# Different Patterns of Codon Usage and Amino Acid Composition across Primate Lentiviruses

**DOI:** 10.3390/v15071580

**Published:** 2023-07-20

**Authors:** Angelo Pavesi, Fabio Romerio

**Affiliations:** 1 Department of Chemistry, Life Sciences and Environmental Sustainability, University of Parma, 43124 Parma, Italy; angelo.pavesi@unipr.it; 2 Department of Molecular and Comparative Pathobiology, Johns Hopkins University School of Medicine, Baltimore, MD 21205, USA

**Keywords:** HIV-1, amino acid diversity, B lymphocytes, codon usage, correlation analysis, monocytes, principal component analysis, primate *Lentiviruses*, T lymphocytes

## Abstract

A common feature of the mammalian *Lentiviruses* (family *Retroviridae*) is an RNA genome that contains an extremely high frequency of adenine (31.7–38.2%) while being extremely poor in cytosine (13.9–21.2%). Such a biased nucleotide composition has implications for codon usage, causing a striking difference between the frequency of synonymous codons in *Lentiviruses* and that in their hosts. To test whether primate *Lentiviruses* present differences in codon and amino acid composition, we assembled a dataset of genome sequences that includes SIV species infecting Old-World monkeys and African apes, HIV-2, and the four groups of HIV-1. Using principal component analysis, we found that HIV-1 shows a significant enrichment in adenine plus thymine in the third synonymous codon position and in adenine and guanine in the first and second nonsynonymous codon positions. Similarly, we observed an enrichment in adenine and in guanine in nonsynonymous first and second codon positions, which affects the amino acid composition of the proteins Gag, Pol, Vif, Vpr, Tat, Rev, Env, and Nef. This result suggests an effect of natural selection in shaping codon usage. Under the hypothesis that the use of synonyms in HIV-1 could reflect adaptation to that of genes expressed in specific cell types, we found a highly significant correlation between codon usage in HIV-1 and monocytes, which was remarkably higher than that with B and T lymphocytes. This finding is in line with the notion that monocytes represent an HIV-1 reservoir in infected patients, and it could help understand how this reservoir is established and maintained.

## 1. Introduction

The genome of *Lentiviruses* shows a strong preference for adenine (A), which accounts for 31.7% (Jembrana disease virus) to 38.2% (Caprine arthritis encephalitis virus) of the genome [1,2,3]. The generation of A-rich genomes may result from selection pressures that favor a less RNA secondary structure, affecting translational efficiency, and selecting amino acids encoded by codons containing A [4]. Thus, the A-pressure can also affect the amino acid usage, resulting in a biased amino acid composition of the Gag-Pol proteins of Human immunodeficiency virus type 1 (HIV-1) and type 2 (HIV-2) when compared to those of Human T-lymphotropic virus type 1 (HTLV-1) and type 2 (HTLV-2) and other *Retroviridae* [5,6].

The strong A-bias, paired with a low content of cytidine (C) ranging from 13.9% (Feline immunodeficiency virus) to 21.2% (Bovine immunodeficiency virus), is directly reflected in the pattern of codon usage in primate *Lentiviruses* [7]. This biased nucleotide composition, together with the CpG suppression typical of all eukaryotic viruses [8,9], led to a strong contrast in codon preferences between *Lentiviruses* and their primate hosts. For example, if we examine the Codon Statistics Database [10], we find that the adenine plus thymine (A + T) percent content in the third codon position of the Simian immunodeficiency virus (SIV) and HIV-1 is remarkably higher (63%) than that (44%) of the respective hosts (Old-World monkeys, African apes, and humans).

Although all members of the genus *Lentivirus* (Feline Immunodeficiency Virus, Bovine Immunodeficiency Virus, Caprine Arthritis Encephalitis Virus, Equine Infectious Anemia Virus, SIV, HIV-1, and HIV-2) have common patterns of codon usage [11], Vidyavijayan et al. detected slight differences between the closely related *Lentiviruses* HIV-1 and HIV-2 [12]. This finding suggests that an extension of the study to a wide phylogenetic range of *Lentiviruses* could reveal the existence of a significant trend in the use of synonymous codons.

In the present study, we assembled a large dataset of genome sequences from primate *Lentiviruses*, which includes SIV species infecting Old-World monkeys and African apes, HIV-2, and the four groups of HIV-1 (M, N, O, and P). We analyzed the codon usage by multivariate statistics, given its ability to condense the information from multiple variables (here the 59 synonymous codons) into a few synthetic variables while preserving most of the information of the original data. We found that the use of synonyms shows a trend—from SIV infecting *Cercopithecus* monkeys to HIV-1 infecting humans—towards a significantly higher content of A + T in the third synonymous codon position. We also found a similar trend (enrichment in A and guanine, G) in nonsynonymous first and second codon positions, which, in turn, affects the amino acid composition of the encoded proteins (Gag, Pol, Vif, Vpr, Tat, Rev, Env, and Nef).

Finally, to test the hypothesis that the use of synonyms in HIV-1 reflects adaptation to expression in particular tissues [13], we compared the codon usage of HIV-1 with that of three different human immune cell types (monocytes, a component of innate immunity, and B and T lymphocytes, components of adaptive immunity), as determined by Ruzman et al. [14] through analysis of transcriptomic data. We found a highly significant correlation between pandemic group M of HIV-1 and monocytes, which was thirty times greater than that with B lymphocytes and nearly five times greater than that with T lymphocytes. This finding is in line with the evidence that monocytes represent an HIV-1 reservoir in infected individuals ([15,16] and references therein).

## 2. Materials and Methods

Sequence data and statistical analysis. From NCBI, we retrieved a first dataset that included 23 genome sequences from virus species representative of the family *Retroviridae*: 20 from members of the *Orthoretrovirinae* subfamily (2 from each genus *Alpha*-, *Beta*-, *Gamma*-, *Delta*-, and *Epsilonretrovirus*, and 10 from the genus *Lentivirus*) and 3 from members of the *Spumaretrovirinae* subfamily. The virus species were as follows: Avian leukosis virus (ALV, NCBI accession number Z46390), Rous sarcoma virus (RSV, AF052428), Mouse mammary tumor virus (MMTV, M15122), Mason–Pfizer monkey virus (M-PMV, M12349), Friend murine leukemia virus (FMLV, M93134), Feline leukemia virus (FLV, M18247), Human T-lymphotropic virus 1 (HTLV-1, D13784), Bovine leukemia virus (BLV, K02120), Walleye dermal sarcoma virus (WDSV, AF033822), Walleye epidermal hyperplasia virus type 1 (WEHV1, AF133051), Bovine immunodeficiency virus (BIV, M32690), Feline immunodeficiency virus (FIV, FIU56928), Caprine arthritis encephalitis virus (CAEV, M33677), Equine infectious anemia virus (EIAV, M16575), Human immunodeficiency virus type 1 (HIV-1, isolate SE7253, AF069670), Human immunodeficiency virus type 2 (HIV-2, isolate BEN, M30502), Simian immunodeficiency virus (SIV, JQ864086), Jembrana disease virus (JDV, NC_001654), Puma lentivirus (PLV, MN531112), Visna/maedi virus (VMV, M60609), Bovine foamy virus (BFV, U94514), Feline foamy virus (FFV, Y08851), and Simian foamy virus human-isolate (SFV, U21247).

From each genome sequence, we selected the non-overlapping region that encodes the Gag, Pol, and Env proteins. We excluded overlapping regions (e.g., the *gag/pol* overlap) with the aim of obtaining a pattern of codon usage not affected by the evolutionary interdependence between overlapping sequences. We analyzed the codon usage of each region using the Nc index [17] and the relative synonymous codon usage (RSCU) index [18]. Nc is a sample measure of the effective number of codons (ENC; Supplementary File S1) that ranges from 20 to 61, where 20 is the value obtained when only one codon is used for each amino acid (i.e., the codon bias is maximum) and 61 is the value obtained when all synonymous codons for each amino acid are equally used (i.e., no codon bias). The RSCU value for each synonymous codon was calculated as follows:

RSCU = (N_codon_/N_amino acid_) × D,

where N_codon_ is the absolute frequency of a synonymous codon, N_amino acid_ is the absolute frequency of the amino acid specified by that codon and its synonyms, and D is the degeneracy of that amino acid (when all synonyms for a given amino acid are used with equal frequencies, a RSCU value of 1 for each codon is expected). We obtained a matrix of 23 rows (number of *Retroviridae*) and 59 columns (RSCU of the 59 codons). We analyzed the transpose of the matrix (59 rows and 23 columns) by means of principal component analysis (PCA) [19,20,21] and using the OriginPro software (OriginLab, Northampton, MA, USA). To further detail the pattern of codon usage in *Retroviridae*, we calculated in each coding region the percent frequency of A, T, G, C, A + T, and G + C at the third position of synonymous codons.

From the NCBI database, we retrieved a second dataset that included 96 genome sequences from virus species covering a wide phylogenetic range of primate *Lentiviruses* (Supplementary Table S1), 68 of which were from a dataset of 124 sequences utilized in our previous study [22]. All 96 sequences were chosen based on the availability of the entire genomic sequence and the absence of an intact *asp* ORF overlapping the *env* gene. Overall, the dataset included 37 sequences from SIV infecting *Cercopithecinae*, 4 from HIV-2, 20 from SIV infecting *Homininae*, and 35 from HIV-1 (9 from group O, 2 from group P, 7 from group N, and 17 from pandemic group M). For group M HIV-1 strains, the dataset includes 2 sequences from subtype A, 4 from B, 5 from C, 1 from D, 1 from F, 2 from G, 1 from H, and 1 circulating recombinant form. The number of sequences representing subtypes A, B, and C approximates the worldwide prevalence of the subtype itself.

From each genome sequence, we selected the nonoverlapping region that encodes not only the structural and enzymatic proteins Gag, Pol, and Env, but also the accessory/regulatory proteins Vif, Vpr, Tat, Rev, and Nef (Supplementary File S2). Importantly, from the sequence of the *env* gene, we also excluded the region corresponding to the Rev Response Element (RRE), a highly conserved structural RNA element with regulatory function that could confound the analysis of coding sequences [23]. Detailed information about the 96 genome sequences (virus isolate, host, length of the selected coding region, NCBI accession number, and the “effective number of codons” ENC [17]) is given in Supplementary Table S1. We analyzed the codon usage of each region using the RSCU index [18]. As input data for principal component analysis (PCA), we obtained a matrix of 96 rows (number of primate *Lentiviruses*) and 59 columns (RSCU of synonyms). We also extended PCA to analyze the amino acid composition, using as input data a matrix of 96 rows and 18 columns (percent amino acid content of the 18 amino acids encoded by the 59 synonyms).

Using the Pearson correlation coefficient *r*, we compared the pattern of codon usage in HTLV-1, HIV-1, and HIV-2 with the overall pattern of codon usage in human monocytes, and B and T lymphocytes, as determined by Ruzman et al. [14] through analysis of transcriptomic data downloaded from public databases: 11644 protein coding genes from monocytes [24], 10940 from B lymphocytes, and 12094 from T lymphocytes. This correlation analysis was extended to SIV infecting Old-World monkeys and African apes. To test if the two correlation coefficients are significantly different from each other, we used the Hotelling *t*-test [25,26]. Using the contingency-table chi-square test [27], we compared the amino acid content of SIV-infected monkeys to that of pandemic group M of HIV-1.

## 3. Results

### 3.1. Principal Component Analysis (PCA) of Codon Usage in the Family of Retroviridae Revealed a Considerable Degree of Variation

**Figure 1:**
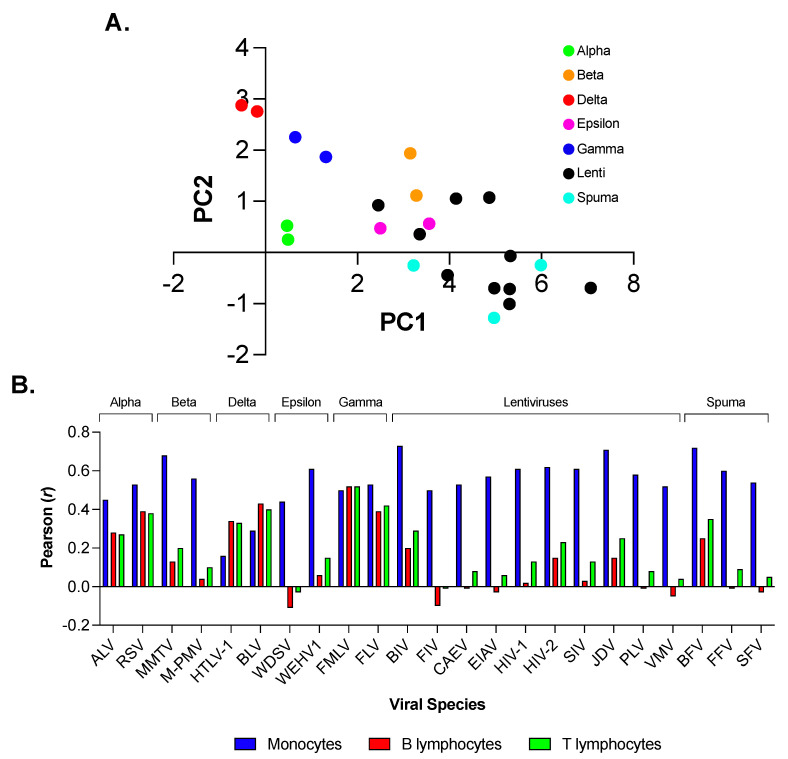
Principal component analysis (PCA) of the codon usage in 23 virus species representative of the family *Retroviridae*. PC1 and PC2 account for 77.1% and 8.5% of the total amount of variation in the input data matrix, respectively. Taken together, the two PCs account for 85.6% of the total variation, namely, the reduction from 59 to 2 variables results in a relatively small (14.4%) loss of information Panel (**A**). Correlation analysis between the RSCU value of 59 synonymous codons in HTLV-1, HIV-1, and HIV-2 and that in human monocytes, B and T lymphocytes. Correlation with monocytes was significant for HIV-1 and HIV-2, but not for HTLV-1. Correlation with B and T lymphocytes was significant for HTLV-1 but not for HIV-1 and HIV-2 Panel (**B**).

By means of PCA, we summarized the information carried by the input data matrix (59 rows and 23 columns; see Section 2) into two synthetic variables, that is, the first (PC1) and second (PC2) principal components. Taken together, the two PCs accounted for 85.6% of the total amount of variation in the use of synonymous codons in the 23 virus species representative of the family *Retroviridae* (77.1% accounted for by PC1 and 8.5% by PC2). We represented the 23 virus species as a swarm of points on a two-dimensional map (Figure 1A). The projection of points on the first axis of the map, from the lowest negative PC1 score (−0.51 for HTLV-1) to the highest positive PC1 score (7.1 for FIV), reflects a strong increase in the A + T content in the third codon position, from 48% in HTLV-1 to 82% in FIV. In detail, the mean A + T content of *Alpha*-, *Delta*-, and *Gammaretroviruses* was 49.4% with a standard deviation (sd) of 2.0%, whereas that of *Beta*- and *Epsilonretroviruses*, *Spumaretrovirnae,* and *Lentiviruses* was 69.2% (sd = 6.6%). The projection of points on the second axis of the map (Figure 1A) brings together Delta- (HTLV-1 and BLV) with *Gammaretroviruses* (FMLV and FLV), because of a mean C content in the third codon position (36.7% and 29.7%, respectively) that is two-fold higher than that in the remaining 19 *Retroviridae* (16.1%; sd = 5.0%).

As the dataset of *Retroviridae* includes three virus species infecting humans (HTLV-1, HIV-1, and HIV-2), we compared their pattern of codon usage with that in human monocytes, and B and T lymphocytes ([14]; see Section 2). We found that HTLV-1 shows a significant correlation with B lymphocytes (*r* = 0.34; t-Student = 2.70; *p* = 0.008) and T lymphocytes (*r* = 0.33; t-Student = 2.61; *p* = 0.011), but not with monocytes (*r* = 0.16; t-Student = 1.21; *p* = 0.23) (Figure 1B). The t-Hotelling test revealed that the correlation of HTLV-1 with B and T lymphocytes was significantly higher than that with monocytes (t = 1.74; *p* < 0.05 and t = 1.89; *p* < 0.05, respectively).

On the other hand, HIV-1 and HIV-2 showed a significant correlation with monocytes (*r* = 0.61; t-Student = 5.81; *p* = 10^−^^5^ and *r* = 0.62; t-Student = 6.01; *p* = 10^−^^5^, respectively), but not with B lymphocytes (*r* = 0.02; t-Student = 0.15; *p* = 0.88 and *r* = 0.15; t-Student = 1.17; *p* = 0.26, respectively) and T lymphocytes (*r* = 0.13; t-Student = 1.03; *p* = 0.33 and *r* = 0.23; t-Student = 1.82; *p* = 0.079, respectively) (Figure 1B). The t-Hotelling test revealed that the correlation of HIV-1 and HIV-2 with monocytes was significantly higher than that with B lymphocytes (t = 8.82; *p* = 10^−^^5^ and t = 6.05; *p* = 10^−^^5^, respectively) and T lymphocytes (t = 7.88; *p* = 10^−^^5^ and t = 5.72; *p* = 10^−^^5^, respectively).

### 3.2. Principal Component Analysis (PCA) in Primate Lentiviruses Revealed a Trend in the Use of Synonymous and Nonsynonymous Codons

Using the RSCU index, we evaluated the pattern of codon usage in the dataset of 96 genome sequences from a wide range of primate *Lentiviruses*: 37 sequences from SIV infecting Old-World monkeys, 20 from SIV infecting African apes, 4 from HIV-2, and 35 from HIV-1, both infecting humans (Supplementary Table S1).

**Figure 2:**
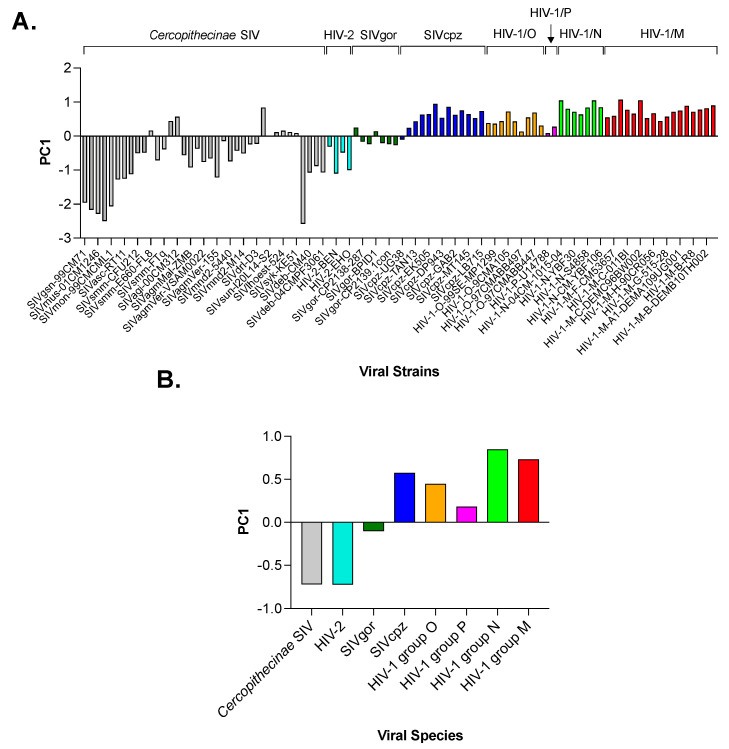
Principal component analysis (PCA) of the codon usage in 96 primate *Lentiviruses*. Panel (**A**) shows the distribution of PC1, which accounts for 36.1% of the total amount of variation. Panel (**B**) highlights the separation between SIV infecting monkeys and HIV-2 at one end (mean PC1 score markedly below zero) and SIV infecting African apes and HIV-1 at the other (mean PC1 score slightly below zero and markedly above zero, respectively).

By analyzing the resulting matrix (96 rows and 59 columns) with PCA, we found that PC1 accounts for 36.1% of the total amount of variation in the use of synonyms. The distribution of PC1 score points out a trend in the pattern of codon usage, which separates SIV infecting Old-World monkeys and HIV-2 (for both a mean PC1 score of −0.72) from SIV infecting African apes and the four HIV-1 groups (Figure 2A). Among the latter viruses, the mean PC1 score ranged from −0.10 in SIVgor to 0.73 in HIV-1 group M and 0.85 in HIV-1 group N (Figure 2B).

**Figure 3:**
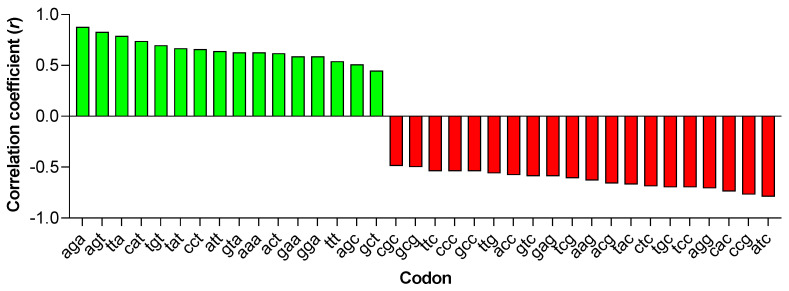
Correlation between the PC1 score of the 96 primate *Lentiviruses* and the RSCU of individual codons. Green columns indicate 16 codons with a highly significant positive correlation (*r* from 0.45 to 0.88). All codons, except AGC, have A or T in the third position. Red columns indicate 20 codons with a highly significant negative correlation (*r* from −0.49 to −0.77). All codons have G or C in the third position.

Using the Pearson correlation coefficient *r* under a t-Student cut-off of significance > 4.84 (*p* = 10^−5^), we found (i) a highly positive correlation, ranging from *r* = 0.51 to *r* = 0.88, between the PC1 score and the RSCU of 16 codons, 15 of them with A or T in the third position (see green columns in Figure 3); (ii) a highly negative correlation, ranging from −0.49 to −0.77, between the PC1 score and the RSCU of 20 codons all ending with G or C (see red columns in Figure 3).

In the case of the two synonyms AGA and AGG for Arg, which are the most preferred codons because of the strong suppression of the remaining four CG-synonyms, the mean RSCU of AGA was 3.4 in SIV infecting monkeys and 3.5 in HIV-2, and it increased to 3.8 in SIV infecting African apes and to 3.9 in HIV-1. The mean RSCU of AGG was 1.9 in SIV infecting monkeys and HIV-2, and it decreased to 1.7 in SIV infecting apes and to 1.6 in HIV-1. Despite these small differences for the AGA and AGG synonyms and for the others, the first principal component identified a clear trend in codon usage (Figure 2A).

**Figure 4:**
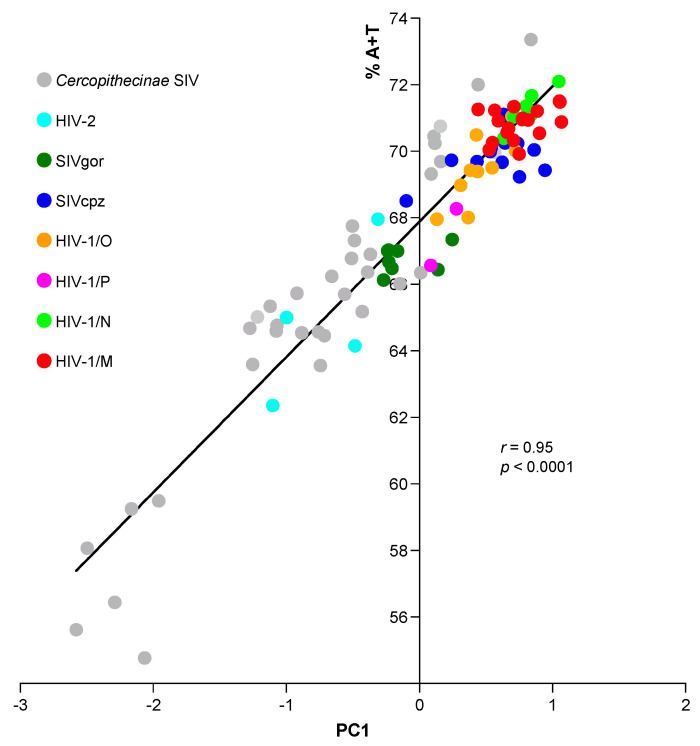
Correlation between the PC1 score of the 96 primate *Lentiviruses* (eight groups) and the respective percent content of A + T in the third position of the 59 synonymous codons (*r* = 0.95).

In line with these findings, the correlation between the PC1 score of each primate *Lentivirus* and the respective percent content of A + T at the third position of the 59 synonyms was extremely high (*r* = 0.95). The dispersion of points shown in Figure 4 shows a significant trend towards an increased A + T content from SIV infecting Old-World monkeys (mean content = 65.4%; sd = 4.4%) to pandemic group M of HIV-1 (mean content = 70.4%; sd = 1.2%). The difference between the two mean contents was statistically significant (t-Student = 6.67; *p* = 10^−^^5^).

Finally, we assessed the presence of a correlation between PC1 and the nucleotide composition at the first and second nonsynonymous codon positions of the 59 synonymous codons. Since a nucleotide substitution in the first codon position does not cause an amino acid change in 4.6% of cases, in the analysis of the first codon position, we excluded the synonyms AGA, CGA, AGG, and CGG for Arg and the synonyms TTA, CTA, TTG, and CTG for Leu. As shown in Table 1, under a t-Student cut-off of significance > 4.84 (*p* = 10^−^^5^), we found a positive correlation between PC1 and the percent content of A (*r* = 0.83) and a negative correlation between PC1 and the percent content of T (*r* = −0.75) and C (*r* = −0.76) (Supplementary Figure S1). In the analysis of the second codon position, where a nucleotide substitution causes an amino acid change in 100% of cases, we found a positive correlation between PC1 and the percent content of G (*r* = 0.74) and T (*r* = 0.52), and a negative correlation between PC1 and the percent content of C (*r* = −0.51) (Supplementary Figure S2).

In addition to the strong correlations between PC1 and the nucleotide composition at the third synonymous codon position (Table 1), the finding of significant correlations also between PC1 and the nucleotide composition at the first and second nonsynonymous codon positions (Table 1) suggests a trend in primate *Lentivirus* that likely affects their amino acid composition.

### 3.3. Principal Component Analysis (PCA) in Primate Lentiviruses Revealed a Trend in the Amino Acid Composition

To avoid the influence of evolutionary constraints on protein regions encoded by overlapping reading frames, we analyzed the amino acid composition in primate *Lentiviruses* using as input data the amino acid sequence obtained from the non-overlapping regions encoding proteins common to all *Lentiviruses*: Gag, Pol, Env, Tat, Rev, Vif, Vpr, and Nef.

By means of PCA, we summarized the information carried by the input data matrix (96 rows for each primate *Lentivirus*, and 18 columns with the percent content of the 18 amino acids specified by the 59 synonyms) into the two principal components, PC1 and PC2. Together, they account for 42.4% of the total amount of variation in the amino acid composition of primate *Lentiviruses* (25.4% accounted for by PC1 and 17.0% by PC2).

**Figure 5:**
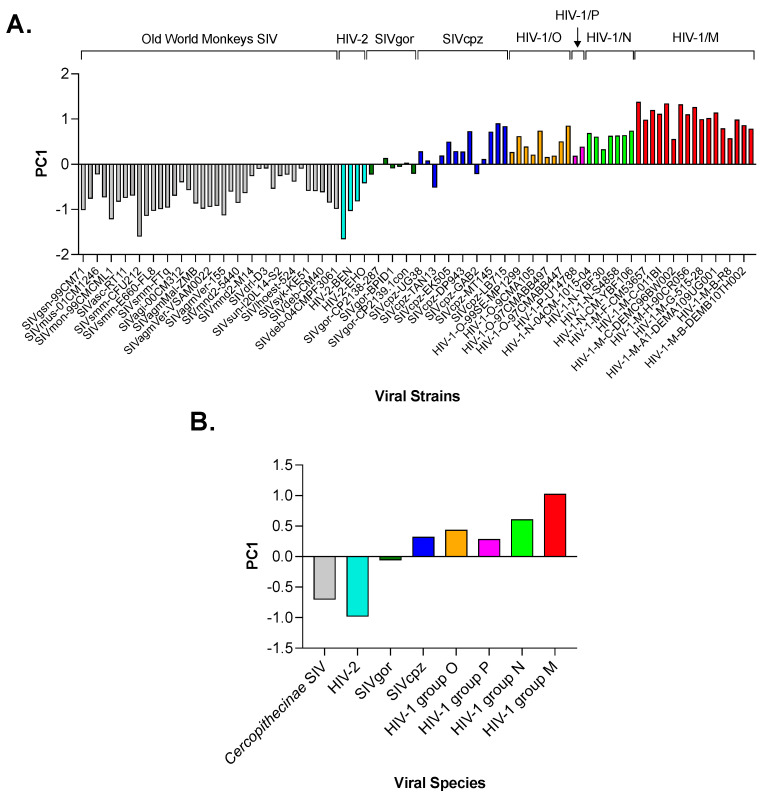
Principal component analysis (PCA) of the amino acid composition in 96 primate *Lentiviruses*. Panel (**A**) shows the distribution of PC1, which accounts for 25.4% of the total amount of variation. Panel (**B**) shows a trend in the amino acid composition that separates SIV infecting monkeys and HIV-2 from SIV infecting African apes and HIV-1. The trend is notably strong for pandemic group M of HIV-1.

However, we found that PC1, but not PC2, shows a substantial trend in the amino acid composition from SIV infecting monkeys and HIV-2 at one end to SIV infecting African apes and HIV-1 at the other (Figure 5A). Subdivision of primate *Lentivirus* into 8 groups and calculation of the mean PC1 score for each group revealed that the trend is remarkably strong for pandemic group M of HIV-1 (Figure 5B).

By correlation analysis under a t-Student cut-off of significance > 4.84 (*p* = 10^−^^5^), we found (i) a positive correlation between the PC1 score and the percent content of Ile (*r* = 0.93) and Ser (*r* = 0.69); (ii) a negative correlation between the PC1 score and the percent content of Tyr (*r* = −0.81), and Leu (*r* = −0.60).

By comparing the overall content of Ile and Ser in SIV-infected monkeys (6.7% and 4.4%, respectively) to that in HIV-1 group M (7.8% and 5.2%, respectively), we found in both cases a statistically significant difference (χ^2^ = 47.98; *p* = 10^−^^5^ and χ^2^ = 38.16; *p* = 10^−^^5^, respectively). By comparing the overall content of Tyr and Leu in SIV infecting monkeys (3.7% and 8.6%, respectively) to that in HIV-1 group M (3.1% and 8.2%, respectively) we found in both cases a statistically significant difference (χ^2^ = 27.08; *p* = 10^−^^5^, χ^2^ = 4.41; *p* = 0.03, respectively).

Therefore, the trend obtained with PCA finds statistical confirmation for a number of amino acids (Figure 5). In particular, pandemic group M of HIV-1 shows a significant increase in the content of Ile and Ser and a significant decrease in the content of Tyr and Leu.

### 3.4. Primate Lentiviruses Have a Codon Usage Significantly Correlated to That of Human Monocytes (Innate Immunity) but Not to That of Human B and T Lymphocytes (Adaptive Immunity)

**Figure 6:**
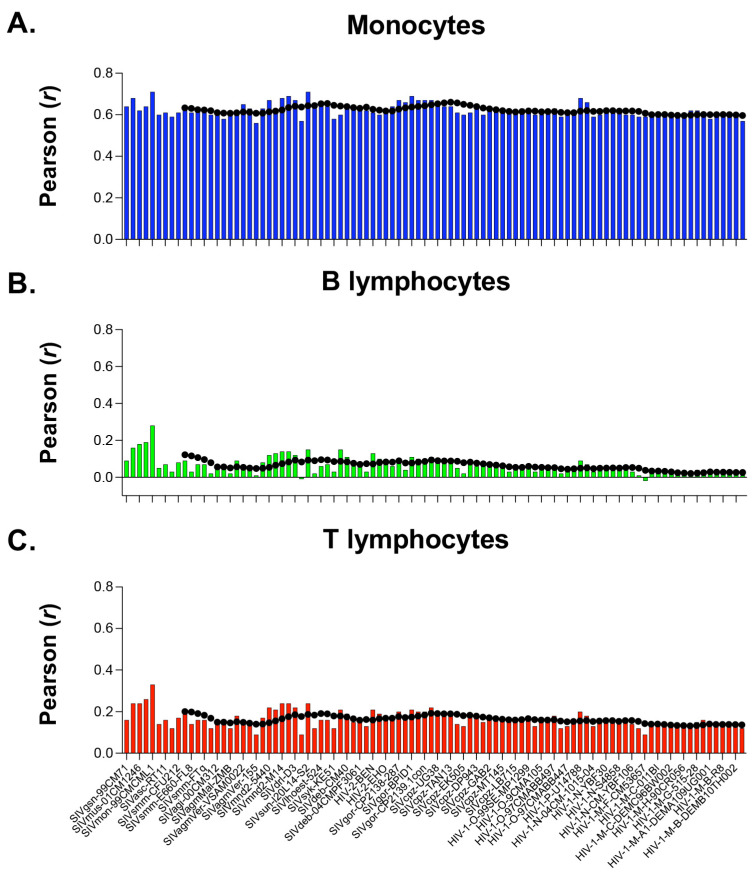
Correlation analysis between the RSCU value of 59 synonymous codons in 96 primate *Lentiviruses* and that in human monocytes, B and T lymphocytes. Panel (**A**) shows the distribution of the correlation coefficients with monocytes; panel (**B**) shows that with B lymphocytes; and panel (**C**) shows that with T lymphocytes.

We found that the codon usage of each of the 96 primate *Lentiviruses* shows a significant correlation with the overall codon usage of human monocytes (Figure 6A; *r* ranging from 0.56 to 0.71; mean = 0.62; sd = 0.03). This highly significant correlation is in line with the similar frequency of A/T-ending codons between genes expressed in monocytes (53.4%) and in primate *Lentiviruses* (from 54.3% in SIV infecting *Cercopithecus mona*, ac. number AY340701, to 72.1% in HIV-1 group N, ac. number DQ017382).

In contrast, we did not find a significant correlation between the RSCU of the 96 primate *Lentiviruses* and that of B lymphocytes (mean value of *r* = 0.06; sd = 0.05; Figure 6B) and T lymphocytes (mean value of *r* = 0.16; sd = 0.04; Figure 6C). The lack of correlation is due to the fact that genes expressed in these two cell types show a frequency of A/T-ending codons of 45.2% and 46.7%, respectively, much lower than in primate *Lentiviruses* (from 54.3 to 72.1%).

More specifically, we found that the RSCU of the 17 isolates from pandemic group M of HIV-1 shows a mean correlation with RSCU from monocytes (*r* = 0.60; sd = 0.01) that is thirty times greater than the mean correlation with B lymphocytes (*r* = 0.02; sd = 0.01) and nearly five times greater than the mean correlation with T lymphocytes (*r* = 0.13; sd = 0.01).

**Figure 7:**
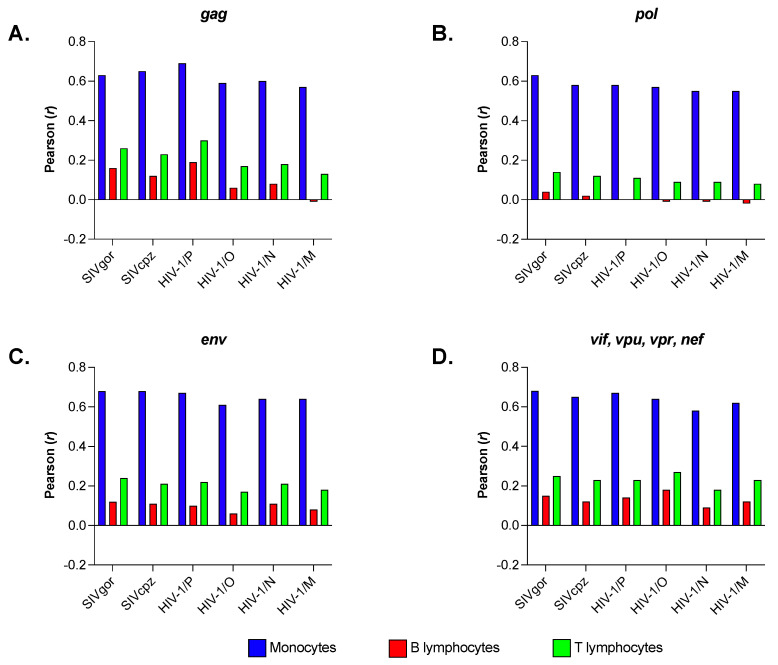
Correlation analysis between the codon usage in individual genes (*gag*, *pol*, and *env*) or in regulatory/accessory genes taken together (*tat*, *rev*, *vif*, *vpr*, and *nef*) and that in human monocytes, B and T lymphocytes. This analysis concerned a subset of primate *Lentiviruses*: SIV infecting gorillas (7 sequences), SIV infecting chimpanzees (13 sequences), HIV-1 group P (2 sequences), HIV-1 group O (9 sequences), HIV-1 group N (7 sequences), and HIV-1 group M (17 sequences). Panel (**A**) shows the extent of correlation in *gag*, panel (**B**) in *pol*, panel (**C**) in *env*, and panel (**D**) in the pool of regulatory/accessory genes.

We carried out the same analysis by comparing the RSCU of individual genes (*gag*, *pol*, and *env*) and the RSCU of the regulatory and accessory genes taken together (*tat*, *rev*, *vif*, *vpr*, and *nef*) with the RSCU of monocytes, B and T lymphocytes. We limited this analysis to SIV infecting African apes and the four groups of HIV-1. We found that the extent of correlation between RSCU of individual genes, or groups of genes, and RSCU of monocytes (see blue columns in Figure 7A–D) does not vary significantly, as does the extent of correlation with RSCU of B and T lymphocytes (red and green columns in Figure 7A–D).

A much greater correlation of codon usage between primate *Lentiviruses* and monocytes compared to T lymphocytes could be revealed in terms of the original cell tropism of these viral species.

## 4. Discussion

The strong divergence we found in the use of synonymous codons in the family *Retroviridae* (Figure 1) confirms previous studies on the compositional features of *Retroviridae* [3,6,28]. However, the poorly homogeneous distribution of the PC1 score that we found in *Lentivirus* (see black circles in Figure 1) raised the hypothesis of a variable pattern of codon usage within this genus.

We tested this hypothesis by investigating the use of synonymous codons in a dataset of primate *Lentiviruses* infecting humans (HIV-1 and HIV-2) and their closest relatives, such as Old-World monkeys and African apes (SIV). The question we addressed is whether there is a significant change in the compositional properties—from SIV infecting monkeys to HIV-1, and in particular to the pandemic HIV-1 strains of group M—or, alternatively, whether the frequency of occurrence of synonymous codons in primate *Lentiviruses* shows small random variations that are not statistically significant.

Using PCA, we found that the distribution of the PC1 score (Figure 2A,B) points out a trend from SIV to HIV-1 towards an increased frequency of A/T ending codons, which was further highlighted by correlation analysis (Figure 3 and Figure 4). A strong bias for A/T in the third codon position of all HIV-1 genes, especially the structural ones, has been detected by Pandit and Sinha [29]. Our study, however, places this feature in a larger evolutionary context, as demonstrated by the clustering of HIV-1, particularly of pandemic group M, with respect to the other primate *Lentiviruses* (Figure 4). Studies have shown that both T cells and myeloid cells express proteins of the APOBEC3 family (primarily APOBEC3G and APOBEC3F), which are part of the cell’s intrinsic immune response [30]. The HIV-1 protein Vif promotes viral infection by counteracting APOBEC3G and F. However, Vif does not seem to have the same effect on other members of the APOBEC3 family, such as APOBEC3B and H, which also limit HIV-1 replication [31,32]. Therefore, it is conceivable that primate lentiviruses may have learned to coexist with these cellular enzymes through a different strategy, namely by evolving a genome with high A content.

There are, however, important exceptions to the strong A/T bias in the third codon position of HIV-1, including at silent codon positions where the presence of any base would encode the same amino acid due to the degeneracy of the genetic code. Indeed, a recent study identified 190 out of 2290 (8.3%) silent third codon positions in the HIV-1 RNA genome where A is found in less than 1% of the sequences (“noA” positions) [33]. The largest cluster of noA positions overlaps the RRE sequence, a highly conserved structural sequence that is essential in the virus lifecycle. However, a number of noA positions and a few smaller clusters are also present in the non-overlapping regions of the *gag*, *pol*, *env* (outside of RRE), and *nef* genes [33]. In silico analyses suggested that the absence of A residues at these silent codon positions may play a structural role in the correct folding of the viral RNA [33]. In the present study, we did not exclude the 190 noA positions above from our analyses, which may represent a confounding factor in the results we report here.

The results in Figure 2 show consistency between PC1 values for HIV-1 group M and SIVcpz, which is in line with the fact that the former originated from the latter [34]. On the other hand, our results do not show much consistency between PC1 for HIV-1 group O and SIVgor. Phylogenetic studies suggest that HIV-1 group O is most closely related to SIVgor, which originated from SIVcpz transmitted to gorillas [35]. However, it has not been conclusively determined whether HIV-1 group O was transmitted to humans from gorillas or from chimpanzees infected with a strain of SIVcpz more closely related to HIV-1 group O [35]. Therefore, the inconsistency between PC1 of HIV-1 group O and SIVgor may not be entirely surprising.

The highly significant correlation we observed between the PC1 score and nucleotide composition in the first and second codon positions (e.g., an increase of A in the first position and of G in the second position; Table 1) is indicative of positive selection in primate *Lentiviruses*. Indeed, PCA of the amino acid composition highlights a change in the amino acid composition (Figure 5A,B). More specifically, when compared to SIV infecting monkeys, pandemic group M of HIV-1 shows a significant increase in Ile and Ser content and a significant decrease in Tyr and Leu content. In a previous study based on a combined computational-experimental approach, Mayrose et al. detected in the genome of HIV-1 a number of regions exhibiting strong purifying selection against synonymous substitutions [36]. Signatures of positive selection in HIV-1 genes have been detected within HIV-1 in infected individuals [37]. Sites under positive selective pressure, in the context of protein structural constraints, have been mapped in the HIV-1 genome by Snoeck et al. [38].

Using the overall pattern of codon usage from genes expressed in human monocytes, B and T lymphocytes [14], we found that the use of synonyms in all primate *Lentiviruses* is significantly correlated with monocytes but not with lymphocytes (Figure 6). Interestingly, we found that pandemic group M of HIV-1 shows a correlation with monocytes that is thirty times greater than that with B lymphocytes and nearly five times greater than that with T lymphocytes. A similar correlation was observed for SIV. Indeed, both HIV-1 and SIV have been shown to infect monocytes, tissue macrophages, and microglial cells [16,39,40].

Several reasons may explain the high correlation between the codon usage of SIV/HIV-1 and monocytes. First, it could be advantageous for SIV/HIV-1 to utilize a codon bias that is more similar to the one in cells that provide a less favorable environment for viral replication. Second, SIV/HIV-1 most frequently utilize the mucosal route of transmission (during both vertical and horizontal transmission), and myeloid cells are the first cells they encounter at the portal of entry. Adaptation to replicate in myeloid cells through various strategies, including codon usage, may be key during primary infection to establish a firm foothold in the new host. Third, the central nervous system (CNS) is an immune privileged site that provides an ideal environment for unchecked viral replication, and microglia are the main cell type targeted by SIV/HIV-1 in the CNS. It is also possible that *Lentiviruses* may have evolved as myelotropic viruses, and that modern day primate *Lentiviruses* conserved the codon bias of their ancestors. On the other hand, there is some evidence that, during the late stages of infection, HIV-1 manipulates the tRNA pool in host cells to its advantage [41]. If that were indeed the case, differences in codon bias between the virus and the various cell types that HIV-1 infects could become irrelevant.

Despite the finding of a significant contribution of monocytes or monocyte-derived macrophages as reservoirs of persistent HIV-1 [15,16], the interaction between myeloid cells and HIV-1 remains elusive. However, Real et al. [42] demonstrated that macrophage tissue reservoirs contain transcriptionally active HIV-1 and viral particles accumulated in vacuoles known as virus-containing compartments (VCCs). This structure is absent from infected CD4+ T cells that are unable to store viral particles [43]. In macrophages, VCCs might serve as storage compartments for infectious viruses produced despite combined antiretroviral therapy (cART) and prone to being transferred to other target cells upon external stimulation during cART [44] and/or once antiretroviral therapy is interrupted ([45] and references therein).

While it could be misleading to draw conclusions about cell tropism from in silico analyses alone, our studies found a significant correlation between the codon usage of HIV-1 and that of genes expressed in monocytes (Figure 6 and Figure 7). These results could provide clues about the origins of HIV-1 and suggest new avenues of research into HIV-1 replication in the myeloid compartment and viral pathogenesis.

## 5. Conclusions

The present study points out a fine bias in the frequency of both synonymous and nonsynonymous codons that is peculiar to HIV-1 (particularly the pandemic HIV-1 strains of group M) and SIV infecting African apes (particularly SIV infecting chimpanzees). An impact on the varying pathogenesis between HIV-1 and HIV-2 can be hypothesized by the different patterns in codon usage and amino acid composition we identified between the two viruses. Finally, the highly significant correlation in codon usage between HIV-1 and monocytes could be a key, together with others known or yet to be discovered, to explaining how the monocyte reservoir is established and maintained.

## Figures and Tables

**Table 1:** Correlation test between the PC1 score of the 96 primate Lentiviruses and the corresponding percent content of A, T, G, C, A + T, and G + C at the first, second, and third codon positions (characters in bold indicate a correlation coefficient r with *p* < 10^−^^5^).

Primate Lentiviruses	A	T	G	C	A + T	G + C
PC1 vs. first codon position	**0.83**	**−0.75**	0.27	**−0.76**	**0.60**	**−0.60**
PC1 vs. second codon position	−0.32	**0.52**	**0.74**	**−0.51**	0.11	−0.11
PC1 vs. third codon position	**0.83**	**0.85**	**−0.88**	**−0.84**	**0.95**	**−0.95**

## Data Availability

The present study was conducted using sequences previously deposited in the National Center for Biotechnology Information (NCBI) database at the National Library of Medicine (https://www.ncbi.nlm.nih.gov/, accessed on 1 July 2023). From the NCBI database, we compiled six sequence datasets that are contained in Supplementary Files S1–S6 within the Supporting Information. Each Supplementary File contains all the NCBI accession numbers of the sequences included in each of the six datasets. The sequences included in three of the datasets (see Supplementary Files S4 and S5) were also included in our previous study [22].

## Supplementary Materials

The following supporting information can be downloaded at: https://www.mdpi.com/article/10.3390/v15071580/s1, Supplementary Figure S1: Correlation between the PC1 score and nucleotide content in the first codon position; Supplementary Figure S2: Correlation between the PC1 score and nucleotide content in the second codon position; Supplementary Table S1: List of 96 genome sequences from primate *lentiviruses*; Supplementary File S1: Dataset of 23 genome sequences from virus species representative of the family *Retroviridae*; Supplementary Files S2–S6: Datasets of 96 nucleotide sequences from primate lentiviruses.
