# Supplementary material for: Different Patterns of Codon Usage and Amino Acid Composition across Primate Lentiviruses
Source: Viruses. 2023 Jul 20;15(7):1580. doi: 10.3390/v15071580 (PMC10385858; doi:10.3390/v15071580)
Supplement: Supplementary file 1 [file viruses-15-01580-s001.zip › viruses-2444075-supplementary.pdf]

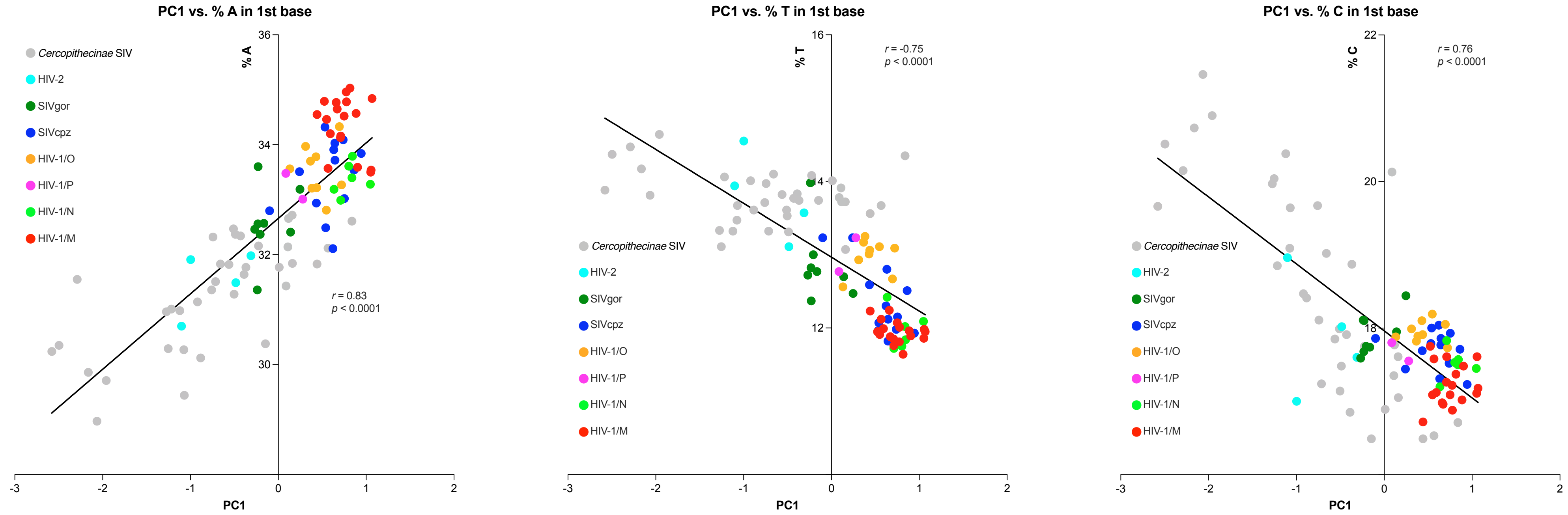

Supplementary Figure S1. Correlation between the PC1 score and nucleotide content in the first codon position.

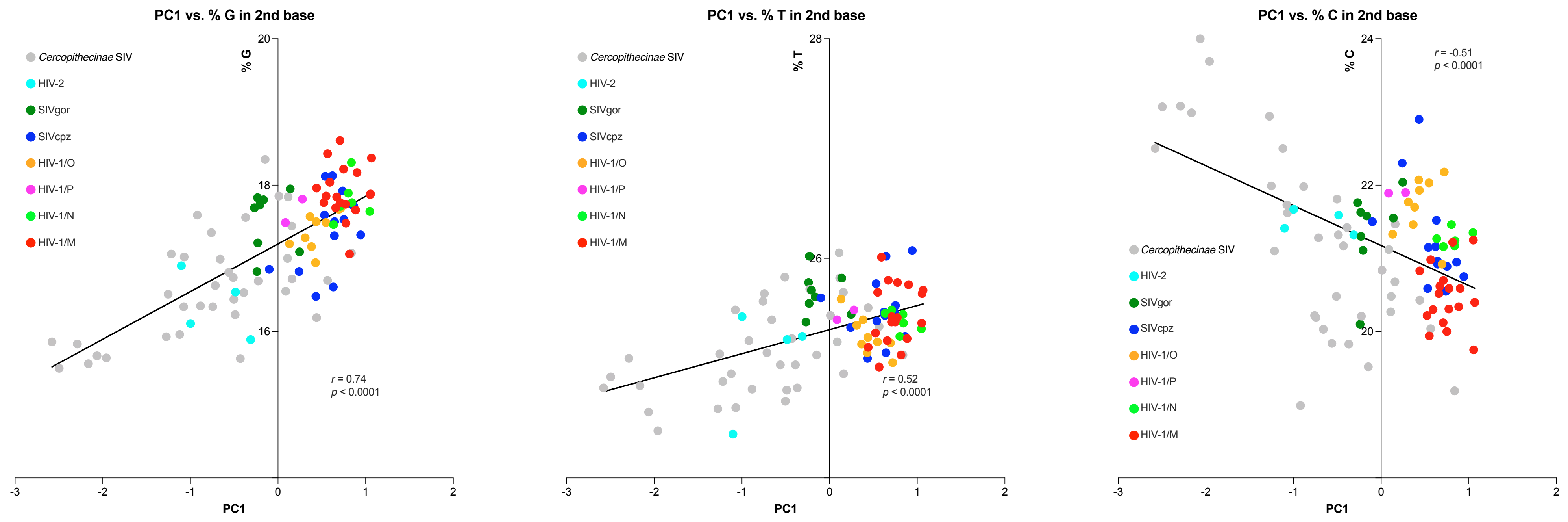

Supplementary Figure S2. Correlation between the PC1 score and nucleotide con-tent in the second codon position

Supplementary Table S1. List of 96 genome sequences from primate lentiviruses: 37 sequences from SIV infecting Old-World monkeys, 4 from HIV-2, 20 from SIV infecting African apes, and 35 from HIV-1 (9 from group O, 2 from group P, 7 from group N, and 17 from the pandemic group M). From each genome sequence we selected the non-overlapping region that encodes the 8 proteins common to all primate lentiviruses (*gag, pol, env, tat, rev, vif, vpr,* and *nef*) and we excluded the coding region of *env* that corresponds to the Rev Responsive Element (RRE, 246 nt). We report the length of the non-overlapping coding sequence, the number of codons within the non-overlapping coding sequence, and the effective number of codons (ENC) determined as in Wright, *Gene*, **1990**, *87*:23-29 (ref. 17).

#	Virus species	Isolate	NCBI acc. #	Length non-overlapping CDS (nt)	Number of codons	Effective number of codons	Host
1	*Cercopithecinae* SIV	SIVgsn (99CM71)	AF468658	7257	2419	49.5	*C. nictitans*
2	*Cercopithecinae* SIV	SIVgsn (99CM166)	AF468659	7182	2394	50.6	*C. nictitans*
3	*Cercopithecinae* SIV	SIVmus (01CM1246)	EF070329	7377	2459	50.3	*C. cephus*
4	*Cercopithecinae* SIV	SIVmus (01CM1239)	EF070330	7335	2445	49.9	*C. cephus*
5	*Cercopithecinae* SIV	SIVmon (99CMCML1)	AY340701	7329	2443	53.3	*C. mona*
6	*Cercopithecinae* SIV	SIVasc (RT03)	KJ461716	7461	2487	46.1	*C. ascanius*
7	*Cercopithecinae* SIV	SIVasc (RT11)	KJ461714	7554	2518	46.3	*C. ascanius*
8	*Cercopithecinae* SIV	SIVasc (RT08)	KJ461715	7464	2488	46.2	*C. ascanius*
9	*Cercopithecinae* SIV	SIVsmm (CFU212)	JX860407	7215	2405	44.6	*C. atys*
10	*Cercopithecinae* SIV	SIVmne (027)	U79412	7287	2429	45.3	*M. nemestrina*
11	*Cercopithecinae* SIV	SIVsmm (E660-FL8)	JQ864086	7218	2406	44.0	*M. mulatta*
12	*Cercopithecinae* SIV	SIVsmm (SL92b)	AF334679	7233	2411	46.6	*C. atys*
13	*Cercopithecinae* SIV	SIVsmm (FTq)	JX860414	7200	2400	45.0	*C. atys*
14	*Cercopithecinae* SIV	SIVrcm (02CM8081)	HM803689	7230	2410	42.1	*C. torquatus*
15	*Cercopithecinae* SIV	SIVagi (00CM312)	HM803690	7203	2401	41.9	*C. agilis*
16	*Cercopithecinae* SIV	SIVgri (677)	M58410	7014	2338	46.8	*C. sabaeus*
17	*Cercopithecinae* SIV	SIVagmMal (ZMB)	LC114462	7218	2406	47.5	*C. cynosuros*
18	*Cercopithecinae* SIV	SIVagmVer (VSAC4004)	KR862336	7239	2413	46.5	*C. sp.*
19	*Cercopithecinae* SIV	SIVagmVer (VSAM0022)	KR862356	7275	2425	48.7	*C. sp.*
20	*Cercopithecinae* SIV	SIVagmVer (VSAG1003)	KR862363	7185	2395	46.7	*C. sp.*
21	*Cercopithecinae* SIV	SIVagmVer (155)	M29975	7350	2450	47.4	*C. aethiops*
22	*Cercopithecinae* SIV	SIVcol (CGU1)	AF301156	6996	2332	45.3	*C. guereza*
23	*Cercopithecinae* SIV	SIVmnd2 (5440)	AY159322	7449	2483	47.6	*M. leucophaeus*
24	*Cercopithecinae* SIV	SIVmnd2 (CM98CM14)	AF367411	7014	2338	45.8	*M. sphinx*
25	*Cercopithecinae* SIV	SIVmnd2 (M14)	AF328295	7422	2474	45.8	*M. sphinx*
26	*Cercopithecinae* SIV	SIVdrl (D4)	KM378564	7386	2462	45.9	*M. leucophaeus*
27	*Cercopithecinae* SIV	SIVdrl (D3)	KM378563	7431	2477	45.3	*M. leucophaeus*
28	*Cercopithecinae* SIV	SIVmnd1 (GB1)	M27470	7464	2488	40.7	*M. sphinx*
29	*Cercopithecinae* SIV	SIVsun (lambda20L14/S2)	AF131870	7113	2371	44.7	*C. solatus*
30	*Cercopithecinae* SIV	SIVlhoest (7)	AF075269	6960	2320	43.4	*C. lhoesti*
31	*Cercopithecinae* SIV	SIVlhoest (524)	AF188116	7026	2342	43.5	*C. lhoesti*
32	*Cercopithecinae* SIV	SIVlhoest (485)	AF188115	7053	2351	42.9	*C. lhoesti*
33	*Cercopithecinae* SIV	SIVsyk (KE51)	AY523867	7185	2395	44.7	*C. albogularis*
34	*Cercopithecinae* SIV	SIVtal (00CM266)	AY655744	7197	2399	50.3	*M. ogouensis*
35	*Cercopithecinae* SIV	SIVdeb (CM40)	AY523865	7326	2442	45.7	*C. neglectus*
36	*Cercopithecinae* SIV	SIVdeb (CM5)	AY523866	7269	2423	45.5	*C. neglectus*
37	*Cercopithecinae* SIV	SIVdeb (04CMPF3061)	FJ919724	7212	2404	46.1	*C. neglectus*
38	HIV-2	HIV-2 (Abt96)	AF208027	7281	2427	46.9	*H. sapiens*
39	HIV-2	HIV-2 (BEN)	M30502	7080	2360	47.1	*H. sapiens*
40	HIV-2	HIV-2 (ALI)	AF082339	7074	2358	45.2	*H. sapiens*
41	HIV-2	HIV-2 (EHO)	U27200	7065	2355	45.9	*H. sapiens*
42	*Homininae* SIV	SIVgor (BQID2)	KP004991	7530	2510	47.3	*Gorilla gorilla*
43	*Homininae* SIV	SIVgor (CP2139.287)	FJ424866	7494	2498	46.1	*Gorilla gorilla*

44	*Homininae* SIV	SIVgor (BPID15)	KP004990	7539	2513	46.8	*Gorilla gorilla*
45	*Homininae* SIV	SIVgor (BPID1)	KP004989	7614	2538	46.7	*Gorilla gorilla*
46	*Homininae* SIV	SIVgor (CP2135con)	FJ424863	7479	2493	46.5	*Gorilla gorilla*
47	*Homininae* SIV	SIVgor (CP2139.1con)	FJ424864	7494	2498	46.5	*Gorilla gorilla*
48	*Homininae* SIV	SIVgor (CP684con)	FJ424871	7500	2500	46.6	*Gorilla gorilla*
49	*Homininae* SIV	SIVcpz (UG38)	JN091690	7527	2509	45.8	*P. t. schweinfurthii*
50	*Homininae* SIV	SIVcpz (TAN1)	AF447763	7578	2526	44.2	*P. t. schweinfurthii*
51	*Homininae* SIV	SIVcpz (TAN13)	JQ768416	7611	2537	44.8	*P. t. schweinfurthii*
52	*Homininae* SIV	SIVcpz (BF1167)	JQ866001	7542	2514	42.6	*P. t. schweinfurthii*
53	*Homininae* SIV	SIVcpz (EK505)	DQ373065	7389	2463	43.2	*P. t. troglodytes*
54	*Homininae* SIV	SIVcpz (US.85.CPZUS)	AF103818	7356	2452	43.9	*P. t. troglodytes*
55	*Homininae* SIV	SIVcpz (DP943)	EF535993	7371	2457	44.3	*P. t. troglodytes*
56	*Homininae* SIV	SIVcpz (CAM13)	AY169968	7425	2475	43.2	*P. t. troglodytes*
57	*Homininae* SIV	SIVcpz (GAB2)	AF382828	7380	2460	43.9	*P. t. troglodytes*
58	*Homininae* SIV	SIVcpz (MB897)	EF535994	7338	2446	44.4	*P. t. troglodytes*
59	*Homininae* SIV	SIVcpz (MT145)	DQ373066	7338	2446	43.9	*P. t. troglodytes*
60	*Homininae* SIV	SIVcpz (LB7)	DQ373064	7356	2452	44.1	*P. t. troglodytes*
61	*Homininae* SIV	SIVcpz (LB715)	KP861923	7566	2522	43.3	*P. t. troglodytes*
62	HIV-1, group O	HIV-1 (O-99SE-MP1300)	AJ302647	7494	2498	43.6	*H. sapiens*
63	HIV-1, group O	HIV-1 (O-99SE-MP1299)	AJ302646	7548	2516	44.1	*H. sapiens*
64	HIV-1, group O	HIV-1 (O-98CMU2901)	AY169812	7479	2493	45.2	*H. sapiens*
65	HIV-1, group O	HIV-1 (O-9CM105)	AY169816	7449	2483	44.0	*H. sapiens*
66	HIV-1, group O	HIV-1 (O-LA30ORBF125)	KU168282	7521	2507	43.8	*H. sapiens*
67	HIV-1, group O	HIV-1 (O-97CMABB497)	AY169809	7509	2503	45.5	*H. sapiens*
68	HIV-1, group O	HIV-1 (O-LA31BCF108)	KU168283	7389	2463	43.8	*H. sapiens*
69	HIV-1, group O	HIV-1 (O-97CMABB447)	AY169813	7488	2496	43.2	*H. sapiens*
70	HIV-1, group O	HIV-1 (O-97US08692A)	AY169805	7551	2517	44.6	*H. sapiens*
71	HIV-1, group P	HIV-1 (P-U14788)	HQ179987	7557	2519	47.6	*H. sapiens*
72	HIV-1, group P	HIV-1 (P-RBF168)	GU111555	7521	2507	47.0	*H. sapiens*
73	HIV-1, group N	HIV-1 (N-04CM-1015-04)	DQ017382	7401	2467	43.0	*H. sapiens*
74	HIV-1, group N	HIV-1 (N-02CM-DJO0131)	AY532635	7434	2478	43.0	*H. sapiens*
75	HIV-1, group N	HIV-1 (N-YBF30)	AJ006022	7428	2476	43.0	*H. sapiens*
76	HIV-1, group N	HIV-1 (N-14003718)	MF767262	7425	2475	43.8	*H. sapiens*
77	HIV-1, group N	HIV-1 (N-S4858)	KY498771	7425	2475	43.1	*H. sapiens*
78	HIV-1, group N	HIV-1 (N-04CM-1131-03)	DQ017383	7434	2478	43.1	*H. sapiens*
79	HIV-1, group N	HIV-1 (N-CM_YBF106)	AJ271370	7404	2468	43.8	*H. sapiens*
80	HIV-1, group M	HIV-1 (M-CRF01-AETH04BKM)	DQ314732	7407	2469	43.6	*H. sapiens*
81	HIV-1, group M	HIV-1 (M-F-CM53657)	AF377956	7374	2458	43.2	*H. sapiens*
82	HIV-1, group M	HIV-1 (M-C-02ZMJM)	AB254156	7467	2489	42.0	*H. sapiens*
83	HIV-1, group M	HIV-1 (M-C-011BI)	MF373132	7482	2494	42.8	*H. sapiens*
84	HIV-1, group M	HIV-1 (M-C-DEMC00IN008)	KP109483	7476	2492	43.5	*H. sapiens*
85	HIV-1, group M	HIV-1 (M-C-DEMC96BW002	KY658708	7443	2481	42.2	*H. sapiens*
86	HIV-1, group M	HIV-1 (M-C-CH131_TF)	KC156114	7422	2474	42.9	*H. sapiens*
87	HIV-1, group M	HIV-1 (M-H-90CR056)	AF005496	7386	2462	43.0	*H. sapiens*
88	HIV-1, group M	HIV-1 (M-G-A1786)	FJ389367	7413	2471	42.7	*H. sapiens*
89	HIV-1, group M	HIV-1 (M-G-515-28)	FJ389365	7344	2448	46.1	*H. sapiens*
90	HIV-1, group M	HIV-1 (M-A-70641)	MH705151	7563	2521	43.2	*H. sapiens*
91	HIV-1, group M	HIV-1 (M-A1-DEMA109UG001)	KF716478	7446	2482	43.1	*H. sapiens*
92	HIV-1, group M	HIV-1 (M-D-92UG001)	AJ320484	7419	2473	42.8	*H. sapiens*
93	HIV-1, group M	HIV-1 (M-B-R8)	KT200357	7392	2464	43.0	*H. sapiens*
94	HIV-1, group M	HIV-1 (M-B-1057-01)	AY331292	7425	2475	43.7	*H. sapiens*
95	HIV-1, group M	HIV-1 (M-B-DEMB10TH002)	KP109514	7356	2452	42.9	*H. sapiens*
96	HIV-1, group M	HIV-1 (M-B-VH)	AF146728	7425	2475	43.3	*H. sapiens*

Supplementary File S1. Dataset of 23 genome sequences from virus species representative of the family *Retroviridae*: 20 from members of the *Orthoretrovirinae* subfamily (2 from each genus *Alpha*-, *Beta*-, *Gamma*-, *Delta*-, and *Epsilonretrovirus*, and 10 from genus *Lentivirus*) and 3 from members of the *Spumaretrovirinae* subfamily. From each genome sequence we selected the non-overlapping region that encodes the structural proteins Gag, Pol, and Env. Each nucleotide sequence contains the name of the virus species, the NCBI ac. number and the length of the coding region. The order of presentation of the sequences is as follows:

1. Avian leukosis virus (ALV), *Alpharetrovirus*
2. Rous sarcoma virus (RSV), *Alpharetrovirus*
3. Mouse mammary tumor virus (MMTV), *Betaretrovirus*
4. Mason-Pfizer monkey virus (M-PMV), *Betaretrovirus*
5. Human T-lymphotropic virus 1 (HTLV-1), *Deltaretrovirus*
6. Bovine leukemia virus (BLV), *Deltaretrovirus*
7. Walleye dermal sarcoma virus (WDSV), *Epsilonretrovirus*
8. Walleye epidermal hyperplasia virus type 1 (WEHV1), *Epsilonretrovirus*
9. Friend murine leukemia virus (FMLV), *Gammaretrovirus*
10. Feline leukemia virus (FLV), *Gammaretrovirus*
11. Bovine foamy virus (BFV), *Spumaretrovirinae*
12. Feline foamy virus (FFV), *Spumaretrovirinae*
13. Simian foamy virus human-isolate (SFV), *Spumaretrovirinae*
14. Bovine immunodeficiency virus (BIV), *Lentivirus*
15. Feline immunodeficiency virus (FIV), *Lentivirus*
16. Caprine arthritis encephalitis virus (CAEV), *Lentivirus*
17. Equine infectious anemia virus (EIAV), *Lentivirus*
18. Human immunodeficiency virus type 1 (HIV-1), *Lentivirus*
19. Human immunodeficiency virus type 2 (HIV-2), *Lentivirus*
20. Simian immunodeficiency virus (SIV), *Lentivirus*
21. Jembrana disease virus (JDV), *Lentivirus*
22. Puma lentivirus (PLV), *Lentivirus*
23. Visna/maedi virus (VMV), *Lentivirus*

```
>#1 Avian leukosis virus (ALV) Z46390 6306 nt
gtgatttcgtccgcgtgtaaaacctattgcggaaaaacctctccttctaagaaggaaatcggggccatgttgtccc
tcttacaaaaggaagggttgcttatgtctccctcagacttatactccccggggtcctgggatcccatcactgcggc
gctctcccagcgggcaatggtacttggaaaatcgggggagttaaaaacctggggattggtgttgggggcattgaag
gcggctcgagaggaacaggttacatctgagcaagcaaagttttggttggggctaggggggagggagggtctctcccc
caggtccggagtgtatcgaaaaaccagcaacggagcggcggatcgacacaggggaggaagcgagagagacaactgt
gcagcaagatgcgaagatggcgccggaggaaacggccacacctaaaaccgttggcacatcctgctatcattgcgga
acagctattggctgtaattgcgccacagcctcggcccctcctcctccttatgtggggagtggtttgtatccttccc
tggcgggggtgggagagcagcagggccaggggggtgacacacctcgggggggcggaacagccaagggcggagccagg
gcacgcgggtctggcccctgggccggccctgactgactgggcaaggatcagggaggagcttgcgagtacaggtccg
cccgtggtggccatgcctgtagtgattaagacagagggacccgcctggacccctctggagccaaaattgatcacaa
gactggctgatacggtcaggaccaagggcttacgatccccgatcactatggcggaggtggaagcgcttatgtcctc
cccgctgctgccgcatgacgttacgaatctaatgagagttattttaggacctgccccatatgccttgtggatggac
gcttggggtgtccaactacagacggttatagcggcagccactcgcgacccccgacacccagcgaatggtcaagggc
gggggaacggactaatttggatcgcttaaagggtttagctgatgggatggtgggcaacccgcagggtcaggccgc
attattaagaccgggggaattggttgctattacggcgtcggctctccaggcatttagagaggttgcccggctggcg
gaacctgctggtccatgggcggacattacgcagggaccatctgagtcctttgttgattttgccaatcggcttataa
aggcggttgaggggtcagatctcccgccttccgcgcgagctccggtgatcattgactgctttaggcagaagtcaca
gccagatatccagcagcttatacgggcagcaccctccacgctgaccaccccaggagagataatcaaatatgtgcta
gacaggcagaagactgcccctcttacggatcaaggcatagccgcggccatgtcgtctgctatccagcccttagtta
```

tggcagtagtcaatagagagagggatggacaaactgggtcgggtggtcgtgcccgagggctctgctacacttgtgg
atccccgggacattatcaggcgcagtgcccgaaaaaacgaaaatcaggaaacagccgtgagcgatgtcagttgtgt
gacgggatgggacacaacgctagacagtgtaggaagcgggatggcaaccagggccaacgcccaggaagaggtctct
cttcggggccgtggcccgcccctgagccgcctgccgtctcgttagcgatgacaatggaacatagagatcgcccctt
ggttagggtcattctgactaacactgggagtcatccggtcaaacagcgctcggtgtatatcaccgcgctgttggac
tccggagcggacatcactattatttcggaggaggactggcctactgattggccggtggtggacaccgcgaacccac
agatccatggcataggaggggaattcccatgcgaaaatctcgtgacatgatagagttaggggttattaaccgaga
cgggtctttggagcgacccctgctcctcttccccgcagtagctatggttagagggagtatcctgggaagagattgt
ctgcagggcctagggctccgcttgacaaatttatagactgttgcgctacatctggctattccgctcaaatggaagc
cagaccacacgcctgtgtggattgaccagtggccccttcctgagggtaaacttgtagcgctaacgcaattagtgga
aaaagaattacagttaggacatatagaaccttcacttagttgttggaacacacctgtctttgtgatccggaaggct
tctgggtcttatcgtttattgcatgacttgcgcgctgttaacgccaagcttgttccttttggggccgtccaacagg
gggcgccagttctctccgcgctcccggctggctggcccctgatggtcctagacctcaaggattgctttttttctat
tcctcttgcggaacaagatcgcgaagcttttgcatttacgctcccctctgtgaataaccaggcccccgctcgaaga
ttccaatggaaggtcttgccccaagggatgacctgttctcccactatctgtcagttgatagtgggtcaggtacttg
agcccttgcgactcaagcacccatctctgcgcatgttgcattacatggatgatcttttgctagccgcctcaagtca
tgacaggttggaagcggcaggggaggaggttattagtacactggaaagagccgggttcaccatttcgcctgataag
gtccaaagggagcccggagtacaatatcttgggtacaagttaggcagtacgtatgtagcacccgtaggtctggtag
cagaacccaggatagccaccttgtgggatgttcaaaagctggtgggatcacttcagtggcttcgccctgcattagg
aatcccgccacgactgatggccccttttatgagcagttgcgggggtcagaccctaacgaggcgagggaatggaat
ctagacatgaaaatggcctggagagagattgtacagctcagcaccactgctgccttggaacgatggaccctgccc
tgcctctggaaggagcggtcgctagatgtgaacaggggcaataggggtcctgggacagggactgtccacacaccc
aaggccatgcttgtggttattctccacccaacccaccaaggcgtttactgcttggttagaagtgctcacccttctg
attactaagctacgtgcttcggcagtgcggacctttggcaaagaggttgatatcctcctgttgcctgcatgctttc
gggaggaccttccgctgccggaggggatcctgctagcccttaagggatttgcaggaaaaatcaggagtagtgatac
gccatctatctttgacattgcgcgtccactgcatgtttctctgaaagtgagggttaccgaccaccctgtgctagga
cccactgtctttactgacgcctcctcaagcacccataagggggtggtagtctggagggagggcccaaggtgggaga
taaaagaaatagctgatttgggagcaagtgtacaacaactggaggcacgcgctgtggccatggcacttctgctgtg
gccgacaacgcccacgaatgtagtgactgactccgcgtttgttgcgaaaatgctactcaagatgggacaggaggga
gtcccgtctacagcggcggcttttattttagaggatgcgttaagccaaaggtcagccatggccgccgttctccacg
tgcggagtcattctgaagtgccagggtttttcacggaaggtaatgacgtggcagatagccaagccacctttcaagc
gtatcccttgagagaggctaaagatcttcataccgctctccatattggaccccgcgcgctatctaaagcgtgtaat
atatctatgcagcaggctagggaggttgttcagacctgcccgcattgtaattcagcccctgcgttggaggccgggg
taaaccctaggggtttgggaccccctacagatatggcagacagactttacgcttgagcctagaatggcccccccgttc
ctggcttgctgttactgtggataccgcctcatcagcgatagtcgtaactcagcatggccgtgtcacatcggttgct
gcacaacatcattgggccacggctatcgccgttttgggaagaccaaaggccataaaaacagataatgggtcctgct
tcacgtctaaatccacgcgagagtggctcgcgagatgggggatagcacacaccaccgggattccaggtaattccca
gggtcaagctatggtagagcgggccaaccggctcctgaaggataagatccgtgtgcttgcggaggggggacggcttt
atgaaaagaatccccaccagcaaacaggggaactactagccaaggcaatgtatgccctcaatcactttgagcgtg
gtgaaaacacaaaaacaccaatacaaaaacactggagacctaccgttcttacagaaggacccccggttaagatacg
aatagagacaggggagtgggaaaaaggatggaacgtgctagtctggggacgaggttatgccgctgtgaaaaacagg
gacactgataaggttatttgggtaccttctcggaaagttaaaccggacatcacccaaaaggatgaggtgactaaga
aagatgaggcgagccctctctttgcaaagaagccgccagcaacaagcaagaaagacccggagaagacacccttgct
gccatcgagaggttacttcttctttcaaatgatacttgtgtgcgtggttattatttccgttgtcccaggggtgggg
ggagttcatctattgcaacaaccaggaaacgtgtgggtcacctgggcaaataagacggccgaacagattttttgcc
ttagtctacagtcagcgacctcaccattccgcacctgcttgataggcattccacagtatcctctgaacacctttaa
gggatatgtcactaatgttactgcttgcgataacaacaccgatttagccagccaaacagcatgcttgataaaggct
ctaaatacaaccctcccttgggacccccaagaattggatattttagggtcccagatgatcaagaacggaacaacac
gtacgtgtgttacctttggttcggtgtgctataaagagaacaatcgcagtagagtctgtcacaattttgatggaa
tgttaatgggactggtggggcggaacgagagttgcgtgacttcatagcaaaatggaaaagtgatgaccttcttata
aggccctatgtcaaccaatcatggacgatggtaagtccaataaacgtagagagtttttcaataagtcgtagatatt
gtggattcaccagtaacgagactcgttactatagaggggacctttctaattggtgtagttcaaaaaggggagaatg
gtcagcggggtacagcaacgggacaaaatgttccagcaacacgacgggttgtggtggtaattgcacaacggaatgg
aattattatgcatatgggtttaccttcggggaacagccagaggtgttgtggaacaatgggactgctaaggcactcc
caccaggtattttcttgatttgtggggacagggcttggcaaggcatcccgcgtaatgccttgggagggccctgtta
tctaggacaattgactatgctctctcctaactttaccacctggataacatatgggccgaacattacgggtcaccac
cgtagcaggcgctcgctgagtcgtctctcgcctgactgcggtgatgagctacagctatggagtgtgacagcccgga
tatttgcttctttctttgctcctggtatagcagcagcacaggccttaaaggagatcgagcgattggcatgttggtc
ggttaagcaagcgaatttaacatcattaatattgaatgcgatgctggaggatacaagcagtatccggcacgcagtg
ttgcagaatcgagcagccatcgatttcttactcctggcgcagggacacgggtgtcaagacgtagaaggatgtgct

gcttcaatctcagcgatcatagtgagtccattcataaggcgctccaagccatgaaggaacatacagagaagatacg
ggtggaagatgatcccataggggattggtttacgcgcacgtttggtgatctaggaaggtggctcgcgaaaggtgtt
aagacgctactgtttgccttgcttgtcatagcctgtctattagctatcattccatgtgtaatcaagtgctttcagg
attgtctatcgagaacaatgaatcagtttatggatgaacgcataagatatcatagaattagggagcagctgtag
>#2 Rous sarcoma virus (RSV) AF052428 6372 nt
gtgatttcgtccgcgtgtaagacctattgcgggaaaacctctccttctaagaaggaaataggggctatgttgtccc
tgttacaaaaggaagggttgcttatgtctccctcagacttatattccccggggtcctgggatcccattaccgcggc
gctctcccagcgggcaatggtacttggaaaatcggagagttaaaaacctggggattggttttggggggcattgaag
gcggctcgagaggaacaggttacatctgagcaagcaaagttttggttgggattagggggagggagggtctctcccc
caggtccggagtgcatcgagaaaccagctacggagcggcgaatcgacaaaggggaggaggtgggagaaacaactgt
gcagcgagatgcaaagatggcgccggaggaaacggccacacctaaaaccgttggcacatcctgctatcattgcgga
acagctattggctgtaattgcgccacagcctcggcccctcctcctccttacgtggggagtggtttgtatccttccc
tggcgggggtgggagagcagcagggccaggggggtgacacacctcggggggcggaacagccaagggcggagccagg
gcacgcgggtctggcccctgggccggccctgactgactgggcaaggatcagggaggagcttgcgagtacaggtccg
cccgtggtggccatgcctgtagtgattaagacagagggacccgcttggacccctctggagccaaaattgatcacaa
gactggctgatacggtcaggaccaagggcttacgatccccgatcactatggcagaagttgaagcgcttatgtcttc
cccgctgctgccgcatgacgttacgaatctaatgagagttattttaggacctgccccatatgccttatggatggac
gcttggggagtccaactacagacggttatagcggcagccactcgcgacccccgacatccagcgaacggtcaagggc
gggggaacggactaacttggatcgcttaaagggcttagctgatgggatggtgggcaacccacagggtcaggccgc
attattgagaccggggaattggttgctattacggcgtcggctctccaggcgtttagagaggttgcccgattggcg
gaacctgcaggtccatgggcggacatcacgcagggaccatctgagtcctttgttgattttgccaatcggcttataa
aggcggttgaggggtcagatctcccgccttccgcacggctccggtgatcattgactgctttaggcagaagtcaca
gccagatattcagcagcttatacgggcagcaccctctacgctgaccaccccaggagagataatcaaatatgtgcta
gacaggcagaagattgcccctcttacggatcagggcatagctgcggccatgtcgtctgctattcagcccttagtta
tggcagtagtcaatagagagagggacggacaaactgggtcgggtggtcgtgcccgaaggctctgctacacttgtgg
atccccgggacattatcaggcgcagtgcccgaaaaaacgaaagtcaggaaacagccgtgagcgatgtcagctgtgt
gacgggatggggcacaacgctaaacagtgtaggagacgggatagcaaccagggacagcgcccaggaagaggcctct
cttcggggccatggcctgtctctgagcagcctgctgtttcgttagcgatgacaatggaacataaagatcgcccctt
ggttagggtcatcctgactaacactgggagtcacccggtcaaacagcgttcggtgtatatcaccgcgctgttggac
tctggagcggacatcactattatttcagaggaggactggcctacggattggccggtggtggataccgcgaacccac
agattcacggcataggaggaggaattcccatgcgaaagtctcgggacatgatagagttgggggttattaaccgaga
cgggtcgttggagcgacccctgctccttttccccgccgtagctatggttaggggagtatcctaggaagagattgt
ctgcagggcctagggctccgcttgacaaatttgtagactgttgcgctacatctggctattccgctcaaatggaagc
cagaccacacgcctgtgtggattgaccagtggccccttcctgaaggtaaacttgtagcgctaacgcaattagtgga
aaaagaattacagttaggacatatagaaccctcacttagctgttggaacacacctgtctttgtgatccggaaggct
tccgggtcttatcgcttattgcatgacttacgcgctgttaacgccaagcttgttccttttgggctgtccaacagg
gggcgccagttctctccgcgctcccgcgtggctggcccctgatggttctagacctcaaggattgcttcttttctat
tcctcttgcggaacaagatcgcgaggcttttgcatttacgctcccctctgtgaataaccaggcccccgctcgaaga
ttccaatggaaggtcttgccccaagggatgacctgttctcccactatctgtcagttggtagtgggtcaggtacttg
agcccttgcgactcaagcacccatctttgcgcatgttgcattatatggatgatcttttgctagccgcctcaagtca
tgatgggttggaagcggcgggggaagaggttatcaatacattggaaagagccgggttcaccatttcgcctgataag
atccagagggagcccggagtacaatatcttgggtacaagttaggcagtacgtatgtagcacccgtaggcctggtag
cagaacccaggatagccaccttgtgggatgttcaaaagctggtggggtcacttcagtggcttcgcccagcgttagg
aatcccgccacgactgatgggcccctttatgagcagttacgagggtcagatcctaacgaggcgagggaatggaat
ctagacatgaaaatggcctggagagagatcgtacagctcagcaccacggctgccttggaacgatggaccctgccc
tgcctctggaaggagcggtcgttagatgtgaacaggggcaataggggtcctgggacagggactgtccacacaccc
aaggccatgtttgtggttattctccacccaacccaccaaggcgtttactgcttggttagaagtgctcaccctttg
attactaagctacgcgcttcggcagtgcgaacctttggcaaggaggttgatatcctcctgttgcctgcatgcttcc
gggaggaccttccgctcccggaggggatcctgttagcccttaggggtttgcaggaaaatcaggagtagtgacac
gccatctattttgacatagcgcgtccactgcatgtttctctgaaagtgagggttaccgaccaccctgtaccggga
cccactgtctttactgacgcctcctcaagcacccataagggggtggtagtctggagggagggcccaaggtgggaga
taaaagaaatagctgatttgggagcaagtgtacaacaactggaagcacgcgctgtggccatggcacttctgctgtg
gccgacaacgcccactaatgtagtgactgactccgcgtttgttgcgaaaatgttactcaagatggggcaggaggga
gtcccgtctacagcggcggcctttattttagaggatgcgttaagccaaaggtcagccatggccgccgttctccacg
tgcggagtcattctgaagtgccagggtttttcacagaaggaaatgacgtggcagatagtcaagccacctttcaagc
gtatcccttgagagaggctaaagatcttcatactactctccatattggaccccgcgcgctatccaaagcgtgtaat
atatctatgcagcaggctagggaggttgttcagacctgcccgcattgtaattcagcccctgcgttggaggccgggg
taaatcctaggggtttggacccctacagatatggcagacagactttacgcttgagcctagaatggcccccgttc
ctggctcgctgtcactgtggataccgcctcatcggcgatagtcgtaactcagcatggccgtgtcacatcggttgct
gcacaacatcattgggccacggctatcgccgttttgggaagaccaaaggccataaaaacagataacgggtcctgct

tcacgtctaaatccacgcgggagtggctcgcgagatgggggatagcacacaccaccgggattccgggaaattccca
gggtcaagctatggtagagcgggccaaccggctcctgaaagataagatccgtgtgcttgcggaaggggacggcttt
atgaaaagaatccccgccagcaaacaggggaactactagccaaagcaatgtatgccctcaatcactttgagcgtg
gtgaaaacacgaaaacaccggtacaaaaacactggagacctaccgttcttacagaaggacccccggttaaaatacg
aatagagacaggggagtgggaaaaaggatggaacgtgctagtctggggacgaggttatgccgctgtgaaaaacagg
gacactgataaggttatttgggtaccctctcgaaaggttaaaccggacatcacccaaaaggatgaggtgactaaga
aagatgaggcgagccctctttttgcatatattctcattattggtgtcctggtcttgtgtgaggttacgggggtaag
agctgatgttcacttactcgagcagccggggaacctttggattacatgggccagccgtacaggccaaacggatttc
tgcctttctacacagtcagccacctccccttttcaaacatgtttgataggtatcccgtcccctatttctgagggtg
attttaagggatatgtctctgataattgcaccactttggaacctcaccggttagtctcgagaggcattcctggcgg
ccctgagaacagcacaaccctcacctatcagaaggtttcatgcttgttgttaaagctgaatgtttctctgttggac
gagccatcagaactacaactgctaggttcccagtctctccccaatataactaatattactcggatccccagtgtgg
ctgggggatgcataggctttaccccatacgatagtccggctggtgtctacggatgggaccggagagaggttacaca
catccttctgaccgacccagggaacaatcctttctttgataaggcctctaactcctcgaaaccgtttacagtagtg
acagcggacaggcacaatctctttatggggagtgagtactgtggtgcatatggctacaggttctgggaaatgtaca
attgctcccaaatgagacagaattggtccatctgtcaggatgtgtggggccgaggcccccccgaaaattggtgcac
aagcacaggaggtacatgggttaatcaatcaaaggaatttaatgagacagagccattcagttttactgtgaactgt
actggcagtaatttgggtaatgtcagcggatgttgcggagaaccaatcacgattctcccaccagaggcgtgggtcg
acagcacacaaggtagtttcactaaaccaaaagcgctaccacccgcaattttcctcatttgtggagatcgcgcatg
gcaaggaattcccagtcgtccggtagggggcccctgctatttaggcaagctcaccatgttagcacccaaccataca
gatattctcaaaattcttgctaattcatcgcggacaggaataagacgtaaacgaagcgtctcacacctggatgata
catgctcagatgaagtacagctttggggtcctacagcaagaatctttgcgtctatcttagccccggggtagcagc
tgcacaagccttaaaagaaatcgagagactagcctgttggtccgttaaacaggctaacttgacaacatcactcctc
ggggacttattggatgatgtcacgagtattcgacacgcggtcctgcagaaccgagcggctattgatttcttgctcc
tagctcacggccatggctgtgaggacgttgctggaatgtgctgtttcaatttgagtgatcacagtgagtctataca
gaagaagttccagctaatgaaggaacatgtcaataagatcggcgtggacagcgacccaattggaagttggctgcga
ggactattcgggggaataggagaatggccgttcatttgctgaaaggactgcttttggggcttgtagttattttgt
tgctagtagtgtgcctgccttgccttttgcaaatcgtatgcggtaacatcagaaagatgattaataactccatcag
ctaccacacggaatataagaagctgcaaaaggcctatgggcagcctgaaagcagaatagtataa
>#3 Mouse mammary tumor virus (MMTV) M15122 6897 nt
atgggggtctcgggatcaaaagggcagaaactctttgtttctgttctacaaagactcctctcagagaggggtcttc
atgtgaaagagagcagtgcaatagagttttatcagttcctaataaaggtctctccttggtttcccgaagaaggagg
attaaatttacaagattggaaaagggtgggaagagagatgaagaggtacgcagcagaacatgggacggatagtata
ccaaagcaggcttaccccatttggcttcagttgagagagatactgacagagcaatcagacttggttttgttatctg
cagaagccaagtccgtgactgaagaggaattagaggaaggtttaaccggactactatcgacaagttcacaggaaaa
aacttatgggactaggggaacagcatatgcagaaatagatacagaggtagacaagctgtctgaacatatttatgat
gaaccatatgaagaaaaggagaaggcagataaaaatgaggaaaaggaccatgttagaaaaataaagaaggtagtac
aaagaaaagaaaatagtgagggtaagagaaaagagaaggattcaaaggcctttttagccacagattggaacgatga
tgacctgtcccctgaggattgggacgatttagaggaacaagcggcacattatcatgatgatgatgagctaatcctt
ccagtaaaaaggaaggtggttaagaagaaacctcaggcactcagaaggaaacccctgcctccggtgggttttgcag
gagcgatggcagaggccagggaaaaaggagatttgacttttacgtttcctgtagtttttatgggagagagtgatga
agatgacacgcctgtttgggaaccgctgccattgaaaaccttaaaggaattgcaatcggcagttaggaccatggga
ccatctgctccctacaccctgcaggtggtagacatggtggctagtcaatggctcaccccgagtgactggcaccaaa
cagccagagctaccttgtctcctggagactatgttttatggagaactgaatatgaagagaaaagtaaagaaatggt
acaaaaggctgcaggcaagcgaaagggcaaggtctctcttgatatgttactggggactggccaattcctgtcccct
tcttctcagataaaattgtctaaggatgtcttaaaagatgtcaccacaaatgctgtgttagcatggaggccattc
cgcctcctggagttaaaaagactgtattagcaggattaaaacagggaaatgaagagtcttatgagactttcatttc
aaggctcgaggaagctgtttaccgaatgatgcccagaggggaagggtcggatatattgatcaaacaattggcgtgg
gagaatgcaaattcattgtgtcaggatctcatccgcccaatacgtaaaacaggaactatacaggattatattcgtg
cttgtctggacgcttctcccgcagttgttcagggtatggcatatgcagcagccatgagaggacaaaagtattctac
ctttgtaaagcaaacatatggtgggggaaaaggaggtcaaggagcagaagggccagtttgtttttcctgtggtaag
acaggacacatcagaaaagactgtaaggatgaaaagggctcaaaaagggcccctcctgggctctgccccagatgta
agaaaggctatcactggaagagtgagtgtaaatctaaatttgacaaagatgggaatccacttcctcccttggaaac
taatgctaaattcaaaaaacttgtaaagggcagtcccctagccccgctcaaaaggggggatggagttaagggctca
ggattaaatcctgaagccccacctttcacaatacatgatttgcctcgaggcacccctggaagtgcaggtttagacc
tgtcatcacagaaggatttgatcctctctctagaagatggagtatcattggtacccaccttagtgaaaggcaccct
ccctgaagggactactggactaataataggtagaagttctaattataaaaagggacttgaggttttaccaggagtc
attgactccgatttccaaggagaaatcaaggttatggttaaggccgcaaaaaatgcggtcatcattcacaaaggag
aaagaatagcacaactgctgttgctgccatatttaaaattacccaatcctgtaatcaaggaagaacgaggctcaga
aggcttcggatcaacaagtcatgtgcattgggtgcaggaaataagtgactccagacccatgcttcacatttactta

aatggaagaagattcctcggtctcttggataccggggcagataaaacttgcatagcaggcagagactggccagcta
attggcccattcaccaaactgagagttctcttcaaggtttaggcatggcctgtggggtggcgcgtagtagtcagcc
actccgttggcaacatgaggataaatcaggaattatacatccttttgtgatccctacactgcctttcaccttatgg
ggaagagatattatgaaagatataaaggtcagattgatgacagactcaccagatttcacaggatttatgatagggg
ccattgagagcaatctctttgcagaccaaatatcttggaagtcagaccagcctgtatggcttaatcaatggcccct
taaacaagaaaagttacaggctttacaacagttagtgacagaacaattacaactgggccacttagaagagagcaat
agcccttggaatacgcctgtttttgtcattaaaaagaagtcaggaaaatggagactgttacaagacctacgtgcag
ttaatgctacaatgcacgatatgggagcattacaacccggcttgccgtcccctgtagcagtccctaaaggatggga
aataatcataatagatctacaagattgctttttaatataaactgcatcctgaagattgtaaaagatttgctttt
agtgtgccctcccctaattttaagagaccttatcaaagattccaatggaaagttttgccccagggtatgaaaaata
gccctactttatgtcaaaaatttgtggacaaagctatattgactgtaagggataaataccaagactcatatattgt
gcattacatggatgacattcttttggcacacccatcaagatccattgtcgatgaaatacttacttccatgatacag
gcccttaacaaacatggccttgtagtatccacagagaagattcaaaaatatgataatctcaaatatttgggaactc
atatacagggtgattcagtgtcttatcaaaaattacagattaggacagataaattaagaaccttaaatgatttcca
aaagctattaggaaatattaattggatacgtcctttcttaaaattaactacgggagagttaaaacctctctttgaa
attcttaatggagattctaatccgatctcaacaagaaaacttactcctgaggcatgcaaagctcttcaattaatga
atgagagactatctaccgctcgggtaaagaggctagatttatcacagccttggtctctatgtatattaaagactga
atataccccccacagcatgcctctggcaggatggagttgtagaatggatacatttgcctcatatttcaccaaaggtg
attactccttatgatatcttttgtacacaacttattattaagggccgacaccgctctaaagaattatttagtaaag
accctgattatattgttgtgccctacaccaaagttcaatttgatctcctattacaagaaaaggaagattggcctat
ttctttattagggttcttgggagaggttcatttccatcttccaaaagaccctttgcttacatttaccctacaaact
gccattatttttcctcacatgacctctaccacaccactagagaaaggaattgtgatttttacagacgggtcagcaa
atggccgttcggtaacatatatacaaggaagggagcctataattaaagaaaatacacaaaacacagcccaacaggc
tgaaattgtggcagtcattacagcctttgaggaagtgagtcaaccctttaatttgtatactgattctaaatatgtg
acagggttgtttcccgaaatcgaaactgcaactttgtcacccagaacaaaaatttacacagaactgaaacatttac
aaaggttaatccacaagagacaagaaaaattttacattggtcatatcagaggacacactggacttcccggtccttt
ggcacagggaaatgcctatgcagattctttaacaagaattctgaccgctttagagtcagctcaagaaagccacgca
ctacatcatcaaaatgccgcggcgcttaggtttcagtttcacatcactcgtgaacaagcgcgagaaatagtaaaat
tatgtcccaattgccccgactgggggcacgcgccgcaattaggggtaaaccccaggggccttaagccccgagttct
atggcaaatggatgttactcatgtttcagaatttggaaaattaaaatatgtacatgtgacagtggatacttattct
cattttactttcgctaccgcccgaacgggcgaagcaaccaaggatgtgttacaacacttggctcaaagctttgcat
acatgggcattcctcaaaaaataaaaacagataatgcccctgcatatgtgtctcgttcaatacaagaatttctggc
cagatggaaaatatctcacgtcacggggatcccctacaatccccaaggacaggccattgttgaacgaacgcaccaa
aatataaaggcacagcttaataaacttcaaaaggctggaaaatactatacaccccatcatctgttggcacacgctc
tttttgtgctgaatcatgtaaatatggacaatcaaggccatacagcggccgaaagacattggggtccaatctcagc
cgatccaaaacctatggtcatgtggaaagaccttctcacagggtcctggaaaggacccgatgtcctaataacagcc
ggacgaggctatgcttgtgtttttccacagacctcacaaactccccaaacctctttgaccttatttcttgctttgt
tgtctgtcctcggcccccgcctgtgacaggggagagttattgggcctacctacctaaaccacctattctccatcc
cgtgggatggggaagtacagaccccattagagttctgacaaatcaaaccatgtatttgggtggttcgcctgacttt
catgggttcagaaatatgtctggtaatgtacattttgaggggaagtctgatacgctccccatttgcctttccttct
ccttttctacccccacgggctgctttcaagtagacaagcaagtatttctttctgatacacccacggttgataataa
taaacctgggggaaagggtgataaaaggcgtatgtgggaactttggttgactaccttggggaactcaggggccaat
acaaaactggtccctataaaaaagaagttgcccccccaaatatcctcactgccagatcgcctttaagaaggacgcct
tctgggagggagacgagtctgctcctccacggtggttgccttgcgccttccctgaccaggggggtgagtttttctcc
aaaaggggcccttgggttactttgggatttctcccttccctcgcctagtgtagatcagtcagatcagattaaaagc
aaaaagaatctatttggaaattatactcccccctgtcaataaagaggttcatcgatggtatgaagcaggatgggtag
aacctacttggttctgggaaaattctcctaaggatcccaatgatagagatttcactgcactagtcccccatacaga
attgtttcgcttagtcgcagcctcaagacatcttattctcaaaaggccaggatttcaagaacatgagatgattcct
acatctgcctgtgttacttacccttatgccatattattaggattacctcagttaatagatatagagaaaagaggat
ctacttttcatatttcctgttcttcttgtagattgactaattgtttagactcttctgcctacgactatgcagcgat
catagtcaagaggccgccatatgtgctgctacctgtagatattggtgatgaaccatggtttgatgattctgccatt
caaacctttaggtatgccacagatttaattcgagctaagcgattcgtcgcagccattatcctgggcatatctgctt
tgattgctattattacttcctttgctgtagctactactgccttagttaaggagatgcaaactgctacgtttgttaa
taatcttcataggaatgttacattagccttatctgaacaacggataatagatttaaaattagaagctagacttaat
gctttagaagaagtagttttagagttgggacaagatgtggccaatttaaagaccagaatgtccactaggtgtcatg
caaattatgactttatctgcgttacacctttaccctataatgctactgagaactgggaaagaaccagggctcattt
attgggcatttggaatgataatgagatttcatataacatacaggagttaaccaacctgattagtgatatgagcaaa
caacatattgatgcagtggaccttagtggcttggctcagtcttttgccaatggagtgaaggctttaaatccattag
attggacacaatatttcatttttataggtgttggagccctgcttttagtcattgtacttatgattttccccattgt

tttccagtgccttgcgaagagccttgaccaagtgcagtcagatcttaacgtgcttcttttaaaaaagaaaaaaggg
ggaaatgccgcgcctgcagcagaaatggttgaactcccgagagtgtcctacacttag
>#4 Mason-Pfizer monkey virus (M-PMV) M12349 6339 nt
atggggcaagaattaagccagcatgaacgttatgtagaacaattgaagcaggctttaaagacacggggagtaaagg
ttaaatatgctgatcttttgaaatttttgattttgtgaaggatacttgtccttggtttccgcaagagggaaccat
agatattaaacggtggcgtagagtaggcgactgtttccaagactattacaatactttggcccggagaaagtccca
gtaactgcattctcttactggaacttaattaaagaattgatagataagaaagaagttaacccacaagtaatggctg
ccgtagcccaaactgaagaaattttaaaaagtaattctcaaacagacctcacaaagacctctcaaaacccagactt
ggaccttatttcccttgatagcgacgatgaaggagctaaaagttcctctctacaagataaaggtttatcaagtact
aaaaaaccaaaaagattcccagttctgttaacagcacaaactagtaaagaccctgaagaccccaacccctcagagg
tagactgggacggcttggaagatgaggcagcgaaatatcataatcccgattggcctcccttcctaacccgtccacc
tccttacaataaagctactccttccgcacccactgtaatggcggttgttaatccaaaagaggagctcaaagaaaaa
attgctcaattagaggaacagattaaattagaagagttacaccaggcactaatttccaaattacaaaaactaaaaa
cagggaatgaaactgtaactcacccagacacagcaggaggcctttctcgcacgcctcactggccagggcaacatat
ccctaaaggaaaatgttgcgccagtcgagaaaaggaagaacaaatcccaaaagatattttcccagtgactgaaacc
gttgatgggcaaggtcaagcctggagacaccataatggttttgattttgccgtcataaaagaattaaaaacagctg
cttcccaatatgggctactgccccatacacattagccatagtggaatctgtagcggacaattggcttacccctac
agattggaatacgcttgttagggcagtcctctcaggaggagatcacttactgtggaaatctgagtttttgaaaat
tgcagagatacggctaaaagaaaccaacaagccggtaatggctgggattttgacatgttaacaggttcgggtaatt
attccagcaccgatgcacaaatgcagtatgatccaggattgtttgctcaaattcaagcggctgctacaaaagcctg
gagaaaacttcccgttaagggagacccaggagcctcccttacaggagtcaaacaaggacccgatgagccatttgca
gatttcgtacacagacttataacaactgctgggagaatctttggaagtgctgaggccggtgtagactatgtaaaac
aactagcatatgaaaatgctaatccagcttgtcaggcagccattcgcccctatagaaagaagacagatttaactgg
ctatatccgtctttgctcggatattgggccctcttatcagcaaggcctggccatggccgccgcctttagcgggcag
actgtaaaagatttcttaacaacaaaaataaagagaaaggagggtgttgctttaaatgcggtaaaaaaggacact
ttgcaaaaaattgtcatgaacatgcacataacaatgctgaaccaaaagttcccggactctgccctagatgtaaaag
agggaaacattgggccaatgaatgcaaatccaaaactgataatcaaggaaacccaataccaccccatcagggaaac
gggtggagggccagccccaggccccgaaacaagcttatggggcagtcagctttgttccagccaacaaaaacaacc
catttcaaagcttaccagagccaccccaggaagtgcaggattggacctctgttccacctcccacacagtattaaca
aaaaaggttttggaaatttttaactgcggccattgacatacttgcaccccaacagtgcgctgaacccatcacgtgg
aaatcagacgaacctgtctgggttgatcagtggccattaaccaatgacaaacttgctgctgcccaacagttagtgc
aagaacagttagaggcaggacatattactgaaagtagttctccctggaacactcccatatttgttataaaaaagaa
atctggtaaatggaggctcttacaagatttacgagccgttaatgccactatggtattaatgggagctttacaacct
ggattaccctccccggtggctatcccacaagggtatcttaaaataattattgatctcaaggattgtttcttttcta
ttccccttcatcctagtgaccaaaaaagatttgccttcagcctaccatccacaaattttaaagaacctatgcaacg
ttttcagtggaaggttttaccacaaggtatggccaacagtcctaccttatgtcaaaaatatgtggccacagccata
cataaggttagacatgcctggaaacaaatgtatattatacattacatggatgacatcctaatagctggtaaagatg
gacaacaagttttgcaatgctttgatcaactcaaacaagagttgactgcagctgggttacatatagccccagaaaa
agttcaattacaagatccctacacatatttaggatttgaacttaatggtcctaaaatcactaatcaaaaagcagtc
attcgtaaagataaattacagactcttaatgatttccaaaaacttttaggagacatcaattggctcagaccatatc
taaaactcactactggagacttaaaacccttattcgacacccttaaaggagactctgaccccaattcccatagatc
cttatcaaaagaagctcttgcctcacttgaaaaggtagagacagccattgcagaacaattcgttactcacataaat
tattcactaccattaatttcctcatattcaacacagccctgacacctactggtttgttttggcaagacaatccta
ttatgtggatccacctgcctgcatcccctaaaaaggtgttacttccctactacgacgctatagcagatttaatcat
actagggagagaccatagtaaaaaatattttggaattgaaccctctacaatcatacaaccatattctaagtctcag
attgattggttaatgcaaaacactgaaatgtggccaattgcctgtgcctcctttgttggcatcctagataaccatt
atccaccaaataaacttatccagttctgtaaactacatacctttgttttccctcaaatcattagtaaaacaccctt
aaacaatgccttattagtttttactgatggctcttccactgggatggccgcatatactcttactgataccaccatc
aaattccaaactaatcttaattcggctcaactagtagaactacaagctttaattgcagtcttatcagccttcccta
atcaacctcttaatatttacactgacagtgcttacttagcccactcaatacccctacttgaaactgtcgcacaaat
taaacacatatcagaaacagctaaactgttcctacagtgccagcaactcatatacaatagatccataccttttat
attggacatgtcagggcccattctggactacctggacccatagctcaaggcaaccaacgagctgacttggcaacta
aaatcgtggctagtaacataaacacaaacctcgaatcagctcaaaatgctcataccttacatcacctcaatgccca
gactttaagacttatgtttaacattcctagagaacaagctagacaaattgttaagcaatgtcctatatgtgtaact
tatctaccagtccctcatttaggagttaatcctaggggattatttcccaacatgatttggcaaatggatgttacac
actactcagaatttggcaatttaaaatatattcatgtatctatagatacattcagtggattcctactggccactct
acaaacaggagaaactacaaaacatgtcataacccatttactccattgcttctctattattggactccctaaacaa
attaaaacggataacggtcctggatacacctctaaaattttcaagaattttgctccacacttcaaattaaacata
ttactggaatcccctataaccccaaggccaaggaatagttgaaagagcccacttatctcttaaaccaccattga
aaagataaaaaaggggggaatggtaccctaggaagggtacccccaggaacatcctcaatcatgcactctttattcta

aatttttaaatttagatgatcaaaataaatcagcagctgatcgtttttggcataataaccccaaaaaacaatttg
ccatggtaaaatggaaagatccattagataatacatggcatggccccgatccagtgttaatttggggcagaggttc
agtctgtgtttactctcaaacctatgatgccgctagatggctaccagaacggttagtaagacaggtgtctaacaat
aaccaatccagggagtgaatgaacttcaattatcatttcatctggagcttagtgatactatctcaaatatctcaag
ttcaagccggttttggagatccgcgtgaagccctggcagaaatacaacaaaaacatggtaaaccttgtgactgtgc
tggaggatatgtttcctccccaccgattaactctcttacaactgtttcttgctctactcatactgcttattcagtg
acaaactccctaaaatggcagtgtgtgtcaactcccactacccctagcaatacacatataggaagttgtcccggtg
aatgcaacacgatctcatatgattctgtacatgcctcttgctataaccactatcaacaatgtaacattggtaataa
aacatatctcactgccactataactggagatagaactcctgccattggtgacgggaatgtccctacagtactaggg
actagtcacaacctcattacagcaggctgtcccaatggtaaaaagggccaagtggtctgttggaatagccgacctt
ctgttcatatatctgatggaggagggcctcaagataaggcccgcgacattatagtaaataaaaagtttgaggaatt
gcacaggtcgctgttcccagaactttcttaccatcctctggccttgcccgaagcccgtggtaaagaaaaaattgac
gcacacactcttgatctccttgccactgtacatagtttactcaatgcttcccaacccagtttagccgaagattgct
ggctgtgcttacagtcaggagatcccgttcctcttgccctgccctataatgatacactctgctctaactttgcctg
tttatctaatcactcctgccctttaacccccccttttttagtacagccctttaacttcactgattccaattgcctt
tacgctcattatcaaaacaactcatttgacatagatgtaggtctagctagctttactaattgctctagctattata
acgtttctacagcctccaaaccctctaattccctatgcgccccaaacagctcggtttttgtatgcggtaacaataa
ggcatacacttatctacccacaaattggacgggaagttgtgtacttgctactcttttgcccgatatagacatcatt
ccaggtagtgagcctgtccccattccagctattgatcatttttaggcaaagccaaaagagcaatccaacttatcc
ccctgttcgtagggttaggtataactactgcagtatctactggggctgctggtctaggggtttccatcactcaata
tacaaaattatctcatcaactaatatcagatgttcaagctatttctagcactatacaagatctccaagatcaggta
gactctctagcagaagtagtactgcaaaacagaagaggattagatctacttacagcagagcagggaggtatctgct
tagccttacaggaaaaatgttgtttctacgccaataaatctggaatcgtcagagacaagattaaaaacctacaaga
cgacttagaaagacgccgaagacaactgatcgacaacccattttggaccagttttcatggattcctcccttatgtt
atgcccctattaggccctttgctttgcttattgcttgtgttatctttcggtccaattattttcaacaagcttatga
cctttattaaacatcaaattgagagcatccaggccaaacctatacaagtccattatcatcgccttgaacaagaaga
cagtggtggctcatatttgaccttaacatag
>#5 Human T-lymphotropic virus 1 (HTLV-1) D13784 5325 nt
atgggccaaatcttttcccgtagcgctagccctattccgcggccgccccgggggctggccgctcatcactggctta
acttcctccaggcggcatatcgcctagaacccggtccctccagttacgatttccaccagttaaaaaaatttcttaa
aatagctttagaaacaccggtctggatctgccccattaactactccctcctagccagcctactcccaaaaggatac
cccggccgggtgaatgaaattttacacatactcatccaaacccaagcccagatcccgtcccgccccgcgccgccgc
cgccgtcatcctccacccacgacccccccggattctgacccacaaatccccccctccctatgttgagcctacagcccc
ccaagtccttccagtcatgcacccacatggtgcccctcccaaccaccgcccatggcaaatgaaagacctacaggcc
attaagcaagaagtctcccaagcggcccctggaagcccccagtttatgcagaccatccggcttgcggtgcagcagt
ttgaccccactgccaaagacctccaagacctcctgcagtacctttgctcctccctcgtggcttccctccatcacca
gcagctagatagccttatatcagaggccgaaactcgaggtattacaggttataaccccttagccggtcccctccgt
gtccaagccaacaatccacaacaacaaggattaaggcgagaataccagcaactctggctcgccgccttcgccgccc
tgccagggagtgccaaagacccttcctgggcctctatcctccaaggcctggaggagccttaccacgccttcgtaga
acgcctcaacatagctcttgacaatgggctgccagaaggcacgcccaaagaccccattttacgttccttagcctac
tctaatgcaaacaaagaatgccaaaaattactacaggcccgagggcacactaatagccctctaggagatatgttgc
gggcttgtcaggcctggacccccaaagacaaaaccaaagtgttagttgtccagccttcccccccacattacagca
agtccttcctaaccaagacccaacatctattctgccagttataccgttagatcccgcccgtcggcccgtaattaaa
gcccagattgacacccagaccagccacccaaagactatcgaagctctactagatacaggagcagacatgacagtcc
ttccgatagccttgttctcaagtaatactcccctcaaaaacacatccgtgttagggggcaggggggccaaacccaaga
tcactttaagctcacctcccttcctgtgctaatacgcctccctttccggacgacgcctattgttttaacatcttgc
ctagttgataccaaaaacaactgggccatcataggtcgtgatgccttacaacaatgccaaggcgtcctgtacctcc
ctatagatctctcatcatcttcccccgggccccctgacttgtccagcctgccaactacactagcccacttacaaac
tatagaccttaaagacgcctttttccaaatcccccctacctaaacagttccagccctactttgctttcactgtccca
cagcagtgtaactacggccccggcactagatacgcctggagagtactaccccaagggtttaaaaatagtcccaccc
tgttcgaaatgcagctggcccatatcctgcagcccattcggcaagccttcccccaatgcactattcttcagtacat
ggatgacattctcctggcaagcccctcccatgcggacctgcaactactctcagaggccacaatggcttccctaatc
tcccatgggttgcctgtgtccgaaaacaaaacccagcaaaccccctggaacaattaagttcctagggcaaataattt
cacctaatcacctcacttatgatgcagtccccaaggtacctatacggtcccgctgggcgctacctgaacttcaagc
cctacttggcgagattcagtgggtctccaaaggaactcctaccttacgccagccccttcacagtctctactgtgcc
ttacaaaggcatactgatccccgagaccaaatatatttaaatccttctcaagttcaatcattagtgcagctgcggc
aggccctgtcacagaactgccgcagtagactagtccaaaccctgcccctcctaggggctattatgctgaccctcac
tggcaccaccactgtggtgttccagtccaagcagcagtggccacttgtctggctacatgccccctaccccacact
agccagtgcccctgggggcagctacttgcctcagctgtgttattactcgacaaatacaccttgcaatcctatggac
tactctgccaaaccatacatcataacatctccacccaaaccttcaaccaattcattcaaacatctgaccaccccag

tgttcctatcttactccaccacagtcaccgattcaaaaatttaggtgcccagactggagaactttggaacactttt
cttaaaacaactgccccattggctcctgtgaaagcccttatgccagtgtttactctttcccctgtgatcataaaca
ccgccccttgcctgttttcagacggatccacctcccaggcagcctatattctctgggacaagcatatattgtcaca
aagatcattccccttccgccaccgcacaagtcggcccaacgggccgaacttctcggacttttgcatggcctctcc
agcgcccgttcgtggcgctgtctcaacatatttctagactccaagtatctttatcattaccttcggacccttgccc
taggcaccttccaaggcaggtcctctcaggccccctttcaggccctcctgccccgcttactatcgcgtaaggtcgt
ctatttgcaccacgttcgcagccataccaatctacctgatcccatctccaggctcaacgctctcacagatgcccta
ctaatcacccctgtcctgcagctctctcctgcagacctacacagtttcacccattgcggacagacggccctcacac
tgcaaggggcaaccacaactgaggcctccaatatcctgcgctcttgccacgcctgccgcaaaaataacccacaaca
tcagatgcctcaaggacacatccgccgtggcctactccctaaccacatctggcaaggcgacattacccatttcaaa
tataaaaatacactgtatcgccttcatgtatgggtagacaccttttcaggagccatctcagctacccaaaagagaa
aagaaacaagctcagaagctatttcctctttgctccaggccattgcctatctaggcaagcctagctacataaacac
agacaatggccctgcctatatttcccaagacttcctcaatatgtgtacctcccttgctattcgccatactacccat
gtcccctacaatccaaccagctccggacttgtagaacgctctaatggcattcttaaaccctattatataagtact
ttactgacaaacccgacctacctatggataatgctctatccatagccctatggacaatcaaccacctaaatgtatt
aaccaactgccacaaaacccgatggcagcttcaccactcccccgactccagccgatcccagagacacattccctc
agcaataaacaaacccattggtattatttcaagcttcctggtcttaatagccgccagtggaaaggaccacaggagg
ctcttcaagaagctgccggcgctgctctcatcccggtaagcgctagttctgcccagtggatcccgtggaggctcct
caagcgagctgcatgcccaagacccgtcggaggccccgccgatcccaaagaaaaagaccaccaacactttctcgcc
actttgattttattcttccagttctgcccccctcatcctcggtgattacagccccagctgctgtactctcacagttg
gagtctcctcataccactctaaaccctgcaatcctgcccagccagtttgttcatggaccctcgacctgctggccct
ttcagcagatcaggccctacagccaccctgccctaatctagtaagttactccagctaccatgccacctattccccta
tatctattccctcattggatcaaaaagccaaaccgaaatggcggaggctattattcagcctcttattcagaccctt
gttccttaaaatgcccatacctagggtgccaatcatggacctgcccctatacaggagccgtctccagcccctactg
gaaatttcagcaagatgtcaattttactcaagaagtttcacacctcaatattaatctccatttttcaaaatgcggt
ttttccttctcccttctagtcgacgctccaggatatgaccccatctggttccttaataccgaacccagccaactgc
ctcccaccgcccctcctctactctcccactctaacctagaccatatcctcgagccctctataccatggaaatcaaa
actcctgactcttgtccagttaaccctacaaagcactaattatacttgcattgtctgtatcgatcgtgccagccta
tccacttggcacgtcctatactctcccaacgtctctgttccatccccttcttctacccccctcctttacccatcgt
tagcgcttccagccccccacctgacgttaccatttaactggacccactgctttgacccccagattcaagctatagt
ctcctccccctgtcataactccctcatcctgccccccttttccttgtcacctgttcccacgctaggatcccgctcc
cgccgagcagtaccggtggcggtctggcttgtctccgccctggccatgggagccggagtggctggcaggattaccg
gctccatgtccctcgcctcaggaaagagcctcctacatgaggtggacaaagatatttcccaattaactcaagcaat
agtcaaaaaccacaaaaatctgctcaaaattgcacagtatgctgcccagaacagacgaggccttgatctcctgttc
tgggagcaaggaggattatgcaaagcattacaagaacagtgctgttttctaaatattactaattcccatgtctcaa
tactacaagagagaccccccttgaaaatcgagtcctgactggctggggccttaactggaccttggcctctcaca
gtgggctcgagaagccttacaaactggaatcacccttgtcgcgctactccttcttgttatccttgcaggaccatgc
atcctccgtcagctacgacacctcccctcgcgcgtcagatacccccattactctcttataaaccctgagtcatccc
tgtaa
>#6 Bovine leukemia virus (BLV) M32690 5022 nt
atgggaaattcccctcctataaccccccgctggtatctcccccctcagactggctcaaccttctgcaaagcgcgc
aaaggctcaatccgcgaccctctcctagcgattttaccgatttaaagaattacatccattggtttcataagaccca
gaaaaaaccatggactttcacttctggtggccccacctcatgtccacccgggagattcggccgggttcccctcgtc
ttggccaccctaaacgaagtactctcaaacgaagggggcgccccgggtgcatcggccccagaagaacaaccccccc
cttatgaccccccgccattttgccaatcatatctgaagggaatcgcaaccgccatcgtgcttgggcactccgaga
attacaagatatcaaaaaagaaattgaaaataaggcaccgggttcgcaagtatggatacaaacactacgacttgca
atcctgcaggccgaccctactccggctgacctagaacaactttgccaatatattgcttccccggtcgaccaaacgg
cccatatgaccagcctaacggcagcaatagccgccgctgaagcggcaacaccctccagggttttaaccccaaaac
gggtaccctaacccaacaatcagctcagcccaacgccggggatcttagaagtcaatatcaaaacctctggcttcag
gccggaaaaatctccctactcgtccttcagctacaaccttggtccaccatcgtccaaggccccgccgaaagctctg
tagagtttgtcaaccggttacaaatttcattagctgacaaccttcccgacggagtcctaaggaacccattattgac
tcccttagttatgcaaatgctaacagagagtgtcagcaaattttgcaggggcgaggccagtggccgcggtggggca
aaaactgcaggcttgcgcacaattgggcccccaagaatgaaacagcctgcacttctcgtccacaccccaggccca
agatgcccgggcctcggcaaccggcccccaaaaggcctcccccaggaccatgctatcgatgcctcaaagaaggcca
ttgggcccgggattgtcctaccaaggccaccggcccacctccgggaccttgccccatatgtaaagatccttcccat
tggaaacgagactgtccaaccctcaaatcaaaaaactaagtgcatgacctacgagctacaaatgctcttacaaagc
ccattccggcactctctcccggaccgccagaccttaccgctatccctacgcaccctccacatatcatttgcctaga
tctcaaagatgccttcttccagattccagtcgaagaccgcttccgcttctacttgtcttttaccctcccatccccc
gggggactccaacctcatagacgctttgcctggcgggtcctacctcaaggcttcattaacagcccagctcttttcg
aacgagcactacaggaacctcttcgccaagtttccgccgccttttcccagtctcttctggtgtcctatatggacga

tatcctttacgcttcgcctacagaagaacagcggtcacaatgttatcaagccctggctgcccgcctccgggaccta
gggtttcaggtggcatccgaaaagactagccagacgccttcgcccgtccccttttgggacaaatggtccatgagc
agattgtcacctaccagtccctacctaccttgcagatctcatccccaatttctcttcaccaattacaggcggtctt
aggagacctccaatgggtctctaggggcacacccactacccgccggcccctgcaacttctctactcttcccttaaa
aggcatcatgaccctagggccatcatccagctttccccggaacagctgcaaggcattgcagagcttcgacaagccc
tgtcccacaacgcaagatctagatataacgagcaagaacccctgctagcctacgtacacctaacccggcggggtc
caccctggtactcttccaaaagggcgctcaatttcccctggcctactttcagaccccccttgactgacaaccaagcc
tcaccttggggcctccttctcctgctgggatgccaatacctgcagactcaggccttaagctcgtatgccaagccca
tacttaaatattatcacaatcttcctaaaacctctctagacaattggattcaatcatctgaggaccctcgagtcca
ggagttgctgcaattgtggccccagatttcctctcagggaatacagcccccgggcccttggaagaccttaatcacc
agggcagaggtttttttgacgccccagttctcccctgatccgattcctgcggccctttgcctctttagtgacgggg
ctacaggacgaggagcatattgcttgtggaaagaccaccttttagactttcaggccgttccggccccagaatccgc
tcaaaagggagaactagcaggtctcttggcgggcttagcagccgccccgcctgaacctgtaaatatatgggtagat
tccaaatacctgtactctttgctcagaaccctagttctgggagcttggcttcaacctgaccccgtaccctcctacg
ccctcctatataaaagcctcctccgacatccagcaatcgttgttggtcatgtccggagccactcttcagcatccca
ccctattgcttccctgaacaattatgtagatcaactgcttcccttagaaactccagagcaatggcataagctcacc
cactgcaactctcgggccttgtctcgatggccgaacccacgtatctctgcctgggaccccccgttcccccgctacgc
tgtgtgaaacctgccaaaagcttaatccaactggaggaggaaagatgcgaactattcagagagggtgggccccgaa
tcatatttggcaggccgatataacccattataaatacaaacagttcacctacgctctgcatgtgtttgtagatact
tactctggagctactcatgcctcggcgaagcgtgggctcaccactcaaacgaccattgagggccttcttgaggcca
tagtgcatctgggtcgcccaaaaaagctaaacactgaccaaggtgcaaactacacctccaaaacctttgtcaggtt
ttgccagcagttcggagtttccctttctcatcatgttccctacaaccccacaagttcggggttagatgaacggaca
aatggactgctcaaacttcttctatctaaatatcacctagacgaaccccaccttcccatgactcaggccctttctc
gagccctctggactcacaatcagattaacctcctaccaattctaaagaccagatgggagctacaccattcaccccc
acttgctgtcatttcagagggcggagaaacacccaagggctctgataaactctttttgtacttgctccccgggcaa
aacaatcgtcggtggctaggaccactcccggccctagtcgaagcctcgggaggcgctctcctggctactgaccccc
ccgtgtgggttccctggcgtttgctgaaagccttcctcactctcactctcctcgctctctgtcggcccatccagac
ttggagatgctccctgtccctaggaaaccaacaatggatgacagcatataaccaagaggcaaaatttccatctcc
attgaccaaatactagaggctcataatcagtcacctttctgtgccaagtctcccagatacaccttggactctgtaa
atggctatcctaagatctactggccccccccacaagggcggcgccggtttggagccagggccatggtcacatatga
ttgcgagccccgatgcccttatgtgggggcagatcggttcgactgcccccactgggacaatgcctcccaggctgat
caaggatccttttatgtcaatcatcagattttattcctgcatctcaaacaatgtcatggaattttcactctaacct
gggagatatggggatatgatcccctgatcaccttttctttacataagatccctgatccccctcaacccgactttcc
ccagttgaacagtgactgggttccctctgtcagatcatgggccctgcttttaaatcaaacagcacgggccttccca
gactgtgctatatgttgggaaccttcccctccctgggctcccgaaatattagtatataacaaaaccatctccagct
ctggacccggcctcgccctcccggacgcccaaatcttctgggtcaactcgtcctcgtttaacaccacccaaggatg
gcaccacccttcccagaggttgttgttcaatgtttctcaaggcaacgccttgttattacctcctatctccctggtt
aatctctctacggcttcctccgcccctcctacccgggtcagacgtagtcccgtcgcggccctgaccttaggcctag
ccctgtcagtggggctcactggcattaatgtggccgtgtctgcccttagccatcagagactcacctccctgatcca
cgttctggagcaagatcagcaacgcttgatcacagcaattaatcagacccactataatttgcttaatgtggcctct
gtggttgcccagaaccgacggggcttgattggttgtacatccggctgggttttcaaagcctatgtcccacaatta
atgagccttgctgtttcctgcgcattcaaaatgactccattatcctccgcggtgatctccagcctctctcgcaaag
agtctctacagactggcagtggccctggaattgggatctggggctcactgcctgggtgcgagaaaccattcattct
gttctaagcctgttcctattagccctttttttgctcttcctggcccccctgcctgataaaatgcttgacctctcgcc
ttttaaagctcctccggcaggctccccacttccctgaaatctccttaacccctaaacccgattctgattatcaggc
cttgctaccatctgcaccagagatctactctcacctctcccccgtcaaacccgattacatcaacctccgaccctgc
ccttga
>#7 Walleye dermal sarcoma virus (WDSV) AF033822 8763 nt
atgggtaactcttcctctactcctcctccctcggctcttaaaaactctgacttatttaagactatgctaagaacac
aatattctggctccgtcaaaaccagacgcattaaccaagacataaagaaacagtacccactatggccagatcaggg
tacctgcgccaccaaacattgggaacaagccgtactaatcccattggattcagtctcagaagaaaccgctaaagta
ttgaattttctacgagtaaaaattcaagcccgaaaaggggaaacagcacgacaaatgacggcacatactattaaaa
aactaatagtagggaccattgacaaaaataagcaacaaacagaaatattacaaaaaacagacgaatctgacgaaga
gatggataccacaaacaccatgctattcatagcaagaaacaaaagagaaagaattgcacaacaacaacaggctgac
ctagcggcacagcagcaagtcctccttttacagcgagagcaacagcgagagcaacgagaaaaggacattaaaaaac
gcgatgagaaaaagaaaaaattattgccggatacaacacaaaaggtggaacaaacagacatcggtgaagcctcttc
ctctgatgcctcagcacaaaaacctatcagtactgacaacaacccggacctaaaagttgacggagttctgactcgg
tcccaacacacaacagtaccttcaaacataaccataaaaaaggatggtacttcggtacaatatcaacatcccatta
ggaattatcctactggcgaagggaaccttactgcacaagttcgaaatccattcaggccattggagttacaacagct
taggaaagattgccctgcactgccagaaggcattccccagctagcagaatggttgacacagaccatggcaatctat

aattgcgacgaggcagacgttgaacaattggccagagtaatcttccccacacctgttagacaaatagcaggtgtta
taaatggccatgcggctgcaaacacagccgctaaaattcagaactatgtcacagcatgtaggcagcattaccccgc
agtatgtgattggggactatacaagcctttacatacaaacctccccaaactgcccacgaatatgttaaacacgct
gagataatattcaaaaataatagtggacttgagtggcaacatgcgacagtacctttcataaacatggttgtacagg
gtttacccccctaaagtcacccgtagtctgatgtctggtaacccggattggagtaccaaaactataccccaaatcat
acccctcatgcagcattacctaaacttgcaaagcagacaggatgcaaaaattaaacaaactcccctagtcttacag
cttgcgatgccagcgcagaccatgaacgggaataaaggatatgtaggctcttaccctacaaacgaaccatattatt
cgttccagcaacaacagcgacctgcaccaagggccccacctggaaatgtacccagcaatacctgcttcttttgcaa
acaaccgggtcattggaaagctgactgccccaataaaacacgtaatcttagaaacatgggaaatatgggacgaggc
ggaaggatgggaggccctccgtataggtcacaaccataccccgcatttatacaacctcctcaaaaccaccaaaacc
aatataatggccgcatggaccggtcacagctacaagcctctgctcaagagtggcttcccggtacatatcctgcata
ggatccaattgactgtccttatgaaaaatctggtacaaaaacaacccaagatgtaattaccactaaaaatgctgaa
attatggttacagtaaaccacacgaaaattcctatgttggtagatacaggcgcctgcctgactgcaataggagggg
cggccacagtggttcccgacctcaaattaacaaacacagaaattatagctgttggtatttccgctgaaccagttcc
acatgtcttggcaaaacctacaaaaattcagattgagaacaccaatatagatatctccccttggtacaaccctgat
caaacatttcatattctaggaagagataccctttctaaaatgagagccatagtatctttcgagaaaaatggagaaa
tgacagttcttttaccaccgacataccacaaacaattgtcatgtcagactaaaaacactcttaacatagacgagta
tttactacaatttccagaccagctatgggcctcactaccaacagatattggtcgaatgttagtgcccccctattaca
ataaaaatcaaagacaatgcatcattaccatcaattcgccaatacccattacctaaagataaaaccgaaggactaa
ggccattaattagttcactagaaaatcaaggaatattgataaaatgccatagcccatgtaatacaccaatctttcc
tattaagaaagcaggtcgggatgagtatcgaatgatacatgacttacgcgcaataaataatatagttgcccccctt
actgcagttgtggcaagtcctaccactgtattatctaacctcgctccgtcactacattggtttacggtgatcgacc
tctcaaatgcattcttttcagtaccaatacacaaggacagtcaataccttttgccttcacctttgagggacatca
atacacatggactgtcttgcctcagggcttcatacatagccctaccctgttttctcaggctttgtaccagagtctc
cacaaaattaaattcaaaatttcctcggaaatctgcatttacatggatgacgttctcattgcctcaaaagataggg
acactaatttaaaagacacagctgttatgttgcaacatttggcttcagaaggacataaagtgtctaaaaagaaatt
acagttgtgtcagcaggaagttgtttatctgggtcaattattgactcccgaggggcgtaaaatactaccagacaga
aaagtaacggtctcacagtttcaacaacctactacgatccgacagattcgtgcctttctaggtctggtgggctact
gcaggcattggataccggaattttctatacatagtaaattttggaaaaacaactgaaaaaggacaccgccgagcc
attccaactggacgaccagcaggtagaggcttttaacaaacttaaacatgcgataacaacagcaccagtattagtg
gtccctgatccagctaaaccttttcaactgtatacgtcacattcagaacatgcatccattgctgtgttgactcaga
aacatgcaggaagaacgcggcctatagctttttatcgtccaaatttgacgctatagagtcggggcttcctccatg
tttgaaagcttgtgcttccatacacagaagtcttacgcaggcggactcgtttattttaggcgcgccactgattatc
tatactacgcatgcaatatgtactcttttacaacgcgatcgatcacagctcgttaccgcttcccgcttcagcaaat
gggaagcggatttattacgacctgaactaacctttgttgcctgctcagcagtctctccggcgcacttatatatgca
gtcttgtgaaaataatattccgcctcatgactgcgttttacttacccacacgatctccaggccccgacctgattta
tctgatctcccaatacctgaccctgatatgactctgttttctgatggctcttatacaaccggcagaggaggagcgg
cagtagtgatgcacaggccggttactgatgattttataattatccatcagcaacctggtggtgcttcagcacaaac
cgccgaattgttagccctagccgcagcatgccacttagctactgataaaactgttaatatatatacagactcccgt
tacgcttatggtgtggttcatgactttgggcatctctggatgcacagaggctttgttacgtccgctggaactccta
taaaaaatcacaaggaaatagagtacctgttaaaacaaatcatgaaacccaaacaggtttccgtaattaaaataga
ggcccacactaaaggtgtaagtatggaggtgaggggtaacgccgctgctgatgaggccgctaaaaatgctgtgttt
ttagtccagagggtactcaagaagggggacgcccttgcgagtacggacttggtcatggaatacagtgagacggatg
agaaattcaccgccggagctgagttacatgacggtgtatttatgcgaggggatcttattgtccctcccctcgaaat
gcttcatgccatcctattggccatacatggagtttctcacacgcacaaaggggaataatgtcttatttttcgaaa
ttttggacacacccaaaagcatctcaaaccatagacttgatactggggcattgtcagatatgtctcaaacacaatc
ccaaatacaaatcacgactacaaggtcatagacctctaccaagcagaccgtttgcccacctacaaatagactttgt
ccaaatgtgcgtgaagaaacccatgtatgccctggttataatagatgtcttttcaaaatggccagaaataatacca
tgtaacaaagaagatgcaaaaactgtatgtgatatattaatgaaagacataatcccaagatggggggttacctgatc
aaatagactctgaccaaggaacgcacttcacggctaaaataagccaggaactgacccactccataggagtggcttg
gaaactacactgccctggacacccacgatccagtggcatagtggaaagaacaaatcgaactctaaaatccaaaatc
ataaaagcacaagaacagctgcaattatccaaatggacagaagtgttgccatatgtcctattggaaatgcgtgcaa
ccccaaagaaacacggactaagtcctcatgagatagttatggggagaccaatgaaaaccacatatctaagtgatat
gtcacccctatgggccactgacacattggtgacgtatatgaataaacttacacgacaattgtcagcatatcatcaa
caggttgtggaccagtggccttctacaagcctcccccccaggcccagagcccggatcctggtgtatgcttagaaacc
caaagaaatcatctaattgggaaggaccttttctgatcctcctctctacaccaacggcggtaaaagttgaaggacg
accaaaagcacttccgactgatggtcccagcctggagaccatagaacataagacagaaatggttaatacaaccagg
agcgaagaacaaagtccggttagaccatcaaaaactagacaacaattaatagacgaaacaccagaaatatgtgcaa
atgcatgggtaataaggttgataaccgaatttcccaccgaattagggaatatgagtcaaaaacaaaaaaccatagc
aatacaggtgcacaataccacaatgactatggaagaaaccgttttctccctagtatctcatgtaaataaaaagaat

tatgagatacacaatgtatcaggaatatgtaccaaataccagttagtacccggaaacttcacgtgtagcacccgaa
aatgcattagtcaaacaaaagaaaaaaggatcattagtactacggtaaaagatacctacgaggtttacctaccttt
cgcatggtcacaaaaacccacaggaggagataaataccccgagccacaaatagggtataatacaggaacagggagg
ctgaaccaatggaacaaagacgaattcgtaataaaacaatgcagaaagaaacgaggaaaaagacagatcacggtac
ctaacagcaccctttcccccacaggtactacagacttcacaaaatttacccctaatcctatatctcccaacagtac
ggccctgaatgagcttgagcaaaagactacaccaataggaacagagcaaccatttaataatgaaaaatggcagaat
ttgatatttgggaatatagttacgaaaatggatccccaatgtgaagccgagctatttcaacaatttaatatatcag
acaaaaccgtacaagtagagtttaaagtgactagcctccccggacaaaacatatcatgtcaagcgatatacaatac
tgaacatggtataaacatagaaaacaaaaactgtgtaataagccttattaaagaaaatcggaaaattaaggcccat
gcctacattacgcgcacaggctcatatgagtggtacgctcagcaagtaacttcaaaaggaataatacaagaagtta
gaaaccttgttactatagtagaatgtgagtgtcctatagtaaaaccattaccacaaggggaataattccattaac
catgcctatgagggtactcaccaacccttcacctatcctaatacattcagcattaaaatttgatttatctaaattt
gggttatccccgtgttcattctcccctatggaatggcagacatatattactaaacctttgaaaagggctatgcatg
gtttcgaggtccatcaacggaagaaacgagacctaggaataggattacatagtacactaaactcatggtggaatgg
agcaaattcattagggttgaccgtagaaagtgccgacagacaaaaatatgatcagaaaatcttaaaggtgttacaa
aaccttgcggtacaacaacgaacggatgtaaaaaaccaacaaacattgggaaaggcattggaaactcccatatata
cgattacgcttcaattagccgacagtcttacagcagcaatattaaagcacgaacaacaacaaaacgtgggaatcac
ttgtaaagatatagccatcctaacagtgacgcaaatcgctacgtacctacgggatattcagcatgagcacttacct
gtatggtttatagaacaaataactaatcaaattttgctaccagtaggacaggtaataatgccggagataacagccc
cccctatattgaacccacttattggatggaaccagtccgtcctggtaataggacttacgcaccagctcactatcac
tactgtacagcagcctttatacaaagcagcaaacatgggaaacttccaagattggactcccttccctccttttatt
ctagcgaataaaacacacggtttcagtatcgattgtcccataatgcgaaactccttttttgtgccatacgctcccca
caccagttaaactttcagaatgggaaagaagtacatcgaccatttatcaaacgtcaccccaggtgtggataacgcc
tgaaggaaaggcctgcctaaatcatcgaaatataacagttcaggacagaacatgcctgataaacaagccagggtgc
tttatccctaaacatccttggagtgcagggaaacaaactatagtcccaacccaatacatacaacagaactttgtgc
cagatactatagatacagaggataatcaaacccgggtattacaaaaggaaatgatagaagcgatatccaaggcaaa
aagggactacggagtactaaaacagggccagatagcattgattcgacatcacgaggctattactaccattcttgga
caagaggcaacatatagcattaaagaaacacaggcattaatttcatctatcgaacaagaggcatggtataataatt
tattctcttggtatgacggctctgtatggtcacagctacaattgataatagttgttatcacatgtaccataccact
tttatgggtattaaatacatgtttatttttttaaattacgaagagccataagacgggaacgggacaacaatatagtg
gtagaataccaagcacaaaccagaggaagaagaacgcatatgactgagcctatcactaaaaaacaacgagcaaaac
tgctacgacacgcaaaaacaaacagacgattgccacgtagcctaagagctacaccagcggtatctgcttttgaaat
ggttacgtttgatccacaagaagaaaccgttgagataaataggatagacccttctcatgagaacaatgaccacgga
ggccccatgaatatggcaccgattataagtgcggacagttatgccctgccgactccttatattaccataatgctag
acagagaacttctaaatcagggcatgcgaaaagtaattacgctcttaaacgatccagcaagagaagtattcaacaa
agcatataatttggttaccactaatcatttcactctggcttatggatgtgacgagtcagcaggatgggttaaccaa
catgctgaatacatgggaaagccggtaatcgtgacattggcgggcctagtaattacgcctgtgggtttggcttgga
taccactcccacaacaagaaccattggaaaagttatttatggtccccaatagcatgccacatgtcaccgtggccat
ggccgattatcatgagacaaaagaaatgggcaaaattgtgaaagacataaacaacgaagaacttcttttggtaaaa
ccacaactctttaagtgggaccgaagggtttttttgtagcctgtccccttgttataagaggagttgtcactggacac
agtttgctgcatatcgcatgccctgctacggcagtacaggccgaagggacctaacgcaatctgcgagcaccttatg
tattatttcattgtttattttatttaaagtaattttatttcagtttatgttgatcccgatgatctaccaacattta
ttattattattagttttatattttttttttattttctttcattatattttattttttattttttcttgatttttctta
cgtacgtatatacaaacgtataa
>#8 Walleye epidermal hyperplasia virus type 1 (WEHV1) AF133051 8742 NT
atggggaacaccaaaacaaaaccgctcacggaagatcagtctaaatacctcaaattagtggaaaccgaacactgcg
agaaaatcccgttgtcaaaaatacataaggaatttcgatcattttgtcctacatggccaaaggacggacctgtga
aacaaagaaatgggaatgcctcgtgcaatcaaagaaagacacaaaattgacaccagaagccattaaattatatggt
tttattaaaaacaggctgcaaatcagaaaagcagttactgcaaaacaattggccacacacgctgtcagacaaatgg
tagtaaaacaaaaggacaccattccttccgctcctcttgaagatgaccagagttcagaagatgaggacaggttaaa
ttcttacatgatgctaagaagaaatagagcagaaaaagaacaaatagctaggcacacacgaaaaaagcaagacgaa
caacgaaagcttgacctaatagaaaaagaaaaaagctcggtggttaagccttcacagaaaactactaaagcagatg
tttcctcgccactagtagtaagacaacgaaccggggctacagcagaactcttccctcaaaaacccgtacgacatta
tcctacaggtaatggagcagacacagtccccatacacagtgcatttaaagctacggacttgcacatgttgagaaaa
gattgtccctccataccggaagatatagctaaggtgggagcatggcttactcaaacctacgagatttatgattgca
accccttagatctgttacaattagcaagagctatttttccggtagcagtagttgaacgggcaacaaatttagcagg
atggcctggatctcctgtccgagacaaaatagatacactaatggccgaattacgtgctcattacccgcctatctgt
gactggggagccattcaaaacttccaatacaaaacaggccagacagcacatgaatatgtctcagaggcggaaaaac
tgttccgtgcaagcagcgggctacaatgggaacacgccctggtcccattcctaaacatgttggtacaaggcttgcc
cgcgaaactcgcttactctctaaagagttccacttatgactgggctgaaaaacaatcccacaaatgacctccctg

atccaacatcacctgtccctacaggaaaagaaagaaggtaaaaacaagcaacaccctgttgttgttcagttggctg
tgccaaacctcacagacatgcaaagacctgctccctatgcccctaggaaccctgaagatgtgtgtttctattgcaa
acagccagggcactggaaggacgaatgtcctaatagaaggggagggagaggtagaggtagaggaaatagcttcagg
ggattcggtttcagaggatttcagagaggaatggggaggggaagaggcatgctgagagggaacggtggaggaggaa
gacattcctttcagtatgatcagatagaaggccaactgccaccaccccaacagtacaatcaacagagatactttca
acaacatcacgaaccagtttatcaacaatctcacatgcaagcgtcagcacaggagtggtaccctaatcaggggcct
tcagcatagagtgttggctgccagggtgctcaggtgtgtacgaacagagaagctgaaatattagtgaaagtaaata
acacaattatccctatgcttgtcgatacaggagcttgtttaaccgccataggaggatcagctcctattgtccctaa
attaaaattaactgacacaactgcctacgcggtaggaatttcttcagatcctgttgaacatataaaaacggagcaa
gtaaaggtaaaagtaaaggatgcttcatgcaatatagagccatggtttaataaagatcaaacctttcatatcctgg
gaagagacacattaactaaaatgagagctatgatagcctttctgccgtgtggtaggatggaagtggtctttccggc
tcggtaccatcagaatgtcattacggatggtgcagaggcgaaatggtcactccagcaattcccagagtctctgtgg
gccagctctcctacggatattgggaaaatgaaaatcacacccattcacatagaagtacttcctgttccatgcccta
gtattagacaatatccactaccgaaagaaaaagtcgaaggacttcgaccaatgatacattctttgctggctcaagg
cgtactaactgaatgtcacagttcctgcaacactcccatcttcccaataaaaaagccaggaagggaggagtatcgc
atgatccacgatctcagagctatcaatgaaatagtagctccactgactgcagtagttgctagcccaaccacggtat
tagcaaatctatcgccagatatgacttgcttcacggtgatagatttatcaaacgcatttttctctgttccaataca
ccctgacagtcaatatctgtttgcatttacctttgaaggaagacagtatacctggactgtattgcctcaagggttt
atacatagccctactttgttttcacaggctttgttcagcagcctttccaaaataaaagactctctgacttcggaaa
tttgtatatacatggatgacgttctaatcgcatctagagacgaagaaacaaactataaagatacagctacgatgct
acatcatttagctgatgaaggacataaagtgtcaaagaaaaaactacagctatgcaaaagcgaagttgtttacttg
ggacagctcatctccaaggcggggcgagacatcctgccagaaagaaagaaaacagtctcgcagtttgccgcgccca
ccacggttagacaggttcgggcgtttcttggtttggcaggctactgcagacattggattgcagattactctgaaaa
cagtaaacatctagaagagttgcttaagaaagaggtcattgaacctttttactctgacaggcgaacaactgcgagca
tttgagcaactcaaaagcactctgttatctgcgcctgtattggcgatcccagattacagaaaggactttgagttgt
acacatctcacactgatcatgttgcagtggcagttctagcacaaaaacaagccggacggacaagaccaatcgcttt
cctatccgcaaaattagatgccattgaacagggacttccaccctgtttaagagcatgcgcatctatacataaaaac
ctaacacaggctgactcctttcttttgggtagaccagtaaaaatatacacaacacactcaatatgtacgttgctac
agagagatagatcacagttagtcacagcatctaggtttagcaaatgggaagcggatcttttaagaccagaacttac
ctttgttacctgtactgctgtttctcctgctcatctcctggcgactgcaacgtccggtgacccacctcacgattgc
gtgttattaactcataccatgtcaagaccacgctcggatctttctgatgtaccactagaaaaacccgaactgatct
tattcacagatggatcttactcaaaggggggaaggtgggtgtgctgttgtccaatacgcacctgaaaaggatacatt
tctgacggtggcagcatgtacagggttcacctccgcacagacagcagagttagcagctattactcttgcttgtcag
tatgcggaagataaatccgttaacatatatacagactccagatatgcctttggcgtattacatgactttgggcatt
tatggcaacatcgaggctttgttacctctgcaggcacgccaatcaaaaatcatgagatgatacaaaaactttgga
agcagtgcaacttccatccaaaatatcactcatgaaatgtcctgcacatacgaaaggtgtgaccttggaaatacgc
ggaaacgctgctgctgatgaagctgccaaaaaggcagtttccatgaggcagggagtactcaaagaggacgctcttg
aggaaaccatgccctctctgtccatgtgttacgacctcatagatgaaaaatactgtgaggatcgaccacagggaca
gggtgtattctggcatgaggatttaattattcctccagtagcaatgttacctagtattatgcgtgctatacatggg
gtttctcatacgcacaaaggggggaatgatggcatactttaaaaaattatggtttcatccattggcagcacaagcta
ttgatgaggtaatagctggatgcgttgtgtgccttaaacataacccaaaatacagaaaaagaaaagataaacaagg
tcatagaccattgccaggaagaccattctcccatttgcaggttgatttcgtgcatatgagcgataagaaacccatg
tatgcacttgtcattgttgatgttttgtccaaatggcctgaagtattttcatgtaacaaagaagatgccactacag
tatgtgacatcttaatgactgacattctccctcgatggggattaccagatcaaattgattctgaccagggaactca
tttcacctctaaaataactcagcaactcgctaactcaattggagtagcttggaagttgcactgtcctggacacccc
caatccagtggaatggtggaaagactaaatagaaccatcaaaacaaaaattgaaaaagtaaaaacacatctacaaa
tagatgcatggcataaagcactgccttatgttttaatggaacttagagcaatccctaagaaaaattacctaagccc
atacgaaattgtaatgggcagaccgatgaagttagacactctaagtaatgtgtctccactatgggccagtgataca
ctagtaacatacatgaataaactcacacatgctctctcagaatatcatacacaggtggccaaccagtggcctacca
gtgttctccctccaggcccagaaccagggacgtggtgtatgataaaatcgtttaccaagaaaccaaccgtatggga
aggaccacacctcatcctgctgtccaccccaacggctgtaaaggttgaaggaaaaccagtgcaggccgcacgacta
tatgaggacacaggagaaggttatgctgttattttcataattaaggatgtggcttacccaatgtctgaaaggtcag
gtcactgttccatgattagaccagatgcgaaagggacgtttgaatgtagtccagtggaatgcaaggaggtacctct
tagaattcacacatgtactgctaaacaggcatccttcttgctattccagccagactaccaaagcttagccgaagaa
aaggagaagctgccatcaccttctctaacaaacagcctgcccccaggttcaaccttaaatgaaacacagataagat
gttctgttagttgggtaatacaattagccagccaacataaacatattgggcataagcactcagatggaacagttac
tataaccattgaaaaagcaatgcatcggtcttcaatggacggaggcaaggtccaggctgtagtgaatggtgtacag
tatgaaacaaccacaagagtaggagcctgtgcttacttcaaactaggcacaggtacgtacacttatgatggcatca
cccgctggtaccttactccttatcctgagtatggtgtagatccccacatgaaaaagctggtactaaagaaagtgag
gagaactagaagggatacctctgctataactactatcctaggggtacgaagtatggatgttgtctggacttgtgca

gtaaaattagcagaatatcaagccataacccaattatctgctgtaattgttgaggagacatgttttatctattggg
tccaaaaggacgcctcaggggcagatcaagattacagggtagcagaagaaacaagttgttgtcttcgttcagactc
gtcagacagctgttgctcgtctacaccagaaacttatgagccaacacattacgtaaatgttccatgtgaagaaaca
ttcagaggaaaaataaaacatctgcctccctattctcccgaaagggagtgggcacatggggcatgttcatactcat
ggtttccccgaggggttatgtgcaatctatcagagtagaagattactgttgtctggatttacttccacctgtacc
tactactgagccaccgccctccacgacaagtataggcaagagcacagcttcgcctttgataaaaacaaaacgcgcg
gttgtaactttggcacctagtactactagtaccactattgccgccaaaataacacccatagagaaaactacctcta
caactgttggcaccacagggactgtcacagtagctgctaccaccactacctccactgtaaaaacctctacagactc
atctcctgatccttatgaaacatttgatggagaacaatgggaaaaactaataatgggcctcactaaaactcaaatt
aaagctacgtgcattggagagataaaagagaaaacaatgatcccagaagacttgacacaactagagtttaaactaa
ctggacgaggagataaagttgtagaatgtcaagttgtatacaatcctgcatatggcacaagctacataaatacatc
ttgtagtatccaactaaacagaactaaatctgtagtatccgtttatgtaccatacattaaggtaggggagtatgac
tggcatcaccttataactacttcaggaggtctctctggaaataaatataccctgattgtagaaaattgtacctgtc
ctaatcttagtccattacttctaggaggaatcgtggcaacctcaaacccacgacctgttctttatgatccatcacc
tatactggtacataccactctgacctatgacctaaaagcatttaaagataccaaactatgtggattctccaataca
gagtggaatacttacatctctaggcctattaatacggctataggagtattcaaaagacctagaaaaatgaaaagag
atcttggtttgcatagttcattaaattcttggtggaacttcgaaaatactctaggcttagggttagaagctcataa
caggcaggaatatgatgaaagaatgttgaacatattaactgatatctctacccagcaagcactggatgttaaaaat
cagttgagcataggaaaagctttgactgtaccttcatatagagtctcgctaagcctgactagtaaggtaactgaag
ctattatggctcatgagaaagatcaaaattttcgaaatcattgctataacatggtaatgatggcagtaagttctat
cacactaaacttaagagatattgagcacgatcacttgccttggtggttactatctggcatacaaactaagctgaaa
ctccccaagggcatgaaactggttcccagtcatcaaagcccgccagttgtaacgcctcaaattggctggaattcaa
caactttaattgtaggtctgactcaccagctacaagctgcaactgagctctctgctctcatgagaggacagaatat
gggaagatttcaggattggacacccatccctgcagtagtaatgtttaatgcaactcatgtcttacctactgaatgt
ccactagttcaaggagtgtttgtgtgtgatgaactacctgccatgataccattgacacagtgggagagtaatacta
caaccatttacaaacataccccacaaatatggttaacgcctgaaggacaggtctgcatcaatgttaagcggatatc
ctatcaaggattacaatgtaaccttaaacaggctggatgctttggtcctacacatttctgggaagcaggaagatta
aatatcattcctactcaacttgcacaagttaactttacgtatgatcttccaaacgaagataagaatcaaactgata
aattacaagaagcattagataaagcaattatggctgcagaaaaagaatatggaaatctgcatgatgtacaattggc
attggtaaaagatcatggaataaaagcaaaactactgtcacaaaaggctcttcaatcagtttctgataccgaagca
gtattaatcaagctcgatacagaaagatggttaaattcattcttttcatggtatgatggaggatggttatctaaag
tacaattactggtggctgctcttaccctttcagttccactcttgtgggtagtaacgctttgcctggcgtgtcgcct
aaagaagaagatagggagacaggggaatcagcaaaatactatgattgtcagaacggacagttatcccatgtctagt
tacactccggttccgcttaggactccgttaagaaggatatgcgtagtatgccatatatctcaattgatttaagtg
cgtcctttatgactgaacgattggttcagacatttccaagatttacgggccaaatgaaagattggtttaatctggc
aacattggttaaaatacacccacatgtaactttagcttatggacaagaggaagtggcagaatggagggagttacat
ccattcattcttggccacagtgtcccagttacggttacaggaataatcgtgggaaaacttggcagtgcatgggtat
taaatgtgaatcaacatagtgttcaggacttgtatgaagttcctggatctattcctcacatttccattgcaattgc
tgaaggacatgaacaaaaagaattaggatggcacttatctcaattgcagcatctggatatggttcctctgtcacct
ggtgtcttctcttggggaccggacacaatctttatgacctctgcatttgtatgtcagggcatagtagcagaaaatt
ag
>#9 Friend murine leukemia virus (FMLV) M93134 7107 nt
atgggccagactgctaccaccccccttaagtttgactttagaccactggaaggatgtcgaacggacagcccacaacc
agtcggtagaggtcagaaaaaggcgctgggttacattctgctctgcagagtggccaaccttcaacgtcggatggcc
acgagacggcacttttaacccagacattattacacaggttaagatcaaggtcttctcacctggcccacatggacat
ccggatcaggtcccctacatcgtgacctgggaagctttagcagtagacccccctccctgggtcaaacccttcgtgc
accctaaacctcccctccttcttccccttcagcccctctctcccacctgaacccccactctcgaccccgcctca
gtcctccctctatccggctctcacttctcctttaaacaccaaacctaggcctcaagtccttcctgatagcggagga
ccactcattgatctactcacggaggaccctccgccttaccgggacccagggccaccttctcctgacggaaaggcg
atagcggagaagtggcccctacagaaggggcccctgactcttccccaatggtatcccgcctgcggggaagaagaga
accccccgtggcggattctactacctctcaggcgttcccccttcgcctgggagggaatggacagtttcaatactgg
ccattttcctcctctgacctctataactggaaaaataataacccctctttctccgaggacccaggtaaattgacag
ctttgatcgagtccgttctccttactcatcagcccacttgggatgactgccaacagctattagggaccctgctgac
gggagaagaaaaacagcgagtgctcctagaggcccgaaaggcggttcgaggggaggacggacgcccaactcagctg
cccaatgatattaatgatgcttttcccttggaacgtcccgactgggactacaacacccaacgaggtaggaaccacc
tagtccactatcgccagttgctcctagcgggtctccaaaacgcgggcagaagccccaccaatttggccaaggtaaa
agggataacccagggacctaatgagtctccctcagccttcttagagagactcaaggaggcctatcgcagatacact
ccttatgaccctgaggacccagggcaagaaaccaatgtgtccatgtcattcatctggcagtccgccccggatatcg
ggcgaaagttagagcggttagaagatttgaagaacaagaccttaggagacttagtgagggaagctgaaaagatctt
taataaacgagaaaccccggaagaaagagaggaacgtgttaggagagaaacagaggaaaaggaagaacgccgtagg

gcagaggatgagcggagagagaaggagagggaccgcagaagacatagagaaatgagtaagttgctggctactgtcg
ttagcgggcagagacaggatagacagggaggagagcgaaggaggccccaacttgaccacgaccagtgtgcctactg
caaagaaaagggacattgggctagagattgccccaagaagccaagaggaccccggggaccacgaccccaggcctcc
ctcctgggaggtcagggtcaggagcccccccctgaacccaggataaccctcaaagtcgggggcaacccgtcacct
tcctagtggatactggggcccaacactccgtgctgacccaaaatcctggacccctaagtgacaagtctgcctgggt
ccaaggggctactggaggaaagcgatatcgctggaccacggatcgccgagtgcacctagccaccggtaaggtcacc
cactctttcctccatgtaccagactgcccctatcctctgctaggaagagatttgctgactaaattaaaagcccaaa
ttcactttgagggatcaggagctcaggttgtgggaccaatgggacagcccctgcaagtgctgaccctaaacataga
agatgagtatcggctacatgagacctctaaagggccagatgtgcctctagggtccacatggctctctgattttccc
caggcctgggcagaaaccgggggcatgggactggccgttcgccaagctcctctgatcatacctctgagggcagcct
ctaccccagtgtccataaaacaataccccatgtcacgagaagccagactggggatcaagccccacatacagagact
gctggatcagggaattctggtaccctgccagtcccccctggaacacgcccctgctacccgttaagaaaccggggact
aatgattataggcctgtccaggatctgagagaagtcaacaagcgggtagaagacatccaccccaccgtgcccaacc
cttacaacctcctgagcgggctcccaccgtcccaccagtggtacactgtgcttgatttaaaagatgctttttttctg
cctgagactccaccccaccagtcagtctctcttcgcctttgagtggagagatccagagatgggaatctcaggacaa
ttaacctggaccagactcccacagggtttcaaaaacagtcccaccctgtttgatgaggccctgcacagggacctcg
cagacttccggatccagcacccagacctgatcctgctccagtatgtagatgacttactgctggccgccacctctga
gcttgactgtcaacaaggtacgcgggccctgttacaaaccctcggggacctcggatatcgggcctcggccaagaaa
gcccaaatttgccagaaacaggtcaagtatctggggtatcttctaaaagagggtcagagatggctgactgaggcca
gaaaagagactgtgatggggcagcctactccgaagacccctcgacaactaagggagttcctagggacggcaggctt
ctgtcgcctctggatccctgggtttgcagaaatggcagccccccttgtaccctctcaccaaaacggggactctgttt
aagtggggcccagaccagcaaaaggcctaccaagagatcaagcaggctctcttaactgcccctgccctgggattgc
cagacttgactaagcccttcgaactttttgttgacgagaagcagggctacgccaaaggtgtcctaacgcaaaaact
ggggccttggcgtcggccggtggcctacctgtccaaaaagctagacccagtggcagctgggtggcccccttgccta
cggatggtagcagccatcgccgttctgaccaaagacgctggcaagctcaccatgggacagccactagtcattctgg
cccccatgcagtagaggcactagttaagcaacccccgatcgctggctctccaacgcccgaatgacccactacca
ggctctgcttctggacacggaccgagtccagttcggaccaatagtgaccctaaacccagctacgctgctccctcta
cctgaggaggggctgcaacatgactgccttgacatcttggctgaagcccacgggactagaccagatcttacggacc
agcctctcccggacgctgaccacacctggtacacagatgggagcagcttcctgcaagaggggcagcgcaaggccgg
agcggcagtaaccaccgagaccgaggtaatctgggccaaagcactgccagccgggacatcggcccaaagagctgaa
ctgatagcgctcacccaagccttaaaaatggcagaaggtaagaagctaaatgtttacactgatagccgttatgctt
ttgccactgcccatattcacggagaaatatatagaaggcgcgggttgctcacatcagaaggaaaagagatcaaaaa
taaggaagagatcttggccctactgaaggctctcttcctgcccaaaagacttagcataattcattgcccgggacat
cagaagggaaaccgcgcggaggcaaggggcaacaggatggccgaccaagcggcccgagaagtagccactagagaaa
ctccagagacttccacacttctgatagaaaactcagcccctatactcgtgaacattttcactatacagtgactga
cataaaagatctgactaaattaggggccacttatgacaatgcacagaagtgctgggtttatcagggaaagcctgta
atgcctgatcaatttacctttgaactattagattttcttcatcaattgacccacctcagtttctcaaaaacaaagg
ctcttctagaaaggagctacagtccttattacatgctgaaccgggatcgaacgctcaaagacatcactgagacttg
caaagcctgtgcacaggtcaatgccagcaagtctgccgtcaaacaagggactagagttcgagggcaccgacccggc
acccactgggaaattgatttcactgaagtaaagcctggcctgtatgggtataaatatcttttagttttcgtagaca
ctttctctggatgggtagaagctttcccaaccaagaaagaaactgccaaagttgtaaccaagaagctactagaaga
aatcttccccagattcggcatgccacaggtattgggaaccgacaatgggcctgccttcgtctccaaggtaagtcag
acagtagccgatttattgggggttgattggaaactacattgtgcttacagaccccagagttcaggtcaggtagaaa
gaatgaataggacaatcaaggagactttaactaaattaacgcttgcaactggctctagggactgggtgctcctgct
tcccctagccctgtatcgagcccgcaacacgccgggcccccatggtctcaccccatatgaaatcttatatggggca
cccccgccccttgtaaacttccctgatcctgacatggcaaaggtcactcataacccctctctccaagctcatttac
aggcactctacctggtccagcacgaagtctggagaccgttggcggcagcttaccaagaacaactagaccggccggt
agtacctcaccctttccgagtcggtgacacagtgtgggtccgcagacaccaaactaaaaatctagaaccccgctgg
aaaggaccctataccgtcctactgactacccccaccgctctcaaagtggacggcattgcagcgtggatccacgctg
cccacgtaaaggctgccgacaccaaaattgagccaccatcagaatcgcccttaattctcttcctgtctctcaaagg
ggccagatccgcagcacccggctctagccctcaccaggtctacaatattacctgggaagtgaccaatggggatcgg
gagacagtatgggcaatatcaggcaaccaccctctgtggacttggtggccagacctcaccccagatttgtgtatgt
tagctctcagtgggccgccccactgggggctagagtatcgggcccccctattcctcgcccccggggccccccttgttg
ctcagggagcagcgggaacagggcaggctgcgccagagactgcgacgagcccttgacctccctcacccctcggtgc
aacactgcctggaacagacttaagctggaccaggtaactcataaatcgagtggggggatttatgtctgccccgggt
cacatcgccccccggaaagccaagtcctgtgggggtccagactccttctactgtgcctcttggggctgcgagacaac
cggtagagcatactggaagccctcctcatcttgggactacatcacagtagacaacaatctcaccactaaccaggct
gctcaggtatgcaaagacaataagtggtgcaatcccttggctatccagtttacaaacgccgggaaacaggtcacct
catggacaattggacactattggggtctacgtctttatgtctctgggcaggacccggggcttactttcgggatccg
actcaaatatcaaaatctaggacctcgggtcccaataggaccgaaccccgtcctggcagaccaactttcgttcccg

ctacctaatcccctacccaaacctgccaagtctccctccgcctctaattcgactcctacattgatttccccgtccc
ccgctcccactcagcccccgccagcaggaacgggagacaggttactaaatctagtacagggagcttaccaggcact
caaccttaccaaccctgataaaactcaagagtgctggttatgcctagtgtctgcaccccccctattacgaggggtt
gcggtcctaggtacttattccaaccatacctctgccccagctaactgctccgcgggctcccaacacaagctgaccc
tgtccgaagtgactggacagggactctgcataggaacagtcccaaaaactcaccaggccctgtgcaacactaccct
taagacaggcaaagggtcttactatctagttgcccccgcaggaactatgtgggcatgtaacaccggactcactcca
tgcctatccgccaccgtgcttaatcgcaccactgactactgcgttctcgtagaattatggcccagggtcacctacc
atcctcccagttacgtctatagccagtttgaaaaatcctatagacataaaagagaaccagtgtccttaaccttggc
cttattattaggtgggctaactatgggtggcatcgccgcgggagtagggacaggaactaccgccctggtcgccacc
cagcagtttcagcagctccatgctgccgtacaagatgatctcaaagaagttgaaaagtcaattactaacctagaaa
agtctcttacttcattgtctgaggttgtactgcagaatcgacgaggcctagacctgttgttcctaaaagaaggagg
actgtgtgctgccctaaaagaagaatgttgtttctatgctgaccatactggcctagtaagagatagtatggccaaa
ttaagagagagactcactcagagacaaaaactatttgagtcgagccaaggatggttcgaaggattgtttaacagat
ccccctggtttaccacgttgatatccaccatcatgggcctctcattatactcctactaattctgctttttggacc
ctgcattcttaatcgattagttcaatttgttaaagacaggatctcagtagtccaggctttagtcctgactcaacaa
taccaccagctaaaaccactagaatacgagccacaataa
>#10 Feline leukemia virus (FLV) M18247 7164 nt
atgtctggagcctctagtgggacagccattggggctcatctgtttggggtctcacctgaatacagggtgttgatcg
gagacgagggagccggaccctcaaagtctctttctgaggtttcattttcggtttggtaccgaagccgcgcggcacg
tcttgtcatttttttgtctggttgcgtcttttcttgtcccttgtctaacctttttaattgcagaaaccgtcatgggc
caaactataactacccccttaagcctcacccttgatcactggtctgaagtccgggcacgagcccataatcaaggtg
tcgaggtccggaaaaagaaatggattaccttatgtgaggccgaatgggtgatgatgaatgtgggctggccccgaga
aggaactttttctcttgataacatttcccaggttgagaaaaagatcttcgccccgggaccgtatggacaccccgac
caagttccgtacattaccacatggagatccttagccacagacccccccttcgtgggttcgtccgttcctacccccctc
ccaaaactcccacacccctccctcaacctctatcgccgcagccctccgcccctcttacctcttccctctaccccgt
tctccccaagtcagaccctcccaaaccgcctgtgttaccgcctgatccttcttcccctttaattgatctcttaaca
gaagagccacctccctatccgggggtcacgggccaccgccatcaggtcctagaaccccaaccgcttccccgattg
ccagccggctaagggaacgacgagaaaaccctgctgaagaatctcaagccctccccttgaggaaggccccaacaa
ccggccccagtattggccattctcagcttcagacctgtataactggaagtcgcataacccccctttctcccaagac
cccgtggccctaactaacctaattgagtccattttagtgacgcatcaaccaacctgggacgactgccagcagctct
tgcaggcactcctgacaggcgaagaaaggcaaagggtccttcttgaggcccgaaagcaggttccaggcgaggacgg
acggccaacccagctgcccaatgtcattgacgaagctttccccttgacccgtcccaactgggattttcgtacgccg
gcaggtagggagcacctacgcctttatcgccagttgctgttagcgggtctccgcggggctgcaagacgccccacta
atttggcacaggtaaagcaagttgtacaagggaaagaggaaacgccagcctcattcttagaaagattaaaagaggc
ttacagaatgtatactccctatgaccctgaggacccagggcaggctgctagtgttatcctgtcctttatctaccag
tctagcccggacataagaaataagttacaaaggctagaaggcctacaggggttcacactgtctgatttgctaaaag
aggcagaaaagatatacaacaaaagggagaccccagaggaaagggaagaaagattatggcagcggcaggaagaaag
agataaaaagcgccataaggagatgactaaagttctggccacagtagttgctcagaatagagataaggatagagag
gaaagtaaactgggagatcaaagaaaaatacctctggggaaagaccagtgtgcctattgcaaggaaaagggacatt
gggttcgcgattgccccaaacggccccggaagaaacccgccaactccactctcctcaacttagaagattaggagag
tcagggccaggaccccccccctgagcccaggataaccttaaaaataggggggcaaccggtgactttcctggtggac
acgggagcccagcactcagtattaactcgaccagatggacctctcagtgaccgcacagccctggtgcaaggagcca
cgggaagcaaaaactaccggtggaccaccgacaggagggtacaactggcaaccggtaaggtgactcattcttttt
atatgtacctgaatgtccctacccgttattaggaagagacctattaactaaacttaaggcccaaatccattttacc
ggagaaggggctaatgttgttgggcccaggggtttaccccctacaagtccttactctacaattagaagaagagtatc
ggctatttgagccagaaagtacacaaaaacaggagatggacatttggcttaaaaactttccccaggcatgggcaga
aacaggaggtatgggaatggctcattgtcaagcccccgttctcattcaacttaaggctactgccactccaatctcc
atccgacagtatcctatgccccatgaagcctaccagggaattaaacctcatataagaagaatgctagatcaaggca
tcctcaagccctgccagtccccatggaatacacccttattacctgttaagaagccagggaccaaggattacagacc
agtgcaggacttaagagaagtaaacaaaagagtagaagacatccatcctactgtgccaaatccatataacctcctt
agcaccctcccgccgtctcacccttggtacactgtcctagatttaaaagacgctttttctgcctgcgactacact
ctgagagtcaattacttttgcatttgaatggagagatccagaaataggactgtcagggcagctaacctggacacg
ccttcctcaggggttcaagaacagccccaccctatttgatgaggctctgcactcagacctggccgatttcagggta
aggtacccggctctagtcctcctacaatatgtagatgacctcttgctggctgcggcaaccaggactgaatgcctgg
aagggactaaggcactccttgagactttgggcaataaggggtaccgagcctctgcaaagaaggcccaaatttgcct
gcaagaagtcacatacctggggtactctttaaaagatggccaaaggtggcttaccaaagctcgcaaagaagccatc
ctatccatccctgtgcctaaaaacccacgacaagtaagagagttccttggaactgcaggttactgccggctgtgga
ttcccggttttgccgagctcgcagccccgctataccctctcactcgaccaggaactctgttccagtggggaacaga
gcaacaattggccttcgagaacattagaaaggccctcttgagttcccctgccctggggttgccagatatcaccaag
ccctttgaattatttattgatgagaactcaggatttgcgaagggggtgttagtccaaaaactgggaccctggaaaa

gaccagttgcctacctatcaaaaaagctggatacagtggcatctggatggccccttgtttacgcatggttgcagc
catcgccatcctagtcaaggatgcagggaagctaaccctaggacagccgctaactatcctgacctcccacccagtt
gaggcacttgtccgacagcctccaaataaatggctctctaatgctagaatgactcattaccaagctatgctcctcg
atgcagagcgagtccatttcgggccgacagtctcccttaaccctgctactttgctccccctccccagcgggaaacc
accacgactgtctccagatttagccgagaccatggcacagaccgacttaactgaccagccgttgccggatgcagac
ctgacctggtacacggatggtagcagcttcatccgtaacggagagagaaaagccggagccgcagtaacaaccgaat
ctgaggtaatctgggctgcttccctcccacccggaacgtcagcccagcgagccgaactgattgccctgacccaggc
actgaagatggcaaaaggtaagaagctaactgtctatacggacagccgatatgcctttgctacagctcatgtacac
ggggaaatctacaggcggcggggcctgctaacttcagaaggaaaagaaattaaaaataaaaatgaaatcctcgccc
tattagaggcgttattcttacccaaaagactgagtatcatccattgcccgggacaccaaaaaggcgatagtcccca
ggcgaaaggaaacagattagccgatgatacagcaaagaaagccgccacagagactcaatcatcactaaccatctta
cccactgaacttatagagggtcccaaaaggcctccatgggaatatgatgacagtgatttagaccttgtgcagaaac
tcgaagctcattatgagccaaaaagaggtacctgggagtaccgagggaaaactataatgcctgaaaaatacgcaaa
ggagttgattagccatctgcataagttaacacacctcagtgctagaaaaatgaaaactttactagaaagagaagaa
actgggttttacctccctaacagagacttacacctccggcaagtaacagagagctgccgggcatgtgctcaaatca
acgcaggaaagataaagtttggacctgatgtaagggcccgaggccgccggcccggaacacattgggaagtagactt
cactgaaatcaagccaggaatgtatggatataaatacctcttggtgttcatagacaccttctctggctgggccgag
gcttaccccgccaaacatgaaacagcaaaagttgttgccaagaaactcttagaagaaattttccccgctacggga
tccctcaggtattgggttcagataatggacccgcctttatctcccaggtaagtcagtctgtggccaccctactggg
gattaattggaagttacattgtgcatatcgaccccaaagttcaggtcaggtagaaagaatgaatagatcaattaag
gagactttaactaaattaacgctagaaactggctctaaggattgggtgctcctcctgcccctggttttataccggg
tacgtaacacgccaggcccccacgggttaactccttttgaaatcctgtacggggcacccccacctatggctcactt
ctttgatactgatatctctacgttcgctacctcccccactatgcaggcacatttacgcgccctgcagctggtccaa
gaagagatccagagacctctagcggcggcctaccgagaaaagctcgaaaccccggttgtgcctcaccccttcaaac
caggagactccgtctgggttcggagacatcaaaccaagaacctcgagccacggtggaaaggaccacatatcgtcct
cctgaccaccccacagccttaaaggtagacggagttgctgcctggattcacgcctcccatgtgaaagctgcagga
ccaaccaccaatcaagacctctcggacagccccagctcagacgatccatcgtttctggtggggatcttattcacaa
tagacataggaatggccaatcctagtccacaccaaatatataatgtaacttgggtaataaccaatgtacaaactaa
cacccaagctaatgccacctctatgttaggaaccttaaccgatgtctaccctaccctacatgttgacttatgtgac
ctagtgggagacacctgggaacctatagtcctaagcccaaccaatgtaaaacacggggcacgttacccttcctcaa
aatatggatgtaaaactacagatagaaaaaaacagcaacagacataccccttttacgtctgccccggacatgcccc
ctcgctggggccaaagggaacacattgtggagggcacaagatgggttttgtgccgcatggggatgtgaaaccacc
ggagaagcttggtggaagccctcctcctcatgggactatatcacagtaaaaagagggagtagtcaggacaataact
gtgagggaaaatgcaacccccctgattttgcagttcacccagaaggggaaacaagcctcttgggacggacctaagat
gtggggattgcgactataccgtacaggatatgaccctatcgccttattcacggtatcccggcaggtgtcaaccatt
acgccgcctcaggcaatgggaccaaacctagtcttacctgatcaaaaaccccccatcccgacaatctcaaacagggt
ccaaagtggcgacccagaggccccaaacgaatgaaagcgccccaaggtctgttgcccccaccaccgtgggtcccaa
acggattgggaccggagataggttaataaatttagtacaagggacatacctagccttaaatgccaccgaccccaac
aaaactaaagactgttggctctgcctggtttctcgaccaccctattacgaagggattgcaatcttaggtaactaca
gcaaccaaacaaaccctcccccatcctgcctatctattccgcaacacaagctgaccatatctgaagtatcagggca
aggactgtgcatagggactgttcctaagacccaccaggctttgtgcaataagacgcaacagggacatacaggggcg
cactatctagccgcccccaatggcacctattgggcctgtaacactggactcaccccatgcatttccatggcggtgc
tcaattggacctctgattttgtgtcttaatcgaattatggcccagagtgacttaccatcaacccgaatatgtgta
cacacattttgccaaagctgtcaggttccgaagagaaccaatatcactaactgttgccctcatgttgggaggactc
actgtaggggggcatagccgcgggggtcggaacagggactaaagccctccttgaaacagcccagttcagacaactac
aaatggccatgcacacagacatccaggccctagaagagtcaattagtgccttagaaaagtccctgacctccctttc
tgaagtagtcttacaaaacagacggggcctagatattctattcctacaagagggagggctctgtgccgcattaaaa
gaagaatgttgcttctatgcggatcacaccggactcgtccgagacaatatggctaaattaagagaaagactaaaac
agcggcaacaactgtttgactcccaacagggatggtttgaaggatggttcaacaggtccccctggtttacaaccct
aatttcctccattatgggccccttactaatcctactcctaattctcctcttcggcccatgcatccttaacagatta
gtacaattcgtaaaagacagaatatctgtggtacaagccttaattttaacccaacagtaccaacagataaagcaat
acgatccggaccgaccatga
>#11 Bovine foamy virus (BFV) U94514 8073 nt
atggctcttaatgacttcgaccctatagctctccaggggtatttacccgcaccacgagttttgcaacataatgata
ttataatatgtagagcaacctctggaccatggggattggagacagatacaatcttattaggatccatttacaaga
tcctgcagggcaacccttgcctattcctcaatgggaacccattcctaatcggactgcaaatcctcggacccaacct
tacccagtggtctctgctccgatggcaactctagaaaacatcttgaataactttcatatacctcatggggtatcta
gatatggtcccctagaaggggtgattatcagccaggagaacaatattcccaggattttgtccagttacacaggc
tgagattgccctactaaatggacaacatctggaggaagagataactattctaagagagataactcatagattgatg
caaggggtgagaccaccagcggtcccacaaggacctgctcctccaccacctccagcccagcctccagctccgttgc

ctgctccgcctattggacctcctccacctgctgctcctgcacctgctccagggcctatgccagttcctcaacacct
tccaatcacacatatacgtgctgtaatcggagaaaccccgcaaacatcagagaagtccctctatggttggcacgg
gcagtacctgccttacaaggtgtttacccggtccaggacgctgttatgcgcagtcgaacagtgaatgctcttacag
ttcggcatcccgggctcgccttggaacccttggaatgtgggtcatggcaagaatgtctggcagcactatggcaacg
tacttttggagccactgctctccatgcgcttggagacacactaggacaaattgccaattcagatgggatagtgatg
gccatagaactggggttattattctccgatgataattgggatctagtctggggtatttgtcgaagattcctcccgg
gacaagctgtttgtgtagccgttcaggctaggctagacccacttcccgataatgcgacacgaattgtgatgatttc
acacatcatacgtgatgtctacgccatcctagggctagatcccctgggcagacctatgcaacaaactcttcccaga
cgcaacaaccaaccacctagacaacagccgcaacggcgtcaacagccaaggcgtaccggaaaccaagaagaaagag
gtcagaggaatcgtggcagacagaacgcccagactccacgtcaagagggaaacagattacaaaattcacaacttcc
tgggccacgggattgcccaaataattctaatcaacctcgttatcccctcaggcccaatcctcaacagccacaacga
tacggacaagaacagaaccgtgggaacaatccgaacccttatcgacagccaaccccctggaaacgggaaccaaaaca
ggaatttcagccggggtcccgcccccgtcaacgagcaaagtcgcggacgtgggagaagcagccaaggaacaaacaa
cacaggaagttctgcggtccacagtgtgagattaacatctgctgctccacccatacccccacaaggctactgggac
tctggggctgaaatcacttgtgtcccagccatatatataatagaagaacaaccagtgggaaaaaaactgattacta
ccatacataatgaaaaagaacatgatgtgtattacgtggagatgaagatcgagaagagaaaagtacaatgtgaagt
aatagctactgccctggactatgtattggtagctcctgtcgatattccctggtataaaccaggaccactggagctg
actattaaaatagatgtggaatcccaaaaacacaccctgataactgaaagcaccttatctcctcaaggccagatga
gattaaaaaaactgttggatcaataccaagctctatggcaatgttgggaaaaccaggtcggacatcggcgtattga
gccacataagatagccactggggccttgaaacctcgacctcaaaaacaatatcatataaatccaagggcaaaagca
gacatacaaatagtcatagatgatttattacgacaggggtattaagacaacaaaatagtgaaatgaacacacctg
tataccctgtccccaaggctgatggacgctggaggatggtgttagactacagagaagtgaacaaagtgacccctt t
ggtggccactcaaaattgccactcagccagcatactaaataccctgtatcgggggccttataaatctactcttgac
ctagccaatggattttgggcacaccccattaaaccagaagattattggatcactgccttcacatggggaggcaaaa
cctattgttggactgtcctcccccagggattcctaaactctccagcattgtttacagcagatgtcgtagacatctt
gaaggacataccaaatgttcaggtatatgtggatgacgtctatgtgagctcagcgactgaacaagaacatcttgac
atactagaaactatttttaacagactgtccacagctggttatatagtctccctgaaaaagagcaaattagcaaagg
agacagtggaattcttggggttttccatttcccaaaatggcagaggactcacagactcatacaagcagaaactaat
ggatctacagcctccgactaccttaagacaattacaaagcatcttgggacttatcaattttgcaaggaatttctta
cctaattttgctgagctagtggctcctctttatcaactgatacccaaagccaaaggccaatgtatcccatggacaa
tggaccatactacacaactgaaaactataatacaggcccttaattcaacggaaaacctggaagaaagaagacctga
tgtggacctaataatgaaggttcacatttcaaacactgctggatatatacgtttttacaatcatggagggcaaaag
cccatcgcatacaataatgctcttttcaccagtactgaactaaagttcacccctacagaaaagataatggctacca
tccacaagggattactgaaggccttagatctgagcttggggaaagaaatacatgtatattcggctattgcctcaat
gactaagttacagaagactccactctccgagcgaaaagcactatctattcgatggctgaaatggcagacttacttt
gaggatcctcggataaagtttcatcatgatgcaactttacctgacctacaaaatttaccagtaccacagcaagaca
cagggaaggaaatgacaatacttcccctgctgcattatgaggcaattttctacacagatggatcggctataaggtc
acccaagcccaacaagacgcattcggcagggatgggcatcatacaggctaagtttgaaccggactttagaattgta
cacttgtggtcttttccattaggagaccatactgctcaatatgcggaaatagcagcctttgagtttgccataagaa
gagcaactggaataaggggaccagttctgatagtgacagatagtaattatgttgctaagagttataatgaagaatt
gccctattgggagtctaacgggtttgttaataataagaagaagacattaaaacacatcagcaaatggaaagccatt
gctgaatgcaagaatttgaaggccgatatccacgtgatacatgagccaggccaccaaccagcagaagcctctcccc
atgctcagggcaatgctctagcagataaacaagctgtttcaggaagctataaggtcttctcaaatgaattgaaacc
cagcctggatgcagagcttgagcaagtccttagcactgggaggcctaacccccagggctacccaaataagtatgaa
tataaattggttaatggactatgttatgtggacaggagaggagaggaaggactcaagataatcccgcctaaagctg
atagagttaaattgtgccaactcgctcatgatgggcccggatctgcccatctgggacgatcggcactcctattaaa
attacaacaaaaatattggtggcccagaatgcacattgacgcctctcgaattgtgctaaactgcactgtctgtgca
caaactaattcaactaatcagaaaccaagaccacccttggttatcccacatgataccaagccatttcaggtttggt
acatggattatattgggccactccctccctccaatggttaccaacatgctctagtaattgttgatgcaggtactgg
attcacttggatttaccccaccaaggcccaaactgccaacgcaaccgtgaaggccctcacacatctcactggcact
gctgtccctaaggtgctgcactccgaccaggggccagcattcacctcctccatcttggctgattggccaaagacc
ggggtatacaattggaacacagtgctccttaccacccccagagtagtgggaaagtggaaaggaaaaacagtgagat
aaagcggctcttaacgaaactgttggcaggacggcccacaaagtggtatccattgattccaattgtacaacttgct
ctgaacaacactcccaacacaagacaaaaatatacccccatcagttgatgtatggggctgattgtaatcttccct
ttgaaaacctggataccttggaccttactcgtgaagaacaactcgcagtcctcaaagaggtccgtgatggtcttct
ggacttataccct tctccttcacagaccactgcccgctcctggactcccagtcctggcctgttggtccaggagagg
gtagccaggccagcacagctgcgtccgaaatggaggaagcccacacctattaagaaggtactaaacgaacggaccg
ttataatagaccacctaggccaggacaaagtagttagtattgataacttgaaacctgcggcacaccagaaacttgc
acaaactccggattcagcagaaatttgcccaagtgcaacaccgtgtccgcccaacacttctctgtggtatgaccta
gatacaggcacatggacctgccaaaggtgtggttaccagtgtcctgacaaatatcatcaaccgcagtgtacatggt

cctgtgaagatcgttgtgggcatcgttggaaggagtgcgggaattgtattccacagctagagacgaccaacctctt
acagatgaaccctaaaatggagagtgtttgcctaccggactttgatccaccgggtgatgaagaagtctccctcaga
ataaggtgcaaatactggatctatctgtgctgtgccacctccacaagaataatggcatggattgtgttcattctca
cagtactcagcatattgctgatctctgtgttgatagcagtatttaggttacaatggaagggagctattgagagccc
aggtcccattttggtgtggaacaatagtaatagcattaatgctcagcctaccacacctccacactattcccgactg
agaagggcaatacacctggcacaaaagcctgtccaggtgaattttacttctataccacagggcttgttcctggaac
ctcacccaaaacccataatttccaaggagagggtattgggactttcacaagtagttatggtggactctagcactct
cacccagaagttgaaccttgaaggagaagctaaatcactcctcataaaaaccataaatgaagaactgatctcgtta
caagatgtagtccttaattttgacctccccctggggatcctcacacacaagaagaatatatagctaagagatgct
atcaacattttgggcactgctacgtggtacatattccaggggaaaagaatggccaactcgggagataatacaaga
ccagtgccctttaaataattcatggttgactgctttacattatgactatttgccagcctgggattattataatcag
cctccgccgaggcaactgtcattgaaggatttcaggaagtataatattagtaataatggttctagatatgaagcct
ataggctgcccttgcaagacaagataggggctgtagccttctgtagccctaccctatactcctcctggtggaatta
cacacaaagtagcagagaaagagaggaattattccgacgcaagttagaaaccttcctgaatccacagactggctgc
cttaaccccgaggcactcccaggaacctggcacactctaggaaaaggtgaatggtttagagatctaacaacatatg
acttttgcaagaaaccagaggccgtctttggattaaacaagacttattatagctggtcattgtgggaaggagactg
tggaagacaaggaaatgacactcaggactatccaccagaatgtcggaattatgagaaaaaggatggagtacacgtc
tatggttgcaggtattggagaacctattctcaaacagcccataccccagacaacctgagttgttatctgtcagaag
actattgtctgttccaaccaaaatgggacagtgctgaaattagatctgatttaggttatttggcctatttaggagc
ctttcccagcccaatttgtattgaggcaaggaatttaactgatcaggattataaggttacatctatctatgctgag
tgtgtaaagcaagggaagcaatatgatattatagatgtaacacgtcagctgacaagcaagctcacccgaagagggg
tcttcttgggcgacctacctgcagaccgagccttctccctcctgactgacttctcgcttccagccagttatcagaa
taaaacgcaggacggaagacgaaaagtctgttcaagtaaaagaacgaggagaagtataaataactggagaaggctc
cagataacaggacaatcaatgaatcaagccattaccacgctttctaagttgtcagacttgaatgatgaaaatctag
ccgcaggaatacatctcctccaggatcatatcgtcaccttaatggaagcaacacttcatgatgtgtcacttttagg
acacatgactagtattcaacacttacatacccacttagccacttttaagaacctgctaataggaaatcgagtggac
tggtcagtactcgaaaataaatggatacaagaagaactgaaatatacagatgaggttatgaatgtaattcgaagaa
ctgcaagaagcatcacttatgatgtccaaaatgtgaagaataccagtgactcgactatgtgggaaatatatattta
ctatgagcttatactaccagaaagaatttggatccgtaattggcaagtggccaatctaggacatttgacccacaat
tccggataccttacccatgtgactatacatcatccgtatgagattgtgaatcaagactgtgaagaactcactttc
tgcatttagttgattgtcacgaacaagattatttaatatgtgaagaggtgatggaagtggaaccttgcggcaacct
gacaggatctgattgtccagtcttggcagaaaatatacaggctccttatgtttatttacatcctttgaaaaatggg
agttatttactcatggcttcccacactgattgttcactccccccctatgaacctgtagtagtgactgtgaatgact
ctctcgaatgctatggtaaaccacttaaaagaccactaacttctcatactgaaatcaagctgtttgccccacaaat
accacaactgagagtccgacttcctcatttagttgggatcattgccaaactaaagagcttaaaaatcaaggtaaca
agcacttgggaatctataaaagaccagatacacagatcagaacaagagctacttcggttggatctccacgaaggag
attactcagactggatcctgcaacttggaaacgccctagaggacgtctggcctgtagctgcgtctgctgtgagtac
aatcgggaccttactggagaaagctgcaggaactctcttcgggaatgttttcagcatactagcctatgctaaaccg
gtcattattgggataatacttatcattttacttttgttagtcatcagaatattgagatggctagcggtggaacgcc
ggagaaagcaagagtag
>#12 Feline foamy virus (FFV) Y08851 7683 nt
atggctcgagaattaaatcctctccaattacagcaactgtatataaataatggcttacaacctaatccaggacatg
gagatattattgctgtcagatttacaggaggaccttggggtccaggtgatagatgggctagagtgacaatacgatt
acaagataacacaggacaacctttacaggttcctggatatgatttggaacctgggataataaatttgagagaggat
atcttgatagcagggccatataatttaataagaactgcctttttggacttagagcctgccagaggacctgaaaggc
atggtccctttggagatggcagattacagcctggagatggtttatctgaaggatttcaacctatcactgatgaaga
aatacaagcagaagtaggaactattggtgctgctagaaatgagataagattattacgagaagctttacagagatta
caagctggaggtgtgggtagacctataccaggagcagttttacaaccacaaccagtaatagggccggtaataccaa
ttaatcatcttaggtcggttattggcaatactccaccaaatccacgagatgtcgccctatggcttggaagatctac
agccgctattgaaggagtatttcccatagtggaccaagtcactcgtatgagggtagttaatgccttagtagcatct
catcccggcctaacgttgactgagaatgaggccgggagctggaatgctgccatatcagctttatggaggaaagctc
acggtgctgcagctcagcatgaattggcaggagtattaagcgatattaataaaaggaaggcatacaaactgcatt
caacctaggaatgcaatttacagatggaaactggtccttagtatggggaataatcaggactcttttaccaggacaa
gccctagtaaccaatgctcagtcacaatttgacctaatgggagatgatatacaacgagcagaaaatttccccaggg
tcattaacaatctatacactatgctgggtctcaatatacatgggcaaagtattagacctcgggtccaaacacagcc
actacaaactcgaccccggaacccgggacgatctcaacaaggtcaactaaatcagccgagaccccaaaatagagct
aaccaatcttatagacccccctagacaacaacagcaacactctgatgttcccgaacagagagatcagagaggaccgt
cgcaacctcctcgtggaagtggaggaggatataattttagaagaaatccgcagcagcctcagcgctacggccaagg
accaccaggaccaaacccgtaccgacgattcggagacggcggtaatcctcaacagcagggaccaccaccaaaccga
gggcctgatcaaggacctcggccaggaggcaatcccagaggaggaggaagaggtcaaggtccaagaaatggaggag

gaagcgctgccgcagtacatacagtaaaagcgtctgaaaacgaaactaaaggttactgggactcccaagccgatat
tacctgtgttccaaaggacttgcttcaaggagaagaacctgttaggcagcaaaatgtgactactatacatggaacg
caggaaggagatgtatattatgtaaatttaaaaatagacggtagaagaattaatacagaagtaatagggacaactt
tggactatgctattataactcctggagatgtaccttggattttgaagaaacctctagaattgactattaaactaga
tttagaagagcagcaagggactttacttaacaattccattttatctaaaaaagggaaagaagaattaaaacaatta
tttgagaaatatagtgccttatggcaaagttgggagaatcaggtgggtcatagaagaattaggccacataaaatag
caactggtacagtaaaacccacacctcagaaacagtatcatattaatccaaaggcaaaacctgatattcagattgt
gataaatgatttactaaaacaaggggtactaattcaaaaggaaagtactatgaacactcctgtctacccagtaccc
aagccaaatggtcgctggagaatggtactggactacagagcagtaaataaagtcacacctttgatagctgtacaaa
atcaacactcgtatggaattttaggaagtcttttttaaaggtagatataaaactacaattgatttatccaatggttt
ctgggcacaccccatagtcccagaggattattggattactgcattcacttggcaaggaaaacaatattgttggact
gttttaccacaaggtttttaaacagccctgggttgtttactggagatgttgtagatcttctacagggaattccca
acgtggaagtctatgtggacgatgtatatattagtcatgattctgaaaaagaacatttggaatatctggatatttt
gtttaatagattaaaagaagcaggatatataatatctcttaaaaaatccaatattgccaattctattgtggatttt
cttggttttcagattactaatgaaggccggggcctgacagatactttaaagaaaaattggaaaatattactgccc
ctaccactcttaaacaattgcaaagcatactaggtcttttaaattttgccagaaattttattcctgactttactga
attaattgctcctttatatgcattgataccaaagtctaccaagaattatgttccttggcaaatagaacattcaacc
actctggaaactttaattactaaactaaacggggcagaatatttacaaggaagaaaaggagataaaacattgatca
tgaaagtcaatgctagttatacaacaggatatataaggtattataatgaaggggaaaagaagccaatatcctatgt
aagtatagtgttcagcaaaactgaattgaaattcactgaactagagaaattgctgaccactgtgcacaagggtctt
ttaaaggccttggacttgtcaatggggcaaaacattcatgtttattccccattgtatccatgcaaaatattcaaa
aaacaccacagactgctaaaaaggctttggcctctcgatggttgagttggctttcttatttggaagatccgagaat
taggttcttttatgatccacagatgcctgctctaaaagatttgcctgctgtagacaccggaaaagataataaaaaa
catcctagcaattttcaacatatattttacactgatggttctgctatcacgtcccctactaaggagggacatttaa
acgctggaatgggaatagtttattttataaacaaagatggaaatttacaaaagcaacaggaatggtccattagttt
ggggaatcatacagcacaatttgcagaaatagctgcttttgagtttgcccttaaaaaatgtttgcctttgggagga
aacattcttgtggttactgacagcaattatgttgcaaaagcatataatgaggaacttgatgtttggcctctaatg
gctttgtgaataacaggaagaaacctttgaaacatattagtaaatggaaatcggttgctgaccttaaaagattaag
gccagatgttgttgtgacccatgagccaggtcaccaaaaacttgactcatctcctcatgcttacgggaataatctg
gctgatcaactggccacgcaagccagttttaaagtacatatgactaaaaatcccaagctggacattgagcaaataa
aggcaattcaagcatgtcaaaataatgaaagattacctgttggttatccaaaacaatatacctatgagttgcaaaa
taataaatgtatggttttaagaaaagacggttggagggaaattcctccttcccgagaacggtataaacttattaaa
gaagcacataacattagtcatgcaggccgagaagccgtgttattaaaaatacaagaaaattattggtggccaaaaa
tgaagaaagatatatcatctttttctttctacatgtaatgtatgtaagatggtaaatcctttgaatttgaaacctat
tagccctcaagctattgtacacccaaccaaacctttgataaattttatatggattacattgggccattgccacca
tcagaaggttatgtgcatgttttagttgtggtagatgctgccactggatttacttggttgtaccccactaaggctc
aaacctccaaggccacaattaaagttcttaatcatctcactggactagcaattccaaaggtgctgcattctgatca
aggatcagcatttacttctgaagaatttgctcagtgggcaaaggaaaggaatatacaattggaattcagtactcct
taccaccctcaaagtagtgggaaagtggaaaggaaaaacagtgaaattaagaaacttttaactaagctcttggttg
ggaggcctttaaagtggtataaccttatatccagtgtgcaacttgctctaaataacactcatgttgtcagcaccaa
gtatactcctcatcaactaatgtttggaattgattgtaatttaccatttgctaataaggataccttggactggaca
agagaagaagaacttgctctcttgcaggaaattcgtgaatctttacaacaccctgtacaacccccacctgctctg
gttggtcaccatacgttggccagctggtccaggagagggtgtacaggccgtcacaattaaggcctaagtggaggaa
gcctacaaaggtcttggaaatattgaatcctagaactgtgattatagtggaccatctaggccaacggaaatctgtg
agtattgacaatttaaaacctacagcacaccagcatttgcatgttgacatacctgaggatattcctttagtaccag
agaaggtacctttgaaaatgaggatgcgatatagatgttatactttgtgtgctacttctactagaataatgttttg
gatactattctttcttctatgtttttcaatagttaccttgagtacaattataagtattcttagatatcaatggaaa
gaagcaataacacatcctggcccagtcttaagctggcaggtgactaattcacatgtaaccatgggaggaaatactt
cctcttcctccagacggagacgtgatatacaataccacaaacttcccgtagaggttaacatctcagggatcccaca
aggtcttttcttcgcacctcaaccaaaacctatatttcacaaagaaagaactttaggtctttctcaagtgattctt
attgactctgatactattactcaaggtcatattaaacaacagaaagcatatttagtctcaacaattaatgaagaga
tggagcaattacaaaagacagtattaccttttgacttacccatcaaggaccctctaactcaaaaggaatacataga
aaaaaggtgctttcaaaaatatggacattgttatgttatagcttttaatggaaataaagtttggccttcacaagat
ttaatacaagatcaatgtccattacctcctcgctttggaaataacttaaagtataggaaccacactatatggaagt
attatataccattgccatttaaagtatcctccaattggacaagagtagaatcctatggtaatattaggataggcag
ctttaaagttcctgatgaatttagacaaaatgccacacatggaatattttgttctgatgcactatatagtaattgg
tatccacgtgatctaccttcttcggtacaacaatcctttgctcaagcatatataacaaaggtacttatgaaaagga
aaaagcaacctactttacgagatatagcttttccaaaggaattgagccctgtaggctctggtatgctattcagacc
tattaacccatatgatatctgtaatatgccaagagcagtattattattaaataaaacatattatactttctcacta
tgggaaggagattgtggatattaccaacacaatcttactcttcatcccgcatgtaagaacttcaatagaactagac

aagaccatccatatgcttgcagattttggagaaacaagtatgactctgagtcagtgcaatgctataataatgatat
gtgttattatagacctttgtatgatggaactgagaatactgaggattggggatggctggcatatactgactctttt
ccatcccccatctgtattgaagaaaagcgaatctggaagaaaaattatactctgtcatctgtattagcagaatgtg
taaatcaagccatggaatatggtatagatgaagtattatccaaactagatctgatatttgggaatctgactcatca
atcagcagatgaggccttcattccggttaataatttcacttggcctagatatgagaaacaaaataaacaacaaaaa
acctcttgtgaaagaaagaaaggtagaagacaaagaaggtccgtaagtacggaaaacctaagaaggatacaagagg
caggcttaggcctggccaatgcaattactactgtggctaagatctctgacctgaatgatcaaaaattagccaaggg
agtacatttgcttagagatcatgttgtcactctaatggaagccaatttggatgatattgtgtccctaggagaggga
atacaaatagaacatatacataatcacttaacctctttgaaattgcttactttggaaaatagaattgactggaggt
ttataaacgattcatggattcaagaagaattaggtgtttcagataatataatgaaagtaataaggaaaactgcaag
gtgcattccttacaatgtcaaacaaactaggaatctaaatacttccactgcatgggaaatatatttatattatgag
atcatcattcctaccactatatatacacagaattggaatataaagaatctaggtcaccttgtaaggaatgcaggat
atttatctaaggtgtggattcaacaaccatttgaagtactaaaccaggaatgtggaacaaatatatatttacatat
ggaagaatgtgttgaccaagactatataatatgtgaagaagtaatggaacttcctccttgtggaaatggaactggt
tcagactgcccagtgctaaccaaaccacttacagatgaatacttggaaattgaacccctaaagaatgggagttatt
tggttttatcaagtactacagactgtggcataccagcttacgtgcctgtggttataacggtgaatgacacaatcag
ctgttttgataaagagtttaaaaggccacttaaacaggaactaaaagtaacaaaatatgcaccatccgttcctcaa
ttagaactaagagttcctcggttaacaagcctgattgcaaaaataaaaggaattcaaatagaaattaccagcagct
gggaaactataaaagagcaagtcgcaagggccaaggcagagcttctacgcttggaccttcacgaaggagactatcc
agagtggctgcagctccttggagaagcaactaaagacgtttggcctacaatctccaacttcgtttctggaataggt
aatttcataaaggacactgctggaggtatttttggaactgcctttagttttctgggatatgtaaaacctgtacttt
tgggatttgtgataatattttgcataattttaattataaaaatcataggatggcttcaaaatacccggaagaagga
ccaataa
>#13 Simian foamy virus human-isolate (SFV) U21247 8133 nt
atggcttcaggaagtaatgttgaagaatatgaacttgatgttgaagctctggttgtaattttaagagatagaaata
taccaagaaatcctttacatggagaagttataggtcttcgccttactgaaggatggtggggacaaattgagagatt
tcagatggtacgtctaatattacaagatgatgataatgaacctttacagagacctagatatgaggtaatacaacga
gctgtaaaccctcatacaatgtttatgatatcaggaccattagctgaacttcaattagcctttcaggatttagatt
tacctgaaggtccattgaggtttggtccattggcaaatggacattatgttcaaggagatccttatagtagttctta
cagaccagtaacaatggccgaaacagcccaaatgactagagatgaactggaagatgttcttaatactcaaagtgaa
atagaaattcaaatgataaatttattggagttgtatgaagttgaaactagagctcttagaagacaattagctgaga
gatctagtacagggcaaggaggaatatccccaggagctcctcgttctcgaccaccagtaagcagcttctcagggtt
accaagtttgccctctatacctgggattcatcccagggcaccttcacctccaagggcaacttctactcccggaaat
attccttggagtttaggagatgatagcccaccttcatctagttttcctggaccctctcaacctcgtgtttctttcc
atccgggaaatcctttgttgaagaagaaggtcatagacctagatcccagtctagagaaaggagaagagaaattct
tcctgctcctgtaccgtcagcacctcctatgattcagtatataccagtaccacctccaccaccgattggcacggtt
atacctattcagcatatcagatctgtaactggagagcctcctagaaacccaagagaaataccaatttggctaggac
gaaatgctcctgctatagatggagtgttccctgttacaacaccggatctaagatgcagaataattaatgctatact
aggaggaaatattgggctatcattaacccctggagactgtttaacatgggactcagcagtagccaccttatttatt
agaacccatggaacttttccaatgcatcagcttggaaatgtaataaaaggcatagttgatcaagaaggagtggcaa
cagcatatactttgggaatgatgctttctggacaaaattatcaattagtttctggaataattagaggatatttgcc
tggacaagctgtagtaactgcattacaacagcgtttagaccaagaaatagataatcaaacaagagcagagactttt
attcaacatctaaatgctgtatatgaaattttaggccttaatgccagaggacaaagtatacgtgcttcagtgactc
ctcaaccccgaccatccagaggtagaggtcgaggtcaaaatacttctagaccctctcaaggaccagctaatagcgg
gcggggacgacagcgccctgcttctggtcaaagcaacagaggatctagtactcagaatcaaaatcaagataattta
aatcaaggaggatataatcttcgaccccgtacttaccaacctcaaaggtacggaggaggacgtggacgaagatgga
acgataatactaacaatcaagagtccagaccatcagatcaaggttctcaaactcctaggccaaatcaagcaggctc
tggggtgcgtggcaatcagtcacaaactcccagaccagctgctggtcgcggaggaagaggtaaccacaaccgaaac
caacgatcatccggtgctggtgactcacgcgctgtcaataccgtgacacagagtgccacgtcctccacattgttag
cccactgggattcaggggcaacaataacttgtattcctgaaagtttttttagaagatgaacaacctattaaaaagac
tttaataaaaacaattcatggagaaaaacaacaaaatgtttattatgtaacctttaaagttaaaggaagaaaagtg
gaagcagaagtgatagcttctccttatgagtatattttgctgtcgccaacagatgttccttggttaacacagcaac
cacttcagttaacaattttagttcctcttcaagaatatcaagagaaaatcttaagtaagactgctcttccagaaga
tcaaaaacaacaattaaaaaccttgtttgtcaagtatgacaatctatggcaacattgggaaaatcaagtcgggcat
agaaaaattaggccacataatatagcaactggtgattatcctcctcgccctcaaaaacaatatcctattaatccta
aggcaaagcctagtatacaaattgtaatagatgacttattgaaacaaggggtgttaacgcctcaaaatagtacaat
gaatacaccagtgtatcctgttcctaaaccagatggaaggtggagaatggtattagattatagagaagtaaataaa
actattccattaacagctgcccaaaaccaacactctgctggtattttagctactattgttagacaaaaatataaaa
ctaccttagatttagctaatggatttgggctcatcctattacaccagaatcttattggttaacagcatttacctg
gcaaggtaaacagtattgttggacacgtcttcctcaaggattttaaatagtccagcattgtttacagctgatgta

gtagatttactaaaagaaatccctaatgtacaagtgtatgttgatgatatatatttaagccatgatgatcctaaag
agcatgttcaacaattagaaaaagtgtttcaaattttactacaggcaggatatgtagtatctttgaaaaaatcaga
aattggtcaaaaaactgtagaattttaggatttaatattactaaagaaggtcgtggcctaacagacacttttaaa
acaaaactgttaaatattactcctccaaaagacttaaagcaattacaaagcatattaggattgttaaattttgcta
gaaattttatacctaattttgctgaactggtacaaccattatacaatttaatagcctcagcaaaaggcaaatatat
tgagtggtctgaagaaaatactaaacaattaaatatggtaatagaagcattaaacactgcctctaatttagaagaa
aggttaccagaacagagactggtaattaaagtcaatacttctccatcagcaggatatgtaagatattataatgaga
ctggtaaaaagcctattatgtacctaaattatgtgttttccaaagcagaattaaaattttctatgttagaaaaact
attaactacaatgcacaaagccttaattaaggctatggatttggccatgggacaagaaatattagtttatagtccc
attgtatctatgactaaaatacaaaaaactccactaccagaaagaaaagctttacccattagatggataacatgga
tgacttatttagaagatccaagaatccaatttcattatgataaaaccttaccagaacttaagcatattccagatgt
atatacatctagtcagtctcctgttaaacatccttctcaatatgaaggagtgttttatactgatggctcggccatc
aaaagtcctgatcctacaaaaagcaataatgctggcatgggaatagtacatgccacatacaaacctgaatatcaag
ttttgaatcaatggtcaataccactaggtaatcatactgctcagatggctgaaatagctgcagttgaatttgcctg
taaaaaagctttaaaaatacctggtcctgtattagttataactgatagtttctatgtagcagaaagtgctaataaa
gaattaccatactggaaatctaatgggtttgttaataataagaaaaagcctcttaaacatatctccaaatggaaat
ctattgctgagtgtttatctatgaaaccagacattactattcaacatgaaaaaggcatcagcctacaaataccagt
attcatactgaaaggcaatgccctagcagataagcttgccacccaaggaagttatgtggttaattgtaataccaaa
aaaccaaacctggatgcagagttggatcaattattacagggtcattatataaaaggatatcccaaacaatatacat
atttttagaagatggcaaagtaaaagtttccagacctgaaggggttaaaattattcccctcagtcagacagaca
aaaaattgtgcttcaagcccacaatttggctcacaccggacgtgaagccactcttttaaaaattgccaacctttat
tggtggccaaatatgagaaaggatgtggttaaacaactaggacgctgtcaacagtgtttaatcacaaatgcttcca
acaaagcctctggtcctattctaagaccagataggcctcaaaaacctttgataaattctttattgactatattgg
acctttgccaccttcacaggatacctatatgtattagtagttgttgatggaatgacaggattcacttggttatac
cccactaaggctccttctactagcgcaactgttaaatctctcaatgtactcactagtattgcaattccaaaggtga
ttcactctgatcaaggtgcagcattcacttcttcaacctttgctgaatgggcaaaggaaagaggtatacatttgga
attcagtactccttatcacccccaaagtggtagtaaggtggaaaggaaaaatagtgatataaaacgacttttaact
aaactgctagtaggaagacccacaaagtggtatgacctattgcctgttgtacaacttgctttaaacaacacctata
gccctgtattaaaatatactccacatcaactcttatttggtatagattcaaatactccatttgcaaatcaagatac
acttgacttgaccagagaagaagaactttctcttttacaggaaattcgtacttctttataccatccatccacccct
ccagcctcctctcgttcctggtctcctgttgttggccaattggtccaggagagggtggctaggcctgcttctttga
gacctcgttggcataaaccgtctactgtacttaaggtgttgaatccaaggactgttgttattttggaccatcttgg
caacaacagaactgtaagtatagataatttaaaacctacttctcatcaggcgcatgaggcacttcaaaatacaaca
actgtgactgaacagcagaaggaacaaattatactggacattcaaaatgaagaagtacaaccaactaggagagata
aatttagatatctgctttatacttgttgtgctactagctcaagagtattggcctggatgtttttagtttgtatatt
gttaatcattgttttggtttcatgctttgtgactatatccagaatacaatggaataaggatattcaggtattagga
cctgtaatagactggaatgttactcaaagagctgtttatcaacccttacagactagaaggattgcacgttccctta
gaatgcagcatcctgttccaaaatatgtggaggtaaatatgactagtattccacaaggtgtatactatgaacccca
tccggaacccatagtggtgaaggagagggtcctaggtctttctcaaattctgatgattaattcagaaaacattgct
aataatgctaatttgacacaagaagtaaagaagttgttaactgaaatggttaatgaagaaatgcaaagtttgtcag
atgtaatgattgactttgaaattcctttaggagaccctcgtgatcaagaacaatatatacatagaaaatgctatca
agaatttgcaaattgttatttagtaaaatataaagaacccaaaccgtggcctaaggagggccttatagctgatcaa
tgcccattaccaggttaccatgctggattaacctataatagacagtctatttgggattactatattaaagtggaga
gtattagacctgcaaattggacaacaaagagtaaatatggacaagctagactaggaagttttatattcctagcag
cctgagacaaatcaatgttagtcatgtactattctgtagtgatcaattatattctaaatggtataatatagaaaat
accatagaacaaaacgagcggtttctgcttaataaactaaataaccttacatctggaacctcagtattgaagaaaa
gagctcttccgaaggattggagttctcaaggtaaaaatgctctgtttagagaaatcaatgtgttagatatctgcag
taaacctgaatctgtaatactattgaatacttcatactattccttctctttatgggaaggagattgtaattttact
aaagatatgatttctcagttggttccagaatgtgatggattttataacaattctaagtggatgcatatgcatccat
atgcttgtagattctggagaagtaagaagaatgaaaaagaagaaactaaatgtagagatggggaaactaagagatg
tctgtattatcctttatgggacagtcccgaatctacatatgattttggttatttagcataccaaaagaattttcct
tcccctatctgtatagaacaacagaaaattagagatcaagattatgaagtctattctttgtatcaagaacgcaaaa
tagcttctaaagcatatggaattgatacagttttattctctctaaagaattttcttaattatacaggaactcctgt
aaatgaaatgcctaatgcaagagcttttgtaggcctaatagatcccaagtttcctccttcctatcccaatgttact
agggaacattatacttcctgtaataataggaaaagaagaagtgttgataataactatgctaagttaaggtctatgg
ggtatgcacttacaggagcagtgcaaaccttatctcaaatatcagatattaatgatgaaaacttacagcaaggaat
atatttattaagggatcatgtaataaccttaatggaagctacattgcatgatatatctgttatggaaggaatgttt
gctgtacaacatttgcatacacatttgaatcatttgaagacaatgcttctagaaagaagaatagactggacctata
tgtctagtacttggctacaacaacaattacagaaatctgatgatgagatgaaagtaataaagagaattgctagaag
tttggtatattatgttaaacaaacccatagttctcccacagctacagcctgggagattggattatattatgaattg

gttatacctaaacatatttacttgaataattggaatgttgtcaatataggtcacttagttaaatcagctggacaat
tgactcatgtaactatagctcatccttatgaaataatcaataaggaatgtgtagagactatatatctgcatcttga
ggactgcacaagacaagattatgtcatatgtgatgtggtaaagatagtgcagccttgtggcaatagctcagacacg
agtgattgtcctgtctgggctgaagctgtaaaagaaccatttgtgcaagtcaatcctctgaaaaacggaagttatc
tggttttggcaagttccacagactgtcagatcccaccatatgttcctagcatcgtgactgttaatgaaacaacgtc
atgctttggactggactttaaaaggccactggttgcggaagaaagattgagctttgagccacgactgccaaatcta
caactaagattaccacatttggttggaattattgcaaaaatcaaagggataaaaatagaagtcacatcctctggag
aaagtataaaagagcagattgaaagagcaaaagctgagctccttcgactggacattcacgagggagatactcctgc
ctggatacaacagctagctgcagcaacaaaggacgtctggccagcagcagcttctgctctacaaggaattggtaac
tttttatctgggactgcccaaggaatatttggaactgcctttagtctcttgggatacttaaagcctatcctaatag
gagtaggggtcattctcttggttattcttatatttaagattgtatcatggattcctacgaaaaagaagaatcagta
g
>#14 Bovine immunodeficiency virus (BIV) M32690 6963 nt
atgaagagaagggagttagaaaagaagcttcgtaaggttagggtgacaccccaacaggataaatattatactatag
ggaatcttcaatgggccattagaatgataaatctaatggggatcaaatgtgtgtgtgacgaggagtgctcggcagc
agaggtagcccttatcataacccaattttcagctttagacttagaaaattctcctatcagaggtaaggaggaggtg
gccataaaaaatactctgaaggttttctggtccctgctggcggggtacaaaccagagagtacagaaacggccctag
gatattgggaggcctttacatatagagaaagggaggccagagctgataaggaaggcgaaattaagagtatttaccc
ttccctaacacagaacacacagaataagaagcagacatcgaatcagacaaacactcaatcattaccagctatcact
actcaagatggtactcctaggtttgatcctgacctcatgaagcagcttaagatctggtcagacgccactgaaagaa
atggggttgaccttcatgcagtgaatatattaggggtcattacagcaaacctagtacaggaagaaattaaactcct
cttgaatagtacacccaagtggagattagatgtacaacttatagaatcaaaagtaagagagaaagaaaatgcccac
agaacgtggaaacagcatcatccagaagccccaaaaacagatgaaatcatcggtaaggggcttagttctgctgaac
aagccaccctgatctcagtagaatgcagagaaactttcagacagtgggtgctgcaggcagctatggaggtggcaca
ggcaaaacatgctaccccaggtcccatcaacattcatcagggacccaaggagccgtacacagactttataaataga
ttagtggcagcccttgaaggtatggcggctccagaaaccacaaaagaatacttactccaacatctatctattgatc
atgccaatgaagactgccagtctattctaagacctttgggacccaacaccccaatggagaaaaaattagaagcatg
tagggtagtgggatctcagaaatcaaagatgcaattttggtagcagctatgaaagaaatggggatccaatcacca
attccagcagtcttgcctcacacaccagaagcatatgcctcccaaacctcagggcccgaggatggtaggagatgtt
acggatgtgggaagacaggacatttgaagaggaattgtaaacagccttttataaaggtgttcataggggaagatg
ggtaaaagggttagtagacactggagcagatgaggtagtgcttaagaacatacattgggataggataaaagggtat
ccagggacaccaattaaacaaattggggtaaatggagtaaatgtggccaaaaggaagacccacgtagagtggagat
ttaaggataagactgggataattgatgtcttgttctcagatactcctgtaaacctttttgggagatctcttctacg
tagcatagtgacttgcttcaccctacttgttcacacagaaaaaatcgaacccctacccgtcaaggtaaggggacca
gggcctaaggtaccccagtggcccttgacaaaagaaaagtatcaggctcttaaggaaattgtgaaagatcttttag
cagaaggaaaaatttccgaagctgcttgggataacccatataataccccagtttttgttataaagaaaaagggaac
gggaagatggaggatgctaatggattttagggaattaaataagataacagttaaaggacaagaattctctacaggc
ttaccttaccctccaggaattaaggaatgtgaacacttaactgcaatagatataaaagatgcctactttactatcc
ctttacatgaggactttagaccctttacagccttctctgtagtccctgtaaatcgagaaggacctatagagaggtt
ccagtggaatgttctaccacaaggatgggtatgtagccctgccatttatcagactaccacccagaagattatagaa
aacattaaaaagagtcacccagatgtcatgttgtatcaatatatggatgatttgttgattgggtctaatagggatg
atcataagcaaatagtgcaggaaatcagggataagttaggatcatatggtttcaagactccagatgaaaaggtcca
ggaagagagagtgaaatggatcggttttgagctcacacccaagaaatggcgttttcagcccaggcaactaaagata
aaaaacccactcacagtaaatgaattacagcaattagtaggtaattgtgtttgggtacagccagaagtaaaaatcc
ctctataccccttaaccgatctactgagggataagaccaatctccaagaaaagatacaactaacaccagaagccat
caagtgtgtagaagaattcaatctaaaactaaaagatccagaatggaaagatagaataagagaaggagcagaatta
gtcataaaaatacagatggttcctcggggcatagtatttgatctgttgcaagatggaaatcccatatggggaggag
taaaaggactaaattatgatcattcaaacaaaataaaaaagatacttagaactatgaatgagctgaacagaacagt
ggtaattatgacaggaagagaagctagtttcctgcttcctgggtcttctgaagattgggaagcggcactccagaag
gaagaaagtctaacacaaatattcccagtaaagttttataggcactcctgcagatggacctccatatgtgggccag
taagagaaaatctaaccacctactatactgacggagggaagaaaggaaaacagctgcagcagtatattggtgtga
aggaaggactaagtcaaaggtatttccaggaaccaatcaacaggcggaattgaaggccatatgcatggctctcttg
gatggaccaccaaaaatgaatatcataacagatagtagatacgcctatgagggaatgagagaagaaccagaaacgt
gggccagggaaggaatctggctggagattgccaagatattgccctttaagcagtacgtgggggtcgggtgggtgcc
tgcacataaagggataggaggaaatacagaggcagatgaaggagttaagaaagccttagaacagatggccccgtgt
agccctcctgaggccattctattaaaaccaggagaaaaacaaaatctggagacagggatctacatgcaggggctta
gaccacaaagcttcctcccaagagcagacttaccagtagccatcacaggaaccatggtagattcagagctacagct
acagctacttaacataggaactgagcatataagaatccaaaaagatgaggtcttcatgacctgtttcctagaaaat
atcccctcagccactgaagatcatgagagatggcatacctcaccagacattttggttaggcagttccatctcccta
agagaatagctaaagagatagtagccagatgccaagaatgtaaaaggacaaccactagcccagtcagaggaacaaa

ccccagaggtcgattcttatggcagatggacaatactcactggaataaaacaattatttgggtagcagtagagaca
aattcaggattagtggaagctcaggtgatccctgaagaaacagcactacaagtagctctctgcattttacagctaa
tccagagatatacagttcttcacttacatagtgacaacgggccgtgctttactgcacacaggatagaaaatctatg
taagtatctggggatcacaaaaactacgggaataccctacaacccacaatcccagggagttgtagaaagagcccac
agagatctaaaagacagattggcagcttatcagggagattgtgaaaccgtagaagcagcccttagcctcgcattag
tttctttaaataaaaaaagaggggaataggggccatacaccatatgaaatatacctagaatcagaacataccaa
ataccaagaccaactagaacaacaattttcaaaacaaaaaattgaaaagtggtgttacgtaaggaacagaagaaag
gaatggaaaggaccctacaaagtgttgtggacggagacggggcagcagtaatagaggaagagggaaaaacagcct
tatatccacaccgtcatatgcgcttcatcccccccccagattcagatatccaagatgggagttcgtgaatggatca
ggacctagacggcgcggaacgcggggaaaggggaggaggatccgaagaactgcttcaggaggagatcaacgaaggg
aggctgacagccagagaagctttacaaacatggatcaataacggtgagatccacccttgggtcctggcaggaatgc
tgtccatgggagtaggaatgctactaggagtatattgtcagttaccagacacactgatttggatactaatgtttca
attatgcctttattggggtttgggtgaaacatctagagaattagacaaggatagttggcagtgggtcagaagtgta
tttataatagcaatattgggaactctcactatggcaggaactgctttggccgacgacgatcaaagtactttaatcc
ccaatatcacaaaaattcctacaaaggacacggaacccggttgcacctatccgtggatattaatcctcttgatttt
ggctttcatactgggaattctgggtataatacttgtcttgagacgcagcaactcggaggatatattggcagccaga
gataccatagattggtggctctcagctaatcaggaaatacctccaaagtttgctttcccaataatattaatatctt
cccctctagcaggcataataggatattatgtcatggaaaggcacttagagatcttcaaaaaggatgtcaaatttg
tgggagcctgagcagcatgtggggaatgcttttggaagaaattggcaggtggctcgcacgtagggaatggaatgtt
agtagagtaatggttatcctcttaatcagcttcagttggggaatgtatgtcaatagggtaaatgcctcagggtcac
atgtagccatggtcaccagccctccagggtaccgcatagtgaatgataccagccaggcaccttggtattgcttctc
ctcggcaccaatcccaacgtgtagttcctctcagtggggagacaaatattttgaggagaaaataaacgagacactg
gtcaaacaggtgtatgaacaggccgcgaaacattcgagagccacatggattgaacctgatctattggaggaagcag
tctatgagctagctctgttatcagctaatgacagtcgtcaggtggtggtagaaaatggtacagacgtatgtagctc
acagaactcgagcacaaacaaaggccacccaatgacgcttctaaagttgagagggcaggtgtcagaaacttggata
gggaattcctccctccagttttgtgtccagtggccatatgtcttggtaggtcttaataatagtgatagtaatatta
gcttcaattcgggagattggatagcaaccaattgtatgcacccaattacactaaataaaagtgcacaagatctagg
aaaaaatttccgagactaacatttcttgacggacaactgtcccagttgaagaacacactgtgcggacataacaca
aactgtttgaaatttggaaacaagtccttcagtacaaattccctaatactatgccaagacaaccccatcggcaacg
acacctttatagcctaagtcattccttctcaaaacaggcctctgcccggtggattcttgtaaaggtccccagcta
tgggtttgtggtagtaaatgacacagatacaccaccatccctccgcatccgaaagcctcgagcagtcggactagca
atattcctgcttgtgctggctatcatggccatcacatcctccttggtggcagctacaacgctcgtgaaccagcaca
cgacggctaaggttgtggagagggttgtgcaaaatgtgtcatatattgctcaaacccaggaccaattcacccacct
gttcaggaatataaacaacagattaaatgtcctacaccatagagtttcatacttggagtatgtagaggaaatcaga
caaaaacaagtattctttggttgcaaacctcatggaaggtattgccactttgactttggaccagaggaagttggat
ggaacaatagttggaatagcaaaacttggaatgatctacaagatgagtatgataagatagaagaaaaaatattaaa
aattcgagtggactggctcaatagctccctgagtgacacacaggacacctttggcctggagacctctatttttgac
catttagtgcaattgtttgattggacttcttggaaagactggataaaaatcattatagtaatcattgtactttggc
ttctgataaagattctcctaggtatgttaagaagctgcgccaaggtcagccagaattaccaacatctcccggcgga
ggaggaggacggggacacagagccagaaagctccccggcgagaggagacccggcttctggaagtctctacgagaat
tggttgaacaaaataggagaaagcaagaacgacgcctatcgggtctggacagaagaatacaacagcttgaggatct
tgttcgccacatgtcgctgggatctcctgacccctcaactccttcagcttccgttctttctgttaaccctcctgct
caaactcctttgggacatcttccgccacgctcctattttaaacttaaaagggtggactgtggggcagggtgggacc
tcaggacaacagcagcccccggacttcccatatgtgaattggactggatccagggaacaaaataacccagaagggg
gattagactctggggcttggtatgaaggcctgagaggttctcagtag
>#15 Feline immunodeficiency virus (FIV) FIU56928 7128 nt
atggggaatgagcagggtaaagaagtgaaggctgcagtcaagagatgtaaagaagtagctgtaggtccggggagtaagag
caaaaaatatggagaaggaaatatcagatgggccataagaatggcaaatgtaactacaggacgagaccctggtaaattgc
cagaaaacatagcacaggtaagaaatttagtatgtgatttaatggaaataagagataagtatggcagcaataaggaaata
gaggccgccataaaaactttaaaagttttaggagtagtgggaattctgtttatgaaggcttctaatacagactcagcagt
aaatttatgggaaataatgggattaaattcaagaccctcagaaaaaggaccaggaggagaggaagaagcaatgccatcag
cttttcaagccaaagagcagaaaggggtaggattaagagatccacaagatattgcaaaagaatatcctatacaagttgtt
aatggacaggctcaatatgttccattgaatccaagaatggtagcaatctttatggaaaaagctagagatggattaggaac
agaagaagttctgttatggttcacagcattttcagcagacttaacacctacagatatggcaacaatattaatgtctgctc
ctggttgtgctgcagataaagaaataattgatacaaaattaaaagaattaactacagaatatgaaagaacacacccctca
gatgctccaagaccattaccttattttacagcaagggagataatgggattggatttgacacaagatcagcaagcacaacc
tcaatttcatgcaggaagagtacaagcaagagcttggtatatagaagcattgcaatatttacaaaaaattaaatcaagaa
gtcctagagcagtgcaaatgaaacaaggtccaaaagaggactatgcaagctttatagatagattatatgctcaaatagat
caagaacaaaatagtccagaagtaaaaatatatttgaaacaatcattaagtttagcaaatgctaatcccgagtgcaaaaa
agccatgtctcatttaaaaccagagagcactctagaagaaaagttgagagcatgtcaggaggtgggatcaacatcctata

aaatgaatatgttagcacaagctttacaacagcaaagtcaagtatgtcaagtacagcaaggaagaggaaagccacaagga
aacaatagaagacctggccagtctttgaaatgtttcaattgtggaaaaccaggacatttagcaaggaattgtagagcacc
tagaaaatgtaatagagatatagaattaatacatagaccaagaattttgatctatgtaaatgggattcctataagatttt
taatggatacaggagcagatataactataatgaatgcagaagattttaatatattaaattcaatcccagatggaatacaa
acaatgataggagtaggtggaggaaaaagaggtagaaaatttagacgagtacatttagaaataagagatcctaatcatag
agctcaatgtttatttggaaatatgtgtatcttagatgacaatagtttaacagaacctctgctagggagagataatatgg
ttagatttggagcaaagttggtaatggcaaatatttcaaataaaattcctatagtaaaagtgaaaatgaaagatcctagc
aaaggaccaaaaattaagcaatggcctctatcaaaagaaaagatagaagcattaacagaaatagtttatagattggaaaa
agaagggaaagtaaaaagggcagatccaaataatccttggaataccccctatttctgtataaaaaagaaatcagggaagt
ggagaatgttaatagattttagaactctgaatgaattaacagaaaaaggtgcagaagttcagttgggactccctcatcca
gcaggattacaagaaaggaaacaagtaacagtattagatattgcagatgcatattttactataccattagacccagacta
tgcaccatatactgcctttactctgcccaaaataaataattcaggtccaggagaaagatttgtatggtgtagtttacctc
aaggatgggtattaagtcccttaatttatcagagtacattaaacaatattttaaaccatttagagaacagcatccagaa
atagatttataccaatatatggatgatatatatataggatcagatttaggaaagaaggagcataaacaaattgtagagga
attaagaaaattattattatggtggggatttgagacgccagaagacaaattacaggagcaaccaccttataaatggatgg
gatatgaattatatcctcggaaatggactatacaaacaaaagaattaataataccagaagaaccaactcttaatgagtta
cagaagttagtaggaataataaattggtcatctcaaataattcctggattaagaattaaggctttaactaatatgatgaa
aggaaatcaagctttagattcaaaaagaaggtggacagaagaggctaagaaagaggcagaagaggcaaaattggcaatag
aacaacacacacaattaggatattatgatcctcaacaacaattacatgcaaaattgagtatagtgggtccacattgtata
gggtaccaagtttatcaaaaagggtctccagataaaatattatggtgtggaagaatgaatagacaaaagaaaaaagcaga
aaatacttgcgacatagccttaagagctatatataagatcagggaagaatcaatagtaaggttaggaaaagaacctattt
atgaaataccatgttctagagaagcatgggaatcaaatttgattaatactccttatttaaaagcttgcccaccacaagta
gagtatattcatgcagcaataatgatacagaggtctttaagtatgataaaagaagaaccaattagaggtgcagaaacatg
gtatattgatggaggaaggaagaagggacaatcagcaaaggcggcatattggactgataaaggaaaatgggaagtaatgc
aaatagaagggagtaatcaaagagcagaggtaatggccctattaatggcattacgatcagggggagaagaaatgaatatt
gtaacagattctcaatatatcctaaatattttgagacaaaaaccagatttgatggagggattatggcaagaaatattgga
agaaatagaaaagaaggtagcaatttttatagattgggtaccaggtcataaaggcattcctgggaatacagaagtagata
acctatgtcaaacaatgatgataatatcaggaaatggaatattagataaaagagaagaggacgcaggatatgatttgctt
gcagaacaagacatacatttaatgccaggagaagtaagaatagtccctacaggagtaagattaatgctgccaaaggaca
ttggggaatggtagtaggaaaatcttcaattgcaaagcaaggattggatgttcttggaggagtaatagatgaaggataca
gaggggaaataggtgtaattatgataaatttacagaaaagatctattactttaaaagaaaagcaaaaggtagcacaatta
ataatcataccttgtaaacatgaagaattgaaacaaggggaaatagaattaaattcagaaagaggagaaaaagggtatgg
atcaacaggtgcatttgcatcttggatgaataacattgaagaggcagaaatcaaccatgaaaaatttcattcagatccag
aatttttaaggactgaatttgggcttcccaaacaagttgcagaagaaataaaaagaaaatgtcctctatgtatagtgcaa
ggggaacaagtaatgggaaaattaaaagtaggaccaggaatatggcaaattgattgtactcatttagaaggaaagattat
actggtcgcagtaaacacagaatcaggatacatttgggcaagaataattcctcaagagacagcagatatgacagtaaaat
atctattacaattaatctcggagcatcatgtgactgaattacaatcagataatggaccaaattttaataatgcaaaagta
gaaggcatgacaggattttgggaataaaacataaatatggaattccaggaaaccctcaatcacaagccttggtagaaaa
taccaatagaatgttaaaagaatggataaagaaatttagaggggaagtaactactttggatgcagcattggcacttgcac
tttatgctcttaactttaaacaaaggggtagaatagggagaatatccccatatgagttacttatacagcaagaatcagac
agaataagagattacttttctaaaataccagcaaataatataaaaaattcttggatttattataaggatagaagagataa
agaatggaagggtccaacacaggtagaatattggggacaaggagcagttttaataaaacatccagagcatgggtatatgc
tcatccctaggagacacataaggagagttccagaaccctgtactcttccagaagtgatggcagaaggaggaagagtagat
gtagtagaaagagcagatgaagaactagggagacaaggagtagaagggcatgaatatgcatttgggatgaatccagattg
gatcggtccttatgagggagagatgttattggattttgatatccttcagtatgtaacagaagaaggaccattcaggccag
gacacaaccctttttagagctcccggaataacggagcaagaaagacaagagttatgtgttatgttacaagacaagctaaaa
gagataaaagggaccataacagaaggacctcacaaaatacctccaggtaagtataggagattaagatatttgcagtattc
agacatgcaggtaacgcagagtctggctttattagtctttgatattagtcactatcttaggaataagttaggaaaagaag
tatatgatatagaaggagatagacaggcagaatataaatttgaaaaaagggttaaaggacgaacttacaataactgtaga
tgtagattacttcttataggtgcaggattcttctatacttgtcttataatagggttgggatgtctcattagagaaacatc
aggagtgatattggcattggatcctccttgggtgattccggtaacaaagatggatgaaataaattttcaatgtcatggaa
attatgaggagtgtccagtgttagagtcggtagcaacctggaagacagattttcaatggaattatagtagaccttttaat
gaaaccataggattagagcaatatgtagatcagatacaagcaaaagcgcttcaagatttacttggatcctgtcaaaagct
atcaaaaaataaattaggggttcttcaatggagatgcttctacgatagaggtatgaagcaactattaggattacaaaaaa
taaggatttgtccaataggaggatatatgttagttaggaaaatagatgaaaataactatactttaagcatgtgcacagag
gaaatagatattaaaatattaaatatgactctaagtcaggaaaaatatgagcattatccatttaatgatattgtttggat
gggaaacaggtattttaatatgacaacagcaaatataactcaacaacaagtaaatataagtataaaatgtgatattatag
tgcctacagtagttaaagtaaagaaagaatttgcaggatacaataatgatttcttgggaccatggggaggattaaagtat
aggtctattcttattaggtataaagattggcaaatgttacagatcccccgttagatttaaattgtactggactacctgg
aatagcacttaatggaacagaagcaaattatacttgtgctcaaaatgctacaattacctacggagatatttgtacacaac
cagaattgtatgtaccatgttatagtccaaattattcaatgcctgtgatggttcaatgtaaattgcatcaagaatatcat
cctaatgatacctatagaaatagtagcaatgatatgcaagtaatgaggtgtagaataatgaaagaggtagaattaagatt
tggggatgaatttatctcattaaactttacattgttaagagatcctttttggctcatttgagggggctataaattta

cttgtaatttgacaggacaattttgggcttataaatttaataatgctacttggggatatgaaggtaatggatcagcatgg
aatgaatctcttaattggttagtgccttataggaactataccaaagaaatgtatgtatgggggcatactctgctataaa
ttataatcatattttgttaaaagattataaacttgttaaaaaaccgttatatactccattaaaatacttaccaccaagaa
agaaaagaggattaggattaactctagctcttgttactgctacaactgcagggttaataggaacaacaacggggacatct
gcactggcagtgtcattaaaattaaaagaagtgatgttacaacaatcacaaataaatgaagcaacattgggaatgttaaa
aatcttacaaagaagactaaaacaggcagaaagagtgattttaacgttacatcagagagtatctaggatagaaagatatt
tagaaattcaatatcagttaagaggaatgtgcccatttaaagacatctgtgagataccggggaatggtaattttacaaat
tataatgattcttgggcaataggtagatgggcagaacaagcagaagaagactggcagcaatttggaacaattgttaaaca
atgcaacagaacaaatgaaaatttgaaaaatgatttagagaagttgagtatagattcctggttatcatggaatccattag
ggaatgtgttccaaatgttaatcacactgataattataattggaatggggtaatattgaaaggatgtatattaaactgt
tgtaaaatcttaatggctagtatgggatataaaagagtagcagaagaaatggtgatattaccagatagtgaattagatag
tgaatcagaaatagaattaaatgtgactgagaaagaaaagaagcccatggtaaattctggaaaggaggagtctgatgagg
aattctga
>#16 Caprine arthritis encephalitis virus (CAEV) M33677 7236 nt
atggtgagtctagatagagacatggcgaggcaagtctccgggggaaaagagattatcctgagctcgaaaaatgta
tcaagcatgcatgcaagataaaagttcgactcagaggggagcacttgacagaaggaaattgtttatggtgccttaa
aacattagattacatgtttgaggaccataaagaggaaccttggacaaaagtaaaatttaggacaatatggcagaag
gtgaagaatctaactcctgaggagagtaacaaaaaagactttatgtctttgcaggccacattagcgggtctaatgt
gttgccaaatggggatgagacctgagacattgcaagatgcaatggctacagtaatcatgaaagatgggttactgga
acaagaggaaaagaaggaagacaaaagagaaaaggaagagagtgtcttcccaatagtagtgcaagcagcaggaggg
agaagctggaaagcagtagattctgtaatgttccagcaactgcaaacagtagcaatgcagcatggcctcgtgtctg
aggactttgaaaggcagttggcatattatgctactacctggacaagtaaagacatactagaagtattggccatgat
gcctggaaatagagctcaaaaggagttaattcaagggaaattaaatgaagaagcagaaaggtggagaaggaataat
ccaccacctccagcaggaggaggattaacagtggatcaaattatgggggtaggacaaacaaatcaagcagcagcac
aagctaacatggatcaggcaaggcaaatatgcctgcaatgggtaataaatgcattaagagcagtaagacatatggc
gcacaggccagggaatccaatgctagtaaagcaaaaaacgaatgagccatatgaagattttgcagcaagactgcta
gaagcaatagatgcagagccagttacacagcctataaaagattatctaaagctaacactatcttatacaaatgcat
cagcagattgtcagaagcaaatggatagaacactaggacaaagagtacaacaagctagtgtagaagaaaaaatgca
agcatgtagagatgtgggatcagaagggttcaaaatgcaattgttagcacaagcattaaggccaggaaaaggaaaa
gggaatggacagccacaaaggtgttacaactgtggaaaaccgggacatcaagcaaggcaatgtagacaaggaatct
cagcaccacctatggttcaggtccgcataggttcccagcagaggaacttgttatttgataccggggcggaccgaac
tatagttagatggcatgagggctcgggaaacccagccggaaggataaaactgcaaggaataggaggaatagtagaa
ggagaaaaatggaataatgtagaattagaatataaaggagaaacaagaaagggaacaatagtagtgttaccacaaa
gtccagtagaagtattaggacgagataacatggcccgatttggaataaagataataatggcaaatttagaggaaaa
aagaatcccaattacaaaagtaaaattgaaagagggatgtacgggtccacatgtcccacaatggccattaacagaa
gagaaattaaaaggtctaacagaaatcatagataaattagtggaagaaggaaaactaggaaaggcacccccacatt
ggacatgtaatactccaatcttttgcataaaaaagaaatcagggaagtggagaatgttaatagatttcagagaatt
gaacaaacagacagaagatttaacagaagcgcagttaggactcccgcatccgggaggactacaaaagaaaaaacat
gttacaatattggacataggagatgcatattttactataccccctatatgaaccatatcgagagtacacatgtttta
ctctattaagtcctaataatctaggaccatgtaaaagatactattggaaagtgctgccacaaggttggaaattgag
tccatctgtatatcaatttactatgcaggagatcttagaggattggatacagcagcatccagaaattcaatttggc
atatatatggatgatatttacataggaagtgatttagaaattaaaaagcatagagaaatagtgaaagatttagcca
attatattgcccaatatggattcactctgccagaagagaagagacaaaaggatatccagcaaaatggctaggatt
tgaactacacccgcagacctggaaatttcagaagcatacattacctgaattaacaaaggaacaataacattaaat
aaattacagaaattagtaggagaattagtatggagacaatccataattgggaaaagcattcctaacattctgaaat
taatggaaggagatagagaattacaaagtgaaagaaaaattgaagaagtacatgtgaaagaatgggaagcatgtag
gaaaaaattagaagaaatggaaggaaattattataataaagacaaagatgtctatggacaattggcttggggagac
aaagctatagaatatatagtgtatcaggagaaagggaaaccattatgggtaaatgtggttcacaatataaagaacc
taagcatcccgcaacaggttattaaagcagcgcaaaaattaacccaagaagtcatcattaggacaggaaaaatacc
atggatattgttgccagggaaagaagaagattggagactagaattgcaattagggaacatcacatggatgccaaaa
ttttggtcctgttatcgaggacatacaagatggagaaaaagaaatataatagaagaagtagtagaagggcctacat
attatacagatggaggaaaaaagaataaagtaggaagtctagggttcatagtatcaacaggggaaaaatttagaaa
gcatgaagagggcacaaaccagcaactagaattaagagccatagaggaagctctaaaacaagggcctcaaacaatg
aatttagtaacagatagtagatatgcatttgaatttttattaagaaattgggatgaagaagtaataaagaatccaa
ttcaagcaagaattatggaaattgcccacaagaaagataggataggagtgcattgggtgccaggacataaagggat
tccccaaaatgaagaaatagacaaatatatttcggaaatatttcttgcaaaagaaggagaaggaattctcccaaaa
agagaagaggatgcagggtatgatttaatatgcccagaagaggttaccatagagccaggacaagtgaaatgcatcc
ccatagagctaagattaaatttaaagaaatcacaatgggctatgattgctacaaaaagcagcatggctgccaaagg
agtgttcacacaaggaggaatcatagactcaggatatcagggacaaatacaggtaataatgtataatagcaataaa
atagcagtagtcataccccaagggagaaaatttgcacaattaatattaatggataaaaagcatggaaaattggaac

cctgggggaaagcagaaaaacagaaggggagaaaaaggatttgggtctacaggaatgtattggatagaaaatat
tcctctggcagaggaagaccacacaaaatggcatcaagatgcccgatcattgcatctagaatttgaaattccaaga
acagcagcagaagacatagtaaatcaatgtgaaatatgcaaagaagcgaggacacctgcagtaattagaggcggaa
acaaaagggggtaaatcattggcaagtggattatacccattatgaaaatatcatactattagtatgggtagaaac
aaattcaggactaatatatgcagaaaaagtaaaaggagaatcagggcaagaattcagaataaaagtgatgcattgg
tatgcattatttggtccagagtcattgcagtcagacaatggacctgcatttgcagcagagcccacacagctgttaa
tgcaatacctaggagtaaaacacacaacaggcataccttggaatccacagtctcaggctatagtagaaagggcaca
tcaactattgaaaagcactttaaagaagttccagccacaatttgtcgctgtagaatcagccatagcagcagcccta
gtcgccataaatataaaaagaaagggtgggctggggacaagccctatggatattttatatataataaagaacaga
aaagaataaataataaatataataaaaattctcaaaaaattcaattctgttattacagaataaggaaaagaggaca
tcaggagagtggaaaggaccaacccaggtactgtggaaaggggaaggagccaattgtggtaaaggatatagaaagt
gaaaagtatttagtaataccttacaaagatgcaaaattcatcccgccaccaacaaaagaaaaggaataaatggatg
ctggggccagatacatgcgcttaactgggaaggaaaactgggttgaagtaaccatggacggagagaaggaaaggaa
aagagaaggtttcactgcgggacagcaaggtaagtatcaaccccaggtaagtaagcaaatagggaacagaaatact
aacccatgctttgcctataaagggatattcctatggaggatatcactaacaatgtggatattgctagggataaata
tgtgtgtcagtgcagaggattacataacactaatatcagatccctatgggttctcacccataaaaaatgtgtctgg
ggtaccagtgacttgtgtaacaaaagaattcgcaaaatggggatgtcaaccactaggagcgtaccctgatccagaa
atagaatacagaaatgtgagtcaggaagtagtgaaagaagtatatcaagagaattggccatggaatacatatcatt
ggcctctctggcaaatggagaatgttaggtactggttaaaagaaaatatgcaagaaaatcaacagagaaaaaataa
tacaaaagagggtatagaggaattattagcaggaactataaggggaagattctgtgtaccatacccatttgccttg
ttaaaatgcacaaagtggtgctggtatacagcggccataaacaacgagtcaggaaaagcaggaaaaataaaaataa
attgcacagaagcaagagcagtctcctgtacagaggacatgccattagcctcaatacaaagagcatattggatga
gaaagacagagagagcatggcctttatgaatatcaaagcatgtgatagcaacctaaggtgtcagaaaagacctgga
gggtgtatggaaggataccctatcccagtaggagcagaaataatccctgaaagtatgaaatacctaaggggagcaa
agagtcagtatggggaataaaagataagaatggagaattaaaattaccattaacattaagagtgtgggtaaaatt
agcaaatgtgtcagaatgggtaaatggacacccccggattggcaagacagaattaacggatccaaaggaataaat
gggacgctctggggagagcttaacagtatgcatcacctaggatttgcccttagccagaacggcaaatggtgtaact
acaccggggaaataaaattagggcaagaaacattccaatatcattacaagccaaactggaactgtaccgggaattg
gacgcaatatccggtgtggcaagtgattagaaacctggatatggtggaacatatgacaggagaatgtgtgcagaga
ccacaaaggcacaatataacagtaggaaatggaaccataacagggaattgcagtacaacaaactgggatggatgta
attgctcacgatcaggaaactacctatataacagctctgagggaggattgttattaattctgtgcagacaaaacag
caccctaacaaggatcctgggaacaaatacaaattggacaactatgtggggaatatacaaaaattgttcaggatgc
gagaatgcaacattagacaacacaggagaaggaaccttaggaggtgtagctaataagaactgtagcttgcctcata
aaaatgagagcaacaagtggacttgtgccccaagacaaagagatggaaaaacagattcgctatacatagcaggagg
aaaaaagttttggacacgaattaaggcccaattcagctgtgaaagtaacataggacaattagatggaatgttgcat
cagcaaatactattgcaaaaatatcaagtaattaaggtaagagcttatacatatggggtgatagaaatgccagaaa
actatgcaaaaacaagaatcataaacaggaaaaaaagagaactcagccacaagaggaagaagagaggcgttggctt
ggtcattatgctagttatcatggcaatagtagctgccgcaggggcttctctgggagtcgcaaacgcgattcagcag
tcttacactaaggcagctgtccagacccttgctaatgcaactgctgcacagcaggatgtgttagaggcaacctatg
ccatggtacagcatgtggctaaaggcgtacgaatcttggaagctcgagtggctcgagtggaagctatcacagatag
aataatgctataccaagaattggattgttggcactatcatcaatactgtataacctctacaaaaacagaagtagca
aaatatatcaattggacgaggtttaaggataattgcacatggcagcagtgggagagaggattacaggggtatgata
caaacttaacaatactgttaaaggaatcagcagcaatgacacaactagcagaagagcaagcaaggaggataccaga
agtatgggaaagtttaaaagacgtctttgattggtcaggatggttctcatggctaaagtatattcctattatagta
gtaggattattaggatgcattctgataagagctgtgatatgtgtatgtcaacctcttgtgcagatatacagaactc
taagtaccccgacataccaacgggtcacagtcatcatggaaacaagagcagacgtcgcaggagaaaatcaggattt
tggcgatggcttagaggaatcagacaacagcgaaacaagcgaaagagtgacagtacagaaagcttggagccgtgcc
tgggagctttggcagaactcaccctggaaggagccatggaaaagggcctgctgaggctgctcgtccttccgctga
cgatgggaatctggataaatggatggcttggagaacaccacaaaaataaaaaaagaaagggtgactgtgagacatg
ggctaaagaggactaa
>#17 Equine infectious anemia virus (EIAV) M16575 7014 nt
atgggagaccctttgacatggagcaaggcgctcaagaagttagagaaggtgacggtacaagggtctcagaaat
taactactggtaactgtaattgggcgctaagtctagtagacttatttcatgataccaactttgtaaaagaaaa
ggactggcagctgagggatgtcattccattgctggaagatgtaactcagacgctgtcaggacaagaaagagag
gcctttgaaagaacatggtgggcaatttctgctgtaaagatgggcctccagattaataatgtagtagatggaa
aggcatcattccagctcctaagagcgaaatatgaaaagaagactgctaataaaaagcagtctgagccctctga
agaatatccaatcatgatagatggggctggaaacagaaattttagacctctaacacctagaggatatactact
tgggtgaataccatacagacaaatggtctattaaatgaagctagtcaaaacttatttgggatattatcagtag
actgtacttctgaagaaatgaatgcattttttggatgtggtacctggccaggcaggacaaaagcagatattact

tgatgcaattgataaaatagcagatgattgggataatagacatccattaccgaatgctccactggtggcacca
ccacaagggcctattcccatgacagcaaggtttattagaggtttaggagtacctagagaaagacagatggagc
ctgcttttgatcagtttaggcagacatatagacaatggataatagaagccatgtcagaaggcatcaaagtgat
gattggaaaacctaaagctcaaaatattaggcaaggagctaaggaaccttacccagaatttgtagacagacta
ttatcccaaataaaaagtgagggacatccacaagagatttcaaaattcttgactgatacactgactattcaga
acgcaaatgaggaatgtagaaatgctatgagacatttaagaccagaggatacattagaagagaaaatgtatgc
ttgcagagacattggaactacaaaacaaaagatgatgttattggcaaaagcacttcagactggtcttgcgggc
ccatttaaaggtggagccttgaaaggagggccactaaaggcagcacaaacatgttataactgtgggaagccag
gacatttatctagtcaatgtagagcacctaaagtctgttttaaatgtaaacagcctggacatttctcaaagta
taatctagagaaaaggcctactacaatagtattaattaatgatactcccttaaatgtactgttagacacagga
gcagatacttcagtgttgactactgcacattataataggttaaaatatagagggagaaaatatcaagggacgg
gaataataggagtgggaggaaatgtggaaacatttctacgcctgtgactataaagaaaaagggtagacacat
taagacaagaatgctagtggcagatattccagtgactattttgggacgagatattcttcaggacttaggtgca
aaattggttttggcacagctctccaaggaaataaaatttagaaaaatagagttaaaagagggcacaatggggc
caaaaattcctcaatggccactcactaaggagaaactagaaggggctaaagagatagtccaaagactattgtc
agagggaaaaatatcagaagctagtgacaataatccttataattcacccatatttgtaataaaaaagaggtct
ggcaaatggaggttattacaagatctgagagaattaaacaaaacagtacaagtaggaacggaaatatccagag
gattgcctcacccgggaggattaattaaatgtaaacacatgactgtattagatattggagatgcatatttcac
tataccttagatccagagtttagaccatatacagctttcactattccctccattaatcatcaagaaccagat
aaaagatatgtgtggaattgtttaccacaaggattcgtgttgagcccatatatatatcagaaaacattacagg
aaatttacaacctttagggaaagatatcctgaagtacaattgtatcaatatatggatgatttgttcgtggg
aagtaatggttctaaaaaacaacacaaagagttaatcatagaattaagggcaatcttactggaagagggtttt
gagacaccagatgataaattacaagaagtgccaccttatagctggctaggttatcaactttgtcctgaaaatt
ggaaagtacaaaaaatgcaattagacatggtaaagaatccaacccttaatgatgtgcaaaaattaatggggaa
tataacatggatgagctcaggggtcccagggttgacagtaaaacacatagcagctactactaaggatgttta
gagttgaatcaaaaagtaatttggacggaagaggcacaaaaagagttagaagaaaataatgagaagattaaaa
atgctcaagggttacaatattataatccagaagaagaaatgttatgtgaggttgaaattacaaaaaattatga
ggcaacttatgttataaaacaatcacaaggaatcctatgggcaggtaaaaagattatgaaggctaataaggga
tggtcaacagtaaaaaatttaatgttactgttgcaacatgtggcaacagaaagtattactagagtaggaaaat
gtccaacgtttaaggtaccatttaccaaagagcaagtaatgtgggaaatgcaaaaaggatggtattattcttg
gctcccagaaatagtatatacacatcaagtagttcatgatgattggagaatgaaattggtagaagaacctaca
tcaggaataacaatatacactgatggggaaaacaaaatggagaaggaatagcagcttatgtgaccagtaatg
ggagaactaaacagaaaaggttaggacctgtcactcatcaagttgctgaaagaatggcaatacaaatggcatt
agaggataccagagataaacaagtaaatatagtaactgatagttattattgttggaaaaatattacagaagga
ttaggtttagaaggaccacaaagtccttggtggcctataatacaaaatatacgagaaaaagagatagtttatt
ttgcttgggtacctggtcacaaagggatatgtggtaatcaattggcagatgaagccgcaaaaataaaagaaga
aatcatgctagcataccaaggcacacaaattaaagagaaaagagatgaagatgcagggtttgacttatgtgtt
ccttatgacatcatgatacctgtatctgacacaaaaatcatacccacagatgtaaaaattcaagttcctccta
atagctttggatgggtcactgggaaatcatcaatggcaaaacaggggttattaattaatggaggaataattga
tgaaggatatacaggagaaatacaagtgatatgtactaatattggaaaaagtaatattaaattaatagaggga
caaaaatttgcacaattaattatactacagcatcactcaaattccagacagccttgggatgaaaataaaatat
ctcagagaggggataaaggatttggaagtacaggagtattctgggtagaaaatattcaggaagcacaagatga
acatgagaattggcatacatcaccaaagatattggcaagaaattataagataccattgactgtagcaaaacag
ataactcaagaatgtcctcattgcactaagcaaggatcaggacctgcaggttgtgtcatgagatctcctaatc
attggcaggcagattgcacacatttggacaataagataatattgacttttgtagagtcaaattcaggatacat
acatgctacattattatcaaaagaaaatgcattatgtacttcattggctattttagaatgggcaagattgttt
tcaccaaagtccttacacacagataacggcactaattttgtggcagaaccagttgtaaatttgttgaagttcc
taaagatagcacataccacaggaataccatatcatccagaaagtcagggtattgtagaaagggcaaataggac
cttgaaagagaagattcaaagtcatagagacaacactcaaacactggaggcagctttacaacttgctctcatt
acttgtaacaaagggagggaaagtatgggaggacagacaccatgggaagtatttatcactaatcaagcacaag
taatacatgagaaacttttactacagcaagcacaatcctccaaaaaattttgtttttacaaaatccctggtga
acatgattggaagggacctactagggtgctgtggaagggtgatggtgcagtagtagttaatgatgaaggaaag
ggaataattgctgtaccattaaccaggactaagttactaataaagccaaattgaatggtcagcatcgcattct
atggggggatcccaggggggaatctcaacccctattacccaacagtcagaaaaatctaagtgtgaggagaacac
aatgtttcaaccttattgttataataatgacagtaagaacagcatggcagaatcgaaggaagcaagagaccaa
gaaatgaacctgaaagaagaatctaaagaagaaaaaagaagaaatgactggtggaaaataggtatgtttctgt
tatgcttagcaggaactactggaggaatactttggtggtatgaaggactcccacagcaacattatatagggtt

ggtggcgataggggaagattaaacggatctggccaatcaaatgctatagaatgctggggttccttcccgggg
tgtagaccatttcaaaattacttcagttatgagaccaatagaagcatgcatatggataataatactgctacat
tattagaagcttatcatagagagataacattcatttataagtcttcttgcacagatagtgatcattgtcaaga
gtatcaatgtaaaaaagttaatcttaattcctctgactcctctaactctgtacgtgttgaggatgtaacgaac
acagcggaatattggggatttaaatggctagaatgtaatcaaacagaaaatttaagactatattagtacctg
aaaatgaaatggtaaatatcaatgatactgatacctggatacctaaggggtgtaatgagacgtgggcaagagt
gaaacgttgtcctatagatattttatatggatacatccaatcaggctgtgtgtacagccaccatttttctg
gtacaggagaaagggattgctgatacttctagaattggcaattgtgggccaacaatatttcttggggttttag
aagataataagggagtagtacgggggattatacagcttgcaatgtgcgtcgcctaaatataaatagaaagga
ttatacagggatctatcaagtacctatatttatacatgtactttcactaacataacttcctgcaataatgag
ccaataatcagtgttatcatgtatgaaacaaaccaggtacaatatttattgtgtaataataataatagtaata
attataattgtgtagtacaaagttttggagttataggacaggcacacttagaactgcctagacctaacaaaag
aataaggaaccaaagctttaaccaatataactgctctataaataacaaaacagaattagaaacatggaagtta
gtaaagacttctggcgtaactcctttacctatttcttctgaagctaacactggactaattagacataagagag
attttggtataagtgcaatagtggcagctattgtagccgctactgctattgctgctagcgctactatgtctta
tgttgctctaactgaggttaacaaaataatggaagtacaaaatcatacttttgaggtagaaaatagtactcta
aatggtatggatttaatagaacgacaaataaagatattatatgctatgattcttcaaacacatgcagatgttc
aactgttaaaggaaagacaacaggtagaggagacatttaatttaattggatgtatagaaagaacacatgtatt
ttgtcatactggtcatccctggaatatgtcatggggacatttaaatgagtcaacacaatgggatgactgggta
agcaaaatggaagatttaaatcaagagatactaactacacttcatggagccaggaacaatttggcacaatcca
tgataacattcaatacaccagatagtatagctcaatttggaaaagacctttggagtcatattggaaattggat
tcctggattgggagcttccattataaaatatatagtgatgtttttgcttatttatttgttactaacctcttcg
cctaagatcctcagggccctctggaaagtgaccagtggtgcagggtcctccggcagtcgttacctgaagaaaa
aattccatcacaaacatgcatcgcgagaagacacctgggaccaggcccaacacaacatacacctagcaggcgt
gaccggtggatcaggggacaaatactacaagcagaagtactccaggaacgactggaatggagaatcagaggag
tacaacaggcggccaaagagctgggtgaagtcaatcgaggcatttggagagagctatatttccgagaagacca
aaggggagatttctcagcctggggcggctatcaacgagcacaagaacggctctgggggaacaatcctcacca
agggtccttagacctggagattcgaagcgaaggaggaaacatttatgactgttgcattaaagcccaagaagga
actctcgctatcccttgctgtggatttcccttatggctattttggggactagtaattatagtaggacgcatag
caggctatggattacgtggactcgctgttataataaggatttgtattagaggcttaaatttgatatttgaaat
aatcagaaaaatgcttgattatattggaagagctttaaatcctggcacatctcatgtatcaatgcctcagtat
gtttag
>#18 Human immunodeficiency virus type 1 (HIV-1) AF069670 5652 nt
gcgagagcgtcagtattaagtgggggaaaattagatgcatgggagaaaattcggttaaggccagggggaaaa
agaaatatagaatgaaacatctagtatgggcaagcagggagctggaaagatttgcacttaaccctagcctttt
agaaacaacagaaggatgtcaacaaataatggaacagttacaatcagctctcaagacaggaacagaagaactt
agatcattatttaatacagtagcagtcctctattgcgtacatcaacggatagagataaaagacaccaaggaag
ctctagataaaatagaggaaatacaaaagaagagcaagcaaaaggcacaacaagcagcagctgacacaggaaa
cagcagcaaggtcagccaaaattaccctatagtgcaaaatgcacaagggcaaatggtacaccagtccttgtca
cctaggactttgaatgcatgggtaaaagtaatagaagaaaaggctttcagcccagaagtaatacccgtgttct
cagcattatcagaaggagccactccacaagatttaaatatgatgctgaacatagtgggggacatcaggcagc
tatgcaaatgttaaaagataccatcaatgaggaagctgcagaatgggacaggttacatccagcacatgcaggg
cctgttgcaccaggccagatgagagaaccaaggggaagcgatatagcaggaactactagtacccctcaagaac
aaataggatggatgacaggcaacccacctattccagtgggagacatctataaaagatggataatcctgggatt
aaataaaatagtaagaatgtatagccctgttagcattttggatataaaacaagggccaaaagaacccttcaga
gactatgtagataggttctttaaaactctcagagctgagcaagctacacaggatgtaaaaaattggatgacag
aaacattactggtccaaaatgcaaatccagattgtaagtccattttaagagcattaggagcaggggctacatt
agaagaaatgatgtcagcatgtcagggagtaggagggcccagccataaagcaaggttttggctgaggccatg
agtcaagcacaacaacatacaactgtaatgatgcagagagccaattttaggggacagaaaaggattaagtgtt
tcaactgtggcaaagaaggacacctagccagaaattgcaggcccctaggaaaaagggctgttggaaatgtgg
gaaagagggacaccaaatgaaggactgcactgaaagacaggctataggggtcagctaagagaagctctatta
gatacaggagcagatgatacagtgttagaagacataaatttgccaggaaaatggaaaccaaaaatgatagggg
gaattggagggttcatcaaggtaaaacagtatgatcagatacttatagaaatttgtggaaaaaaggctatagg
tacagtattagtaggacctacacctgtcaacataattggaagaaatatgttgactcagattggttgtactcta
aatttcccaattagtcctattgagactgtaccagtaaaattaaagccaggaatggatggcccaaaggttaaac
aatggccattgacagaagaaaaaataaaagcattaacagaaatttgtacagaaatggaaaaggaaggaaaaat
ttcaaaaattgggcctgagaatccatataatactccaatatttgctataaagaaaaagacagcactaaatgg

aggaaattagtagatttcagagagctcaataaaagaactcaagacttttgggaagttcaattaggaataccgc
atccagcaggcttaaaaaagaaaaaatcagtaacagtgctagatgtgggggacgcatattttcagttccctt
agatgaaagctttagaaagtatactgcattcaccatacctagtacaaacaatgagacaccaggagtcaggtat
cagtacaatgtgcttccacagggatggaaaggatcaccggcaatattccagagtagcatgacaaaaatcttag
agcccttcagattaaagaatccagaaataattatctatcaatacatggatgacttgtatgtggggtctgattt
agaaatagggcagcatagaacaaaaatagaagagttaagagctcatctattgagctggggatttactacacca
gacaaaaagcatcagaaagaacctccatttctttggatgggatatgaacttcatcctgacaaatggacagtcc
agcctatagagctgccagaaaaggaaagctggactgtcaatgatatacagaaattagtgggaaaactaaattg
ggcaagtcaaatttatgcaggaattaaagtaaagcaattgtgcagactcctcaggggagccaaagcactaaca
gatatagtaacactgactgaggaagcagaattagaattggcagagaacagggaaattctaaaggaccctgtgc
atggggtatattatgacccatcaaaagacttaatagcagaaatacagaaacaagggctagaccaatggacata
tcagatttatcaggagccatttaaaaatctaaaaacaggaaaatatgcaagaaagaggtctgctcacactaat
gatgtaagacaattagcagaagtggtgcaaaaagtggtcatggaaagcatagtaatatggggaaagactccta
aatttaaactacccatacaaaaagaaacatgggaaacatggtggatggactattggcaggctacctggattcc
tgaatgggaatttgtcaatacccctcctctagtaaaattatggtaccagttagagaaagaccccatagcagga
gcagagaccttctatgtagatggggcagccaatagggaaactaagctaggaaaagcagggtatgtcactgaca
gaggaaggcaaaaggttgtttctctaactgagacaacaaatcaaaagactgaactacatgcaatccatctagc
cttgcaggattcagggtcagaagtaaacatagtaacagactcacaatatgcattaggaatcattcaagcacaa
ccagacagtagtgaatcagagatagttaatcaaataatagagaagctaataggaaaggacaaagtctacctgt
catgggtaccagcacacaaggggattggaggaaatgaacaagtagataaattagtcagctctggaatcaggaa
ggtgttattttagatgggatagataaagctcaagaagagcatgaaagatatcacagcaactggagagcaatg
gctagtgattttaatctgccacctgtaatagcaaaggaaatagtagccagctgtgataaatgccagctaaaag
gggaagccatgcatggacaagtagactgcagtccagggatatggcaactagattgcacacatctagaaggaaa
agtaattctggtagcagtccatgtggccagtggctatatagaagcagaagttattccagcagaaacaggacag
gagacagcatactttctactaaaattagcaggaagatggccagtaaaaatagtacacacagataatggcagca
atttcaccagcgctgcatttaaagcagcctgttggtgggcaagtatccaacaggaatttggaattccctacaa
tccccaaagtcaaggagtagtggaatctatgaataaggaattaaagaaaatcatagggcaggtaagagagcaa
gctgaacatcttaagacagcagtacaaatggcagtattcattcacaattttaaaagaaaaggggggattgggg
ggtacagtgcaggggaaagaataatagacataatagcaacagacatacaaactaaagaattacaaaaacaaat
tacaaaaattcaaaatttcgggtttattacagggacagcagagatcccatttggaaaggaccagcaaaacta
ctctggaaaggtgaaggggcagtggtaatacaggacaacaatgatataaaggtagtaccaagaagaaaagcaa
agatccttagggatactgcagacaacttgtgggttactgtctactatggggtacctgtgtggaaagatgcaga
gaccaccctattttgtgcatcagatgctcaagcatataagacagaaatgcataatgtatgggctacacatgcc
tgtgtacccacagaccccaacccacaagaactgcatttgaaaaatgtgacagaagagtttaacatgtggaaaa
atagcatggtagagcagatgcatacagatataatcagtctatgggacgaaagcctaaagccatgtgtaaagtt
aacccctctctgcgttactttaaattgtaccaatgccaacggaacccagaatgtcaacatcacgaatgtagga
atgagaaactgctctttcaatatgaccacagagctaagggataagaaacagaaaggatattcactttttata
agcttgatatagtacaaattaatgataatggtaataacagtaataacagtagtgagtataggttaataaattg
taatacctcagccattacacaggcttgtccaaaggtatcctttgagccaattcccatacactattgcccca
gctggttttgcgatcctaaagtgtagggataaggagttcaatggaacagggccatgcaacaatgtaagcacag
tacagtgcacacatggaatcaagccagtagtatcaactcaactgctgttaaatggcagtctagcagaagaaaa
gataatgattagatctgaaaatatctcagacaatgccaaaaccataatagtacaacttaccgagcctgtaaca
attaattgtaccagacctagcaacaatacaagaacaagtatacgtataggaccaggacaagcattctatgcaa
caggtgacataacaggggatataagacaagcacattgtaatgtcagtagatcaagctggaataaaactctaca
agacatagttacacaattaagagtatactggaatagaacaataatctttaatagctcctcaggaggggattta
gaaattacaacacatagttttaattgtggaggagaattcaaacagctccaggcaagagtcctggctgtggaaa
gatacctaaaggatcaacagctcctaggaatttggggctgctctggaaaactcatctgcaccactaatgtgcc
ctggaactctagttggagtaataaatctcagaatgaaatatgggaagacatgacctggctacaatgggacaga
gaaattagcaattacacagaaataatatataaactaattgaagaatcgcagaaccagcaggaaaagaatgaac
aagacttactggcattggacaaatgggcaaatctgtggaattggtttgagataacaaaatggctgtggtatat
aaaaatatttataatgatagtaggaggcttaataggattaagaatagtttttgctgtgttttctgttataaat
agagttaggcagggatactcacctttgtcgtttcaggctattagtttgtttgataccccagcaatagcagtag
ctggctggacagataggggtatagaattaatacaaagaattggtagagctattctcaacatacctagaagaat
tagacagggctttgaagaggctttgctataa
>#19 Human immunodeficiency virus type 2 (HIV-2) M30502 6297 nt
atgggcgcgagaaactccgtcttgagagggaaaaaagcagacgaattagaaaaagttaggttacggcccggcg
gaaagaaaaagtacaggttaaaacatattgtgtgggcagcgaatgaattggataaattcggattggcagagag

cctgttggagtcaaaagaaggttgccaaaagattctcagagttttagatccattagtaccaacagggtcagaa
aatttaaaaagcctttttaataccgtctgcgtcatttggtgcttgcacgcagaagagaaagtgaaagatactg
aggaagcaaagaaactagcacagagacatctagtggcagaaactggaactgcagagaaaatgccaaatacaag
tagaccaacagcaccacctagtgggaaaagaggaaactaccccgtgcaacaagcgggtggcaactatgtccat
gtgccactgagcccccgaactctaaatgcatgggtaaaattagtggaggaaaagaagttcggggcagaagtag
tgccaggatttcaggcactctcagaaggctgcacgccctatgatattaatcaaatgcttaattgtgtgggcga
tcaccaagcagctatgcaaataatcagagagattattaatgaagaagcagcagactgggattcgcagcaccca
ataccaggccccttaccagcaggacagctcagagacccaagagggtctgacatagcaggaacaacaagcacag
tagatgaacagatccagtggatgtataggccacaaaatcccgtaccggtagggaacatctacagaagatggat
ccaaatagggctgcaaaagtgtgtcagaaagtacaacccaactaacatcttagacataaaacagggaccaaaa
gaaccgttccaaagctatgtagacaggttctacaaaagcttgagggcagaacaaacagacccagcagtaaaaa
attggatgacccaaacgctgctaatacagaatgccaacccagactgcaagttagtactaaaaggactggggat
gaatcccaccctagaagagatgctaaccgcctgccaggggtaggcggaccaggccagaaagccaggctaatg
gctgaagccctaaaagaggctatgggaccaagccctatcccatttgcagcagcccaacaaagaaaggcaatta
ggtattggaactgtggaaaggagggacactcggcaagacagtgccgagcacctagaagacagggctgctggaa
gtgtggcaagccaggacacatcatggcaaactgcccggaaagacaggcaggtttttttagggttgggcccacgg
ggaaagaagcctcgcaacttccccgtgacccaagcccctcaggggctgataccaacagcacctccggcagatc
cagcagcggaactgttggagagatatatgcagcaagggagaaagcagagggagcagagggagagaccatacaa
agaggtgacggaggacttgctgcacctcgagcagagagagacacctcacagagaggagacagaggacttgctg
cacctcaattctctctttggaaaagaccagtagatgacaggagataccccaatcaacatctttggcagaaata
ttctgacagccttaggcatgtcattaaatttaccagttgccaagatagagccaataaaagtaacattgaagcc
agggaaagatggaccaaggctgaaacaatggcccctaacaaaagagaaaatagaagcactaaaagagatctgt
gaaaaaatggaaaaagagggccagctagaagaggcacctccaactaatccttataataccccccacatttgcaa
ttaagaaaaaggacaagaacaaatggaggatgctgatagattttagagaactaaataaggtgactcaagattt
cacagaaattcagctaggaattccacacccggcaggactagccaaaaagaaaaggatctctatattagatgta
ggggatgcctatttttccataccactacatgaagattttaggcagtatactgcatttaccctaccagcagtaa
acaatatggaaccaggaaaaagatatatatataaagtcttgccacaaggatggaagggatcaccagcaatttt
tcaatacacaatgaggcaagtcttagaacctttcagaaaagcaaacccagatgtcattctcatccagtacatg
gatgatatcttaatagctagtgacaggacaggtttagagcatgacaaagtggtcctgcagctaaaagaacttc
taaatggcctagggtttctactccagatgagaagttccaaaaagaccctccatttcaatggatgggctgtga
actatggccaactaaatggaagctgcagaaactacaactgccccagaaagacatatggacagtcaatgacatc
caaaagctagtgggagtcttaaattgggcggcacaaatctattcaggaataaaaaccaaacacttatgtagac
taattagaggaaaaatgacactcacagaagaagtgcagtggacagaactagcagaagcagagctagaagaaaa
caaaattatcttgagccaggaacaagaaggatattattaccaagaagaaaaagaattagaggcaacaatccaa
aaaagccaaggacatcaatggacatacaaaatacaccaggaagagaaaatcctaaaagtaggaaagtatgcaa
agataaaaaatacccataccaatggggtcagattactagcacaggtagttcagaaaataggaaaagaggcact
agtcatttggggacggataccaaaatttcacctgccagtggagagagagacctgggagcagtggtgggataac
tactggcaagtgacatggatcccagagtgggactttgtatctaccccaccactggtcaggttaacatttaacc
tagtaggagatcctataccaggcgcagagaccttctacacagatggatcatgcaatagacagtcaaaagaggg
aaaagcaggatatgtaacagatagaggaaaagacaaagtaaaagtattagaacaaactaccaatcagcaggca
gaattagaagtctttcggatggcactggcagactcaggcccaaaggttaatatcatagtagattcacagtatg
taatggggatagtagcaggccagccaacagagtcagaaaatagaatagtgaaccagatcatagaagaaatgat
aaagaaggaagcagtctatgttgcatgggtcccagcccataaaggcataggaggaaaccaggaagtagaccat
ttagtaagtcaaggcatcagacaagtattattcctggaaaagatagagcccgctcaagaggaacatgaaaaat
atcatagcattataaaagaactaacccataaatttggaataccccttctagtagcaagacagatagtaaactc
atgtgcccaatgccaacagaaaggagaagccatacatgggcaagtaaatgcagaaataggcgtttggcaaatg
gactacacacacttagaaggaaaaatcattatagtagcagtacatgttgcaagtggattcatagaagcagaag
tcatcccacaggaatcaggaaggcagacagcactcttcctattaaaactggccagtaggtggccaataacgca
cttgcacacagacaatggccccaacttcacttcacaggaagtgaagatggtggcatggtgggtaggtatagaa
caatcctttggagtaccttacaacccacaaagccagggagtagtagaagcaatgaatcaccacctaaagaatc
agataagtagaattagagaacaggcaaatacaatagaaacaatagtactgatggcagttcattgcatgaattt
taaaagaaggggaggaataggggatatgaccccagcagaaagactaatcaacatgattaccacagaacaagaa
atacaattcctccaaagaaaaaattcaaattttaaaaatttccaggtctattacagagaaggcagagatcagc
tgtggaaaggacctggtgaactactgtggaagggagaaggagcagtcatagtcaaggtagggacagacataaa
agtagtaccaagaaggaaggccaagattatcagggacatggagcctggtaggaatcagctgtttgttgtcatt
ttactaacaagtgcttgcttagtatattgtagccagtatgtgactgttttctatggcatacccgcgtggaaaa
atgcatctattcccttattttgtgcaactaaaaatagagacacttgggggaccatacagtgcttgccagacaa

tgatgattatcaggaaataatttaaatgtgacagaggcttttgatgcatggaataatacagtgacagaacaa
gcagtagaagatgtctggcatctatttgagacatcaataaaaccatgtgtcaagctaacacctctatgtgtgg
caatgaattgtagcagggttcaagggaataccacgaccccgaatcccaggacctcgagttccacaacctcgag
accacccacatccgcagcctccataataaatgaaacttctaactgcatagaaaacaacacatgcgcaggatta
gggtatgaggagatgatgcaatgtgagttcaatatgaaggggttagaacaagataagaaaaggaggtataagg
acacatggtatttagaagatgtggtttgtgacaacacaacagctggcacatgttacatgagacattgcaacac
atcaatcatcaaagagtcatgtgataagcactattgggatgctatgaggtttagatactgtgcaccaccgggc
tttgccctattaagatgtaatgataccaactattcaggctttgaacctaagtgcactaaagtagtagctgctt
catgcacaaggatgatggaaacgcaaacttctacttggtttggctttaatggcactagagcagaaaatagaac
atatatctattggcatggcagagataataggactatcattagcttaaacaagtattataatctcacaatgcgt
tgtaagagaccaggaaataagacagttttaccaataacacttatgtcaggattagtgtttcactctcagccaa
tcaacacaaggcctaggcaggcatggtgccggtttggaggcagatggagggaagccatgcaggaggtgaagca
aacccttgtacaacatcccagatacaaaggaatcaatgatacagggaaaattaactttacgaaaccgggagca
ggctcagacccggaagtggcatttatgtggactaactgcagaggagaatttctctactgtaacatgacttggt
tcctcaattgggtagaagacaagaaccaaacacggcgcaactattgccatataaagcagataattaatacctg
gcataaagtagggaaaaatgtatatttgcctcctagggaaggggagttggcctgtgaatcaacagtaaccagc
ataattgctaacattgacatagataaaaatcggactcataccaacattacctttagtgcagaagtggcagaac
tgtaccgattagaactgggagactacaaattaatagaaataacaccaattggcttcgcacctacagatcagag
aaggtactcctcaactccagtgaggaacaaaagaggtgtgttcgtgctagggttcttgggttttctcgcgaca
gcaggttctgcaatgggcgcgcggtccctgacgctgtcagcccagtcccggactttactggccgggatagtgc
agcaacagcaacagctgttggacgtagtcaagagacaacaagaaatgttgcgactgaccgtctggggaacgaa
aaacctccaggcaagagtcactgctatcgagaagtacctaaagcatcaggcacagctaaattcatggggatgt
gcgtttagacaggtctgccacactactgtaccgtgggtaaatgactctttatcgcctgactggaaaaatatga
catggcaggagtgggagaaacaagtccgctacctagaggcaaatatcagtcaaagtttagaagaagcccaaat
tcaacaagaaaagaatatgtatgaattacaaaaattaaatagctgggatattcttggcaactggtttgactta
acctcctgggtcaagtatattcaatatggagtgcatatagtagtgggaataatagctttaagaatagcaatct
atgtagtgcaattgttaagtagatttagaaagggctataggcctgttttctcttccccccccggttatctcca
acaggcggcccaactgcaa
>#20 Simian immunodeficiency virus (SIV) JQ864086 6300 nt
atgggcgcgagaaactccgtcttgtcagggaagaaagcagatgaattagaaaaaattaggttacggcccaacg
gaaagaaaaagtatatgttgaagcatgtagtatgggcagcaaatgaattggacagattcggattagcagaaag
cctgttggataacaaagaaggttgtcaaaaaattctttcggttttagctccattagttccgacaggttcagaa
aatttaaagagcctttataatactgtctgcgtcatttggtgcattcacgcagaagagaaagtgaaacatactg
aggaagcaaaacaaatagtgcagagacatctagtggtggaaacaggaacagcagacagaatgccagtaacaag
cagaccaacagcaccacctagtggcagaggaggaaattacccagtgcagcaagtaggtggcaattatgtccac
ctacccttaagtccaagaacattaaatgcttgggtaaaattggtagaagagaaaaaatttggggcagaggtag
tgccaggatttcaagcgctatcagaaggctgcactccctatgatatcaatcaaatgctaaattgtgtaggaga
acatcaggcagccatgcaaattattagagagattataaatgaagaagctgccgattgggatttacaacaccca
ccgccaggtccaccaccagcagggcaacttagagagccaagaggatcagacattgcaggaactactagtacag
tagatgaacaaatccaatggatgtacaggcaacaaaaccctataccagtaggcaacatttatagaaggtggat
ccaattagggctgcagaaatgtgtaagaatgtataacccaacaaacattttagatgtgaaacaaggaccaaaa
gagccatttcaaagctacgtagatagattctataaaagtctaagagcagagcaaacagatccggcagtaaaga
attggatgacccaaacactgctgattcaaaatgctaacccagattgtaaattggtgctcaagggtctgggtat
gaatcccactttagaagaaatgctgacagcctgtcagggaataggagggccaggacaaaaagctagattaatg
gcagaagcattgaaagaggcactgagaccagaccaactcccatttgcagcagtccaacagaaaggacaaagga
ggacaatcaagtgttggaattgtggaaaggagggacactctgcaagacaatgcagggcccctagaagacaggg
ctgctggggggtgcggaaaaacgggtcatgttatggccaaatgccctgaaagacaggcgactgcctacattgaa
gaacagcccgtagaagtattattagatacaggggctgatgattcaattgtagcagggatagaattaggtccaa
attataccccctaaagtagtaggaggaataggaggcttcattaataccaaagaatataaagatgtaaaaataaa
agtcttaggcaaggtgattaagggaacaattatgacgggagataccccaattaatattttggcagaaatttg
ctaacagctatgggcatgtctttaaatctccccatagctaaggtggagcctataaaagtaacactaaaaccag
ggaaagaaggaccaaaattaagacagtggccgctatcaaaagaaaagataattgcattaagagaaatctgtga
aaaaatggaaaaagatggccagttagaggaagcccctccaaccaatccgtataacacccccacttttgctata
aagaagaaagacaaaaataaatggaggatgctaatagattttagagaattaaataaggtcactcaagacttta
cagaagtacagttaggaataccacaccctgcaggactagcaaagagaaggaggatcacagtattggatgtagg
tgatgcatatttctccatacctctagatgaagaattcaggcagtacactgcctttactttaccatcagtaaat
aatgcagaaccaggaaaaagatacatctataaggtattacctcaagggtggaaggggtcaccagctatttttc

agtatactatgagaaatgtattagaacctttcaaaaaagcaaatccagatgtgaccctgatccaatacatgga
tgacgtcttaatagctagtgatagaacagatttagagcatgacagggtagttttacagttaaaggaacttctg
aacggcataggattctctaccccagaagagaagttccagaaagatcccccattccagtggatgggatatgaat
tgtggccaaccaaatggaaactgcagaaaatagagttgccacaaagagagatttggacagtaaatgacataca
aaaattagtaggagtgctaaattgggcagcacaaatttatccaggaataaagactaaacatctttgcagacta
atcagaggaaaaatgactttaacagaagaggttcagtggactgagatggcagaggcagaatatgaagaaaaca
agataattctcagtcaagaacaagaaggatgttactaccaagagggaaaaccaatagaggcaacagtaataaa
gagtcaggataatcaatggtcatataaaattcaccaagaagacaaagtactgaaagtaggcaaatttgcaaag
gttaaaaatacacatacaaatggagtcagattactagcacacgtagtgcagaaaataggaaaagaagcactag
taatttgggagaggtgccaaaattccatttgccagtagaaagagaaatttgggaacaatggtggacagatta
ttggcaagtaacctggataccagattgggactttgtgtcaacacctcccttagtcagattagtcttcaaccta
gtaaaagagcctatacagggggcagaaacattctatgtagatggatcctgtaataggcagtcaagagaaggaa
aagcaggctatgtggcggataggggcagagacaaagcaaaactttagaacagactaccaaccaacaagcaga
gttggaagccttctatctagccttagcagattcgggaccaaaagcaaatattatagtagattcccaatatgtt
atgggcataatagcaggtcaacccactgaatcagaaagtaggttagtaaaccagataatagaggagatgatta
aaaaagaagcaatttatgtagcatgggtgcctgcacataaaggaataggaggaaatcaagaagtagatcacct
ggttagccagggaattagacaagtcctattcttagaaaaaatagaaccagcacaagaagagcatgaaaagtac
catagtaatgtaaaagaattggtattcaaatttggtttacctaggctagtagcaaaacagatagtagacacat
gtgataaatgccaccagaaaggagaagccatacatgggcaagtaaatgcagaactagggacttggcaaatgga
ctgtacgcacctagaaggcaaaataattatagttgcagtacatgtggctagtggattcatagaggcagaagta
atcccgcaggaaacaggaagacaaacagcactgtttctgttaaagttagctggcagatggcctatcacacatc
tgcatactgataatggtgccaatttcacatcacaagaagtaaaaatggttgcctggtgggcagggattgaaca
gacctttggggtgccttataatccacagagccaaggagtagtggaagcaatgaaccatcatttaaaaacccag
atagatagaattagagaacaagcaaactcaatagagactatagtactaatggcagttcattgtatgaattta
aaagaagggaggaataggggatatgactccagcagaaagattagtcaatatgatcaccacagaacaagaaat
acaattccaacaatcaaaaaattcaaaatttaaaaattttcgggtctattacagagaaggcagagaccagctg
tggaaaggacccggtgagctattgtggaaaggggaaggagcagtcatcctaaaggtagggacagagatcaagg
tagtaccaaggaggaaagctaaaattatcaaagacatgggatgtcttgggaatcagctgcttatcgcgctctt
gctagtaagtgctttagagatttattgtgttcaatatgtaacagtattctatggtgtaccagcatggaagaat
gcgacaattcccctcttctgtacaaccaggaatagggacacttggggaacaacacaatgcttgccagataatg
atgattactcagaattggcaatcagtatcacagaggcttttgatgcttggaataatacagtcacagaacaagc
aatagaggatgtgtggaacctctttgaaacatccattaagccctgtgtaaaactcaccccactatgtatagca
atgagatgtaataaaactgagacagataggtggggtttgacaaggaacgcagggacaacaacaacaacaacaa
caacaacaacagcagcaacaccaagtgtggcagaaaatgttataaatgaaagtaatccttgcataaaaaataa
tagttgtgcaggcttggaacaggagcccatgataggttgtaaatttaacatgacagggttaaaaagggacaaa
aggatagaatataatgaaacatggtattcaagagatttaatctgtgagcagtcagcgaatgaaagtgagagta
aatgttacatgcatcattgtaacaccagtgttattcaggaatcctgtgacaagcattattgggatgctattag
atttagatattgtgcaccgccaggttatgctttgcttaggtgtaatgattcaaattattcaggctttgctcct
aactgttctaaggtagtggtttcttcatgcacaagaatgatggaaacgcaaacctctacttggtttggcttca
atggtactagggcagaaaatagaacatacatttattggcatggcaaaagtaatagaaccataattagcttaaa
taagtattataatctaacaatgagatgtagaagaccaggaaataagacagttttaccagtcaccattatgtca
gggttggtcttccattcgcaacccataaatgagagaccaaaacaggcctggtgctggtttggaggaagctgga
aagaggccatccaggaagtgaaggaaaccttggtcaaacatcccaggtatacgggaactaatgatactaagaa
aattaatctaacagctccagcaggaggagatccagaagtcacttttatgtggacaaattgtagaggagaattc
ttatactgcaaaatgaattggtttcttaattgggtagaggacagagaccaaaagagtagcagatggagacaac
aaaatacgagagagcgacagaagaaaaattatgtgccatgtcatattagacaaataatcaacacgtggcacaa
agtaggcaaaaatgtatatttgcctcctagggaaggagacctgacatgtaattccactgtaactagtctcata
gcagagatagattggaccaatagcaatgagaccaatatcaccatgagtgcagaggtggcagaactgtatcgat
tggagttgggagattacaaattagtagagattactccaattggcttggcccccacaagtgtaagaaggtacac
cacaactggtgcctcaagaaataagagaggggtctttgtgctagggttcttgggtttctcgcgacagcaggt
tctgcaatgggcgcggcgtcgctgacgctgtcggctcagtcccggactttgttggctgggatagtgcagcaac
agcaacagctgttggatgtggtcaagagacaacaagaattgttgcgactgaccgtctggggaactaagaacct
ccagactagagtcactgctatcgagaagtacctgaaggatcaggcgcagctaaattcatggggatgtgctttt
aggcaagtctgtcacactactgtaccatggccaaatgaaacattggtgcctaattggagcaatatgacttggc
aagagtgggaaagacaggttgacttcctagaggcaaatataactcaattattagaagaagcacaaattcagca
agaaaagaatatgtatgaattgcaaaaactaaatagctgggatatctttggcaattggtttgaccttacttct
tggataagatatatacaatatggtgtactaatagttttaggagtagtagggttaagaatagtaatatatgtag

tgcagatgttagctaggttaagacagggttataggccagtgttctcttcccctcccgcttatgttgtcatcag
aattggaatagcctacctacaa
>#21 Jembrana disease virus (JDV) NC_001654 5748 nt
atgaagttgagcaagcttgagaaggcccttaaaaaggtaagggtgacgccccagagagatgatacttatacaa
taggaaacgtcctgtgggccatcaggatgtgtagattgatggggctggattgctgcatagatgaggctacagc
agcggaggtggcaattttgataggacgtttccagtcgttggatttacaagactcccctttaaagggaaaggat
gagaaagccatattgaccactctgaaagttttgtggagcctgctggcgggccaccacccagaaaattcagata
tggcagaaaagtattgggaagcatggacaatcagagaaagggagtcccaaaaggaggaggaaggagaaattac
cagcatctacccacaacttagaaagaacttccccgctgtatccacatctgacgggtctcctagatatgaccct
gatttaacaaaacagctaaagatttgggcagatgcgacggaaaaacatggggtggaccaccacgccgttaata
ttttaggggtgatcacagcaaacttaacacaaagtgaaattaggctattactacaaagcaccccacaatggag
attggatattcagctcatagaatcaaaattaaatgccagggagcacgcccatagggtctggaaagagtcccac
ccggaggccccaaagacagatgagataattggaaaggggctaaccgcagcagagcaagccaccttaaccaccc
aagaatgcagagacacttataggcagtgggttttagaggcggcgttagaggtagcacaaggaaaacatgaccg
gcccggacctattaatatccaccaggggccgaaagagccatatcctgagtttgtaaataagttagttacagca
ttggagggtatggcagcacccgaaactaccaaacagtatctattagaccacctgtcggtggatcatgcaaatg
aggactgccgcgcagtgttgctacctttagggccctcagccccgatggagaggaagttagaggcatgcagggc
ggtaggttcaagtaaacagaagatgcaattcttggcagaggcatttgcagctataaacgtaaagggtgatgga
gaagtacaaaggtgttatggatgtgggaagcctggccatataagaagggattgcaagaaccagaaatgtttca
aatgtggaaaaccgggccacttacaaaggaactgcaaatcaaaaaggcaacctttcataaaaatttttcttagg
agggaggtgggttagggctttgatagatacaggggcagatgaggtagtgctgaaagacattcattgggataga
atcaagggtgtgcccgctgcctcagtagtgcaggttggagtaacaggcagaaatatagcaaggaggaaaagta
atgtagagtggagattcaaaaacagatatggcatagtggacgtcctgttctccaacactccagtaaatttgct
aggccgatcagtactgcaaagtatagtgacaaaatttactctagctgcacacaccaaacagattcaaccactt
ccggtcaagctacatgggccaggtccacgggtgcctcagtggcccctcacattagaaaaatataaagccctta
aggaaattgttgaggaactactaaaagatggaaaaatctctagaaccccttgggataatcctttcaatacccc
tgtttttgtaataaaaaagaaaggggggaagtaagtggagaatgctaatggatttcagggccttaaataaagtg
acaaacaaaggacaagaattccaaatcgggctgccatacccccaggaattcagcaatgtgaacatataacag
ctatagacataaaagatgcctactttaccatccctttagatgagaattttagacaatacactgctttctcggt
ggtcccggtgaatagagaagggcctctagaaagatatcattggaatgtgttacctcaagggtgggtgtgtagc
cctgccatatatcaaacaaccactcaggagataattgcagagataaaagatagatttcctgacattgtgctct
atcaatatatggatgaccttttaattggatcagacagaccagaccataaaagggtggtgagtgaaattaggga
agaattaggggcttatggttttaaaactccagaagaaaaaatccaggaagaacaggtgcaatggttggggtat
gaacttaccccgaaaaggtggcggttccagcctaggcagataaaaataaaaaaggtggtaacagtgaatgagc
tacaacagatgataggaaactgtgtatgggtgcagccggaggtaaaaatccctttgagtcccctctcagattt
actaaaagggaaaactgacctaaaagataagataaaattgactgaagaggctatccaatgcctagagacagta
aataagagattaaaagaccctgaatggaaggaaaggataaaggaaggaactgagctggtagttaaaattcaat
tgatccctgagggcgttgtttatgatctactacaggatggaaaccccatatggggtggagtgaaggatggga
ttataatcacgctaataagataaagaaaatgttaagtataatgaaaaaattgtcccggatagtcatgattatg
accggaagggaagtctccttcctgatacccggagattcagaagactgggaatctgccttacagaggatcaata
cactaacagagatacctgaagtaaaattctacaaacatgcctgtaggtggacctctgtctgcggtcctgtaat
agaaagatatcctacttattatactgatggaggcaagaaagggtctaaggcagcagcagcctactggagggag
ggaaaaataaggagggaagttttcccaggtacaaatcaacaggcagaacttaaggccgtcttgatggctctac
aagatggccctgcaaaaatgaatattattacagacagtagatatgcttttgaggggatgcgagaggaacccga
gacttggggaagagaaggactttggaaggaaatcggagaagagctaagaaggaaagagtatgtaggagtcagt
tgggttcccggacataaggggatcggaggaaacacagaagtagaccaagaggtacaaaaagcactccagggcc
ccataacagtgagtctcccacaagaaatactattagaagcaggggagacaaaacttgtgaagacagggatctt
ctgggaagggctgaggccatgtaagctcaggcccgaagaaggcctgaagcttaagggaagcttaatagatgaa
gaattacagctggaaattacaaatactcaaaacagcagggtgggaataagacaagggcaaacaattggcacat
gtttcattgaagctattccacaagccatagaagaacatgaaaaatggcatactacagcggagatattggctcg
agagttccaactacccaggagagtagctagagaaattgtacacaggtgtcaagcatgtaaaagaacagtaagt
tgccccaggaggggaacaaacccaagagaaagattcctatggcaaatggataatacccatctagaagggaaaa
ttatatgggtagctgtggaaacaaattcagggttgattgaggcgcgggttattccagaagaaagcgcacaatc
aatagtgttttgcattttaatgctcgtatacaggtacaccgtataccacatacatagtgacaacggccctgt
ttcatagcacaaaaagtagaagccttatgtaagtatctaaagattactaaaacaactggtataccttacaacc
ctcaagcacaagctatagtagaaagaacacacagagacatcaaagataaaatagcagcatttaggaagattg
tgagacagtagaggcggctttgagcctgaccctagtggccctaaataaaaaaagagggggaatagggggtcat

accccatatgaaatatacctagaatcagaatataacaaataccaagaacaacagaatcactataacaattta
aaacggaaaaatgggcctatgttagggacaaaaggaaagagatccacccttggataattcttttctgttttgc
aggggctataggggtgattggagggtggggcttacgggggaattgaatgtttgcatgctaattgttttagta
gtgttagtaccaatatactggggaataggggaagctgcaagaaacattgatagcttagattggaagtggatac
ggaaggtattcatagtaataattttgtacttgtggggttgctagggggctgctcagctcagagacagcatgt
ggctatgctgctgagcccccgggaataaggctgccaagtacggttgatatcccatggttttgcattagcaat
gctcccatacccgactgtgtacactggacagtgcagaagccagatcaaaaacaccaacagattgaaaacgtga
tggaattacaggaagtgctagataatgctaccttttttgaagtaccagacctatttgacagagtgtacctaga
attggcccgcttagatgcaaacagtactggggtccctgtaaatattccccctaccggaataagccaggttaag
ggagactgctcaacgggagacattcaagggatgaatgaaacactgagcactagaggaactctaggggagagaa
ctttcctgagcataaggccaggaggatggttcaccaacactactgtatggttctgtgtccattggccatttgg
atttatccaaagaaaggaaaatctgtcagaagggagtgctcaggtaagaaattgcctcgacccaataaatgtg
accgagccaagagtagctaattattcctactgccccttggaatacaaagggaagaactatataaacaaggggt
tgaagtgtgtaggaggaagggtagacttgagtagtaacccggagcaacatacagacctcctagcttgtggaac
attctgccagaactttcgtaattgtgatatggtgtcaagggacatcttaataggataccacccttcccagcag
aaacagcatatttatataaaccatacattctgggagcaggcaaatacacaatggatattggtgcaggtcccaa
actacgggtttgtgccagtcccagatacagaaagaccttggaaaggaggcaaacctaggggaaagagagccgt
ggggatggtcatattccttctcgtgttagctatcatggcgatgacagcatctgtcaccgcagctgctacgctc
gtgaagcaacacgccacggcacaagtagtggggagactgagcacgaatctaacttatatcactaagatacaga
atcaatacctgcacctgtttcaaaatctgaacacacgagttaataatctacatcatagggttacttaccttga
gtttttggcagaagtccatgaggtacaaactgggctagggtgtgtgccgagaggaagatattgccattttgac
tggcgcccagaggaggtaggattaaacatgacgctctggaattctaccacctggcaacaatggatgtcctact
acgatcagatagaagaaaacatatggaatttgaaatacaattggtctgaggccttagagaaaggaaagagtaa
tactgacggtttggagccggatgtcttccgatacttggcagacctatcctccagttttacatggggtagttgg
gtggataagcttgtatggctagcttatatacttttggcttattttgcatttaag
>#22 Puma lentivirus (PLV) MN531112 6951 nt
atggggaatgagtcagggaaagaggagagagtaattgcaaaaaggtgcaccaatgttgtgacaggcagtgggg
ggaagagtcccaaatattccataggcaatataagatgggctatgagatttgccttggtagctacaggacgaga
ccctcaggttagtcccacatctgtggaagacctgagagaacttattgcagacttagcagaaaaagaagataaa
tttgggggaagtaaagagcttacttgtgcaataaagactttaaaagtgttagtaatagcaggatgtctaaata
tgaaatgtgctaatacagactcagctatacatctatttaaaatattgggactggaagccagaccatcaaagaa
agaaggaattgaaaaggaagaaccagctcaagtggctcctatagtagcaggacccatgggagctacatataca
gcatttaatccaagaacagtagccatttggatggaaaaagctagagagggtatacatagtgaagaagctattt
tgtggtttacagcattctccacagacttaacacctaccgacatggcacaattgcttatgtcagctccaggatg
tgctgcagataaagaactaatagatcaaaagctgaaggaactgactaaagaatatgagaggacacatccttca
gatggtcccaggcctctgccatatcctactgcagcagaaataatgggaatcggaatccctcaagaacaacagg
ctatggctcactttgagcctgctagaaggcaatgtagagagtggtatttaatagccttaaatgaattaagaaa
aattagagcaggagcaccaagagcagtctctgttaaacaaggacctaaagagctatattcagaatttgtagat
agactatttaagcaaatagaagcagaatctgctcctcaggatgtcaaacaatatttaaaggattcattaagta
taagtaatgcaaatccagagtgtaaaagagcaatgagtcaccttagaccagaagacaaattggaagacaagat
tagggcttgccaagatatagggtcaacaacatacaagatgaatatgcttgcacaagcgttacaacaagttagg
gtacagcaagtgcaagtaaaacctcgaggtgctccaggaccacaaagaaagaaagggcctctcaaatgtttta
actgtggaaaaattggacatatggcaaaaatgtgtaaagctcctaagagatgcaataaatgtggaaaactagg
tcatattgcaatttgggaaatgaacacacgaccccgtgttgaggcctggataaatggaaaaaagattaatttt
ttattagattcaggggctgatatctcagtaataaatttaaaagattggaatcaaggtcaggctattaaagaag
ggcctcaacatatgacaggggttgggggagtgcaaaatgggatgaggtattctaatgtgcatatagaaattca
tacagaaaatgaaggaaaatgttgtaatggttatatgtgtgtccttccacaaaataatttagtagataattta
ttaggtagagataatttacaaaaactaggtataagaatggtaatggcaatgatttctgataaaataccaatcg
ttaaagcaaggatgaaagatccaaataatggaccaagagtcaaacaatggccattatcaaaagaaaaaataga
ggctctgacagaaatagtagaaagattagaaaaggaaggaaaagtagggagagctgatcccaataatccttgg
aatactcccgtattttgtataaaaaagagatcaggaaagtggagaatgttaattgattttagggaacttaatg
caaaaactgagaaaggagcagaggtgcagttggggttaccccatccatctggattacagaaaagaaagaatgt
aacagtcctggatataggagatgcctattttacaatccccttggatccagattatcaaccatatacagcattt
actttgccatctaaaaacaatcaaagcccaggaagaagatatatttggaaatctcttcctcaggggtggcttt
taagtcctttgatctatcaaagtactttagataatattttacaaccttttagacaaaaatatggaaaagaaat
tgatatatatcaatatatggatgatatttacattggttcagataaagaagtgaagacacataggaaaatagtt
caagaattaagggaattacttttatggtggggatttgaaacaccagaagataaactacaagaggcaccacctt

ataaatggatgggatatgaactccatcccaatgggtggaaaattcaaacagcaaagttggaaatacctaaaaa
ccccactttaaatgaactgcaaaagttagtaggaaaaattaattggcgactcaaataataggagggttgcct
attaaaaatttgactgaaatgatgaaaggcaatcccgacttaaattcaaaaagagaatggacttctgaagcta
gacaagaggcagaacaggcaaaagaagctatagaaaatctgcctaatggaaactattatgacaaaaataaaga
attatatgctattttgagtattagtggtccattacaaataagttatatggtctatcagttagaggggattaaa
aaacttcctttatggtatgggagaatgaatagagttaagagaaaaatagaaaatacttgtgatattgctatga
gagctataaataagattaaagaagagactgtaattaggttggggagagagccactctatcaaattccttgctc
aaaagagaattgggaaagttatattcagactagtaggtatctaaaaaatgtccctcctcaagtggaatttatt
aatagtagtctgatgatagaaagacatttagcatgtttaatggaggaccccatacaagatgaagaagcagaaa
catggtatattgatggaggaaggaaaaagggacaaaaggcaagagcaggatggtggaaaaataaccaggaatg
gcaaataatggaaatagaaggatctaatcaagtagcagaagcacaagctttaaatatggcattaaagtcaggg
ccagaaaagatgaatattataacagatagccaatatatatataatatggtaagagcaaggccagaacccaatg
actccctgtggaaagaaattgtagaagaacttcaaagaaaagagaaaatctttattgattgggttcctggaca
cagaggcatcccaggaaataaggaaatagatgaattagtacaatgtaacatgattattgaaggagcaggaata
cttcaaaaaagagaagaagatgcaggttatgatcttatagcatcagaaaagatgtattttcttccttcagaaa
caagaaaagttcctatagattgtaaaatggaattgcctcctgggacagtcggtctagtactaggaaaatcatc
tataggatgtaaaggattagatgtattaggaggagtaatagattcaggatacagaggagagatatcagtaatt
atgatgaattcttcaaaaaaggggcaatggatagaacaaggccaaaaaatagctcaattacttattatgccag
tacagcatgaaatattggaacaaggaaaagtacaaatgaactcagaaagaggagaacaaggatttggtagtac
aggagcatttgcattgcagtcctggatggattatatagaacaggcagaaatagagcatgaaaagtttcataca
gatgaaaagacattgcaagagaagtttggattacctaaggtagtagctaatgaaattttgcagaaatgtacaa
tctgcagaatgacaggaataggaagtggaaacagaggagggaaattaaagatgggacctggagtatggcaggt
agactgtactcatcttgaaggacaaataatattagtagccattaatgtggaatcaggattactatggacaaaa
ttattaaaacaagagacagctaaagaaacagtattagctgtattagaattagtacagacacataacgttgtgg
aaatacaaacagataatggacccaattttgcaaattcaaggatggaagggttaacagcattttttaggaatata
tcataaatatggaattccattgaatccacaaagtcaagcattagtagaaaatgttaatagcactttaaaagt
tggataaagaagtttagaccagaggtagaaactttacaggcagcagtagcattagctacatttgctctaaata
aaaaaagaaagggtaatataggggggtatgtctccctatgaactatatatatggcaggaaaacaacttaataca
acaatactactccaatatcatatttaaaaaatttggcacgaaacaatggatatattataaggacattaaagat
aaggtgtggaagggcccagtagaagttctctattgggcgaaggctcagttttgataaaagatcctgaaagag
gacttttcttagtccctaataggaggattcgaagagtacctccccctcaagaattagaggtaagtatggagaa
ccagcaggccagttggataggaccttatgaaggagaagccctgctagattggcaggtccttgcagccttagga
gaggatcagagaatcccatcgggaaaccccttccgggaagctggaatgactcaagaacagagagaggaattag
ctaaattcctccaacccatgctccaaggtattaagcaacaaatgttggaaggaggtgagttgaatccccatca
aagaggtaagtggcttatgcaaaaaaggatagatcaatattgggactctttggttaaattgagtacagggcgt
cgggtcaaaaacaaaataggcccacaagtaatagatatagaagcaggtcaaatagttaaacaatccaaagctc
cattgactcaatgtatcataagttttattttaataggattaggagcattagtatatgtaggattatttttagc
tataacttcatcaataagtaaggtcaatgctgtaaaaattgctattaaccctccttgggtggtgccagtcaaa
aataaagaggaaataaattttcaatgtataggtaatcatagtgaatgtatactatcagaggaatggggaagat
ggtctgcccctttaactggacttttcagactcatttaaatgagacacaaacgatggcagattatgaagcaga
gataagaggacaacaatatatgaatttgttaaaagaatgtagaagaggcccattcgattctgcttggagtgtg
gcctggtggacatgtttttatgatagagcactaagacaattaattggatatgataaagtaaggctgtgtccac
ttgggggatatatgttttataataatcagaccagaagattacaattatgcacagatgaattacatattggaat
tttgaatatgaccattagtcctataccttactggaacagaacaaatgagacagacccagataccccattaaat
ggatatctgtttgtagcgaacagacatttctatcttatgaacaatgctaccaacattacagaagaaagggtta
acattaacattacatgcaaggtgattgttccaacctcacatataacctataaaaaggaatttataggatataa
aaatgaattcttgggaccttggggaggctcaaggtataggtctataatgatcagacatcaatcctttgccaat
atgactaatccacctttggacttagattgctcagggatacctggaacaccatttaatggcacagaggcaaatt
actcttgtgcaaacaataccacaccatatggagaaatttgtactcagccaactctgtattttccatgctttaa
tcctaattatacaatacccatattggtgcaatgtgctttacatcaagaatatagaacaaatgatagtcatcgt
aatcaatcagatgatatgcaagttataagatgtagaatagcaagagagattgatattcaagaaatagatcaaa
ttacaaggttaaatctgacattagttaaagatccatttctaatctatttacaaggggctaataattttacatg
tacattacaaggacaattttggctatttaaaatgaataatgcttcatggaattggaacaccccaaatgctaca
tggctaacgccttggaaaaattttactcaagaaaattatgtttgggggcatattcctcagcaatatggaatt
ggtttcatttaaaacattactatttggtaaaacaaccagtgtatactatattaaaagatgatcctcatagaag
aaaaagaggaataggattaacagtggcattaatagggacctcggtggcagggctgataggagcaaccactggc
accacggccttagtagtatctcagaacctgagagggattatgctccaacaaacagagataaatgagcaaacct

tcaggatgctgaagctactgcagaggcgtgtggaacagtcagaacggatgatcctgatgcttcaccaacgggt
gtctaaaatagaaaactttttacaattaatacaaaatctaagaggaatgtgtcccttcaaagatatatgtgac
attccaatgcaatggaactttacaaattataatgaaagcgatacaataggaaagtgggcagaacaaagtgaaa
aagatagaattgaatttgaacaaattttaaataatgcgtcaaaagcaaatgagaacatgaaatatgacttaca
gaatatggatttgcagaactggctgagttggatgaattatagtacattagtaaaaacagtaatagtgatcata
atagggataataggaataatgctggcaccagtggtagtgcagataattaaaaatgtattgtatatgttccagg
gatacaaacctgttatggccaatctgatagaaataaaagaagaaacaggcaacgaagaaagagaagaagaaga
tggagagcaattatga
>#23 Visna/maedi virus (VMV) M60609 7530 nt
cccttcagtgaaggagaaagtgttgcttgggcacaggaggagggttcgcgaccccttagaaagacggaggggc
gcgggcgtcctcttgggccgaggggacacaagagcaacactggtaaggaagccgccgtggtgaggctagctag
agacatggcgaagcaaggctcaaaggagaaaaagggataccccgagctcaaggaagtaattaaagcaacttgt
aaaataagggtagggcccgggaaggagaccttgacggaagggaattgtctatgggcattaaaaactatagact
ttatatttgaggatttaaaaacagagccgtggacgattacaaaaatgtatacagtatgggatagattaaaagg
gctaactccggaggaaacaagcaaaagagaattcgcctccttgcaagctacgttggcttgcataatgtgtagt
caaatgggcatgaagcccgagacagtgcaggcagcaaagggaataataagtatgaaagaaggactacaagaaa
ataaggaggccaaggggggagaaggtagagcaactctaccccaacttagagaaacatagggaagtttaccctat
tgtgaatttgcaagcaggagggagaagttggaaggcggtagagtcagtagtcttccagcaactgcaaacagtg
gcaatgcagcatggacttgtgtccgaggattttgagaggcaattggcatattatgctactacctggactagta
aagatatattagaagtattggctatgatgcctgggaatagagcacagaaggaattaatacaaggaaaattaaa
tgaagaagcagaaaggtgggtaagacaaaatccacccgggccgaatgtcctcacggtggatcaaataatggga
gtgggacaaaccaatcagcaagcatctcaagccaatatggatcaggcaagacagatatgccggcagtgggtaa
taacagcgttaagatcagtgaggcatatgtcacatagaccaggaaaccctatgttagtgaagcagaagaatac
tgagagttatgaagacttcatagctcgcctactagaggctattgatgcggaaccagtgacggaccctataaaa
acatatttaaaagtaacattgtcatatacaaatgctagcacagactgtcaaaagcagatggataggacattag
ggacgagggttcaacaagcaacggtagaagaaaagatgcaagcatgtcgagatgtgggatccgaaggatttaa
gatgcaattattagcacaagctttgagaccgcaagggaaggcaggacacaaaggggtaaatcaaaagtgttat
aattgtggaaaaccaggacatctcgcaagacagtgtgaagcaccacccaaaatagaaataaaagtaggaacaa
ggtggaaaaaattattagtagatacgggggcagataaaactatagtaacatcccatgatatgtcagggatacc
aaagggaaggataatattacagggcatagggggaataatagaaggagaaaaatgggaacaagtacacttgcaa
tataaagataaaataatcagaggtaccatagtggtgttagctacgagtccggtagaagtattaggaagagata
atatgagcgaattgggaataggattaattatggcaaatttagaagaaaagaaaattcccattacagaagtaag
attaaaagagggatgtaagggaccccacatagcgcaatggcctttgacgcaagaaaaattagagggattaaaa
gaaatagtagacagattagagaaggaagggaaagtaggaagagcgcccccacactggacttgtaatacccctа
tattttgtattaagaagaaatcaggaaaatggaggatgttaatagattttagagagttaaataagcaaacaga
agatttagcagaagcacagttagggttaccgcatccaggaggattacagagaaagaaacatgtaacaatatta
gatataggagatgcatatttttacaataccattatatgagccatatagacaatatacatgctttaccatgttaa
gtccaaataatttaggaccatgtgtaagatattattggaaagtgttaccacaaggatggaaattaagtccttc
agtgtatcaatttacaatgcaaaaaatattaagaggatggatagaagaacaccctatgatacaatttggaata
tacatggatgatatctatataggagtgatttaggattagaagagcacaggggtatcgtgaacgaactagcat
catatatagcgcaatatggatttatgctgcctgaagataagaggcaagaaggatacccggctaaatggcttgg
atttgaattacatccggagaaatggaaatttcaaaaacatacgctcccagagattacagaaggacccataacc
ttgaataaactacagaaattagtaggagatttagtttggagacaatccctaataggaaaaagcatcccaaata
tcttaaaattaatggaaggagatagggctttacaaagtgaaagatacatagagagtatacatgtaagagaatg
ggaagcctgtagacaaaagctgaaggaaatggaaggaaattattatgatgaagagaaggatatctatggcaa
ctagattggggaaataaagcaatagaatacatagtatttcaagaaaaaggaaaacctttatgggtaaatgtag
tacatagtattaagaatttgagtcaagcccaacaaattatcaaagcagcacaaaaactgacacaagaagtaat
aataagaacaggcaagataccctggattttgttgccgggaagggaagaagattggatattagagttacaaatg
ggaaacataaattggatgccatcattttggtcatgttataaaggctcggtgaggtggaaaaagaggaatgtaa
tagcggaagtagtctcaggaccaacatattataccgatggaggaaagaaaaatgggcggggaagcctggggta
tattgcctccacaggtgaaaagtttagaatatatgaagaagggacaaatcagcagttagaattgagggccata
gaagaggcatgtaaacagggaccagaaaaaatgaatatagtaacagatagcaggtatgcatatgaatttatgt
tgcggaactgggacgaagaagtaataagaaaccctatacaagcgagaatcatggaattaatgcataataaaga
aaaaatagggggtacattgggtgcctggacacaaggggattcctcaaaatgaagaaatagataggtatatctca
gaaatatttttagcaaaagagggaagagggattttacaaaaaagggcagaagatgctggatatgacttaatat
gtccacaggagataagcattccggcgggacaagtgaaaaggatagcaattgacttgaaaataaatttgaaaaa
ggaccaatgggccatgatagggaccaaaagcagttttgcaaataagggagtatttgtacaaggaggtatcata

gattcgggatatcaaggaacaatacaagtagtaatatataatagtaataataaagaagtagtaataccacagg
gaagaaaatttgcacagttgatcctcatgcctctaatacatgaagagttggagccatggggagaaacaagaaa
aacagaaagaggggaacagggatttggatcaacagggatgtattggatagaaaatattcccctagcagaagaa
gagcataacaaatggcatcaagatgctgtgtccttgcatttagaatttgggattcccaggacagctgcagaag
atatagttcaacaatgtgatgtgtgtcaagaaaataaaatgcctagtacattaagaggcagtaataaagggg
catagaccattggcaagtagactatacccactatgaagacaagataatattggtctgggtagaaacaaattca
ggactaatctatgcagaaagggtaaaaggagaaacaggacaagaatttagggtgcaaactatgaaatggtatg
cgatgtttgccccgaaatcattgcagtctgataacggaccagcatttgtagcagaatctactcagctcttaat
gaaatatttgggcatagaacatactacagggatcccctggaacccacaatctcaagcattagtagagagaaca
catcagacgttaaagaatacattagaaaaacttatacctatgtttaacgcgtttgaatcagccctcgcaggga
ccctcattactctaaatataaaaagaaagggtgggctagggacaagccctatggatatatttatatttaataa
ggaacaacaaagaatacagcaacaaagtaaatcaaaacaagaaaaaattcgattttgttattacagaacaaga
aaaagagggcatccaggagagtggcaaggaccaacacaggtactttggggcggggacggtgcgattgtagtga
aagacagaggcacagatagatatctggtgatagctaacaaagatgttaagttcataccgccaccaaaagaaat
acaaaaagaataaactgtgataagtctgtctaggatgaccagcaaagaaagtaagccaagcagaacaacgtgg
agaggcatggagcccccactccgagaaacatggaatcaagtattacaggagctagtaaaaagacaacaacagg
aggaagaagaacaacagggactggtatcaggtaagaaaaaatcatgggtaagtattgacctcctggggacaga
aggaaaagatattaagaaggtaaacatatgggaaccatgtgaaaagtggtttgcacaggtgatatgggggta
ttgtgggtgctgcaaattgtgttgtggggatgtctcatgtgggaaatgagaaaaggaaaccagtgtcaagcag
aagaagtgatagcattggtgagtgatcctggaggatttcagagggtgcaacacgtagaaacagtgcccgtgac
ctgtgtgacaaaaaatttcactcaatggggatgtcaacccgagggggcctatccagatcctgaactagagtat
agaaatatatctagggaaatcttagaggaagtatataaacaagactggccctggaatacttatcattggcctt
tatggcaaatggaaaatatgaggcaatggatgaaagaaaatgaaaaggagtataaggaaagaacaaacaaaac
gaaggaagatatagatgatctggtagcaggaagaataaggggaagattttgtgtgccatatccctatgcttta
ctacgatgtgaggaatggtgttggtaccctgaatctattaatcaggaaacaggacatgctgaaaaaataaaaa
taaactgcacaaaagcaaaagcagtttcttgtacagaaaaaatgcccttagcagcagtccagcgggtatattg
ggaaaaagaggatgaagaaagcatgaagtttttgaatataaaagcctgtaatatatcattgaggtgtcaagat
gaaggaaagagtccgggaggatgtgtacaaggataccccattccaaagggagcagaaataataccagaagcta
tgaaatacctcaggggaaagaaaagtcgatatggagggatcaaggataaaaatggggaattaaaattacctct
ctctgtaagggtgtgggtaaggatggctaatctgtcaggatgggtgaatggaacgcctccttactggagtgca
agggttaatgggagtacagggataaatggaacaaggtggtatggagtaggaacactacatcacttagggtata
acattagcagtaacccagagagagggatatgtgactttacaggagaactttggataggggtgacaaattccc
atattattataagccctcatggaattgttcacaaaattggacaggtcacccagtatggcaagtatttagatac
ttggatatgacagaacatatgacaagcagatgtatacaaagacctgaaagacataatataacagtaggaaatg
gaacaataacggggaattgtagtgtaacaaattgggatggatgtaactgtacaaggtcaggaaatcatttgta
taacagcacaagcggaggactgttagtaatcatatgcaggcaaaatagcaccataacaggaataatgggaacg
aacactaattggacaacaatgtggaacatctaccaaaattgctcaaaatgtaacaacagtagcctagacagaa
caggtaatgggaccttgggaacagtgaacgaccttaagtgtagcttaccacacagaaatgagagtaacaagtg
gacatgtgcggcgagaaggaaggggagccgaagagactcattgtatatagctggaagagacttctgggcaga
gtaaaagcaaaatacagttgtgagagtaatttagggggtctagatagtatgatgcatcagcaaatgttactgc
aacgataccaagtaataagagtaagagcttacacatatggtgttgtagaaatgccacaatcgtatatggaggc
acaaggggaaaacaggaggtcaagaaggaacctccaaaggaagaaaagagggataggcttggttattgtgctg
gccatcatggcaataatcgctgctgcaggagctggtctcggtgtcgcaaacgccgtgcagcaatcctatacca
ggacggctgtccagtctcttgctaacgcaactgctgcccagcaggaagtgttagaagcatcgtatgccatggt
acagcatatagccaaaggaataagaatcctagaggcaagagtggctagggtggaagccctggtagatcgaatg
atggtataccatgaattggattgctggcattatcaacactactgtgtaacctcaacaagaagtgaagtagcca
attatgtaaactggacaagatttaaagataactgtacctggcaacagtgggaagaagaaatagaacaacacga
gggaaacttgagtctattactcagagaagcagcattacaagtacacatagctcaaagagatgctaggagaatc
ccagatgcttggaaagcaatacaagaagcatttaactggtcctcttggttctcgtggctcaaatatataccct
ggattatcatgggaattgtaggattaatatgttttagaatactaatgtgtgtgatatcaatgtgtttgcaggc
ttacaagcaagtaaagcagatcagatatacacaggtaacagtggtgatagaagcaccggtggaattggaggaa
aaacaaaaaagaaacggggatggtacaaatggctgcgcaagcttagagcgcgagagaagaacatcccatcgca
gttttatccagatatggagagcaacatggtgggcatggaaaacctcaccttggagacacagctggaggacaat
gccctatataaccttgctacccatgttggtgatatggcagtggatggaagagaatggatggaatggagagaat
cagcacaaaaagaaaaaagaaagggtggactgtcaggacagagaacaaatgcctaccctggaaaatgactatg
tagagttatag

Supplementary File S2. Dataset of 96 nucleotide sequences from primate lentiviruses: 37 sequences from SIV infecting *Cercopithecinae* (#1 - #37), 4 from HIV-2 (#38 - #41), 20 from SIV infecting African apes (#42 - #61), and 35 from HIV-1 (9 from group O, #62 - #70, 2 from group P, #71 and #72, 7 from group N, #73 - #79, and 17 from the pandemic group M, #80 - #96). From each genome sequence we selected the non-overlapping region that encodes the 8 proteins common to all primate lentiviruses (*gag*, *pol*, *env*, *tat*, *rev*, *vif*, *vpr*, and *nef*) and we excluded the coding region of *env* that corresponds to the Rev Responsive Element (RRE, 246 nt). Each nucleotide sequence contains the NCBI acc. number and the length of the coding region.

```
>#1  AF468658  7257 nt
atgggagcgaggcactcggcgatgctgacgggtactaagttggataggtatgagaaagtgagtctcagaccgaga
ggaaagaagaagtacgcgatcaagcacttagtatgggcctccaaggagcttgagagatacgcgctatcaggcagt
cttcttgaatctaaagaagggtgtcaacaggtattggaagtcacctccccattagaaccaaatgggtccgaatct
ctcaaaagcctctatggaatcgtgtcagtgctctattgtatccatgcagacatatgggtagaagatacagagcag
gctaagaagcaagttcagataaggtgtcatctagtaggaacaaaagagaaagaaacacagaacaaagacccccca
ggggccgcaggggacaggcggtagttagtcagaactaccccatagttagaaatgctcagggacagtttcaacac
caggccctcaacagcagagtccttaagacttgggtaagcatagtggaagaaaagaagtttgctccagagacagtc
gctctgtttcaggctctaacagaaggctgtataccttatgatatgaaccaaatgctcaacgctgttggagactat
caaggagcagtacagattattaaagatgtcatcaatgaacaggcagcagagtgggatctcttacatccgcagccc
gcggcaccccaaccagtagcagggcttagagaccctagcggagcagacatagcaggagtaacatcaactcctaat
gagcagatagagtggattacaaggcagaacaatccagttaatgtggcagatatatatagaaaatggatcatcttg
ggcttgcagcgatgtgtaaaaatgtataaccctgtcaatattttgggcatcaaacagggccctaaggaaccattt
aaggaatatgtagacagattctttaaatgcttaagggctgaacaggcagatcaggcagtaaagaattggatgaca
caattgctgctggttcaaaatgctaatccagaatgcaagctgatcttgaaggccatgccaggagccagcttggaa
gaaatgcttacagcatgccaaggggtaggggaccaactcataaatctagactcttggcagaggccatggcaaca
gcactcaaaggcagctcatacaatatggtgcaaagaggcccccaggaaggcaaggaaaaccccaaaatgttac
aattgtggaaagtttggacatatcgcaagagactgtcccaaacccaaggaaagaaagtgttttaaatgtggcaag
gcaggacacttagctaggcaatgcaaaacaggaacaccagccaaggagcttgaagtctccatgggggacaagtg
gtgtccatgttattagacacaggggcggacgataccattgtccaagaatcagcaattgaattagacacaccatgg
acaccaaaaacggtagggggatagggggtttaatacaagtaaaggaacatagacatgtagaagtgatctttaat
gaaaaaagaatcaaagccactgtacttgtagggcccacaccagtgaatatattgggaagaaattgtttaagcaag
ttaggagtaaccttaaacatggtgcagcagaaactagagcccgtagaagtgcacctaaaatcaggaaaagaagga
ccaaagattaaacaatggcccttgtctaaggaaaagatagaggccttaacccaaataacacaagaaatgctcaag
ttaggccaattggaaaagatagggccagaaaatccatacaactcgccagtctttgccataaaaaagaaagacaaa
tcacaatggagaatgttaatagatttcaggaaattgaatgaggctacgcaagacttggctgaggtccagttgggg
attccgcacccagcaggactagaacaaaaagagcacgtaactataatagatatgaaagacgcttattacagtgta
ccattgtatgaagaattcagaaagtacactgcattttcagtaccatcagtaaacaatcagaccccagctgagagg
tatcagttcaaagttttacctcagggttggaaggcgtctccaaccatttttcaggccactgtagcttccttgtta
tatcagattagagaccaggagccagatgtggtaataatacagtacatggatgacttgttgataggatcagatagg
aaattggcagagcataggcaagtagtgcacaaaataagaaatttgttgacgtcctataacatccagacccccgag
gccaaacaccaacaagattatccagtcagatggctcgggtatgagttgcaccctaagggatggaggctccaacca
gtggagctcccagatcaagacatctggacagtaaatgacatccaaaaattggtagggaagctcaattgggtatcg
caaatacatcctggcataaggacaaaacagttatgcagatgtattagaggagccaaagggctaacagaacaggtg
gagctcactgaggaagcacaaatagagttagcagaaaacagagagattctaaaacaatcctcagagggcagctac
tacgacgcagaaaagccacttgtggtagaaattacctccctaggagaacagcaatggggatacatgttcagtcag
gacagcaagatgctaagatcaggcaaatttgcaaaaaccagaactgcacatataaactcttatcagcaattggca
```

gatgctatgaccaaagtaggtagggaaagtcaggttacttggggaaaagtacctgacaaattcaggatcccagta
gtcaaggagcaatgggatacgtggtggatgaactattggcaggcaacatggattcccaccatagaagcagtacac
acaccacatttgttgagacagtggtacaccctagtctcggaacccctagaaaatgcagtcacctattatgtagac
ggggcagcaaataggacctctaaattaggaaaagctggctatgtgaccaatacaggaaaatatagagccatagaa
ctagaaggaactacaaatcaacaagcagaactccaggcagttcttttggcattaaaagaaggaccaccaagaatg
aacttggtcacagattcccaatacgtaatggggatcttacaaagccaaccagaagtatccacctctcccttagtg
gaacaaatcatacaagaactgttagggaaagaggccgtctacttgagttgggttccagcacacaaagggatagga
ggaaacacagaggtagacaaattagtcagcagagggatccgccaagtactcttcatggagaacatagagcccgca
gtagaggaccatgaaaaatatcatagtaattggaagtatttaagggatcaatacaaaatcccggcactattggct
aaagaaatagtaaacaagtgctccaaatgtcagatacatggggaaccaaaacatgggcaagtaaatgcagaatta
ggcatatggcaaatggactgcacacatctggaaggcaaagttatcttagtggcagtccacgtagccagtgggtat
gtttgggctaggataataccccaagaaacaggcagacaaactgcattaaagctattagaattagcagcaacatgg
ccagtaactcacctacatacagataatggccccaatttcatctctaaggaactggaggcagcttgttggtgggca
aacatacaacacagtacaggggtgccatacaacccgcaaagtcaaggggtagtagaaaacatgaacaaacaactc
aaggaaacaatccaaaagattagagatgaggtcacatacttagaaacagcagtagcacaagcatgctacattcat
aattttaaaagaaagggaggaataggggatatgtgccctacagagaggttagtgaacatgatccacacagaatta
gaaacacaacacttaaacacacaaagttccaaatttcaaaaatttcgggtttattacaggcaaggagctaatcct
cattggcaaggaccagcagtacttctctggaagggtgaaggagcagtagtggtccaaacccaagcaggtgagatc
atcacagtccctagaaggaaagcaaagatcatcaagccagcaactaaacatcatatatggtcaggaaaaactcca
tttgtgtatatccaccactatcaactacaacatcaaaggtttacacaaaacaaaattcgattagcattagatact
aggcaggtaggagaggaagtagaagccacatatattgagataaccatcctatgggacacaactagtcatggacca
gccagtctatcccgatccacctactaccaacaggcagtcacaatagagtgggtttacaacagaagtaagctagga
gcaagggaaacagacataacatggtacagtactaatttgaccccagacgtagcaatgcaaatcatacacacaagg
tactttccatgttttcagaacgaagacgtacggagggcaataaggggtgaacagctcttaggatattgtgaccac
ccggaagctcacccacaggtaggaacacctcacacgcttgaaaggctagccttctttgcgtacgttagatacgta
acggagctccctgccgatccggattggacagtggatcaggcagcaatagcatgtgcaatagattatgtcaggaga
gtgcagacattactctttagacactttagagatgggtgtttccacagatacaatagaattgtcagaaggtaccca
gtaatcaggcccttaaggggtaccgccccaccagatagtaactctgttcctgacccggcagagtgtcttttgcct
aagtggcaacagccaggagccgcgccaaacaccccgtgctctgcttgctattgcaaaaggtgtgcttttcattgt
cagttgtgttttctaaggaagggactaggtctttccctttccgggaactggacgacagtgttctatggggtaccg
gtatggagagatgcaaaaccaccattgttctgtgcctcagatgcagacataactagtaatgaaccaggaaacatt
tggatctctacggcatgcttgccctcagacccctcaccgtctgaggtgccactaaacataacagaggaatttaat
atctataaaaattatatggtagatgaggtgagagatgacatggtatctctatttaatcaggccctaagaccatgt
gtcaaactgactccgatgtgtgtacgaatgaaatgtaagctcccaaatacgagcacaacaacaacaccgtccaca
acaacagccacaacatcaccctcatccccaacgccaacaccatggggaaattggggaggtaatggaacaggacag
ccaatatataactgttcctttaaccagactacagaatttagggacaaaaagagacagatgtatagcctcttttgg
aaagatgatattatgagggcacaggatggtaatgacactgactattatattataaattgcaacacatcatatgtc
acgcaaaagtgtgtcaagacctccttccaaccggttcccatacactactgtgctccgcccggctttgctatgcta
aaatgcaatgatgccaatttcacaggagtgggcaagtgttccaatgtatcagcagtaacatgcacgcatggcatt
caacctttagtagcaacatggcttcatctaaatgggacttatcagccagggaacaataccagagtaatgatgaat
ggcaaaaagaatgaatctatagtcataggctttggggaagattatcagcttactcttacatgtatcagacctgga
aataaaaccctaaaaaatttacagataggagcaggaatgacattctactcccaaattatagtagggggagatact
aggaaggcctattgcaaactcaaccatacaaagtgggatatagcaataaggcaagccatgaaggccatgaagaac
cattgggaaaagatcaacaatgataccacccctaataagactcaaataaggtggacctccgaacctaaaggagat
ctagaggtccaaactcattggttccaatgtcagggagaatttttctattgtaatttgtcagtactctttcaattc
aacaacaatattacggcagtggatgacacaaacattaacaatgtcacaagcaaatacaaaggacaatggatggct
tgcagaattaggcaatttgtcacacaatggggatatgtgtctaggtcgatctacttacctccacggcaaggtcat
ataaattgtacatctaacatcactggcctcctaatagacggagcgatgtatggacattccatcaatatgacccca
tcggccgatgtagccgatgcatggaagtatgagttatccaggtacaaggtggtggagatagatccactttcgctg
gccccaacctcggctcaaaggagaccacatccaggggtgcatgtgaaaaagaggcaagcttggggctgtgcgaat
cagccaatctgtcacaccatagtaccttggaatgactcatgggctaagaactcgacgccagattgggagcacatg
acctggcaggagtggagtaagttaattgaaaatgacacatataccatacagcaattattagaaaatgcaaaccat
caacaatcaaagaacatgaatgacttattaaagttatccaaatgggactccctttggagttggtttgatatttcc
aattggctctggtacattaagattttcataatggtagttgcagctttagtagctttgagaataataatgtttgtt
cttaatatgcttagaagggttgaatcaccctccaggcaaaattgcatcgattatgaaccatcatgcccagtgaga

ccacaggtgccgcttagagaccccacctacaaacttatggtggatctctcgcacttttgaaagaaaagggagga
ctggaggccatgttttattgtgaagatagacaccaaaagctagagagctattgctattatgagtggggcattgtg
cctggatggctccagtggactccaggaccaggaatccgctacccaactatgccaggcttctgctggtgcttacgg
ccagtggccatgactgaggactctgagcccggggatgatcaatatctgttgaaccatcccgcctatcagggacag
caagaggaccaccatagggagatcttggtcttctccttctgctctaggctagccttgaaatcaggctggcagatg
aaccagctgcagcaggaggagcggaagaagcgcctaaccgcaaaccgcttcctatag
>#2 AF468659 7182 nt
atgggagcgaggcactcggcgatgctgacaggtactaagttggataggtatgagaaagtgcgtctcagaccaaag
ggaaagaaaaagtacatgatcaagcacttagtatgggcctccaaagagcttgagagatatgcgctgtccgacgct
cttcttgaaaatcaagaggggtgtcgacggatattagaaattgtcttcccccctagaaccaacagggtcagaagct
ctcaaatctctctttggaatcgtgtcagtgctctattgtatccatgcagacatacaggtggaggatacagagcag
gctaagcagcaagtcaggataagatgtcatctagaggagaccaagggtaaagaaaagcagaataaagacccccg
ggggccgcaggggacaggcggtagttagtcaaaactaccctgtgattaggaatgcccaaggacagtatcaacat
caggccctcaatagtagaattcttaagacttgggtaagcatagtggaggagaaaaagtttgctccagagacagtt
gccttgtttcaggctttatcagaaggctgtataccctatgatatgaaccaaatgctcaatgctataggagattat
caaggggcagtacagattattaaagatgtcatcaatgaacaggcagcagagtgggaccttttacatccggtacca
gcagcacctcaaccggtggcagggcttagagaccctagcggagcagacatagcaggagttacttcaactcccaat
gagcagatagagtggatcacaaggcagaaccaacctgtcaatgtgtcagatatatatagaaagtggatcatttg
ggcctgcagcgatgtgtaaaaatgtataaccctgtcaatattttggacatcaaacaaggccctaaagagcccttt
aaggaatatgtagacagattctttaagtgcttaagggccgaacaagcagatcaggccgtgaaaaattggatgaca
caatcgctgctggttcaaaatgctaacccagaatgcaagctgatcttgaaagccatgccaggagccaacttggaa
gaaatgcttacagcatgccaaggggtaggggaccaactcacaaatctaggctcttagcagaagccatggcaaca
gccctcaaaggcactagttcatacaacatggtgcagaaaggcccccctggaaggcaaggaaaaaccccaaaatgt
tataattgtggacaattcggacacctagcaagagattgtcctaaacccaaagaaagaaagtgctttaaatgtggc
agagcagggcactttagcaaaccatgtagaacagggacaccaatcaaggagcttgaagtctccataggggggacaa
gtggtgtccatgttattagacacaggggcggacgacaccatcgtccaagactcagcaattgaattagatacacca
tggacaccaaaaatggtaggggggaatagggggtttaatacaagtaagggagcataaacatgtagaagtaatcttt
aatgagaaaagaatcaaagctactgtactggttggacccacaccagtaaatatattaggaagaaattgcttaagt
aagttaggggtaactttaaacatggtgcagcagaagatagaacccatagaagtgcacctaaaggcaggaaaagag
ggaccaaagatcaaacagtggcccctgtctagggaaaagatagaggccttaacccaaataacacaagagatgctt
agattaggtcaattggaaaaaataggaccagaaaatccatacaactcaccagtctttgccataaaaaagaaggac
aaatcacaatggagaatgctaatagattttagaaaattaaatgaggctacgcaagacttggctgaggtccagttg
ggaattccgcacccagcaggaattccgcaccctgcaggactagagcaaaaggagcatgtaactataatagatatg
aaagatgcttattatagtataccattgtatgaggatttcaggaagtacaccgctttttcagtaccttcagtaaat
aatcaggccccagctgaaaggtatcagttcaaagtcttaccgcagggttggaaagcgtcgccaaccatctttcaa
aacattgtagcttccttgctacgacagattagagatcaggagccagatgtggtattaatacagtacatggatgat
ttactgataggatcagataggaaactgggagaacacaggcaagtagtgcacaaaataagaaatttactaacatcc
tataacatccaaacccccgaggccaaacaccaaccagattatccggtcaagtggctcgggtatgagttgcatcct
aagggatggaggctccagccggtggaactcccagaccaggatacctggacagtgaatgacatccagaaattggta
gggaaattaaattgggtatcacaaatacaccctggtataaggacaaaacagttgtgcaaatgtattaggggagct
aaagggctaacagaccaggtagagctcactgaggaggcacagatagaattagcagaaaatagggagatcttaaaa
caatcttcagagggtggctattatgacgcagaaaagccgctcgtggtggaaattacctccctaggagaacagcaa
tggggggtacatgttcagccaagaaggcaagatgttaaaatcagccaaattcgcaaagaccagaaatgcacacata
aactcctatcagcaatttgcagatgctttgaccaaagtaggtagggagagtcaggttacctggggaaaggtacca
gacaaatttaggatcccagtagtcaaggaacaatgggatgcatggtggatgaattattggcaggcaacatggata
cccaccatagaagcaatacacacaccacacctactgagacaatggtacaccctagtctcggaacccctagaagat
gcagttacttattatgtagatggagcagcaaataggacctccaaactaggaaaagctggttatgtgaccaataca
ggaaaacatagggccatagaattagaagaaactacaaatcagcaagcagaactccatgcagttcttttggcatta
aaagaagggccaccaaaaatgaatctggtcacagattcccagtatgtaatggggatcttgcaaagtcagccagag
gtatccacctctgcgctagtggaacaaatcatacaagaactgctaaaaaaggaagccatctacttaagttgggtt
ccggcacacaagggaataggaggaaacacagaggtggacaaattagtcagcaagggaatccgccaagtgcttttc
ctggaaaacatagagcctgcagtagaagaccatgaaaagtatcatagtaattggaagtacctaagagatcaatac
aaaatccccgcactattggctaaagaaatagtgaacaaatgttccaagtgtcaggtacatggggaaccaaaacat
gggcaggtaaatgcagaattaggcatgtggcagatggactgcacgcatttggaaggaaaggtcatcttagtggcg
gtccacgtagcaagtgggtatgtctgggctaggataataccccaggaaacaggcagacaaactgcattgaagctc

ttagaattagcagcaacatggcctgtaacccacctacatacagataatggccccaatttcgtctccaaggaattg
gaagcagcctgctggtgggcagacatacaacacaccacaggggtgccatacaacccgcaaagtcaaggggtagta
gaaaacatgaacaaacaactcaaggaaacaatcacgaaaatcagagaagaggtcacatacttagaaacagcagta
gcacaggcatgctacattcataattttaaaagaaagggaggaataggggatatgtgccctacagaaagaatagta
aatatgattcacacagaattagaaacacaacacttaaacacacaaaattccaaatttcaaaaatttcgggtttat
tacaggcaaggagctaaccctctttggcaaggaccagcagtactcctctggaaaggtgaaggagcagtagtagtc
caaactcaagcaggtgagattattacagtccctagaaggaaagcaaagatcatcaagccaaggaaggtagactgg
ttgcttagagctactaaacaccacatatggtcaggcaaaacaccatttgtatatgtccatcactaccaattgcaa
caccaaagatttacacaaaacaagattcgattagcaatggatactaggaaggtaggggaagaagtagaagcaaca
tatatagagataactatcctatgggacacaaccagtcatggaccagctagcttatcccggtccacctattaccaa
caagcagtcataatagaatgggtatacaacagaagcaaactaggagcaagagagggagacataatttggtacagc
actaatctgaccccaggagtagcaatgcaaattatacatggaaaatactttccatgctttcagaacgaagacata
cggagggcaataaggggtgagcagatcctaggaaattgtgaacacccggaagctcacctgcaggtaggaacacct
cagacgcttgagcagttagccttctttgcatatgtcaaacatgtgacggagctccctgccgatccagattggaca
gtggatcaggcagcaatagcatgtgcaatagattacatcagggtgcaaacacttctctttagacactttaaagac
ccggaagagtgtctcttgcccaactggcagcagccaggagcggcgcccgccaccccgtgctctgcttgctactgc
aagaagtgtgcccttcactgtcagttgtgctttctaaggaagggactaggtctttccctccataggtccagtaat
tggacgacagtgtactatggggtaccggtatggagggacgcaactccaccattgttttgcgcctcagatccagat
atagctagtaatgaaccaggaaacatttggatttctacagcatgtttgccctcggacccctcaccggcagaggtg
ccgttaaacataacagagaaattcaacatctataaaaattacatggtagatgaggtgcgagatgacatggtatca
ctatttaatcaggccctgaaaccatgtgtcaaattgactccaatgtgtgtggggatgtattgtaacctcacaaac
acatccacaagcgaaccaacaacaactccaaggccaccaaatgtctccacaaccacgcagtggggaagttggggg
ggggaaaatggaacaggacaacccttatataactgttcctttaatcagaccacagaatttagagaccaaaagaaa
cagatgtatagcctcttctggagagaagacattatggaagagacccatggtaaccagagtggatattatattaga
aattgtaatacctcatatatcactcaaaaatgtgtcaagtcctccttccaaccggttcccatacactactgtgct
ccacccggctatgctatgctaaaatgcaatgatgttaatttcacaggagtaggcacatgctacaatgtgtcagca
gtaacctgcacacatggcattcaacccttagtagcaacatggctccatttaaatgggacctatcagccaggaaat
aataccagaataatgatgaatggtatgaagaatgaatctatagttataggctttggagaagattatcatcttaat
cttacatgtatcagacctggaaataaaaccataagaaatttgcaaataggagcgggaatgacattttattcccaa
gtgatagtaggaggaaatactaggaaagcctattgccgacttgatcctgataggtggaatagggcaataagagaa
gctatgaaagcaatgaatgaacattgggaaaataaaactgggagaaatgatactcaaataagatggacctcagag
cctaaaggagatctagaggttcaaactcattggttccaatgtcaaggagaattttctattgcaatttgtcaata
ctctttcaacttaataataacaccattaacagcagcaatattgggaacatcacaagcaaatacaaaggacaatgg
ctggcttgcaaaattaggcaatttgtcacccaatggggatatgtgtctaaatcaatctacttacctccacggcag
ggtcatataaattgcacatccaacattacaggtctgttgatagatggcgctatgtatgaaagctctatcaacatg
accccgtcggccgatgtggctgatgcgtggaactatgaattgtccaggtacaaagtggtggagatagatccgctc
tcgatggccccaactccggctaaaggaaagaacaccccgctgtggagaaaagacaagcatggggctgtgcgaac
aggccaatctgtcacaccatagtaccttggaacacctcatgggctaatggctcgcttccagactgggagaacatg
acctggcaaaagtggagtatgttagttgagaatgacacatatacaatacagcagttgttagagcaagcaaaccag
caacaagcatcaaacctgaatgagctgatgaagttgtccaaatgggactccctttggagttggtttgatatttcc
gattggcagaggtacattaagatctttgtaatagtagttgcagccttaatagctttaagaatagtaatgtttatc
cttaatatgcttagaagggagccaaaacccaagagaggaagcctcgaggagtatgaaccatcatgcccagtaaga
ccacgggtaccacttagagaccccacctacaaagtcatggtggatctctctcacttttgaaagaaaaggagga
ctggagggcatgttttactgtgaggacaggcaccagaagttggaacaatatgcctatctggagtggggcctggtg
cctggatggctctcattcactccaggcccaggaacccgctatcctacgataccaggattctgcatctgtctgcgg
ccagtagccacaactgaggactctgagcctggggatgatgaatatctgctgacccaccctgcctatcagggacgg
agtgaagaccagcataaggagttcttggtcttctccttctgctccaagctggccataaagtcaggcatacagcta
gaccagctgcagcaggaggagcggaagatgcgcctaaccgcaaaccgcttcctatag
>#3 EF070329 7377 nt
atgggggcgaggcactcggcgatgctgaaaggtactaagttgaataaatatgagaaggtcagattaaggcctaaa
ggaaagaagaagtacctaataaagcatatagtatgggcttccagagagcttgagcggttcggtctctcagatacg
ctcctagagagcaaggacggatgccaacgcatcctagaagtaatctgccccttggaagaaactgggagcgaggcg
ctcaaatcactctttggaatcgtgtcagtactctggtgtatccatgcagacgtaacagtgaaagacacagaggag
gctaaaaagcaaattaggataaggtgccacctagcggagaaaaagaagactcaaaaggagactcaggagcaaaa
gccaccaccagtggccagaattacccagtcataagaacagcacagggacagtatcagcatcagtctcttagccct

aggctattaaaaacctgggtatcaacagtagaggagaaaaaatttgccccagaagtagtggccctgttccaggcc
ctatcagaaggatgcatcccctatgatgtaaatcagatgctcaacgccataggagaccaccaggggcaatacaa
ctaataaaagatgtggtcaatgagcaggcagcagacagagacgttttacatccccaaccaacacagccacaacca
aatgcagggctaaggtacccatcaggggctgatgtggcgggagtgtcatcaacgccagcagaacaaatagaatgg
atgactagacagcaagacccagtaaatgtagggaatatatatagaaaatggatcatcttgggcctacagagatgt
gtcaaaatgtacaatccagtaaacatcttggatattaaacaaggccctaaagaaccattcaaagattatgtggat
cggttctttaaatgcttaagagctgaacaggctgaccaagcagtcaaaaattggatgacacagtccctgctagtg
caaaatgccaaccccgagtgcaaactcgtcttgaaagccatgccaggggccacattaaaagagatgttacaggcc
tgccaggggattgagggacccatgcacaagtcaaggctgatggcagaagccatgactaatgctttgaggcaaaac
accattaacaccatcaacatggtacaaagacagagtcccaggggagtaatgggaaaaaagagggaaaacagcaca
cgttgctacaactgtggacaatttggacatttggcaagagattgccccaagcctaagtctaccagatgttttaag
tgtggtaaggaaggacacttggccagacagtgccgaaccgacacaggcaaaagcgctgtgattaatgggcagcca
gtgtccatgctcctggacaccggagcggatgacaccatcatccaagactcagatataaaattagtggggaattg
actcccaaagtagtgggaggtttagggggctgatcaatgtaaaacaatataagggggtgatagtaaaattcaat
gagaaaagaatagtagccacagtcttggtgagtcctacacccatcaatatcttagggagaaattgcttatcaaaa
ttggaaataacactaaacatggtaatggcaggacagcaattgaaacccactaaagttagccttaaagaaagaaaa
aaagggccaatggtaaaacaatggccactctctaaggagaagatagaggcactaaaagaaattactcagaaaatg
ataaaattaggtcaactggaggaagcagggcctaataacccatataattcaccagtattcgccataagaaagaaa
gacaagacaaaatggcgaatgttaatagatttcagaaagcttaatgaggtgacacaagaatttgcagaagtccag
acagggatcccacatccctcagggttagcccaaaaggcacatgtcactatagtagatatgcaggatgccttttat
agtgtcccattagacaaagaattccgaccctacacggccttctcggtccctgcagtgaacaacatgggaccagct
aaaagattccaatttaaggtgttgccacaggggtggaagggatcacccaccatttttcaagccacaacagccaag
cttctagagcaagttaggaaggacaatcaggacgccttaatagtccaatacatggatgacctgcttatagggtca
gacagagaaataggagaacatagaaggctagtgaaaaagatcagagatctcctctcaagtaaggggatacagacc
ccagaagacaaacaccagccagactacccggtagaatggctagggtacgagttacatccgaaaggatggagaatc
aaaccagtggagctgccagaccaggacacctggacagtgaatgaaattcagaagctggtagggaagttaaactgg
gcagcccaagtctattcagggatcaaaactaaacatttatgcagatgtatcaggggaataaagggggctaacagag
ctagtggagctcagtgagcaagcccaattagaactagcagaaaatagggaaatcctgaagcaagaggccggtggg
gcctattatgacccagaaaaacccctagtcttagagatagtgtccttaggggaacaacaatgggggtacaccttt
acccaagacagaaacatgctaagaacaggaaagtttgccaagatcagaacagcccatagtaacccctaccaacag
ctggcagaagccctatctagagctagtaaggaggccctagtctgttggggaagaccccagacaaatgcaggata
ccggtagtcaaggaacagtgggacaattggtgggcagactcatggcaaacaacttggattcccgacatagaagca
gtacacaccccataccttctgagacagtggtttaccttggtcccagagcccatagaaacagcacccacctactat
gtggatggagcagcaaataggaactctaaactaggaagagctggatatgtaaccgataggggacaggagagagct
ataaatttggaaaacacaaccaaccagcaagcagagctccaagccattctcttggctctaatggatgggccacca
gaaatgaacctagtaacagattctcaatatgcattgggaataatcacagcagcaccagaggtatcagagtcccct
ctggtggagcagataatacagcaaatgctgagcaaaaatgccatctttatatcccgagtaccagcccacaagggc
ataggagggaatgaggaagtagaccacctagttagcaggggaatccgccaggtactatttatggaaaatatagac
ccggcagttgaagaccatgagaagtaccattctaattggaagtatctcagggataaatacaacatacccaccatc
ttagccaaggaaatagtaaacaaatgctcagcatgtcaaacacatggggaaccaaaacatgggcaagtcaatgca
gacttgggggtatggcaaatggattgcacacatctggaaggcaaggtcatcttggtggcagtacatgtagccagt
agtttcatatgggccaggataatttcccaagaaactggaaggctaacagccctagagctgtcaaacttggcagcc
acatggccaattagtcagatacacccagacaatggtaccaactttactagcaaggaattccaagctgttgcatgg
tgggctaacatacagcacaccacaggtgtcccctacaatccacagagtcaaggagtagtagaaaatgcaaacaaa
cagttaaaggaaaccattcacaaagtaagggaagaagtaacatatttagaaacagcagtagcacaagcagtcttc
attctaaataataaaagaaagggagggataggggatatgacccctacagaaaggttagttaatatgctatacaca
gaactagaaatacaacaactacaaaatcacaatacaaaattttcaaaatttcgggtttattacagaagaggagca
aacccttattggctgagaccagctcgacttctttggaaaggagaaggagctctagtgatcaaaacaaaggaaggt
gagatcgtaacagtcccaagaagaaaagcaaaaataatcaaggattgcactaaacattatatcttctcaggaaa
gctccatttacatatgtccaccactatcaattgcagcatcagagattcacccagaacaaaataaaaatacctttg
agcataaacagaatagaaggaggcccaacagagactacatacatagagataaccatattgttagatgtcacaaac
gtagggccagccagtttatcaaggtccacctattggcagcaatcctatatcctgaagtggagatacatcagggca
gccccacaaaatagagagatagacatggtgcattacgagaccttcctagacccagaagtgaccatgcagattata
cacacacactacttctcttgtttccaacagagagacatccaaagggcaatcagaggagaacagctcctaagaaag
tgtgagcacatcaagacacactacccgaaggtaggaactcctctatctctagaaaagctagcattctttgcatac

atcaaacatacagtcacagagctcccagcagatccacaatggtctgtagatcaggcagccataacatgtgccatt
gattacattaaatcagtccaaaccctcttgttcagacactatagggacggatgttaccatagctatgctcaaaca
atcaggagatacccaccgttgcgcccattgagagggacgcagcccccagactctaattccatgccaaatgctgac
cccacaccatctttgagaccctccagatatagaatggatgaaatggacccttcagttgaaggcctgcctccggac
cagcgtcccggagcggcacccccacaccttgcaccaactgttactgcaaatgttgcgcgtttcactgtatattg
tgcttccagaaaaaagcattaggtatctcactaatattgagaaaacatgccaagcaatgggtcacggtgttttat
agggtaccggtatcaaagaatgccacaccacctctcttctgtgcatcaaatgctaatatagcaaacagagagcca
gggaacgtgttgataacgacagcatgcctacccacggaccccgcgccacaggaggtacaactaaacatctcaaat
gagcactttaatatatataagaactatatggtagatcagatgactgatgatgtgaagtccatctttactcaggcc
cttaagccgtgtgtgaaactaacacctatgtgtgtaaaaatgaagtgtgaccttctcaatattagtatgtctagt
accaccgcagcaagcgccacaacaagcgggcatacaaccgtcaccccttggggcagatgggcagacaataatgag
acacaattgaccatgtacaattgttccttcaatcagaccaccgagttcagagatgtaaaaaaacagatgtactct
ctcttctttgtaaaagacctcatgaaaggggagaatgagacatattacattacaaactgcaacacttcttatatc
acacagcagtgtgagaagagcagcttccaaccggtcccaatacaatactgtgcccctgcaggatatagtctctta
aaatgcaatgatgctaatttcacaggacaaggcatttgtaataatgtaaccgctaggcactgcactcatgacata
ttaccgctaattgctacttggctacaattgaatggtacctacctaccaggcaatgacacagctgtaatgatgaat
ggtgataaaaatgaatctattgccataaaatttggagaaaactttagggtaaatctaacctgtattaggccagga
aataagacaattagaaatctacagataggagcaggcatgaccttctattctcagctgatagtggatggagacacc
cgcagggcatattgcaagatcaataaaactcaatgggatcctgctttgagacaggctatgatagctatgagagac
cattggcacaatagactccaaaaagctaataagacaataaacaaaacggaaataaaaataaggtggacctcagag
ccaaagggagatagggaggttcagacacactggtttaattgccaaggggaattcttctactgcaacttgtcagta
ttattccaatttgagaataacaccagagaaattaatgactcaaatatacatacagtaaaaaccaaatatcgccat
gaccaatggatggtctgcagaataaggcagtttgtaacccagtggggttacgtatcaaagtccatatacctgccc
cctcgaaaaggtcatgttcaatgtacatcccatgtaacagggctattaatagatggggccatgtacggcaataca
ataaacatgacccccctcagccaatgtctggacgcgtgcgtctagactgagagatacagtggtagagatagaccct
ctttccgtggcaccgacagaagtgcgcaggagacctgtgccccacgtcaaggctaggaatctgtgggggtgcgcc
aataggcaaatctgccatacctcggtgccatggaatgattcttgggcaaaccacacccaaccaggatgggacaac
atgacctggcaacagtggagcagattagtggacaatgacacagcagtcatccatggtctattggaaatggctcaa
caacaacaggaagaaaaccaacacaagttgcagaagcttttagaatgggattccctttgggaatggtttgacatt
tccaaatggctctggtatgtcaagatattttgcatggtagtagcaggcttaatagctttaagaatagttatgttt
atattaggaatgcttagaaggatttcctttagggaagccttagggcgcttcatcgcacacgcacaaaacaagggc
cgcaggcaaatcatagaagaacatgagtgctcctgcccagtacgcccgaaagtcccgattcgtgatgtcaactac
aaactcatgatagacctctctcactatttgaaaaaaaagagaggactagaaggaatgtttgtgtgcatagacaga
ctgcaaaaactagagacttatgcctatgtaaaatcaaaaatagtcccaagatcactaatatacacggatggccca
ggcacccgttacccacggcaaccagggttcctgtggtgcttgagaccggtagccatgacagaggagtcggagcca
ggagatgaccagtacctcctgacacacccggcctatgtgggtcgggatgaagaccaccacaaagagttcctggtc
ttctcctttgctctaggctggccctaaagtcaggcccgcagcttaatcaaatacaacaaaaagagcggaagaga
cgcttaaccgcaaaccgcatcctctag
>#4 EF070330 7335 nt
atgggagcgaggcactcggcgatgctgacaggtaccaagttggataggtacgaaaaggtgagactaagaccaaaa
ggaaagaagaggtacctaatcaagcacatagtatgggcttccaaggagcttgaaaggtttggactctcggactcc
cttttggagacaaaagagggatgtcaaaagatcttagaagttctcataccattagaaccaacagggtccgaatct
ctcaaatcactctttggaatcacgtcagtgctctggtgtatccatgcagaggtggaggtggaagacacagagcag
gctaggaagcaaatcaggataaggtgtcatctggcggacaagcaaggggaagaaaagacagcacagaatcaaact
agtgctaattaccccatcataaggaacccacagggacaatttcaacaccagtcccttaatgctagaatcttaaag
acatgggtgtcaatagtggaagaaaagaaatttgcagcagaagtagtggcaatgtttcaagcactagcagaagga
acaataccctatgacatgaatcagttgctaaatgccataggggatcatcagggggctatacagatcataaaagat
gtcataaatgagcaagcagcagaatgggatctgctacacccacagcctcagcagccgcagcccaatgcaggcctg
cggagcccatcaggatcagacatagcaggggtaacatccacccccaatgaacaaatagaatggataactaggcaa
aacgatcctataaatgtggcagacatctataagaaatggataatcatggggttacagaggtgtgtcaaaatgtat
aaccctgttagtatattggatatcaagcagggaccaaaagagccatttaaggattatgtggacaggttctacaaa
tgcttaagggcggagcaatcagatcaagcagttaagaattggatgacctccactttgttagtgcagaatgctaac
ccagagtgtaagctcatcctgaaaagcatgccaggagccaccttagaagaaatgttgcaggcatgccaaggggta
ggagggccagcacataagtccaggctaatggcagaggctatggcctcggcccttagacaaaacagtcagctgaac
atggtgcaaggagccagagggaaaggcagccagggaggtcccaggggaaaccctaggtgttacaactgtgggcag

tttggacacatggcaagaagctgccccaagccaaaaactaggaaatgcttcaaatgtggaagggagggacacttg
gcaaaacagtgcaggtctgaaggtgccaaatcggctatagaaggacaagtggtgcacatgctgctcgacacaggg
gcagatgacaccatagtccaggattccaacattcagatagataaaccatggaatcccaaattggtgggggaatc
gggggaaacatatcagtcagagaatatagaggggtacaagtatctttcaatgagaaaacaattaaggcaacagtg
ttggtgggacctacacccatcaacataatgggagaaattgtttaagtaaatttggaatcaccttgaacatgata
caggaaaaaatagaacccataaaagtggccttaaaggaaggggcaaaaggaccaatggtcaaacagtggccactg
actcaagaaaagattaaggccttagaaggcatagttcagcaaatgttaaaattagatcagatagaagagataggg
ccagacaatccttacaactctccatgttttgcaatcaggaagaaagataaatctaaatggagaatgttaatagat
tttagacagctaaatgaggccacccaagagttcacagaggtacagctagggatcccacatccagcaggattagcg
gaacatgagcatgtcacaatagtagatatcaaggacgccttttacagcgtcccattagatccagcattcagaaaa
tacacagccttctcgctgccccaggtaaataaccagggaccagcaagaaggtatcagttcaaagtgctgccacag
gggtggaaaggatcccccaccatcttccaatacacggcagcaaaattattacaggaaatcagggaagcaaatcca
gatatcaccctcatccagtacatggatgatctcttaataggatccaacagagagatatcgggacataggagagta
gtagcacagattaggaacatgctcttgaaactggggatacaaaccccggaagataagtaccagaaagactaccca
gtccagtggctagggtatgagttacaccccagagggtggaaaatacagccagtaacactcccacagcaagaaaac
tggacagtaaatgatatacagaagttggtaggcaaattgaattgggcagcgcaagcctacccaggagttaaaacc
aaacagctatgcaaatgtataaggggagttaaatccctgacagaagaagttcaactaacagaggaagctcaatta
gaattggcagaaaaccaggaaattcttaagcaggcagtccagggggcatactttgatccagaacaacccctggtg
gctgaaattgtctcgttaggagactcacaatgggggtataacttcctccaaaataagggaatattaaaatctgga
aaatttgccaaagtcagatcagtccataccaacagttatcagcagttatctgatgccatagccagaataggaagg
gaatcattagtcatttgggggaagcccccgaaaaggtcaggattccagtgatcaaggagcagtgggatcaatgg
tggacagaacattggcaggtatcatggatacctgacatagaggcagtacataccacccacctgctgcgacagtgg
ttcacattggtgccagagccattgtcagaagctcccacatattatgtagatggagcagcacataaagtatctaag
ttagggaaggcaggatatgttactaacacaggaaaagaaaaggtggttagcttagaaaatactaccaatcaaaag
gcagaactagaagctgtcttgttggccttgaaagaaggccctcccagcatgaatatagtcacagattcccaatat
gtgctggggatagtggcttctcaaccccaagaatccacctcgcccttggtagaagaaataattcaacaattactg
accaaggaggcggtatacctatcgtgggtgccagcccataaaggtataggaggaaatgaggatgtggacaaactg
gtaagccatggaattaggcaggtgctattcatggagcaaatagagcctgcaaaagaggaccatgaaaagtaccat
agcaattggaagtatctcagggataagtacaacatcccagctttactggctaaggaaatagtaaacctttgtccc
aaatgtcagacacatggggagccaaaaacaggacaggtcaatgcagaattgggagtgtggcaaatggattgtact
cacttagaaggaaaaatcatcttagtagccgtccacgtggccagtggatacacgtggcaaaaatattacccagg
gaaacaggaaggcaaacagccctaggcctgttagaattagcagctctttggcctgtaacacaaatacacacagat
aatggagccaacttcattagtgaagaattcggagctgcatgttggtgggctagcatagatcacaccacaggggta
ccatacaaccctcaaagtcagggagtagtagaaaacaaaaacaaacagttaaaagaaactatacagaaaatcaga
gaagaagtcacctatttagaaacagcagtagcacaggcagtcttcatcatgaattttaaaaagaagggaggaata
ggggacctagcagctgctgagagaataattaatatgttacatacagaactagaactacaacacttacaatcacaa
aaatccaaatttcaaaattttcgggtttattataggacaggctctgacccatcttggaagggacctgctgccttg
ttgtggaagggtgaaggagcagtagtcgtcaaaacagaacaaggacaggtaatcactgtacctagaagaaaggca
aaaataatcaagccagctacaaaacatcatatcttttcaggaaaaacaccatttgtgtatgtacaccactaccag
gtacaacaccaaagattttcccaaaacaagatcaagctagcattgacacaaaacaccttagaatcaggagagaca
gaagtcaccttcatagagataacagtcctatgggatgtgactaatgtaggaccagcaagcctatccaaaagtacc
tattggaagcagtcatacatcctggaatgggtatacatgaggaaaggtccaagagatagagaacgggactatgtt
tggtatcacacatacctcacacctgagatagccatgaagatcatacacacccaccacttctcttgtttctatagc
caggatatcccgagggtcatcagaggacaaccacccttaggggattgtgaacacccaggagcacatacaaaggta
ggacccccctcccactctacaggtattagcactattaacattaaagaaagtaacggagctaccagccgatcctaat
tggacagtggatcaggctgcaatagcatgtgccatagattacattaggagaacacaaacactccttttcaggcat
tatcgagaggatgttatcataggtactctaacacaatcaggagataccccaacataagacccttgagaggaca
caagctcctccttctaattcaatgcctaatgctgatcctactcctcctttgagaccctctagatataggatggat
gaggatccttcagtagaaaaccttcccccggaacagcgccccggcgcagcgccgaccacgccttgcagctcctgc
tattgtaaacggtgtgcttttcattgcatgttgtgcttccaaaagaaagctttaggtatctccataatatggggc
aatcagtgggtcacagtgttctatggggtaccggtatggaaagatgcaactccaccactcttctgtgcatcagat
gctaacatagcaaacagggaaccagggaatgtgtggatcaccacagcatgtcttcccacagatcccgcaccccag
gaagtacaattaaatatctctggggaatattttaatgtctataaaaattacatggtgacccagatggttgatgat
gtaaggtccatattcaaccaggccctaaaccctgtgtcaaattgacccctatgtgtgtcaaaatgaaatgcagt
agaataaatgtaacaaggccatcaacaacaacaacagcatcaacaccaacaaaaacaacaccagtgaccccttgg

gggaaatgggacgccaatggtacagagcaaagcatgttcaattgttcattcaatcagactacagaattcagagac
aaaaagaagcagatgtactccttattttacactgatgatctgatgcgtgaggataatgatacttattacatatta
aactgtaacacctcatacataactcagcaatgtgaaaagagcagcttccaaccggttccgatacaatactgtgca
cctgcagggtattctctattgaaatgtaatgatgctaattttacaggacaaggaaaatgtaacaatgtgacagct
acgcattgcacgcatggcatattacccttggttgctacctggttacagcttaatggcacatatttagaaggaaat
aatacagcagtaatgatgaatggggacaaaaatgaatctataggaataaagtttggggaaaactttagagttaac
ttaacctgcttaaggccaggcaataagactataaggaacctccagataggggcaggcatgactttctattcacag
cttatagtaggaggggacacacgaagagcatactgcaagataaacaaaacacagtgggatgtagcattaaggcaa
gctatgttagcaatgagaaagcattggcatggccgcttggagaaacaaggagaaaatattacagaagaaaagata
aaaatcaggtggacctcccagcccaaaggggataatgaggtccaaacacattggtttaattgtgcaggggaattc
ttttactgcaatctatcagtgctgtttcaatttaacaacaatatcacacaaattaacgagagcaatatacataat
gtgacaaccaaataccaccaagatcaatggatggtctgtaggattagacagtttgtcacgcaatggggctatgtg
tcaaaatccatctacttgcctccacgacggggacatgtgcagtgtacttctaatgtgactgggctccttattgat
ggagctatgtataaagacacagtcaacatgactccatccgcgaatgtgatggacgcctggcgaatggaactaaaa
aattacaaggtagtggaaatagacccgctttccatggctcccacagaggtgcagaggaggcctgttccccatgta
aaagcaagaaacctttggggatgtgcgaacaggcagatctgtcacactagggtgccatggaatacatcatgggcc
aatcataccttaccaggctgggaaaacatgacctggcagcagtggagcaagttggtggataatgatactaatacc
atacagggcctcctagaagcagcacaaaagcaacaggaagaaaaccaacacaaattgcaaaaattattagaatgg
gactctttatgggaatggtttgacatctccaagtggctttggtatattaagatcttttgtatggtagtagcaggt
ttagtgcttttagaatattcatgtttgtgctaggcatacttagggaacaagcccgccgctgcagtgaactctta
gcgcgcctcttagcatacattcaaggaagcaatgagcggccactcatcgaagaacatgagcgttcatgcccagtg
cggccgcgagtgcccctacgggatcccacatacaagatgatgattgacctctctcattatttaaaagaaaaggga
ggactggaagggatgttttattgtgaggaaagacaccaaaaattggagacttatacctatgtggactggggaatt
gtaccaggatggctacagtttacagagggcccggggacccggtacccaacactgccagggttcctgtggtgccta
agggaagtcgccataactgaggactctgaggaggggatgaggaatatctcctcacccatgcagcctaccaaggc
aaggaggaggacccccataagcagttcttggtcttctcattctgctctcgcctcgccatgaagtcaggcaggcaa
ttagatcaaatacagcaagaagaacggaagaggcgcttaaccgcaaaccgcatcctatag
>#5 AY340701 7329 nt
atgggggcgaggcactcggcgatgcttagtggtactaaattagacaagtatgagaaggtgcggcttagaccgcgc
ggtaagaagaagtacctgattaagcatatagtatgggctgccaaggagcttgatagattcggtctctcggactcg
ctcctggagacacaggacggctgtaagaagattttggaagttattcttcctctacagccgacaggatccgagtct
atcaaatctctctttggtatcgcttcggtattgtattgcatacatgccggtatagaaattgaggacacagaacag
gctaagcagcaagtaaaaatcaggtgtcacttagcaggggaacagggagaacagaaagctgcagccgcggccgct
ccaccaacaggcggcgtacccagtggaaactaccctgtcgtgagaacacagggggaggcttccaacatcaggca
gtggagcctcggctcctcaagacatgggtacaggtcattgaggagaagaaatttgccccagaagtggtggccctc
ttccaggctctatcagaaggcatgattccctatgacatcaatcagctactaaacgccataggtgatcatcagggg
gcactccaaattattaaggatgtcatcaatgaggaagcggccaattgggacttagtgcacccacagccgccacaa
ccgcaacccaatgcagggctgggagacccaacgggggccgatatagcaggagtctcgtccacaccccaacagcag
atagagtggataaccagggctaacaaccccgttcaggtctcagacatctacagaaaatgggtcatcttgggcctg
cagcgatgcgtcaaaatgtacaaccctgtcaacattttggatatcaaacagggacctaaagaaccattcaaagat
tatgtagacagattctacaaatgcctcagagcagaacaaacagatcaggcagtcaaaaattggatgacgcagacg
ctgctggtccaaaatgccaacccagattgcaaactcatcctaaaggccatgccaggtgctagcttggaggacatg
ctacaggcatgtcagggagtgggaggtccaatgcacaaatctaggattctggcagaagctatggcaggggcaata
gccaacatgcccatgaatatggtacaggccagaggaccaccccaaaggaagggacaacccaaatgcttcaattgt
ggaaaatttgggcacatggcgaaaaattgcaaagcccccaaaggaggaaatgttacaattgtggccaaccggga
cacctcgccaaagactgcccgcagccccccaaacaaaacaaaggggtgtctgtgctgataggggacaaaaggtg
tccatgctattggatacaggtgccgacgacacaattatacaagatcagcacgtaaatttaggggagaattggaca
cctaaagtagtaggggggaatagggggaatgatagaagttaaacaatacaaagcaatacaagtgatatttgaagac
agagaggtttgggcaacagtattagttggaccaactcctataaatattttagggagaaatgtcttagctaagatg
ggggtaaccctcaacatggctgctgggggagacctccaaccccccatctttagaggtcactttaaaagcaggaaaa
gagggaccaaagttgaaacagtggccattatccagagaaaagatagaggctcttacacaaattactcaagagatg
cttaaattaggacaattggaaccaaccgagcccaataacccatataactcaccctgttttgccataaagaaaaag
gacaagagcaaatggagaatgctcatagatttcagagaacttaacaaggcaactcaggacttctttgaggtgcag
ttgggaatcccacacccagcgggacttgagaagatggaccatgtgacaatagtagacatgaaggacgccttctac
agcattcccttgtgggaaccgttcagaaaatacacggcgttctcaataccctctttaaacaatgctgaacctgct

aaaaggtatcagttcaaggtcctcccgcagggctggaaagggtccccaactgtgtttcaacatacagcggcagag
ctaccacaggagatcaggaaaaaatacccagaggtaactttaatacagtacatggatgacctactgataggatca
aatcactctctgccagaacacaggaaaatagtaggggtcataagagcgaccctactaaggaaagggattcaaaca
cccccagagaaatttcaggatgactacccggtgcaatggttagggtatgagctacacccatcccattggaaaatt
gccaaaatagagctaccagaccaggaacaatggactgtcaatgagatccaaaagttgataggaaagcttaattgg
gcagcacaaatctattcaggaataaaaaccaaaaacctgtgcaaaagtatcagaggggcacggagcctgacagat
acagtggtcctctcagatttagctcaagctgagctagcggaaaatagggagattctcaaggaggaatccaccggg
ggatattacgaccccaacgagccattaaaggtggagttaacctcactagcagaaaaccaatggggctacagattc
ttccaagaaaaatcagtattgaagacaggaaaatttgccaaaattagaagtacccactccaatacctatcagcag
ttagcagatgcgctagccaaattgggaaaagaggccatagtcacatggggacgacttcccatttttcgcctacca
gtggtgaaagagcaatgggacgcatggtgggcagataattggcaaatcaattgggtaccagacatagaggcggtg
tattccccacacctgctcagacagtggtatactctggtacaagaccccatagaaggggctccaacatactacgtg
gatggggccgccaataggacatcaaaggaagggaaagcaggttatgtaaccaactttggggcaagaaaggccgtc
acattagaacagaccaccaaccaaaaggcagagctagaggccgtgttactagccctcagagatggaccacctcag
atgaacataattacagattctcagtatgtactgggcattctagcctcctgcccggagatctcagaaagtcccata
gtggaggccatcatacaggaactcttaaaaaaggaccaggtattcttgtcatgggtaccagctcacaaaggaata
ggaggaaatgaagaagtagatcgcctagtgggccagggaattaggcaagtcctgtttatggaaaatttggaaccc
gcaagggaagatcatgaaaagtatcatagtaattggaaatacttaagagatacctaccacattcctactctgttg
gccaaagaaatagtcaaccattgtcacaaatgtcaaaaccatggagaacccaaatcaggacaggtaaacgcagaa
gtaggagtatggcagatggactgcacccatctagagggaagcataattttggtagcagtacatgtagccagtggt
tatgtatgggccaagatcctaccaagggaaacaggaaaatgtacagggatagccctgttggagctggccgctatg
tggccggtcactcagatacatacagataatgggccaaatttcaccagccaagaattcgaagcagctgcatggtgg
gcaaatattcatcacaccacaggggtaccctacaacccacaaagccaaggggttgtagagaatatgaatagacaa
ctaaaagagaccattaagaaaatcagggatgaggttgaatacctcccaacagcattagcacaagcactgttcatc
ttaaattttaaaagaaagggaggaataggggatatggcccctgtagacagatttataaacatgatacacacagaa
ttagaactacaaacctcaaacaaccaaattacaaaattttcaaaatttcgggtttatttcaggacaggtgccgac
cctcagtggaaaggaccagcgcgcctactctggaaaggtgaaggtgccgtagtcataaaaactgaggagggcgat
atcctgacagtccctaggagaaaggcaaagataatcaaaccctgcactaaatggcacatactcacaggaagtgca
ccctttgtctatgtacatcactaccagctgcacaatcagagattctcacagaacaaaattaaactccccttagac
ctaggaatcactcaagaaggagagtcatgggcaacatatttggagataacaatttattgggacttaacaaacgtg
ggacctagtgcactctcaccaacagtgtacaacaaacaggcctacacaatagaatgggtttattggatgcaaaag
gtggaacgggtccctttagatcccgtgtggaatccaaacccaagagatagagaggtcagaacatggtatgctacc
catctgacgccggacctggcgaatcagatagtacatacacattattttgcatgcttccaacagttggacgtgcgc
agagcaatcagaggagagaagctcttgggacagtgtcaacatctagcaacacactacccgaaggtagggaccccg
ttgaccttagagaaattagccttccttgccctagtgcgaccggcctggaatgcagaccaggcatgggcagcatgt
gccattgattatactagatgggtccaaaccatcctttataggcactacagggaaggctgttaccatagatatgca
gagcaaataagaagataccctgttcttaggccaatgagagggactgctcccggcccaaccagttctgttccacag
gccgatccagacaatcccagaagaccttccagatatagaatggatgaacctgttgacccagacttacccaaagaa
caacacccccagcaaccccacgcacaccttgtactaactgcttttgtagagtttgttgctttcactgtcagatt
tgcttcctcacaaaaggcctcgggatttcatggaccaccgtctactacggtgttccagtatggaaaccggcaact
cctcctctcttttgtgcctctgatccaaattacgggtctaaagaggcagggaacaattggctggcgtcttcctgc
ctcccgacagaccctaccccgcaatctctatatttgaacatcactgaggagttcaatgcttatcaaaactacatg
gtggaagaaatggtagaggatatgaagtctctgttctcgcaggctttaaagccttgtgtgaaattaacacccatg
tgtgttaggatgctctgtgttgaagttaacaccgtttcgaatgccagtaccactcctgcccctagtacccccaca
ccctggggaaattggggagggaatggaacaggacagccagtatataattgctcatttaaccagactacagaattc
agggataaaaagaagcaaatgtatagcttattttggaaggaagacataatgaaggaggaaggaagcaatggcagt
cactattacatcctgaattgcaacacatcatacataacccaagcttgtgagaagtccaattatgagccagttcct
ttgcattattgtgcacccccaggctatgctttattgagatgtgatgaccccgcctttacgggacaagggagttgt
tctaatgtttctgcagtaacatgtactcatgctatccaacctatagtagctacttggttccagttgaacagtacg
ggtaatgctcccaatacaacagtaatgatgaataagcagaaaaatgagtctatagtagttagattggctaagcac
ctacatgtcaacattacttgcattaggccaggaaacaaaactattaggaatttacagataggagcaggcatgaca
ttctattcgcaacttatagtgggaggtaatactcgtaaagcctactgcaaagttaataagacccaatgggagact
gccctacaggcagttcacgaggcagtaaaaactgagtgggaaaagaaaaacaatggaaccaatgtgaccacaata
tcttggagatttcaaccccaaggggacaaggaggtccagactcactggtttaactgccagggagaattcttttat
tgtaatgtctcagctttattcattaatagaagaaccaacaaaacggacgggatatctccattcgacgtgaataac

aagcccaacaccacgtatcatggtgggtggctagcatgtaccataagacaaatggtgacacaatggggatatgtg
tcaaagagtatctacctacctccacggaaaggccatgtgcagtgtacatctaatatcactgccctcctgattaca
ggggagttgtatcaaaacaacgtgacccttgttccctcggcacaggtgagtgactcatggagatcagagctcagt
cgatataaggtggtggaaattgaccccttatctatggcacctacaacagcgcagagaaggactggggtacacagg
gaaaagagaaatgcatggggatgtgccaataaagccgtgtgtcacacaacagtgccgtggaacaattcttgggca
aaaggtcacttccctgagtgggacaatatgacatggcaacagtggagtgagttagtagataatgacacaatgacc
attcagcagctcttggaagctgcgcaagagcagcaaggcaaaaaccaacatgagttaatgaagccgggacaatgg
gacttcctgtggaattggtttgacatctccaaatggttgtggtacatcaaaatattcattatagtagtagcagct
ttgataggcttaagaatacttatgttcatactaggagttatcagtaggttacgcttgctagcctactgccagggc
agaaatggcaaacaacgccggcaacctctggacgacgacgacgacgacgacggtgtcggttgtccagtaaggccc
cgagttccgcttcgcgaccctacttggaagctcatgatggacctctctcattatttgaaagaaaaggaggactg
ggagagatgttttactgtgaagacagacatagaaagatagagcaatatgcctatctggaatggggcttaatccca
ggatggctacaatatacggaagggccaggggtcaggtacccgactatgcccgggttcctgtggtgcttgcgcccg
gtagccaccacggaggattccgaggagggagatgaggacttcctcctaacccatccggcctatcaaggccggatg
gaggacccccatagacagttcttggtcttctcattctgctccaagctggcagtcaagagcggcagacagctagca
cagcttcagcaagaggagcggaagaagcgcttagccgcaaaccgcatcctctag
>#6 KJ461716 7461 nt
atgggggcgaggcactcagcgatgcttactgggaaaaagcttgataagtatgagaagatcaggctaaggccgaaa
ggcaaaaagaagtaccagataaagcatatagtttgggcatccaaagagttggaacgatttggactatcagactcc
ttattggaaaataaggagggctgccaaaagatcttagaggtcattgttccattggaagaaacagggtccgagtct
ctaaaatccctctttggattagtttcagttttgtggtgtgtacatgcagatgttacagtagaagacacagaggaa
gctaaaaaacagatcagaattaggtgccacatgagtggcaagaaggaggagacctccgcgggcggaggtgcctca
ggacaaaactatccaattgttaggaatgcacagggacagcatcagcatcaggctttgtccccaagaatcctcaag
acatggataacaaccattgaggagaaaaaattttcagcagaagtagttgccctattccaggcactgtcagaaggc
tgtataccctatgatataaatcagctcatgaatgccataggggaacatcaaggagcggtacagcttatcaaagat
gtagttaatgaggaagcagccgcttgggatcagttgcacccacagccagcacagccgcagcccaacgcaggcttg
agatacccatcagggagtgacattgcaggggtatcatcatccatacaagaacagctagaatggatgacaagagca
gtagacccagtaaatgtaggagaaatatacagaaaatggatagtaatgggtctgcagagatgtgtcaaaatgtac
aacccagttaacattctggacataagacaagggccaaaagagagcttcaaagactatgtagaccgattctacaaa
tgtctgagagcagaacaggctgatcaatcagtcaaaaattggatgactacaaccctcttggtccagaatgctaac
ccagaatgcaaggccatcttaaaggccatgccaggagccagcttagaagaaatgcttacagcatgccaaggcgta
ggagggccacactataagaccaagctgttagcagaagccatggcatcagctttccaaaagacaggaaattacaac
atggtgcagggcccacgaggcccgaaaggggctcgcaggggggccaatgaaaaaccctaatcctaggtgtttcaat
tgtggacagttcggacacatgtccaaacagtgcccacacccgaaaaagataaagtgctttaagtgtggcagagag
ggccatatggcaagacagtgtcgagcctcagaaggtgcacagggaggagcatcctgcttagtggagaggcaagaa
gttgacctactattggacacgggtgcagatgatacaataatccatgatcaggacatagaactaggagataactgg
cacccaaaaattataggggagtaggtggcaatattagagtaaaagcatatcatagtgttgaaatagaatggcag
ggaaaaagagtaacagaggaagtattggtgggagacactcccatcaatataatgggagaaatttcttaaccaaa
tttggggtgactttaaacatggtagtaggcaaatttaagccgacagaagtacggctaagagaagggaaagatgga
cccaaagtcaaacaatggccattaagtgcagagaaaatacaggcactaaaagagatagtacaagatatggtagat
aaaggacaattggaaaaaatagggccagaaaacccatacaattctccagtattttgtattaggaagaaggacaaa
acaaaatggaggatgctaattgatttcaggaaactaaatgagcacacacaggacttagcagaagtacagttgggg
atcccacacccagcaggtttacccaagaaggcacaggtttccatagtagacatcaaagatgcctactatgcaatc
ccgctacatgaggactttcaaaaatacacagcattcaccataccttcagtgaacaacatgggaccaggagaaaga
tatcagtttaaggtactgccacaggggtggaaggcatctccaactatatttcagaacacaataggagacctgttg
caacagatcaggaaaaaatacccacaaatccttatgattcagtacatggatgacctcctcatagggtcagatgaa
acagtaacagaacacaggaaaatagtagagcaaattaggaacatccttctaaaacaagggttacagacccctgag
gaaaaataccagccacaaaggccagttaattggttagggtatgagcttagacctagagagtggacaattcctaga
ataacattgccagttaagcaggtctatacagtcaatgagatccaaaagttagtaggacaattaaattgggccagc
caaatatatccaggataaaaacaaaggctctgtgtaaattaattagaggaacaaaaggcttaacagaagaagta
caggtcacagaagaggcagaaacagagcttgcagaaaaccaagccattttagaccaagaagctaaaggggatac
tatgatgcagagaagccattagaagtagatatcatacagttgggaggaacccagtgggggtacacagtaagacaa
gacaaagaagtactaaagacaggtaaatttgcaaaacaaagaagtgcacatagtaacccattcaaacaattggta
gatgccattatgaaaataggaaaagaaagcttagtatactggggagaataccacaattcaatgtcccagttaac
aaagagcagtgggacgcatggtggaatgaacattggcaggtgtcctggattccggatataaaaccagtgcacact

ccacctttgatacaaatgtggtaccaattagtacaggatccaatttcagaagcagaaacttggtatattgatgga
gcagcaaatagggaatctaaattagggaaagcaggatatgtgacagatagaggaaaagagaaagtagtagcatta
gaacataccaccaatcaaaaagcagagttacaagcattacttctagctctacaagatggggaacaaaacagaac
atagttacagactcccaatatgttctaggaatcataacaggagctcccacagaaacagaccatccaatactagag
caaataataacacagttacaaagcaaagaagcaatatacctctcatgggtaccagcccataaaggcataggggc
aatgaagcagtagacaaactagtaagcaaaggaatcagaagggtattgttcctggaacaaataccacaagcccag
gaagaacatgaaaggtatcataacaattggcaagaccttagagacaggttccagataccagcattaatagcaaaa
gagatcttaaaagcatgccccaagtgtcaaggaaaaggagagccaatgcatgggcaggtgaacatggaagtggga
ctctggcagatggattgtacacacttggaagggaaaatcataatagtagcagtacatgtagccagtggatacaca
gaagccaagctaatcccacaagaaacaggaaaagagacggctatattcctgttacaactatgtgctagatggcca
gtcacacagatacacacagataatggaccaaatttcaccagccaggaattagcagcagcagcatggtgggctaat
gtacaacacagcacaggtgtgccttacaatccacagagtcagggagtagtagaaaacaagaacaagcaattaaag
gaaacaataagcaaaatcagagatgaagtacaatacttagagacagcagtagcaatggcactcctcattcttaat
tttaagaaaaggggaggaataggggggatgacaccagcagaaaggctaataaacatgatacacacagacttagaa
ctacaacaaaccaaatttccaaaattttcgaattttcgggtttattacaggacagggaagagccctgagtggcta
gggccagcacaactcctgtggaaaggagaaggtgcagtggtaatcaagacaactacaggtgagctgttgacagtc
ccaaaaaggaaagcaaagatcattaagcccctaactaagtatctcaagtataaaacaaaggaattagaggaagta
caatggatacatcattaccaattatatgcacataggtatacacaaaacaaagcaatcatcccacttaccttgaaa
gtatcacaggattatgtggaagcaacagccatagaagtacaaatattgtgggatctgtcaagttgtagaagtagg
gatccacctggaacaagagacacacaagcccttactattagttggacttacaccaggttgactaagggagacaga
gagatagatagaatcagctatgagacagagattactccgcacttagctgatgcaatgatacatgtggagcatttc
tcttgttttacagctcaggatttcactagagcaatcagaggacagcaattgctaggtgagtgccatcatccacat
tatttaggcaaagagatacccacattgcaatacctagcactaagggcaatacaatggacagaacagcaggcaatg
gcagctactgcaatagattacataaggatcgttcaaaagttattgtgggatcattataagaaaggatgctatcac
agataccatgctagagtaaggaggtttccacaactgagaccactgagaggtacagcaggacaggacacgccccat
gacagtgatccgccacaggtgctaagaccctctcgttatcatccagataacgatccatctcaagaggggttagaa
aaatggcatagggcgcctgcacaacctccaactgcttgcactccatgcttttgcaaaaactgcatatatcactgc
atattgtgcttccaacgaaaaggacttggcatccgcatgacaaagtataagcttatagtattaggtatagcaatt
ataataggtttaataacaatagatttgcaacaggggatgaaaatgatggatgggtaacagttttctatggggta
ccagtgtggcataatgccacccctcctttgttttgcacagcagatgcaagacatacatgggtcactactaattgc
ttgccaacagatccagcccctatagagacacctatgaatatctcaggagaatggttcaatgtacacaccaattat
atggtggaccatatggacaaagatatggcagcattgtttctacaggctacaaagccatgtaccaaattgacccct
atgtgtgttaaaatgaagtgtcagaattatactaaagaagctacaaccacaacaacaccaacatcatcaacaaca
tcaaaagcaccaccaacaacaacccaaaggcctgcaacagaatggtggggaggaaaagatcctcaatctttactc
aattgcacttttaacatgaccccaggttttaaagataggaaagctcattattgggctcctttttatacattggat
ttatggcaacaatctaatggtactaatggaacaggggaatattatgtaaagtattgcaacacgtctgctatgaca
caagcctgtgataaatttcattttcagcctttcccagttcattactgtccaccagcagggtatgcattgttcaaa
tgcaatgacataccatggaatggacaagggccatgtaaaaatgtgacagcagtacattgtacacatgcaattaat
acattggctagtacatggctccagttaaatggcacatatgaaaagaatacagatgaggtgcaggttatgagaaag
tataattccaattacagtgtagcttttatcttctcagagagccaaatagtaaatttaacatgtgtaagaccaggg
aataaatcaataaggaacttgcaaataggagcaggcatgacctttactcacaactcatagtaggggaaatact
agaagagcttattgcaaaataaatggcacacagtggtacaatgctttaaatgctacctatagggccataaagaag
gagtataatcttacacggatcaaccaatcacctggagatctcaaccaagggggatagagaagtggagagtcac
tggttccagtgtcaaggggagttcttttactgtaatttatctaggatctttcagaaaaccaatttcacaaatgat
accttctatccacgaaacataacaaaggaaggggcagagttgagcaacatatggttcacatgtaccatcaggcaa
gtagtgaacagatggtcacatgtagaaaagttgatgtacttaccaccaagaaggggacatgttcagtgtacatct
aatgtcacaggaatcttggtggaatctgaatactatccaggaagtccatttaacatgaccccatcagccaatatc
aaggacttgtggaaaatagatttaagaagatataaggtggtggaaatagatccaattggcctagcacccactcca
ataaaaagatatgaaccaccaccaagcaaaagcgttgtcaaaagagcttcatatgggtgccaattcaaacaaatt
tgttatacctcagttccttggaataaatcctttgctgatggagcacttcctgcttggcacaacatgacctggata
gagtgggaaagaaaagtgactaatcatacaggtatcatcaatactttgttggtagaggcccaaaggaggcaagaa
gaaaacacacataagttacagaagttaggagaatgggataatctttggaattggtttgacatctccaagtggttt
cagtggattaaaatagcagtgttgatagtaataggtttaatagcattaagaatagtaatgtggcttattaatata
ttgtggtggtttggacgtgcttatcctcgcttgatactagcagcagaagcaacagcagaagcaaccagaagagca
gtagcttacattcacgaagaaacagccgagacagccaagagaggggaagttgtacctgtaagatacaacgaaggt

gacagcacccgagatgatgatgaagaaggagtgggtttcccagtatgcccacaagtaccccctcagacaaatgaca
ttcaagacggctgtggatttctcctggtttttaaaagaaaagggaggactggaagggttgttttattcccctgag
agacacaagaaattggacctatatgcttataatgtatggggactagttccagggtggcaaggatacacccagggg
ccaggcacaaggttcccaacatgctttggcatcctctgggaattggtcccggtggagatagggccacagggcctg
ggagaaggagatgaaagagcatcgttgctccatgcggggcaacaagtctaccaggacccccattgtgagaccctg
gtatggcacttcaatcccacccttgcctttgagccaggcatactgaaggctgacaagttgggacagaagcctttg
cctgggcaaaagacctttctaacaggaagagactag
>#7 KJ461714 7554 nt
atgggggcgaggcactcagcgatgcttactgggaaaaagcttgataagtatgagaaaatcaggctaaggccgaaa
ggcaagaaaaggtaccagataaagcatatagtttgggcatccaaagagctggaacgatttggactatcagactcc
ttattggaaaataaggagggctgccaaaagatcttagaggtcattgttccattggaagacacagggtccgagtct
ctaaaatccctctttggattagtttcggttttgtggtgtgtacatgcagatgttacagtagaggacacagaggaa
gctaaaaaacagatcagaattaggtgccatatgagtggcaagaaggaggaaacctccgcgggcggaggtgcctca
gggcaaaattatccaattgttaggaatgcacagggacagcatcagcatcaggctttgtccccaagaatcctcaag
acatggataacaaccattgaggagaaaaaattttcagcagaagtagttgctttgtttcaggcactatcagaaggc
tgtataccctatgatataaatcagctcatgaatgccataggggaacatcaaggagcagtacagcttatcaaagat
gtagttaatgaggaagcagccaattgggaccagctgcacccacagccagtacagccgcagcccaacgcaggctta
agatacccatcagggagtgacatagcaggggtatcatcatccatacaggaacagctagaatggatgaccagaaca
gtagaccaagtaaatgtgggagaaatatacagaaaatggatagtaatgggtctgcagagatgtgtcaaaatgtac
aatccagttaacattctggacataagacaagggccaaaagaaagcttcaaagactatgtagacagattctacaaa
tgtctgagagcagaacaggctgatcagtcagtcaaaaattggatgactacaaccctcttggtccagaatgctaac
ccagaatgcaaggccattctaaaggccatgccaggagccagcttagaagaaatgcttacagcatgccaaggcgta
ggagggccacactataagaccaagctgttagcagaagccatggcatcagctttccaaaagacagggaattacaac
atggtgcaaggcccacgaggcccgaaaggggctcgcaggggaccaatgaaaaaccctaaccctaggtgtttcaat
tgtgggcagttcggacacatgtccagacagtgcccacagccaagaaagataaagtgctttaagtgtggcagagag
ggccatatggcaagacagtgtcgagcctctgaaggtgcacagggaggagcatcctgtttagtagagggacaagaa
gttgacctactattggacacgggtgcagatgatacaataattcatgatcaggacatagacttaggagataactgg
cacccaaaaattatagggggagtaggtggcaatattagggtaaaagcttatcatagtgttagcatagaatggcag
ggaaaaagagtaacagaggaagtattggtgggagacactcccatcaatataatgggggagaaatttttaaccaaa
tttgggggtaactctaaatatggtagtaggcaaatttaagccaacagaggtacggttaaaggaagggaaagatgga
cccaaagtcaaacaatggccattaagtgcagaaaaaatacaggcactaaaagagatagtacaagatatggtagac
aaaggacaattggaaaaagtagggccagaaaacccatacaattctccagtattctgtattaggaagaaggacaaa
acaaaatggaggatgctaattgatttcaggaaactaaatgagcacacacaggacttagcagaagtacagttgggg
atcccacacccagcaggtttacccaagaaagcacaggtttccatagtagacatcaaggatgcctactatgcagtc
ccgctacatgaggactttcaaagatacacagcattcacaataccctcagtgaacaacatgggaccaggagaaaga
tatcagtttaaggtactgccacaggggtggaaggcatctccaaccatatttcagaacacaataggagacctgtta
caacagatcaggaaaaaatacccacaagtcctcatgattcagtacatggatgacctcctcatagggtcagacgag
acaataacagagcacaggaaaatagtagagcaaattaggaacatccttctaaaacaagggctacagacccctgag
gaaaaataccagccacaaaggccagttaattggttagggtatgagcttaggcctagagagtggacaattcctaaa
ataacattgccagttaagcaggcctatacagtcaatgagatccaaaagctagtaggacaattaaattgggccagt
caaatatacccagggataaagacaaaggctctgtgtaaattaattaggggaacaaaaggtctaacagaagaagta
caggtcacagaagaggcagaaacagagcttgcagaaaatcaagccattttagaccaagaagctaaaggggggatac
tatgatgcagagaagccattagaagtagatatcatacaattaggggggaacccagtggggggtatacagtaagacaa
gacaaagaagtactaaagacaggtaaatttgcaaaacaaagaagtgcacatagtaacccattcaaacaattggca
gatgctattatgaagataggaaaagagagcttagtatattgggggagaataccacaatttaatgtcccagttaac
aaagagcagtgggacgcgtggtggagtgaacattggcaggtgtcctggattccggacataaaaccagtgcacacc
ccacctttgatacaaatgtggtaccaattagtacaggaaccaatcccagaagcagaaacttggtatattgatgga
gcagcaaatagggaatctaaattagggaaagcaggatatgtgacagatagaggaaaagagaaggtggtagcatta
gaacacaccaccaatcaaaaggcagagttacaagcattgctcctagctttacaagatggggggaacaaaacagaac
atagttacagactcccaatatgttctaggagtcgtaacaggagctcccacagaaacagaccacccaatactagag
caaataataacacagttacagagcaaagaagcaatatacctctcatgggtaccagcccataaaggtataggggggc
aatgaagcagtagacaaattagtaagcaaaggaatcagaagggtattgttcctggaacaaataccacaagcccag
gaagaacatgaaaggtatcataacaattggcaagaccttagagacaggttccaaataccagcattaatagcaaaa
gagatcttgaaagcatgccccaagtgtcaaggaaaaggagagccaatgcatgggcaggtaaacatggaagtgggg
ctctggcagatggattgtacacacttggaagggaaaatcataatagtagcagtacatgtagccagtgggtacaca

gaagccaaactaatcccacaagaaacaggaaaagagacggctatattcctgttacaactatgtgctagatggcca
gtcacacagatacacacagacaatggaccaaatttcaccagccaggaactagcagcagcagcatggtgggctaat
gtacaacatagtacaggtgtgccttacaatccacagagtcagggagtagtagagaacaagaacaggcaattgaag
gaaacaataggcaaaatcagagatgaagtacaatacttagagacagcagtagcaatggcactcctcattcttaat
tttaagaaaaggggaggaatagggggatgacaccagcagaaaggataataaacatgatacacacagaattagaa
ctacaacaaaccaaaattccaaaattttcgaattttcgggtttattacaggacagggaagagccctgagtggcta
gggccagcacaactcctgtggaaaggagaaggtgcagtggtaatcaagacaactacaggtgagctgttgacagtc
ccaagaaggaaagcaaagatcattaagcctctaactaagtatctcaagtataaaacaaaggaattagaggaagta
caatggatacatcattaccaattatatgcacataggtatacacaaaacaaagtaatcatcccacttaccttgaaa
gtatcacaggattatgtggaagcaacagccatagaagtacaaatattgtgggaactatcaagttgtagaagtagg
gatccacctggagtaagagacacacaaggcctcactattagttggacttacaccagattgcacaagggagacaga
gagatagatagaatcagctatgagacagagattactccgcaattagctgatgcaatggtacatgtggagcatttc
tcttgttttacagctcaggatttcaatagagcaatcagaggacagcaattgctaggtgagtgccatcatccacat
tatttaggtaaagaggtacccacattacaatacttagcactaagggcaatacaatggacagaacagcaggcgtgg
gcagctactgcaatagattacataaagatcattcaaaagttattgtgggaccactataagaaaggatgctatcac
agataccatgctagagtaaagaggtatccacacctaagaccactgagaggtacagcaggacaggatacgccccat
gatagtgatccaccacaggtgctaagaccctctcgttatcatccagacaatgatccctcccaagaggaggtagaa
aaatggcatagggcgcctgcacaacctccaactccttgcaattcctgcttttgcaaaagctgcatatatcactgc
atattgtgctttcaacgaaaaggacttggcatccgcatgacaaagtatacgcttatagcattaggtatagcaatt
ataataggtttagtaataatagatttgcaacaagggaagaaaataatggatgggtaacagttttctatgggta
ccagtgtggcagaatgcctcccctcctttgttttgcacagcagatgcaagacatacatgggtcaccatgaactgc
ttgccaacagacccagcccctgtagagacacctatgaatatcacaggagaatggttcaatgtgcacaccaattat
atggtggaccacatggatcaagatatggcagcgctgtttctacaggctacaaagccatgtaccaaattgacccct
atgtgtgttaagatgaagtgtagcaattatactagagaggctacaacagcagcaccatcaacaacagcaccaacg
accaaagggcatacaacagaatggtggggaggaaaagatcctcaatctttactcaattgttcttttaacatgacc
ccaggttttaaagataggaaagctcattattgggctcctttttataaattggatttatggcaacataataatggt
actaatggcacaggggaatattatgtaaagtattgcaacacgtctgcaatgacacaagcctgtgacaaatttcat
tttcagcctttcccagttcattactgtccaccagcagggtatgcattgttcaaatgcaatgacataccatggaat
gggcaagggcaatgtaaaaatgtgtcagcagtacattgtacacatgcaattaatacattggctagtacatggctc
cagttaaatggcacatatgaaaagaacacagatgaggtgcaggtcatgagaaagtataattccaattacagtgta
gcttttatcttttcagagagccaaatagtaaatttgacatgtgtaaggcctgggaataaaacaataaggaatttg
cagataggatcaggcatgacctttactcacaactcatagtagggggagatactagaagagctttttgcagaata
aatggcacacagtggtaccatgctttaaatgctacttatagggctataaagaaggagtataatcttacacagaac
caaccaatcacctggagatctcaaccaagggggacagagaagtagagagtcactggttccaatgccaaggggaa
ttcttttattgtaacttatctaggatctttcagcaaaccaattttaccaatgacaccttctatccaagaaacata
acagaggaaggggaaaggttgagcagaacatggttcacatgtaccatcaggcaagtagtgaataggtggtcacat
gtagaaaagttgatgtacttaccaccaagaaggggacatgttcagtgtacatctaatatcacaggagtcttggtg
gaatcagaatactatccagggagtccatttaatatgaccccatcagccaacatcaaggacctgtggaaaatagat
ttaagaagatataaggtggtggaaatagatccaattggcttagcacccactccaattaaaaggtatgaaccacca
tcaaacaccaaaagtgttgtcaaaagagcttcatatgggtgccacttcaaacaaatttgttacacctcagttcct
tggaatatatcttttgctgatggaggagtgcttcctgactggaataatatgacctggatagaatgggaaagaaaa
gtgaccaatcatacaggcatcattaataatttgttggtagaggctcaaaggaggcaagaagaaaacacacataag
ttacagaagttaggagaatgggataatctttggagttggtttgatatctccagatggtttcagtggattaaaata
gcagtgttaatagtaataggtttaatagcattaagaatagtaatgtggcttattaatatattggggttcttagca
gttgtctgggacgacctgagagcgatcgtcttgtggatccaccggacattggtcgacttaggctggctgattcgt
catctgtggagcagtctaactgactggtggtttggaagtgcttatcctcgcttgatactagcagcagaagcaaca
gcagaagcaactagaagagcaatagcttacattcaagaagaagcagcagcagccaagagaggagaagttgtaccc
atgaggtacaatgaaggtgacaacacccgagatgatgatgaagaagaagtgggtttcccagtacgcccacaagta
cccatcagaaacctgacattcaaaatagctgtggatttctcctggtttttaaaagaaaagggaggactggaaggg
ttgtattattcccctgagagacacaagaaattggacctatatgcttatcatgtatggggagtagttccagggtgg
caaggatacacccctggcccaggcccgaggttcccaacatgttttggcatcctctgggaattggtcccggtggat
gtggggccacagggcctgggagaaggagatgaaagggcattgttgctccatgcggggcaacaagcataccaggac
ctccatggtgagaccctggtgtggcacttcaatcccacccttgcctttgagccaggcatactgaaggctgacaag
ttgggccagcagccattgcctgggatgaagagctgcataacaggaagagactag
>#8 KJ461715 7464 nt

atggggcgaggcactcagcgatgcttactgggaaaaagcttgataagtatgagaagatcaggctgaggccgaaa
ggcaaaaagaagtaccagataaagcatatagtttgggcatccaaagagctggaacgatttggactatcagactcc
ttattggaaagtaaggagggctgccaaaagatcttagaggtcattatcccattggaagaaacagggtccgagtct
ctaaaatccctctttggattagtttcagtcttatggtgtgtacatgcagatgttacagtagaggacacagaggaa
gctaaaaaacagatcagaattaggtgccacataagtgacaaaaaggaggagacctccgcgggcggaggtgcctca
gggcaaaattatccagttgttaggaatgcacagggacagcatcagcatcaggctttgtccccaagaatcctcaag
acatggataacaaccattgaggagaaaaaattttcagcagaggtagttgccttattccaggcactatcagaaggc
tgtataccctatgacataaatcagctcatgaatgccataggggaacatcaaggagcggtacagcttattaaagat
gtagttaatgaggaagcacacacttgggaccagttgcacccacagccagcacagccgcagcccaacgcaggcttg
agatacccatcagggagtgacatagcaggggtatcatcatccatacaagaacagctagaatggatgacaagggca
gtagaccaagtaaatgtgggagaaatatatagaaaatggatagtaatgggtctgcagagatgtgtcaaaatgtac
aacccagttaacattctggacataagacaagggccaaaagaaagcttcaaagactatgtagaccgattctacaaa
tgtctgagagcagaacaggctgatcaatcagtcaaaaattggatgacttcaacccttttgatccagaatgctaac
ccagaatgcaaggccatcttaaaggccatgccaggagctagcttagaagagatgcttacagcatgccaaggcgta
ggagggccacactataagaccaagctgttagcagaagctatggcatcagctttccaaaggacagggaattacaac
atggcgcaaggcccacaaggcccgaaaggggctcgaaggggggcccatgaaaaaccctaatcctaggtgtttcaat
tgtggacgatttggacacatgtccagacaatgcccacagccaaaaaaagatgaagtgctttaagtgtggcagagag
ggccatatggcaagacagtgtcgagcctctgacagtgcacagggaggagcatcctgtttagtggagggcaagaa
gttgacctactattggacacgggtgcagatgatacaataattcatgatcaggacatagaattgggagataactgg
cacccaaaaattataggggggagtaggtggcaatattagagtaaaagcatatcataatgttgacatagaatggcag
ggaaaaagagtaacaggggaagtgttggtgggagacactcccatcaatataatggggagaaatttcttaactaaa
tttggggtgactctaaatatggtagtaggcaaatttaagccgacagaagtacggttaaaagaagggaaagacgga
cctagagtcaaacaatggccattaagtgcagaaaaaatacaggcactaaaagagatagtgcaagatatggtaaat
aaaggacaattggaaaaaatagggccagaaaacccatacaattctccagtattttgtattaagaagaaggacaaa
acaaaatggaggatgctaattgatttcaggaaactaaatgagcacacacaggacttagcagaagtacagttgggg
atcccacacccagcaggtctacccaagaaggcacaggtttccatagtggacattaaagatgcctactatgcagtc
ccgctacacgaggactttcaaaaatatacagcattcacgataccttcagtaaacaacatgggaccaggagaaaga
tatcagtttaaggtgctgccacaggggtggaaggcatctccaactatatttcagaacacagtaggagacctgtta
caacagatcaggaaaaatacccacaaatcctcatgattcaatacatggatgacctcctcatagggtcagatgag
acagtaacagaacatagaaaaatagtagagcaaattaggaacatccttctaaaacgagggttacaaacccctgag
gacaaatatcagccacaaaggccagttaattggttagggtatgaacttaggcctagagaatggacaattcctaga
ataacattgccagttaagcaggtctatacagtcaatgagatccaaaagttagtaggacaattaaattgggccagc
caaatatacccaggataaaaacaaaggctctatgtaaattaattagagggacaaaaggcttaacagaggaagtg
caggtcacagaagaggcagaaacagagcttgtagagaaccaagccattttagaccaagaagctaaaggggatac
tatgatgcagagaagccattagaggtagatatcacacaattgggagaaacccagtgggggtacacagtaagacaa
gataaagaagtactaaagacaggtaaatttgcaaaacaaagaagcgcacatagtaacccattcaaacaattggta
gatgccattatgaaaataggaaaagaaagcttagtatattggggaagaataccacaatttaatgtcccagttaat
aaagaacagtgggacgcgtggtggagtgaacattggcaagtgtcctggattccggatataaaaccagtgcacacc
ccacctttgatacaaatgtggtaccaattagtacaagatccgatcccaggagcagaaacttggtatattgatgga
gcagcaaatagggaatctaaattagggaaagcaggatatgtgacagacagagggaaagaaaaagtagtagcatta
gaacacaccaccaatcagaaagcagaattacaagcattacttctagctctacaagatggggaacaaaacagaac
atagttacagattcccaatatgttctaggaatcataacaggagctcctacagaaacaaaccacccaatactagag
caaataataacacagttacaaagcaaagaagcaatatacctctcatgggtaccagcccataagggtataggaggt
aatgaagcagtagataaattagtaagcaaaggaatcagaaaggtattgttcctggaacaaataccacaagcccag
gaagaacatgaaaggtatcataacaattggcaagaccttagagacaggttccgaataccagcattaatagcaaaa
gagattttaaaagcatgccccaagtgtcaaggaaaaggagagccaatgcatgggcaggtaaacatggaagtggga
ctctggcagatggattgtacacacttggaagggaaaatcataatagtagcagtacatgtagccagtgggtacaca
gaagccaaactaatcccacaagaaacaggaaaagagacggctatattcctgttacaactatgtgctagatggcca
atcacacagatacacacagataatggaccaaatttcaccagccaagagttggcagcagcagcatggtgggctaat
atacaacacagcacaggtgtgccttacaatccacaaagtcagggagtagtagaaaataagaacaggcaattaaaa
gaaacaataagcaaaatcagagatgaagtacaatacttagagacagcagtagcaatggcactcctcattcttaat
tttaagaaaaggggaggaataggggggatgacaccagcagaaaggctaataaacatgatacatacagacttagaa
ctacaacaaaccaaatttccaaaattttcgaattttcgggtttattacaggacagggaagagcccagagtggcta
gggccagcacaactcctgtggaaaggagaaggtgcagtggtaatcaagacaactacaggtgagctgttgacagtc
ccaaaaaggaaagcaaagatcattaagcctctaacaaagcatctcaaatataaaacaaaggaattagaggaagta

caatggatacatcattaccaattatatgcacataggtatacacaaaacaaagcaatcatcccacttaccttgaaa
gtatcacaggattatgtggaagcaacagccatagaagtacaaatattgtgggaactgtcaagttgtagaactagg
aatccacctggagtaagagacacacaagctcttactattagttggacttacaccagattgaataaaggagacaga
gagatagatagaatcagctatgagacagagattactccgcaattagctgatgcaatggtacatgtggagcatttc
tcttgttttacagctcaggatttcactagagcaatcagaggacagcaattgctaggtgagtgccatcatccacat
tatttaggcaaagagatacccacattgcaatacctagcactaagggcaatacaatggacagaacagcaggcatgg
gcagctactgcaatagattatataaagatcattcaaaagttattgtgggatcattataagaaaggatgctttcac
agataccatgttagagtaagaagatttccacagcttagaccactgagaggtacagcaggacaggaaacgccccac
gacagtgatccgccacaggtgctaagaccctctcgttatcatccagataatgatccatcccaagaggggttagaa
aaatggcatagggcgcctgcacaacctccaactccttgcaattcatgcttttgcaaaagctgcatatatcactgc
atattgtgcttccaacaaaaaggacttggcatccgcatgacaaaacataagcttatagtattaggtatagcaatt
ataataggtttaataatagtagatttgcaacaaggggaagaaaataatggatgggtaacagttttctatggagta
ccagtgtggcataatgccacccctcctttgttttgcacagcagatgcaagacacacatgggtcactactaattgc
ttgccaacagatccagcccctatagagacacctatgaatatctcgggagaatggttcaatgtacataccaattat
atggtggaccatatggaccaagatatggcggcattgtttctacaggctacaaagccatgtactaggttgacccct
atgtgtgttaagatgaagtgtagtaattttactaaaccaacttcaacaccacaaccaactacatcatcaaaacca
acaacattgacagcgacaaccaaaaagcttgtaacagaatggtggggaggaaaagatcctcaatctttactcaat
tgctcttttaacatgaccccaggttttaaagataggaaagctcactattgggctcctttttataaattggattta
tggcaacataagaatgatagcgaaactaatgtcacaggggaaggggaatattatgtaaagtattgcaacacgtct
gcaataacacaagcctgtgataagtttcattttcaacctttcccagttcattactgcccaccagcagggtatgca
ttgttcaaatgcaatgacataccatggaatgggcaagggccatgtaaaaatgtgacagcagtacattgtacacat
gcaattaatacattggctagcacatggcttcagctaaatggcacatatgaaaagaacacagataaggtgcaggtc
atgagaaagtataattccaattacagtgtagcttttatcttctcagaaagccaaatagtaaatttaacatgtgta
agaccagggaataaatcaataaggaacttgcaaataggagcaggcatgaccttttactcacaactcatagtggga
ggaaatactagaagagcttattgcaaaataaatggcacacagtggtactctgctttaaatgctacctatcgggct
atacagaaggagtataatcttacagagaaccaatcaatcacctggagatctcaaccaaggggggatagagaagta
gagagtcactggtttcagtgccaaggggaattcttttattgtaatttatctaggatctttcagaaaaccaatctt
acaaataacacctttacccacaaaacataacacaggaaggggaagcgttgagcaacatatggttcacatgtacc
atcaggcaagtagtgaacagatggtcacatgtagaaaagttgatgtacctaccaccgagaaggggacatgttcag
tgtacatccaacatcacaggaatcttggtggagtcagaatactatccaggaagcccatttaacatgaccccatca
gccaatatcaaggacctgtggaaaatagatttaagaagatataaggtggtggaaatagacccaattggcctagca
cccactccactaaaaaggtatgaaccaccaccaagcaaaagcattgtcaaaagagcttcatatgggtgccaattc
aaacaaatttgttacacctcagttccttggaataaatcttttgctgatggtgaagcgcttcctgaatggaacaac
atgacctggatagagtgggaaagaaaagtgactaatcatacaggtatcatcaatgggttgttggtagaggcccaa
aggaggcaagaagaaaacacacataagttacagaagttaggagaatgggataatctttggagttggtttgacatc
tccagatggtttcagtggattaaaatagcagtattaatagtaataggtttaatagcattaagaatagtaatgtgg
cttattaatatattatggtggttcggaagtgcttatcctcgcttgatactagcagcagaagcaacagcagaagca
actagcagagcagtagcttacattcacgaagaagcagcccaaccaggagaagtagtacccttaaggtacaacgag
ggtgactacacccgaactgatgatgaagaagaagtgggtttcccagtacgcccacaagtacccatcagaacaatg
acattcaaaacagctgtggatttctcctggtttttaaaagaaaagggaggactggaagggctattttattcccct
gagagacaccagaaattggacctatatgcttatcatgtatggggactgactccagggtggcagggatacacccaa
gggccaggcccgaggttcccaacatgttttggcatcctctgggaattggtcccggtggaggtagggccacagggc
ctgggagaaggagatgaaagggcaaagttgctccatgcagggcaacaatcattccaggacccctatggtgaggtc
ctggcgtggcacttcaatcccacccttgcctttgagccaggcatactaaaggctgacaagttgggacagcagcct
ttgcctgggcaggagacctttctaacaggaagagactag
>#9 JX860407 7215 nt
atgggcgcgagaagctccgtcttgtcagggaaaaaagcagatgaattagaaaaggttaggttacggcccggcgga
aagaaaaagtacatgttgaagcatgtagtgtgggcagcaaacgaattggacagattcggattggcagagagcctg
ttggaaarcaaagaaggttgtcaaaarattctttcagttttagctccattaacaccaacagggtcagaaaattta
aagagcctctacaatactgtctgcgtcatytggtgcattcacgcagaagagaaagtgaaacatacygaggaagca
aaacaaatagtgcagagacatctagtggtagaaacaggaactgcagatcccatgccgaaaagyagtagaccaaca
gcaccacctagtggcatgaagggaaattacccagtgcagcaagtrggtggcaattatgtycacctaccattaagy
cccaggacattaaacgcctgggtaaaattagtggargaaaagaaatttggggcagaggtagtgccagggtttcag
gcactrtcagaaggctgtactccttatgatatcaatcagatgctaaattgtgtgggagaacaccargcagccatg
cagatcataagggaaatcatcaatgaagaggcagctgattgggacctgcaacatccacaaccaggtccaatacca

gcaggacagcttagggacccaagaggrtcagatatagcaggaaccaccagcacagtggaagaacaaattcagtgg
atgtacaggcagcagaatccgataccggtaggaaacatctacaggagatggattcaacttgggttgcaaaaatgt
gtgaggatgtataatccaacaaacattctagatgtgaaacagggacccaaagagccatttcaatcttatgtagac
aggttctacaaaagtttgagrgcagarcaaacagatccrgcagtwaagaattggatgactcagacactgctgatt
caaaacgccaacccagaytgtaaattggttttaaagggtctgggaatgaaccccactctagaagaratgctaacr
gcctgtcaaggggtaggggaccaggacaaaaggctaggctaatggcagaagccctgaaagaagccctaacacca
gggccactcccatttgcagcagcccagttaagaggaggaccaaaaagaacagtcaaatgttggaattgtggaaaa
gatggacacactgcaagacagtgtaaagctccaagaagacarggctgctggaaatgtggaaaaatgggccacgtc
atggctaaatgcacagaaagacaggcaacagcgcaggtcgagggtcagcctgtggaagtattactggacacaggg
gctgatgactctatagtagcaggagtagaattaggaacaaattacaccccaaagatagtaggaggaattggaggt
tttattaatactaaagaatataaaaatgtagaaatagaagtactagggaaaagaattagaggaacactcatgaca
ggagataccccaatcaatatttttggcagaaatttactaacagctttgggtatgtccttaaacttcccaatggcc
caagtggagccggtaaaagtaacactaaaaccaggaaaagatggaccaaaagtaaggcaatggccactatcaaaa
gaaaagatacaggcattaaaagagatctgtgaacagatggaaaaggatggtcaattagaagaagctccacctacc
aatccatacaatacacctacttttgctataaagaagaaagacaaaaacaagtggagaatgctaatagatttcagg
gaactaaataaagtcactcaggatttcacagaagtgcagttgggaattccacacccagcaggattggcaaagaga
aaaaggattacagtcctggatgtaggggatgcatatttctctatacccttagatgaggacttcaggcagtatact
gctttcaccctaccatcggtaaataatgcagagccaggaaaaagatatatctacaaggttctaccacaagggtgg
aaaggatcaccagcaatttttcaacatacaatgagaggtgtattggagccatttagaaaagcaaatccagatgta
accctaattcagtacatggatgacatcctgatagccagtgacagaacagacttagaacatgacagagtagtgctg
cagttaaaagaactcttaaacggaatgggcttctctactccggatgagaagttccagaaagaccctccatatcaa
tggatggggtatgaactgtggccaacaaaatggaaattgcaaaaaatagaattgcctcagaaagacaattggaca
gtaaatgacattcagaaactagtaggagtgttaaattgggcagcgcaagtctatccaggaatcaaaaccaaacat
ctttgtaaactgatcagaggaaaaatgactctgacagaggaagtccaatggactgaattggcagaagcagagtat
gaggaaaacaagatcattctcagtcaggaacaagaaggatgttactaccaagagggaaagcctttagaggcaacg
gtagtgaaaaatcaggataatcagtggtcttacaaaatccaccaggaagataaagtattgaaagtaggcaaattt
gccaaaataaagaatacacatacaaatggagttaggctattggcacatgtggtacaaaagataggaaaggaggct
atagtgatctggggacggatcccaaaattccatctaccagtagagagagaaatctgggagcaatggtggacagac
tactggcaggtgacctggataccagactgggactttgtatcaacacccccttggtcagattagttttcaaccta
gtaaaggatcctcttgaaggagaagaaacattctacacagatggatcatgcaacaggcagtctaaagaagggaaa
gcaggttatgtaacagatggaggaaaagacagggtaaaggtgttagaacagaccactaatcagcaagcagagcta
gaggcctttctcatggcattaacagactcaggaccaaaagcaaatatcatagtagattcacaatatgtaatggga
atagtagcaggtcaacctactgaatcagaaaataagatagtaagccaaatcatagaagaaatgatcaaaaagacg
gcaatttatgtcacatgggtaccagctcacaaaggcctaggaggaaatcaagaagtagaccacctagtcagtcag
ggaatcagacaagtattgtttctagaaaagatagaaccagctcaagaagagcatgaaaaataccacagcaatgta
aaagagttggtattcaaatatggcataccaagattggtagcaaaacaaatagtagatacctgtgacaaatgccac
caaaaaggggaagctatacatggacaagttaatgcagatctaggaacctggcaaatggattgcacacacttagaa
ggaaaaatagtcatagtggcagtacatgtggctagtggattcatagaagcagaagtaattccacaagagacaggg
agacagacagcattattcctgttaaaattggcaagtagatggcctatcacacatttgcatacagacaatggtgcc
aactttacttcacaagaagtaaaaatggtagcttggtggacaaatatagagcacacctttggagtaccctataat
ccacagagccaaggagtagtggaagctatgaatcaccacttgaaaacacaaatagacagaattagagagcaagca
aattcagtagaaaccatagtactaatggcagttcattgcatgaattttaaaagaaggggaggaataggggatatg
accccagcagaaagactaattaatatgattaccacagaacaagaaatacaattccagcaatccaaaaattcaaaa
tttaaaaatttcgggtctattacagagaaggcagagatcagctgtggaaaggacccggtgaactattgtggaaa
ggggaaggagcagtcatcttaaaggtagggacagatataaaggtagtgcccagaagaaaagctaagatcataaaa
gatctcataaagcatttaaaatataacactaaagacctacagcaggcatgttatgtccccatcataaagttgga
tgggcatggtggacttgcagcagggtaatctttccattacaaggagaatctcatttagaagtacaagggtactgg
aacttaacaccagagaaaggatggctcagtacttatgctgtaaggataacttggtacagcagaaattttggaca
gatgtaacaccagattatgcagatacattactgcatggatcttatttctcttgctttacagcgggagaagtaaga
agagccatcaggggagaacagttactgtcttgctgcaagttcccaagagctcatagaaatcaggtaccaagtctg
caatatctagcactgagtgtagtaagtatggcagaagctcccccagaggatgaggccccacagagagagccgtgg
gatgaatgggtaatggaagtcctggaggagctcaaagaagaagctaaaaggcattttgatcttcgcttgctaact
gcgcttggtaactacatttatgataggatgggatgtcttgggaatcaactgcttatcgccatcttgctagtaagt
gcttatgggatctattgtgctcaatatgtcacagtattctatggtgttccagcatggaggaacgcgacaattccc
ctcttctgtgcaaccgagaatagggacacttggggtacaactcaatgcttgccagataatgatgactattcagaa

ctagcaatcaatgtcacagaggcttttgatgcctggaacaatacagtcacagagcaagcaatagaggatgtctgg
aatctctttgagacatccataaaaccatgtgtaaaattaaccccattatgtatagcaatgagatgcaataaagct
gagacagacaagtggggactaacaggaacagcagcaccaccaacaacaaagacactaaagacaacatcaacaaca
aaaccaccagtactcgaagtaaatgataccgaaccctgtgtaaaacttaacaactgcacaggattggaaccagaa
ccaatgataggatgtaaattcaatatgacaggattgaaaagaggcaggaaaaaggagtacaatgaaacatggtac
tctagtgatttggtctgtgagcaaggtggtaacaacacaaatgaaagcagatgttatatgaatcattgtaataca
agtgtaatccaagagtcctgtgacaaacattattgggatgctattagatttagatactgtgcaccaccaggctat
gctttgcttagatgtaatgattcaaattattcaggttttatgcctaattgttctaaggtagtagtctcttcatgt
acaaggatgatggaaacacaaacctctacatggtttggttttaatggcactagggcagaaaatagaacttacatt
tattggcatggtcataggaatagtactataattagtttaaatagctattacaatttaacaataagatgtagaagg
ccagggaataaaacagtactaccagtcaccataatgtcagggttagttttccattcacaacccataaatgagagg
ccaagacaggcatggtgctggtttggaggaaaatggaagtcagcaataaaagaggtgaaggagaccttggtcaaa
catcccaggtatacaggaaccaatgatacaagtaaaattaatctgacagcaccaggaggaggagaccctgaggtc
accttcatgtggacaaattgtagaggagaattcctctattgtaaaatgaattggttcctaaattgggtagaagat
agaaacagaacgcagatggaggcgagaaacaacaacgataaagagaggagaaagaggaactatgtgccatgtcac
attagacaggtgatcaatacatggcacagagtaggtaaaaatgtatatttgccgcctagagaaggagacctgaca
tgtaactccactgtgaccagtctaatagcagaaatagactggtatgataaaaaccaaactaatatcaccatgagt
gcagaggtggcagaactgtatcgattggaattggggggattacaaattagtagaaatcactccaattggcatagcc
ccaacaaatgtaaagaggtacaccactactactacctcaagagctaaaaattcatggggatgtgcgtttaggcag
gtctgccacactactgtcgaatggccagccgcaaatgtgacaccagattggaataacatgacctggcaagagtgg
gagagaaaggttgactatctagaggcaaatataacgcttctattagaagaagcacaaattcaacaagaaaagaac
atgtatgagttgcaaaaactaaatagctgggatgtatttggcaattggtttgatctcacctcttgggtaaaatat
atacagtatggaatatatatagtagtaggaattatattgttaagaatagtaatctatgtagtacagatgttagct
aggttaaggaaagggtataggccagttttctcttccccacctaattatcgccagcagctcacctatattggacct
cagatgtataatgaaggccagtacatgaatacccccctggagaaaccctgcaagagaaagagccaaattagcatat
aggaaacaaaatatggatgatgtagatgaggaggatgatgaattagtaggggtagcagtacatccaaaagttcca
ttaagagcaatgtcatacaaattggcaatagacatgtcacattttataaaagaaaaggggggactggatgggatt
tattacagtgagagaagacataggattctagacatgtacctagaaaaggaagaaggaataataccagattggcag
aattacacctctgggccagggataagatacccaatgttctttggctggttatggaaactggtaccagtagatgta
tcagatgaagctcaagaagatgagacccactgtctggtgcatccagcacagacatatcagtgggatgacccttgg
ggagaagtcctagcatggaagtttgatccaacattagcttatacctatcaggcatttgttaaatacccagaagag
tttggatataagtcaggcctgtcagaggaagaggttaagagaaggctaaccgcaagaggccttttaaaatggctg
acaagaaggaaacca
>#10 U79412 7287 nt
atgggcgcgagaaactccgtcttgtcagggaagaaagcagatgaattagaaaaaattaggctacgacccggcggg
aagaaaaagtacatgttgaagcatgtagtatgggcagcaaatgaattagatagatttggattagcagacagcctg
ttggagaacaaagaaggatgtcaaaaaatactttcggccttagctccattagtgccaacaggctcagaaaattta
aagagcctttataatactgtctgcgtcatctggtgcattcacgcagaagagaaagtgaaacacactgaggaagca
aaacagatagtgcagagacacctagtggtggaaacaggaacagcagaaactatgccaaaaacaagtagaccaaca
gcaccatctagtggcaaaagaggaaattacccagtacaacaaataggtggtaactatacccacctaccattaagc
ccgagaacattaaatgcctgggtaaaattgatagaggagaagaaatttggagcagaagtagtgccaggatttcag
gcactgtcagaaggctgcatcccctatgacattaatcagatgttaaattgtgtgggagaacatcaagcagctatg
cagattatcagagaaattataaacgaggaggctgcagattgggacctgcagcacccacaacaagctccacaacaa
ggacagcttagggagccgtcaggatcagacattgcaggaacaactagtacagtagatgaacaaatccagtggatg
tacagacaacagaaccccataccagtaggcaacatttataggagatggatccaactggggttgcaaaaatgtgtc
aaaatgtataacccaacaaacattctagatataagacaagggccaaaagagccatttcagagctatgtagacagg
ttctacaaaagcttaagagcagaacaaacagatccagcagtaaagaattggatgactcaaacactgctgattcaa
aatgctaacccagattgcaagctggtgctgaagggggctgggtatgaatcccaccctagaagaaatgctgacggct
tgccaaggagtaggaggaccaggacaaaaggcaaaattaatggcagaagccctgaaagaggcccttgcaccaggg
ccactccctttttgcagcagcccaacagaagggaccaagaaagccaattaagtgttggaattgtgggaaagaggga
cactctgcaaggcaatgcagagccccaagaagacagggctgctggaaatgtggacaaatgggccatgttatggcc
aaatgcccagacagacaggcaggtttttttaggctttggcccatggggaaagaagccccgcaatttccccatggcc
caaatgcatcaggggctgacaccaactgctcccactgctcatattgagggacagcctgcagaagtactattggat
acaggggctgatgattctattgtagcaggaatagagttaggtccacattataccccaaaaatagtaggaggaata
ggaggttttattaatactaaagaatacaaaaatgtagaaatagaagttttaggcaaaaggattaaagggacaatc

atgacaggggacaccccgattaacattttggtagaaatttgctaacagctctgggaatgtctctaaatttcccc
atagctaaggtagagcctgtaaaagtcaccttaaagccaggaaaagatggaccaaaattgaggcagtggccatta
tcaaaagaaaagatagttgcattaagagaaatctgtgaaaagatggaaaaggatggtcagttggaggaagctccc
ccgaccaatccatacaacaccccccacatttgctataaagaaaaaggacaagaacaaatggagaatgctgatagat
tttagggaactaaataaggtcactcaggactttacagaagtccaattaggaataccacaccctgcaggactagca
aaaaggaagaggatcacagtactggatgtaggtgacgcatatttctccatacctctagatgaagaatttaggcag
tacactgcttttactttaccatcagtaaataatgcagaaccaggaaaacgatacatttataaggtcctgcctcag
gggtggaaggggtcaccagccatcttccaacacactatgagaaatgtgctggaacccttcaggaaggcaaatcca
gatgtgaccctagtccagtatatggatgacatcttagtagctagtgacaggacagacctggaacatgacagggta
gttttacagttaaaggaactcttaaatagcatagggttttctaccccagaagagaagttccaaaaagatcccccca
tttcaatggatggggtatgaattatggccaacaaaatggaagttgcaaaagatagagttgccacaaaaagagacc
tggacagtgaatgatatacagaagttagtaggagtattaaattgggcagctcaaatttatccaggtataaaaacc
aaacatctctgtaggttaattagaggaaaaatgactctaacagaggaagttcagtggactgagatggcagaggca
gaatatgaggaaaataaaataattctcagtcaggaacaagaaggatgttattaccaagaaggcaagccattagag
gccacggtaataaagaatcaggacaatcagtggtcttataagattcaccaagaagacaaaatactgaaagtagga
aaatttgcaaagataaaaaatacacataccaatggagttagactattagcacatgtaatacagaaaataggaaag
gaagcaatagtgatctggggacaggtcccaaaattccacttaccagtcgagaaggatgtatgggaacagtggtgg
acagactattggcaggtaacctggataccagaatgggattttatctcaacgccgccactagtaagattagtcttc
aatctagtgaaggaccctatagagggagaagaaacctattatgtagatgggtcatgtaataaacagtcaaaagaa
gggaaagcaggatatatcacagataggggcaaagacaaagtgagagtcttagaacagactactaatcaacaagca
gaattggaagcatttctcatggcattgacagactcagggccaaaggcaaatattatagtagattcacaatatgtt
atgggaataataacaggatgccctacagaatcagagagcaggctagttaaccaaataatagaagaaatgattaaa
aagacagaaatttatgtagcatgggtgccagcacacaaaggtataggaggaaaccaagaaatagaccacctagtt
agtcaagggattagacaagtcctcttcttggaaaagatagagccagcacaagaagaacatgataaataccatagt
aatgtaaaagaattggtattcaaatttggattacccagactagtggccaaacagatagtagacacatgtgataaa
tgtcatcagaaaggagaagctatacatgggcaggtaaattcagatctagggacttggcaaatggattgtacccat
ctagagggaaaaataatcatagttgcagtacatgtagctagtggattcatagaagcagaagtaattccacaagag
acaggaagacagacagcactatttctgttaaaattggcaagcagatggcctattacgcatctacacacagataat
ggtgccaactttacttcgcaagaagtaaagatggttgcatggtgggcagggatagagcacacctttggggtacca
tacaatccacagagtcagggagtagtggaagcaatgaatcaccatctaaaaaatcaaatagatagaatcagggaa
caagcaaattcaatggaaaccatagtattaatggcagttcattgcatgaattttaaaagaaggggaggaataggg
gatatgactccagcagaaaggttacttaacatgatcactacagaacaagaaatacaattccaacaatcaaaaaac
tcaaaatttaaaaatttcgggtctattacagagaaggcagagatcaactgtggaagggacccggtgagctattg
tggaaaggggaaggagcagtcatcttaaaggtagggacagacattaaggtagtacccagaagaaaggctaagatt
atcaaagatctcataaaatatctgaaatataaaactaaagatctacaaaaggtttgctatgtgccccatcataag
gtcggatgggcatggtggacctgcagcagagtaatcttcccactacaagaagaaagccagttagaagtacaaggg
tattggaatttaacaccagaaagagggtggctcagtacttatgcagtgagaataacctggtactcaaggaacttt
tggacagatgtaacaccagactatgcagacattttactgcatagcacttatttcccttgctttacagcgggagaa
gtgagaagggccatcaggggagaacaactgctgtcttgctgcaggttcccgagagctcataagaaccaggtacca
agtctacagtacttagcactgagagtagtaagtatggaagaaagacctccagaagatgaaggcccacaaaggaa
ccatgggatgaatgggtagtggaggttctggaggaactgaaagaagaagctttaaaacattttgatcctcgcttg
ctaactgcgcttggtaattatatctatgatagaatgggatgtcttgggaatcagctgcttatcgccatcttgttt
ctaagtgcctatgggatctattgcattcaatatgtcacagtcttttatggtgtaccagcttggaggaatgcgaca
attcccctcttctgtgtaaccaggaatagggatacttggggaacaactcagtgcctaccagataatgatgattat
tcagaattggcccttaatattacagaaagctttgatgcttgggagaatacagtcacagaacaggcaatagaggat
gtatggcatctctttgagacctcaataaagccttgtgtaaaattaaccccattatgcattactatgaaatgcaac
aaaagtgaaacagataaatggggattgacaaaatcatcaacaacaacagcagcaccaacaacaaaaacaacaaca
acaaaggaaatagaagtggtcaatgaaaatagtacttgtgtaaatcgtgataattgcacaggcttggaacaagag
ccaatgataagctgtaaattcaacatgacagggttaaaaagagacaagaaaagagagtacaatgaaacttggtac
tctgcagatttggtttgtgaacaaggtaatagcactgaagatgaaagtagatgttacatgaatcactgtaacact
tctgttattcaagaatcttgtgacaaacattattgggatgctattagatttaggtattgtgcacctccaggttat
gctttgcttagatgtaatgacacaaagtattcaggctttatgcctaactgttctaaggtggtggtctcttcatgc
acaagaatgatggagacacagacttctacttggtttggctttaatggaactagagcagaaaatagaacttatatt
tactggcatagcaaagataataggactataattagtttgaataagtataataatctaacaatgaaatgtagaaga
ccaggaaataagacagttttaccagtcaccattatgtctggattggttttccactcacaaccaatcaatgaaagg

ccaaaacaggcatggtgtaggtttgaaggaaattggaaggaggcaataaaagaggtgaagcagaccattgtcaaa
catcccaggtatactggaactaacaatactgataaaatcaatttgacggctcctcgaggaggagatccggaagtt
accttcatgtggacaaattgcagaggagagtttctctactgtaaaatgaattggtttctaaattgggtagaagat
aagaatctgactggaactacccagaagccacaggaacagcataaaaggaattacgtgccatgtcatattagacaa
ataatcaacacttggcataaagtaggcaaaaatgtttatttgcctccaagagagggagacctcacgtgtaactcc
acagtaaccagtctcatagcaaacatagattggattgatggaaaccaaactaatatcaccatgagtgcagaggtg
gcagaactgtatcgattggaattgggagattataaattagtagagatcactccaattggcttggcccccacaaat
gtgaagaggtacactactggtggcaccccaagaaataaaaatgcttggggatgtgcatttagacaagtctgccat
actactgtaccatggccaaatgcaagtctaacaccaaattggaacaatgagacttggcaagagtgggagcgaaag
gttgacttcttggaggaaaatataacggcccttctagaagaggcacaaattcaacaagaaaagaacatgtatgaa
ttacaaaagttgaatagctgggatgtgtttggcaattggtttgaccttgcttcttggataaggtatatacaatac
ggagtttatatagttgtaggagtaatactgttaagaatagtcatctatatagtacaaatgctagctaagttaagg
caagggtataggccagtgttctcttccccaccttcttatttccagcagctaacctacctacaaggacagaaatac
aatcagggacagtttatgaatactccatggaaaaacccagctggagagagggaaaaattagcatacagaaaacaa
aatatggatgatatagatgaggaagatgatggcttggtaggggtaccagtgaggccacgagttcccttaagagca
atgacttacaaattggcagtagatatgtctcattttataaaagaaaaggggggactggaagggatttattacagt
gaaagaagacataaaatcttagacatatacttagaaaaggaagaaggcatcgtgccagattggcaggattacacc
tcgggaccaggacctagatacccaaagacatttggctggctatggaaattagtccctgtaaatgtatcagatgag
gcacaggagggtgaggagaattatttactgcatccagctcaaacttcccagtgggatgacccttggggagaggtt
ctagtatggaagtttgatccaactctagcctacacttatgaggcatatattagatacccagaagagtttggaagc
aagtcaggcctgtcagaggaagaggttagaagaaggctaaccgcaagaggcctcttaaaaatggctgacaagaag
gaaactagctga
>#11 JQ864086 7218 nt
atgggcgcgagaaactccgtcttgtcagggaagaaagcagatgaattagaaaaaattaggttacggcccaacgga
aagaaaaagtatatgttgaagcatgtagtatgggcagcaaatgaattggacagattcggattagcagaaagcctg
ttggataacaaagaaggttgtcaaaaaattctttcggttttagctccattagttccgacaggttcagaaaattta
aagagcctttataatactgtctgcgtcatttggtgcattcacgcagaagagaaagtgaaacatactgaggaagca
aaacaaatagtgcagagacatctagtggtggaaacaggaacagcagacagaatgccagtaacaagcagaccaaca
gcaccacctagtggcagaggaggaaattacccagtgcagcaagtaggtggcaattatgtccacctacccttaagt
ccaagaacattaaatgcttgggtaaaattggtagaagagaaaaaatttggggcagaggtagtgccaggatttcaa
gcgctatcagaaggctgcactccctatgatatcaatcaaatgctaaattgtgtaggagaacatcaggcagccatg
caaattattagagagattataaatgaagaagctgccgattgggatttacaacacccaccgccaggtccaccacca
gcagggcaacttagagagccaagaggatcagacattgcaggaactactagtacagtagatgaacaaatccaatgg
atgtacaggcaacaaaaccctataccagtaggcaacatttatagaaggtggatccaattagggctgcagaaatgt
gtaagaatgtataacccaacaaacattttagatgtgaaacaaggaccaaaagagccatttcaaagctacgtagat
agattctataaaagtctaagagcagagcaaacagatccggcagtaaagaattggatgacccaaacactgctgatt
caaaatgctaacccagattgtaaattggtgctcaagggtctgggtatgaatcccactttagaagaaatgctgaca
gcctgtcagggaataggagggccaggacaaaaagctagattaatggcagaagcattgaaagaggcactgagacca
gaccaactcccatttgcagcagtccaacagaaaggacaaaggaggacaatcaagtgttggaattgtggaaaggag
ggacactctgcaagacaatgcagggcccctagaagacagggctgctgggggtgcggaaaaacgggtcatgttatg
gccaaatgccctgaaagacaggcgactgcctacattgaagaacagcccgtagaagtattattagatacaggggct
gatgattcaattgtagcagggatagaattaggtccaaattataccccctaaagtagtaggaggaataggaggcttc
attaataccaaagaatataaagatgtaaaaataaaagtcttaggcaaggtgattaagggaacaattatgacggga
gataccccaattaatatttttggcagaaatttgctaacagctatgggcatgtctttaaatctccccatagctaag
gtggagcctataaaagtaacactaaaaccagggaaagaaggaccaaaattaagacagtggccgctatcaaaagaa
aagataattgcattaagagaaatctgtgaaaaaatggaaaaagatggccagttagaggaagcccctccaaccaat
ccgtataacacccccacttttgctataaagaagaaagacaaaaataaatggaggatgctaatagatttagagaa
ttaaataaggtcactcaagactttacagaagtacagttaggaataccacaccctgcaggactagcaaagagaagg
aggatcacagtattggatgtaggtgatgcatatttctccatacctctagatgaagaattcaggcagtacactgcc
tttactttaccatcagtaaataatgcagaaccaggaaaaagatacatctataaggtattacctcaagggtggaag
gggtcaccagctatttttcagtatactatgagaaatgtattagaacctttcaaaaaagcaaatccagatgtgacc
ctgatccaatacatggatgacgtcttaatagctagtgatagaacagatttagagcatgacagggtagttttacag
ttaaaggaacttctgaacggcataggattctctaccccagaagagaagttccagaaagatccccccattccagtgg
atgggatatgaattgtggccaaccaaatggaaactgcagaaaatagagttgccacaaagagagatttggacagta
aatgacatacaaaaattagtaggagtgctaaattgggcagcacaaatttatccaggaataaagactaaacatctt

tgcagactaatcagaggaaaaatgactttaacagaagaggttcagtggactgagatggcagaggcagaatatgaa
gaaaacaagataattctcagtcaagaacaagaaggatgttactaccaagagggaaaaccaatagaggcaacagta
ataaagagtcaggataatcaatggtcatataaaattcaccaagaagacaaagtactgaaagtaggcaaatttgca
aaggttaaaaatacacatacaaatggagtcagattactagcacacgtagtgcagaaaataggaaaagaagcacta
gtaatttggggagaggtgccaaaattccatttgccagtagaaagagaaatttgggaacaatggtggacagattat
tggcaagtaacctggataccagattgggactttgtgtcaacacctcccttagtcagattagtcttcaacctagta
aaagagcctatacaggggcagaaacattctatgtagatggatcctgtaataggcagtcaagagaaggaaaagca
ggctatgtggcggataggggcagagacaaagcaaaactttagaacagactaccaaccaacaagcagagttggaa
gccttctatctagccttagcagattcgggaccaaaagcaaatattatagtagattcccaatatgttatgggcata
atagcaggtcaacccactgaatcagaaagtaggttagtaaaccagataatagaggagatgattaaaaaagaagca
atttatgtagcatgggtgcctgcacataaaggaataggaggaaatcaagaagtagatcacctggttagccaggga
attagacaagtcctattcttagaaaaaatagaaccagcacaagaagagcatgaaaagtaccatagtaatgtaaaa
gaattggtattcaaatttggtttacctaggctagtagcaaaacagatagtagacacatgtgataaatgccaccag
aaaggagaagccatacatgggcaagtaaatgcagaactagggacttggcaaatggactgtacgcacctagaaggc
aaaataattatagttgcagtacatgtggctagtggattcatagaggcagaagtaatcccgcaggaaacaggaaga
caaacagcactgtttctgttaaagttagctggcagatggcctatcacacatctgcatactgataatggtgccaat
ttcacatcacaagaagtaaaaatggttgcctggtgggcagggattgaacagacctttggggtgccttataatcca
cagagccaaggagtagtggaagcaatgaaccatcatttaaaaacccagatagatagaattagagaacaagcaaac
tcaatagagactatagtactaatggcagttcattgtatgaattttaaaagaaggggaggaataggggatatgact
ccagcagaaagattagtcaatatgatcaccacagaacaagaaatacaattccaacaatcaaaaaattcaaaattt
aaaaattttcgggtctattacagagaaggcagagaccagctgtggaaaggacccggtgagctattgtggaaaggg
gaaggagcagtcatcctaaaggtagggacagagatcaaggtagtaccaaggaggaaagctaaaattatcaaagac
ctcatcaaacacctgaaatataacactaaagacctacagatggcttgttatgtgccccatcataaagttggatgg
gcatggtggacttgcagcagagtaattttcccattaagagatgagactcatttggaagtacaaggatattggaat
ttggcaccagaaaaaggatggctcagtactcatgcagtaagaataacctggtactccagaaatttctggacagat
gtaacaccagattatgcagacactttactgcatagcacttatttcccttgcttttcagagggagaagtacgaagg
gccatcaggggagagaaattgctgtcttgctgcaagttcccgaaagctcataaaaatcaggtaccaagcctacag
tatctagcactaacagtagtaagtatggcagaaagacctccagaagatgaagccccacagagggaaccatgggat
gaatgggtagtggaagttctggaggaattaaaagaagaagccctgaaacattttgatcctcgcttgctaactgcg
cttggtaactatattatgggatgtcttgggaatcagctgcttatcgcgctcttgctagtaagtgctttagagatt
tattgtgttcaatatgtaacagtattctatggtgtaccagcatggaagaatgcgacaattcccctcttctgtaca
accaggaatagggacacttggggaacaacacaatgcttgccagataatgatgattactcagaattggcaatcagt
atcacagaggcttttgatgcttggaataatacagtcacagaacaagcaatagaggatgtgtggaacctctttgaa
acatccattaagccctgtgtaaaactcaccccactatgtatagcaatgagatgtaataaaactgagacagatagg
tggggtttgacaaggaacgcagggacaacaacaacaacaacaacaacaacagcagcaacaccaagtgtggca
gaaaatgttataaatgaaagtaatccttgcataaaaaataatagttgtgcaggcttggaacaggagcccatgata
ggttgtaaatttaacatgacagggttaaaaagggacaaaaggatagaatataatgaaacatggtattcaagagat
ttaatctgtgagcagtcagcgaatgaaagtgagagtaaatgttacatgcatcattgtaacaccagtgttattcag
gaatcctgtgacaagcattattgggatgctattagatttagatattgtgcaccgccaggttatgctttgcttagg
tgtaatgattcaaattattcaggctttgctcctaactgttctaaggtagtggtttcttcatgcacaagaatgatg
gaaacgcaaacctctacttggtttggcttcaatggtactagggcagaaaatagaacatacatttattggcatggc
aaaagtaatagaaccataattagcttaaataagtattataatctaacaatgagatgtagaagaccaggaaataag
acagttttaccagtcaccattatgtcagggttggtcttccattcgcaacccataaatgagagaccaaaacaggcc
tggtgctggtttggaggaagctggaaagaggccatccaggaagtgaaggaaaccttggtcaaacatcccaggtat
acgggaactaatgatactaagaaaattaatctaacagctccagcaggaggagatccagaagtcacttttatgtgg
acaaattgtagaggagaattcttatactgcaaaatgaattggtttcttaattgggtagaggacagagaccaaaag
agtagcagatggagacaacaaaatacgagagagcgacagaagaaaaattatgtgccatgtcatattagacaaata
atcaacacgtggcacaaagtaggcaaaaatgtatatttgcctcctagggaaggagacctgacatgtaattccact
gtaactagtctcatagcagagatagattggaccaatagcaatgagaccaatatcaccatgagtgcagaggtggca
gaactgtatcgattggagttgggagattacaaattagtagagattactccaattggcttggcccccacaagtgta
agaaggtacaccacaactggtgcctcaagaaataagaattcatggggatgtgcttttaggcaagtctgtcacact
actgtaccatggccaaatgaaacattggtgcctaattggagcaatatgacttggcaagagtgggaaagacaggtt
gacttcctagaggcaaatataactcaattattagaagaagcacaaattcagcaagaaaagaatatgtatgaattg
caaaaactaaatagctgggatatctttggcaattggtttgaccttacttcttggataagatatatacaatatggt
gtactaatagttttaggagtagtagggttaagaatagtaatatatgtagtgcagatgttagctaggttaagacag

ggttataggccagtgttctcttcccctcccgcttatgttgtcatcagaattggaatagcctacctacaacctcag
aaatatagtgagggacaatatatgaataccccctggaggaacccagcaacagaaagagaaaaattaggttataga
caacaaaacatggatgatgtggatgatgaagatgatgacttagtaggtgtctcagtgcacccaagagtcccctta
agggccatgacatacaaattggcaatagacatgtctcattttataaaagaaaagggggactggaagggatttat
tacaatgagaaaagacatagaatattagatatgtacatggaaaggaagaaggaataataccagattggcaaaat
tacacatcagggccaggaactagataccctatgtactatgggtggctctggaaattagtcccagtagatgtctca
gatgaagctcaggaagacgagacacattgcctgatgcatccggcacagactcatcagtgggatgaccccctggga
gaggtactggcatggaagtttgatccagaattagcttatagctataaggcatttattaagtacccagaagagttt
ggtagtaagtcaggcttgtcagaggaagaggtaaagagaaggctaaccgcaagaggccttattaaaatggctgac
aagaaggaaacaagctga
>#12 AF334679 7233 nt
atgggcgcgagaggctccgtcttgtcagggaaaaaagcagatgaattagaaaaagttaggttacggcccggcgga
aggaaaaagtacatgcttaagcatattatatgggcagccagagaattagacagatttggatcggcagaaagcctg
ttggaaagcaaagaaggatgtcaaagaatattagcagtactagctccattaatgccaacaggctcggaaaattta
aaaagcttgtttagtactgtctgcgtagtttggtgtctgcacgcagaaatgaaagtgaaagacacagaggaagct
aagaaaacagtacagagccatctagtggtagaaagtggaactgcagaaaaattgccagctcaaagcagaccaaca
gctccacctagtggaggaaactatccagtgcagcaagtaggaaataattatgtgcacactccactgtccccacgg
acactcaatgcgtgggtcaaactggtagaagaaaagaaatttggagcagaagtagtgccaggatttcaggcacta
tcagaaggctgtacaccctatgacattaatcagatgcttaattgtgtaggtgaacatcaggcagccatgcaaatt
atcagagaaataattaatgaggaagcagcagactgggacctacaacacccaagaggacaacagccggcgcaaccg
gctggagggctaagggaaccatcaggatcagacatagcagggacaactagcaccccaagtgaacagatagaatgg
atgtatagggcacaaaacccagtcccagtgggagacatctatagaaggtggatccagctgggactccaaaaatgc
gtcaggatgtataacccaacaaacatcctggatgttaaacaaggtccaaaggagccctttcaaagttatgtagat
aggttctacaaaagtttgagagcagagcagacagatccggcagtgaagaattggatgacacaaacactgctgatt
cagaacgccaaccctgattgcaaactagtattaaaagggttgggcatgaatcccaccctagaagagatgctaaca
gcctgtcaaggggtaggaggcccaggacagaaggcccgcctcatggcagaggccatgaaagacgccctcacgggg
tctttagtggcagcacagtttaggggagcagcgaaaggccaagggaataagcctatcatcaggtgttttagagca
ctaatagagggtcagcccctagaggttctactggacacaggggcagacgactccatagtagcagggatagagtta
ggtccagggtacacaccaagaattgtaggggggattggaggattcataaatacaaaagaatatagaacagtaaaa
atagaagttttaggaaaggtaataagagaaacactaatgacaggagataccccgataaatatttttggcagaaat
attctagcagcattaggggtctctttaaattacccagtggcaaaggtagaatatacaaaagtaaagttgaaggag
ggaatggatgggccaagattaaaacaatggcccttatctaaggaaaagatacaagcattaacagagatctgcgag
aaaatggaaaaggagggacaattggaacgagcccctcctacaaatccttataatacaccgacctttgctattaag
aaaaagaataaggacaagtggagaatgctcatagatttcagagaattaaacaaaatgacacaagaattcacagaa
gtacagctgggtatcccacatccggcaggcctaaaggaaatggaaaggataacagtgctagatatagggggatgcc
tatttttcagtaccccctagacccggaatttagacaatacacagcattcactataccatcagtgaacaaccaagaa
ccaggaaagaggtatatttacaaggtcctgccacaaggatggaagggatctcctgcaattttccaggcaaccatg
aggcaggtattagaacccttcagaaaagctaacccagatgtgcttctcgtccagtatatggatgatttactaatt
ggcagtaacagaggcctaacagagcatgacaaaatggtaacacagttaagagacatgctcaacaatctggggttc
agtaccccagaagacaagtttcaaaagaatccaccattacagtggatgggatatttgttataccccaagaagtgg
aaattacagaaaatagaactccctgaaaaggagcgatggacagtaaatgacatacaaaaactagtgggagttcta
aattgggcagcacagatatatccagggattaaaacaaaaaacctgtgcaaaatgatcagagggaaaatgactctg
acagaagaagttcagtggacagaattagcagaagcagaattggcagagaatagaattattctaaatcaagagcag
gaaggaagatattacagagaagatgaaccgctagaagcaacggttctaaagaatcaagacaatcagtggagttat
aagattcatcaaggtgacaggattctcaaggtagggaagtttgcaaaaataaaaaatacacatacaaatggaata
aggttactggctaatgtagttcaaaagataggaaaagaaagtctagtgatatggggaaagacaccatttttcac
cttccagtagaaagggaggtttgggatcaatggtggacagattattggcaagctacttggattccagagtgggac
ttcatctccacaccaccgttaatcaggttagtgtttaatttagtaaaagatccaatagaaaaggaggaagtatat
tacattgatggctcctgcaatagaaacagtaaagaagggaaagcaggatatgtcacagacaggggaaaagaaaag
gtcctgccattagaacaggctaccaatcagcaagcagagctgcaggccctgttattggccttgaaagattctcca
agtaaggtcaatgtagtaacggactcacagtatgtcttgaacataataacaggacagccatcagaatcggattca
gatatagtggcacaaattattgaacaactagttcagaaagaagcagtatacataggatgggttccagctcataaa
ggcataggagggaacaatgaggtagaccgtttagttagtcaaggaataagacaggtcctgttcctagaaagtata
gagccagcacaagaagaccatgacaaatatcatagcaatgtaaaggagttagctcagaaatataacatcccacaa
ttagtagctaaacagatagtaaatgcttgtaacaaatgccaacagaagggagaagcgatacatggtcagacaaat

gcagaggtaggcacatggcagatggactgcacccacttagaaggaaaggttatcatagtagcagtacatgtggct
agtggctttatagaagcagaggtaatacctagggaaacaggaagacagacagcattgttcctcttaaaaatagca
tcaagatggcctataaagcatctgcatacagacaacggtgccaatttcacctcccaggaggtgaagatggtagca
tggtggttaggagtggaacagtcctttggagtaccgtacaacccacaaagtcagggagtagtagaagccatggac
ttacatcttaagaaaaacatagataaaataagggaacaagcagagtcagtagagaccctagtactaatggcagca
cattgcatgaattttaaaagaaggggaggaataggggatatgactcctgcagaaagaatagtaaatatgatcacc
acagaactagaaacccaatacttaaattcacaaaattcaaaatttcaaaattttcgggtttattacagagaagga
agagaccaactgtggaagggaccagcagagctcctctggaaaggagaaggggctgttgtaattaaggtagggaca
gaaataaaagtggtaccaagaagaaaagcaaaaatcatcaaagatagaatagtggagcgctggcatagctgcatt
aaattccataagtataaaacaagagagctagagaaagcctgctatgtgccacatcacaaagtaggatgggcatgg
tacacagcctctagggtgatttccccttggaggagggaagtcacttggaggtacaagtgtactggaatctgacc
ccagaaaaaggatggttatcaagctatgcagttaggattacttggtactcagaaaaattttggacagatgtaacc
ccagatgtagcagatcagttagcacatagtacttatttcccttgctttgcagcgcacgcggtgagacaagccatc
agaggagagcaggtgttatcctactgtggctacgcggtagcccatcactctagtgtccaaagcctacagctgttg
gccctaaaggtagtattacaaatggaacatgctccagaggatgagaccaacccaagagaaccatgggatgagtgg
ataagagatgtactggaagaattgaaagaagaggcactgaaacattttgaccctcgcttgcttactgctttagga
aattacgtatatgatacttacggggatacaatagaaggggcaggggaaataattaagatattacaaaaggcattg
ttcttacactttaggatggcgtgtcctggacttcacctgcttatagacatcttgtttttaagtgtgttagggacc
tggtgtgcacagtatgtaacaatcttttatggtattcctgcatggaggaacgctacgatcccccctcttctgtgcg
acccagaatagagacacatgggggaccgttcagtgcttgccagataatggagactattcagaattggcccttaat
gttacagaggccttcgatgcttgggataatacagtaactgaacaagcaatagaagatgtttggaatctctttgaa
acatctattaaaccttgtgtaaagttaactccattatgtattgctatgaaatgtaataaaaatgagacagacaga
tggggggttgacaagagcagctactactactagctcaccaactactactagccccttaactgctgctagcccatca
ggagaggaaatcgttaacgacactatgtcttgtacaaagaacaacaattgctctggcatagagcaggaaccaatg
ataggttgtcaatttaacatgacaggactaaaaagagaccagaaaaggcagtacaatgaaacctggtactccaga
gatctagtctgtgagcaaggaggaaatgaaagcagtagatgttacatgaatcattgcaatacaagtgttatacaa
gagtcatgtgacaagcactattgggatgccatcagatataggtactgtgcaccaccaggctatgctttgcttaga
tgtaatgatacaaattattcaggctttgcacctaattgtagtaaggtagtagtatcatcatgtacaaggatgatg
gaaacacagacttctacatggtttggctttaatggtactagagcagagaatagaacatatatatattggcatggc
ggaagtaatagaacaataattagcttaaataagtattataatttgacaataaagtgcagaagaccaggaaataag
acagtcctaccagtcaccattatgtcaggtttggtctttcattcgcaaccaataaatgagaggccaagacaagca
tggtgctggtttggaggaaaatggagagaagctatgcaggaggttaagaaaaccatagtcaaacaccccaggtat
actggaaccaatgatactaggaaaattaatctaacggccccgggaggaggggatccggaagtcacattcatgtgg
acaaattgcagaggagaattcctttactgtaaaatgaattggttcctcaattgggtagaggataggaatacgagc
agcccaaggtggacaactcaaaccaaaaaggaacagcacaaaagaaattatgtaccttgccacatcaggcaaata
attaatacgtggcacagggtaggaaaaaatgtctatttgcctccaagggaaggagacttaacttgtaactccaca
gtgaccagcctaatagcaaatattgattggattgacaacaatgagaccaatattaccatgagtgcagaagtggca
gaattgtatcgattggaattggggggattataaattggtagagatcactccaattggcatggctcccacacatgtg
aaaaggtacaccacaagtacctcaaagaataaaaattcatggggatgtgcatttaggcaagtctgccacactact
gtactatggccaaatgacagtcttgtcccagactggaacaatatgacatggcaagaatgggagaaaaaggttgaa
ttcctagaggcaaatataactcaaatgttggaggaagcacgactacagcaagagaaaaacatgtatgaattacag
aaattgaatagctgggatgtgtttggaaattggtttgaccttacctcctgggtaagatacatacagtatggagtc
tttctagtcataggaatagtattgttaagaatagttatctatgtagtacaaatgttaagtaggttaaggcagggt
tataggcctgttttctcctcccccccatcttatcatcagcaggagcttgtcagaagagaagcaggctatatccgt
gatggatataaatacaatgtaggggaatttatgaacaccccatggagaaaccctgctacagaaagacaaaagcaa
cagtataggcagcagtgtatggatgatatagatgaggatgatgatgagctggctggagtagttgtctggccaaaa
gtacccctaagagcaatgtcatacaaattggcaatagacatgtcacattttataaaagaaaagggggactggat
gggatttattacagtgagaggagacatagaatcctagatatatacctagaaaaagaggaaggtatcatcccagat
tggcagaattatacaagtgggccaggaataaggtacccaaagttctttgggtggctctggcagctggtgccagta
gacgtctcggatgaggcaaacaatgatgagacccattgcttggtacatccagcccagacatatcagcattcagac
ccttggggaaaggtcctagcctggaagtttaatcctcacctagcctacacatatgaagcatttgtaaggcaccca
gaagagtttgggtggaagtcaggcttgacagaggaagaggttgagagaaggttagctaacaagccaaaaccgcag
aagaagatggcggataagaaggaaacaagctga
>#13 JX860414 7200 nt

atgggcgcgagaagctccgtcttgtcagggaaaaagacagatgaattagaaaaggttaggttacggcccaacgga
aagaaaaagtacatgttgaagcatgtagtgtgggcagcgaacgaattggatagatttggattggcagaaagcctg
ttggagaacaaagaaggttgtcaaaaaattctctcggtcttagctccattagtaccaacaggttcagaaaattta
aagagcctttataatactgtctgcgtcatttggtgcatccacgcagaacagaaagtgaaacatacagaggaagca
aaacaaatagtgcagagacatctagtggtggaaacaggaactgcagatacaatgccagccacaagtagaccaaca
gcaccacctagtggcagaggaggaaattaccctgtgcagcaaataggcggcaattatgtccacctacctttaagt
ccaagaacactgaatgcatgggtaaaattggtggaagagaagaaatttggagcggaggtagtgccaggattccag
gcactatcagaaggctgtaccccatatgatattaatcaaatgttaaattgtgtggggggatcatcaggcagccatg
caaattataagagaaatcataaatgaggaagcggctgattgggacctacaacacccacagccgatgggcccaata
ccagcagggcagctcagagacccgaggggatcagacattgcaggaactactagcacagtagaggagcagatagaa
tggatgtataggcagcaaaatcctataccagtaggtaacatctacagaagatggatccagcttgggctacaaaaa
tgtgttagaatgtataatccaacaaacattctagatgtaaagcagggaccaaaagaaccttttcaggcctatgta
gatagattctacaaaagcttaagggcagagcaaacagatccggcagtaaagaattggatgactcaaacgctgctg
attcaaaatgcaaacccagattgtaaagtgatcttaaaaggtctgggtatgaaccccaccctagaggaaatgatg
tcagcctgtcaaggagtgggtggaccaggacaaaaggctaggttaatggcagaggccttaaagagtgctttagca
ccaggagtacttccatttgcagcggcccaacagaaaggaggactgagacatacagtaaagtgctggaattgtgga
aaggagggacactctgcaaaacaatgcagagcccctaggagacaaggctgctggaaatgtggaaaaatgggccat
atcatggctaaatgtccagagagacaggtgacagcccatattgaaggtcagccagtagaagtattattagacaca
ggggctgacgactcaattgtagcaggaatagaattaggattaaattataccccaaaagtagtaggagggatagga
ggattcattaataccaaggaatacaaaaatgtagcaatagaagtattaggtaaaacaattaggggaacagtcatg
acggggggataccccaatcaacattttggcagaaatctgttaacaagattagggatgtctttaaattttccagta
gctagggtagaaccagtaaaagtgacactaaaaccagggcaggatggaccaaaattaagacagtggccattatct
aaagaaaaaatagaggcattgaaagagatctgtgaaaaaatggagaaggagggccagttggaggaagctcctcct
actaatccatacaatacgcccacctttgccataaagaaaaaggacaaaaataagtggagaatgctaatagacttc
agagaactaaatagggttacacaggatttcacagaagttcaactaggaataccacaccctgcaggactagcaaaa
agaagaagaattacagtactggatgtaggggatgcatacttttccataccactggacccagaattcagacaatat
actgcctttaccctaccatcagttaacaatgcagagccaggaaagagatacatctacaaggtcttaccacaagga
tggaaagggtcaccagcgattttccaacacacaatgagaaatgtattggagccttttagaaaggccaatccagat
ataaccttagttcagtacatggatgacatcttgatagctagtgataggacagacttggaacatgacagggtggtc
ctgcagttaaaagaactattgaatggcctagggttctccaccccagatgagaaatttcagaaagaccctccatat
cattggatgggatatgagttatggcctacaaaatggaagctgcaaaagatagagctaccacaaagagaaagctgg
acagtaaatgacattcagaagttagtaggagtactaaattgggcagcacaaatttatccagggataaagacaaaa
cacctctgcaagctaattagaggaaagatggcattgacagaagaagtccaatggacagaaatggcagaggcagag
tttgaggaaaatagaatcattcttaatcaagaacaagaagggtgttactaccaagaggataagcccttagaagca
accatagtgaaaagccaagataatcagtggtcttataagatccatcaagaaaataaaatactgaaagtaggtaaa
tatgcaaaaataaagaatacacatacaaatggagtcaggttattggcacatgtagtacaaaagataggaaaagaa
gcaatagtaatttggggaaagtgcctaaatttcatatgccagtagaaagagaaatttgggagcagtggtggaca
gactattggcaggtaacatggataccagaatgggatttcatatcaacacccccttttagttaggctagtgtttaat
ctggtaaaagaccctatagaaggggcagaaacattctatgttgatggttcttgtaataggcagacgaaggtagga
aaagcaggatacataacagacagaggaaaagataaggtaaaactcttagaacagactaccaatcaacaagcagaa
ttagaagcttttctcatggcagtaacagattcaggaccagaagcaaacattatagtagactctcagtatgtcatg
ggaatagtagcaggacaacctactgaatcagaaagcagggtagtaaaccaaataatagaggagatgatcaaaaag
acagcaatctatgtggcatgggtaccagcccataaaggcataggaggaaatcaggaaatagatcatttagttagt
caaggaatcagacaagtcctgtttctagagaaaatagagccagcacaagaagaacatgagaagtaccatagtaat
gtaaaagaactggtgttcaaatttagaatacccagattagtagcaaaacagataattgatacttgtgacaaatgc
caccaaaaaggggaggcaatacatggacaagtaaatgcagagttagggacttggcaaatggactgcacacactta
gaaggaaaggtaatcatagtagcagtgcatgtagctagtggattcatagaggcagaagtaataccacaggaaaca
ggaagacagacagcattgttcctgttgaaattggcaagcagatggcccatcacacatttgcatacagacaatggt
gccaatttcacctcgcaagaggtgaagatggtagcctggtgggcaggcatagaacagacctttggggtgccttat
aatccacagagccaaggggtagtagaagcaatgaatcatcacttaaaaactcaaatagacagaatcagagaacaa
gcaaactcagtagaaacaatagtactcatggcagttcattgcatgaattttaaaagaaggggaggaataggggat
atgaccccagcagaaagattaatcaatatgattaccacagaacaagaaatacaattccaacaatcaaaaaattca
aaatttaaaaattttcgggtctattacagagagggcagagaccaactgtggaaaggacccggtgagctattgtgg
aaaggggaaggagcagtcatcataaaggtagggacagacattaaagtagtaccaaggagaaaagcaaaaattatc
aaagacctcataaagcatttgaaatataacactaaagagctacagaaagcctgctatgtgccccatcataaggtt

ggatgggcttggtggacttgcagcagagtgatcttcccattagaaggggaatctcacttagaggtacaaggatat
tggaacttgacaccagagaagggatggctcagtacttatgcagtaagaatcacctggtacaccaggaggttctgg
tcagatgtaacaccagactatgcagacacgttactgcatggcacttatttcccttgttttacagcgggagaagtg
cgaagggccatcaggggagaacaattgctgtcttcctgcaggttcccgaaagctcataagaaccaggtaccaagc
ttacagtatctagcattaagaatagtaagcatggcagaaagacctccagaagatgaaggcccacagagagaacca
tgggatgagtgggtagtggaagttctggaagaattaaaagaagaagctctgaagcattttgatactcgcttgcta
actgcgcttggtaattatatctatgataggatgggatgtcttggcaatcagctgcttatcgccagcttgctacta
agtgcttgctcgatatattgcaagaaatatgtaactgttttctatggcattcccgcatggaggaacgcgacagtt
cccctcttctgtgcaaccaagaatagggatacttggggaacagtccaatgcttgccagataatgatgattactca
gaattagctctcaatgtcacagaggcctttgatgcttgggagaacacagtcacagaacaggcagtagaggatgta
tggaacctctttaagacttctataaaccctgtgttaaactaacaccactatgtatagctatgagatgcaatagg
acagagacaaatcgatggggactaacggcaactccgacacaacaacagtaacacctagcgccaaaactacaaca
gaaaaaggtgaaataaatgacactgacccctgcttcaaaggtaacaactgtacagggataggaagtgaagaattg
gtatcttgcaaattcaatatgacagggttaaagagagacaaaaagaaggagtacaatgagacctggtactcaagg
gatttggtctgtgaacgagacggaaatgaaatagcaaatgaaagtagatgctacatgaatcattgcaatacttct
gtaatccaggaatcttgtgacaagcattattgggatgctataagatttagatattgtgcaccacctggctatgct
ttgcttagatgtaatgatacaaattattcaggctttgcacctaattgcagtaaggtagtggtctcttcatgcaca
aggatgatggaaactcaaacctctacatggtttggatttaatggcactagagcagaaaatagaacatacatatat
tggcatgggagagacaataggactataattagtttaaataagtattataatctaacaataaaatgtagaagacca
ggaaacaagacagttatgcctgtgacaatcatgtcaggcttggtcttccattcccaaccaataaatgacaggcca
agacaagcctggtgttggtttggtggaaaatggaaatcagctatgcaggaggtgaaacaaacccttgtaaaacat
cccagatatactggaaccaatgatactaaaaacataaactttactgagccaggaaaaacctctgacccagaagta
gcttttatgtggacaaattgcagaggagaattcctctattgtaaaatgacttggttcctcaattgggttgaagac
agaaacatgtcttcacaaaacaaggcagaacaaaaaagacgtaactatgtgccttgccacattcggcagataatt
aacacttggcacaaggtagggaaaaatgtatatttgcctcctagggaaggagaactgacatgtaattcaacagta
accagtcttattgctaacatatattggattgatggaaacagaacaaatatcaccttcagtgcagaggtggcagaa
ctgtacagattggagttggggggattacaaattagtagaaattactccaattggctttgcacctaccgatgtaaaa
agatattcctctgtaacgacaccgaggaataaaaattcatggggatgtgcattcaggcaagtctgccacactact
gtaccgtgggtcaatgattcactccaaccagactggaacaatatgacttggcaagaatgggagaaacaggtagct
tacctagaggcaaatattacacagcaattggaagaagcacaaatactacaagaaaagaatatgtatgatttacaa
aaattaaatagttgggatgtttttggcaactggtttgacctcacgtcatgggtaaaatatgtctatttcggattc
tatgtagtagcaggagtaatagtattaaggatagtaatctatgtaatacagatgttaggtaagttaagaaagggc
tataggccagtgttttcctcccctcccacttatcgccagcagctgacctacctccaaggagagagttacagtcag
ggtcagtttatgaatacccccctggagaaacccagctagagagagagaaaaattggcatataggcaacaaaatata
gatgatatagatgatgataatgatgatttaataggagtaccagtacactcaaaagtccccttaagatgcatgact
tacaaattagcagtagacatgtcacattttataaaagaaaagggggactggaagggatttattacaatcagaga
agacatagaatactagacatttacttagaaaaggaggaaggaatcatccctgactggcagaattacacatcagga
ccaggaataagatacccaatgatgttcggatggctgtggaaactagtcccagtagaggtctcagatgaggcacag
aatgatgagacacattgtctggtgcatccagcacagacgggccaatgggatgacccgtggggaaaggtcttggca
tggaagtttgaccccactctggcttatacctatgaggcatatattaagtacccagaagattttggggacaagtca
ggcttgtcagaggaagaggttaagagaaggctaaccgcaagaggccttttaaaaatggctgacaagaaggaaact
>#14 HM803689 7230 nt
atgggtgcgagagcgtcggttttgtcagggaaaaaactagatgcctgggaacaaattcggcttcgtcccggtagc
aagaaaaagtatatgctgaaacatctagtgtgggcaagtagggagctagagagatttggcctaagtgatacgttg
ttagattcagcagtaggatgtcagaaattttaggagtcctgttgcctctagttccgacagggtcagaggggcta
aaaagcctctttaatttatgctgcgcactttggtgcgtacattcggaagtgaaagtgagagacacagaggaagct
atacaatatgtaaagaaacagtgccatctagaggacagagaagatagtgcagcagaaaaagaaaaaggtgccaca
gcgacatctagtggccaaaaaggaaattacccaataataacagtaaatcagcagccagagcaccagcccatttca
ccacggacacttaatgcttgggtgaaagtggtagaagagaaaaagtttggggctgaagtggtgcccatgttttcg
gcactttcagaaggatgcataccttatgatataaatcaaatgttaaatgcaataggagaacatcaaggagctttg
caaatagtgaaagaagtcatcaatgaggaagcagcagattgggatgctagacatcctaatctaggaccactacct
gcagggcagcttagagagcctacaggaagcgatattgcaggaacaacaagcagtgttgcagaacagatagcttgg
tcaacaagagctaacaacccaataatagtagggaacatttatagaaattggattattctggggttgcaaaaatgt
gtaaggatgtataacccagtaaatgttctggatattaaacagggaccaaaagagccattcaaggactatgtagat
aggttctataaagccttgagagcagagcaaacagatccagcagtaaaaaattggatgactcagacactgctgatc

cagaatgccaatcctgactgtaaaacagttctaaaggtctaggcatgaacccctccttggaagaaatgttgcta
gcatgccaaggagttggaggaccccaacacaaggctagagtattggcagaagccatgcagcatgctaatagcatg
atgatggctcaaatgagagggcaaggaaggtcagggccaccaagaagaggtggagaaagaccagtaccaaaatgt
tacaattgtggaaaaacaggacatatttccaggttttgtaaggcccctagaagaaaaggatgctggaagtgtgga
gatgaggatcatcttatgaaggattgtaccaagcaggttatggtcaatatagaagggcaaaaggtagaagccctc
ttagatacaggggcagatgacacagtaattaatgggatagaattaggagaaaattggaagccaaaaattataggg
ggtattggtggatatataaatgtgaaacaatattacaattgtaagatagaaatagcagggaaagtaacacatgcc
catgtattggtagggccaacacctgtaaatattattggaagaaatgttttacacaaattaggagcctctttaaat
tttccaattagcaaggcagaaacaattgaagtgaaattgaaaccaggacaagatgggcctaaagtaaaacaatgg
cctttaagcaaagaaaagatagaagcgttaacagaaatttgtaatgctatggaaaaagaaggaaaaatatccaaa
atagggccagaaaatccttacaatacgcctatttttgcattaggaaaaaggattctacaaaatggagaaaattg
gtagatttcagagaattaaataagagaactcaggacttctttgaggtacagctgggaataccacatcctggggt
ttgaaaaaatgtaaacagataacagttttggacatagggggatgcttatttttcatgtcctctatatgaaccttt
agaaaatatactgcattcacaataccagcaatcaataatcagggaccaggagttaggtatcaatacaatgtgctc
ccacagggatggaagggatctccagctatatttcaaacaactgcaaatagaatcttagagccattcagaaaagaa
aatccagatgtagtaatctatcaatacatggatgatttatttgtaggatctgataggacagaattagaacataat
cagatgataaaaagactcagagaacatttgttgatctggggtttgaaaccccagataaaaaatttcaggacaaa
cctccctttgaatggatgggtatgtactccatccagacaaatggacagtacaagaaattaaattacctgagaaa
gaggaatggacagtaaatgatattcagaaattagtgggaaaattaaactgggccagtcaaatctatacaggaata
aagacaaaagaattatgtaaattaattagaggagtaaaaaggctagatgaaaaggtagaattcaccagagaagca
gaactagaatatgaggaaaacaagctattgctgaaagaacaaatgcatggagtctactatgatccagaaaaacct
ctgatagctaaagtacaaaagttaacaggagggcagtggtcttatcaggtagaacaaggagatggtaaaccatta
aagacagggaaatatgccaagcaaaagactgcacacaccaatgagatcaggatgctagcaggattggtacagaaa
atatgtaaagaagcagtagtcatttggggaaaattgcccaaatttgagctgccaatagagagagaggtatgggaa
caatggtgggctgattattggcaggtatcatggattcctgagtgggaattcgtgagcacgcccccattaattagg
ttatggtataatctggtaaaagatcccataccaggggaagaagtctattatgtagacggggcagctaacagaaat
agcaaacagggaaaagcaggatacattacagacagaggaagagaaaaggtaaaagaattagaagataccactaat
caaaaggcagaattggaggcagtcttattagcattaaaagattcagggaaaaaggtaaatatagtaacagattca
cagtatgtgtttggcatattggcaggaagtccagacactagtgatagtcctcttgtacagcagatcatagaacaa
ttaatagggaaagaggaagtgtacctctcctgggtacctgcacataagggcataggaggaaatgaggaagtagac
aaattagtaagtaaaggaattagacaagtcctcttttagatggaatagaaaaggctcaagaagagcatgaaaaa
tatcataataattggagagcaatggcagaagattttcagataccacaagtagtagcaaaggagatagtagcccaa
tgccctaaatgtcaggtgaaaggagaagcaatgcatggacaggtagatgccagtccaaaaacatggcagatggac
tgcactcatttagaaggaaaggtcatcatagtagcagtccatgtagccagtgggtatatagaagcagaagtttta
ccagcagaaacaggaaaagaaacagctcacttcctgctaaagctagcagccagatggccagtaaaacatttgcac
acagacaatggagcaaattttacgagctcagcagtacaagcagtttgttggtgggctcagatagagcacaccttc
ggggtaccctacaacccacaaagtcaaggagtagtagagtccatgaatcaccagctaaaaactataataacacaa
ataagagatcaagcagaaaagatagagacagcagtacaaatggcagtgctcattcacaattttaaaagaaaaggg
gggattggggggtacagtgcaggagaaagaataatagatataatagcatcagacttacaaacaactaaattacaa
aatcaaatttcaaaaattcaaaattttcgggtttatttcagagaaggaagggatcaacagtggaaaggacctgcc
actctaatctggaaaggagaaggagcggtagtaatccaggacggacaagatttaaaggtagtcccaagaagaaag
tgtaaaatcataaaagatcagtggcactccctagtaaaatatcatatgtataaaggaaagaaagaagcaagagaa
tgggaatatgtaccccatttcaaagtaccttggggggtggtctcattcagaggtccacattcccttggaaat
aacactaagataaaggtgaccacctattggaatctaacaacagaaaaggggtggttgggaacttatggggcagct
cttgcttatatagaccagaaatgtgaccctccctacttcacagatatagacccaatagtggcagacagcttaata
cataagatatatttcccctgttttacagataaggcaattagacaagctatattgggagaaaaggttttgctgtgt
ggattccagaggggacatagagatcaggtagggacactccaatacctagcaatacaagcttgggctagagagcaa
gtaaagaaaatggagatgcccccggaagatgaaggccctcagagagagccaatgaatgaatggctactagacacg
cttatggagctacaggaagaagcagctaagcatttacaatggagcttttacatgcagtagggaattacatatat
gagcaatatggggatagcttagagggagttagagtactaattactttgcttcaaaggctctgttcttgcactat
agaaaatgttattgcaagaaatgtgcatatcactgccagctttgctttcttcaaaaggcattaggcattaacatg
aagtgcctaagcttaatagtgttaagtagtctgattcttttagctttagcaaaacagaagccacaatatgtgaca
gtattttatggagtaccagtatgggtcaacagtacagtacctatgttttgtgttactgacaacacaaactcatgg
ggtactttaaattgcatcccagaaggaggaatatcaccagaagtaccagttaatgtgtcagaaaaatttgatgct
tgggaaaatgccctgtatgaacaagcaaaagataatgtgtggaatctttatgattctaccctaaaaccttgtgtt

aggttatcccctttatgtgtaacaatgaattgtactgtaataaatgggagttgggatggtataacaacaccagct
ccatcaccagcacctacaacaccaacagcagcaaaaactacaacaaaagtagattgccctatgaataatgaaaca
tgtgcagcagttccagatgaggatgttatgaattgtgagtttgcagtggcaggattaaaaagggatgaaaaatat
aaagctaatgacacgtggtactcaagagatctttggtgtgagaaagataaaaataataataagactactaagagg
caatgttttatgcgacattgtaatactactagtattcagcaattctgtgagcctaaatactgggaacctttttagg
attagatattgcgctccaccaggatttgccctattggtatgcaaagataaaaattatacaggatttgatacctgt
cataatgtgacagccacttcatgcacagatatgataaatacaacagtatcaacctcatttggactaaatggatca
attagtgaaaacagaacttggatttatcagagaaagcaatcgaatcggacagtaatagggttaaatagagagtat
aatctaacagtggaatgtagaaggccatcaaataggacagtgaagggaatctcattggctacaggagtctttatc
tccttaagagtagagaaacgaccaaaaggagcttggtgtagattttatggcaattggacaggtgcttgggaagaa
gttaaaaaagaggtaataaaaacgaagggatataaagggacaaatgatactaagaaaatatacataagatcacat
tatggaggggatgatgaagctaaatacttttggttaaattgtgatggggaattcttatattgcaaattgaattgg
tttctcaacctattaaataatagaactgatggaaacataaatgagcgaagacaagccatgtttgtgccttgtatc
acaaaaatggtggttaatgattggtatacagtgtcaaaaaaggtatacacaccaccaagacctgatgcattgaaa
tgcagggccacggtcacttacttgttggcagacatagattatatacataccaatgaaacaaatgttactctgact
gcagaggtaggagatctatgggcagcagagttaggaagatacaaagtagtagaaattaaaccaattggctatgca
cctacagatgttaaaaggtacgagacaagacagaaaaatacatttggctgtgcatggaggcaagtctgtcataca
acagtgcagtggactttcaatgcaacacctgaatggaataaacagacatggcttgaatgggaaaggaatatatca
ataatagaagggaatatctctttagcattacaggaagcccaggatcaacatgagagaaatgtgcatgaactggag
aaattaaataattggggagatgcctttagttggctgaggcttgactggtggatggaatatctaaaaataggagtt
tttataattttaggagttataggactaagaatttttctttcttctatggggatgtatcagtaagattagagcaggg
tatactcctttgttgtcgcccccaccttattatcatcagcagatacaaggaggaataacaatagaaacacagcaa
agtgtagatgctatagattggtatgaggagacagatgataccttggtaggtttcccagtgaaaccacaagtacca
ttaagacctatgacttttaaattggcaatagatctctcacatttttttaaaagaaaaggggggactggaagggatt
tattactccaccaggagacatagaatcttagatatatacttagaaaatgagcatggaattatccctgattggcag
aattacacaaaaggaccaggtattagatacccaatgatgtttggatggctgtggaaattagtaccagtagatgtg
attgatgaggctaaggaagatgaagagcatagcctgctacacccagctgagacttgtgggatggaagacccctgg
ggagaggtcttagcttggaagtttgatcctatgctagcaatagattatgtgggctataaactgcacccagaggac
tttggagagaagaagaacaagacccaataa
>#15 HM803690 7203 nt
atgggtgcgagagcgtcaattttgacagggaaaaagctagatgcctgggagcagattcggcttcgccccgggagt
aagaaaaaatatatgttaaaacatctagcatgggcatgtagggaactagagagatttggcttaagtgatacattg
ttagattcagcaggggatgtgaaaaaatcttaggagtccttttgcctctggttccgacagggtcagaggggctg
aaaagcctctttaatttgtgctgcgcactttggtgcgttcacaaggaagtgaaagtgaaagacacagaagaagca
gtgcagtatgttaagaaacagtgccatctagtggagaaagaggatactgcaggagaaaaagaaaaaggtgccaca
atgacatctagtggtcaaagaggaaattaccccatagtaacaattaatcagcagccagaacaccagcctatttct
cccaggacgttgaatgcctgggtgaaggtggttgaggaaaagaagtttggagcagaagtagtgcccatgttttcg
gcactttcagagggctgcataccttatgatgtaaatcaaatgctaaatgccataggagaacatcaaggagcatta
caaatagtgaaagaagtaataaatgatgaagcagcagactgggatcttaggcatcccaatccagggccactacca
gcaggacagctaagagagccgacaggtagtgatattgcaggaaccaccagcaatattgctgaacagatagcctgg
acaaccagagcaaataacccaatagcagtaggaaacatttatagaaattggatagtactagggcttcaaaagtgt
gtaaaaatgtataacccagtaaacatcttggatataaagcagggaccaaaagaacctttaaagattatgtggat
aggttttataaagcacttagagcagaacaggcagatccagcagtaaaaaattggatgactcaatcacttttgatt
cagaatgcaaacccagactgcaagacagtgctgaaggggctaggaatgaatcccactctagaagaaatgctaaca
gcctgtcaaggagtaggaggaccacagtataaagccagggtcctagcagaagccatgcaacaagcgaatagcgct
atcatggctcaaatgagaggtgggcaaagatcaggcccgccaagaggacaaggaggaaagacattcttgaaatgt
tacaactgtggaaagttggggcacatttcaagatattgtaaagcccccagaagaaaaggatgctggaagtgtgga
gaggaggatcacatccttaaggattgccctaagcaggtatgggtcaccatagagggtcaaaaagtagaagccctt
ttagacacaggtgctgatgatacagtcatcagtgacatagaattaggagataattggaagccaaaaattatagga
ggaataggggatatataaatgtaaaacagtattataattgtaaaatagaaatagcagggaaagttacacatgct
catgtattagtaggtccaacaccggtaaatataattgggagaaatgtgttacacaaattaggagcttcattaaat
tttccaataagtaaagcagaaataattaaagtagagttaaaggaaggtcaggatggaccaagagtaaagcagtgg
cccctcagtaaagaaaaaatagaagccttaacagaaatatgcaatgcaatggaaaaggagggaaaaattagtaga
ataggaccagagaatccatataatactccaatattttgtattagaaagaaagattcgaccaaatggagaaagcta
gtagatttcagagaactaaataagaggacacaggatttctttgaggtacagctggggattccacatccaggagga

ttgaagaaatgcaagcagattactgtgctggacataggggacgcatatttttcctgccccctctatgagcccttc
agaaagtatacagcttttactataccagcaataaataatcaagggccaggtatcagatatcaatacaatgtgctt
cctcagggatggaagggctccccagccatatttcaaaccactgctaacaaaattttagaaccagtcaggaaacaa
aatccagatttagtcatctatcagtatatggatgacctgtttgtaggatcagacagaacagaattagaacacagt
caaatgataaagaaattgagggaacatttgctcgcctggggatttgagacaccagaaaagaaatttcaagataaa
ccaccatttgaatggatggggtatgtactccacccagacaagtggacagtgcaggaaattaagttgccagaaaag
gaagaatggacagtgaatgacatacagaagttggtaggcaaattgaactgggcaagtcagatctatgcaggaatc
aaaactaaagaattgtgcaaattaattagaggaataaaaagattagatgataaggtagaatttacaaaagaagca
gaattagagtatgaagaaaataaactgctattaaaagaaaaattgcatggggtatattatgacccagaaaaacca
ttgatagcaaaagtacagaaattagaaggaggacagtggtcatatcaggtagaacaaggagatggaaaacctcta
aaaacaggaaaatatgccaagcagaagacagcacataccaatgagattagaatgctggcagggttagttcagaaa
atttgtaaagaagcaatagttatttggggaaaattgcctaaatttgaattgcctgtagaaagagaggtatgggaa
caatggtggctgattattggcaagtctcatggattccagaatgggaatttgtgagcacgccgcctttaattagg
ctgtggtataacctagtaaaggatcccataccaggagaggaggtgtattatgtggatggggcagcaaacagaaat
agtaaattaggaaaagcaggatacataacagatagaggaaaagaaaaagtaaaagaattagaagataccaccaat
cagaaggcagaattagaggctgtacttttagctttaaaagattcaggaaaaagagtaaatatagtaacagactct
caatatgtgtttggcatattagcaggaagtccagacactagtgagagtcccttagtacagcagataatagagcaa
ctaatagggaaagaagaagtgtacctctcctgggtaccggcacataagggcataggaggaaatgaagaggtagat
aaattagtgagcagaggaattagacaagtattattccttgatggcatagaaaaagcacaagaagaacatgataaa
tatcatagcaattggagagctatggcagaagatttcagaatacctcaagtagtagctaaggaaatagtggcccaa
tgtcctaagtgccaggtgaaaggggaggcagtccatgggcaaacagatgctagtccagggacatggcaaatggat
tgtactcatttggaaggaaaggtaatcatagtggcagtccatgtagccagtggatatatagaagcagaagtcata
ccagcagaaacaggaaaagagacagcatacttcctgttaaaattagcagcaagatggccagtgaaacacttacat
acagataatggagctaatttcacaagtgcagcagtacaggcagtctgttggtgggctcagatagagcacaccttt
ggagtaccctacaatcctcaaagtcagggagtggttgaaagtatgaatcatcaattgaagacaataatagggcaa
attagagaccaagcagagaaattagaaacagcagttcaaatggcagtgctcattcacaattttaaaagaaaaggg
gggattgggggtacagtgcaggagaaagaataatagacataatagcatcagagttacaaacaaataaattacaa
aatcaaatttcaaaaattcaaaattttcgggtttattttagagaaggcagggatcagcagtggaagggaccggcc
actctcatctggaaaggagaaggagcagtggtgatccaggatggacaggatctgaaggtagtccctagaagaaag
tgtaaaattgtaaaagatcaatggcactctctagtaaaataccatcagtacaaaggaaaaaaggaagcaaaggaa
tgggaatatgtgccccattttaaagtaccttggggatggtggtctcattcagaaatccacgttcccttgggaaaa
aacactaaggttaaaattaccacttattggaatctcacagcagaaaagggatggttaagtacttatggagtaggg
atagcctatatagataacacttgtgacccttgctactttacagatatagatcctattctggctgacaagatgata
cataatgtctattttccctgttttacagatcaggcaattaggaaagctcttttaggagagaaagtgttagtgtgt
ggctttcagcgaggacacagagatcaggtagggactcttcagtaccttgctttacaagcttgggttaaatcccag
ttacagaaaatggaaattccccaggaggatgaaggccctcaaagggaaccatatgataaatggcttttagacacg
cttatggagctacaagaagaggcagctaagcattttaccatggagcttttgcaggcagtaggaaattacagttat
gagcagcatggagaaagtatagaaggggttaagaaaatgataactttgttgaatagagctttgtttttgcactat
agagcttgctattgtaagaagtgtgcttaccactgtcagctttgctttcttcaaaaggcaataggcatacaaatg
aaaagtgcaataggaatagtgataatagctagtctaataagtgctatagtaagtaaacaatgggtaacagtattc
tatggggttccggtatggaaaaatagtacagcaccaatgttttgtgttacagataatgcaaattcatggggcact
ctaaattgtataccagaaggggggactgtcaccagaggtacctgtaaatgtctcagagcagtttgatgcatgggaa
aacagtttgtatgaacaggccaaggacaatgtgtggcatctatatgattccaccctgaagccttgtgttagacta
tcacccttatgtataaccatgaactgcacagcattaaatggaagctgggatggatcagtaacaacaacaacacaa
ccaagaacaacaccatccgcaacagcatcaacaaatatagactgtggaataaacaatgaaacatgtagtgcagta
gaggatgaaaatgttatgcagtgtacatttgcagtagcagggttgaaaagagatgaaaagcataaatacaatgat
acttggtattcaagagacctctggtgtcagaaagctaacaatgtgacccaatgttttatgagacactgtaacaca
actagcatacaccagttttgtgaaccaagatactgggaaccctttaggttaagatactgtgctccaccaggcttt
gccttgctaaaatgcaatgattataattacacgggttttgatacttgtcataatgtaacagctacttcatgtaca
gacatgataaacacaacagtgtctactagttttggattaaatggatcaataagtgaaaatagaacttggatctat
caaagaaaacagagtaacagaacaatcatagggttgaatagtttctataacttgacagtaagttgtagaagacca
tcaaataggacaataaagggaatttccttagccacaggagtatttatatctctaaaagtagaaaaaaggccaaaa
ggagcttggtgcagattttatggaaattggaaaggagcttggaaagaggtaaaagagaaggtcatacaaacaaag
ggatataagggaacaaatgacacaactaaaataaacataaggtcagtttatggaggagatgatgagactaaatat
ttttggctaaattgcgatggagaattcctctattgtaaactcaattggtttttgaattatttgaacaataagaca

gagggtaatgagaaggaaagaaggcaagccatgtttgttccttgcgtcacgaaaatgattgttaatgattggtac
acggtgtcaaggaaagtttatacaccgccaaggcaagatgcgttaacatgtaatgctacagtaagctatttgtta
gcagacatagagtatgtgaatcaaaatgagactaatgtgacactgtctgcagaagtaggagacctatgggcagca
gaattgggaagatacaaagtagtggaaattaaaccaattggatatgcaccaacagctataaaaaggtacgagaca
aaacagaaaaatacatttggttgtgcctggagacaagtgtgtcacacctcagtgccttggagctttaataagaca
ccggactggcagaaacagacatggcttgaatgggaaaggaatgtctcatatttagaagcaaacataacagcctca
ttacaagaagcacaagatcagcatgagaaaaatgtacatgaactagaaaagttgaacaattggggagatgcattt
agctggctaaggctggactggtggatggagtatataaaaataggaatctttgttatactaggcattatagggtta
agaatagtcttcttgctatggagctcygttagtaagcttagagcaggatatactcctttgctctctcccccaccc
tattatcattcacaggtgcaaggagggtttactatagaaacacagcagtcagtagatgctatggattggtatgag
gagacagatgacaccttggtaggatttccagtcaagccacaagtaccattaagagcaatgagtttcaaattggca
atagacatgtcccactttttaaaagaaaagggggactggaagggatttattattcaataagaagacatagaatt
ctagatatatacttagaaaatgagcatggaataattcctgattggcagaattacactccaggacctggtcccagg
tacccgaccttcttcggatggttgtggatgctggtaccagtggatgtgagtgatgaggcaaaagaggatgaggaa
catagtctcctgcatccagcagaaagtagtgggattgaggacccttggggagaaacattggcttggaagtttaat
cctatgttagcagtagattacataggctatagactgcaccctgacttctttggagagagaaagaacaagacccag
taa
>#16 M58410 7014 nt
atgggcgggggtcactcagcactgtcagggagaagcctcgacacgttcgagaagattaggctacgtccgaacggg
aaaaagaagtaccaaattaaacatttaatatgggcaggaaaagaaatggaacgatttgggttacatgagaaactt
ttagaaacaaaagaaggctgtcaaaaaatcatagaagttttaaccccgttggaaccgacaggctccgagggggcta
aaagctctgtttaatttgtgctgcgtcatttggtgcattcacgcagaacagaaagtgaaagacacagaggaagct
gtagtaacagttaagcaacactaccatctagtggacaaaaatgagaaagcagctaaaaagaaaaatgagacaaca
gcgccacctggtggcgaatcaagaaattacccagtagtaaatcagaataatgcctgggtacaccagcctttgtct
ccgcgcacgttaaatgcgtgggtcaaatgcgtggaggaaaaaaggtggggagcagaagtagtccccatgttccaa
gcactctcagagggatgtctctcctatgatgtaaatcagatgctcaatgtaataggagaccatcagggggcatta
caaattcttaaggaagtcattaatgaagaagcagcagagtgggacaggacacacagaccaccagctggcccgtta
ccagcagggcagctaagagacccgacagggtcagatatagcaggaactaccagctcaattcaggaacaaatagag
tggaccttcaatgccaatccaagaatagacgtaggggcacaatacagaaaatgggttattttgggcttacaaaag
gtagtgcagatgtacaatccccaaaaggtcctagacattcgacagggacctaaagaacccttccaggactatgta
gacagattctataaagccctgagagcagaacaagcaccacaggatgttaaaaattggatgacacaaactttgctt
atccagaatgccaatccggattgtaaattgattctgaaaggattgggaatgaatccaaccttggaggaaatgcta
atagcttgccagggagtaggagggccacaacataaggctaagctaatggtagaaatgatgagtaatggacagaat
atggtccaagtgggacctcagaaaaagggcccccgagggccgctaaaatgctttaattgtggcaaatttggacat
atgcaaagggaatgcaaggcaccaagacagatcaaatgctttcgagtcatcatagaaggaacgccagtgcaagcc
ttgttagatacaggagcagatgacactataattcaagaaaaggacttgcactttcccccacataaaccatggcgt
tccaaggtagtaggaggtataggaggagggattcatgtcaaagaatatcagggggtacaagtacaattggaggat
aaaatcatcaccggctcaattctaataggaagtacaccaatcaatattataggaagaaatatttagctcaggca
ggcatgaaattagttatgggagttctatctagtcagattgaggaaacaaaagtacaactaaaagaagggaaagat
ggacctaaattgaaacaatggcccttatcaagagaaaaaattgaagctttaacagaaatatgcaaacaaatggaa
gaggagggaaaattatctaggataggaggagaaaatccttataatacaccagtgtttgccataaagaaaaaggat
aaaacacaatggagaatgcttgtagatttcagggaactaaacaaagctactcaagactttttgaggttcagctg
ggaattcctcacccagcgggccttcagaaaaagaagcaaatcacagtaatagacatagggggatgcctattattca
ataccattatgcaaggaattcagaaaatatacagcatttaccatcccctcagtaaataatacagggccaggata
aggtatcagttcaattgtctgcctcagggatggaaaggatctcctacaattttccagaatacggcagcaaacatt
ttagaggagatcaaaaggcacactcctgggttagaaattgtccaatacatggacgatttgtggttggcgtcagac
catgatgagactagacataatcaacaggtagacatagtaagaaagatgctgctagaaaaaggtctagaaacccca
gacaagaaagtccaaagagaaccgccatgggaatggatggggtataaattgcatccgaataaatggaccattaac
aaaatagaattacccccttagaaggagaatggacagtaaacaaaatacagaaggtagtaggagttctaaattgg
gcaagtcaaatttatccaggaattaaaaccaaacatacctgtgccatgttgagagggaaaaagaacctcctagaa
gaaatagtatggacagaagaggcagaggcagaatataagaacaatcaagggatagtgcaggaaacacaagaagga
acatactatgaccctctcaaagaattaatagcaacagttcaaaagcaaggagaagggcaatggacataccaattc
acccaagaaggggcagtattaaaggtgggaagatatgccaagcaaagagaaactcatactaatgatctaaggact
ctagcacaccttgtccaaaaaatctgtaaggaagcacttaccatttggggaagacttccacgagtacaactccca
gtagacaagaaaacatgggatatgtggtggcaggactattggcaagtatcctggataccagaatgggagtttgtt

agcacaccactcctagtaaaactgtggtattccttagtaaaagaaccaatcaaaggagaagatgtttattatgtg
gatggggcagcatccaaagtgaccaaattaggtaaggcaggatatctgtcagagagaggaaaaagtagaattagg
gaattagaaaacaccactaaccaacaagcagaattaacagcagttaagatggcattggaggacagtggagaaaat
gtaaatatagtcacagattctcaatatgtaatgaacatcttgacagcatgtccacaggaaagtaactcaccctta
gtggaacagataatacaagccctaatgaaaaagaggcaggtctacttacaatgggtaccagctcataaggggata
ggaggcaatacagaaatagataaattagtaagcaaaggaataagacagatcctcttcttagatagaatagaagaa
gcacaagatgaccatgcaaagtaccataacaattggagaagtatggtacaggaatttggattacctaatatagta
gcaaaagagatagtagcggcatgtcccaaatgccaaataagaggagaacctaagcatggacaggtagacgcctcc
attgaaacttggcagatggactgcacccatttagaaggaaaagttataatagtagcagtacatgtagccagtgga
ttcatagaagcagaggtgatcccaagagaaactgggaaggagacagcacactttctgctgaaactgttagcaaga
tggccagtgaaacatctacacactgataatggcccaaactttacctctcagaatgtggcagcggtgtgctggtgg
ggtaatatagagcacaccactggaataccttataacccacagtcacagggtagtgtagaaagcatgaacagacag
ctcaaggaaatcatctctcaaataagagatgattgtgagagattggagacagcagtgcaaatggctacgcatatc
cacaattttaaaagaaagggaggaatagggggtatctctagtgcagaaagattggttaatatgctaacaacacaa
ctagaactaaatactctacaaaaccaaatccaaaaaattttgaattttaaggtctactacagagaaggtagagat
ccagtgtggaaaggaccagcgcgactcatctggaaaggagaaggcgcggtggtaattaaagaggggaagacatc
aaggtagtccccaggagaaaggctaagattatcaaagatcaattgccttgggaatacagacatcattggcaggtg
caatggcagttttggacctacagccagttcattatccccttatcaaaagatgattacatagaagtgaatatttat
cacaacctcaccccagaaagaggatggctctcaagtcatggagtagggttatcctattaccatcaaaaggatat
aagacagaagtagatccaggaacagcagacagaatgatacacctatattattttaactgttttacagatagagcc
atccaacaggctatcagaggggagaagtatacgtggtgcacattcaaggaaggacataaaggtcaggtacaatca
ctgcaacttttggcactagttgcatatacaaccccatggaatgagataggctacaagtactatagaattgttcaa
aagtctatgtttgtacatttcagatgtggttgtagaaggagaggacctttttccccttacgaagagaggagacca
ttacaaccatgtactaataaatgctattgcaaaaaatgctgttatcactgtgagctttgcttcctgcagaagggc
ttaggagtgcgttatatggggagattgcttataaaaatactaataatagcaatagggataagtataggaataggt
aacctgtatgtgacagtgttttatggaatcccagtatggaaaaattcaacagttcaggcattttgcatgacgccc
aataccaatatgtgggcaaccaccaactgcataccagatgatcatgataatacagaggtgcctctaaacattaca
gaagctttcgaggcttgggataatccgctggtaaaacaagcagagagtaatatacatctactctttgaacaaacg
atgaggccttgtgttaagctctcccccatatgtattaaaatgtcctgtgtagagctgaatggtacagccacgaca
aaggccaccactactgcaactacaacaatgactacccccctgtcagaattgcagtacagagcagatagaaggagaa
atggcagaggaaccagcatccaactgcacttttgcaattgcaggatatcaaagagatgtaaaaaagaattatagc
atgacctggtatgatcaggagttagtctgcaataataaaacaggaagtgaaaagggaagtaaggattgttacatg
atacattgtaatgattcagtgataaaagaagcttgtgataaaacatattgggatactttaagagtaagatactgt
gcaccagcagggtatgctttgctaaaatgtaatgataaggattatagaggctttgctccaaagtgcaagaatgtt
tcagtagtgcattgtactagattaatcaatactactataactacagggataggattaaatggtagtagatcagaa
aatagaacagagatatggcagaaaggaggaaatgataatgatacagttataataaagttgaataagttttacaac
ttgacagtgagatgccgaagacctggtaataaaacagtgttgccagtaacaatcatggcagggttagtatttcac
tctcagaaatataataccaggttaaaacaagcgtggtgccacttccaaggagattggaaaggggcatggaaagaa
gtcagagaagaagtaaagaaagtgaaaaatcttacagaagtaagcatagaaaatatacatctgagaaggatatgg
ggagatccagaatcagcgaatttttggttcaattgtcaaggtgaatttttctattgtaagatggactggtttatc
aattatctaaacaatcgaacagaagatgcagaaggtactaataggacctgtgacaaaggaagccaggaccagga
ccatgtgttcagagaacttatgttgcctgccatatacgacaagtagtaaatgattggtacactgtctctaaaaag
gtatatgctccaccaagggaaggtcatttggagtgtaactcatcagtcacggcactatacgtggcaatagattat
aacaacaagtctggcccaataaatgtgaccctaagtcctcaggtacgcagcatatgggcgtacgaactgggagac
tataaattagtagagataacaccaattggctttgctcctacagatgtaagaagatatactggccccacaagagaa
aaaaattcatggggatgtgcgtggaaacaagtatgtcacaccacagtgccatggaagtataataacactcctaag
tgggacaatatgacttggttggagtgggagagacaaattaatgccttggaaggcaacataactcaactattggaa
gaagcacaaaatcaggaatcaaagaatctggatctgtaccagaaattggatgattggtcagggttctggtcatgg
ttctcactgtcaacttggttaggctatgttaaaataggattttagtgatagtgattattctaggattaagattt
gcatgggtattatggggatgtatcagaaatattaggcagggatataatcctctcccccagggtaaaatgactcca
gacggccgccgcctgcaagaaggagacacctttgatgagtgggatgatgatgaagaagaagtaggcttccctgtg
caacctcgagtccccttaagacagatgacctataaattagcagtggacttttcccactttttaaaatcaaagggg
ggactggatgggatatattactctgaaagaagagaaaagatcctgaatttgtatgccttgaacgagtggggaata
atagatgattggcaagcttactcaccaggcccggggataaggtacccgagagtctttggcttctgctttaagcta
gtcccagtggacctgcatgaggaggcacgcaactgtgagagacactgtctgatgcatccagcacagatgggggaa

gatcctgatggaatagatcatggagaagtcttggtctggaagtttgacccgaagttggcggtggagtaccgcccg
gacatgtttaaggacatgcacgaacatgcaaagcgctag
>#17 LC114462 7218 nt
atgggtgcgggtacctcagcattgtcagggagaaaattagatgaatttgagaaaatacgacttcgcccgaacggt
aagaaaaaataccgattaaaacatataatatgggcaggcaaagaaatggagcgcttcggcctccatgagaaactc
ttagaaacagaggaaggatgtaaaaggataatagaagttctattacctttagaaccaacaggatcagaaggtttg
aaaagcttgtttaatctcgtgtgcgtgctgtattgcattcaccgagaacagaaagtgaaagacacagaggaagct
gtagtaacagtaaaacaacactgccacctagaggaaaaacaaaaaagtgcagctgcgccacctggtggcgaaaag
aaaaattacaacagtagagagacatctggtggccaaagtcagaactacccagcgcaacaacagggaaatgcttgg
gtacatgtgccgctctcccccaggacgttaaatgcctgggtgaaagcagtagaagagaaaaaatttggggcagaa
atagtgcccatgtttcaagccctctcggagggatgcaccccttatgacatcaatcaaatgctaaatgttttagga
gaccatcagggggcactacagatagtaaaagaaatcatcaatgaggaagcagcaaattgggacttacaacatccc
ccgccggctggaccccctaccagcagggcaattgagagaccctaggggatcagacatagcaggtaccaccagcaat
gttcaagaacagctggagtggatctataccgccaaccctagagtagacgtaggggccatttataggagatggatc
atcttggggttacaaaaaatgtgttaaaatgtacaacccagtctcagtgctagacatcagacaagggcctaaagag
cccttcaaagattatgtagatagattctacaaagctataagagcagaacaaacatcaggagaagtaaaacaatgg
atgacagagagtctgctcatccaaaacgccaacccagaatgtaaagtaattctaaagggtctgggaatgcaccca
acattggaagaaatgctcacagcctgtcaaggggtaggaggaccccagtacaaagccaaattgatggtggaaatg
atgcagcaaatgcagggacaaaacatggtgcagcaaggggggcccaagaggaccaccgcgaggaaaccctcgctgc
ttcaattgtggaaaatttggccatttacaaagaaattgcgcagagccaagaaaagatgaaatgctttaaatgtgga
aaaataggacacatggccaaggactgcaaaggacaggttattgttagaatagagggtcagccaatcaaagtcttg
ttagatacaggagcagatgataccatcataaaagaacaggacttagcattaacgggatcatggcgacccaaaatc
ataggaggaataggaggaggtttacaagttagggagtataataatgtagaagtaatattagaagacaaaacatta
aaaggggcagtcttattaggaccaacacctataaatattataggaagaaatttttttagcccaggcaggagtcaaa
cttgttatggggcaattatcagaagcaatccccataactccagtcagactaaaagaaggggccaaaggaccttgt
gtaaagcaatggcctctatctagggaaaagataaaagctttgacagatatttgtgactccttagaaaaagaggga
aaaatatcaagaataggaggggaaaatgcatataacaccccagtattctgcataaaaaagaaggatcaatcgaaa
tggagaatgctaatagactttagggagctaaataaggccacccaggacttctttgaagtccagctaggaatacct
catccctcagggctacagaaaaagaaacaagtaactgttttagacataggagatgcttattacagcattcctcta
gatccagagtttagaaaatatacagcctttactattcctagcataaacaatcagggacccggacagaggtatcaa
tttaattgtttgccacaaggatggaaaggatcccccacaatttttcagaatacagcagcaaaaatactggcccag
attaaagaagaattaccggggctagaaattgtccagtatatggatgacctctgggtagggtcaaactactcagaa
agagaacatgatgaaaaagttagacagcttagagagaaactattacagtggggctttgagaccccagaaaagaaa
gttcaatcagaacctccctatgaatggatgggatataaactttggccaagcaaatggcagttacaaaagttagag
atagaagaaaaagatcaatggacagtaaatgatattcagaaattggcaggaaaacttaactgggcagcacaacta
tatccagggcttaaaaccaaacacatttgccgcctcattaggggaaaaaggggattgttagatccagtcctatgg
actcctgaggcagaactagaacttgaagagaatagaagcattcttaagacagaacaagaaggggcatattatgac
ccacaaaagcccctaagagcagcagtccaaaagttaggaaatggacagtggggatatcaaataaaacaggagcaa
aaggtattaaaagtaggaaaatatgagaaacagaaagcaatacatactaatgaacttagaatattagcaggactt
gtgcaaaagatagctaaagagagcctagttatctggggaaccttacccaaatttgagttaccagtagaaagggag
gtatgggaacaatggtggagtgattattggcaagtctcttggataccagaatgggaattcgtcagtgtcccacct
ctagtaaaattgtggtactctctggtcaaagatccaattccaaaagaagatgtctattatgtagatggggcctgt
aatagacaatcaaaagaaggaaaagcaggttatatcactcaatatggaaaacaaagagtcaaaactcttgagaat
actactaatcaacaggcagaactgcaggctatcctcatggctctagaggatagcgggtctaaagttaatatagta
acagactcccaatatgcgttagggatcttgacagctcaccccacacagagtgattccgccttagtaaatcaaata
attgagcaattaatcaagaaggagcaagtataccttcaatgggtcccagcacataaaggcataggaggaaatgaa
gaggtagacaaattagtcagtaaaggaattaggcaagtgttattcttagaaaggatagaagaagcacaggaagaa
catgagaggtaccataacaattggaaggaaatggcggacacatttggcatcccacaagtagtggctaaagaaata
gtagcagcatgtcctaaatgccaagtcaaaggagagcccgttcatggccaggtagacgcgtccccaggggtctgg
cagatagactgtacccacatagaaggacacatcatcatagtagctgtccatgtggccagtggcttcatagaagca
gaggaaataccaagggaaacaggaaaggctacagccaaattcctattaaagttactatcaagatggccagtaaaa
cagattcacactgacaatggaccaaattttgtatcacaagaagtcaaagctatttgttggtggggaaaaatagaa
catacaacaggagtaccctataatcctcaatctcaagggtcagtagaatctatgaataaacaattgaaagaaata
ataggaaaaattagggatgactgccaatatgtcgaaacagcagtattaatggcatgccacattcacaattttaaa
agaaagggaggaataggggggtatgaccagtgcagaaagactactaagtatactaactgcacaaatagaaacacaa

accttacaacaaaaaatccaaaaaattttgaattttagagtctactacagagaaggaagagaccctgtgtggaag
ggaccagcacagctcatttggaaaggagaaggagcagtggtaataaaagaagcagacggagacttgaaagtaata
ccaagaagaaaggctaagattattaaagaccatataagtagatggcaaggcatagtaagatactggatggaaaaa
aggggaatacagtgggaatataagatgcactataaaattcattgggcatggtacactatgtgtcaatacattatc
cctctggaaacaggagacatctatgtagacatgttctggcacttgaccccacagacgggatggctctccacatat
gcagtagggatacagtatttaagttaccaggggaattataggacagaattagatccagggacagctgacagtatg
atccacctccactacttcaattgctttacagaaagagccatccagaaagccatcaggggagaaaggttcgtcttc
tgcaactacccagaaggacacaaacagacagggcaagtacagacccttcaattcctagccctacaagcagtacaa
gtgccaatgagtgagcgagcctataggtattataggattgttcagaaagctctgtttgtacatttcaggtgtgga
tgtagaaggaggacaccatttgaaccttacgaggagaggagaaatttacaaccctgtaataataagtgcttttgc
aagcgctgctgttatcattgccagctttgtttcctacaaaaaggattgggtataaatatgaacataaagtacata
aattttctaatagttataggtataggtataggactagtgttaagtaaacagttatatgtgacagtcttttatgga
gtaccagtatggaaaaacagcacagtacaggccttttgtatgacacctaccaatagattatgggcaacaactaat
tgcatcccagatgatcatgactatacggaagtgcctctgaatattacagagccatttgaagcatgggctgacaga
aaccctttggtagcacaggcagggagtaatattcacctgctgtttgagcagacactcaaaccatgtgtaaaatta
agcccattatgtattaagatgtcatgtgtacccttaaatggctccaagacagggtcaacaacggcaccaagtaca
acacaaaccacaaagtcaacacaaaggccttgcaagcaatatgagaaaaacaacacgttgcaggcatgtaacgac
accattatagaaagagaaatggatgaagagcccgcttcaaattgcacctttgccatggcaggatatattagggac
cagaaaaagaattattcagtagtatgggatgatagggaaatttattgtaaaaatggaagcgacaatagctccaag
gtgaaagagtgctatatgattcattgcaatgactctgtgataaaggaagcttgtgacaagacctattgggatgaa
ttaaggctgagatattgtgctccagcaggatatgctttgcttaaatgtaatgatcatgattataatggctataag
caaaattgctctaatgtgtcagtagtacattgcacaggattaatgaacaccacagttagtacaggcctattgctt
aatggtagttattcagaaaatagaactcagatatggcagaaacatagagtgaataatgattcagtgattatcctc
tttaacaagttttataatcttacagtaacttgtaaaaggccagggaataagacagtcttgccagtcaccattatg
gcaggattagtcttccactctcagaagtataacatgaggttaagacaagcttggtgccacttccagggcaattgg
aaaggagcctggaaggaagtacgagaggaaatagtgaaattaccaaaagataggtatcaaggaaccaacaataca
gagcagataaaattgctgcgacaggatggggatccagaagcagctaacttgtggttcaattgccaaggagaattt
ttctattgtaaaatggattggtttctaaattacataaacaatgccacaactacgtttgatggaaacaagtgtggt
aataaaacaaagaaaggaaatgctccaggaccctgtgttcaaaggacatatgttgcctgccacataagatcagtt
gtaaatgattggtatacagtaagcaaattaacctatgccccgccaagagaagggcacttacaatgtacttccacg
gtaacgggtatgacagtagagctaaattacaacaataagaacgtaacaaatgtgaccctaagtccccaaatagaa
gggatttgggcagcagaattgggtcgatacaaattggtggagattaccccaattggctttgcacccacagaggtc
agaagatacacaggggggacatgagagacaaaaaaatgcatggggggtgtgcatggaaacaggtgtgtcataccacg
gtaccatggaaatggaacaatactcctagttgggacaatatgacctggctcgaatgggaaaggcaaattcaaaaa
ctggaaggcaacataacagggcttctggaggaagcaagggcacaggaagagaaaaatttggatgcgtatcaaaaa
ttaacctcatggtcagacttctggagttggtttgacttaagcaagtggtttaacatcttaaaaataggattctta
atagtaataggaattataggattaagactcttatatagtgtatacctttgtatagctagggttaggcagggggct
ttccagtacctgcaaaagagaccaacaaagggtaggatgactccggaaggacgacgtctgcaagagggagacact
tgggaagaatggagtgatgatgaagaagaaataggcttcccagtcaagcctagagtgcctctccgccaaatgact
tacaaacttgcagtggatttctcgcacttttaaaagaaaagggaggactggaagggatttattactcagaaagg
agagataagatcctgaatttatatgccttaaatgaatggggaatcttggatgattggaatgcatggacaccagga
ccaggaataagatatccgcgctgctttggcttctgctttaagctagtaccagtagagttacatgaagaagcacag
aattgtgagaggcactgcctggtccaccccgcccagatgatagaagaccccgatggaatcaaccatggagaagtc
ctggtttggaagtttgacccaatgctggcggttgagtttaaggcagggatacacttttcagacatgcatgcgaca
gttggtaaaggaaactag
>#18 KR862336 7239 nt
atgggtgcgggttcctcagcattgtcagggagaaaattagaccgatttgaagaaatacgtttacgcccgaacgga
agacaaaagtataaattgagacacttaatatgggcaggcaaggagatggagcgcttcggcctccacgagaagtta
ctagaaacagaggaaggctgtaagaaaatcatagaagtactcttcccccctagaaccaacagggtcggaaggttta
aaaagtctgtttaatctcgtatgtgtgctttactgcattcatcgagaacagaaagtgaaagacacagaggaagct
gtagtaacagtaaaacaacagtgccatctagtggagaaagaaaaaactgcagcagcgccacctggtggcgaaaag
aaaaatagcaacaccacagtgacatctagtggtcagagtcaaaattacccagtacaacaacaagggaatgcctgg
gtccataccccactctctccaaggaccctaaatgcttgggtaaaagcagtagaggaaaagaagtttggagcagag
atagtccccatgttccaagcactctcggaggatgcactccgtacgatataaatcaaatgcttaatgtgcttgga
gaccaccaaggggccttacaaatagtaaaagagatcattaatgaggaagcagccaattgggaccaaacacatcca

ccaccagcaggtccattaccagcaggacagctgagagatccgagaggatcggacattgcaggaaccactagtaca
attcaggaacaaatagaatggatctacacagcaaatccaagaacagatgtgggagccatttacaggagatggata
atcctagggctgcaaaagtgtgtaaagatgtacaatccagtcagtgttttggatattaggcaagggccaaaagaa
gcctttaaagactatgtagacagattttacaaagccataagagcagaacaggcctcaggagaggtgaaacaatgg
atgacggaaactctcttaactcaaaatgccaacccagaatgcaaagtcatcctgaaaggcctaggtatgcacccc
acccttgaagaaatgttaacagcttgtcaaggagtaggaggacctcagtataaggctaaactgatggtagaaatg
atgcagcaattacagtcaaatcaaaacatggtccagcaggggacaggaggcccaaggggaccaaaaggcctaga
ggaccaccaagatgctttaattgtggaaaatttggacacttgcagagaaattgtccagaaccaagaaagagcaag
tgtttcaaatgcggcaaaacaggccacatggcaaaagattgcaggggacaggtgaattttgaaattctcataaat
ggcctcccagtcatggtccttttagatacaggggcagatgatacaattattaaagaaacagatattaatttggca
ggaccatggcggcccaaattaataggaggcattggggaggcttaagagtaaaagaatataacaatgttacagta
caaatagaggacaaaacattgattggcacgatcttaatagggccaaccccaattaatataataggaagaaacttt
ttggctagtgcagggatgaaattagtaatggacaattaagcgataaaatacccattacacctgctaaattaaag
gagggagccaaaggtcccaaaataaagcaatggccactctcagcagaaaaaatcaaagccctcacggacatatgt
gaggacatggaaaaggaaggaaaactaaccagaataggaggagaaaatccttataacaccccaatcttctgtatt
aagaaaaaggatacctcacaatggaggatgttagtagattttagggaattaaataagatgacccaagactttttt
gaggtacaattagggatcccacacccagcgggactgaggaaaatgaaacagatcacagtgctagacataggcaat
gcctattacagtgtaccattagaccctgacttccgcaaatatactgcatttactatacctgcaattaataatgag
ggaccagggaaaaggtaccaattcaactgtctcccgcagggatggaaaggatcaccaaccatctttcaaaatacg
gcagcagggattctagagaaaattagggaagagttaaaagagttaactattatccagtatatggatgatttgtgg
gtaggatccaattgtggggaaaggaagcatgatgaactgatagagcgactaaggacagagcttctagcctgggga
tttgaaaccccagacaaaaaggtacagaaacaacctccctttgaatggatggggtataaattatatcctcagaaa
tgggtagtacaacccatagaattagaagagaaagaacagtggacagttaatgatattcagaaactagtaggaaaa
ctaaattgggctgcacaaatatatccagggttaaaaactaaaagaatttgtcaattaataaaaggaaagaaaaat
ttgttagaccctgtagtctggacagatgaagcagaggcagaatatgaagaaaataagattattctaaaaacagaa
caagaagggacatattatgatccagaaaaacctctaaaggtagcagtgcaaaagctgggagatggacaatgggga
tatcagttcaaacaagacaaaatcttaaagacagggaaattcatgaaacagaggaccactcatagtaatgagcta
agaattttggcaggattggtacaaaagatagccaaagaaagcctagtaacatggggaatactccctaagtttgaa
ctcccagtagaaagggaggtatgggaacaatggtgggcggagtattggcaagttggctggatacctgaatgggaa
tttgtcagtgtaccaccattggtcaccttatggtaccgcctcaccaaagaccccatacctaaacaagatgtctac
tatgtagatggagcctgtaacagaaactccaaaatgggcaaagcaggatatatcactcaatatggaaacagaga
gtgaaagaattggaaaatactaccaaccaacaggcagaattaacagctatccttatggcactacaagatagtgga
gatcaagtaaacatagtaacagactcccagtatgccttagggatcatatcaagtcaccccacacaaagtgactcg
cctctggtagaacagatcatccaagaattagtgaagaaaaaggcggtatacctacaatgggtaccagcacacaaa
ggcataggggggaaatgaagaggttgataaactagtcagcaaaggaataagacaagtactattcatagaaaaaata
gaagaggcccaagaagaacacgaaagatatcacaacaattggagagaaatggcagatacatttggtattccccaa
gtagtagccaaagaaatagtagctcagtgccctaagtgccaaataaaaggagagcctatacatggacaagtggat
gcttcaccaggagtgtggcagatggactgtacccatatggaaggacatgtgatcatcacagccgtacatgtagcc
agtgggttcatagaagcagaaataatacctagagaaacaggaaaagagacagcaaagttcctgttaaaaattctc
tccagatggccaataacaaagctacacacagacaatggccctaacttcatttcacaggaagtgaaggcaatgtgt
tggtggggaaaaatagagcacacaacaggagttccctataatccacaatcacaagggtcagtggaaagtatgaat
aaacaattaaaggaaatcataggcaaaattagagatgactgtcaatacctagaaacagcagtattaatggcctgc
cacatccacaatttaaaagaaagggaggaataggggatatgactagtgcagatagactcctgaacatcctaacc
acacaaatagaaacaaaacaactacaacaaaaaattcaaaaacttttgaatttcaaggtgtacttcagagaaggg
agagacccaatttggagaggcccagcaacactcatctggaaaggagaaggagcagtggtaattaaagaccaagga
gaactaaaggtagtcccaagaagaaaagcaaagatcataaaagattggcaaggaatagttaggttttggatgaaa
aaacgaggattaaattggcaatatcagatgcattatcagatacattgggcatggtatacaatgagcagatacaca
attcctcttacttcaggagatatagaagtagacatctattggcacttgacaccagaaaaaggatggctctctact
tatgcagtaggaatacagtatctttccaagataggacaatatagaacagaattagacccccaaacagctgacagt
atgattcactgtcattactttaattgttttacagaaagagccattcaaaaggcaatcagaggggaaagattcgtc
ttctgcacttatccagagggacataaacagacagggcaagtacaaacccttcaatttcttgccttggtagcagta
caaagagcatacaaatattatagactggtgcaaaaagccctgtttgtacatttcagatgtggatgtaagcgcatg
acagcctttgatccttacgaggagagaagaccattgcaaaaatgtgataataagtgctactgtaaaagatgttgt
tatcattgccagctttgctttctgcagaagggtttaggtgtgaatatgcatttgcaaaagctcctatctacattc
ttagtgttaggcttttagcatgggtaggaataggtaaacaatggatcacagtgttttacggagtaccagtatgg

aaaaacagttcagttcaagctttctgcatgacacccactaacagactatgggcaacaacaaactgtataccagat
gatcatgattacacagaaatacccttgaacattacagaaccttttgaggcttggccagatggaaaccctttagtg
gctcaagcagaaagtaatatacacttgttgtttgagcagacactcaaaccatgtgtgaaattatctcccttgtgc
ataaaaatgagctgtgtgcctctaggatctagtaaagctggtcctactactactcctgctagtagtaccaccgcc
aaaaacctaccatgtgtgaagaatgagactgatccacagctgagggattgtaatgcaacaatcatagaacaggaa
atggaggaggaaccagcatcaaattgtacatttgcaatggcaggatatgttagggatcagaagaaaaactactca
gtagtatggagtgatgcagaaatttactgtaagaacagtacctacagtgccaccagtaacaccaaagactgttac
atgatccattgtaatgattctgttattaaggaagcatgtgataaaacttattgggatgagttgcaacttaggtat
tgtgctccagcaggatatgctttgctgaaatgtaatgattatgattataatggatataagcaaaactgcagtaat
gtttcagtagtacattgcacaaagcttataaatacaacaattagtacaggcttattgcttaatggaagctattca
gaaaataggacacagatttggcaggaacatggagtgagcaacgactcagtgataatccattttaacaaacattac
aatctgacagtaacatgtagaagaccaggaaataaaacggtcttaccagtaacaatcatggcaggcttggtattt
cattctcaaaaatataatacaaaattaagacaagcttggtgtcacttccagggcaattggaaaggagcctggaag
gaagtaagagaagaaattgtaaaactaccaaaggaaagatatcagggaaccaataacacagagaatataaaattg
ttaaggcaagatggagatccagaagcagccaatttatggtttaattgtcagggagaattcttctattgtaaaatg
gattggttccttaattacatcaacaatcagacagttacctttgatggaaatacgtgtaataatactaagactaga
ccgagaaagggagcgcctggaccttgtgtgcaaagaacatatgttgcctgccacattcgatctgtcgtaaatgat
tggtacaaagtggcaaaattaacctatgctccaccaagagaaggtcatttgcaatgtacctccacggtgacgggt
atgacagtagaactgaattacaattcaggaaatcggacaaatgtgacactcagccctcaaataagaggcatatgg
gcagcagaattgggtaggtataaattagtggaaattactccaattggctttgcaccaacagatgtcaggcgatat
gaaggaccccaaagacagaaaaacgcctggggatgtgcatggaaacaagtctgtcatacaatagtcccgtggcaa
tggcacaatgtaacaccaaggtgggataacatgacctggttggagtgggaaaggcagatagcaaacttggaaggc
aacatatcgcgacaattagaagaggccagggctcaggaagagaaaaacatggatgcatatcaaaaactgtcagat
tggtcaagcttctggagctggtttgacctgagtcgttggttcaactatctaaagataggcttcttagtgatagta
ggaattataggattgagattactctatacagtatacttgtgcatctctagggttaggcagggttataccccttttg
tctcaacagaaagcttggcagtaccttcacggagaagaaaaaggcagaccaaggaatggaaaaatgagaccagaa
ggtagatatctacaggaaggagacacctgggatgaatggagtgatgatgaggaggaagtaggctttccagtgaag
ccaagagtaccaataagacagatgacttacaagcttgcagttgatttttcgcactttttgaaagaaaaggagga
ctggatgggatttattactccgagaggaggaataagatcctggatctgtatgccctgaatgaatggggaatcatt
gatgattggaatgcctggacaccaggacctggcattcgttacccgcgctgctttggtttctgctttaagttggta
ccagtagatctgcatgaagaagcacaaacatgtgaaagacattgccttgttcacccagctcagatgggagaagac
cccgatggaataaaccatggagaagtcctggtgtggaagtttgatccaatgcttgcttgtcaggcccggccaggg
caatttaatgacatgcatgagctggtgaagaggaagtag
>#19 KR862356 7275 nt
atgggtgcgggttcctcagcactgtcagggagacaattggaccaatttgagaaaatacgtttacgcccgaacgga
aagaaaaattacaaattgaaacatttaatatgggcaggcaaagagatggagcgctttggcctccatgagaagtta
ctagaaacaaaggaaggctgtcagaagatcattgaggttctctttcctttggaaccaaccggatctgaaggacta
aaaagtctctttaatttggtgtgcgtattatattgtttgcacacagaacagaaagtgaaagatacagaggaagcg
gtggcagtagttagacaacactgccacctagtggaaaaagaaaaaagtacaacaacgccatctggtggccaaact
ggaaataacatgcagacagcgccacctggcggcaggagtcaaaactttccagtacaacaacagggaaatgcatgg
gtccatgtacccctctccccacgcaccttaaatgcctgggtaaaagcggtggaggagaaaaaatttggagcagaa
atagtgcccatgtttcaggcattatcagaaggctgtacaccctatgatataaaccagatgcttaatgtgctagga
gaccatcaaggggcattacaaatagtgaaggaaatcatcaatgaggaggccgcccagtgggacctgacacatcct
ccacaagcaggaccaataccagcaggacaattaagggacccacgaggctcggacattgcagggaccaccagctca
ttacaggaacagatcgaatggatttatacggccaaccctaaagtagatgtaggagccatctatagaagatggatt
atattaggcctacaaaaatgtgtaaaaatgtacaatccagtcagtgtcctagatatcagacagggtcccaaagaa
cctttcaaggattatgtagatagattctataaggcaataagagcagaacaggcttcaggagaagtaaaacaatgg
atgactgagagtctcctgatacagaatgccaatccagagtgtaaaatcatcctcaagggattgggcatgcacccc
accctggaagaaatgttgacagcatgtcaaggagtagggggcccgcaatacaaggccaaattaatggtggaaatg
atgcaacaactgaatcaacagaatcaggtcatggtccaacaaggagggggaggacccagacgtggcccccaaaa
ccaattaggtgctttaattgcggaaaatttgggcatatccaaaagaattgtacagaaccaaggaaacttagatgt
ctaaaatgtggcaaaccaggccacatggcaaaagactgcaggggacaggtgaattttacagtgtatattaacggc
ctccccattaaggctctcttagacacaggagcagatgatactatcataaaagagacagatatacaattagcagga
cattggagaccaaagttaatagggggaataggaggaggccttaatgtaaaggaatatttaaatgtcaccctaaat
ttagaaggaaaagagttaatagggaccattttagtaggagaaacacctatcaatattataggaagaaacttctta

gccaatgctggtatgagattggtaatgggacaattatctgatgctatacccattaccaaagtagctctaaaagaa
ggagctaaaggaccgtgtataaaacaatggccactttcaagggaaaaaatagaagccctacagcaaatatgcaca
caaatggaaaaggagggaaaattaaccaagataggggagagattgcatataacacccctgtatttgcaataaga
aagaaagacaaaacacaatggaggatgctaatagattttagggaattaaataaagtaacacaagacttctttgag
gtccagttggggataccacatccaggaggccttcataaaaagaagcaaataacagttttggatatagggatgct
tattatagcattccattggacccggatttcagaaaattcactgcttttacgatacctagtgtaaacaaccagggt
ccagggactagatacatatttaactgcctcccgcaaggatggaagggatccccaactattttcaaaatactgca
gcaaagatattagaagaaataaaaacagaacttcctgctcttacaattattcagtatatggatgatatgtgggta
ggatcagatttaacaccatatgagcatgataaacagatcacaatactgagagaaaggctatctcaatggggcttg
gaaactccagaaaagaagctccaaaaggaaccccgtattcatggatgggatataagttgtggccacagaaatgg
cagctacagcccatagagctaccaaaccctgaggaatggactgtaaatgacatacaaaaattagtagggatgcta
aactgggctgcacaaatttacgcaggtcttaaaactaaacaaatatgcaaactgatcagagggaagaaagacctg
ttggaaaaggtaacatggactcaagaagctgaaatagagctagcagaaaatagagaaattctcaaaaccacacag
gagggagcatactatgaccctacaaaaagtcttagggcaacaatacagaaattgccagatggacaatggagttat
caaataaaacaagaaggaaaccaaattttgaggacaggaaaatacatgaaggctaaagctacacatacaaatgac
ttaagagtactggccggtctagtacagaaaattgctaaggagagtataacttgttggggactttacccgtcttt
gagttaccagtagaaagggaagtatgggaacagtggtggagtgattattggcaagtcagctggataccagaatgg
gaatttgttagtgttccaccattagtcaccctatggtataggctaacgaaagatcctatacccaaggtggacacc
tattatgtagatggatcctgcaacaggaatagcaaggaaggaaaggccggatatatcacacaacaggggaagcaa
aggattaggaccttagaaaataccaccaaccaacaagcagaattacaagcagtattgatggccctagaagatagt
ggaaaacaggttaacataataacagattcccaatatgtgttaggggttatatcgtcacatcccacgcaaagtgac
tcgccactggtagaacaaattattgctgcattaattcaaaaggaagcggtgtatttaaactgggtaccagctcac
aaagggataggaggaaatgaggaaatagacaaattagtgagccaaggattagacaggtcctcttcctagaaaaa
atagaagaagcacaagaggaacacgaacggtaccacaacaattggaaggagatggcagatacctttggaatacct
caagtagtagcaaaagaaatagtagcacaatgtccaaaatgtcaaataagaggggaaccaatacatgggcaagta
gatgcctcaccggggtttggcaaatggactgtactcatctagaaggaaaaattattataacagcagtacatgtt
gcaagtggctttatagaagcagaaattatacctagagaaacaggaaaggaaacagcaaaatttttactcaagcta
ctttctagatggcctatcacaaaactacacacagataatgggcccaactttgtgtcgcaagaagtacaagcaatg
tgttggtgggggaaaatagagcacacaacaggtataccatataaccctcaatcacaaggttcggtagagtctatg
aatagacagttaaaggacattattggaaaaatcagggaagactgccaatacttagaaacagcagtactcatggcc
tgccacattcataattttaaaagaaagggaggaataggggatatgactagtgctgatagattactaaacatatta
accacccaactagaaaccaaaactttacaacaaaaaattcaaaaattcttgaatttcaaggtctactacagagaa
ggccgtgatcctgtgtggaaaggaccagccgtgctcatttggaaaggtgaaggggcagtggtgatcaaagaacag
ggagaactgaaagtcatacccagaagaaaggctaagattattaaggattggcagggaattgttagattctggatg
accaaaagacaactcccatggaagtatgaaatgcattaccaaattcactgggcatggtatacgatgagcaggtac
aggattccactcaaaaatggagaaattgttatagatctctattggcatttaaccccagaaaaaggatggctctca
acttatgcagtagggatacaatatttaggtaatcaaggacactatagaacagaaatagacccccatacagcagat
agtatgatacatacagagtatttcacttgttttacagaaagagccatccaaaaggcgatcagaggagagagattc
atcttctgcaactacccagagggacataaacaactagggcaggtacagaccctacagtttctagccttacaggta
gttcaaattcctagactagagagggcatataagtattacagactggtgcaaaaagccttgttcgttcacttcagg
tgtggctgcaggcgcaggacaccttttgaaccctacgaagagagaaggatggacaaggggaggcgcagcgcctc
ctccagggcttgcatgagcctttgaaggcatgtactaacaaatgctattgcaagaactgctgttatcattgtcag
ttgtgtttctgcaaaaagggttaggtgtgaatatgctcttgcttaagatctgtctcttaggcttaatagggata
ttagtttatagccaccaccaacaatatatcacagtattctatggggtaccagtatggaaaaatagcacagtccaa
gccttctgcatgacacctactactagattatgggctacaaccaattgcataccagacgatcatgattatacagag
gttcctcttaacataacggaaccctttgaggcatgggcagatagaaatccattagtggcacaagcagaaagcaat
atacacctgttgtttgagcagacactcaaaccatgtgtcaaattaagcccactctgcataaggatgagttgtgta
gaattaaaatcctcaagaccaacaaccacggcgtcaacaagtactgtaacagcaccactatctgacccatgccag
agaaacgagagtgagactgtaattggaaagtgtaatgacacaataatagaaaaggagatggaggaagagccagct
tctaattgcacctttgctatggctgggtatgtaagagatcaaaagaagaactactcagtagtctggaatgatgca
gaaattatgtgtaagggaaatgggaccaacgaggccggaaaaagagaatgctatatgattcattgtaatgattca
gtaataaaggaggcttgtgaaaagacatactgggatgagttaagactgagatattgtgcacctgcaggcttcgct
ctactcaaatgtaatgatatggactataatggatataagcagaattgctctaatgtatcagtggtacattgcaca
ggcttgatgaataccacggttagcacaggcttattgcttaatggtagttattctgagaatagaacacaaatatgg
caaaagcatagagtaaataatgattcagtgatagtactcttcaataagttttataatctcacagttagatgtaga

agacctggcaataagacagtattgccagtgaccattatggcaggtctggttttccactcccagaagtataacacg
aggctaagacaagcgtggtgtcatttccaaggcgattggaggggagcctggagggaggtaaaagagcacatagtt
aaattaccaaaagacaggtataaggggacaaatgacacagaaaagatctatctgcagagacaatttggtgaccca
gaagcagcaaatctttggttcaattgtcaaggagaattcttttattgtaagatggattggtttcttaattatcta
aacaacctaacagtagatgcagatcataataagtgcaacaattcgccaaaaggaaaagcaccaggaccctgtgtt
cagagaacatacgttgcctgccatatccgatctgtcataaacgattggtataccatctctaaaaggacttatgct
cctccaagagaaggtcacttggagtgtctttccacggtcactggtatgacagtagaacttaattatatatccaac
aatagaacaaatgttaccctatcccctcagataggggtatatgggcagccgaattggggagatacaaattagtt
gaaattacgccaattggttttgcacccacggacgtcaggcggtatacaggagggcacgacagagcaaaaaatgcc
tgggggtgcgcatggaaacaagtatgtcataccactgtaccttggcaatggcaaaacaggaccccacaatgggac
aacatgacttggttagaatgggaaagacaaatagcggatttggagagcaacataacagggcaactggtagcggct
agagaacaagaagagaaaaatttggatgcttatcaaaaacttacctcgtggtcagattttggagctggtttgac
ctgtcaaaatggtttaatattctaaaattaggcttctttatcatcataggcataatagggttaagattgctttac
acagtatacacatgcatagctagggttaggcagggttattctcctctgtctccacagacagctttacagcttcta
caaccaagaaaaaggaatggaaaacttacagaggacggaaggagactacaagcaggagataactgggaagattgg
actgatgacgaagatgaagtgggcttcccagtgagaccacaagtccctctgagacaaatgacttacaaattggca
gtggatttctctcatttttgaaagaaaagggaggactgagtgggatttactactcagaaagaagagataagatc
ctgactctctacgcactcaacgaatggggaatcatagatgattggaacgcttggaccccgggacctgggatccgc
tatccccggaccttcgggttctgcttcaaattggtacctgtaaacctgcatgaggaggcagagacttgtgagaga
cattgccttgtccaccccgcccagctgggagaagaccctgatggcataaaccatggagaggtgctggtttgggta
ttcgaccccatgttggcctgtcaggcccgaccaggacagtttaatgacatgcatgagcttgttaagaggaagtag
>#20 KR862363 7185 nt
atgggagcgggttcctcagcattgtcaggtaggcaattagaccaatttgaacaaatacgcttacgcccgaacgga
aagaaaaagtataaattaaaacatataatatgggcaggcaaggagatggagcgcttcggcctccatgaaaagtta
ttagaaacaaaagaaggctgtcaaaagatcatagaagtcctttttcctctagaaccaacgggctcggaaggacta
aaaagtcttttaatctggtatgcgtactttactgcctgcatacagaacagaaagtgaaagacacagaagaggca
gtaacaacagtaaggaaacagtgccacctagtggaaaaacaaaaaattgcaacagagacatctagtggccaaaaa
gaaaatagcagctcaaaagagacacctagtggcagaagtggtaattacccagtgcaacaacagggaaatgcatgg
gtacacgttcctctatccccgcgcactctgaatgcgtgggtgaaagcagtggaggagaaaaagtttggggcagag
atagtacccatgttccaagctctatcagaagggtgcacaccatatgacatcaatcagatgttgaatgtgttagga
gatcaccaaggagccctgcagatagtaaaagaaatcattaatgaagaagcagcccagtgggacttgacacatcca
ccgccagcagggccattaccagcaggtcagctaagggatcccagaggatcagacatagcaggaacaactagtaca
ttacaggaacaaattgaatggatctacactgctaatcctaaggtagatgtaggtgctatatataggagatggatt
attttgggggctgcaaaaaatgtgtaaaaatgtacaacccggtcagtgtcctagacatcagacaaggaccaaaagaa
cctttcaaagactatgtggacagattctataaggccattagagccgaacaagcctcaggggatgtaaaaatgtgg
atgacagagaccctcctcgttcaaaatgccaaccctgagtgtaaagtcattctgaaagggttaggcatgcatccc
actttggaggagatgctaactgcctgccaaggggtaggggaccccagtataaggcaaaattgatggtggaaatg
atgtcccaaatgaatcaacagcaacagatcatggtacagcaggggaaccctagaaagggaccgccaagaccgtcc
gcaaaatgttttaattgtggaaggtttggacatctgcaaagaaactgcccagaacccagaaaaataaagtgcctt
aaatgtggaaagccaggacatatggcaaaagactgcagggggcaggtaaattttaccatacatataaatggcctc
ccaataaaagtacttcttgatacaggagcagatgacactattatcaaagaaacagacattcaattacaaggttca
tggagacccaaattaataggaggtataggggaggtattaatgtaaaagaatataataatgtagtagtagaaata
gaagggaaagaactaattggcactgttttagtaggagaaactcctattaacattatcggaagaaatttcttagct
aacgcagggatgaaattagtaatgggagcattatctacaaatataccaatcacaaaagtgaatttgaaagaagga
gcaaaaggaccttgcttaaaacaatggccactgtctaaagaaaaaattctagcattacaacaaatttgtcaggaa
atggaaaaagaaggaaaattgactaagattggagggaaaatgcctacaacacaccagtatttgcaataaggaag
aaagacaaaacacagtggcgaatgttaatagactttagggagctaaataaagtcactcaagacttctttgaagtg
caattaggaataccacatccgggggattaaaaaagaagaaacaaatcactgtgctagatataggcgatgcctat
tatagcatccccctggatccagactttagaaaatacacagccttcactataccaagcataaataatgaaggacca
ggcactaggtatcaatttaattgtctaccacaaggatggaaaggctcacccaccatcttccaaaatacagcagca
aagatattggaagaaataaaggcagacttacctgcattaactatcattcaatatatggatgatttatgggtagga
tctgacttaaccccatatgaacatgataaacaaattgaaaccctcagacagagattaggacggtggggactagaa
actcccgagaagaaattacagaaacaacctccctatgaatggatggggtacaaactatggcctcataaatggcag
ttacaacctatagaattaaaagagccagaagaatggacagttaatgacattcagagagtggtaggacaattaaat
tgggcagcacaattgtacccaggattaaaaacaaagaacatttgtaaactgataagaggaaaaaagaaccttttg

gaaccagtacaatggaccccggaagcagaggtagaatatgcagagaataaagagattctcaaaacttcacaggag
ggagcctattatgacccctcaaaaaatctcaaagtagcagtacagaagcttccagaaggacaatggagctatcag
tttaaacaagaaggaggaaacattctaaaaacaggaaaatacatgaaaaataaagcaactcacactaatgatctt
agagtattggcaggattagttcaaaaaatagctaaagaaagcatagttatctggggacaattacccatttttgaa
ttacctgtagaaaaagagacttgggagcaatggtggagtgactactggcaagtagggtggataccagattgggaa
tttgtcagtgttcctccattagttacactttggtatcgactgacgaaagaccccatcccaaaggaagatgtttat
tatgtagatgggtcatgcaacagagaaagtaagttaggcaaagcaggatacatcactcagcaaggaaagcagaaa
gtcatcactttggaaaacacaacaaaccaacaggcagaattgcatgcagtactgctggccttacaagacagtgga
aaaagggtaaacattgtgacagattcacaatatgttttaggtatcatctcttcacaccccacacagagtgattca
ccactggtagaacaaataatagcccagttgattcaaaaggagttagtatatttaaattgggtgccagcccataaa
ggtataggggaaatgaggaaatagacaaattagtaagtcaaggaattaggcaagtactgttcctagaaaaaata
gagcatgcacaggaagaacatgaaagataccataacaattggaaggaaatggccgacacatttgggataccacag
gtggtagcaaaagaaatagtagcacagtgccccaagtgccagataaggggagaaccaatacatgggcaaattgat
gcctcaccaggtgtgtggcaaatggattgtacccacttagaaggaaaaataattatcacagcagtccacgttgcc
agtgggttcatagaagcagaggtcataccaagggagacaggaaaagaaaccgccaaattccttctaaaaataata
tcaaggtggcctatcacaaagttacacacagataatggcccaaattttgtatcacaggaagtacaagccatgtgt
tggtggggaaaggttgagcatacaacaggagtaccttacaatccacaatcacaaggttcagtagaatcaatgaat
agacaattaaaagagattattggcaaaatcagagatgactgtcaattcttggaaacagcagtacttatggcctgc
cacattcataattttaaaagaaagggaggagtaggggatatgactagtgcagaaagattattgaatatgttaacc
acacaattagaaatcaatcacttacaaaaacaacaacaaaaattttcaaatttcaaggtctacttcagggaaggc
agagatcctgtctggaaaggacccgcaacactcatctggaagggagaaggagcagtggtaataaaagaccaagaa
gaattaaaagtagtcccaaggaggaaagcaaaaattataaaagatttttggatgaataagaggggcctcccatgg
caatatacaatgcattacaaaattcactgggcatggtacacaatgagtagatacacaatacctgtagggaggga
caaatagtaatagatctttattggcatttgaccccagaaaaaggatggctctctacatatgcagtaggcatacag
tatttggacttgcaaagctcctactggacagaagtggacccagaaacagcagacagtatagtacattgtcactat
tttaattgctttacagagagagccgtccagaaggcaatcagaggggaaagattcatcttctgcaactacccagag
ggacataaacagacagggcaagtacaaactctccaactcctagctctcattaaagttcagagagcatataaatat
tacagattagttcaaaaggccttgtttgtccatttcagatgtggttgtagacgtaggggaccttttgatccctac
gaagaaagaagacctttggaagcatgttttaataaatgctactgtaagaaatgttgttatcactgccagctttgc
tttttgcaaaagggattaggtatcaatatgactatagtacctgtactagtttatttaggaatagggcttttatta
gcaaaggagcagtatattacagtattctatggagtaccagtatggaagaatagttcagttcaagcattctgcatg
acacccaccacaagattatgggctactacaaattgtatcccagatgatcatgactatacagaggttcctttgaat
atcacagaacctttttgaagcatgggctgatagaaatcccttggtagcacaagcagggagcaacatacatctattg
tttgagcagacactcaaaccttgtgtgaaattgtccccctatgtataaaaatgtcctgtgtagagcttaactct
tctagaatcactacaaccagtactccatcagccactgacaagccctcagacccttgtggtggtaatagtgcgacc
ataggaagatgtaacaatactcttatagagcaagaaatggaggatgagccagcttctaattgtacctttgcaatg
gcagggtatgtgagagatcagaaaaagaattattcagtggtatggaatgatgcagaaattatgtgcaaaagcaag
aacagcagtgacaataagactaaagagtgctatatgatacactgcaatgactcagtgatcaaagaggcatgtgaa
aaaacgtactgggatgagctgagattaagatactgtgctccagcaggatatgctttattaaaatgtaatgatgaa
gattataatggatataagagaaattgctcaaatgtgtcagtagtgcattgcacaggactgatgaataccacagtc
agtacaggcttactgcttaatggtagttatagtgcaaataagactgaaatatggcagaagcacagggttaataat
gattcagtaatcatactgttcaataaacattataacctgacagtgagatgcaggagaccagggaacaaaactgta
ttacctgtaaccataatggcaggattggtcttccactcccagaagtataacacgaggttaagacaagcatggtgt
cacttccaaggcaattggcgaggggcctggaaggaagttaaggagaaaatagtacagttaccaaaggacagatat
caaggaaccaatgatacaaaacaaatatttttgcaaagacaatttggagatcctgaggcagcgaatctatggttt
aattgtcaaggagagttcttctattgtaagatggattggttcctaaactaccttaataaccttacagtagatgca
aatcacaataaatgcacaaacagcaccaagaaggacacgcaccaggaccttgtgtacagaggacctatgttgct
tgccatatccgaacagttattaatgattggtacacagtatcaaagagaacatatgccccaccaagagaaggacac
ttggagtgtttatctacagtcacagggatgacagtggaattgaattataattcaaagaacagaactaatgtgaca
ttgagtccccaaatagaaggcatttgggcagcagaattgggcagatataaattagtggaaataacaccaattggc
tttgcacccacggatgtcaggcgatatacgggaggacacgaaagacaaaaaaacgcctgggggtgcgcttggaaa
caagtttgtcataccactgtcccctggacttggagtaacaggactccagagtggaacaacatgacttggcttgag
tgggaaagacaaatagcggacctggaaagcaacataacaggacaattagtcgctgccagggaacaagaggaaaag
aatttagatgcatatcaaaaattgacctcgtggtcagacttctggagttggtttgacttatctaaatggtttaat
atccttaagttaggcttctttgtaatcataggaataataggcttaaggttgctatatagtgtgtatctttgca
a

gcaagggttaggcagggttattctccccttcttccgcagacagcttttcaatacttgcagaagcccaggaagaaa
aatgggaacatgactgcagaagggaggagattacaacaaggagatgagtggagcgaatggtccgatgatgaagaa
gaagtcggcttcccagtcaaaccacgagttccacttaggcagatgacctataaacttgcagtggatttctctcac
tttttaaaagaaaagggaggactgagtgggatttactactcagaaagaagaaataagatcctgaccctttatgct
ctcaatgaatggggaatcattgacgactggaatgcctggaccccaggtccaggaatcaggtacccgcgcaccttc
ggcttttgctttaagcttgttccagtagtcctgcacgaggaagcagaaacatgtgagagacattgcctcgtccac
cccgcgcagatgggagaagacccggatgggatatcccatggagaggtcttgatctggcagtttgatcccatgcta
gcctgtcaggctaggccaggacagtttactgatatgcatgatcttgtcaagaggaagtag
>#21 M29975 7350 nt
atgggggcggctacctcagcactgaataggagacaattagatgaatttgagcatatacgacttcgcccgaacgga
aagaaaaagtatcaaattaaacatttaatatgggcaggcaagaagatggaccgcttcggcctccatgagaagtta
ttggagacagaggaaggttgtaaaaagatcatagaagttctctctcccctagaaccaacagggtcggaaggaatg
aaaagtctgtataatctggtgtgcgtattgctttgcgtccaccaagaaaagaaagtgaaagacacagaggaagct
ttagcaatagtaagacaatgctgccacctagtggacaaagaaaaaactgcagttacgccacctggtggacagcag
aaaaataacacaggaggaacagcgacacctggtggcagccaaaattttcccgcacaacagcaagggaatgcatgg
gtgcatgtaccactttcacctcgcaccctaaatgcatgggtaaaagcagtagaagagaaaaaatttggggcagaa
atagtacccatgttccaagccctctcagaaggctgcaccccatatgacatcaatcagatgcttaatgtcttagga
gatcatcagggggccttgcaaatagtgaaagaaataattaatgaggaagcagcccagtgggatgtaacccaccca
ccgccggcaggccccttgccagcgggacagctcagggatccgggggatcagatatagcagggaccactagtaca
gtgcaagagcagctagagtggatctatactgctaacccaagggtagatgtaggggccatctatcgaagatggatc
atcctagggttacaaaaatgtgtaaaaatgtacaatccagtgtctgttttagatatcagacaagggcccaaagaa
ccattcaaagattatgtagacagattctataaagcaataagagcagaacaagcttcaggagaagtcaaacaatgg
atgacagaatctttgctcattcagaatgccaacccagattgcaaagtaattttgaagggcctaggatgcacccc
actcttgaagaaatgctgacagcctgtcaaggggtgggaggcccaagttacaaagccaaagtcatggcagaaatg
atgcagaacctgcagagtcagaacatggtacagcagggaggtggaaggggaagaccaagaccccccgccaaagtgt
tacaactgtggaaaatttggccacatgcagaggcagtgtcctgagccaagaaaaataaaatgtcttacctgtata
ataggaggaactgccgttaaggcattattagatacaggggcagatgacactataataaaggatacagatttacaa
ttaaggggatcatggagaccaaaaatagtaggaggaattggggagggttaaacgtaaaagaatatgataatgta
gaagtacaattggaagacaagatattaagaggaacagtcctcataggagcaactcccatcaatatcataggaaga
aactttttagcccaggcaggagccaaattagtgatggggcaattgtcgcagacaataccaatcaccccggtacgc
ttaaaggaaggggccagaggaccacgattgaagcaatggccactctctaaagaaaaaataatagccctgcaagaa
atttgcaaaacattagaggaagaaggaaaattaagcagggtagggggagacaatgcatacaatacaccagtattc
tgtataaggaaaaaagacaaatcacagtggagaatgctggtagatttcagggaactcaacaaagctacacaagac
ttctttgaagtccaattaggtataccccatccagcagggttaaagaaaatgaagcaaataaccattatagatgtg
ggggatgcatattatagcataccactggatcctgagtttagaaaatacacagctttcaccatccctacggtaaac
aatgagggaccaggcataagatatcaatttaattgcctaccgcagggctggaaggatccccgacaattttccaa
aacacagcatcaaaaattctagaagaaataaagaaagaattaaaacagctgacgattgtccagtacatggatgac
ctctgggtaggatcacaagaagagggtccaaagcatgatcagctagtacaaacacttaggaatagattgcaagaa
tggggattagaaacaccagagaaaaaggtgcaaagagaacctccctttgagtggatgggatataaattatggcct
cataaatggaagttacaaagtatagaattagagaagaaagaacaatggacagtgaatgatcttcagaaattggta
gggaaattaaattgggcagcacaattatatccaggattgagaacaaaaaatatctgtaagctacttagaggaaag
aaaaatttattagacgtggtagaatggaccccagaggcagaagcagagtacgaagaaaacaaggagatcctaaaa
acagagcaagaaggtacttattatgcaccagaaaaaccccttagggcagcagtacagaaattaggagatgggcaa
tggtcataccaattcaagcaggaaggaaaaatcttaaaggtagggaagttcgccaaacagaaagctactcacacc
aatgagttgcgtgtactagcaggagtagtacagaaaatagggaaagaggccctagtaatttggggacaattaccc
acttttgaactcccagtggagagggacacatgggaacaatggtgggcagactattggcaagtcagttggataccc
gaatgggactttgtcagtgttccgcccttagtaactttgtggtatacactgactaaggaacccatcccgggagag
gatgtctactatgtagatggagcctgtaatagacagtcgaaagagggaaaagcaggctacataacccaacaaggc
aaacaaagagtacaacagctagaaaacacaacaaatcaacaagctgaactgacagccataaaaatggccttggag
gatagcggccctaaagtcaatatagtaacagattcacaatatgcgatgggcatattgacagcacagcccacacag
agtgactccccactagtagaacaaataatagcacagatggtacagaaagaagccatctatctgcaatgggtacct
gctcataaaggtataggggcaatgaagaaatagacaaattagtaagcaagggagttagaagaatattgttcatt
ggcaggatagaagaagcacaagaagaacatgataggtatcacagtaactggagaaatctagcagacacatttgga
ttgccacaaatagtagctaaagaaattgtagcaatgtgcccaaaatgtcaagtaaaggggaaccaatacatgga
caagtagatgcttcaccaggagtgtggcagatggactgcacacatatagaaggaaaaatagtgatagtagcggtc

catgtagccagtgggtttatagaagcagaggttatccctagggaaacaggaaaagagacagcaaagttcttgtta
aaaataataggaagatggcccatcactcacctccatacagataatggaccaaatttcacttctcaggaagtagct
gctatgtgctggtggggaaaggtagaacacacaacgggggtaccatataatccacagtcccagggatctatagaa
agtatgaacaaacaattgaaagagataattggaaaaataagagatgactgtcaatatacagaaacagcagtactt
atggcctgccacattcacaattttaaaagaaagggaggaatagggggctaacagctgcagagagactaataaat
atgataacaacacaattagaaatcaacactctacaaaccaaaatccaaaaaattttgaattttagagtctactac
agagaaggcagagatccagtgtggaagggacctgctcgcctgatctggaaaggagaaggcgcggtagttctcaag
gaaggtgaagaactgaaggtagttccgagaaggaaagcaaaaatcataaaagactattggatgaataaaaggaat
ctgaaatgggaatacaaaatgcattatcaaatcacttgggcatggtacactatgagcagatatgtaatacccctc
ccaggaagtggagaaatccatgtggatatctattggcatttagctccaaaacaaggatggctctcaacttatgca
gtaggaatacaatatgttagcctagtaaatgataaatatagaacagaattagatcccaatacagcagactccatg
atacattgtcattattttacctgttttacagatagagccatccaacaggcactaaggggaaacaggttcatcttc
tgtcaatttccaggaggacataaactaacaggtcaggtaccctccttgcaatatttagcattactagcccatcaa
tattatcgcttagttcagaaagctctctttgtgcatttccggtgtggatgtcgcaggagacaaccctttgagcca
tacgaggagaggagacaatacaggaaaccccttgagacatgtacaaataaatgcttttgcaaaaaatgctgttat
cattgccaattctgcttcttacggaaaggactaggtattacctatatgacaaagttcttaggaattttatagta
ttaggaatagggataggaatagggataagtacaaaacagcagtggataacagtgttctatggagtaccagtatgg
aaaaacagctcagtccaagctttttgcatgacacctactactaggttgtgggcaactactaattgcataccagat
gatcatgactatacagaagtaccactgaatataacagagccatttgaagcatgggcagacagaaatcccttagta
gcacaagcaggaagtaacattcacctgctgtttgaacagacattaaagccctgtgtaaagctatcacctctatgt
atcaaaatgaattgtgtagagttaaaaggctccgcaacctctaccccagcaacctctactacggcaggaaccaaa
ctaccctgtgttagaaataaaacagactccaacctacagtcatgcaacgacaccatcatagaaaaggagatgaat
gacgaggcagcgtcaaactgcacctttgctatggctgggtacattagggaccaaaagaagaattactcagtagta
tggaatgatgcagaaatcttttgtaagcgtagtacatcgcataatgggacaaaagagtgctatatgatccactgt
aatgattcagttataaaggaagcttgtgataagacatattgggatgaattaagactaagatattgtgctccagca
ggatacgctttgcttaaatgtaatgattgggattatgcaggatttaagccagaatgttctaatgtttcagtagtg
cattgcacaactttaatgaatacaacagtaaccactggtctgttattgaatggaagctattcagaaaatcgaacc
cagatctggcaaaaacatggagtgagcaatgactcagtgttaatcttgctcaataagcattataacctgacagtt
acatgcaaaaggccagggaataagacagtcttgccagtaacgataatggcaggattagtcttccactcacagaag
tataatacaagactaaggcaggcctggtgccacttccagggcaattggaaaggagcttggaaggaagtacaagag
gaaatagtaaaattaccaaaagaacggtaccaaggcaccaatgatacaaacaaaatctttttgcaaagacaattt
ggagacccagaagcagcaaatctatggttcaactgtcaaggggaattcttctactgtaaaatggactggtttta
aattatctgaataatttaacagtggatgctgatcataatcattgtaaaaacaacgcagggaaaggtcgaagtcca
ggtccctgtgtacagagaacttatgttgcctgccatatccgatctgtcataaatgattggtatactatatcaaag
aaaacatatgctccaccaagagaaggacatttgcagtgcacgtccacagttactgggatgacagtagagctaaac
tataataaccagaacaggacaaatgtaacattgagtccccagatagaaaccatctggcggcagaattgggcaga
tacaaattggtagagattacaccaattggatttgcacccacagaagtcaggcgatacacgggaggccaagagagg
caaaaaaacgcttgggggtgcgcgtggaaacaagtatgtcatacaacagtaccctggacgtggaataatacacca
gagtggaataatatgacctggttggagtgggaaaaacagatagaaggattggagggcaacataacaaaacaattg
gaacaggcaagggaacaagaggaaaagaatttggatgcttatcaaaagttgtcagactggtcgagtttttggtct
tggttcgatttttcaaaatggctgaacattttaaagataggctttttggcagtaataggcgttatagggttaaga
ttgctttacacattatatacttgcatagctagggttaggcagggttactctcctttatctcctcagcaattggtt
cttgacactcagcatctggttacacaacagctgcctgaccctccttctcaagcttagatgggcttggggagctca
aagccgcagcacaagaagcagttaaccatctggcgagctttgcacgcaacgcggcacaccagatatggcttgctt
gcagatccgcttatcgggcaatcatcaactctccaagaagagtgcgacaagggcttgaggaagtccttaattagg
aagagaaatggcaacatgactccagaaggaagacgtctacaggacggggaccaatgggatgaatggtcagatgaa
gaagatgaagtgggatttccagtaagaccaagagtgccactaagacaaataacatacaaacttgcagtagatttt
tcgcacttttaaaagaaaagggaggactggatgggatttattactccgataggagaaataagatccttaatctg
tatgccctcaatgaatggggaatcattgatgattggaacgcatggtcaaaaggacctgggataagatacccgagg
tgctttggcttctgcttcaagctagtaccggttgccctgcatgaggaagcagaaacatgtgaaaggcattgcttg
gtacacccagcacaactgcatgaagaccctgatggtataaatcatggagaaatattggcatggaagtttgatcca
atgttggctgttcagtacgacccctcaagggagtactttacagacttatattcaacagttggtacaggaaactag
>#22 AF301156 6996 nt
atgggcaacgagcagggactgttagggaagaaaacattagaagacctacagaaagtccggctgaagaaagggaag
aaaggatgttataagataaaacatgttagatggatgtgcacagaggtaagccgctgtgttttaatatttgagctg

ttaaagtcagcaacaggagtggctcaaatattggaaaaggtgacgccattagtggacacaggatcagaagtgttg
aggtccctatatggatgttctgtgtgttactgcctgcacagaaaatggaatattgaggacactcaagaggcagaa
aagaaagtagaagaagcttataaaaagcaggccatgatagaaatggccagcaaggaggaggagaaagcaaaaaag
gaagcagagaaattggacatgccaatagtaacagggcctcaagggccagtgcatcaacccctgagccccaggacg
ttaggagcctgggttaagtgtgtagagggaggaattgctccttccttggctcccatgttcttagcttattctacg
gggcaatagcttatgatatgaacttgatgctcaatattttggatactcatcaaggattccctcaggtcttgaag
gatgagattaataaaaaggcagaagagtatgacctactacatccggtccagcagccacaacagcaaggagcatta
aggcagcctacagcatcagatatcacaggcaacacaagctcagtagcagaacaagtggcttggggagagcccata
gccaacatttataggggctggatagtacagagtctggaaaaggtaatccagattgcaagaccatcctctgtacta
gatattagacagggaagtaaagaggattttaaaagctatgtagacagattttattcagccctaagggcagaacct
gcagcaggagaaatcaaggcctggatggctaataatttgctaatacagcatgccaaccctgactgcaaaagaatt
ctgaaaggactgcagaagccatctttggaagacatgctagcagcatgccagggagtgggaggaccagaccacaag
gcaaaggtgctagcagaggctatgcagcagttccagcaagagagaaccaacatgattgaagtaaagacagcaaaa
tgcttcaattgtcagggaatagggcacttagccagaatgtgtcccaaaagaccaataggaggagccggtcgagga
agaggccgaggacggggaggctttagaggagctcccagaaggccagtgaggtgttttacctgcaatcaggaagga
cacatgcaaagagattgtcccaacaagcaggcaatagtagaagtagaaggacaaaagtgccaagcgctgttagat
acaggagcagatgatacagtttttaaagaagaagatgttcaattaacaggcgcttggacagcagtacaaattcag
ggaataggggggagcaatcagagtaaaacagtatagagataaaattctaaagatgggaggaaaagaatacagagga
gatatcttaataggacacacccccataaatatagtaggtagaaatatgttaaaagaagggaaattagtagtagct
caattatcagataggatcccagtcaccaaggtgacgttaaaagagggcatggatgggccaaaagtaaagcagtgg
cccttaagcaaagaaaaaatagaaggtttacaaaagatatgtgaaagattggaagcagagggaaaaatagaaaaa
gcagaattagggaatccatataatacaccaatattttgcattaggaaaaaagataaaaatgaatggaggaaatta
atagatttcagggagctaaacaagagaacacaagacttcatggaagtccaattagggataccacacccaggaggt
cttatagaaaagacccatataacagtattagatataggagatgcctatttctcaattcccttggatccggattat
caaaagtacacagcttttacaatcccatcagtgaacaatttggcaccagggactagatatgtgtacaaagtgcta
ccgcagggttggaaagggtctccaacaatcttccaaggaacagtagcaagaatcctagaaccgttcaggaagaga
ggacaattgcaaatagctcagtacatggatgatttatatctaggatcagatctgcctttagaggagcatagaagg
gcagtacaggagctcagagatgcactcttgtactatgggttagagacaccagaaaagaaataccaggcagatcct
ccatacaagtggatgggctatgaattacatcctaagcaatggaaattacaaaaggtcaaaattccagaacaggat
aagtggacagtaaatgatatacaaaaattggtaggagccttaaattggttaagccaattatacccaggaatcagg
acaaaggagataagtaaatgcactaaaggtaagaagcatttgttggaggaagtgcagcttagtgcagaagcagaa
gaggagctacaagataacaaacagataatcaaagaagaagttcagggagtgtactatataccacatgaggacatt
tgggtagacattttcaggttagggaaaggacaatggggctatgcagtctgccaaaaacatggaacgctaagaaga
ggaaaacataacacaggcaaaaatcagcactttaatagtatgcaggagttagcttcagttatacaaaagatаggt
agggaagccatagtcacctggggaaaggtgcccaaaatgaaagtcccagcaaagagagaggattgggaacagtgg
tggagtgactattggcaaagtgcttggatcccagagatagagtttgtctcatcctcttatgtagtaaaattagta
tggaacttggttttagagcctctgaaggaggcaccaacttattggacagatggaggatgctccaggaaattagga
gcgggaaaggcaggatggataaattcaaaaggagaagaagaagtagtcaatcttcatgaagggtcaaatcagcaa
gcagagctgacaggagttttgctagctctaaaacatgggccaaaaagaatgaatttggtaacagacagtctttat
gcattagggataattactgggcagccatatgatcagcttccatctagtagaggggagattattcaggcaggtatg
gctaaagaagccattcatgtagcctggtgtccagcacataaaggataggaggaaatgaactgatagatcaaaag
gtaggagtaagacaggtaatgtggatagataaaatagaagcagcagaagaagaccatcaaaagtttcatagtaat
gtgcagtatctaaaagaacaatttgggttacccactgtagtagcaaaagaaatttgggaaagatgttcagagtgt
cagaacaaaggacaagctgttcatggccagctagattatagctatggcttatggcaattagattgtactcatgag
gaaggaaaggttatcttagtagcagtacacgtctgtactctgttctgctgggcaaccatattaaagagagaaaca
ggggaagaaacaggtagggccctcataaaattggctagtcagtgggaggttagacaagttcacacagataatggg
ccaaattttgtaagtcagcactttaaggcagcagtgtggtggctaggaatagcacacaccacaggacacccctat
aatcctcaatcacaaggagttgtagagcagagaaacaaggatgttaaaagaaagattaagaaaatgaaagatcaa
gcagaaacattagaaagcaaagtagcaatggcagtctatgcgctcaattttaaaagaaagggaggattagggggg
aagagtccatgggaaagacaagtagaaagagcaataatagaattagatacacaaaacctaacaaaattacaaaat
caaaaatttaaaattttaaggcctactggaaagagcacacaggagagtggcaaggaccaggagaactggtgtgg
aaaggtgaaggagcggtagtcatcagaaattctcaaggtaccttgtttgtgaaacctagaagaaaagtaaagatc
accagactccaatatggagaagatgtgggcagtgagaatttgttatccaagcagtttccagaatgccaatattat
tatttaggaaaaacagagtcacccgaatggtggacagaaggtaaaatagagttttacttagggtcagctattaag
ttaagcataatatacttgggcactttaaccccagatagatatcatagaggaccaccagtctcttatgaattacag

atagtaagtgggaaacagatttaagctggcaacaagcagtagggtacatgcacatcataaggggaagtgtgta
cgcatagaggaagaagcaagaaaagctataaggggcttaccctggaatccttgtgatttccaggtaggacaccta
ggagtgttgaagttgcaagaaaacataacccgggaggccagaaggcacttccctttgactcacatctcagtgata
atgtggggttgtactagaaggcactgggggagaccgtggacggctggtctggcttatgtgagaatcatggagtgt
gccctctacttgcacgttcatgcgggggcgtgtatgcttagatatctctttataaacttgctagtattaggtata
gtgttaagtaataaatgggtaacagtatatcaaggagtacctgcatgggaagaagcagatgtaaatgatcagcag
ttcttctgcttttcttcctccccagagattcaacaggtgttggggtgtttaccacctccgcctggaaagccagta
gaacaaaacatgccaaatgtaacagaagcttttgatctttttaagaatagcttcagtggggaggtatggataata
acacagactaccttagaacaaaggctaagaccatgtgctaagttgacagcatactgtgctccaatgatttgcacc
aaagtaaataggactgaaaatggcactagtacagtagccccaacaacaacaaataatagtgcttcagattgggat
gagtcaaattggaaagaatatccgtggtataattgcagaatgaattcaacagctttcttacttaaagatagaaag
gagttagaattaggattttcagtagaagatttaactgtgcttggaaataaaaatgacagtaatagtatcagggca
acaatgaaagattgtgcaaattatacagttacccaagtatgtgatatgactatagtggatccagttaggacaggg
ttctgtgcagctccaggatacatgcttttaagatgtgatgataaaaagtgggatgggacaggagcatgcaacaat
gtgacagcagtgtcctgtacccatgagtttaatataacagtcatgtctcatgtattagtcaatgcctcaaaagag
ttgtctgattgggcgaaagatagagagggagtctggaagaatgactcagggacaattgagtattattggtttcca
aaagatatagctttagggtgtataagaagaggaaatagttctcacagaatctaaacactgccaatggagcaaaa
ttctactatgagcttattccttattcaaaaggcatttatggaaggtgtcagtttgtaccaatgactgggcagaat
aagaaaaacaaaacacaattcgcaatagaaatcaaaaagaatttaacagcttggctagagcgcatttcacgcaaa
aatataactatcactccaaggaatggaaatagaacaagtgatccagaggctactttcacctttgtgatttgtcat
aggttattcttttactgtaatgcatctagtttatggaaacatgattccccagtcatgaactgtaccattagaaag
ctggtgaatagttgggtcactcatgccaggatcctgtatgggccgcctccaggaggtcatctacaatgtaactgg
gagaaacagccagttattgcgttcatgggcaccatagaaggagacaatgatggtaatgggtgtgcttatccagca
gcaccaaattttaagcatgcattaagtactttggaattgggaagatacaaattggtaaaaatgaggacaactact
tatgtcccaacagatataaaaagatcagtgaatgtaaattggcatcatgggagacaaaagagaggcatctttgca
ttcgccagcattgggtgtgcaaacatgcaaatttgtagaactattgtgccctggaacaagacctggggagaggag
gatccatggcagaacatgacctggaaacagtggcatgagcgagtaagaaattacacagatataatagaggcagac
ttagtagaggcatatgacttacaagaggaaaatgaaaagaaattggcagaattaggggattggactaattggttt
tcaggctttggcctattcaacatctttaagtatgtactctatgctgcatatgttgtaggaggtttaataggactt
agaattattatggtagtaatagcttgcataagaggagctttcagagttaagggatttcagcagataggcaggact
aatgtgtcttcacagatctgcccttgcccgctttgctatagacatccagatgtatattaccacaacccagaacca
gaggacgtagaagatgtagaagcaggatgttatgtgatgtctcgactgccagtacgacaggcgaccattaagcta
ctggtggacatctcttgctttttgaaagaaaagggtggactggaggggattattagaacccctgagagagatgac
ctgatagagcagtatgcctacattgagtggggctgcttaaaaggttggttggagtatgaagatgagctgggagag
gatggagcattaaaagaggacagaaagcctctggttgctggatggctttggaaattagtctacatagagcaactt
ggagagtatgcatacagctatgacttgagtctgttgagcgttacaagcaggaagaagaagaaaccacaacaggtt
gccatagagatggtggactga
>#23 AY159322 7449 nt
atgggcgcgagcgcgtcaggacttaggggagaaaaattggatgaactggaaaagattaggttacggccctccgga
aagaaaaagtaccagttaaaacatgttatatgggtaagcaaggaactagacagatttggcctacatgagaagttg
ttagaaagtcaggaaggatgcgaaaaaattcttagcgtactctttccactagttcctacagggtcagaaaattta
atttcgctgtacaacacctgttgttgcatttggtgcgtacatgcgaaagtgaaagtaacagatactgaggaagca
aaagagaaagtaaaacaacggtaccacctagtggttgaaagagagaatgcagcctcagaagaagaaaaaggagca
acagcgacacctgctgttcgctcaaaaaattaccccattcaggtaataaatcaaacaccagtacatcagggaatt
tcaccaagaaccttgaatgcatgggtaaaatgtatagaggagaaaaagttcagcccagaaatagtgcctatgttt
attgccttgtcagaaggatgcatcccgtatgacctcaatggcatgctcaatgccattggagatcatcagggagct
ctccaaatagtgaaggatgtcatcaatgaggaagctgcagactgggatcttagacaccccctgtgggacctatg
ccccaaggagtgctaagaaacccaacaggaaatgacattgcaggaacaacaagctccatagaagagcagatagaa
tggacaaccaggcagcaagaccaggtaaacgtaggaggtatatacaaacaatggatagtactagggctgcaaaaa
tgtgtaagcatgtataaccctgtaaatattttagacatcaaacagggaccaaaagagccttttaaggactatgtg
gatagattctacaaagctctgcgggcggagcgcacagatccacaggtcaaaacttggatgacgcagacattactc
atccagaatgccaacccagattgtaaagctaccctcaaagggttaggcatgaatcccacattggaagaaatgttg
ctggcctgccaaggggtaggaggacctaaatacaaggcacaaatgatggcagaggccatgcagcaagcacaggca
gctgtaatgatgcagaactcgggggaccgccgcggggtcccccgagacaacccccctagaaaccctagatgcccc
aattgcggaaagtttggacatgtactgagggattgcagagccccaagaaagagaggctgttttaagtgtggagat

ccaggacatctaatgaggaactgcccaaagatggtggaaatagaggggcaaaaggtagaggcacttctagatact
ggagcagacgatacagtaattaaagatttagagttaacaggaaattggaagccacgaattataggaggaattgga
ggagcaatcagagtaagacagtattttaattgtaaagtagaaatagcaggaaaaattactcatgctgcagttctg
gtgggccctacccctgtaaatattataggtagaaatgtacttaagaagttaggatgtactttaaattttcctatt
agtaagatagagacagtaaaagtaacactaaaaccaggaacagatggaccgagaataaaacagtggccactgtct
aaagaaaagatcctagccttacaagaaatatgcaatcaaatggaaaaagaaggcaagatctctagagtaggtcca
gaaaatccatacaacaccccagtattctgtataaaaaagaaagatggaaccagctggagaaaattagtagatttt
agacaactgaacaaagtcactcaggatttctttgaggtgcagttaggaatcacacatccaggaggcctaaagcaa
tgtgagcaaattacagtattggacataggagatgcctatttttcatgtcccttggatgtggactttagaaagtat
actgcgttcaccattccatcggtgaacaaccagggcccaggaatcagataccagtataatgtcctaccacaggga
tggaaaggctctccagcaattttccaggcaacagcagacaaaattctaaaggcttttaaggaaaagcatccagaa
gtcttaatctatcagtatatggatgacttgtttgtggggagtgatctaagtgcctctgaacacagtaaaatgata
gtagaattaagggaacatttgcagttctgggggctcgagaccccagacaaaaagttccaaaaggaacctcccttt
gaatggatgggatatgtgctgcacccaaagaaatggacagttcagaaaatacagctaccagaaaaagaaagctgg
acagtaaatgatattcagaaattagtagggaaacttaattgggcaagccaaatctattccggaattaaaacaaaa
gagctctgtaaattgataagggggcaagacccctagataaaatagtggaatggactagagaagcagaactagaa
tatgaagaaaacaagctaatagtgcaggaggaagtacatggagtgtactaccagccagaaaaacctttaatggca
aaggtgcaaaagttaacacaaggacagtggagttaccaaatagaacaggaagacaacaaacctctcaaggtagga
aagtatgccaggacaaagaatgcccacacaaatgaattgagggtacttgcagggttggtacaaaaggtagctaag
gagtgtctagtaatctggggaaagctaccgaaattctatctcccgttagagagggaggtatgggaccaatggtgg
catgactattggcaagtaacatggatcccagagtgggacttcatttcaacaccaccattaataaggttatggtac
aacctgctaaaagacccaataccaggggaagatgtatattatgtagatggggcagcaaacagaaactccaaggaa
ggcaaggcaggctattatacctctagaggtaaaagcaaggtagtagccttggaagaaacgaccaatcaaaaggca
gaattgacagctataaagatggctttgcaagattcaggacccagggtgaatatagtcactgattcccagtatgca
ctaggcatactttcagcagccccagatcagtcagacaaccccatagttagggaaataatagaactcatgataggc
aaggaagagatttatctctcatgggtcccagcccacaagggtataggaggtaatgaacagatagataaattagta
agtaagggaataagacaggtactatttttagagggaatagataaagcccaggaagaacatgacaaatatcacaat
aattggagagcgttgtctcaggactttaacctacccccatagtggcaaaagagatagttgcacaatgtccaaaa
tgtcagacaaagggagaacctgttcatgggcaggtaaatgcagatccagggacatggcagatggactgcacccat
ttagaaggaaaaatcatcatagtagcagtccatgtagccagtggatatttagaagcagaggtaataccagcagag
acaggaaaagaaacagcgcacttcctgttaaagctagcaggtaggtggccagtaaaacatctacacactgacaat
ggtcctaactttgtcagtgaaaaggtggccacagtctgctggtgggctcaaatagagcacaccacaggagtacct
tacaacccacagagtcaaggagtagtagaagcaaaaaatcatcatcttaagaagatcatagaacaagttagggat
caagctgaaaggctagaaacagcagtgcaaatggcagtgctaattcacaattttaaaagaaaaggggggataggg
gagtacagtcctggagaaagaatagtagacataataaccacagacatcctaacaaccaaattacgacaaaatatt
tcaaaaattcaaaattttcaggtttattacagagaaggaagggaccaacagtggaaaggaccagcagagctcatt
tggaaaggagaaggcgctgtggtaatcaaagaaggagttgatttaaaggtggttcctagaagaaaagcaaaaata
attaaggaccaggacgtcaagttgagagattggaattcaatagtaaaatatcataagtataaaggagagaaacat
ttagaaaattggcaattataccccccattttcaatgctcaggatggtggacacactctcaaaagaagatcccttt
aaggatggttcaaagattatcatcaccgtcttgtggaacttgaccccagaaaaagggtggctgtctcaatacgcc
tttacagtagaatatcaaaaggaaaattatttcacttacatagacccagtgacggcagatagaataatccacggg
gaatatttcccatgttttacagatcaagcaataagaaaagccttgtttggagaaagactggtagcttgttacttc
ccctggggacatagaggtcaggtagggactctacagtttttggccttacaagcctatctcagaggtagaaagatg
gaacagccacctgaggatgaggctccccagagaggaccatataataaatggctggtaggtactttggcagaaata
caagaggaagctttaaagcatttcgataggcgcttgctacatgcagtaggctcatgggtgtatgagcaacaagga
gacactttagaaggtgtccaacagctaataagcatttgcaaagagcactgttcttacactttagaaagtgcttc
tgtaaagtttgttgctatcattgtcagctgtgcttcttgcagaaagctttaggggtacattatatgttaagatat
cttaggtatatagtcttaggaataatagtaagtgtaatagtaggagaacaatgggtgacagtgtactatggcaca
cccaaatggcataaggcaaggacacacctgttttgtgcaacagacaataattcattctgggtgaccacaagctgt
gtccccagtctgttgcactatgaagaacagcatatccccaatataacagagaattttacaggccctatagaagag
aatgaaatagtgacacaagcttggggagcaatttcttccatgatagatgcagtattaaaaccatgtgtaaggtta
acaccttattgtgtgaagatgaaatgtacagaaggtcaaaatgaaacagagcaagcaacagctaagaccacaaca
cctgtacccacaacaacaacccccctccactaccactagctccagcacaaataagacaacaactcctgtgttggtt
gttgagaaacaaaataatgagacaacaacacagcaaaatagagtatgtaaattcaacacgacaggattatgtagg
gactgcaaattggaaatagaggaaaactttagatatgaagatgtaacttgcacaaagcttaataaaactggtagt

gctactaatagtactgaacctgaatatgagtgttacatgacttcatgtaacgcaacagtaataacacaggaccgt
aacaaagcttccacagatagaatgacatttaggttgtgtgcacccccagggtttgtcttactaaaatgtaatgaa
aaattgaacaagacaaaattatgtgggaatgtgtctgcagtgcagtgcacagcaccgctgccagccacaatctct
acaatgtttggcttcaatggaactaagcatgattatgatgagcttattcagacaaacccacgaaaaggaaaagat
gagtttcatgaccataaatatgtatatagagtggataaaaaatggggattacaggtaaggtgtagaaggaaggga
aatagatctatcatttccacaccaagttctacaggattgctgttctatcatgggttggagccaggaaagaatctt
agaaagggcaagtgtcagttagaagggaaatggggacaggctttacatagtctatcattagagctaagaaagata
aatgatagcatttacaaagacaaccacaatatgacatgtaaaagcagtaacaacaaaaagaacacaacagggtgt
catcttaaaactataagtataagtgagtccaccgttaaaggcgaaccaggagctgaaactattatgctcctctgt
ggaggagaatatttcttttgtaattggactaaaatatggaaggcatggaatagcaaacaaagttcagtttggtac
ccttacatgtctcgcaatattagacaaatagtaggtgattggcataaagtaggcaagaaaatttatatgcctcct
gtgtctgggtttaataatgaaataagatgtactaatgatgtgacagaaatgttctttgaggtccaaaaaactgat
gatgacaatggatatatcataaaatttataccacaagattggattcaaaatcaatacacagcagtgggggctcat
tacaaattggtgaaggtggatccgataggttttgcccccacagacatacacagacatcatttgcctaatacaagg
caaaaatcggtatggggatgttcattctcccaagtttgtcatactagtgtaaaatggcccaataatagtatagtg
cccaattggacctcagagacatggctggaatgggacagaagagtaaacagcatagtgacaaatatgaccatagac
ttacagagggcatatgaattagaacagaggaacatctttgagttgcaaaaattaggggacttaaacttccatggg
ctaactgggttcgaccttacatggtggcttaagtatgttaagataggactgttagtagtagtagttatcatagga
ttaagaatgttagcttgcttatggtcagtattagggaagtttaggcagaagccttggaagccagagcaagtcagg
agctggctgaaaaggtcgacgctttacatttggctgaagaacctccaagcagtaattgaacgggatctaaggcac
cagcaggatgtaactctcccctcagaaaaagagcaaccaagtgatgaagaggaagaggtaggctttccagtctac
ccaacgcagccagtgcctgaagcaacctacaaggacttgatagacatgtcccactttttaaaagaaaagggggga
ctggaagggatttggtggtctaaaagaagagaagagatcttggacctttatgcacaaaacgagtggggactaata
cctaattggcagaactacacaaagggaccggggataaggtatccgaagactttcgggttcttgttcaagctggtc
ccggtggcagtgtcgcccccccctggaggaggatgaatgcaacaggctgctaaactcttctcaaatgggaatccag
gaagacccagagggagagaggttgatgtggaagtttgattcagggcttgcttatacgttctatgctcctataatt
aggccagaggaatacaagtgtgtgacaagtctgagctatgaagcctacaagaaggaggaaaagcctgactgctgc
aagaggaagtggtggcagttctag
>#24 AF367411 7014 nt
atgggcgcgagcgcgtcaggtcttaggggagaaaaattggacgagctggaaaagattaggttacggccctccgga
aaaaagaaataccagctgaaacacataatatgggtaagcaaggaactagacagatttggccttcatgaaaagctg
ttagaaagtaaggaaggatgcgagaaaattcttagcgtactctttccactagttcctacagggtcagaaaattta
atctcgctgtacaacacctgctgctgcgtatggtgcgtacatgcgaaagagaaagtaacagatacagaggaagcg
aaagagaaagtaaaacaaaagctccatctagtggccgaaaaggaaaatgcagcatcagaaaaagaacaaagagca
atagtgacacctagtggccgctcaaaaaattacccaatacagataataaatcagaccccagtccatcagggaatt
tcaccgcgcacgctgaatgcgtgggtaaaatgcatagaagagaagaagtttagcccagaaatagtgcccatgttc
atagccttgtctgaagggtgcctcccctatgatctcaatggcatgctcaatgctattggagaccatcagggagcg
cttcaaatagtgaaggatgtcatcaatgaggaagctgcagactggaacttgagacacccacaagtgggtcctatg
ccccaaggtgtcttgagaaacccaacgggcagtgatattgctggaacaacaagctccatagaagagcagatagaa
tggactacaagggagcaagatgcagtaaatgtgggaggaatatataaacaatggatagttttagggcttcagaag
tgtgtaagcatgtataatccagtaaacatcctggacataaagcaaggaccaaaggagccgttcaaggactatgtg
gacaggttttacaaagctctgcgggcggagcggactgatccacaagtcaaaacctggatgacacaaactttgctc
atccagaatgctaacccagattgtaaatccatccttaaagggctaggcatgaatccctctttagaagagatgccg
ctagcctgccaaggggtaggaggccctaaatataaagcacaaatgatggcagaggctatgaaggaagcccagtca
gctgtaatgatgcagaattcgggaggccaccgcgggggcccccgagacaacctcctagaaacatcagatgccct
aactgtggaaaatttggtcatgggctgagggactgtataagccctcgaaaaaagggatgcttcaagtgtggagat
ctaggacacataatgagaaattgcccaaagatggtggagatagaagggcaaaaagtggaggccttactggacaca
ggagctgatgatacagtaatcaaagatctagatttaaaaggtaattggaaaccacagattattggaggaattgga
ggatcaatcaatgtaaaacagtttttcaattgtaaagtaacaatagcaggcaaaactacacacgcttcagtccta
gtgggccccacacctgtaaatattgtaggtagaaatgtgctgaagaaattaggatgtacactaaattttccagtt
agtaaagtagaaacagtaaaggtaacactaaaaccaggaactgacggacctaaaataaaacaatggccattgtct
aaggaaaagatttagccttacaagaaatatgtagtcaaatggagaaggaaggccagatctctaagataggtcca
gaaaatccttacaacacaccggtgttttgcatcaaaaagaaagatggaaccagctggagaaaattggtagatttt
agacagttaaacaaagtaactcaggacttctttgaggtgcagttgggaatcccacaccctggaggtctcaaacaa
tgtgagcaaattacagtactggacataggggatgcctatttctcatgtcctttggatgaggactttagaaagtac

actgcattcaccattccatcggtgaataatcagggcccaggaatcaggtaccagtataatgtcctaccacaggga
tggaagggctccccagccattttccaggcaacagcagataaaatcttacagcccttcagagagagacatccagat
gtagtgatctatcaatatatggatgatctctttgttgggagtgatagagttgccccagaacacagcagaatgatt
caagagttaagagaccacctcttgttttggggctcgagaccccagacaagaagtttcaaaaggaaccaccctttt
gagtggatgggatacatactgcaccctaagaaatggacagtgcagaaagtacagcttccagagaaagaagaatgg
acagtaaatgacatccaaaagttggtagggaaacttaattgggcaagtcagatttattccggaattaaaacaaaa
gagctctgtagactaattagaggggcaaaaccccctagatgaaaaagtagaatggactagagaagcagaattagaa
tatgaagaaaacaaactcatagtgcaggaggaagtgcatggagtatactatcaaccagagaagcctttaatggca
aaggttcaaaaattgacacaagggcaatggagttaccaaatagagcaggaggataataagccgctgaaggtaggg
aaatatgccagaacaaagaatgctcatacaaatgagcttagagtgcttgcaggactagtacagaagattgcaaaa
gaagctttagtgatctggggaaaactccctaagttttatctgccaatagaaagagaagtctgggaccagtggtgg
ccagaatattggcaagccacatggattcccgaatgggaatttgtatccactccacaccttatcgggttgtggtat
aacctgttgagagaacctgtgccaggagaagatgtgtattatgtggatggagcagctaacagaaactccaaagaa
gggaaagcaggatatgtaacagctagaaataaatccagagtaatagccctagagaataccacaaaccaaaaagca
gaattggaggcaataaaaatggcattacaagactcaggaccaaaagtaaatatagtgacagattcacagtatgct
atgggtatcctatcggcagcacccgaccaatcagacaaccccatagtaagagaaattatagaactcatgatccac
aaggaagcagtatacctagcttgggtaccagcacacaaaggcataggagggaatgagcaagtagataaactagtg
agcagaggagtaaggcaagtactgtttttagaaggcatagataaagcacaagaagagcatgataaatatcacaat
aattggagggcattagcacaagacttctgtataccaaacatagtggcaaaagaaatagtagcccagtgtccaaag
tgtcaaacaaaaggagagccaatacatggccaggtagatacatccccaggaacctggcaaatggactgcacacat
atggaaggaaaagtaatcatagcagcagtccatgtagcaagtaggtatctagaagcagaagtaatacccacagaa
acaggaaaagaaacagcacacttcctgttaaaattagcaggtagatggccagtaaaacatctacacacagataat
ggccctaatttcaccagtgaaaaagtagccacagtctgttggtgggctcaaatagagcacactacaggaattccc
tacaatcctcagagtcaaggagtaatagaggcaaaaaaccatcatctcaaacaaatcatagggcaagttagagac
caagcagagaaactagaaacagcagttcaaatggcagtattaattcacaattttaaaagaaaaggggggataggg
gaatacagtccaggagaaagaatagtagacattatagcaacagacctcctaacaactaaattacaacacaatatt
caaaaaattcaaaatttcgggtttattacagagaaggaagggaccaacagtggaaaggaccagcagaactcatt
tggaaaggagaaggagcggtggtaatcaaagaggggacagacttgaaggtagtaccaaggagaaaagcaaaaatc
atcagagatagacatgcagtagaaagatggaattcactggtaaaataccacaaatataaaggagaaaaacatttg
gacaaatgggatttaaaggcacattttcaatgctcaggatggtggacacactcacaaaaatatattcccctagaa
gaagatgaacagatcataatcaccatcctgtggaacttaaccccagaaaaaggatggttatcaacttatgccatg
acaatagaatataagttcacaaactactacactcacatagacccccaaacagcagacaggatgatacattggaaa
tatctcccctgttttacagaacaagcaataagacaagccctgctaggaaagagactaacagtgtgctacttccac
tggggacataaagggaaggtagggagcctgcagtacttggctttgctaagttatacagcctactgtaacatggag
caaccacccgaggacgaggcccctcaaagagaaccatacaatgaatggctaattgacaccttagcagaagttcaa
gaagaagctttaaagcattttgatagacgcttattgcatgcagtaggaagctggatctacaaaacctatgggat
acattagaaggggtgcgacagcttatctcaattcttcaaaaagctctgtctatgcactatagaatgcttaggtac
ataatattaggaataattgtaggactagggttaggaaaccaatgggtgacagtatattatggcacacctaaatgg
cataaggcagagacacacctgttctgtgcaacagaaaatgattccttatgggtaacaactagttgtgttcccagc
ttgttgcactatgaagagcaaccaattccgaacataacatggaatttcacaggtcccatggaagaaaatgaggta
gtaatgcaagcatggggagctatctcctccatgatagacgcagtattaaaccttgtgtgaaactgaccccatac
tgtgtcaagatgcagtgtacaaaaggaaaagaccttgctacccctatacccactacatccaccacaactaccaca
actaccaccaagacggtggcaaatactactgagttggacatagacaccaataacactgaaactacaacacaacag
aacagggtctgtaaattcaatactacaggattatgtagggattgcaaattggagatagaagaaaattttagatat
gaagatgtaacttgtacaaatggcacaaataacacatattcttgttatatgacacaatgcaatacatcagtaata
acccaggactgtaataaagcatccacagatgaaataaaatttagactgtgtgcacctccaggatatgttctcttg
aggtgcctagaaagattgaatgtctctaaaaaatgtaccaatatcacagcagtacagtgtacacagccattgcca
gccaccacctcaactatgtgtggatctagtgggacaaaacatgattataatgaattaatacagacaaatacaaag
aaaggcaaagaggagtttcatgaccataagtatgtgtatagagtagatgagaagtacaacttacaagtagtatgt
agaagaaaaggaaacagatctgtgatctcaacacctagtgcaacgggattgatcttttatagtgggttagaacca
gggaaaaatcttaagaaaggtatgtgtcagttgaagggacaatggggaatagccatgcatgatctagcaatagaa
ttaagaaaaatagacagctccatatggagaaatgtaacaaagggctattgcaaggggttaaaaagaaaggaaaat
agaacgggttgtgcccttaaaacaataaaggtctctgactacaccacgaaaggcgaaccaggagcagaaactatt
atgctcctctgtggaggagaatacttcttttgtaattggacaaaaatttggagaacatggaatgatcaaaattca
tcagtatggtatccatggatgtcatgtaatattagacaaataatagatgattggcataaagtaggaaagaagatt

tatatgcctcctgtatctggatttaataatgagataagatgttctaatgatgtaacagaaatgttctttgaggtt
caaaagacagaagagggatacatcatcaaatttgtgccacaagatgaggtacaaaaccgatttacagcggtagga
gcacactatcaactagtaaaagtggacccaattggctttgctcctacagaagtggcaagatatcatctgccagaa
gctaggcagaagtccatgtggggctgctcattcgcccaggtatgccacacacatgtaccatggccaaatgactct
ataacaccaaattggacctcagagacatggctggaatgggataaaagagtaacagctctaactgacaatatgaca
gtaaacttacagaaggcatatgaattggagcaaaagaatatatatgagctagaaaaattaggagattggacttcc
tgggctagctggttcgactttacgtggtggcttaaatatgttaagataggtttactaatagtaatagtgattata
gtgttaaggatttagcttgcttatggtcagtcttaggaaagtttgaagaggaagaaacgggctttccagtatat
ccacagtgcccagtaagagaacccacctacaaggacttggttgatatgccccacttttaaaagaaaagggggga
ctggaagggatttggcattctaaaagaagagaagagatcttggacttatatgctcaaaatgaatggggattcatt
cctacctggcaatcttatactgatggcccagggataagatacccgaagacatttaggttcttattcaagctttgc
ccggtggcagtgcctccagaccaggagaacaatgagtgtaacaaattgctccagtcctctcagctgggaatccag
gaagaatccttgggggagagaggctagtgtggaagtttga
>#25 AF328295 7422 nt
atgggcgcgagcgcgtcaggtcttaggggagagaaattggatgaactggaaacaattaggttacggccctccgga
aagaagaaataccagttaaaacatataatatgggtaagcaaggaactagatcgatttggccttcatgaaaaacta
ttagaaactcaggaaggatgcgagaaaattcttagcgtactctttccactagttcctacagggtcagagaatctt
cttgcgctgtacaacacctgttgttgcatctggtgcgtacatgcgaaagtgaaagtaactgatacagaagaggca
aaaaagaaagtagaacaatgcttccatctagcggccaaaggagaaagtgcagcctcagaaaaagaagagaaagca
acagcgacacctagtggccgctcaaaaaattatcctattcagataataaatcaaacaccagtgcatcaaggaatt
tcaccaagaactttaaatgcctgggtcaaatgtattgaggaaaagaaattcagtccagaaatagtacctatgttc
atagccctatcagaaggatgcctcccatatgacctcaatggtatgttaaatgctattggggaacatcagggagct
ctccaaatagtgaaggatgtcatcaatgaggaagctgcagactgggatcttagacacccccaagtaggacctttg
cctcaaggggtgctgagaaacccaacaggaagtgacattgcaggaacaacaagcagcatagaagaacagatagaa
tggacaactagacagcaggaacaggtaaatgtaggggcaatctataagcagtggatagtcctagggctgcaaaaa
tgtgtgagcatgtacaacccagtgaacattctggacataaaacaagggccaaaagagcctttcaaggactatgtg
gacagattttataaagctctgcgggcggagcgaacagatccacaagtcaaaacctggatgacacaaacattgctc
atccaaaatgccaacccagattgtaagagcatcttgaaaggattgggcatgaacccctctttagaagaaatgtta
ctagcatgtcaaggagtggggggaccaaaatataaggcacaaatgatggcagaggccatgcagaagatgcagtca
gaagtcatgatgcagaactcgggggggaccaccgcggggtcctccgagacaacccccctagaaaccccagatgtccc
aactgtggaaggtatggacatgttctgagagactgtagactccccagaaagaagggatgctttaagtgtggggac
acaggacatatgatgagaaactgcccaaaaatggtggaaatagagggacaaaaggtagaagccctactggacact
ggagcagatgatacagtaatcaaagatgtagatttaacaggaaattggaaaccccaaattataggaggaattgga
ggagcaattagggtgaaacagtatttcaattgtaaaataacagtagcaggaaaaagtactcatgcttcagtgcta
gtgggccctacccctgtaaatattataggtagaaatgttcttaagaagttaggatgtactctgaattttcctatt
agtaagatagagacagtaaaagtaacactaaaaccaggaatggatggaccaagaattaaacagtggccactgtct
aaagaaaagatttagccttacagacaatatgtgatgaaatggaaaaggaaggcaaaatctctagaataggtcca
gaaaatccatataatacaccagtatttgtataaaaaagaaagatggaaccagctggagaaaattagtagatttt
aggcaattaaatagagtgactcaggatttctttgaggtgcagctaggaatcccacatccgggaggcttaaaacaa
tgtgaacaaatcacaatattggatataggagatgcctatttttcatgtcctttggatgagagctttagaaagtac
actgcatttaccattccatcggtaaataatcaaggcccaggaatcaggtaccagtataatgttcttccacagga
tggaaaggctctccggcaatatttcaagcaacggcagacaaaattctaaaaacattcaaagaggaatacccagaa
gtattaatttatcaatacatggatgatctgtttgtgggaagtgatctgagtgcgtcagagcacaacaaaatggta
aataggttgagggaacatttaaggttctggggggctcgagaccccagacaaaaagttccaaaaagaacctcctttt
gagtggatggggtatgtgctgcacccacagaaatggacagtccaaaagatacagctgccagaaaaagagacatgg
acagtaaatgatattcagaaactggtaggaaaactcaattgggcaagtcaaatctattccggaattaaaacaaaa
gagctctgcaaattgatcagaggggcaaaacctctagatgaagtagtagaatggactagggaagcagaattagaa
tatgaagaaaataagttaatagtgcaggaggaagtacatggagtttattatcagccaggaaaacctctgatggcc
aaggtacagaaactgacacagggacaatggagctatcagatagaacaggaagagaataagcctcttaaggtaggg
aagtatgctaggacaaaacatgcccacacaaatgagttaagggtgcttgcaggattagtacaaaaaatagccaaa
gagtgcatagtgatttggggagaattgccaaaattctatctccccctggagagggaagtatgggatcagtggtgg
catgattattggcaggcaacatggatcccagaatgggaattcatctcaacaccaccactaataaggttgtggtat
aacctgttaaaagaaccaataccaggggaggatgtctattatgtggatggggcagcaaatagaaactctaaggaa
gggaaagcaggatactatacagctaggggaaaaagtaaagtaatagccttagaaaacacaactaatcagaaagca
gaattagaggcaatactgttagccttgaaagattcaggtcctagggcaaatatagtcacagactcacaatatgca

ttaggcatactttcagccactccagatcgatcagacaaccctatagttagagaaataatcaaccaaatgatagcc
aaagaagctgtatacttagcatgggtaccagctcataaaggcataggaggcaatgaacaaatagacaaattagta
agcaaaggaatcagacaagtactattcctggaaggaatagataaagctcaggaggaacatgacaaataccataat
aattggagatcattaagtcaggaatttagcataccccctatagtggcaaaagaaatagttgcacagtgcccaaag
tgtcaggtaaaggagaacctatccatggacaggtggatgcggccccaggaacatggcaaatggattgcacccat
ctagaaggaaaggttatcatagtagcagtccatgtggccagtggatatctagaagcagaagtgataccagcagaa
acaggaaaagaaacagcacatttcctgctaaaactagcaggaagatggccagtgaaacatttacatactgacaat
ggtccaaactttgtcagtgagaaggtagccacagtgtgttggtgggctcagatagagcacaccacaggaataccc
tataacccccaaagtcaaggagttgtggaagcaaaaaatcatcatcttaaggtaattatagagcaagttagggat
caagcagagagactggagacagcagtacaaatggcagtactcattcacaattttaaaagaaaagggggatagggg
gagtacagtccaggagaaagaatagtggatataataacaacagatattctaactaccaaattacaacaaaatatt
tcaaaaattcaaaattttcgggtttattacagagaaggaagggagcagctctggaaaggaccagcagaactcatt
tggaaaggagaaggagcagtggtaatcaaagaaggaacagacttaaaagttgtgcccagaagaaaagctaaaata
atcagggataaatggaattcattagtcaaataccacaaatataaggaagaaaaacacttagataagtgggaacta
ttccaccactttcaatgctcaggatggtggacgcactctcaaaagattatccccttcaaagatggctcaaagatt
attattactgccctatggaacttgaccccagaaaaaggatggttatctcaatatgcaatcacagcagaatacaac
aaaggggactattatactcacatagacccagtaacagcagacagaatgattcattgggaatatttcccatgtttt
acagctgccgctgtgagaaaggtgctgtacggagaaagaatagtagcgtgttacagcccctggggacataaaggt
caggtagggactctacaactcttggctttaagagcctacatcaagttttgcagaatggaacagccgccagaggat
gaggctccacaaagagaaccatataatgagtggctgatagatacattagcagaaattcaagaagaagctttgaag
cattttgataggcgcttattgcatgcagtaggaagctggatttatgaaacatatggggacaccttggagggggtg
cagaagcttatcaccctcttacagagagctctatttctgcattttagacatggatgcagggaaagccgaattgga
caagaaggaggcaaatataaccccccttagatcctttccaaggccgaacaaccccttgtaaatgttattgcaagaa
atgttgctatcactgccagtgttgcttcttgcagaaagctttagggatacattatcatgtctacagaaccaggag
aactcggaagagacttctgggaaaaatatcacaatgcttagacatttaatcacagcaatagtaataataggtata
ggtataggaaaacaatgggtaacagtatattatggcacacctaaatggcacccagcaagaacgcatctttttttgt
gcaactgacaacaactccctttgggtaaccacaagttgtgtaccaagcttattgcattatgaggagcaactcatc
cccaatataacagaaaattttacagtccctatacaagagaatgaagtggtaaggcaagcatggggagctatttcc
tctatgatagatgcagttctaaaaccgtgtgtaaaattgacaccatactgtgtcaagatgcaatgtacaaaggga
gaaaacagtagtaccagcactaccactcccatgcccactaccaccactcccaccactccagcagtgacggggctg
gaggtgacggtacagaataatgagagtaccacacaagaaaatagggtatgtaaatttaacacaacaggattatgt
agggattgtaaaatagagattaaagagagctttagatatgaggatgtgacctgtactaaaaaggaaaatgacact
gaaactgaagaatgttatatgacacactgtaatacatcagtaataacacaggattgtaataaggcctcaacagac
aaaatgacatttagattatgtgcccctccagggtacattctgctaagatgtcgtgagaaattgaataaaactaaa
ttatgtgccaatgtctcagcagtacagtgtactgacccaatgcctgctactatatcaactatgtttggctttaat
ggaacaaaacataattatgatgagttaattttggtgaaccctcaaaaggagtttcatgaccataaatatgtttat
agggtgaacaaaaaatggggggttgaaggtggtatgtgataggaagggaaacaggtctatagtttcagtcccaagt
gcatcaggattaatttctatcatgggttagaaccaggcaggaatctgaaaaaaggaatgtgcaggttaatagga
caatggggaagagctttgaacgcattatcacgtgaattaaagaaagtaaatgcaagtatttatagaggttttaat
gcaagtggcccttgcaggggggataaacaatagaggagtggtaaacaaaacagggtgtgccttgaaaacaatagaa
gtaagtaactacaccaccattggcgaaccaggagcagaaacaattatgatcctctgtggaggagaatatttcttt
tgtaattggacaagaatttggaaaacctggaataaccaaaactcaaatgtttggtatccatatatgtcatgtaaa
atcagacagatagtggatgattggcataaagtgggaaggaagatttatatgcctcctgtgtctggatttaataat
catataagatgcactaatgatgtcacagagatgtttttttgaggtccaactagttgaaggaaaaagatacctaata
aagtttctgccacaagatgaggtacaaaatcaattcacagcagtgggagcacattataaattggtaaaagtggat
ccgataggattcgcccctactgatgtacacagatatcatctaccagatgcaaagcagaaggcagtatggggatgc
tcattctcccaagtgtgtcatactaatgttccgtggccaaatgaaagtataactcctaattggacctcagaaaca
tggctggaatgggatagaagagtgacagctatcactaacaatatgacaatcgatttacagagggcatatgaattg
gaacaaaagaatatgtatgaattacaaaaattgggagatttgacttcatgggctagctggttcgacctcacgtgg
tggctcaaatatgttaagataggaatattgataataatggtagtaataggacttagaattttagcttgtttatgg
tctacaataggtaggtttggagactatctccgacctcgcaacctcagacagccagacgaagaaagagaaggagag
ctcaacttagaagaacagaacatcaagtcagagagctccaagaaagaatttggcagaccttggaggccagagcaa
ataaggaattggttgaaggagtcaacaatttacatttggctgaagaatctgcaggcagtaattgaagtagatgat
gagcaaaaagaagaaaggggctttccagtttatcccacacagcctgtgcgtgaagcaacctacaaaaacctgata
gatatgtcccacttttaaaagaaaagggggactggaagggatttggttctctagaagaagagaagagatctta

aacctgtatgctcagaatgaatggggattcataccagattggcaagaatacacttcagggccagggataagatac
ccgaagagatttgggttcttattcaagttaatcccagtacaagtaccaccagaccaggagaaccaggagtgtaac
aggctgcttaactcctctcagctaggaatccaggaagaatccatgggggagaggctgatgtggaagtttga
>#26 KM378564 7386 nt
atgggagcaagcgcgtcaggacttaggggagagaagcttgatgcttttgaaaaggttaggttacggccctccgga
aaaaagaaatacaggctcaagcatcttgtctgggtagcgaaggaactagatcgattcggcctacatgagagatta
ttagaaagtcaggaagggtgccaaaaaattttaggagtactctttcctctagttcctacagggtcggagaattta
atttctctctttaatacctgctgcgtagtttggtgcgttcactcgaaagtgaaagtatcagacacagaggaagct
gttcaaaaggtaaagcaacactgccatctagtggacaaaaatgagaatgcagcttcaaaaaatgaaaatggggaa
acagcgacatctagcggcaggtcaagaaattaccccattcaagttgttaatcaacaggcagtgcaccaggctatt
tcaccaagaactttaaatgcctgggtgaaggtgattgaggaaaagaagttcagtgcagaagtggtgcctatgttt
ttggccctgtcagaaggctgcattagctatgacatcaatcaaatgttgaatgctattggagatcaccaaggtgcc
ctccaaatagtaaaagacatcatcaatgaggaagcagcggactgggataggagacatcctcaagtaggacctctc
ccacaaggggtgcttaggaacccctcagggtcagacattgcagggaccaccagctctatagaagagcagatagaa
tggaccaccagagcccaagatgccatcaatgtgggagcaatttataggcaatgggtggtcttagggttgcaaaga
tgtgtaacaatgtataatccagtcaacatcttggatgtaaaacagggtcctaaagaaccattcaaggactatgta
gatagattttataaagccctcagagcagaacacacagatgcagcagtaaaaaattggatgacacaaacgctgctg
atacagaatgcaaatccagactgtaaagtgattctcaaaggcctagggatgaaccccagtctggaagagatgctg
ctggcttgccaaggagtaggaggaccaggccataaggcaagggtcatggcagaagctatgaaagaacaacaagca
gctataatgatgcagcagaattccctcagaggtccaccgcggggacccaggggacccccctccaagaaaccctaaa
tgtccaaactgcgggcagttcggacataacctaagacaatgtacaaagcctagaaaaaagggatgctttaggtgt
ggagctttagatcatatgttaagaaattgcccaaaacaggtgaatgagatagaagggcaaaaggtggaggcttta
ctagacacaggagcagatgatacagtaattcaaggattagaattaacaggaaaatggaagccacaaattatagga
ggaattggaggagctattaatgtgaagcaatattttaattgtgaaatcaaggtagcaggtaaaattactcatgct
tcagtgttggtaggccctacccctgtaaatattataggtagaaatgtgctgtgtaaattaggagctactcttaac
tttcctgttagtcaagtagaaccagtaaaagtaacactaaaaccaggaatggatgggccaaagataaaacaatgg
cccctctccaaggaaaaaattgaagccttaaaagagatatgtgagcaaatggagaaggaaggtcaaataagtaga
ataggtccagaaaatgcatacaacacaccggtgttttgtattaaaaagaaagatggtaccaaatggagaaaatta
gtagatttcaggcagttaaataaagcaacacaggacttctttgaggtgcagttggggataccacacccggagga
ttaaaagagtgcgaacagatcacagtactggatgtaggtgatgcctacttctcatgccctctggatccggacttt
agaaagtacactgcattcaccattccatcggtgaataatcagggcccaggaatcaggtatcagtataatgtcctc
ccacagggatggaaaggctcacctgcaatcttccaagctacagctgacaagatcttacagccattcagagagaaa
aatccagatgtgattatttatcagtacatggatgatctgtttgtaggaagtgatagaccaaagcatttacatgat
cagatgattaaagaattgagaacacatttaaagttctgggggctcgagaccccggacaaaaaatttcaggacaat
cctccttatgagtggatgggataccagctgcatccaaagaaatggacagtgcaagaaattcgtttgcctgataaa
gaaatttggactgttaatgatattcagaaattagtaggaatactcaattggcctagtcaaatttattccggaatt
aaaacaaaagagctctgtaaattaattaggggagccaagcccctggatgaagaagtagaatggtctagggaagca
gaactagaatatgaagaaaacaaactgatattaaaagaacaaatgcatggggtatattatcagccagaaaagccc
ctaaaagccaagatacaaaaattgggcaatggacaatggtcatatcagatagaacaagatgacaacaagccacta
aaaacaggaaaatatgctaaggtaaagaatagtcacaccaatgatatgaggatgttagcaggactagtgcaaaag
gtagcaaaagaagcactggtaatctggggaagattgcccatttctacctaccggtagaaagggaggtttgggaa
caatggtggcaggagtactggcaggtaacatggattcctgattgggaatttgtatcaacacccccctctcatcaag
ttatggtataacttgttgagtgacccaataccaggagaagaagtttattatgtggatggagctgctaacagagtt
agcaaagaaggaaaagctggctatgttacctccaggggaaaagaaaaggtaatagccttagaagaaaccaccaat
cagaaggctgaattacaggctattctgttagccttgaaagactctggtccaaaagtcaacatagtgacagattca
caatatgccatgggcataatatcttcctccccagaaatcagtgacaaccctatagtgaatcaaattatagaacaa
atgatcagtaaagaagcagtttacttaaattgggtaccagcacataaagggataggaggtaatgaggaggtggac
aaattagttagtagaggaataagacaagtgttattcctagataatatggaaaaggcacaggaagaacatgattta
taccataataattggagatccttagcacaagaatttggtctaccagggatagtggcaaaagaaatagtggcacag
tgtcccaaatgtcaaatacatggagagccaatacatggccaagtagatgcaagcccaggaacttggcaaatggac
tgcacacatctagaaggaaaaataatcatagtggcagtccatgtagcaagtggatacatagaagcagaagtaata
ccagcagaaacaggagagaaaacagcttactttctgctaaagttagctggcagatggccagtatcacacttgcat
acagataatggaccaaatttcaccagtgaaaaggtagcaacagtatgctggtgggctaaaatagagcataccaca
ggagtaccctataaccccccagagtcagggagtggtagaatcaatgaacaatcagctaaagaaaattataggacaa
atcagagatcaggcagaaaaactagagacagcagttcaaatggcagtactgattcacaattttaaaagaaaaggg

gggataggggagtacagtgcagcagaaagaatagtagacatcatagccacagatctcttaacaagcaaactacaa
caaaatattctcaaaattcaaattttcgggtttattacagagagggaagagatcagttgtggaaaggaccagca
gaacttgtctggaaaggggaaggagcagtggtcatcaaggaaggtactgacttgaaagtaatacctagaagaaag
gcaaagatcatcaaagataggtacatggtagagagatggaactccctgtgtaaatatcacaagtataagggagaa
aagtatttagaaagatggcattatgcacctcacttccaatgctcaggttggtggactcatagtcagtggaccatc
cccttccaagacaaaagtaaaatagtagtaacagtcctgtggaacctcaccccagacaaagggtggttaagtaag
tatgccataacaatagaacatattcaggagaagttttgcaccttcatagacccaacaacagcagataaaataatc
cacttggaatatttcccttgttttacagatcaagcaattagaggtgtcctgctgggatttagaattcaagcttgc
tatttccctagagggcataagggtcaggtagggagtcttcagtatcttgccttgcaagcacacataaaatatctt
aagatggaaaggccacctgaggatgaaggaccgccaagggaaccctatgatgagtggctagtagatactttagta
gaaatacaagaggaagctttgaagcattttgataggcgcttactgcataatgtaggatcatgggtctatgaaaga
tacggagattcattagaaggagtacagcagcttataacaatcttgcaaagagcgcttttcttgcattttagaatg
gatgccaggaaggtagacttggaccagcaggacgctggaatccatttcgaacctttccaagggcgaaccagcaat
tgtatgattagacatatagttataggcttatgcattttaggattttaggattaagtataggaaaacattgggta
acagtgttttatggaacccccaagtggagaccagcatccacacatctgatctgtgctacagataatcattcattc
tgggtaactacaagttgcataccaagtctattgcattatgaagaaacacaaattgaaaatatagaagaaaatttt
actgtccctatgacaaaaaatgaagtgataaaacaagcatggggtgcattatcatctatgatagatgcagtgctt
aaaccatgtgtcaaaatcaatccttattgtgtaaaaatggaatgtactgatggcccaccacaaccaacaacaact
acagcaactagtactagtactactagtgctgccccaaatacaacagcagatttggagatagacagaaataacaca
gaaccaaaaacagaaacaaatagagtatgtaagtacaatgtgacaggattgtgtagggactgtaaagaagagatc
acagaaaatttaggtatgatgatgtagtttgtaaatgtggcagtagagctgaatcaccagaagagaacaactgt
actgaaagtagaatatgttatgttcaccattgcaactcaaccgtgattactcaggattgcaataaggcttctact
gatatgatgaaattcagattgtgtgctccaccaggatatatatttctaagatgtaatgagaagctaaataaaact
agaaagtgtagaaatataacagcagtgcagtgtactagacacatgcctgccacaatctctagcatgtttggcttc
aatggcacaaagcatgatcaagatgagttaatagagacaaggggaagaccagactttattgatcataaatacgtg
tttagggttgataggaaatggaaccttagaattctctgtagaagaaaaggaaataggagtatagtgtcaacgccc
tcagccacagggttgttgttttatcatgggttagaaccaggaagaaatctaaaaagaggaatgtgtatatttcag
ggacactggggaatggctttagaaagcctagccaaagaattgaaaaaagtaaatgcatccatttggagaaatact
aataagaccagccaatgtaacgggctcaaaggaaagacaaacgcaacagggtgcctgttgaagcagcttagggta
gataattacaccgccgagggagatctggcatcagagaacctcatgatgatgtgtggaggagaatatttcttctgc
aatgttactaaaatatggaaaacatggaataatagatcaagtagtgtgtggtatccatatgcatcctgccacata
aaacaaataatagatgattgggcaaaagttggcaggaaaatttatatgcctcctgtatcaggatttaataatgag
ataagatgtacacaagaagtcacagaaatgttctttgaggttcaccgtttggacacaaatgtaaccgatgattct
cattatcagattaaatttataccacaagatgaagtgcaaaatcagtccacagcagtaggagcacattacaaattg
gtcaaggtggagccaattggctttgctccaactgacgtgcatagatacaacctgccggaacacaggcagaaaagc
ctctggggatgctcctttgcacaagtatgtcacacaaatgtagagtggcctaatgaaacagtgactccaaattgg
acctcggagacatggatggagtggcaaaaaagagtggatagtatatcaaacaacattactcttgatttacagaag
gcatatgaacaagaacaaaagaatatctttgaattgcaaaaattaggagatttaacatcatgggctaactggttt
gattttacttggtggtttaaatatattaagataggattctttgtagtgatagtaataatagggttaagaatctta
gcagctttatggagtacagtaggtaggtttaggcagggttatcgccctcttccttatatctttaagagcttagat
actactcttatgcaagcagcagcagcagcagagaaagatgtcacccttcccactgaagaagaacaaccctcagaa
gaagaagaagaagtaggctttccagtatatcctcgatgtcctctaagagaaccaacatataaagatctggtagac
ttttcccactttttaaaagaaaagggggactgaagggatttggtggtctcacaagagagagacaatcttggac
ttatatgctcaaaatgaatggggcttcataaaaggctggcaaagctacaccagaggacctggtatcaggtaccca
aaagtgtttgggttcctgttcaagctggtccctgtggaggtagatgaagatgtggtaaatcagccgtgcaacagc
ctgttaaactccagccaaatgggtcctattgatgacccaaacggagaaagactcatgtggcagtttgatccaacc
ctggcatattccttcaaggcttgtattcaccatccagaggagtttggacatgctagaagtatagagcacatagca
gaggagccagcctgctgcaagaggaagtggtggcag
>#27 KM378563 7431 nt
atgggagcaagcgcgtcaggacttaggggagagaagcttgatgcttttgaaaaggttaggttacggccctccgga
aaaaagaagtataagctcaagcatcttgtctgggtagcgaaggaactagatcgattcggcctacatgagagatta
ttagaaagtcaggaagggtgccaaaaaatttaggagtactctttcctctagttcctacagggtcagagaatcta
atttctctctttaatacctgctgcgtagtttggtgcgttcactcgaaagtgaaagtaacagacacagaggaagct
gttcaaaaagtaaaacagcactgccatctagtggacaaaaatgagaatgcagcttcaaaaaatgaaaatggggaa
acagcgacatctagcggcaggtcaagaattaccccattcaggttgttaatcaacaggcagtacaccaggctatt

tcaccaagaactttgaatgcctgggtgaaggtaattgaggaaaagaagttcagtgcagaagtggtgcctatgttt
ttggccctgtcagaaggctgcatcagctatgacatcaatcagatgttaaatgctattggagatcaccaaggtgct
ctccaaatagtaaaagacatcattaatgaggaagcagctgactgggataggagacatcctcaagtaggacctctc
ccgcaaggggtgcttaggaacccctcagggtcagacattgcaggaaccaccagctctatagaagagcagatagag
tggaccaccagagcccaggactccatcaatgtgggagcaatttatagacaatgggtggtcttagggttgcaaaga
tgtgtgacaatgtacaatccagtcaacatcttggatgtgaagcaaggtcctaaagaaccattcaaggactatgta
gatagattctacaaagccctcagggcagaacatacagatgcagcagtaaaaaattggatgacacaaacgttgctg
atacaaaatgcaaatccagactgtaaagtgattctcaaaggcctagggacgaaccccagtctggaagagatgctg
ttagcttgccaaggtgtaggaggaccaggccataaggcaagggtcatggcagaagctatgagagaacaacagaca
gctataatgatgcagcaaaatcccccaggggtccaccgcggggacccaggggacccctccaagaaaccctaga
tgtccaaactgcgggcagtatggacataccctaagacaatgtacaaagcctagaaaaaagggatgctttagatgt
ggagctttagatcatatgttaagaaattgcccaaaacaggtgaatgaaatagaagggcaaaaggtagaggcttta
ctagacacaggagcagatgatacagtaattcaaggattggaattaacaggaaaatggaagccacaaattatagga
ggaattggaggaaccattaatgtgagacaatattttaattgtgaaatcaagatagcagggaaaattactcatgct
tcagtgttagtaggccctacccctgtaaatattataggtagaaatgtactctgtaaattaggagctactcttaac
tttcctgttagtcaagtagaaccagtaaaagtaacactaaaaccaggaatggatggaccaaagataaaacaatgg
cccctctccaaggagaaaattgaagccttgaaagaaatatgtgaacaaatggagaaggaaggtcaaataagtaga
ataggtccagaaaatccatacaacacaccggtgttttgtattaaaaagaaagatggtaccaaatggagaaaacta
gtagatttcaggcaattaaacaaagcaacacaggacttctttgaggtgcagttagggataccacacccaggagga
ttaaaagcatgcgaacagatcaccgtactggatgtaggtgatgcctacttctcatgccctctggatccggacttt
agaaagtacactgcattcaccattccatcggtgaataatcagggcccaggaatcaggtatcagtataatgtcctt
ccacagggatggaaaggctcacctgcaatcttccaagctacagctgacaagatcttacagccattcagagagaag
aatccagatgtgattatatatcagtacatggatgatctgtttgtagggagtgatagaccaaagcatttacatgag
cagatgattaaggaattaagaacacacttaaagttctgggggcccgagaccccagacaaaaaatttcaggacaat
cctccttatgagtggatggggtaccagctgcacccaaagaagtggacagtgcaagaaattcgcttgcctgataaa
gaaatttggactgttaatgatattcagaaattagtaggagtgcttaattgggctagtcaaatttattccggaatt
aaaacaaaggagctctgtaaattaattaggggagccaaacccctagatgaagaagtagaatggaccagggaagca
gaactagaatatgaagaaaacaaactgatattaaaagaacaaatgcatggggtatattatcagccagaaaaaccc
ctaaaggccaagatacaaaaactgggcaatggacaatggtcatatcagatagaacaagatgacaacaagccacta
aaaacaggaaaatatgctaaggtaaaaaatagtcacaccaatgatatgagaatgttagcaggactagtgcaaaag
atagcaaaagaggcactggtgatctggggaagattgcccactttctacttaccagtagaaagggaagtttgggaa
caatggtggcaggagtactggcaggtaacatggatcccggattgggaattcgtatcaacaccacatctcatcaag
ttatggtataatttgctgagtgacccaataccaggagaagaagtttattatgtggatggagccgctaacagagtt
agtaaagaaggaaaagctggctatgttacctccaggggaaagagaaggtgatagccttagaagaaaccaccaat
cagaaggctgaattacaggccatcctgttagccttaaaggactccggtccaaaagtcaacatagtgacagattca
cagtatgccatgggcataatatcatcctccccagaaatcagtgacaacccgatagtgaatcaaattatagaacaa
atgatcagtaaagaagcagtttacttaaattgggtaccagcacataaaggaataggaggtaatgaggaggtggac
aaattagttagtagaggaataagacaagtgttatttctagataatatggaaaaagcacaggaagaacatgattta
taccataataattggagatccttagcacaagaatttggtctaccagggatagtggcaaaagaaatagtggcacaa
tgtcctaagtgtcaaatacatggagaaccaatacatggccaagtagatgcaagcccaggaacttggcaaatggac
tgcacacatttagaaggaaaaataatcatagtggcagtccatgtagcaagtggatacatagaagcagaagtaata
ccagcagaaacaggagagaaaacagcctactttctgctaaaattagccggcagatggccagtgtcacacttgcac
acagataatggaccaaatttcaccagtgaaaaggtagcaacagtatgctggtgggctaaaatagagcataccaca
ggagttccctataaccccccagagtcaaggagtggtagaatcaatgaacaatcagttaaagaaaatcataggacaa
attagagatcaggcagaaaaactagagacagcagttcaaatggcagtactaattcacaattttaaaagaaaaggg
gggataggggagtacagtgcagcagaaagaatagtagacattatagcctctgatctcttaacaaataaactacaa
caaaatattctcaaaattcaaaattttcgggtttattacagagagggaagagatcagttgtggaaaggaccagca
gaacttgtctggaaggggaaggagcagtggtcatcaaggaaggtactgacttgaaagtaatacctagaagaaaa
gcaaagatcattaaagataggtacatggtagaaaggtggaactccctgtgcaaatatcataagcataaggagaa
aagtatttagaaagatggtattatgcacctcacttccaatgttcaggttggtggactcatagtcagtggaccatc
cccttcaaagacaagagtaaaatagtagtaacagtcctgtggaacctcaccccagacaaagggtggttaagtaaa
tatgccataacaatagaacatattcaggagaagttttgtaccttcatagacccaacaacagcagatagaataatc
cacttggaatatttcccttgttttacagatcaagcaataagaggtgtcttactgggatatagaattcaagcttgc
tatttccctagagggcacaagggccaggtagggagtcttcagtatcttgccttgcaagcacacataaagtacctt
aggatggaaaggccaccggaggatgaaggaccgccaagggaaccctatgatgagtggctagtagatactttagta

gaaatacaagaggaagctttgaagcattttgataggcgcttattgcataatgtaggatcatgggtctatgaaaga
tatggagattcattagaaggagtgcagcagcttataagaatcttgcaaagagcactttcttgcattttagaaaa
tgctggtgcaaacactgcgcttaccactgtcagctttgctttcttcaaaaaggcttagggatacgttatatgatt
agacatatagttataggcttacgcattttaggattttaggaataagtataggaaaacattgggtaacagtgttc
tatggaacccccaagtggagaccagcatccacacatctgatctgtgctacagataatcattcattctgggtaact
acaagttgcataccaagtttattacattatgaggaaacaaaaattgaaaatatagaggaaaattttactgtccct
atgacagaaaatgaagtgataaaacaagcatggggtgcgctgtcatctatgatagatgcagtgcttaagccatgt
gttaaaatcaatccttactgtgtgaaaatgatatgtaatgataccaaagaaataccaactacaacaacagtagct
acaacagtggctactactactagtactacagctccaaaaaatgctagtgttgccccaacaacagcaaatggcact
ttggatatagatacaaataatacagaaaaagtaacagaagcaaacagagtatgtaagtacaatgtgacaggattg
tgcagggactgtaaggaagaaatcacagaaaattttaggtatgatgatgtagtttgtaaatgtggcaatgtaact
gaatcaccagaaaataataattgtaatggtacaagaacatgttatgtgaccagttgcaactcaacagtgattacc
caggattgtaataaggcctccacagatatgatgaagttcagattgtgtgctccaccagggtatatattgctaaga
tgtaatgaaaaactgaataaaagtagtaagtgtaaaaatataacagcagtgcagtgtactagacacatgcctgcc
acaatctctagtatgtttggcttcaatggcacaaagcatgatcaagatgagttaatagaaacaagaggaagacca
gaatttgtggatcataaatatgtgtttagggttaataagaaatggaacctcaaaatcatgtgtagaagaaaagga
aacaggagtgtaatatctacaccctcagctacagggttgttgttttatcatgggttagaaccagggaaaaatcta
aacagaggaatgtgtaaatttgagggacaatggggaaaggctttagaaagcttagccaaagaattaaaagcagta
aatgcctccatttggagaaatactactattaaagggaaagaatgtaaacgtctcaaagaaaccaaagacacaaaa
acagttgggacagggtgcatgttgaagcagcttaaggtagataattacaccaccaaggggggatctagcatcagag
aacctcatgatgttgtgtggaggagaatacttcttctgtaatatctctaaaatatggagaacatggaataataga
tcaagtaatgtgtggtatccatatgcatcctgccacataaaacaaatagtagatgattgggcaagagtgggcagg
aaaatttatatgcctcctgtatcaggatttaataatgaaataagatgtacacaagatgtcacagaaatgttcttt
gagatccaaaaattggagacaaatgaaatcaatgattccaactatcagattaaatttgtgccacaagatgaagtg
caaaatcagttcacagcaataggagcgcattacaaattggtcaaggtggagccaattggctttgctccaactgat
gtacatagatacaacctgccggaacacaggcagaagagcctctggggatgctcctttgcacaagtgtgtcacaca
aatgtggtgtggcctaatgagtcagtgactccaaattggacctcagagacatggatggagtggcaaaaaagagta
gatagtatatcaaacaacattactcttgatttacagaaggcatatgaacaagagcaaaagaatatttttgagtta
caaaaattaggggacttaacatcatgggctaactggtttgatttcacttggtggtttaaatatattaagatagga
ttctttatagtaatagcaataataggattaagaattttagcagctttatggagcacagtaggtaggtttaggcag
ggttatcgccctcttccttatgtctttaagagcttagatactactcctatacaagcagcagcagcagcagagaaa
gatgtcacccttcccacagaagaagaacaaccctcagaagaagaagaagaagaagtaggctttccagtatatcct
caatgcccgttgagagaaccaacttacaaagagttagtagacttctcccacttttaaaagaaaggggggactg
aaagggatttggtggtctctcagaagagaatcaatcttggatctttatgctcaaaatgaatggggcttcataaaa
ggctggcaaagctacaccaagggacctggcatcaggtacccaaaggtatttgggttcctgttcaagttggtccct
gtggatgtagatgatgacctgatagatcagccgtgtaacaggctgttgaactccagccagatgggtcctattgat
gactcagaaggagaaagactcatgtggcagtttgatgcaagcctggcatacacctttcaggcttgtattcaccat
ccagaggagttcggacatgttacaagtataagacatgataaaagagaagagccagattgttgcaagaggaagtgg
tggcag
>#28 M27470 7710 nt
atgggtaatgggaactctgccttgttagggactgatttggataaatttgagaaaataagattaaagagaggtggt
aaaaaatgttatagattgaaacacctctgttggtgtaaaggtgaattagatagatttggcttatcggataaactc
cttgaaacacagcaaggatgtgaaaaaatcctctcagtatgttggccattatatgaccaaggatcagataatcta
aaagctttggtagggacagtctgtgttgtagcctgcatacacgcaggtatagaaattaagagcacacaagatgct
ttaaaaaaattaaaagtcataacaagaaaggaagaaaagcaggaggatgaaagtaagaatttccctgtacaaagg
gatgcagcaggacagtatcagtatactccaataagtcctaggattatacagacatgggtaaaaacagtggaagaa
aagaagtggaaaccggaggtcatccctctattctcagcattgacagaaggagcaatcagtcatgatttgaatatc
atgctgaatgcagtaggagatcatcagggagcaatgcaagtcttaaaagatgtaattaatgagcaagcagcagaa
tgggatctaacacatcctcaacaacaaccagcacaaccaggaggaggattaaggaccccttcaggctctgatata
gcaggaactacttctacagtggaagaacaattggcatggatgaatatgcaacaaaatgcaatcaatgtaggaaca
atctataagagttggattatactgggcatgaatagattggtaaaaagtcattgtccaataagtataacagatgta
agacagggaccaaaggaagcttttaaagactatgtagatagattctacaatgtaatgagagcagaacaagcttca
ggagaagtaaagatgtggatgcagcagcatctgcttatagaaaatgcaaacccagaatgcaagcagattttgaga
agcttagggaaaggagcaactttagaggaaatgttggaagcatgtcagggagtaggtggccacaacataaagcc
agattaatggcagaaatgatgagaacagtggtaggacaatcacaaaattttgtgcagcagagagggcctcaaaga

ggaccagttagacaacctactggaaggaaacctatctgcttcaactgtaataaagaagggcatgtagcaaggttc
ttcaaggcccctagaaggaaagggtgctggaattgtggagcaatggatcatcagaaagctcaatgccctaagcca
gctcagcagcagagggttgaggtctcagtggatggtgtcactataagagctctactagatacaggagctgatgat
accatctttaatgaaagaaatataaaattaaaaggaaattggcagccaaaaattataggggaataggtggaaac
ttaagagtaaaacagtatgataatgtatatgtagaaataagagggaagggaacatttgggacagtattgatagga
cctactccaatagatataatagggagaaacataatggaaaaattaggaggaaaattaatattggcacaattgtct
gataaaataccaataacaaaagtgaaattaaaaccaggagtagatggacccagaataaaacaatggcctttaagt
aaagagaaaatagttggtcttcagaaaatatgtgatagattagaggaggaaggaaaaattagtagggtagatcca
ggaaataattacaatacacctatctttgccataaagaagaaggataaaaatgaatggagaaaattaatagacttt
agagaattaaacaagttaacacaggattttcatgaattacagttaggtatacctcacccagcaggaataaaaaag
tgtaaaagaataacagtcctagatataggggatgcctattttagtatacctctggatccagattatagaccctat
actgcctttacggtaccatcagttaataatcaagcaccaggaaaaagatacatgtataatgttcttcctcaaggg
tggaagggaagtccatgtatctttcaagggacagtagcatcactgctggaggtatttagaaagaaccatccaaca
gtacagttatatcaatacatggatgatttgtttgtagggtcagactatacagcagaagagcatgagaaagctata
gtagaattaagggctttattaatgacatggaacttagaaacacctgaaaagaaatatcagaaagaacctcccttt
cattggatggggtatgagttacacccagataagtggaagatagaaaaggttcaactaccagaattagcagaacag
ccaacagtaaatgaaatacagaaattggtaggtaaattaaattgggctgcacagttatatcctgggatcaaaaca
aaacaactgtgcaagctaataagaggaggactaaacataacagagaaagtcacaatgacagaagaagcaagactg
gaatatgaacaaaataaagagatcttggctgaagaacaagaagggtcttattatgatcctaataaggaattatat
gtaagatttcagaaaacaacaggaggagatatatcatttcaatggaagcaaggaaataaggttttaagagcaggg
aaatatgggaaacagaaaacagcacatagtaatgacctcatgaaattggcaggtgctacgcagaaggtaggaaga
gaaagtatagtaatctggggttttgtaccaaaaatgcagatacccactacaagggagatatgggaagattggtgg
catgagtattggcagtgtacatggataccagaagtagaatttatcagcacacctatgttagaaagggaatggtat
agcttgtccccagaacctctagaggggtagaaacatattatgttgatggagcagctaacagggacagtaaaatg
ggaaaagcaggatatattacagatagaggttttcaaagggtagaagaatatctaaataccaccaatcagcagaca
gaattacatgcagtaaaactagctctagaagatagtggaagttatgttaacatagtaacagattcacaatatgta
gtaggtatactagcaagcagacctactgaaacagatcaccccatagtaaaggaaataatagaattaatgaaagga
aaagaaaaaatttatttaagttggctaccagcacacaaagggataggagggaatgagcaaatagataagctagta
agttcaggaatcagaaaagtcttattcctacaaaatatagaaccagcacaggaagaacatgagaaatatcatagc
aatgaagcacaattaagagagaaattccacttaccagctctagtagccaaacagattgtgcaaagttgcagtaag
tgctgtcatcatggagagcccataaagggacagacagatgcttcacttggagtctggcagatagattgcacacat
ctggaaaatcaaattattatagtagcagtgcatgtagcttcaggcttcatgaaggcagaagttataacagcagaa
actggaaaaaagacagcagagtttctgttaaagttagcagcacaatggcctattagtaaactacacacagataat
gggcctaactttactagtcaggaagtagaaaccatgtgttggtggttagggatagaacacacatttggaataccc
tataacccacaaagtcaggggtagtggaaaataaaaataagtatctaaaagaattgattgagaaaataagagaa
gattgcaaagaattaaaaacagcagtagccatggccacattcattcataattttaaacaaagggaggactaggg
gggatgacagcaggagagagaatagtaaatatgatcaatacagaattagaatatcaatatcaacaaaatcaaatt
tcaaaaaatttaaattttaaggtttacttcagagaaggaagagatcagctgtggaaaggacctggtatcctttg
tggaaaggagaaggggcagtagttttaaaatatcaagaagagataaagatagtacctagaagaaagtgtaaaata
ataaaagatcagagaatagaaaagtggcactggttagtaagaagacagatggcatgggccactgcaaataatgag
gaaggatgttggtggctgtatcctcattttatggcttataatgaatggtatacttgcagtaaagtagtgattata
ataaatagggacataagattaatagttagaagctattggcatttgcaaatagaggtaggatgcttaagtacttat
gcagtaagcatagaagcagtagttagaccgccaccctttgagaaagagtggtgtacagagataactccagaggta
gcagatcatctaatacatttacattttatgactgcttcatggacagtgcagttatgaaagccatcaggggagaa
gaagtgttaaaagtttgtagatttccagctggccataaagcacaaggtgttctctctttgcagtttctctgcttg
agagtcatccaagtatcagaagatcaaggacctcccagagagccatacaatcagtggctagcagatactatggag
gaaataaaggaagaagcaagaaagcacttccctctcattatcctaaatgcagtatcagaatattgtgtgcaaaac
acagggagtgaggaagaggcctgtgagaaatttattaccttaatgaatagagccatttgggtccacctagctcaa
gggtgtcaactgctagaagagtattatcagcctttgcaagcttgtgagaatatgtctacaggaaacgtgtaccag
gaactaataagaagatacctggtagtggtgaagaagctatacgaaggtaagtatgaagtgtccaggtctttttct
tatactatgtttagcctactagtaggtattataggaaaacaatatgtgacagtcttctatggagtaccagtatgg
aaggaagctaaaacacatttgatttgtgctacagataattcaagtctctgggtaaccactaattgcataccttca
ttgccagattatgatgaggtagaaattcctgatataaaggaaaattttacaggacttataaggaaaatcagata
gtttatcaagcatggcatgctatgggaagtatgttagataccatacttaagccatgtgtaaagattaacccatat
tgtgttaagatgcaatgtcaggaaacagaaaatgtatcagcaacaacagctaagcctataactacacctactact

acatctacagttgcaagtagtacagagatttacttagatgtagataaaaataatacagaagaaaaggtagagagg
aatcatgtatgtaggtataacataacaggactatgcagggattcgaaggaagaaatagtaacaaattttagaggg
gatgatgtgaaatgtgaaaataatacttgctatatgaatcattgtaatgagtcagttaatacagaagactgtcag
aagggacttttgataagatgtattttaggttgtgtgcctccaggatatgtcatgttaagatataatgagaagtta
aataataataaattgtgtagcaatatatcagcagtgcagtgtactcagcacttagtagccacagtaagtagcttt
tttggctttaatggaactatgcataaggaaggagaattgatacccatagatgataaatatagggggcccagaggaa
tttcatcaaaggaagtttgtctataaggtgccaggaaaatatggcttaaagatagaatgtcacagaaaaggaaat
aggtcagtagtgagtactccatcagctacaggattattattttatcatgggttagaacctggaaagaatttaaag
aaaggcatgtgcaccttcaaaggacgttggggttagcactttggagtctagctaaagaactaaataaattaaat
gactccatcaaagtgaaccagacctgtaaaaattttactagcactggagaggagaacaaacaaaacacggacaag
caaaaggagtttgccaaatgcataaagactcttaagatagataattatactacatcaggagatagagcagcagaa
atgatgatgatgacatgtcaaggtgaaatgttcttctgtaatgtaacaagaatcatgagggcatggaatgatcct
aatgagaagaagtggtatccttatgcctcatgtcaaattaggcaaatagtagatgactggatgcaagtaggaaga
aagatatatttaccacctacatcaggatttaataatcacataaggtgtacacatagggtaacagaaatgtacttt
gaaatgcaaaagatagatagtaatgaaacaaaaatgcaaattaaattcttgcctcccagtgaaacctccaatcaa
tttgttgcttatggagctcattataaattagtcaaaataatgccaattggcatagcacctacagatgtgaaaaga
cacactttacctgaacatcataaagagaagagaggagcagtaatacttggtatccttggtctgctctcgctggca
ggatccgcgatgggctcagtgtcggtggcactgactgtccaatctcagtctttggtgactgggatagtggaacaa
caaaaacagttgttgaagctcatagagcaacagtctgaactcttaaaactcaccatatggggagtaaagaattta
cagactcgcctgaccagtttggagaattatatcaaggaccaagctttgctgtctcaatgggggtgttcatgggca
caggtgtgtcatacttctgtagagtggactaatacaagcatcactccaaattggacatcagaaacttggaaggaa
tgggagacaagaactgattatctgcaacaaaacattacagaaatgttaaaacaggcatatgatcgagagcaaaga
aacacatatgaattacagaagttaggagaccttacatcttgggcaagttggtttgactttacttggtgggttcaa
tacttaaaatggggagttttcttagtgttaggaattataggattaagaattttgttagccttatggaatacaata
agtaggtttaggcagatgggttcctcgcagtccaagaagcgatcagaagcttgggttcgctactcgtcagctttg
cggcaattagttggagggccggttacaccggatggctacaagcaaatagaatcttcacagggtgcagagaagcaa
tcattgctgcggggacgtgcatatggcacatactcagaaggattagacaaagtgcagaacgaccccttaactaaa
gatgagaaacttgacttaacacagcaggatccagaagaggaggaagaagttggatttcctgtgtgtcgccaagtt
tccttaagagtgccatcatacaaagatctgatagacttctctcattttataaaagaaaagggggactgggaggg
atatattatagcaggagaagagaagaaatcctagatctctatgcagagaatgagtggggatttgaacctggatgg
caacagtatacgacaggtccaggaaccagatatcctaagacatttggattcctgtttaagctggaaccagtgagc
agagctataggagatgagtatgcagctaacaatcatctgttacactcctcccagttatgtcctcaggaagatcca
gaaggagagaccctcatgtggtctgggaccctcatcttgcctatgactttgcagcattaa

>#29 AF131870 7113 nt

atgggagcgggaaactccacagtggatagggaagttgtgaggagctttgaaagagtagctctgaaacctggaggg
aaaaagacctaccaggcaagacatgtagtatgggcaggtaaagaattagataggtttggacttaacaaggaactg
ttaagaacagtggaaggctgtcagaagattctgtcagtctgttggcctttgtatgcatctggttctaagaacctg
aaggccttagtaggtacagtatgtgtgcttgcctgttgtcatcaaggcattccagtaaaagacacacaggaggct
ctaaaaaaggtcagactagagccagcaaaaaatgaagaagcacaagcttctcaaaattttccagtgcagagagag
ggacaaaattacattcatcagcctctctcgcctagaacagtgcaaacatgggtgaagatagtggaagagaaggga
tggaaaccagaaacagtggcaatgtttgcagctttaatgacaggagcaattccagaagacatcaatgtgatgctg
aatgccataggggagcatcaaggagctatgcagatgataaaagatcatatagtggaggaaagtgcagaatgggac
agaatgcaccctcagcagcagcctgcacagccaggaggaggcctaaggactcctaatggtacagacatagcaggg
gtaacatcaacagtagaagagcagatacagtggatctatggccttaatggagcaacaagagttgcagtacaagac
atctataaaggctgggtcatagagggcatggaaagagtagttagactccatcagactacctcagtagtagaaatt
agacagggaccaaaagagcctttcagagactatacagataggttcttcaaggctttaagagcagaatcaggctca
gaggaagtaaaagagtggatgaaagagaaaatgctagtgcaaaatgcaaacccagattgtaaattggtaataaag
gcactgggagaaactccatcattagaggagatgatgagagcctgccaaggggttgggggccgagccataaagga
aagatcctggcagaagcaatggcctctgcttttaggcagcaagtaggaagacaagctatggtgcagcaaaactta
ccacccagaaacagccaggggaggtttgtaagaataggaggaggaggacctaggaagcccatgacttgttttaat
tgtggtaaaccaggacatctagcaaatcagtgcagagagccaaagaaaggaccacccggctcctgctggaaatgt
ggaaagatgggacataaggtcttcattaatgggcaacctgtgactgctttattggacacgggagcagatgacaca
atagtgtctgaagattctgtgtccatagaaggtcactggaagcccagggtagtgggtggtataggggacaaatt
agagtaaaagaatatgcagatgtctttgtagaaattagggataaaacagctacaggaacagtgttggtggggcca
acaccagtagatatcatagggagaaatattctttcagccttaggggaagattagttttagccactcttagtgaa

aagatcccaattacaaaagttaagttaaaagaaggggccgctggaccaaaagtgaaacaatggcccctgtcaagg
gagaaaatagaaggcctacagaaaatttgtgatagattggaagcagaaggaaagattagcagggcagacccaggg
aacccttataacacaccaattttgctataaagaagaaagacaaaaatgaatggagaaagttaatagattttaga
gtactaaatggtatgactcaagactttcatgagttacagttagggattcctcatccagcaggattaaagaagtgt
aaacagattacagtagtagatgtgggggatgcatacttctcaataccattggatccagattatagaaagtataca
gcattcacaataccgtcagtaaataatcaggcaccaggaaaaagatatgtgtataatgtcctaccacagggctgg
aaaggaagtccttgcatctttcagggaacggtagcgagcctcttagagaaatttagaagacagtatccagaagtt
caattgtaccaatacatggatgatttactcataggatctgattatgagaaaaagaagcatgaagaaatagtgaaa
caattgaggcagctactcatggaatggaatttagaaacaccagagaaaaaatatcagggagagccaccctataag
tggatggggtacatattacacccagacagatgggagatagagaagataaaattacccccctctagaagaagagcca
acagtgaatgacatacagaaaattgtaggagttatcaattgggcttcacaattatatgaagggctcagaacaaaa
gaattatgtaaactaattagagggaataaaccattagcagagaaagtaaagatgacagaagaagctagggaagaa
taccagagtaatcaggaggtattacaggagtcagtatcaggcagttactatgaaccagataaagaattaatttgc
agagtgcaaaaggtaaagcaaggaatattaacctttcaatggttgcagggaaaacaagtattaagggtaggaaga
tatcaaaagagaggggcagcacatgagaatccttgtcaacagttagcagctgccctacaaaagataggaagagaa
agcatagttatatggggatttgtgccaaagatacaggtacccatacaaagagagatatggagtcagtggtgggca
gactactggcagtgtacatggatccctgagttagaatttgtcagcactcctaagttggagcaagagtggtatact
ctagccacagaaccagtgccaggagacacctactatgtggatggagcagctgaaaagctagaaaagagagggaaa
gcaggatatataacccagggaggaagatccagagttaagaaattggaaaacaccaccaatcagcaagcagaacta
gaggctataaaaatggccttggaggatagtaggagctcagttaatatagtaacagactcacagtatgccttaaga
ctgctcagtaagagacccacagaaacagattcagagctagtaaaggagatcgtagagctcataaggcaaaaggac
caggtatacctaggttgggtaccggcacacaaaggaataggaggaaatcaagaaatagatcaactagttagccag
ggaatcagaaagagacaggtaatgtttatagaaaagatagaaccagctgtagaagaacatggaagttccataac
aatgcagcctctttgcaggagatgtttgacataccccctagtggtagcaaagcagatagtcaatgaatgtgcacaa
tgtcaacagaaaggggaagccataacaggacaagtagatgcctcagtgggcatctggcagatagattgcacccac
atggaagaaaaagtcataatagtagcggtgcatgtggcttcagggtatatggtagcagaagtactacccaatgag
caagggaagacaacagccacttggctgttaaagctctgtgccatgtggccagtaaaacagatacatacagataat
gggccaaatttcatcagtaaagatgtggaggcagtatgttggtggttaggcatacagcatacaacaggatacct
tacaaccctcagagtcaaggagtagtggaagcaaagaacaaggtattaaaacaaattatcagcagaattagagaa
gatgcacaggaattgaaaactgcagtactcatggctctgcacattcataattttaaacaaaggggaggactaggg
ggaatgacagcagcagagaggtttatcaacatgattaatgcagatctagaaacacaatacttacagaaaataaat
tcaaaaattttaaaatttaaggtctactacagagaaggaagagatcctcagtggaaaggacctgctaaacttctg
tggaaaggggaaggagcagtagtcatcaaacagggtgagaacatcctggtagtacctaggagaaaagcaaaacta
gtaaaggacagagaatgggaagatagaagacagtataagatagttagaatagtatggcttatagatagaattgct
gtagagaaattttagatttaagaagaatgcatagggaaacaaaagatgattgggtatctatgtatggaacaggt
actggatgggagtggtacacatacaataaaattattattccagtaacttatgggacagtagtagtaaggatctat
ggacacctcacaccagcaaaagggttgattaaccagtggggatgtagtatggaatggatttataacagttatcaa
acagaaatagatcctcttgtggcagatcagatgatccactttaagtattttgattgttggacctctaggtgtatc
agaagagctatgttagggaaaaaatactacatgagtgcagaaatcaagtagctcacaaaggcctggttctctct
ttgcagtttctctgcttgagagttctcgccttgttcattcatctgccaatgtgtccttcttacatcagaatcaga
agtggatcaggattggaggcctgtgataacaaatgctggtgtaggagatgctgctttcactgtcaactttgcttt
ctgcagaaagggttagggatccactatatgcggtgccctgaaatattgataggtttctctttgctgttaggtagt
atagcaatacagtatgtaacagtattctatgggactcctaagtgggaaccagcagtagtgccattaatatgtgct
tcagctaataatagcttgtgggtgactacttcatgcttgccagatttacaaacttatgcagaagtgcccataaca
gggttagaagagaattttacagagggaataagcaataatcaaatagtgcagcaagcatggcaagctatgacctct
atggtagatgccattatgaagccttgtgtaaagatcaatccctattgtgtcaaaatgaagtgtccaccgaaaccg
acaactccctcctccaactcaacagtcaaaagtagctgtgattattggacgaccaccacggctaagactacaact
cagactacaagtagtaccagctccactgctagtacaactactccaatgcctctagattggaattgtactgacaca
gagaacatagcggagagcaataaagtatgtaaatataatgtgacagggctttgtagagattgtaaaacggaggtg
gaacaaaactttagggatacagaagttacttgcaatggtaatgacacatgttatatgacacattgcaatgattca
attattacacaagactgccataagggtattatgcaaaatgcttacttcaggctttgtgcacctgcggggtatatg
ttattaagatgtgatgaaaaattaaatgctactaagaaatgtaaaaatataactgctaccccttgtactaattat
atgacaagtacagttagtagctttttttggctttaatggaactagacataaggaagatgagttaataccaatcaat
aacaaggtaggacaaggggctgagggagaatatgtatggaaagtagcagctaagtgggggttagtaattcagtgc
ataaggaaagggaataggtcacaagtaagtactataagtagtacaggactgctgttttattatgggctagagcca

ggttctaagttaagactggcacagtgtaaatttgtaggacaatggggaagagcctttgccaccttagggaaacta
ttgaggcaggtggaacctttagctaacatgagtaatggctgtacatttgataatataacaaagacatgcagattt
accaatggtacagattttaaaaagatgattaagtttaaaccaatggagaagcatggagcagatgcagcaacagag
atgttgatgatgacttgtggagaagagatgttcttttgtaatttaacaagaattttcaaagtatggaatgacaca
acctccaataaatggtatccttgggccaattgccacataaaaagtgtgatagatgattgggcgtcagtgggcaag
aagatttacttgcccccaacatcagggtttaataacagaataagatgtgcaaacagagtgacagaagcatggttc
acactggaaagggtggaagactggaaggtaaatggatcaaacatctcagttgtagccttccagccacctaccaac
accttaaatcaatttgtgagcacaggagcacattacaaactagtaagaattaggccaattgggtttgctccaaca
gatgagcaccggtacgcacccaggaaggaaaagagggcttcttgggggtgtcaatggaaacaagtatgccatact
aatgtgccctggaattataatatcacgcccaattggactaaggacacctggatggaatgggataggcaagtaaaa
atgtatgatgataatataacagctcttctccaggaggcatatgtaacagagttagagaatcaaaacaagttcaaa
cagctacaagaatttaacttctggagttggttggacctgtctcaatggtttctatacataaaatatgcagtcctg
attataggcattataatagcagctagaattcttagttttatcatacaacagatctataggatgtgtcagggatat
agggtgttgtccccttctgcttatgttgaacagccaacatataagcagctaatagatctctcacattttataaaa
gaaaagggtggactggaagggctctggtactccagaaccagagaagaaatcttggatttgtatgcagaaaatgag
tggggcttcataactggctggcaagattacaccaaagggcctggagtgagatatccaaaggcctttggctggcta
tggaagctagcaccagtcaccattgatgaagacagggatcctaatcatccttgtcaagccctactgcattccagc
cagcaaggggttaatgaagacccttggggagagagacttatctggacatttgatcccactctggcctatgatttt
agggccatacaaaagcaccctgaagagttcaagcatgttactagtctgcagtgggaggtgtag
>#30 AF075269 6960 nt
atgggatcgggtaactctgtcctcagtaggcaaattgaaaaagatttttgtagtgtcaggctaagacctgggagt
aaaaagacttatcaaaaaagacatgtagaatgggcaacgaaagaattagacagatttggattgggatcacaactc
ttggaaacagcagaagggtgtaagaaaatcctatctgtctgctggcctttatatgcaactggttctaagaaccta
aaggccttagtagggacagtatgtgtcatctgctgctgtcacctaggagtaagaatatcagacactcaggaagcc
atcagtaaagtaaaaatagaaccagcaccagaagcagcagggaagaaacaacagacaggggaaattatcccctg
ataagagaaaatcagagatgggtacatacacctttatcgcccagaacaatacaaacatgggtgaaaatagtggaa
gacagaggttggaaaccagaaacagtagcaatgttttcagccttaacagaaaaagctttgcctgatgatttaaat
gttatgttaaatgcaattggagatcatcagggcgccatgcaaataataaaagatcacatagtggaggaaggtgca
gagtgggatagacagcacccgcaacaacagccagctcaaccaggagggggtttaagaactcctaatgcaacagat
gtggcaggggtcacctcaacagtagaagaacaactagcctggactacagcagacactcctgtagatgtaggaaaa
atttataaagaatgggtcattcaggctatggaaaaagtagtcagaatccatcaaccagtaagtgtcatggacatc
aaacaaggacccaaagagccttttaaagaatatgcagataggttctttaaagcactgagagcagaaggaggctct
catgaagtaaaggagtggatgaaggagaaaatgcttgtgcagaatgcaaaccctgactgcaggttagttataaaa
gcactaggagaaggggcatctctagaagagatgatgaaagcttgtcagggagtaggaggacctgctcataaagga
aaaatattggcagaagcaatggcttcagccatacagcaacaagtaagacaaaacatggtgcaggtcactccgctt
agaaatgcgcaaggcaggtttgtgagaacaggaggaggaggtcccaggaaacccctcacttgcttcaattgtgga
aagccaggtcacacagcaagaatgtgcagacagcccagacaggaaggttgttggaactgtggcagtaaagagcat
agtatattggtcaatggcaaccagtgtcagcactgttggacactggggctgatgacacaatttttcagaaaat
tcagtaagaatagaaggaccctatactccaagagtagtagggggcataggggggcaaattagggtaaaagaatat
agagatgtctttatagaaatagcaggaaaaactacagtagggacagtcttattaggacctaccccagtagatatt
gtagggaggaatatttaactgcaatgggggccaaattgattttagctcagttaagtgataaaattcctattaca
aaggtatctttaaaacctgggtgtgatggaccaagagtaaagcagtggcctctatcaaaagagaaaatagaaggc
ctacaagctatttgtgataggttagaaaaagaaggaaaaatatctccagtggatccagggaacccatacaatact
ccaatatttgctatcaagaagaaagacaaaaatgaatggagaaagttaattgatttcagaaagctcaatgaatta
actcaagactttcatgagctacaattaggtattccacacccagcaggtatcaagaaatgcaaacagattacagta
gtagacataggagatgcctatttcagtatccctttagatccaaattacagaaagtatacagcatttactatacct
tctctcaataatcaagagccaggaaaaagatatcagtacaatgtgctgccacaagggtggaaggggagcccttgt
atatttcaggaacagtagcaggacttctctcagagtttaggaaattaaatccagacatgatcatttaccaatat
atggatgatttattcataggatcagatagagagagaaaaggacatgatcaggcagtaaaagaactcagagaactt
cttatgacatggaacttagagacaccagaaaagaagttccaagcagagccaccctatcattggatgggttatgta
ctgcatcctgataggtgggaaatagaaaagattaaattaccagagatggatctaacaaaaactacagtaaatcaa
atacagaaactggtgggagtacttaattggcagctcaattgtatgatggtattaggacaaaagaactctgcaaa
ttaataaggggagtaaaacccttggaagaaatcataaactggacagaggaagccttggaagaatatggcagaac
aaagaggtacttaaagaaaagatgcagggagcctattatgacccagaaaaggaacttattgtcagggtacagcaa
aacaaaaaggggataattactttccagtggagacaaggaaataacatcttaagagctgggaggtatcaaagacag

aaggcagcacacacaaatcccctacagaaattagtagaagctattcagaagataggaaaagaaagcatagtcatc
tggggctttgtgccaaaaattcaagtcccagtgactagagaagtctgggagcactggtggagcgaccactggcag
gttacatggattccagacttagaattcatttctaccccgcaattagaacaagagtggtacatttgggaggcagaa
cccataataggggtagacacctactatgtagatggagcagcagaaaaggtaggaaaaacaggaaaagcaggatat
ataacacaatcagggaaagagaaagtaaaggagttaactgacaccacaaatcagcaagcagaactagaggcagtt
ctaatggcattacaagatagcaatagtaaagtaaatatagtaacagattcacaatatgtaatgaaaatattgtca
caaagaccaacagaaacagaacaccccatagtaaaagacatcatagaacaatgcaagcagaaagatcaagtttat
ctaggatgggtgcctgctcataaaggaataggaggtaatcaagaggtagaccacctagtaagtaaaggtataaga
cagaaacaggtcatgtttctagaaaagatagaacctgcagtagaagaacatagcaaattccataacaatgcaaag
gatctagaagaaaaatttaacctacccccatggtagccaaacaaattgtcaatgactgtgcaaactgtcaaaag
aaaggagaagccataacaggacaagtggatgtctcagtgggtatttggcagctagactgtactcacttagaagga
caggtcattataaatgcagtccatgtggcctcaggcttcatggttgcagaagttataccagatgagacaggaaaa
acaacgtcacatttcctgttaaaattatgcagtagatggcctgtaaaacaaatacatacagacaatggtccaaat
tttgtaagtaaggaggtacaggcagtaacctggtggatagggatagaacatacaactgggataccttataaccct
cagagtcaaggggttgtagaagcaaaaaacaaagttttaaaaagtattatagaaagagtaagagaagatgcacag
caactaaaaacagcagtactaatggcagtccacattcataattttaaacaaaggggaggactagggggcttaca
ccagcagagaggtttattaatatgattaatgcagaactagaaacacaatatctacaaaaattaaattcaaaaatt
ttaaaatttaaggtttattacagacaaggaagagatcctcagtggaaaggaccagcgcaacttctgtggaaagga
gaaggtgctgtagtggtaaaagaaggggagaacatcttctcagtccccagaagaaaagcaaaactagtaaaagat
tccttaatagaaaagtttttagacatagtcaggttaaacacagaggcaaaagaaaattgggtaggcttacatgga
acaggtgtaggttgggaattctatacctatcataaattagtgatacctctagagtcaggaactttagtagtcaga
atatatgcacatttggcagcaggaaggggatggatctcacaatgggcagtttccatagaatgggtctatagagac
tatcaaacagaaatagaccccatactagcagaccaaatgatccactataaatattttaactgcttcatacaaaga
gacattagaagggcacttctgggagaaagaataactatgtgttgccagcctttaggacataaaggttgtgttctc
tctttgcagtttatctgcttgagacaactacaagctttgtttcttcacctcccaacttgtcccagcgccctgcaa
gcgtgtcagaataccatggcatgtccaggattaggaattctgcttttgcttcttggcataatatggggaaaacaa
tatgtaacagtattttatggggtgccaaattgggatgataatgtttcagtgcccttgatttgtgcttcagccaat
actagcctctgggtcacgacatcttgcttgccagatttgcaatcttatgcagaggtacctatctataatatttca
gaaaactttacaataccagtaaaagacaatcaggttatacagcaagcctggtctgctatgaatgctatggtagac
agtattatgaaaccatgtgtaaaaatcaatccatattgtgtgagaatgcagtgtggggaagttacaaaaacacca
acaacaacaccaaaaactactacacagatgccttgttttatcaacgaacaggtaacagttaagaacccggggaat
gaaacaagattagaggaagatcttaattgcacaagagggcttaatgagaccacagagaggaatgcagaatgccag
tataatgtaacagggttatgcagagactgcaggacggaaataaaacaaagctttagatatgatgatgtaacgtgc
tcaggagagagggagaacagaacctgttacatgactcattgtaatgattcaataataacacaagattgtaataag
ggggtaatgcaaaatgcttattttaggctttgtgctccagcaggctatatgctattgagatgcaatgaacaatta
aactttagtaaaaaatgtgaaaatataacagctacaccatgtactggatatatgcttagtagtgtaagtagtttc
tttggctttaatggaactaatcataccagggatgagcttattccactaactccaaataagatggaagatctaaat
ggtgcaaagtttgtgtataaagtagcaggaaaatggggtttaattattagatgcataagaaaaggaaatagatca
gaagtatccacaattagctctacaggatatttattttattatggcctagaacatggcagcagattaagattagca
cagtgtaaatttgaaggccaatggggaagaatgtttaataatctaggcaaaatgctaaaggagttgaatgcagaa
gccatgaattatacagaagggacagggacatgtgattctaagaaaacaacttgtggacggaaattaaaaggctta
ccaatagctaatatgactaggcatggagcagacttagcaacagagatgttaatgcatacttgtggagaggaaatg
ttcttttgtaatgtaactaggatattccaagaatggaacaataaaaattcagataaatggtatccttgggccaac
tgccacataaaaagcatcattgatgattggctacaataggaaagaagatttatctaccacctacttcaggtttc
aacaatagaatcagatgtactcatagagtaacagaaatgttctttgagatggaaaaatgggaaccacatgaggac
ttaggaggcaacctaagtataaagttcttgcctccatcatgggaaactaatcaatttgtggcagaagggtctaaa
tataaattgataaaattgaatccaattgggtttgctcctacagatgagcacaggtacgcacccaggggaaggcaa
accagagcctcttgggggtgtcaatggaaacaggtttgtcatactaatgtggagtggacatataatatcacaccc
aattggaccaaggatacctggagagaatgggaatcaaaggtagcaatttatgacaagaacattacttctctgtta
caagaagcatacaccacagagctagagaatcagaataaatttaagaagttacaagaatttaacttttggagttgg
ttggatatctcacactggtttacttatgttaagtatgcagtgttaattatacttgtaatcatagggttaagagta
ttgagctttataatacaaaatgtagtaaaaatgtgtaggggggtatagggtgctctccccctctgtttatattgaa
caggactacaagtgggagaaagaacctacctataagactcttatagacctctcacattttataaaagaaaagggt
ggactggaagggctatattggtcccagagaagacaggacatcttaattctctactgtgagaatgagtggggtctc
attggagacttcatgaactacacagatggaccaggaacaagataccctctgacatttggatggttgtggcagtta

gaaccagtggcttgtgatgagtataaagatccctcagatgagacacaatgccttttgcattcaagccagctgggg
gtcctggaagaccccctggggagagaggcttatctggcacttcaatccaatgttggcagtggactttatagccctc
aaaaagcagcctgcgaagatacaaaatactgctttcgcttttgactgcaagaggaagtag
>#31 AF188116 7026 nt
atgggatcggggaactccgtcctcagtaggcaaattgagaaagattttgtaatgtcaggctaagacctgggagt
aaaaagacttatcagaaaagacatgtagactgggcaacgaaagaactagataggtttggctaggagcaaacctt
ctagaaacagcagagggctgtaagaagattttgggagtccgttggcctttatatcctacaggctctaaaaatctg
aaagctctagtagggacagtgtgtgtcatctgttgctgtcacctaggaataagagtcaatgatacacaggaagca
attaataaagtgaaaattgagcagaacaaacctgtgccagaaagtaaaaattttcctttgattagagaaaatcaa
agatgggtccatacccctctatcacccagaacaattcaaacatgggtgaaaatagtagaagataggggatggaag
ccagaaacagtggctatgttttcagctctgacagaaaaagccttgccagaagacttaaatgtcatgttaaatgct
ataggagaccatcaaggggcaatgcagataataaaagatcatatagcggaagaaggagctgaatgggatagacag
catccccagcagcaaccagcacaaccaggaggagggttaagaactccaaatgcaacagatgtggcagggatcaca
tcaacagtagaagaacagcaagcctggacaacagcagacacacccatagatgtagggaaaatctataaagaatgg
gtaatccaggctatggagaaagtggtaagaatacatcagccagtcagtgtaatggacataaaacagggtccaaaa
gaaccattcaaggactatgctgataggttctttaaggccttaagagcagaaggaggttcacatgaagtgaaagag
tggatgaaagagaaaatgctagtgcagaatgcaaaccctgactgcaaaatggtaataaaggccttaggagaaggt
gccactttagaagaaatgatgaaagcatgtcagggagtaggaggacctgcccataaaagaaaaatcttagcagag
gcaatggcaacagccatgcaggggcagatgaaacatctcatggtgcagcaaacaccacctagaaatgcacaaggg
agatttgtaagaacgggggaggtggcccaagaagacctctcacttgtttcaattgtgggaagccaggacattca
gcaaaaatgtgcaggcagcctagacaggaaggctgttggaactgtggtgctaaagtactggtcaatgggcaagaa
gtaactgccctattggacactggagcagatgatacaatcttttctgaaaattcagttagaatagaaggaccttat
actccaagagtagtaggggggtataggggggacaaattaaagtaaaagaattcagggatgtttttatacaaatagca
gggaagaccacagtagggacagttttgctaggaccaaccccagtagatatagtaggaagaaacattttaaaccct
ctaggggctagattagtgttagcacaattaagtgagaaaatacctataacaaaggtgcatttgaagccagggtgt
gatgggccaagagtaaaacagtggccactatccaaagaaaaaatagaaggattacaagcaatttgtgatagatta
gaaaaggaagggaaaatatccccagttgatccaggaaatccttacaacaccccaatctttgctataaagaaaaag
gacaaaaatgaatggagaaagttgatagatttcagaaaattaaatgagttaacacaagactttcatgagctacag
ttaggaataccacacccagcaggaataaagaaatgcaaacaaattacagtagtagatataggagatgcctatttc
agcatacctttagatcctgattacagacaatatactgcgttcactataccgtcactaaacaatcaggaaccagga
aaaagatatcagtataatgtgttgccacaaggatggaaggggagtccttgtatttttcaagaaacagttagtgga
ctccttgcagagttcaggaaattaaatccagacatactcctctaccagtacatggatgatttgttcataggttca
gacagggagaaagaaggacatagtcaggcagtaaaagaattaagagaacttttaataacttggaacttagaaact
ccagagaagaaatttcaagacaaaccaccctaccattggatggggtatgtcctgcacccagatagatgggaaata
gagagagtaaaactgccagagatagacacaaggaaaactacagtaaatgaaattcagaaactggtaggagtttta
aattgggcagcacaactgtatgatggaatcagaacaaaagagctctgtaagttgattagaggagtaaagcccttg
gaacaactagtgacttggacagaggaagcactagaagaatatgagaccaataagacaattctaaaagaaaaagta
caggggggcttactatgatccaagtaaagaacttgtagtaagggtacaacagaacaagaaaggggttataactttc
caatggaggcaaggaaatgccattctaagagcaggaagatatcaaagacaaaagagtgctcacatgaatcccctt
cagcaattagcagaagctgtacagaaaataggaaaagaaagcatagtcatttggggttttgtaccaaagatacaa
gtacctgtcactagagaaacatgggaaaattggtggataaacttttggcaagtaacttggataccagacttagaa
ttcatctccacacctcaattagaacaggaatggtatagcttggaagcagaacccatagaaggtacagatacctac
tatgtggatggagctgcagaaagagtaggtaaaacaggaaaagcagggtatataactcaatcaggaaaagagaaa
ataaaagaattacaagatactactaatcagcaggcagaattagaggcagtattgttagctttacaagatagcaaa
tcaacagttaacatagtaactgactcccaatatgtaatgaaggtcttaagtcagagacctacagaaactgagcat
cctatagtgaagaacatcataaaggagctgcagaagaaagatgcagtttacctaggatgggtaccagctcataaa
ggtataggggggaaaccaggagatagatcaattagttattaaaggaattaggacaaaacaggtcatgtttctggag
aagatagaaccagctgtagaagagcatggaaagtttcacaataatgcaaaagacttagaagaaaagtatggata
ccccccatggtagctaagcaaatagtaaatgagtgtgctaagtgtcagcagaagggagaagccataacaggtcaa
gtagatgtatcagtgggtatttggcaactggattgtactcacctagaagggcaaatcatcatgaatgcagtccat
atggcatcaggcttcatggttgcagaagtgattccagatgaaacagggaaaactacagcaaatttttgttaaag
ttatgtagcagatggcctgtaaaacaaattcacacagataatggtccaaattttgtgagtaaagatgtacaggca
gtgacctggtggttaagtatagagcacacaacagggataccatataaccctcaaagtcaaggagtagtagaggca
aaaaataaagttttaaagcaaatcatagaaagagtaagagaagatgcacagcagctaaagacagcagtgttaatg
gcagtccacattcataattttaaacaaaggggaggactggggggttatcaccagcagagagatacatcaatatg

atcaatgcagatttagaaactcaatacatacaaaaattaaattcaaaaattttaaaatttaaggtttactacaga
caaggaagagatccacagtggagaggacctgctcaacttctgtggaaaggagaaggtgctgtagtggtaaaggag
ggagagaacattttctctatccccaggaggaaagcaaaattagtgaaagatagcttagtagagaaatttctagat
atcaaaaggaatcattcagaaagtaaaaatgattgggttagtttacatggtacaggagtaggatgggagttttat
acttacaataaattactaattccattagatacaggaactttagtagtaagaatttatgctcatctggcagcaggg
agaggatggatttcacagtgggcagtatcaatagaatggatctatggaaactatcagacagaagtggatcccatt
ctagctgatcaaatgcttcatgtgaagtattttgactgttgggtgcatagggacatagttagggccataagggga
gaaaagattctgcacagctgtaaaatgcctactggacataaaggttgtgttctctcattgcagtacctctgcttg
agacaactacaaagagctctctatttacatctgcctcaatgtcccagtccactccaatcctgccagaataagtgc
cactgcaaagtttgttgctttcactgtatactttgcttccagcagaaggcgttagggatacggtatatggcatgt
ccaggtaacatattgctattgctgctctgcttgggactaacagcaggagaaaaggagaaacaatatgtaacagta
ttctatggagtaccaaaatgggaagatgctgtagtacctttaatctgtgcttcagctaataatagcttgtgggtc
actacatcttgtctcccagatctgcaatcttatgctcagatccctattcataatattactctaaattttactcaa
gaaattaaggacaatcaaataatacagcaggcatggggagctatgacgagcatggtggatgctattatgaagcca
tgtacaaaaataaatccttattgtgttcgcatgaaatgtacaggggaaggactaggtaagaagagaacaacaaca
acaacaacaccaacaacaacacccatgccttgctttataacagaatctactactactactacaaagagttcaggg
actgaacaaccatctttaatagaagatacaaattgtacacgatggttaaatgagactacagaaattaatagggaa
tgttcatataatgtgacagggctttgtagagattgcaaggaagagataaagcaaaattttaggtatgatgagaca
acatgtaatgagaatggtacttgctacatgacccattgcaatgatagtattattctgcaagactgcaataaggga
gttatgtcaaatgcttatttaggctttgtgcaccagctggctacatgcttttaaaatgcaatgaaaagctaaac
ttttcagctaattgtactaatataacagctacaccatgcacagattacatgataagttcagttagtagcttcttt
ggctttaatggaaccaatcatacagaagacgagttaattccactaactccaaaaaagatgggagatcttaatgga
gctaaatttgtttacaaggtagcaggaaaatggggactaatcattagatgtataagaaaaggaaataggtcagaa
gtctccacaatcagttcaacaggacttctgttttactatgggttagaacatgggtctagactaagattggcacaa
tgcaaatttgagggtcaatggggaagaatgtttaacaatttaggaaggatgttaaagaagcttaatgagactgct
atgaattataaagaaggaaattgttccacaccaggaaaaccatgtggtagacaattaaagggtctacccatagca
aatatgacgagaagaggagtagatctcgcaacagagatgctcatgcacacttgtggtgaggaaatgttcttttgt
aatgtcacaagaatcttccaggaatggaacaataaaaactcagataaatggtatccctgggccaattgccacata
aaatcaattatagatgattgggcaactgttgggaagaaaatttatcttcctccaacatcaggatttaacaataga
ataagatgtactcacagagtgacagaaatgtggtttgaaatggaaaaatgggaaccacatgaagacttaggagga
aatctgtctattaagtttttgcctccctcatgggagacaaatcaatttgtggcaacaggagctcattacaaattg
atcagactgagaccaattggctttgctccaacatcagagcatcggtacgcacccaggggaaggcaaacaagagcc
tcttgggggtgtcagtggaaacaagtgtgtcatactaatgtaccatggaattataatgttactcctaattggaca
agggatacctggatagaatgggaaagacaagttggatctttagaagcaaacatcaccacactgttgcaagaggct
tacacaacagagttagagaataggaataactttaaaaagttacaagattttaatttttggagttggatggacctc
actacttggttccaatatattaagtatgcagtgcttataatcataggtataataatactaagaattctaagcttt
atcatacaaagtgtagtgaagatgtgtaggggctatagggttctcgccccctctgcttatgttgaacaggacttc
aagtgggagaacggatctacctataaaaccttgatagacctctcacattttataaaagaaaagggtggactggaa
gggctatattggtcccagagaaggaatgacatcttaacactctactgtgagaatgaatggggtctcattggagac
ttcatgaactacacagatggaccaggaactcgttacccactcgcctttggatggctgtggcagttggagccagta
gcatgtgatgagtttaaagatccctcagatgagagacaatgtttgctacattcaagccagttggggggtcctagaa
gacccctggggagaaaggctcatctggcactttaatcccatgttagcagtggactatgtagccctgagaaagcaa
ccagcaagtgttcaagcaactgctatgagatttaactgcgagaggaagtag
>#32 AF188115 7053 nt
atgggatcggggaactctgtcctaagtaggcaaattgagggagaattctgcagtatcaggctaagacctggcagt
aaaaagacgtatcagaaaagacatattgtgtgggcaacaaaagaattagataggtttggtttaggagcacacttg
ctggagacagcagatggctgcaagagaattcttggagtctgttggccactatactccacaggctcaaagaattta
aaagcacttgtaggaactgtatgtgtgctgtgttgctgccatttagggataaaaatagcaaacaccgcagaagct
atgcaaaagataaaaatagaaccaactcaggaagacaggcagcagaaaagtggcaattatccactcatcagagaa
aatcagagatgggtacacacgccattgtcacctagaacaatacaaacttgggtaaaaatagtagaagatagagga
tggaaaccagaaacagtggcaatgttttctgccctcacagaaaaagctttgccagatgacttaaatgtcatgtta
aatgccataggagaccatcaagtggcaatgcagataattaaagatcatattgtggaagaaggggcagaatgggac
aggacacatccgcaacaacaacctgcacaaccaggaggagggcttagaacccctaatgcaacagatgttgcagga
gtcacctcaacagtagaggagcagctagcctggaccactgcagacaccccagtggatgtaggaaaaatttataaa
gaatgggtaattcaagctatggaaaaagtagttagaattcatcaaccagttagtgtaatggacatcaaacagggg

ccaaaagaaccattcaaagaatatgcagataggttctttaaagcattaagagcagaaggaggctcacatgaggta
aaagaatggatgaaagagaagatgctggtacaaaatgcaaacccagattgtaggatggtaataaaagccttagga
gaaggggctagtttagaagacatgatgaaggcctgtcagggagtaggaggaccagcccataaaggaaagattttg
gcagaggcaatggcaacagccatgcaaagtcaaatgagacagaatatggtacaagtgacaccaccaaggaatgag
cagggaaggtttgtaagaacaggaggaggaggccctagaaagcctttaacttgctttaattgtggaaagccaggt
catacagcaagaatgtgtagacagcctagacaggagggatgctggaactgtggtagcaaagaacataggtttgct
caatgtccaaagcctaacattctggtcaataggcagccagtgtctgctcttcttgacactagggcagatgatacc
atcttttcagaaaattcagttaaaatagagggaccttatacacctagagtagtaggggggaataggggggacaaatt
agagtaaaagaatacaaggatgtctttatagaaatagcagggaagacaacagtagggacagtactattaggacct
acaccagtagacattgtaggaagaaatattttaactagtgtgggagcaaaattagtactagcacaattaagtgac
aaaatacctatcacaaaggtcagattgaagccaggctgtgatggtcccagagtaaaacagtggcctttgtcaaaa
gaaaagatagagggcctacaagcaatttgtgatagattagaaaaagaagggaaaatatcaaaagcagatgcagga
aacccatataacacaccaatctttgccataaagaagaaggacaaaaatgagtggagaaaactgattgattttaga
caactaaatgctttaacacaagaccttcatgaagtacaattaggcattccacatcctgcaggaataaagaaatgc
aagcaaataacagtggtagatataggagatgcctattttagtattcccctggatccagactatagacagtacaca
gcattcacagtaccatctattaataaccaggaaccagggagaagatatcagtataatgtactaccccaaggatgg
aaggggagtccatgcattttcaagctacagtagcaggactcctatcagaatttagaaattaaaccctgacata
atcctgtatcaatacatggatgatttactcatagggtcagatagggacaagaaagggcatgatcaggcagtaaaa
gaattaagagagctcttaatgacatggaatttagaaacaccagaaaagaaatttcaagcagagcctccctatcat
tggatggggtatgtacttcatccagataggtgggaaatagagaaagttaaactgccagacatagaccttcagaag
acaacagtaaacaacatacagaagctagtaggagtcttgaattgggcagcacaactttatgagggaattagaact
aaagaactttgtaaattaattaggggggtaaaagctctagaagaagttgtaacctggacagatgcagcattagaa
gaatatgaacaaaaccaacagatactaaaagaaaaactgcagggagcttattatgaccctactaaagaattggta
gtaagagtacagcagaataaaaaggggtcattaccttccagtggaggcaaggaaattctattctgagagcagga
aggtatcaaaggcaaaaagcagctcacacaaatcccctacaaaagttggcagaggcagtacaaaaaataggaaaa
gagagtatagtcatttggggtcatgtgcctaagatccaggtaccagtaacaagagaagtgtgggatcaatggtgg
agtgatcattggcaggtcacctggatacctgaattggagtttatctcaaccccacagttagaacaggaatggtat
agcttagaagcagaacccatagaaggggtagatacctattatgtagatggagctgcagaaaaagtagggaaaaca
ggaaaagcaggttatgtcacacaatcaggaaaggagaaagttaaagaattaacagataccaccaatcaacaagca
gaattagaggctgtcttattggcattaaaagacagtaaaagcagggtcaacatagtaacagactcacaatatgtg
atgaaagtcttatctcaacggcctacagaaacagaacaccctgtagtgaagcagattatagaggaatgtaagaag
aaagaccaagtatatctaggatgggtaccagctcacaaaggtataggggggaaatcaagaagtagaccacctagtc
agtaaaggaattagacaaatacaagtactgtttctagaaaaaatagagccagcacaggaagagcatagcaaattt
cataataatgctaaagatctagaagaaaaatttcacctacccccctatggtagctaagcaaatagtgaacagttgt
aataaatgtcagacaaagggagaagccataacaggacaagtagatacttcaccaggtatttggcaaatagactgc
actcacatggagggacaggtaatcattaatgcaatccatgtggcctgtggatttatggtagcagaagtaattcca
gatgaaacagggaaaacaacagcaaacttcctgttaaaactgtgcagcagatggccagtacagcaagtccataca
gacaatggaccaaactttgtcagcaaggaggtacaagcagtaacctggtggctaggtattgaacactcaacaggg
attccttacaatccccagagtcagggagtagttgaagccaaaaataaagtgctaaagcaaatcatagaaagggtt
agagaagatgctcaacaattaaagacagctgtcctaatggcagtacacattcataattttaaacaaaggggagga
ctaggggggctttcaccagcagaaaggtttattaatatgattaatgcagacttagaaacacaatatttacaaaaa
ctaaattcaaaaattttaaaatttaaggtttactacagacaaggacgagatcctcagtggaagggacctgcacaa
attctgtggaaaggagaaggtgctgtagtggtaaaagaaggagataccatcttctcagtccccagaagaaaagct
aagctagtaaaagataacctgatagagaaattcctagatattaggagagcacataaagaaactcaggttgattgg
gtaagcttacatggaacaggggtaggctgggaattttatacttataataaaatagtaatacctttagagatagga
accttagtagtaagatgctatgcacacttggcagcaggaagaggatggatctctcagtgggcagtatcaatagaa
tggatttatgggaactatcaaacagaaatagatcccattctagcagatcaaatgatacactgtaaatattttaac
tgcttttctacaagggatattaggaaggctttatggggagaaaagataatggcaatctgcgctatgcccactgga
cataaaggttgtgttctctcattgcagtttctctgcttgagacaactacaagcactctttattcatttgcctacc
tgtcccagcccactgagtgtctgtgacaacaagtgctgctgcaaaaggtgttgctttcattgtattctttgcttt
cagcagaagggattagggatacgatatatggactgtcttaagctcctacttttcttgctctttttaggagcacaa
ggggaggcaaagaagcagtatgtaacagtattttatggggtgcctaagtgggaagatgctgtagtgccattaatt
tgtgcttcagctaataatagcttatgggtcacaacatcatgcctaccagatttacagtcttatgcacagattcct
attcacaacataagtttaaatttcactcaagagataaaagataatcaaatcatacagcaagcatggagtgctatg
accagtatggtggatactatcatgaagccttgtgtaaagataaatccatactgtgtgagaatgaaatgtacaggg

ggaggaataggaccaaaaacaacaccatcaccaataggaaaaacaacaacacccatgccttgctttatagcagaa
acaaccacaaccaccaccaaagggaatacaactatggaaagtttaatagaggacacaaattgtactagatggtta
aatgagactactgagattaatagagaatgtaaatataatgtaacaggactatgtagggactgtaaagaagaaata
aagcagagttttaggtatgatgagacaacctgtgataagaatggaacctgttatatgacccactgtaatgatagt
attattcagcaagattgtaataagggagtaatatctaatgcttattttaggctttgtgcaccagcaggctatatg
cttttaaaatgcaatgaaaaattgaatttctcagctaattgtaccaatataacagcaactccttgcacagactat
atgattagtacagtgagtagtttctttggctttaatggaaccaatcatacagaggataagttaattccactaact
ccaaaaaagatgggaaatctcctggacgcgaagtttgtatataaagtatcaggaaaatggggactcattatcaga
tgtattaggaaaggaaacaggtcagaagtctctaccatcagttcaacaggacttctgttttactatggcttggaa
catggtagcaggctgagattggctcagtgtaaatttgaaggacaatggggaaggatgtttaataatttagggaaa
atgcttcaaaagcttaatgcaacagcaatgaattatacaaatggacaatgtcctgagggaaaacagccttgtggt
agacatctaaagggattacctatagcaaatatgacaagacgtggggaaggtcttgcaacagagatgttgatgcat
acatgtggagaggaaatgtttttctgcaatataacaagaatactccaagaatggaataataaaaactcagataaa
tggtatccctgggccaattgccacataaaatcagtcatagatgattgggcaactgtagggaagaaaatctatatc
cctccaacatcaggctttaataacagaataagatgtactcatagagtaacagaaatgtggtttgaactggaaaag
tgggagccagaggaaaacctgggggaaatttgtctgtcagattcttgcctccctcatgggagacaaatcaattt
gtggcaacgggagctcattataaattaataaggattagaccaattggctttgctccaacagctgagcaccagtac
gcacccagtagtaagcagaagagagcttcctggggtgtcaatggaaacaaatatgtcatactaatgtggaatgg
aattataatataactcctaattggacaagggatacctggattgagtgggacagacgagttggagtattagaagca
aatatctccaccctgttgcaagaagcctataccacagagttagaaaatagaaatgccttcaagaagctacaggag
tttaacttttggagttggttggatattttatcatggttccaatatattaagtatgcagtactaataataataggt
attatagtgttaagaatagtaagctttattgtgcagaatatagtaaaaatgtgtaggggatatagggttcttgcc
ccctctgcttatgttgaacaggactacaagtgggagaaaggacctacttataagactctgatagacctctcacat
tttataaaagaaaagggtggactggaagggctatattggtcccagagaagacaggacatcttgtgtctttactgt
gagaatgaatggggtctcattggagacttcatgacctacacagatggaccagggacacgttacccattaacattt
ggatggttgtggcagctggaaccagtggcctgtgatgagtacacagatccctcagatgccagccaatgcctgctg
cactcaagccagctgggggtccaggaggacccctggggagagaggcttatctggcacttcagtcctatgctagca
gtggactttgtagccctcagaaaacaacctaagagcatacaagcatcatctcttagcttgaactgtaagaggaag
tag
>#33 AY523867 7185 nt
atgggagcaggggggatcggcgatcctaacaggtagaaagcttgataaatatgagaaaattcggcttcggccgaag
gggaagaagcgctacctagttcgacatttagtttgggcaggaaaggagctggacagattcggcctctcagaccag
cttcttgaaagtaaagaaggttgtgaacaaatcataaaaaccatcttaccattagaaaaacatggctcagaaaat
ttaaaatcattatttggcataacagcagtagtctgggccgtccacgcagaaaaagacgtggaagatacggaacaa
gcaaaacagaaagtgaaagaggcttgcaattgggaagatgaggaaacagtgacatctagtggccaaaaagaaaat
agcaacgacacagcgacatctagtggcagagaaggaaaaatgcagctgccagcagcaatgccatctagtggcggc
tcaggaaattaccctctaattagaaacccacaaaatcaatggattcatgtaggggtcaacaccaggactttaaaa
acctgggtagaggcagtgaacagcaaaaaatttgatgcctcactggtcccactattccaattttaacagaaggt
tttataccttatgacctcaatgacatgcttaatgccattggagatcatcaaggagcaatgcaagtgatcaaagat
gtgattaatgaggaagggtctgaatgggacctacaacacccacaacctcaacagcagcagcctgtagcagggcta
agagatcctagtgcctcagacatagcaggaacaaccagtaccatccaagaacaaatagagtggataactagacag
aacaacccaattcaggttggacagatctacaggcagtggataattctaggattacaaaagtgtgtgcaagtctac
aatcctgtcaatattttggacatcaggcaaggacctaaagaaacatttaaggattatgttgatagattctatcat
tgtcttagagcagaacaagcggacccggctgtaaaaaattggctcacacagtcattactaattcagaatgcaaac
cctgagtgcagaagcattctgaaagcaatggtcaaaccaaccttggaagaaatgcttcaagcttgccaaggggta
ggaggaccacaatacaaagcaaaactattagcagaagcaatggtaatgacacaacacagcttgggcatgatacaa
gggccaagacaaggaagcaatcccaggagagggccaacaagatgctttaattgtgggcagttaggacatcttcaa
aaagactgccctaggccaaaaaagcttaagtgctttaactgtggaggaacgggacacatagcaaggcagtgtaga
caaccaagaaaaggacaaggcaaccctcctccacaggccgaaataaatgtagaagggaccaaggtaacaatgttg
ctggacacaggggcagatgacaccataataagagaacaagacatccagttacatcaaccttggaccccaaaaata
gtaggggggattaggaggaaacataagggtaagacaatataaccagatcagatttcaaatagaaagaccagatggg
agaataaaggaagtagagggtagtcttctagtaggacctacaccagtaaatattttaggaagaaacatattgagc
aaattgggagcaaaattagtaatgatagcacaaaaattagaaccaatgaaagtccaactaaagccaggaaggag
ctacctaaattaaaacagtggcctctgacaagagaaaaacttgaggcactgcgagcaatagtttcagatatgctt
gaaaggggcaattggaaaaggcctcccctacaaatccttataataccccagtttttgtaatacaaaagaaggac

aaattaaaatggaggatgcctatggattttaggaaactcaatgaagcaacacaggacttttgggaagtacagtta
ggaatacctcatccaggagggctcagtacaaaaagaatgacagtattagacttgaaagacgcctattacacagta
cctctagatgaagagttcagacagtatacagcatttacagtgcccagtataaacaattcttctccaggagagaga
taccagttcaaggtgttgccacaaggatggaagggatcgcccactatctttcaggcaacagtaaatcagatactc
caacctataagggaaaagaattcagatataataatagtacagtacatggatgaccttttggtagggtctaacaga
tcagaaaaggaacatggtcaaattgtaagtgaaattgtaaaaagtctattagcagtaggattctctattccacca
gaaaaatggcaggacaaattccccttgcaatggttagggtacacgctacaccctgataagtggagtctacaaaag
gttcaactaccagaaattacagaatcaccaacagttaatgagttacagaaaataataggagtcctaaattgggca
agtcaaatatatcctggtatcaaatgtaaagcattgagtaagtgcataagaggggcaaaagcacttaccgacatt
gtagaactgacagaagaggcagaagcagaaatggctgaaaatagagagatactcaaagtagaacaaacagggtct
tattatcagccagaaaaaccattagaagcacacattagcaagttaggacaacaacagtggggtacataattaaa
caagggccacaagaaaaacctctgatcacaggaaaaacaggaaaagcatatgcgacccatcacaatgattaccag
gcattggctcagctgatgaataaaataggaatacaagccctatggatttggggaaaaatccctgaattccacctc
ccagtaaaaagggaagaatgggaaaaatggtggacagatcactggcaagcaacatggttgccagaagtaaaatgt
gtgcacaccccccatattagttagatggtattacaatttggtttcagagccagtagaagcagaaacattttatgtc
gatggggccgctaataggaatagcaaagaaggacaagcaggttacataacagataggggtaaacagacattaaag
aggttggaaaacacaaccaatcaaaaggctgagttagaagcagtattaatggcattacaagactcaggaccagaa
gttaacatagtaacagattctcagtatgcattaggaatattaatgaactgtcctacatcctcggaacacccagta
gtcgaacagattatgcaaaaggctatggagaaagaaaagatctacataacctgggttccagcccataaaggaata
ggaggaaatcaagaagtagatcaattagtaagtaagggaattaggaaaatactgtttctggataacatcccaaaa
gcacaggaagaacatgagaaatatcacactaatgtagaatacataaggcaagaatttcatctacccagacaggta
gccaaagctatagtagaaatgtgtcccaaatgccaaatcagaggagaaccaaaacatgggcaagtagacacagat
ctaggtacatggcaaatggattgtacccacttagaaggaaaagtaatttgtgtagcagtaaacacagccagtggg
tatacagaaactaaaatattgaaaagagaaacagggcaagaaacaggactattccttttgcaaatagcagcaaga
tggccaattaaacatatacatacagacaatggtccaaacttcatcagtgatgcctttgcggcagcctgttggtgg
gcaggagtcgaacacaccacaggaataccatacaatccacaaagccagggaatagtagaaaacaaaaatagacaa
ttaaaagaaaccatcaatcaaattagggaagaagtagaaagattagagacagcggtggcaatggccacgttcatt
ttaaattttaaaagaaagggaggaatagggggactacaccagcagataggtatattaacatgctatacacagaa
ctacaactacaaaatacacacacacaaaaattttcgaattttaaagtatattatagacatggtacttctgattgg
cagggaccagcttctctactgtggaagggagaaggagcagtagtgatccagacaccagatcaacaggtaatagca
gtacctagaaggaaagcaaaaatcatcagcagtaaaatgcagcatatattaaaggtacacaagtataaaagcaaa
caattagagaaagccacatataaacatcattatcagattacatgggagtggtggactcgagcacaatgggaaata
ccagtaggacaaggtgtattaattataaaattctatcataatttgacaccagagaaaggcctgttaagaacagag
ggagtaggattaatttgggcccatccgtctggatggagtacagaagtaacaccacaaacagcagatgcattagta
catagtcaatacttcccttgtttttcagatagggcagtccaacaagcacttagaggggagaagttgacttcgcac
tgctggaatttccataaagaacaggtactttcattgcaatatttggctttacaaaagtatttaagcaagtgcatg
ctagatatgcatagagcactcaactttatgctttttgaacattttgctgcattagaaggaacatttctctcaaat
gggcctcagactccttgtagcagatgctattgcaaaaagtgttgctatcattgttatagatgctttcttcaaaaa
ggcttagggataacaatgaaactttttaatatcataagtgtagtgttaggtatagtattaagtttaggcatagta
gctaggttagaacaatatgtcacagtattttatggagttcccaattgggaagatgctcaagtacctatgttttgt
gcaacccccccatacgggaggttgggcaacaaagaactgtgtaccatcttctgaacagatagaagtcagagttaat
attagtggggaatatttcactgcgtggaattcttcccatggcataagacaacagcttttgcaggacatgtctgat
cttttcctgcaggctgataggccttgtgtaaaattatctcctatgtgtatcagaacgttatgtgtagaggacaag
gacagtagtagtggtaatcttaccacaacaatcagtccaacaacaacaacaaacaaagcagcaacaccacctgca
tggtgggggacaactctacagaaactaggtacaattgttcatttaacatgacaggggttttaaggataagaaa
catcagtataatgcattcttttacaagtcagatataatgagagaggaaggaaatgaaagttattactatttacag
cattgtaatactagtgtaatgcgcaatgcttgtgaaaaacagacttttcgcccatttcccattcaatactgtgct
ccaccgggatactcactgttgaaatgtaatgatacagaatttgaaggagatgctgagtgtcaaaatgtaacagca
gtttcatgcacacatcctttcaacactttggctagtacatggtttcagttaaatggcacctataaagcaaaagac
aaagttaggtttattagacaaaaggataaaaatgaatcagtgattatactagtgccagaaaaattaggattacaa
ctggtgtgtgaaagaccaggaaatgagtctataaaaaacatccaattggcagcaggatttttcttgccagtgatt
caagggagattgaaaacagggaaagctgcgaaaagagcattttgcaaagtcaaaggagaatgggaaagtttttt
gagcaggtccataatgagagtataaaggtgtggaaaaatgtgacctcaacatcatggaggtcgcaaccacaagga
gacctggaagtgagaacacattggtttcaatgtggcggagaatttttttactgcaatgtttcaaagattttgca
aatgtgtcaaatggctatgctaatccaagcaattatgcaaaaaatttatatctctcgtgtgccatacgacagatt

ataaactattgggggtatgtaaccaagctaatgtatttgccacctacagaagggcacattaagtgcacttctaat
attactgctgtattaacagatatagagtactatccaaattcacagttgaattttgcacctactgctaatgtggaa
gatgtgtggagagctgatttattcaattataaattgattcggattaaacctattggctttgcgcctacaagtcaa
aggcgctatgagctgcctacgaaacagaaaagatcaaattggggatgcgcatttaagcaaatttgtcacacagca
gtaacgtggcagcaagcatgtggcaataactctcgttgcccaacaccccaatgggaaaacatgacatggcataca
tgggaacggcaggtagataatttgacagaccacatagataatttactgagagaagctcaggagcagcaagagaag
aatgtgcatgatcttactaaattacaagaatgggattccctgtggagctggtttgatctgtcgaaatggttccag
taccttaagataggattctttgccatagcagctatagtgatattaagagtacttagttttgcttggggcatagta
agaaatatgttgggaggctattctcctcttttgcagggacttgtaattgcctggggattcatcagtcccatcttg
gatcccaaaagagactttgactctgggggaaaaaatggaatgcgggagacatctgtcatgatgagggagatgag
gatctagtaggctttcctgttttaccacaagtacccttgagacccttaacttacaaacttgccatagatctctcc
cattttataaaaagaaagggaggactgcagggcatgtttttctgtcaaaagagacatgagattctacagctgtat
ttaaagaatgagcatggtgtgatagatgacatcacctatacgagtggccaggaacaagatacccgctgatcttc
gggtggttatgggaattggcccctaatgaaatagaagggtacttggtagatgaggaagacacactgatgatgcac
ccagcagcaggagtgggggcttcagaagacccacatagggagaatctcatgtggaacttcaatccacacttagcc
tatacaccaggctgggaaatggctcgtcagcaattagagagacaaacaggaaagcgctag
>#34 AY655744 7197 nt
atgggggcacggcactcggcgatgctcaccggtagggccttagataaatatgagaaagtgagactgaggccgaaa
gggaaaaagaagtatatggtgaagcatattgtatgggcttccaaagagatggagaggtttggcctgagtgacgcc
ctgttagagaccaaagaagggtgcgttaagatcatagaagccatttacatgttagaaccgaatggctcagaaggg
ataaagtctctctttggaatcgtgtgtgttctatactgcattcatgctggtatagacattgaggatacagagcag
gccaaggcagaagtaaggaaacgctgccacctaggggagaaaaaggaaacaacagaggaggaaaaagtaagaaag
gcaacagtgacatctagtggccaaacaggaaattaccccataataagaaatgcacagcagcagtatcaacatcag
gcactgtcacctaggctgctaaagacatggattgccactatagaggagaaaaaatttgcccctgaaacagtggcg
ctcttctcggcactagcggaagggtgcatcccctatgacatcaatcaaatgcttaatgcagtaggagagcatcag
gggcaatgcagatcataaaggacatcattaatgagcaggcatcagagtgggatcagatgcatccccagataggt
ccactaccagcaggacagttaagggaaccagcagggagtgacatagcagggacaacaagcactcccgctgaacag
gtggaatggaccaccagagcacagaaccccgtagatgtgggagcaatatacaaaagatgggtcatcatgggctta
cagcgctgtgtaaaaatgtacaatccagttaacatactggatgtgaaacaaggccccaggaaccattcaaagac
tatgtagacaggttctacagatgtcttagggccgaacaaacagatcaggcagtaaaaaattggatgacacagacg
ctgttggtccagaatgctaacccagagtgcaaggccatcttaaaagccatgggcccaggagccaccttagaagaa
atgctccaggcctgtcaaggggtaggaggaccccagcataagagcaggcttatggcagaagctatggcagaagcc
ttaaaacaaaacacccaacaagtcatagccatggtacaacaggggcaaggaggcccaaggggagaggggacct
agaaggaacccccccagggcagattaggtgttataactgtggaaaatttggccatatagcaaagaactgcccggcc
ccgccgagacagaaggtgccgccaggcacttgctataagtgtggcaaaccaggccacatagcaaaacaatgctcc
tcagcacaggccaatttttttagggaaagtgctgatagaaggacaagaggtggaggtactcctagacacaggagca
gatgacacaattatacaagtaggagaaatagaattggaaggagtaccccaccaaaaaacagtagggggattggca
ggatacatacaagtgcaaagttatagtgccatagaaatacagtgggaaaacaaaagagcaaggacacagttttg
attggaccaacgccaattaacattctagggagaaatttttagcaaaatttagtgttacacttaacatggtcagc
tcagacataccgataaccaaggttaaattaaaagagggtaaggaacccccaaaatcaagcaatggcccttaacc
agagaaaaaatagaagggctacaaatcatcatagacgacatggtgaaggcaggacagttagaggaggttggaccg
gaaaatccatataattctccagtgttcgctatcagaaagaaggacaaaactcaatggagaatgctcatagacttc
cgcgccctaaatcaagcaacccaagacttcgcggaagtacaacaggggatacctcacccctcaggcctgcctcag
atgaagcaaataacggtcctagacatgaaggatgcctattacagcatccccttagacccagacttctcacagtac
acagccttcaccatcccgtctctgaacaatgataggccaggaagaagatatcaattcaaggtgctaccccaagga
tggaagggttcgcccaccatcttccaacatacctcggatcaattattgaggaaattcagggacaagtaccctcag
gtagcactctttcaatacatggatgacctattggtaggaagcaatgagagcttggaggaacatagaaggatagta
ggagaactaagagctctcttaaacaaggtagggatccagaccccagaggctaaataccaagacaaaccgcccatt
aaatggctagggtatgaattgtatccagataagtggaaagtacagcctattgaattgccagataaactcacatgg
acagtcaatgagttacagaagttaataggaaaacttaattgggcatgccaaatatattctggtattaaaacaaag
gccctctgcaggtacctcaggggagtaaaaggacttctagaagaagtacagttatcagaggaagcagaggcagaa
ttagcagaaaacagggaaatcttagcagaagaaactcacggtacatattaccaggaaggagagcccctggaggca
gaactgaccaaattggcagaaggtcaatggggctatatgataaaacaaggcaaacggctcttaaaaacaggcaag
tttgccaaacagagaacagcacacagtaacccctatcaacagttaataggagctatgcagaaaataggcaaggag
tccatagtcttttgggggcaggtaccagtattcaggatcccagtggtcaaggaggaatgggaacagtggtggaca

gaacactggcaatgctcatggatacccaccatcgtgcccattcacacaccaccattagttaggctatggtataac
ctagtgcaggagcccatagaaggagcagacacgtattacgtagacggtgcagcccaccgagagtcaaaagaaggc
aaggcaggatacgtgactgcaacaggaaaagaacatgtcatcaagttagaaaaaactaccaaccaaaaggcagaa
ttggaagcagtactattggcactcaaagattcagggcccaaggtaaatatagtcacagactctcagtatgtctat
ggcatattggcaggaagccccacagaaacagataacaagataatagaagacattatccagctactactaagcaag
gaggcagtctacttggcttggctgccggcccacaaaggcataggaggaaatgaacaggtggataagttagtaagc
caaggaatcaggaagatcctttttgtagaacagatcccagaagcacaagaagaacatgagagataccataataat
tggagggatcttaaggcaagatttaagttacccaccatagtagccaaggctattatagaagcatgccccaaatgt
caagtacaaggagaacctaagacaggacagaacaatgcagctgtagggacatggcaaatggattgcacccatttg
gagggacaaatcatctgtgtagcagtacacgtggccagtgggtacatagagactaagattctgccaaggggagacg
gggcgggagaccgccctctttctcttacaagtggccagcagatggcctataagccacctgcatacagacaatggg
ccaaactttgtctcagcagaaatgcaggccatggtatggtggctcaaaatagagcactccgcaggagttccatat
aatcctcaatcccagggagcagtagaaaacaaaaataaacagctcaagaagacaatcacacaaattagggatgaa
gtacaatacctgtccacagcagtggcacaggcgactttcattttgaattataaaagaagaggggggattaggggat
atgtgcccagcagaagcaataattaacatgatctacacagaactacaaactacacaactacaaaaccaaattcaa
aatttttcggattttaaggtctattacagaaagggtgctaatcctctttggcagggaccagctcacctcatttgg
aaaggagaaggagcggttgtcttgcgaacagacgaaggagaggtaatcacagtccctagaaggaaggcaaaaata
attaagccctttcacatacataaaaccaagtacttacaaaagttgatttgggttcatcactttcaattgagacac
gcctcctatacacaagcaaagtggatactcccgttagaagaaaaggacaccagagtagaatatttaaccataaca
cagtattggtgtttagcaccagaccatggatatacgtccgattgggacacaatatccattgagtggtcagtctcg
gatatagtccgagacgaaaaggtgtacgccactgaagtaaccccaaatgtggcagatcacttgattcataccaaa
tatttcccatgttttacagcgtcagcagtcagacaagccatcagaggggaggtgctactagcagtttgtggagtc
agacacaaaggtagggacacgccaccgtctctacagttcctagccttgacagcagcagttagatggacaggagaa
caggcatgggctgcctcagtcatagattttgctaggaagggtaatcaaatgttactcctacatttccagcaaggg
tgctattgctattgcaagaaatgttgttaccactgccagctttgcttcttgcagaagggtttaggtatccggatg
aaatacttagcagttatttttttgcttagcttacttaggaaggtttataatagggaaccagtatgtcaccatctat
tatggagtaccagtgtggaaaaatgcaacacccccattgttttgtgccaccatggcaagggaccaatggtcaaca
tcccaatgcgtcccatcagatcctcagaatctcgaggtaccagtgaatctcactgagtacttcgatgtgtataaa
aattacatggtggaccacatagcagaagacatggatagcctctttttgacagccacaagaccatgtactaagtta
acaccaatatgtgtaaggatgaagtgtgaggacataacaaagaaccttacaatcagtgccaataccacaacacca
gccccgacggccatgagcaatagcaatatgccgtggtggggagacaatacaacagaaccagtatataattgtaca
tggaacatgacaggagcatttagggataagaggtatacctactttctcactggtacctagcagacctaatgaag
gaaccaggcaatggatccttctattatgccacaggatgcaatgtgtcagttatagcacaagcttgtgaaaaaact
cactaccaggcatttcctatacagtactgtgctccagcaggctttgccctgataaagtgtaatgatagcccatgg
acaggtagaggaaagtgtcacaacatctcagcagtacactgtacagaccctatcaccacaatagcagctacgtgg
ttaattctcaatgggacaaaggaaaatcacacacaggtcatacatcaggctccaaagaatgaaagtgctataatc
ctcctaggaaaggaacacaacctcacaatcacatgcataagaccaggaaataggacaataaaggacttacagata
gcagcaggactaatgtttcacagccagataatagcagggaagaacctcaacagggcctattgtaagctagagggc
aattggactaaagccatggaatcggccaatgaagaaatagcaaaaaggtacaaggagcatatcaacaacacagat
aaagtgaacatcacatggacggcagtcaggagaggagacctagaggtacaacttttctggcatgcatgtcaggga
gaattcttctattgcaacataagttctatgttcctgaatacttcctatgagtataactacacatcaaaccaaggc
aaattacaccaaggggtcataggaaagaagccgaggtcgagtgataggaccaagttctacatgtcatgccaactg
aggcaaatgatcaacaggtggagcagggtggagaagttaatgtacctcccacccagggaaggacacctgcagtgc
aaaagtaacatcacaggattgttggtggatatcaaccaccaccgcggctctagtgacataacagtagagccctcg
tcacagatacgtgactcgtggcgagcagcagtttcacggtacaaggtgatagagatcgtgccttttgggtatgcg
ccgacaaatatcagaaggtacgatgggcccgctaacaagcaaaaacgaaattcatgggggtgcgcatggaaacag
atctgctacacctcggtaccgtggaacaaaacatggtcgaattacacagatcctcaatggcagaatatgacatgg
caagaatgggagatgaaagtagacaatcacacagggctcatatcacagttgttacaagaagcacaagagcaacag
gaaagaaatgtccatgagttgcaaaagttaaatgactgggacagtctctggagctggttcaatttgtctgcctgg
tttagatggcttaggatagcagtaatagtagtagctagtcttatcttacttaggattgtaatgtacatagttaat
gtgctcaagttgctcaggcagggctacttacctcttccgtttgaagaatggcaagcaacaggagcggactacatg
ggcaaggcattaacacaaggagaggtttggtatgaaaaagaacaagaagaaaatgacatggaactagtgggcttc
cctgtgaggcctaaagttccgttgagaacctgtacttataaactaggtatagacttctcgttctttttgaaagaa
aagggaggactgaagggatattttacaacaaacgtaggcatgcaatcttgaatctgtatgcccacaatgaatgg
ggcatactgccagactggcagaactacacagagggaccaggaacaagatacccattgtgctttggtatcctgtgg

aaactctgcccagtagagatccatgaggatgacacggaggacggacatctcctcccgcatccagcatatgatggg
caggcagaagacccatggggagaatccctggtctgggtctttgatgagaagctggcctacacccccggagcgaag
atggccgagtgggacaggttggagagagagaagcggatgctgctagctccaccgcaaaccgcatcctcttga
>#35 AY523865 7326 nt
atgggggcggggcggtcggcaccattaaccggggcggctttagacagatttgaaaaagtaagattgagaccaaaa
ggcaaaaagaagtatatgataaagcacctcgtctgggcaggtaaggaactagacagatttggccttgcagcagca
ttattagaaaataaggaaggctgtcaggaaattttgactaggttacagcctttagaagcaacaggcagtgagaat
ttgaaatctctatatggcatagtctgtgtcatctgggcatgccatgccaacctctcggtggatgacaccgaggag
gccaaaaaggcagtgtacaggaggataaagcaggtggaacaggaggaagaattagaaatggccctaatccaaaga
cagaaagagaaggaagtagaacagaaacaacaaaagcaacaggaacaaagacagcaggagcctcaggcagcaccc
cagacaacaccatcagttaactaccctatcttaagacaggggcaacagtttgtgcatatgccattaagccccagg
atagtaaaaacctggataaatgctgtagaggaaaagaaatttctccagagatagttcctctttttcaagtcttg
gcagagggctgtacccccctatgacattaatggtctacttaatgcaattggagatcttcaggggggctatgcagatc
attaaagatgtaattaatgaggaagcagcagagtgggatttacaacacccacaacaacagccgccacaggggcag
cttagggaaccatcaggagcagacatagcaggaacaaacagcacagtggaagaacagatagcatggatgaccaga
ccagcaggtcaaggacaaggacccatagatgtaggacaaatatatagaagatgggtcatactaggtctgcaaaga
tgtgtgaaaatgtacaatccaaccaacatcttggatgttaaacaagggcccaaggaaccattcaaagattatgtt
gacaggttctataagaccttaagagctgaacaagcagatcaggcagtaaaaaattggatgaccaccaccctcatg
attcaaaatgcaaaccctgattgcaggatcatcctgaaaggattaggtcagaatcccaccttggaagagatgcta
catgcatgccaaggggtgggaggcccacagcaaaaggccagacttatggcggaggctatggcctcagccttaaaa
gaggcagggtccctaggcatggtacaacaaaggagaggacccccctggttctagaagacagattagatgcttcaat
tgtggacaaattggacatctccaaaaggactgcaaaagacctagaaagaccaaatgttttaaatgtgggcaggaa
ggccacattgcaaaaaattgtggacagactccgagggcaacagtacacatagaaggaagaaaagtccaggtgcta
atagatacaggggcagatgataccattatttcagaaaaagatatagatttagaggcaccttggaccccaaaaaca
gtagggggattaggaggtttcataaatgtcaaatgttacccagggatagaaattgcaatggctaataaggtagca
gtatcagatctattagtaggagataccccaattaatattctaggaagaaattatttagcaaaaatggggggtatct
ataaattttccagtattagattataccccagtaaaattaaaagaggggcagatggaccaaagattaaacaatgg
ccactttcaagggaaaaaatagaggccttaaaagatatcttaaagcccatgatagaggcaggacagatagtcccg
gcagcgcccaccaacccatataactctccagtgtttgtaataaagaagaaggacaaaagtaagtggcgaatgcta
atagacttaagagccttaaacaaggccacacaggaggtgtgggaggtgcagacaggtataccacacccatctgca
ctaccacaaatggaacagatcactgtcttagatcttgcagatgcctattattcaatagcactagacccaacattt
gcaaaatacacagcattcacagtaccatctgtcaataatatacaaccaggagaaagatatgagttcagagtactg
cctcagggatggagcgcttcaccagccatttttcaagcctcagtagggagacagttacagatctttagggaaaag
aatccagagctaataatagtccagtatatggatgatttactagtagggtcagatttaaggaaagatcaacaccta
aagaaggtagcacaattaagacagttcctcctagagaggggattgaggacaccacctgagaagtatcaggaagac
ccacccttttcactggatggggtatgagttacaccctaaaaaatggcggttgatgccagttaccctaccagatgaa
gaagaatggacagtgcataaaattcagaagttagtaggacagttaaattgggcaagccaaatttattcagggata
aaaacaaaacacttatgtagggcaatcagaggggtcccgggattaacagacccggttaccctctctgaagaagct
caagcagaacttagagagaacaaggaaatcctgaagcaagaagtgtcaggagtttactataaagagcaggaacca
ttaatagcagaattatccaaattagggaaaggacaatggggatatgtgatcagacaacctaagggaatattaaag
acaggaaaattctctagagataaaggagctcactataatgatttccatcaattagctaaggctatgtataaaata
ggaacagaaagcatagtttttttggggaagaataccacagttcaggctcccagtggtaaaagaagaatgggacaat
tggtggcataatcactggcaagccgcctggatcccagactgggaggcaatacataccccacatctggtcaagctc
tggtatgagctggtgtcagaacccataccagatgcagacacctactatgtagatggggcagctaacagggagtcc
aaattagggaaagctggctatgtcacagagtggggaaaacaaagtgtaaaatgcttagagaataccactaatcag
aaagcagaattagaagccatattgttggccttggaagaaggcccatcaaaaatgaatatagtaacagactcccag
tatgcactggggataatttttagaacacccatctgagacagaacataagatagtagaaaaggtaatacaggccttg
caagggaaagaacaaatttatctagcatgggtaccagcccacaagggaataggaggaaatgaccaggtagataaa
ttagtcagtaaaggaataaggaggatcctcttcttggaaagggtagaggaagcccaagaggaccatgaaaaatac
catgggaattggaaacaattaagggatgaataccaactacctacactcatagctaagcaaattataacacaatgc
cccaaatgtcagacaagaggggagcccacacatggtcaggtggatgcctcaatgggcatctggcagatggactgc
acccatctggaaaacaaaataataatagtggcagtccatgtggctagtggatatatagagaccaagatcctacca
gcagaaacaggaaaagagacagctctgttcctgttacagttaggagcaagatggccagtaactcagatacacaca
gacaatggacccaactttatcagtcaaccagtcaaggcagcttgctggtggctaggtattgagcacaccacaggg
gtaccatataacccacaaagccagggagtggtagaaagtaagaacaggattctaaaagataccatcaaacaaatc

agagatgatgcagagagattggaaacagcagtagcaatggctacctacataattaattttaaaagaaggggagga
atagggatatgtctccgatagaaagactagtaaacatgataacaacagaaatagaaacaaaaactttacaacaa
aaaatttcaaaactttttaggcttcaaggtctattacagagaaggtgctgatccaacttggaaagggccagcaacc
ctgctgtggaaaggagaaggtgcagtggtgtgcaagacagaggtaggagatatcaaagtggtccctagaaggaaa
gcaaaagtaatcaaagaattagataggtggtgcagtgctataaaaagagaaaaatacaagaccaaagaattagat
actgtacaatggatacaccattttgaattaaggaactcatattatacacaaacaaaaatagtattccccatcaca
aaagaaggggagtagtagtagaattactatggtgcttgaccccagagaaaggatggctacccactatggcagta
gcaatagcatgggaaacaaaaacatggaagacagaactaacaccagatttagcagatcaccttatacacctaaga
tactttccatgtttctcccaacaggcagtgagacaagccataagggagagatcctactgcaacagtgtcagcag
taccatagggaccagaaggtaaaggaccaccttcactacagtacatagcacttagagtagcagtaaaatacata
aaaatagttcaaaaaatgttatggttgcacctgagagaagcttgctttcacagggagagagaggctaccagaggg
tatcctaacattaggccattgacaggaagaaatagagaagttagggacggagaataaatggagagcatagatcca
tttgaagaaagagctaatacaccccaccccgcttgtatgtgcaaagcctgcattctacattgccagctgtgtttt
atacaaaagggtctagggatttccatgaggcttatacagcttttgctacttattttagggttaatagtgataggt
acacaggggaaacagaaaaagcaacaatacttgacagtatattatggagtaccagtctgggtagatgccaaagtt
gatttgttctgtacagccaactcatcagagtcaggatgggcagtgacagcatgcctgccacatgccttagtgaga
gaagaagtcccaatgccaaatgtgacccaaaactttaatgcttttgataaccccatagaggagcagctatggcag
gatatgactagtttgtacaaacagtcattcaaaccatgtgtaaagctaacaccatattgtgttagcatgcagtgc
ataaaaacatcaacaaatccaacaccaacaaacacatcaacaacaacaacaacaatagcaacaacaacaaaaaca
actacagattggtcaggggaaaacataaccatgacacaatattggaattgttcatttaatgtctcaggtccttat
agagataagaaagaaaagagctcagcagtatggctagaggatgatatccagtgggcggataataaagatggtagt
gggaacagaacagggtacatgaaacactgtaatgactctgtcatcacccagtcttgtgaaactagccgctttaaa
ccttttaagatcaggtactgtgcaccagctggatatggtctactaagatgtgatgataaaaattttaatggtaca
gggttatgcaacaacgtgactgcagtggcatgtacaaatctcatacacaccatggcatccacatgggtacaattt
aatgggtctgatgaggagagggcagaagagcttcatataataagaaaagaggtaaaaggtgaggtgcagaatgga
agcattactataagagtgcccgctaaatacaacttgacattaacatgtgtaaggccaggaaataagacctaccga
gccatacacatggctacaggtctgtccttttatactacctttatacaaaggctgaggataaagagagcacattgt
agactaaacggaagctgggcaaacgcaacaaaggaaatgcgacaaaagatattggaaatatttggaaaggcaaat
aggacaaataatctgacaatacactacccaaaaggagatagggaagtacaaagtgtctggtttcagtgtcatgga
gaattcttctattgtaacatttccaaagccctagatctcctactcctacaaaataacactagaaatagcacttgg
tctgacaagtggttgatgccttgtaggataaatcagttagtaaacacttggtatacggtagggcaacatatctat
ctacctccgaaagaaggagagttgaagtgcagctcacacatttctgcatttgtttttgatgtagatcactataat
gggagtataaccccttacaccatcagcagacatcagggcagtatggagagcagacctatttaaatataaaatcata
gaagtaaaacctataggattcgctccctccgcagtgagaaggtacgagggaccggagtcggtaagacacaagagg
aatgaatggggctgcgccttcaaacagatctgtcataccacggtcccttggaataactctctggaggatccagac
tgggacaatatgacctggcaggagtgggaaatgaaggtagcaaattatacagatgagtgggaaggagctttacag
agagcacaagagcagcaggagaggaatgtacatgccctacaatcactacaagattgggactctttatggaattgg
tttgacctgtccagatggttctggtggattagattagtagtatacataatagctgctttaatattgctaagaata
gctatgtttggagtgaatattggaagtagaggctttgaattactgcaagaaggaatatatagactgtcatttgct
ctttggcaaagactgagactcttgggggaacgctttagagtgcttgggggtacttacagttctggggaagtcct
agaagagaaataaagtgcaccaataagcaacaacaagacttagatcagacagaagcagtaggcttcccagtacgc
ccccaggtatccatccgagaccccacatataagttaatgatagactactctcatttttttgaaagaaaagggtgga
ctggaggatgtttttttactctgccagaaggcatgctattttagagcttcatgcccagaatgaatggggaattata
ccaggctggctacagtacacaaagggacctggagttaggtatccaatttacttcgggttcctctttaaactggtg
ccagtagaaatagcagacccagattatgagaatgatgagaggaacatcttgttacatgatgcccaccaggggatg
atggaagacccccatagagagaggttggtttggaagtttgacagcactctggcctattgttataaggcaggccat
gctgaacaaagagagcatactagaagatgtatgttccctaagaggaagtag
>#36 AY523866 7269 nt
atgggggcggggcgatcggcgccattaacgggggcagctttagacagatttgagaaaataagattgagaccaaaa
ggcaagaaaaagtatttgataaagcacctcgtctgggcaagtagggaactagacagatttggccttgcaggagca
ttattggaaaccaaagaaggatgtcaagagatcttaactagattacaacctttagaggcatcaggcagtgaaggc
cttaagtccctatacggcatagtatgtgtcgtttgggcatgccacgccaacctctcggtagaagataccgaggag
gcaaaaaaggcagtggctagaaggataaagcaggcagagcaggaggaagagttagagatggcattaattcagaag
aaagcagaaaaggagttagagaaaaagcagaaaactcaggcagcagcagcagacacacagcaaaaaacaaaccaa
ccagctaattttccaatcctgagacaaggacagcaatatgtacatatgcctttaagtcctaggattgtgaaaacc

tggataaatgcagtggaggaaaagaaattttccccagagatagtgcccctatttcaagtattggcagaagggtgt
actccctatgatattaatgggctgctaaatgccataggagatctacaaggggcaatgcaaattataaaagatgtg
attaatgaggaagcagcagagtgggacttacaacacccacagcaacagccaccacaaggtcagcttagagagcca
tcaggagctgacatagcaggaacaaacagcacagtggaagagcagatagcttggatgaccaggccagcaggacaa
ggagcaggcccaatagatgtaggacagatatacaggagatgggtcatcctaggcttacagagatgtgtaaaaatg
tacaatccaactaacattttggatgttaagcagggtcctaaggaacccttcaaagactatgtagatagattctac
aaaaccttgagggctgaacaagcagatcaggcagtcaaaaattggatgaccaccaccctaatgattcagaatgct
aacccagattgtagaataattctaaagggttaggacagaaccccactttagaagaaatgttgcatgcatgccag
ggagtaggaggtccccaacagaaagcccgtctgatggcggaggctatggcctcggccttaaaagaagcaggctcc
ctggggatggtgcagcaaagaggaggacaaggaggaggtcctagaagacagcttagatgctttaattgtggacag
attgggcatgtgcaaagggattgtaaaaagcctagaaaagttaaatgcttcaaatgtggtaaggagggccacata
gcaaagaactgtggacaagtaccaagggcagcagtatatgtggaaggaaaaagggtccaggtgctaatagacaca
ggtgcagatgatacaattatttcagagaaagatttagatttagatgcaccttggaccccgaaaacagtgggggga
ttaggggggatttatcaatgtaaaatgctacccagggatagagattaaaatggcagacaaggtagctgtttcagat
gtgttagtaggtgataccccaatcaacatattgggaaggaattatttggcaaagatgggggtatccataaatttt
ccagtattggactttacaccagtaaagttgaaggagggagctgatggcccaaggattaaacagtggcccctttca
agagaaaaaatagaagccttaaaagagatcataaggcctatggtagaagcaggacagatagtccccacagagccc
actaacccatatagctctcctgtgtttgttataaagaagaaggacaagaataaatggagaatgcttatagacctg
agagcccttaataaggctacccaggatgtctgggaagtgcaaacagggatcccgcacccctcggggttgccacaa
atggaacagataacagtcctagacttggcagatgcctattattcaatacccttggatccacagtttgcacgttat
acagcattcacaataccttcggtgaacaatacacaaccaggagagagatatgagttcagagtactacctcaaggc
tggagtgcatcaccagccatattccaggcaacagtgggcaggcagctgaaactttttaaggaaaaatacccagac
ttaattatagtccaatatatggatgacctattggtaggatcaaatttagataaggtctcacatttagaacaggta
aaacagcttaggaaatttctcctagaaaaggggttaaggacacccccggagaaataccaggaggatcccccatac
cattggatggggtatgagttacatccaaagaaatggcacttgttgccaatagagctaccagaagaggaggagtgg
acagtccacaaaatccagaaactggtaggacagttaaattgggcaagtcagatatactcaggaataaagaccaaa
cacttgtgcaaggcaatcagaggggttccagggctaacagataaagtaagcctttcagaggaagcacaagcagaa
ctgagggagaataaagaaactcccaaacaggaagtctcaggggtatactacaaagaagatgagcccctaatatca
gagatttccaaaataggaagaggacagtggggatatgtaattagacaaacaaagggattgctaaaaacagggaaa
ttctcaaaggacaagggaactcattataatgactttcatcaactagctaaggcaatgtataaaataggcacagag
agtatagtctattggggaaaagttcccaaatttaggttgccagtagtaaaggaagaatgggataactggtggcat
aaccactggcaggccacttggatccctgactgggaggcggtgcataccccacaactagtcaggctatggtatgag
cttgtatcagagccaataccagatgctgacacctattatgtagatggagcagctaatagggaatcaaaaatggga
aaagcaggctacatatcagaatggggaaaacagggagtaaaaagtctggaaaatactaccaatcaaaaggcagaa
ttagaagccatcctactggccttggaggagggaccctccaagatgaacatagtaacagactcccagtatgcttta
ggaatattgttagaacaccccacggaaacagaacatgagatagtagaaaagatcatacaagccttacaagaaaag
gaacaggtatatcttaattgggtaccagcccacaaaggtataggagggaatgatcaggtagacaaattggtaagt
agaggaataaggagaatcctattcttggaaagagtagaagaggctcaagaggatcatgagaaatatcatggtaat
tggaaacaattgagagatgaatttcagttacctaccttaatagccaagcagattattgcgcagtgtccaaagtgc
cagataaagggagagcctatgcatggtcaagtagatgcctccatgggggtatggcaaatagattgcacccaccta
gaaaacaaagtcatcatagtagcagtgcatgtagctagtggatttatggagactaaggtcttatcagcagaaact
ggaaaggaaacagccttattcttattacagttgggggcaagatggcccattaaacagatacacacagacaatgga
ccaaatttcataagtcagccagtcaaggcagcatgttggtggttgggaatagatcatactacaggggtaccatat
aacccacaaagtcaaggggtagtagaaagcaaaaatagggtcctaaaagaaactatcaaacaaattagagaagat
gcagaaagactagaaacagcagtagcaatggccaccttcattattaattttaaaagaaggggaggaataggggat
atggcagcaatagagagactagtaaacatgataactacagacttggaaacacaaaccttacaacaaaaaatttca
aaacttttaggttacaaggtatattacagagaaggagctgacccaacttggaagggacccgctaccttactgtgg
aaaggagaaggagcggtggtgtgtaaaacagaggtaggagacataaaggtagtaccaagaagaaaagccaaggtg
ataaaagatttagataagtggtgtagtgccataaaaagaggaaagtataagaccaaagaattacaagcaataaag
tggatacaccattatgaattaagaagggcattctacactcagacaaaaatagtcttccccgtcacagaagaagga
aaagtagtagtggaaatattctggagcctggctccagaaaaagggtggatgccctctatggcggtaggaatctct
tgggagcagacagggtggcagacagaattaacacctgacctagctgatcatttgatacacttaagatacttccct
tgtttctcccaacaggcagtcacacaagccctaaagggagagaaactactacaacagtgtcagcaataccatagg
ggaccagaaggtaaaggaccaccctcattacagtacttagcactaaggacagcagtaaaatatataaaaatagta
caaagaatgctttggcttcatctgagagaagcatgcttccatagagagagggaagctactaggagataccccaat

attagacccctaacaggaagaaacagggaggtcagggatggagaataaatggaagaaatagatccattcaaggaa
acagctaatacaccccatcctaattgtatgtgcaaagcctgtactatgcattgccagctgtgctttatgcaaaaa
ggtctagggatctacatgcttagcttattaataataatagcaagtctgaccctggtaggggcagcaccaacaaag
caacaatgggtgacagtatattatggagtgcccgtatggcacaatgccactgttggcttattctgtacagctaat
agttcagaatcaggatgggctgtgacagcatgcttgccacatgcaatggttagagaacaagtccctatgcctaat
gtgtcccagtattttgactcttttcaaatgttatggaacaacaattatgggacgacatgactagcttatacaga
caatcttttaaaccctgtgtaaagttaaccccatattgtgtgcggatgaactgtagtagtacaacaacaacaaca
acagcagcaacaacaacaataaaaacaacaactgactggtcaggagagaataagacagtgacacagtattggaat
tgtaccttcaatgttacagggccatatagggacaagaaggaaatgcaaagtgcagtgtggttagaggatgacata
cagtgggagacagaaaaccaaacaaatactaacaaaagtataagacatgggtatatgaaccactgcaacaattca
gttataacacagtcctgtgaaaccagtcgctttaagccttttaagataagatactgtgcaccagctggatatggt
ctgttaagatgtgatgacagaaactttaatggtaccggcttgtgtaccaatgtgactgcagtggcatgtacaaat
ctcatacataccatggcatcaacatggatgcaattcaatggttcagatgaagaaagggcagaagacatacatatc
ataaggaagggcctggaaaataagaccattactgtcagaatcccaccaaagtataatctatctttggattgtgtt
aggccagggaacaagacgtatagagctgttcatatggccacagggctatccttttatacaaccttcataccaagg
ctgaggataaagagagcacattgtaggatgagaggtaattggacaggagcagttaaggagataaggagcagatta
aaggagatatataatgtgacaaaaataacagtacactatccaggaggtgatagggaggtacaaaacacgtggttt
caatgccatggagagttcttttattgtaatgtttccaaagcatttaatctgctcatgaataaaactaataacata
acctggagtaataacactttgatgccttgtaagattaatcaattggtaaacacatggttcaaggtaggacaacat
gtatatttgcctccaaaggaaggagaattgaagtgtcattcttctgtttctgcacttgtttttgaggtagctcat
tatgggcaaaacatgaacataacaccctcctcagatattaaggcagtttggagggcagatctatacaagtataag
atcatagaagttaaacccatcgggatggcccctacaagtataagaaggtacgagggaccggagtctgcaagacaa
aaaaggaatgaatggggatgtgcctttaaacaaatctgtcacactacggtcccttggaaccatacatggggagaa
cctgattggaataatatgacctggcaagagtgggaaaggaaagtagcaaactatacagatgaatgggaaggagcc
ttacagagagcacaggaacaacaagagagaaatgtgcatgccttacagtcactgactgattgggattcattatgg
aattggtttgacctatccagatggttctggtggattagattagtagtgtatataatagcagctttgatattgctt
aggatagcaatgtttggggttaatatcgggactaagagaggctttgagctcctgcaagaaacaatatatagactt
tcatttgcattttggcaaagactgagactcttggggaacatttaagagtggtctggggttattgtcaagaacca
ctctatgcagacccccacagagagataaaatgcactcagagagcccaggagtgttatgatgaggaacatgggata
ggctttgctgtcagaccaagggtcccaattagggaacccacttacaaattgatgatagactactctcattattta
aaagaaaagggtggactggaggatatttttactctgctagaagacatgctatattagaaatccatgcccaccat
gaatggggaataataccaggttggttgaagtacacagaaggcccaggaccaaggtatccaacatactttgggttc
ctatttaagctagtgccagtagagatagcagatcctgactatgagaatgatgagagaaatatcctcttacatgat
gcccaccaaggacaagcagaagacccccataaggagaggctagtatggaagtttgattcctcccttgcctattgc
tataaagcaggacatgaagcccataaaccagagcataacagaagatgcatgttccctaagaggaagtag
>#37 FJ919724 7212 nt
atgggagcggggcgatcagcaccgttaacggggaaagctttagacaaatttgaaaaggtgaggttaaggccaaaa
ggtaaaaagaagtatatgataaagcacctcgtctgggcagctagggaattagataggttcggcctctcagaggca
ttgttagaaaacaaggagggatgtcaggagattctaaccaggttaacacctttggaagcaacaggcagtgagaat
ctcaagtctctttatgggacatgttgtgtcatatgggcctgccatgcaggtctccaaatagaggacacagaagaa
gcaaagaaggcagtaaaacttagaattgctcaagtagaagcagaggaccaattagaaatgcaggtcctgcagaaa
aaagcagaggcggaagcagcaaagaaacaaggggcagtatcagctcaagcccaaccagcccaggttcaatcagca
gccccacaacagggaacagtagtaaggggcaactacccaatcctcagacaaggggacagtatgtgcatatgcct
ctaagtccaaggatagtaaaaacttggataaatgcagtagaggagaaaaagttctccccagagatagtccctctt
ttccaagttctagcagagggctgtaccccatatgacatcaatgggttactcaatgccataggagatttacaaggg
gcaatgcagataataaaggatgtaatcaatgaggaagcggccgagtgggatttacaacacccacaacagcagcca
ccccaaggacagttgagagaaccctcaggagcagatattgcagggaccaacagcacagtagaagaacaaattgca
tggatgacaagagcccagggagtgggaggaggacccatagatgtaggacaaatttatagaagatgggtaattttg
ggtctacaaagatgtgtaaaaatgtataatcctaccaacatcttggatgtgaaacaagggcccaaagagccattc
aaggactacgtagacaggttctacaagaccttgagggcagaacaggcagaccaggcagtaaaaaattggatgacc
accacccttatgattcaaaatgctaacccagattgcaggacaatcttgaaggctttgggacctaatcctactcta
gaggagatgctgcatgcatgccaaggggtaggaggaccccaacagaaagcaaggctcatggcggaggcaatggtc
tcagccatgagggagggaggaggccttaccatggtacaacagaggggaccaggagggccaagacctgggaataga
attagatgctataattgtggacaaattggccatgttcaaaaggactgtaggaagcccagaacagttagatgcttc
aaatgtcaacagacagggcacatggcaaaaaactgcccaaaagcagctcctcagtcaaagaatacaatagagata

gagggaagaagggtgcaggttctattagatacaggggcagatgatactataattcatgagaaagatattcaactt
acctcaccctttgtcccaaaaacagtagggggtctgggaggttttataaatgtaagatgttatccaggatagaa
atccaatgtcaaggtaaggtagcagtaggggaagtcctagtgggagagacacccattaatatttagggaggaat
ttcttagcacccctaggcttctcgataaacatgctagagagtcagattcaatataccccagtgaaattaaaagaa
gggaagtcaggaccaaaagtcaaacaatgggcctctagccagggaaaagatagaggctctcagggaaatagtggg
aaagatggtggaggaaggccaatggctcctgcctcacccaccaatccatataactcacctgtgtttgccattaaa
aagaaagacaagaacaaatggagaatgttaatagatcttagagaattgaacaaggcaacacaaccagtttgggag
gtccagacagggatcccgcacccagcaggtctgccacagaaaaggcagataacagttttggacatagcagatgca
tactattctataccattagatccatcctttgctcagtatacggcatttactataccatcagttaataacacagcc
cctggagagaggtatgagttcagggtgctgccacaaggatggagtgcatcaccagctatattccaacacactgtt
catcgggtattggaaggatttagaaagaaatacccagatgtactcatagtccagtacatggatgacctattggta
gggtcagacagaacagaagaacaacacagactagtagtcaaaacactaagagattatctcctagagagggggtta
aaaacaccccccagagaaatttcaggcagagccccccctatcagtggatgggatatgtccttcatccaaaaaagtgg
actacccaaccaattcagatccctgaggaagaggaatggactgttaataaaatacagaaactagtaggaatcttg
aattgggcaagccaaatatattcagggataaaaacaaaacacctgtgcaagagtattagaggggttccagggtta
acagaccctgtaaagttaacagaagaggcacaggccgagctggaagaagccagacagatcttaaaagaacaagtg
tcagggacctattacaaagaacaggaggacctcatagcagacattactaagctttcagaagggcaatggggatac
accattagacagtcaaagggaatcctgaaaacaggcaaatatgcaaaggcaaaaggggcacatttcaatgatttt
catcagatagcaaaattgatgatgaaggtaggaacagaaagcatagtaacatggggggaggttacccacatttagg
ttacctgtccagaagcaggactgggatggatggtggcatgagcattggcaagccacttggatcccagaatgggag
gcggtacacactcctcccctagtcagattatggtattccttggtttcagaacctataaaagaagcagatacatat
tatgtggatggagcagctaacagagaatccaaggaagggaaagcagggtacatttcagagagaggaaaacagagt
gtaaaggccttagaaaacactaccaatcaaaaggcagaattaacagcagttctcatggccttagaagatagtggg
gaaaaggtgaacatagtaacggactcccagtatgtactaaacatactaacagaacaccccacaaccactgaacat
gaattagtagagaaaataattcaacaactacagaagaaacaggaagtatatcttcagtgggtgccagctcataaa
ggtctgggaggtaatgaacaaatagataaattagttagtaagggaattagaaagatcctttttctagaaagagta
gaagaggctcaggaagatcacgataaataccacagtaattggaaacagctaagggaggagtttaatctccccacc
ttagttgcaaagcaaataattaatcaatgtcccaaatgtcaaatacatggggaacccaaacatggacaagtcaat
gcagacttgggtatttggcaaatggactgcacacatttagaaggaaaagtcataatagtagcagttcatgtggct
agtggatttacagaaacccaggtgttaaaggaggaaacaggaaaacagacagctttattcctgttacaattaggg
gctaggtggcccatacaacaagttcacacagacaatggacccaactttattagtcaggcatttgctgccgcctgt
tggtggttagggatagagcacaccacaggggttccatacaacccacagagtcaaggagtagtagaaaacaaaaat
agacaattaaaggatacaatcacacaagttagggaagatgcacaacgattggaaacagcagtagctatggctact
cacattcttaattttaaaagaaggggaggaataggggatatgaccccttcagaaaggatagtcaatatgattaat
acagaactagaaataaaatacctacaacaaaaaatttcaaaattttcaggattcaaagtctactacagagaagga
gctgatcccagttggaaaggcccagcaacactcctgtggaaaggagaaggagcagtggtaatcaaattggacaca
ggggatttaaaagtagttcccagaagaaaagcaaaagtaataaaggacttagatagatggtgtagtgctataaaa
aagggaaaatataaaaccaaagaattggctcctataacatggcaacatcactttgaactcagaaatagttatttt
actcagacaagaataatatttcctatcacaaaagaaggaaaagtagtagtagaactcttctggtgcctagcgcca
gaaaaaggatggttgccaggaatggcagtaggtctcagttgggagcagggaaactggcagacagagctatctcca
gaattggcagaacacttaatacacttaagatatttcccctgttttactcaacaggcggtgaggcaggccattctg
gggagaagctgctcacgtgctgccagcaatatcatagggggagcaggaggtaacggaccaccttcattacagtac
atttgcttaagggtagccataaaaagggcagcagcttcaagacattaccctaacatcagaccactgtcaggaaga
aatcaggaggttagggatcaggaataaatggaggaggaaatggacctattccaagggagagggagaggagaagca
aatcaccctagttgctattgcaaaatctgtacttttcattgctacttgtgcttcacgcaaaaaggcttagggatc
acgatgcttgctttcatattagctattatagtaattatcttaggacaaaagacagaacaggttagccagcaacaa
cagtatgtgacagtattttatggggttccagtttggaaaaatgcctcagtgggattgttttgcacagcaaatggt
tcagagtcaggatgggcagtgacagcctgcctacccacactccgtgataagagaacaggtacacatgccaaatgta
tcacagtactttgatgcattcaagaatcccatagaagaccaattatgggaagatatgacagggttatataaacaa
acatacagaccctgtgtaaagttaacaccttattgtgtaaagatgcggtgtaagactgaaacaaccctaacatca
aacctaacaactccaaagacaacaactgattggagtggagaaaacaacaccatgtcacaatattggaattgctct
ttcaatgtctcaggtccatatagagataagaaagaagagggacatgccatgtggctaacagatgatctggaatgg
gcaaatggaaatgagacaggggatactaggggagcttatatgaggtattgcaacaggtctgtaataactcaagcc
tgtgacaaaagtcactttaaacctttcaaaataaggtactgtgcaccagctggatatggtctaatgagatgtgat
gacaaaaatttcaatggtacaggactttgtaccaatgtgtctgcagtggcatgtacaaacttaatacacacaatg

gcatccacctggttacagttcaatgggtcagatgaggaaagagcagaagaaattcatgtcataagaaagggtctt
gaaaataagacaataacaattaggattcctcccgagtacaacctccgtttagactgtgtccgtccaggtaataag
acatatagagcaactcacatggccacaggattatcattttacacaacctttgtagaaaggctaaggatcaaaaga
gcacattgcagaatgcgaggtaattggagtggcgcagtttctgacataaaacaagtattaaagggcatatataat
gttactaacataacaatgcattacccaggaggagatagagaggtacaaagggtatggttccaatgccatggagaa
tatttttattgcaatgtcagtaaggcctatcaacttttcaccaatgacaccataacatggagtaacaacactttg
atgccctgcaaaattaatcaattggtaaacacctggtacaagatagggcagcatatatacttgccaccaagagaa
ggggagttgaggtgtgactcacatgtttctgcaataatttttgatgtggctcactatggacacatgttgaatttg
accccatcatcagatattaaggcagtgtggagagcagatctgtataagtacaaaatcatagaagtgcagccaata
ggctttgcacctactgctgtcagacgctacgagggaccagagtctgtgagacaaaaaagaaatgagtggggatgt
gcttttaaacagatctgccacaccacagtgccttggaatggatcaataggacaaccagattggaataacatgaca
tggcaggaatgggaacagaaggtagcaaactacacagatatatgggaagaagcattacagaaagcacaggaaaaa
caggagcagaacgtacatgccctacagtcactacaagattgggactctttgtggaattggtttgacctatccagg
tggttctggtggattagaatagtggtgtatgtaatagcaggattagtattgcttagaataataatgtttgtagta
aatataattagtctgagctttgctatttggactttgctacaaagacttggggaacaactaagagtactttgggga
tacttgcaagaaacctttataggaaatccaagaagagaaatcaagtgtacacagagacaacaacaagattttgac
caacaagaggaagtgggatttcctgtcaaacccagggtcccaattagagaccccacctataaattaatgatagac
tactctcatttttaaaagaaaagggtggactggaggatatttttactcagccaggaggcatgcaatcttggaa
ctgcatgcccaaaatgaatggggaataatacctggttggctccagtacactgagggcccagggattagatatcct
aagtactttgggttcctctttaagctagtgccagtagagatagcagacccagactatgagaatgatgaaaggaac
atcttgttacatgatgcccaccagggtcagatggaagacccttataaggagaggctggtatggaagtttgattca
caactagcctactgctataaagcaggccatgaagctcatactaaagagacacatactaggagatgtatgttccct
aagaggaagtag
>#38 AF208027 7281 nt
atgggcgcgagaagctccgtcctgtcagggaaaaaagctgatgaattagaaaaagttaggttacggcccggcgga
aagaaaaaatacatgttaaaacatgttgtgtgggcagcgaacgaattggacagattcggattagcagagagcctg
ttggaatcaaaagaaggttgccaaaaaattctttcagtattagctccattagtaccaacaggttcagaaaattta
aagagcctttataatactgtctgcgtcatttggtgtttgcacgccgaacagaaagtaaaacatactgaagaagca
aaacaagtagtgcagagacatctagtggtagaaactggaactacagaaaaagtgcctgccacaagcagaccaata
gcaccacctagtggcagaggaggaaactacccagtgcagcaagtaggcggcaactatgttcatctgccattaagt
cctcgaactttaaatgcatgggtaaaattggtagaagaaaagaagttcggggcagaagtggtgccaggatttcag
gcgctgtcagaaggctgcctaccatatgatatcaatcaaatgctaaattgtgtaggagaacatcaagcagccatg
caaattattagagaaataattaatgaggaggcagcagaktgggatcttcaacatccacaacaaggccgccgcca
gcaggacaamtcagggaaccgacaggatcagatatagcagggaccactagtacaatagaagaacaaattcagtgg
acacacaggcagcaaaaccctataccagtaggaaatatytatagaagatggatccarttaggacttcaaaaatgt
gtcaggatgtayaatccaacaaacatcctagatgtaaaacaagggccaaaagaaccctttcagtcttatgtagat
agattctacaagagtctragagcagaacaaactgatccagcagtaaaaaattggatgacacagacactactgatt
caaaatgccaacccagattgtaaattagtactaaaggggttgggaatgaatcctactttagaggaaatgttgaca
gcctgtcaagggrtaggaggaccagggcaaaaagccaggcttatggctgaagcattaaaagaagccttaaaccct
acagccctaccatttgcagctgctcaacaaaaaacaggaggaaaaaggagcacaataaaatgttggaattgtggc
aaggagggacatacagttagacagtgcagagcccccagaagacaggggtgctggaagtgtgggaagccaggccac
attatggccaaatgtccagagactgcatacatagaaagtcagccagtagaagtactattagacacaggggctgat
gactctatagtagcaggaatagaattaggaagtaattatagtccaaaaatagtaggaggaataggaggatttatt
aataccaaagaatacaaaaatgtagaaattaaagtggtaggaaaaagaatacaggctactgttatgacaggggat
acccctataaatatctttggaaggaacattttagttaagctaggcatgtctctaaattttccaattgcaaaggta
gagccagtaaaagtaaggctaaaacctggcatggatgggccaaaaatcaggcaatggccccctatctaaggaaaaa
atacaagcactcagggaaatttgtgacaaaatggaacaggaagggcaattggaagaagcacccccccaccaatccg
tataacacacccacctttactatcaagaagaaggacaaaaacaaatggagaatgttaatagacttcagagaactt
aacagggtaacccaggactttactgaagtccagttgggcatacctcaccctgcaggtttggcagaaaaaaggaga
ataacagtactggatatagggggatgcctacttcagcattcctctagatgtagatttcaggcagtacacagctttc
actttaccatcagtaaacaatgcagaaccaggaaaaagatacatctacaaggtcctaccgcaaggatggaagggc
tcaccagctatcttccaacatacaatgaggaaggtactagaccccttcagaaaagccaatgaggatrtaattaty
atccaatatatggatgacattcttgtggccagtgacagaactgatttagaacatgatcaagtagttttgcagcta
aaggaactcttaaataaaacrgggttctctaccccctgatgaaaagttccaaaaagaccctccatatcaatggatg
ggatatgaattrtggccaaaaaagtggaaactacaaaagattaacctgccmgaaaaagagacttggacagtaaat

gatattcaaaaactagtaggggtactgaaytgggcagctcagctatatccaggaatcaaaacaaaaaacctgtgy
aaattaattagaggtaaratgaccttgacggaagaagtacagtggacagaattggcagaagcagaattgcaagaa
aacaagattatcctaraacaggarcaggaaggcgcctattacaaagaagragagcctttrgaagccactgtacag
aaaaatttagacaatcagtggacatacaaagttcatcaaggaaacagaatcctaaaagtaggtaaatatgcaaag
ataaaaaatacccatacaaatggagtaagattattggctcatgtggtccaaaaratagg aaaggaagcactagta
atatggggaaaattacccttctttcatctgccagtagaaagggacacctgggaacaatggtggacagactattgg
caagtaacttggatcccagattgggactttgtgtctacaccgcatctagtaaggttagtctacaacctagtcaaa
gaacctctagagcagacagagacctattatacagacgggtcctgcaataaaacctccaaggarggaaaagcaggc
tacgtcacagacagaggaaaagacaaggtaaargtcttagagcagacaacaaaccagcaggcrgaacttgaggca
tttgcaatggccttgcaggactcaggtccaaaagtcaatatcatagtrgattcccagtatgtcatgggratcata
gcaggrcaaccaacggaaacagaatccccgttagtaaataaaataattgaagaaatrattaaaaaagaarctcta
tatgtaggatgggtaccagctcataaaggaataggaggtaatcaagaggtagaccrtctagtaagccagggaatt
agacaagtactattcttgraaaaaatagaacctgctcaagaagaacatgaaaartttcatagtaatgtaaaagag
ttaactcataagtttggcattcctcaattagtagcaaaacagatagtaaattcataccacagttgccaacagaaa
ggagaagccattcatggacaggtaaatgcagaattgggtacttggcaaatggactgcacccatttagaaggaaaa
gtaatcatagtagcagttcatgtggccagtggctttgtagaagcagaagtaatcccacaagaaacaggaaggcaa
acagcattattcctgttaaaattagcagggagatggcctatcacacacctacatacagacaatggtgccaacttc
acctcgcaagaggtgaagatggtagcctggtgggtaggcatcgagcaagcatttggagtaccatacaacccccag
agtcaaggggtagtagaatcaatgaatcatcatctaaagaaacaaatagataaaattagagatcaagcaaattca
atagaaaccatagtgttaatggcagtacattgcatgaattttaaaagaaggggaggaataggggatatgacccct
gcagaaagaataattaacatgattactacagaacaagaaatacaatttcaacaaacaaaaaattcaaaatttaaa
aattttcgggtctattacagagaaggcagagaccagctctggaaaggacctggtgagcttttgtggaaaggggaa
ggagctgtcatcataaaggtagggacagaaatcaaagttgtacccagaagaaaagcaaaaatcataaaagatctg
ataaaattcttaaagtttaaaacaaaagacctacagaaggcggtttatgttcctcatcacaaagttggatgggct
tggtggacttgcagcagggtaatattcccattaacaaaagaagcacatttggaaatacaagggtattggaacttg
acccctgaaaaggatggttaagccagtatgcagtaagattaacctggtacacaagaaaattttatacagatgtg
acaccagagacagcagatcaattattgcatggatcttattttgattgctttacagctggtgaagtaagaagagcc
atcaggggagagcagatattgtcctgctgcaactatccaacagctcataaaaggcaggtgcctagcttgcaattt
ctagccttacaagtagtgcaaaaaggaatggcagaagaaatcccccagaagatgaggcccccagagagagcca
tgggatgaatgggtagtagaagttctggaagaaatcaaagaagaagccttaaaacattttgaccctcgcttgcta
actgcgcttggtaactatatctatgataggaaatgttattgcaaaaaatgctgctatcattgtcagttatgcttt
cttaacaagggactcggagtatgtatggcatatcttggcaatcagctgcttatcgcaaccttrctcttaagtact
tatgggttctattgtaccaaatatgtcactgtttttatggcatacccgcatggaggaacgcgagcgtacctctc
ttctgtgcaaccaaaaatagggacacatggggcactacacagtgccttcctgataatgatgattacagtgaacta
gcattgaatgtaacagaagcttttgatgcctgggataacacagtaacagaacaggcagtagaagatgtttggaat
ctgtttgagacatctacaaaaccttgtgttaaattaacacctctgtgcattgccatgagatgcaataaaactgag
acaaacaaatggggtctaacagggaccaccactgtaacccctgcaacagttcccacaactaaaatggtgacagct
gaactagtgaatagcactagccagtgcttaatgtacgataattgtacaggtatacagtcagaatccatggttggc
tgcaaatttaacatgacagggctaaaaagggatcagagaaaggaatacaatgagacttggtattcacaagatttg
caatgygaacagtccaataagagtgagaatgagagtagatgctatatgaggcactgtaatacatcagtcattcag
gaatcatgtgataaacattattgggatgctataagatttagatattgtgctccgccagggtttgctttgctaaga
tgtaatgatactaactattcaggatttgcacctaattgctctaaagtagtggtctctacatgtacaagaatgatg
gaaacacaaacctctacatggttcgggtttaatggaacaagggcagaaaatagaacatatatctattggcatggg
agagacaataggactataataagtttaaataagtattataatttaacaataaratgtaggagaccaggaataaa
acagtattaccaattactattatgtcagggctagtcttccactctcaaccaatcaatgaaagacccagacaggct
tggtgttggtttggaggcaaatggaaggaagccatgcaggaggtaaaggaaaccgtggtcaagcaccccagctat
aaaggaacaaatgacacaaagaamataacctttacaacaccaggagaaggttcagatccagaagtaaagttcatg
tggactaattgcagaggagaattcttgtattgtaaaatgacttggtttctaaattggatagaggacagaaatatg
actctcctaaagccccaggaacgacaaagacgcaattatgtaccctgccatattaggcagataattaacacatgg
cacaaagtaggaaaaaatgtgtacttgcccccagagaaggtgatctgacgtgtaattcaacagtcaccagccta
atagcgaatatagaagagaataggtctaacaaccatacaaatatcattttcagtgcagaggtggcagaactgtat
cgattagaattggggattataaattagtagaaataactccaattggctttgcacccacaaatgtaaagagatat
tcctctgtgacaccgaaaaataaaaattcatggggatgtgcgtttagacaggtctgccacactactgtaccttgg
gatgcccttggtgctaataaaacattggagccacaatggaataatatgacatggcaagaatgggaaaagcagatc
aactttttggaggacaacatcacacggctattagaagaagcacaaattcagcaagaaaaaaatatgtatgagtta

cagaaattaaatagctgggatgtatttggcaattggtttgacctaacctcctgggttaaatacgtttacttaggc
ctttatgtagtggcaggagtaatagtgttaagaatagtcatatatgtagtacaaatactaggaagacttaggcaa
gggtataggcctgtttyttcttcccctccctcttatgttcagcaggaacatctccggctagaagcagcttatttc
agcgggcaacaatacacgcagggtcaatttatgaataccccatggagaaacccagcaacagaaagagcaaagtta
gcatacaggyagcaaaataatgatgatgtagatagtgatgataatgacttagtaggagtaccagtctaccccaaa
gtcccgctaagagtaatgagttacaaattggcaatagacatgtcacattttataaaagaaaagggggactggaa
gggatttattacagtgaaagaagacatagaatcctagacatatacatggaaaaggagcaaggtataattccagat
tggcagaattacacagcaggaccaggtataagatatccartgacgtttggctggctatggaaactagtaccagtc
acggtctctgatgaggcacaggaggacgagactcattgcttggtacatccagcacaaactagcccatgggatgac
ccggagaccgaagtgttggcgtggaagtttgaccctacattagcctatgactacagggcatttatattgcatcct
gaggagttcaggtggaagtcagggctaccagaagcagtatggaaagagaaactaaaacagagaggactgcctata
gaataa
>#39 M30502 7080 nt
atgggcgcgagaaactccgtcttgagagggaaaaaagcagacgaattagaaaaagttaggttacggcccggcgga
aagaaaaagtacaggttaaaacatattgtgtgggcagcgaatgaattggataaattcggattggcagagagcctg
ttggagtcaaaagaaggttgccaaaagattctcagagttttagatccattagtaccaacagggtcagaaaattta
aaaagccttttaataccgtctgcgtcatttggtgcttgcacgcagaagagaaagtgaaagatactgaggaagca
aagaaactagcacagagacatctagtggcagaaactggaactgcagagaaaatgccaaatacaagtagaccaaca
gcaccacctagtgggaaaagaggaaactaccccgtgcaacaagcgggtggcaactatgtccatgtgccactgagc
ccccgaactctaaatgcatgggtaaaattagtggaggaaaagaagttcggggcagaagtagtgccaggatttcag
gcactctcagaaggctgcacgccctatgatattaatcaaatgcttaattgtgtgggcgatcaccaagcagctatg
caaataatcagagagattattaatgaagaagcagcagactgggattcgcagcacccaataccaggccccttacca
gcaggacagctcagagacccaagagggtctgacatagcaggaacaacaagcacagtagatgaacagatccagtgg
atgtataggccacaaaatcccgtaccggtagggaacatctacagaagatggatccaaatagggctgcaaaagtgt
gtcagaaagtacaacccaactaacatcttagacataaaacagggaccaaaagaaccgttccaaagctatgtagac
aggttctacaaaagcttgagggcagaacaaacagacccagcagtaaaaaattggatgacccaaacgctgctaata
cagaatgccaacccagactgcaagttagtactaaaggactggggatgaatcccaccctagaagagatgctaacc
gcctgccaggggtaggcggaccaggccagaaagccaggctaatggctgaagccctaaaagaggctatgggacca
agccctatcccatttgcagcagcccaacaaagaaaggcaattaggtattggaactgtggaaaggagggacactcg
gcaagacagtgccgagcacctagaagacagggctgctggaagtgtggcaagccaggacacatcatggcaaactgc
ccggaaagacaggcaggtttttagggttgggcccacggggaaagaagcctcgcaacttccccgtgacccaagcc
cctcaggggctgataccaacagcacctccggcagatccagcagcggaactgttggagagatatatgcagcaaggg
agaaagcagagggagcagagggagagaccatacaaagaggtgacggaggacttgctgcacctcgagcagagagag
acacctcacagagaggagacagaggacttgctgcacctcaattctctctttggaaaagaccagtagatgacagga
gataccccaatcaacatctttggcagaaatattctgacagccttaggcatgtcattaaatttaccagttgccaag
atagagccaataaaagtaacattgaagccagggaaagatggaccaaggctgaaacaatggcccctaacaaaagag
aaaatagaagcactaaaagagatctgtgaaaaaatggaaaaagagggccagctagaagaggcacctccaactaat
ccttataataccccccacatttgcaattaagaaaaaggacaagaacaaatggaggatgctgatagatttagagaa
ctaaataaggtgactcaagatttcacagaaattcagctaggaattccacacccggcaggactagccaaaaagaaa
aggatctctatattagatgtaggggatgcctatttttccataccactacatgaagattttaggcagtatactgca
tttaccctaccagcagtaaacaatatggaaccaggaaaaagatatatatataaagtcttgccacaaggatggaag
ggatcaccagcaatttttcaatacacaatgaggcaagtcttagaacctttcagaaaagcaaacccagatgtcatt
ctcatccagtacatggatgatatcttaatagctagtgacaggacaggtttagagcatgacaaagtggtcctgcag
ctaaaagaacttctaaatggcctagggttttctactccagatgagaagttccaaaaagaccctccatttcaatgg
atgggctgtgaactatggccaactaaatggaagctgcagaaactacaactgccccagaaagacatatggacagtc
aatgacatccaaaagctagtgggagtcttaaattgggcggcacaaatctattcaggaataaaaaccaaacactta
tgtagactaattagaggaaaaatgacactcacagaagaagtgcagtggacagaactagcagaagcagagctagaa
gaaaacaaaattatcttgagccaggaacaagaaggatattattaccaagaagaaaaagaattagaggcaacaatc
caaaaaagccaaggacatcaatggacatacaaaatacaccaggaagagaaaatcctaaaagtaggaaagtatgca
aagataaaaaatacccataccaatggggtcagattactagcacaggtagttcagaaaataggaaaagaggcacta
gtcatttggggacggataccaaaatttcacctgccagtggagagagagacctgggagcagtggtgggataactac
tggcaagtgacatggatcccagagtgggactttgtatctaccccaccactggtcaggttaacatttaacctagta
ggagatcctataccaggcgcagagaccttctacacagatggatcatgcaatagacagtcaaaagagggaaaagca
ggatatgtaacagatagaggaaaagacaaagtaaaagtattagaacaaactaccaatcagcaggcagaattagaa
gtctttcggatggcactggcagactcaggcccaaaggttaatatcatagtagattcacagtatgtaatggggata

gtagcaggccagccaacagagtcagaaaatagaatagtgaaccagatcatagaagaaatgataaagaaggaagca
gtctatgttgcatgggtcccagcccataaaggcataggaggaaaccaggaagtagaccatttagtaagtcaaggc
atcagacaagtattattcctggaaaagatagagcccgctcaagaggaacatgaaaaatatcatagcattataaaa
gaactaacccataaatttggaataccccttctagtagcaagacagatagtaaactcatgtgcccaatgccaacag
aaaggagaagccatacatgggcaagtaaatgcagaaataggcgtttggcaaatggactacacacacttagaagga
aaaatcattatagtagcagtacatgttgcaagtggattcatagaagcagaagtcatcccacaggaatcaggaagg
cagacagcactcttcctattaaaactggccagtaggtggccaataacgcacttgcacacagacaatggccccaac
ttcacttcacaggaagtgaagatggtggcatggtgggtaggtatagaacaatcctttggagtaccttacaaccca
caaagccagggagtagtagaagcaatgaatcaccacctaaagaatcagataagtagaattagagaacaggcaaat
acaatagaaacaatagtactgatggcagttcattgcatgaattttaaaagaaggggaggaataggggatatgacc
ccagcagaaagactaatcaacatgattaccacagaacaagaaatacaattcctccaaagaaaaaattcaaatttt
aaaaatttccaggtctattacagagaaggcagagatcagctgtggaaaggacctggtgaactactgtggaaggga
gaaggagcagtcatagtcaaggtagggacagacataaaagtagtaccaagaaggaaggccaagattatcagggac
acttatttctcttgctttacggcaggtgaagtaagaagagccatcagaggggaaaagttattgtcctgctgcaac
tatccccaagctcataaagcacaggtaccatcacttcaatacctagccctagtagtagtacaacaaactgaagca
ccaacagagtttcccccagaagatggaccccacggagggacttagggagtgactgggtaatagaaactctgagg
gaaataaaggaagaagccttaagacattttgatccccgcttgctaattgctcttggctactatatccataataga
caaggagaagaactccttgcccagctgcaccgacccctagagccatgcactaacaaatgctattgtaagcgatgc
agtttccattgccagctgtgtttctcgaaaaggggctcggaatatcaatggagcctggtaggaatcagctgttt
gttgtcattttactaacaagtgcttgcttagtatattgtagccagtatgtgactgttttctatggcatacccgcg
tggaaaaatgcatctattcccttattttgtgcaactaaaaatagagacacttggggaccatacagtgcttgcca
gacaatgatgattatcaggaaataattttaaatgtgacagaggcttttgatgcatggaataatacagtgacagaa
caagcagtagaagatgtctggcatctatttgagacatcaataaaaccatgtgtcaagctaacacctctatgtgtg
gcaatgaattgtagcagggttcaaggaataccacgaccccgaatcccaggacctcgagttccacaacctcgaga
ccacccacatccgcagcctccataataaatgaaacttctaactgcatagaaaacaacacatgcgcaggattaggg
tatgaggagatgatgcaatgtgagttcaatatgaaggggttagaacaagataagaaaaggaggtataaggacaca
tggtatttagaagatgtggtttgtgacaacacaacagctggcacatgttacatgagacattgcaacacatcaatc
atcaaagagtcatgtgataagcactattgggatgctatgaggtttagatactgtgcaccaccgggctttgcccta
ttaagatgtaatgataccaactattcaggctttgaacctaagtgcactaaagtagtagctgcttcatgcacaagg
atgatggaaacgcaaacttctacttggtttggctttaatggcactagagcagaaaatagaacatatatctattgg
catggcagagataataggactatcattagcttaaacaagtattataatctcacaatgcgttgtaagagaccagga
aataagacagttttaccaataacacttatgtcaggattagtgtttcactctcagccaatcaacacaaggcctagg
caggcatggtgccggtttggaggcagatggagggaagccatgcaggaggtgaagcaaacccttgtacaacatccc
agatacaaaggaatcaatgatacagggaaaattaactttacgaaaccgggagcaggctcagacccggaagtggca
tttatgtggactaactgcagaggagaatttctctactgtaacatgacttggttcctcaattgggtagaagacaag
aaccaaacacggcgcaactattgccatataaagcagataattaatacctggcataaagtagggaaaaatgtatat
ttgcctcctagggaaggggagttggcctgtgaatcaacagtaaccagcataattgctaacattgacatagataaa
aatcggactcataccaacattacctttagtgcagaagtggcagaactgtaccgattagaactgggagactacaaa
ttaatagaaataacaccaattggcttcgcacctacagatcagagaaggtactcctcaactccagtgaggaacaaa
aattcatggggatgtgcgtttagacaggtctgccacactactgtaccgtgggtaaatgactctttatcgcctgac
tggaaaaatatgacatggcaggagtgggagaaacaagtccgctacctagaggcaaatatcagtcaaagtttagaa
gaagcccaaattcaacaagaaaagaatatgtatgaattacaaaaattaaatagctgggatattcttggcaactgg
tttgacttaacctcctgggtcaagtatattcaatatggagtgcatatagtagtgggaataatagctttaagaata
gcaatctatgtagtgcaattgttaagtagatttagaaagggctataggcctgttttctcttcccccccggttat
ctccaacaggcggcccaactgcaaggacagcagtatcagcagggagagtacatgaacagcccatggagaaaccca
gcaacagaaagacagaaagatttgtataggcagcaaaatatggatgatgtagattctgatgatgatgacctaata
ggagttcctgttacaccaagagtaccacggagagaaatgacctataaattggcaatagatatgtcacattttata
aaagaaaaaggggggactgcaaggatgttttacagtaggaggagacatagaatcctagacatatacctagaaaaa
gaggaagggataataccagattggcagaattatactcatgggccaggagtaaggtacccaatgtacttcgggtgg
ctgtggaagctagtatcagtagaactctcacaagaggcagaggaagatgaggccaactgcttagtacacccagca
caaacaagcagacatgatgatgagcatggggagacattagtgtggcagtttgactccatgctggcctataactac
aaggccttcactctgtacccagaagagtttgggcacaagtcaggattgccagagaaagaatggaaggcaaaactg
aaagcaagagggataccatatagtgaataa
>#40 AF082339 7074 nt

atgggcgcgagaaactccgtcttgagagggagaaaagcagacgaattagaaagaattaggttacggcccggcgga
aagaaaaaatatcagctaaaacatattgtgtgggcagcgaatgaattggacagattcggattggcagaaagcctg
ttggagtcaaaagaaggttgccaaagaattcttaaagttttagaaccattagtgccaacaggatcagaaaattta
aaaagccttttaatactgtctgcgtagtttggtgcgtgcacgcagaagagaaagtgaaagatactgaaggagca
aaacaaataatacagagacatctagcggcagaaatagaaacagcagagaaaatgccaagcacaagtagaccaaca
gcaccacctagtgaacagggggaaacttccccgtacaacaagtagccggcaactacacccatgtgccgctgagc
cccgaaccttagatgcttgggtaaaattagtagaagaaaagaagttcggggcagaagtagtgccaggatttcag
gcactctcagaaggctgcacgccctatgatattaatcaaatgcttaattgtgtgggcgaccatcaagcagccatg
caaataatcagggagattatcaatgaagaagcagcagactgggatgttgcacatcccataccaggccccttacca
gcagggcagcttagagaaccaagagggtctgacatagcaggaacaacaagcacagtagaagaacagatccagtgg
atgttcaggccacggaatcctgtgccagtagggaacatctatagaagatggatccagatagggctacagaagtgt
gtcaggatgtacaacccaaccaacatcctagacataaaacaaggaccaaaggagccattccaaagctatgtagat
agattctacaaaagcttaagggcagaacaaacagatccagcagtaaagaattggatgactcaaacactgctggta
cagaatgccaacccagactgcaaattagtgctgaaaggattagggatgaatcctaccttagaagagatgctaacc
gcctgtcagggagtaggggaccaggccagaaagccagattaatggcagaagccttaaaggaggccatgacacca
gctcctatcccatttgcggcagcccaacaaagaaggacaattaagtgctggaattgtggaaaggaagggcactcg
gcaagacaatgccgagcacccacagcctacattgagggccagccagtggaagttttactagacacaggggctgac
gactcaatagtagcaggaatagagttagggagcaactataccccaaaaatagtaggggaatagggggattcata
aataccaaagaatatgaagatgtagaaataaaagtactaaataaaagagtaaaagccaccataatgacaggtgac
accccaatcaatatttttggcagaaacattttgacagccttaggcatgtcattaaacctaccagttgccaagata
gagccaatagaggtaagattaaagccaggaaaagacgggccaaaattaagacaatggcccttaacaaaagaaaaa
atagaggcactaaaagaaatctgtgaaaaaacggaaagagaaggccaattagaggaggcacctccaactaaccct
tataataccccacatttgcaataaagaagaaggacaaaaacaaatggagaatgctaatagattttagagaatta
aacaaggtaactcaagatttcacagagattcagttagggattccacatccagcaggattagccaagaaaagaaga
atcactgtgctggatgtaggggatgcttacttttccataccactgcatgagagctttagacagtatactgcattt
actctaccatcagtaaacaatgcagaaccaggaaaaagatatatatataaagtcttaccgcagggatggaaggga
tcaccagcaatttttcaacacacaatgagacagatcttagagccattcagaaaggcaaaccaggatgtcattctc
attcaatacatggatgatatcttaatagctagtgacaggacagatttagaacatgacaaggtggtcctgcagtta
aaggaactcctaaatggcctaggattttccaccccagatgagaagttccaaaaagaccctccatacaaatggatg
ggctatggactgtggccaactaaatggaagctgcaaaaaatacaattgccccagaaagaagtatggacagtcaat
gacatccaaaaacatgtgggtgtcctaaattgggcagcacaaatctacccaggaataaagaccaaacacttatgt
agactaattagaggaaaaatgacactcacagaaggagtgcagtggacagaactagcagaagcagaactagaggag
aacagaattatcttaagtcaggaacaagaggggcactattaccaagaagaaaaggagttagaagcaacagtccaa
aaagatcaagacaatcaatggacatataaaatacaccagggagaaaaaattctaaaagtggaaaagtatgcaaaa
atgaaaaatacccataccaacggggtcagattgttagcacaggtagttcaaaaaataggaaaagaagcactggtc
atttggggacgaataccaagatttcacctaccagtagaaagagaaacctgggaacagtggtgggatgactactgg
caagtgacatggatcccagactgggactttgtatctaccccaccactggtcaggctagcatttaacctagtaaaa
gatcctatactaggcgcagagaccttctacacagacgggccctgtaataggcaatcaaaagaaggaaaagcagga
tatataacagatagagggagagacaaggtgaaggtactagaacaaactaccaatcagcaagcagaattagaagcc
ttcgcgctggcagtaacagactcaggtccaaaagccaatattatagtagattcacagtatgtaatgggaatagta
gcaggccagccaacagaatcagaaaatagaatagtaaatcaaatcatagaagaaatgataaaaaaggaagccatc
tatgttgcgtgggtcccagcccacaaaggcataggggaaatcaggaagtagaccatttagtaagtcagggcatc
agacaagtattgtttctagaaaaaatagagccagctcaggaagaacatgaaaagtatcatagcaatgtgaaagaa
ctatcccataaatttggattacccaatctggtggcaagacagatagtaaacacatgtgcccaatgtcagcagaag
ggagaggctatacatgggcaagtgaatgcagaactaggcacttggcaaatggactgcacacacttagaaggaaaa
gtcatcataatagcagtgcatgttgccagtggattcatagaagcagaggtcatcccgcaggaatcaggaagacaa
acagcactcttcctattaaaactggctagtagatggccaataacacacttgcacacagatagtggtgtcaacttc
acctcacaggaagtaaagatggtagcatggtgggttggtatagagcaatcctttggagtaccttacaatccacaa
agccaaggagtagtagaagcaatgaatcaccacctaaaaaatcagataagcagaattagagagcaggcaaataca
gtggaaacaatagtactaatggcagttcattgcatgaattttaaaagaaggggaggaatagggggatatgacccca
gcagaaagactcatcaatatgatctccacagaacaagaaatacaattcctccaaacaaaaaatttgaaatttaaa
aatttcccggtctattacagggaaggcagagatcagctgtggaaaggacctggggagctactgtggaaaggggac
ggagcagtcatagttaaggtagggacagacataaaagtagtaccaagaaggaaggccaagatcatcagagacctt
gtcaagtacctaaagtacagaacaaaagatctagaaaaggtgtgctatgttccccaccataaggtgggatgggca
tggtggacttgcagcagggtaatattcccattacaaggaagaagtcatctagagatacaggcatattggaaccta

acaccagaaaaaggatggctctcctcttatgcagtaagaataacttggtatacagaaaagttctggacagatgtt
accccagactgtgcagactccctaatacatggcacttatttctcttgctttacggcaggtgaagtaagaagagcc
atcagaggggaaaagttattgtcctgctgcaattatccccaggcccataagtcacaggtaccgtcactccaattt
ctggccttagtggtagtgcaacaaactgaagcaccaacagagtttcccccggccggaatgggaccccaccaggga
gccagggatgagtgggtaatagaagtcctgagagaaataaaagaagaagctttaaggcattttgaccctcgcatg
ctaattgctcttggcggctatatctatactagaatgatgtctagtagaaatcagctgcttgttactatcttacta
gctagtgcttgcttagtatattgtaaacaatatgtgactgtttttttatggcgtgccagcatggaaaaatgcatcc
attcccctcttttgtgcaaccaaaaatagagatacttggggaaccatacagtgcttaccagacaatgatgattat
caggaaatagctttgaatgtgacagaggctttcgatgcatgggataatacagtaacagaacaagcagtagaagat
gtctggagactatttgagacatcaataaaaccatgtgtcaagttaacacctttatgtatagcaatgaagtgtagc
aacataagcacagagagcacaaccacatccccgagcccagggagcacactcaaaccccctgataaatgagagcgat
ccatgcataaaggcagacaactgccccaggggactaggggatgaagagatggtcaattgtcggttcaacatgaca
ggattacagagagataagccaaaacagtataatgaaacatggtactcaaaagatgtggtttgtgaaccatttaac
accaccacaaaccagaccaggtgttacatgaaccattgcaacacatcagtcatcacagagtcatgtgataagcac
tattgggatgctataaggtttagatactgtgcaccacctggttacgccctactaagatgcgatgatatcaattat
tcaggctttgcacccaattgctctaaagtagtagctgctacatgcacaaggatgatggagacgcaaacttctact
tggtttggctttaatggcactagggcagaaaatagaacatatatctattggcatggtagagataatagaactatc
atcagcttaaacaaacattataatcttactatgcattgtaagaggccaggaaataagacagttgtaccaataaca
cttatgtcagggttaatatttcactcccagccaatcaataaaagacccagacaagcatggtgctggttcaaaggc
gaatggaggaaagccatgcaggaggtgaaggaaacccttgtaaaacatcccaggtataaaggaaccaatgacaca
aaccaaattaactttacaaaaccaggaagaggctcagatgcagaagtggtatatatgtggactaactgcagagga
gaatttctccattgcaacatgacttggttcctcaattgggtggaaaacaaaacgggtcaggaacagcacaattat
gcaccgtgccatataaagcaaataattaatatctggcacaaagcagggaaaaatgtatatttgcctcctagggaa
ggagagttgacctgcaactcaacagtaaccagcttgattgctaacattgacacggatggcaaccagacaaatatt
acctttagtgcagaggtggcagaactataccgattagaattggggggattataaattagtagagataacaccaatt
ggcttcgcacctacatcagaaaggagatactcctctactccaaggaggaataaaaattcatggggatgtgcattt
agacaagtctgccacactactgtaccatgggtaaataactccttaaaacctgattgggacaacatgacgtggcaa
gagtgggaacaacaagtccgttacctagaggcaaatatcagtgaacagttagaacgggcacaaattcagcaagaa
aagaatacgtatgaactacaaaaattaaatagctgggatgtttttaccaactggcttgacttaaccgcctgggtc
aagtatattcaatatggagtttatataatagtaggaatagtagctcttagaatagtaatatatgtagtgcaaatg
ttaagtagactcaggaagggctataggcctgttttctcctcccctcccggttacatccaacaggcagcctacctg
cagggacagcggtatcagcagggagattttatgaataccccatggagaaccccagcaacagaaagagagaaagaa
ttgtacaagcaacagaatatggatgatgtagatttagatgatgatgatagcctagtaggggtctctgtcacacca
agagtacaattaagaacaatgacatacaaattggcagtagatatgtcacatttaataaaagaaaggggggggactg
gaagggatgttttacagtgagagaagacatagaatcttagacatatacttagaaaaggaagaagggataattcca
gattggcagaactatactcatgggccaggaataaggtacccgatgttctttgggtggctgtggaagctagtacca
gtagatgtcccacaagaaggggaggacactgagactcactgcctgctacacccagtacaaacaagcaggcatgat
gacacgcatggggagacattagtttggagatttgaccctaagctggctcatgattacaaagcctttattctacac
ccagaggaatttgggtacaagtcaggcctgccagaagatgagtggaaggcaagactgaaagcaagagggatacca
tttagtaagaacaggaacagctga
>#41 U27200 7065 nt
atgggcgcgagaggctccgtcttgtcagggaagaagacagatgaattagaaaaggttaggttacggcccggcgga
aagaaaaagatacatgctaaaacatatagtatgggcagtgaatgaattagagagatttggattggcagagagccgg
ttgggatcaaaagaaggatgccggaaaattcggaaagtcttaggaccattagtaccaacaggttcagaaaattta
aaaagtctttataataccgtctgcgtcatcttttgcctgcacgcagaagagaaagtgaaagatactgaggaagca
aaaaagatagcacagcgacatctagcagcggacacagaaaaaatgccagctatgagtaaaccaagtaaaccaact
agccgcctagcttatccagtgcagcaaatagctggcaattattcccacctgccgctaagcccccgaaccttaaat
gcctgggtaaaattagtagaagagaagaagttcggggcagaagtagtaccaggatttcaggcactatcagaaggg
tgcacccccttatgatattaatcagatgttaaattgtgtaggagaacatcaggcagccatgcaaattattaggag
ataattaatgaggaagcagcagactgggaccaacagcatccatcgccaggcccaatgccagcagggcaactcagg
gaaccaagagggtcagacatagcgggaaccaccagcacagtagaagaacagatacagtggatgtacagaccccaa
aatcctgtcccagtgggaaacatctatagaaggtggattcagttagggctacagaaatgtgtccggatgtacaat
cctactaatatactggacataaagcaagggccaaaagaacccttccaaagctatgtagatagattctacaaaagc
ttacgggcagagcaaacagacccagcagtaaaaaattggatgacacaaacactgctgattcagaatgctaaccca
gattgcaaattagtgcttaagggcttgggaatgaaccccacattagaagaaatgctaacagcctgccaggggata

ggaggcccagggcagaaggcaaggctaatggctgaggctttaaaagaagccttgacaccttccaccaatccgttt
gccgccgctcaaccaagagcagggaagaggacagtgacatgctggaactgcggcaaggcgggacatacagccagg
cagtgcaaggcccctagaaggcagaaagcaactattgagggtcaatcagtagaagtattactagacacaggagct
gatgactcaatagtagcagggatagaattaggcagcaattacaccccaaaaatagtaggtgggataggaggattt
ataaataccaatgaatacaaaaatgtagaaatagaagtagtaggaaaaagagtaagagcaacagtaatgacaggg
gacaccccaataaacatttttggcagaaatattttaaatagcttaggcatgactctaaatttcccagtagcaagg
atagaaccagtaaaagtccagttaaagcctgaaaaagatgggccaaaaatcagacaatggcccctatccaaagag
aaaatactagccctcaaagaaatctgtgaaaaaatggaaaaagagggacagttagaagaggcgcctcctactaat
ccatacaattcgcccaccttcgccataaaaaagaaagacaaaaacaaatggaggatgctaatagatttcagagaa
ctaaacaaggtaacccaagaatttacagaggtccagctgggtattcctcacccagcaggactggcatcaaagaaa
agaataacagtactagatgtaggagatgcctacttcagtgtcccactagatccagacttcagacaatatacagca
tttactttgccagcagtaaataatgcagaaccaggaaagagatatctttacaaagtcctaccacagggatggaag
ggatccccagcaattttccagtacaccatggcaaaggtactagaccctttcagaaaagccaacaatgatgtcact
ataatccagtacatggatgacattctcgtggcaagtgacaggagcgatctggagcatgacagggtagtgtctcaa
ctaaaagagctattaaataacatgggattctctactccagaagaaaagttccaaaaagaccctccattcaaatgg
atggggtatgagctctggccaaagaaatggaaactgcaaaaaatacagctaccagaaaaagaggtttggacagta
aatgacattcagaagttagtgggagtattaaattgggcagctcaacttttcccggggattaagaccaggcatata
tgtaaactaataaggggaaagatgaccctaacagaagaggtacaatggactgaattggcagaggcagaattccag
gaaaacaaaatcatcctagaacaagagcaggaaggatcctattacaaagaaggggtacctttagaagcaacagtg
cagaaaaatctagcaaatcagtggacatacaagattcatcagggagataaaatcctaaaagtaggaaaatatgca
aaggttaaaaacactcacaccaatggagtaagactattggctcatgtagtccaaaaaataggaaaggaagcattg
gtcatctggggagagataccaatgttccatctaccagtagaaagagagacatgggatcagtggtggacagattac
tggcaagtaacctggatcccagaatgggattttgtctcaaccccaccattaataaggttagcctataacctggtc
aaagacccccctagaaggagtagaaacttactacacagatggatcctgtaacaaagcctcaaaagaaggaaagca
ggatatgtcacagacagggggaaaggataaagttaaaccattagaacaaacaacaaatcagcaagcagagcttgaa
gcatttgcactagcactacaggactcaggaccacaggtcaatatcatagtagattcacaatatgtcatgggaata
gtagctgcacaaccaacagaaacagaatcaccgatagtaagagaaataattgaagaaatgatcaaaaaggaaaaa
atatatgtaggatgggtaccagctcacaagggactgggtggtaatcaggaagtagaccacctagtgagccaagga
attagacaaatcctatttctagaaaaaatagaaccagctcaagaagaacatgaaaaatatcataataatgtaaaa
gaactagtccataaatttgggattccacaattagtggcaagacaaatagtaaattcctgtgataaatgccaacaa
aaaggggaagctattcatggacaggtaaattcagaactagggacatggcaaatggactgtacacatttagaggga
aaggttataatagtggcagttcatgtagccagtggattcatagaagcagaagtaataccccaagaaacaggaaga
cagacagctctcttcctgttaaagctggccagcagatggcctatcacacacctgcacacagacaacggtgccaac
ttcacttcacaagatgtgaaaatggcagcctggtggatagggatagaacaaacattcggagtgccctataatcca
gaaagtcagggagtagtagaagcaatgaaccatcatctgaaaaatcagatagacagaattagagatcaggcagta
tcaatagagacagttgtgttaatggcaactcactgcatgaattttaaaagaaggggaggaataggggatatgacc
cctgcagaaagaatagtcaacatgataactacagaacaagaaatacaattcctccaaacaaaaaatttaaaattc
caaaatttccgggtctattacagagaaggcagagatcaactctggaagggacctggtgatctattgtggaaaggg
gaaggagcagtcatcataaaggtagggacagaaatcaaagtaatacccagaagaaaagcaaagatcataaggaac
tacctgaaatataggacaaaagacttgcaacaggtctcttatgttcctcaccataaggtaggatgggcttggtgg
acttgcagtagagtaatatttcccctgaaagaaggagcacatctagaagtccaaggatactggaacctgacccca
gaaaggggattcttgagttcctatgctgtaagactaacatggtatgagaggagcttttatacagatgtaactcct
gatgtagcagaccgattactgcatgggtcttatttctcttcgtttacagctaatgaagtaaggagagccatcagg
ggagaaaagatattgtcccactgcaactacccatcagctcatacaggccaggtaccaagtttacagtttctagcc
ctaagagttgtacaagaaggaaaagcagaagcagtcccagagattcctccagaggataaaaacccacaaagagaa
ccgtgggaacagtgggtagtggacgtcctggaggaaataaaacaagaagccttaaagcattttgacccccgctta
ttaactgcacttggcaattttatctacaatagggcatgcagtaatacatgctactgtaaaaaatgttcctaccat
tgccagctttgctttcttaaaaagggcctggggatatgtatggcacatgttaataattacctacttgttacactc
ctgcttataagtatctatgggtatatgggcaagaactttgtcactgtcttctatggtatacccgcatggaaaaat
gcatcaattcccctcttttgtgctaccagaaacagagatacctggggaactgtacaatgcctcccagataatgat
gactataccgaaatccaattaaatataacagaggcttttgatgcatgggataatacagtgacagatcaggcaaca
aaggatgtgtggagtctctttgagacctcaataaaaccatgtgttaaattaaccccactgtgtgtgacaatgaag
tgtaataagacgtggagctcagctagcaaagagaccactacgtcctccgcgtccctcagatcttctactcagacc
ctactcaacgaagatagcaaatgtattcaaaatgacagctgtgcagggataggactagaagaaatgatagactgt
caattcaaaatgacaggattaaaaagagatgagtcaaaacaatataaggacacctggtataaacaagatttagtg

tgtgaaaaggggacaaggagtaatgaaagcaaatgttatataaaaacctgtaatacatcaattatccaagaatca
tgtgacaaacattattgggatagtttaagatttaggtattgtgctcccccaggatttgctttgctaagatgcaat
gatactaaatattcaggcttcatgcccaactgtagtaaggtagtagtatctctgtacagaatgatggaaacacag
acctctacatggtttggcttcaatggtacaagggcagaaaacaggacatatatctattggcatggtaaagacaat
aggactatcataagcttaaattcatattataatctgacaatgcactgtaaaaggccaggaaacaaaatggttgtg
ccaataagaaccgtgtcaggtattctcttccattcacagcctatcaataaaagacctaaacaagcttggtgctgg
tttaaaggaaactggacagaagccatacaggaggtgaaagagaccattaaaaatcatcccagatattcaggaaca
acaaatatctcacagataaggttagcagagcacgcgagaagctcagatccagaagtaagatatatgtggactaac
tgtaggggagagtttctctactgtaatatgactttttttcttaaactgggtagaaaatagaactgggctcaagaga
aattatgcgtcatgccacatcagacagatagtcaacacgtggcacaagattggaagaaatgtgtatttgcctcca
agagagggtgaactctcctgtaattccactgttaccagcctcatagccaacattgactggatagataagaacctt
actaatattactgtgagtgcagaagtgtcagaactgtataaattggaactgggggactacaaattagtagagata
acaccaattggctttgcacctacaagtataaaaagatattcctcagtgacaccgaggaataaaaattcatgggga
tgtgctttcagacaggtctgtcacacgactgtaccatgggtaaatgaatcccttaagccagactggaataacatg
acatggcaacaatgggagaggcaagtccgcttcttggatgcaaatataacaaaattactagaagaggcacagata
caacaagaaaagaacatgtatgagttacagaaattaaatcaatgggatattttcagtaattggtttgacttcacc
tcctggatggcatacatcaggttaggattatatatagtaataggaatagtagtattaagaatagcaatatacatt
atacagatgctagcaaggcttaggaagggctataggccagtattctcctcccctccctcttatactggacaaaag
actttgggagcagaagggggaggaaaacaagattcagatgaggatgatgaggacaatgaagtaggggtccgtgta
agacccggggtcccactgaggccaatgacattcaaactagcagtagacatgtctcatttttttaaaagaaaagggg
gaactggaagggattttctatagtgagagaaggcataaaatactagacacatacttagaaaatgaagaaggcatt
gtgtctggatggcagaactacacacatgggccaggagtaagatatcccaagttctttggctggctatggaagcta
gtgccaataaatatgatagcagaaccagaggacgaggaaacccattgtctggtgcatccagcacagacctccgca
tgggatgacccccacgaagagacccttgtctggcagtttgactccctcctagcatatgactatgtggccttcagc
aggttcccagaggagtttgggtatcagtcaggaatgccagagaaagagtggaaggctaaactgagagcaagagga
atacctacagagtag
>#42 KP004991 7530 nt
atgggtgcgagagcgtctgtgttgtcagggagtaaattggatgagtgggaaaaaatccggttaaggccaggatct
aaaaagaaatatctgctaaaacatttagtatgggcaagcagggagctggaaagatttgcttgtaatcctgagcta
ttagaaacagcagaagggactgagaagctactgcagcagctagaaccagctctcaagacagggtcagaaagcctg
cagtcactctggaacacgatagtagtactctggtgtgttcacaacagattcaaagtggctgatacacagcaggca
atacaaaagttgaaggaagtgaaggggcataaaaacacaagtgcgcagcagacagatcaaaggagctcgcagcag
acaggacaaaattaccctatagtaccaaatgcacagggacaaatgacgcatcagcccctctcccccagaacttta
aatgcatgggtaaaggcagtagaagaaaaggcctttaaccctgagattattcctatgtttatggcattatcagaa
ggagctatcccatatgacatcaatactatgctaaatgccataggaggacatcaggggggccctacaagtgctgaag
gargtcatcaatgaagaagcagcagattgggacagaactcacccgccagtggtgggaccattgccaccagggcaa
ataagggaaccagcaggaagtgacattgctgggacaactagcacccagcaagagcaggtccactggactactagg
gctaacaatcctatcccagtaggagatatctacagaaaatggatagtaatggggttaaacaagatggtaaaaatg
tacagtccagtgagcatcttagacataaagcagggaccaaaagaaccatttagagattatgtagacaggttctay
aaaactttaagagctgaacaagcaacccaagaagtaaaaaattggatgacagagacactgcttgttcagaatgcc
aatccagattgcaaacagattctaaagtcattagggccaggggctaccttggaggaaatgatgatagcctgtcaa
ggggtaggaggaccaactcataaggctagagtactagcagaagcaatggcctctgtccaccaagatctaaaagga
ggatacacagcagtattcatgcaaagagggcaaaacccaaataagagagggcctataaaatgttttaactgtgga
aaagaagggcatttagcaaaaaattgtcgagcacctagaagaaagggttgctggaaatgtggacaggagggtcat
caaatgaaagattgcaaaaatggaaggcaggcaacagcgaaaatcgggggccatctatgtgaagttttgctggat
acagggggcagatgatacagtgctacatgacatacaattagaaggaaaatggacaccaaaaatgatagggggtata
gggggttttataaaggtgaaagaatttaacaatgtgacagtacaaatagaaggcagggaagttcagggaacagta
ttggtgggacctactcctgttaatattgttggaagaaacatattgacagggttgggttgcacattaaatttcccc
ataagccccatagccccagtgccagtaaaactaaaaccaggaatggatggaccaaaagtaaaacaatggccccta
tctagagaaaaaatagaagcattaacagcaatatgtcaggaaatggaacaagaaggaaagatatcaagaataggg
cctgaaaatccttataatacacccatatttgctataaaaaagaaagatagcactaagtggagaaaattagtagat
ttcagagagttaaataaaagaacacaagatttctgggaggtacagttaggtatcccacatcctgggggtttaaag
aaaaagcaatctgtcacagttttagatgtaggagacgcctatttctcatgtccattagacccagatttcagaaaa
tatacagcttttactattcctagtgtaaacaatgagacaccaggagtgagataccaatacaatgtcctcccgcaa
ggatggaaaggctctccagctatatttcaaagttcaatgactaagattctagatccattcagraaagacaatcca

gaaatagaaatttatcagtacatggatgacctctacgtagggtcagatttaccactagcagaacatagaaggaaa
gttgaggtgcttagggagcatctataccagtggggatttactaccccagayaaaaarcaccaaaaggagcctccc
tttttgtggatgggctatgagctccacccagataaatggacagtacagcccatcaaattgcctgacaaagaagta
tggacagtaaatgatatacaaargctggtaggaaaattaaattgggcaagtcaaatctatcaaggaatcagagta
aargaactatgtaagttgattagaggaaccaagtcattracagaagtagtacctctaagcaaagargcggaacta
gaactagargaaaatagggaaaggctaaaggaaccagtgcatggggtatattaccaacctgacaaagacttatgg
gttaatattcaaaaacarggagaagggcaatggacgtaccaggtctatcaagatgagcataaaaaccttaaaaca
ggaaaatatgctaggcaaaaagtctcacatacaaatgatataagacaactagcagaagtaattcagaaagtgtct
caagaatctatagttatttggggaaagttgcctaagtttaagctgccagttactagagaaacttgggaagcatgg
tgggcagactattggcaagccacctggattcctgaatgggaatttgttagtacacctccattgatcaaattatgg
tatcaattagaaaagtgagcctatttcaggggcagagacatactatgtagatggggcagccaatagggacacaaaa
ttaggaaaagcagggtatgttacagaacaaggaaaacagaagataatcaaattagaggagactactaatcagaaa
gctgaattaatggcagtcttagtagcactgcaagactccaaaaaagaagtaaatatagtaacagactcgcagtat
gtattaggtgttatctcagcccaacctacacaaagtgattccccccctagttcagcagataatagaggaattgaca
aaaaaggaaagagtataccttgcctgggttcctgcccataagggcatagggggaaatgaaaaaatagacaaacta
gtaagtaaagatattagaagagttctattcctagaagggatagaccaggcacaagaggatcatgaaaaatatcat
agcaattggagggcgttggccagtgactttggactgccaccagtggtagccaaagaaatcattgctagctgccct
aaatgtcatgtgaaaggggaagctatgcatggtcaagtagattgcagtccagggatatggcaactggactgcacg
catttagaaggaaaaattatattagtggcagttcatgtggccagtggcttcatagaagcagaagtaatccctgca
gaaacaggacaggaaactgcttacttcctgcttaaattagcagcaaggtggccagtcaaagtaatacacaccgat
aatggaccaaactttaccagtgctgcagtaaaggctgcttgttggtggcttaatataactcatgaatttggaatt
ccatataatccacaaagtcaaggagtagtagaatctatgaataaagagttaaagaaaataatacagcaagttaga
gatcargcagagcagttgaaaacagcagttcaaatggcagtatttgtccacaattttaaaagaaaaggggggatt
ggggggtacactgcaggagataggataatagacataatagcttcacaaatacaaacaacagaattacaaaaacaa
attttaaaaattcaaaattttcgggtctattacagagacagcagagaccctatttggaaaggaccggcacagctc
ctgtggaaaggtgagggagcagtagtcatacaagacaagggagacattaaggtagtaccaagaagaaaggcaaaa
ataatcagggattataggtccaaaaaggccaaagattggttttacagacaccattatgaagccacaaatccaaga
gtcagttcaggtgtatatattccagtaggtggtgcttgcataatagtgaccacatattggggtttgatgccargg
gaagmagctgarcaattgggaaatggggttagtatagaatggcagctaaggaattatagaacacagattgatcct
gaaacagcagacaggatgatacacctgtattatytttcmtgttttacagagtcagcaatcaggaaagccatctta
gggcaaaaggtgttacccacttgtgaataccctgcaggacatagtcaggtagggacccttcaattcctagctttg
agggtattagtaaaaacaagaaaatttagacctcccttacccagtgtccagaagttagcagaagatgcagaagca
ttaaaacatttcctagaccttggctacacgccttgggacaacacatttatgaaacttatggagacacttgggag
ggagtaacagccattataaggatcttacaacaattaatatttacccattatagaattggctgccatcatagtaga
ataggaattatcccatctaacgcaagaggaagaagaagaagagtgccccccttggcatcatcctgggagtcaaccc
caaaccccttgtaataactgctyttgcaaaaawtgcgcctatcattgctatctttgtttcacaaagaagggtttg
gggatctccccactgtaygcaacagtctattatggggtccctgtatggagagatgcacaaactacattattctgt
gcagcagatgcaaaaatggcaagctcagagatgcataatgtatgggckacacaagcatgtgttcccacagaccca
carccaatagaactgaaactaaccaatgtgacagagacctttaacatytggaaaagtggaatggtagagcaaatg
caggaggatattattagcttatgggaccaaagtttaaagccmtgtgtcaagttaacagtcatgtgtgtgaccatg
aattgcakccgawttaccccctaayacgacaacccatagccttaacatgacaggtccagcccctaggacaagtamt
ccaaccactagcagccataacatgacaagtactccaaacattccagagctagaagtttacaactgtacttttaat
gtcaccacagtattgaaagataaaaaaagtcaacaacaggcgttgttttacagagaagacttatctcagataggt
gatrasaacagcacctataggctgattaactgtaatacttctaccatctctcaagcttgtcccaaagtgtccttt
gagccattgccaatacagtattgtgcaccggcaggctatgctttaatgaaatgtaaccaaagcaagtttaatggg
acaggacatgtaatgagactttaataactcactgtacacatgggattagaccgacagtgtcaacacaattcatc
ttcaatgggacgttagaaaaggaactcctgatgctaagtaaaaacatttcagacagtggaaaaacaattatagtt
aagttaaaaaaggcagtaacattcaagtgtgaaaggacagggaacaacacgcgagggcaaattcagatagggccc
atgacaatttacaattcagaaaacattgtgggcaaaaccaggaaagcctattgtgcatataatagaacagaatgg
gaacaggctctgaaaaccatcagtgaagcctttgcaaagttagagaatgtgacaaaggtacagtggaagaacagt
tcaggaggtgatctagaggtagcaatgctgcattttaattgccatggtgagttcttttattgcaacaccacgaca
atgtttaattatacttacaattgtagccaccatagctgtaaagctcaatcaaagaacatttcagagggcagcggg
gactcgtatattccctgtaaattgaaacaagtagtaaattcatggatgagagtaggatcaggtctttttgcccca
cctatacggggaactctcaaatgtatgtcaaatataacaggtttactactagaaagagatgtcccattkaacata
ackgccactracagaaatataactctcagaccaacgggggagaaatgaaagatatatggagaagtgaactatac

ccatacaaggtggtgcaagtcaaagcattatctgtggcacccacaaagataaaaagacccataattggtctgaac
agagaaaaaagaggactgtggggatgtgcagggaaattgrtttgttatactaatgtgccatggaataatacttgg
actaataaaagtratacagaattagagcacatytgggagaacctaacatggcaggaatgggacaaactrgttgac
aattacactgawgacatcttcctcaagatacagsaggcgaacacacaacaagaggttaatgaaaagaagttacta
gagttagacaaatgggcagacttgtggagttggtttgacataactaaatggctgtggtacataaaaatagctata
atgatagtagragctttaataggactaagagttgtcatggtagtgcttagtttagtaaagaacattaggaaggga
tatcaacctctctcgttacaggaagcttgcaaaacacttaaggctacagcacaatattggctacaagaattgcaa
agaagcrctactaacttactagatactgttgcagtggcagttgccaattggactgacagcattatcttaggtgtg
cagagattcggaaggggaattcttaacatcccaagaagaataagacaagggttagaactcagtttattgtaaatg
gggaatgcctggaagaaaagcagctttgtgggatggccagcagtaagggatagaatgaggcaaacttcccctgac
cctgagccatgtgcccctggggtaggaatagtttctagagaattagcacagaaaggaggcataccaagctcatac
acacctcagaacaatgcagcccttgcgttcctagacagtcatacagaggaagaagtaggtttcccagttacacct
caagtgcctctaagaccaatgacctttaaaggagcgtttgacctcagcttctttttaaaagaaaagggaggactg
gatgggctaatttactccccacagagagcagagattctggatctttgggtcyatcacactcagggattcttccct
gattggcagaactacacaccagggccaggaaytagatacccactgacctatgggtggttgtttaagttagtacca
gtatcagaagctgaggcagaagaaatgggcaataagaatgagagggctaaactcctacacccagcttgyaatcat
ggctatggggatgagcatggacaaatactaaagtggcagtttgatagatcactaggcaacaaccatgttgctctg
gcccttcacccagagtactttaagaactaa
>#43 FJ424866 7494 nt
atgggtgcgagagcgtcagtgttgacaggggacgattggatgcatgggaacgaattaggcttaagccaggaagt
aagaaaaagtatatgctaaaacatgtagtatgggcaagcagggagctggaaagatttgcatgtaatcctgagctt
atggaaacagccgagggctgtgaaaaattattagggcaactagaatcagctctcaaaacagggtcagatggcttg
cgatctctgtggaacactttggtagtgctgtggtgtgtccaccggagaattgatgtatcagacactcagcaagcc
ttagaaaaatttaaggaagtcatgtcaaaaagaaagtcagacagccgccctgaaacaagtcagaactatccagtg
gtagcaaatgctcaagggcagatggtccatcagccgctgtctcccaggactctaaatgcctgggtgaaagcggta
gaagaaaaggccttcaatccagaaatcatccctatgttcatggcattatcagaagggtctatcccttatgatatt
aatactatgctgaatgccattggaggacatcaggggcattacaggtattaaaggaagtcatcaatgaggaagca
gcagaatgggacaggatgcacccggcagtggtgggacctctgccagtgggacaactgagggagccgtcaggggga
gatattgcagggacaacaagtactcaacaagaacaagtagcttggataacaaggaataacaaccctgtgccagtg
ggtgacatatataggaaatggatagtgctagggttaaacaaagtagtaaaaatgtactgtccagtcagtatccta
gatattaagcagggacctaaggaaccattccgagactatgtagacagattctacaaaccctcagagcagaacag
gcaagtcaagatgtaaaaaattggatgacagaaactttgttaattcagaatgctaatcctgattgtaagcaaatt
ctaaaagccctaggcccaggggcaaccctagaagaaatgatgaatgcttgtcagggagtgggggacctactcac
aaagcaagagtcttggcagaggcaatggcagcagcccactcagatttaaaaggaggatatacagcagtctttatg
caaaaggggggaaacaatcaaggattaagaagggaccagtaaagtgcttcaattgtgggaaagtaggacatata
gcaaaaaattgtagggcacccagaagaaaggatgctggaaatgtgggcaagatgggcaccaaatgaaagactgc
agatcaggcagacaggcgcctgtgaagataggggacatctatgtgaggctttgttagacacaggggctgatgat
acagtagtagaacacttatctttagaaggtaggtggaaaccaagaatgataggaggcataggaggatttattaaa
gtaaaagaatatgaagatgtagaaatagaaatagaacataggaaagtagtagggacagttctagttggaccaacc
cctgtaaatattataggaaggaatattttaacaaaaattggatgtaccctaaattttcccataagctccatagat
gtagtacccgtatctttaaagccaggatggatggacccaaagtcaagcaatggcctttgtcaaaagaaaaaata
gaagccttaacagctatttgtcaggaaatggaacaggaaggcaagatatcaaaaattgggcctgaaaatccatat
aatacacctatttttgccataaaaaagaaagacagtactaaatggagaaaattggtagattttagagaattaaat
aaacgaacacaggattttgggaagttcagttagggattcctcacccaggagggcttaaaaagaaaaaatcagta
acagtactggatgttggggacgcttatttttcttgtcccttagacccagattttagaaaatatacagctttcaca
attcctagtgtgaataatgagacaccaggggtaaggtatcagtacaatgtactaccccagggatggaaaggatct
ccagcaattttccagcactccatgacaaaaatacttgatcctttcagaaagaaaaacccagagatagaaatctat
cagtacatggatgacttgtatgtagggtcagatttacctataacagaacataggcagaaggtagaaaaactcagg
gaacatttatatgcatggggattcaccactcctgacaaaaagcatcaaaaggaacctcccttttttgtggatgggg
tatgagctgcaccctgataagtggacagtgcagcctataaagttaccagaaaaggaaagttggacagtcaatgat
atccagagattggtaggtaaactaaattgggctagtcaaatatatccagggataagagtaaaagaactctgcaaa
ttaattaggggtactaaatcacttacagaagtagtgacccttagtaaagaagcagaattagaactagaagagaac
agggaaatattaaaagaacccgtacatggagtgtattaccagcctgaaaaggaattaatagtgaatgtgcaaaaa
caaggagaaggacagtggacttatcagatcttccaggatgaatacaagaatctaaagacaggaaagtatgctaga
caaaaggccactcatactaatgacattagacagttggcagaagtgatacaaaaggtctcacaagaaagcatagtt

atctggggaaaactgcctaagttcaggctacccatatgtcggagtacctgggacgctttttggactgactattgg
caggcttcctggatccctgagtgggaatttattagtacacctcccctcatcaagctctggtatcaattggaaaag
gaacccataccaggagcagaaacctttatgtagatggagcagctaatagagaaagtaaacaaggaaaagcagga
tatgtaacagataagggtagacagaaaataagaaaactagagggcacaactaatcagaaagcagaactagaggca
gtcctcttggccttagaagaatcaggaaaaaatgttaacatagtgacagactcccagtatgtactagggattatt
gcagcaagtcctgatcaaagtgactccccttagtgcagaaaataatagaggaaatgacaacgaaggaaaaagtc
tatttagcttgggtaccagctcacaaaggtataggggaaatgaagaaatagacaaattagtcagtaaagatatc
agaagagtcttattcctagaaggcatagatcaagcgcaggaagatcatgaaaaatatcacagtaattggagaaca
ctagccagtgactttggtctgccgccagtggtagccaaagaaatcatcaatagctgccctaagtgccatgtaaaa
ggggaagctaggcatggacaagtagattgcagcccaggtatctggcagttggattgcactcacttagaagggaaa
atcatttagtagcagtccatgtggccagcggcttcatagaagctgaagttattccagcagaaacagggcaggaa
acagcctacttcctgctcaagttagcagctaggtggccagtaaaaataatacacacagacaatggcctaatttt
actagtgccacagtcaaggctgcctgttggtggctcaatgtaacacatgagtttggaattccctataatccccaa
agtcaaggagtagtggaatctatgaacaaagaattaaaaagattatacaacaagtgagagatcaagcagaacat
ttaaagacagcagtgcagatggcagtatttgtccacaattttaaaagaaaaggggggattggggggatacactgca
ggagacaggataatagacatgttagccacacaaatacaaacaacagaattacaaaaacaaattttaaaaattcaa
aattttcgggtctattacagagacagcagagaccctatttggaaaggaccagcgacgctcctgtggaaaggtgaa
ggggctgtagtcattcaggacaaaggggacattaaagtagtccctaggagaaaggcaaaaataattagggattac
cataaatatagaactaaacaagcaaaacagtggttctataggcatcactatgaagcagtcaatcctagggtaagt
tcaggggtatacataccagtaggccccgctgaaattatagtaaccacatattggggactcatgccaggagaaaag
gaagaacaattaggccatggggtaggcatagaatggagacagggaaaatacataacacaattagatccagaaaca
gcagataggataatccatctttattactttcaatgttttacagaatctgcagtgaggaaagcaattgtgggggaa
aaactcctgtacaagtgtaactacccggcaggacatagtcaggtagggacattgcaatacttagcattaagagct
ttagtagggaaagtcagggtaagaccaccttttccaagtgttcagaaattagcagcagatatgttagaagaactt
aagcaagaagcagtaagacactttcctaggccctggttgcaccaattgggacagcatgtttatgatacttatgga
gacacttgggaaggagtgacagcaataatcagaatcttacagcaagtaatctttgttcattatagaattgggtgt
catcatagcagaatagggattttgccaccctctagaagaggaagaagactgccaccatggcagcaaccagggagc
cagcccccaaccccatgcaacacatgctattgtaaaaagtgctgttatcactgctatgtttgtttcacaaagaag
ggtttgggaatctccgctacgcattatgctactgtgtattatggagtgccagtctggcgagaggcaaatgtaact
ttattctgtgcagcagatgctaattatgtgagcaaagaacaacacaatatttgggcaacgcaggcatgtgtgcct
accgacccaaggccaacagagtttccattggaaaatgtaacagaaacctttaatatctggaaaaattacatggta
gatcaaatgcaggatgatatagttagcttatgggatcagagcttaaagccttgtgtaaaactaacagttatgtgt
gtcactctgaattgtacacaactaaatacaacaaatacaacacagactgagatgacaaatacaactataccacca
catacaactataccaccacacatcccacagttagacatttacaactgtagctttaatgttaccacggttctaaaa
gataagacaacaaagcagcaagctttattttatagacaagatataatagaaacaggtgaaacaggtacaaatggg
atcaaagggtatagattaatcaattgtaatacctctactatatcgcaagcttgtcccaaagtctcctttgagcca
ctacctatacagtattgtgcaccagcaggctatgccctgatgaaatgtaatgatatacaatttaatggtacagga
gaatgcagaaacgtgtcaatagttcattgtacccatgggatcaggccaacagtctccacacagttgatactcaat
ggaactttagcagaagggaatgccacaataatcagcaaaaatagtagtgatagtggaaaagatattatagtcaaa
ctagccagaccagtagaaattacctgtgaaaggacaggtaataatacaagaggacagatccaaatagggcccatg
acaatttataactcagaaaacatagtaggaaacacaagaaaggcttttttgcaagtataatgagacaaactggcag
ggtgcgttgaaagacacagtgcaggctctcaacgagagcctgatccacttccggaattcttcaggaggagatcca
gaagttacctttctgcattttaattgtcatggagaatttttctactgtaataccactaaaatgtttagctacaat
tgcacaacagagggttgcaataccactgaaacaagcaataccactgaaacaactaaatatattccctgccatcta
aaacaggtagtcaggtcctggatgagagtaggctcaggcctatttgctccgcctattaggggaactctaagatgt
atatctaacattacaggaataatattacaaagggatgcacccctaaaaactgatgagaacagcaccttaagacca
ataggaggtgacatgacaaacatttggaggagtgaactgtaccccctacaaggtggtgagggtcaaggcattgact
gtggcaccaacaaaagcaaagaggcctgttattggccataacagggaaaaacgtggactgtggggatgcacaggg
agattaatttgttacactaatgtgccatggaacaagacctggaccgggaaaaatgacactgaactagatgacatt
tggggaaacatgacttggcaacaatgggacaaattggtagacaactacactgacacaatattttttagaaatacaa
agggcacaggaacagcaggaggctaatgaaaaggccctactagagttggataaatgggcagatctttggagttgg
cttgacataacacaatggctatggtatataaaaatattcataataataatagcagggctagtaggactaagaatt
ctaatggctataataaatatgtgtcacagggttaggcagggttactcacctctgtcatttcagttgacaagagac
tgctttgcttttattgcttactggggacaagaacttaagcaaagtgctattagcttgctagactgtgttgctgta
tggaccgctaattggactgatcaagtaatagcaatagctcaaagaataggtaggggcatcttaaacatacccaga

aggattagacaaggattagaaagaagcctattataaatgggaaacacatggaaaaagagtagcttcataggatgg
ccagcagtaagggaaagaatgagacaaaccaaccctgagccaaagccggctccaggggtgggtgaagtgtctaga
gcattagcacaaagaggagggatacctgcaaaatatactccacaaaacaatcaagctttagcattcctagaagct
catgaagatgaagaggtaggatttccagtcagacctcaagtgccgttaagaccaatgacctttaaaggggcattt
gacctcagcttctttttaaatgaaaaggggggactggaagggttagttttctctcaggccagaagtgatatctta
gacctctgggtttatcatactcagggatacttccctgactggcagaattacacaccagggccagggataagatat
cccctgacctttggatggctgtttaagttagtgccagtctcagaggcagaagcggaggagatgggaacatcaaat
gagaaggccatgctgctgcatccagcgtgcagccatgggagagacgacccatatggagagatccttgtctggaga
ttcgatagagcattgggaacaactcatgttgccttacagaagcacccagaactgttcaagagagactaa
>#44 KP004990 7539 nt
atgggtgcgagagcgtcagtgttaacaggggtcgattggatgcatgggaaagaattaggctcaagccaggaggt
aagaaaacctatatgctaaaacatgtagtatgggcaagcagagagctggaaagattcgcttgtaatcctgagctt
atggagacagcagaaggttgtgagaaattactaggacagcttgagccagctctcaaaacaggttcagaaggcctg
cagtcgctttggaacaccctggtagttctatggtgtgttcacaagcgaatagacgtagccgacacccagcaggcc
attacaaaatggaaggaggaaatgcaaaaacgaagaaaagagaaagaaagcagccccggaacaagtcaaaactat
cctatagtgcagaatgcgcaggggcaaatgatacatcagccactgtctcccaggactttaaatgcttgggtgaag
gcagtagaagagaaagccttcaaccctgagattatccctatgtttatggccttatcagaaggatctataccttat
gacattaatactatgcttaatgcaataggcgggcatcagggagcattacaggtgttgaaagatgtgattaatgag
gaagctgcagaatgggacaggttacaccctgcccccgtagggccgttaccgccagggcagataagggaacctaca
ggaggggacattgcaggaaccaccagtactcagcaagagcagatagcatggataacaaaaaatcctcctatacca
gtaggagacatctatagaaaatggatagtgttgggactcaataaagtggtaaaaatgtatagccctattagcatc
ctggacataaagcagggacctaaggagccattcagagattatgtagataggttctataaaaccctcagagcagaa
caagccagtcaggatgtaaaaaattggatgacagataccttattagtacaaaatgctaatcccgattgcaagcag
attctgaaagccctaggaccaggggccaccttggaggaaatgatgaatgcttgtcaaggagtaggggaccaaca
cataaggccagggtcttggcagaagctatggcagcagccaaccaagcyagccaagacttgaaaggggatacaca
gcagtctttatgcaaagaggacagagagggccagttaagtgctttaactgtggaaagctagggcatatagcaaag
aactgtagagcaccgaggagaaaaggatgctggaaatgtggacaagagggtcatcagatgaaagactgcaaaata
ggaaaacaggcacctgtaaaaataggggacatatttgtgaagctttattagatacaggggctgatgacacagta
gtagataaaataccattagaaggaaggtgggtaccaaaaatgataggaggaataggaggatttattaagtaaaa
gagtttgaaaatgttaaaatagaaatagaaggtagagaagttcatgggacagtattagttggcccgaccccagtt
aatataataggaaggaacattttgacatccattggctgtactttaaattttcctatcagccccatagaagtagta
ccagtaaaattgaaaccaggcatggacggcctaaggtgaagcaatggcctttatcaaaagagaaaatagaagca
ttaactgctatctgccaagatatggaacaggaaggaaaaataacaagaattgggcctgaaaatccctataacacc
cctatttttgcaataaaaaagaaagatagcagtaaatggagaaagctagtagattttagagagttaaataaaaga
actcaagattttgggaagtccagctgggaatccctcacccaggaggtcttaagcagaagaagtcagtaacagta
ctagatgtgggagatgcttatttctcctgtcccttagatccagatttcaggaagtacacagcttttactatacct
agtgtaaataatgaaacaccaggggtcagataccagtacaatgtgttacctcaagggtggaaggatcaccagcc
atcttccaacattccatgaccaagatcctagatccattcaggaaaaataatccagaaatagaaatttatcagtac
atggatgatttatatgtagggtcagacttgccactatctgaacataggcaacgagtcgaaaagcttagagaacat
ctctatgcttggggatttacaactcctgataaaaaacatcagaaggaacccctttcctttggatggggtatgaa
ctccaccctgacaagtggacagtgcaacccattaagttgccagaaaaagaaagctggacagtgaatgatatccag
ggactagtagggaaactaaattgggcaagtcaaatttatccaggaataaggataaaagagttatgcaaactcata
aggggaactaaagctttaacagaagtagttaccttcactagagaagcagagttagaactagaagaaaataaagaa
atcttaaaagaaccaaggcatggagtttattaccaaccagaaaaagaattaatagtagatatacagaaacaagga
gagggacaatggacttatcagatatttcaggaagaacataagaatttgaagacagggaaatatgccaggcaaaag
gctactcacaccaatgacataaggcaattggcagaagtaatacagaaggtatcacaagagagcatagtaatatgg
gggaagctgcccaaattcaggttaccagtaaatagaaccttgtgggaagcctggtggtcagattattggcaagcc
acctggataccagagtgggagtatgttagcacacccctcttattaaactctggtaccagctagaaaaggacccc
ataccagagacagaaacttttatgtagatggggcagcaaatagagaaaccaagctaggtaaggctggctatgta
acagataggggaagacaaaaaatagtcaaactagaaggaaccaccaatcaaaaagcagaactagaagcagtgcta
ttagccttaaaagaatcaggaaagcaggctaatatagtaacagactctcagtatgtgttagggattattgcagca
actccagatcagagtgactccccccctagtacaaaagataatagaagaaatgacaaaaaaagaaaaggtgtaccta
tcatgggtaccagcacacaaaggcataggggtaatgaagaaatagacaaattagtcagtaaagacatcaggaga
gtcttatttttagaaggcatagatcaggcgcaggaagatcatgagaaatatcatagtaactggaggaccttagcc
agtgactttgggcttccaccaatagtggcaaaagagattattaacagttgccctaaatgccatgtaaagggagaa

gccatgcatgggcaggtggactgcagtccaggaatatggcaattggactgtacccacacagaaggaaaaatcatc
cttgtagcagtccatgtggccagtggcttcctggaagctgaagtaattccagcagaaacaggacaggaaactgca
tacttcctgcttaagctagcagcaagatggcctgtaaaagccatccacacagacaatgggcctaacttcgctagt
gctgcagttaaagcagcttgttggtggctcaacataactcatgaatttgggataccctataatccccaaagtcaa
ggagtagtagaatccatgaacaaggaattaaagaaaattatactgcaagttagggaccaagctgaacatttaaaa
acggcagttcaaatggcagtatttgtccacaattttaaaagaaaaggggggattggggggtacactgcaggagat
aggattatagatataatggctacacaaatacaaacaacagaactacaaaaacaaattttaaaaattcaaaatttt
cgggtctattacagagacagcagagaccctatttggaaaggaccagcgacgctcctgtggaaaggtgaaggggca
gtagtcatacaagataagggagacattaaagtagtccctagaaggaaagcaaaaatcattagggattacaggtct
aaaagagcaaagaattggttttatagacatcattatgaggcagttaatcctagagttagttcaggggtctatatt
ccagtagggacagcaatgattattgtaaccacctactgggggctcatgcctggtgaaaaagaagatcagttgggg
catggagcaagtgtagtatggagtcaaggcaaatacagcacacaaatagatccagagacagcagatagactaatt
cacctccactacttccaatgtttttcagattcggctgtgaggaaggcaatactaggggatagattactgaccagg
tgtgaatattcagcaggacatagtcaggtaggctccctacagtacttagccttaagagtgttagtaggaaaagta
agaaggaagccacccctccctagtgttcagttattgacagaggacaccttggaggaactcaaagcagaggctgta
agacactttcctaggccttggttacactccctaggacaacatatatatgatacctatggggacacctgggaagga
gtaactgcaattattagaatcctacaacaattaattttatccactatagaattggatgccctcatagcagaata
gggatagcgccacaccctcaaaggggagaagattgcctccatggcaacagccagggagtcagccctcaagcccg
tgtaacaattgtttctgtaaagcttgctgctatcactgctatgtttgtctcgtaaagaagggtttgggaatctcc
actggaagctatgttatgggtacacgttatgtcactgtatattatggagtgccagtgtggagggaagccaaaact
acattattctgtgcagcagatgcaaacctagctagcaaagagcagcataatatttgggccactcaagcctgtgtg
cctacagaccctactccaataaaggtcaagctaaatatatcagaaccctataacatttgggaaaattatatagta
aagcaaatgcaaaaaaacatcattagcttattaaatcagagcttaaaaccttgtgttaagctaacagtcatatgt
gttacaataaattgtagtaagtttaaaccagctactaccaatacaactagtgcaccaattaacgctagtctacaa
agtagcactactgcacaaaataacctaaacttttataactgttcttttaatgtaactacagtgctaaaagataaa
aaagaaaagaagcaagctctcttctataaagaaaaccttgtaccacttaatcaaacatctaaagaaaaactatat
aggctaattaattgtaacactactacaattacgcaagcctgtccaaaagtgtcttttaaaccattaccaatacag
tattgtgcaccagcaaaatatgcattaataaaatgtaaacaaaaaggttttaatrgtacarractttgcaatraa
acagtaataacacattgcacacatggaattagaccaactgtgtccacacagttaatatttaatggaagtttagca
gaagaagaggctctagtgatgtcaaaagatgttacggccactgggaaaaacatcataataaaattaagtacagga
gtaaacataacgtgtatcagaacaggtaataatacaagaggacaaacacaaataggacccatgacctggtataat
agtgtgaattatataggtaatatcaggagggcttattgccaggtggaggaaacagactggaagggaattttgaaa
aatgttagcaaagctctcataaaacgttacaatggcaaggtaaatgaaacccatacgtttgagtttaagaacgct
tctggggggagacccagaggttacacacctgcacttcaattgtcatggggagtttttttattgtaatacctctaaa
ctcttcaactatacctatgtctgtaatcagacagatcaaggtaatgctaattattcctgtcatagtactagtaca
ttagaaaatggaaccttagtaataccctgcaaattaaggcaggtagttaattcatggatgagagtgggatcagga
ctttttgcaccacctgtcccgggaagcctaacgtgccactccaatatcacaggtctaatccttcaaagggactgg
ccactaaacaataatacgactactatttttgaggcctgaaggaggagacatgaaagacatctggagaagtgagcta
tatccatataaggtagtccaagtaaaagctttggctgtggcacctacaaaaatctccagaccaacaattatggca
cagactcatcagagggaaaaaagaggcctgtggggatgtacaggacaaatagtctgctataccaatgtgccatgg
aatcaaacctggactggtaggaatgacactgaattagatagcatttggaacaagttgacatggcaggaatgggat
aaattagtagataattacactgacaccatttttgtagagatacaaaaagcaaatgaacaacagaaagaaaatgag
aaaaaactgttagagttagaccaatggacacagctgtggagctggtttgacataacaaaatggttatggtacata
aaaattttcatcatgatagtaggaggcctcatagggctaaaatttttgctagctataattaacatagttaaaaga
gtcaggcagggatactcacctttgtcattacagtgcttgagagactgcttggctgtgtgtggctattgggcacaa
gaactacaacagagtgcaactagcttattggacactgttgctgtgagagttgctgactggactgaccaagtgatc
ctagtagggcaacgaatagggagaggcatcttgaacataccaagaagacttagacaaggactagagaggagcttg
ctataaatggggaatgcatggaagaaaagtagtttagtagggtggccagcagtcagagacagaatacaccagact
accccagagatagacccagcacctggggtggggaaaatctccagagaattggcaaaagagaaagcaatacctagc
aagtacaattcaagaaataatgaggcattagctttcttagaggctcatgaagatgaggaagtagggtttccagtg
aagccccaagtaccccctaaggccaatgactttcaaaggagcatttgatctcagcttctttttaaatgaaaaggga
ggactggatgggttagtttattcacctgagagagccgagattctagatctctgggtctatcacactcagggcttc
ttccctgattggcagaattacaccccaggtccaggagtcagatggcccctgacctttgggtggatgtttaaacta
gtacccgtctctgaggcagaagctgagtacatgggaaataaggatgagagagctaagttgttacacccagcttgc

acctatgggttttcagatccacacaaggagatcctagtctggaagtatgacagatcactaggaacacagcatgtt
gccctgatgaaacacccagagctgtttatcaaagactaa
>#45 KP004989 7614 nt
atgggggcgagagcgtcagtgttaacaggggccgattggatgcatgggagagaattaggcttaagccaggaagt
aagaaaacatatatgctaaaacatgtagtatgggcaagcaaagagctggaaagatttgcatgtaatcctgagctt
atggaaacagcggatggctgtragaaattattagggcagttagaaccagctcttaaaacaggctccgatggcctg
cagtctctttggaacaccctagtagttctgtggtgtgttcacagaagacaggaagtaagtgacacccagcaggct
attgcaaaatggaaggaggaaatgcagaagaggaaaaaggcacaagagggcagcactggaacaagtcaaaattat
cctattgtgcagaatgcccaaggacaaatggtccatcagccgctgtctcccaggacrttraatgcctgggtaaag
gcggttgaagaaaaagccttcaaccctgaaatcatccctatgtttatggccttatcagaagggtctataccctat
gacatcaatactatgctcaatgcagtaggtgggcatcagggagcgttacaggtgttgaaggatgtaatcaatgag
gaagctgcagaatgggatagaacacatcccgccccagtagggcctttaccaccagggcaaattagagaaccaaca
ggagggatattgcaggaagcactagcactcagcaggaacaaatagcatggataacaagagccaatcctatacca
gtaggagacatctatagaaagtggatagtgctgggacttaataaggtggtaaaaatgtacagccccattagcatc
ttggatataaagcagggacctaaagaaccatttagagattatgtggatagattctacaaaaccctcagagcagaa
caagccagccaggatgtaaagaattggatgacagaaactttactaatrcaaaatgctaatccagattgtaagcaa
attttgaaagctctgggaccaggggccaccctggaagaaatgatgaatgcctgtcaaggagtaggggggaccaaca
cataaggccagggtcttggcagaagctatggcagcagccaatcaagccagccaagacttgaaaggagggtataca
gcagtgttcatgcaaagaggacagagagggccagttaaatgctttaactgtggaaaggtcggacatatagcaaag
aactgtaaggcacctaggagaaaagggtgctggaaatgtggacaggaaggtcaccaaatgaaagattgtaaatca
gggagacaggcwccagtaaagataggggggacatatttgtgaagctttattagacacaggggctgatgacacagta
gtagataaaatacctttagarggtagatggaagccaaaaatgataggggggaataggaggttttattaaagtaaaa
gaatttgaraatgttaaaatagagatagaagggagagaagtttctgggacagtattagttggcccaaccccagta
aatatagttggaagaaatatcctaacattagtgggttgtactctaaattttcccattagccctatagaagtagta
ccagtaaaattgaagccaggaatggacggcctagagtaaagcaatggcctctatcaaaagaaaaaatagaagct
ttaactgctatctgtcaagagatggaacaagagggaaaaataacaaaaattgggcctgaaaatccatataacacc
cctatctttgcaattaaaaagaaagatagcagcaaatggaggaaattagtagacttcagggaattaaataaaaga
acacaagacttctgggaagtccagctgggaatccctcacccaggtggtcttaaacaaaggaaatcagtaacagtg
ttggatgtgggagatgcttatttctcctgtcctttagatccagatttcggaagtatacagcttttacaatacct
agtgtgaataatgaaacaccaggagttagatatcagtacaatgtacttcctcagggctggaarggatctccagcc
attttccagcactcaatgactaaaatcctggaaccctttagaaaagagaacccagaaatagaaatttatcagtac
atggatgatttatatgtagggtcagatttgcctctgcctgaacataggcagcgrgtagagaagcttagagaacat
ctctatgcatggggrtttacaactcctgataagaaacaycaaaaggaacctccattcctctggatgggctatgag
ctccatcctgataaatggacagtacaacccatcaagctaccagaaagagaaagctggacagtaaatgatatccag
aaattagtaggcaaattaaattgggccagtcagatatatccaggaataagagtaaaagagttatgcaaactaatt
agagggactaaagccttaacagaggtgattcctcttactagagaagcagaattagagttagaagaaaataaagaa
atcttaaaggaaccagtgcatggrgtctattatcagccagaaaaagatttaatagtagatatacaaaaacaagga
gagggacaatggacttatcagatatttcaggaagaacataaaaacttgaaaacwggaaagtatgctaggcagaag
gctacccataccaatgatataaggcaattggcagaagtaatacagaaagtatcacaagaaagtatagtaatatgg
ggaaagctrcccaaatttaggttaccagtaaataggactttatgggaagcctggtggtcagactattggcaggcc
acctggatacctgagtgggaatttgttagcacacccccctctcattaagctttggtatcaattggaaaaggacccc
ataccaggaacagaaactttttatgtagatggggcagcaaacagagaaaccaagctaggtaaagctggatatgtg
acagataggggtaggcagaaaatcattaaattggaagaaacaactaatcagaaagcagaattagaagctgtacta
ttagccttaaaagaatcaggaaaacaagctaacatagtaacagactcccaatatgtattagggattatctcagca
actccagatcaaagtgactccccccctagtgcagagaataatagaagaaatgacaaaaaaggacaaggtgtacctt
tcatgggtaccagcacataaaggcataggggggtaatgaggaaatagacaaattagtcagcaaggacattagaagg
gtgttatttctagaaggcatagatcaggcacaagaagatcatgaaaaataccacagcaattggaagaccctagct
agtgactttgggcttccaccaatagtagcaaaagagatcatcaatagttgccctaaatgtcatgtaaaaggagaa
gccatgcatggacaggtagattgcagtccagggatatggcagttagattgtacccacacagaaggaaaaattatc
cttgtggcagtccatgtagccagtggcttcctggaagccgaggtaattccagcagaaacaggacaggaaactgca
tactttctgctcaagttagcagcaagatggcctgtgaaagtcatacacacagataatgggcctaacttcactagt
gctgcagtaaaagcagcatgttggtggctcaacataacacatgagtttggaataccctacaatccycaragtcaa
ggagtrgtagaatctatgaacaaagaattaaagaagattatacttcaagtacgagaccaggctgagcatttaaag
acagcagttcaaatggcagtatttgtccacaattttaaaagaaaaggggggattgggggtacactgctggagac
agaatcatagatatgatggctacacaaatacaaacaacagaactacaaaaacaaattttaaaaattcaaaatttt

cgggtctattacagagacagcagagaccctatttggaaaggaccagcgacgctcctgtggaaaggtgaaggggca
gtagtcatacaagataagggagacattaaagtagtccctagaaggaaagcaaaaataattagggattatagatct
aaagaagcaaaaaattggttttataggcaccactatgaggcaactaatcctagagttagttcaggagtttacata
ccagtaggaacagcaactattattgtaactacatattggggactcatgcctggggaaagaaaagarcaattaggg
catggggcaagtgtggagtggagacaaggtagatacatcacacagatagatccagaaacagctgataggctaatt
catctccactacttccaatgtttttcagattcggctgtgaggaaggcaatactaggggacagattattgcacagg
tgtgaatactcagcaggacatagtcaggtaggatccttacagtacctagccttaagggtgttagtagggaaagta
aaaaggaggccaccccttcctagtgtccaggtgttgacagaagatacactagaggaactcaaggcggaagcagtg
agacacttccctaggccttggttgcaattattagggcagcacatctatgatacctatggggacacctgggaggga
gtaactgcaattattaggatcctacaacaattggtctttgttcattatagaattggatgccagcatagtaggata
ggtatattgccaccctcacgaagaggaagaagactgcctccatggcagcagccagggagtcagccctcaagccca
tgtaacacttgcttctgtaaagtctgctgctatcattgctatgtttgtttcgtaaagaagggtttgggaatctcc
atgaaggatatgacagtaatgaagaagaagaagagcagcttaggaaactggggaatttgcttggctttgataata
tactttaatgctatcagttatgttaattgcacacrttatgttactgtatattatggagttccagtttggcaagat
gctaagactaccttgttttgtgcagcagatgcagatctagctagcaaagagcagcataatatttgggccacacaa
gcctgtgtgcccctagatccaacaccaatagagcttaagttgaatatcacagagtcctttaatatttgggaaaat
tatatggtagagcaaatgcargaggacatcgttagtttatgggatcagagtttaaagccttgtgttaaattgaca
tttctttgtgtcactatgaattgtagtgagtataaaggttataattgttctacagaagggaacacaacagctgca
ccatgcaatacaaccgaaaacactacaaaagagaacagcacaggaatgttgacctgtaattttaatgtgactaca
gtattaaaagataaaaaggaacagaaacaggcactcttctatagagaagacctagcaagcttagaaagcaataat
agttatagactaattaattgcaatagttctaccatcacacaagcatgtccaaaagtgtcttttgagccactacca
atacaatactgtgcaccagcagggtacgcactaatgaaatgcaacaggacagactttaatggcacaggaacttgt
aatgagacatctatagtacactgcacacatggaatcaggccaacagtttcaacccagttagtgctcaatggaact
ctagcaaaaggaaaacctttagtaattactaagaatgtctcggagactggagcgcctatcatagtgaaattgagt
gcaagtactgcgataacctgtataagacctggtaataataccaggggagaagttcaattaggacccatgacatgg
tacaacatgagacactacataggcgatatcagaaaagcacattgcactgtctctcggggaaattggacaaaggtc
ctacaaaatgtcagtgcagctctctgggaagcctatccagcagagtggaaaaataaaacacaagataaaaatcac
acaatactatttagggcaagctctggaggagacccagaagttgcttctttgcacttcaattgtcatggggaattc
ttttattgcaacacctctgcactgtttaaccatagctgtacaaagaatatgacaaagaacgaatggatatgtaca
ccaaacaatgccactggcacattaagattaccttgcaggctaaaacaggtagtgaactcatggatgagagtrggg
tcaggcttgtttgcgccacctatcccaggaagtttaacctgtaaatctaacatcacaggtatactgttggagaga
gaccttcctctgggaaatatgaccaacaccaccttaagacccataggaggggacatgaaaaatatttggagaagt
gaattgtacccctacaaggtggtccaagtaaaagctttaggggtggcacctacaaaaatctccagacccacaatt
atgggtcctcacagagaaaaaagaggcctgtggggatgttcaggaaaattagtatgttatactaatgtgccatgg
cagaaaaattggacgacttaccaaagtgacagtcaattagatgctatttgggataatttaacatggcaggaatgg
gacaaacaggtaaacaattacactgatctaatttttcttgaaatacaaatagcacaggaacagcaagaacagaat
cagaagaaattgttagaattggaccaatgggcacagttgtggagctggttggacataacacagtggttgtggtac
ataaagattttatcatgatagtaggagggcttataggactaaggattttatagctgtagttaatgtagttaag
agagtcaggcagggatactcacctttgtcattgcagtgtgtgagagactgcttggctgtgtgtggctactgggtg
caagaattacaacgcagtgcaaccaatctgttggacactactgcagtggcagttgctgaatggactgatcaagtg
atcttaatagggcaaagaatagggagaggtatactgaacatacccagragaattagacaagggctagagagaagt
ttactctaaatggggaacgcatggaagaaaagtagtctggtaggatggccagcagtcagagacaggatgcaccag
actaccccagaagaagcagcacctggggttggggaagtctccagagaattggcaaagggaaaaggaatacccagt
aaatacaatgcaaggaacaatgcagccttagctttcttagatgctcatagagaggaagaagtaggattccccgtc
agacctcaagtgcccttgagatgcatgaccttcaaaggggcatttgacctcagcttctttttaaatgaaaaggga
ggactggatgggttaatttactcacctgagagagcagaaatcctagatctctgggtctatcacactcagggattc
ttccctgactggcagaattacactccagggccaggggtcagatatcctttgacctttgggtggctgtttaagcta
gtgccagtctctgaggcagaagctgaggaaatgggaaataagaacgagagagctaagctgttacacccagcctgt
gcttatggattctcggatccacataaggagattttgatgtggaagtttgacagatcactaggaatcacacatgtt
gccctacaaaagcacccggaactgttttctaaagactga
>#46 FJ424863 7479 nt
atgggtgcgagagcgtcagtattgacaggggacgattggatgcgtgggaacgaattcggcttaagccaggaagt
aagaaaaagtatatgctaaaacatgtagtatgtgcaagcagggagctggaaaggtttgcatgtaatcctgagctt
atggaaacagcygagggctgtgaaaaattattagggcaactagaaccagctctcaaaacagggtcagatggctta
cgatctctgtggaacactttggtagtgttgtggtgtgtccaccggagaattgatgtatcagatactcagcaagcc

ttagaaaaatttaaggaagccatgtcaaaaagaaagtcagacagccgccctgagacaagtcagaactatccagtg
gtagcaaatgctcaagggcagatggtccatcagccgctgtctccaaggactctaaatgcctgggtgaaagcagta
gaagaaaaggccttcaatccagaaatcatccctatgttcatggcattatcagaagggtctatcccttatgatatt
aatactatgctgaatgccattggtggacatcagggagcattacaggtattaaaggaagtcatcaatgaggaagca
gcagaatgggacaggctgcacccggcagtagtaggacctctgccagtaggacagctgagggagccgtcagggga
gatattgcagggacaacaagtactcaacaagaacaagtagcttggataacaaggaataataaccctgtgccagtg
ggtgacatatacaggaaatggatagtgctagggttaaacaaagtagtaaaaatgtactgtccagtcagtatccta
gatattaagcagggacctaaggaaccattccgagactatgtagacagattctacaaaaccctcagagcagaacag
gcaagtcaagatgtgaaaaattggatgacagaaactttgctaattcagaatgctaatcctgattgtaagcaaatt
ctaaaagccctagggccgggggcaaccctagaagaaatgatgaatgcttgtcagggagtgggaggacctactcac
aaagcaagagtcttrgcagaggcaatggcagcagcccactcagatytaaaaggggggatatacagcagtctttatg
caaaaggggggaarcaatcaagggattaagaagggaccagtaaaatgcttcaattgtgggaaagtaggacatata
gcaaaaaattgtagggcacccagaagaaagggatgctggaaatgtgggcaagaggggcaccaaatgaaagactgc
agatcaggcagacaggcgcctgtgaagataggggggacatctatgtgaggctttgttagacacaggggctgatgat
acagtagtagaacacttatctttagaaggtaggtggaaaccaagaatgataggaggcataggaggattcattaaa
gtaaaagaatatgaagatgtagaaatagaaatagaacatagaaaagtagtagggacagttttagttggaccaacc
cctgtaaatatcataggaaggaatattttaacaaaaattggatgtaccttaaattttcccataagctccatagat
gtagtacccgtatctttaaaaccagggatggatggacccaaagtcaagcaatggcctttgtcaaaagaaaaaata
gaagccttaacagctatttgtcaggaaatggaacaggaaggcaagatatcaaaaattgggcctgaaaatccatat
aatacacctattttcgccataaaaaagaaagacagtackaaatggagaaaattggtagattttagagaattaaat
aaaaggacacaagattttgggaagttcagttagggattcctcacccaggagggcttaaaaagaaaagatcagta
acagtactggatgttggggacgcttatttttcttgtcccttagacccagatttagaaaatatacagctttcaca
attcctagtgtgaataatgagacaccaggggtaaggtatcagtacaatgtactaccccagggatggaaaggatct
ccagcaattttccagcactccatgacaaaaatacttgatcctttcagaaagaaaaacccagaaatagaaatctat
cagtacatggatgacttgtatgtagggtcagatttacccataacagaacataggcagaaagtagaaaaactcagg
gaacatttatatgcatggggattcaccacccctgacaaaaagcatcaaaaggaacctccctttttgtggatgggg
tatgagctgcaccctgataagtggacagtgcaacctataaagttaccagaaaaggaaagttggacagtcaatgat
atccagaaactggtagggaaattaaattgggccagtcaaatatatccaggaataagagtaaaagaactctgcaaa
ttaattaggggtactaaatcacttacagaagtagtgacccttagtaaagaggcagaattagaactagaagagaac
agggaaatattaaaagaacccgtgcatggagtgtattaccagcctgaaaaggaattaatagtgaatgtgcaaaaa
caaggagaaggacagtggacttatcagatcttccaggatgaatacaagaacttaaagacaggaaagtatgctagg
caaaaggccactcatactaatgacatcagacagttggcagaagtaatacaaaaggtctcacaggaaagcatagtt
atctggggaaaattgcctaagttcaggctacccatatgtcgaagtacctgggacgctttttggactgactattgg
caggcttcttggattcctgagtgggaatttgttagtacaccccccctcattaagctctggtatcaattggaaaaa
gaacccataccaggagcagaaacctttatgtcgatggagcagctaatagagaaagcaaacaaggaaaagcagga
tacgtaacagataggggtagacagaaaataaggaaactagagggcacaactaatcagaaagcagaactagaggca
gtcctcttggccttggaagaatcagggaaaaatgttaatatagtgacagattcccagtatgtactagggattatt
gcagcaagtcctgatcaaagtgactccccccttagtgcagaaaataatagaggaaatgacaacaaaagaaaaggtc
tatttagcttgggtaccagctcacaaaggtataggggggaaatgaagaaatagacaaattagtcagtaaagatatc
agaagagtcttattcctagaaggcatagatcaagcacaggaagatcatgaaaaatatcacagtaattggagagca
ctagccagtgactttggtctgccgccagtggtagccaaagaaatcatcaatagctgccctaagtgccatgtaaaa
ggggaagctaggcatggacaagtagattgcagcccaggtatctggcagttggactgcacccacttagaagggaaa
atcatttagtggcagtccatgtggctagcggcttcatagaagctgaagttattccagcagaaacagggcaggaa
acagcctacttcctgctcaagttagcagctagatggccagtaaaaataatacatacagacaacgggcctaatttt
actagtgccacagtcaaggctgcctgttggtggctcaatgtaacacatgagtttggaattccctataatccccaa
agtcaaggagtagtggaatctatgaacaaagaattaaaaaagattatacaacaagtgagagatcaagcagaacat
ttaaagacagcagtgcagatggcagtatttgtccacaattttaaaagaaaaggggggattggggggatacactgca
ggagacaggataatagacatgttagccacacaaatacaaacaacagaattacaaaaacaaattttaaaaattcaa
aattttcgggtctattacagagacagcagagaccctatttggaaaggaccagcgacgctcctgtggaaaggtgaa
ggggctgtagtcattcaggacaaaggggacattaaagtagttcctaggagaaaggcaaaaataattagggattac
cataaatatagaactaaacaagcaaaacagtggttctataggcatcactatgaagcagtcaatcctagggtaagt
tcaggggtgtacataccagtaggccccgctgaaattatagtgaccacatattggggactcatgccaggagaaaag
gaagaacaattaggccatggggtaggcatagaatggagacagggaaaatacataacacaattagatccagaaaca
gcagataggataatccatctctattactttcaatgttttacagaatctgcagtgaggaaagcaattgtgggggaa
aaactcctgtgcaagtgtaactacccagcaggacatagtcaggtagggacattgcaatacttagcactaagagct

ttagtagggaaagtcagggtaagaccaccttttccaagtgttcagaaattagcagcagatgtgttagaagarctt
aagcaagaagcagtaagacactttcctaggccctggttgcaccaattgggacagcatgtttatgatacttatgga
gacacttgggaaggagtgacagcaataatcagaatcttacagcaagtaatctttgttcattatagaattgggtgc
catcatagcagaatagggattttgccaccctctagaagaggaagaagactgccaccatggcagcagccagggagc
cagcccccaaccccatgcaacacatgctattgtaaaaagtgctgttatcactgctatgtttgtttcacaaagaag
ggtttgggaatctccgctacgcattatgctactgtgtattatggagtgccagtctggcaagaggcaaatgtaact
ttattctgtgcagcagatgctaattatgtgagccaggaacaacacaatatatgggcaacgcaggcatgtgtgcct
accgacccaagcccaatagagtttccattggaaartgtaacagagacctttgatatctggaacaataacatggta
gatcaaatgcaggatgatataattagcttatgggatcagagcttaaagccttgtgtaaaactaacagttatgtgt
gtcactttgaattgtacaaagacaacagagacgacaacrcagacaaatryaacaaatacaactataccaccatac
atcccacagttagacatttacaactgtagctttaatgttaccacggttctaaaagataagaaaacaaagcagcaa
gccttattttataagcaagatataataaaaacagatgagacgaatgagaccaaagagtatagtgagtataggtta
atcaattgtaatacctctactatatcgcaagcttgtcccaaagtctcctttgagccactacctatacagtattgt
gcaccagcrggctatgccctgatgaaatgtaatgataaacaatttaatggtacaggagaatgcaaaaatgtgtca
atagttcattgtacccatgggatcaggccaacagtctccacacagttgatactcaatggaactttagcaaaagag
catgccacaataattagcaaaaatagtactgatagtggaaaagatattatagtcaaactagccagatcagtagaa
attacctgtgaaaggacaagtaacaatacaagaggacagatccaagtagggcccatgacaatttataactcagaa
aacatagtaggaaacacaagaaaggctttctgcaagtataataagacaaactggcagaatgcattgaaagataca
gtacgggctctcaaagcgaatcagatccgcttccagaattcttcaggaggagatccagaagttacctttctgcat
tttaattgtcatggagaattttctactgtgataccactaaaatgtttaactacaattgtacaaaggagagctgt
gattgcataacagggaattgcacaaattatatcccctgccatctgaaacaggtagtcatgtcctggatgagagta
ggctcaggcctatttgctccgcctattaggggaactttaagatgtaaatctaatattacaggaataatattacaa
agggatgtaccccctgaataagactaatgagaatagtactgattacaacaccttaagaccaataggaggggacatg
acaaacatttggaggagtgagttgtacccctacaaagtggtaaaggtcaaggcattgtctgtggcaccaacaaaa
gcaaggagacctgttattagccataacagggaaaaacgtggactgtggggatgcacagggagattaatttgttac
actaatgtgccatggaacaagacctggaccgggaaaaatgaaactgaactagatgacatttggggaaacatgact
tggcaacaatgggacaaattggtagacaactacactgacacaatatttttagaaatacaaaaggcacaggaacag
caggaggttaatgaaaaggccttactagagttggataaatgggcagatctttggagttggcttgacataacacaa
tggctatggtatataaaaatattcataatgataatagcagggctagtaggactaagaattctaatggctataata
aatatgtgtcacagggttaggcagggttactcacctctgtcatttcagttgacaagagactgctttgcttttatt
gcttactggggacaagaacttaagcaaagtgctattagcttgctagactgtgttgctgtatggactgctgattgg
actgatcaagtaatagcaatagctcaaagaataggtaggggcatcttaaacatacccagaaggattagacaagga
ttagaaagaagcctattataaatgggaaacacatggaaaaagagtagcttcataggatggccagcagtaagggaa
agaatgagacaaaccagccctgagccaaagccggctccgggggtgggtgaagtgtctagagtgttagcacaaagg
ggagggatacctgcaaaatatactccacgaaacaatcaagctttagcgttcctagaagctcatgaagatgaggag
gtaggatttccagtcgyacctcaagtgcctttaaggccaatgacctttaaaggggcatttgacctcagcttcttt
ttaaaagaaaaggggggactggaagggttagttttctctcaggccagaagtgagatcttagatctctgggtttat
catactcagggatacttccctgactggcagaattatacaccagggccagggataagatatcccctgacctttgga
tggctgtttaagttagtgccagtctcagaggcagaagctgaggagatgggaacagcaagtgagagagctatactg
ctgcatccagcgtgcagccatgggagagacgacccacatggagagatccttgtctggagattcgatagagcattg
ggaacaactcatgttgcattacagaagcacccagaactgttcaagagagaataa
>#47 FJ424864 7494 nt
atgggtgcgagagcgtcagtgttgacaggggacgattggatgcatgggaacgaattaggcttaagccaggaagt
aagaaaaagtatatgctaaaacatgtagtatgggcaagcagggagctggaaagatttgcatgtaatcctgagctt
atggaaacagccgagggctgtgaaaaattattagggcaactagaatcagctctcaaaacagggtcagatggcttg
cgatctctgtggaacactttggtagtgctgtggtgtgtccaccggagaattgatgtatcagacactcagcaagcc
ttagaaaaatttaaggaagtcatgtcaaaaagaaagtcagacagccgccctgagacaagtcagaactatccagtg
gtagcaaatgctcaagggcagatggtccatcagccgctgtctcccaggactctaaatgcctgggtgaaagcggta
gaagaaaaggccttcaatccagaaatcatccctatgttcatggcattatcagaagggtctatcccttatgatatt
aatactatgctgaatgccattggaggacatcaggggcattacaggtattaaaggaagtcatcaatgaggaagca
gcagaatgggacaggatgcacccggcagtggtgggacctctgccagtgggacaactgagggagccgtcaggggga
gatattgcagggacaacaagtactcaacaagaacaagtagcttggataacaaggaataacaaccctgtgccagtg
ggtgacatatataggaaatggatagtgctagtgttaaacaaagtagtaaaaatgtactgtccagtcagtatccta
gatattaagcaggggcctaaggaaccattccgagactatgtagacagattctacaaaaccctcagagcagaacag
gcaagtcaagatgtaaaaaattggatgacagaaactttgctaattcagaatgctaatcctgattgtaagcaaatt

ctaaaagccctaggcccaggggcaaccctagaagaaatgatgaatgcttgtcagggagtgggggacctactcay
aaagcaagagtcttggcagaggcaatggcagcagcccactcagatttaaaggggatatacagcagtctttatg
caaaagggggaaacaatcaagggattaagaagggaccagtaaartgcttcaattgtgggaaagtaggacatata
gcaaaaaattgtagggcacccagaagaaagggatgctggaaatgtgggcaagatgggcaccaaatgaaagactgc
agatcaggcagacaggcgcctgtgaagataggggacatctatgtgaggctttgttagacacaggggctgatgat
acagtagtagaacacttatctttagaaggtaggtggaaaccaagaatgataggaggcataggaggatttattaaa
gtaaaagaatatgaagatgtagaaatagaaatagaacataggaaagtagtagggacagttctagttggaccaacc
cctgtaaatattataggaaggaatattttaacaaaaattggatgtaccctaaatttcccataagctccatagat
gtagtacccgtatctttaaagccagggatggatggacccaaagtcaagcaatggcctttgtcaaaagaaaaata
gaagccttaacagctatttgtcaggaaatggaacaggaaggcaagatatcaaaaattgggcctgaaaatccatat
aatacacctatttttgccataaaaaagaaagacagtactaaatggagaaaattggtagattttagagaattaaat
aaacgaacacaggattttgggaagttcagttagggattcctcacccaggagggcttaaaaagaaaaaatcagta
acagtaytggatgttggggacgcttatttttcttgtcccttagacccagatttagaaatatacagctttcaca
attcctagtgtgaataatgagacaccaggggtaaggtatcagtacaatgtactaccccagggatggaaaggatct
ccagcaattttycagcactccatgacaaaaatacttgatcctttcagaaagaaaaacccagagatagaaatctat
cagtacatggatgacttgtatgtrgggtcagatttaccyataacagaacataggcagaaggtagaaaaactcagg
gaacatttatatgcatggggattcaccactcctgacaaaaagcatcaaaaggaacctccctttttgtggatgggg
tatgagctgcaccctgataagtggacagtgcagcctataaagttaccagaaaaggaaagttggacagtcaatgat
atccagagattggtaggtaaactaaattggcctagtcaaatatatccaggaataagagtaaaagaactctgcaaa
ttaattaggggtactaaatcacttacagaagtagtgacccttagtaaagaagcagaattagaactagaagagaac
agggaratattaaaagaacccgtacatggagtgtattaccagcctgaaaaggaattaatagtgaatgtgcaaaaa
caaggagaaggacagtggacttatcagatcttccaggatgaatacaagaatctaaagacaggaaagtatgctaga
caaaaggccactcatactaatgacatcagacagttggcagaagtgatacaaaaggtctcacaagaaagcatagtt
atctggggaaaactgcctaagttcaggctacccatatgtcggagtacctgggacgctttttggactgactattgg
caggcttcctggatycctgagtgggaatttattagtacacctcccctcatcaagctctggtatcaattggaaaar
gaacccataccaggagcagaaacctttatgtagatggagcagctaatagagaaagtaaacaaggaaaagcagga
tatgtaacagataagggtagacagaaaataagaaaactagagggcacaactaatcagaaagcagaactagaggca
gtcctcttggccttagaagaatcaggaaaaaatgttaacatagtgacagactcccagtatgtactagggattatt
gcagcaagtcctgatcaaagtgactccccccttagtgcagaaaataatagaggaaatgacaacaaaggaaaaagtc
tatttagcttgggtaccagctcacaaaggtataggggaaatgaagaaatagacaaattagtcagtaaagatatc
agaagagtcttattcctagaaggcatagatcaagcgcaggaagatcatgaaaaatatcacagtaattggagaaca
ctagccagtgactttggtctgccgccagtggtagccaaagaaatcatcaatagctgccctaagtgccatgtaaaa
ggggaagctaggcatggacaagtagattgcagcccaggtatctggcagttggattgcactcacttagaagggaaa
atcattttagtagcagtccatgtggccagcggcttcatagaagctgaagttattccagcagaaacagggcaggaa
acagcctacttcctgctcaagttagcagctaggtggccagtaaaaataatacacacagacaatgggcctaatttt
actagtgccacagtcaaggctgcctgttggtggctcaatgtaacacatgagtttggaattccctataatccccaa
agtcaaggagtagtggaatctatgaacaaagaattaaaaaagattatacaacaagtgagagatcaagcagaacat
ttaaagacagcagtgcagatggcagtatttgtccacaattttaaaagaaaggggggattggggggatacactgca
ggagacaggataatagacatgttagccacacaaatacaaacaacagaattacaaaaacaaattttaaaaattcaa
aactttcgggtctattacagagacagcagagaccctatttggaaaggaccagcgacgctcctgtggaaaggtgaa
ggggctgtagtcattcaggacaaaggggacattaaagtagttcctaggagaaaggcaaaaataattagggattac
cataaatatagaactaaacaagcaaaacagtggttctataggcatcactatgaagcagtcaatcctagggtaagt
tcaggggtatacataccagtaggccccgctgaaattatagtgaccacatattggggactcatgccaggagaaaag
gaagaacaattaggccatggggtaggcatagaatggagacagggaaaatacataacacaattagatccagaaaca
gcagataggataatccatctttattactttcaatgttttacagaatctgcagtgaggaaagcaattgtgggggaa
aaactcctgtacaagtgtaactacccggcaggacataggcaggtagggacattgcaatacttagcattaagagct
ttagtagggaaagtcagggtaagaccaccttttccaagtgttcagaaattagcagcagatatgttagaagaactt
aagcaagaagcartaagacactttcctaggccctggttgcaccaattgggacagcatgtttatgatacttatgga
gacacttgggaaggagtgacagcaataatcagaatcttacagcaagtaatctttgttcattatagaattgggtgt
catcatagcagaatagggattttgccaccctctagaagaggaagaagactgccaccatggcagcaaccagggagc
cagcccccaaccccatgcaacacatgctattgtaaaaagtgctgttatcactgctatgtttgtttcacaaagaag
ggtttgggaatctccgctacgcattatgctactgtgtattatggagtgccagtctggcgagaggcaaatgtaact
ttattctgtgcagcagatgctaattatgtgagcaaagaacaacacaatatttggcaacgcaggcatgtgtgcct
accgacccaaggccaacagagtttccattggaaaatgtaacagaaacctttaatatctggaaaaattacatggta
gatcaaatgcaggatgatatagttagcttatgggatcagagcttaaagccttgtgtaaaactaacagttatgtgt

gtcactctgaattgtacacaactaaatacaacaaatacaacacagactgagatgacaaatacaactataccacca
catacaactataccaccacacatcccacagttagacatttacaactgtagctttaatgttaccacggttctaaaa
gataagacaacaaagcagcaagctttattttatagacaagatataatagaaacaggtgaaacaggtacaaatggg
atcaaagggtatagattaatcaattgtaatacctctactatatcgcaagcttgtcccaaagtctcctttgagcca
ctacctatacagtattgtgcaccagcaggctatgccctgatgaaatgtaatgatatacaatttaatggtacagga
gaatgcagaaacgtgtcaatagttcattgtacccatgggatcaggccaacagtctccacacagttgatactcaat
ggaactttagcagaagggaatgccacaataatcagcaaaaatagtagtgatagtggaaaagatattatagtcaaa
ctagccagaccagtagaaattacctgtgaaaggacaggtaataatacaagaggacagatccaaatagggcccatg
acaatttataactcagaaaacatagtaggaaacacaagaaaggcttttttgcaagtataatgagacaaactggcag
ggtgcgttgaaagacacagtgcaggctctcaacgagagcctgatccacttccggaattcttcaggaggagattca
gaagttacctttctgcattttaattgtcatggagaatttttctactgtaataccactaaaatgtttagctacaat
tgcacaacagagggttgcaataccactgaaacaagcaataccactgaaacaactaaatatattccctgccatcta
aaacaggtagtcaggtcctggatgagagtaggctcaggcctatttgctccgcctattaggggaactctaagatgt
atatctaacattacaggaataatattacaaagggatgcacccctaaaaactgatgagaacagcaccttaagacca
ataggaggtgacatgacaaacatttggaggagtgaactgtacccctacaaggtggtgagggtcaaggcattgact
gtggcaccaacaaaagcaaagaggcctgttattggccataacagggaaaaacgtggactgtggggatgcacaggg
agattaatttgttacactaatgtgccatggaacaagacctggaccgggaaaaatgacactgaactagatgacatt
tggggaaacatgacttggcaacaatgggacaaattggtagacaactacactgacacaatattttagaaatacaa
agggcacaggaacagcaggaggctaatggaaaggccctactagagttggataaatgggcagatctttggagttgg
cttgacataacacaatggctatggtatataaaaatattcataataataatagcagggctagtaggactaagaatt
ctaatggctataataaatatgtgtcacagggttaggcagggttactcacctctgtcatttcagttgacaagagac
tgctttgcttttattgcttactggggacaagaacttaagcaaagtgctattagcttgctagattgtgttgctgta
tggaccgctaattggactgatcaagtaatagcaatagctcaaagaataggtagggggcatcttaaacatacccaga
aggattagacaaggattagaaagaagcctattataaatgggaaacacatggaaaaagagtagcttcataggatgg
ccagcagtaagggaaagaatgagacaaaccaaccctgagccaaagccggctccaggggtgggtgaagtgtctaga
gcattagcacaaagaggagggatacctgcaaaatatactccacaaaacaatcaagctttagcattcctagaagct
catgaagatgaagaggtaggatttccagtcaaacctcaagtgccgttaagaccaatgacctttaaaggagcattt
gacctcagcttctttttaaatgaaaagggggggactggatgggttagttttctctcaggccagaagtgatatctta
gacctctgggtttatcatactcagggatacttccctgactggcagaattacacaccagggccagggataagatat
cccctgacctttggatggctgtttaagttagtgccagtctcagaggcagaagcggaggagatgggaacatcaaat
gagaaggccatgctgctgcatccagcgtgcagccatgggagagacgacccatatggagagatccttgtctggaga
ttcgatagagcattgggaacaactcatgttgccttacagaagcacccagaactgttcaagagagactaa
>#48 FJ424871 7500 nt
atgggtgcgagagcgtcagtgttgacaggggggacgattggatgcatgggaacgaattaggcttaagccaggaagt
aagaaaaagtatatgctaaaacatgtagtwtgggcaagcagggagctggagagatttgcatgtaatcctgagctt
atggaaacagccgagggctgtgaaaaattattagggcaactagaaccagctctcaaaacagggtcagatggcttg
cgatctctgtggaacactttggtagtgctgtggtgtgtccaccggagaatagatgtctcagatactcagcaagcc
ttagaaaaatttaaggaagccatgtcaaaaagaaagtcagacagccgccctgaaacaagtcagaactatccagtg
gtaacaaatgcgcaagggcagatggtccatcagccgctgtctccaaggactctaaatgcctgggtaaaagcggta
gaagaaaaggccttcaacccagaaataattcctatgttcatggcattatcagaagggtctatcccttatgatatt
aatactatgctgaatgccattggaggacatcagggggcattacaggtcttaaaggaagtcatcaatgaggaagca
gcagaatgggacaggcagcacccagcagtggtaggacctctgccagtgggacaactgagggaaccatcaggggga
gatattgcaggaacaacaagtactcaacaagaacaagtagcttggatgacaaggaataataatcctgtgccagtg
ggtgacatatacaggaaatggatagtgctagggttaaacaaagtagtaaaaatgtactgtccagtcagtatcctg
gatattaagcagggacctaaggaaccattcagagactatgtagacagattctacaaaaccctcagagcggaacaa
gcaagtcaagatgtaaaaaattggatgacagaaactttgctaattcagaatgccaatcctgattgtaagcaaatt
ctaaaagccctaggcccaggggcaaccctagaagaaatgatgaatgcctgtcagggagtgggaggacctactcac
aaggcaagagtcttggcagaagcaatggcagcagcccactcagatctaaaggggggatatacagcagtctttatg
caaaaaggggaaacaatcaagggattaagaagggccagtaaaatgcttcaattgtgggaaggtaggacatata
gcaaaaaattgtagggcacccagaagaaagggatgctggaaatgtgggcaagatgggcaccaaatgaaagactgc
agatcaggcagacaggcgcctgtgaggataggggacatctatgtgaggctttgttagacacaggggctgatgat
acagtagtagaacacttacctttagaaggtaggtggaaaccaagaatgataggaggcataggaggatttattaaa
gtaaaggaatatgaagatgtagaaatagaaatagaacatagaaaagtagtagggacagtcttagttggaccaacc
ccggcaaatattataggaagaaatgtgttaacaaaaattgggtgtaccctaaattttcccataagccccatagag
gtagtacccgtatctttaaagccaggatggatggacctaaagttaagcaatggcccttgtcaaaagaaaaaata

gaagccttaacagccatttgtcaggaaatggaacaggaaggcaagatatcaaaaattgggcctgaaaatccatat
aatacacctattttgccataaaaagaaagacagtaccaaatggagaaaactggtagattttagagagttaaat
aaacgaacacaagattttgggaagttcagttagggatccctcacccaggagggcttaaaaagaaaaaatcagta
acagtactggatgttggggacgcttatttttcttgtcccttagacccagattttagaaaatatacagctttcaca
attcctagtgtgaataatgagacaccaggggtaaggtatcagtacaatgtactaccccagggatggaaaggatct
ccagcaattttccagcactccatgacaaaaatacttgatcctttcagaaagaaaaacccagaaatagaaatctac
caatacatggatgacttgtatgtagggtcagatttacccataacagaacataggcagaaagtagaaaaactcagg
gaacatttatatgcatggggattcaccacccctgacaagaagcatcaaaaggaacctcccttttttgtggatgggg
tatgagctgcaccctgataaatggacagtgcagcctataaagttaccagaaaaggaaagttggacagtcaatgat
atccagaaattggtaggcaaattaaattgggctagtcaaatatatccaggaataagagtaaaagaactctgcaaa
ttaatcaggggtactaaatcacttacagaagtagtgacccttagtaaagaagcagaactagaattagaagagaac
agggaaatcttaaaagaaccagtacatggagtgtattaccagcctgaaaaggaattaatagtgaatgtgcaaaaa
caagaagaagggcagtggacttatcagatcttccaggatgaacacaagaacttaaagacaggaaaatatgctagg
caaagggccactcatactaatgacatcagacaattggcagaagtgatacaaaaggtctcacaggaaagcatagtt
atctggggaaaactgcctaaattcaggctacccatatgccgaagcacctgggacgctttttggactgactattgg
caggcgtcctggattcctgagtgggaatttattagtacacccccctctcattaagctctggtatcaattggaaaaa
gaacccataacaggagcagaaaccttttatgtagatggagcagctaatagagaaagcaaacaaggaaaagcagga
tatgtaacagatagggtagacagaagataagaaaactagagggcacaactaatcaaaaagcagaactagaggca
gtcctcttggccttggaagaatcagggaaaaatgttaatatagtgacagactcccagtatgtgctggggattatt
gcagcaagtcctgatcaaagtgactccccccttagtacagaaaataatagaggaaatgacaacaaaagaaaaagtc
tatttagcctgggtaccagctcacaaaggtataggggggaaatgaggaaatagacaaattagtcagtaaagatatc
agaagagtcttattcctagaaggcatagatcaagcacaggaagatcatgaaaaatatcacagtaattggagagca
ctagccagtgactttggkctgccaccagtggtagccaaagaaatcatcaatagctgccctaagtgtcatgtaaaa
ggggaagctaggcatggacaagtagattgcagcccaggtatctggcagttggattgcactcacttagaagggaaa
atcattctagtagcagtccatgtagctagtggcttcatagaagctgaagttattccagcagaaacagggcaggaa
acagcttacttcctgctcaagttagcagccaggtggccagtaaaagtaatacacacagataatgggcctaacttt
actagtgccacagttaaggctgcctgttggtggctcaatgtaacacatgagtttggaattccctataatccccaa
agtcaaggagtagtggaatctatgaacaaagaattaaaaaagattatacaacaagtgagagatcaagcagaacat
ttaaagacagcagtgcagatggcagtatttgtccacaattttaaaagaaaaggggggattggggggatacactgca
ggagacaggataatagacatgttagccacacaaatacaaacaacagaattacaaaaacaaattttaaaaattcaa
aattttcgggtctattacagagacagcagagaccctatttggaaaggaccagcgacgctcctgtggaaaggtgaa
ggggctgtagtcattcaggacaaaggggacattaaagtagttcctaggagaaaggcaaaaataattagggattac
cataaatatagaactaaacaagcaaaacagtggttctataggcatcactatgaagcaatcaatcctagggtaagt
tcaggggtatacataccagtaggccccgctgaaattatagtgaccacatattggggactcatgccaggagaaaag
gaagaacaattaggcaatggggtaggcatagaatggagacaaggaaaatacataacacaattagatccagaaaca
gcagataggataatccatctctattactttcaatgttttacagaatctgcagtgaggaaagcaattgtgggrgaa
aaactcctgtgcaagtgtaactacccagcaggacatagtcaggtagggacattgcaatacttagcattaagagct
ttagtagggagagtcagggtaagaccaccctttccaagtgttcagaaattagcagcagatttgttagaagaacta
aaacaagaagcagtaagacactttcctaggccctggttgcaccaattgggacagcacgtttatgatacttatgga
gacacttgggaaggagtgacagcaataatcagaatcttacagcaagtaatctttgttcattatagaattgggtgt
catcatagcagaatagggattttgccaccctctagaagaggaagaagactgccaccatggcaacagccagggagc
cagcccccaaccccatgcaacacatgctattgtaaaaagtgctgttatcactgctatgtttgtttcacaaagaag
ggtttgggaatctccgctaagactgagcattatgctactgtatattatggagtgccagtctggcgagaggcaaaa
gtaactttattctgtgcagcagatgccagttttgtgagcaaagaacaacacaatatttgggcaacgcaggcatgt
gtgcctaccgacccaagcccaacagagtttctattggaaaatgtaacagagacctttgatatctggaaaaattac
atggtagaacagatgcaggaggatataattagcttatgggatcagagcttaaagccttgtgtaaaactaacagtc
atgtgtgtcactatgacttgttcaaacgacacaaaggggagcaataaatcaactgcaactacaactagcgcaaac
acacctacaagcacagagacaactataccaacaaacatcccagagttagatattttcaattgtacttttaatgtc
accacggttctaaaagataagaaaacaaagcagcaagccttattctacaagcaagatataacagaaacagataaa
aatgagtataggttaataaattgtaacacctctactatatcacaagcttgtcccaaagtctcctttgagccacta
cctatacagtattgtgcaccagcaggctatcccctgctgaaatgtaatgatgaacaatttaatggtacaggagaa
tgcaacaatgtgtcaatagttcattgtacccatgggatcaggccaacagtctccacacagttgatactcaatgga
actttagcaaaaggaataatgccacaataatcagcagcaatagtagtgatagtgcaacagatattatagtcaaa
ctagccaacccagtagaaatcacctgtgaaaggacaggtaataatacaagaggacagatccaagtaggcccctg
acaatttataactcagaaaacataataggaaacacaagaaaggctttctgcaagtataatragacagcatggcag

aaggcgttacaagctacagtgcgggctctcaaaacgaacctgacccactacttcaacaagtcttcaggaggagat
ccagaggttacctctctgcactttaattgtcatggggaattttctactgtaataccactaaaatgtttaactac
aagtgtacaggggaacactgcaattgtacagacaaggagctttgcaataagactgcagaaatggagtatattcca
tgccgcctaaaacaggtagtcaactcctggatgagagtaggctcaggcctatttgctccwcctmttaggggaact
ttwagatgtatatytwatattacaggaatamtattacaaagagatacaccccysgctgagaataataataacacc
accttaagaccattaggaggtgaaatgagaaacatttggaggagtgaattgtacccctacaaggtggtaaaggtc
aaggcattgtctgtggcaccaacaaaagcaaagaggcctgttgttagccataacagggaaaaacgtggactgtgg
ggatgcacagggaaattgatttgttacactaacgtgccatggaacactacctggaccgggaaaagtgatagtgaa
ctagatggcatttggggaaacttgacttggcaacaatgggacaaattggtagacaactacactgacacaatattt
ttagaaatacaaaaggcacaagaacagcaggaggctaatgaaaaggccttactagagttggataaatgggcagat
ctttggagttggcttgacataacacaatggatatggtacataagaatattcataatagtaatagcagggctagta
ggactgaaaattctaatggctataataaatatatgtcacagggttaggcagggctactcacctttgtcatttcag
ttgacaagagactgctttgcttttattgcttactggggacaagaacttaagcaaagtgctattagcttgctggac
tgtgttgctgtatggactgctgattggactgaccaagtaatagcaatagctcaaagaataggtaggggcatctta
aacataccaagaaggattagacaaggattagaaagaagcctattaggaaacgcatggaaaaagagtagctttgta
ggatggccagcagtaagggaaagaatgaggcaaaccagccctgagccagatccggctccaggggtgggtgaagtg
tctatagcattagcacaaagaggagggatacctgcaaaatatactccaaaaaacaatcaagctttagcattccta
gaagctcatgaaaatgaggaagtaggctttccagtcgtacctcaagtgcctttaaggccaatgacctttaaaggg
gcatttgacctcagcttcttttaaaagaaaagggaggactggaagggttagttttctctcaggccagaagtgat
atcttagacctctgggtttatcacactcagggatacttccctgactggcagaattacacaccagggccaggggta
agatatcccctgacctatggatggctgtttaagttagtgccagtctcagaggcagaagctgaggagatgggaaca
gcaagtgagagagctatattgctgcatccagcgtgcagccatgggagagacgacccacatggagagacccttgtc
tggaggttcgatagagcattgggaacaactcatgttgccctacagaagcacccggaactgtttcagagagactaa
>#49 JN091690 7527 nt
atgggtgcgagagcgtcagtgttgcggggagagaagcttgatagatgggaagccataaagcttagacccggaggc
aggaaaagatatttgttaaaacacctaatatgggccgggagtgagttacagcgcttcgcgatgaatcccggcctc
atggagagcaaagaaggctgctggaaaatcatcctccagctacagccatctgtagacattggttctccagaaata
atctccttgtttaacaccatttgcgtgctttattgcgtacatgaaggagaaaaagtgcaaaatacggaagaagca
gttaaaattgtgaaagtgaaactaacaaagcaagaaagtagctccgcagcgacatctagcggacagaataaaaat
acagaagagacacctagtggtagccaagagaatgcaggaagagcagcagcgccatctagtggcagactgtatcca
gttatcacagacatgcagggagcagcaaggcatcagcctatatcacccagaactttaaatgcttgggtaaggaca
gtggaagacaaagggttcaatccagaagtgataccaatcttttcagctttatctgaggggggctaccccttatgat
ttaaatactatgcttaatgctattggggatcatcaagcagcaatgcaaatgctaaaggaagtaatcaatgaggag
gcagcagactgggatagaacacacccagctcatgcaggaccccaacaagcggggatgctgcgagagccaacagga
gctgatattgcaggaaccactagctcagtacaagagcaagtgcagtggatgacaacccctcaggcacaaggagga
gtaccagtaggggacatctataaaagatggataattatagggttaaacaaattagttaggatgtacagccctgtt
agtattttagacattaaacagggaccaaaagaaccattcagagattatgtagacaggttctacaaatgcatcaga
gcagaacaggcctctcaaccagtaaagacttggatgacagaaactctgttagtccagaatgcaaatccagactgt
aaacacattttgaaggcactaggacaaggagcaaccttagaggaaatgcttacagcttgccagggagtaggagga
ccttcccataaggcaaaaatactggcagaagcaatggcatcagcaaacagtgcagttggggggaataaatatgtta
caaggaggaaagaaacccctgaggaaaggccagctgcagtgtttcaattgtgggaaaccagggcatacagccaga
aactgcagagccccaaggaaaaagggctgctggaggtgtggtcaggaaggacatcagatgaaagactgcacagga
agacccaacaattcaggggtaactgtgaaagtacaaggacaagtctgtcaagctcttttagacactggagcagat
gacagcgtttttttgtaatcttaagctaaagggacagtggactccaaaaactataggggggaatagggggctttgtg
ccagtgaatgaatattacaacattccagttcagataggaaataaagaagtaacagccactgttctagtaggggac
acccccattaatataataggaagaaatattctacaacaattaggatgtactctaaatttccccattagtaaagtt
gatgttgtaaaggtgaagctaaaagagggaatggatggcccaaaggtaaaacaatggcccctctctaaagaaaaa
attgaagctttaacagagatctgcaattccttagaagaggaaggaaagatttctgcaattggccagaaaatcca
tataatacaccaatctttgcaattaagaaaaaggatggttctaaatggagaaaattggtagattttagagagtta
aacaagagaacacaggatttctgggaattgcagctaggaataccccatccagcaggactaaagaagaggggcatg
gtaacagtactggatgtaggggatgcttatttttctattcctctggatccagacttcagacagtatactgctttt
accattcccagtcttaacaacaatacaccaggaaaaagatttcagtataatgtgttgcctcaagggtggaagggg
tcaccagcaatttttcaaaacagtatgacaaaaattttagatcccttcagaaaagaccacccagaggtggatgtc
tatcagtacatggatgatctttatgtagggtcagatttaaatgaaaatgaccataggcagctcataaaaaggtta
aggcagcatttgttagcctggggactagagactccagacaaaaagtatcaggaaaaacctccatttctgtggatg

ggttatgagctgcatccaaacaaatggacagtgcaaaatattacactgccagagccagagcagtggacagtaaat
catattcagaaattagtaggtaaattaaattgggctagtcaaatttacccaggaataaaaactaaggaactgtgc
aagctcattagaggagtaaaagggctgactgacccagtacagatgactagggaagcagaactagaattagaagaa
aacaaagaaattctaaaagaaaaggtacaaggggcttattatgatcctaagctacccctgcaggcagcagttcaa
aagcaggggcaaggacaatggacataccagatttatcaggaagaaggaaaaaacctgaaaacaggaaaatatgca
aaatcagcaggcactcatactaatgagataaggcaactagcaggattgatacagaaaataggtaatgaaagcatc
ataatttggggaattgtgcctaaattcttgctgccagtatccaaagaaacatggagtcaatggtggactgattat
tggcaagttacctggatacctgagtgggagttcattaataccccaccattaatcagattatggtataatttgtta
gataaccccatcccagaagcagaaactttttatgtggatggagcagctaatagagatagcaaaaaagggaaagca
ggatatgtcacagatagggggaggtacaaggcaaaagagttagaaaacaccacaaatcagcaagcagaattatgg
gcagtggatttggcattaaaagattcaggagcacaagtaaacatagtgacagactcccaatatgttataggaatt
ctacaaggactgcctgatcaaagtgactcccccatagtagaacaaatcatacaaaaattaaccaataaagcagca
gtctatttagcctgggttccagcccatagaggaataggggcaatgaagaagtagacaagttagttagcaaaaat
atcagaagagtactctttctagaaggaattaatgaggcacaggaagatcatgataaatatcatagtaattggaaa
gcattggctgatgaatacaatcttcctcctgtagtagcaaaagaaatcatagcccaatgccccaagtgtcacata
aaaggagaagccatacatggacaagtagactgtagtccagaagtatggcagctagattgtacccacttggaaggg
aaaatcatcatagtagcagtacatgtggccagtggattcatagaggcggaggtcatacctgaggaaacaggaaaa
gaaacagcttacttcatattaaaattagcaggcagatggcctgtaaagaagattcatacagataatggccaaat
ttcactagtacagcagtaaaggctgcatgttggtgggcgcaaatccaacatgaatttgggattccatacaatccc
caaagtcaaggggttgtggaatccatgaataagcaattgaagcaaattattcagcaggttagggaacaagcggaa
caactgaaaacagcagttgtaatggcagtctacattcataattttaaaagaaaaggggggattggggagtatact
gcaggagaaagactattagacctactgactacaaacatacagacaaagcaattacaaaaacaaattttaaaaatt
caaaattttcgggtttattatagggacggcagagatccggtttggaagggaccagcgcgactgctgtggaaaggt
gaaggagcagtagtaataaaggagggggaagatattaaagtagtgcccagaagaaaagttaagataataaaagac
tatggattagttaaacaccatatattcacaagtaaatgttgcagcagctggaaatatagacaccattatgaaact
gatataccaaaaagagcaggagaaatacatattcccattaatgagaaagcaaaaatagtagtgttacactattgg
ggtctagcctgtggggagagaccatggcatttaggacatggagtaggattggaatggagacaaggaaaatatgtc
acacaactagaccctgcaacagctgaccagttaattcatactaagtattttgcatgctttactgcaggagcaatt
cgtcaagcaatcttaggagagagaattctgacattctgccaatttcgatcaggacacagacaggtaggaacttta
caatatttagccttttttgaaggtagtggagattcaatacaagacagctccaagacacaggaggccgttgccatct
gtactaaagttaacagaggacattctagaagaaataaaacaagaagctgttaggcattttccaaggcaaatatta
caaggagtagggaattgggtctttacagtgcatggagactcctgggaaggagtacaagaattaatcagaattctg
cagaaagctttgtttgctcactatcgacatggttgtgttcacagcagaataggacctatagatcctcaagtagcc
ccatggaatcatccaggagctgcacctaagacaccatgttcaaattgttattgtaaaaagtgctgctttcattgc
ccgctttgctttacgaaaaaagcattaggaatctccgtccattatgctacagtattttatggggtgcctgtttgg
aaagatgcaaaacctcaattgttttgtgcctctgatgcagacattactagtagagacccacataatatatgggct
actcataactgtgtaccccctagatccacatccctatgaagttccattatcaaatgtctcagtagactttgacatg
gaaaacaactatatggtggaagaaatgaaaaaggatttaattgcactcttccaacaaagctttaaaccctgtgtt
aagttaacacctttctgtgtaacaatgaactgtgtaaaacttaaggagacaacaactacaatagctaccatcaca
aaccccccttgtaaacttgacaggaatagaagattataaagtgtataattgtagttttaatcagaccactgagttt
agagacaagaaaaagcagatttattctttattttacagagaagatattatgaaaacagacaataacgatagttat
tatttgcataattgcaacacctcagccattacccaagaatgcgaaaagtcaagctttgaaccaattcctattaga
tattgtgctccagcaggatatgctatgttgaaatgtaacagtgcaaactttacaggagtaggaacttgtaacaat
gtaagtgtagtacattgcacacatggaatatatcccatgatagccacagccttacatctaaatggaacacttgaa
aagcacaacacaacagcttattttgcaacacccacacacaataagccactgttgataaaattcaataagtcagta
gacatgaattgtacaagaacaggcaataattcaaggggacaagtacaaatcggtccaggcatgaccttttataat
atagaaaacatagttggaaataccaggaaagcttattgtacggtaaactaccaggaatggagtcatgccatagga
gaagctaaaaaagtggccgaagaagccctaaagaagaatattacctttcggtggaaccaaggaggagatctagaa
gttaccaacttctggtttaattgtcaaggagaattttctattgcaacctcactaactggacaaatataacgtgg
ctagagacgtggctaaagactaacaatagttcccgtccccttgtagcaccatgtaaacttaggcagatagtaaac
cattggggtatagtatctaaaggtatctacctacctcctagaaggggaaatttaacatgcagatctaacattaca
gggttcattacgacatgggataatgacacggagaacgtgcttgttaccttttcagccaaagtagaagactattgg
aaggtagaaatgtcacgatacaaagtagtggaaattcagcccttggccatagctcctactacaggaaaaaggccg
gagattagagccaatcacacaaggtcaaaaagaagtgtttggggatgtgccaataaattggtatgccacagtaat
gtagtgtggaatatgacatgggctgaaaatggaactacttgccctcagggttcagaagactattataattgcatc

tggaacaacttgacatggcagcagtgggaaaagctagtggctaattctacagaagaaatatatacattattagag
aaagcacaagtgcaacaagaaactaacaaaaaagaactctttgagttagataagtggagcactctttgggattgg
tttgacattacacaatggctgtggtatattaagatagctatacttatagtagcagggttagtaggacttagaatt
gttatgtttatagttaatgtaattaggcaagttaggcagggttatatgcccctgttttcacagagtagacaactt
ttcgattggcttagcaatacatatagtatcttaagaacctcgcttatacaggcaatagataggttagctaacttc
acaggctggtggacggacttggttatagcaggagtagcatttgtagcccaaggcatcaggaatatcccaaggaga
attagacagggcctagaaatagccttaaattaaatgggaaacatctttggcaagtggcctggagcccaaagggca
atccaagcgatacatgagagtaatcctacaataaaagggcaagcatcacaggacctccaggcaagggggagggttg
actactagcactctaggaacatcagcagatgtaataaattactctcatgaccactctgaagaggaagtcggcttc
ccagttaggccagcagtaccaatgagacccatgacagaaaaactagcagtagatctgtcatggttcctaaaagaa
aaggggggactggatgggttacctttctccacaaagagagcagccatcttagatatgtggatgtataatacacaa
ggaatattcccagactggcagaactacactcctggaccaggaatcagatacccactatgcagagggtggctgttt
aaactggtaccagtagacccacctgaagatgatgagaggaacatcttgctacacccttactgttctcatggaaga
gaagatcctgcaggagaaggagagaatctgatctggtgctttgacagcagtctatcaagaagacacatagccaga
gagagatatccggagtacttcaaataa
>#50 AF447763 7578 nt
atgggtgcgagagcgtcagtgttgaggggagataagctggatacatgggaatccataaggcttaaatccagaggc
aggaaaaaatatttaataaaacatctagtatgggccggaagcgaactacagcgtttcgcgatgaatcccggtctc
atggagaacgtagaaggctgctggaaaatcatcctccagctgcagccttcggtagacattggttctccagaaatc
atttctttgtttaataccatctgtgtactctactgcgtacacgcaggagaaagagtccaagatacggaagaagca
gtcaaaattgtgaaaatgaaactaactgtacagaaaaataactccacagcgacatctagtggacaaagacagaat
gcaggtgaaaaagaggaaacagtgccacctagtggcaatacaggaaacacagggagagcaacagagacacctagt
gggagtagactatacccagtgataactgatgcacagggagttgcaaggcatcagcctatttcacctagaactcta
aatgcctgggtaagggtaatagaagaaaaagggtttaatccagaagtaataccaatgttctcagcattgtctgag
ggagcaaccccttatgatctaaatagtatgctcaatgctgttggggaacatcaagcagcaatgcaaatgttgaag
gaagtcatcaatgaggaagcagcagagtgggacagagcacatcccgctcatgcaggaccccagcaagcagggatg
ctaagagagcccacaggggcagatattgcagggaccactagtacgctacaagaacaagtactgtggatgacaacc
ccacaggcacaaggaggagtgccagtaggagacatctataaaaggtggataattttaggattaaataaattagtc
agaatgtacagccctgttagcattttggacataaaacagggaccaaaagaaccattcagagattatgtagacaga
ttctacaaaacaatcagagcagaacaagcatctcaaccagtaaaaacttggatgacagaaactttactggtacaa
aatgcaaacccagattgtaagcatatcttaaaagccttggggcaaggagcaacattagaagaaatgctcacagcc
tgtcaaggagtgggaggaccctctcataaggcaaagattctggctgaagcaatggcctcagcaacagcaggggga
gtaaatatgctgcagggaggaaaaagaccacccttaaaaaagggtcagctgcagtgttttaactgtgggaaagta
ggccatacagcaagaaattgtagggctccaagaaagaaaggttgctggaggtgtggacaagagggacatcaaatg
aaggactgcaccaccagaaacaacagcactggggtaacagtaaaagtccagggacaagtctgtcaagctcttta
gatactggagcagatgacagtgtttttgtaacatcaaattaaagggacagtggacaccaaaaaccataggagga
ataggaggatttgtaccagttagtgagtactataatattccagtacaaattggcaataaagaagtcagagccact
gtcctagtgggagaaaccccattaatataataggtagaaatattttaaagcaattaggatgtaccttaaatttt
cctattagcccaatagaggtagtaaaagtacaattaaaagaaggaatggatgggccaaaagtaaagcagtggccc
ctctccaaggagaaaattgaggcattaacagaaatatgtaagacattggaaaaggaaggaaaaatttctgcagtt
ggaccagaaaacccatataacacaccaatttttgccattaagaaaaaggatacctctaaatggagaaaattagta
gatttcagagaactgaataaaagaactcaagatttttgggagttacagctaggaataccccatccggcagggtta
agaaaaagaaatatggtgacagtactggatgtaggggatgcctacttttccattcccctggatccagacttcaga
aagtatacagcttttaccatacccagtctcaataataacacaccagggaaaagatttcagtataacgtgttacct
caaggttggaagggatctccagcaatttttcagagcagtatgacaaaaatcctagatcctttcagaaaagaacac
ccagatgtggacatttaccaatatatggatgatctttacataggttcagatcttaatgaagaggaacataggaaa
ctgataaagaagctgagacagcatctgttaacatggggattagagacccctgacaaaaagtatcaggaaaaacct
ccattcatgtggatgggctatgagctacatccaaataaatggacagttcaaaatatcacattaccagaaccagag
cagtggacagtgaatcatatccagaagttggtaggcaaacttaattgggccagtcaaatttatcatggaataaa
actaaagaactatgcaaattgattagaggagtaaaaggattaactgagccagtagaaatgaccagggaagcagaa
ttggagttagaagaaaataagcagattctaaaagaaaaggttcaaggagcatactatgatcctaaattacctctg
caagcagcaatacagaagcaggggcaaggacagtggacatatcagatatatcaggaagaagggaaaaatttaaaa
acaggaaaatatgcaaaatcaccaggtacccacaccaatgagataagacaattagcaggactgatacagaaaata
ggcaatgagagcataataatttggggtattgtgcctaaattttttattacctgtatccaaagagacatggagccag
tggtggactgattactggcaagttacctgggtacctgagtgggaatttattaacaccccaccactaatcaggcta

tggtacaatctgttgtctgaccccatcccagaagcagaaacctttatgtagatggggcagcaaacagagacagt
aaaaagggaagagcaggatatgtaacaaacagaggcagatacaggtcaaaggacttagagaacaccactaatcaa
caagcagaattatgggcagtagatctagccttaaaagactcaggagcacaggtaaatatagtcacagattcccaa
tatgttatgggagttttacagggattaccagatcaaagtgactcccccatagtagagcaaattattcaaaagtta
acacaaaagacagcaatttatctagcatgggtaccagcccataaaggtataggggggtaatgaagaagtagacaaa
ttggttagtaaaaatattagaaaaatattattcctggatggaattaatgaagcacaggaagaccatgataaatat
cacagtaattggaaagctttagctgatgaatataatctgccccccagttgtggctaaagaaattattgctcagtgt
ccaaaatgccatataaaggagaggctatacatggacaggtggactacagtccagaaatctggcaaatagactgt
acccacctagaaggaaaggtcatcatagtagcagtgcatgtagctagtggtttcatagaagcagaagtcatacca
gaagaaacaggaagagaaaccgcttacttcatcctaaaattggcaggaagatggcctgtaaagaaaatacataca
gataatggaccaaattttactagtacagcagtgaaggcagcctgctggtgggcacaaattcaacatgaatttggg
attccatataatcctcaaagtcaaggagtagtagaatctatgaataaacaattaaagcaaattatagagcaagtc
agggaccaagcagagcaactgaggacagcagtaatcatggcagtgtatatccacaattttaaaagaaaagggggg
attggggagtacactgcaggggaaagactattagacatactaactacaaatatacagacaaaacaattacaaaaa
caaattttaaaagttcaaaattttcgggtttattatagggacgccagagatccaatttggaagggaccagcgcga
ctactgtggaaaggtgaaggggcagtagtaataaaagaaggagaagacattaaagtagtacccaggagaaaagca
aaaatcataaaagagacatggacaagcctagttaaacatcatatctttacaaccaaatgctgtaaagattggaag
tatagacatcattatgaaactgatacaccaaaaagagcaggggaaatacacatacctctaacagaaagatcaaaa
ttagtggttttacattattggggtctagcctgtggagaaagaccatggcatctaggtcatggcataggattagaa
tggagacaaggaaaatacagtacacaaatagaccctgaaacagcagaccaattgattcacactaggtattttacc
tgttttgctgcaggagcagttcggcaagcaatattaggagaaagaatattgacattctgccactttcaatcagga
cacagacaggtagggactctgcaattcttagctttcagaaaggtagttgagagccaagataaacagccaaaggga
ccaaggaggcccttgccatctgttacaaaactaacagaggacatcctagaagaaataaaacaagaagcagtgaaa
cactttccaagaccaatattacaggggtaggaaattgggtcttcaccatttatggagactcctgggagggagta
caggaattaatcaagatcttgcagagagctttgtttacccactatcgccatggttgtatccacagcagaatagga
cccatagatcctcaggtagcaccatgggaacatccaggagctgcacctgaaacaccttgtacaaactgttactgt
aaaaaatgctgctttcattgcccagtttgctttacgaaaaaagcattaggaatctccacttattacaccacagtg
ttttatggagtacctgtttggaaagaggcccaaccaaccttgttttgtgcctctgatgctgatattactagtaga
gataaacacaacatatgggcaacacataactgtgtgcctttagatcccaatccttatgaagtaaccctagccaat
gtgtcaataaggtttaatatggaagaaaattacatggtgcaagagatgaaagaagatatattatcactttttcaa
cagagttttaagccttgtgtaaaattaacaccattttgcataaagatgacatgtacaatgactaataccacaaat
aaaaccctgaattcggcaacaacaaccttaacaccaacagtaaatttgagttctatacctaactatgaggtgtat
aattgttcatttaatcagacaactgagtttagagataagaaaaaacaaatatattccttgttttatagagaagat
attgtaaaagaggatggtaacaataatagttattatttacataattgcaatacctcagtcattactcaagaatgt
gataaatctacttttgaaccaattcccatcagatactgtgctccagcaggctttgccctgttaaaatgtagagat
cagaatttcacagggaaaggacaatgctccaatgtctcagtagttcactgtacacatgggatttatcctatgata
gccacagcattacacttaaatgggtccctggaagaagaagaaacaaaagcttactttgttaatacctcagttaat
acacccttattagtaaaatttaatgtatcaataaatttaacgtgtgaaagaacaggaaacaatacaagaggtcaa
gtacagataggtccaggtatgaccttttataatatagaaaatgtagtaggggacaccaggaaagcttattgttca
gtcaatgcaacaacatggtacaggaacttagattgggctatggctgccataaacacaaccatgagggccagaaat
gaaacggtacaacaaacgttccaatggcagagggatggagaccctgaggtcactagcttctggttcaattgtcaa
ggagaattcttttactgtaatctcacaaattggactaatacctggacagctaatagaaccaataatactcatggt
actcttgttgcaccatgcagactgaggcagatagtaaatcattggggtatagtgtcaaaaggggtttaccttccc
ccaaggaggggaacagtaaaatgtcactcaaacatcacaggacttatcatgacagcagaaaaagacaacaataat
agttataccccccaatttctgctgtagtagaagactattggaaagtagaattagcaagatataaagtggtggaa
attcagcccttgtcagtggctccaaggccaggaaaaaggcctgaaattaaggccaatcatactaggtcaagaaga
agcctctggggatgtgctaacaaattggtgtgtcacagtagtgtgccatggaacctcacctggctgaagattct
acaaagtgcaatcacagtgatgcaaagtactatgactgtatatggaacaatttgacttggcaggaatgggatcga
ttagtagaaaactctacaggaaccatatactccctgttagagaaagcacaaacacaacaggagaaaaacaaacaa
gagttgttagaattagacaaatggagcagtctttgggattggtttgatataacacaatggctgtggtatataaaa
atagctataatcatagtagcaggattagtaggacttagaattctcatgtttatagttaatgtagttaagcaagtt
aggcagggttatacaccccctattttcacagactatcatagtaggagttagacagatcattgagtggagcagtaat
acttatgctagcttaagagttttgctaatacaagccatagacagacttgctaactttacagggtggtggacagat
ttaatcatagaaggagtggtttacatagccaggggaatcagaaatattcctagaagaattagacagggtctggaa
ctagccttaaattaaatgggaaacatatttggtagatggcctggggcccggaaagccatcgaagatcttcataac

acctcaagtgagcctgtaggacaggcctcacaagacctccagaataaaggaggtctcactactaacaccctaggt
acctcagcagatgtgttagaatactctgcagaccatactgaagaagaagtaggttttccagtcagaccagcagta
cccatgagacccatgacagagaagctagcaatagatctgtcatggttcttaaaagaaaagggggggactggatggg
ctatttttctctccaaaaagagcagccatcctagacacctggatgtataatacacagggtgtctttccagactgg
cagaactacacccctggaccaggaatcagatacccactgtgtaggggatggttatttaagttggtaccggtagac
ccaccagaagatgatgagaagaacatcttgctacatccagcctgtagccatggaactaccgatccagatggagag
actctgatctggcgctttgacagcagcctagcaagaaggcacatagccagagaaagatatccggagtacttcaaa
taa
>#51 JQ768416 7611 nt
atgggtgcgagagcgtcagtattaaggggagaaaagcttgatacttgggaaagcattaggcttagacccggaggc
aagaaaaagtacttaataaaacatctagtatgggccggaagcgagctcgaacgcttcgcgatgaatcccggtctt
atggaaaacaaagaaggctgctggaaaatcatcctccagctacagccctcaatagacattggctctccagaaata
gtctccttgtacaacactgtctgtacactattttgtgtacacgcaggagaaaaggtacggacacggaagaagcc
gttaaaattgtgaaagtaaaactaactacagtaaaagatgatgccgcagcgacatctagcggacaaggtcagaat
gcaggaggaagagggacagcagtgacacctagtggtaaaagtgaaaatgccagtagagtaacagtgccacctagt
gagggaggaaggttatatccaattgtgactgaccttcagggaacagcaagacatcagcctatttcacctagaact
ttaaatgcttgggtaaaaacagtggaagaaaagggatttagtccagaagtcattccaatgttctctgctttatct
gaaggggcaacccctttatgatttaaattccatgcttaatgctattggagaacatcaggctgcgatgcaaatgtta
aaggaagtaatcaatgaggaggcagcagagtgggacagactacatcccgctcatgcagggccccagcaggcaggg
atgctaagggagccaacaggagcagacattgcagggaccactagtacaacacaagaacaaatacagtggatgaca
acacctccagcacaaggaggagtaccagtaggggatatctataagagatggatcattctagggcttaataaattg
gttagaatgtataacccagtgagtattctggacataaagcaagggccaaaggaggcatttagagattatgtagat
agattctacaaaactatcagagcagaacaagcctctcagccagtgaaaacatggatgacagaaaccttgctgata
caaaatgcaaatccagattgcaagcatattctaaaagccttaggacagggagcaacattggaagaaatgctaaca
gcctgtcagggggtaggaggccctacccataaggcaaaaatcctggctgaagcaatggcaacagctactaataca
gtaggagtaaatatgctgcagggaggtaagaaaccattaagaaaggggcagcttcaatgttttaattgtggaaaa
ttagggcatacggcaaaaaactgtagagccccaaggaaaaaaaggctgttggaggtgtggacaggagggacatcaa
atgaaagactgcacagccagaactaacaatactggggttgcagtcagagttcaaggacatctctgtcaagctctg
ttagacacaggtgctgatgacagtgttttctgcaatttacagctaaaaggacagtggaaaccaaaaaccataggg
ggtataggggggtttataccagtaagtgagtactataatattccggtgcaaattggcaataaagaagtaagagcc
actgtcttagtgggggatacacccctaaatataataggaagaaacattttacaacaattgggctgtactttaaat
ttccccattagtaaagttgaagtagtaaaagtagaactaaaacaaggcatggacgggccaaaagtaaaacaatgg
cctctgtctaaagagaaaattgaagccttaacagaaatatgtagtactttagaaaaggaaggaaaaatctctgcc
ataggaccagaaaatccttacaatacacccatatttgcaatcaagaaaaaggactcttctaaatggaggaagtta
gtggacttcagagaattaaataaaagaacacaggatttctgggagttacaactagggataccacaccctgcagga
ttaaaaaagagaaaagtggtaacagttctggatgtaggggatgcttatttttccattcctctagatccagacttt
agaaaatatacagcatttaccatccctagtctcaataacaacacaccagggaagagatttcagtacaatgtgtta
cctcaaggatggaagggatcacctgctatatttcagagtagcatgacaaaaatttttagaccctttcaggaaaaaa
catccagaggtagatgtgtaccaatacatggatgatctttatgtaggatcagactacagtgaagaggagcacaga
aagctaatgaaagagctaagacagcacttgttagcatggggattagaaaccccagacaaaaaatatcaagaaaaa
cctccattcttatggatgggctatgagctacatccagataaatggacagtgcagaacatcaccctgccagaacca
gaacagtggacagtgaatcacatccaaaagctggtaggcaagttaaattgggcaagtcaaatctatgcaggaata
aagacaaaagaattatgcaaattaataagaggagtaaaggactaactgaccctgtggaactaacaagagaagca
gaattagagttagaagaaaataaagaaattctaaaagaaaaggtgcagggtgcatactatgaccctaaactacca
ttacaagcagcagtacaaaaacagggacagggtcaatggacatatcaaatatatcaggaagaaggaaaaaattta
aaaacaggcaagtatgcaaaatctccaggcacccacactaatgagataaggcaattagcaggtcttatacagaaa
ataggtaatgaaagcatagtaatttggggcactgtaccaaagtttctcttgccagtgactaaggaaacatggagt
cagtggtggacagattactggcaagtcacctgggtacctgaatgggaattcattaacacccccccattgattaga
ctatggtacaatttgctctctgaccccattccagaagcagaaacattttatgtagatggggcagcaaatagggaa
agtaaaaagggaaaagcaggatatgtcacagacaggggtagatataaaaccagggaactagaaaataccacaaat
cagcaagcagaactatgggctgtagatttagctctaaaggattcagggtcacaagtaaacatagtaacagactcc
cagtatgtcatgggggtactgcaaggatgcctgatcaaagtgactcccccatagttgaggaaatcatacaaaaa
ctcatgcagaaaaatgcagtgtatctagcatgggtacctgctcataaaggaataggaggcaatgaggaagtagat
aagctagtcagtaaaaatattaggaaagtcctattttagaagggataactgaggcacaggaagatcatgataaa
tatcatagtaattggaaagcaatggctgatgaatacaatcttcccccctatagtagccaaggaaatcattgcccag

tgttctaaatgtcacaaaaaaggagaagccattcatggccaagtagactgtagcccagaagtatggcagctagat
tgcactcacctagaagggaaagtcatcatagtggcagtgcatgtggccagtggtttcatagaagctgaggtcata
ccagaggaaacaggaaaagaaacagcttacttcattcttaaattggcagggagatggcctgtaaaaagaatacat
acagacaatggaccaaattttactagcacagcagtaaaggcagcatgttggtgggcacaaatccaacatgaattt
ggcataccttataatccccaaagtcaaggagtagtggaatctatgaataagcaattaaagcaaataatagagcaa
attagagaccaagcagagcagttaaaaacagcagtagtaatggcagtctacattcacaattttaaaagaaaaggg
gggattggggagtatactgcaggagaaagactgctagatatactaaccacaaatatacagacacaacaactacaa
aaacaaattttaaaagttcaaaattttcgggtttattatagggacgccagagacccgatttggaagggaccagcg
cggctactgtggaaaggtgaaggagcagtagttataaaagagggtgaggacatcaaagtagtacccagaaggaaa
gcaaaaataatcaaagaaacctggaatagtctagtgaaacatcatatctttacaactaaatactgcaaagagtgg
aaatatagacaccattatgagacagatgtgccaaaaagagcaggggaaatacacatacctcttgaaggaaaggca
agaatagttgtgctacattattggggattagcttgtggggagagaccatggcatttgggacatggagtagggata
gaatggagacaaggaagatatagcacacagatagaccctgaaacggcagaccagcttattcacactaagtacttt
gcatgttttactgcaggagcagtgcggcaagcaatcttaggagaaaggatactgacgttctgccattatcaaaca
ggacatagacaggtagggactttacaatatctagcttttctaaaagtagttgaagctcaatacaagacagcaaag
aaacctggaagacctttgccagctgtagttaaattaacagaagacgtgttagaggaattaaaacaagaagcagtt
aggcactttccaagaccagtactgcagggagtaggaaattgggtgtttacagtttatggagattcctgggaagga
gctcaagagttaatcaaaattctgcaaagggccttgttttctcattatcgacatggttgtgttcacagcagaata
ggacctatagatcctcaggtagccccatggatgcatccaggagctgcacctgagacaccttgtacaaattgttac
tgtaaaaagtgctgctttcattgcccgctttgctttacgaaaaaagcattaggaatctccacatactatgctaca
gtcttttatggagtacctgtttggaaagatgctacaccaccactgttttgtgcttctgatgcagatgttgctagt
acacaggcacacaacatctgggcaacacataattgtgttcccctagatcctcatccttatgaagtgcctttacca
aatgtatccatagaatttgatatgtctactaattatatggtagaagagatgaaaacagatttgatctcactttt
cagcaaagttttaaaccatgtgtaaaattaacacccttttgtataagaatgacatgtgcaaatgtaacagaagca
acaacaacaacaccaccaacaacgtcaactagcacaacagaaccaccaacatcaaagaccacagtgaatacgtca
gagatagaagattataaggtgcttaattgcagctttaatcaaacaactgagtttagggataagaaaaagaacatt
tactctttattctatagggaagatgttatgaaagaaagccagaataaaacaaacaatagcgaatattattatttg
cataattgcaataccacagccataactcaaagttgtagtaagtcaagttttgaaccaattcccattagatactgt
gctccagcagggtatgctatgctaaaatgtaatgatgcaaattttacaggggcaggagaatgcacaaatgtaagt
gtagtccactgtacacatgggataatgcccatgatagcctcatggttacatttaaatggaacatatgaaaaggag
aaaacgaaggtgtatcatacaaatgtaacaaataatccaccactattagttaagttcaatgagacaatacacata
acctgtgaaagaacagggaacaatacaagaggtcaggtacagataggaccagggatgaccttctataacagagaa
aacatctttggagatactaggaaagccttttgctatgttaatgcaactgagtggcggagaactcttgagatggcc
aaaacagccctcagagaagccacaaaaaaacctaccctgaatatcacacggccaaaggggacccagaggtagag
aacttttggtttaattgtcagggagaattttctactgcaatctaacaaaatggattaacgagacctggcttaac
gagacggaccagaccaatcttgtagcaccctgtagactcagacaaatagttaaccattggggcattgtggctaaa
gccatctacctccctccaagaagaggaacagtaaaatgtgtgtcaaacatcacgggtttcctgatgacaaacgag
gaaacgggtgctaccttctccggtaaagtagaggattattggagggtggaattagcaaagtacaaaattgtagaa
attcagcccctatcagtggctcctaccacaggaaaaaggccagagattaaggccaatcatacaagatcaaaaagg
agtgtttggggatgtgccaacaaattggtgtgtcatagtactgtggtatggaatgacacttgggctgctaggaaa
ccctgttcaaacacatctggacctacagattatgattgcatttggcaaaacttaacatggcaacaatggagtgaa
ttagtagacaactctacagacacaatttacactttattagaggttgcacaaatacaacaggaaaagaataagaaa
gaattattagaactagataaatggagcactctctgggactggtttgatattacacaatggctgtggtatattaaa
ttggctataatcataatagcaggcttaataggacttagaattctaatgtttatagttaatgtgcttaggcaggtt
aggcagggttatatgcccttgttttcacagttcatacagaccataataatagtacatagaaacttagtgcttaag
tggagactatttagagattggcttgccaatgcttatgtgattgtaaagacttcttttataaggaacttagatagg
cttgcaaatttcacagcctggtggactgacatactaatagaaggagcagttaacattttagaggcattagaaac
atcccaaccagaatcagacaaggtttagaaatagctttaaattaaatgggaaacattttggcagatgggctggt
gctactgaggtcctacgcagattgcatgctgcacctgctcatgatgggcaggcctcaaaagaacttcatgaaaga
ggagggctcactaacaataccataggaacagaaaaagatgttgcccattactctcaagaccatacagaagaggaa
gttgggttcccagtcagaccagctgtaccaatgagacccatgacagaaaaattagcaacagatctgtcatggttc
ttaaaagaaaaggggggactggatgggttaatttactctacacgtagagcagctatcctggatacctggatgtat
aacacacaaggagtattcccagactggcagaattacacccctgggccaggagtaagatacccactctgcagaggg
tggttatttaagctagtaccagtaactccacctgaggacgatgaaaggaacatcttgctgcatccagctaactct

catggaagagaggacccagatggagacggcgagactctcatgtggcagtttgacaccagcctagcaagaagacac
atagccagagagagacatccggagtacttcaaataa
>#52 JQ866001 7542 nt
atgggtgcgagagcgtcagtattgaggggagagaagttagatcgatgggaatcggtaaggcttcgcccaggaggc
aagaaaaaatatcttataaagcatctagtgtgggccggcaatgaattacagcgattcgctttgaatcccggcctg
atggaaacagcagatggttgcagaaagatcattatgcaactctcaccatcagtagaaatagggtcaccagaaata
atctccttatttaacaccattgcagtgttacattgcatacatgaaggagaaaaaattcaggacactgaagcagca
gtaaaaattgtgaaagagaaactaaaaataacgagcagtgaaacaacaggaaatgaggaaaccgcaaaaggaaa
aaagagacagcagtgacatctagtggtcagacagaaaaaatctaccccacattgccatctagtggtcagacaggg
agaaactaccctattgtttttgatgctgcgcaacagccaaggcatcagccaatttcacccagaactcttaatgct
tgggtaaaaacagtagaggagaaaaatttaagccagaagtaatcccaatgttctcagccttagcagagggagcc
atcccacatgatcttaatacaatgctaaatgcaattggggaccatcaaggggcaatgcaagtgctgaaggaagtt
atcaatgaagaagcagctgattgggacaggttacacccaactcatgcagggccgctagtcccagggactctaaga
gagcccacagggagtgacattgcaggaacaacaagtaccattgtggagcagatacaatgggtaactactcctcaa
aatcaaggaggagttccagtaggagatatctataaaagatggatcatcctagggctgaataaattagtgaaaatg
tacagccctgtaagcattctagatataagacagggaccaaaagaaccattcagagattatgtagacaggttctac
aaaactatcagagcagaacaagcttcccagccagtaaaaaattggatgacagaaactttactggtacaaaatgct
aacccagattgtaagaccatactgaaagcattgggaccaggggcaaccttggaagaaatgctgactgcctgtcag
ggagtaggggggaccagcacataaggcaagggtcctagcagaagccatgcagcaaacacagcagaatgttaatatg
gtacaggggagagcagcgcctcaaggaaaaagaggggcaggaaacataaaatgttttaattgtggaaagatagggg
catacagcaagaaattgcagagccccaagaagaaagggatgttggaagtgtggacaggaaggtcatcagctgaag
gactgcacacagtcaaaaaagtggagtagaagtaacagtgcagggacaggtctgcacagccccttttagacacaggg
gctgatgatacagtatttgcagaccttaagttaaaaggatcatgcaaccctaggacaattggaggaatagggggga
tttgtccctgtacaagaatattacaatgtaccaattaccatagcagggaaaacagtgaaaacaaaagtactaata
ggaccaaccccagtaaatataattggaagaaatgttttaagacaacttggttgtactctaaatttccctattagt
acagtagaaatagtaaaagtgaagctcaaggaaggaatggatggcctaaggtaaaacaatggccactatccaga
gagaaaattgaagccctaacagaaatttgtgaaaatttaagagcagaagggaaaatcattgaagtaggacctgac
aatccttacaatactcccatctttgctattaaaaagaaagacagtacaaaatggagaaaattagtagattttaga
gagctcaataaaaggacacaagatttttgggaggtacaattaggaataccacacccagcagggttaaaaaagaga
aaagtcattactgtgttggatgttggggatgcttatttttcaatcccattagacccagaatttcaaaagtataca
gcatttaccataccaagtgtcaataatgaaaggcctggtaaaagatattgttacacagtgctaccacagggatgg
aaggggtctccagcaatcttccaatacagtatgacaaaaatttttagatccattcaggaagaagcatcctgaaata
gatgtttaccaatacatggatgacctgtatgtaggatctgatttgacagctgaaaaacataaagaagtggttcag
gagttaagaaaccacttgaaagcatggggcttagaaacccccgataaaaaatatcaagagaaaccaccatttctc
tggatgggatatgaattacacccagagaagtggacagtgcagcagataaaattaccagagccagaaacatggaca
gttaatgatattcagaaattagtgggtaaattaaattgggcaagtcaaatttatccagggatcagaacaagagaa
ctctgcaaattaattaggggagtcaaaggactaacagatgtagtacagttgactgcagaagcagaattagaatta
gaggaaaataaacaaattttaaaagaaacagtacaggggacctactatgacccaaaggaacctcttaaggcagct
gtacagaagcaaggaaaaggacaatggacttatcaagtctatcagaaagaaaatcaaatattaaaaacaggaaaa
tatgcaagaaaccatggtactcatactaatgaattaaaacaattagcaggggtagtgcaaaaaattgggacagaa
agcattataatatggggaagagtacctgtattccaacttccagttacaaaggaagtgtggcaacaatggtggtct
gactattggcaggtcagctggattccagaatgggagtttgttaacacccccccattaataaagttatggtacaat
ctgttaacagaacctatgccagaagcagagacctattatgtagatggagcagcaaacagggactctaaattaggg
aaagcaggctatgtgacagatagaggaaaaagcagggtaatagaattaacagaaactactaatcagcaggcagaa
ttacaggctatactgctggcattaaaagatgcaaaagaaaaggtcaacatagtgacagattcccaatatgtgcta
ggaatcttacaaggaactcctgaccaaagtgactccccccctagtagaggaaataatacaagaattaatgaataaa
gaagcagtttatttaaattgggtacctgctcacaaaggaataggagggaatgaagaagtagataaattagtaagc
aaaaacattagaaaagttcttttcctagaaggaatagatcaagcacaagaagatcatgaaagattccatagtaat
tggaaaatgttagctgatgaatataatttacccccccatagtagctaaggaaatcatagctcaatgcaataaatgt
cacaggaaaggagaagccatacatggacaagtggattgtagcccagaaatatggcaactagactgcacacacata
gaaggaaaagtggttctagtagcagtccatgtagccagtgggttcatagaagcagaagtgttatcaagtgaaaca
ggcaaggaaacagctttcttcattttaaaactagcaggaagatggccagtaaaaaagatacatacagataatgga
gccaattttactagtgctgcagtaaaggcagcatgctggtgggcaaacatccaacaggaatttggaattccctac
aatccacaaagtcaaggagtggtagaatccatgaataaacaattaaaaattattgtagaccaaattagagaacaa
gcagaacatttaaggacagcagtagctatggcagtgtacattcacaattttaaaagaaaaggggggattgggggg

tacacaccaggagatagaatattagacatattaacaacagacatacaaactaaagaattacaaaaacaaatttt
aaacttcaaaattttcgggtctattatagggactcaagagacccagtgtggaaaggaccagcccgtctcctgtgg
aaaggtgaaggggcagtagtcataaaggagggagaagaaatcaaagtagtaccaagaaggaaggctaagattata
aaggatgcttggaacagcctagtaaaacaccatatttataagagtaaacattgtaaagaatggaaatatagacat
cattatgatatagaccaccccagaaagggaggagaagtgcatatacccataactgagaaagcaaaattggtaata
acaatatattggggcctacactgtggggaaaaagcttggcatcttggacatggggcaagcatagaatggagacaa
gggagatactggacagaagtagacccagaaacagctgaccaaattatacatagtagatattttccttgttttaca
gaaagggccataagacaagctattttgggagaaaagatcctacagcattgccatttccctacaggacattctaag
gtagggagcctacagtttctagcctttaaaaaagtgctagaacagaacaagcagaagcctcatagacctccatta
ccctcagtaactgtcctggcagaggatacattggaagaaataaaaaatgaggcaatgagacattttccaagaaat
acactacaaaacataggcaactgggtgtttcagaactatggagattcatgggaaggagtacaagtgcttataact
ctcttacaaaaagccctgtttactcattatagatacgggtgtgcccatagcagaatagggataggaccaaggagg
ccttggttacacccaagctctacaccaagcacgccctgcaattcttgctattgtaaagtatgctgttatcactgc
caactttgctttaccaagaaggctctcggcatctcactaggaattggaacatattacaccacagtctattatggg
gtgccagtatggaaagaggcaacaccaacattgttctgtgcttcctcagcaaaggtagcaagcacacaaccacat
aatatttgggccacacataattgtgtgcctcttgatcctcagccatatgaaatacctattaacattactactgaa
tttaatatggaaactaattatatggtagaagagatgaaaaaagatttaatctctctgtttcaacagagtttgaaa
ccttgtgtaaagttgacaccctttgtgtaaaaatgaactgcagtgaattcacaagaaatagtacaacaggaaat
aatgcaacaaaaaataatacaagtctagaagataaaataaaaagcaatcttacaagtctagaaatgtatgaatgc
tttttaatcaaactacagaatttagagacaaaaagaagcagatatattcattgttctataaagaggatataatg
aaagacaaaaacgacagtactaatacctcttattatttaataaattgcaataccacagccataactcaggaatgt
gagaagtcttcatttgaaccagttcctattcagtactgtgctccaccaggatatgctatgctaaagtgtaaagat
gaaaatttcaggggaaaaggaaactgtacaacagtgaggattgtacattgtacacatagtatatttccaatgata
gctacagctttacatttaaatgggtctgtagaagaaggagaaacaaaggcatactatgtagaagccaaacataat
gcaccattattaataaaattcaataaagcaacggtctttaacataacttgtgtaagacccggcaacaacacaaga
ggacaggtacagatagggccaggcatgaccttctataatataagaaatgttattggagacactagaaaggcattt
tgctatgtaaacaaaacactatgggaaaatgctactgaggcagcaaaagtagcaataaacgaaactcttaaaaac
accacgttcaaaaacaccacctatacatttaggacagatggggatctaaagtggatctcaggaggagaccaagag
actcagactcattggttcaactgtcaaggagaattttctattgtgacctcaaaaattggacaaacaatggcatc
gagatcgatggaatgctgatagcaccatgtcgacttagacagatagtaaatcattggggaatagtatccaggga
atttatctccctcccagggagggacaggtcaaatgtgtatctagtattacaggattcataatgacagctgagacg
aatgacactgatggcactatcacacccaccttctctgcaaaggtggaagactactggaaagtagaaatgagtaga
tacaaagtagtggagatacagccattatcagtggcacccactaaaggaaaaagaccagtagttggtgacccagaa
gcaaaaagtagaagtaaaagaggtctatggggatgtgctaacaaattgatctgtcatagttcattagaatggaat
agctcttggttaaacaaaaccctgtgcggtaataagacaaacaattatgaaaacaattatgagtgcatatggtct
caatacacctggcaacagtgggatgaggaaataaggaacatctcagatgttatctaccttagtttagaaacagct
caggtgcaacaagaaagaaatcacaagcaactcctagaactagataaatggagctcattatggaactggtttgac
atcactaattggctgtggtatattaaaatagctataataattgtagccagcttagtaggcttaagaatagtaatg
tttatcataaatttagtggggaagcttaggcagggctattcacttttgtctccacagcagcattgcattgtttgc
atagatactttagcagagttcactggctggtggacagacggggttatagaagcactaagagtagcagtagacata
ataagacatataccaactagaatcaggcagggattagaaattgctctaaactaaatgggagctgtactaggaacc
agattcccagggatagacaacatgttgaacagaattagaaacacaccagcagcagatgcaccagggtcacaagat
ttgcataacagaggaggccttacaacaaacaccataggaacagaacatgatgtattacaacattcaggagaccat
acagaagaagcagtaggcttcccagtaagacctcaagtgccaatgagacctatgacagaaaaattggcagtagac
ctctcatggtttttaaaagaaaagggggggactggaagggctatattggtctccaaagagagcagcaatactagat
acctggatgtttaacactcaaggaatattccctgattggcagaattacacaccagggccaggagttagatatcct
ctgtgtagaggttggttatttaagttagtaccagcagacccaccagaagaggatgaaaggaatctactcatgcac
cctgcttgctcctatggaagagatgaccctaatggagaactgttggtatggaagtttgactctgaactggctaga
agacacatagccagagagagacatccggagtacttcaaataa
>#53 DQ373065 7389 nt
atgggtgcgagagcgtcagtattaacaggaggaaaattagatcaatgggaaaaaatttatttgagaccaggggga
aagaaaaaatacatgatgaaacacttagtatgggcaagcagggagctggaaagattcgcgtgtaacccaggtctc
atggacacagcagaaggttgtgcccaattactcagacaattagaaccagctctcaaaacagggtcagaaggactg
cgctctctatttaacaccctggcagttctttactgtgttcataataacataaaggtacagaatacacaggaagct
ttagaaaaattaagagagaaaatgaaagcagaacaaaaagaacctgaaccagaacaagcagcaggggcagcggca

gcacctgaaagcagtatcagtaggaactatcctctggtacaaaatgctcaagggcaaatggtacatcagccgctg
tcacccagaaccttaaatgcttgggtaaaagtggtagaagaaaaggcctttaatccagaagtaataccaatgttt
atggccttgtcagaaggggcaacgccccaagacttaaacaccatgttaaatacagtaggggacatcaggcagca
atgcagatgctgaaggaagttatcaatgaggaagcagcagagtgggacaggggacatccagtacatatggggcca
ataccaccaggacaagtaagggagccaagaggaagtgatatagcaggaacaactagtaccctggcggagcaagta
gcatggatgactgccaatcctcctgttccagtaggagatatttatagaagatggatagtcttgggattaaataag
attgtaagaatgtatagtcctgccagcattctagacatcaaacagggaccaaaagagacttttagagattacgta
gataggttttacaaaactctaagagcagagcaggccactcaggaagtaaagaattggatgacagagacactctta
gtacaaaatgcaaatcctgattgtaaaaatatcctgagagcattaggaccaggagcctccctggaggaaatgatg
acagcatgccaaggggtgggaggaccagctcataaggcaagagtactagcagaagccatgacacaggcacaaaca
gcaactagtgtttttatgcaaagaggaaattttaaaggcataagaaagaccatcaaatgctttaattgtggtaaa
gagggccatctagcaagaaactgtaaggcacctagaaaaagggctgctggaaatgtgggcaagagggacatcaa
atgaaggactgcagaagtggagaaaggcaggcgataggaggagaaataaaagaagctcttctagatacaggagct
gatgatacagtaatagaagaaatacagttggaaggaaaatggaaaccaaaaatgataggggaattggagggttt
atcaaagtaaaacagtatgataatgtgataatagagatacaagggaaaaaagcagttggcacagtcttagtaggg
ccaacacctgttaatattattggaagaaattttctgacacaaattggttgtactctaaattttccaataagtcct
attgaaaccataccagtgaaattgaagccaggtatggatggcccgagagtaaaacagtggcctttgacagaagaa
aaaataaaggcactaacagaaatttgtacagaaatggaaaaagaaggaaaaatttccagaatagggcctgaaaat
ccatataatactccaatttttgctataaaaaagaaagatagcactaaatggagaaaattagtagatttcagagag
ttaaacaaaagaactcaagattttgggaagtacaattaggaatcccacatccagcagggctaaagaagaaaaaa
tcagtaacagtgctagatgtaggagatgcttacttttcatgtcccttagatgaaaattttagaaagtatacagct
tttaccatacctagtgtaaataatgaaacacctggtattagataccagtacaatgtgctgccacaaggctggaaa
ggatcaccagcaatctttcagagcactatgacaaaaatattagaaccatttaggaagaacaatccagaactagtc
atatatcagtacatggatgatctctatgtaggctctgatctagaaataacacaacatagagaggcagtggaaaga
cttaggagtcatcttttgacttggggatttacaacccctgacaaaaagcatcagaaagaaccaccattttgtgg
atgggatatgaactccatccagataaatggacagtccagacaatacaattaccagagaaagacacatggactgtc
aatgatatacaacagttagtaggaaaattaaactgggctagccagatctacccagggattaaagtaaaacaactt
tgcaaattaattagaggagcaaaagctctaacagaagtagtcacacttacaagagaagcagaattagagttagct
gagaatagggagatattaaaagaacctgtacatggggcctattataacccagacaaagaattaatagcagagatc
cagaagcagggacaaggtcaatggacatatcagatttatcaagacttacataaaaatttaaagacaggaaaatat
gcaaaaatgaggtctactcatactaatgacataagacaattaactgaagtggtacaaaaggtggcactagaaagt
atagttatctggggaaaaactcctaaattcagattaccagtgcaaaaagaagtatgggagacctggtggacagag
tattggcaggcaacttggattcctgattgggagtttgtcaacacccctccactagtaaaattatggtatcagtta
gagacagaaccaattagcggggcagaaacctattatgtagatggagcagctaatagggaaactaaattagggaaa
gcaggatttgtgacagataggggtaggcaaaaggtgacctctatctcagaaactaccaaccaacaagctgaatta
caggctgtcctcatggccttacaagatgcaggacaagaagtaaatatagtcactgactctcagtatgtcctagga
ataattcactcacaaccagataaaagtgaatcagaattagtaaatcagataatagaagaacttataaaaaaggaa
agaatatatctttcctgggtacctgcacataaaggtattggaggaaatgaacagatagacaaattagtcagcaca
ggaatcaggaaagtcttattcctagatggtatagataaggctcaagaagaacatgaaagatatcatagtaattgg
aaggcaatggccagtgattttaatctacccccatagtagctaaagaaatagtagccagttgtgataagtgtcaa
ttgaaaggagaagccatacatgggcaaatcaactgtagtccaggggtatggcaattagattgcacacatttggag
ggaaaaattatccttgtagcagtccatgtggccagtggctacttagaagcagaagtcattcctgcagaaacagga
caggaaactgcatattttatcctaaagttagctggaagatggccagtaaaagtcatacatactgataatgggagc
aacttcaccagtgccactgtaaaagcagcctgttggtgggcaaatatccaacaggagtttggaataccctacaat
cctcaaagtcagggtgcagtagaatccatgaataaagaattaaagaaaattataggacaaattagagatcaagca
gaacatttaaaaacagcagtacaaatggcagtcttcattcacaattttaagagaaaggggggattggggggatac
actgcaggggaaagaataatagacataatagcaacagatatacaaacaacaaagctacaaacacaaattttaaaa
gttcaaaatttcgggtttattacagagacagcagagagcccacttggaaaggaccagccaaactactgtggaaa
ggagaaggggcagtggtaattcaagataacggggatataaaagtagtcccacgtagaaaggccaaaataattaga
gatttggtaaagcatcacatgtatgtgtcaaagaaggcacgaggttggttctatagacatcattatgaaacagat
aatccaaaaataagttcagaaatacacatcccattaggagaagcaaaattagtaatagttacctattggggatta
atgccaggagaaaggccatggcatttaggacatggagtatccatagaatggagacaaggaatatacaggacacaa
atagatcctgaattggcagacaagctaatacacctttattattttgattgttttacagcctctgccatcaggcaa
gcggtcttagggagaccagtaatacctaagtgtgaataccctgcagggcataaacaggtaggctccctacaatat
ctggcattaatagcctgggtgggagtacagaagagaagaccacccttacctagtgtgaccaagttaacagaggat

ttgttggaagacctaaagaatgaggctctgcgccactttcctcggccttggctacatggactagggcaatacttc
tataatacatatggagatacctgggagggagtagaggccatcattaggacactacaacaactgttgtttatacat
tataggattggctgtcaacatagcaggataggaatcactcctcaaaggagaaggttagagccctggaatcaccca
ggaagtcaacctaagacagcttgcaataattgccattgtaaagtttgttgctatcactgtgtgtattgcttcaca
aaaaaaggcttaggcatctcattgtgggtaacagtgtactatggggtacctgtctggagagatgcagagacagtt
ctattctgtgcttcagatgctaaggcccatagtacagaggctcacaatatttgggccacgcaagcatgcgtcccc
actgaccccaacccacaagaagtactaataccaaatgtgacagaacgttttgatatgtggaaaaacaatatggta
gatcaaatgcaagaagacattattagcttgtgggaacagagtttaaaaccctgtgttaagttaaccccattatgt
gtaaccctatcatgtagctcatggaggagtgtgaacaatagtgttaaccaaacaaaccacgtacaaatgcaaaat
tgctcatttaatgtgaccactgagttaagagataaaaagaagcaagtctactctctgttttatatggagatata
ataccettagacactaataatagcagtggtaataacagtcaatataggttaataaattgtaacaccacagctgtg
acacaggcctgtcctaaaatttcctttgagccaattccaatatattattgtgcaccaccaggatttgccattata
aaatgtaatgatcaagattttaatggaactggggaatgtaacaatgtaagtactgtacaatgtacccatggaata
aagccagtgatatccacacagttgatcctaaatggcagcttagcaacatcaaatatcgtaataagaaataattca
aaagacacactcttggtgcaattaaatgaaagtatcccaataaattgcacaagaccaggaaataagacaagagga
caagtacaaataggacctggtatgacattctataacatagaaaatataataggagacactaggcaagcatattgt
gaggtaaacaggacatgggaacaaatatggaatacaacaaaacaaataataataaacaacagaaagaatattaca
tttatacccaatccaggcggagacctagaagtaacaaacttaatgatcaattgtggaggagaattcttttactgt
aataccagtcaattgtttacaaatcaaaatggcaacacaactggcaatatcaccttacaatgcagaataagacaa
attgtaaatttatggacaagagtaggaaaaggaatttatgctccgccaattaaaggacctattaactgtctctct
aacatcactgggataatacttgactatacaaagtctggaactgagaagtacaccatatatcccacagggggagac
atgactaacctctggaggcaggagttgtataagtataaggtggtcagcatagaacccataggagtagcaccagga
aaagctaaaagacacacagtgactagacaaaaaagagcaggcctatggggctgctcaggaaaatctgtgtgttac
accaatgtgccttggaataccacttggagcaacaataactcctatgacacaatatggggaaatatgacatggcaa
aattgggatgagcaagtaagaaattattcaggtgtcatttttgggctgctagagcaagcacaagagcaacaaagc
ataaatgaaaagtcactcttggaattggatcaatggtcaagtttgtggaactggtttgacattacaaaatggctg
tggtacataaaaattttcataatggtagtagcaggcattgtaggcataagaataataagcataataatgtctatg
gtagcaagagttaggcagggatattctcccctctcgttgcaggaactcagaatatatttgtggggaataatagct
tactggggaagggagctaaaaattagtgctataaacttgcttgatactacagctgttgcagtagcagaaggaca
gatagaatcatagagctagtgcaaagaataggacggggaatactacacatacctaggagaatcagacaaggtcta
gaaagagcacttttataaatgggaaaagtttggtcaaagagtagccttgtggggtggccacaagtcatagataga
atgagaagacagcaagatccagcagaaggggtaggagcagtttctcaagatctagctaatcgaggggctattacc
ataagaaatactaaggagaataatcaagccctagcttggctagaagagcaaaaagaggaagcagtaggctttcca
gtatgtcctcaggtaccattaagaccaatgacatacaaagaagcttttgatctttccttctttttaagagaaaag
gggggactggaagggctagtttggtcaaccaagagacaagaaattctagacctctgggtttatcatactcaagga
atcttccctgactggcagaactacactccaggaccaggcgtcagatacccccctgacctttggatggtgcttcaag
ctagtaccactgtcacctgaggaggtagaagaagctaatcaaggagacaacaatgtcctgttacatcccatgtgc
caacatggaatggaagatccagacaaagaagtgctggtgtggcgctttgacagctccctagcaagagtacacaga
gcaagagagctgcatccggagttctaccagaactgctga
>#54 AF103818 7356 nt
atgggtgcgagagcgtcagttctgacaggggacgattagatgcttgggaaaagattcggcttagacctgggga
aagaaaaagtatatgatgaaacatttagtttgggcaagcagggagctggaaagatttgcttgtaacccaggtcta
atggaaacagcagacggctgtctccaattattaaaacagttagaaccagctctaaaaacaggatcggaaggcctg
cgatcgctctttaacaccttagcggtcctttggtgtgtccacagtagggtgacagtggaggatacgcagcaggcc
ttagtgaaactgaaagaggtagtgcagttgcagaaacaacaggaagaaaaagaacaacagcaacaggaggcaagt
ggcagtaacattggtagcagcaattacccagtaatacagaacgctcagggacagatggtacatcaggcaatgtct
cccagaactttaaatgcctgggtaaaagcggtagaagaaaaggcctttaacccggaagtcatcccgatgttcatg
gccttatcagaaggggcgactccccaagatgtcaacactatgctcaatgctataggggacatcagggagcaatg
caagttttaaaggaagccatcaatgaggaagcagcagattgagatagaacacatcctctgcatgcaggccctata
gcaccagggcagatgagagaacccaggggaagtgacattgcaggaaccaccagcaccttgcaagaacaagtgggc
tggatgacagcaaacccacccattccagtaggagacatatatagaagatgggtggtcctagggcttaataaagta
gtaaaaatgtactgtcccgttggtatcttagatatcaagcagggacctaaagagccctttagagactatgtagac
aggttctacaaaacattaagagcagaacaagcttctccagaagtaaaaacctggatgacagagacccttttggta
caaaacgctaatccagattgtaaaactattttaagagccttgggacctggagccaccctagaagaaatggtgaca
gcatgtcaaggagtgggaggacccgctcataaggcaagggtcttagctgaagccatgtgccaaatgaaaaacccc

tcaagcgtatttctacagaaaggaaacgctgggaagccagggagaaaaatcaaatgttttaattgtggcaaagag
ggacatctagccagaaattgcagggctccaagaagaaaagggtgctggaagtgtgggcaggaaggacatcaaatg
aaagactgcacagcaggaaacagacaggctgtagcagggcagatagtagaagctcttttagacacaggagctgat
gatacagtactagacaacatacaaattgaagggacatggagaccaaaaatgatgggggaattggaggttttata
aaagtaaaacaatatgatcacgtcaatatagaaatagagggaagaaaagcacagggttcagttttagtgggacca
acaccagtaaatattattggcaggaatattttaacacaaattggatgtactttaaattttcctatcagctctatt
aaaactgtaccagtaagattaaaaccaggaatggatggccctagagtaaagcaatggccattgacagcagaaaaa
attaaggcattaacagaaatttgccaagaaatggaaaaagaaggaaagattacaagagtagggccagaaaatcct
tacaatacaccaattttgctatcaaaaagaaagatagcactaaatggagaaaattagtagactttagagaatta
aataaaaggacacaagacttctgggaagttcagttaggcataccacacccagcaggattaaagaagaaaagatca
gtaacagtcctagatgtaggggatgcctatttttcttgtccactggataaggaatttagaaaatacacagcattt
accatccctagtgtaaacaatgagaccccaggaattagataccagtataatgtgttaccacaaggatggaaaggg
tcaccagcaattttccagagcagtatgacaaaaattctagatccttttagaaaacaacatccagatgttataatc
tatcaatatatggatgatctctatgtagggtcagatctaaacttagaaaagcatagggaaaaggtagaactgctc
agacaatatttgcttacttggggattcactacccctgataagaagcatcaggaggaaccaccattcttatggatg
ggctatgaactccatccagataagtggacagtgcagcccatacagttaccacaaaaagaaatttggacagtcaat
gatattcagaaattagtagggaaactgaactgggcaagtcagatatatccaggaataaaaataaagcagttatgc
aaattaataaaaggagctaaagctctaactgaagttgtaaattttacacatgaggcagaaatggagttagaagaa
aacagagaaattctaaaggaaccagtacatggggtctattatgacccagaaaaagaattagtagcagaagtacaa
aaacaaggaaggagtcagtggacataccaaattttcaagaacggcataagaatctaaagacaggaaaatatgcc
agacaaagatcagcacatactaatgatatcagacagctagttgaagtggtgcaaaaaatagctactgagagcatt
gtcatttggggaaaggtaccaaaatttaaattgctagtgcagaaggaagtctgggaaacatggtggtcagaatat
tggcaggccacctggattccagattgggaatttgtcaatacccctcccctcgtaaaactttggtataagttagag
acagaggccatagagggggcagaaacattttatgtggatggagcagcccaacgagaaaccaagaaaggaaaggca
ggatatgttactgataggggtagacaaaaaattataactttagaaaatactactaatcaaaaggcagagctcaca
gcggtatacttagcattaaaagattcagagaatacagttaatgtagtcactggctcccaatatgtcttaggaatc
atccactctcagccagaccaaagtgaatcagaattggtcaatcagataatagaagaattaataaagaaagaaaaa
agttatatctcatgggtaccagcacataaaggaattggaggaaatgaacaaatagataaactagtcagttcaggg
atcagaaaagttcttttcctagatggcatagataaagcacaggaagaccatgacaaatatcatagtaattggaca
gctatggccagtgattttaacctgccaccagtggtcgctaaggagatagtggccagctgtgataaatgtcagcca
aaaggagaggccatacatgggcaggtagattgcagtccaggtatctggcagctagattgtacacatttagaaggg
aaaatcattatagtggcagtacatgtggccagtggatacctagaagcagaggtcattcctgcagaaacaggacaa
gagacagcctattttatcttaaaattagcaggaagatggcctgtaaaagtgattcatactgataatggatctaac
tttacaagtagtacagttagagcagcttgctggtgggcaggcatacaacaagagtttggaattccatacaatcca
caaagtcaaggagtggtagaatccatgaataaagaattaaagaaaatcataggacaaatcagggatcaagcagag
catttaaagacagctgtacagatggcagtattcattcacaattttaaaagaaaaggggggattgggggtatact
gcaggagaaagaatcatagacatcatagcatcagaactacaaacagacttattacaaaaacaaattttaaaagtt
caaaatttcgggtctattacagggacagcagagatccaatttggaaaggaccagccaaacttctgtggaaaggt
gaaggggcagtagtaatcaaggaaaacgaggaggttaaagtagtacccagaagaaaagcaaaaattataaaagac
gcttggaatagtctggtaaaatatcacatgtatagatcaaagaaagctaaaggatggttctacaggcatcattat
gagagcactaatcccagagtaaattcagaagtacatatccctatagataaagcaaaaatagtgattataacgtat
tggggttgaaaacaggagaacaagcctggcacttagggcatggagtatcaatagaatggagacaaggaacttac
aagacacaaatagatcctgagatggcagacaagctaattcatctgtattattttgattgttttacagcctctgcc
atcagaaaagctgttttagggagaccagtaattcccaaatgtgaataccctacagggcacaataaggtaggctct
ttgcaattcttggctttaaaggccttagtaggacagagcaaaagaagaccacccttacctagtgtgactaaattg
acagaagatgtcttagaagaactaaaagaagaggctttgcgccattttcctagaccctggctacaaggcttagga
cagtacatctacagtacttatggagacacttgggagggagtagaagcaattataaggatcttacaacaacttttg
tttatccattataggattggctgtaatcatagcaggatagggatttccttggcaaggagaactcctcaagggaga
aggatagagccctggaagcagccaggaagccagcctaagactgcttgcaataattgctactgtaaaaagtgttgc
tttcactgtgtggtttgttttacgaagaagggcttaggaatttccttatgggctacagtatattatggggtacct
gtatggagagatgtagagacaaccttattctgtgcctctgatgcaaaggcatacaagcaggaggcccacaacatt
tgggccacacaggcatgtgtccctactgaccccaatccacaagaagtacatttgccaaatgtgactgaaaagttt
gacatgtgggaaaataatatggcagaacaaatgcaggaagatattattagcctatgggaccagagcttaaaacct
tgtataaagttaaccccattgtgtgttactatgacttgtcttaaccccgatagtaatagtagtgctgtaaatact
actgatataatgagaaactgttcttttaatataactactgaattaagagacaaaaagaaacaagtgtattcctta

ttttatgtagatgatctagctcatatcaataataatacctatagactgataaattgcaacaccaccgctatcaca
caggcttgtcctaagacctcctttgagccaattccaatacactattgtgcaccaccaggctttgccatcctaaaa
tgtaatgaaaaagatttcaaaggaaagggagagtgtaaaaatgttagtacagtgcagtgtactcatggcataaaa
ccagtggtgactacacagctcataataaatggcagtttagcaactaaaaatgttactgtaagaagtaaaaacttt
gcagacattattctagtacaattctcagagggagtcaatatgacttgtattagaccaggaaacaatacagtggga
aatgtacaactaggaccaggaatgacttttataacataccaaagatagtaggagatgtaagagaagcacattgt
aacatctcaaaactgacatgggagaaacagagaaaatacactttagagataataaaaaggaagcaaacctgaca
aaggtagagttaattccaaatgcaggaggagacccagaagtggtaaatatgatgcttaattgtggaggagaattt
ttttattgtaatacaattcccctatttaacatgacctacaacaataccgacaacaccactatcacacttaagtgt
agaataagacaaattgtaaatcagtggatgagagtaggaaaaggaatctttgccccaccaatcaaaggtgtgcta
agttgtaactcaaatataacgggaatgattcttgacataagcataagcgcagtcaataacgatagtaggaatata
acagtgatgcctacaggaggagatatgacggctttatggaaaaatgaattacataagtataaggtggtcagcata
gaacctataggagtggcaccaggtaaggccaaaaggcatacagtgaaaagagaaaaaagagcaggtctgtggggc
tgctcaggaaaaacaatttgttataccactgtgccttggaatgatacctggagtaacaacctctcctatgatgct
atttggggcaatctaacttggcaagaatgggacagaaaagtaagaaactattcaggtactatttttagtcttata
gaacaagcacaagaacagcagaatacaaatgaaaaatcactcttggaattggatcaatggtcaagtctatggaac
tggtttgatattaccaactggctgtggtatataaaaatatttttaatagtagtagcaagcttagtaggaatcaga
attgtaggtgtgatattttcactagtagcaaaagttaggcagggttattctcccctctcgttacaggctacaagc
ttattagatacaacagcaatagcagtagctgaaggaactgatagaattatagaattaacaagaaggctctttcta
ggtattatacacataccaagaagaattaggcaaggcctagagagaagcttattaggtaacaagtggtctaagagt
agcatagtaggatggccagaagtcagaaacagattgagacaaactcagacaacagcagcagcagaaggagtagga
cctgtctctcaagacttagcagaacatggggctatcactactagaaatacaccacagaacaaccaaactctggca
tggttagatgagatgactaatcatgaaagtgaagtaggcttccctgttcgccctcaagtaccattaaggcccatg
acttacaaacaagcattcgatttaggcttcttttaaaagaaaagggggactggaagggttagtttattccaga
agaagacaagagatcctagatctctgggtttaccatacacaaggaatcttccctgactggcagaattacacccca
gggcctggagtaagatatcccctaacatatgggtggtgctttaagttagtccctctcacagaagaggaagtagag
caggccaataaaggagagaccaacatactgctgcaccccatgtgccagcatggaatggaagatgaacatggcgaa
gtcttaatctggcagtttgacactgaactggctcggagacacagagctaaagagctgcatccagagtacttccgg
aactga
>#55 EF535993 7371 nt
atgggtgcgagagcgtcaatattaacgggggacgattagatgcttgggaaaaaattaggctaaggccaggggga
aagaaaaaatatatgatgaaacatttagtctgggcaagcagggagctggaaagattcgcttgtaaccctggtcta
atggaaacaggagagggatgtgaacagctattaaaacagttagaaccagctctcaaaacaggatcagaaggcctg
cgatcgctttttaacactctagcggtactttggtgtgttcataaaagaattccagtggaagacacacagcaggct
ttaactaaactgaaagaaacagcggcccgrgaacaggaagtggcccaaccacagccgcagcaagacagcaatgtc
agtagaaacttcccagtagtacagaatgcacaagggcaattgatgcatcagcccatgtcacctagaactttaaat
gcttgggttaaggtagtggaagaaaagaattttaatccagaagtgataccaatgtttatggccctgtcagaggga
gcgactccccaggatgtcaatactatgcttaatgctataggagggcaccagggagctatgcaggtgttaaaggaa
gtcatcaatgaggaagcactggaatgggacaggacccatcctgtgcatgcaggtccaatagcaccaggacaactg
agagaggcacgaggaagtgacatagctggaaccactagcactctagcagaacagattgcttggatgacagcaaat
ccacccgttccagtaggagaaatctatagaagatgggtggttctagggcttaacaaagtggtaagaatgtatagc
ccagtcagtatcctagacattaagcagggacctaaggagcctttcagggactatgtagatagattctacaaaacc
ctgagggcagaacaggcaacacaggaggtaaaaacctggatgactgataccctcytggtacaaaatgctaaccca
gactgtaaaaatatcttgagggccttaggaccaggagccaccttagaagagatgatgacagcatgccaaggagtg
ggaggaccagcccataaggctagggttttggctgaagctatgagccaagtacaaaactccacaagtgtgtttttg
cagaggggcaataatgggagaccaactaggaaaatcaaatgctttaattgtggcaaagaagggcatttagcaaga
aactgcagggccccaagaaggaagggggtgctggaaatgtggacaagaggggcatcagataaaagattgtacagct
ggagacagacaggctatagaggggcagctgatagaagctctgctagacacaggagctgatgatacagtgatagat
aatgtacaattaacaggaagatggaaaccaaaaatgatagggggaattggaggttttatcaaagtaaaacaatat
gataacatagcaatagaaattgaaggcagaaaagcaacaggcacagtgttggtgggaccaacaccagtgaatata
attggcagaaatatattgacacagattggctgtactttaaatttccctataagtcccattggaactgtgccagta
aaattaaagccaggaatggatggccctagagtaaaacaatggcccttaacagaagagaaaatcaaagctttaaca
gaaatttgtcaggaaatggaaaaagaaggaaagatttcaaaaatagggccagaaaatccttataatacaccaatt
tttgccataaaaaagaaagatggtgaaaaatggagaaaattggtagattttagagaattaaatagaagaacacag
gacttctgggaagtccaattaggcataccacacccggcaggattaaaaaagaaaaagtcagtaacagtactagat

gtgggagacgcttattttcctgcccactagatgaaagttttagaaaatacacagcatttaccatacctagtgta
aacaatgagacaccaggaattagatatcaatataatgtgctaccacaaggatggaaagggtcaccagcaattttt
caaagcagcatgacaaaaatcttagaaccatttagacaacagaatccagagttgacaatttaccagtacatggat
gatctttatgtaggctctgayctagaaataggagaacatagaaaaaaggtagagttacttagacaacatctactc
acttggggttttactactcctgataagaagcatcaaaaagagccacctttttttgtggatgggctatgagcttcat
ccagataaatggacagtgcaaccaatacagttaccacaaaaagagacctggactgtcaatgatatccaaaagctg
gtaggaaaattaaattgggcaagtcaaatatatccagggataaaagtaaaacagctatgcaagctcataaaagga
gcaaaagctttaactgaagtagtggccatgacacaggaagcagaaatggaattggaagaaaatagagaaatttta
aaagacccagtgcatggagtgtactatgacccagagaaggagttaatagcagaagtacaaaaacagggaaacagt
cagtggacttatcaaatttttcaggaacagcataagaacttgaaaacagggaagtatgctaggcaaagatcagca
catactaatgacataagacaattagcagaggtagtacaaaaagtagcaactgaaagtatagtaatctggggaaaa
acaccaaaattcagattacctgttcaaaaggaggtctgggaaacctggtggtcagaatactggcaagccacctgg
atcccagactgggagtttgtaaatacccctcccctggtaaaattatggtaccarttagaaacagaacccatacca
ggggcagaaacattctatgtagatggggcagccaacagagacacaaagaaaggaaaagcaggttatgtcacggat
agaggtagacagaaaatagtcaatctagaagataccactaatcagaaagcagagctgacagctgtttatctagcc
ctacaagatgcagaacatatagttaacatagtcactgattcccaatatgtgttaggaataattcactctcagcca
gatcaaagtgagtcagaattggttaatttaataatagaagagttaataaaaaaggaaaaaagctacctctcatgg
gtgccagcacacaaaggaattggaggaaatgaacaggtagataaattagtcagctctggaattaggagagttctc
ttcttagatggtatagataaggcacaagaagaacatgaaaaatatcataataattggagagctatggcaagtgac
ttcaacctcccacctgtagtggccaaagaaatagtagccagttgtgacaagtgccaattaaaaggagaagccatg
catgggcaagtagactgcagcccaggaatgtggcaattagactgcacacatctagaagggaaagtcatcctagtg
gcagtacatgtagccagtggatacctagaggcagaggtcatccctgcagaaactggacaggaaacagcttatttt
attctaaaattagcaggaagatggccagtaaaagtaattcacactgataatggatctaattttaccagtaataca
gttaaggcagcctgttggtgggcaggcatccaacaggaatttggaattccttataacccacaaagccaaggagta
gtagaatcaatgaacaaagaattaaagaagatcataggacaaattagagaacaagcagagcatttaaagacagca
gttcaaatggcagtgttcattcacaattttaaaagaaaaggggggattggggggtacactgcaggagaaaggatt
atagacattatagcaacagacatacaaacaactaatttacaaaaacaaattttaaaagttcaaaattttcgggtt
tattacagggacagcagagatccaatttggaaaggacctgccagacttctgtggaaaggtgaaggggcagtagta
atcaaagataaagaggaagttaaagtagtgcccaggaggaaagcaaaaataatcagagatctagtaaaataccat
atgtacagaacaaagaaagcaagaaaatggttttataggcaccattatgaaagttctaaccccagagtaagttca
gaaatacatatccctttagaaaaagcaaaattagtggtcaccacctattggggattaatgccaggagagcatgaa
tggcacctaggacatggagtgtccatagaatggagatatgggtcatacagaacacaaatagatcctgaaatggca
gatagactaattcaccttcattattttgattgttttgcagcttctgccatcagaaaagctgttttagggacacaa
gtagttcctaagtgtgagtacccagcagggcaccaacaggtagggtccctacaatatttagccttaaaggcctgg
gtaggagtagaaaagaaaagaccacccttgcctagtgtcgccaagttagcagaagatctattggaggaactgaaa
aatgaggctgtacgacactttcctcgagcatggttacatggattaggacaacacatatatgacacatatggagat
acatgggcaggtgtagaagctatcattagaatactgcaacagctcttgtttattcattataggattggctgtcaa
catagcagaatagggattaaccctgggaggaggagactagagccctggcaacatccaggaagccaacctagaact
gcttgcaataattgctattgtaaatgttgctgctttcattgcatggtctgttttgagaaaaaaggcttaggaatc
tccgatcagttgtgggcaacagtgtactatggagtacctgtgtggaaagatgcggagacaacactcttttgtgca
gcagatgcttcagcacttaacaaagaagcccacaatatttgggcctcgcaagcctgtgttcccacagaccctaac
ccacaagaagtgcatgtaccaaatataactgaaacctttgacatgtgggaaaataacatggtagaacaaatgcag
actgatattattagtctatgggatcaaagtctaaaaccatgtgttaagttgacaccactatgtgtcactatgcat
tgtattccatacaacataagcagcactgggaatgacactaggaatagctctacaagccagatggtaaaaaaatgc
ttctttaatatgactactgaattaagagataaacagaagcaagtatattctttattttatgtagatgatatagta
smkattaatggtamtaaktcctataggctaataaactgtaataccacagctattacacaagcatgccctaaaacc
tcctttgagccaattccaatacattattgtgcaccccccaggttttgcaatcatgaaatgcaatgagccagatttt
aatggaacaggaaaatgtagaaatataagtacagtacaatgtactcatggcatcaagccagtggtaactacacar
ttaattcttaatggaagtctagctgagaacagcacgaytgytaggactgaasacatgamaraaatggatragacc
atcatagtacagtttcataaaagggtccagatgacatgtataagaccaggaaataacagcagaggacagatacaa
ataggaccagcaatgtcattttataacctagaaaatattataggagataccagacaagcatattgtaatrtcagc
gccgcagaatggaacaaaggttmaawgacactrtgagggctataaaaagtcttaagccaggcagtaacatcacc
ttcacagatcaacaaggaggagacccggaaatagtaaacatgatgttcaattgtgggggagagtttttttattgt
aacaccaccccattgttcaataacagttggacaaatacaactagctcagatataaatagctcaaatataataata
aactgtagaataaggcaaattgtaaactcctggatgagagtgggcaagggaatttacgccccaccaattagagga

actataagttgcacatcaaatattactgggctcctgctcgagttagacagaggactcccgagaaatggactaat
accaataacaagttaacactataccctacaggaggagagatgagagatttgtggaggctggaattacataaatac
aaagtagtcagcatagaacccataggagtggcaccaagtaaagctaaaaggcacacagtgacaagagaaaaaaga
gcaggtctgtggggctgctcaggaaaatccatctgttatactactgtgccttggaatagtacttggggtgctaat
acctcctatgatgaaatctggaataacctaacatggcaagactgggacaagagagtaaaaaattattcaggagtc
atctttgaccttatagagcaagcacaagagcaacaraacacaaatgagagaagcttattggaattggatcaatgg
tcaagtttgtggaattggtttgatatttctaattggctgtggtacataaaaatatttctaatggcagtagcaggc
ttaataggcataagaatagtaggagttatcatgtccttaatagcaaaggttaggcagggatattctcccctctcg
ttgcaggctacaagcttattagatacaacagcaattgcagtagctgaaggaacagatagaattatagaagttgtg
caaagaataggtagaggtattctacatataccaagacgcattagacaaggcttagagagaagcttgctataaatg
ggcaacaaatggtcaaaaagcagcctggtaggatggccagaagtaagagctagaataagacaagctcaaccagct
gcaccaggggtaggatcagtctcacgagatttagaggcacatggagcaataaccactagaaataccccacaaaac
aatcaaactctagcatggctagaagaaatgcagggacatgaagaggaggtaggctttccagttagaccacaagta
ccccttagacctatgacatttaaaggagcttttgatttaggctactttttaaaagaaaaggggggactggaaggg
ctaatttactccagaagaagacaagagatcttggatctctgggtctatcatacacaaggattcttccctgattgg
cagaattacacaccaggaccaggagtcagatatccactaacatttgggtggtgctttaaactggttccactgaca
ccagaagaagtggaaaaagccaatgaaggagataccaacatactgcttcaccccatctgccagcatgaaatagaa
gatgaacatggagaagtcttggtttggagatatgacagcaggctagctttgagacacattgctagagaacaacat
ccggagtactaccaggactga
>#56 AY169968 7425 nt
atgggtgcgagagcgtcagtgttaacaggggggaaattggatcgctgggaaaaagttcggcttagacccgggga
aggaaaaaatatatgatgaaacatttagtatgggcaagcagagagctggaaagattcgcatgtaaccccgggcta
atggaaagtgcagaaggatgtcttaaattgctacagcagttagagccagctctgaaaccggctcggaagggctg
cgatctttatttaacactctagcagtgttatggtgtgttcacagtaacatcgaggtagaagacacacagaaagca
ttagaacagcttaagaaacaccatggagaacaagtaagaaaaaatgagaacacagaaaacaatgaaagcagacag
ggcgctagtgcttcctctggcactagtgggaattaccctatagtacagaatgctcaagggcagatggtacatact
cccatctccccctagaaccttaaatgcatgggtaaaagtagtggaagagaaggcttttaatcctgaggtaatacca
atgttttcagctctgtcagaaggagctctacctttggatgttaacacaatgctgaatgctgtaggggggacatcag
ggagccatgcaagttctaagagaggtcatcaatgaagaagctgcagaatgggatcggatgcatcctgttcatgcc
ggccccatagctccaggacagctaagagaaccacggggtagtgatatagcagggactaccagcacattgcaggag
caaattggatgggcaacagcaaacccacccatcccagtaggagacatctatagaagatgggtaattctagggcta
aacagagtagtaaaaatgtattgtccagtaagcatccttgatataaagcaaggaccaaaagagccctttaaagat
tatgtagacaggttttataaaaccatgagagctgagcaagcttctcaggaagtgaaaacttggatgacagacacc
cttttggtgcaaaatgctaacccagattgcaagcaaattctaaaggccctgggtccgggggcctccctagaagaa
atgatgacagcctgtcaaggggtaggaggaccagcccataaggcaagagtcttagctgaagctatgacaatggtc
caaagtcaagcaagaacagacattttctttcagaaagggccaggggcaaccccaagaagaaaaataaaatgctat
aattgtggaaaggaaggccatctggccagaaactgtaaggcaccaaggagaaagggctgctggaaatgtgggcag
gagggacaccaaatgaaagactgttcagggagacaagtaatagaagggcaattatgtgaagccctgttagacaca
ggggctgatgatacagtaatacaagacattcaattacagggaatttggaaaccaaaaatgatagggggaattgga
ggctttattaaagtgaaacaatatgataatgtatcaatagagatagaggggaaaaaagtgcaaggacagtgttg
gtgggaccaacacctgtcaatataattgggagaaatattttaacacagatagggtgcacactggtattccctatc
agttcaattgaaactgtcccagtcaaattgaaaccaggaatggacggccaagaataaagcaatggcctttatca
gcagaaaaaattaaagccttgacagaaatatgtcaggaaatggaaaaggaagggaagatatctaaaataggacct
gaaaacccatacaatactcctattttgcaattaaaaagaaagatagttcaaaatggagaaaattagtagatttc
agagaattaaataaaagaacccaagatttctgggaagtacaattgggcataccccacccagcagggttaaaagag
aagaaatcagtaacagtactagatgtgggtgatgcatatttctcttgtcctctagataaggacttcaggaagtat
acagcattcacaattcctagtgtaaataatgaaactccagggatcagatatcagtataatgttttaccacaagga
tggaaagggtctccagctatctttcaaagtagtatgacaaaaattctggaacccttcagagcaaaaaaccctgat
atcactgtctaccaatacatggatgacttatatgtagggtctgatcttgacattacccaacacagacagaaggta
gaagagttaaggcaacatttgcttcactggggggtttacaaccccagataaaaagcatcaaaaagaaccacccttc
ttatggatggggtatgagcttcacccagataaatggacagtgcagccaataaagctaccagaaaaggaggtatgg
acagtcaatgatcttcagaaactcataggaaaattaaactgggcaagtcagatttatccaggaataaaaataaaa
cagttgtgcaaattaataagaggaacaaaaaggttaacagatgtagttcctctcacaccagaagcagaattagaa
ttagcagaaaacagagaaatagttagtacaccagtacatggggtttattataatccagataaggagctcatagca
gaagtacaaaaacagggagaagggcagtggacatatcagatctttcaggaacaacatagaaatttaaagacagga

aaatatgccagacagagatcaacacatacaaatgatattagacaattggcagaagtcatacaaaaaattgctgtg
gaaagtatagtcatttggggaaaaacacctagatttagattaccagtacaaaaagaaagttgggagacatggtgg
gcagagtactggcaggccacctggatccccgagtgggagttcattaacacccccccattagtaaaattatggtat
agtttagagacagaccctataccaacggcagacacatactatgtagatggagcagcaaatagggaaacaaagaaa
ggaaaagcaggatacataacagacaagggaaaacaaaaaataatcagtctagagaacaccactaatcaacaggca
gagttaggggccttgtatctagctttacaagactctgaacaacaagtgaacatagtaactgactctcaatatgtg
ttagggattattcagtcacaaccagatcacagtgaatcagaattagtaaatcaaataatagaagaattaattaaa
aaggacaaggtttacctttcctgggtacctgcacataagggcataggaggaaatgaacaagtagataagttagtc
agttctggaatcaggaaagtactattcctggatggaatagataaagcacaggaagaacatgaaagatatcatagc
aattggaaagctttggcgggtgaatttaacttaccacccatagtagcaaaagagatagtggctaactgtgataaa
tgtcaggtaaaaggagaggctctgcatgggcaggttgattgtagcccaggaacatggcagatggattgcacacat
ttagaaggcaaaatcatcatagtagcagttcatatagccagtggatacatagaagcagaagttatcccagcagaa
acaggacaagaaactgcatacttcatattaaaattagcaagcagatggccagtgaaagtcttacacacagataat
gggacaaattttacaagtaatgcagttaaagcagcatgttggtgggccaacatcaaacaaaagtttggaattccc
tacaacccacaaagtcaaggagtggtagaatccatgaacaaagaattaaagaaaatcattgaacaagttagagat
caggcagaacatctaaaaacagcagtacaaatggcagtgttcagtcacaattttaagagaaaaggggggattggg
gggtacactgcaggggaaagaataatagacataatagcaacagacatacaaacaaaagaactacaaaaacaaatt
ttaaaaattcaaaaatttcgggtttattacagagacagcagagacccgatttggaaaggaccggccaccctgctg
tggaaaggtgaaggggcagtagtaattcaggatcaaggagaaataaaagtagtacctagaaggaaagcaaaaata
attagagatgcatggaatagcctagttaaatatcatatgtataaaacaaagaaagcaaaagattggttctacaga
caccactatgaacatcctaatccaagagtttcttcagaggtacacattccttttaaagataattcaaaattaata
atcacaacctattgggctttaaccacaggagaaagagcgtggcatttaggtcatggagtctccatacagtggagg
atgggatcatatgttacacaggtagatccttatgtagcagataaattgatccactctcaatattttgattgtttt
gcagactctgccatcagaagagctatattagggcatattgtagaaccccggtgtgcttacagagaaggacacagg
caggtaggcaccctacaattcctagctttaaaagctttagtaaaagaaaaaaggcttagaccacccttacccagt
gtagctaaattaacagaggatatattagaagacttaaagaatgaagctgtaaaacactatcccagaagttggctt
caccaattaggccagcatatttataccacctatggggacacgtgggaaggggtggaggcaattataagaatccta
caacaactactgtttgttcattttagagttgggtgccaccatagcagaataggtataataccacaaaggagaaga
gcattggagccatggaagcatccaggcagccagccaaaaacagcttgtaataattgctattgtaaagcttgttgt
ttccattgtatgtattgcttcacaaagaaaggcttaggaatttccgaatgggtaacagtgtattatggagtacct
gtgtggagagaagtcaacacagtactcttctgtgcttcagatgctaaagcacatagcacagaggctcataacata
tgggccacacacgcatgtgtacctacagaccctaatcctcaagaagtagttcttcataatgtaacagaggacttt
aatatgtggaataatcagatggtagaacagatgcaagaagacatcagtagtctctgggaccagagtcttaaacca
tgtgtaaagctaactcctttatgtgtaacaatgaaatgtagtaacgttactagacaaaatagcactagcaactct
aacaataaccctaccctcaagaacaatacttcaggtaagaatgaaactggagttttacaaatgaaaaactgtaca
tttaacacaacaacagaattgagagataaaaagaaacaggtctattcattattttatgtagatgatctgcaaagc
ctaggaagtggaactggtgatacatatacaatgattaattgtaacaccacagccataacacaggcatgtccaaaa
gtgtcctttgagcctattccaatacactattgtgcaccagcaggatttgcaattctcaaatgtaatgatgtaaac
ttttcagggaaaggaaaatgtagaaatgtaagcacagtccattgtacacatggaatcaaaccagtagtaacaacc
caattgatcattaatgggagtctagctacagaaaacataacagtaagagtaaacaatgcctctaaaaatacccat
gattggatagtacaactaagcacagcagtgaatctgacgtgtaagagggtagggaataacacaagaggcaaagta
cagataggaccaggaatgaccttctataatatggaccatatatttggtgatacaaggaaagccttttgcgaactc
aatgggacaacatggaatgaaacactgcaaaaagttagagaaagcttaataaaggaaataaaagcaaatgcaaat
ggaacctacaatataacgtttgagccaagcagtggaggtgacccagaaatagcaaatcacatgttcaactgtgga
ggagaattcttttactgcgatactagaaaaatgtttaatgaaagtgaaccttttcacgagaacatgacaatacca
tgcagaattagacaaatagtgaattcatggatgagagtaggaaggggaatctatgcacctccaataccaggacat
ataacttgtaactctctaattactgggctcatattgacacgagaccatgttaataatactaataatacatttaga
ccaataggaggagacatgaaaaacatttggaggagtgaactctataaatacaaagtggtgcgcatagaaccatta
tctgtggcaccaaccaaagcaaaaaggcatacagtaggagaaaggagacagaagagagcaggcctttggggctgc
tctggaaagtctatctgttataccactgtgccttggaacaaaacatggtcaggcaagtctatgagtgacatttgg
aacaatttaacatggcaacaatgggacaaattgatcactaattacacagggacaattttggtttattagaagag
gcacaatcacaacaagagaaaaatgaaaaggacttattggagttggatcaatgggcatctttgtggaactggttt
gacataaccaactggctgtggtacataaaaattttccttatggcagtaggagggattataggtttaagaattatc
atgtcagttgtctcagtgatcaggagagtcaggcagggttactcacccctctcgttgcaggctacaagcttatta
gatgcaacagctattgcagtaggagaaggcacagatagaatcataagagcagtgcaaatagtattcagaatcata

ggcaacataccccggcgcataagacaaggcctagagagaactttactttaaatgggagctcagtggtctaaaagt
agcctagtaggatggcctgaaatcagaaaaagaataaaagaggctccaatagcagcagaaggagtaggagaggtt
tctaaggatctagaaaaatatggagctatcactagtagacatacaccaagtactaatcaaactttagcatggtta
gatgaaatgaaacatcatgaagaagaagtgggctttccagttagaccacaggtgccattaagaccaatgacttac
aaaggagcctttgacctttcccactttttaagaggaaaggggggactggaagggttagtttactcccagaaaagg
caagaaatcttagatctctgggtttatcatacacaaggcttcttccctgactggcagaactacaccccaggacca
ggagagagatacccaccctgctttggatggtgctttaaactagtacctttgacaactgagcaagtggaagcagcc
aatgagggagataacaactgcttactgcaccctatatgtcagcatggaatggaggatgaaagtaaagaagtctta
atatggcgctttgacagcagactggctttaagacatatagcaagagagaaacatccggagtattacaaagactga
>#57 AF382828 7380 nt
atgggtgcgagagcgtctatattaaccggcgggcgattagatgcctgggagaaaattaggctcagaccaggggga
aggaaaagatacatgatgaaacatatagtatgggcaagcagggagctggaaagattcgcttgtgaccctggtcta
atggaaacaaaagaaggctgttatcagttattgaaacagctagaaccagctcttaaaacaggatcagaaggatta
cggtccttatttaatactttggctgtgttatggtgtgttcatgctagaatagaagtagaagatacacagcaagcc
ttacaaaagttgaaagaatttgtgcaaaaggaaacagcaacagcacaggaagcagcagcaaagcaaagtgagtca
aacaccactgagataagcagaaattacccagtggtgcagaatgcacagggacagatgatacatcaggccatgtca
cccagaactttaagtgcctgggttaaagcagtagaagaaaaagcctttagcccagaagtcattccaatgttcatg
gctttatcagaaggggcaacaccccaagatgtgaacacaatgctgaatgctataggaggacaccaaggagctatg
caggtactaaaagaggtaatcaatgaagaagcagcggagtgggatagattacacccattacatgcaggaccagtg
gcaccaggtcaaatgagagagccaagaggcagtgacattgcaggtactactagtaccttgcaagagcaagtagga
tggatgacatccaatccaccagtccctgtaggagagatttacagaagatgggtggtattagggcttaataaggtg
gtaagaatgtattgccctgttagcatcttggacataaaacaagggccgaaggagccttttagagactatgtagat
agattctacaaagttttaagagcagaacaggcttcacaagatgtgaaaaactggatgacagagaccctcctggta
cagaatgccaacccagattgtaagcaaatacttagggcactaggaccaggagctaccctagaggaaatgatgaca
gcctgccaaggagtaggaggcccggcacataaggctagagttctagcagaagctatgagccaaatacaacagaca
agctcaatattcatgcaaagagcaggggggaagaactcctcctaggagagtcaagtgcttcaattgtggaaaggaa
ggtcatatagcaagaaattgtaaagcccctagaagaaagggatgttggaagtgtggaaaagaaggacatcagatg
aaggactgccaaaatgaaggaagacaggtggtagcaggacaactatgtgaagccctactagatacaggggctgat
gatacagtattagataatatacaactagaagggaaatggagaccaaaattaataggaggcattggaggatttatt
aaagtaaaacaatatgataatgtagaaatagaaatagagggaaaaagagtagaaagcacagtactagtgggacca
actccagttaatattataggaagaaatgtgttaacccaattaggatgtactctaaattttccaataagtccaatt
actcctgtgccagtaaaattaaagccaggaacagatggaccaagggtaaagcaatggccattgtcaaaagaaaag
atagaggcattaacagaaatttgtagggaaatggaaaaagaaggaaaaatttcaagaataggtcctgaaaatcca
tataatactcccatatttgcaataaggaagaaagacagtacaaagtggagaaaattagtagattttagagaattg
aataaaagaacacaagatttttgggaagtacagttaggcattccacaccctgcaggtttaaagaaaaagaaatca
gtaacagtgccggatgtgggagatgcttatttttcctgccccttagatgaaaactttagaaagtatacagccttc
accatccctagtgtaaataatgaaactccaggtataaggtaccagtataatgttctaccacaaggatggaaaggg
tcaccagcaatcttccaaagcagcatgacaaaggttttagaccctttcagacagaaaaacccagaattggtaatt
taccaatacatggatgacttatatgtaggatcagatttatctttggaagaacacaggaaaagagtagaacaactc
agagaacatttactcagatggggttttaccacccctgacaagaagcaccaaaaggaacctccttttctctggatg
gggtatgagcttcatccagacaaatggacagtgcagccaatacagctgcctcagaaagagaattggactgtaaat
gacattcagaaactggtaggtaaattaaattggcaagtcagatctatccaggaattaagataaaacaattgtgt
aaattaatcagaggagctaaggctttaacagaaatagtcgagatgactagagaagcagaattagagttagaagaa
aatagggaaattttaaaagaaccagtgcatggcacctactatgacccagaaaaagaactaatagcagaagttcaa
aagcagaataatgggcaatggacttatcagattttcaggaaacaggaaggaatctaaaaacaggaaaatatgcc
agacaaagagcagctcctactaatgacataagacaactagctgaagtggtgccaaaagtttcccctgaaagtata
atcctttggggaactgttcctaaatttaagttaccagtgcaaagggaagtatggaagcctggtggacagattat
tggcaagccacctggatccctgaatgggaattcgtcagaaccccaccccttgtaaagttatggtatcaattggaa
aaagaacctatacaaggagcagaaacctattatgttgatggggcagccaatagggagacaagaagaggaaaagca
ggatatgttattaataatggaaaacagaaggtcatttccttggaagagaccactaaccaaaaggcagaattagag
gcactcagaatagccctacaggactcagggcaagaagtaaatgtggtaacagactcccaatatgtgttgggaatc
ataaattcccaaccagaccaaagtgagtcagaaatagtgaatcaaataatagaagagctaataaagaaagagaag
gtctatttgacatgggtaccagcacacaaaggaataggaggaaatgaacaggtagataagttagtcagtagtggc
atcagaaaggtgctattttagatggtatagataaggcacaagaagaccatgagagatatcatagcaactggaga
gccatggctgatgaattccaactcccaccggtagtagccaaagaaatagtagccacatgtgataaatgtcagctt

aaaggagaagcaatacatggacaagtggattgcagtccagggatctggcagctggactgcacccacttagaaggg
aaaataatcctggtggcagtccatgtggcaagtggatacttagaagcagaagtaattccagctgagactggacag
gaaactgcatattttatcttaaaattagcagggagatggccagtaaagaagatacacacagataatggaacaaat
ttcaccagtgcagcagtaaaagcagcctgttggtgggcaggcataaaacaagaatttggaataccttataatcca
caaagtcaaggggtagtagaatccatgaacaaagaattaaaaaagattatagggcaagttagagaccaagcagaa
cacttaaagacagcagtacaaatggctgtattcattcacaattttaaaagaaaggggggattgggggtacact
gcaggagaaagaatagtagacatgctagcaacagacttacaaacacaagaactacaaaaacaaattctaaaatt
caaaaatttcgggtttattacagagacagcagagaccccatttggaaaggaccagcctctctcctgtggaaaggt
gagggtgcagtagttattcaggacaacggagatataaaagtagtacccagaagaaaagctaaaattattaaacat
ttagtaaagtttcatatgtacaagtcaaaaaaggccagaggatggttttataggcaccattatgatagtcccaat
cctaaagtaagttcagaagtccacatacccataagagatgcaaaaatagtggtctgtacatactggaatttaatg
ccaggagaacaaccatggcatctaggccatggagtgtccatagagtggaggcagagaacatatagaactcagata
gatcctcaaacagcagatcggctgatacactgttattattttgattgttttgcagcctctgctgtcagaagagcc
atcctaggggagattgtgacaaaggtgtgtgaattccctaggggcatcctcaagttcagtcactacaatatctg
gccatagtacaagtagtaaagacaaagaaaaagaagccacccctacctagtgtcactatcttagcagaagatata
ttggaggacttaaaaaacgaggctgtcagacattttcctagaccatggctacatcagttaggacaattaatctac
aataattatggagatacatgggaaggagtagaggccataatcagaatacttcaacacttgctgtttgtgcatttt
aggattggctgtcaacatagcaggatagggattgaacgacagaggagaagaaggagacttgagccctggaatcat
ccaggaagtcagccaaaaacagcttgcaattcatgctactgcaaaaggtgctgctatcattgtatgctttgcttc
accagcaaaggcttaggtatttcccaatggtgggtaacagtatactatggggttccagtctggaaggaggctact
acaccattgttctgtgcagcaaatgcaacagctttaaaagaggaacctcataatatttggccacacaagcctgt
gtgcctacagacccctctccagaagaggtcatcctaattaatgtgacagaagaatttaatgtatgggacaatgct
atggtagagcaaatgcaggaggatattactagcctatgggaccagagccttagaccttgtgtaaaattgaaccct
ttatgtgtgcaattaacgtgtacttctataaatgctactggaaacgaaactaatgctactggaaacggaattgaa
aaggatgggttggcacaagacatgagaaattgtactttcaatacaaccacagaattaagagataaaaaacagcaa
atttattccttattctggaaaaatgatcttgtaggtactaacaatacgtttaggttaattaattgcaatactaca
gcaatcacacaagcttgtccaaaaacctcctttgaaccaattccaatacattactgtgcaccagctggctttgcc
ctgttgaaatgtaatgataaagattatccaggaaaaggaaagtgtaaaaatgtaagtacagtgcattgcacacat
ggaattaagccaactgtaactactcagttgatactcaatggcagcttagcagaagaagaagaggtggtgttcaga
actaagaatatgacagctccaggtctcagtgacacagttatagtacagttaaaaaggcaattcccattaattgc
agtagaccaggaaacaatacaggaagagcaataaaccttagcccagggacaacattcttcaacacggaagcccta
ataggaaaccctaggaaagcgtcctgccaccttaatggtacactatggaataacattctaaacagaataaaacaa
aagattaaaaacagcacaacgtggcacaggggggacatcacattcacaaagcatccaggaggagacccagaagtg
gtaaatttcatgtttaactgtggaggggagttcttttattgtaatacatcaagattaattacctgtaacagtagt
gacacaagtgagtacattctcccatgcaaaattaggcaagtagtaaattcttggatgagagtggggaaaggtatc
tttgccccgcctagaagaggaactataacctgtaattccaccattacaggactcctcttagaagtacagaatggc
acggggaacaacacagaagtttacctgtcaggggagatatgagagatatttggaggagtgaattatataaatat
aaaatagtaaaaattgagccactgggggtggcaccaacaaaagcaaagaggtacacagtggcaaaagcacttgat
agatccaaaagagcagcactatggggctgctctggaaaaactgtgtgctactctactgtaccctggaatacttcc
tggaacagtaacaaatcttatgaggacatttggagtaatctgacctggcagcaatgggataagttagtagaaaat
catacaggaaccattttcaattattacagagagcacatgagcaacagaatagcaatgaaaaagagctattggaa
ttggatcaatggtcctcattatggaattggtttgacataacaaactggctgtggtatataaaaatgtttataatc
ttagtgggaggggttataggtttaagaattgtaataggtgtagtaaatataataagaagaagtaggcagggatac
tcacctttatcgtttcaggctattagcctgcttgatactacagcaattatagtagccgaaagaactgatacaata
atagaagtagcaactagaatagggagaggcatattgcatataccaagaagaatcaggcaaggtttagaaagagct
ttactagaataaatgggcaacaaatggtctaaaagcagcattgtaggatggccgcaggtcagagagagacttagg
cggactcaggaagcagcagaaggagtaggagaagtgtctcaggatttagcaaggcgtggagcaataacttccaga
catacccctcagactaatcaaactcttgcatggctagaagaaatggaacaggaggatgtagggtttccagtaaga
cctcaggtacctttaagaccaatgacatataaagcagcctttgatctcagccacttttaaaagaaaagggggga
ctggaagggttagtttattccaagagaagacaagagatcctggacctctgggtttatcatacccaaggatacttc
cctgattggaataactatactcctggaccaggagtgcgttatccgttgacctatggatggtgcttcaagctagtg
ccagtatcaccagaggcagtagaagaagccaacaagggagaggataatctcctattacatcccctgtgcactcat
ggatttgaggatgaagagaaggaggttctgatatggaagtttgacagtcaactagccctgcggcatcttgcaaga
gaaaaacatcctgagtattacagagactga
>#58 EF535994 7338 nt

atgggtgcgagagcgtcagtattaacgggaggaaagttagatgattgggaaaaaatttatctccggccaggggga
aagaaaaaatatatgatgaaacatttagtatgggcaagcagggagctggaaagattcgcttgtaaccccggccta
atggacacaacagaaggatgtgaacaattaattaaccagttgcagccagctctcacaacaggatcagaaggacta
agatctttatttaacaccctagcagtcctctggtgcgtacatcaaagaattagagtaaaagacacaaaagaggct
ttagacaaactagaagaagtgcagcaaaaaagcaaagtgaaagtaaaacaggaagccgcggcggcagaccccagc
ggcggcgagagcagaaacttcccgatagtaacaaatgctcagggacaaatggtccatcagtcgctctcacccaga
actttaaatgcatgggtaaaagtagtagaagaaaaggcatttagcccagaagtcatacccatgtttatggctcta
tcagaaggagccacccctcaggatttaaacaccatgctaaatacaataggggacatcaggcagcaatgcaaatc
ttaaaagaagtgatcaatgaggaagctgcagagtgggacaggacacaccctatacatgcagggccaattcctcca
ggccaaatgagggaaccaagaggaagtgacatagcagggacaactagcaccattcaggaacaagtgggatggatg
accagtaaccctcctaccccggtaggagacatttacaagagatggatagttttagggcttaataaaatagtaaga
atgtatagccctgtcagtattctagatataaagcaaggaccaaaagaaccatttagagattatgtggacagattt
tacaaaactctaagagctgaacaagcaacccaagatgtaaaaaattggatgacagagaccttgctagtgcaaaat
gccaacccagattgtaagcaaattttaagagctctgggtccaggggcaaccttagaagaaatgatgacagcctgc
cagggagtagggggcccctctcataaagccagagtattggcagaagcaatgagtcaggcaaacaccactgttatg
atgcaaagaggtaatgttaagattcagaaaagaattgtgaaatgctttaactgtggcaaggaaggtcacatagca
agaaactgtagagcccctagaagaaaaggctgttggaaatgtgggcaagaaggacaccaacagaaaaactgcccc
gatatgcaacgggcgggagttataaaagaggtactattagacacaggggcagatgatacagtactagaggaaata
gaattagagggaaaatggaggccaaaaatgataggggggaattggaggttttataaaagtaagacaatatgatcag
atacccatagaaatttgtggaaaaagagcaataggtacagtattagtagggcccacacctgtgaatattataggg
agaaatatcttaactcaaattgggtgtactctaaattttcccattagcactatagaaactgtaccagtaaaatta
aagccagggatggacggtccaagagttaaacagtggcccctaacagaagaaaaaataaaagcattgacagaaatt
tgtacagagatggaaaaggaaggaaaaatttcaagaatagggccagaaaatccatataatacaccaatatttgct
attaagaaaaaggatagcacaaaatggagaaaattagtagacttcagagaattaaataaaagaacccaagatttt
tgggaagtccagctaggaataccacaccctgcgggactgaaaaagaaaaaatcagtaacagtgctggacgtaggg
gatgcttatttttcatgcccccctagatgaagagtttagaaaatacactgccttcaccatacctagtgtaaataat
gagacaccaggaataaggtaccagtacaatgtactcccgcagggatggaaaggatcaccagctatttttcaaagc
agtatgactaagattctggaaccttatagaaaacagaatccagaaatagtcatctaccagtacatggatgatcta
tatgtaggatctgatttacatatagagcaacatagagaaaaggtggaagaattaagagctcatctattaaagtgg
ggctttactacacctgacaaaaagcatcaaaaagaaccaccattcctttggatgggatatgagctccatccagac
aaatggacagtccagcccatacagctgccagamaaagacagctggactgtcaatgacatacagaaattagtagga
aagttaaattgggcaagtcaaatatatccaggaattaaggtaaaacaattgtgtaagcttattagaggaacaaaa
gctttaacagaggtagttrccctcacacaggaagcagaattagagttagcagaaaacagggagatattaagagac
cctgtacatggggcctactatgacccctcaaaagaattgatagcagaaatacagaaacaaggtcaaggccaatgg
acttatcagatttatcaggaacagcataaaaatctgaaaacaggaaaatatgcaaaaatgagagctgcccattca
aatgatgtaaaacagttgactgaagtagtacagaaagtagccctggaaagtatagtcatctgggaaaaacaccc
aagtttagactgcccatacagaaagaaacatggaggcttggtggacagattactggcaagcaacttggatccct
gaatgggaatatgttaacacccctcccctggtaaagctgtggtaccagttagaaacagaacccatagtgggggca
gaaaccttttatgtagatggggcagcaaatagagaaaccaaattaggaaaagcaggatatgtaactgatagaggg
agacaaaagattatctccctaacagaaacaactaatcaaagagcagaactgcaagctatatacctagccctacag
gattcagaagtagaggtaaacatagttactgactcacaatatgcattagggatcattcaatcacagcctgacatt
agtgaatcagacatagtaaatcagataatagagcagctagtgaaaaaggaaaagatttatctttcatgggtacca
gcacacaaagggattggaggaaatgaacaaatagacaaattagttagtgcaggaatcaggaaagtcctttttcctg
gatggaatagataaggcccaagaagaacatgagaaataccacaacaattggaaagctatggcagatgaatttaat
ttgccacccatagtagccaaagaaatagtagccagctgtgataaatgccaattaaaaggagaagccatgcatgga
caggtagactgtagtccaggaatatggcaactagactgcacacatttagaaggaaagatcatcttagtagctgta
catgtagccagtggatacatagaggcagaagtcatacctgctgaaacagggcaagagacagcttattttgtgctg
aaattagcaggaagatggccagtaaaggttatacacactgataatggcagcaactttactagtgctgctgtcaag
gctgcatgttggtgggcaaatatccaacaggaatttggaattccctacaatcctcagagtcaaggagtagtagaa
tcaatgaacaaagagttaaagaaaatcatagggcaggtaagagatcaagcagaacatttaaagacagcagtgcaa
atggcagtcttcattcacaattttaaaagaaaaggggggattggggggtacagtgcaggggaaagaatcatagat
ataatagcaacagatatacaaactagagaactacaaaaacaaatttcaaaagttcaaaaatttcgggtttattac
agggacagcagagaacctatctggaaaggaccagccaagcttctctggaaaggtgagggagcggtagtgcttcaa
gataaggaagatataaaagtagtcccacgcagaaaggctaaaataatcagggacgcatggaacagtttggtaaaa
catcacatgtatgtgtctaaaaaggcacgaggttggttttacagacatcattatgaaagtgagaatccaaaagtt

agttcagaaattcacattccagtaggagatgccaaattagtaatagtaacctattggggactcatgccaggagaa
agagactggcatttaggacatggggtctctatagaatggagacagaaaagatatagcacacaaatagatcctgaa
ttggcagaccacctaattcatttgcattattttgattgttttgcagagtctgccataaggaaagccatactagga
caaatagttagccctagatgtgaattcccagcaggacacacaaaggtaggcactctacaatatttggcaatctca
gcactgctaaaaggaagccaaagaaaccacctctacctagtgttagtaaattagcagaagacttattagatgaa
ctcaaacaggaagcagtaagacattttcctagacagtggcttcatgatttaggacagcacatttataacacatat
ggagacacttgggcggggggttgaggctatcataaggatcctgcaacaattgctgtttattcattacagaattggc
tgccaacatagcagaataggcattctgccacaaggaagaaggagattagaaccctggaatcatccaggaagccag
cctaaaacccccctgtaacaattgctattgtaaaatgtgttgctatcactgtcaattttgcttcacaagaaaaggc
ttaggaatttactctgaaaataatttatgggtaacagtgtactatggagtacctgtatggaaagaggcaaaaaca
actctttttttgtgcatcagacgctaaagcacagatagctgaggcacacaacatatggcttctcaagcttgtgtg
cctacagatcctaaccctgaagaaataacgttagaaaatgtaacagaagagtttgatgcatggaacaataacatg
gtagaccaaatgcaagaagatctcattagcctatgggatcaaagcctaaagccatgtgtaaaactgacccccctc
tgtgtgaccttaaactgttccacaaatacaactacagaggcatctcaggtgcagtataattgttctttcaatgta
actacagaattaagagataagaagaagcaagtgtactccttattttatagagaggacataacaagtcttgatagc
aataagacagttaaaaatggaacttataggttaattaactgtaataccacagccataacccaggcttgtccaaaa
acatcatttgaaccaattcctatttactattgtgcaccagctggatttgccctttttaaaatgtaatgaccagaat
ttcaagggaaaaggacatgtagaaatgttagcacagtgcattgtacacatggtattaagcctgtagtctctacc
caattcctcttgaatggaagcctggcagaaggaaacaagactgtagttagggtaagaagtaaatctaatacagag
actatcattgtgcagctagctacagcaatatatatcaattgtaccagactaggtaataaaaccatagagggcata
cctatagggccaggacaaatttttatataggactaaaacggtggtaggcgacaccagaggagcagagtgcagaatt
aatgggacagcctggaatgagactctgagacaggtaaaggaggcattaaacaacacatatcgaaatctcaatctc
agtctcacagaaataaactttgaaggtgcatccggcggagatctagaagtaactacacattatttcaattgtgga
ggagaattcttttactgtaatacatcaaacttattcaatcaatccactattaacaatattacacacattccgtgc
agaataagacaaattgtaaatcagtggcaaggagtaggaaaaggaatctttgcacctcctattagaggaacaatt
caatgtaattccacaatcacaggcctactcttaacaagagatggtaaaaatgagactgaaacttttagacctaca
ggaggagacatgagaaataattggagaagtgaattatacaaatacaaggtagtaaaaattgaacccctaggatta
gcaccaaccaaggcaaaaaggagaacagtgcaaagagaaaagaggggactatggggctgctctggaaaactcatt
tgcactactaatgtgccttggaataatacttggagtaaaaaattaggaacaaacctatcctttttgggataacatg
acatggatgcaatgggagaaagagattgataactacacagaaactatctatgaacttctaacacgatctcagaat
caacaggaagtcaatgagcaagaattgttagctctggataaatggtcaagcctgtggaattggttcgatataaca
cagtggctgtggtatataaaattatttgtcatgatagtaggaggcttaataggcataagaatagtttttgctatg
ctttctatagtcaatagagttaggcagggatactcacctttgtcgttccaggctataagcttgcttgacgccaca
gcaatagcagtagcagagggaacagatagaatyatagaaatagcacaaagagttggcagaggcattttgcatata
cctagaagaattagacaaggtttagaaagagctttgctataaatgggaaacaaatggtcaaaaagtagcctggta
ggatggcctgcagtaagggacagacttagaaggacacctccagcagcagaaggagtagggccagtctcgcgagac
ctagagagacatggggctataacaagtaggaatactccacagacaaatgagactcttgcctggttagaggagcaa
agggacgaagaagtaggatttccagtaagaccccaagtgcctttaaggcccatgacctataaagcggcctttgat
cttagccactttttaaaagaaaaggggggactggaagggctaatctactcccaaagaaggcaagagatccttgat
ctctgggtctatcatacacaaggcttcttccctgattggcaaaactacacaccaggaccaggggtcagatacccg
gtgaccttcggctggtgcttcaagctagtgcctgtagatccaacagatgtggaggaagcaaatgaaggagacaac
aatgtgctattacatcctatgtgccagcacggacaagaagatgaacacagagaagtgctgatgtggcagtttgac
agccacttggccctgacacacagagcccgagagctgcatccggagtactacaaagactgctga
>#59 DQ373066 7338 nt
atgggtgcgagagcgtcaatattaaccgggggaagattagatgcttgggaaaaaattaggctaagaccaggggga
aagaaaaaatacatgatgaaacatttagtatgggcaagcagagagctggaaagattcgcttgtaaccctggtcta
atggaaaatgcagaaggttgtgagaagttattgaaacagctagaaccagctcttaagacagggtcagaaggactg
cgctctttatttaacactttggcagtcctttggtgtgtacataagagaatagaagtggaagacacacaacaggct
ttacagaaactgaaagctgccgtgaacccagaacagggtgaaacaccatcagctagcgcgaacttccctgtagta
cgaaacgctcagggacagttgacgcataatccaatatcacccagaaccctaaatgcctgggtaaaagcagtagaa
gaaaaagctttcaatccagaggtcataccaatgttcatggcattatcagaaggggcaacgcctcaagacctcaat
actatgctaaatacaataggaggacatcaggcagccatgcaaattctaaaagaagtaatcaatgatgaggctgct
gagtgggacaggacacaccctacccatgcagggcctatagctccaggacaaatgagggaaccaagaggcagtgac
atagcaggcactactagctccctagaggagcaagtagggtggatgacagcaaaccctcctattccagtaggagat
atttatagaagatggatagtcctggggttaaataaaatagttaaaatgtacagtcctgttagcattttggacatt

aaacaaggaccaaaagagcctttcagggactatgtagacaggttctacaagaccttaagggctgaacaagcatca
caggatgttaaaaattggatgacagacaccctgttgatacagaatgctaacccagactgtaaaaacatcttaaga
gcattaggaccaggagccactctagaagaaatgatgacagcttgtcaaggggtaggaggaccctcacataaggct
agagtgctggcagaagccatgagccaggtaaacaaaacagaaatctttatgcaaagaggggacaaaatagacct
cctaataaaaagattaaatgctttaattgtggaaaagaaggacacctagcaaggaactgcagggcaccccgaaga
aagggctgctggaagtgtggacaggagggacatcagatgaaagattgtaatcaaaataatggaaggattattgaa
ggacaattaattgaagccttgctagacacaggggctgatgacacagtaattgaagacttaaatttgccaggaaaa
tggagtccaaaaatgataggggggaattggaggtttattaaagtaaaacaatttgaaagagtaaacatagaaata
gaaggaaaaaagataaccagtactgtattagtaggaccaacccctgtcaatataataggaagaaatgtcctaaca
aaattagggtgtactctaaatttcccaataagtccaatagacacagtaaaagtgtttctaaagccaggatggac
gggcccaaggtaaaacagtggcctctgtcaaaagaaaaaatagaggcattaactgaaatatgtaaagaaatggaa
aaggaaggaaaaatttcaaaaatagggccagaaaatccttacaatacacctattttgcaattaagaaaaaggac
agcaccaaatggagaaaacttgttgatttcagagaacttaataagaggactcaagaattttgggaagtacaactg
ggcataccacacccagctgggttaaagaagaaaaaatcagtaacagtcttggatgtgggagatgcctatttttca
tgtcctcttgacccagactttagaaaatttactgcattcaccattcctagcataaataatgaaactccaggaatt
agatatcaatataatgtacttcctcaaggatggaagggatctccttccatctttcagagtagcatgacaaagatc
ttagaaccctttagaacaaagcatccagaaataattatttaccagtacatggatgacttgtatgtgggctcagat
ttacctatagaggaacatagaaagagagtagaagagctcagagcacatctgttaaaatggggatttacaacacca
gacaagaagcatcagaaagaaccccatttctctggatgggatatgagttacatccagataagtggacagtccag
caaataaaattgccagaacaggaagtgtggactgttaatgatattcagaagttagtaggcaaattaaattgggca
agtcaaatatatccaggaattaaaattaagcagctttgtaaattaattaggggagcgaagggtctgacagaagta
gtagctctcaccccagaagctgaactagaactggcagaaaatcaagagatactaaaagaaccagtacatggagcc
tactatgaccctgaaaaggaattaatagcagaaatacagaagcaaggacagggtcagtggacatatcaaatattt
caggagccacataaaaacctaaagacaggaaaatatggcaaacaaagagctgcacatactaatgatattaggcag
cttgctgaaacagtacaaaaaatagctacagaaagcattgtaatatggggaaaaacaccaaaatttagactccct
gttcaaaaagaaatgtgggaagcctggtgggcagattactggcaggctacctggattcctgagtgggaattcata
aacaccccacccctagtaaagttatggtaccagttagagacagagcctatagaaggagcagaaacatactatgta
gatggggcagccaacagagaaacaaaaagaggaaaagcaggctatgtgacaaacaggggggagacaaaagattcat
agtcttgaagataccactaatcaaaaagcagaattacaagcagttttaatggccttacaggactcaggacaggaa
gtcaatgtggtcactgactcccaatatgtgctgggaatcttacaatcacacccagaccacagtgaatcagatata
gtaaatcagataatagaagagttaataaaaaaggaaaaaatttatctatcatgggtaccagcacacagaggtata
ggaggaaatgaacaggtagataaactagttagttcagggatcagaaaagttcttttcttagatggaatagaaaaa
gctcaagaagaacatgaaagataccactctaattggaaggcaatggctcaggactttaacttaccaccaatagtg
gctaaagaaatagtagcccaatgtgataaatgtcagctaaaaggagaagcaatgcatggacaagttgactgtagc
ccaggaatatggcagattgattgcacccatttagaaggaaaaataattatagtggcagtccatgtggccagtgga
tacatagaagcagaagtcatccctgctgaaacaggacaggaaacagcatattttgtccttaagttagcaggaaga
tggccagtaaagactatacacacagacaatgggcctaatttcactagtaatgcagtaaaggcagcatgttggtgg
gcaggtattagacaggaatttggaattccatacaacccacaaagtcaaggagtagtggaatctatgaataaagaa
ttaaaaaagatcatagagcaagtcagagatcaggctgaacatttaaaaacagcagtacaaatggcagtcttcatt
cacaattttaaaagaaaaggggggattggggggtacagtgcaggagagagaataatagacatcatagcaacagac
atacaaacaaaagaactacaaaaacaaattttaaaaattcaacaatttcgggtctattacagagacagcaaagat
ccaatttggaaaggaccagcgaaactcctgtggaaaggtgaaggggcagtggtaattcaagatcaggacgaaata
aaagtggtgcctagaaggaaagctaagataattagggatacctggaatagtttagtgaaatatcatatgtacaga
tcaaagaaagcaaaaaattggttctatagacatcactatgatggcaccaaccctaggatggcttcacagatatat
attccaataaatcaggctaagctaattgtcacaacatattggggactaatgccaggagaaagagattggcatctg
ggacatggagtagcaatagaatggagagaaagaggttacagtacccaggtagaccctgaaacagcagataggcta
atacaccttcattattttgattgttttacagcaactgccatcaggaacgctatcttagaaaaggcagtcctccct
aagtgtgaattcccagaagggcacagcaaggtagggtctttacaatactggctctaaagcagttggttaaaacc
aagaaaaatagaccacctctgccctcagtagcaaagttagcagaagatacattggaagaaattaaacaagaagca
gttagacactttcccagaccctggttacatgcactaggagaacatatttatgaaacatatggagactcctgggcg
ggagtacaagctataataagaattttgcaacaattactgtttgtccatttcagaattggctgccatcatagcaga
ataggcataaaccctcagagaagaaggagattagaaccatggaaccatccagggagcaaacctagaacagaatgc
aataagtgcttttgcaaaaagtgttgttatcattgtatactttgctttacaaagaaaggcttaggcatctccgaa
aattggtgggtcactgtttattatggggtcccagtatggagggaggcaaaaaccaccctttctgtgcctccgat
gctaagagttatagtacagaagctcataacatctgggccacccaagcttgtgtacctactgatcccactcctcaa

gaggtactcttacctaatgtcactgaagagtttaacatgtgggaaaattacatggtagatcaaatgcaggaagac
ataataagtctctgggaacaaagtcttaaaccatgtgtaaaattaacacccttatgtgtgacccttacttgtaat
aatcccactaatacttcctgcactaactctactgacgatcgcttgggagacatgagaaattgtagctttaatgtc
acaactgaactaagagataagaaaagacaagtgtattccttattctatgtggaggatataacagcaataggaaat
aatagtacttacaggttgattaattgtaataccacagccatcactcaagcttgtccaaagacttcctttgaacca
attccaatacattactgtgcaccagcaggatttgcactcctaaaatgtaatgatatagattacaaaggaatgaa
acttgtaaaaatgtaagtacagtacattgtacacatggcattaaaccagtggcaaccacccaactgattttgaat
gggagtacagcagacaatcagactgtggccagaatagacccttcagagaatttagcaataatacagctcaaagat
ccagtaaagattacatgtaggagaccaggaaataacacaagaggtcaaatacaaataggaccagcaatgactttt
tataacatagaaaatgtagtaggggacactagaaaagcatattgtgagataaatgggacacagtgggcaaaagcc
ctcaatgaaacaaaagaagttctaaggaacatactgaggaagaacataagctttatggtcccttctgggggagat
ccagaagtgactaaccatcactttaactgcgggggagaattcttctactgtaatacaagtgagatcatcaatatc
actaagataaacaaaactgagaatatgacaataatcccttgtagaataagacaaatagtgaattcctggatgcgt
gtagggaaaggaattttgcaccaccaattagaggtaacattacctgcacttctaatatcacaggtatgctacta
gagatacataagaaccgagaagatcagggagaagatcaggatcagaataacacatacgtttgtctaacaggggt
aacatgaaagatatttggagaagtgaactatacaaatacaaaatagttgagatacaaccactaggagtagcacct
actaaaagtagaagatatgcagtagaaaaacaacaccatagagaaaagagaggtctatgggctgtacaggaaaa
actatttgtcccactgctgtacgctggaacaagacatggggaaatattagtgactatcaggttatttggaataat
tacacatggcaacaatgggatagagaagtaaataattacacaggactcatctacacattgctagaggaggctaat
acacagcaggagaaaaatgaaaaggagttattggaattggattcatgggctaatttatggagttggtttgacata
acaaattggttgtggtacataaagatgttcttaatagtagtaggaggcattataggcttaagaatatgctttgca
ataggatcactaatcaatagggttaggaagggatactcacctttgtcattgcaggccataagcttattagatact
accgctattgcagtaggagaaggaactgatagaatcttagaagcaataacacgcttgggcaggggcattttacat
atacctagaagaattagacaaggcttagaaagagctttgctttaaatgggagccaagtggtcaaaaagtagccta
gtaggatggccagaaataagagagagagaataaaaagagctccaacagcagcagatggagtaggggaagtatcacaa
gacatagcaagccgtggagcagtcacaattagaaataccccacaaaccaatgagactttagcttggctagaagaa
atgcaggatgaagaagtgggatttccagtcaaaccacaagtgcccttaagacctatgacctttaagggtgccttt
gatctctctcacttttaaaagaaaagggggactggaagggttaatctggtcaaggaaaagacaggagatcctt
gatttatgggtttatcacacacaaggttacttccctgactggcaaaactacaccccggggccaggagtgcgctac
ccactaaccttcgggtggtgctttaaactggtccctttgccacctgaggaggtggagaaagccaatgaaggagaa
aacaactgcctgctacatcccatgtgccagcatggaatggaggatgaggaaagagaagtactggtatggactttt
gatagcaagctggctctcagacatctagcaagagagaaacatccggaatattacagagactga
>#60 DQ373064 7356 nt
atgggtgcgagagcgtcaatcttaacaggcggaaagttagatgattgggaaaaagttcggctaaggcccgggga
agaaaaagatatatgatgaaacatttagtatgggcaagcagggagctggagagatttgcttgtaaccccgggctt
atggaaacaacagaaggctgtgagcaattgattacacagttagaaccagctcttaagacagggtcagaaaacctt
aaatctttatttaacaccatagtggtcctctggtgcgtgcatcagagaatcgcgataaaagacaccaaggaggct
ttagatacactaacagcagttagacagaaacagcaaaaggaaaaggaagcagctgcagccgcggcaagggacaat
gacagtggtagcagaaactaccctatagtgacaaatgcacaagggcagccggtacaccaggccctctcacctaga
actttaaatgcctgggtcaaggtagtagaagaaaaggcatttagcccagaagtaatacccatgttcgcagcctta
gcagaaggagccacccctcaagatttaaataccatgctaaatacagtaggggacaccaggcagccatgcagatc
ttaaaagaggtcattaatgaggaagcacaagaatgggataggctgcatcctatccatatggggccaatagcacca
ggccaaatgagagaaccaaggggaagtgacatagcaggaaccactagtactgtacaagaacaaataggatggatg
actagcaaccctcctataccagtaggagaaatttacaaaagatggataattatgggctgaataaggtagtcaga
atgtacagccctgttagcatcctggacataaagcaagggccaaaagaaccattcagagactatgtggacaggttt
tacaaaacattaagagcagaacaagccacacaggaagtaaaaaattggatgacagacaccttgcttgtacagaat
gctaacccagattgcaaaagtatcctgagagcattgggtccaggagcctccttagaggaaatgatgacagcatgc
cagggagtcgggggtccagcccataaagccagggtcttagcagaagctatgagtcaagcaaatgcagcaacagtc
attatgatgcaaaggggaaattttaaaggaccaaaaagagttattaaatgtttcaattgtggcaaagaagggcac
atagcaagaaattgtaaagcccccaggagaaaaggctgttggaaatgtgggcaggaaggacatcggatgcaagat
tgtaacatggggaaggttgtcgggggctacttgaaaggagcactgttagatacaggggcagatgatacagtgcta
gaagatttagaattaggggaaggtggaaaccaaagatgataggaggaattggaggttttatcaaagtaaagcaa
tatgataatataccaatagaaataggaggaaaaaaggcactaggtacagttttagtaggacccacaccagtaaat
atcataggaaggaacattttaactcaaattggatgtactctaaattttcctattagccctattgaaactgtacca
gtacaattaaagccaggaatggatgggccaaaagtaaaacaatggcccttaacagaggaaaaaattaaagcttta

acagaaatctgtacagaaatggaacaggaaggaaaaatttcaagaatagggcctgaaaacccatataatactcct
gtgtttgctataaaaaagaaagatagcacaaaatggagaaaattagtagattttagagaattaaataaaagaact
caagacttttgggaagttcaattaggaatacctcaccctgcgggattaaagaagaaaaggtcagtcacagtattg
gatgtaggagatgcctacttttcatgcccccctagatgaaaactttagaaaatatactgcattcaccatacctagt
ataaacaatgaaacaccaggaatcagatatcaatacaatgttctaccgcagggatggaaaggatcaccagctata
tttcaaagtagcatgacaaagattttagagccattcagaagacagaacccagatatagtaatttatcagtacatg
gatgatttatatgtagggtcagatttgaaaatagagcaacacaggaacaaggtagaagagctgagagctcacttg
ctaaagtgggggtttaccacccctgacaaaaaacatcagaaggaaccaccatttctctggatgggatatgaactc
catccagacaaatggacagtacagcccatacagttaccagaaaaggaaagttggactgtcaatgacatacagaaa
ttggtgggaaaattaaattgggcaagtcaaatatacccaggcatcaagataaaacagatatgcaggcttcttagg
ggagtaaagaattcaacagaagtagtcacctttactagagaagcagaattagaattagcagaaaatagggaaata
ttaaaaaacccagtacatggggtctattatgacccggctaaagacctgatagcagaagtacaaaaacaaggacag
agtcaatggacctatcaaatctatcaggaacaatttaaaaatctgaaaacaggaaaatatgccagacaagggtct
gctcataccaatgatgtaaaacagttagcagaggtaatgcaaaagatagccatagaaagcatagtcatatgggga
aaaataccaaagtttagattgcccattcaaaaagaagcatgggaagcatggtggacagattattggcaagccact
tggatccctgaatgggaatatgttaatacccccttcccatgtcaagttttggtaccaattagagcaagaacctata
ccaggggcagaaacatttatgtagatggggcagctaacagagaaactaagttaggaaaagcaggatatgtaaca
gataggggggaagcagaaaataatccccttaacagaaacaaccaatcagaaggctgagttacaagccattcaatta
gccttacaagactcaggacctgaggtaaatatagttactgattcacaatatgcattaggaatccttcaagcccaa
ccggatcagagtgattcagaattggtgaatcaaatcatagaaaatctaataaaaaaggaaaaagtttacttaaca
tgggtacctgcacataagggaataggaggaaatgaacaagtagacaaattagtcagttcaggaattaggaaagtc
ctttttctggatggaatagatcgagcccaggaagagcatgaaaaataccataataattggagagctatggccagt
gatttcaatctgccacccattgtagcaaaagagatagtagctagttgtgataaatgtcagctaaagggagaggca
atgcatggacaggtagactgtagcccaggaatatggcagttagattgtacacacctagaaggaaaagtcatctta
gtagcagtgcatgtagctagtggctatatggaagcagaagtcatacctgcagaaacaggacaggaaacagcttat
tttatcttaaaattagcatcgagatggccagtaaaaattatacatactgataatggtagcaattttactagcact
gcagtcaaggctgcatgttggtgggcgcagatccagcaggaatttggaattccctacaatccccagagtcaagga
gtggtagaatcaatgaacaaagaacttaagaaaataatagggcaggtaagagatcaggcagagcacctaaaaaca
gcagtacaaatggcagtattcattcacaattttaaaagaaaaggggggattggggggatacagtgcaggagaaaga
ataatagacataatagcaacagatatacaaactacagaattacaaaaacaaatttcaaaagttcaaaaatttcgg
gtttattacagggaccgcagagatcctatttggaaaggaccagccaaactactgtggaagggtgaaggagcagta
gtgctgcaagatcaggaagaaataaaggtagtcccacgtagaaaagctaaaattattagggatgcatggaacagt
ttggtaaaatatcatatctatgtatccaggaaagcaagaggctggttttatagacatcattatgagagtgaaaat
ccaaaagtaagttcagaaatacatatcccactaggggaggccaaattagtaatagtaacttattggggacttaca
ccaggagaacgggactggcacttaggtcatggggtatctatcgagtggaaacagaagaaatatagtacacaaata
gaccctgagttggcagatcatatgattcatttatattattttaattgttttgcagaatctgccataaggaaggcc
atattaggacgagtagttagccctagatgtgaatttccagcaggacacaagaaggtaggaaccctacaatatttg
gcaatctctgccttattacaaagacagccaacaaggccgcctttgcctagtgtcagcaaattagcagaagatgta
ttagaagaactcaaacaggaagcagtaagacactttcctagaccatggctccaccaattgggacaatatatatat
gacacctatggagacacctgggagggagttcaagctatcataagaatcctgcaacaactcctgtttattcattac
agaattggctgtcaacatagcagaataggcattacaccacaaagaagaaggagactagatccctggaatcatcca
ggaagtcagcctaaaactccctgtaacaattgcttttgtaaaaagtgttgctatcactgtccattttgcttcaca
aagaaaggcttaggaatttccagcactaaaaatgaaaatttatgggtgacagtgtactatggagtgcctgtctgg
aaagaagcagaagcaactctcttttgtgcgtcagatgcaaaagcacaaaaggcagaggcacacaatatatgggcc
tctcaagcttgtgtacccacagatcctgatcctaaagcaatagaactaagcaatgtaacagaaaattttaatatg
tggacaaatgcaatggtaaatcagatgcaacaggatgtaattagcttatgggatcaaagcctaaaaccatgtata
aagctaacccctctttgtgttactttacattgcagtatccctaaatttgataactctagcattaatagctctaat
gagactatgaaaaattgtagcttcaatacaacaacagaactaagagataagaagaaacagacttactcattgttt
tatgtagatgatttaactcagataaacaagacaaccgagagctataagcttataaattgtaatactacagccata
acccaggcttgcccaaagatttcttttgagccaatacctatacattattgtgctccagctggttttgctcttcta
aggtgtaacaataaaacatataatggtagtggtccatgtaacaatgttagtacagtacactgtactcatggaatt
aaaccagttatttctactcaattgatcctgaatggtagtctagcagaagaaaaaatcatgattaggtacaaggct
gataaggttctagtacagctcaatacaagtatacacatcaattgtactagggtaggaaataagacaataaaagga
atccccctaggaccaggacagctgttctatggaacagaaactgtagtaggagacacaagagaggctcattgtgaa
attaaccagacacattggtataaaatactaaaccaggtgaaaagagagctaaccacggtcttcaatgaatctaac

aaaactgtacactttgcaaacagttcaggtggagatccagaagtggcaaaccttcattttaattgtggaggagaa
tttttttattgtaacacctcggccttgttcaatgacaccttgttacgcaataaaaccttgctcaatgaaaccttg
cacaataacttgacactaccttgtaggattagacaaattgtaaacctatggctaagagtaggaaagggaatattt
gctccacctattagaggtaacatccgctgtaattccactattactgggttgatcctagaaaaacacacaaacaca
gacaatatcaccttccgtccaataggaggggatatgacagacatctggagaagtgaattatataactataaaata
gtaaaaattgaaccattaggagtagcgcctaccagggcgagaaggaggactgtggatagagaaaaaagaggaata
tggggttgctcaggaaaactcatctgcaccacttctgtgccatggaatagaacttggagtaacaaaacatataat
gaaatttgggacaacatgacatggatggaatgggacagggaagtaagaaattacacagaaattatttatgggtta
atagagcaagcacaagaccagcaggaaaataatgagaaaaaacttttagaactggatcattggacaagcctgtgg
aattggtttgacataagtcactggctgtggtacataaaaatattcattatgataataggaggcttaatagtgtgt
aggataatttttgctgtgctcgctatagtaaatagagtcaggcagggatactcacctctgtcatttcaggccact
agcttgcttgatacaacagcaatagcagtagctgagggaacagatagaatcatagaaatagtacaaagaattggt
agaggaatattacatatacctagaaggattagacaaggtttcgaaagggctttattataaatgggaagcaaatgg
tcaaaaagtagcctggtaggatggcctgaagttagaagaagaattagacaagaaactgtagcagcagaaggagta
ggaccagcctcacaagacctagctaggcatggggcaattacaagtagtaataccccacaaactaatgagaccatt
gcctggttagaggagcaaaaggaggaagaaatatggtttccagtaaaacctcaggtaccattaaggccaatgact
tttaaagaggcttttgatcttggctactttttaaaagaaaaggggggactggaagggttagtttactctaaaaag
agacaagaaatccttgacctcggggtttatcacacacaaggaatcttccctgactggcagaattacaccccaggg
ccagggattagatatccaataacctttggcgggtgcttcaagctagtaccactaaccccagaagaggtggaggag
gctaataaaggagagaacaatctactactgcacccaatatgccagcatggaatggaagatgaagacaaagaagtg
ctgatatggaaatatgacagccagctcgccctaagacacatagctcgagagctgcattcggagtactacaagaac
tgctga
>#61 KP861923 7566 nt
atgggtgcgagagcgtcagtattaaccggtagtaaattggatgattgggagaaaattaggttaaggccaggggt
aagaaaaaatatatgatgaaacatttagtatgggcaagcagggagctggacagatttgcttgtaaccccggcctt
atggaaacaggagaaggctgtgaacagttaattaaacagctagaaccagctctcggaacagggtcagaggattt
agatctctctttaatactttagtagtcctctggtgtgtacatcagagaattcaggtaaaagacaccaaagaagcc
ttagaccagctggcagaatggcaacaaaagcaaaagaaaaaggaagcagcagcgacagcagcagacagctcagtc
agcagaaactaccctgtagtagcaaatgcacagggacagatggtgcatcagccattatcacccagaaccttaaat
gcctgggtgaaagtggtagaagaaaaggcatttagcccagaagtaatacctatgttcatggcattagcagaagga
gccaccccctcaagatttaaataccatgctgaatacagtaggggggacatcaggcagccatgcaaatattaaaagaa
gtaattaatgaggaagcatcagaatgggataggatacatcccattcatatggggcctatagcaccaggccaaatg
agagagccaaggggaagtgatatagcaggaactactagtaccatacaggagcagataggctggatgactagtaac
cctcccattccagtgggagagatttataaaagatggatagttctggggctaaataaaatagtaagaatgtatagc
cctgtcagtattctagacataaagcagggaccaaaagaaccattcagagattatgtggacagattttataagacc
ttaagggcagagcaggccacacaggaagtaaaaacttggatgacagacaccttgcttgtgcagaatgctaaccca
gattgtaagaacatcttgagagcactgggtccaggagccaccctagaggaaatgatgacagcatgccagggagtg
gggggccctgcccataaggctagagtactggcagaggccatgagtcaagcaaatgctacagtaatgatgcagagg
ggaaattttaaaggccctaagagaactattaaatgctataactgtggcaaggaaggccacctagccaggaactgc
aaagcccccagaaggaaaggctgttggaaatgtggacaggaaggacacagaatgcaggattgtaaactaagcaag
gttataggaggacacttaaaagaggcactgttagacacaggggcagatgatacagtgctagaagagctagaatta
gggggaaaatggaaaccaaaaatgataggggggaattggaggttttatcaaagtaaagcagtatgaaaatatacca
atagaaatatgtggaaaaagagcagtaggtacagttttagtaggacccacacctgttaatataatagggagaaac
atattaactcaaattggctgtactttaaattttcctatcagtcctattgaaactgtaccagtagcattaaagcca
gggatggatggaccaagagtcaaacaatggccattgacagaagaaaaaataaaagcattgacagaaatttgcaca
gaaatggaaaaggaaggaaagatttctaaaattgggcctgaaaatccatacaatacaccagtatttgctataaaa
aagaaagatagtacaaaatggagaaaattagtagatttcagagaattaaataaaagaactcaagatttctgggag
gttcagttaggaataccccaccctgcggggttaaagaagaaaaaatcagttactgtattggatgtgggggatgct
tatttttcatgcccccctagatgaaaattttaggaaatatacagcattcactatacctagtgtgaataatgagaca
ccaggaatcaggtatcaatacaatgtactcccgcagggatggaaaggatcaccagccatatttcaagctagtatg
acaaaaattctagagccttacagaaagcagaatccagagataattatttaccaatacatggatgatttgtatgta
ggctcagatttaaaaattgagctacacagacaaaaggtagaagagctgagagatcatctactaagatggggattt
actacccctgacaagaaacatcagaaggaaccaccattcctctggatggggtatgagctccatccagacaaatgg
gcagtacagcctatacagttaccagacaaggagagctggactgtcaatgatatacaaaaactagtgggaaagtta
aattgggcaagtcaaatatatccaggaattaaagtaaagcaattatgcaagctaattagaggaacaaagagttta

acagaggtagttaccctcacaaaagaagcagaattagaattggctgaaaacagggagatattggcaaacccagta
catggggtctattatgacccttcaaaagaattgatagcagaagtacaaaaacagggacagagtcagtggacatat
cagatatatcaagaacagtttaagaatttaaagacaggaaaatatgctaaaacaagatcagcccataccaatgat
gtaaagcaattaacagaagtagtgcaaaaaatagcattagaaagcattgttatctggggtaagataccaaagttt
agactgcccatacaaaaggaaacatgggaagcctggtggactaattattggcaggctacctggatccctgaatgg
gaatatgttaatacccctcccctagtaaaactctggtaccagttagaaacagaaccaataacaggtgcagagaca
ttctatgtagatggggcagctaatagagataccaagttagggaaagcagggtatgtaacagataggggaaacaa
aagataatctccctaacagaaacaactaatcagaaagcagagttacaagctattcagctagctttacaggattca
ggatcagaagtaaacatagtaactgactcacaatatgcattaggaattatccaaggccagccagataaaagtgaa
tcagaaatagtaaatcagataatagaaagtttaataaacaaggaaagggtctatctctcatgggtacctgcacac
aaaggaattggaggaaatgaggaagtagacaaattagtcagttcagggattagaagagttctcttcctggatgga
atagacaaagcacaggaagagcatgaaaaataccataataattggaaagcaatggccagtgactttaatataccc
ccaatagtagcaaaagaaatagtagccagttgtgataaatgtcaattgaagggagaagcaatgcatggacaggta
gactgcagtcctggaatatggcaactagattgcacccacctagaaggaaagatcatcctagtagcagtacatgtt
gccagtggctacatagaagcagaagtcatacctgcagaaacagggcaggagacagcatacttcatattaaaatta
gcaggaagatggccagtaaaagttatacacactgataatggcagcaactttaccagtaatgcagtcaaggctgca
tgttggtgggcacagatccaacaggaatttggaattccctacaatccccaaagtcaaggagtagtagaatccatg
aataaagaactaaagaagattataggacaagtaagagatcaggcagaacatttaaagacagcagtacaaatggca
gtattcattcacaattttaaaagaaaaggggggattggggggtacagtgcaggagagagaataatagacatcata
gcaacagacatacaaactacagaactacaaaaacaaattttaaaagttcaaaaatttcgggtttattacagggac
agcagagatcctatttggaaaggaccagcaaaactactttggaagggtgaaggagcagtagtgcttcaagatcag
gaagaaataaaggtagtcccacgtagaaaagcaaaaattattagggatttggtaaaacaccacatgtatgtgtcc
aaaaaagcaaaaaattggttttatagacatcattatgaaagtacaagtccaaaagtaagctcagaaatacacatt
ccaataggagatgctaaattagtaatagtaacttattggggtctgcaaccaggagaaagagactggcatctggga
catggagtgtctatagaatggagacagagaagatatagtacacagatagatcctgagttggcagaccacctgatt
catttgcattattttaattgttttgcagaatctgccataaggaaggccctattaggacacatagttagccctaag
tgtgaatatccagcaggacacttaaaggtaggctccttacaatatttggcaatatcagccttgttcaaaagcaac
ccaaaaagaccacccttgcctagtgtaagtaaactaacagaggatgtgctagaagaacttaaacaagaagcagta
agacattttcctagacagtggcttcatggcttaggacagtacatatacaacacatatggagatacctgggcggga
gttgaagccataataaggattctgcaacaattgctgtttattcatttcagaattggctgtcaacatagcaggata
ggcattgtggttcgaagaaggacaatagagccctggaatcacccaggaagccaacctaaaactgcatgtaacaat
tgttattgtaaaagctgtagctttcattgtccactttgcttcatgaaaaaaggcttaggcatttccagtaaggag
gaactgtgggtgacagtgtactatggagtacctgtatggagagatgcagaaacaactctcttttgtgcatcagat
gcaaaggcacaagaaacagaggcacataatatctgggcctctcaagcttgtgtacccacagatcctaaccctgaa
gaaatacacctaccaaatgtaacagaatactttgatatgtggaaaaataatatggtggaacaaatgcaggaagat
ataattagtttgtgggatcaaagtttaaaaccatgtgtaaagttgactcctctctgtgttactttaaactgcact
aattgtatttggggaaatggcacttttagggaatggaatgacacaagggaagacttgagaaactgcagctttaat
gctggggatataagagacaagaaaaagagggtccattcactattttatgctgaagatattatactaatagaccgc
accagaactacaaagcagggaaattcaacacaagcccccgccacgacgcctatagcgagtaccaaggtacccgat
agcaatgacacatgcagccccaacaatactctagaatatagacttataagttgtaatacctcagtcataacccag
gcctgcccaaaaacttcttttgaaccaatacctatacactattgtgctccagctggttttgctcttttaaaatgt
aatgatacaaattttagtggacagggtatatgtagaaatgtcagcacagttcattgtacacatggaattaggcca
gtggtgtcaactcacctacttttaaatggcagtttagcaaaaggggatatcatgataagggtaaagaacaaaaac
caaagtactgacaccattatagtacaattgaacaagagtataaatattacttgtattagaccagggaataacacc
ataaaaggcattcctatagggccaggacaacacttctatggtacagaaacggtagtaggtgatacaaggaaagcc
tactgcaaaatcaatggaacacaatggtttgatacattaaagagagtagcttgggaattaaaggaaacattcaat
cttaccagagtaacatttgatcaagcatcacccggagatccagagatacaaaaacactacttcaattgtggagga
gaattcttttattgtgagacatcttcattgtttaatgctacaatagatattacaccaaatactacactgaatgag
acgacctgggattatccagctgagaacaatattaaccaagaaatgataatccattgtagaataaaacaaattgta
tcaatgtggcagagagtgggaaaaggaatattttctcctcctattagaggaaccattcaatgtaactccactatt
acaggactactgctaactcgcgatggtaataacaaagcagatagtgagaacaaaacaaggattgaaacaggacag
aacaaagcagaagagattaataggactgacattttttagacctgcaggaggagaaatgagagacaattggagaagt
gaattatatagatacaaggtggtgaaaattgaaccactaggactagcacccaccaaggcaaaaagaaggactgta
aatagagaaaaaagaggaatctggggatgctcaggaaaactcatctgcactactgatgtaaagtggaatgaaaca
tggagtaataaatcttataatgagatctgggataacatgacatggatgcagtgggacaaagaagtcagaaattac

acagaaaccatttatgatttaatagaacaagcacaaaaccaacaggagtacaatgaaaaaagtttgttagagctt
aaccagtgggcaagcctgtggaattggtttgatatatcaaattggctgtggtatataaaaatattcattatgata
gtaggaggattaataggctgtagaatagtttttgctttgctatctatagtaaatagagtcaggcagggatactca
cctctgtcatttcagggtgttctacagtattggggaagggaactaaaagttagtgctataagcttgcttgataca
acagcaatagcagtagcagaaggaacagataggataatagaagtagcacaaagatttggtagaggcatacttaat
atccccacacggattagacaaggattagaaagagctttgctataaatgggagccaaatggtcaaagagcagccta
gtaggatggcctgaggtgagagaaagaatcagacaaacacaaccagcagcagaaggagtaggaccagtgtcaaga
gacctagacaatcatggagcaatcacagtcagaaatactccacaaactaatcaaacccttgcctggttagaagag
cagaaagaggaagaagtaggtttcccagtaaggcctcaggtaccattaagaccaatgaccttcaaaggtgcgttt
gatctcagccacttttaaaagaaagaggggactggaagggctaatttattccagaagaagacaagagatcctt
gatctctgggtttatcacacacaaggcttcttcccagactggcaaaactacacaccaggaccaggcgtcagatat
ccactgacctttggctggtgcttcaagttagtaccagtgcctccagaggaagtggaaagagccaatgaaggagag
aacaatgtgctgctgcatccaatgtgccagcatggcatggaagatgaagatcgagaggtcctgcaatggcgcttt
gacagtcacctcgccctgagacacatagccagagagcaacatccggagtactacaaagactgctga
>#62 AJ302647 7494 nt
atgggtgcgagagcgtctgtgttgtcagggagcaaattggatacatgggaacaaattaggttaaagccaggatgt
aaaaagaaatacagactaaaacatttagtatgggcaagcagggagctggaaagattcgcatgtaatcctgagcta
ctagaaactgcagagggcaatgaggaactgttacagcagttagagccagctctcaagacagggtcagaaagcctg
cagtcactctggaacacaatagcagtgctctggtgtgttcacaaaagatttaaagttgaagatacacagcaggca
atacagaaactaaaggaagtaatggggagcaggaagtctgcaggtgccgctaaggaagacacaagcgcaaggcag
acgggtcaaaactaccctgtagtagcaaatgcacagggacaaatggtacatcagtccctctcccccaggacttta
aatgcatgggtaaaggcagtagaagaaaaggcctttaaccctgaaatcatccctatgttcatggcattgtcagag
ggagctattccttatgatactaataccatgctaaatgccataggaggacatcaaggggctttacaagtgctaaaa
gaagtaatcaatgaggaagcagcagaatgggatagaactcacccacaagcggcagggccattgcctccagggcag
ataagggaaccaacaggaagtgacatcgctgggacaactagcacccagcaagagcaagttcactggattactagg
cccaaccaacctatcccagtaggagacatctatagaaaatggatagtgttagggttaaacaaagtagtaaaaatg
tacagcccagtgagcatcttagatattagacagggaccaaaggaaccatttagagattatgtagatcggttctac
aaaacattaagagctgaacaggcaactcaagaagtaaaaaattggatgacagaaaccctgcttgttcaaaatgcc
aacccagattgcaaacagattttgaaatcattagggccaggagctaccttagaagaaatgatgatagcttgtcag
ggagtaggaggaccaactcataaggccagagtactagcagaagcaatgtctgcagcccaagatctgaaggagga
tactcagcagtatttatgcaaagagggcaaaacccaggtaggaaagggcctataaaatgtttcaattgtggaaaa
gaaggacatctagcaagaaattgtcgagcacctagaaagaaaggttgctggaaatgtggacaggaaggtcaccaa
atgaaagattgcagaaatggaaaacaggcaccagcaagggttgggggccatctatgtgaagttttactggataca
ggggcagatgatacagtactaaataacatacaattggaaggaaaatggacaccaaaaatgatagggggtatagga
ggttttataaaggtaaaagaatataatcaagtgccagtagaaatagagggaagggaagtactgggaacagtattg
gtgggacctactcctgttaatattattggaagaaacatattgacaggattgggttgtacactaaatttccctata
agtcccatagccccagtaccagtaaaactaaaaccaggaatggatggaccaaaaataaaacaatggcccctatct
aaagaaaaaatagaagccttgacagcaatatgtcaggaaatggaacaagaaggaaaaatttcaagaataggacct
gaaaatccttataatacacctatctttgctataaaaaagaaagatagcactaagtggagaaagctggtagacttt
agggaattaaacaagagaacacaagatttctgggaggtacagttaggtatcccacatccgggggtttaaagcaa
aagcaatctgttacagtcctagatgtaggagatgcttatttctcatgccccttagacccagatttcagaaaatat
actgccttcactattcctagtgtgaacaatgagaccccaggaataagataccagtacaatgtcctcccgcaggga
tggaaagggtcgccagctatattccaaagttcaatgacaaaaattttagatccatttaggaaaaacaacccagaa
ttagaaatttgtcaatacatggatgacttatatgtaggatcagatttaccccctgacagaacatagaaagagagta
gaattgcttagagaacacttatatcagtggggattcactaccccctgataaaaagcatcaaaaggaacctcccttt
ttgtggatggggtatgagctccatccagacaaatggacagtacaacccatccaattgcctaacaaggaggaatgg
acagtaaatgatatacaaaaactagtaggaaaattaaattgggcaagtcaaatctatcaaggaattagagtaaaa
gaattgtgtaagttaattagaggcaccaagtcattgacagaagtagtacctttaagtaaagaggcagagctagaa
ttagaggagaatagagaaaagttaaaagaaccagtgcatggtgtatactatcaacctgacaaagacttatgggtt
aatattcagaagcagggaaaaggacaatggacttatcagatatatcaggatgaacataagaacctcaaaacaggg
aaatatactaagcaaaaggcctctcacacaaatgatataagacaattagcagaagtactccagaaggtgtctcaa
gaagctataattatctggggaaaattgcctaaatttaagctgccaatcactagagaaacttgggaaacatggtgg
gcagactattggcaagccacctggattccagaatgggaatttgtcagcacagccccattgatcaaattatggtac
cagttagaaagtgaacctattatagggggcagaaacctattatgtagatggagcagctaacagagatacaaaacta
ggaaaagcaggatatgttacagaaaaagggaaacagaaaatagtaaaattagaggagaccaccaatcaaaaggct

gaattaatggcagtattattagccttacaggattccaaggaaacagtaaatatagtaacagattcacaatatgta
ttgggcatcatctcctcacagcctacacagagtgagtcccctctagttcagcagataatagaggaactaacacaa
aaggaacaggtgtttcttacatgggttcctgcccataaaggcataggaggaaatgaaaaaatagataaattagta
agcaaggatattagaagagtcctattcctagaaggaatagaccaggcacaagaagatcatgaaaagtatcatagc
aattggagagcattagctagtgattttggattgccaccagtggtggccaaagaaatcattgctaattgtcctaaa
tgtcatataaaaggggaagcaattcatggtcaggtagactacagtccagaagtatggcaaatagattgcacacat
ctagaaggcaaaatcataatagttgctgttcatgtggcaagtggattcatagaagcagaagtaataccagcagaa
acaggacaagaaactgcctacttcctgttaaaattagctgcaagatggcctgttaaaataatacatacagacaat
gggcctaatttcacaagtgcaaccatgaaggctgcatgttggtggacaggcataaaacatgagtttggaatacca
tataatccacaaagtcaaggagtagtagaggccatgaacaaggaattaaaatcaattatacagcaggtgagggac
caagcagaacacttaaaaacagcagtacaaatggcagtatttgttcacaattataaaagaaaaggggggattggg
gggtacactgcaggagaaaggataatagacatattagcatcacaaatacaaacaacagaattacaaaaacaaatt
tttaaaattcaaaaatttcaggtctattacagagacagcagagatcctatttggaaaggaccggcacagctcctg
tggaaaggtgagggagcagtagtcatacaagataaaggagacattaaggtagtaccaagaagaaaggcaaaaata
atcagacattacaggtcagggaagaccagagactggtattacagacaccattttgaatctaaaaatccaagagtc
agctcaggtgtatatattccagtaggagggccttggatagtagtgaccacatattggggattgatgccaggggaa
agagatgaacatttgggacatggggttagcatagactggaggtacaggaagtatacaacacagattgaccctgaa
acagcagacagaatgatacatacatattattttgcctgttttacagagtcagcaatcaggaaagccatcttaggg
cagagagtactgaccaggtgtgaatactctgcaggacatagtcaggtagggacactgcaactactagctctaaga
gtggtagtaaaagaaaaaagacataagcctcccctacccagtgtccagaagttaacagaagatctcctagaagaa
ctaaaagcagaagcagtaagacatttccctagaccttggctacaggccttgggacaatacatttatgacacttat
ggggacacttgggtaggagttatggccattataagaatcttacaacacctgctatttgcccattttagaattgga
tgccaacatagtagaataggaattaacccatctaacacaagaggaagaggaagaagaatgccccccttggcatcac
cctggaagtcagccccagaccccttgtaataagtgctattgcaaagcgtgctgctaccattgctatgtttgtttt
gcaagcaagggtttgggactctcccaacactatgcaacagtctatgctggggtacctgtatgggaagaggcaacc
ccagtactattctgtgcttcagatgttaacttaacgagcactgagcagcataatatttgggcatcacaagcctgt
gttccaacagacccctctccatatgaatatcctttaaaaaatgtaacagataacttcaatatatgggaaaattac
atggtagaacaaatgcaggaagatattattagtttatgggaacagagtttaaaaccttgtgttcaaatgactttt
ctgtgtgtacaaatgaattgtacaaatgtaaatgatgagaccaacagctccgtaaagaatgataccagcagctca
gagaaccttatgaaaaagtgtgagtttaatgtaaccacagttctcaaagacaaaaaggagaaacaacaggctcta
ttctatgtatcagatttgatgaaagtgaatgaaaataatgacacaatgtatacattaattaattgtaattccaca
accattaagcaaacctgtccaaaggtatcgtttgaggcaattccaatacactattgtgctccagcgggatatgcc
atctttaagtgtaacaacacagggtttaatggaacaggcccatgcacaaatgttacagtagttacttgtacacat
ggcatcagaccaacagtaagtactcaactaatattaaatgggacaatctctgaaggaaagataagaattatggga
aaaaatatttcagacactgggaaaaatatcatagtgaccataaattctactataaacatgacctgtgagagacca
ggaaatcagacagtacaaaagatactaacaggtccagtggcctggtacagcatgggcctgaaaaataacctaact
aactcaagggcagcctcttgcaagtataacagctctgtttgggaagaagccttaaaacaaacagctgaaaggtat
ttagaacttatgaacaatacaaatacagttaacataacattcaatcacagcactggtggagacccagaggtaacc
catttgcattttaactgtcatggagaattcttctattgtaacacatctcagatgtttaattataccttttcatgt
acaagaaccaactgtattagacaaagtaacagtagcattaatggcacaatatcttgcaggataaaacaggtggta
aggtcatggatacagggagggtcgggactttatgcacctcccaggccaggttacctaacatgcaactcatccata
actggaatgattctacaattggataagacatggaaccgcaccaacaacagcgaatccacatttagaccaataggg
ggagatatgaaagatatatggagaactgaattgttcaaatacaaagtagtaaagataaaaccttttagtgtggca
cctacaaaaattgcaagaccagtcataggcactggcactcgaagagaaaaaagaaacctatggggctgtaaagga
aggctagtctgctacacatcagtaaaatggaacaggacatggacaaataataatactgatttagatacaatttgg
ggaaatctaacatggcaggaatgggatcagcagataagcaacataagcgccaccatatatgatgaaatacaaaag
gcacaagtacagcaggaacacaatgagaaaaagttgctggagttagatgaatgggcttctatttggaactggctg
gacataactaaatggttgtggtatataaaaatagcaataatcatagtaggggcactaataggggtgagaattgtt
atgatagtacttaatctagtgagaaacattaggcatggatatcaacccctctcatttcaggcttgcagaatttgc
atagctgtaatacaatactggctacaagagttgcagaatagtgctacaagcctactagatacccttgcagtggca
gttgccaattggactgacggtataattttagggctacaaaggataggaagaggaattcttaacatcccaagaaga
attagacagggcttagaaagagctttattgtaaatgggaaacgtattgggtaaagataaatttaaggatggtca
gcagtcagagaaagaatgagaaaaacttcccctgagcctgaaccatgtgcacctggagtaggacaagtctccagg
gaattagcagctagaggagggatatcaaattcccatactcctcaaaacaatgcagcccttgcattcctagaaagt
caccaagatgaagacgtaggcttcccagtaagacctcaagtgcctctaaggccaatgacctataaaggagcattt

gacctcagcttctttttaaaagaaaagggaggactggatgggttaatttactcccctgaaagagcagagatcctg
gatctttgggtgtatcacactcagggattcttccctgattggcagaattacacaccaggaccaggaacaagattc
ccactgacatttgggtggctatttaagctagtaccagtgtcagaagctgaggcagaagaactaggaaataagtgt
gacagggctaaactcctgcatccagtttgcaaccatggctttgaagatccacacaaggagatgctgaaatggcag
tttgatagatcactaggcagcacccatgttgctctgataacccacccagagctctttctcaaggactaa
>#63 AJ302646 7548 nt
atgggtgcgagagcgtctgtgttgacagggagcaaattggatgcatgggaacaaattaggttaaagccaggatgt
aaaaagaaatacagactaaaacatttagtatgggcaagcagggagctggacagatccgcatgtaatcctgagcta
ctagaaactgcagagggcaatgagaaactgttacagcagttagagccagctctcaagacagggtcagaaagcctg
cagtcactctggaacactatagcagtgctctggtgtgttcacaacagaattaaagttgaagatacgcagcaggca
atacagaaactaaaggaagtaatggggagcaggaagtctgcaggtaccgctaaggaagacacaagcgcaaggcag
acgggtcaaaactaccctatagtaacaaatgcacagggacaaatggtacatcagtccctctcccccaggacttta
aatgcatgggtaaaggcagtagaagaaaaggcctttaaccctgaaatcattcctatgttcatggcattgtcagag
ggagccattccctatgatactaataccatgctaaatgccataggaggacatcaaggggctttacaagtgctaaaa
gaagtaatcaatgaggaagcagcagaatgggatagaactcacccaccagcggcagggccattgcctgtagggcag
ataagggaaccaacaggaagtgacattgctggaacaactagcacccagcaagagcaagttcactggattaccagg
cccaaccaacctatcccagtaggagacatctatagaaaatggatagtgttaggattaaacaaagtggtaaaaatg
tacagcccagtgagcatcttagatattagacagggaccaaaagaaccatttagagactatgtagatcggttctac
aaaacattaagagctgaacaggcaactcaagaagtaaaaaattggatgacagaaaccctgcttgttcaaaatgcc
aacccagattgcaaacagattttgaaatcattagggccaggagctaccttagaagaaatgatgatagcttgtcag
ggagtaggaggaccaactcataaggccagggtattagcagaagcaatggctgcagcccaagatctgaagggagga
tacacagcagtatttatgcaaagagggcaaaacccaagtaggaaagggcctataaaatgtttcaattgtggaaaa
gagggacatctggcaagaaattgtcgagcacctagaaagaaaggttgctggaaatgtggacaggaaggtcaccaa
atgaaagattgcaaaaatggaagacaggcaacagcaagggttgggggccatctatgtgaagttttactggataca
ggggcagatgatacagtactaaccaacatacaattggaaggaaaatggacaccaaaaatgataggggggtatagga
ggttttataaaggtaaaagaatataatcaagtgccagtagaaatagagggaagggaagtactgggaacagtattg
gtgggacctactcctgttaatattattggaagaaacatattgacaggattgggttgcacactaaatttccctata
agtcccatagccccagtaccagtaaaactaaaaccaggaatggatggaccaaaaataaaacaatggcccctatct
aaagaaaaaatagaagccttgacagcaatatgtcaggaaatggaacaagaaggaaaaatttcaagaataggacct
gaaaatccttataatacacctatctttgctataaaaaagaaagatagcactaagtggagaaagctggtagacttt
agggaattaaacaagagaacacaagatttctgggaggtacagttaggtatcccacatccggggggtttaaagcaa
aagcaatctgttacagtcctagatgtaggagacgcttatttctcatgccccttagacccagatttcagaaaatat
actgccttcactattcctagtgtgaacaatgagaccccaggaataagataccagtacaatgtcctcccgcaagga
tggaaagggtcgccagctatattccaaagttcaatgacaaaaattttagatccatttaggaaagacaacccagaa
ttagaaatttgtcaatacatggatgacttatatgtaggatcagatttacccctgacagaacatagaaagagagta
gaattgcttagagaacacttatatcagtggggattcactacccctgataaaaagcatcaaaaggaacctcccttt
ttgtggatggggtatgagctccatccagacaaatggacagtgcagcccatccaattgcctaacaaggaggaatgg
acagtaaatgacatacaaaaactagtaggaaagttaaattgggcaagtcaaatctatcaaggaattagagtaaaa
gagttgtgtaagttaattagaggcgccaagtcattgacagaaatagtacctttaagtaaagaagcagagctagaa
ttagaggagaatagagaaaagttaaaagaaccagtgcatggtgtatactatcaacctgacaaagacttatgggtt
aatattcagaagcagggatggggacaatggacttatcagatatatcaggatgaacataagaacctcaaaacaggg
aaatatactagacaaaaggcctctcacacaaatgatataagacaattagcagaagtactccagagggtgtctcaa
gaggctataattatctggggaaaattgcctaaatttaagctgccaatcactagagaaacttgggaaacatggtgg
gcggactattggcaagccacctggattccagaatgggaatttgtcagtacacccccattgatcaaattatggtac
cagttagaaagtgaacctattatgggggcagaaacctattatgtagatggagcagctaacagagatacaaaacta
ggaaaagccggatatgttacagaaaaggggagacagaaaataattaaattagaggagaccaccaatcaaagggct
gaattaatggcagtattattagccttacaggattccaaggaaacagtaaatatagtacaagattcccaatatgta
ttgggcatcatctcctcacagcctacacagagtgaatcctctctagttcagcagataatagaggaactaacaaaa
aaggaacaggtgtatcttacatgggttcctgcccataaaggcataggaggaaatgaaaaaatagataaattagta
agcaaggatattagaagagtcctattcctagaaggaatagaccaggcacaagaagatcatgaaaagtatcatagc
aattggagagcattagctagtgattttggattaccaccagtggtggccaaagaaatcattgctaattgtcctcaa
tgtcatataaaaggggaagcaattcatggtcaggtagactgcagtccagaagtatggcaaatggattgcacacat
ctagaaggcaaaatcataatagttgctgtccatgtggcaagtggattcatagaagcagaagtaataccagcagaa
acaggacaggaaactgcctacttcctgttaaaattagctgcaagatggcctgttaaggtaatacatacagacaat
gggcctaatttacaagtgcagccatgaaggctgcatgttggtgggccaacataaaacatgagtttggaatacca

tataatccacaaagtcaaggagtagtagaggccatgaacaaggaattaaaatcaattatacagcaggtgagggac
caagcagaacacttaaaaacagcagtacaaatggcagtatttgtgcacaattataaaagaaaaggggggattggg
gggtacactgcaggagaaaggataatagacatattagcatcacaaatacaaacaacagaattacaaaaacaaatt
tttaaaattcaaaaatttcaggtctattacagagacagcagagatcctatttggaaaggaccggcacagctcctg
tggaaaggtgagggagcagtagtcatacaagataaaggagacattaaggtagtaccaagaagaaaggcaaaaata
atcagacattacaggtcagggaagaccagagactggtattacagacaccattttgaatctagaaatccaagagtc
agttcaggtgtatatattccagtagggggccttggatagtagtgaccacatattggggattgatgccaggggaa
agagatgaacatttgggacatggggttagtatagaatggaggtacaagaagtataaaacacagattgaccctgaa
acagcagacagaatgatacatatatattattttgcctgttttacagagtcagcaatcaggaaagccatcctaggg
cagagagtactgaccaggtgtgaataccctgcaggacatagtcaggtagggacactgcaactactagctctaaga
gtggtagtaaaagaaaaaagaaataagcctcccctacccagtgtccagaagttaacagaagatctcctagaagaa
ctaaaagcagaagcagtaagacatttccctagaccttggctacaggccttgggacaatacatttatgacacttat
ggggacacttgggtaggagttatggccattataagaatcttacaacaactgctatttgcccattttagaattgga
tgccaacatagtaggataggaattaccccatctaacacaagaggaagaggaagaagaaatggatccagtagatcc
tgaatgccccccttggcatcaccctggaagtcagccccagaccccttgtaataagtgctattgcaaagcatgctgc
taccattgctatgtttgttttgcaagcaagggtttgggaatctcccagcactatgcaacagtctatgctggggta
cctgtatgggaagaggcaaccccagtattattctgtgcttcagatgctaacctaacaagcactgagcagcataat
atttgggcatcacaagcctgtgttcccacagacccctctccatatgaatatcctttaaccaaggtgacagataat
ttcaatatatggaaaaattacatggtagaacaaatgcaggaagacattattagtttatgggaacagagcttaaaa
ccttgtgttcaaatgactttcctgtgtgtacaaatgaattgtacaaactatgtacaagggaattatacaaacaat
agctccataaataatgataccagcagtccagagaaccttgtgaaacagtgtgaatttaatgtaaccacagttgtc
aaagacaaaaaggagaaaaaacaggctctattctatgtatcagatttgatgaagataaatgaagcaaatgacaca
aaggacatgtatacattaattaattgtaactccacaaccattaagcaagcctgtccaaaggtatcgtttgagcca
attccaatacactattgtgctccagcgggatatgccatctttaagtgtaacagcacagagtttaatggaacaggc
ccatgcaacaacattacagcagttacttgtacacatggcatcaaaccaacagtaagtactcaactaatattaaat
gggacactctctgaaggaaacataagaattatggaaaaaatatttcagacaatatgaaaaatatcatagtgacc
ctaaattctactataaacatgacctgtgtgagacaaggaaatcagtcagtacaagagatacaaataggtccaatg
gcctggtacagcatgagcctggcccaagagggaaaaccaaataactcaaggatagcctattgcaagtataacatc
tctgattgggaaaaagccttaaaacagacagctgaaaggtatttagaccttaggaacaatacaaatacagttaac
ataacattcgagcgcagcattggtggagattcagaggtaacccatttgcatttcaactgtcatggagagttcttc
tattgtaacacatctaagatgtttaattataccttttcatgtataggaaccaactgtactagtaaccaaaatagc
agcaacagtaatgacactcggatatattgcaggataaaacaggtggtaaggtcatggatacagggaggtcagga
ctctatgcacctcccaggaaaggtaacctaacatgcagctcactcataactggaatgattctacaattggatatg
ccatggaacagcaccaacaacagcaacgccacatttagaccaacaggggggagatatgaaagatatatggagaact
gaattgttcaaatacaaagtagtaaaggtaaaaccttttagtgtggcacctacaaaaattgcaaggccagtcata
ggcactggcactcaaagagaaaaaagaaacctatggggctgtaagggaaagctaatctgctacacatcagtaaaa
tggaacacgacatggacaaattgcacaaataccaataagttagatgatatttgggacaagctaacatggcagcaa
tgggatcagcagataagcaacgtaagttccatcatatatgaggaaatacgaaatgcacaagtacagcaggaacaa
aatgagaaaaagttgctggagttagatgaatgggcttctatttggaactggctggacataactaaatggttgtgg
tatataaaaatagcaataatcatagtaggggcactaataggggtgagaattgttatgatagtacttaatctagtg
aaaaacattaggcagggatatcaacccctctcatttagagcttgcagactttgcatagctgtaatacaatactgg
ctacaagagttgcagaatagtgctacaagcctactagataccattgcagtggcagttgccaattggactgtcaca
ataattttagggatacaaaggataggaagaggaattcttaacatcccaagaagaattagacagggcttagaaaga
agtttattataaatgggaaacgtattgggtaaagatatatttaagggatggtcagcagtcagagaaagaatgaga
ggaacttcccctgatcctgaaccatgtgcacctggagtaggacaaatctccagggaattagcagctagaggaggg
ataccaagttcctatactcctcaaaacaatgcagcccttgcattcctagaaagtcaccaagatgaagaagtaggc
ttcccagtaagacctcaagtgcctctaaggccaatgacctataaaggagcatttgacctcagcttctttttaaaa
gaaaagggaggactggatgggttaatttactcccataaaagagcagagatcctggatctttgggtatatcacact
cagggattcttccctgattggcagggctacacaccaggaccagggccaagattcccactgacatttgggtggtta
tttaagttagtaccagtgtcagaagctgaggcagaggaactaggaaacaagtgtgagagggctagcctcctgcat
ccagcttgcaaccatggctttgaagacaaccacgggcaaatactgaaatggcagtttgatagatcactaggcagc
acccatgttgctatggtaaccaacccagagctctttaacaaggactaa
>#64 AY169812 7479 nt
atgggtgcgagtgcgtccgtgttgacagggagtaaattggatgcatgggaacgaattcggttgaagccaggatct
aaaaagaaatatagactaaaacatttagtatgggcaagcagggagctggacagattcgcatgtaatcctgagcta

ctagaaactgcagagggcaatgagaagttgttacaacagttagaaccagctctcaagacagggtcagaaagcctg
cagtcactctggaacacaatagcagtgctctggtgtgttcacaacagatataaagttgaagatacgcagcaggca
atacaaaagttaaaggaagtaatggggagtaggaagtctgcaagtgccgctaaggaagaaacaagtgcaaggcag
acgggtcaaaattaccctgtagtaccaaatgcgcagggacaaatgatacatcaggccctctcccccaggacttta
aatgcatgggtaaaggcagtagaagaaaaggcctttaaccctgaaatcattcctatgttcatggcattgtcagag
ggagctattccctatgatattaataccatgctaaatgccataggaggacatcaaggggccttacaagtgctaaag
gaagtaatcaatgatgaagcagcagattgggatagaagtcacccaccagtggtagggccgttgccaccagggcaa
ataagggaaccaacaggaagtgacattgctgggacaactagctcccagcaagagcaagttcactggattactagg
gccaaccaccctatcccagtaggagacatctatagaaaatggatagtgttgggactaaacaaaatggtaaaaatg
tacagtccagtgagcatcttagatattaaacagggaccaaaagaaccatttagagattatgtagatcggttctac
aaaacattaagagctgagcaagcatctcaagatgtaaagaattggatgacagaaaccctgcttgttcagaatgcc
aacccagattgcaaacagattttgaaagcattagggccaggagctaccttagaagaaatgatggtagcctgtcag
ggagtaggaggaccaactcataaggccaaattgctagctgaagcgatggttaccgcccatcaagatctaaaagga
ggatacacatcagtattcatgcaaagaggacaaaatccaatgaggaagggacctataaaatgtttcaattgtgga
aaagagggacatatagcaaaaaattgtcgagcacctagaaagaaaggttgctggaaatgtggacaggaaggtcac
cagatgagagattgcagaaatggaaaacaggcaacagcaaaagttgggggccatctatgtgaagtkttrctggat
acaggggcagatgatacagtactaaacaacatacaattggaaggaaaatggacaccaaaaatgatagggggtata
gggggytttataaaagtaaaagaatatgatgatgtgacagtagaaatagagggaagacaagtacagggaacagta
ttggtgggacctacccctgttaatattattggaagaaatatattgacaggattaggttgtacactaaattttcct
ataagccccatagccccagtrccagtaaaattaaaaccaggaatggatggaccaaaaataaaacaatggcccta
tctaaagaaaaaatagaagcyttgacagcaatatgtcaggaaatggaacaagaaggaaaaatttcaagaatagga
cctgaaaatccttataatacacctatctttgcaataaaaaagaaagatagtactaagtggagaaaattggtagac
tttagggaattaaacaagagaacacaagatttctgggaggtacagttaggtatcccacatccaggggggtttaaag
caaaagcaatctgttacagtcctagatgtaggagatgcttatttctcatgccccttagacccagatttcagaaaa
tatactgccttcactattcctagtgtgaacaatgagaccccaggaataagataccagtacaatgtcctcccgcaa
ggatggaaaggttcaccagctatattccaaagttcaatgataaraattctagatccatttaggagggacaaccca
gaattagaaatttgtcagtacatggatgacctatatgtaggatcagatttacccctgacagaacatagaagaagg
gtagaaatgcttagagaacacttatataagtggggattcactacccctgataaaaagcatcaaaaggaacctccc
tttytgtggatggggtatgagctccatccagacaaatggacagtacagcccatccaattacctaacaaggatgtg
tggacagtaaatgatatacaaaaattagtaggaaagttaaattgggcaagtcaaatctattcaggaattagagta
aaagaattgtgtaaattaattagaggcaccaaatcattgacagaagtagtacctttaagtaaagaggcagagctg
gaattagaggagaacagagagaagctaaargaaacagtgcatggtgtatactatcaacctgamaaagamttatgg
gttcagattcagaagcatgaawcagggcaatggacttaccagatatatcaggatgaatataagaacctcaaaaca
ggaaartatactaggcaaaaagcctcccacacaaatgatataagactattaacagaagtagtccagaaggtggct
caagaagccatagttatctggggaaaattgcctaaatttaagctgccagtcactagagaaacttgggaaacatgg
tggacagactwttggcaagccacctggattccagaatgggaattcgttagtacacccccattgatcaaattatgg
tacaggttagaaagtgaacctatcataggagcagaaacctattatgtagatggagcagctaatagaaatacwaaa
ctagggaaggcaggatatgttacagaacaagggaaacagaaaatagtaaaattagaggagaccaccaatcaaaag
gctgarttaatggcgatactattagccctacaggaytccaargaaacagtaaatatagtaacagattcacaatat
gcattgggtatcatctcctcccaacctacrcagagtgattccccattagttcagcagataatagaggaactaaca
aaaaaggaacaggtgtatcttgcatgggttcctgctcataaaggcataggaggaaatgaaaaaatagataaatta
gtgagcaaggatattagaagagtcctattcytagaaggaatagaccaggcacaagaagatcatgagaaatatcat
agcaattggaaagcattagctagtgactttgggctaccaccagtagtggccaaagaaatcattgctagttgtcct
aaatgtcatataaaaggagaagcaatgcatggtcaggtcgactgcagtccagaagtatggcaaatagattgcaca
catttagaaggcaaggtcataatagttgctgtccatgtggcaagtggattcatagaagcagaagtaataccagct
gaaacaggacaagaaactgcctacttcctgttaaaattagctgcaaggtggcctgttaaaattatacatacagac
aacgggcctaattttacaagtgcaaccatgaaagctgcatgttggtggaccaacatacaacatgagtttggaata
ccttataatccacaaagtcaaggagtagtagaagccatgaataaggaattaaaatcaattatacagcaggtgagg
gaccaagcagaacacttaagaacagcagtacaaatggcagtatttgttcacaattttaaaagaaaaggggggatt
gggggtacactgcaggagaaaggataatagacatactagcatcacaaatacaaacaacagaattacaaaaacaa
atttttaaaattcaaaatttcaggtctattacagagacagcagagaccctatttggaaaggaccggcacagctc
ctgtggaaaggtgagggagcagtagtcatacaagataaaggagaaattaaagtagtaccaagaagaaaggcaaaa
ataatcagacattacaggtctgggaaggccaggaactggtattacaggcatcattttgaatctagaaatccaaga
ttcagttcaggtgtacatattccagtagggatggcttgtatagtagtgaccacatattggggactgatgccaggg
gaaagagaggatcagatgggacatggggttagtatagaatggcagtacaaacagtatacaacacagattgaccct

gaaacagcagacaggataatacatctgtactattttacttgttttacagagtcagcaatcaggaaagccattcta
gggcagagagtattgcccaggtgtgaatacactgcaggacatagtcaggtagggacactgcaactattagctcta
agagcagtagtaaaagcaaaaagaaataagcctcccctacccagtgtccagaaattaacagaagatactctagaa
gaactaaaaacagaagcagtaagacattttcctaggcyttggctacakagcttrggacaatacatttatgagact
tatggggacacttgggtaggagttatggcaattataagaatcttacaacacctgctattcacccatttagaatt
ggatgccaacatagtagaataggaattaacccatctaacaccagaggaagaggaagaagaatgccccccatggcat
caccctggaagtcagccccygaccccttgcaacacttgctattgcaaaagatgctgctatcattgctatgtttgt
ttcacaagaaagggtttgggaatctcccagctgtatgcaacagtttatgctggggtacctgtatgggaagaggca
gccccagtattattctgtgcctcagatgctaacctaacaagcactgagaagcataatgtttgggcatcacaagcc
tgtgttcccacagaccccactccacatgaatatttattaaaaaatgtgacagatarcttyratatatggaaaaat
tacatggtagaacaaatgcaggaagacattattagtctatgggaccagagtttaaaaccttgtgttcaaatgact
ttcatgtgtgtacaaatgaattgtacagacatagaaaacaaaaacagtagctcagaaaaccctatgaaaaagtgt
gagtttaatataaccacagttctcaaggacaaaaaggagaaaaaacaggctttattctatagagcagatttgact
gaaataagagacaacagcacaaacagcacaatatatacattaattaattgtaattccacaaccattaagcaagcc
tgtccaaaggtatcttttgagccaattccaatatactactgtgctccagcaggatatgccatttttaagtgtaac
aatgcagaatttaatggaacaggcaaatgcaacaacatttcagtggtgacttgtacacatggcatcaagccaaca
gtgagtactcaactgatattaaatgggacactctcaaaagaaaaaataagaattatgggacaaaatatttcggcc
actgcacaaaatatcatagtgaccctaagttctgctgtaaacataacctgtgtgagaccagggaataagacagta
caagagatgagaataggtccgatggcctggtacagcatggccataagtggaacaaacagctcaagggtagcctat
tgccagtataacaccactgagtgggaaaaagccttaaaaaacacagctgaaaggtatttggaacttataaataag
acagaagagaatacaacaataatgttcaaccggagccatgatggcgatgtagaggtaacccatttgcattttaac
tgtcatggagagttcttctattgtaacacatctgggatgtttaattatacctttttatgtaatgggaccaactgt
aaaaaccaaagtacccatagcaataatggcacgataccttgcaagttgaaacaggtggtaagatcatggatgagg
ggagggtcgggactctatgcacctcccatcccaggtaacctaacatgcatgtcacacataactggaatgattcta
caaatagatgggccatggaacaacaacaccacaaacaatacatttagaccaatagggggagatatgaaagatata
tggagaaatgaattgttcaactacaaagtagtgagggtaaaacctttagtgtggcacctacatcaattgcaagg
ccagtcataggcactggcactcatagagagaaaagaaacctatggggctgtaarggaagactaatctgctacaca
tcagtaaaatggaacgasacatggagwrgtacaactaatatagaccaaatttggggaaacttaacatggcaggaa
tgggatcagcagatagacaacgtaagtgccrccatatatgaggaaatacaaagggcacaagtacagcaggaacaa
aatgagaaaaagttgctggagttagaygaatgggcttctctttggaattggcttgacataactaaatggttgtgg
tatataaaaatagcaataatcatagtaggagcactaataggtgtgagaattgtcatggtagtgcttaatctagtg
agaaacattaggcagggatatcaacccctctcgttacaggcttgcagaatttgtgcagctgtaatacatttctgg
ctacaagaattgcagaagagcgctactagcctactagatacctgtgcagtagcagttgctaattggactgacggc
ataatcttagggrtacaaaggctaggaagaggaattcttaatatcccccgaagaataagacagggcttggaaaga
agtttattgtaaatgggaaacgtattggggaaagataaatttaaaggatgggcagcagtaagagagagaatgaga
aaaacttcctctgatcctgatcctcaaccatgtgcacctggagtagggccagtgtccagggaactagcagctaga
ggaggagtaccaagttcctatactcctcaaaacaatgcagcccttgcattcctagaaagtcatgaagatgaagat
gtaggtttcccagtargacctcaagtgcctctaaggccaatgacctataaagcagcatttgacctcggtttcttt
ttaaaagaaaagggaggactggatgggttaatttactcccatargagagcagagatcctagatctttgggtvtat
cacactcarggatttttccctgattggcagaactacacaccaggaccagggactagatacccactgacatttgga
tggttatataagctagtaccagtgwcagaaacygaggcaaargaactaggaaacacatgtgagagggctatgctc
ctgcatccagcttgtgcacatggctttgahgatccacacggagaaatactgaaatggcagtttgatagatcacta
gcctccacccatgttgctaagataactcacccagagctcttccccaaggactaa

>#65 AY169816 7449 nt

atgggtgcgagtgcgtctgtgttgacaggaagtaaattggatgcatgggaaagaattaggttaaagccaggatct
aaaaagacatataagctaaaacatttagtatgggcaagcagggagctggacagattcgcatgtaatcctgggcta
ctagaaactgcagagggtaatgagaagctgctacagcagttagagccagctctcaagacagggtcagaaagtctm
cartcactctggaacacaataacagtgctctggtgtgttcacaacagatatcaagttatagacacgcaggaggca
atacaaaagttaaaggaagtcatggggagcaggaartctgcggaggccgctaaagaaaacaaaagcacaaagcag
gagggtcaaaattaccctatagtaacaaatgcgcaggggcaagtaacgcatcagcccatctcccccaggacttta
aatgcatgggtaaaggcagtagaagaaaargcctttaaccctgaaatcattcctatgttcatggcattgtcagag
ggagcaattccctatgatattaatactatgctaaatgccataggaggacatcagggagctttacaagtgctaaag
gaagtaattaatgaggaagcagcagattgggatagaactcacccaccaccggtagggccgttgccaccagggcag
ataaggaaccaacaggaagtgacattgctggaacaactagcacccagcaagagcaaattcaatggattaccagg
cccaatcaacctatcccagtaggagacatctacagaaaatggatagtgttaggactaaacaaaatggtgaaaatg

tacagcccagtgagcatcttagatattaagcagggaccaaaagaaccatttagagactatgtagatcgattctac
aaagtattaagagctgaacaagcatctcaagaagtaaaaaattggatgacagaaaccctgcttgttcaaaattcc
aatccagattgcaaacagattttgaaatcattaggrccaggagctaccttagaagagatgatggtagcctgtcaa
ggagtaggaggaccaactcataaggccagagtactagcagaagcaatggctacagcccagcaagatttgagagga
gggtgcacagcagtattcatgcaaagagggcaaaatcaaggaaggaaaggacctataaaatgtttcaactgtggg
aaagagggacatatagcaaaaaattgtcgagcccctaggagaaagggttgctggacagcaagagttgggggccac
ctatgtgaagttttgctggatacaggggcagatgatacagtattaaccaacatacaattggaaggtaaatggaaa
ccaaaaatgataggaggtataggaggctttataaaggtaaaggaatatgagaatgtgacagtagaaatagaagga
agagaagtacagggaacagtattggtgggacctactcctgttaatattattggaagaaatatattgacaggacta
ggttgtacactaaatttccctataagccccatagccccagtgccagtaagactaaaaccaggaatggatggacca
aaagtaaaacaatggcccctatctaaagaaaaaatagaagccttaacagcaatatgtcaggaaatggarcaagaa
ggaaaaatttcaagagtaggacctgaaaatccwtataatacacctatctttgctataaaaaagaaagatggtacc
aagtggagaaagctggtagactttagagaattaaacaagagaacacaagatttttgggaggtacagttaggtatc
ccacatccgggggtttaaagcaaaagcaatctgttacagtcctagatgtaggagatgcttatttctcatgtccc
ttagatccagatttagaaaatatactgctttcactattcctagtgtgaacaatgagaccccaggaayaagatac
cagtacaatgtcctcccgcaaggatggaaagggtcaccrgctatattccaaagttcaatgacaaaaattctagay
ccatttagaaaagacaacccagaattagaaatatgtcagtatatggatgacttatatgtaggatcagatttaccc
ctgacagaacatagaaaaargattgaattgcttagagaccacttatatcagtggggattcactacccctgataag
aagcatcaaaaggaacctccttttctgtggatggggtatgagctccacccagacaaatggacagtgcagcctatc
caattgcctaacaaggatgtgtggacagtaaatgatatacaaaaactaataggaaagttaaactgggcaagtcaa
atttaccaaggaattagagtaaaagaattgtgtaaattaattagaggcaccaagtcattaacagaagtagtaccc
ttaagtaaagaggcagagctagaattagaggaaaacagagaraggctaaaagaaccagtgcatggggtatattat
caacctgamaaagacttatgggttgatattcagaaacagggagaagggcaatggacttaccagatatatcaggat
aaatataagaacctcaaaacagggaaatatacwaggcaaaaggcctcccacacaaatgatataagacaattagca
gaagtactccagaaggtgtctcargaatctatagttatctggggaaaattgcctaaatttaagctgccagtcact
agagaaatttgggaagcatggtgggcggactattggcaagccacctggattccagaatgggaatttgtyagcaca
cccccattgattaaattatggtaccagttagaaagtgaacctattatgggggcagaaacttattatgtagatgga
gcagctaatagagagacaaaactaggaaaggcaggatatgttacagaactagggaaacagaaaataataaaatta
gatgaaaccaccaatcaaaaggctgaattaatggccatattattagccttacaggattccaaagaaaaagtaaat
atagtaacagattcacaatatgcattgggcatcatttcctctcaacctacacagagtgaatctcctatagttcag
cagataatagaggaactaacaagaaaggaacaggtatatcttgcatgggttccggctcataaaggcataggggga
aatgaaaaaatagataaattagtaagcaaggatattagaagagtcctgttcttagaaggaatagaccaggcacaa
gaagatcatgaaaagtatcatagtaattggagagcattagctagtgaatttggactaccaccagtagtggccaag
gaaatcattgctagctgtcctaaatgtcatataaaggggaagcaatgcatggtcaggtagactgcagtccagaa
gtatggcagatggattgcacacatctagaaggcaaaatcataatagttgctgtccatgtggcaagtggattcata
gaagcagaagtaataccagcagaaacaggacaagaaactgcctacttcctgttaaaactagctgcaagatggcct
gttaaaatattacacacagacaatgggcctaattttacaagtgcaactatgaaggctgcatgttggtgggccaac
atacaacatgagtttggaataccatataatccacaaagccaaggagtagtagaagccatgaataaggaattaaaa
tcaattataggacaggtgagggaccaagcagaacacttaagaacagcagtacaaatggcagtatttgttcacaat
tataaaagaaaaggggggattgggggtacactgcaggagagaggataatagacatattagcaacacaattacaa
acaacagaattacaaaaacaaattttaaaaattcaaaattttcgggtctattacagagacagcagagaccctatt
tggaaaggaccggcacagctcctgtggaaaggtgagggagcagtagtcatacaagayaagggagacattaaggta
gtaccaagaaggaaggcaaaaataatcagagattacaggtctagaaagaccaaggactggtattacagacatcat
tatgaatccagaaatccaagagtcagttcaagtgtatatattccagtagagaragcctatatagtagtgaccaca
tattggggattgatgccaggagaaagagatgwacatmtgggacatggagttagtatagaatggcagtataaaaaa
tataaaacacagattgaccctgaaacagcagacaggatgatacatctgcattatttcacctgttttacagacaca
gcaatcaggaargccatcctagggcagagagtactgaccaagtgtgaataccctgcaggacatagtcaggtaggg
acactacagctactagctctaacagcaatagtaaaaacaagaaacagtaggccccccctacccagtgtccagaaa
ttaacagaagatctcttagaagaattaaaarcagaagcagtaagacattttcctaggccctggctacatgccttg
ggacaatacatttatgagacttatggggacacttgggaaggagtkatggcaattacaagaatcttacaacaactg
ctgtttacccattatagaattggatgccaacatagtagaataggaatttacccaactaatacamgaggaagagga
agaagagtgcctgcttggcaccaccctggaagtcaaccccagaccccttgtaataagtgctattgcaaagaatgc
tgctatcattgctatgtttgtttcacaagcaagggtttgggaatctcccagctgtttgcaacagtctatgctggg
gtgcctgtatgggaagatgcagcaccagtattattctgtgcttcagatgctaatctaacaagcactgagcagcat
aatatttgggcagcacatgcctgtgttcctacagatcctctccatatgaatatccattggacaatgtgacagat

tattttaatgtatggaaaaattatatggtagaacaaatgcaggaagacattattagtttatgggaacagagtctt
aaaccttgtgttcaaatgactttcctgtgtgtacaaatgaattgtaccaacttaaatgacacagtaaatggaacc
agcagctcagaaagcgatatgcaaaggtgtgagtttaatgtaaccactgttgtaaaagacaaaaaggagaaaaaa
caggctttattctatagatcagatttgatggaattaaaggataaggataatagcacaaacacaacaatgtataca
ttaattaattgtaactccacaaccgtcacgcaagcctgtccaaaggtatctttccagccaattccaatacactat
tgtgctccagcaggatttgctatctttaagtgtaacagcacagaattcaatggaacaggcacatgcgaaaacata
acagtagttacttgtacacatggcatcaagccaacagtaagtactcaactaatattaaatgggacactctctaaa
ggaaaaataagaatgatgtcaacaaatatttcagacagtgggaagaatatcatagtgaccctaaattctaccata
aacatgacctgtagtagaccagcattggaagtacagagcatgggcgtaggtccaatggccgtatacagtgcacac
ctaagggaatcagggacaaacggatcaaggatagcttactgtgagtataataccagtgattgggaaaaagcatta
agacaaacagctgagaggtatttagaacttgtaaacaacacaggtagtattagcatgacattcaacaaaagcaac
gatggtggagatccagagacaacccatttacattttaattgtcatggagaattcttttattgtaacacatctaat
ttatttaattatacctttttcatgtaatggaaccacctgtaatgatagccaaagtaccaccaatgacacacagata
ccttgcaaactgagacaggtagtaaggtcatggataaaaggacagtcaggattctatgcacctcccatcaagggt
aacctgacatgtacatcaaacataacaggaatgattctacaaatggatgcaccatggaacagcaccagaaacaac
agcgaaaaccaaactgcaacatttagaccaacagggggagaaatgaaagatatatggagaactgagttgttcaac
tacaaagtagtaagagtaaaacctttagtgtggcacccacatcaattgcaaggccagttgtaggcactagcact
catagagaaaaaagaaacctgtggggctgcaaaggaaagctagtctgctacacatcagtaaaatggaatgaatca
tggacaggaaacgaaagcatttgggaagaactaacatggcaggaatgggatcggcagataagaaacataagctcc
accatatatgaggaaatacaaaaggcacaagtacagcaggaagtaaatgagagaaaggtgctggagttggatgaa
tgggcttctctttggagttggcttgacataactaaatggttgtggtatataaaaatagcaataatcatagcagga
gcactaataggcctgagagttatcatgatagtacttaatctagtgaagaacattaggcagggatatcaacccctc
tcgttgcaggcttgcagaatttgtgcagctgtaacacaatattggctacaagaattgcagaatagtgctacaagt
ttgctagatacagttgcagtggcagtagccaattggactgacggtataatctcagggatacaagcaataggaaga
ggaattcgtaacatcccaactagaattagacagggcttagagagaagtctattgtaaatgggamacgtattggga
aaaaataaatatgagggatgggcagcagtaagacaaagaatgaggagaacwagaatttcccatgagcctgaacca
tgtgcacctggagtaggacagatttccagggacttagcagctagaggrgggataccaatttcccatactcctgaa
aacaatgcagcccttgcattcctagaaagtcaccaagatgaagaagtgggtttcccagtaaggcctcaagtacct
ctaaggccaatgacctataaaggagcatttgacctcagcttctttttaaaagaaaagggaggactggaagggtta
atttactcccataagagagcagaaatcctggatctttgggtgcatcacactcagggattcttccctgattggcag
aactatacaccaggaccaggaactagattcccactgacatttgggtggctatttaagctagtaccagtgacagca
gaagaagcagaaagactaggaaatacatgtgagagggctcatctcctgcacccagcgtgttcccatggctttggt
gatccacatggggaaatactaatgtggaagtttgatagatctctaggcaacacccatgttgctcagataacccac
ccagagctcttccagaaggactaa
>#66 KU168282 7521 nt
atgggtgcgagagcgtctgtgttgacagggagtaaattggatgcgtgggaacaaattaggctaaagccaggatct
aaaaagaaatataggctaaaacatttagtatgggcaagcagggagctggaaagattcgcatgtaatcctggtcta
ctagaatctgcagagggtaatgagcaactgttacagcaattagagccagctctcaagacagggtcagagagcctg
caatctctctggaactcagtagcagtgctctggtgcgttcacaacagatttgatgttcaagatacacagcaggca
ataaaaaaagttaaaggaagtaatggcgagcaggaagcctgccactgccgctaaggaggaaacaggctcaaggcag
acaagtcaaaattaccctgtagtaacaaatgcgcagggacaaatggtacatcaagccctttcccccaggacttta
aatgcatgggtaaaggcagtagaagaaaaagcctttaaccctgaaattattcctatgtttatggcattatcagaa
ggagctatttcctatgatatcaataccatgatgaatgccataggagatcaccaaggggctttacaagtgctgaag
gaagtaatcaatgaggaagcagcagaatgggatagaactcatccaccagcaatggggccgttaccaccagggcag
ataagggaaccaacaggaagtgacattgctggaacaactagcacacagcaagagcaaattcactggattactaga
gggaataaccctatcccagtaggagatatctatagaaaatggatagtgctaggattaaataaagtggtaaaaatg
tacagtccagtgagcatcttagatattaagcagggaccaaaagaaccattcagagactatgtagatcggttctac
aaaacattaagagctgagcaagctactcaagaagtaaaaaattggatgacagaaaccctgcttgttcagaattcc
aacccagattgtaaacaaatttttgaaatcattaggaccaggagctactctagaagaaatgatggtagcctgccaa
ggagtaggagggccatctcacaaggcgagagtactagcagaagcaatggtttctgcccaacaagatctaaaagga
ggatacacagcagtattcatgcaaagagggcaaaatccaaaaagaaaagggcctataaaatgtttcaattgtgga
aaagagggacatatagcaaaaaattgtcgagcacctagaagaaaagggtgctggacagcaagggttgggggccat
ctatgtgaggctttactggatacaggggcagatgatacagtattaaatagcatacaattagaaggaagatggaaa
ccaaaaatgataggggggtataggaggctttataaaagtaaaagaatatgataatgtgacagtagaaatacaagga
aaggaagtasaggggacagtattggtgggacctactcctgttaatattattgggagaaatatattgacaggatta

ggatgtacactaaatttccccataagtcccatagccccagtaccagtacagctaaaaccaggaatggatggacca
aaagtaaaacaatggcccctatctagagaaaaaatagaagcactaacagcaatatgccaagaaatggaacaggaa
ggaaaaatctcaaggataggacctgaaaatccttataatacacctattttgctataaaaaagaaagatagcaca
aaatggagaaagttggtagacttcagggaattaaataaaagaacacaagatttctgggaagtgcaattaggtatt
ccacatccaggggggtttaaagcagaggcaatctgttacagtcttagatgtgggagatgcttatttctcatgtcct
ttagacccagacttcagaaaatacactgccttcactatacctagtttgaacaatgagaccccccggagtaagatac
cagtacaatgtcctcccgcaagggtggaaaggttcaccagccatatttcagagttctatgacaaagattttagat
ccatttagaaaagacaacccagaaatagaaatttatcagtacatggatgacttatatgtaggatcagatctacca
ttggcagaacatagaaagagggttgaattgcttagagaacatttatatcagtggggattcactacccctgataaa
aagcaycagaaagaacctccctttctatggatgggatatgagctccatccagacaagtggacagtacagcccatt
caattgcctaacaaggaagtatggacagtaaatgacatacaaaaagtaataggaaaattaaattgggcgagtcaa
atctatccaggaatcagagtaaaagaattgtgcaagttaattagaggaactaaatcattgacagaggtgataccg
ttaagtaaagaagcagaactagaattagaagaaaacagagaaaggctaaaagagcctatacatggagtatattat
caacctgacaaagacttatgggttaatattcagaagcaaggagaagggcaatggtcttaccagatatatcaggat
gaacataagaaccttaaaacagggaaatatactaggcaaaaggcctctcacacaaatgacataagrcaattagca
gaagtggtccagaaggcatctcaagaatctatagttatatggggaaaattacctaaatttaagttgccagtcact
agagaaacttgggaagcctggtgggcagattattggcaagccacctggattcctgaatgggaatttgtcagcacr
cccccattgatcagattatggtaccggttggaaacagaacctattgtaggggcagaaacctattatgtagatgga
gcagctaataggaatacaaaactaggaaargcaggatatgttacagaacaagggaaacagaaaataataaagtta
gaggagacaaccaatcagaaggctgaattaatggccatactattagccttgcaggattccaaaaaacaagtaaac
atagtaacagactcacaatatgcattgggcatcatagcctcccaaccaacacaaagtgactcccctatagttcag
cagataatagaagaactaaccaataaggaacaagtgtatcttacatgggtaccagctcacaaaggcataggagga
aatgaaaaaatagataagttagtaagcaaagatattagaagagtcctgttcctagaaggaatagatcaggcacaa
gaagatcatgaaaaataccatagcaattggagagcattagctagtgactttggaataccaccagtagtagctaag
gaaatcattgctagttgtcctaaatgccatataaaggagaagcaatgcatggccaagtagactacagcccagag
atatggcaaatggattgcacacatttagaaggcaagatcataatagttgctgtccatgtggcaagtggctttata
gaagcagaagtaataccagcagaaacaggacaggaaactgcctatttcctgttaaaattagcagcaagatggcct
gtcaaagtaatacatacagacaatgggcctaattttacaagtgcagccatgaaggctgcatgttggtggacaggc
atacaacatgagtttggaataccatataatccacaaagtcagggagtagtagaagccatgaataaggaattaaag
tctattatacagcaggtgagagaccaagcagagcatctaaaaacagcagtacaaatggcagtctttgttcacaat
tttaaaagaaaaggggggattggggggtacactgcaggagagagattaatagatatactagcatcacaaatacaa
acaacagaattacaaaaacaaattttcaaaattcaaaattttcaggtctattacagagatagcagagaccctatt
tggaaaggaccggcacaactactgtggaaaggtgaggggcagtagttatacaagataaaggagacattaaagta
gtaccaagaagaaaggcaaaaataatcagaaattacagatctagaaaggctaagagatggaattatagacatcat
tatgaaacaaggcatccaaaaattagttcagttatatacattccaatagcagaagctgagataatggttaccaca
tattggggattaatgccaggggaaagagaggaacacttgggacatggggtcagtatagaatggcaatacaagaag
tataaaacacagattgatcctgaaacagcagacaggatgatacatctgcattattttacctgttttacagagtca
gcaatcaggaaggccattctagggcagagagtgctgaccaggtgtgaatacactgcaggacatagtaaggtaggg
acactacagttcttagccttaaaagcattagtgaaagtaaagaaaaacaagcctcccctacccagtgtccaaaag
ttaacagaagacagatggaacaagcaccagagaatcaagggccagctagagagcaatccaatgtgtggacatctc
ctggaggagctaaaacaagaagcagtgagacatttccctaggacttggctacaagccttagggcaatacatttat
gatacttatggagacacttgggagggagttatagcaattataagaatcttacaacaaatactgtttatccattat
agaattggatgccagcatagtagagtaggaattattccatctaatacaagaggaagaggaagaagaatgcctcct
tggcatcaccctgggagcaggccccaaaccccttgtaataattgctattgcaaacgctgctgctatcactgcctc
ctttgtttcacaaagaagggtttgggaatctccccacaaatgtatgcaacagtctattctggggtaccggtatgg
gaagatgcaaaaccaacactattctgtgcttcagatgttaacttgacaagcacagagaaacataatatttgggca
tcacaagcctgtgttcccacagaccctactccaaatgaatatcgtctagagaatgtgacagataaatttgatata
tggaaaaattatatggtggaacaaatgcatgaagacattattagtttatgggatcagagtttaaagccttgtgtg
cagttgactttcttatgtgtacaaatgaactgtacagatgtaaaatcaaatacaacaaatacaaccatcctaaaa
gaagaagcaacagccataaaaagctgtagctttgatgtaactacagttctcaaagacaaaaaggagaaaaaacag
gctctattttatgtaacagatctggctaaggttgacccaacagatccaaattcaacagatacaacaacctataca
ttaattaattgtaactccacaaccatcaggcaagcctgtccgaaggtaagctttgagcccatccctatacattat
tgtgctccagcaggatatgccatctttaagtgtaatgaaacaaactttaatggaacaggcacatgccacaatgtt
acaatggttacttgtacacatggcatcaagccaacagtaagtactcagctaatattgaatggacaatatctaaa
gacaaaacaagaattatgggacaaaatatcacagacagtgggaagaatatcataataaccctaaatactactata

aaaatgaattgcacgaaagaaggaaataatgagatacaagaaataaaaatagggcccatgtcatggctcagtatg
gcagttactgcaaatgaaactggctcaaaatcaaggatagcttattgtaattatagtagcaccgaatgggaaaag
gccttaaaactaacagctgaaagatatgtacaacttttaaacataacagaaaaagttaccataacattcaaaaat
agcagtggggagatgcagaaataagccgtttgcattttaactgtcatggagaattcttttattgtaacacatct
aagatgtttaactatacttttgactgtacaagtggcaactgtactgagatcaattccatgaataaaactgatgag
cataatggactaccttgcaggttaagacaggtagtaagatcatggataaaggagagtcaggactttatgcacct
cccatcagaggcaacctcacatgccagtccaacataactggaatgattctacaactagatgaaccatataaccgc
acatcagatgcaaatgtcacacttagaccaagtggggagacatgcaagatatatggagagctcaattgtacaac
tacaaagtagtacagataaaacctttagtgtagcacctacaagaatctcaaggccaacaataaaccttaacacc
cctcgcagagaaaaaagaaacctatggggctgtaagggaaggataatctgttacacatcagtaaaatggaacaca
tcatggggagattacaatgacagtatttggaaccactatacatggcagcaatgggaccaacaaatagataatata
agctccattatctatgatgaaatacaagcagcacaagaccaacaggaaaggaatgtaaaggcactgctggagcta
gatgaatgggcctctctttggaactggtttgacataactaaatggctgtggtatataaaaatagctataatcata
gtaggagcactgataggtataagagttatcatggtagtacttaatctagtgaggaacattaggcagggatatcaa
cccctctcgttgcaggctttcaaactttgtggagctgtaacacaatactggctacaagaattgcagactagtgct
ataaatctgtttgatacttttgcagtggcagttgctaattggactgacagctttatcttaggtatacaaagaata
ggacgaggattccttaacatcccaagaagggttagacaaggcgcagaaatagcattattgtaaatggggaatgcc
tggagcaaaagcaaatttgcaggatggtcagaagtaagaaggagaatgagacaaacctcccctgagcctcagcca
tgtgcacctggagtaggagaagtctccagggcattagcagacagaggggaataccaaattcctacaatcctcaa
aacaatgcagctctcgcattcctagaaagccacacagatgaggaagtaggtttcccagtaaaacctcaagtgcct
ctaagaccaatgacctacaaagcagccgttgacatcagcttcttttaaaagaaaagggaggactggaagggtta
atttactcccataagagagcagaaatcctggatctctggatatatcacactcagggattttccctgattggcag
tgttacacaccgggaccaggaactagattcccactgacatttggatggctatttaagttagtgccagtgtcaaga
gaggaggcagaaggactgggtaatacacgtgaggatgctagtctgctacatccagcttgtaatcatggcgctgag
gatgaacacggggagatgctaaaatggcagtttgatagaacattaggcagcacacatatagccctgcaaaagcac
ccagagctcttccccaagtaa
>#67 AY169809 7509 nt
atgggtgcgagagcgtctgtgttgaaagggagcaaattggatgcatgggaacaaattaagttgaagccaggatct
aaaaaagcatataggctaaaacatttagtatgggcaagcagagagctggaaagattcgcatgtaatcctgagcta
ttagaaactgcagagggtaatgagaaactgttacaacagttagagccagctctcaagacagggtcagaaagcctg
cagtcactctggaacacagtagcagtgctctggtgtgttcacaacagatttaaagttgaagatacgcagcaggca
atacaaaagttaaaggaagtaatggggaaaaggaagcctgtagatactgctaaggaagacacaaactcaaggcar
acaggtcaaaattaccctgtagtaccaaatgcacagggacaaatgatacatcaggccctctcccccaggactttg
aatgcatgggtaaaagcagtagaagaaaaagcctttaaccctgaaattattccaatgttcatggcattgtcagag
ggagctgttccctatgatatcaatactatgctaaatgccataggagggcatcaggggggctttacaagtgctgaag
gaagtaatcaatgaggaagcatcagactgggatagaacgcacccaccagcgatggggccgttgccaccagggcaa
ataagggaaccaacaggaagtgacattgctggaacaactagcacccagcaagagcaagttcactggactaccagg
cctaacaaccctatcccagttggagatatttatagaaaatggatagtgttaggattaaacaaaatggtaaaaatg
tacagtccagtgagcatcctagatattaggcagggaccaaaagagccatttagagactatgtagatcggttctac
aaaacattaagagctgagcaagcaactcaagaagtaaaaaattggatgacagaaaccctgcttgttcaaaatgcc
aacccagattgcaaacagattttaaaatcattagggccaggagctaccttagaagaaatgatgatagcctgtcaa
ggagtaggagggccaacacataaggccaaaatactagcagaagcaatggctacagcccagcaagatctaaaagga
ggatacacagcagtgttcatgcagagagggcaaggcccaattaggaaagggcctataaaatgtttcaactgtgga
aaagagggacatatagcaaaaaattgtcgagcacctagaaagaaaggctgctggaaatgtggacaggaaggtcat
caaatgaaagattgcagaaatggaaaacaggcaacagcaagggttgggggccatctatgtgaagttttactggat
acaggggcagatgatacagtactaaacaacatacaattggaaggaaaatggacaccaaaaatgatagggggtata
ggaggctttataaaggtaaaagaatatagcaatgtgaaagtagaaatagaaggaagggaagtacagggaacagta
ttagtgggacctacccctgttaatattattggaagaaatatattgacaggattaggttgtacactaaatttccct
ataagccccatagccccagtaccagtgaaactaaaaccaggaatggatggaccaaaagtaaaacaatggccccta
tctaaagaaaaaatagaagctttaacagcaatatgtcaggaaatggaacaagaaggaaaaatttcaagaatagga
cccgaaaatccttataatacacctatctttgctataaagaagaaagatagtaccaagtggagaaaattggtagac
ttyagagaattaaacaagagaacacaagatttctgggaggtacagttaggtattccacatccggggggtttaaag
caaaagcaatctgttacagtcctagatgtaggagatgcttatttctcatgtcctttagacccagatttcagaaaa
tatactgccttcactattcctagtgtgaacaatgagaccccaggagtaagataccagtacaacgtcctcccgcaa
ggatggaaaggatcgccagccatattccaragttccatgacaaaaattctagatccatttaggaaaaagaaccca

gaaatagaaatttatcagtacatggatgacttatatgtaggatcagatttgcccttggcagaacatagaaaaagg
gttgaattgcttagagagcatttatatcagtggggattcacgacccctgataaaaaacatcaaaaggaacctccc
tttctgtggatgggatatgagctccacccagacaagtggacagtacagcccatccacttgcctaataaggacgtg
tggacagtaaatgatatacaaaaactagtaggaaaattaaattgggcaagtcaaatctatcaagggattagagta
agagaattgtgtaagttaatcaagggcaccaagtcattgacagaggtggtacctttaagtaaagaggcagaacta
gaattagaagaaaayagggaaaagctaaaagaaccagtacatggrgtatactatcaacctgacaaagatttatgg
gttaatattcagaagcaaggagagggacagtggacttaccagatatatcaggatgaacataagaacctcaagaca
gggaaatatactaggcaaagagcctcccacacaaatgatataaggcaattggcagaagtactccagaaggtatct
caggaatcgatagttatctgggggaaattgcctaaatttaagctgccggtcactagagaaacttgggaaacatgg
tgggcagactattggcaagccacctggattccagaatgggaatttgttagcacacccccattgatcaagttatgg
taccagttagaaagkgaacctattagggggcagaaacatattatgtagatggagcagctaatagagatacaaaa
ctaggaaaggcaggatatgttacagagcaagggaaacagaagataataaaattagaagagaccaccaatcaaaag
gctgaattaatggcagtgttagtagccttacaggattccaaggagaaagtaaacatagtaacagattcacaatat
gtattgggtatcatctcctcccaacctacacagagtgaatcccctatagttcaacagataatagaagaactgaca
aaaaaggaacaggtgtaccttacatgggtccctgctcataagggcataggggggaaatgaaaaaaatagataaatta
gtaagcaaagatattagaagagtcctattcctagaaggaatagaccaggcacaagaagatcatgagaaataccat
agcaattggagagcattagccagtgactttggactaccaccagtggtggccaaggaaattattgctaattgtcct
aaatgtcatataaagggggaagcaattcatggtcaggtagactgcagtccagaagtatggcaaatggattgcaca
catgtagaaggcaaagttatcatagttgctgtccatgtggcaagcggattcatagaagcagaagtgataccagca
gaaacaggacaggaaactgcctatttcctgttaaaattagcagcaagatggcctgttaaagtaatacatacagac
aacgggcctaattttacaagtgcaaccatgaaagctgcatgttggtggactaacatacaacatgagtttggaata
ccatacaatccacaaagtcaaggagtagtagaagccatgaataaggaattaaaatcaattatacagcaagtgagg
gaccaagcagagcacttaaggacagcagtacaaatggcagtatttgttcacaattttaaaagaaaaggggggatt
ggggggtacactgcaggagagagattaatagacatattagcatcacaaatacaaacaacagaactacaaaaacaa
attttgaaaattcaaaaatttcgggtctattacagagacagcagagaccctatctggaaaggaccggcacagctc
ctgtggaaaggtgagggagcagtagtcatacaagacaaaggagacattaaggtagtaccaagaagaaaggcaaaa
atactcagggactacaggtctaggaaaaccaaggactggttttacagacatcattatgagtctagaaatccaaga
gttagttcaggtgtatatattccagtagggrtaggttgtataatagtgaccacatattggggattaatgccaggg
gaaagggatgagcagttgggacatggagttagtatagaatggcagtacaagaggtataaaacacagattgaccct
gaaacagcagacaggatgatacatctgtattatttcacctgttttacagagtcagcaatcaggaaagccaccctа
gggcagagagtgctgaccaggtgtgaataccctgcaggacatagtcaggtagggacrctgcaactattagckctg
agaatagtagtaaaagaaaaaaggaataagccccccctacccagtgtccagaagttaacagaagatctcctggaa
gaactgaaagcagaagcagtgagacatttccctaggaattggttacaagacctgggacaattcatttatgagact
tatggagacacttgggtaggagttatggccattataagaatcttacaacagatactatttacccattttagaatt
ggatgccracacagtagaataggaattaacccatctaacacaagaggaagaggaagaagagtgccccccttggcac
caccctggaagtcagccccagaccccttgtaatgcttgctattgcaaaaagtgctgctatcattgctatctttgt
ttcacaagcaagggtttgggaatctcccagttgtatgcaacagtctatgctggggtgcctgtatgggaagaagca
aacccaatactattctgtgcttccgatgctaacttaacaagcactgagaagcacaatatttgggcagcacaagcc
tgcgttcccacagaccccactccacatgaatatccattatccaatgtgacagataacttcaatatatgggaaaat
tacatggtggaccagatgcatgatgatatcactgatttgtggaagcagagtttaaagccatgtgttgaattgact
ttcctgtgtgtacaaatgaattgcacagatgttactcatactaataatgtaaccatctcgcaggctgtaaccagc
cccgtaaaaaagtgtgatttcaatgtaaccacaattttcaaagacaaaagggagaaaaaacaggctctattctat
aaagcagatttgcttgaaacaaaagaattagaatcaaacagaacaatgtatacattaattaactgtaactccaca
accatcaagcaagcctgtccaaaagtaacttttgaacctattccaatacactactgtgctccagcaggctatgcc
atctttaagtgtaacaatagcgaatttaacggaaaaggtgcatgcagtaatattacagtagttacttgtacacat
ggcatcaagccagcagtaagtactcaactaatactaaatgggacactctctaaaggaaaaataagaattatggga
aaaaatatttcagacagtgggcaaagtatcctagtgactctaaattctactataaacataacctgtgagagacca
ggaaatcggaaaagagaggagataaaaataggtccaatggcttggtacagtatgtcagtagaaaacaacataaca
tcaagggaagcttattgcacatataatgtcagtaaatgggcaacagccctaaagcaaacagctgagaggtatcta
gaacttgtaaacaatacacaggctagcataacattcaatcaaagcagtggtggagatccagagataaccaattta
catttcaattgtcatggagaattcttttattgtaacacaacggggttgtttaattatacctttctgtgtgaaaac
tccatctgtaacttcacccataacaatacttcagaggagcgcaatctaacttgtagaattagacaggtggtaagg
tcatggatgaagacagggtcgggactctatgcacctcccatcgaaggtaacctaacatgtaggtcaaacataact
ggaatgattctagaaatggataaaccatggaacaaaagtacaaacaatgaaacagccatatttagaccaataggg
ggagatatgaaagaaatatggagaactgaattgttcaactacaaagtagtaagggtaaaacctttagtgtggca

cctacaaaaattgcaaggccagtcataggcactggcactcctagacagaagagaaacctgtggggctgtaaggga
caattagtctgctacacatcagtaaaatggaacacaacatggggaagcaatattcctaatattactaatctrgag
gaaatttggggcaaactaacctggcaggaatgggatcagcaggtaagcaatgtaagctccatcatatatgaggaa
atacaaaaggcacaagtacagcaggaacaaaatgagaagaagttgctggagttaaatgaatgggcctctatttgg
aattggcttgacataactaaatggttgtggtatataaaaatagctataatcatagtaggagcactagtaggtgtg
agagttgtyatgataatacttaatctagtgagaaacattaggcagggatatcaacccctatcgttacaggcttgt
agaattggtagagctgtaatacaatactggctacaagaattgcagaatagtgctataagtctggtagataccttg
gcagtggtagttgccaattggactgacggcatcatttaggactacaacgcataggaagaggaattcttaacatc
ccaacaagaattaggcagggcctagaattaggtttattgtaaatgggaaacgcattraagaaaagtagctttgtg
ggatggccagcagtaagagaaagaatgagaaaagctcccagtagtgatcctgagccatgtgcacctggagtagga
gaactctccagagaattagcagctagaggagggataccaggctcatatactcctcaaataatgcagcccttgca
ttcctagaaagccaccgagaagaagaagaagtaggtttcccagttaaacctcaggtgcctttaaaaccaatgacc
tataaaggagcatttgacctcagcttctttttaaaagaaaagggaggactggatgggttaatttactcccatact
agagcagagatcctggacctttgggtgcataacactcagggattcctccctgattggcagaactacacaccagga
ccaggaaccagattcccactgacatttggatggttatttaagctagtaccagtgtcagaagctgaggcagaagaa
ttaggcaataagtgtgaaagggctagactcctccatccagcttgtgatcatggttttggagatcaccacggggaa
atactgaaatggcagtttgatagatcactaggcaacactcatgttgctatgataacccacccagagctcttccac
aaggactaa
>#68 KU168283 7389 nt
atgggtgcgagtgcgtctgtgttgaaagggagcaaattggatgcatgggaacaaattaggttaaagccaggatct
aaaaagaaatatagactaaaacatttagtatgggcaagcagggagctggaaagattcgcatgtaatcctgagcta
ctagaaactgcagagggcaatgagaaactattacaacagttagagccagctctcaagacagggtcagacagcctg
cagtcactctggaacacaatagcagtgctctggtgtgttcataacagatataaagtagaagatacacagcaggca
acacaaaagctgaaggaagtaatggggagcagaaagtctgcagcggccgctaaggaagacacaagcggaggcag
acgggtcaaaattaccctgtagtaccaaatgcacaaggacagatggtgcatcagcccatctcccccaggacttta
aatgcatgggtaaaggcagtagaggaaaaggcctttaaccctgaagtcatccctatgttcatggcattatcagag
ggagctattccctatgatattaatactatgctaaatgccataggaggacatcaaggggccttacaagtgctaaaa
gaagtaatcaatgaggaagcatcagattgggatagaactcatccaccagcgatgggaccgttaccaccagggcag
ataagggaaccaacaggaagtgacattgctggaacaactagcacccagcaagagcaagttcactgggttaccagg
gccaacaaccctatcccagtaggagatatctatagaaaatggatagtgctaggattaaacaaaatggtaaaaatg
tacagcccagtgagcatcttagatattaaacagggaccaaaagaaccatttagagactatgtagatcggttctat
aaaacattaagagctgagcaagcaagtcaagacgtaaaaaattggatgacagaaacattgcttgttcaaaatgcc
aacccagattgcaaacagattttgaaatcattagggcaaggagctaccttagaagagatgatgryagcctgtcag
ggagtgggaggaccaactcataaggccagagtgctagcagaagcaatggctacggcccagcaagatctgaaggga
ggatgcacagcaatatttatgcaaagagggcaaaatccaattaggaaaggacctataaaatgtttcaattgtgga
aaagagggacatctagcaagaaattgtcgagcgcctagaaagaaaggttgctggacagcaagggttgggggccat
ctatgtgaagctttactggacacaggggcagatgatacagtactaaccaacatacaattagagggaaaatggaca
ccaaaaatgataggggggtataggaggttttgtaaaagtaaaagaatatgatgacgtgacagtagagaaagaggga
agaagagtacagggaacagtattggtgggacctactcctgttaatattattggaagaaatatattgacaggatta
ggttgtacactaaacttccctataagccccatagccccagtgccagtaaaactaaaaccaggaatggatggacca
aaagtaaaacaatggcccctatctaaagaaaagatagaagccttgacagcaatatgtcaagaaatggagcaagaa
ggaaaaatttcaagagtaggacctgaaaatccttataatacacctatctttgctataaaaaagaaggatagtact
aggtggagaaagctagtagactttagagaattaaacaagagaacacaagatttctgggaggtacaattaggtatc
ccacacccggggggtttacagcaaaggcaatctgttacagtcctagatgtaggagatgcttatttctcatgcccc
ttagacccagatttcagaaaatatactgctttcactattcctagtgtgaacaatgagaccccagggataagatat
cagtacaatgtcctcccgcagggatggaaagggtcaccagctatattccaaagttcgatgacaaaaattctagat
ccatttaggaaaaacaacccagagctagaaatttgtcagtacgtggatgacctatatgtagcatcagatttaccc
ctgacagaacatagaaaacgggtagaattgcttagagaacacttatatcagtggggggttcactaccccctgataaa
aagcatcaaaaggaacctccctttctgtggatggggtatgagctccatccagacaaatggacagtacagcccatc
caattgcctaacaaggacgtatggacagtaaatgatatacaaaaactagtaggaaagttaaattgggcaagtcaa
atctatccaggaattagagtaaaagaattgtgtaagttaattagaggcaccaagtcattgacagaaatagtaccc
ttaagtaaagaagcagagctagaattagaggagaacagagaaaggctaaaagaaccagtgcatggtgtatactat
cagcctgacaaagacttatgggttaatattcagaagcaaggagaagggcaatggacttatcagatatatcaggag
gaacataagaacctaaaaacaggaaatactctaggcaaaaggcctcccacacaaatgatataagactattagca
gaagtagtccagaaggtgtctcaagaagctatagttatttggggaaaattgcctaaatttaagctgccagtcgct

agggagacttgggaaacatggtgggcggactattggcaagccacctggattccagaatgggaatttgtcagcaca
cccccattaatcaaattatggtacaggttagaaagtgaccctattagggggcagaaacatattatgtagatgga
gcagctaatagagatacaaaattaggaaaggcaggatatgttacagaamgagggaaacagaaaataataaaatta
gargagaccaccaatcaaaaggctgaattaatggcagtattattagccttacaggattccaaggaagcagtaaac
atagtaacagattcacaatatgcattgggcatcatctcctcccaacctacacagagtgattcccctatagttcag
cagataatagaggaactaacaagaaaggaaagggtgtatcttgcatgggttcctgctcataaaggcataggagga
aatgaaaaaatagataaattagtaagcaaggatattagaagagtcctatttctagaaggaatagaccaagcacaa
gaggatcatgaaaagtatcatagcaattggagagcattagctagtgactttggactaccaccagtggtagccaaa
gaaatcattgctaattgtcctaaatgtcagacaaaaggagaagcaatgcatggacaagtagactgcagtccagaa
gtatggcaaatagattgcacacatacagaaggcaaagtcataatagttgctgtccatgtggcaagtggatatata
gaagcagaagtaataccagcagaaacaggacaagaaactgcctacttcctgttaaaattagctgcaagatggcct
gttaaagtaatacatacagacaacggcctaattttacaagtgccaccatgaaggctgcatgttggtggaccaac
ataaaacatgagtttggaataccatataatccacaaagtcaaggagtagtagaagccatgaataaagaattaaaa
tcaattatacagcaggtaagggaccaagcagaacacttaagaacagcagtacagatggcagtatttgttcacaat
tttaaaagaaaaggggggattggggggtacactgcaggagaaaggataatagacatattagcatcacaaatacaa
acaacagaattacaaaaacaaatttttaaaattcaaaaatttcaggtctattacagagacagcagagatcctatt
tggaaaggaccggcacagctcctgtggaaaggtgaaggagcagtagtcatacaagataaaggagacattaaggta
gtaccaagaaggaaggcaaaaataattagacattacagatctagaaagaccaaggaatggtattacagacaccat
tttgaatccagaatccaagattcagttcaggtgtacatatcccagtagggaaggcttgggtagtagtgaccacc
tattggggattgatgccaggggaaaaagatgaacatttgggacatggggttagtatagaatggcagtacaagaga
tatacaacacagattgaccctgaaacagcagacaggataatacatcagtattattttacctgttttacagagtca
gcaatcaggaaagccgtcctagggcacagagtgctgaccaaatgtgaatactctgcaggacatagtcaggtaggg
acattgcaactactagctctaagagcagtagtaaaagaaaaaagaaataagcctcccctacccagtgtccagaaa
ttaacagaagataccctagaagatctaaaagcagaagcagtaagacattttcctagaccatggctacatgccttg
ggacaatacatatatgaaacttatggagacacttgggtaggagttatggcaattatgagaatcttacaacaactg
ctatttacccatttagaattggatgccaacatagtagaataggaattaacccatctaacacaagagaaagagga
agaagaatgccccccttggcatcaccctggaagtcaaccccagaccccttgtaataagtgctattgcaaaagatgc
tgctatcattgctatgtttgtttcacaagaaagggtttgggaatctcccagctgtatgcaacagtctatgctggg
gtacctgtatgggaagaggcaaccccagtattattctgtgcttcagatgcgaacctaacaagcactgagcagcat
aacatttgggcatcacaagcctgtgtccccacagatcccactccatatgaatatccattaaagaatgtgacagat
aacttcaatatatggaaaaattacatggtagaacaaatgcaggaagacattattagtttatgggaacagagctta
aaaccttgtgttcaaatgactttcctgtgtgtacaaatgaattgtacagacataatgaaacagtgtgcctttaat
ataaccacagttctcaaagacaaaaaggagaaaaaacaggctctgttctatgtatcagatttacataaattctat
gataacgagacaaatagcacaatgtatagattaactaactgtaattcctcaaccattaggcaagcctgtccaaag
gtatcttttcagccaattccaatacactattgtgctccagcaggatatgccatctttaagtgtaacagctcagaa
tttaatggaacaggtacatgccgcaacattacagtagttacttgtacacatggcatcaagccaacagtgagtact
caactaatattaaatgggacactctctgaaggaaaaatcagaattatggggaaaaatatctcggacagtggcaaa
agcatcttagtgaccctaaattctactataaacataacctgtgagagaccatggaatctgtcattacaagagata
cgcataggtccaatggcctggtatagcattgctttacagacacaaccccaggcaaggttagctcattgcacgtat
aataccactgattgggaaaaagccttaaaacaaacagctgaaaggtacttagaacttgtaaactatacaagtggt
aatgttaacataacattcaatagaagcaatgakggaggagatgcagaaataacccagctacattttaactgtcac
ggagagttcttctattgtaacacatctagtctgtttaattatagcttttcatgtaacggaaccaactgtactatt
aaccaaactaaaaatgagacttggataccttgcaggataaaacaggtagtaaggtcatggatacagggagggtca
ggactctacgcacctcccatccgaggtgcactaaaatgtgagtcaaacataactggaataattctagaaatggat
cagccatggaataaaagcagcccagatgtcacatttagaccaataggggagacatgaaagacatatggagaact
gaattgttcagatacaaagtagtaaaggtaaaaccttttagtgtggcacctacaaaaattgcaaggccagtcata
ggcactaacactcatagagaraaaagaaacctatggggttgtaagggaaggataatctgctacacatcagtaaaa
tggaacagtacatggggaaatgtcaccagtatgagtgaagtttgggacaaactaacctggcaggaatgggatcag
cagatagacaacataagcaatgttatatttgatgaaatacaaagagcacaagtacagcaggaacaaaatgagaag
aagttgctggagttagatgaatgggcttcaatttggaattggcttgacataactaaatggttgtggtatataaaa
atagcaataatcatagtaggagcactaataggtgtaagaattgtcatggtagtacttaatctagtgagaaacatt
aggcagggatatcaacccctctcgtttcaggcttgcagaatttgtgcagctgtaggacaatactggctacaagaa
ttgcagaatagtgctacaaacctattagataccattgcagtggcagttgccaattggactgacggcataatatta
ggaatacaaaggataggaagaggaattcgtaacatcccaaggagaactagacagggcttagaacaaagtttattg
taaatgggaaacgcattgaggaaaagcccatttccaggatgggcagcgataagagagagaatgagaagaacctcc

cctgagcctgaaccatgtgcacctggagtaggacaaatctccaaggaattagcagctagaggagggataccaagt
tcctatactcctcaaaacaatgcagcccttgcattcctagaaagtcaccaagaggaagaagtaggtttcccagta
agacctcaagtgcctctaaggccaatgacctataaagcagcatttgacctcagcttctttttaaaagaaaaggga
ggactggaagggctaatttactcccatcagagagcagagatcctggatctttggatttatcatacccaaggattc
ttccctgattggcagaattacacaccaggaccaggaactagattcccactgacatttgggtggttatttaagcta
gtaccagtgtcagaagctgaggcagaagaactaggaaataaatgtgagagggctaaactcctgcatccagcttgc
aaccatggctcggaagatgcacacggacagatactgaaatggcagtttgatagatcactaggcaacacccatgtt
gctatgataacccatccagagctcttcaacaaggactaa
>#69 AY169813 7488 nt
atgggtgcgagagcgtctgtgttgacagggagtaaattagatgattgggaacaaattaggttaaggccaggatct
aaaaagaaatataggctaaaacatttagtatgggcaagcagggagctggaaagattcgcatgtaatcctgagctg
ttagaaactgcagaaggtaatgagaaactgttacaacagttagaaccagctctcaagacagggtcagaagacctg
aagtcactctggaacgcaatagcagtactctggtgcgttcataacagatataaagttggagatacacagcaagca
gtgcaaaaattaaaagaagtaatgggaaacaggaagtctgcagataccgctaaagaaaccactagctcaaagcag
acaagtcaaaattacccagtagtagcaaacatgcaagggcaaatgatacatcagcccctctcccccaggacttta
aatgcatgggtaaaggcagtagaagaaaaggcctttaacccggaaattatccctatgtttatggcattgtcagaa
ggagctattccctatgacattaatactatgctaaatgccataggaggacaccaggggctttacaagtgttgaag
gaagtcatcaatgaggaagcagcagattgggatagaactcacccaccggtggtagggccgttaccaccagggcaa
ataagggaaccaacaggaagtgacattgctggaacaactagcacccagcaagagcaagtccactggattaccagg
gctaacgcccctatcccagttggagatatctatagaaaatggatagtgctaggactaaataaaatggtaaaaatg
tacagtccagtgagcatcttagatattaaacagggaccaaaagaaccatttagagattatgtggataggttctac
aaaacattaagagctgaacaagcaacccaggaagtaaaaaattggatgacagaaaccctacttgttcagaatgcc
aatccagattgcaaacagattttgaaatccttagggccaggagctaccttagaagaaatgatggtagcatgtcag
ggagtaggagggccaactcataaggcaaaaatactagcagaagcaatggcctcagtccaacaagatctaaaagga
ggctacacagcagtatttatgcaaaggggacaaaatccaactaggggaagacctataaaatgtttcaattgtgga
aaagatggacatctagcaaaaaattgtcgagcacctagaaaaaaaggttgctggaaatgtggacaggaaggtcac
caaatgaaagattgcagaaatggaaaacaggcaacagcaaaggttgggggccatctatgtgaggttttgctggat
acaggggcagatgatacagtactaaacaacatacaattggaaggaagatggacaccaaaaatgatagggggaata
ggaggttttataaaagtaaaagaatatgataatgtgacagtagaaatagaaggaaaaaaggtgcagggaacagta
ttggtgggacctactcctgttaatattattggaagaaatatattgacaggattgggttgcacattaaacttccct
ataagccccatagacacagtaccagtaaaactaaaaccaggaatggatggaccaaaagtaaaacaatggccccta
tctagagaaaaaatagaagccttaacagcaatatgtcaggaaatggaacaagaaggaaaaatttcaagaatagga
cctgaaaatccttataatacacccatctttgctataaaaaagaaagatagtactaagtggagaaaattggtagac
ttcagagaattaaataaaagaacacaagacttctgggaggtacagttaggtatcccacatccgggggtttaaag
caaaaacaatctgttacagttttagatgtaggagatgcttatttctcatgtcctttagatgagaatttcagaaaa
tatactgctttcactattcctagtataaacaatgagaccccaggaataagataccagtacaatgtcctcccacaa
ggatggaaagggtcaccagctatattccaaagttcaatgacaaaaattctagatccattcagaaaaaacaatcca
gaaatagaaatttgtcagtacatggatgacttatatgtagggtcagatttgcccttggcagagcatagaaaaagg
gtagaatcacttagagagcatttatatcggtggggattcactaccccagataaaaagcatcaaaaagaacctcca
tttatgtggatgggatatgagctccacccagacaaatggacagtacagcccatccaattgcctgacaaggaagtg
tggacagtaaatgatatccaaaaattagtaggaaagttaaattgggcaagtcagatatatcagggaattagagta
aaagaattatgcaagttaatcagaggaaccaagtcattgacagaagtggtacctttgagtaaagaggcagaacta
gaactggaagaaaacagggaaaggctaaaagaaccagtacatggtgtatattatcaacctaacaaagacttatgg
gttaatattcagaaacaaggacaagggcaatggacttatcaggtgtatcaggatgaacataagaacctcaaaaca
ggaaaatatgctaggcaaaaggcctcccatacaaatgagataagacaaatagcagaagtagtccagaaggtggct
caagagtctatagtcatctggggaaaattgcctaaatttaagctgccagtcactagagaaacttgggaagcatgg
tgggcagactattggcaagccacctggattcctgaatgggaatttgtcagcacgcccccattaatcaaattatgg
taccaattggaaagtgaacctattgtaggagcagaaacctattatgtagatggagcagctaatagggagacaaaa
ttaggaaaggcaggatatgttacagaacaaggaaaacagaaaataataaatttagaggagaccaccaatcaaaag
gctgaattaatggcagtactaatagcattacaggattccaaagaaaaggtaaacatagtaacagactcacaatat
gtattgggcatcatctcctcccaacctacacaaagtgattcccctatagttcagcagataatagaagaactgaca
aaaaaggaacaaacgtatattacatgggttcctgctcataagggcataggaggaaatgaaaaaatagataaatta
gtaagcaaagatattagaagggtcttattcctggaaggaatagatcaggcacaagaagatcatgaaaaatatcac
agcaattggagagcattagctagtgactttggactaccaccagtagtagccaaagaaattattgctagttgtcct
aaatgtcatactaaaggggaagcaattcatggtcaggtagattacagtccagagatatggcaaatggattgcaca

catttagaaggcaaagtcataatagttgctgtccatgtggcaagtggattcatagaggcagaagtgataccagca
gaaacaggacaagaaactgcctatttcctgttaaaattagcakcaagatggcctattaaaatattacatacagat
aatgggcctaattttacaagtgcaaccatgaaggctgcatgttggtgggcyaacataaaacatgagtttggcata
ccatataacccacaaagtcaaggggtagtagaagccatgaacaaggaattaaagtcaattatacagcaggtgagg
gaccaagcagagcatttaaaaacagcagtacaaatggcagtatttgttcacaattttaaaagaaaaggggggatt
ggggggtgtactgcaggagagagattaatagatatgttagcatcacaaatacaaacaacagaactacaaaaacaa
attttaaaaattcaaaaatttcgggtctattacagagacagcagagaccctatttggaaaggaccggcacaactc
ctgtggaaaggtgagggagcagtagtcatacaagataaaggagatatcaaggtagtaccaagaagaaaagcaaaa
atactcaggtgtctggtgaaataccataagtacaggtctaaaaaggcccagagatggcattatagacatcattat
gaaaccagaaatccaagaattagctcaggtgtatatattccagtaggtacagctaatatagtggtgactacatat
tggggattaatgccaggggaaagagatgagcatttaggacatggagtcagtatagaatggcaatacaaaaaatat
agtacacagattgaccctgaaacagcagatagamtratacatctgcattatttcacctgttttacagaracagca
atcaggagtgccattttagggcagagagtgctgatcaggtgtgaataccctgcaggacatagccaggtagggaca
ctgcaactcctagcattaagagcagtggtaaaagacaaaagaagtaaacctcccctacccagtgtccagaagtta
acaggagacaccctggaagaactaaaagcagaagcagtaagacactttcctaggccttggctacaaaacttagga
caatacatctatgatacttatggagacacctgggaaggagttatggcaatcataagaatcttacaacagttgata
tttatccatttagaattggatgtcaacatagtagaataggaattaccccatctaacgcaagaggaagaggaaga
agagtgccccccctggcatcaccctggaagtcagccccccaaccccttgcaacgcttgctattgcaaaaaatgctgt
tatcattgctatctttgtttcacaaagaagggtttgggaatctccaatctatatgcaacagtctattctggggtg
cctgtgtgggaagatgcaasccccaacattattctgtgcttcagatgttaaccttactagcactgagagccataat
atttgggcatcacaggcctgcgtccccacagacccctcaccacatgagtatccactaattaatgtgacagataga
ttcaatatatggaagaattacatggtagatcaaatgcaggaagacattattagtttatgggagcagagtctaaaa
ccttgtgttcaaatgactttcctgtgtgtacaaatgaattgtacagatgtcaatcaaacattgagtaatgcgaca
caagggatggaagtaaaaaagtgtgaatttaatgtaactacagttttaaaagacaaaaaggaaaagaaacaggct
ctattctatgtatcagatttggctaaggttcaagatagtggtcagaatggaacatatacattaattaattgtaat
tccacaaccatcaagcaagcctgcccaaaggtttcttttgagcccatcccaatacattattgtgctccagcagga
tatgccatctttaagtgtaatgacacagcatttaatggaacaggcttatgcaagaacatttcagtagttacttgt
acacatggtatcaagccaacagtaagtactcaactaatactaaatggaacaatctctgaaggaaacataagaatg
ctgggaaaaaatctgacaagtaatgagaatatcctagtgactctaaattccactataagcataacctgtgagaga
ccatacaatcagtcaatacaagagttaaggataggtccaatggcttggtacagcatgacattagaacaaaacaaa
acaggcagtaacataaggacagcttattgcaattataatggctctgactgggaaggtacattaaaaggagtagct
gaaagatatttagaacttataaatcagacaaacaatgctaccgtgacattcagtaatagtacgggtggagatcca
gagataagctttttgcattttaactgtcatggagaattcttttattgtgacacaagtaagatgtttaattatacc
tttaattgtaacaagaccaaatgtaataatagtaaaaatagtacccataataacactatgacaacaataccttgc
agaataaaacaggtagtaaggtcatggataaggggagagtcgggactctatgcacccccccattaaaggtaattta
acctgcatatcaaacataactggaatgattctattaagggacgagccagggaataacagcagacacaccccttagg
ccagtaggggggagatatgaaagatatatggagaactgaattagtcaattacaaagtagtaaaaataaaaccttttt
agtgtagcacctacaaaaattacaaggccagtcataagcacccatagagaaaaaagaaacctctggggctgtaag
ggaaagctaatttgctatacatcagtaaaatggaacaaaacatggggaggagatgagtcaatctggggtaaccta
acatggcagcagtgggatcagcagatagagaatgtaagctccatcatatatgaagaaatacaaaaggcacaagaa
caacaggaacaaaatgagaagaagttgctggaattagatgaatgggcctctctttggaattggcttgacataact
aaatggttgtggtatataaaaatagctataattatagtaggagcactaataggtgtgagaattatcatggtaata
cttagtctagtgaagaacattaggcagggatatcaacccctctcgttacaggcctgcagacaaacaagagcttta
ttacaatattggctacaagaattgcaaaaaagtgctacaagcttgctagataccattgcaataatagttggcaat
tggactgatagtatcatcttaggactacaaagaataggaaggggaattcttaacatcccaagaagaattagacag
ggccttgaacgctgcttagtgtaaatggggagtgcatggagtaaaagtaaattttcaggatggtcagcagtaaga
gatagaatgagacaaacctcccctaatcctgagccmtgtgcacctggggtaggagaaatttcyagacagttatca
gaaagagggggataacaaattcttatactcctcaaaacaatgcagcccttgcattcctcgaaagccaccaagag
gaggaagaagtagggttcccagtaagacctcaagtgcctctaaggccaatgacctataaaggagcatttgacctc
ggcttcttttaaaagaaaagggaggactggatgggttaatttactcccatagaagaaaagagatcctggatctt
tgggtgtatcacactcagggattcttccctgattggcagaactacacaccaggaccaggaaccagatacccattg
acatttggatggccatttaaactagtaccagtgacarmagaagaggcagaaagaatgggcaatcagtgtgaaagg
gctatgctcctacatccamtttgtaaccatggtagtgaggatgaagaaggggaaatactacaatggaagtttgat
agttcattagcgcgcacccatgttgctttaataactcacccagagctcttcaacaaggactaa
>#70 AY169805 7551 nt

atgggtgcgagtgcgtcagtgttgacaggaagtaaattggatgcatgggaacaaattaggctaaagccaggatct
aaaaagaaatataggctaaaacatttagtatgggcaagcagggagctggacagattcgcatgtaatcctgagcta
ctagaaactgcagagggcaatgagcaactgttacagcaattagagccagctctcaagacagggtcagaaagcctg
cagtcactytggaacacaatagcagtgctctggtgtgttcacaacagatataaaattggagatacgcagcaggca
atacaaaagttaaaggaagtcatggggaacaggaagtctgcgagtgccgctaaggaagacacaagtgcaaagcag
gcaggtcagaattaccctgtagtatcaaatgcgcagggacaaatgatacatcagcccatctcccctaggacttta
aatgcatgggtaaaggcagtagaagaaaaggcctttaaccctgaaatcattcctatgttcatggcattgtcagag
ggagctattccctatgatattaatactatgctaaatgccataggaggacatcaaggggctttacaagtgctaaag
gaagtaatcaatgaggaagcatcggagtgggatagaactcacccaccaccgatagggccgttaccaccagggcag
ataagggacccaacaggaagtgacattgctgggacaactagcacccagcaagagcaagttcactgggttaccagg
aaccccaaccctatcccagtaggagacatctatagaaaatggatagtgttgggactaaacaaaatggtaaaaatg
tacagcccagtaagcatcttagatattagacagggaccaaaagagccatttagagactatgtagatcggttctac
aaaacattaagagctgagcaagcaactcaagaagtaaaaaattggatgacagaaaccctgcttgttcaaaatgcc
aacccagattgcaaacagatttaaaatcattagggccaggagctaccttagaagagatgatggtagcctgtcag
ggagtaggaggaccaactcataaggccaaaatattagcagaagcaatggctgcagcccatcaagatctgaaagga
ggatacacatcagtattcatgcaaagagggcaaaacccaattaggaaagggcctataaaatgtttcaactgtgga
aaagagggacatatagcaaaaaattgtcgagcacctagaaagcgaggttgctggaaatgtggacaggaaggtcat
caaatgaaagattgcagaaatggaaarcaggcaatagcaagggtgggagrccatctatgtgaagttttactggat
acaggggcagatgatacagtactaaacaacatacaattggaaggaaaatggacaccaaaaatgatagggggtata
ggaggttttataaaagtaaaagaatatgataatgtgacagtagaaatagagggaagagaagtacagggaacagta
ttggtgggacctactcctgttaatatcattggaaggaatatattgacaggattaggatgtacactaaatttccct
ataagccccatagccccagtgccagtaaaactaaaaccaggaatggatggaccaaaaataaaacartggcccta
tctaaagaaaaaatagaagccctgacagcaatatgtcaggaaatggaacaggaaggaaaaatttcaagaataggr
cctgaaaatccttataatacacccatctttgctataaaaaagaaagatagtactaagtggagaaarttggtagac
tttagggaattaaacaagagaacacaagatttctgggaggtacagttaggtatcccacatccgggggtytaaag
caaaagaaatctgttacagtcctagatgtaggagatgcttatttctcatgccccttagacccagacttcagacca
tatactgctttcactatcccaagtgtaaacaatgagaccccaggaataagataccagtacaatgtcctcccgcaa
ggatggaaaggttcaccagccatattccaaagttcaatgataaaaattctagatccatttaggaaagacaaccca
gaattacaaatttgtcaatacatggatgacctctatgtaggatcagatttacctctgacagaacatagaaaaagg
gttgaattgcttagagaacacttatatcagtggggattcactaccccctgataaaaagcatcaaaaggagcctccc
tttctgtggatggggtatgagctccatccagacaaatggacagtacagcccatccaattgcctaacaaggaagtg
tggacagtaaatgatatacaaaaactaataggaaagttaaattgggcaagtcaaatctatcaaggaattagagta
aaagaattgtgtaagttaattagaggcaccaagtcattgacagaagtagtacctttaagtaaagaggcagagttg
gaattagaggaaaacagagagaagctaaaagaaccagtgcatggtgtatactatcaacctgacaaagacttatgg
gttaatattcagaagcaaggagaagggcaatggacttaccaaatatatcaggatgaacataagaatctcaaaaca
gggaaatatgctaggcaaagagcctcccacacaaatgagataagacaattagcagaagtactccaaaaggtgtcc
caagaagctatagttatctggggaaaattgcctaaatttaagctgccaattactagggaaacttgggaagcatgg
tgggcggactactggcaagccacctggattccagaatgggaatttgtcagtacacccccattgatcaaattatgg
taccaattagaaagagaacctattatagggcagaaacttattatgtagatggagcagctaatagaaatacaaaa
ctaggaaaggcaggatatgttacagaacaagggaaacagaaaataataaaattagaagagaccaccaatcaaaag
gctgaattaatggcagtattattagccttacaggattccaaagaaaaggtaaatatagtaacagattcacaatat
acattgggcatcatctcatcacaacctacacagagtgaatcccctatagttcaacagataatagaggaactgaca
aaaaaggaaaaggtgtatcttacatgggttcctgctcataaaggcataggaggaaatgaaaaaatagataaatta
gtgagcaaggatattagaagagtcctattcctagarggaatagaccaggcacaggaagatcatgaaaaatatcat
agcaattggagagcattagctagtgactttggactaccaccagtggtggccaaggaaatcattgctagctgtcct
aaatgtcatataaaaggagaagcaatacatggtcaggtagactgcagtccagaagtatggcagatagattgcaca
catgtagaaggcaaagtcataatagttgctgtccatgtggcaagtggattcatagaagcagaagtaataccagca
gaaacaggacaggaaactgcttacttcctgttaaaattagctgcaagatggcctgttaagataatacatacagac
aatgggcctaatttacaagtgcaactatgaaggctgcatgttggtggactaacatacaacatgagtttggaata
ccatataatccacaaagtcaaggagtagtagaagccatgaataaggaattaaaatcaatyatacagcargtgagg
gaccaagcagaacacttaagaacagcagtacaaatggcagtatttgttcacaattttaaaagaaaagggggggatt
ggggggtacactgcaggagagaggataatagacatactagcatcacacatacaaacaacagaattacaaaaacaa
attttaaaaattcaaaattttcgggtctattacagagacagcagagaccctatttggaaaggaccggcccagctc
ctgtggaaaggtgagggagcagtagtcatacaagataaaggagacattaaagtagtaccaagaagaaaggcaaaa
ataatcagagattacaggtctaggaagaccaaggactggtattacagacatcattatgaatgtagaaatccaaga

atcasttcwagtgtatatattccaatagggccggcttttatagtagtgaccacatattggggattgatgccagga
gaaagrgatgaacatatgggacatggggttagtatagaatggcagtacaagaagtatgcaacacagattgaccct
gaaacagcagacaggatgatacatctgcactattttacctgttttacagaatcagcaatcaggaaagccatccta
gggcaaagagtactgaccaagtgtgaataccctgcaggacatagtcaggtagggacactacaactactagctcta
agagctgtagtgaaagaaaaaagacataagcctcccctacccagtgtccagaaattaacagaagatctcctagaa
gagctaaaagcagaagcagtaagacatttccctaggccttggctacagggcttgggacaatacatttatgayact
tatggggacacttgggaaggggttatggcaattataagagtcttacaactactgatatttgcccattttagaatt
ggatgccaacatagtagaataggaattaacccatctaacacaagaggaagaggaagaagaatgccccccttggcat
caccctggaagtcagcccctgaccccttgtactaagtgctattgcaaaaaatgctgctatcattgctatgtttgt
ttcacaagcaagggtttgggaatctccctgtatgcaacagtctatgctggggtacctgtatgggaagaggcagaa
ccagtattattctgtgcctcagatgctaacctaacaagcactgagcagcataatgtttgggcatcacaagcctgt
gtccccacagaccccactccacatgagtatccattacgcaatgtgacagataaatttaatatatggaaaaattac
atggtagaccaaatgcaggaagacattattagcctatgggaacagagcttaaaaccttgtgttcaaatgactttc
ctgtgtatacaaatgaattgtacagacatacaaatcaattctaaagaaaacaactctacaaacgatagctcaata
aaccctataagcacagagaaccctaacagcatagagaaccctatgaaaaattgtgagtttaatgtaactacagtt
ctcaaagataaaaaggagaaaagacaggctctattctatgtagcagacttggatagagtgaataatagtgaaata
ggtaacacagcatatacattaattaaytgtaactccacaaccatcaagcaagcctgtccaaaggtatcttttgag
ccaattccaatacactattgtgctccagcaggatttgccatcttcaagtgtaacagcacagagtttaatggaaca
ggcacatgccacaacattacagtagttacttgtacacatggtatcaagccaacagtaagtactcagctaatatta
aatggaacactttcggaaggaaagataagaataatagcaaaaaatatttcagctactggcaacaatatcatagtg
accttaaatactactataaacatgacctgccagagaccaggacatcaagatgtacaagagataatgacaggtcca
ctggcctggtatagcatggaactaaagaaagacacaaacacatccaaatcaagaatagcttattgtgagtataat
accaagaagtgggaagaggccttaagacagacagctgaaaggtatttagaattagtaaacaatacaggtgttaga
gaaatgatattcaattacaatactggaggagatccagagataacccagttgcattttaattgtcatggagagttc
ttttactgcaacacatctcagatgtttaattatatctttaactgtaccggaaccacctgtaattataccaaaaac
atccaaaattatacctttagccaaaataccactagcactaagataccttgcagattgaggcaggtagtaaggtca
tggatgaggggaggatcgggaccctatgcacctcccatccgaggtaacttaacatgtggatcaaacataactgga
atgattctagaaatggatgagccatggaacgagagccacagggaaagcatatttagaccaacaggggggaaatatg
aaagatatatggagaactgaattgctcagatacaaagtagtaaggataaaacctttagtgtggcacctacaaaa
attacaaggccagtcataggctttggcacccgcagagaaaaaagaaactcatggggctgtaaaggaaggctagtc
tgttacacatcagtaatatggaacacatcagaatggaaaggaaatgagtctatttggggcaacctcacatggcag
caatgggatcaggagatagacaatgtaagtgccaccatatttgaagaaatactaaaagcacaaatacagcaggaa
acaaatgagaaaaagctgctggagttagatgaatgggcttcyatttggaattggcttgacataactaaatggttg
tggtatataaaaacagcaataatcatagtaggagcactaataggtataagaattgtcatggtaatacttaattta
gtgagaaacattaggcagggatatcaacccctctcgttccaggcttgcagaatttgtgcagctgtaatacaatac
tggcttcaagaattgcagaatagtgctacaaatctagtagatactattgcagtagcagtagctaattggactgac
agtataatcttagggatacaaagaataggaagaggaatctggaacgtccccaggagaattagacagggatttgaa
agaagtttgttataaatgggaaacgcattgaggaaaagcaaatttgcaggatggccagcagtaagagaaagaatg
agaagggcttcccctaaacctgaagattgtgcacctggagtaggagaagtctccagggaattagcatctagagga
ggaataccaagttcctatactcctcagaacaatgcagcccttgcatttctagagagccaccaagatgaagatgta
ggtttcccagtaacaccccaagtgcctctaaggccaatgacctwtaaaggagcatttgacctcagcttcttttta
aaagaaaagggaggactggatgggttaatttattcccagcaaagagcagagatcctggatctttggatctatcac
actcagggattcttccctgattggcagaactacacaccagggccaggaactaggttcccactgacatttggatgg
ttatttaagctagtaccagtgacagaagatgaggcaaaaagactaggaaatgtgtgtgagagggctcatctcctg
catccagcttgtaaccacggctttgaagatccacacggagagatactgaaatggcagtttgacagatcactgggc
aacacccatgttgctaagataacccacccagagttcttccccaaggactaa
>#71 HQ179987 7557 nt
atgggtgcgagagcgtcagtgttgacaggggggccgattggatgcatgggaaagaattaggcttagaccaggaggt
aagaaaaaatataagctaaaacatgtagtatgggcaagcagagagctggaaagatttgcatgtaatcctgggctt
atggaaacagcggaaggctgcgaaaaattattagagcagttagaaccagctctcagaacaggctcygatggcctg
cagtctctttggaacaccctagtagttytatggtgtgttcacaaaagaatagagataagtgacacacagcaggcc
attacaaaatggaaggaggaaatgcagaaaagaaagaaaacatcagagggcagcactggaacaagtcaaaactat
cctatcgtgcagaatgcccaggggcaaatgacccatatgccgctgtcccccaggacgctgaatgcctgggtgaag
gcagtagaagaaaaagccttcaaccctgaaattatccctatgtttatggccttgtcagaaggggctatacctgat
gacatcaataccatgcttaatgcagtaggcggacatcagggagcgttgcaggtgttgaaagaggtaattaatgaa

gaggctgcagaatgggatagaacacatcccgttccagtaggaccattaccaccagggcagcttagagaaccaaca
ggaggggatattgcaggaaccactagtaccaaacaggaacagataacctggatgacaaggaacaatcctgtacca
gtaggggacatctatagaaartggatagtgttggggctcaataaagtggtaaaaatgtactgccccgttagcatt
ctggacataaagcagggacctaaggaaccatttagagattatgtagayagattctacaaaaccctcagagcagag
caagccagtcaggaagtaaaaagttggatgacagacaccttactagtacaaaatgctaatccagattgyaagcag
attttgaaagctctgggaccaggtgccaccttagaagaaatgatgaatgcctgtcaaggagtaggggggaccaaca
cataaggccagggtcttggcagaagctatggcagcagctaatcaagctagccaagaattaaaaggagggtataca
acagtttttatgcagagtggacagagaaagccagttaagtgctttaactgtggaaaagtaggacacatagcaaag
aactgcaaggcacctagaagaaggggrtgttggaagtgtggacaggaaggtcatcaaatgaaagactgcaaatca
ggaagacaggcaccagtaaaaatagggggcatatttgtgaggctctattagatacaggggctgatgacacagta
gtagataacttacctttagaaggaagatggaaaccaaaaatgataggggggaataggrggttttataaaagtaaaa
gaatttgaagaagttaagrtagaaatagaaggaagacaagtttatggractgtattagtgggyccaaccccagta
aatataataggaagaaatattttgacattaattggatgtactttgagtttccccatcagccccatagaaatagta
ccagtaaaattaaaagcaggaatggatgggcctagggtgaagcaatggcccttgtcaaaggaaaaaatagaagct
ttaagagccatctgtcaagagatggaacaggaaggaaaaataacaaaaattgggcctgaaaatccatataacacc
cccatttttgcaataaaaaagaaagatggcagcaagtggaggaaattagtagacttcagagaactaaataaaaga
acacaagatttctgggaagtccaattgggratccctcatccaggaggtcttcagaaaagraaatcagtgaccata
ttagatgtaggrgatgcttatttctcctgycctttagatccagattttagaaaatatacagctttcactatacct
agtgtaaataatgacacaccaggacttagrtatgtgtayaatgttctgcctcagggatggaagggatcaccagcc
attttycagcattcaatgactaagatcttagaaccctttagraagagtaatccagaagtagaaatctaycaatac
atggatgatytatatgtagggtcagatytgcctttgtctgaacatagacagcgggtagagaaactyagggarcac
ctctatgtatggggttcacaactcctgacaaaarcatcaaaaggagcctcctttcctctggatgggatatgag
ctccatcctgacaagtggackgtgcaacccatcaaattaccagaaaaggaaagttggacagtaaatgacattcag
aagctagtaggaaagttaaattgggctagtcaaatttatccaggaattagggtaaaagagttatgyaaactaatt
agaggaactaartccttaacagaagtagttgcytttactagagaagcagaattagaactagaggaaaataaagaa
attttaaaagaaccagtgcatggagtttattaccaaccagaaaaggaattaatagtagacatacagaaacagggg
gcgggacaatggacttatcaggtattccaggaagaacataaaaacttgaaaacagggaaatatgccaggcaaaag
gctacccacaccaatgatataaggcaactggcagargtaatacagaaggtatcacaagaaagcatagtgatatgg
ggaaaactacccaagtttagactaccagtaaatagaaatgtgtgggagacttggtggtcagactattggcaagcc
acctggatacctgagtgggaatttgttagcacacccctcttattaagctctggtatcagctagaaaaagacccc
ataccaggaacagaaacctttttatgtagatggggcagcaaacagagagaccaagttaggyaaagctggatatgta
acagataggggtaggcagaagataatcaaactagaagaracaactaatcaaaargcagaattagaagcagtgttg
ttagccttaaaagaatcaggagaacaggctaacatagtaacagactcccaatatgtgttaggrattatctcagca
actccagatcaaagtgactcccccytagtgcaaaaaataatagaagaaatgacaaaaaaggaaaaggtatacctr
tcatgggtaccagcacacaaaggcataggggggtaatgaraayatagayaaattagtcagyaargacattagaaga
gtgttattcttggaaggcatagaccaagcacaggaggatcatgaaaaatatcacagtaattggagagccctagct
agtgartttggccttccaccaatagtggcaaaagagattatyaacaattgccctaaatgtcatgtaaaaggggaa
gccatgcatggacaggtagactgcagtccaggaatttggcaactggactgtacccacatggarggaaaaattatc
ctcgtggcagtccatgtrgccagtggcttcatggaagcagaagtaattccagtagaaacaggacaagaagctgca
tactttgtgctcaaattggcatcaagatggcctgtaaaagtaatacacacagacaatgggcctaactttactagc
gctgcagtaaaagcagcctgttggtggcttaatataactcatgagtttgggataccctacaatccccaaagtcag
ggagtagtggaatcaatgaataaagaattaaagaaaattatacatcaggtacgagatcaagctgagcacttaaag
acagcagttcaaatggcagtatttgtccacaattttaaaagaaaaggggggattgggggtacactgctggagac
aggatcatagatattctggctacacaratacaaacaacagaattacaaaaacaaattttaaaaattcaaaatttt
caggtctattacagagacagcagagaccctatttggaaaggaccagcgacactcctgtggaaaggtgaaggggca
gtagtcatacaagacaaaggagatattaaggtagtccctaggagaaaagcaaaaataattagaaattatcacaaa
tataggtctaaaaagacaagaaagtggttttataggcatcattatgagacaacccatcctaggattagttcagca
gtttatattccagtaggaacagcaaccattattgtgactacytattgggggctcatgcctggggaaagagaagaa
caattaggacatggagcaagtgtggagtggagacaaggtaaatacaccacacagatagatccagaaacagcagat
aggctaattcatctccactactttcaatgtttttcagattcggctgtgaggagggcaatactaggggacagggta
ttgaayamatgtgaatactcagcaggacatagtcaggtaggctccttgcagtatttagccttaaaagtggtagta
gggaaggtaaaraggaagccacccctccctagtgtccagatattgacacaagacgaactcaaggaggaagcagta
agacacttccctaggccttggttacactcattaggacagtatatctataatacctatggggacacctgggaggga
gtaactgcaattattaggatcctacaacaattaatytttatccattatagaattggatgccaacatagtagaata
ggtatcttgacaccctctcgaagaggaaggagactgcctccatggcaacagccagggagtcagccctcaagccca

tgtaacaattgctactgcaaagcctgctgctatcattgctatgtttgtttcacaaagaagggtttgggaatctcc
gctattagctatactagtggaacacgttatgttactgtatattatggggttccagtttggagagatgctaaaatt
accttattctgtgcagcagatgcatcgctaactagcarwgagcagcataatatttgggccacccaggcctgtgtg
cccacagaccctagaccaatagaggtcaggatagataatgtaacagagtcttttaatatttgggacaattatatg
gtgacacaaatgcaggaagacatcattagcttatgggatcagagccttaaaccttgtgtaaaattgacagttcta
tgtgttactatgrattgtagcgactgcagcacggttgactgtactaataactcctccagggttaataacagcacc
gacagcaccaacaccagcaaaaccaatccattagaattatttcagtgcagttttaatacaaccacaatagtaaaa
gataaacagcagacacagcaagcactcttttatagagcagacctaacaaaattggataatgacaatagtacatat
agattaattaattgcaacaccactaccattacacaggcatgcccaaaagtgaactttgagccgctacccatacag
tattgtgcaccagcagggtatgcactaatgaaatgcaatcagacaggatttaatggtacaggaccttgtaataag
acagttataacacactgtacacatggaattaagccaacagtgtcaacacaattaatactcaatggaactctagca
aagggagagcccttagtaattactcagaatgtgtcagatacaggaaaagtcatcatagtaaaattaaatgagagt
gtgtcactcacctgtataagaccaggtaataacacaagaggacaagtgcaactaggagtcatgacatggtataat
atgaaacactacgtaggcgatatcagggcagctcattgtaatgtttctagggaaaattggaaaagaaccttagaa
tgggtcagtgaggccatctggaaggcttatcctcaacattccaaccatacatccaaacacaaccatacatttgtt
tttaaaaacagtacaggggggagacccagaggtttcttcgctgcacttcagttgtcatggggaattcttttattgt
aacaccagcagcctgtttaactttagttatacttggaatgacaccacctggcgggctgcaggcaatcataataca
agtgaaaatataacactgtcttgtagactgaagcaagtagttaattcatggatgagagtgggatcaggcttgttt
gcgacacctattgaggggggaattaaagtgtcaatccaacattacaggtttaatgttagaaagagaactaccttac
aatgacagcagcaagaacaccactttaagtcctataggggggagacatgacaaatatctggagaagtgagttatat
ccctacaaagtagttcaagtaaaagctctggccgtggcacctacaaaaatttccagacctacaattatggctcat
gatggacataggaagaaaaggggactgtggggatgctcaggacaaatartatgttatactaatgtgccatggaat
agtacctggaccaacaaaaatgaaacagaattagatggcatttggggtaatctaacatggcaggagtgggacaaa
ctggtggataattacactgacacaatttacttggaaatacagaaagcacaagagcagcagaaggaaaatgaaaga
aagctattagaattagacaaatgggcacaattgtggagctggatggacataacaaaatggttgtggtacataaaa
attttcattatgatagtaggaggaattataggactaaaaatcttaatggctataggtaatgtagtcaggaaagtc
aggcagggataytcacctgtrtcgttgcagtgcctgcgagactgctttgctgcgtgtggctattggactcaagaa
ttaaaacaragtgcaaccagcytgttgaacactgttgctatatcagttgcaaattggactgatcaagtaatagca
gtagggcaacaaataggaagaggcttcttgaacataccaagaaggttaagacaagggctagaaagaagcttactg
taaatggggaatgcatggaagaaaagtagcttagtgggctggccagcagtcagggaaaaaataaagcagactacc
ccgactacccctgacccgactaccccagtaacacctgcacccggggttggggaaatttccaaagaattagcacaa
ggaaaaggaatacccagtaaatttagttcaaagaacaatgcagcattggccttcttggatgctcatgaggaagaa
gaagtaggrttcccagtcaggcctcaagtacccttaagatgcatgacatacaaggcagcatttgacctcagcttc
tttttaaaagaaaagggaggactggatgggttagtttactcacctgagagagcagagatcctagatctctggatc
tatcacactcagggattcttccctgactggcagaattacactccagggccaggagaaagatatcccctgacctttt
gggtggctgtttaaactagtaccagtctctgaggtagaagctgaggaaatgggagataagcaggagaaagctaag
ctgctacatccagcctgcacttatgggttttcagatcctcataaggagatcctagtgtggaagtttgacagctca
cttggaagagaacatgttgccttacaaaagcacccggaactgtttattaaagactaa
>#72 GU111555 7521 nt
atgggtgcgagagcgtcagtgttgacaggggggccgattggatgcatgggaaagaattaggcttaagccaggaagt
aagaaaaaatatatgctaaaacatatagtatgggcaagcagagagctggaaagatttgcatgtaatcctgagctt
atggaaacagcagaaggctgtgaaaaattattagagcagttagaaccagctctcagaacaggttctgatggcctg
cagtctctttggaacaccctagtagttttatggtgtgttcacagaagagtrgatataagtgacacacagcaggcc
attacaaaatggaaggaggaaatgcagagaagaaagaaagcactagagggcagcactggaactggaacaagtcaa
aactatcctattgtgcagaatgcccaggggcaaatgacccatctgccgctgtctcccaggacgctgaatgcctgg
gtgaaggcagtagaagagaaggccttcaaccctgaaattatccctatgtttatggccttgtcagaagggtctata
cccgatgacattaatactatgctcaatgcagtaggcggacatcagggagcrttrcaggtgttgaaagargtgatt
aatgaagaggctgcagaatgggatagaacacatcctgttccwgtrggaccgttaccaccagggcagcttagagac
ccaacaggaggggatattgcaggaaccactagtaacaagcaggaacagataacctggacaacaaggaacaataat
cctataccagtaggagatatctatagaaagtggatagtgttggggctcaataaagtggtaaaaatgtatagcccc
attagcatcctggacataaagcagggacctaaggaaccatttagagattatgtagatagattctacaaaacyctc
agagcagaacaagctagtcaggaagtaaaagagttggatgacagacacattattaatacaaaatgctaatccagat
tgtaagcagattttgaaagctctgggaccaggggccaccttagaagaaatgatgaatgcctgtcaaggagtaggg
ggaccaacacataaggccagggtcttagcagaagctatggcagcagctaatcaagctaaccaagatttaaaagga
gggtatacaactgttttcatgcagagtggacagagawagccagttaagtgctttaactgtggaaaaataggacac

atagcaaagaactgtaaggcacctaggagaaaaggatgctggaagtgtggacaggaaggtcatcaaatgaaagac
tgcaaatcaggaagacaggcaccagtaagagtagggggcatatttgtgaggctttattagatacaggagctgat
gacacagtagtagataaattacctttagaaggaagatggaaaccaaatatgataggaggaatagggggttttata
acagtaaaasaatttgaagwagttaaggtagaaatagaaggaagagaagtttacggaactgtattagtrggccca
accccagtaaayataataggaagaaatattttgacattaattggatgtactctaagtttccccatcagctccata
gaaatagtaccagtrawattaaaggcaggaawggatgggcctaaggtaaagcmatggcccttrtcaaargaaaaa
atagaagctttaagagstatttgtcaagagatggaacaggaaggaaaaataacmaaaattgggcctgaaaatcca
tataacacccccatctttgcaataaaaaagaaagatagcagcaartggaggaaattagtakacttcagagaacta
aataagagaacacaagattttgggaagtacaghtgggaatccctcatccaggaggtcttaagaaragraaatca
gtgacagtattagatgtgggggatgcttatttctcctgtcctttagatccagattttagaaaatatacagctttt
actatacctagtgtaaataatgagacaccaggaattagatatcagtataatgttctgcctcagggatggaaagga
tcgccagccatctttcagtattcaatgactaagatcttagaaccctttagggagagtaayccagaagtagaaatc
tatcaatacatggatgatctatatgtagggtcagatttgcctttgtctgaacatagacagcgggtagagaaactt
agggaacacctctatgtatggggtttacaactcctgacaaaaaacatcaaaaggagcctcctttcctctggatg
ggatatgagctccatcctgacaagtggacggtgcaacccatcgtattaccagaaaaggaagcttggacagtaaat
gacatccagaagctagtaggaaagttaaattgggcaagtcagatttatccaggaataagggtaaaagaattrtgc
aaactaattagaggaactaaatccttaacagaagtagttgctcttactagagaagcagaattagaactagaggaa
aataaagaaattttaaaagaaccagtgcatggagtttattaccaaccagaaaaggaattaatagtagacatacag
aaacagggagagggacaatggacttatcagatataccaggaagaacataaaaacttgaagacagggaaatatgcc
aggcaaaaggccactcacaccaatgatataaggcaactggcagaagtaatacagaagacatcacaagaaagcata
gtaatatggggaaaattacccaagtttagactaccagtaaatagaagtatgtgggagacttggtggtcagactat
tggcaagccacctggatacctgaatgggaatttgttagtacacccccctcttattaagctctggtatcagctagaa
aaagaccccataccaggaatagaaacctttatgtagatggggcagcaaacagagagactaaattaggyaaagct
ggatatgtaacagataggggtaggcaraagataatcaaactagaaggaacaaccaatcaaaaagcagaattagaa
gcagtgctgttagccttaaaagaatcaggaaaacaggctaacatagtaacagactcccaatatgtrttagggatc
atctcagcaactccagatcaaagtgactccccccctagtgcaaaaaataatagaagaaatgacaaaraaggaaaaa
gtatacctatcatgggtaccagcacacaaaggcataggggggtaatgaagatatagayaaaytagtcagtaaagac
attagaagrgtgttattcctagaaggcatagaycaagcacaggargatcatgaaaaatatcacagtaattggaaa
gccctagctagtgactttggccttccaccaatagtggcaaaagagattatyaacaattgtcccaaatgtcatgta
aaaggggaagccatgcatggacaggtagactgcagtccaggaatttggcarctggactgtacccacacagaagga
aaaattatccttgtagcagtccatgtagccagtggtttcctggaagcagaagtgattccagcagaaacaggacar
gaaactgcatayttttgtgctcaaattggcagcaagatggcctgtaaaagtgatacacacagacaatgggcctaac
ttyactagygctgcagtaaargcagcctgttggtggcttaatataactcatgagtttgggataccctacaatccc
cagagtcagggagtagtggaatcaatgaataaagaattaaagaaaatcatacttcaagtacgagatcaagctgaa
cacttaaagacagcagttcaaatggcagtatttgttcacaattttaaaagaaaaggggggattgggggtacact
gctggagacaggatcatagatattatggctacacaaatacaaacaacagaattacaaaaacaaattttaaaatt
caaaattttcgggtctattacagagacagcagagaccctatttggaaaggaccagcgacgcttctgtggaaaggt
gaaggggcagtagtcatacaagacaaaggagatattaaagtagtccctaggagaaaagcaaaaataatcagagat
ctggtcaaatatcacaaatataggtctaaaaagacaaagaattggttttataggcatcattatgagacagtccat
cctaggattagttcaggagtttacattccagtaggagcagcaaccattgttgtgactacttattgggggcttatg
cctggggaaagagaagaacaattaggacatggagcaagtgtggagtggagacaaggtaaatacaccacccagata
gatccagaaacagcagataggctaattcatctccactactttcaatgtttttcagattcggctgtgaggagggca
atactaggggacagggtattgaacaagtgtgaatactcagcaggacatagtcaggtaggctccttgcagtttta
gccttaaaagtggtagtagggaaagtaaaaaggaagccacccctccctagtgtccagatattgacacaagatgaa
ctcaaggcggaagcagtaagacccttccctytgcaatacttacactcattaggacaatacatctataatacctat
ggggacacctgggagggagtaactgcaattattaggatcctacmacaattaatttttatccattatagaattgga
tgtcaycatagtagaataggtatattgccaccctctcgaagaggaaggagactgcctccatggcagcagccaggg
agtcagccctcaagcccatgtaacaattgctactgcaaagcctgctgctatcattgctatgtttgtttcacaaag
aagggtttggggatctccgctattagctgtgctagtggaatacactatgttactgtatattatggggttccagtt
tggagaaatgctgaagttaccttgttttgtgcagcagatgcatcgctaactagcaaagagcagcataatatttgg
gccacccaggcctgtgtgcccaccgaccctacaccaatagagatcccgataaatgtaacagagtcttttaatatt
tggaaaaattatatggtgacacaaatgcaggaagacatcattagcttatgggatcagagccttaaaccttgtgta
aaattgacaatcctatgtgtcactatgaattgtagtgaatgccacagggaggctagctgtaataataacagtagc
tcctctgtgaatakcaccgccgacaatccattagatctatttaagtgcagttttaatacaaccacagtattgaaa
gataaaaagcagggacagcaagcactcttctatagaacagacctaatagcactgaatgagacagcaaataataca

ctatatagattaattaattgcaacaccactaccattacacaagcatgcccaaaagtgacttttgagccactaccc
atacagtattgtgcaccagcagggtatgcactaatgaaatgcaatcagaaaggatttaatggtacaggaccttgt
aatcagacagtcataacacactgtacacatggaattaagccaacagtatcaacacaattgatactaaatggaact
ctagcagaggaagagcccttagtaattactcaaaatgtgtcagacacaagatatgttatcatagtaaaattaaat
aagaatgtgtcaatcacctgtgtaagaccaggtaataacacaagaggacaagtgcaaataggacccatgacctgg
tataatatgaaattctacacaggtgatatcaggaaagcttattgtaacatttccatgcaaaattggacagaaacc
ttaaaatctgtcagtgaggccatctggaaagcttatcctcaacccccaaccaaaaccatacatttgtctttcga
aacagtacaggggagacccagaggtttcttttctgcactkcaattgtcatggggaattcttttattgtaacacc
agtaccctgtttaattatagctatacttgtaatacctcgggtgcatgtacaggtaaacacacaggtgagaacatg
acaataacactgccttgtagactaaagcaagtagttaattcatggatgaaagtgggatcaggcttgtttgcgcca
cctatcgaggacaattgcagtgccactccaacattacaggtttaatattagacagagcatcaccttataatgcc
aacagcagcaacaccactttaagccctacaggggagaaatgagaaatatctggagaagtgagttatatccctac
aaggtagttcaagtaaaggctctggctgtggcacctacaagagtctccagacctacaatcatggctcatgatgct
cataggaaaaaaaggggactgtggggatgctcaggacaaacagtatgttatactaatgtgccatggaataggagc
tggaccaacaaaagtgagactgaattagatggcatttggactaatctaacatggcaggaatgggacaaamtggtg
gataattacactgacacaatttatttagaaatacaacgagcacaagatcarcaaaaggctaatgaaaaaaagtta
ttagagttagaccaatgggcacaattgtggaactggttggacataacacaatggttgtggtacataaagatttc
attatgatagtaggaggaataataggactaagaatcttgttagctataattaatgtagtcaggagaatcaggcag
ggatactcacctgtatcgttacagtgcctgcgagactgctttgctgcgtgtggctattggactcaagaattaaaa
caaagtgcaaccagcttattggacactgttgctatatcagttgcagggtggactgatcaagtaataatagtaggg
caacaaataggaagaggcttttgaacataccaagaaggataagacaagggatagaaagaagcttgctagggaat
gcatggaagaaaagtagcttagtgggctggccagcagtcagagaaagaatgaatcagactgccccagaaacacct
gcatctggggttggggagatttccaaagaattagcacaaggaaaaggaatacccagtaaatataattcaaagaac
aatgcagcattagccttcttagaagctcatgaagatgaagaagtaggattcccagtcaggcctcaagtacccgta
agacccatgacatacaaggcagcatttgacctcagcttcttttaaaagaaaaggggggactggaagggttaatt
tattcacctgggagagcagaaatactagatctctgggtctatcacactcaggggttcttccctgactggcagcat
tacactccagggccaggagaaagattcccactgacctttgggtggctgtttaaactagtaccagtctctgaggta
gaagttgaggaaatgggagatgagcaggagaaagctaagctgctacatccagcctgcacttatggggattcagat
catcatagggagatcttaatgtggaagtttgacagatcactgggagaaagacatgttgccttacaaaagcacccg
gaactgtttactaaagactaa
>#73 DQ017382 7401 nt
atgggtgcgagagcgtcagtgttaacaggggaaaattagataaatgggaagcaatttatttgagaccaggggga
aagaaaaaatacaaaatgaaacatttagtatgggcaagcagggagctggaaagattcgcttgtaacccaggtctc
atggacacagcgcacggctgtaaccagttactaaaccaattagaaccagctctcaatacagggtcagaaggactg
cgctccttatataayaccatagcagttctttattgtgtccatagtacaataccggtactcaatacacaagaggct
ttggacaagataaaagagaaaaaggaacagcacaagtctgaggcaaaaaatccagaagcaggggcagcggcagca
gctgataacaatatcagtaggaactatcctctagtccagaatgctcaaggacaaatggtacatcatccgctgaca
cccagaactttaaatgcttgggtaaaagtaatagaggagaaggcctttaatccagaagtaataccaatgtttatg
gccttgtcagaaggggcaacaccctcagatctaaattctatgttaaatacagtaggaggacatcaggcagctatg
caaatgctaaaggaagtcatcaatgaggaagcagcagaatgggacaggacgcatccagtccctgtgggaccacta
cccccagggcaactgagagaacctagaggragtgatatagcagggacaactagtactctgccagaacagataaca
tggatgacttctaatcctcctattccagtaggagatatttatagaagatggatagttttggggttaaacagaatt
gtgagaatgtatagtcctgtcagcattttagagatcaagcaaggaccaaaagaaccctttagagattatgtagac
aggttctacaaaactttaagagcagagcaggcaacacaggatgtaaagaattggatgacagaaacactcttagta
caaaatgcaaatccagattgtaarcagatcctaaaagcattaggaccatcagctagcttagaagagatgatgacg
gcctgccaaggagtgggggaccagggcataaggcaagagtgctagcagaggctatggcacaggcacaggcagca
actagtgtctttgtacaaagaggaaactttaaggcacaaggagaaccattaaatgtttcaactgtggcaaagag
ggccatttggcaaggaactgtaaggctcctagaagarggggctgttggaagtgtgggcaagaaggacatcaaatg
aaagattgtaaaaatgaaggaagacaggctataggaaatgaagtaagagaagctcttttrgatacaggagctgat
gatacagtaatagaagaaatacaattagaaggaagatggaaaccaaaaatgataggaggaattggaggatttatc
aaagtaagacaatatgataatataacaatagacatacaaggaaggaaagcagttggtacagtgttggtaggacca
acacctgttaatattataggaagaaactttttaacacagattggctgtaccctaaattttccaataagtcctatt
aaaactgtaccagtaaaattaaaaccaggaatggatggcccaaaagtaaaacaatggcctttgacagcagaaaaa
atagaggcattaagagaaatttgtacagaaatggaaaaggaaggaaaaatttctagaatagggcctgaaaatcca
tataacactccaattttgctataagaaagaaagatagcaccaaatggagaaaattagtagatttcagagaatta

aataaaaggacccaagacttttgggaagtgcagctaggaattccacatccagcaggattaaagcagaaaaagtca
gtaacagtcctagatgtaggagatgcttatttttcatgtcccttagatgaagatttagaaaatatacagctttt
accatacctagtgtaaataatgagacacctggtattagataccagtataatgtgttaccacagggatggaaggga
tcaccagcaatttttcaaagttcaatgacaacaattytagaaccatttagaaagaagcatccagaaataatcatt
tatcagtacatggatgacctctatgtgggatctgacttagacctagcacaacatagagagacagtagaagaactt
agaggtcatcttttgaagtggggctttacaacccctgacaaaaaacatcaaaaggagccaccgttcctctggatg
ggatatgagctccatccagataaatggacagtccagccaataaagttaccagaaaaggatacatggactgtcaat
gatatacaaaaattagtaggaaagttaaattgggcaagtcagatctatccaggaatcaaagtaagacagctttgt
aaattaatcagaggaaccaaggctttgacagaagtagttacctttacagaagaagcagaattagaactagcagaa
aatagggagatattaaaagaacccctacatggagtctattatgacccagaaaaagaattaatagcagaaattcaa
aaacaaggacaaagtcagtggacatatcagatttatcaggaatcayataaaaatttaaagacaggaaagtatgca
aaaatgaaatctgcccatactaatgatataaaacagttaactgaagtagtacaaaaggtagcaacagaaagtata
gtaatttggggaaagactcctaagtttaaattaccmgtacaaaaggaagtgtgggaggcatggtggactgagaat
tggcaagcaacttggattccagagtgggaattcgttaacactcctccccttgtaaaattatggtatcagttagaa
acagagccaattagtggggcagaaacatactatgtagatggagcagctaataaagaaacaaaattgggaaaagca
ggttttgtgacagatagaggaagacagaaagtggtctctcttgaaaacaccaccaatcaaaagactgagttacaa
gctatccttatggccttacaagatkcaggacaggaagtaaacatagtcactgattctcagtatgctatgggaata
attcactcacaaccagataagagtgaatcagaattggtgagccaaataatagaagaactcataaaaaaggaaaga
gtctatctctcttgggtrcctgcacataaaggtattggaggaaatgagcaggtagacaaattagttagctcagga
attagaaaagtattatttctagatggtatagaaaaagcccaagaagagcatgaaagatatcacagtaactggaaa
gcaatggccagtgatttaacttgcccccccatagtggcaaaagaaatagtagccagctgtgacaaatgccagcta
aaaggggaagccatgcatggacaggtcaattgtagtccaggagtgtggcagttagattgtacacacttagaagga
aaaatcattcttgtagcggtccatgtggccagtggctacttagaagcagaagttattcctgcagaaacaggacak
gaaacagcatattttattttaaagttagctggaagatggccagtaaaagttatacacactgataatggacccaat
ttcactagtgccactgtaaaagcagcctgttggtgggcaagtatcaaacaggaatttgggataccctacaatcct
caaagtcagggagcagtagagtccatgaataaagaattaaagaaaattataggacaaatcagagatcaagcagaa
catctgaaaacagcagtacaaatggcggtcttcattcacaattttaaaagaaaaggggggattggggggtacact
gcaggggaaagaataatagacataatagcaacagacatacagacaacaaaattacaaacacaaattttaaaagtt
caaaatttcgggtttattacagagacagcagagatcccatttggaaaggaccagccaaactgctgtggaaagga
gaaggggcagtggtaatccaagataacggggatataaaagtagtcccacgtaggaaagcaaaaataattagagat
ttagtaaaacatcatatgcatgtgtcaaaaaaggcaaaaggatggtattatagacatcattatgaatcaaggcat
ccaaaaacaagttcagaagtacatatcccagtaggtcaggcaacattagtgatagtcacttattggggattaaca
acaggagaacagccttggcatctaggacatggagtatccatagaatggagactaagaaaatacaagacacaggtt
gatcctgaaatggcagacaagctaatacatctttattattttgattgttttacagattctgccataaggcaagcg
gtcttagggagacaagtatttcctaggtgtgaatatcctgcagggcacaaccaggtagataccttacaatatcta
gcattaacagcttgggtgggaataaagaagagaaagccacccttacctagtgtggccaagttaacagaagattta
ttagaagacttaaaaaatgaagctgtgcgccatttccaaggatttggctacatgggttagggcaacacatctat
gacacttatggagacacctgggagggagtagaggcaattatcaggatgytacaacaattactgtttatccattat
aggattggttgccagcacagcagaatagggattactcctcaaaggagaagattagagccctggaatcatccagga
agtcaacccaaaacagcttgcaataagtgctattgtaaaaattgctgctatcattgtgtggtttgcttcacaaag
aaaggcttaggcatctcacaacattgggtaacagtgtactatggggtgccagtatggagagacacagagacagtt
cttttctgtgcttcagatgctaaagcccatagtacagaggctcacaacatctgggccacacaagcatgtgttccc
actgatcccaatccacaagaagtgccattaatcaatgtgactgaacattttgatatgtggaaaaataacatggca
gaacaaatgcaagaggacattatcagtttatgggaacagagcttaaagccctgtgttmaattaaccccattatgt
gtaactatgcattgtwacaatagcaatgggkttaatgrgacagttgacaatggggaattagatataggatacaaa
caaatgaaaaattgttcattcaatgtaaccactgagagaaaagataaaaagaagctagcttactctctgttttat
gcagaagatgtagtgcaactcaatgagagtgacagtacgaatcaaacatataggctaataagttgtaaaaccaca
tctgtaacacaagcctgtcctaagaccacctttgagccaattccaatacattactgtgcaccaccaggctttgcc
attatgaratgtaatgaaggaaactttagtggaaaaggggaatgtaaaaatgtgagtactgtacaatgcacacat
ggaataaagccaacgatatctactcagttaatcmtaaatgggagcctagatacagatgatattgttmttagayrt
sataawgataatatgttggtgcaatggaatmagacagtgtcaataaattgtacaaggccagggaataatayagga
ggacaggtgcagataggacctgctatgacattttataacatagagaaaataataggggacattagacaagcacac
tgtaatgtctctgaagaatggaaatcaatgtgggatagaacaaaagagaaaataaagggcctcctgggaaacaac
acaacctttaagtctcgggtgaacattggaggagacccagaagtaagacacttcatgttcaattgtggaggagag
tttttcctttgtaacacttccagattatttgatgagaacgggactgtaaatgggactattattctgccctgtaga

ataaaacaaattgtaaatttgtggacaagagtaggaaaaggaatttatgcaccaccaattcggggaaatattacc
tgtaactccagtattactggactaattctagaagttagtggaaatagtacagtatatccttcaggaggaaacatg
gttaatctctggagacaagagttgtacaagtacaaagtagttagcatagaacccataggagtagcaccaggtaaa
gctaaaagacgcacagtgaatagagaaaagagagcaagtctatggggctgctcaggaaaaactatatgctatacc
actgtgccttggaatgagacttggagcaatcatacctcctatgattcaatctggggaaatttaacctggcaacaa
tgggatgagaaagtaagaaactattcaggtgtcattttttgaccttatagagcaggcacaagagcagcagaacaca
aatgagaaatcactcttggaattggatcaatggacaagtctgtggaactggtttgatatttcaaactggctgtgg
tacataaaaatagctataatggtagtagcaggcattataggcataagaatcataagtgtaataataactataata
gcaagagttaggcagggatattctcccctctcgttgcagctcagaaaacgcttgtggggaatactagcatattgg
ggaaaagagttaaaagatagtgctatcagcttgcttaatacaacagctattgcagtagcaggggg racagatagg
cttatagaattagcacaaagaataggaaggggaatattacacatacctagaagaatcagacagggcctagaaaga
acacttttataaatgggaagagttggtcaaagagcagtgtagtaggatggccagaaatcagagaaagaatgaga
agacaaagacaaactcaagcagcagcagcagcagtaggagtaggagcagcttctcaagacctagctaatcgaggg
gccatcaccacaagcaatactagagataataatgaaactgtagcttgggtagaagcacaagaagaagaagaggaa
gtaggctttccagtacgccctcaagtaccattgaggccaatgacctttaaagcagcttttgatctttccttcttt
ttaaaagaaaagggggggactggaagggctagtttggtccagaaaaaggcaagagattctagacctctggatttat
aacacacaaggctacttccctgactggcagaactacacaccagggccaggagtcaggtatcccctgacatttgga
tggtgcttcaaactagtaccattgtcagctgaagcagtagaagaagctaatgaaggagacaacaatgccctctta
catcccatatgtcaacatggagtagatgatgatcacaaagaagtgctggtgtggcggtttgacagctccctggca
agaagacacatagcaagagagctgcatccggacttctacaagaactgctga
>#74 AY532635 7434 nt
atgggtgcgagagcgtcagtgttaacaggggaaaattagatcaatgggaagcaatttatttgagaccaggggga
aagaaaaaatacagaatgaaacatttagtatgggcaagcagggagctggaaagattcgcttgtaacccaggtctc
atggacacagcgagcggctgtgcccagttactaaaccaattagaaccagctctcgcaacagggtcagaaggactg
cgctccttatataacgccctagcagttctttattgtgtccatagtaacatacaggtacacaatacacaagaggcg
ttggacaagataaaagagataaaagagaaaaaggaacggcacaagtccgagccaaaaaacccagaagcaggggca
gcggcagcagctgataacaatatcagtaggaattatcctctagtccagaatgctcaaggacaaatgatacatcag
ccgctgacacccagaactttaaatgcttgggtaaaagtgatagaggagaaggcctttaatccagaagtaatacca
atgtttatggccttgtcagaaggggcaacacccgcagatctaaatactatgttaaatacagtaggaggacatcag
gcagctatgcagatgctaaaggaagtcatcaatgaggaagcagcagaatgggacaggacgcatccartccctgyg
ggaccattaccccaggacaactgagagaacctagaggaagtgatatagcagggacaactagtactctgmtggaa
caggtgacctggatgacttctaatcctcctgttccagtaggagaaatctataaaaaatggatagttctggggtta
aatagaattgtgagaatgtatagtcctgtcagcattctagagatcaaacaaggaccaaaagaaccctttagagat
tatgtagacaggttctacaaaactttaagagcagagcaggcatcacaggatgtaaagaattggatgacagaaaca
ctcttagtacaaaatgcaaatccagattgtaaacagatactaaaagcattagggccagcagctaccttagaagaa
atgatgacggcctgtcagggagtgggggggaccagcgcataaggcaagagtgctagcagaggctatgtcacaggta
cagacagcaactagtgtctttgcacaagggagaaactttaaaggcataaggagaaccattagatgtttcaattgt
ggcaaagagggccatttggcaagaaactgtaaggcccctagaagagggggctgttggaaatgtggcaagaagga
catcaaatgaaagattgtaaaaatgaaggaagacaggctataggaaaagaagtaagagaagctcttctagataca
ggagctgatgatacagtgatagaagagatacaattagaaggaaaatggaagccaaaaatgataggaggaattgga
ggatttatcaaagtaagacaatatgataatgtaacaatagacatacaaggaaggaaagcagttggtacagtgtta
gtaggaccaacacctgttaatattataggaagaaactttttaacacagattggctgtactttaaattttccaata
agtcctattgaaactgtaccagtaaaattaaaaccagggatggatggcccaagagtaaaacaatggcctttgaca
gcagaaaaaatagaggcattaagacaaatttgtgcagaaatggaacaggaaggaaaaatttctagaatagggcct
gaaaatccatacaacactccaatctttgctataaaaaagaaagatagcactaaatggagaaaattagtagatttc
agggaattaaataaaaggacccaagattttgggaagtacagctaggaattccacatccagcaggattacagaag
aaaaaatcagtaacagttctagatgtaggagatgcttattttcatgtcccttagatgaagattttagaaagtat
acagccttcaccatacctagtacaaataatgagacacctggtattagataccagtataatgtgttgccacaggga
tggaaggggtcaccagcaattttcaaagttcaatgacaaaaattttagaaccatttagaaagaaacatccagag
ataatcatttatcagtacatggatgacctctatgtgggatctgactcagaactagcacaacatagagagacagtg
gaagaacttagaagtcatcttttgaagtggggctttacgacccctgacaaaaaacatcaaaaggagccaccgttc
ctctggatgggatatgagctccatccagataaatggacggtccagacaataaagttaccagaacaggatgtatgg
actgtcaatgatatacaaaaattagtaggaaagttaaattgggcaagtcagatctatccaggaatcaaagtaaaa
cagctttgtagattaatcagaggaaccaaagctttgacagaagtagtcaactttacaaaagaagcagaattagaa
ttagcagaaaacagggagatattaagagaacccctacatggagtctactatgacccaggaaaagaactaatagca

gaaattcaaaaacaaggacaagatcagtggacatatcagatttatcaggagccatataaaaatttaaaaacagga
aaatatgcaaaaaggagatctgcccatactaatgatataaaacagttagttgaagtggtacaaaaagtagcaaca
gaaggtatagtaatttggggaaagattcctaagtttaaattaccagtacaaaaggaagtgtgggaggcatggtgg
actgacaattggcaagcaacctggattcctgagtgggaattcgtcaacactcctccccttgtaaaattatggtat
cagttagaaacagagccaatcactggggcagaaacatactatgtagatggagcagctaataaagaaacaaaattg
ggaaaagcaggttttgtgacagatagaggaaaacagaaagtggtctctattgaagacaccaccaatcaaaagact
gagttacaagctatccttctggccttacaagattcaggacaggaagtaaacatagtcactgattctcagtatgct
ctgggaataattcactcacacccagataaaagtgaatcagaattggtgagccaaataatagaagagctcataaaa
aaggaaagagtttatctctcttgggtacctgcacataaaggtattggaggaaatgagcaggtagacaaattagtt
agctcaggaattagaaaagtattattcctagatggtatagaaaaagcccaagaagatcatgaragatatcacagt
aactggagagcaatggccagtgattttaacttaccctctgtagtggcaaaagaaatagtagccagctgtgacaaa
tgccagctaaaaggggaagccatacatggacaggtcaattgtagtccaggagtgtggcagctagattgtacacat
ttagaaggaaaaatcattcttgtagcggtccatgtggccagtggctacttagaagcagaagttattcctgcagaa
acaggacaggaaacagcatattttattttaaaattagctggaagatggccagtaaaagttatacacactgataat
ggacccaatttcactagtgccactgtaaaagcagcctgttggtgggcaaatatcacacaggaattcgggataccc
tacaatcctcaaagtcagggagtagtagaatccatgaataaagaattaaagaaaattataggacaaatcagagat
caagcagaacatctaaaaacagcagtgcaaatggcggtgttcattcacaattttaaaagaaaaggggggattggg
gagtacactgcaggggaaagaataatagacataatagcaacagacatacaaacaacaaaattacaaacacaaatt
ttaaagattcaaaattttcgggtttattacagagacagcagagatcccatttggaaaggaccagccaaacttctg
tggaaaggagaaggggcagtggtaatccaagataacggagatataaaagtagtcccacgtaggaaagcaaaaata
attagggatttagtaaagcatcatatgtatgtgtcaagaaaggcagaagggtggtattatagacatcattatgaa
acaaatcatccaaaaataagttcagaagtacatatcccagtaggtctggcaaggttagtgataaccacttattgg
ggactaacaacaggagaaaggtcttggcatctaggacatggagtatccatagaatggaaactaggaaaatacaag
acacaagttgatcctgaaatggcagacaagctaatacatctttattattttaattgttttacagcctctgccata
aggcaagcggtcttagggagaccagtattccctaggtgtgaatatccggcagggcacaatcaggtaggcactttg
caacatctagcactcacagcctgggtgggagtaaagaagagaaagccacccttacctagtgtgactaagttaaca
gaagatttattagaagaattaaaaaatgaagctgtgcgccattttccaaggacttggctacatgggttagggcaa
cacatctataacacatatggagacacctgggaggggtagaggcaattattaggatactacaacaattactgttt
atccattataggattggctgccagcatagcagaatagggattactcctcggggagaaggttagagccctggaat
catccaggaagccaacccaagacagcttgcaatggatgctattgtaaatattgctgctatcactgtatgtgttgc
ttcacaaagaaaggcttaggcatatcaaaatattgggcaacagtgtactatggagtaccagtatggagagatgta
gaaacagttcttttctgcgcctcagatgctaaagcccatagtacagaggctcacaacatctgggccacacaagca
tgtgtccccactgatcccaatccacaagaagtaccactagacaatgtgactgaaccttttaatatgtgggaaaat
aaaatggcagaacaagtgcaagaggacattattagtttatgggaacagagcttaaagccctgtgttaaattaacc
ccattatgtgtaactatgaattgtagcaatagcaatggaaatcggacaacagatgagaaagaaaaaccaggaaat
gggacagacctagaagcaagacacatgaaaaattgctcattcaatataaccactgaaatacatgacaaaaagaag
caagcttactctctgttttatgtagaagatgtagtgccactcaatgatgggaataatagtacatacaggctaata
aattgtaataccacagctgtaacacaagcttgtcctaagactacctttgagccaattccaatacattactgtgca
ccaccaggctttgccattatgaaatgtaatgaagcaaattttaatggaacaggggaatgtaaaaatgtgagtact
gtacaatgcacacatggaataaagccagtgatatccactcagttaatcctaaatgggagcttagataaagatatt
gttattagaaataatagtggtggaaatctgttggtgcaatggaatgagatagtgacaatgaattgtacaaggcca
gggaataatacaggaggacaggtacaaataggacctgctatgacattttataacatagaaaaatagtaggagac
attagacaagcacactgtaatgtctctaacgaatggagatcaatgtggaataaaacaaaagagaaaataaagagc
ctcctgggaaacaacataacattcaaggctcaggaaaagaatggaggagacccagaagtaacacacttaatgttc
aattgtggaggagagtttatctattgtaatacttccagattattcaatgagagcatgaatacaaatgggactaat
gggactattactctgccctgtagaataagacaaattgtaaatttgtggacaagagtaggaaaaggaatttatgca
ccgccaattcggggaaatcttacctgtaactccactattactggactgattctagaacatagtggtggcagtaat
ggcacagtatatcccacaggaggaaacatggtcaatctctggagacaagagttgtataagtacaaaacagtcagt
atagaacccataggagtagcaccaggtaaagctaaaagacgcacagtgagtagagaaaaaagagcaagtctatgg
ggctgctcaggaaaaactatatgctataccactgtgccttggaatgagacttggagcaataatacctcttatgat
acwatctggggtaatttaacctggcagcaatgggataggaaagtaagaaactattcaggtgtcattttttgagctt
atagaraaggcacaagaacaacagaacacaaatgagaaatcactcttggaattggatcagtgggcaagtctgtgg
aactggtttagtatcacaaactggctgtggtacatcaagatagctataatggtagtagcaggcattataggcata
agaatcataagtgtaataataactataatagcaagagttaggcagggatattctcccctctcgttgcagctcaga
acacgcttgtggggaataatagcatattggggaaaagagttaaaggatagtgctatcagcttgcttaacacaata

gctattgtagtagcagagggaacagacaggcttatagaattagcacaaagaataggaaggggaatattacacata
cctagaagaatcagacagggcctagaaagagcacttctataaatgggaaaaatttggtcaaagagcagcatagta
ggatggccagaaatcagagaaagaatgagaagacaacgacctcacgagccagcagtagagccagcagtaggagta
ggagcagcttctcaagatctagccaatcgaggggccctcaccacaagcaatactagaaccaataatccaactgta
gcttgggtagaagcacaagaagaagaaggggaagtaggctttccagtacgccctcaggtaccattgaggccaatg
acctataaagcggctgttgatctttccttcttttaaaagaaaaggggggactggaagggctaatttggtccaga
aaaaggcaagacgttttagatctctgggtttatcacacacaaggcttcttccctgactggcagaactacacacca
gggccaggaactagattcccccctgacctttggatggtgtttcaaactagtaccattgtcagctgaagcagtagaa
gaggctaatgaaggagacaacaatgccctcttacatcccctatgccaacatggagtagatgatggacacaaagaa
gtgctgcagtggcagtttgacagctccctggcaagaagacacatagcaagagagctgcatccggactttacaag
aactgctga
>#75 AJ006022 7428 nt
atgggtgcgagagcgtcagtgttaacaggggaaaattagatcaatgggaatcaatttatttgagaccaggggga
aagaaaaaatacagaatgaaacatttagtatgggcaagcagggagctggaaagattcgcttgtaacccaggtctc
atggacacagcggacggctgtgccaagttactaaatcaattagaaccagctctcaagacagggtcagaagaactg
cgctctttatataacgctctagcagttctttattgtgtccatagtaggatacagatacacaacacacaggaagct
ttggacaagataaaagagaaacaggaacagcacaagcccgagccaaaaaacccagaagcaggggcagcggcagca
actgatagcaatatcagtaggaattatcctctagtccagactgctcaaggacaaatggtacatcagccgctgaca
cccagaaccttaaatgcttgggtgaaagtgatagaggagaaggcctttagtccagaagtaataccaatgtttatg
gccttgtcagaaggggcaacgccctcagatctaaatactatgttaaatacagtaggggggacatcaggcagcaatg
cagatgctgaaggaagtcatcaatgaggaagcagcagactgggataggacacatccagtccctgtgggaccacta
cccccagggcaactgagagaccctagaggaagtgatatagcaggaacaactagcaccctggcagaacaggtggct
tggatgactgctaatcctcctgttccagtaggagatatttatagaagatggatagtcctggggttaaacagaatt
gtgagaatgtatagtcctgtcagcattctagagatcaaacaaggaccaaaagaacccttcagagactatgtagac
aggttctacaaaactctaagagcagagcaggcaacacaggaagtaaagaattggatgacagaaacactcttagta
caaaatgcaaacccagattgtaaacagctcctaaaagcattagggccaggagctaccttagaagagatgatgacg
gcctgccagggagtgggggggaccagcacataaggcaagagtgctagcagaggctatgtcacaggtgcagcagcca
acaactagtgtctttgcacaaaggggaaactttaaaggcataaggaaacccattaaatgtttcaattgtggcaaa
gagggccatttggcaagaaactgtaaggcccctagaagaggaggctgttggaagtgtgggcaagaaggacatcaa
atgaaagattgtaaaaatgaaggaagaatagggaaagaagtaagagaagctcttttagatacaggagctgatgat
acagtaatagaagagctacaattagagggaaaatggaaaccaaaaatgataggaggaattggaggatttatcaaa
gtgagacaatatgataatataacagtagacatacagggaagaaaagcagttggtacagtattagtaggaccaaca
cctgttaatattataggaagaaatcttttaacccagattggctgtactttaaattttccaataagtcctattgaa
actgtaccagtaaaattaaaaccaggaatggatggcccaaaggtaaaacaatggcctttgacaacagaaaaaata
gaggcattaagagaaatttgtacagaaatggaaaaggaaggaaaaatttctagaatagggcctgagaatccatat
aacactccaatttttgctataaaaaagaaagatagcactaaatggagaaaattagtagatttcagggaattaaat
aaaaggacccaagattttgggaagtgcagctaggaattccacatccagcaggattaaagcagaaaaaatcagtg
acagttttggatgtaggagatgcttatttttcatgtcccttggacaaagattttagaaagtatacagcttttacc
atacctagtataaacaatgagacacctggtattagataccagtataatgtgctgccacaaggctggaaaggtca
ccagcaatttttcagagtacaatgacaaaaattctagaaccattcagagagaaacatccagagataatcatttac
cagtacatggatgacctctatgtgggatctgacttagaactagcacaacatagagaggcagtagaagaccttaga
gatcatcttttgaagtgggggctttacgacccctgacaaaaaacatcagaaggaaccccccgttcctctggatggga
tatgaactccatccagacaaatggacagtccagccaataaagttaccagaaaaggatgtatggactgtcaatgat
atacagaaattagtaggaaagttaaattgggcaagtcagatctatccaggaatcagagtaaaacagctctgtaaa
ttaatcagaggaaccaaagctttgacagaagtagtcaactttacagaagaagcagaattagaactagcagaaaac
agggagatattaaaagaacccctgcatggagtctattatgacccaggaaaagaattagtagcagaaattcaaaag
caaggacaaggtcagtggacatatcagatttatcaggagttacataaaaatttaaaaacaggaaagtatgcaaaa
atgagatctgcccatactaatgatataaaacagttagttgaagtggtaaggaaagtggcaacagaaagtatagta
atttggggaaagactcctaaatttagattaccagtacaaaaggaagtgtgggaggcatggtggaccgatcattgg
caagcaacttggattcctgagtgggaatttgtcaacactcctccccttgtaaaattatggtatcagttagaaaca
gagccaatcagtggggcagaaactttctatgtagatggagcagctaatagggaaacaaaattgggaaaagcaggt
tttgtgacagatagggggaagacagaaagtggtctctattgcagacaccaccaatcaaaaggctgagttacaagct
atccttatggccttacaagagtcaggacgggatgtaaacatagtcactgactctcagtatgctatggaataatt
cattcacagccagataaaagtgaatcagaattggtgagccaaataatagaagagctcataaaaaaggaaagagtt
tatctctcttgggtacctgcacataaaggtattggaggaaatgagcaggtagacaaattagttagctcaggaatt

agaaaaatattattcctagatggtatagaaaaagcccaagaagatcatgacagatatcacagcaattggaaagca
atggccagtgatttaacttacccccatagtggcaaaagaaatagtagccagctgtgacaaatgccagctaaaa
ggggaagccatgcatggacaggtcaattgtagtccaggagtgtggcaattagattgtacacacttagagggaaaa
atcatccttgtggcggtccatgtggccagtggctacttagaagcagaagttattcctgcagagacaggacaggaa
acagcatatttatttaaagttagctggaagatggccagtaaaagttatacacactgataatggatccaatttc
actagtgccactgtaaaagcagcctgttggtgggcaaatatcaaacaggaatttgggataccctacaatcctcaa
agtcagggagcagtagagtccatgaataaagaattaaagaaaattataggacaaatcagagatcaagcagaacat
ctaaagacagcagtgcaaatggcggttttcattcacaattttaaaagaaaaggggggattggggggtacactgca
ggggaaagaataatagacataatagcaacagacatacagacaacaaatttacaaacacaaattttaaaagttcaa
aattttcgggtttattacagagacagcagagatcccatttggaaaggaccagccaaacttctgtggaaaggagaa
ggggcagtggtaattcaagataacggggatataaaagtagtcccacgtaggaaagcaaaaataattagggattta
gtaaaacatcatatgtatgtgtcaaaaaaggcaaaaggatggtattatagacatcattatgaaacacatcaccca
aaaataagttcagaagtacatatcccagtaggtcaggcaagattagtgacagtcacttattggggggctaacaaca
ggagaacagtcttggcatctaggacatggagtatccatagaatggagactaagaaaatacaagacacaagttgat
cctgaaatggcagacaagctaatacatcttcattattttgattgttttacagcctctgccataaggcaagcggtc
ttagggagaccagtattacctaggtgtgaatatccagcagggcacaaacaggtaggcaccctacaatatctagca
ctaacagcctgggtgggagcaaagaagagaaagccacccttacctagtgtgactaagctaacagaagatttatta
gaagaattaaaaaatgaagctgtgcgccattttccaaggatttggctacatgggttaggacaacacatctataac
acatatggagacacctgggaggggtagaggcaattatcaggatactacaacaattactgtttatccattatagg
attggctgccagcacagcagaatagggatcactcctcaaaggagaaggttagagccctggaatcatccaggaagc
caacctaaaacagcttgcaataattgctattgtaaaagatgttgctatcactgcttatattgcttcacaaagaaa
ggcttaggcatctcacaacattgggtaacagtgtactatggggtaccagtatggagagaagcagagacaactctt
ttctgtgcttcagatgctaaagcccatagtacagaggctcacaacatctgggccacacaagcatgtgttcctact
gatcccaatccacaagaagtgctattacccaatgtaactgaaaaatttaatatgtgggaaaataaaatggcagac
caaatgcaagaggatattatcagtctgtgggaacagagcttaaagccctgtgttaaattaaccccattatgtgta
actatgctttgtaacgatagctatggggaggaaaggaacaatacaaatatgacaacaagagaaccagacatagga
tacaaacaaatgaaaaattgctcattcaatgcaaccactgagctaacagataaaaagaagcaagtttactctctg
ttttatgtagaagatgtagtaccaatcaatgcctataataaaacatataggctaataaattgtaataccacagct
gtgacacaagcttgtcctaagacttcctttgagccaattccaatacattactgtgcaccaccaggctttgccatt
atgaaatgtaatgaaggaaactttagtggaaatggaagctgtacaaatgtgagtactgtacaatgcacacatgga
ataaagccagtgatatccactcagttaatcctaaatggaagcttaaatacagatggaattgttattagaaatgat
agtcacagtaatctgttggtgcaatggaatgagacagtgccaataaattgtacaaggccaggaaataatacagga
ggacaggtgcagataggacctgctatgacattttataacatagaaaaaatagtaggagacattagacaagcatac
tgtaatgtctctaaagaactatgggaaccaatgtggaatagaacaagagaggaaataaagaaaatcctggggaaa
aacaacataaccttcagggctcgagagaggaatgaaggagacctagaagtgacacacttaatgttcaattgtaga
ggagagtttttctattgtaacacttccaaattatttaatgaggaattacttaacgagacaggtgagcctattact
ctgccttgtagaataagacagattgtaaatttgtggacaagggtaggaaaaggaatttatgcaccaccaattcgg
ggagttcttaactgtacctccaatattactggactggttctagaatatagtggtgggcctgacaccaaggaaaca
atagtatatccctcaggaggaaacatggttaatctctggagacaagagttgtataagtacaaagtagttagcata
gaacccataggagtagcaccaggtaaagctaaaagacgcacagtgagtagagaaaaaagagcaagtctatggggc
tgctcaggaaaaacaatatgctataccactgtgccttggaatgagacttggagcaacaatacctcttatgataca
atctggaataatttaacctggcaacaatgggatgagaaagtaagaaactattcaggtgtcattttggacttata
gaacaggcacaagaacaacagaacacaaatgagaaatcactcttggaattggatcaatgggacagtctgtggagc
tggtttggtattacaaaatggctgtggtatataaaaatagctataatgatagtagcaggcattgtaggcataaga
atcataagtatagtaataactataatagcaagagttaggcagggatattctcccctttcgttgcagctcagaaca
cacttgtggggaatacttgcatattggggaaaagagttaagggatagtgctatcagcttgcttaatacaacagct
attgtagtagcagaaggaacagataggattatagaattagcacaaagaataggaaggggaatattacacatacct
agaagaatcagacaaggcctagaaagagcactgatataaatgggaaagatttggtcaaagagcagcctagtagga
tggccagaaatcagagaaagaatgagaagacaaacgcaagaaccagcagtagagccagcagtaggagcaggagca
gcttctcaagatctagctaatcgaggggccatcaccataagaaatactagagacaataatgaaagtatagcttgg
ctagaagcacaagaagaagaagaggaagtaggctttccagtacgccctcaggtaccattaaggccaataacctat
aaacaggcttttgatctttccttctttttaaaagataaggggggactggaagggctagtttggtccagaaaaagg
caagatattctagacctctggatgtatcacacacaaggcatcctccctgactggcataactacacaccaggcca
ggaattagataccccgtaacctttggatggtgcttcaaactagtaccattgtcagctgaagaagtagaagaggct
aatgaaggagacaacaatgccctcttacaccccatatgtcaacatggagcagatgatgatcataaagaagtgttg

gtgtggcgatttgacagctccctagcaagaagacatgtagcaagagagctgcatccggagttttacaagaactgc
tga
>#76 MF7672 7425 nt
atgggtgcgaaagcgtcagtgttaacaggggaaaattaaataaatgggaagcaatttatttgaaaccaggggga
aaaaaaaaatataaaaggaaacatttagtatgggcaagcagggagctggaaagattcgcttgtaacccaggtctc
agggacacagcgagtggctgtgcccagttaataaaccagttaaaaccacctctcaagacagggtcaaaaggactg
cgctctttatataacaccctggcagttctttattgtgtccatagtaacatatcggtacacaatacacaagaggct
ttggacaagataaaagagaaacaggaacagcacaagtctgagccaaagcaaccagaagcaggggcagcagcggca
gctgataacaatgtcagtaggaactatcctctaatccagaatgctcaaggacaaatggtacatcaggcgctgtca
cctagaactttaaatgcttgggtaaaagtaatagaggaaaaggcctttaatccagaagtaataccaatgtttatg
gccttgtcagaaggggcaacgccctcagatctaaattctatgttaaatacagtaggggacatcaggcggctatg
cagatgctaaaggaagtcatcaatgaggaagcagcagaatgggacaggacgcatccagcccctgtggggccacta
cccccaggacaattgagagaccctagaggaagtgatatagcagggacaactagtactctggcagaacaggtggca
tggatgactgctaaccctcctattccagtaggagatatttatagaagatggatagttctggggttaaacagaatt
gtgagaatgtatagtcctgtcagcattctagaratcaagcaaggaccaaaagaaccctttagagattatgtagac
agattctacaaaactttaagagcagagcaggcaacacaggatgtaaagaattggatgacagaaacactcttagtr
caaaatgcaaayccagattgtaaacagatyctaaaagcattagggccaggagctaccttagaagagatgatgacg
gcctgccagggagtgggggaccagcacacaaggcaagggtcctagcagaggctatgtcacaggcacaggcagca
actagtgtctttgcacaaggaggaaactttaaaggcacaagaaaaaccattaaatgttttaattgtggcaaagag
ggccatttggcaagaaactgtaaggcccctagaagaggagggtgttggaagtgtgggaaagaaggacatcaaatg
aaagattgcaaaagtgaaggaagacaggctataggaaaggaagtaagagaagctcttttagatacaggagctgat
gacacagtaatagaagaaatacaattagaaggaaaatggaagccaaaaatgataggaggaattggaggatttatc
aaagtaagacaatatgataatataacaatagacatacaaggaaggaaagcagttggtacagtattagtaggacca
acacctgtcaatattataggaagaaactttttaacacagattggctgtactttaaattttccaatcagttctatt
gaaactgtaccagtaaaattaaaaccaggaatggatggcccaagggtaaaacaatggccattgacagaagaaaaa
atagaggcattaagagaaatttgcacagagatggaaaaggaaggaaaaatttctagaatagggcctgaaaatcca
tataacaccccaatttttgctataaaaaagaaagatagcactaaatggagaaaattagtagatttcagagagtta
aataaaaggactcaagattttgggaagtgcagctaggaattccacatccagcaggattaaagcagaaaaaatca
gtgacagttttagatgtaggagatgcttatttttcatgtcccttagataaagagtttagaaagtatacagctttt
accatacctagtgtgaataatgagacacctggtattagataccagtataatgtgttgccacagggatggaaggga
tcaccagcaatttttcaaagttcaatgacaaaaattttagaaccattcaggaagagacacccagagatagtcctt
tatcartacatggatgacctctatgtgggatcagacttagaaatagcacaacatagagagacagtagaagaactt
agaggtcatcttttgaagtggggctttacaaccccctgacaagaaacatcaaaaggagcctccattcctctggatg
ggatatgagctccatccagataaatggacggtccagccaataaagttaccagaaaaggaggtatggactgtcaat
gatatacagaaattagtaggaaaattaaattgggcaagtcagatctatccaggaatcaaagtaaaacatctttgt
agattaatcagaggagccaaggctttgacagaagtagtcacttttacacaagaagcagaattagaactagcagaa
aacagggagatattaaaagaaaccctgcatggagtctactatgacccagaaaaagaattaacagcagaaattcaa
aaacaaggacaaggtcagtggacttatcagatttatcaggagcaatataaaaatttaaaaacaggaaagtatgca
aaaatgaaatctgctcatactaatgatataaaacagttagctgcagtggtacaaaaagtggcgacagaaagcata
gtaatttggggaaaaattcctaagtttaaattaccagtacaaaaggaagtgtgggaggcatggtggactgataat
tggcaagcaacttggattcctgagtgggaatttgtcaacacccctcccctwgtaaaattatggtatcagttagag
acagagccaattattgggtagaaacatactatgtagatggagcagctaatagagaaacaaaattggggaaagca
ggttttgtgacagacagaggaagacagaaagtggtctctattgaaaacaccaccaatcagaaggctgagttacat
gctatccttatggctttacaagagtcaggacaggaagtaaacatagtcactgactctcagtatgctatgggaata
attcactcacaaccagatacaagtgaatcagacttggtgggtcaaataatagaagaactcataagaaaggaaaga
gtctatctctcttgggtacctgcacataaaggcattggaggaaatgagcaggtagataaattagttagctcagga
attagaaaagtattattcctagatggtatagaaaaagcccaagaagagcatgaaagatatcacagtaattggaaa
gcaatggccagtgattttaacttacccccctgtagtagcaaaagaaatagtagccagttgtgacaaatgccaaata
aaaggggaagccatgcatggacaggtcaattgtagtccaggagtgtggcaattagattgtacccacttagaagga
aaaatcattctggtagcggtccatgtggccagtggctacttagaagcagaagttattcctgcagaaacaggacag
gaaacagcatattttattttaaagttagctggaagaaggccagtaaaagtcatacacactgataatggaaccaat
ttcactagtaacactgtaaaagcagcctgttggtgggcaagtatcaaacaggaattcgggataccctacaatcct
caaagtcagggagcagtagaatccatgaataaagaattaaagaagattataggacaaatcagagatcaagcagaa
catctaaaaacagcagtgcaaatggcagtcttcattcacaattttaaaagaaaaggggggattgggggtacact
gcaggggaaagaataatagacataatagcaacagacatacaaacaacaaaattacaaacacaaattttaaaagtt

caaaattttcgggtttattacagagacagcagagatcccatttggaaaggaccagccaagctactgtggaaagga
gaaggggcagtggtaatccaagataacggggatataaaagtagtcccacgtaggaaagcaaaaataattagggat
ttagtgaaacatcatatgtatgtgtcaaaaaaggcaaaagggtggttctatagacatcattatgaaacaaatcat
ccaaaaataagttcagaagtacatatcccaatgggtgaggcgaaattagtgataatcacttattgggggctaaca
acaggagaacagtcttggcatctaggacatggagtatccatagaatggagacaaggaaaatacaggacacaagtt
gatcctgaaatggcagacaagctaatacattgttattattttaattgttttacagcctctgccataaggcaagcg
gtcttagggagaccagtattacctaggtgtgaatatccagcagggcaccaacaggtaggcactctacaatatcta
gcactaaaagcctgggtgggaataaaaaagagaaagccacccttacctagtgtttctaagctaacagaagactta
ctagaagaattaaaaaatgaagctgtgcgccattttccaaggacttggttacatgggttaggagaatacatctat
cacacatatggagacacctgggaggggtagaggcaattatcaggatactacaacaattactgttcatccattat
aggattggctgtcagcacagcagaatagggattactgttcaaggaagaaggttagagccctggaagcatccagga
agccaacccaagacagcttgcaataattgctattgtaaaaaatgctgctatcactgtgtgtgttgcttcacaaag
aaaggcttaggcatctcaaattattgggtaacagtgtactatggagtaccagtatggaaggaagcacataccgtt
cttttctgcgcctcagatgctaaagcccatagtgcagaggctcacaacatctgggccacacaagcatgtgtcccc
actgatcccaatccacaagaagtgctattaactaatgtgactgaatattttaatatgtggaaaaataaaatggca
cagcaaatgcaagaagacattatcaacttatgggaacaaagcttaaagccctgtgtttcattaactccattatgt
gtaactatgctttgtaacgatagcaatggatggaatgagacagtaaaccakactgcaagcccggacwtgaaacaa
atgagaaattgctcatataatgtcaccaatgagataagagataaaaagaagcaagtttactctctgttttatgta
gatgatgtagtaccactcaatgacagtgtgacaacatataggctaataaattgcaataccacagctgtgacacaa
gcttgtcctaagacctcctttgagccaattccaatacattactgtgcaccaccaggctttgccattatgaaatgt
aatgaaggaaattttagtggaaatggaacttgtaaaaatgtgagtactgtacaatgcacacatggaataacacca
ataatatccactcagttaatcctaaatggaagcttagatacaaaggatatagttattagacatcatagagataga
ggagntagaggtgatctgttggtgcaatggaatgagacagtgtcaatgaactgtacaaggccaggaaataattca
gtaggacaggtgcagataggacctgctatgacattttacaacatagagaaaatagtaggagacattagacaagca
cactgtaatgtctctggaaactgggcatcaatgtggaacaaaacaaaagaaaaaatacagaacctactgggaaaa
aayacaacakaattcraggttcggggggaaaaatggaggagacccagaagtaacacacttaatgttcaattgtaga
ggagagtttttcctctgcaacactactaacttatttaatgacagcaagaatgcaactaatgggggtattactctg
ccctgtagaataaaacaatttgtgaatttgtggacaagagtaggaaaaggaatttacgcaccaccaattcgggga
gagctttcctgtaacttcaatattactggactaatgctagaacatagtggtaacaccaacagtacaatattccta
ctatatccctcaggaggaaacatggttaatctctggagacaagagctgtataagtacaaagtagtcagtatagag
cccataggagtagcaccaggtaaagccaaaagacacacagtgggtagagaaaaaagagcaagtctatgggctgc
tcaggaaaacttatatgctacaccactgtgccttggaatagcacctggagcaataatatctcttatgatgrcatc
tggaataatttaacctggcaacaatgggatgagaaagtaaaaaactattcaggtgtcattttcgrtcttatagag
caggcacaagaacaacaaaacacaaatgagaaatcactcttggaattggatcaatgggcaagtctgtggaactgg
tttgatattacaaactggctgtggtacataaaaatagctataatggtagtagcaggcattataggcataagaatc
ataagtgtagtaataactataatagcaagagttaggcagggatattctcccctctcgttgcagctcaggatacgc
ttgtggggaataatagcatattggggagcagagttaaaagagagtgccattagcttgtttaatacaacagctatt
gtagtagcagagggaacagataggattatagaattagcacaaagaataggaaggggaatcttacacatacctaga
agaatcagacagggcctagaaagagcacttttataaatggggaagagttggtcaaagagcagaataccaggatgg
ccagaaatcagagaaagaatgagraaacaaacgcaagaatcagcagtagagccagcagtaggagtaggagcagcc
tctcaagacctagctaatcgaggagccctcaccaccagcaatactaaagataataatcaaactgtagcttgggta
gaagcacaagaagaagaggaggaggtaggctttccagtacgccctcaagtaccggtgaggcctatgacctataaa
gcagcctttgacctctccttctttttaaaagaaaaggggggactggaagggctagtttggtccagaaaaaggcaa
gagattctggacctctgggtttatcacacacaaggcttcttccctgactggcagaactacacaccagggccagga
atcagatacccnctgacctttggatggtgcttcaaactagtaccattgtcagatgaagcagtagaagaagccact
gaaggagacaacaatgccctcctgcatcccatatgtcaacatggagtagatgatgatcacaaacaagtgctggtg
tggaggtttgacagcgccctggcaagaagacacatagcaaaagagctgcatccggacttttacaagaactgctga
>#77 KY498771 7671 nt
atgggtgcgagagcgtcagtgttaacaggggggaaaattagatcaatgggaatcaacttacttgagaccagggga
aagaaaaaataccaaatgaaacatttagtatgggcaagcagggagctggacagattcgcttgtaacccaggtctc
atggactcagcggaaggctgtgcccagttaatgaaccaattagaaccagctctcaagacagggtcagaaggactg
cgctctttatataacaccctagcagttctttattgtgtccatcgtagaatatcagtacaaaatacacaagaagct
ttggacaaagtacatggggaaaaggaacagcacaacaagccaaaaaactcagaagcaggggcagcggcagcagtt
gacagcagtatcagtagaaattatcctctagtccagactgctcaaggacaaatgatacatcagccgctgacaccc
agaaccttaaatgcttgggtaaaagtgatagaggagaaggcctttagtccagaagtaataccaatgtttatggcc

ttgtcagaaggggcaacacccggagatctaaatactatgttaaatacagtaggaggacatcaggcagctatgcag
atgctgaaggaagtcatcaatgaggaagcagcagaatgggacaggctgcatccagtccctgtgggaccactgccc
ccaggacaactgagagaccctagaggaagtgatatagcaggaacaactagtactctgcaagaacaggtggcatgg
atgactgctaatcctcctgtcccagtaggagatatttatagaagatggatagtcctggggttaaacagaattgtg
agaatgtatagtcctgtcagcattctagagatcaaacaaggaccaaaagaaccctttagagactatgtagacagg
ttctacaaaactttaagagcagagcaggcaacacaggaagtaaagcagtggatgacagaaacactcttagtacaa
aatgcaaacccagattgtaaacagctcctaaaagcattagggccaggagctaccttagaagagatgatgacrgcc
tgccagggagtgggggaccagggcataaggcaagagtactagcagaggctatgtcacagatacagccaacaact
agtatctttgcacaaagaggaaactttaaaggcataaggagaaccattaaatgtttcaattgtggcaaagagggc
catttggcaagaaactgtaaggcccctagaagaaagggctgttggaaatgtgggaaagaaggacatcaaatgaaa
gattgcaacaatgaaggagacaggctatagggaaagaagtaagggaagctcttttagatacaggagctgatgat
acagtaatagaagaactacaattagagggaaaatggaaaccaaaaatgataggggaattggaggatttatcaaa
gtaagacaatatgataatgtaacagtagatatacagggaagaaaagcagttggtacagtattagtaggaccaaca
cctgttaatattataggaagaaatcttttaacacagattggctgtactttaaattttccaataagtcctattgaa
actgtaccagtaaaattaaaaccaggaatggatggcccaaaggtaaaacaatggcctttgacagcagaaaaaata
gaggcattgagagaaatttgtacagaaatggaaaaggarggaaaaatttctaaaatagggcctgagaatccatat
aacactccaatttttgctataaaaaagaaagatggcactaaatggagaaaattagtagatttcagagaattaaat
aaaaggacccaagattttgggaagtgcagttaggaattccacatccagcaggattaaagcagaaaaaatcagtg
acagttttggatgtaggagatgcttatttttcatgtcctttggatgaagatttcaggaagtatacagcttttacc
atacctagtataaacaatgagacacctggtattagataccagtataatgtgttgccacagggctggaaagggtca
ccagcaattttcaaagttcaatgacaaagattctagacccatttagaaagaaacatccagagataatcatttat
cagtacatggatgacctctatgtgggatctgacttagaactagcacaacatagagaggtagtagaagaacttaga
aggcatcttttggattggggctttacgaccccctgacaaaaaacatcaaaaggagccaccgtttctctggatgga
tatgagctccatccagataaatggacggtccagccaataaagttgccagaaaaagagatatggactgtcaatgat
atacaaaaattagtagggaaattaaattgggcaagtcagatctatccaggaatcaaagtaaaacagctgtgtaga
ttaatcagaggaaccaaagctttgacagaagtagttgactttacaaaagaagcagaattagaactagcagaaaac
agggagatattaaaagaaaccatgcatggagtctactatgacccaggaaaagaattaatagcagacattcagaaa
caaggacaaggtcaatggacatatcagatctatcaagagccatataaaaacctgaaaacagggaaatatgcaaaa
atgagatctgcccatactaatgatataaaacagttagttgaagtagtacagaaggtggcaacagaaagtatagta
atttggggaaggactcctaaatttagattaccagtacaaaaggaagtgtgggagacatggtggactgatcattgg
caagcaacttggattcctgagtgggaatttgtcaatacyctccccttgtaaaattatggtatcagttagaaaca
gagccaatcagtggggtagaaacatactatgtggatggagcagctaatagagaaacaaaattgggaaaagcaggt
tttgtgacagatagaggaagacagaaagtggtttctcttgcagacaccaccaatcaaaaggctgaattacaagct
atccttatggccttacaagagtcaggacaggcagtaaacatagttactgactcccagtatgctatgggaataata
catgcacaaccagataaragtgaatcagaattggtaagccaaataatagaagaactcataaaaaaggagaaagtt
tatctctcatgggtacctgcacataaaggtattggaggaaatgagcagatagacaaattagtcagctcaggaatt
aggaaagtattattcttggatggcatagacaaagcccaagaggatcatgaaagatatcacagtaattggagagca
atggctagtgattttaacctacccccgtagtggcaaaagaaatagtagccagctgtgacaaatgccagctaaaa
ggggaagccatacatggacaggtcaattgtagtccaggagtatggcagttagactgtacacacttagaaggaaa
atcattcttgtagcagtccatgtggccagtggctacttagaagcagaagttattcctgcagaaactggacaggaa
acagcatattttattttaaagttagctggaagatggccagtaaaagttatacacactgataatggacccaatttc
actagtgccactgtaaaagcagcttgttggtgggcaaacatcaaacaggaatttgggataccctacaatcctcaa
agtcagggagcagtagagtccatgaataaagaattaaagaaaattataggacaaatcagagatcaagcagaacat
ctaaaaacagcagtgcaaatggcggttttcattcacaattttaaaagaaaggggggattgggggtacactgca
ggggaaagaataatagatataatagcaacagacatacagacaacaaaattacaaacacaaattttaaaagttcaa
aattttcgggtttattacagagacagcagagatcccatttggaaaggaccagccaaacttctgtggaaaggagaa
ggggcagtggtaatccaagataacggggatataaaagtggtgccacgtaggaaagcaaaaataattagggattta
gtaaagcatcatatgtatgtgtcaaaaaaggcaaaagggtggtattatagacatcattatgaaacaactcatcca
aaaataagttcagaagtacatatcccagtaggtcaggcaagattagtgataaccacttattggggactgacaaca
ggagaacagccttggcatctaggacatggagtatccatagaatggagactaagaaaatacaggacacaagttgat
cctgaaatggcagatcagctaatacattttttattattttaattgttttacagcttctgccatcaggcaagcgatc
ttagggagaccagtatcacctaggtgtgaatatccagcagggcataaacaggtaggcaccttacaatatctagca
ctaacagcctgggtggaagtaaagaagagaaagccacccttacctagtgtgactaagttaacagaagatttatta
gaggaattaaaaaatgaagctgtgcgccaytttccaaggatttggctacatgggttaggacaatacatctacaac
acttatggagacacatgggaggggtagaggcaattattaggatgctacaacaattactgtttatccattatagg

attggctgtcagcacagcagaatagggatcactcctcaaaggagaaggatagagccctggaatcatccaggaagc
cgacccaagacagcttgcaataattgctattgtaaaaaatgttgctatcactgtgtggtttgcttcacaaagaaa
ggcttaggcatctcacaacattgggtaacagtgtactatggggtaccagtatggagagacgcagagacagttctt
ttctgcgcctcagatgctaaagcccatagtacagaggtccacaacatctgggccacacaagcatgtgttcccact
gatcccaacccacaggaagtgctattacccaatgtaactgaaaattttaatatgtggaaaaataacatggcagaa
caaatgcaagaagacatcatcagtctatgggaacagagcttaaagccctgtgttaaattaaccccattatgtgta
actatgctttgcaacaatagcaatggaaatatgtcagttgagggtaccacccagaggactacaacaagtagtaca
ggtataggaaataagctaatgaaaaattgctcatttaatgcaaccactgagagaaaagataaaaagaagacagtt
tactctctgttttatgcagaagatgtagtaaaaatctcaaatgatagtacttataggctaataagttgtaatact
acagttgtgacacaggcttgtcctaagatctcttttgaaccaattccaatacattactgtgcaccaccaggcttt
gccattataaaatgtaatgaaggaaattttagtggaaatggaacttgtaaaaatgtgagtactgtacaatgtaca
catggaataaagccagtgatatctactcagttaatcctaaatggaagtttaagcacaggtgacattgttattaga
aatagcagtcgcggtactctgttggtgcaatggaatgagacagtgcaaataaattgtacaaggccaggaaataat
acaagtggacaggtgcagataggacctgctatgacattttataacatagagaaaatagtaggagacattagacaa
gcgcactgtaatgtctcctgggaaagttggggaaaaatgtggaatagaacaaaagacaaaataaacagggaggtc
aactggaccaactggatctaccgggatagggagaagaaggaaggagatccagaagtaacccaattcatgttcaat
tgtagaggagaattttctattgtaacacttctaaattgtttaaggagagtaaatcgtctaacaggacagggagt
attactctgccctgtagaataaaacaatttgtaaacttgtggacaagggtaggaaaaggaatttatgcaccacca
gttaggggaaatcttacttgtaattccagtattactggactgrttytagaatatagtrakgyagataatggtact
aatgttacagtatatccctcaggaggaaacatgattgatctttggagacaagagttgcatagatacaaagtagtt
agtatagaacccataggagtagcaccaggtaaagctaaaagacgcacagtgagtagagaaaagagagcagtcttt
ggactgggtgcgctgtttcttgggtttcttggagcagcagggagcactatgggcgcagcgtcaataacgctgacg
gtacaggcccggacattactatctgggatagtgcaacagcagaacaatctgttgagagctatagaggcgcaacaa
catttgttgcaactctcaatttggggcattaaacagctccaagcaaaagtccttgctatagaaagataccttagg
gatcagcaaatcctaagtctatggggctgctcaggaaaagctatatgttataccactgtgccttggaatgagact
tggagcagtaatacctcctatgagacaatctggaataatttaacctggcaagaatgggataagaaagtaagaaac
tattcaggtgtcattttgaccttatagagcaggcacaagaacaacagaacacaaatgaaaaatcactcttggaa
ttggatcagtgggcaagtctgtgggactggtttagtattacaaactggctgtggtatataaaaatagctataatg
atagtagcaggcatcataggcataagaatcataagtgtaataataactataatagcaagagttaggcagggatat
tcacctcccctctcgttgcagctcagaatatacttgtggggaataatagcatattggggaaaagagttaaaagat
agtgctatcagcttgcttaatacaacagctattgtagtagcagaaggaacagataggcttatagaattagcacaa
agaataggaaggggaatattacacatccctagaagactcagacaaggcctagagagagtacttgtataaatggga
aagagttggtcaaaaagcagcatagtaggatggccagaaatcagagaaagaatgagaagacaaccagcagagcca
gcaataggagtaggagcagcttctcaagacctagctaatcgagggggcaatcaccacaagtaatactgcaagcaat
aatgaaactgtagcttggctagaagcacaagaagaagaggaagaagtaggctttccagtacgccctcaggtgcca
ttaaggccaatgaccatgaaagcggctgttgacctttccttctttttaaaagaaaggggggactggaagggcta
ccttggtcccagagaaggcaagacattctagacctctgggtttataacacacaaggcttcttccctgattggcag
aactacacaccagggccaggagttagatacccccctgacctttggatggtgcttcaaattagtgccaatgtcaact
gaagaagtagaagaagctaataaaggagaaaacaatgccctattgcatcccatatgtcaacatggagtagatgat
gctcacaaagaagtgctggtgtggcagtttgacagctccctggcaagaagacacgtagcaagagagctgcatccg
gagttctacaaggactgctga
>#78 DQ017383 7434 nt
atgggtgcgagagcgtcagtgttaacaggggaaaattagataaatgggaagcaatttatttgagaccaggggga
aagaaaaaatatagaatgaaacatttagtatgggcaagcagggagctggaaagattcgcttgtaacccaggtctc
atggacacagcgaagggctgtgcccagttactaaaccagttagaaccagctctcaatacagggtcagaaggactg
cgctccttatataacaccttagcagttctttattgtgtccatagtacaataccggtacttaatacacaagaggct
ttggacaaaataaaagagaaaaaggaacagcacaagcctgaggcaaaaaatccagaagcaggggcagcggcagca
gctgataacaacatcagtaggaattatcctctagttcagaatgctcaaggacaaatgatacatcagccgctgaca
cccagaactttaaatgcttgggtaaaagtgatagaggagaaggcctttaatccagaagtaataccaatgtttatg
gccttgtcagaaggggcaacaccctcagatctaaattctatgttaaatacagtaggaggacatcaggcagctatg
caaatgctaaaggaggtcatcaatgaggaagcagcagaatgggacaggacgcatccagtccctgtgggaccacta
cccccagggcaactgagagagcctagaggcagtgatatagcagggacaactagtactctgccagaacagataaca
tggatgacttctaaccctcctattccagtaggagatatttatagaagatggatagttttggggttaaacagaatt
gtgagaatgtatagtcctgtcagcattctagagatcgagcaaggaccaaaagaaccctttagagattatgtagac
aggttctacaaagttttaagagcagagcaggcaacacaggatgtaaagaattggatgacagaaacactcttagta

caaaatgcaaatccagattgtaaacagatcctaaaagcattagggccaggatcctccttagaagagatgatgacg
gcctgccagggagtgggggaccagggcataaggcaagagtgctagcagaagctacggcacaggcacaggcagca
accagtgtctttgtacaaaggggaaactttaaaggcacaaggagaaccattaaatgtttcaattgtggcaaagag
ggccatttggcaaggaactgtaaggcccctaggagaggaggctgttggaaatgtgggcaggaaggacatcaaatg
aaagattgtaaaaatgaaggaagacaggctataggaaatgaagtaagagaagctcttttagacacaggagctgat
gatacagtaatagaagaaatacaattagaagggagatggaaaccaaaaatgataggggaattggaggatttatc
aaagtaagacaatatgataatataacaatagacatacaaggaaggaaagcagttggtacagtgttagtaggacca
acacctgttaatattataggaagaaacttttaacacagattggctgtactctaaattttccattaagtcctatt
aaaactgtaccagtaaaattaaaaccaggaatggatggcccagaagtaaaacaatggcctttgacagcagaaaaa
atagaggcgttaagagaaatttgtacagaaatggaaaaggaaggaaaaatttctagaatagggcctgaaaatcca
tataacactccaatttttgctataaaaaagaaagatagcactaaatggagaaaattagtagatttcagagaatta
aataaaaggacccaagacttttgggaagtgcagctgggaatcccacatccagcaggattaaagcagaaaaagtca
gtaacagtcttggatgtaggagatgcttatttttcatgtcccttagatgaagattttagaaagtatacagtttt
accatacctagtgtaaacaatgagacacctggtattagataccagtataatgtgttaccacagggatgggagga
tcaccagcaatttttcaaagttcaatgacaacaattctagaaccatttagaaagaagcatccagagataatcatt
tatcagtacatggatgacctctatgtgggatctgacttagaaatagcaaaacatagagagacagtagaagaactt
agaggccatctgttgaagtggggctttacaacccctgacaaaaaacatcaaaaggagccaccattcctctggatg
ggatatgagctccatccagataaatggacagtccggccaataaagttaccagaaaaggatacatggactgtcaat
gatatacagaaattagtagggaaattaaattgggcaagtcagatttatccaggaattaaagtaagacagctttgc
aaattaatcagaggaaccaaggctttgacagaagtagttacctttacagaagaagcaggattagaactagcagaa
aacagggagatattaaaagaacccctacatggagtctattatgacccagaaaaggaattaatagcggaaattcaa
aaacaaggacaaagtcagtggacatatcagatttatcaggagtcacataaaaatttaaagacaggaaagtatgca
aaaatgagatctgcccatactaatgatataaagcagttaactgaagtagtacaaaagatagcaacagaaagtata
gtaatttggggaaagactcctaagtttaaattaccagtacaaaaggaagtgtgggaggcatggtggactgagaat
tggcaagcaacttggattcctgagtgggaattcgtcaacacccctcccccctgtaaaattatggtatcagttagaa
acagagccaattagtggggcagaaacatactatgtagatggagcagctaatagagaaacaaaattgggaaaagca
ggttttgtgacagatagaggaagacagaaagtggtctctattaaagataccaccaatcaaaaaactgagttacat
gctatcctcatggccttacaagagtcaggacaggaagtaaacatagtcactgactctcagtatgctatgggaata
attcactcacagccagataaaagtgaatcagaattggtgagccaaataatagaagaactcataaaaaaggaaaga
gtctatctctcttgggtacctgcacataaaggtattggaggaaatgagcaggtagacaaattagttagctcagga
attagaaaagtattattcctagatggtatagaaaaagcccaagaagagcatgaaagatatcacagtaactggaga
gcaatggccagtgattttaacttrcccccccatagtggcaaaagaaatagtagccagctgtgacaaatgccagcta
aaaggggaagccatgcatggacaggtcaattgtagtccaggagtgtggcagttagattgtacacacttagaagga
aaaatcattcttgtagcagtccatgtggccagtggctacttagaagcagaagttattcctgcagaaacaggacag
gaaacagcatattttattttaaagttagctggaagatggccagtaaaagttatacacactgataatggacccaat
ttcactagtgccactgtaaaagcagcctgttggtgggcaagtatcaaacaggaattcgggataccctacaatcct
caaagtcagggagcagtagagtccatgaataaagaattaaagaaaattataggacaaatcagagatcaagcagaa
catctaaaaacagcagtgcaaatggcagtcttcattcacaattttaaaagaaaaggggggattggggggtacact
gcaggggagagaataatagacataatagcaacagacatacagacaacaaaattacaaacacaaattttaaaagtt
caaaattttcgggtttattacagagacagcagagatcccatttggaaaggaccagccaaacttctgtggaaagga
gaaggggcagtggtaatccaagataacggggatataaaagtagtcccacgtaggaaagcaaaaataattagagat
ttagtaaaacatcatatgcatgtgtcaaaaaaggcaaaaggatggtattatagacatcattatgaaacaaggcat
ccaaaaataagttcagaagtacatatcccagtaggtcaggcaagattagtgatagtcacttattggggactaaca
acaggagaacagccttggcatctaggacatggagtatccatagaatggagactaagaaaatacaagacacaggtt
gatcctgaaatggcagacaagctaatacatctttattattttgattgttttacagcgtctgccataagacaagca
atcttagggagacaagtatttcctaggtgtgaatatcctgcagggcacaatcaggtaggcaccttacaatatcta
gcattaacagcttgggtgggaataaagaagagaaaaccacccttacctagtgtggctaagttaacagaagattta
ttagaagacttaaaaaatgaagctgtgcgccattttccaaggatttggctacatggattagggcaacacatctat
gacacatatggagacacatgggaaggagtagaggcaattatcaggatgctacaacaattactgtttatccattat
aggattggttgccagcacagcagaatagggattactcctcaaaggagaagattagagccctggaatcatccagga
agccaacccaaaacagcttgcaataattgctattgtaaaaaatgctgctatcattgtgtggtttgcttcacaaag
aaaggcttaggcatctcacaacattgggtaacagtgtactatggggtaccagtatggagagaaacagatacagtt
cttttctgtgcttcagatgctaaagcccatagtacagaggctcacaacatctgggccacacaagcatgtgttccc
actgatcccaacccacaagaagtacatttaatcaatgtgactgaaaaatttgatatgtggaaaaataacatggca
gaacaaatgcaagaggacattattagtttatgggaacagagcttaaaaccctgtgttaaattaaccccattatgt

gtaactatgcattgtaacgatagcaatgggtttaatgagacagttgggaatgggacattagacataggatacaaa
caaatgaaaaattgctcattcaatgtaaccactgagagagaagatagaaagaagaaagcttactctctgttttat
acagaagatgtagtgcaactcaatgacagtgacagtgacagtaatacatatactctaataaattgtaagaccaca
gttgtaagacaagcttgtcctaagaccacctttgagccaattccaatacattactgtgcaccaccaggctttgcc
attatgagatgtaatgaaggaaactttagtggaaaaggggaatgtaaaaatgtgagtactgtacaatgcacacat
ggaataaagccagtgatatccactcagttaatcatgaatgggagcctagatgagacagatattgttattagaaat
gatagtggtactatattgggtaatatattggtgcaatggaataagacagtattaataaattgtacaaggccaggg
aataatacaggaggacaggtgcagataggacctgctatgacattttataacatagagaaaataatagggggacatt
agacaagcacactgtaatgtctctgaagaagaatggaaatcaatgtggaatagaacaaaagaaaaaataaaggac
attctgggaaacaaaacaacctttgaggctcggggggaacgatggaagtgacccagaagtaacacacttaatgttc
aattgtggaggagagtttttcctttgtaacacttccagattatttaatgagagcaagaacgagactacaaatggg
actattattctgccctgtagaataaaacaaattgtaaatttatggacaagagtaggaaaaggaatttatgcacca
ccaattcggggaaatattacctgtaactccaatattactggactgattctagaatatagtaatagcacaaatggt
acagtagtatatccttcaggaggaaacatggttaatctctggagacaagagttgtataaatacaaagtagtgagt
atagaacccataggagtagcaccaggtaaagctaaaagacgcacagtgagtagagaaaagagagcargtctatgg
ggctgctcaggaaaaactatatgctataccactgtgccttggaatgagacttggagcartmatacctcytatgak
rcaatctggggwaatttaacctggcarmaatgggatragaaagtaagaaactattcaggtrytattttgamctt
mtagagcaggcacaagaacaacaraacacaaatgagaaatcactcttggaattggatcaatgggcaartctgtgg
aactggtttgatattacaagctggctgtggtacataaaaatagctataatggtagtagcaggcattataggcata
agaatcataagtgtaataataactataatagcaagagttaggcagggatattctcccctctcgttgcagctcagr
awacgcytgtggggartactagcatattggggaaaagagttaaaagatagtgctatcagcttgcttratacaaya
gctattgcagtagcagagggaacagataggmtyatagaattagcacaaagaataggaaggggaatattacacgtc
cctagaagaattagacagggcctagaaaggacacttgtataaatggggaaaatttttgtcaaagaacagagtagca
ggatggtcagaaatcagagaaagaatgagaaggcaaactcaagagccagctcaagagccagcagcagtaggagta
ggagcagcttctcaagacctagctaatcgaggggccatcaccacaagcaatactagagataataatgaaactgta
acttggctagaagcacaggaagaggaagaggaagtaggctttccagtacgccctcaagtaccaataaggccaatg
acctataaagcagcttttgatctttccttctttttaaaagaaaaggggggactggaagggctagtttggtccaga
aagaggcaagagattctagacctctgggtttatcacacacaaggcttcttccctgactggcagaactacacacca
gggccaggagttagataccccctgacctttggatggtgcttcaaactagtaccattgtcagctgaagcagtagaa
gaagccaatgaaggagacaacaatgccctcctgcaccccatatgtcaacatggagtagatgatgagcacaaagaa
gtgctggtgtggcggtttgacagctccctggcaagaagacacttagcaagagagctgcatccggacttttacaag
aactgctga

>#79 AJ271370 7404 nt

atgggtgcgagagcgtcagtgttaacaggggaaaattagatcaatgggaagcaatttatttgaggccaggggga
aagaaaaaatacagattgaaacatttagtatgggcaagcagggagctggaaagattcgcttgtaacccaggtctc
atggacacagcgaatggctgtgcccagttaataaaccaattagaaccagctctcaagacagggtcagaaggactg
cgctctttgtwtaacaccctggcagttctttattgtgtccatagtaacataccggtacacaatacacaagaggct
ttagacaagataaaagagaaacaggaacagcacaagtccgagccaaagaaaccagaagcagggacagcggcggca
gctgacagcagtatcagtaggaattatcctctagtccagaatgctcaaggacaaatggtacatcagccgctgaca
cctagaactttaaatgcttgggtaaaagtaatagaggaaaaggcctttaatccagaaataataccaatgtttatg
gccttgtcagaaggggcaacgccctcagatctaaatagtatgttaaatacagtaggaggacatcaggcagctatg
cagatgctaaaggaagtcatcaatgaggaagcggcggagtgggacaggacgcatccagcccctgtgggaccacta
cccccagggcaaatgagagaccctagaggaagtgatatagcagggacaactagtactctggcagaacaggtggca
tggatgacttctaatcctcctattccagtaggagatatttatagaagatggatagttctggggttaaacagaatt
gtgagaatgtatagtcctgtcagcattctagagatcaagcaaggaccaaaagagccctttagagattatgtagac
aggttctacaaaactttaagagcagagcaggcaacacaggatgtaaagaattggatgacagaaacactcttagta
caaaatgcaaacccagattgtaaacagatcctaaaagcattaggaccaggagctaccttagaagagatgatgacg
gcctgccagggagtaggggggaccagcacataaggcaagagtgctagcagaggctatggcacaggcacaaacagca
actagtgtctttgtacaaaggggaaactttaagggcataagaaaaaccattaaatgttttaattgtggcaaagag
ggccatttggcaagaaactgtaaggccccctagaagaaggggctgttggaagtgtgggcaagaaggacatcaaatg
aaagattgtaaaaaatgagggaagmcaggctaatataggaaaggaaataagagaagctcttttagatacaggagct
gatgatacagtaatagaagaaatacaattagaaggaaaatggaaaccaaaaatgataggaggaattggaggattt
atcaaagtaagacaatatgataatataacaatagacatacaaggaagaaaagcagttggtacagtgttagtagga
ccaacacctgttaatattataggaagaaacttttaacacagattggctgtactttaaattttccaatcagtcct
attgaaactgtaccagtaaaattaaaaccagggatggatggcccaagggtaaaacaatggcctttgacagcagaa

aaaatagaggcattaagagaaatttgtacagaaatggaaaaagaaggaaaaatttctagaatagggcctgaaaat
ccatataacactccaattttgctataaaaaagaaagatagcactaaatggagaaaattagtagatttcagggaa
ttaaataaaaggacccaagaattttgggaagtgcaactaggaattccacatccagcaggattaaagcagaaaaaa
tcagtaacagttmtagatgtaggagatgcttatttttcatgtcccttagataaagatttagaaagtatacagct
tttaccatacctagtataaataatgagacacctggtattagataccagtataatgtgttaccacagggatggaag
ggatcaccagcaattttcaaagttcaatgacaaaaattctagaaccatttaggaagaaacatccagagataatt
atttatcagtacatggatgacctctatgtgggatctgacttagaaatagcacaacatagagagacagtagaagaa
cttagaggtcatcttttgaagtggggctttacgacccctgacaaaaaacatcaaaaggagccaccgttcctctgg
atgggatatgagctccatccagataaatggacggtccagccaataaagttaccagaaaaggaggtatggactgtc
aatgatatacaaaaattagtaggaaagttaaattgggcaagtcagatctatccaggaatcaaagtaaaacagctt
tgtaaattaatcagaggaaccaaggctttgacagaagtagtcacttttacacaagaagcagaattagaactagca
gaaaacagggagatattaaaagaacccctacatggagtctattatgacccaggaaaagaattaatagcagaaatt
caaaaacaaggacaaggtcagtggacatatcagatttatcaggagccatataaaaatttaaaaacaggaaagtat
gcaaaaaygagatctgctcatactaatgatataaaagagttagctgcagtggtacaaaaggtggcaacagaaagt
atagtaatttggggaaagactcctaagtttaaattaccagtacaaaaggaagtgtgggagacatggtggactgag
cattggcaagcaacttggattcctgagtgggaattcgtcaacactcctccccttgtaaaattatggtatcagtta
gaaacagagccaattagtggggcagaaacatactatgtagatggagcagctaataaagaaacaaaattaggaaaa
gcaggttttgtgacagatagaggaagacagaaagtggtctctattgagaacaccaccaatcaaaaggctgagtta
caagctatcctttggccttacaagagtcaggacaggaagcaaacatagtcactgactctcagtatgctatggga
ataattcactcacaaccagataaaagtgaatcagacttggtgggccaaataatagaagaactcataaaaaaggaa
agagtctatctctcttgggtacctgcacataaaggtattggaggaaatgagcaggtagataawttagttagctca
ggaattagaaragtattattcctagatggtatagaaaaagcccaagaagaacatgaaagatatcacagtaattgg
aaagcaatggccagtgattttaacttaccccccatagtagcaaaagaaatagtagccagctgtgacaaatgccag
ctaaaaggggaagccatgcatgggcagatcaattgtagtccaggagtgtggcagttagattgtacacacttagaa
ggaaaaatcattcttgtagcagtccatgtggccagtggctacttagaagcagaagttattcctgcagaaacagga
caggaaacagcatattttattttaaagttagctggaagatggccagtaaaagttatacacactgataatggaccc
aattttattagtgccactgtaaaagcagcctgttggtgggcaggtatcaaacaggaattcgggataccctacaat
cctcaaagtcagggagcagtagagtccatgaataaagaattaaagaaaattataggacaaatcagggatcaagca
gaacatctaaaaacagcagtgcaaatggcggttttcattcacaattttaaaagaaaggggggattgggggtam
acggcaggggaaagaataatagacataatagcaacagacatacaaacaacaaaattacaaacacaaattttaaaa
gttcaaaattttcrggtttattacagagacagcagagatcctatttggaaaggaccagccaaacttctgtggaaa
ggagaaggggcagtggtaatccaagataacggggatataaaggtagtcccacgtaggaaagcaaaaataattaga
gatttagtaaaacaccatatgtatgtgtcaaaaaaggcaaaagggtggtattatagacatcattatgaaacaaag
catccaaagacaagttcagaagtrcatatcccagtaggtcmggcaarattagtgatagtcacttattggggacta
acaacaggagaacagccttggcatctaggacatrgagtatccatagaatggagacaaggaaaatacaagacacaa
gttgatcctgaaatggcagacaagctaatacattgttattattttaattgttttacagcttctgccataaggcaa
gcggtcttagggagaccagtgttacctaggtgtgamtatccggcagggcacaamcaggtaggcactctacaatat
ctagcamtaacagcctrggtgggagtaaagaagagaaggccacccttacctagtgtgactaagttaacagaagat
ttattagaagaattaaaaaatgaagctgtgcgccattttccaaggatttggctacatgggttaggacaacacatc
tataacacatatggagacacctgggaggggtagaggcaattatcaggatactacaacaattactgtttatccat
tataggattggctgccagcacagcagaatagggattactccccaaaggagaaggttagagccctggaatcatcca
ggaagccaacccaagacagcttgcaataagtgctattgtaaaaaatgctgctatcactgtatgtgttgcttcaca
aagaaaggcttaggcatctcaccacattgggtaacagtgtactatggagtaccagtatggagagacgcagagaca
gttcttttctgcgcttcagatgctaaagcccatagtacagaggctcacaacatctgggccacacaagcatgtgtc
cccactgatcccaatccacaagaagtgctattaaccaatgtgactgaatattttaatatgtgggaaaataaaatg
gcagaacaaatgcaagaggacattatcagtttatgggaacagagcttaaagccctgtgttaaattaaccccatta
tgtgtaactatgctttgtaacaatagcaatgggaatagtgcagggaatagtactaccaataggacagaggatcta
gaagacagacaaatgaaaaattgctcattcaatataaccactgagataagagatagaaagaagcaagtttactct
ctgttttatgtagaagatgtagtgccaatcaaagatgggactgacaataatacatataggctaataaattgtaat
accacagctgtgacacaagcttgtcctaagactacctttgagccaattccaatacattactgtgcaccaccaggc
tttgccattatgaaatgtaatgaaggaaattttagtggaaatggaagctgtacaaatgtgagtactgtacaatgc
acacatggaataaagccagtgatatccactcagttaatcctaaatggaagcttagatacagatgatattgttatt
agacatcatggggtaatctgttggtgcaatggaatgagacagtgtcaataaattgtacaaggccaggaaataat
acaggaggacaggtgcagataggacctgctatgacattttataatatagaaaaaatagtaggagacgttagacaa
gcatactgtaatgtctctgaagaatggggatcaatgtggaataaaacaaaaaagaagataaaaagactcctggga

aacaacacaactttcaaagctcaggataaaaatggaggagacctagaagtaacacacttaatgttcaattgtrra
ggagagttttctattgcaacacttccagattatttaatgagagcgagaacaagactaataagactattattctg
ccctgtagaataaaacaaattgtaratttgtggacaagagtagkaaaaggaatttatgcaccaccaattcgggga
aatcttagctgtarctccagtatcactggactgattctagaacatagtggtgaaaacggtaacaagacagtatat
ccctcaggaggaaacatggttaatctctggagacaagaactgtataagtacaaagtagttagtatagaacccata
ggagtagcaccaggtaaagccaaaagacgcacagtgagtagagaaaagagagcaagtctatgggctgctcagga
aaaactatatgctataccactgtgccttggaatgatacytggagcagtaatacctcctatgatacaatctggrgt
aatctcacctggcaacaatgggatcggaaagtaaggaactattcaggtgtcatttttgatcttatagagcaagca
caagaacaacaaaacacaaatgagaaagcactcttggaattggatcaatgggcaagtctgtggaactggtttgat
attacaaaatggctgtggtacataaaaatagctataatggtagtagcaggcattataggcataagaattataagt
gcaataataactataatagcaagagttaggcagggatattctcccctctcgttgcagctcagaatacgcttgtgg
ggaataatagcatattggggaaaagagttaaaagatagtgctatcagcttgcttaatacaacagctattgtagta
gcagagggaacagataggtttatagaattagcacaaagaataggaaggggaatattacacatacctagaagaatc
agacagggcctagaaagagcacttttataaatggaaaaatttggtcaaagagcagcctagtaggatggccagaa
atcagagaaagaataagaagacaaactccagagccagcagtaggagtaggagcagtttctcaagacctagctaat
cgagggccatcaccacaagcaatactaaagataataatcaaactgtagcttggctagaagcacaagaagaasag
gaggtaggctttccagtacgccctcaagtaccgctgaggccaatgacctataaagcggcttttgatctttccttc
tttttaaaagaaaagggggactggaagggctagtttggtccagaaaaaggcaagaaattctagacctctgggtt
tatcacacacaaggtttcttccctgactggcagaactacacaccagggccaggagttagataccccytgtgcttt
ggatggtgcttcaaactagtaccattgtcagaggaagcagtagaagaagctaatgaaggagacaataatgccctc
ctgcatcccatatgtcaacatggagtagatgatgatcacaaacaagtgctggtgtggcggtttgacagctccctg
gcaagaagacacgtagcaaaagagctgcatccggacttctacaagaactgctga
>#80 DQ314732 7407 nt
atgggtgcgagagcgtcagtattaagtgggggaaaattagatgcatgggaaaaaattcggttacggccaggggga
aggaaaaaatataagatgaaacatctaatatgggcaagcagagagttggaaagatttgcaattaaccctggcctt
ttagaaacagcagagggatgtcaacaaataatagaacagttacagtcaactctcaagacaggatcagaagaactt
aaatcattatataatacagtagcaaccctctggtgcgtgcaccagaggatagaggtaaaagacaccaaagaagct
ttagacaaaatagaggaagcacaaaataagagtcagcaaaagaaacagcaagcagctgctggcacaggaagcagc
agcaaagtcagccaaaattaccctatagtgcaaaacgcacaagggcaattggtgcatcagcctttatcacctaga
actttgaatgcatgggtgaaagtagtagaagaaaagggttttaacccagaggtaatacccatgttctcagcatta
tcagagggagccgccccacaagatttaaatatgatgctaaatatagtgggggacaccaggcagcaatgcaaatg
ttaaaagaaaccatcaatgaggaagctgcagaatgggatagggtacacccagtacatgcagggcctattccacca
ggccagatgagggaaccaaggggaagtgacatagcaggaactactagtacccttcaagaacaaataggatggatg
accaacaatccaccgatcccggtgggagacatctataaaagatggataatcctgggattaaataagatagtaaga
atgtatagccctactagcattttggacataagacaagggccaaaagagcccttcagagactatatagataggttc
tataaaactctcagagcagaacaagctacacaggaggtgaaaaactggatgacagaaaccttgctagtccaaaat
gcgaatccagactgtaggtccattttaaaagcattaggaagaggcgctacattagaagaaatgatgacagcatgc
cagggagtgggaggacctagccataaagcaaggttttggccgaggcaatgagccatgtacaacaagcaaatata
atgatgcagagaggcaattttaagggccagaaaagaattaagtgcttcaactgtggcaaagaaggacacctagcc
agaaattgcagggcccctagaaaaaagggttgttggaaatgtgggaaggaaggacatcaaataaaagactgcact
gagagacaggctataggaggacagctgaaagaagctctattagatacaggagcagatgatacagtattagaagat
ataaatttgccaggaaaatggaaaccaaaaatgataggggggaattggaggttttatcaaggtaaggcagtatgat
caaatacctatagaaatttgtggaaaaaaggctataggcacagtattagtaggacctacacctgtcaacataatt
ggacgaaatatgttgactcagcttggttgtactttaaatttcccaattagtcctattgacactgtaccagtaaca
ttaaagccaggaatggatggaccaaaggttaaacagtggccattaacagaagaaaagataaaagcattaacagaa
atttgtaaagagatggaagaggaaggaaaaatctcaaaaattgggcctgaaaatccatacaatactccagtattt
gctataaagaaaaaggacagcaccaaatggagaaaattagtagatttcagagagctcaataaaagaactcaggac
ttttgggaagttcaattaggaataccgcatccagcaggtttaaaaaaggcaaaatcagtaacagtactagatgtg
ggagatgcatatttttcagttcctttagatgaaagctttagaaagtatactgcatttaccatacctagtataaac
aatgagacaccaggaatcagatatcaatacaatgtgctgccacagggatggaaaggatcaccggcaatattccag
tgtagcatgacaaaaatcttagagccctttagaataaaaaatccagaaatggttatctatcaatacatggatgac
ttgtatgtaggatctgacttagaaatagggcagcacagagcaaaaatagaggagctaagagctcatctattgagc
tggggatttactacaccagacaaaaagcatcagaaggaacctccgttcctttggatgggatatgaactccatcct
gacagatggacagtccagcctatagaactgccagaaaaagacagctggactgtcaatgatatacagaaattagtg
ggaaaactaaattgggcaagtcaaatttatgcagggattaaggtaaagcaactgtgtaaactcctcaggggagct

aaagcactaacagacatagtaccactgactgaagaagcagaattagagttggcagagaacagggagattctaaaa
caccctgtgcatggagtatattatgacccgtcaaaagacttagtagcagaagtacagaaacaagggcaggaccaa
tggacatatcaaatttatcaagagccatttaaaaatctgaaaacagggaaatatgccagaaaaaggtctgctcac
actaatgatgtaagacaattaacagaagtggtgcaaaaaatagccacagaaggcatagtaatatggggaaagacc
cctaaatttagactacccatacaaggagaaacatgggaaacatggtggatggagtattggcaggctacctggatt
cctgaatgggagtttgtcaatacccctcctctagtaaaattatggtaccaattagaaaaagaccctatagtagga
gcagagactttctatgtagatggggcagccagtagggagactaagctaggaaaagcagggtatgtcactaataga
ggaagacaaaaggtagtttccctaactgagacaaccaatcaaaagactgagttacatgcaatccatttagccttg
caggattcagaaccagaagtaaatatagtaacagactcacaatacgcattaggaatcattcaggcacaaccagac
aggagtgaatcagaagtagtcaaccaaataatagaggagctgataaaaaaggaaaaggtctacctgtcatgggta
ccagcacacaaggggattggaggaaatgaacaagtagataaattagtcagttcaggaatcaggaaggtgctattt
ttagatgggatagataaggctcaagaagaacatgaaagatatcacagcaattggagaacaatggctagtgatttt
aatttgccacctatagtagcaaaggaaatagtagccaactgtgataaatgtcaactaaaaggggaagctatgcat
ggacaagtagactgtagtccagggatatggcaattagattgcacacatctagaaggaaaagtcatcctggtagca
gtccacgtggccagtggatatatagaagcagaagttattccagcagaaacaggacaggaaacagcatactttctg
ctaaaattagcaggaagatggccagtaaaagtaatacacacagacaacggtagcaatttcaccagcgctgcagtt
aaagcagcctgttggtgggccaatgtccgacaggaattcgggatcccctacaatccccaaagtcaaggagtagta
gaatctatgaataaagaattaaagaaaatcatagggcaggtaagagagcaagctgaacaccttaagacagcagta
caaatggcagtattcatacacaattttaaaaggaaaggggggattggggagtacagtgcaggggaaagaataata
gacataatagcaacagacatgcaaactaaagaattacaaaaacaaattacaaaaattcaaaattttcgggtttat
tacagggacagcagagacccaatttggaaaggaccagcaaaactactctggaaaggtgaaggggcagtagtaata
caagacaatagtgatataaaggtagtaccaagaagaaaagcaaagatcattagggatctagtaaaacatcatatg
tatatctcaaagaaagctaaaaagtggttttatagacatcattatgaaagtcagcatccaaaggtaagttcagaa
gtacatatcccactaggagaggctacattagtaataagaacatattggggtctgcagacaggagaaaaggactgg
caattgggtcatggagtctccatagaatggaggcaaagaaaatatagcacacagatagatcctgacctagcagac
caactgattcatctacattattttgactgtttttcagaatctgccataaggaaagccatattaggacaagtagtt
agacgtaggtgtgaatacccatcaggacataacaaggtaggatccctacaatatttggcactgaaagcattaaca
acaccaaaaaggataaagccacctctgcctagtgttaagaaattaacagaagatctgttagaggagcttaaaaac
gaagctgttagacattttcctaggccttggcttcatggcctaggacagcacatctatgacacttatggggatact
tgggaaggggttgaagctataataagaattttgcaacaactactgtttgttcatttcagaattgggtgtcaacat
agcagaataggcattatacgagggagaagaggcaggctagagccctggaatcatccgggaagtcagcctacaact
gcttgtagcacgtgttactgtaaaatatgttgctggcattgccaactatgctttctaaaaaaaggcttaggcatc
tccgcctcaaacaacttgtgggttacagttcattatggggttcctgtgtggagagatgcagataccaccctattt
tgtgcatcggatgccaaggcacatgaaacagaagtgcacaatgtctgggccacacatgcctgtgtacccacagac
cccaacccacaagaaatatacatggaaaatgtaacagaaaattttaacatgtggaaaaataacatggtagagcag
atgcaggaggatgtaataagtttatgggatcaaagtataaaaccatgtgtaaagttaactcctctctgcgttact
ttaaattgtaccaataaggttcgggcgatcaatgtcacatcaagctctagcataggaaatataacagatgaatta
agaaactgttcttttaatataaccacagaactaagagataagaaacagaaggtccatgcactttttttataagctt
gatatagtacctattgaaaataacaatagtagtaacaatagtagtgagtataggttaataaattgtaatacttca
gtcattaagcaggcttgtccaaagatatcctttgatccaattcctatacattattgtactccagctggttatgcg
attttaaagtgtaatgataagaatttcaatgggacagggccatgtaaaaatgtcagctcagtacaatgcacacat
ggaattaagccagtggtatcaactcaattgctgttaaatggcagtctagcagaagaagagatagtaatcagatct
gaaaatctcacaaacaatgtcaaaaccataatagtacatcttaataaatctgtagaaatcaattgtaccagaccc
tccaacaatacaagaaaaagtataactataggaccaggacaaatgttttacagaacaggagaaataataggagat
ataagaaaagcatattgtgagattaatggaacagaatggaatgaaactttaagacaggtagctgtaaaattaaaa
gagcactttaataagacaataatatttaaaccaccctcaggggggagatctagaaattacaatgcatcattttaat
tgtaaaggggaatttttctattgcaatacaacacaactgtttaatagtacttgggtaggaaatgaaaccaaaaag
ggggataatggcactataagaaatgaaaccaaaaaggggggataatggcactatcatacttccatgcaggataaag
caaattataaacatgtggcaggaaacaggacaagcaatgtatgctcctcccatcagtggaacaattaattgtgta
tcaaatattacaggactactattgacaagagatggtggaggtaataatgactcgagtcagaacgagaccttcagg
cctggaggggggaaatataaaggacaattggagaagtgaattatataaatataaagtagtagaaattgaaccacta
ggaatagcacccaccagggcaaagagaagagtggtggagagagaaaaaagaggactttggggctgctctggaaaa
atcatctgcaccactgctgtgccctggaactccacttggagtaataaatcttatgaagagatttgggagaacatg
acatggatagaatgggagagagaaattagcaattacacaaaacaaatatatgagatacttacagaatcgcaaaac
cagcaggataagaatgaaaaggatttgttagaactggataaatgggcaagtctgtggacttggtttgacataacc

cattggctgtggtatataagaatatttataatgatagtaggaggtttaatagggttaagaataattttgctgtg
cttgctatagtacatagagttaggcagggatactcacctttgtctttccaggctatttctttgtttgatgcttca
gcaatagcagtagcggggtggacagatagggttatagaagtagcacaaggagtttggagagctattctccacata
cctaggagactcagacagggcttggaaagggctttgctataaatgggaggcaagtggtcaaaaagtagcatagtg
ggatggcctcagatcagggagaaaataaagcaaactcctccagcagcagaaggagtaggtgcagtatctcaagat
ctagataaacatggagcagtaacaagtagtaatatgaataatgctgattgggactggctgaaagcacaagaggaa
gaggaggtaggctttccagtcaggccacaggtacctctaagaccaatgacatttaagggagctttcgatctaagc
ttctttttaaaagaaaaggggggactggaggggctaatttactccaagagaagacaagagatccttgacttgtgg
gtctatcatacacaaggtttcttccctgattggcacaactacacaccagggccagggatcagatatccactatgt
tttggatggtgcttcaagctagtaccagttgacccaagagaagtagaggaggacaacaaaggagaaaacagcagc
ctgttacaccccataagccagcatggaatagaggacgaagaaagagaagtgctgatgtggaagtttgatagtgcc
ctagcacgaagacacatagcccgagaaatgcacccagagttctataaaaactgctga
>#81 AF377956 7374 nt
gcaagagcgtcactattaagcgggggaaaattagatgatttggaaaaaattcggttaaggccagggggaagaaa
aaatataggctgaaacatatagtatgggcaagcagggagctagaaagatttgcacttaatcctggccttttagag
acaaaggaaggctgtaaacaaataataggacaactacaaccatcccttcagacaggatcagaagagcttaaatca
ttattcaacacaatagtagtcctctattatgtacatcaaaggataaaaataggagacaccaaggaagctttagat
aagctacaggaagaacaagacaaaagtcagcaaaaacacaaccagcagcggctgacaaaggggtcagtcaaaat
taccctatagtacagaatcttcagggacaaatggtacaccagtctctatcacctagaactttaaatgcatgggta
aaagtaatagaagagaaggctttcagcccagaagtaatacccatgttttcagcattatcagaaggagccacccca
caagatttaaacaccatgctaaacacagtgggggacatcaagcagccatgcaaatgttaaaagataccatcaat
gaggaagctgcagaatgggacagattacatccagtgcaggcaggacccatcccaccaggtcagataagagaacct
aggggaagtgatatagcaggaactactagcaacctacaggaacaaatagcatggatgacaagcaacccacctgtc
ccagtaggagaaatctataaaagatggataatcctaggattaaataaaatagtaagaatgtatagccctgtcagc
attttggacataaaacaagggccaaaagaaccctttagagactatgtagacaggttctttaaaactctaagagct
gagcaagcttcacaggaagtaaaaggctggatgacagacaccttgttggtccaaaatgcgaacccagattgtaag
atcattttaaaaggattaggaacaggggctacactagaagaaatgatgacagcatgtcagggggtgggggacct
ggccataaggcaagaattttggctgaggcaatgagccaagtaacatctacatccatattgatgcagaaaagcaac
tttaagggccaaagaagaaatgttaagtgtttcaactgtggcaaagaaggacatatagctaaaaattgcagggcc
cctagaaaaagggctgttggaaatgtggaaaggaaggacaccaaatgaaagactgcactgagagacaggctaca
ataaaagtagaggggcaactaagggaggcactattagatacaggggcagatgatacagtattagaagatataaat
ttgtcaggaaaatggaaaccaaggatgataggggggaattggaggttttatcaaagtaagacagtatgaccaaata
cccatagaaatttgcggacaaaaggctataggtactgtattagtaggacctacgcctgtcaacataattggaaga
aatatgttgactcagattggttgcactttaaattttccaattagttctattgaaactgtaccagtaaaattaaag
ccaggaatggatggcccaaaggttaaacaatggccattgacagaagaaaaaataaaagcattaacagaaatctgt
acagagatggaaaaagaaggaaaaatttcaaaaattgggccagaaaatccatacaatactccagtatttgccata
aagaaaaaggacagtactaaatggagaaaattagtagatttcagagaacttaataaaagaactcaagatttttgg
gaggttcaattaggaataccacaccctgcagggttaaaaaagaaaaaatcagtaacagtactagatgtggggat
gcatatttttcagttcccttagataaggagttcaggaagtacactgcattcaccatacctagtatcaacaatgag
acgccaggaattagatatcagtacaatgtgcttccacagggatggaaagggtcaccagcaatattccaaagtagc
atgacaaaaatcttagagccctttagagtaaaaaatccagaaatagttatttaccaatacatggatgatttgtat
gtagggtctgacttagaaataggacagcataggacaaaaatagaagagttaagagaacatctattgagatgggga
tttactacaccagataaaaaacatcagaaggaacccccatttctttggatggggtatgaactccatcctgacaaa
tggacagtacaggctatacaattgccagacaagagcagctggactgtcaatgatatacagaagttagtgggaaaa
ctaaattgggcaagtcagatttatccagggattagagtaaagcacctatgcaaactccttagaggagccaaagcg
ctaacagacgtagtgccactaacagtagaagcagaattagaaatggcagagaacagggaaattctaaaagaacca
gtacatggggtatattatgacccatcaaaagatctaatagcagaagtacagaaacaagggcacgaccaatggaca
tatcaaatttatcaagagccacataaaaatctgaaaacaggaaagtatgcaagaaggaggtctgcccacactaat
gatgtaaaacaattaacagaagtagtgcaaaaaatagccacagaaggcatagtaatatggggaaaagttcctaaa
tttaggctacccatacaaaaagaaacatgggaaacatggtggacagagtattggcaggccacctggattcctgaa
tgggagtttgtcaatacccctcctctagtaaaattatggtatcaattagaaacagagcccatattaggagcagaa
actttctatgtagatggggcagctaatagagagaccaaaataggaaaagcaggatatgttactgacagaggaaga
caaaaagttgtccccctaactgagacaacaaatcagaagactgaattacaagcaattcatttagctttgcaggac
tcaggatcagaagtaaacatagtaacagactcacagtatgcattaggaatcattcaagcacacccagataagagt
gaatcagagttagtcaaccaaataatagagcaattaatacaaaaagaaagggtctacctgtcatgggtaccagca

cataaaggaattggaggaaatgaacaagtagataaattagtcagtgctggcatcaggaaagtactgtttttggat
gggatagataaggctcaagaagaacatgaaaaatatcacagcaattggagagcaatggctagtgattttaatttg
ccacctgtagtagccaaagaaatagtagccagctgtgataaatgtcagctaaaaggggaagccatgcatggacaa
gtagactgcagtccagggatatggcaactagattgtacacatttagaaggaaaaattatcttggtagcagtccat
gtggctagtggttatatagaagcagaagttatcccagcagaaacgggacaggaaacagcctacttcctactaaag
ttagcaggaagatggccagtaaaaataatacatacagataatggcagcaatttcaccagtactgtggttaaggca
gcctgttggtgggcaggtatccagcaggaatttggaattccctacaatcctcaaagtcaaggagtagtagaatct
atgaataaagaattaaagaaaattataggacaggtaagagatcaagctgaacatcttaagacagcagtacaaatg
gcagtattcattcacaattttaaaagaaaaggggggattggggggtacagtgcaggggaaagaataatagacata
atagcaacagacatacaaactaaagaattacaaaaacaaattacaaaaattcaaaactttcgggtttatttcagg
gacagcagagacccaatttggaaaggaccagcaaagctactctggaaaggtgaaggggcagtagtcatacaagac
aataatgaaataaaagtagtaccaagaagaaaagcaaaaatcattagggatacatggaacagtttagtaaaacat
catatgtatgtttcaaggagagctaaaggatggttttatagacatcactatgaaagcaggcatccaagagtaagc
tcagaagtacacatcccactaaaggatgattctaaattagtaatagtaagctattggggtctacatacaggagaa
agagattggcatttgggtcaaggagtctccatagaatggaggcagaaaagatataggacacaagtagaccctggc
ttggcagaccaattaattcatttgtattactttgattgtttttcagaatctgccataaggaaagccatattagga
cagagagttagtcctaggtgtaactatcaagcaggacataacaaggtaggatccctacaatatttggcactaaca
gcattaataaccccaaagaagataaagccgcctttgcctagtgtcaggaaactagtagaggatcttttagaggag
cttaagcatgaagctgttagacatttccctagggagtggctccatggcttaggacagcatatctacaacacctat
ggggatacttgggagggagttcaagctataataagaacactgcaacaactactatttatccatttcagaattggg
tgtcatcatagcagaataggcattattcgacaaagaagactaagactagagccctggaatcatccaggaagtcag
cctgagactccttgtaataaatgttactgtaaaaagtgttgctttcattgccaattgtgctttacaaggaagggc
ttaggcatctccgctgcagataacttgtgggtcacagtctattatggagtacctgtgtggaaagaagcaaccact
actctattttgtgcatcagatgctaaagcatatgaaagagagatacataatgtctgggctacatatgcctgtgta
cctacagaccccaacccacaagaattagttctgggaaatgtaacagaaaattttaacatgtggaaaaataacatg
gtagaccagatgcatgaagatataatcagtttatgggatcaaagcctaaagccatgtgtacagataaccccactc
tgtgtaaccttaaattgtactgatgttcctgttaacatcactaatgggaacagcaccctggataacatcaccctg
gaagaacaaggggaaataaaaaactgttctttcaatatcaccacagagataaacgatattaagaaaaaagaatct
gcaatttttataggcttgatgtagtaccaatcaataatagtactagtgaatataggctactaagttgtaatacc
tcaaccgttacacaggcttgtccaaaggtgtcctttgatccaattccaatacattattgtgctcctgctggtttt
gcgattctaaagtgtaatgataaagagttcaatgggacagggttatgtaggaatgtcagcacagtacaatgtaca
catggaattaaaccagtagtgtcaactcaactactgttaaatggcagcctagcagaaggagatatagtaattaga
tctgaaaatatctcagataatgcaaaaaccataatagtacagtttaatagatctgtagcaattaactgtacaaga
cccaccaacattacaagaagaagtatgcgtataggaccaggacgagtattttatgcaacaggtaccgtactagga
gatataagaaaggcatattgtaccattaatggaacactgtggaataaaactttagaaggagtagctaaagaggtc
caaagccaccttaataaatcaataacatttgcgccatcatcaggaggggacctagaagttacaacacatagtttt
aattgtagaggagagtttttctactgcaacacagtagctctgtttaatgcaactaacatgactaatgcaatgaac
aggtccaatggcattatcactcttccatgtagaataagacaaattgtaaacatgtggcaaagagtaggacgagca
atgtatgccgctcccattgctggacaaattcagtgtaactcaagcatcacaggtctaatattgacaagagatggt
gggaaaaataataccaataatgacaccctcagacctggaggggggagatatgagagacaattggagaagtgaactg
tataaatataaggtagtaaaaattgaaccactaggagtagcacccaccaaggcaaaaagacaagtggtgaagaga
gaaagagaaaaaagagggatttggggatgctctggaaaactcatctgcaccactaatgtgccctggaattctagt
tggagtaataaatctcagaatgaaatttgggagaacatgacctggatgcagtgggaaaaagagatcagtaattac
acaggcacaatatacaaattaatagaaaatgcacaaaaccagcaggaaaagaatgaacaggacttattggcattg
gacaaatgggacaatctgtggagttggtttactataacaaattggttgtggtacataaaattattcataatgata
gtaggaggcttgataggattaagaatagtttttgctgtgcttgctgtaataaatagggttaggcagggatactca
cctttgtcgttacaggctattgatctgcttaataccacagcaatagtggtagctgaaggacagatagaatcata
gaagttctgcaaagagctggtagagctattctccacatacctagaagaataagacagggtgcagaaagggctttg
ctataaatgggtggcaagtggtcaaaaagtagtaaagttggatggcctactataagggaaagaataagacaaacc
cctgcagcagcaccaggggtgggagcagtgtctcaagacttagataaacatggggcaattacaagcagccagact
agggatactaatagtgacttggcatggctagaagcgcaagaggatgaggaggtaggtttcccagtcagacctcag
gtacctttaagaccaatgacttataagggagctttcgatctcagtttctttttaaaagaaaaggggggactggaa
gggttaatttactccaagagaagacaagaaatccttgatctgtgggtctaccacacacaaggctacttccctgat
tggcagaactacacaccagggccagggactagatatccactgacctttggatggtgcttcaagctagtaccagtt
gagccagaggaggtagaaaaggccaatgaaggagagaacaactgtttattgcaccctatgagccaacatggaatg

gaggacgaagacagggaagtgttaaagtggaagtttgacagctccctagcacggagacacagagccagagagatg
catccggagtactacaaagactga
>#82 AB254156 7467 nt
atgggtgcgagagcgtcaatattaagaggcggaaaattagatgcatgggaaaaaattaagttaaggccaggagga
aagaaacattatatgctaaaacacttagtatgggcaagcagggagctggaaagatttgcacttaaccctggcctt
ttagagacagcagaaggctgtaaacaaataatacaacagctaactccagctcttaagacaggaacagaggaactt
agatcattatttaacacagtagcaactctctattgtgtacataaaaatatagaggtacgagacaccaaggaagcc
ttagacaaaatagaggaagaacaaaagaaatgtcagcaaaaaccacagcaagcagaagcagctgacaaaggaaaa
gtcagtcaaaattaccctatagtgcagaatctccaagggcaaatggtacaccaaagcctatcaccgagaacttta
aatgcatgggtaaaagtaatagaggagaaggctttcagcccagaggtgatacccatgtttacagcattgtcagaa
ggagccaccccacaagatttaaacaccatgttaaatacagtgggggacatcaggcagccatgcaaatgttaaag
gataccatcaatgaggaggctgcagaatgggatagggtacatccagtgcatgcagggccagttgcaccaggccaa
atgagagaaccgaggggaagtgacatagcaggaaccactagtacccttcaagaacagatagcatggatgacaagt
aacccacctattccagtaggagacatctataaaagatggataattctggggttaaataaaatagtaagaatgtat
agccctgtcagcattttggacataaaacaagggccaaaggaaccctttagagactatgtagaccggttctttaaa
actttaagagctgaacaatctacacaagaagtaaaaaattggatgacagacaccttgttggtccaaaatgcgaac
ccagattgcaagaccattttaagagcattaggaccaggggcttcattagaagaaatgatgacagcatgtcaggga
gtgggaggacctagccacaaagcaagagtgttggctgaggcaatgagccaagcaggcaatgcaaacataatgatg
cagagaggcaattttaagggccctagaaaaattgttaaatgtttcaactgtggcaaggaagggcacatagccaga
aattgcagggcccctaggaaaagggctgttggaaatgtggaaaggaaggacaccaaatgaaagactgtactgaa
agacaggctgtaggggggccagataaaggaagctctcttagatacaggagcagatgatacagtattagaagaaata
aatttgccaggaaaatggaaaccaaaaatgataggagggattggaggttttatcaaagtaagacagtatgatcaa
ataactatagaaatttgtggaaaaagggctgtaggttcagtattagtagggcctacacctgtcaacataattgga
agaaatatgttgactcagcttggatgtacactaaattttccaattagtcctattgagactgtaccagtgaaatta
aagccaggaatggatgggccaaaagttaaacaatggccattgacagaagaaaaaataaaagcattaacagcaatt
tgtgaggaaatggagaaggaaggaaaaattacaaaaattgggcctgaaaatccatataatactccagtacttgcc
ataaaaaagaaggacagtactaagtggagaaaattagtagatttcagggaactcaataaaagaactcaagacttt
tgggaagttcaattaggaataccacacccagcagggttaaaaaaggagaaatcagtgacagtgctagatgtgggg
gatgcatatttttcagttcctttagatgagaacttcaggaaatatactgcattcaccatacctagtataaacaat
gaaacaccagggattagatatcaatataatgtgcttccacagggatggaaaggatcaccagcaatattccagagt
agcatgacaaaaatcttagagccctttaggacaaaaaacccagaaatagttatctatcaatatatggatgacttg
tatgtaggatctgacttagaaatagggcaacatagagcaaaaatagaggagttaagagaacatttattgaaatgg
ggatttaccactccagacaaaaaacatcagaaagaaccccccatttctgtggatggggtatgaactacatcctgac
aaatggacagtacagcctatacagctgccagaaaaagaaagctggactgtcaatgatatacagaagttagtggga
aaattaaactgggcaagtcagatttatccaggaattaaagtaaagcaactgtgtaagctccttaggggagccaaa
gcactaacagacatagtaccactgactgaagaagcagaattagaattggcagagaacagggaaattctaagagaa
ccagtacatggagtatattatgacccatcaaaagacttaatagctgaaatacagaaacagggacatgaccaatgg
acatatcaaatttaccaagacccattcaaaaatctgaaaacagggaagtatgcaaaaatgaggactgcccacact
aatgatgtaagacagttagtagaggcagtgcaaagaataactatggaaagtatagtaatatggggaaagactcct
aaatttagactacccatccaaaaagacacatgggagacatggtggacagactattggcaagccacctggattcct
gaatgggagtttgttaatacccctcccctagtaaaattatggtaccagctagagaaagaacccatagtaggagca
gaaactttctatgtagatggagcagctaatagggaaactaaaataggaaaagcaggatatgttacagatagagga
agacagaaaattgtttctctaactgaaacaacaaatcagaagactgaattacaagcaattcagctagctttgcag
gattcaggatcagaagtaaacatagtaacagactcacagtatgcattaggaataattcaagcccaaccagataag
agtgaatcagagttagttaatcaaataatagaacagttaataaacaaggaaaaagtctacctgtcatgggtacca
gcacataaaggaattggaggaaatgaacaagtagacaaattagtaagtagtggaatcaggagagtgctgtttcta
gatggaatagataaggctcaagaagagcatgaaaaatatcacagcaattggagagcaatggctagtgaattcaat
ctgccacccatagtagcaaaagaaatagtagctagctgtgataaatgtcagctaaaaggggaagccatacatgga
caagtggactgtagtccagggatatggcaattagattgtacacatttagaaggaaaaatcatcctagtagcagtc
catgtagccagtggctacatagaagcagaggttattccagcagaaacaggacaggaaacagcatacttcatacta
aaattagcaggaagatggccagtcaaagtaatacacacagacaatggcagtaatttcaccagtgctgcagttaag
gcagcctgttggtgggcaggtatccaacaagaatttggaattccctacaatccccaaagtcagggagtagtagaa
tccatgaataaagaattaaagaaaatcatagggcaagtaagagagcaagctgagcaccttaagacagcagtacaa
atggcagtattcatccacaattttaaaagaaaaggggggattgggggggtacagtgcaggggaaagaataatagac
ataatagcaacagatatacaaactaaagaactacaaaaacgaattatacaaattcaaaattttcgggtttattac

agagacagcagagaccctatttggaaaggaccagccaaactactctggaaaggtgaaggggcagtagtaatacaa
gataatagtgacataaaggtagtaccaaggaggaaagtaaaaatcattaaagacacatggaatagtttagtaaag
caccatatgtatgtttcaaagagagctaagggatggttctacagacatcattttgaaagcagacatccaagagta
agttcagaagtacacatcccattaggggatgctagattaatagtaaagacatattggggtttgcagacaggagaa
agagattggcatttgggtcatggagtctccatagaatggagattgagaaggtatagcacacaagtagaccctagc
ctggcagaccaactaattcatatgcattattttgattgttttgcagactctgccataaggaaagccatattagga
catatagttagccctagatgtgactatcaagcaggacataacaaggtaggatctttacaatacttggcactgaca
gcattgataaaaccaaaaaagagaaagccacctctgcctagtgttagcaaattggtagaggatcttttagaagag
ctcaagcaggaagctgtcagacactttcctagaccatggcttcatggcttaggacagcatatctatgagacctat
ggggatacatggacgggagttagagtgataataagaatcctgcaacaattactgtttactcatttcaggattggg
tgccagcatagcagaataggcattttgcaacagagaagagcaagactagagccctggaagcatccaggaagtcag
cctagaaccgcttgtaattcatgttactgcaaacgctgcagctatcattgtccagtttgctttctgacaaaaggc
ttaggcatttccatggagggcttgtgggtcactgtacactatggggtacctgtgtggaaagaagcaaagactact
ctattttgtgcatcagatgctaaagcatatgagacagaagtgcataatgtctgggctacacatgcctgtgtaccc
acagaccccagcccacaagaagtgtatttaaaaaatgtaacagaaaattttaatatgtgggaaaataacatggtg
gatcagatgcatgaggatataatcagtttgtgggatcaaagcctaaaaccatgtgtaaagttaaccccactctgt
gtcactttagactgtagtcataatattaccattaggaatagtacctccagtgagaataatagtacctccagtacc
aacagtacaatcttgagtgaaataatgagaaattgtactttcaatgtaaccacagaactaaggataagcagaca
aaagaatatgcactttttataaacttgatatagtaaaacttgagaagaactctagtgaatatagattaataaat
tgtaattcatcaaccgtaacacaggcctgtccaaaagtctcttttgacccaattcctatacattattgtgctcca
gctggttatgcgattctaaagtgtaataataattatacattcaatggaacaggaccatgtaataatgtcagcaca
gtacaatgtacacatggaattaaaccagtggtgtcaactcaactactgttaaatggtagcctagcagagggaggg
atagtaattagatctaaaaatctgacagacaatgccaaaacaataatagtacatcttaatgaatctgtaagaatt
gtgtgtacaagacctggcaataatacaagaaaaagtgtaaggataggaccaggacaagcattctttgcaacagga
gaagtaataggagatataagaaaggcacattgtaacattactgagaggacatggaataacactttacaaaagata
agtagaaaattagaaaaacactttccgaacaaaactataaaatttgcaccatcctcaggaggggacccagaaatt
acaacacatagctttaattgtagaggagaatttttctattgcgacacatcaaacctgtttaacagcacatttaat
gttaacagcacatttaatggtacagataatgcaaatagcacacagaacatcacactccaatgcagaataaaacaa
attataaacatgtggcaggaggtaggacgagcaatgtatgcccctcccattgcaggaaacataacatgtatttca
aatatcacaggaatactattaacacgtgatggaaatgataattacacagagacattcagacctataggaggaaat
atgaaggacaattggagaagtgaattatacaaatataaagtggtagaaattaagccattaggagtagcacccacc
aaggcaaaaaggagagtggtggagagacaaaaaagagggatttggggctgctctggaaagctcatctgcaccact
gctgtgccttggaactctagttggagtaataaaacatataatgagatttgggataatatgacctggatgcagtgg
gatagagaaatcaataattacacagatacaatatactggttgcttgagaaatcacaaaaccagcaggaacaaaat
gaaaaagatttactagaattggacagttgggatagcctgtggagttggttaagcataacaaattggctgtggtat
ataagaatattcataatgatagtaggaggtttgataggtttaagaataatttttgctgtgctttctatagtaaag
agagttaggcagggatactcacctttgtcgtttcagtatttgggaagccttgtgcagtattggggtccggaacta
aaaaagagtgctattagcctgcttgataccatagcaatatcagtagctgagggaacagataggattatagaattg
ctaagaagaatttgtagagctatctgtagcatacctagaagaataagacagggctttgaagcagcattgcaataa
atggggaacaagtggtcaaaaggcagtatagttggatggcctgctgtaagagaaagattaagacgagccagacca
gcagcagagggagtaggaacagcagcagagggagtaggaacagcatctcaagacttagataaacatggggcactt
acaaccagcaacacagccaccaacaatgctgattgtgcctggctggaagcacaagaggaagaagaagtaggcttt
ccagtcagacctcaggtacctttaagaccaataacttataaggcagcattcgatctcagcttcttttaaaagaa
aaggggggactggaagggttaatttactccaagaaaaggcatgagatccttgatttatgggtctatcacacacaa
ggcttcttccctgattggcaaaactacacaccgggaccaggggtcagatatccactgacctttggatggtgcttc
aagctagtgccagttgacccaagtgaagtagaagaggccaatgaaggagagaacaactgtttgctacatcctata
agccagcatggaatagaggatgaagacagagaagtcttaaagtggcagtttgacagcagcctagcacgcagacac
ctggcccgcgagctacatccggagtactacaaaaactgctga
>#83 MF373132 7482 nt
atgggtgcgagagcgtcaatatttagaaggacggaattggatacttggaaaaaaattaagttaaagccaggggga
aagaaaaagtatatgatgaaacatctagtatgggcaagcagagagctggaaagatttgcacttgaccctggcctt
ttagaaacatcagaaggctgtaaacgaataatacaacagctacagccatctcttcagacaggaacaaaagaactc
atatccttgtataatacagtagtagttctatattgtgtacataacaatatagcggtaaatgacactaaggaagcc
ttagacaaagtaaaggaagaacaaaacaaaagtcagcaaaaaacacagcaggcagcaacggctggcaacaaacag
gtcagtcaaaattatcctatagtgcagaaccttcaagggcaaatggtacatcaaaacatatcaccaagaaccttg

aatgcatgggtgaaagtaatagaggagaaggctttcagcccagaggtaatacccatgtttacagcattatcagaa
ggagccactccacaagatttaaacaccatgttaaatacagtgggggacatcaagcagccatgcaaatgttaaaa
gacaccatcaatgaggaggctgcagaatgggataggttacatccagtgcaagcagggcctgtcgcaccaggccaa
ataagagatccaaggggaagtgacatagcaggaactactagtacccttcaggaacaaataacatggatgacaagt
aacccacctatcccagtaggagacatctataaaagatggataattctagggttaaataaaatagtaagaatgtat
agccctgttagcattttggacataaaacaagggccaaaggaaccctttagagactatgtagaccggttctttaaa
accttaagagctgaacaagctacacaagatgtgaaaaattggatgacagacaccttgttggtccaaaatgcgaac
ccagactgtaagaccattttaagggcattagggccaggagctacattagaagaaatgatgacagcatgtcaggga
gtgggaggacctggccacaaagcaagagtattggctgaggcaatgagccaagcaaacaatgtaaacataatgatg
cagagaaacaatttaagggcccaaaaagaattattaaatgtttcaactgtggcaaggagggcacatagccaga
aattgcagggcccctaggaaaaagggctgttggaaatgtggaaaggaaggacaccaaatgaaagactgtactaat
gaaaggcaggctgtaggggacagataaaggaggctctcttagacacaggagcagatgatacagtattagaagaa
ataaaattaccaggaaattggaaaccaaaaatgataggggaattggaggttttatcaaagtaagacagtatgat
caaatacttatagaaatttgtggaaaaaaggctataggttcagtattagtaggacctacacctgtcaacataatt
ggaagaaatatgttgactcaacttggatgcacactaaattttccaattagtcctattgaaactgtgccagtaaaa
ttaaagccaggaatggatggcccaaaggtcaaacaatggccattgacagaagagaaaataaaagcattaatagaa
atttgtgaagaaatggaaaaggaaggaaaaattacaaaaattgggcctgaaaatccatataacactccagtattt
gccataaaaaagaagaacagtactaagtggagaaaattagtagattttagggaactcaataaaagaactcaagac
ttttgggaagtgcaattagggataccacacccaggagggttaaaaaagaacaaatcagtgacagtactagatgta
ggggatgcatatttttcagttcctttagatgaaggtttcaggaaatatactgcattcaccatacctagtctaaac
aatgagacaccagggattagatatcaatataatgtgcttccacagggatggaaaggatcaccagcaatatttcag
agtagcatgacaaaaatcttagaaccctttagggcacaaaatccagaaatagtcatctaccaatacatggatgac
ttgtatgtagcatctgatttagagatagggaaacatagagcaaaaatagaggaattaagagaacatctatggagg
tggggattttacacaccagataagaaacaccagaaagaaccaccatttctttggatggggtatgaactccatcct
gacaagtggacagtacagcctatacagctgccagaaaaggatagctggactgtcaatgatatacagaagttagtg
ggaaaattaaactgggcaagtcaaatttatccagggattaaagtaaagcaactatgtaaactccttaggggaacc
aaagcattaacagacatagtaccactgactgaagaagcagaattagaattggcagagaacagggaaattctaaaa
gaaccagtacatggagtatattatgacccatcaaaagacttaataactgaaatacagaaacagggaaatgatcaa
tggacatatcaaatttaccaagaaccattcaaaaacctgaaaacagggaaatatgcaaaaatgaggactgcccac
actaatgatgtaaagcagttaacagaggtagtgcaaaaagtagccatggaaagcatagtaatatggggaaaaact
cctaaatttagattacctatccagaaagaaacatggaggcatggtggacagattattggcaagccacctggatt
cctgagtgggagtttgtcaatacccctcccctagtaaagttatggtaccagctggagaaagaacctatagcagga
gtagaaactttctatgtagatggagcagctaatagggaaactaaaatgggaaaagcaggatatgttactgacagg
ggaaggcagaaaattgtttctctaactgaaacaacaaatcagaaaactgaattacaagcaattcagctagcttta
caggactcaggatcagaagtgaacatagtaacagactcacagtatgcattaggaattattcaagcacagccagat
aagagtgaatcagagttagtcaatcaaataatagaacagttgataaaaaaggaaagggtctacctatcatgggta
ccagcacataaagggattggaggaaatgagcaaatagataaactagtaagtactggaatcaggaaagtgctgttt
ctggatggaatagataaggctcaagaagagcatgaaagatatcacaacaattggagagcaatggctgatgagttt
aatctgccacccatagtagcaaaagaaatagtagccagctgtgataaatgtcagctaaaggggaagccatgcat
ggacaagtagactgtagtccaggaatatggcaattagattgtacacatctagaaggaaaaatcatcctggtagca
gtccatgtagccagtggctacatagaagcagaggttattccagcagaaacaggacaagagacagcatactacata
ttaaaattagcaggaagatggccagtcaaagtaatacatacagacaatggcagtaatttcaccagtagtgcagtt
aaggcagcctgttggtgggcaggtatccaacaagaatttggaattccctacaatccccaaagtcagggagtagta
gaatccatgaataaagaattaaagaaaatcatagggcaggtaagagatcaagctgagcaccttaagacagcagta
caaatggcagtattcattcacaattttaaaagaaaaggggggattggggggtacagtgcaggggaaagaataata
gacatgatagcaacagatatacaaactaaagaactacaaaaacaaatcacaaaaattcaaaattttcgggtttat
tacagagacagcagagaccctatttggaaaggaccagccaaactactctggaaaggtgaaggggcagtagtaata
caagataacagtgacataaaggtagtaccaagaaggaaagtaaaaatcattaaggacacatggaatagtttagta
aagcaccacatgtatatctcaaagagaaccaggggatggttttatagacatcattttgaaagcagacatccaaaa
ataagttcagaagtacacatcccattaggggaggctaaattagtaataataacatattggggtttgcaaacagga
gaaagagattggcacttgggtcatggagtctccatagaatggagactgagaagatatagcacacaagtagaccct
agcctggcagaccaactaattcatatgcactattttgattgttttgcagactctgccataaggaaagccatatta
ggacatatagttagccctaggtgtgactatcaagcaggacataataaggtaggatctctacaatatctggcactg
acagcattgataaaaccaaaaaggattaagccacctctgcctagtgttaagaaattagtagaggatcttttagag
gaactcaagcaggaagctgtcagacactttcctagaccatggctccatagcttaggacaacatatctatgaaact

tatggggatacttggatgggagttgaagccataataagaattttgcaacaattactgtttatccatttcagaatt
gggtgtatgcatagccgaataggcattatgcgacaaagaagaacaagactagagccctggaaccatccaggaagt
cagcctaaaactccttgcaataattgctattgtaaatactgtagctatcattgtctagtttgctttcagacaaaa
ggcttaggcatttccgggaaggggacaaggaggacttgtgggtcacagtatattatggggtacctgtgtggaga
gatgcacaaacctctctgttttgtgcatcagatgctaaagcatatgatacagaggtgcataatgtctgggctaca
catgcctgtgtacccacagaccccaacccacaagaaatggttttaaaaaatgtaacagaaaattttgacatgtgg
aaaaataacatggtggatcagatgcatcaggatataatcagtttatgggatcaaagcctaaagccatgtgtaaag
atgaccccactctgtgttactttaaactgtacaaatgctacaactgctatgaatgctacaattgataataacatg
cagggagaaataaaaaattgtactttcaatgtaaccacagaaataagagataaggtaaagaaggtacatgcactt
ttttataaacttgatatagcaccacttggtaacagtgctagtagtgataacagttctagtggtggtaaaggtact
aggaataatgagtataatgattatatattaataaattgtaatacctcagccatagctcaagcctgtccaaaggtc
tcttttgacccaattcctatacattattgtgctccagcgggttatgcgattctaaagtgtaatgataagacattc
aatggaacaggaccatgcaataatgtcagcacagtacaatgtacacatggaattaagccagtggtatcaactcaa
ttactgttaaatggtagcctagcagaaggagacataataattagatctgagaatatgacaagcaatactaaaaca
ataatagtacatcttaatgaatctgtatcaattaattgtacaagacccaacaataatacaagagaaagtttaagg
ataggaccaggacaagcattatatgcaacaggagatataataggagatataagacaagcacattgtaatattagt
aaagaaaggtggaataaaactttagaaagagtaaagaaaagattagcagagtacttccctaatacaacaatactc
tttgcaccacccccaggaggggacctagaagttacaacacatagctttaattgtagaggagaatttttctattgc
aatacatcaaacctgtttaatagtagtaaaactaatataacagcaaatgaaaatggaaccatcacactcccatgc
agaataaaaccaattgaaaccttggggcaggggtaggaaaagcaattattgccccctccattaaaggaaaatta
cctggtacttccaaattcccaggcttaccactggccccgagtaggggtgaaaaaataggaacctatggaaatttg
gcaaaaagagaattttccccggggggggagatatgagtgacaattggagaagtgaattatataaatataaagtg
gtagaaattaatccattaggagtagcacccactaaggcaaaaaggagagtggtggagagagaaaaaaagagggatt
tggggctgctctggaaaactcatctgcaccactgctgtgccttggaatgaaagttggagtaataagtctcaaaat
gagatttgggataacatgacctggatgcagtgggatagggaaatcaataattacacaaatgaaatatacagattg
cttggggaatcgcaaatccagcaggagaaaaatgaacaggatttactagcgctggacaagtggcaaaatctgtgg
agttggtttgacataacaaactggctgtggtatataaaaatgttcataatgatagtaggaggcttgataggttta
agaatagtttttgctgtgctttctatagtaaatagaattaggcagggatactcacccttatcgttgcagtatctg
ggaagtcttgtgcagtattggggttctgagctaaaaaggagtgctattagtctgcttgataccacagcaatagca
gtaggtgaaggaacagataggattgtagaatttatactacgagtttgtagagctatctacaacatacctcgaaga
ataagacagggctttgaagcagctttgcaataaatgggggcaaatggtcaaaatgtagtccagtaggatggtct
gagataagagacagaataaaaagaactcccccggcagcagagggagtaggagcagcatcccgagacttagagaga
catggggcactgacaaccagcaacacacccagcaataatgctacttgtgcctggctggaagcgcaagaggaggaa
ggagatgtaggctttccagtcagacctcaggtacctttaagaccaatgacttataaggcagcattcgatctcagc
ttctttttaaaagaaaaggggggactggaagggttaatttactctaagcaaaggcaagacatccttgatttgtgg
gtctatcacacacaaggtttcttccctgattggcaaaactacacaccaggaccaggggtcagatatccactgacc
tttggatggtgcttcaagctagtgccagttgacccaaaggaagtggaagaagacaataaagatgagaacaacagc
ttgctacaccctgtgagcctgcatggagtggaggatgaagacaaagaagtactaatgtggaagtttgacagtcac
ctagcacgcagacacatggcccgcgagctacatccggagtattacaaagactgctga
>#84 KP109483 7476 nt
atgggtgcgagagcgtcaatattaagaggggaaaattagataaatgggaaaaaattaggttaaggccaggggga
aagaaacattatatgttaaaacacttagtatgggcaagcagggagctggaaagatttgcacttaaccctggcctt
ttagagacagcagaaggctgtaaacaaataataaagcagctacacccagctctccagacaggaacagaggaactt
aaatcattattcaacacagtagcaactctctattgtgtacatgcagggatagaggtacggacaccaaggatgcc
ttagacaaaatagaggaagaacaaaacaaaagtcagcaaaaaacacagcaggcaaaagaggctggcgggaaggtc
agtcaaaattatcctatagtgcagaatctccaagggcaaatggtacaccaggccatatcacctagaactttgaat
gcatgggtaaaagtagtagaggagaaggcttttagcccggaggtaatacccatgtttacagcattatcagaagga
gccaccccacaagatttaaacaccatgttaaatacagtggggggacatcaagcagccatgcagatgttaaaagat
accatcaatgaagaggctgcagaatgggatagaacacatccaatacatgcagggcctattgcaccaggccaaatg
agagaaccaaggggaagtgacatagcaggaactactagtacccttcaggaacaaatagcatggatgacaagtaac
ccacctgttccagtgggagacatctataaaagatggataattctggggttaaataaaatagtaagaatgtatagc
cctgttagcattttggacataaaacaagggccaaaggaaccctttagggactatgtagatcggttctttaaaact
ctaagagctgaacaagctacacaagatgtaaaaaattggatgacagacaccttgttgatccaaaatgcgaatcca
gattgtaagaccattttaagagcattaggaccagggcttcaatagaagagatgatgacagcatgtcaaggagtg
ggaggacctagccacaaagcaagagtgttggctgaggcaatgagccaaacaaacaatgccataatgatgcaaaga

agcaattttaaaggctctaaaagaactattaaatgtttcaactgtggcaaggaagggcacttagccagaaattgc
agggcccctaggaaaaagggctgttggaaatgtggaaaggaaggacaccaaatgaaagactgtactgaaagacag
gctgtaggggtcagataaaagaggctctcttagacacgggagcagatgatacagtgttaggagaaataaatttg
ccaggaaaatggaaaccaaaaatgataggaggaattggaggttttatcaaagtaagacaatatgatcaaatacct
atagaaatttgtggaaaaaggctataggctcagtattagtaggacccacacctgtcaacataattggaagaaat
atgttgactcagcttgggtgcacactaaattttccaatcagtcccattgaaactataccagtaaaattaaagcca
ggaatggatggcccaaaggttaaacaatggccattgacagaagagaaaataaaggcattaacagcaatttgtgat
gaaatggaaaaggaaggaaaaattacaaaaattgggcctgaaaatccatataacactccaatatttgccataaaa
aagaaggacagtactaagtggagaaaattagtagacttcagggaactcaataaaagaactcaagattttgggaa
gttcaattaggaataccacacccagcagggttaaaaaaaaaaaaatcagtgacagtactggatgtgggggatgca
tatttttcagttcctttatatgaagacttcaggaaatatactgcattcaccatacctagtataaacaatgaaaca
ccagggattaggtatcaatataatgtgcttccacagggatggaaaggatcaccagcaatattccagagtagcatg
acaagaatattagagccctttagggcacaaaatccagaaatagtcatctatcaatatatggatgacttatatgta
ggatctgacttagagatagggcaacatagagcaaaaatagaggagttaagagaacatctgttaaagtggggattt
accacaccagacaagaaacatcagaaagaacctccatttctttggatggggtatgaactccatcctgacaaatgg
acagtacagcctatacagctgccagaaaaggatagctggactgtcaatgatatacagaagttggtgggaaaatta
aactgggcaagtcagatttacccaggaattaaagtaaggcaactttgtagactccttaggggggctaaagcacta
acagacatagtaccactaactggagaagcagaattagagttggcagaaaacagggaaattctaaaggaaccagta
catggagtatattatgacccatcaaaagacttgatagctgaaatacagaaacaggggcaggaccaatggacatat
caaatttaccaagaaccattcaaaaatctgaaaacagggaagtatgcaaaaatgaggactgcccacactaatgat
gtaaaacagttaacagaggctgtgcagaaaatagccatggaaagcatagtaatatgggaaaagactcctaaattt
agattacccatccaaaaagaaacatgggagctatggtggacagactattggcaagccacctggattcctgagtgg
gaatttgttaatacccctcccctagtaaaattatggtatcggctggaaacagaacctatagcaggagcagaaact
ttctatgtagatggagcagctaatagggaaactaaaacaggaaaagcagggtatgttactgacagaggaagaaat
aaagttgtttctctaactgaaacaacaaatcagaagactgaattgcaagcaatttgtctagctttgcaagattca
ggatcagaagtaaacatagtaacagattcacagtatgcattagggatcattcaagcacaaccagataagagtgaa
tcagagttagttaaccaaataatagaacagttaataaagaaggaaagggtctacctgtcatgggtaccagcacat
aaaggaattggaggaaatgaacaagtagataaattagtaagtaatggaatcaggaaagtgctatttctagatgga
atagataaagctcaagaagagcatgaaaaatatcacagcaattggagagcaatggctagtgactttaatctrcca
cccatagtagcaaaagaaatagtagccagctgtgatcaatgtcagctaaaaggggaagccatgcatggacaagta
gactgtagtccagggatatggcaattagattgtacacatttagaaggaaaaatcatcctggtagcagtccatgta
gccagtggctacttagaagcagaggttattccagcagaaacaggacaagaaacagcatactatatactaaaatta
gcagcaagatggccagtcaaagcaatacatacagacaatggtagtaatttcaccagygctgcagttaaggcagcc
tgttggtgggcaggcatccaacaggaatttggaattccctacaatccccaaagtcagggagtagtagaatccatg
aataaagaattaaagaaaattatagggcagataagagatcaagctgagcaccttaagacagcagtacaaatggca
gtattcattcacaattttaaaagaaaaggggggattggggggtacagtgcaggggaaagaatagtagacatgata
gcaacagacatacaaactaaagaattacaaaaacaaattataaaaattcaaaattttcgggtttattacagagac
agcagagaccccatttggaaaggaccagccaaactactctggaaaggtgaaggggcagtagtgatacaagataat
aatgacataaaggtagtaccaaggaggaaagcaaaaataattaaagacacatggaatagtttagtaaaacaccat
atgtatatttcaaagagagctaagggatggttttacagacatcattatgacagcagacatccaaaagtaagttca
gaagtacacatccccttaggggatgctagactagtaataaaaacatattggggttgcaaacaggagaaagagat
tggcacttgggccatggagtatccatagaatggagattgagaggatatagtacacaagtagaacctggcctggca
gaccagctaattcatatgcattattttgattgttttgcagactctgccataagaaaagccatattaggacacata
gttattcctaggtgtgactatccagcaggacataataaggtaggatctctacaatacttggcactgacagcactg
ataaaaccaaaaaagataaggccacctctgcctagtattaggaaattagtagaggatcttctagaggaactcaag
caggaagctgtcagacactttcctagaccatggcttcatggcttaggacaatatgtctatgagacctatggggat
acttggacaggagttgaaactatggtgagactactgcaacaattactgtttattcatttcagaattgggtgccag
catagcagaataggcattttgcgacagagaagagcaagactagagccctggaaccatccaggaagtcagcctaaa
actgcttgcaataactgttattgtaaaaaatgtagctaccactgtctagtttgctttcagaaaaaaggcttaggc
atttccgtgggaggaaacttgtgggtcacagtttattatggggtacctgtgtggagagaagcaaaaactactcta
ttctgtgcatcagatgctaaagcatatgagaaagaagtgcataatgtctgggctacacatgcctgtgtacccaca
gaccccaacccacaagaaatagatttgaatgtaacagaaaattttaacatgtggaaaaatgacatggtggagcag
atgcatgaggatgtaatcagtttatgggatcaaagcctaaagccatgtgtaaagttgaccccaatttgtgtcact
ttagaatgtacagatgctaatattacctgcaatagtactactagcagtaataattgtaccagctatgagatcaac
aaggaggacatgggggaaataaaaaattgctctttcaatacaaccacagaattaatagataagcagaagaaagtg

catgcactctttatagacttgatatagtatcactagaaaggacaactctagtaagaagaacgactctaatgag
tattatagattaataaattgtaatacctcagccataacacaagcctgtccaaaggtcacttttgacccaattcct
atacactattgtgctccggctggttatgcgattctaaagtgtaaaaataagacattcaatgggacgggaccatgc
aataatgttagcacagtacaatgtacacatggaattaagccagtggtatcaactcaactactgttaaatggtagc
ctagcagaagaagagataataattagatctgaaaatctgacaaacaatgccaaaataataatagtacatcttaat
caagctgtagaaattgtatgtacaagacctggcaataatacaagaaaaagtataaggatagggccaggacaaaca
ttctatgcaacaggagacataataggagacataagacaagcacattgtaacattagtgaagctaagtggaataaa
accttgcgagaggtaagtaaaaaattagcagaacacttccctaataaaacaataatatttaactcatcctcagga
ggggacctagaaattacaacacatagctttaattgtggaggagaatttttctattgtaatacatcaagcctgttt
aatagtacatttaatagtacatacatgactaatgatacagacatgaattcaaactcaaccatctcaatcccatgc
cgcataaaacaaattataaacatgtggcaggaggtaggacgagcaatgtatgcccctcccattgcaggaaacata
acatgtaaatcaaatatcacaggactactattggtacgagatggaggaaactcaaatgatacaaatgagccagag
atattccgacctcaaggaggagatatgagggacaattggagaagtgaattatataaatataaagtggtagaaatt
aagccattgggagtagcacccactgcagcaaaaaggagagtggtgggaagagaaaaaagagggatttggggctgc
tctggaaaactcatctgcactactgctgtaccttggaactccagttggagtaacaaatctcaagaccagatttgg
aatgatacgacctggatgcagtgggataaagaaattagtaattacacagacataatatacacgttgcttgaaaaa
tcgcaaaaccagcaggaaataaatgaaaaggatttattagcattggacagttggaacagtctatggaattggttt
gacataacaaagtggctgtggtatataaaaatattcataatgatagtaggaggcttgataggtttaagaataatt
tttgctgtgctttctatagtgaatagagttaggcagggatactcacctttgtcgtttcagacctatctaggaagt
cttgtgcagtattggggtctggaactgaaaaagagtgctattagtctgtttgataccatagcaatagcagtagct
gaaggaacagataggcttatagaattcatacaaagaactggtagagctatctgcaacatacctagaagaataaga
cagggttttgaagcagctttgcaataaatgggggcaagtggtcaaaacgcagcatagttggatggcctaatata
agagagagaatgagacgagctgagccagcagcagaaggagtaggagcagcatctcaagacttagataaacatgga
gcgcttaccagcagcaacacagacaccaataatgctgattgtgcttggctgagagcgcaagaggaggaagaagat
gtaggctttccagtcagacctcaggtgcctttaagaccaatgacttttaagggagcatttgatctcagcttcttt
ttaaaagaaaaggggggactggaagggttaatttactctaagaaaaggcaagagatccttgatttgtgggtctat
cacacacaaggctacttccctgactggcaaaactacacaccaggaccaggggtcagatacccactgacctttggg
tggtgcttcaagctgctaccaattgacccaagggaagtagaagaggccaacaaagaagaagacaaccgcttgcta
caccctatgtgccagcatggaatggaggatgaacacagagaagtattaaagtggaagtttgacagtcaactagca
cttaaacacagggcccgcgagctacatccggagttttacaaagactgctga
>#85 KY658708 7443 nt
atgggtgcgagagcgtcaatattaagaggcgaaaaattagataaatgggaaaaaattaggttaaggccaggggga
aagaaacattatatgctaaaacatctagtatgggcaagcagggagctggaaggatttgcacttaaccctggcctt
ttagaaacagcagaaggctgtaaacaaataatgaaacagctacaaccagctcttcaaacaggaacagaggaactt
aggtcattatacaacacagtagcaactctctattgtgtacatgcagggatagaggtaagagacaccaaggaagcc
ttagacaagatagaggaagaacaaaacaaaagtcagcaaaaaacacagcaggcaaaagaggctgatgggaaggtc
agtcaaaattatcctatagtacagaatctacaagggcaaatggtacaccaggccatatcaccwagaactttgaat
gcatgggtaaaagtaatagaggagaaggcctttagcccagaagtaatacccatgtttacagcattatcagaagga
gccaccccacaagatctaaacaccatgttaaatacagtgggggacatcaagcagccatgcaaatgttaaaagat
accatcaatgaggaggctgcagaatgggatagattacatccagtacatgcagggcctgttgcaccaggccaaatg
agagacccaaggggaagtgacatagcaggaactactagtacccttcaggaacaaatagcatggatgacaagtaac
ccacctgttccagtgggagacatctataaaagatggataattctggggttaaataaaatagtaagaatgtatagt
cctgtcagcattttggacatcagacaagggccaaaggaaccctttagagactatgtagatcggttctttaaaact
ttaagagctgaacaagctacacaagatgtaaagaattggatgacagaaaccttgttggtccaaaatgcaaaccca
gattgtaagaccattttaagagcattaggaccaggggctacattagaagaaatgatgacagcatgtcagggagtg
ggaggacctggccacaaagcaagagtgttggctgaggcaatgagccaggcaaacagtgtaaacataatgatgcag
aaaagcaattttaaaggccctagaagaattgttaaatgtttcaactgtggcaaggaagggcatatagccaaaaat
tgcagggcccctaggaaaaaaggctgttggaaatgtggaaaagaaggacaccaaatgaaagactgtactgagaga
caggctgtaggggccagataaaggaggctctcttagacacaggagcagatgatacagtattagaagaaataaat
ttgccaggaaaatggaaaccaaaaatgataggaggaattggaggttttatcaaagtaagacagtatgatcaaata
gttatagaaatttgtggaaaaaaggctataggtacagtattagtaggacctacacctgtcaacataattggaaga
aatatgttgactcagcttggatgtacactaaattttccaattagtcccattgaaactgtaccagtaaaattaaag
ccaggaatggatggcccaaaggttaaacaatggccattgacagaagagaaaataaaagcattaacagaaatttgt
gaagaaatggagaaggaaggaaaaattacaaaaattgggcctgaaaatccatataacactccagtatttgccata
aaaaagaaggacagtactaagtggagaaaattagtagattttagggagcttaataaaagaactcaagacttttgg

gaagttcaattaggaataccacacccagcagggttaaaaaagaagaaatcagtgacagtactggatgtggggat
gcatattttcagttcctttagatgaaggcttcaggaaatatactgcattcaccatacctagtataaacaatgaa
acaccagggattagatatcaatataatgtgcttccacagggatggaaaggatcaccagcaatattccagagtagc
atgacaagaattttagagccctttagagcacaaaatccagaaatagtcatctatcaatatatggatgacttgtat
gtaggatctgacttagaaatagggcaacatagagcaaaaatagaagaattaagagaacatctgttaaagtggga
tttaccacaccagacaaaaaacatcagaaagaaccccatttctttggatggggtatgaactccatcctgacaaa
tggacagtacagcctataaagctgccagacaaggatagctggactgtcaatgatatacagaagttagtgggaaaa
ttaaactgggcaagtcagatttacccagggatcaaagtaaggcaactttgtaaactccttaggggggccaaagca
ctaacagatgtagtaccactaactgaagaagcagaattagaattggcagagaacagggaaattctaaaagaacca
gtacatggggtatattatgacccatcaaaagacttaatagctgaaatacagaaacaggggcatgaccaatggaca
tatcaaatttaccaagaaccattcaaaaatctgaaaacaggaaagtatgcaaaaatgaggactgcccacactaat
gatgtaaaacagttaacagaggcagtgcaaaaaatagcccaggaaagcatagtaatatggggaaagactcctaaa
tttagactacccatccaaaaggaaacgtgggagacatggtggacagactattggcaagccacctggattcctgaa
tgggagtttgttaatactcctcccctagtaaaattatggtaccagttggagaaagaacccataccaggagtagaa
actttctatgtagatggagcagctaatagggaaactaaactaggaaaagcagggtatgttactgacagaggaagg
cagaaaattgtttctctaactgaaacaacaaatcagaagactgaattgcaagcaattcagctagctttgcaagat
tcaggatcagaagtaaacatagtaacagactcacagtatgcattaggaatcattcaagcacaaccagataagagt
gaatcagagttagtcaaccaaataatagaacaattaatacaaaaggaaagggtctacctgtcatgggtacctgca
cataaaggaattggaggaaatgagcaagtagataaattagtaagtcagggaatcaggaaagtgctgtttctagat
ggaatagataaggctcaagaagagcatgaaaaatatcacaacaattggagagcaatggctgatgagtttaatctg
ccacccatagtagcaaaggaaatagtagctagctgtgataaatgtcaactaaaaggggaagccatacatggacaa
gtagactgtagtccagggatatggcaattagattgtacacatttagaaggaaaagttatcctggtagcagtccat
gtagccagtggctacatagaagcagaggttatcccagcagaaacaggacaagaaacagcatactacatactaaaa
ttagcaggaagatggccagtcagagtaatacatacagacaatggtactaatttcaccagtgctgcagttaaggca
gcctgttggtgggcaggtatccaacaggaatttggaattccctacaatccccaaagtcagggagtagtagaatcc
atgaataaagaattaaagaaaattatagggcaggtaagagatcaagctgagcaccttaagacagcagtacaaatg
gcagtattcattcacaattttaaaagaaaaggggggattggggggtacagtgcaggggaaagaataatagacata
atagcaacagacatacaaactaaagaactacaaaaacaaattataaaaattcaaaattttcgggtttattacaga
gacagcagagaccctatttggaaaggaccagccaaactactctggaaaggtgaaggggcggtagtaatacaagat
aacagtgacataaaggtagtaccaaggaggaaagtaaaaatcattaaggacacatggaatagtttagtaaagcac
catatgcatatttcaaaaagagctaagggatggttttacagacatcattatgaaagcagacatccaaaagtaagt
tcagaagtacacatcccattaggggaggctagattagtaataaaaacatattggggtttgcaaacaggagaaaga
gattggcatttgggtcatggagtctccatagaatggagattgagaaaatatagcacacaagtagaacctggcctg
gcagaccagctaatccatatgcactattttgattgttttgcagactctgccataagaaaagccatattaggagaa
atagttattcctaggtgtgactatcaagcaggacataatcaggtaggatctctacaatacttggcactgacagca
ttgataaaaccaaaacagagaaagccacctctgcctagtgttaggaaattagtagaggatcttctagaggaaatc
aagcaggaagctgtcagacactttcctagaccatggcttcatggcttaggacaatatgtctatgaaacctatggg
gatacttggacaggagtagaaactctaataagaatactgcaacaactactgtttattcatttcagaattgggtgc
caacatagcagaataggcattttgcgacagagaagagcaagaatggagccagtagatcctaacctagagccctgg
aaccatccaggaagtcagcctagaactgcttgtactaaatgctattgtaaatactgttgctaccattgcctagtt
tgctttcagacaaaaggcttaggcatttccagtgtggtgggaaacttgtgggtcacagtttattatggggtacct
gtgtggaaagatgcaaaaactactctattctgtgcatcagatgctaaagcatatgaaaaagaagtgcataatgtc
tgggctacacatgcctgtgtacccacagaccccaacccacaagaaatagttttggaaaatgtaacagaaaatttt
aacatgtggaaaaatgacatggtggatcagatgcatgaggatataatcagtttatgggaccaaagcctaaagcca
tgtgtaaagttgaccccactctgtgtcactttaaaatgtgtaaatgttagtaataaatttaacctacaaaatagc
atgaatgaagatccgaaggaagaaataagaaattgctctttcaatgcaaccacagaattaagagataagaaacag
aaagtgcatgcactcttttataaacttgatatagtaccacttcatggcaacaactctagtgagtatagattaata
aattgtaatacctcaaccataacacaagcctgtccaaaggtctcttttgatccaattcctatacattattgtact
ccagctggttatgcgattttaaagtgtaataatcagacattcaatgggacaggaccatgcaataatgtcagctca
gtacaatgtacacatggaattaggccagtagtatcaactcagttactgttaaatggtagcctagcaaaaggagag
ataataattagatctgaaaatctgacaaacaatgccaaaataataatagtacatcttaataaacctgtaaaaatt
gtgtgtgtaaggcccaacaataatacaagaaaaagtgtaaggataggaccaggacaaacattctatgcaacaggt
gaaataataggagacataagacaagcatattgtatcattaataaaactgaatggaataacactttacaaggggta
agtaaaaaattaggagaacacttctctaataaaacaataaaatttgaaccgtcatcaggaggggacctagaaatt
acaacacatagctttaattgtagaggagaatttttctattgcaacacatcacaactgtttaatagtacatacagt

cccagttttaatggtacagaaggtaaattaaatgggaccatcacaatcccatgtagaataaaacaaattataaac
atgtggcagaaagtaggacaagcaatgtatgcccctcccattgcaggaaacctaacatgtgaatcaaatatcaca
ggattactattgacacgtgatggaggaaaaacaggttcaaatcacccagagatattcagacctggaggaggggat
atgagggataactggagaagtgaattatataaatataaagtggtagaaattaagccattgggagtagcacccact
gaggcaaaaaggagagtggtggagagagaaaaaagagggatttggggctgctctggaaaactcatctgcaccact
gccgtgccttggaactccagttggagtaatagatctcatgatgagatttgggataacatgacctggatgcagtgg
gatagagaaattaataattacacagacacaatatacaggttgcttgaagaatcacaaaaccagcaggagaaaaat
gaaaaggatttattagcattggacagttggcaaaatctgtggaattggtttagcataacaaattggctgtggtat
ataaaaatattcataatgatagtaggaggcttgataggtttaagaataatttttgctgtgctttctatagtgaat
agagttaggcagggatactcacctctgtcgtttcagtatctgggaagccttgtgcagtattggggtctagagcta
aaaaagagtgctattagtctgtttgataccatagcaatagcagtagctgaaggaacagataggattatagaatta
atacaaagaatttgtagagctattcgcaacatacctagaagaataagacagggctttgaagcagctttgcaataa
atgggggcaagtggtcaaaatgcagtggatggcctgctgtaagagaaagaatgagaagaactgagccagcagca
gagggagtaggagcagcatctcaagacttagataaatatggagcacttacaagcagcaacacaagcaccaataat
gctgattgtgcctggctggaagcgcaagaggaggaaggagatgtaggctttccagtcagacctcaggtgccttta
agaccaatgacttataaaggagcattcgatctaggcttcttttaaaagaaaaggggggactggaagggttagtt
tactccaagaaaagacaggaaatccttgatttgtgggtctatcacacacaaggcttcttccctgactggcaaaac
tacacaccgggaccaggggtcagatatccactgacctttggatggtgcttcaagctggtgccagttgacccaggg
gaagtggaagaggccaacaagggagaagacaactgtttgctacaccctatgagccagcatggaatggaggatgaa
cacggagaagtattaaagtggaagtttgacagtcacctagcacgcagacacatggcccgcgagctgtatccggag
tattacaaagactgctga
>#86 KC156114 7422 nt
atgggtgcgagagcgtcagtattgaaaggcggaaaattagatgcatgggaaaaaattaggttaaggccaggggga
aagaaaaaatataggctaaaacacttagtatgggcaagcagggagctggaaaaatttgcacttaaccctggcctt
ttagaaacatcagaaggctgtaagcaaatactgacacagctacaaccagctcttcaaacaggaacagaggaactt
aaatcattattcaacacagtagcaactctctattgtgtacataaaaggatagaggtacatgacaccaaggaagcc
ttagacaaggtagaggaagaacaaaacaaaagtcagcaaaaaacacagcaggcagaagcggctgacaaggaaaa
gtcagtcagaattatcctatagtgcagaatctccaagggcaaatggtacaccaggccttatcaccaagaactttg
aatgcatgggtaaaagtaatagaggagaaagctttcagcccagaggtgatacccatgtttacagcattatcagaa
ggagccaccccacaagatttaaacaccatgctaaatacagtgggggacatcaagcagccatgcagatgttaaaa
gataccatcaatgaggaggctgcagaatgggataggatgcatccagtgcatgcagggcctgctgcaccaggccag
atgagagagccaaggggaagtgacatagcaggaaccactagtaaccttcaggaacaaatagcatggatgacaagt
aacccacctattccagtgggagacatctataaaagatggataattctggggttaaataaaatagtaagaatgtac
agccccgtcagcattttggacataaaacaaggaccaaaagaaccctttagagattatgtagatcggttctttaaa
accttaagagctgaacaatcctcacaagaggtaaaaaattggatgacagacaccttgttgatccaaaatgcgaac
ccagattgtaaaaccattttaaaagcattaggatcaggggcttcattagaagaaatgatgacagcatgtcaggga
gtgggaggacctggccacaaagcaagagttttggctgaggcaatgagccaagtaaacaataccagtataatgatg
cagaaaagcaactttaaaggctctagaaaaattgttaaatgtttcaactgtggcaaggaagggcatatagccaga
aattgcagggcccccaggaaaagaggctgttggaaatgtggaaaggaaggacaccaaatgaaagactgtactgag
aggcaggctgtaggggacagataaaggaggctctcttagacacaggagcagatgatacagtattagaagacata
aatttgccagggaaatggaaaccaagaatgataggaggaattggaggttttatcaaagtaagacaatatgaacaa
atacctatagaaatttgtgggaaaaaggctataggtacagtgttagtaggacctacgcctgttaacataattgga
agaaatatgttgacacagcttggatgtacactaaattttccaattagtcctattgaaactgtaccagtaaaatta
aagccagggatggatggcccaagggttaaacaatggccattgacagaagagaaaataaaagcattaacagcaatt
tgtgaagaaatggagaaggagggaaaaattacaaaaattgggcctgaaaatccatataacactccaatatttgcc
ataaagaagaaggacagtactaagtggagaaaattagtagatttcagggaactcaataaaagaactcaagatttt
tgggaggttcaattaggaataccacacccagcagggttaaaaaagaaaaaatcagtgacagtgctagatgtgggg
gatgcatatttttcagttcctttagatgaaagctttaggaaatatactgcattcaccatacctagtataaacaat
gcaacaccaggaattagatatcaatataatgtgctaccacagggatggaaaggatcaccagcaatattccagagt
agcatgacaaaaatcttagagcccttcaggacacaaaatccagatatagttatctatcaatatatggatgacttg
tatgtgggatctgacttagaaatagggcaacatagagcaaagatagagcaattaagagaacatttactgcaatgg
ggacttaccacaccagataaaaaacatcaaaaggaaccccccatttctttggatggggtatgaactccatcctgac
aaatggacagtacagcctatacagctaccagacaaggatagctggactgtcaatgatatacagaagttagtggga
aaattgaactgggcaagtcagatttacccagggattaaagtaaggcaactttgtaaactccttaggggaaccaaa
gcactaacggacatagtaccactaactgaagaagcagaattagaattggcagagaacagggaaattctaaaagaa

ccagtacatggggtatattatgacccatcaaaagacttgatagctgaaatacagaaacaagggcatgaccagtgg
acatatcaaatttaccaagaaccattcaaaaatctgaagacaggaaagtatgcaaaaatgaggactgcccacact
aatgatgtaaaacagctaacagaggcagtgcaaaaaatagcccaggaatgcatagtaatatggggaaagactcct
aaatttaggttacccattcaaaaagacatatgggaaacatggtggacagactattggcaagccacctggattcct
gaatgggaatttagtaatacccctcccctagtaaaattatggtatcagcttgagaaagatcccatagcaggagca
gaaactttctatgtagatggagcagctaatagggaaactaaactggggaaagcagggtatgtaactgataaagga
aggcagaaaatcattactctaactgaaacaacaaatcagaaggctgaattacaagcaattcagcttgccttgcag
gattcaggatcagaagtaaacatagtaacagactcacagtatgcattaggaatcattcaggcacaaccagataag
agtgaatcagagttagtcactcaaataatagagcagttaataggaaaggaaaggatctacctgtcatgggtacca
gcacataaaggaattggaggaaatgaacaagtagataaattagtaagtagtggaatcaggaaagtactgtttcta
gatggaatagacaaggcccaagaagagcatgaaaaatatcacagcaattggagagcaatggctagtgagtttaat
ctgccacccatagtagcaaaagaaatagtagctagctgtgataaatgtcaacaaaaaggggaagccatgcatggg
caagtagactgtagtccagggatctggcaattagattgtacacatttagaaggaaaaatcatcctggtagcagtc
catgtagccagtggctacatagaagcagaggttatcccagcagaaacaggacaagaaacagcatactacatacta
aaattagcaggaagatggccagtcaaggtaatacatacagacaatggcagtaatttcaccagtaatgcagttaag
gcagcctgttggtgggcaggtatccaacaggaatttggaattccctacaatccccaaagtcagggagtagtagag
tccatgaataaagaattaaagaaaatcataggacaggtaagagatcaagctgagcacctaaagacagcagtacaa
atggcagtattcattcacaattttaaaagaaaaggggggattggggggtacagtgcaggggagagaataatagac
atgatagcaacagacatacaaactaaagaattacaaaaacaaattataaaaattcaaaattttcgggtttattac
agagacagcagagaccctatttggaaaggaccagccaaactactctggaaaggtgaaggggcagtagtaatacaa
gataacagtgacataaaggtagtaccaaggaggaaagcaaaaatcattaaggacacatggaatagtttagtaaag
caccatatatgggcctcaaagaaagctagtggatggttttacagacatcattatgaaagcagacatccaaaagta
agttcagaagtacacatcccattaggggatgctagattaataataacaacatattggggtttgcaaacaggagaa
agagattggcatttgggtcatggagtctccatagaatggagattgaaaagatatagcacacaagtagaccctggc
ctggcagaccagctaattcatatgcattattttgattgttttgcagactctgccataagaaatgccatattagga
cacatagtttttcctaggtgtgactatcaagcaggacataataaggtaggatctctacaatatttggcactgata
gcattgataaaaccaaaaaggataaagcctcctctgcctagtgttaggaaattagtagaggatcttttagaggaa
ctcaagcaggaagctgtcagacactttcctagaccatggctccataacttaggacaacatatctatgaaacctat
ggggatacttggacaggagtcgaagtgataataagaattctgcaacaactactgtttatccatttcagaattggg
tgccagcatagcagaataggcatcttgagacagagaagagcaagactagatccctggaaccatccaggaagtcag
cctaagactgcttgtaataattgttattgtaaaggctgcagctatcattgtctagtttgctttcagaaaaaaggc
ttaggcatttccgggatggggcaattgtgggtcacagtctattatggggtacctgtatggaaggaagcaaaaact
actctattttgtgcatcagatgctaaagcatattcccaagaagcacataatatctgggctacacatgcctgcgta
cctacagaccccaacccacaagaaatggttttagaaaatgtaacagaaaattttaacatgtggaaaaatgacatg
gtagatcagatgcatgaggatataatcagtttatgggatcaaagcctaaagccatgtgtaaagttgaccccactc
tgtgtcactttaaactgtactactgttaatattacccaggaaaataatccccttaatgatagtgatagtaattac
atgaaaaactgctctttcaatatgaccacagaactaaaagataaaaagaaggaagagagggcattgtttcataga
cttgatctagtaccacttaatgagaatagtagcaagtatgagaatagtaacaagtatatattaacaaattgtagg
acctcaaccatagcacaggcctgtccaaagatctcgtttgatccgattcctatacattattgtgctccagctggt
tatgcgattctaaaatgtaataataaggcattcaatggatcaggaccatgcactaatgtcagcacagtacaatgt
acacatggaattaagccagtggtatcaactcagctactgttaaatggtagcctagcagaagagataataatcaga
tttgaaaatctgacaaacaatgtcaaaacaataatagtacaccttaatgaatctgtagaaatttcgtgtacaaga
cccaacaataatacaagaaaaagtgtgaggataggaccaggacaaacattctatgcaacaggagaaataatagga
gatataagacaagcatattgtaccattaatgaaggaaaatggaataaaactttagaaaggataaggaacaaatta
gcagagtacttccctaataaaacaatacaatttcaaagcgcctcaggaggggacctagaaattacaacacatagc
tttaattgtggaggagaatttttctattgcaacacatcaagattgtttaatcatacatttaatgagacatcagac
gaagacatcacactccaatgtaggataaaacaaattataaacatgtggcagggggtaggacgagcaatatatgcc
cctcccattgcaggaaacataacatgtaactcaaatatcacaggactactattgacacgtgatggagggaagagt
aatagtagtaatcagacagagatattcagacctgcaggaggaaatatgagggacaattggagaagtgaattatat
aaatataaagtggtagaaattaaaccattaggaatagcacccactaaggcgaaaaggagagtggtggagagagaa
aaaggaatttgggctgctctggaaaactcatctgcaccactaatgtgccttggaactctagttggagtaataaa
cctgaagaagaaatttgggggaacatgacctggatgcaatgggagagagaaattgataattatacagacacaata
tacagtttgcttgaaaaatcgcaaaaccagcaggaacaaaatgaacaagatttactagcattggacagttggaaa
aacctgtggaattggtttagcataacaaaatggttgtggtatataaaaatattcataatgatagtaggaggctta
ataggtttaagaataatttttgctgtgctttctatagtgaatagagttaggcaggatactcacctttatcgttc

cagtatctaggaagtcttgcgcagtattggggtctggaactaaaacaaagtgctattaacctgcttgataccata
gcaatagcagtagctgaaggaacagataggatcatagaagtaatacaaagaatttgtagagctatctgcaatata
cctacaagaataagacagggctttgaagcagctttgcaataaatgggggcaagtggtcaaaaagcagtatagtt
ggatggcctgctgtaagagaaagaataagacaagctaggccagagccagcagcagagggagtaggagcagcgtct
caagacctagataaacatggggcacttacaaccagcaacacagtttataacaatgctgcttgtgcctggatagaa
gcgcaagaggagggagaagaggtaggctttccagtcaaaccccaggtgcctttaagaccaatgacttataaagga
gcattcgatctcagcttctttttaaaagaaaaggggggactggatgggttaatttactcccagcaaagacaggat
atccttgatttgtgggtctataacacacaaggctacttccctgattggcaaaactacacaccaggaccagggatc
agatatccactgacctttggatggtgctacaagttagtgccagttgacccaaggacagtagaagaggccaacagt
ggagaaaacagctgcttgctccaccctatgagccagcatggaatggaggatgcagacagagaagtattaatatgg
aagtttgacagcagcctagcacgcagacacctggcccgcgagctacatccggagtattacaaagattgctga
>#87 AF005496 7386 nt
atgggtgcgagagcgtcagtattaagcggcggaaaattagatgcttgggagaaaattcggctaaggccaggggga
aagaaaaaatataggctaaaacatctagtatgggcaagcagggagctggaaagatttgcacttaaccccggcctt
ttagaaacaccagaaggctgtctacagataatagaacagatacagccagctattaagacaggaacagaagaactt
aaatcattatttaatctagtagcagtcctctattgcgtacatcgaaaaatagatgtgaaagacaccaaggaggct
ttagataagatagaggaaatacaaaacaaaagtcagcaaaaaacacagcaagcagcagctgataaggaaaaagac
aacaaggtcagtcaaaattatcctatagtacagaatgctcaagggcagatggtacaccaggccatatcacctagg
accttaaatgcatgggtaaaagtagtagaagaaaaggcttttagcccagaagtaatacccatgttttcagcatta
tcagaaggagccaccccacaagacttaaatgctatgctaaatacagtgggggacatcaagcagccatgcagatg
ttaaaagatacaatcaatgaggaagctgcagaatgggacagggtacatccagtgcatgcagggcctattccacca
ggccaaatgagagaaccaaggggaagcgatatagcaggaactactagtaccctgcaggaacaaatagcatggatg
acaggcaatccagctatcccagtgggagacatctataaaagatggataatcctgggattaaataagatagtaaga
atgtatagtcctgtcagcattctggacataaaacaagggccaaaagaaccctttagagactatgtagacaggttt
tttaaaactttaagagctgagcaagccacacaggatgtgaagaattggatgacagaaaccttgttggtccaaaat
gcaaatccagattgcaagactatattaagagcattaggacaaggggcttcaatagaagaaatgatgacagcatgt
cagggagtgggaggacctagtcataaagcaagagttttggctgaggcaatgagccaagtaacaaatacaaataca
gccataatgatgcagaaaggcaactttaagggccaaagaaaatttgttaaatgcttcaactgtggcaaagaggga
cacatagccagaaattgcagggcccctaggaaaaagggctgttggaaatgtggaagagaaggacatcagatgaaa
gactgcacagagagacaggctatagagggacagttaaggaagctctattagatacaggagcagatgatacagta
ttagaagagataaatttgccgggaaaatggaaaccaaaaatgataggggggaattggaggttttatcaaagtaaga
cagtatgagcaagtagccatagaaatctgtggaaaaaaggctataggtacagtattagtaggacctacacctgtc
aatataattggaaggaatatattgactcaaattggttgcaccttaaattttccaattagtcctattgaaactgta
ccagtaaaattaaagccaggaatggatggcccaaaggttaaacaatggccattgacagaagaaaaaataaaagca
ttaacggaaatttgtacagagatggaaaaagaaggaaaaatctcaagaatagggcctgagaatccatacagcact
ccaatatttgccataaaaaagaaggatagtactaaatggagaaaattagtggatttcagagaactcaataaaaga
actcaagacttctgggaagttcagttaggaataccacacccagcagggttaaaaaagaaaaaatcagtatcagta
ctggatgtgggggatgcatatttttcagtccctttagataaagaattcagaaagtatactgcattcaccatacct
agtataaacaatgagacaccagggattagatatcagtataatgtgcttccacagggatggaaaggatcaccagca
atattccagagtagcatgacaaaaatcttagcgccctttagagaacaaaatcctgaaatggttatttaccaatac
atggatgatttgtatgtaggatctgacttagaaatagggcaacatagagcaaaaatagaggagttaagagctcat
ttgttgaaatggggatttaccacaccagacaaaaaacatcagaaagaaccccccatttctttggatgggatatgaa
ctccatcctgacaaatggacagtacagactgtaaaactgccagaaaaagacagctggactgtcaatgatatacag
aagttagtgggaaaactaaattgggcaagtcagatttatccaaatattaaagtaaagcaactatgtaaactcctt
aggggggccaaagcattaacagacataataccactgacaaaagaggcagaattggaattggcagaaaacagggag
attctgagagaaccaatacatggagtatattatgatccatcaaaagacttaatagcagaaatacggaagcaaggg
caaggccaatggacatatcaaatttatcaggagccatttaaaaatctgaagacaggaaaatatgcaaaaatgaga
actgcccacactaatgatataaaacaattaacagaagcagtgcaaaagatatctacagaaagcatagtaatatgg
ggaaaaattcctaaatttagactacctatacaaaaagaaacatgggagacctggtggacagagtattggcaagcc
acatggattcctgaatgggagtttgttaacacccctcatctagtaaaattatggtatcagttagaaacagagccc
atagcaggagcagaaacttactatatagatggggcagctaatagggaaactaaattaggaaaagcaggatatgtc
actgatagaggaaagcaaaaagttgtctccctaacggaaacaacaaatcagaagactgaattacaagcaatttat
ctagctttgcaagattcagggttagaagtgaacatagtgacagattcacagtatgcactaggaatcattcaagca
caacccgataagagtgaatcagagttagttaatcaaataatagaggaattaataaagaaggaaaaggtctacctg
tcatgggtaccagcacacaaaggaattggaggaaatgaacaagtagataaattagttagttctggagtcagaaaa

gtgctatttctagatgggatagataaagctcaagaagaacatgaaaggtatcataacaattggagagcagtggct
agtgattttaatctaccacctatagtagcaaaagaaatagtagctagctgtgataaatgtcagctaaaggggaa
gccatgcatggacaagtagactgtagcccaggaatatggcaattagattgcacacatttggaaggacaagttatt
ctggtagcagtccatgtagccagtggctatatagaagcagaagtcatcccagcagaaacaggaaaggaaacagca
tacttcctgttgaaactagcaagcagatggccagtaaaagtaatacatacagacaatggcagcaatttcacgagt
gctgcggttaaggcagcctgttggtgggcagatatccaacaggaatttgggattccctacaatccccaaagtcag
ggagtagtagaatctatgaataaagaattaaagaagatcatagggcaggtaagagaccaagcagaacaccttaag
acagcagtacaaatggcagtattcattcacaattttaaaagaaaaggggggattggggggtacagtgcaggggaa
agaataatagacataatagcaacagacatacaaactaaagaattacaaaaacaaatttcaaacattcaaaattt
cgggtttattacagggacagcagagacccaatttggaaaggaccagcaaaactcctctggaaaggtgaaggggca
gtagtaatacaagacaatagtgaaataaaagtagtaccaagaagagaggcaaaaatcattagggatacatggaaa
agcttagtaaagtaccatatgcatatttcaaggaaagctagaggatggttttatagacatcattttgaaagcact
catccaaggataagttcagaagtacacatcccattaggagaagctaggttagtcataaccacatactggggtctg
aatacaggagaaagagaatggcatttaggccagggagtctccatagaatggagactgaaaaggtatagcacacaa
gtagagcctggcctggcagaccaactaattcatatgcattattttgattgtttttcagaatctgccataaggaaa
gccatattaggacgtgtagttagacctaggtgtaactatccagcaggacataaacaggtaggaactctacaatac
ttggcattaacagcattagtggcaccaaaaaagataaagccacctttgcctagtgttagaaagctagtagaggat
cttttagaggagattaagaatgaggctgttaggcattttcctagagtatggctccatcaattaggacagcatatc
tataacacctatggagatacttgggtaggagttgaagctttaataagaacgctgcaacaactactgtttattcat
ttcagaattgggtgccaacatagcagaataggaattactcgacagagaagagtaagactagagccctggaaccat
ccaggaagtcagcctcaaactgcttgtaacaattgttattgtaaaaagtgctgctatcattgccaaatgtgcttt
ttaaagaaaggcttaggaatttccgctgcacaaaacttgtgggttacagtatattatggggtacctgtgtggaaa
gaggcaaaaaccactctattctgtgcatcagatgctaaggcatatgagacagaaaagcataatgtctgggctaca
catgcatgtgtacccacagaccccaacccacaagagatggtcatggagaatgtaacagagagctttaatatgtgg
gaaaataacatggtggagcagatgcatacagacataatcagtttatgggatcaaagcttgaaaccatgtgtaaaa
ttaaccccactctgtgttactctaaactgtactaatgtcagaaacaatacctctaacagcactagcagtatggag
gcaggaggggaactaacaaattgctctttcaatgtaactacagtactaagagataagcagcagaaagtacatgca
ctcttttatagacttgatgtagtaccaattgataacaatagtactcagtataggctaataaattgtaatacctca
gtcattacacaggcttgcccaaaggtgtcctttgaacctattcccatacattattgtgctccagctggctttgcg
attctaaagtgtaacaataaaacattcaatggaacaggattatgtacaaatgtcagtacagtacaatgtacacat
ggaattagaccagtggtatcaactcaactgctattaaatggaagcctagcagaagaacagatcataattagaact
aaaaatatctcagacaataccaaaaacataatagtacagcttaagacaccagtaaacattacatgtaccaggcct
aacaataatacgagaacaagtatacatttagggccaggacgagcattctatgcaacaggtgacatcataggagat
ataagacaagcacattgtaatattagtagaacagactggaataagactttacaccaggtagttacacaattagga
atacacttgaacaatagaacaataagctttaagccaaactcaggaggggacatggaagttagaacacatagtttt
aattgtagaggagaatttttctattgcaatacatcagggctgtttaatagtagttgggaaatgcatactaattac
acatcaaatgacacaaagggaaacgaaaacattacactgccatgcagaataaaacaaattgtaaacatgtggcag
agagtaggacgagcaatgtatgcccctcccatccaaggaaacattatgtgtgtatcaaatattacaggactaata
ttgacaattgacgagggtaacgcgtctgcagaaaattataccttcagacctggaggaggagatatgagggacaat
tggagaagtgaattgtataaatataaagtagtaaaaattgaaccactgggaatagcacccaccaagacaaggaga
agagtggtggagagagaaaaaagagggatttggggctgctctggaaaactcatctgcaccactaatgtgccttgg
aactctagttggagtaataaatcacagagtgaaatctgggacaacatgacttggatggaatgggataaacaaatt
agcaattacacagaggaaatatacaggttgcttgaagtctcgcaaacccagcaggaaaagaatgaacaggactta
ttagcattggacaaatgggcaagtctgtggacttggtttgacatatcacattggctgtggtatataaaaatattc
ataatgatagtaggaggtttaataggtttaagaataatttttgctgtgctttctatagtaaatagagttaggcag
ggatactcacctttgtcttttcaggctattgatttgcttaacaccacagcaatagcagtagctgagggaacagat
gggattatagtaatagtgcaaagagcttggagagctattctccacatacctagaagaataagacagggctttgaa
agaagcttgctataaatgggaggcaaatggtcaaaaagtaggatgggtgggtggtctactataagggaaagaatg
aggcgagctgaaccagtagcagaaggggtaggagcagtgtctcgagatttggatagacgcggggcagtcacaatt
aataatacagcatctactaatcgtgatgccgcctggctggaagcacaagaggacggggaggaagtaggctttcca
gtcaggcctcaggtacctttaagaccaatgacctataagggagcttttgatctcagccatttttaaaagaaaag
gggggactggatgggttaatttactccaagcaaagacaggacatccttgatttatgggtctataacacacaaggc
tacttccctgactggcagaactacacaccagggccaggggagagatttcccctgacctttggtggtgcttcaag
ctagtaccagtaaatccacaggaggtagaacaggccaatgaaggagagaacaacagcttgctacaccccatgagc

ctgcatggaatggaggatgacgggagagaagtgctgatgtggaaatttgacagtcgactagcattgacacacttg
gcccgagtaaagcatccggagtacaaagactgctga
>#88 FJ389367 7413 nt
atgggtgcgagagcgtcagtattaagtggggaaaattagatgcatgggaaaaaattcggttgaggccaggggga
aagaaaaaatatagaataaaacatctagtatgggcaagcagggagctggaaagatttgcactcaaccctggcctt
ttagaaacaacagaaggttgtcaacaaatattggaacagttgcaaccaactcttaggacaggaacagaggagatt
aaatcattatataatgcartagcaactctctattgtgtacatcaaaggatagaggtaaaagacaccaaagaagct
ctagaagaagtggaaaagatacaaaagaaaagtcagaaaaagacacagcaggcagcaatgggtgaaggaaacagc
agccaagtcagccaaaattatcctatagtgcaaaatgcacaagggcaaatggtacatcagcccctatcacctaga
actttaaatgcatgggtaaaagtagtagaagagaaggcattcaatccagaagtaatacccatgttttcagcacta
tcagaaggagccaccccacaagatttaaataccatgctaaacacagtgggggacatcaagcagctatgcaaatg
ctaaaagatactattaatgaggaagctgcagagtgggacaggacacatccaccacaggcagggcctatcccacca
ggccagataagggaaccaaggggaagtgatatagcaggaactactagtaacctgcaggaacaaataagatggatg
accagcaatccacctatcccagtgggagaaatytataaaagatggataatcctgggattaaataagatagtaaga
atgtatagccctgtcagcattttggatataagacaagggccaaaagarccttttagagattatgtagataggttc
tttaaaactttaagagctgaacaagctacacaggaagtaaaaaactggatgacagacaccttgttggtccaacat
gcaaacccagattgtaagaccatcttaagagcattagggccaggagctacactagaagaaatgatgacagcatgt
cagggagtgggaggacccggccataaagcaagggttttagctgaagcaatgagccaggcatcaaattcagcagca
gctataatgatgcaaaaaggcaattttaagggcccaagaagaattaagtgtttcaactgtggcaaggaaggacat
ctagccagaaattgcagggcccctaggaaaaagggctgttggaaatgtggaaaagagggacatcaaatgaaagac
tgcacagaaagacaggctacagtaagagtaggggacagctaatagaagccctattagacacaggagcagatgat
acagtattagaagatataaatttacaaggaaaatggaaaccaaaaatgataggggaattggtggttttattaaa
gtaaaacagtatgatcaaatacttatggaaatttgtggaaaaaaggctatagggacagtgttagtaggacctaca
cctatcaacataattggaagaaatatgttgactcagattggttgtactttaaattttccaattagtcctattgaa
actgtaccagtaaaattaaagccaggaatggatggcccaaaggttaaacaatggccattgacagaagagaaaata
aaagcattaacagaaatttgtacagaaatggaaaaagaaggaaaaatttcaaaaattgggcctgaaaatccatac
aacactccaatatttgccataaagaaaaaagacagtaccaagtggagaaaattggtagatttcagagagctcaat
aaaagaactcaagacttctgggaggtccaattaggaatacctcatcccgcggggttaaaaaagaaaaaatcagta
acagtactagatgtgggggatgcatatttttcagtgcccttatatgaagactttagaaagtatactgcattcacc
atacctagtgtaaataatgagacaccagggattagatatcagtacaatgtgcttccacagggatggaaaggatca
ccagcaatatttcagartagcatgacaaaaatcttagagccctttagaataaaaaatccagaaatagaaatctac
caatatatggatgatttatatgtaggatctgacttagaaatagggcagcatagarcaaaaatagaggagttaaga
gaacatctgttaaaatggggtttaccacaccagataaaaaacatcagaaagaacctccattcctttggatggga
tatgagctccatcctgacaaatggacagtacaacctatacagctgccaaataaagaaagctggactgtcaatgat
atacaaaagttagtgggaaaactaaattgggcaagtcagatttatccaggaatyaaagtaaaacaactatgtaaa
ctccttagggggccaaagcactaacagacatagtaccactgactgcagaagcagaattggaactggcagagaac
agggaaattctgaaagaacctgtacatggagtctattatgacccatcaaaagacytaatagcagaagtacagaaa
caagggttagaccaatggacatatcaaatctatcaggagccatacaaaaatctgaaaacaggaaaatatgcaaaa
aggggtctactcacactaatgatgtaaaacaattaacagaagtagtgcaaaaaatagccacagagagcatagta
atatggggaaagactcctaaatttaaactacccatacgaaaagaaacatgggaagtatggtggacagaatattgg
caggccacctggattcctgactgggagtttatcaatactcctcctctagtaaaattatggtatcagttagaaaca
gaacccatmgcaggagtagaaacttattatgtagatggggcagctaatagggaaacaaagttaggaaaggcagga
tatgtcactgacagaggaaaacaaaaaattattaccctaactggaacaacaaaccaaaagactgaattacaagca
attcagctagctttgcaggactcaggatcagaagtaaacatagtaacagactcacagtatgcattaggaattatt
caagcacaaccagataggagtgaatcagaattagtcaatcaaataatagagcagctaataaaaaaggaaagggtc
tacctgtcatgggtaccagcacacaaagggattggaggaaatgaacaagtagataaattagtyagtaatggaatc
aggaaagtgttattttagatggcatagataaagcccaagaagagcatgaaagatatcacagcaattggagagca
atggctagtgattttaatctgccacctatagtagcaaaagaaatagtggccagctgtgataaatgtcagctaaaa
ggggaagccatgcatggccaagtagactgtagtccaggaatatggcaattagattgtactcatttagaaggaaaa
attatcctggtagcagtccatgtggccagtggctatatagaagcagaagttatcccagcagaaacaggacaggaa
acagcattcttcatattaaaattggcaggcaggtggccagtgaaaatgatacacacagacaatggcagcaatttc
accagtgctgcagtaaaggcagcatgttggtgggcagatatcacacaagaatttggaattccctacaatccccaa
agccaaggggtagtggagtctatgaataaagaattaaagaaaatcattgggcaggtcagggatcaagctgaacac
cttaagacagcagtacagatggcagtattcattcacaattttaaaagaaaggggggattgggggggtacagtgca
ggggaaagaataatagacataatagcatcagacatacaaactaaagaactacaaaaacaaattacaaaagttcaa

aattttcgggtttattacagggacagcagagacccaatttggaaaggaccagcaaaactactctggaaaggtgaa
ggggcagtagtaatacaagacaataacgaaataaaggtagtaccaaggagaaaagcaaagatcattagggataca
tggaacagyttagtraaacatcatatgtatgtctcaaagaaagctaaaggctggttttatagacatcactatgaa
agcaggcatccaaaagtaagttcagaagtacacatcccaataggagatgctaaaatagtagtaagaacatattgg
ggtctgcatacaggagaaaaagattggcacctaggtaatggggtctccatagaatggaggcagagaagatatagc
acacagatagatcctgacctagcagaccaactgattcatctacattattttaactgtttttcagactctgccata
aggaaagccatattaggagaaatagttagacctaggtgtgaatatcaagcaggacataataaggtaggatcccta
caatatttggcactgaaagcattagtaacctcaccaagggcgaagccacctttgcctagtgttaggaaattaaca
gaagatctgttagaagaacttaaggctgaagctgttagacattttcctaggccctggcttcatggcttaggacag
catatctataacacttatggggatacttgggaaggagttgaagccataataagaatactgcaacaactattgttt
gtccatttcagaattgggtgccaacatagcagaataggcattgttccacaaagacaaagaagagtaaggctagag
ccctggaatcatccggggagtcagcctaaaactgcttgtaccaaatgctattgtaaagcgtgttgctggcattgc
caagtttgctttctgaacaaaggcttaggcatctccgcctcaaataccttgtgggtcacagtctattatggagta
cctgtgtgggaagatgcagataccaccctatttgtgcatctgatgctaaatcatatagtactgaaggccataat
gtctgggctacacatgcctgtgtacccacagaccccaacccacaagagatatctctgataaatgtaacagaaaat
tttaacatgtggaaaaataacatggtagaacagatgcatgaggatataatcagtttatgggacgaaagcctaaag
ccatgtgtacagctaacccctctctgtgttactttaaactgtgtgccagcaaatggcactaacataactggctct
ggcactgtgaatataaccgaacagatgaaaaactgctcgttcaatataaccacagaaataagggataggaagaag
cargaatacgcgctttttataaacttgatatagtaccaatagaggataatagtaatagtaataataatagttat
aggatgataaattgtaatgtctcaaccattaaacaagcttgtccaaagatgtcctttgacccaattcccatacat
tattgtgctccagctggttttgcgattttaaagtgtagggacgaggagttcaatggaacaggaacatgtaaaaat
gtcagtacagtacaatgtacacatggaattaagccagtggtatcaactcaattactgctgaatggcagtttggca
gaaggagacataatgattagatctaaaaacatcacagacaatgccaaaatcataatagtgcagcttaataaatct
atagaaattaattgtaccagacctggcaataatacaagaaaaagtgtaagaatcggaccaggacaagtgttctat
acaacaggtgaaataataggagatataagacaagcatactgtaatattgcaaaaaaagactgggataatatgcta
agggaagtggctataaaactaaggaaaagccttaacagcaacaagaccataacctttaactcatctgcaggaggg
gacctagaaattgcaacacatagttttaattgtagaggagaatttttctattgtaatacatcaggcctgtttaat
atatcatataatagtacagagaatagtacttataatgaagggaatgagacttatacactcccatgtaaaataaaa
caaattgtgagaatgtggcaaagagtgggacaagcaatgtatgcccctcccatcaaaggaaacattacatgtaga
tcaaacattacaggactactattaacaagagatggtggggagaatgataataataacagtagtgaggtcttcaga
cctgcaggaggagacatgagggacaattggagaagtgaattatataagtataaaatagtaaaaataaaaccatta
ggagtagcacccaccaaggcaaggagaagagtggtggggagagaaaaaagagcagggatctggggctgctctgga
aaactcatctgcaccactaatgtgccctggaatgctagttggagtaataaatcttatgaggccatttgggagaac
atgacctggatagaatgggaaagggaaattaacaattatactcaacaaatatacagcctaattgaagaatcgcag
aaccagcaggaaaagaatgaacaagacttattagcattagaccaatgggcaagtttgtggaattggtttgacata
tcaagatggctatggtatataaaaatatttataatgataataggaggtttaataggtttaagaatagtttttgct
gtgcttgctataataaatagagttaggcagggatactcacccttgttgttccaggctattaatttgcttgatacg
gtagcaatagcagtagctaattggacagatagggttatagaagtagtacaaagagcttgtagggcttttctcaac
atacctagaaggataagacaaggcgtagaaagagcattgccataaatgggaggcaagtggtcaaaaggatgcatg
gccggatggcctgaggtaagggaaagaatgagacaaaccactccagcagaaggagtaggagcagcatctcaagat
ttagctaggcatggagcgatcacaagtagcaatacaccatccactaatgctgcttgtgcctggctagaagcacaa
gaggaagactcagaggtaggctttccagtcagaccacaggtacctctgagaccaatgacttttaagggcgctttt
gatctcagcttcttttaaaagaaaaggggggactggatgggctaatttactccaagaaaagacaagatatcctt
gacctgtgggtctataatacacaaggattcttcccagattggcaggactacacaccagggccagggactagactc
ccactgacctttgggtggtgcttcaaactagtaccactggaaccaacagagatagaggaagccaataaaggagag
aacaacagtttattacaccccatctgccagcatggaatggaggacgaggacagagaagtgctagtatggagattt
gacagtagcctagcacggagacacttggcccgagagctgcatccggacttctacaaagactga
>#89 FJ389365 7344 nt
attcggctgaggccmgggggaargaaammrtayaractaaaacatwtagtatgggcaagcagggagctggayaga
tttgcacttaaccccggcctcttagaaacaggagaaggwtgtcagcagatamtgrmamagttgsaaccakctctc
magacaggaacagaggarattaaatcattatataatacagtagcaacyctctattgtgtacatcacaggatagar
ataaragacaccaaagaagctctagatgtartrgaaaaamtacaaaagaamagtcagcaagaaacacagcaggca
gcaaggagtacaggaaataacagccaagttagccaaaactatcctatagtgcagaatgcacaagggcaaatggta
caycaggccatatcaccyaggactttaaatgcatgggtaaaagtaatagaagaaaaggccttcagcccagaagta
atacccatgtttacagcattatcagaaggagccaccccacaagatttgaataccatgctaaacaccgtgggggg

catcaagcagctatgcagatgctaaaggatactattaatgaagaagctgcagagtgggataggatgcatccacca
caggcagggcctcttccaccaggccagmtaagagaacccaggggaagtgatatagcaggaactaccagtaccctg
caggaacaaataacatggatgaccagcaacccacctatyccagtgggagaactatataaaagatggataatcctg
gggttaaataaaatagtaagaatgtatagccctgtcagcattttggacataaaacaagggccaaaagaaccyttc
agagattatgtagatagrtttttaaaactttgagagctgarcaagccacccaggatgtaaaaaattggatgaca
gacaccttgttggtccaaaatgcgaatccagattgtaaaaccatcttaagagcattaggaccaggagctacatta
gaagaaatgatgacagcatgtcaaggagtgggaggacccagccacaaagcaagagtgttagctgaggcaatgagc
caggcaacargtgcagcagcaaacrtaatgatgcagaaaagcaattttaagggcccgagaagaaatattaagtgt
ttcaactgtggcagggaaggacatctagccagaaattgcagggcccctaggaaaaagggctgttggaaatgtgga
caggagggacatcaaatgaaagactgcacagaragacaggctgtgggggacagctaatagaagccctrttagac
acaggrgcagatgatacagtattagaacaaataaatttaccaggaaaatggaagccaaaaatgataggggaatt
ggaggatttatcaaagtaaaacagtatgatgatatacatatagaaattgarggraaaaaggctatagggacagta
ttagtaggacctacacctgtcaacataattggaagaaatatgttgactcagattggytgtactttaaattttcca
attagtcctattgaaactgtaccagtaaaattaaagccaggaatggatggcccaagggttaaacaatggccattg
acagaagagaaaataaaagcattaacagaaatttgtaatgaaatggaaaaggaaggaaaaatttcaaagattggg
cctgaaaatccatacaacactccaatatttgccataargaaaaargacagtactaaatggagaaagttggtagat
ttcagagagctcaataaaagaactcaagatttctgggaagtccaaytaggtatacctcatccckcggggttaaaa
aagaaraaatcagtaacagtactggatgtgggagatgcatatttttcagttcccytacatgaagactttagaaag
tatactgcattcactatacccagtayaaataatgagacaccagggaytagatatcagtayaatgtgctyccmcag
ggatggaaaggatcaccagcaatatttcagagtagtatgaccargatcttagagccctttaggayaaraaatcca
gaactggtgatctaycaatacatggatgatttatatgtaggatctgacttagaaatagggcagcatagagcaaaa
atagaggagttaagarakcatctrytgarrtggggattyaccacaccagataaraaacatcagaaagaacctcca
ttcctttggatgggataygagctccatcctgacaaatggacggtacaacctatacagctgccagacaaggaaagc
tggactgtcaatgatatacaraaattagtgggaaaactaaattgggcaagycagatytatcaagggattaaagta
aggcagctatgtaaactccttagggggccaaagcactaacagacatagtaccactgactgargaagcagaatta
gaattggcagagaacagggagattctaaaagaacctgtacatggagtctactatgacccatcaaaagatttaata
gcagaagtacagaaacaagggctygaccaatggacatatcaaatttatcaagagccatataaaaatctgaaaaca
ggaaagtatgcaaaaaggggtctgcccacactaatgatgtaagacaattaacagaggcagtgcaaaaaatagcc
acagaragcatagtaatatggggaaaagttcctaaatttaaactacctataaggaaagagacatgggaattatgg
tggacagaatattggcaggccacctggattcctgattgggagtttgtcaatacycctcctctagtaaaattrtgg
taycagttagagacagarcccatakyaggagcagaaacttactatgtagatggggcagctaatagggaaacaaaa
ttaggaaragcaggatatgttactgacaaaggaaagcaaaargttatyaccatacaggaaacaacaaaccaaaag
gctgaattacatgcaattcagctagccttacargactcagggtcagaggtaaacatagtaacagaytcacaatat
gcattaggaatcattcaagcacaaccagatagragtgaatcagaattagtcaatcaaataatagarcagctrata
aamaaggaaaggrtctacttarcatgggtaccagcacacaaaggattggrggaaatgaacaagtagataaatta
gtcagtagtggaatcagaaaagtactatttttagatggcatagataaagcccaagaagaacatgaaagatatcac
agcaattggaaagcaatggctagtgattttaatctgccaccggtagtagcaaaagagatagtggccagctgtgat
aaatgtcagctaaaaggagaagccatgcatggacaggtagactgtagtccaggaatatggcaattagattgtaca
catttagaaggaaagattatcctggtagcagtccatgtagccagtggctatatagaagcagaggttatcccagca
gaaacaggacaggaaacagcatactttatattaaaattagcaggaaggtggccagtaaaagtaatacatacagac
aatggcagcaattttaccagtaatgcagtaaaggcagcttgttggtgggcaagtatcacacaagaatttggaatt
ccctacaatccccaaagccaaggagtagtggaatctatgaataaagaattaaagaaaattattgggcaggtcaga
gatcaagctgaacatcttaagacagcagtacagatggcagtattcattcacaattttaaaagaaaaggggggatt
ggggggtacagtgcaggggaaaggataatagacataatagcatcagatctacaaactaaagaactacaaaaacaa
attacaaaaattcaaaattttcgggtttattacagggacagcagagacccaatttggaaaggaccagcaaaacta
ctctggaaaggtgaaggggcggtagtaatacaagacaataacgaaataaaagtagtaccaagaagaaaagcaaag
atcattagggatrcatggaacagtttartaaaacatcatatryatatctcaaagaargctaaagrytggtcttat
agacaccactatgaaagcaggcatccaagagtaagttcagaagtacacattccactaggagaagctaaaatagta
gtaagaacatattggggtctgcatacaggagaaaaagactggcarttgggtcatggggtctccatagaatggagg
cagggaaggtatagcacacaaatagatcctgacctagcagaccaaytgattcatctgcagtattttgattgtttt
acagactctgcmataagacaagccatattaggacaaagagttagacctaggtgtgaatatcaagcaggacataat
aaggtaggatctytacaatatytggcactgcaagcattagtaaaaycaaraaagagaaggccacctttgcctagt
gttaggaaattaacagaagatgaacttaaaaatgaagctgttagacattttcctagaccctggctccatggctta
ggacaatatatctataacacttatggagatacttgggaaggagttgaagccataataagaatgctacaacacttg
ctgtttatccatttcagaattgggtgccaacatagcagaataggcattagtcctctacggagaagagtaaggcta

gagccctggaatcatccrgggagtcagcctaaaactgcttgtaacaactgctattgtaaagtgtgctgctggcat
tgtcaagtttgctttctgaacaaaggcttaggcatctccgcttcctcagacttgtgggtcacagtctattatggg
gtaccagtgtgggaagatgcagatactcctctattttgtgcatctgatgctcaagcacatagtactgaaagccat
aatgtctgggccacacatgcctgtgtacccacagatcccaacccacaagaaatgcttctgaaaaacgtaacagaa
ccttttaacatgtggaaaaataacatggtagaacagatgaatgatgatataatcagtttatgggatgaaagccta
aagccatgtgtacagctaacccctctctgtgttactttaaactgtgctgatgtttgtcttaagaacagcactgct
actaatgattgtcctaagaacagcactgggaataacactgtgagtaatagtattacaaaagaaatgacaaactgc
tctttcaatataaccacagaaataaaagataagcagaagaaagaatacgcgctttttataaacttgatatggtg
caactggatggtagaaatgactcttatgggttaataaactgtaatgtctcagccattaaacaagcttgtccaaag
gtgtcttttgacccaattcccatacattattgtgctccagctgggtttgcgattttaaagtgcaaggataagaat
ttcaatggaacaggaacatgtaaaaatgttagttcagtacaatgcacacatggaattaagccagtggtatcaact
caattactactgaatggtagtctagcagaagaagaaatagtaattagaactgaaaacatctcagacaatgccaaa
atcataatagtgcagcttaataaatccatagaaattaattgtaccagacccagtaacaatacaagaagaagtata
agtcttggacctggacgggcgttctatgcaacaggtgatgtagtaggagatataagacaagcacactgtaatgta
agtagacaacaatggaatgacacggtacagaaagtaactgcatcactacaaaagatctttaaaaaaggcaatata
acctttaagccacccacaggaggggacctagaaatgacaacacatagttttaattgtagaggggaatttttctat
tgtaatacatcagacttgtttaatatcagtacaacaaatagtactaatgatactatcacactcccatgtaagata
aaacaaattgtgagaatgtggcagagagtgggacaagcaatgtatgcccctcccattgcaggagaaattacatgc
aggtcaaatattacaggactactattaacaagagatggtgggggtggaggtaatcaaacaaatgaaaccttcaga
cttgcaggaggaaatatgagggacaattggagaagtgaattatataagtataaagtagtaaagattaaaccacta
ggaatagcacccaccaagtcaaggagaagagtggtggagagagaaaaaagagggatttggggctgctctggaaaa
ctcatctgcaccactaatgtgcgctggaacactagttggagtaataratcttttratgagatttgggataacatg
acctggatacaatgggaaagggaagtcagcaattacacacaagaaatatacagactaattgaagaatcgcaaaac
cagcaggaaaagaatgaacaagacttattagcactggaccagtgggcaagcttgtggaattggtttgacatatca
aggtggctgtggtatataagaatattcataatgatagtaggagggttaataggtttaagaatagtttttgctgtg
ctttctatagtaaatagagttaggcagggatactcacctttatctttccagttgcttgatacaattgcaatagca
gtagctaactggacagatagggttatagaagtagcacaagcagctggtagagcttttcttcacatacctagaagg
ataagacaaggctttgaaagagctttgctataaatgggatgcaagatgtcaaaagaatggtccagagtaagggaa
aggatgagacgaacccccccaacagcagatggagtaggagcagaagcagcagcagatggagtaggagcagcatct
caggatttagctaggcatggagcactcacaagcagcaatacagcatccaccaatgctgcttgtgcctggctagaa
gcacaacaggaggactcagaggtaggctttccagtcagaccacaggtacctttgagaccaatgacctttaaaggt
gcttttgatctcagcttcttttaaaagaaaaggggggactggatgggctaatttactccaagcaaagacaagat
atccttgacctgtgggtctataatacacaaggatacttcccagattggcagaattacacttcagggccaggaatt
aggtacccactgacctttgggtggtgcttcaaactagtaccaatggatccagcagaggtagaggaagccaataga
ggagagaacaacagtctattacaccccatctgccagcatggaatggaagatgaagacagaaccgtgctggtatgg
aagtttgacagtggcctagcacggagacacttagcccgagagctgcatccggagtattataaagactga
>#90 MH705151 7563 nt
atgggtgcgagagcgtcagtattaagcgggggaaaattagatgcatgggagaaaattcggttaaggccaggggga
aagaaacattataaactgaaacatctagtatgggcaagcagggagctggaaagattcgcagttaaccctggcctt
ttagactcagcagaaggctgtcaacaaataatggaacagttacaaccagctctcaagacaggatcagaagaactt
acatcattatttaatacagtagcaaccctctattgtgtacatcaaaggatagatgtaagagacaccaaggaagct
ttagataaaatagaagaaataaagagcaagcaaaagacacagcaggtagcagctgacacaggaaacagcaacaag
gtcagtcaaaattaccccataatacaaaatacacaagggcaaatggtatatcagagcttatcacctaggactttg
aatgcatgggtaaaagcaatagaagaaaaggctttcagcccagaagtaatacccatgttcacagcattatcagag
ggagccgccccacaagatttaaatatgatgctgaacatagtgggggacaccaggcagctatgcaaatgttaaaa
gataccattaatgaagaagctgcagaatgggatagggtacatccagtacatgcagggcctattcaaccaggccag
atgagagaaccaaggggaagtgacatagcaggaactactagtacccttcaagaacaaataggatggatgacaagc
aatccacctatcccagtgggagaaatctataaaagatggataatcctgggattaaataaaatagtaagaatgtat
agccctgttagcattttggacataagacaagggccaaaagaacccttcagagactatgtagataggttctttaaa
actctcagagctgagcaagctacacaagaggtaaaaggttggatgacagaaaccttgctggtccaaaatgcgaat
ccagactgtaagaccattttaagagcattaggagcaggggctacattagaagaaatgatgacagcatgtcaggga
gtgggaggacccggccataaagcaagggttttggctgaggcaatgagtcaagtacaacatacaaacataatgatg
cagagaggcaattttaggggccagaaaagaatgattaaatgtttcaactgtggcaaagaaggacacctagccaga
aattgcagggcccctaggaaaaagggctgttggaaatgtgggaaggaaggacatcaaatgaaagaatgcacagaa
agacaggctataggaggacagctaaaagaagctctattagatacaggagcagatgatacagtattagaagacata

aatttgtcaggaaaatggaaaccaagaatgataggggggaattggaggttttatcaaagtaaggcagtatgatcag
atacttatagaaatttgtggaaaaaaggctataggtacagtattggtaggacctacacctgtcaacataattgga
agaaatatgttgactcagattggttgtactttaaattttccaattagtcctattgaaactgtaccagtaaaatta
aagccaggaatggatggtccaaaggttaaacaatggccattgacagaagagaaaataaaagcattaacagaaatt
tgtaaagaaatggaaaaggaaggaaaaatttcaaaaattgggcctgaaaatccatacaatactccagtatttgct
ataaagaaaaaggacagcactaaatggaggaaattagtagatttcagagagctcaataaaagaacccaggacttc
tgggaagttcaattagggataccacatccagcgggtttaaaaaagaaaaaatcagtaacagtactagatgtgggg
gatgcatatttttcagttcctttacatgaagacttcagaaagtatactgcattcaccatacctagtataaacaat
gagacaccaggaatcaggtatcagtacaatgtgcttccacagggatggaaaggatcaccagcaatattccaaagt
agcatgacaaaaatcttagagccctttagattaaaaaatccagaattagttatctatcaatacatggatgacttg
tatgtaggatctgatttagaaataggacagcatagaacaaaaatagaggagttaagagatcatctattgagatgg
gggtttactacaccagacaaaaagcatcagaaagaacctccatttctttggatgggatatgaactccatcctgac
aaatggacagtccagcctatacagctgccaaacaaagacagctggactgtcaatgatatacagaaattagtggga
aaactaaattgggcaagtcaaatttatccagggattaaagtaagacaactgtgtaaactcctcaggggagccaaa
gcactaacagatatagtaacactgactgaggaagcagaattagaactggcagagaacagggagattctaaaagaa
cctgtgcatggagtatattatgacccatcaaaagacttagtagcagaaatacagaaacaagggcaagaccaatgg
acatatcaagtttatcaagagccattcaaaaatctgaaaacaggaaaatatgcaagaaaaaggtctgctcacact
aatgatgtaaggcaattagcagaagtggtgcaaaaagtgtccacagaaagcatagtaatatggggaaaaacccct
aaattcagattacccatacaaagagaaacatgggaagcatggtggatggagtattggcaggctacctggattcct
gaatgggagtttgtcaatacccctcctctagtaaaattatggtaccagttagaaaaggaccccatattaggagca
gagacttttatgtagatggggcagctaatagagagactaagctaggaaaagcagggtatgtcactgacagagga
agacaaaaggttgtttccctaactgagacaacaaatcaaaagactgaattgcatgcaatctacctagccttgcag
gattcagggtcagaagtaaatatagtaacagattcacagtatgcattaggaatcattcaggcacaaccagacaag
agtgaatcagagctagttaatcaaatgatagagaagctaatagaaaaggaaaaagtctacctgtcatgggtacca
gcgcacaagggaattggaggaaatgaacaagtagataaactagttagttctggtatcaggaaggtactgtttta
gatgggatagataaagctcaagaagaacatgaaagatatcacagcaactggagagcaatggctagtgattttaat
ctgccacctatagtagcaaaggaaatagtagccagctgtgataaatgtcaactaaaaggggaagccatgcatgga
caagtagactgcagtccagggatatggcaattagattgcacacatctagaaggaaaaataatcatagtagcagtc
catgtagccagtggctatatagaagcagaagttatcccagcagaaacaggacaggagacagcatactttctgcta
aaattagcaggaagatggccagtaaaagtaatacacacagacaatggcagcaatttcaccagcgctgcagttaaa
gcagcctgttggtgggcaaatatccaacaggaatttggaattccctacaatccccaaagtcaaggagtagtggaa
tctatgaataaagaattaaagaagatcatagagcaagtaagagagcaagctgaacaccttaagacagcagtacaa
atggcagtattcattcacaattttaaaagaaaaggggggattggggggtacagtgcaggggaaagaataatagac
ataatagcaacagacatacaaactaaagaattacaaaaacagattacaaaaattcaaaattttcgggtttattac
agggacagcagagacccaatttggaaaggaccagcaaaactactctggaaaggtgaaggggcagtagtaatacag
gacaatagtgacataaaggtagtaccaagaagaaaagcaaagatcattagggatacatggaacagtttagtaaaa
catcatatgtatgtttcaaagaaagctcaaggttggttttatagacatcactatgaaagcaggcatccaaaaata
agttcagaagtacacatcccactaggtgatgctaggatagtggtaagaacatattggggtctgcatacaggagaa
agagactggcacttgggtcatggggcctccatagaatggaggcagagaagatatagcacacaaatagatcctgac
ctagcagaccaattaattcatctgcattattttgactgttttgcagactctgccataagaaaagccatcttagga
gaaatagttagacctaggtgtgaatatcaagcaggacataaccaggtaggatctctacaatatttggcactgaaa
gcattagtaaaaccaaaaagggcaaagccacctttgcctagtgttaagaaattaacagaagatctgttagaagag
ctgaagcatgaagctgttagacatttccctaggccgtggctccatggattaggacaacatatctataacacctat
ggggatacttgggaaggggttgaagctataataagaatgttgcaacaactactgtttgttcatttcagaattggg
tgccaacatagcagaataggcattgttcgagggagaagagtcaggctagagccctggaatcatccgggaagtcag
cctaaaactgcttgtaataaatgtcattgtaaaaagtgttgctatcattgccaagcttgctttctcaagaaaggc
ttaggcatctcctatggcaggaagaagcggagaccccgacgaagaactcctcagggcagtaaggatcatcaaaat
cctatacaaaaggctacaggaaacttgtgggtcacggtatattatggggtacctgtgtggaaagacgcagagacc
accctatttgtgcgtcagatgctaaagcatatgatacagaagtgcataatgtctgggctacacatgcctgtgta
cccacagaccccagcccacaagaaatagacctggaaaatgtaacagaaaagtttaacatgtggaaaaataacatg
gtagagcagatgcatatggatataattagtctatgggatcaaagcctaaagccatgtgtaaagttaacccctctc
tgtgttactttagattgtagccatagcatcaccaccatcaatagcaccaccatcaatagcaacagcacaagtgat
atatcaggagaaataaaaaactgctctttcaatatgaccacagaactaagagataagacaaagaaagtatattca
ctttttatagacttgatgtggtacaaattgatgaaaataatggtagtaataacagtgagtatagactaataaat
tgtaatacctcagccattacacaggcttgtccaaaggtaacctttgagccaattcccatacattattgtgcccca

gctggttttgcgattctaaagtgtaaggaggagaatttcaatggaacagggatatgcaggaatgtcagcacagta
caatgcacacatggaattaagccagtagtatcaactcaactgctgttaaatggcagcctagcagaaggagaggta
gtgattagatctgaaaacttcacaaacaatgccaaaatcataatagtacagtttaatgagtctgtacaaattaat
tgtaccagacctaacaacaatacaagaagaagtttccgtataggaccaggacagacattctatgcaacaggtgaa
ataataggaaatataagacaagcacattgtaatgtcagtaaaacagactgggagagcactttacaaaaggtagct
gcacaattaggcaaagaatttaaaaccaacacaacaatctttgctaactcctcaggaggggatatagaaattaca
acacatagttttaattgtagaggggaattttctattgcaatacatcagacctgtttaatagcacttggcggaaa
ctggcaaatggcacttggagcaacataaataatgcaacgtcaaatgacactataactctccaatgcaaaataaag
caaattgtaaacatgtggcagagagtaggacaagcaatgtatgcccctcccatccaaggagtaatacagtgtgta
tcaaacattacaggactactcttaacaagagatggtgggagtagtaacagggcaaatgagaccttcagacctgga
ggaggagatatgagggacaattggagaagtgaattatataagtataaagtagtaaaaattgaaccactaggagta
gcacccaccagggcaaggagaagagtggtggagagagaaaaaagaggaatttggggttgctctggaaaactcatc
tgcaccactactgtgccctggaactctagttggagtaatagatctcaggaggaaatatggaacaacatgacctgg
ctgcaatgggaaaaagaaattagcaattacacagacataatatatagtctacttgaagaatcacagaaccagcag
gaaaagaatgaacaagacttattggcattggacaagtgggcaagtctgtggaattggtttgacataacaaattgg
ctatggtatataaaaatatttataatgatagtaggaggcttaataggattaagaatagtatttgctgtgctttct
ataataaatagagttaggcagggatactcacctctctcgtttcagtacctgtggaaccttctgttatattgggtt
cgggaactgaaaattagtgctattaatttgcttgataccacagcagtagcagtagctgagtggacagataggatt
atagaaataggacaaagacttgctagagctattctcaacatacctagaagaatcagacagggcttagaaagggct
ttgctataaatgggtggcaagtggtcaaaaagcagcatagtaggatggcctgatgttagggaaagaatgagacga
tgtcctgaagttagggaaagaatgagacgagctcctccagcagcagaaggagtaggagcagtgtctcaagattta
gataagcatggagcaatcacaagcaggaatacagcaactaccaatgctagttgtgcctggctggaagcacaagag
gaagaaggggaggtaggctttccagtcaggccacaggtacctttaagaccaatgacttataaggcagctgtggat
ctcagccacttttaaaagaaaaggggggactggatgggttaatttggtcccagaaaagacaagacatccttgat
ctgtgggtctacaacacacaaggcttcttcccagattggcagaattacacaccagggccagggcctagattccca
ataacatttggatggtgctttaagctggtaccagttgatccagctgaagtagaagaggctactgaaggagagaac
aacagcttattacaccctatatgccaacatggagcggaggacactgagagagaagtattaaagtggaagtttgac
agtcgcctggcattaaaacacagagctcaagagctgcatccggagttctacaaagactgctga
>#91 KF716478 7446 nt
atgggtgcgagagcgtcagtattgagtggtggacaattagataggtgggagaaaattcggctaaggccagggggga
aagaaaaaatatagactaaaacatctagtatgggcaagcagggagctggatagattcgcacttaaccctagcctt
ttagaaacaacagaaggatgtcaacaaatattagaacagttacaaccagctattaagacaggaacagaagaactt
agatcattatataatacagtagcaaccctctattgcgtacatcgaaagatagaggtaaaagacaccaaggaagct
ctagataaaatagaggaactacaaaagaaaagcaagcaaaagacacaacaggcagcggctgacacaggaagtaac
agcagcacggtcagccaaaattaccctatagtgcaaaatgtacaagggcaaatgatacaccagcccttgtcacct
aggactttgaatgcatgggtaaaagtaatagaagaaaagggtttcaatccagaagtaatacccatgttctcagca
ctatcagaaggagccaccccacaagatttaaatatgatgctaaacatagtggggggacaccaggcagctatgcaa
atgctaaaagataccatcaatgaggaagctgcagaatgggacaggttacatccagtacatgcagggcctgttgca
ccaggccagatgagagaaccaaggggaagtgatatagcaggaactactagtaacactcaagaacaaatagcatgg
atgacaggcaatccacctatcccagtgggagacatctataaaagatggataatcctgggattaaacaaaatagta
agaatgtatagtcctgttagcattttggatataaaacaagggccaaaagaacccttcagagactatgtagatagg
ttctttaaagctctcagagctgaacaagctacacaggaggtaaaaggttggatgacagaaacattactggtccaa
aatgcaaatccagattgtaagtctattttaagagcattaggatcaggggctacattagaagaaatgatgacagca
tgccagggagtgggaggacccagccataaagcaagggttttggctgaggcaatgagtcaagtccaacagccaaac
ataatgatacagagaggcaattttagaggccagaaaaagattaagtgtttcaactgtggcaaagaaggacaccta
gccagaaattgcagggcccctagaaaacagggctgttggaaatgtgggaaggaaggacaccaaatgaaagactgc
actgagagacaggctatagggggccagctaagagaagctctattagatacaggagcagatgatacagtattagaa
gacataaatttgccaggaaaatggaaaccaaaaatgatagggggaattggaggttttatcaaagtaagacagtat
gatcagatactcatagaaatttgtggaaaaaaggctataggtacagtgttggtaggacctacacctgtcaacata
attggaagaaatatgttgacccagattggctgtactttaaattttccaattagtcctattgaaactgtaccagta
acattaaagccaggaatggatggcccaagaattaaacaatggccactgacagaagaaaaaataaaagcattaaca
gaaatttgtacagaaatggaaaaggaaggaaaaatttcaaaaattgggcctgaaaatccatacaatactccagta
tttgctataaagaaaaaagacagcactaaatggagaaaattagtagatttcagagaacttaataaaagaactcaa
gacttttgggaagttcaattaggaataccgcatccagcgggcttaaaaaagaaaaaatcagtaacagtactagat
gtgggggacgcatatttttcagttcccttagataaaaatttagaaagtatactgcattcaccatacctagtata

aacaatgagacaccaggaatcaggtatcagtacaatgtgcttccacagggatggaaaggatcaccggcaatattc
cagagtagcatgataaaaatcttagagccctttagagcaaaaatccagaaataattatctatcaatacatggat
gacttgtatgcaggatctgatttggagatagggcagcatagagcaaaagtagaagaattaagagatcatctatta
agttggggatttaccacaccagacaaaaagcatcagaaggaacctccatttctttggatgggatatgaactccat
cctgacaaatggacagtacagcctatacagctgccagaaaaagaaagttggactgtcaatgatatacagaaatta
gtgggaaaactaaattgggcaagtcaaatttatgcagggattaaggtaaagcaactgtgtaagctcctcagagga
gccaaagcactaacagatatagtaccactgactgaggaagcagaattagaattggcagagaatagggaaattcta
aaagaccctgtgcatggagcatattatgacccctcaaaagaattaatagcagaaatccaaaaacaagggcaggat
caatggacatatcaaatttatcaagagccatttaaaaacctaaaaacaggaaaatatgcaagaaaaaggtctgct
catactaatgatgtaaaacaattaacagaagtggtgcaaaaagtggtccaggaaagtatagtcatatggggaaag
acccctagatttaaactacccatacaaaaagagacatgggaaacatggtggatggactattggcaggctacctgg
attcctgaatgggagtttgtcaatacccctcccctagtaaaattatggtaccagttagagaaagaccccataata
ggagcagagactttctatgtagatggggcagccaatagggagactaagctaggaaaagcagggtatatcactgat
aaaggaagacaaaaggttgtttccctaactgagacaacaaatcaaaagactgaactacatgcaatcaatctagcc
ttgcaggattcaggatcagaagtaaatatagtaacagactcacaatatgcattaggaatcattcaggcacaacca
gacagaagtgagtcagagttagtcaatcaaataatagaaaagctaataggaaaggacaaagtctatctgtcatgg
gtaccagcacacaaggggattggaggaaatgaacaagtagataaattagtcagctctggaatcaggaaggtacta
tttttagatgggatagataaggctcaagaagaacatgaaagatatcacagcaattggagagcaatggctagtgat
tttaatctgccacccatagtagcaaaggaaatagtagccagctgtgataaatgtcagctaaaaggggaagccatg
catggacaagtagactgcagtccagggatgtggcaattagattgcacacatctagaaggaaaagtaattctggta
gcagtccatgtagccagtggctatatagaagcagaagttatcccagcagaaacaggacaggagacagcatacttt
ctgctaaagctagcaggaagatggccagtaaaagtagtacacacagacaatggtagcaacttcaccagcgctgca
tttaaagcagcctgttggtgggcaaatgtccaacaggaatatgggattccctacaatccccaaagtcaaggagta
gtagaatctatgaataaggaattaaagaaaatcataggacaggtaagagatcaagctgaacaccttaagacagca
gtacaaatggcagtattcattcacaattttaaaagaaaaggggggattggggactacagtgcaggggaaagaata
atagacataatagcaacagacatacaaactagagaactacaaaaacaaattacaaaaattcaaaattttcgggtt
tattacagggacagcagagagccactttggaaaggaccagcaaaactactctggaaaggagaaggggcagtggta
atacaggacaataatgatataaaggtagtgccaaggagaaaagtaaagatcattaaggatacatggcacagttta
gtaaaacatcatatgtatgtctcaagaaaagctaaagattggcattatagacatcactatgaaagtaggcatcca
aaagtaagttcagaagtgcacatcccactaggggaggctagaatagtagtaagaacatattggggtctgcataca
ggagaaaaggactggcaattgggccatggggtctccatagaatggagactaaaaagatatagcacacaaatagat
cctgacctggcagatcaactaattcatctgcattattttaactgtttttcagaatctgccataaggagagccata
ttaggacaagtagttagccctagttgtgaataccaagcaggacataacaaggtaggatccctgcaatatttggca
ctgaaagcactagtaacaccaacaaggacaaagccacctttgcctagtgttaagaaactaacagaagatctgtta
gaagagcttaagcatgaagctgttagacattttcctaggccatggctccatggactaggacaacatatctatgaa
acctatggggacacttgggaaggagttgaagctataataagaattttacaacaattactgtttgttcatttcaga
atcgggtgccaacatagcagaataggcattaacattcgcaggagacgaggcaggctagagccctggaaccatcca
ggaagtcagcctgcaaccgcttgtaataagtgttactgtaaaaagtgttgctttcactgtcaagcgtgctttctg
aacaaaggcttaggcatctccgctacagacaaattgtgggttactgtctactatggggtacctgtatggaaagac
gcagagaccaccttattttgtgcatcagatgcgaaagcatatgagaaagaaatgcacaatgtctgggctacacat
gcctgtgtacctacagaccccaacccacaagaagtagctttggaaaatgtgacagaagagtttaatatgtggaaa
aataacatggtagaacagatgcagacagatataatcagtctatgggaccaaagcctaaagccatgtgtagagtta
acccctctctgcgtcactttggagtgtagcaatatcaacagcaccaataacaccgccaccaatttcaccaatttc
atgggagaagacataaaaaactgctctttcaatatgaccacagaattaagggataaggagaagaaagtatattca
cttttctataaagtggatataacaccattggaaaacagcaacgaaactaagtataggttaataaattgtaatacc
tcagccattaaacaagcctgtccaaaggtatcctttgagccaattcccatacattattgtgctccagctggtttt
gcgatcctaaagtgtagagatgaggagttcaatggaacagggccatgcaaaaatgtcagcacagtacaatgcaca
catggaatcaagccagtagtatcaactcaactactattaaatggcagtctagcagaaaagaaggtaatgattaga
tctgaaaatttcacaaacaatgctaaaaacatcatagtacaatttaataagtcagtagaaattaattgtaccaga
cctaacaacaacaccagaaaaagtgtacatataggaccaggacgagcattctatgcaacagatataataggggac
ataagatgggcatattgtaatgtcagtagatcagaatggaaggaaactttacaacaggtagtcaaacaattagga
aaccattggaataaaacaatacactttaataattctgcaggaggggatttagaaattacaacacatagttttaat
tgtggaggagaatttttctattgtaatacatcaagcctgtttaatagtacttgggtatatggtaatggaagctgg
gggtcaaatagcacggggttaaatagtacaatgataaataatactattttcagtgataccataattcttcaatgc
agaataaagcaaattataaatatgtggcagagagcaggacaagcaatatatgcccctcccatcaaaggagtaata

agctgtagatcaaacatcacaggactaatattaacaagagatggtgggagtaacagcaatgcaagtacaaatgga
agtgaaaccttcagacctggaggaggagatatgagggacaattggagaagtgaattatataagtataaagtagta
aaagttgaaccactaggaatagcaccctccaaggcaaggagaagagtggtggcgagagaaaaaagaggaatttgg
ggctgctctggaaaactcatctgccccactactgtgccctggaactctagttggagtaataaaagttacagtgag
atatgggataacatgacctggctgcagtgggataaagaaattagcaattacacagacctaatatatgggctaatt
gaagaatcgcaaaaccagcaggaaaagaatgaactagacttattagcattaaacaagtgggcagagctgtggaat
tggtttgacttatcaaactggctgtggtatataagaatatttataatgatagtaggaggcttaataggattaaga
atagttttgctgtgctttctataataaatagagttaggcagggatactcacctttgtcgttccaggctattagt
ttgcttgataccatagcaatagtagtagctggctggacagatagggctatagaaataggacaaggaattggtaga
gctatcctcaacatacctagaagaatcaggcagggcttagaaagggctttgctgtaaatgggtagcaagtggtca
aaaagcagcatagtgggatggcctcaggttagggaaagaatgagacgagctcctccacaagctcctccaactcct
ccagcagcaaaaggagtaggagcagtatctcaagatctagagaaacatggagcaatcacaagcagtaatataaat
cacccgagttgcacctggctggaagcgcaagaggaagaggaggaggtaggctttccagtcaggccacaagtacct
ttaaggccaatgacttacaagggagctcttgacctcagccacttttaaaagaaaaggggggactggatgggcta
gtttactccaagaagagacaagagatccttgatctgtgggtatacaacacacaaggctatttccctgattggcag
aattacacaccagggccaggggtcagatacccactaacatttggatggtgtttcaagctagtaccagtagatcca
gaggaagtagagaaggctaatgagggagagaacaatagcctgttacaccctgtgtgccaacatggaatggatgat
gaagagagagaaacattgatatggagatttgacagccacctggcttttaaacacagagcccaagagttacatccg
gagtactacaagaactgctga
>#92 AJ320484 7419 nt
atgggtgcgagagcgtcagtattaagcgggggaaaattagatgcatgggaaaaaattcggttacggccaggagga
aagaaaaaatatagactgaaacatatagtatgggcaagcagggagttagaacgattcgcacttaatcctggcctt
ttagagacatcagaaggctgtaaacaaataataggacagctacaaccagctgttcagacaggatcagaagaactt
aaatcattatttaatacagtagcaaccctctattgtgtacatgagaggatagaggtaaaggacaccaaggaagct
ttagaaaaaatagaggaagaacaagccaaaagtaagaaaaaagcacagcaagcaacagctgacacaagaaacagc
agccaggtcagccaaaattatcctatagtgcaaaacctacagggacaaatgatacaccaggccatatcacctaga
actttgaatgcatgggtaaaagtaatagaagagaaggctttcagcccagaagtaatacccatgtttacagcatta
tcagaaggagccaccccgcaagacttaaacaccatgctgaacacagtagggggcatcaagcagccatgcagatg
ttaaaagagaccatcaatgaggaagctgcagaatgggataggctacatccaccgcaggcagggcctgttgcacca
ggccagatgagggaaccaaggggaagtgatatagcaggaactactagcacccttcaggaacaaatagggtggatg
acaagcaatccgcctgtcccagtaggagagatctataaaagatggataatcctggggttaaataaaatagtaaga
atgtatagtcctgtcagcattttggacataagacaaggtccaaaggaaccctttagagattatgtagatcggttc
tataaaactctaagagccgagcaagcttcacaagatgtaaaaaattggatgaccgaaaccttgttggtccaaaat
gcaaacccagattgtaaaactatcttaaaagcactgggaccaggggctacattagaagaaatgatgacagcatgt
cagggagtaggggacccagtcataaagcaagagttttggctgaggcaatgagccaagcaacaaatttgaatggt
gccgcaatgatgcagagaagcaattttaagggcccaaggaaaattgttaagtgtttcaactgtggcaaagaaggg
cacatagcaagaaattgcaaggctcctaggaaaaagggctgttggaaatgtggcagtgaaggacaccaaatgaaa
gattgcactgaaagacaggctatagggggacagctaaaggaagctctattagatacaggagcagatgatacagtg
ttagaagacataaatttgccaggaaaatggaaaccaaaaatgatagggggaattggaggttttatcaaagtaaga
cagtatgatcaaatacccatagaaatctgcggatataaagctataggtacagtattaataggacctacacctgtc
aacataattggaagaaatttgttgactcagattggctgcactttaaattttccaattagtcctattgaaactgta
ccagtaaaattaaagccaggatggatggcccaaaagttaaacaatggccattgacagaagaaaaaataaaagca
ctaacagaaatttgtgcagacatggaaaaagaaggaaaaatttcaagaattgggcctgaaaatccatacaatact
ccagtatttgccataaagaaaaaagacagtactaagtggagaaaattagtagatttcagagaacttaataagaga
actcaagatttctgggaagttcaactaggaataccacatcctgcagggctaaagaagaaaaaatcagtaacagta
ctggatgtgggtgatgcatatttttcagttcccttagatatagactttagaaagtatacagcattcaccatacct
agtacaaacaatgaggcaccaggaattagatatcagtacaatgtgcttccacagggatggaaaggatcaccagca
atattccaatgtagcatgacaaaaatcttagaaccctttagaaaacaaaatccagaaatagttatctatcaatac
atggatgatttgtatgtaggatctgacttagaaatagggcagcatagaacaaaaatagatgaattaagggaacat
ctattgaggtggggacttatcacaccagacaaaaaacatcagaaagaacctccatttctttggatgggttatgaa
ctccatcctgataaatggacagtgcagcctataaagctgccagaaaaagaaagctggactgtcaatgatatacag
aagttagtgggaaaattaaattgggcaagccagatttatccaggaattagagtaaaacacttatgtaaactcctt
aggggagccaaagcactgacagaagtaataccactgacaaaagaagcagaattagaactggcagaaaacaaggaa
attctaaaagaaccagtacatggagtgtattatgacccatcaaaagacttaatagcagaagtacagaaacaaggg
caagaccaatggacatatcaaatttatcaagagccatttaaaaatctgaaaacaggaaagtatgcaaaaatgagg

agtgcccacactaatgatgtaaaacaattaacagaggcagtgcaaaaaatagcccaagaatgcatagtaatatgg
ggaaagactcctaaatttagactacccatacaaaaggaaacatgggaagcatggtggaaagaatattggcaggcc
acctggattcctgagtgggagtttgtcaatacccccccttagttaaattatggtaccagttagagaaggaaccc
atactaggagcagaaactttctatgtagatggggcagctaatagagagaccaaattaggaaaagcaggatatgtt
actaacaaaggaagacagaaagttgtctctctaactgacacaacaaatcagaagactgagttacaagccattaat
ctagctttgcaggattcgggattagaagtaaacatagtaacagattcacaatatgcattaggaatcattcaggca
caaccagataaaagtgaatcagagttagtcagtcaaataatagagcagttaataaaaaaggaaaaggtctaccta
gcatgggtaccagcacacaaggggattggaggaaatgagcaagtagataaattggtcagtaatggaatcaggaaa
gtattatttttggatggaatagataaggctcaagaagaacatgagaaataccacaacaattggagagcaatggct
agtgattttaacctgccacctgtggtagcgaaagaaatagtagctagctgtgataaatgtcagctaaaaggagaa
gccatgcatggacaagtagactgtagtccaggaatatggcaattagattgtacacatctagaaggaaaagttatc
ctggtagcagttcatgtagccagtggctatatagaagcagaagtgatcccagcagaaacagggcaggaaacagcc
tactttatcttaaaattagcaggaagatggccagtaaaagtagtacatacagacaatggcagcaatttcaccagt
tctgcagttaaggctgcctgttggtgggcaggcattaagcaggaatttggaattccccacaatccccaaagtcaa
ggagtagtggaatctatgaataaagaattaaagaaaattataggacaggtaagagatcaagctgaacatcttaag
acagcagtacaaatggcagtattcattcacaattttaaaagaaaaggggggattggggagtacagtgcaggggaa
agaataatagacataatagcaacagacatacaaactacaaaattacaaaaacaaatcataaaaattcaaaatttt
cgggtttattacagggacagcagagatccaatttggaaaggaccagcaaagcttctctggaaaggtgaaggggca
gtagtactacaagataatagtgacataaaggtagtaccaagaagaaaagtaaagatcattagggatacttggaaa
agtttagtaaaacaccatatgtatgtttcaaagaaagctcaaggatggttgtatagacatcactatgacagccct
cacccaaaaataagttcagaagtacacatcccattaggagacgctagactggtagtaaaaacatattggggcctg
catacaggagaaagagaatggcatctgggtcaaggagtctccatagaatggagaaaaaggagatatagcacacaa
gtagaccctggcctagcagaccaactaattcatatgtattattttgattgttttgcagaatctgctataagaaaa
gccatattaggacaattagttagtcctaggtgtgaatatcaagcaggacataacaaggtaggatccttacagtat
ttggcactaacagcattaataaaaccagaaaaaacaaagccacctttgcctagtgttaggaagctaacagaagat
cttttagaggaacttaagagtgaggctgttagacattttcctaggatatggctccatggattagggcaatatatc
tatgaaacttatggggatacatgggcaggagttgaagctctaataagaaccctgcaacaactactgtttattcat
ttcagaattgggtgtcaacatagcagaataggcattattcaacagagaagaccaagattagagccctggaaccat
ccaggatgtcagcctaggacttcttgtaacaattgctattgtaaaaagtgttgctatcattgccaaaaatgcttc
ttaacgaaaggcttaggcatctcagttgcagagaagaagtgggtcacagtgtattatggggtacctgtgtggaaa
gaagcaacaaccactctattttgtgcatcagatgctaaatcatataaaacagaggtacataatatctgggctaca
catgcctgtgtaccaacagaccccaacccacgagaaatagaactggaaaatgtcacagaaaactttaacatgtgg
aaaaataacatggtggagcagatgcatgaggatatcatcagtttatgggatcaaagcctaaaaccatgtgtaaaa
ttaaccccactctgtgtcactttaaactgcactgatgcaaggaggaatgagactaggaataatattacaggaatg
gaaaacaatgatcaaatagaaatgaaaaactgctctttcaatataaccacaaaattaatagataagaagaagcaa
gtacatgcactttttttatagacttgatgtggtacaaatagataatgatactagtaatagcaactatagcaactat
agattaataaattgcaatacctcagccattacacaggcttgtccaaaggtaacttttgagccaattcccatacat
tattgtgccccagctggttttgcaattctaaagtgtagagataagaagttcaatggaacaggaccatgcaaaaat
gtcagcacagtacaatgcacacatggaattaggccagtagtgtcaacccaactgctgttgaatggcagtctagca
gaagaagagataataattagatctgaaaatctcacaaacaatgctaaaaccctaatagtacagcttaatgagtct
gtagaaatcaattgtacaaggccctactacaaccagataagacaaagaacatctataggacaagggcaagcactc
tatacaacaagagtaacgggagatataagaaaagcatattgcaatattagtaaagcaggatggaataaaacttta
cagcaggtagcaaaaaaattaggagacctctttaaccagacaacaataatttttaaaccatcctcgggaggagac
ccagaaattacaacacacagctttaattgtggaggggaatttttctactgcaatacatcaaaactgtttaacagt
gcatggaatgacagtacatggaatatagggaataataatacagggtcagataatgagacaatcattatcccatgc
agaataaaacaaattataaacatgtggcagggagtaggaaaagcaatgtatgcccctcccatcgaaggatggatc
aattgtgcatcaaatattacagggctcttactggtaaggatggtggtggtgcaaatgatagtcagaacgagacc
ttcagacctcaaggaggagatatgagagacaattggagaagtgaattatacaagtataaagtagtaaaaattgaa
ccactaggaatagcacccaccaaggcaaagagaagagtggtggaaagagaaaaaagaggaagttgggggtgctct
ggaagacacatttgcaccactactgtgccctggaactctagttggagtaataaatctatagatgacatttggaat
aacatgacctggatggagtgggaaaaagaaattgacaattacacaggtgtaatatacagattaattgaggaatcg
caaacccagcaagaaaagaatgaacaagaactattgcaattggacaaatgggcaagtttgtggaattggtttagc
ataacaaaatggctgtggtatataaaaatattcataatgatagtaggaggcttaatagggttaagaatagttttt
actgtgctttctttagtaaatagagttaggcagggatactcacctctatcgtttcaggctattagcttgtttaac
gctacagcagtagcagtagctgaggggacagatagggttatagaagtagtacaaagatttttagaggtattctt

aacgtacccacacgaataagacagggcttggaaagggcgttactataaatgggtggcaaatggtcaaaaagtaaa
attgggtggcctgctgtaagggacagaatacaaagaactagtccagcagcagaaggggtgggagcagcatctcga
gacctggaaaaacatggggcaatcacaagtagcaatacagcagagactaatcctgactgtgcctggctagaagca
caagaagaggaagaggaggtgggttttccagttagacctcaggtaccgttaagaccaatggcttacaaggcagct
tttgatctaagccactttttaaaagaaaaggggggactggaagggttaatttactccaagaaaagacaagaaatc
cttgatctttgggtctaccacacacaaggctatttccctgattggcaaaactacacaccagggccagggattaga
tacccactgacctttggttggtgcttcgagctagtaccagtggatccagaggaggtagaagaggccaataaagga
gagaacaactgcttgttacaccctatgagccagcatggaatggaggacccggagagagaggtgttagtgtggaga
tttaacagcagactagcatttgaacacaaggcccgaatacagcatccggagttctacaaagactgctga
>#93 KT200357 7392 nt
atgggtgcgagagcgtcagtattaagcgggggagaattagatagatgggagaaaattcggttaaggccaggggga
aagaaaaaatataggttaaaacatatagtatgggcaagcagggagctagaacgattcgcagtcaatcctggcctt
ttggagacatcagaaggctgcagacaaatactgggacagttacaaccgtcccttcagacaggatcagaagaactt
agatcactatataatacagtagcaaccctctattgtgtacatcaaaagatagatgtaaaagacaccaaggaagct
ttagacaaggtagaggaagagcaaaacaaaagcaagaaaagggcacagcaagctgcagctggcacaggaaacagc
aaccaggtcagcgtcagccaaaattaccctatagtgcagaaccttcaggggcaaatggtacatcaggccatatca
cctagaactttaaatgcatgggtaaaagtggtagaggagaaggctttcagcccagaagtaatacccatgttttca
gcattatcagaaggagctaccccacaagacttaaacaccatgttaaacacagtgggggacatcaagcagccatg
caaatgctaaaagagaccatcaatgaggaagctgcagaatgggatagattgcatccagtacaggcagggcctgtc
gcaccaggccagataagagaaccaaggggaagtgacatagcaggaactactagtacccttcaagaacaaatagca
tggatgacacataatccacctatcccggtaggagaaatttataaaagatggataatcttgggattaaataaaata
gtaagaatgtatagccctgtcagcattctggacataagacaaggaccaaaggaacctttagagactatgtagat
cggttctataaaactctaagagctgaacaagcttcacaggatgtaaaaaattggatgacagagaccttgttagtc
caaaatgcgaacccagattgcaagactattttgaaagcactgggaccagcagctacactagaagagatgatgtca
gcatgccaaggagtggggggcccggccataaagcaagagttttggctgaagcaatgggccaaataacaaataca
gctaccataatgatgcagaggggcaattttaagaaccaaaaaaggactgttaagtgtttcaattgtggcaaggaa
ggacacatagccagaaattgcagggcccctaggaaaaaggctgttggaaatgtggaagggaaggacaccaaatg
aaagattgcactgagagacaggctgtagggggcaactaaaggaagctctattagatacaggagcagatgataca
gtactagaagaaataaatttgccagggaaatggaaaccaaaaatgataggggggaattggaggttttatcaaagtg
agacagtatgatcaggtacccatagaaattagtgggcataaagccataggtacagtattagtaggacctacacct
gtcaacataattggaagaaatctgttgacacagattggatgcactttaaattttcccattagtcctattgaaact
gtaccagtaaaattaaagccaggatggatggcccaaaagttaaacaatggccattgacagaagaaaaattaaat
gcattaatagaaatttgtgcagaaatggaaaaggaaggaaaaatttcaaaaattgggcctgaaaatccatacaat
actccaatatttgccataaagaaaaaagacagtactaaatggagaaaattggtagatttcagagaacttaataag
agaactcaagacttctgggaaattcaattaggaataccacatccttcagggttaaaacagaaaaaatcagtaaca
gtattggatgtgggtgatgcatatttttcagttcccttagataaagactttaggaagtatactgcatttaccata
cctagtgtgaacaatgagaaaccagcaattagatatcagtacaatgtgcttccacagggatggaaaggatcacca
gcaatattccaaagtagcatgacaaaaatcttagagccttttagaaaacaaaatccagacatggttatctatcaa
tacatggatgatttgtatgtcggatctgacctagaaatagagcaacatagaacaaaaatagaggaactgagacaa
catctgttgaggtggggtttacaacaccagataaaaaacatcagaaagaacctccattcctttggatgggttat
gaactccatcctgataaatggacagtacagcctataaagctgccagaaaaggacagctggactgtcaatgacata
caaaaattagtgggaaaattaaactgggcaagtcagatttatccaggattaaagtaaggcaattatgtaaactc
cttaggggaaccaaagcactaacagaagtagtatcgctaacaagagaagcagaactagaattggcagaaaacagg
gagattctcaaagaaccagtacatggagtgtactatgacccatcaaaagacttaatagcagaaatacagaagcag
gggcaaggccaatggacatatcaaatttatcaagagccatttaaaaatctgaaaacaggaaagtatgcaagaacg
aggggtgcccacactaatgatgtaaaacaattaacagaggcagtgcaaaaaatagccacagaaggcatagtgata
tggggaaagattcctaaattcaaattacccatacaaaaagaaacatgggaaacatggtggacagagtattggcaa
gccacctggattcctgagtgggagtttgtcaatacccctcccttggtgaaattatggtatcagttagagaaagaa
cctatagtaggagcagaaactttctatgtagatggagcagctaatagggaaactaaaataggaaaagcaggatat
gttacagacagaggaagacaaaaagttgtccctctaacggacacaacaaatcagaagactgagttacaggcaatt
catttagctttgcaggattcaggattagaagtaaatatagtaacagactcccaatatgcattaggaatcatccaa
gcacaaccagatcagagtgaatcagagatagtcagtcaaataatagaacagttaataaaaaaggaaaaggtctac
ctggcatgggtaccagcacacaaaggaattggaggaaatgagcaagtagataaattggtcagtagtggaatcagg
aaagtgctattttagatggaatagataaggcccaggaagagcatgagaaatatcacaataattggagagcaatg
gctagtgattttgacctgcccccctgtagtagcaaaagaaatagtagccagctgtgataaatgtcagctaaaagga

gaagccatgcatggacaagtagactgtagtccaggaatatggcagctagattgtacacacttagaaggaaaagtt
atcctagtagcagttcatgtagccagtggttatatagaagcagaagttattccagcagagacagggcaagaaaca
gcatactttattttaaaattagcaggaagatggccagtaaaaacaatacatacagacaatggccccaatttcatc
agtaatacagttaaggccgcctgttggtgggcggggatcaagcaagaatttggcattccctacaatccccaaagt
caaggagtagtagaatctatgaataaagaattaaagaaaattatagggcaggtaagagatcaggctgaacatctt
aagacagcagtgcaaatggcagtattcatccacaattttaaaagaaaaggggggattggggggtacagtgcaggg
gaaagaataatagacatgatagcatcagacatacaaactaaagcattacaaaaacaaattacaaaaattcaaaat
tttcgggtttattacagggacagcagagatccactttggaaaggaccagcaaaacttctttggaaaggtgaaggg
gcagtagtaatccaagataatagtgaaataaaagtagtgccaagaagaaaagtaaaaatcattagggatacatgg
aaaagtttagtaaaacaccatatacatgtttcaaagaaagctaagaaatgggtttatagacatcactatgaaagc
attaatccaaaaataagttcagaagtacacatcccactaggggatgatacattagtagtaacaacatattggggt
ctgaacacaggagaaagagaatggcatttgggccagggagtctccatagagtggaggaaaaggagatatagaaca
caagtagaccctgacctagcagaccaactaattcatctgtattattttgattgtttttcagaatctgctataaga
catgccatattaggatatagagttagccctaggtgtgaatttcaagcaggacataataaggtaggatccttacag
tacttggcactagtagcattaataacaccaaaaaggacaaaaccacctgtgcccagtgttagcaaactaacagag
gatcttttagaagaacttaagagagaagctgttagacattttcctaggacatggctccatgacttaggacaatat
atctatgaaacttatggggatacctggactggagtggaagccataataagaatattacaacaactactgtttatt
catttcagaattggatgtcatcatagcagaataggcattattcgacagagaagagcaaggctagagccctggaaa
catccaggaagtcagcctaagactccttgtaccaaatgctattgtaaaaagtgttgctttcattgccaagtttgt
ttcataacaaaaggcttaggcatctccgctacagaacaattgtgggtcacagtctattatggggtacctgtgtgg
aaggaggctaccaccactctattctgtgcatcagatgctaaagcctataaaacagaggcacataatgtttgggcc
acacatgcctgtgtacccacagaccctaacccacaagaagtagaattggtaaatgtaacagaaaattttaacatg
tggaaaaataacatggtagaacagatgcatgaggatataattagtttatgggatgaaagcctaaaaccatgtgtg
aaattaaccccactctgtgttactttaaattgcagtaatgctaatttcaattgcaataatactaataacgctagt
tgtctagagatgcaggcagaagtaaaaaattgctctttcaatatctccacaagcataaaaaacaaagaaaagaaa
gaacatgcactttttatagtcttgatgtaatgtcaatagataatacaagctatgcattgagacagtgtaacacc
tcagtcattaaacaggcctgtccaaaggtatcctttacaccaattcccatacattattgtgccccagctggtttt
gcgattctaaaatgtaaggataagaaattcaatggaacaggaccatgtaaaaatgtcagcacagtacaatgtaca
catggaattaagccagtagtgtcaactcaactgctgttaaatggcagtctagcagaagaagaggtagtgattaga
tctgaaaatctcacagacaatgctaaaaccataatagtgcagctgaatgaaactgtaaaaattaattgtacaaga
cccagtaacaacacaagaaaaggtatacacataggaccggggagtgcattttatgcaacaggagaaataatagga
gatataagacaagcacattgtaacgttagtagaacagaatggaacaaaactttaggatacatagttgaaaaatta
agagaacaatttaagaatacaacaataatctttaaccagtcctcaggaggggacccagaaattgaaatgcacagt
tttaattgtggaggggaatttttctactgtaatacaacacagctgttcaatagtacttgggagccagggaagaat
actactacagagggaaatgacactatcacgctcccatgcagaataaaacagattataaacaggtggcaggaagta
ggaaaagcaatgtatgcccctcccatcagaggacgaattaattgtacatcaaatattacagggctgttattaaca
agagatggtggtaacaataataaccagacaaataggacagagacctttagacctcaaggagggaatatgaaggac
aattggagaagtgaattatataaatataaagtagtacaagttgagccattaggaatagcacccaccaaggcaagg
agaagagtggtgcagagagaaaaaagaggcatttggggttgctctggaaaactcatttgcaccactaatgtgcct
tggaatgctagttggagtaacaaaacccgtagtgagatttgggaaaacatgacctggatgcagtgggaaagagaa
attagcaattacacagcaacaatatactccttaattgaagaatcgcaaatccaacaagaaaagaatgaacaagaa
ttattagaattagataagtgggcaagtttgtggaattggtttgacataacaaaatggctgtggtatgtaaaaata
ttcataatgatagtagcaggcttagtaggtttaagaatagcatttactatatttctttagtaaatagagttagg
cagggatactcaccattatcattccaggcagttagtttatttaatgccacagctataacagtagccgaaggaca
gatagggctatagaattacttcaaagcattggtagagctattctccacatacctagaagaataagacagggctta
gaaagggctttgcaataaatgggtggcaagtggtcaaaacttagtaggggatggaggttgtaagggaaagaatg
agacgaaccacaccagtagaaccagcagcaagaggggtgggagcagtatctcaagatttggaaaggcatggagca
atcacaagtagcaatacagcagctactaatcctgattgtgcctggctagaagcacaggaggaagaggacgtgggt
tttccagtcagacctcaggtacctttaagaccaatgacttacaaaggagcacttgatcttagccacttttaaaa
gaaaaggggggactggaagggctaattcactcccagagaagacaagatatccttgatctgtggatctaccacaca
caaggctacttccctgattggcaaaactacacaccagggccaggggtcagatttccactgacctttggatggtgt
ttcaagctagtaccagttgatccagatcaggtagaaaaggccaatgaaggagagaacagctgcctcttacacccc
atgggcctgcatggaatggatgacccagagaaagaagtgttaatgtggaggtttgacagccgcctagcatttcat
catgtggcccgagagctgcatccggagtactttaaaaactga
>#94 AY331292 7425 nt

atgggtgcgagagcgtcagtattaagcgggggaaaattagataaatgggaaaaaattcggttaaggccaggaggg
aaraaaaagtataaattaaaacatatagtatgggcaagcagggaactagaacgcttcgcagtcaatcctggcctg
ttagaaacaacagaaggctgcagacaaatactgggacagctacaaccatcccttcagacaggaacagaagaactt
agatcattatataatacaatagcagtcctctattgtgtacatcaaaggatagatataaaagacaccaaggaagct
ttagataagatagaggaagagcaagacaaaagtaagcaaaaggcacagcaagcagcagctgacacaggaaacagc
aacaaccaggtcagccaaaattaccctatagtgcagaacctccaggggcaaatggtacatcaggccatatcacct
agaactttaaatgcatgggtaaaagtagtagaagagaaggctttcagcccagaagtaatacccatgttttcagca
ttatcagaaggagccaccccacaagatttaaacaccatgctaaacacagtggggggacatcaagcagccatgcaa
atgttaaaagagaccatcaatgaggaagctgcagaatgggatagattgcatccagtgcatgcagggcctattgca
ccaggccagatgagagaaccaaggggaagtgacatagcaggaactactwgtacccttcaggaacaagtaggatgg
atgacaagtaatccacctatcccagtaggagaaatttataaaagatggataatcctgggattaaataaaatagta
aggatgtatagccctaccagcattctggacataagacaaggaccaaaagagccctttagagactatgtagaccgg
ttttataaaactctaagagctgagcaagcttcacaggaggtaaagaattggatgacagaaaccttgttggtccaa
aatgcgaacccagattgtaagactattttaaaagcattgggaccagcagctacactagaagaaatgatgacagca
tgtcagggagtgggaggacccggccataaagcaagagttttggccgaagcaatgagccgagtaacaaattcaaat
gccataatgatgcagagaggcaattttaagaaccaaagaaagactgttaagtgtttcaattgtggcaaagaaggg
catatagccagaaattgcagggctcctaggaaaaagggctgttggaaatgtggcagggaagggcaccaaatgaaa
gattgtactgagagacaggctatagggggcaactaaaggaagctctattagatacaggagcagatgatacagta
ttagaagaaatgaatttaccaggaagatggaaaccaaaaatgatagggggaattggaggttttatcaaagtaaaa
cagtatgatcagatacttatagaaatctgtggacataaggctataggtacagtattagtaggacctacacctgtc
aacataattggaagaaacctgttgactcagattggttgcaccttaaattttcccattagtcctattgaaactgta
ccagtaaaattaaagccaggaatggatggcccaaaagttaaacaatggccattgacagaagagaaaataaaagca
ttagtagaaatttgtacagaaatggaaaaggaagggaaaatttcaaaaattgggcctgaaaatccatacaatact
ccagtatttgccataaagaaaaaagacagtactaaatggagaaaattagtagatttcagagagcttaataagaga
acacaagacttttgggaagttcagttaggaataccacatcccgcaggattaaaaaagaaaaaatcagtaacagta
ttggatgtgggtgatgcatatttttcagttcccttagataaagaatttaggaagtacactgcatttaccatacct
agtataaacaatgaaacaccagggattagatatcagtataatgtgcttccacagggatggaaaggatcaccagca
atattccaaagtagcatgacaaaaatcttagagccttttagaaaacaaaatccagacatagttatctatcaatac
atggatgatttgtatgtaggatctgacttagaaatagggcagcatagaacaagaatagaggaactgagacaacat
ttgctgaagtggggatttaccacaccagacaaaaaacatcagaagaaacctccattcctttggatgggttacgaa
ctccaccctgacaaatggacagtacagcctatagtgctgccagaaaaagacagctatactgtcaatgatatacag
aagttagtgggaaaattaaattgggcaagtcagatttacgcagggattaaagtaagacaattgtgtaarctcctt
aggggagccaaagcactaacagaagtaataccactaacaaaagaggcagagctagaactggcagaaaacagggag
attctaaaagaaccagtacatggagtgtattatgacccatcaaaagacttaatagcagaaatacagaaacagggg
caaggtcaatggacatatcaaatttatcaggagccatttaagaatctgaaaacaggaaagtatgcaagaatgagg
ggtgcccacactaatgatgtaaaacaattaacagaggcagtgcaaaaaataaccacagaaagcatagtaatatgg
ggaaagattcctaaatttagactccccatacaaaaagaaacatgggaaatatggtggacagaatattggcaagcc
acctggattcctgagtgggagtttgtcaatacccctcccctagtgaaattatggtaccaattagagaaagaaccc
ataataggagcagaaactttctatgtagatggggcatctaacagggaaactaaattaggaaaagcaggatatgtc
actaacagaggaagacaaaaagttgtctccctaactgacacaacaaatcagaaaactgagttacaagcaatttat
ctagctttgcaggattcgggatcagaagtaaacatagtaacagactcacaatatgcattaggaatcattcaagca
caaccagataagagtgaatcagagctagtcaatcaaataatagagcagttaataaataaggaaaaggtctacctg
gcatgggtaccagcacacaaaggaattggaggaaatgaacaagtagataaattagtcagtgctggaatcaggaaa
gtactattttagatggaatagataaggcccaagaagaacatgagaaatatcacagtaattggagagcaatggct
agtgattttaacctgccacctgtagtagcaaaagaaatagtggccagctgtgataaatgtcagctaaaaggagaa
gccatgcatggacaagtagactgtagtccaggaatatggcaactagattgtacacatttagaaggaaaagttatc
ctggtagcagttcatgtagccagtggatacatagaagcagaagttattccagcagagacagggcaggaaacagca
tattttcttttaaagttagcaggaagatggccagtaacaacaatacatacagacaatggcagcaatttcatcagc
actacagttaaggccgcctgttggtgggcggggataaagcaggaatttggcattccctacaaccctcaaagtcaa
ggagtagtagaatctatgaataaagagttaaagaaaattataggacaggtaagagatcaggctgaacatcttaag
acagcagtgcaaatggcagtattcatccacaattttaaaagaaaaggggggattggggggatacagtgcaggggaa
agaatagtagacataatagcaacagacatacagactaaagaactacaaaaacaaattacaaaaattcaaaatttt
cgggtttattacagggacagcagagatccactttggaaaggaccagcaaagctcctctggaaaggtgaaggggca
gtagtaatacaagataatagtgacataaaagtagtgccaagaagaaaagcaaaaatcatcagggatacatggaaa
agtttagtaaaataccatatgtatagatcaaagaaagctaggggatggttttatagacatcattatgacagcact

catccaaaaataagttcagaagtacacattccactagggaatgcgagattggtaataacaacatattggggtctg
catacaggagaaagagactggcatttgggtcagggagtctccatagaatggagggaaaggaaatatagaacacaa
gtaacccctgacctagcagaccgactaattcatctgtattactttgattgtttttcagaatctgctataagaaag
gccatattaggacatatagttagtcctagttgtgaatatcaagcaggacataacaaggtaggatctctacagtac
ttggcactagcagcattaataacaccaaaaaagataaagccacctttacctagtgttacaaaactgacagaggat
cttttagaggagcttaagagtgaagctgttaggcactttcctaggatatggctccatggcttagggcaatatatc
tatgaaacctatggagatacttgggaaggagtggaagccataataagaactttgcaacaactgctgttcattcat
ttcagaattgggtgtcaacatagcagaataggcattactcgacagaggagagcaagattagagccctgggagcat
ccaggaagtcaacctaagactgcttgtacctcttgctattgtaaaaagtgttgctatcattgccaagtctgcttc
ataacaaaaggcttaggcatctccgctgaaaaagactcgtgggtcacagtctattatggggtacctgtgtggaaa
gaagcaatcaccactctattttgtgcttcagatgctaaagcatataagacagaggtacataatgtttgggccaca
catgcctgtgtacccacagaccccaacccacaagaagtagtattggcaaatgtgacagaacattttaatatatgg
gaaaataacatggtagaacaaatgcatgaggatataatcagtttatgggatgaaagcctaaagccatgtgtaaaa
ttaaccccactctgtgttactttaaattgcactgactatattaataacagtactgaaagtaagaaaaccaatgaa
ggcttgaacagtactatagaaatgaaaaactgctctttcaaaataacccacaggcatgagagataagatgcagaaa
gcatatgcactttttttataagcttgatatagtaccaatagatgatgataatattaataagactagcaccaaccat
aattataccagctataggttaataagttgtaacacctcagtcattacacaggcctgtccaaaggtatcctttgag
cccattcccatacattttgtgccccggctggttttgcgattctaaagtgtaacaacaagagttacacaggaaaa
ggaccatgtacaaatgtcagcacagtacaatgtacacatggaattaggccagtagtatcaactcaactgctgtta
aatggcagtctagcagaagaagagatagtrattagatctgccaatttctctaacaatgctaaaaccataatagta
cagctaaataaaactgtagtaattaattgtataagacctaacaacaatacaagaaaaagtatacatataggacca
gggagagcattttatgcaacaggagaaataataggagatataagacargcacattgtacccttaatggaacacaa
tggaataacactttaagacagatagctttaaaattaaaagaacaatttaagggtaaaacaatagtctttaatcac
tcctcaggaggggacccagaaattgtaaagcactcttttaattgtgcaggggaatttttctactgtgatacaaca
aaactgtttaatagtacttggcatggtaatgaaacatcttggaataatactgaaaagttaaatgacactatcata
ctcccatgcagaataaaacaaattataaacatgtggcaggaagtaggaaaagcaatgtatgcccctccaatcgaa
ggactaattaactgttcatcaagtattacggggctgctattaacaagagatggtggtaagagcaataatcagact
gagatcttcagacctggaggaggagatatgagggacaattggagaagtgaactatataaatataaagtagtaaaa
attgaaccagtaggagtagcacccaccaaggcaaagagaagagtggtgcagagagacaaaagagggatttggggt
tgctctggaaaactcatttgcaccactgatgtgccttggaatgatagctggagtaataaatctctggataagatt
tggaataacatgacctggatgcagtgggaaagagaaattgacaattacacagatgtaatatacaacttacttgaa
gaattgcaaaaccaacaagaaaagaatgaacaagaattattgcaattagataaatgggcaagtttgtggacttgg
tttgacataacaaaatggctgtggtatataaaaatagtcataatgataataggaggcttaataggattaagaata
gtttttgttgtactttctatagtgaatagagttaggcagggatactcaccattgtcgtttcaggctgttagcttg
cttaattccactgccatagtagtagctgaggggacagataggattatagaagtagtactaagagcttttagagct
gttcttcacatacctacaagaataagacagggcttagaaagggctttgctataaatgggtggcaagtggtcaaaa
agtagtatggttggatggcctgctgtaagggagagaatgagacgagctgaaccagcagcagagggggtgggagca
gtctctcgagatctggaacgacatggagcgatcacaagtagcaatacagcaaaaaacaatgctgctcttgcctgg
ctagaagcacaagaggaggaggaggtgggttttccagttagacctcaggtacctttaagaccaatgacttacaag
gcagctatagatcttagccactttttaaaagaaaaggggggactggaagggctaatttactcccagaaaagacaa
gatatccttgatctgtgggtttaccacacacaaggctacttccctgattggcagaactacacaccagggccaggg
accagattcccactgacctttggatggtgcttcaaactagtaccagttgagccagagaaagtagaagcagccaat
gaaggagagaacaactgcttgttacaccctatgagcctgcatgggatggaggactcggagggagaagtgctacag
tggaagtttgacagccgcctagcgcttcgtcacatggcccgagagaagcatccggagtactacaaggactgctga
>#95 KP109514 7356 nt
atgggtgcgagagcgtcagtattaagcgggggagaattagatagatgggaaagaattcggttaaggccaggagga
aggaaaaaatatcaattaaaacatatattatgggcaagcagggagctagaacgatacgcacttaatcctggcctg
ttagaaacatcagatggctgtaaacaaatattggcacaactacaaccagcccttcagacaggatcagaaggattt
agatcattattcaacacagtagcagtactctattgtgtgcatcaaaagatagagataaaagacaccaaagaagct
ttagataagatagaggaagagcaaaacaaatttaagaaaaaggcacaacaagcagcagctgacacaggaaacagc
agccaggtcagccaaaattaccctatagtgcagaacatgcaggggcaaatggtacatcagcccatatcaccaaga
actttgaatgcatgggtaaaagtagtagaagagaaggctttcagcccagaagtaatacccatgttttcagcattg
tcagaaggagccaccccacaagatttaaacacaatgctaaacacagtgggggggacatcaagcagccatgcaaatg
ttaaaagagaccatcaatgaggaagctgcagaatgggatagattacatccagtggctgcagggcctgttgcacca
ggccagctgagagacccaaggggaagtgacatagcaggaactactagtacccttcaggaacaaataggatggatg

acacataatccacctatcccagtaggagagatctataaaagatggataatcctgggattaaataaaatagtaagg
atgtatagtcctaccagcattctggacataaaacaaggaccaaaagaaccctttagagactatgtggaccggttc
tataaagttctaagagccgagcaagcatcacaggatgtgaaaaattggatgacagaaaccttgttggtccaaaat
gcaaacccagattgtaaaactattttaaaagcattaggaccagcagctaccctagaagaaatgatgacagcatgt
cagggagtgggaggacccagccataaggcaagaattttggcagaagcaatgagccaagtaacaaacccaactacc
atactgatgcagagaggcaattttaagaaccaaaaaagaattgttaagtgtttcaattgtggcaaagaagggcat
atagccaaaaattgcagggcccctagaaaaagggctgctggaaatgtggaaaggagggacaccaaatgaaggac
tgcactgagagacaggctataggggggcaactaaaggaagctctcttagatacaggggcagatgatacagtatta
gaggaaatgaatttaccaggaagatggaaaccaaaaatgataggggggaattggaggttttatcaaagtaagacag
tatgatcagatatccatagaaatctgtgggcataaagctataggtacagtgttagtaggacctacacctgtcaac
ataattggaagaaacttgttgactcagattgggtgcactttaaattttcccattagtcctattgaaactgtacca
gtaaaattaaaaccaggaatggatggcccaaaagttaaacaatggccattgacagaagaaaaaataaaagcacta
acagaaatttgtgcagagatggaaaaggaagggaaaatttcaaaaattgggcctgaaaatccatataatactcca
atatttgccataaagaaaaaagacagtactaaatggagaaaattagtagatttcagagaactcaataagagaact
caagacttctgggaagtgcagttaggaataccacatcctgcagggttaaaaaagaaaaaatcagtaacagtactg
gatgtgggtgatgcatatttttcagttcccttagataaagaattcagaaagtatactgcatttaccatacctagt
ataaacaatgagacaccaggggttagatatcagtacaatgtgctcccacagggatggaaaggatcaccagcaata
ttccaaagtagtatgacaaaaattttagatccttttagaaaacaaaatccagacctagttatctatcaatatatg
gatgatttgtatgtaggatctgacttagaaataggacagcatagaacaaaaatagaggaactaagacaacatctg
ttgaggtggggacttaccaccccagacaaaaaacatcagaaagaacctccatttctttggatgggttatgaactc
catcctgataaatggacagtacagcctatagtgctgccagaaaaagacagttggactgtcaatgacatacagaag
ttagtgggaaaattaaattgggcaagtcagatctactcagggattaaagtaaaacagttatgtaaactccttagg
ggaactaaatcactaacagaaataataccactaacagaagaagcagagttagaactggcagaaaacagggagatt
ctaaaaacaccagtacatggagcgtattatgacccatcaaaagacttaatagcagaaatacagaagcaaggggaa
ggccaatggacatatcaaatttatcaggaaccttttaaaaatctgaaaacaggaaaatatgcaagaatgaggggt
gcccacactaatgatgtaaaactattaacagaggcagtgcaaaagatagccacagaaagcatagtaatatgggga
aagactcctaaatttagactacccatacaaaaagaaacatgggaaacatggtggacagagtattggcaagccacc
tggattcctgagtgggagtttgtcaatacccctcccttagtgaaattatggtaccagttagagaaagaacccata
gtaggagcagaaactttctatgtagatggggcagctaacagggaaactaaattaggaaaagcaggatatgtaact
gacaaaggaagacaaaaggttgtctccctaaatgacacaacaaatcagaagactgagttacaggcaattcatcta
gctttgcaggattcaggattagaagtaaacatagtaacagattcccaatatgcattgggaatcattcaagcacaa
ccagataaaagtgaatcagagttagtcaatcaaataatagagcagttaataaaaaaggaaaaggtctacctagca
tgggtgccagcacacaaaggaattggaggaaatgaacaagtagataaattagtcagtgctggaattaggaaaata
ctatttttagatggaatagataaggcccaagaagaacatgagaaatatcacaataattggagagcaatggctagt
gattttaacataccacctgtagtagccaaagaaatagtagccagctgtgataaatgtcagctaaaaggagaagcc
atgcatggacaagtagactgtagtccagaaatatggcaactagattgtacacacttagaaggaaaagttatcctg
gtagcagttcatgtagccagtggatatatagaagcagaagttatcccagcagagacagggcaagaaacagcatac
ttcatcttaaaattagcaggaagatggccagtaaaaacaatacatacagataatggcagcaatttcaccagtgct
gcggttaaggccgcctgttggtgggcggggatcaaacaggaatttggcattccctacaatccccaaagtcaaggg
gtagtagaatctctaaacaaagaattaaagaaaattgtaggacaagtaagagatcaggctgaacatcttaagaca
gcagtacaaatggcagtattcatccacaattttaagagaaaaggggggattggggggatacagtgcaggggaaaga
atagtagacataatagcaacagacatacagactaaagaattacaaaaacaaattacaaaaattcaaaattttcgg
gtttattacagggacagcagagatccactttggaaaggaccagcaaagcttctctggaaaggtgaaggggcagta
gtaatacaagataatagtgacataaaagtagtgccaagaaggaaagcgaaaatcattagggatacatggcacagt
ttagtaaaacaccatatacatgtttcaaagaaagctcaggatggatttatagacatcattatgaaaacactcat
ccaaaaataagttcagaagtacacattccaataggggaagctaaattggtgataaagacatattggggtctgcat
acaggagaaagagactggcatttgggtcagggagtctccatagaatggaggaaaaggagatataacacacaagta
gaccctggtttagcagaccaactaattcatctgtatcactttgattgtttttcagaatctgctataagaaatgcc
atattaggaaatagagttagtcctaggtgtgaatatcaagcaggacataacaaggtaggatctctacaatatttg
gcactaacagcattaataacaccaaagaagacaaagccacctttgcctagcgttacaaaactgacagaggatctt
ttagaggagcttaagcatgaagctgttaggcattttcctagaccatggcttcatggcctagggcaacatatctat
gaaacttatggaatacttgggcaggagtggaggctataataagaatactgcaacaactgctgtttattcatttc
agaattgggtgtcaacatagcagaataggcattattccacagagaagagcaagactagagccttggaagcatcca
ggaagtcagcctaaaactgcttgtaacaattgctattgtaaaaagtgttgctttcactgccaagtttgtttcaca
aaaaaaggcttaggcatctccgctgaggaattgtgggtcacagtctattatggggtacctgtgtggaaagaagca

gtcaccactctattttgtgcatcagatgctaaagcatatgacaaagaggtacataatgtttgggccacacatgcc
tgtgtacccacagaccccaacccacaagaaatagaattggaaaatgtgacagaaaattttgacatgtggaacaat
aacatggtagaccagatgcatgaggatataattagtttatgggatcaaagcctaaaaccatgtgtaaaattaacc
ccagtctgtgttactttacattgctgtaagttgaatgatactaagtgccatgatttgaatgtcactaatagtact
acccctgaaggtaacaataccactaatattactaacaatggtggtgatgatttaagaaattgctctttcaatatg
accacaataatgacagagaagatgcagaaaaattatgcactttctatacatctgatatagtacctatagataat
acagataatactagtgactataggttgataagttgtaacacctcaatccttacacaagcctgtccaaaggtatcc
tttgagccaattccaatacattattgtgccccggctggttttgcaatcttaaagtgtaaagacaagacattcaat
ggaacaggaccatgtacaaatgtcagcacagtacaatgtacacatggaattaggccagtagtatcaactcaattg
ttgttaaatggcagtctagcagaggaagacatagtaattagatctaaaaatttctcggacaatgctaaaaccata
atagtacagctgaatgaatctataccaattgtgtgtgtaagacccaacaacaatacaagaaaaagtatatctata
ggaccagggagaacatggtatgcaacaggagacataataggagacataagacaagcatattgtaacattactgtt
gggaattggagtaacactttacaacggatagataaaaaattaaaagaacaatttagaaataaaacaataaacttt
aatcaatcctcaggaggggacccagaaattgtaatgcacagctttaattgtggaggggaatttttctactgtaat
acaacacagctatttaataatacttggcttacttggaaggccaatgcaagtgatcctgaaaaaaataacacaaaa
aatatcacactcccatgcagaataaaacaaattataaacatgtggcaggaagtaggaaaagcaatgtatgcccct
cccatcgcaggacaaattaaatgtgtatcaaatattacagggttactattaacaagagatggggtcaggacaat
aacactaacggtaacgagaccttcagacctggaggaggagatatgagggacaattggaggagtgaattatataaa
tataaagtagtaaaaattgaaccattaggaatagcacccactaaggcaaagagaagagtggtgcagagagaaaaa
gggatttggggttgctctggaaaactcatttgcaccactactgtgccttggaatgctagttggagtaataaatct
ctggataccatttggaataacatgacctggatgcagtgggatagagaaattaacaattacacaagcttaatatac
accttacttgaaaaatcgcagaaccagcaagaaaagaatgaacaagaattattggaattggataagtgggcaaat
ctgtggaattggtttgacataacaaattggctgtggtatataaaaatattcataatgatagtaggaggcttgata
ggtttaagaatagtttttactgtactttctataataaatggagttaggcagggatactcaccattatcgtttcag
gcaattagcttgatcaacgccgcagcagtagcagtagctgaggggacaggtaggccacgcacctagatgggtagc
aagtggtcaaaattgggtggatggcctactgtaagagagagaatgagacgagctcaaccagctcaaccagcagca
gatggggtgggagcagcatcgcgagacctggaaaaacatggagcaataacaagtagtaatacagcagctaacaat
cctgacagtgcctggctagaagcacaagaggaagagcaggaggtgggctttccagtcagacctcaggtaccatta
agaccaatgacttacaagggagctttagatcttagccactttttaagagaaaaggggggactggaagggctaatt
tactcccagaaaagaagagacatccttgatctgtgggtctacaacacacaaggctacttccctgattggcagaac
tacacaccagggccagggactaggtggccattaacctttggatggtgcttcaagttagtaccagtggagccagag
aacagtgaaggagagaccaacagtttgctacaccctgcaagcctgcatgggatggaggacccagagggagaagta
ttagtgtggaagtttgacagccgcctagccttccatcacatggcccgcgagctgcatccggagtactacaaggac
tgctga
>#96 AF146728 7425 nt
atgggtgcgagagcgtcagtattaagcgggggagaattggatagatgggagaaaattcggttaaggccaggggga
aagaaaacatataaattaaaacatatagtatgggcaagcagggaactagaacgattcgcagttaatcctggcctg
ttagaaacatcagaaggctgtagacaaatactggtacagctacaaccatcccttccgacaggatcagaagaactt
aaatcattatttaatacagtagcaaccctctattgtgtgcatcaaaagatagagataagagacaccaaggaagct
ttagaaaagatagaggaagagcaaaacaaaagtaagaaaaaggcacagcaagcagcagctgacgcagcagcagct
gatgcaggaaatagcagcaaggtcagccaaaattaccctatagtgcagaacctccaggggcaaatggtacatcaa
cccatatcacctagaactttaaatgcatgggtaaaagtagtagaagagaaggctttcagcccagaagtaataccc
atgttttcagcattatcagaaggagccaccccacaagatttaaataccatgctaaacacagtaggggggacatcaa
gcagcagccatgcaaatgttaaaagagaccatcaatgaggaagctgcagactgggatagattacatccagtacat
gcagggcctattgcaccaggccagatgagagaaccaaggggaagtgacatagctggaactactagtacccttcag
gaacaaataggatggatgacaaataatccacctatcccagtgggagaaatctataaaagatggataattctggga
ttaaataagatagtaagaatgtatagccctaccagcattctggacataaaacaaggaccaaaggaaccctttaga
gattatgtagaccggttctatagaactctaagagccgagcaggcttcgcaggaggtaaaaaattggatgacagaa
accttgttggtccaaaatgcgaacccagattgtaggactattttaaaagcattgggaccagcagctacactagaa
gaaatgatgacagcatgtcagggagtaggggggacccggccataaagcaagagttttggccgaagcaatgagccaa
gtaacaaattcagctaccataatgatgcagagaggcaattttaggaaccaaagaaagactgtcaagtgtttcaat
tgtggcaaagagggccacatagccagaaattgcagggccccgaggaaaaggggctgttggaaatgtggaaaggaa
ggacaccaaatgaaagattgtactgagagacaggctatagggggcaactaaaggaagctctattagatacagga
gcagatgatacagtattagaagaaatgtgtttaccaggaagatggaaaccaaaaatgataggggggaattggaggt
tttatcaaagtaagacagtatgatcaggtattagtagaaatttgtggacataaagctataggtacagtattagta

ggacctacacctgtcaacataattggaagaaatctgttgactcagcttggatgtactttaaattttcccattagt
cctattgagactgtaccagtaaaattaaagccaggaatggatggcccaaaagttaaacaatggccattgacagaa
gaaaaaataaaagcattagtagaaatttgtacagaaatggaaaaggaaggaaaaatttcaaaaattgggcctgaa
aatccatacaatactccagtatttgccataaagaaaaaagacagtactaaatggagaaaattagtagatttcaga
gaacttaataagagaactcaagacttctgggaagttcaattaggaataccacatcccgcagggttaaaaaagaaa
aaatcagtaacagtactggatgtgggcgatgcatatttttcagttcccttagataaagacttcagaaagtatacc
gcattcaccatacctagtataaacaatgagacaccagggattagatatcagtacaatgtgcttccacagggatgg
aaaggatcaccagcgatattccaaagtagcatgacaaaaatcttagagccttttagaaacaaaatccagacgta
gttatctatcaatacatggatgatttgtatgtaggatctgacttagaaatagggcagcacagaacaaaaatagag
gaactgagacaacatctgttgaggtggggtttaccacaccagacaaaaaacatcagaaagaacctccattcctt
tggatgggttatgaactccatcctgataaatggacagtacagcctatagtgctgccagaaaaagacagctggact
gtcaatgacatacagaaattagtgggaaaattaaattgggcaagtcagatttattcagggattaaagtaagacaa
ttatgtaaacttcttaggggaaccaaggcactaacagaagtaataccactaacagaagaggcagagctagaatta
gcagaaaacagggagattctaaaagaaccagtacatggagtgtattatgacccatcaaaagatttaatagcagag
gtacagaagcaggagcaaggccaatggacatatcaaatttatcaagaaccatttaaaaatctgaaaacaggaaag
tatgcaagaatgaagggtgcccacactaatgatgtaaaacagttaacagaggcagtgcaaaaaatagccacagaa
agcatagtaatatggggaaagactcctaaatttaagctacccatacaaaaggagacatggaagcatggtggatg
gagtattggcaggccacctggattcctgagtgggagtttgtcaatacccctcccttagtgaaattatggtaccag
ttagagaaagaacccatagtaggagcagaaactttctatgtagatggagcagctaatagggagaccaaattagga
aaagcaggatatgttactgacagaggaagacaaaaggttgtcccccctaagtgacacaacaaatcagaagactgag
ttacaagcaattcagctagcgttgcaggattcgggattagaagtaaacatagtaacagactcacaatatgcatta
ggaatcattcaagcacaaccagataagagtgaatcagagttagtcaatcaaataatagagcagctaataaaaaag
gaaaagatctatttggcatgggtaccagcacacaaaggaattggtggaaatgataaggtagatagtttggtcagt
gctgggatcaggaaagtactattcttggatggaatagataaggcccaagatgatcatgagatatatcacagtaat
tggagggcaatggctaatgattttacctgccacctatagtagcaaaagatatagtagccagctgtgataagtgt
cagcaaaaaggagaagccatgcatggccaagtagactgtagtccaggtttatggcaactagattgtacacattta
gaaggaaaagttatcctggtagcagtccatgtagccagtgggtatatagaagcagaagtcattccagcagagaca
gggcaggaaacagcatatttctattaaaattagcaggacgatggccagtaaaaacagtacatacagacaatggc
cccattttcatcagtactgcggttaaggccgcctgttggtgggcagggattaagcaggaatttggcattccctac
aatccccaacgtcaaggagtggtagactctatgaataacgacttaacgacaattataggacaggtaagagatcag
gctgaacatcttaagacagcagtacaaatggcagtattcatccacaattttaaaagaaaaggggggattgggggg
tacagtgcaggggaaagaataatagacataatagcatcagacatacaaaccaaagaattacaaaaacaaattaca
aaagttcaaaatttcgggtttattacagggacagcagagatccactttggaaaggaccagcaaagctcctctgg
aaaggtgaaggggcagtagtaatacaagataatagtgacataaaagtagtgccaagaagaaaagcaaagattatt
agggatacatggaaaagtttagtaaaacaccatctatataagtcagggaaagctaggagatgggtttatagacat
cactatgaaagcactcatccaagaataagttcagaagtacacatccctctaggggaagctagattggtaataaca
acatattggggtctgcatacaggagaaagagactggcatttaggccagggagtctccatagaatggagaaaaaga
agatatagcacacaagtagaccctggcctagcagaccaactaattcatatgtattattttgattgtttttcagaa
tctgctataagaaatgccatattagaacgtatagttagtcctagttgtgaacatcaagcaggacataacaaggta
ggatctttacagtacttagcactagcagcattaataacaccaagacagacaaagccacctttgcctagtgtcacg
aaactgacagaggatcttttagaggaactcaagagtgaagctgtcagacattttcctaggatatggctacatggc
ctagggcagcatatctataacacctatggggatacttgggcaggagtggaagccttaataagaagtctgcaacaa
ctgctgtttattcatttcagaattgggtgtcgacacagcagaataggcattaatcctcagaggagagcaagacta
gagccctggaagcatccaggaagtcagcctaagactgcttgtaccaattgctattgtaaacaatgttgctttcat
tgccaagtttgcttcatacgaaaaggcttaggcatctccgcaacagaaaaattgtgggtcacagtctattatggg
gtacctgtgtggaaagaagcaaccaccactctattttgtgcatcagatgctaaagcatatgataaagaggtacat
aatgtttgggccacacatgcttgtgtacccacagaccccaacccacaagaaatattactagaaaatgtgacagaa
gagtttaacatgtggaaaaataacatggtagaacagatgcatgaggatataatcagtttatgggatcaaagccta
aagccatgtgtacaattaaccccactctgtgttactttaaattgcactgatgagttgacgaatgttacttttact
aatagtaggcatgtgactaatagtagttatgtgggaagtatggaaaaaggagaaatgaaaaactgctctttcaac
atcaccacaagcataagagataagaggcacaaagaatttgcactttttttataaacttgatgtagtacaaatagat
ggtagtaatactagctatagattaataaattgtaatacctcagtcattacacaggcctgtccaaaggtatccttt
gagccaattcccatacattattgtgccccggctggttttgcgattctaaagtgtaacaataagacgttcaatgga
aaaggaccatgtgcaaatatcagcacagtacaatgtacacatggaattaggccagtagtgtcaactcaactgttg
ttaaatggcagtctagcagaaaaagagatagtaattagatctgacaatttcacggacaatgctaaaagcataata

gtacagctgaatgaatctgtagaaattcattgtatgagacccaacaacaatacaagaaaagggatatatgtagga
ccagggagacacatctatgcaacagagaaaatagtaggagatataagacaagcacattgtaacatcagtagaaca
aactggactagcgtgctaagacagatagctgtaaaattaagagaacgatttaagaataaaacaatagtctttaat
cactcctctggaggggacccagaaattgtaaggcacagttttaattgtggaggggaatttttctactgtaattca
acacaactgtttaatagtacttggtttaatagtactgggaatgatactgaaagggcaactaacaatacagaaaat
atcacactcccatgtagaataaaacaaattataaacatgtggcagaaagtaggaaaagcaatgtatgcccctccc
atcaacggacagattagatgttcatcaaatattacagggctgatactaacaagagatggtggtaatcaagagaac
aagaccgagatcttcagacctggaggaggagatatgagggacaattggagaagtgaattatataaatataaagta
gtaagaattgaaccattaggagtagcacccaccaaggcaaagagaagagtggtgcagagagaaaaaagagcaggg
atttggggttgctctggaaaactcatttgcaccacttctgtgccttggaatgctagttggagtaataaatctcta
agtgacatttgggataacatgacctggatgcagtgggaaagagaaattggcaattacacaggcttaatatatcat
ttacttgaagaatcgcagaaccaacaagaaaagaatgaacaagaattattggcattagataagtgggcaagtttg
tggaattggtttagcataacaaaatggctgtggtatataaaaatattcataatgatagtaggaggcttagtaggt
ttaagaatagtttttgctgtactttctctagtgaagaaagttagggagggatactcaccattgtcattgcaggct
attagcttgctcaacgccacagctatagcagtagctgagggaacagatagggttatagaagtagtacaaagagct
tgtagagctattctccacatacctagaagaatcagacagggcttggaaaggcttttactataaatgggtggcaag
gggtcaaaacgtattaggtctgaatggcctactgtaagggaaagaataatacaagctgagccagcagcagctggg
gtgggagcagcatctcgagacctggaaaaacatggagcaatcacaagcagcaatattaataacgctgattgtgtt
tggctacaagcacaggaggaggaggaggtgggttttccagtcagacctcaggtacccttaagaccaatgactttc
aaggcagctcacgatcttagcttctttttaaaagaacaggggggactggaagggctaatttactcccaaagaaga
caagatatccttgatctatggatctatcacacacaaggctacttccctgattggcagaattacacaccagggcca
gggacccgatatccactgacctttggatggtgcttcaagttagtaccagttgagccagatcaggtagaaaaggcc
aatgaaggagagaacatcagtttgttacaccctatgagcctgcacgggatggaggacaaagagaaagaagtgtta
atgtggaaatttgacagccgcctagcagttcatcacatggcccgagagctgcatccggagtattacaaaaactga

Supplementary File S3. Dataset of 96 nucleotide sequences of the gene *gag* from primate lentiviruses: 37 sequences from SIV infecting Old-World monkeys (#1 - #37), 4 from HIV-2 (#38 - #41), 20 from SIV infecting African apes (7 from SIVgor, #42 - #48, 13 from SIVcpz, #49 - #61), and 35 from HIV-1 (9 from group O, #62 - #70, 2 from group P, #71 and #72, 7 from group N, #73 - #79, and 17 from the pandemic group M, #80 - #96). From each genome sequence we selected the non-overlapping region that encodes the Gag protein. Each nucleotide sequence contains the NCBI ac. number and the length of the coding region.

```
>#1  AF468658  1326 nt
atgggagcgaggcactcggcgatgctgacgggtactaagttggataggtatgagaaagtgagtctcagaccgaga
ggaaagaagaagtacgcgatcaagcacttagtatgggcctccaaggagcttgagagatacgcgctatcaggcagt
cttcttgaatctaaagaagggtgtcaacaggtattggaagtcacctccccattagaaccaaatgggtccgaatct
ctcaaaagcctctatggaatcgtgtcagtgctctattgtatccatgcagacatatgggtagaagatacagagcag
gctaagaagcaagttcagataaggtgtcatctagtaggaacaaaagagaaagaaacacagaacaaagacccccca
ggggccgcaggggacaggcggtagttagtcagaactaccccatagttagaaatgctcagggacagtttcaacac
caggccctcaacagcagagtccttaagacttgggtaagcatagtggaagaaaagaagtttgctccagagacagtc
gctctgtttcaggctctaacagaaggctgtataccttatgatatgaaccaaatgctcaacgctgttggagactat
caaggagcagtacagattattaaagatgtcatcaatgaacaggcagcagagtgggatctcttacatccgcagccc
gcggcaccccaaccagtagcagggcttagagaccctagcggagcagacatagcaggagtaacatcaactcctaat
gagcagatagagtggattacaaggcagaacaatccagttaatgtggcagatatatatagaaaatggatcatcttg
ggcttgcagcgatgtgtaaaaatgtataaccctgtcaatattttgggcatcaaacagggccctaaggaaccattt
aaggaatatgtagacagattctttaaatgcttaagggctgaacaggcagatcaggcagtaaagaattggatgaca
caattgctgctggttcaaaatgctaatccagaatgcaagctgatcttgaaggccatgccaggagccagcttggaa
gaaatgcttacagcatgccaaggggtaggggaccaactcataaatctagactcttggcagaggccatggcaaca
gcactcaaaggcagctcatacaatatggtgcaaagaggcccccaggaaggcaaggaaaaaccccaaaatgttac
aattgtggaaagtttggacatatcgcaagagactgtcccaaacccaaggaaagaaagtgttttaaatgtggcaag
gcaggacacttagctaggcaatgcaaaacaggaacaccagccaaggagctt
>#2  AF468659  1329 nt
atgggagcgaggcactcggcgatgctgacaggtactaagttggataggtatgagaaagtgcgtctcagaccaaag
ggaaagaaaaagtacatgatcaagcacttagtatgggcctccaaagagcttgagagatatgcgctgtccgacgct
cttcttgaaaatcaagaggggtgtcgacggatattagaaattgtcttcccccctagaaccaacagggtcagaagct
ctcaaatctctctttggaatcgtgtcagtgctctattgtatccatgcagacatacaggtggaggatacagagcag
gctaagcagcaagtcaggataagatgtcatctagaggagaccaagggtaaagaaaagcagaataaagacccccccg
ggggccgcaggggacaggcggtagttagtcaaaactaccctgtgattaggaatgcccaaggacagtatcaacat
caggccctcaatagtagaattcttaagacttgggtaagcatagtggaggagaaaaagtttgctccagagacagtt
gccttgtttcaggctttatcagaaggctgtataccctatgatatgaaccaaatgctcaatgctataggagattat
caaggggcagtacagattattaaagatgtcatcaatgaacaggcagcagagtgggaccttttacatccggtacca
gcagcacctcaaccggtggcagggcttagagaccctagcggagcagacatagcaggagttacttcaactcccaat
gagcagatagagtggatcacaaggcagaaccaacctgtcaatgtgtcagatatatatagaaagtggatcattttg
ggcctgcagcgatgtgtaaaaatgtataaccctgtcaatattttggacatcaaacaaggccctaaagagcccttt
aaggaatatgtagacagattctttaagtgcttaagggccgaacaagcagatcaggccgtgaaaaattggatgaca
caatcgctgctggttcaaaatgctaacccagaatgcaagctgatcttgaaagccatgccaggagccaacttggaa
gaaatgcttacagcatgccaaggggtaggggaccaactcacaaatctaggctcttagcagaagccatggcaaca
gccctcaaaggcactagttcatacaacatggtgcagaaaggccccctggaaggcaaggaaaaaccccaaaatgt
tataattgtggacaattcggacacctagcaagagattgtcctaaacccaaagaaagaaagtgctttaaatgtggc
agagcagggcactttagcaaaccatgtagaacagggacaccaatcaaggagctt
>#3  EF070329  1335 nt
```

atggggcgaggcactcggcgatgctgaaaggtactaagttgaataaatatgagaaggtcagattaaggcctaaa
ggaaagaagaagtacctaataaagcatatagtatgggcttccagagagcttgagcggttcggtctctcagatacg
ctcctagagagcaaggacggatgccaacgcatcctagaagtaatctgccccttggaagaaactgggagcgaggcg
ctcaaatcactctttggaatcgtgtcagtactctggtgtatccatgcagacgtaacagtgaaagacacagaggag
gctaaaaagcaaattaggataaggtgccacctagcggagaaaaagaagactcaaaaggagactcaggagcaaaa
gccaccaccagtggccagaattacccagtcataagaacagcacagggacagtatcagcatcagtctcttagccct
aggctattaaaaacctgggtatcaacagtagaggagaaaaatttgccccagaagtagtggccctgttccaggcc
ctatcagaaggatgcatcccctatgatgtaaatcagatgctcaacgccataggagaccaccaggggcaatacaa
ctaataaaagatgtggtcaatgagcaggcagcagacagagacgttttacatccccaaccaacacagccacaacca
aatgcagggctaaggtacccatcaggggctgatgtggcgggagtgtcatcaacgccagcagaacaaatagaatgg
atgactagacagcaagacccagtaaatgtagggaatatatatagaaaatggatcatcttgggcctacagagatgt
gtcaaaatgtacaatccagtaaacatcttggatattaaacaaggccctaaagaaccattcaaagattatgtggat
cggttctttaaatgcttaagagctgaacaggctgaccaagcagtcaaaaattggatgacacagtccctgctagtg
caaaatgccaaccccgagtgcaaactcgtcttgaaagccatgccaggggccacattaaaagagatgttacaggcc
tgccaggggattgagggacccatgcacaagtcaaggctgatggcagaagccatgactaatgctttgaggcaaaac
accattaacaccatcaacatggtacaaagacagagtcccaggggagtaatgggaaaaaagagggaaaacagcaca
cgttgctacaactgtggacaatttggacatttggcaagagattgccccaagcctaagtctaccagatgttttaag
tgtggtaaggaaggacacttggccagacagtgccgaaccgacacaggcaaaagcgctgtg
>#4 EF070330 1311 nt
atgggagcgaggcactcggcgatgctgacaggtaccaagttggataggtacgaaaaggtgagactaagaccaaaa
ggaaagaagaggtacctaatcaagcacatagtatgggcttccaaggagcttgaaaggtttggactctcggactcc
cttttggagacaaaagagggatgtcaaaagatcttagaagttctcataccattagaaccaacagggtccgaatct
ctcaaatcactctttggaatcacgtcagtgctctggtgtatccatgcagaggtggaggtggaagacacagagcag
gctaggaagcaaatcaggataaggtgtcatctggcggacaagcaaggggaagaaaagacagcacagaatcaaact
agtgctaattaccccatcataaggaacccacagggacaatttcaacaccagtcccttaatgctagaatcttaaag
acatgggtgtcaatagtggaagaaaagaaatttgcagcagaagtagtggcaatgtttcaagcactagcagaagga
acaataccctatgacatgaatcagttgctaaatgccataggggatcatcaggggctatacagatcataaaagat
gtcataaatgagcaagcagcagaatgggatctgctacacccacagcctcagcagccgcagcccaatgcaggcctg
cggagcccatcaggatcagacatagcaggggtaacatccacccccaatgaacaaatagaatggataactaggcaa
aacgatcctataaatgtggcagacatctataagaaatggataatcatggggttacagaggtgtgtcaaaatgtat
aaccctgttagtatattggatatcaagcagggaccaaaagagccatttaaggattatgtggacaggttctacaaa
tgcttaagggcggagcaatcagatcaagcagttaagaattggatgacctccactttgttagtgcagaatgctaac
ccagagtgtaagctcatcctgaaaagcatgccaggagccaccttagaagaaatgttgcaggcatgccaaggggta
ggagggccagcacataagtccaggctaatggcagaggctatggcctcggcccttagacaaaacagtcagctgaac
atggtgcaaggagccagagggaaaggcagccagggaggtcccaggggaaaccctaggtgttacaactgtgggcag
tttggacacatggcaagaagctgccccaagccaaaaactaggaaatgcttcaaatgtggaagggagggacacttg
gcaaaacagtgcaggtctgaaggtgccaaatcggct
>#5 AY340701 1323 nt
atggggcgaggcactcggcgatgcttagtggtactaaattagacaagtatgagaaggtgcggcttagaccgcgc
ggtaagaagaagtacctgattaagcatatagtatgggctgccaaggagcttgatagattcggtctctcggactcg
ctcctggagacacaggacggctgtaagaagattttggaagttattcttcctctacagccgacaggatccgagtct
atcaaatctctctttggtatcgcttcggtattgtattgcatacatgccggtatagaaattgaggacacagaacag
gctaagcagcaagtaaaaatcaggtgtcacttagcaggggaacagggagaacagaaagctgcagccgcggccgct
ccaccaacaggcggcgtacccagtggaaactaccctgtcgtgagaacacagggggaggcttccaacatcaggca
gtggagcctcggctcctcaagacatgggtacaggtcattgaggagaagaaatttgccccagaagtggtggccctc
ttccaggctctatcagaaggcatgattccctatgacatcaatcagctactaaacgccataggtgatcatcagggg
gcactccaaattattaaggatgtcatcaatgaggaagcggccaattgggacttagtgcacccacagccgccacaa
ccgcaacccaatgcagggctgggagacccaacgggggccgatatagcaggagtctcgtccacaccccaacagcag
atagagtggataaccagggctaacaaccccgttcaggtctcagacatctacagaaaatgggtcatcttgggcctg
cagcgatgcgtcaaaatgtacaaccctgtcaacattttggatatcaaacagggacctaaagaaccattcaaagat
tatgtagacagattctacaaatgcctcagagcagaacaaacagatcaggcagtcaaaaattggatgacgcagacg
ctgctggtccaaaatgccaacccagattgcaaactcatcctaaaggccatgccaggtgctagcttggaggacatg
ctacaggcatgtcagggagtgggaggtccaatgcacaaatctaggattctggcagaagctatggcaggggcaata
gccaacatgcccatgaatatggtacaggccagaggaccaccccaaaggaagggacaacccaaatgcttcaattgt

ggaaaatttgggcacatggcgaaaaattgcaaagccccccaaaggaggaaatgttacaattgtggccaaccggga
cacctcgccaaagactgcccgcagccccccaaacaaaacaaaggggtg
>#6 KJ461716 1326 nt
atgggggcgaggcactcagcgatgcttactgggaaaaagcttgataagtatgagaagatcaggctaaggccgaaa
ggcaaaaagaagtaccagataaagcatatagtttgggcatccaaagagttggaacgatttggactatcagactcc
ttattggaaaataaggagggctgccaaaagatcttagaggtcattgttccattggaagaaacagggtccgagtct
ctaaaatccctctttggattagtttcagttttgtggtgtgtacatgcagatgttacagtagaagacacagaggaa
gctaaaaaacagatcagaattaggtgccacatgagtggcaagaaggaggagacctccgcgggcggaggtgcctca
ggacaaaactatccaattgttaggaatgcacagggacagcatcagcatcaggctttgtccccaagaatcctcaag
acatggataacaaccattgaggagaaaaaattttcagcagaagtagttgccctattccaggcactgtcagaaggc
tgtataccctatgatataaatcagctcatgaatgccataggggaacatcaaggagcggtacagcttatcaaagat
gtagttaatgaggaagcagccgcttgggatcagttgcacccacagccagcacagccgcagcccaacgcaggcttg
agatacccatcagggagtgacattgcaggggtatcatcatccatacaagaacagctagaatggatgacaagagca
gtagacccagtaaatgtaggagaaatatacagaaaatggatagtaatgggtctgcagagatgtgtcaaaatgtac
aacccagttaacattctggacataagacaagggccaaaagagagcttcaaagactatgtagaccgattctacaaa
tgtctgagagcagaacaggctgatcaatcagtcaaaaattggatgactacaaccctcttggtccagaatgctaac
ccagaatgcaaggccatcttaaaggccatgccaggagccagcttagaagaaatgcttacagcatgccaaggcgta
ggagggccacactataagaccaagctgttagcagaagccatggcatcagctttccaaaagacaggaaattacaac
atggtgcagggcccacgaggcccgaaaggggctcgcagggggccaatgaaaaaccctaatcctaggtgtttcaat
tgtggacagttcggacacatgtccaaacagtgcccacacccgaaaaagataaagtgctttaagtgtggcagagag
ggccatatggcaagacagtgtcgagcctcagaaggtgcacagggaggagca
>#7 KJ461714 1326 nt
atgggggcgaggcactcagcgatgcttactgggaaaaagcttgataagtatgagaaaatcaggctaaggccgaaa
ggcaagaaaaggtaccagataaagcatatagtttgggcatccaaagagctggaacgatttggactatcagactcc
ttattggaaaataaggagggctgccaaaagatcttagaggtcattgttccattggaagacacagggtccgagtct
ctaaaatccctctttggattagtttcggttttgtggtgtgtacatgcagatgttacagtagaggacacagaggaa
gctaaaaaacagatcagaattaggtgccatatgagtggcaagaaggaggaaacctccgcgggcggaggtgcctca
gggcaaaattatccaattgttaggaatgcacagggacagcatcagcatcaggctttgtccccaagaatcctcaag
acatggataacaaccattgaggagaaaaaattttcagcagaagtagttgctttgtttcaggcactatcagaaggc
tgtataccctatgatataaatcagctcatgaatgccataggggaacatcaaggagcagtacagcttatcaaagat
gtagttaatgaggaagcagccaattgggaccagctgcacccacagccagtacagccgcagcccaacgcaggctta
agatacccatcagggagtgacatagcaggggtatcatcatccatacaggaacagctagaatggatgaccagaaca
gtagaccaagtaaatgtgggagaaatatacagaaaatggatagtaatgggtctgcagagatgtgtcaaaatgtac
aatccagttaacattctggacataagacaagggccaaaagaaagcttcaaagactatgtagacagattctacaaa
tgtctgagagcagaacaggctgatcagtcagtcaaaaattggatgactacaaccctcttggtccagaatgctaac
ccagaatgcaaggccattctaaaggccatgccaggagccagcttagaagaaatgcttacagcatgccaaggcgta
ggagggccacactataagaccaagctgttagcagaagccatggcatcagctttccaaaagacagggaattacaac
atggtgcaaggcccacgaggcccgaaaggggctcgcaggggaccaatgaaaaaccctaaccctaggtgtttcaat
tgtgggcagttcggacacatgtccagacagtgcccacagccaagaaagataaagtgctttaagtgtggcagagag
ggccatatggcaagacagtgtcgagcctctgaaggtgcacagggaggagca
>#8 KJ461715 1326 nt
atgggggcgaggcactcagcgatgcttactgggaaaaagcttgataagtatgagaagatcaggctgaggccgaaa
ggcaaaaagaagtaccagataaagcatatagtttgggcatccaaagagctggaacgatttggactatcagactcc
ttattggaaagtaaggagggctgccaaaagatcttagaggtcattatcccattggaagaaacagggtccgagtct
ctaaaatccctctttggattagtttcagtcttatggtgtgtacatgcagatgttacagtagaggacacagaggaa
gctaaaaaacagatcagaattaggtgccacataagtgacaaaaaggaggagacctccgcgggcggaggtgcctca
gggcaaaattatccagttgttaggaatgcacagggacagcatcagcatcaggctttgtccccaagaatcctcaag
acatggataacaaccattgaggagaaaaaattttcagcagaggtagttgccttattccaggcactatcagaaggc
tgtataccctatgacataaatcagctcatgaatgccataggggaacatcaaggagcggtacagcttattaaagat
gtagttaatgaggaagcacacacttgggaccagttgcacccacagccagcacagccgcagcccaacgcaggcttg
agatacccatcagggagtgacatagcaggggtatcatcatccatacaagaacagctagaatggatgacaagggca
gtagaccaagtaaatgtgggagaaatatatagaaaatggatagtaatgggtctgcagagatgtgtcaaaatgtac
aacccagttaacattctggacataagacaagggccaaaagaaagcttcaaagactatgtagaccgattctacaaa
tgtctgagagcagaacaggctgatcaatcagtcaaaaattggatgacttcaacccttttgatccagaatgctaac
ccagaatgcaaggccatcttaaaggccatgccaggagctagcttagaagagatgcttacagcatgccaaggcgta

ggagggccacactataagaccaagctgttagcagaagctatggcatcagctttccaaaggacagggaattacaac
atggcgcaaggcccacaaggcccgaaaggggctcgaagggggcccatgaaaaaccctaatcctaggtgtttcaat
tgtggacgatttggacacatgtccagacaatgcccacagccaaaaaagatgaagtgctttaagtgtggcagagag
ggccatatggcaagacagtgtcgagcctctgacagtgcacagggaggagca
>#9 JX860407 1302 nt
atgggcgcgagaagctccgtcttgtcagggaaaaaagcagatgaattagaaaaggttaggttacggcccggcgga
aagaaaaagtacatgttgaagcatgtagtgtgggcagcaaacgaattggacagattcggattggcagagagcctg
ttggaaarcaaagaaggttgtcaaaarattctttcagttttagctccattaacaccaacagggtcagaaaattta
aagagcctctacaatactgtctgcgtcatytggtgcattcacgcagaagagaaagtgaaacatacygaggaagca
aaacaaatagtgcagagacatctagtggtagaaacaggaactgcagatcccatgccgaaaagyagtagaccaaca
gcaccacctagtggcatgaagggaaattacccagtgcagcaagtrggtggcaattatgtycacctaccattaagy
cccaggacattaaacgcctgggtaaaattagtggargaaaagaaatttggggcagaggtagtgccagggtttcag
gcactrtcagaaggctgtactccttatgatatcaatcagatgctaaattgtgtgggagaacaccargcagccatg
cagatcataagggaaatcatcaatgaagaggcagctgattgggacctgcaacatccacaaccaggtccaatacca
gcaggacagcttagggacccaagaggrtcagatatagcaggaaccaccagcacagtggaagaacaaattcagtgg
atgtacaggcagcagaatccgataccggtaggaaacatctacaggagatggattcaacttgggttgcaaaaatgt
gtgaggatgtataatccaacaaacattctagatgtgaaacagggacccaaagagccatttcaatcttatgtagac
aggttctacaaaagtttgagrgcagarcaaacagatccrgcagtwaagaattggatgactcagacactgctgatt
caaaacgccaacccagaytgtaaattggttttaaagggtctgggaatgaaccccactctagaagaratgctaacr
gcctgtcaaggggtaggggaccaggacaaaaggctaggctaatggcagaagccctgaaagaagccctaacacca
gggccactcccatttgcagcagcccagttaagaggaggaccaaaaagaacagtcaaatgttggaattgtggaaaa
gatggacacactgcaagacagtgtaaagctccaagaagacarggctgctggaaatgtggaaaaatgggccacgtc
atggctaaatgcacagaaagacaggca
>#10 U79412 1383 nt
atgggcgcgagaaactccgtcttgtcagggaagaaagcagatgaattagaaaaaattaggctacgacccggcggg
aagaaaaagtacatgttgaagcatgtagtatgggcagcaaatgaattagatagatttggattagcagacagcctg
ttggagaacaaagaaggatgtcaaaaaatactttcggccttagctccattagtgccaacaggctcagaaaattta
aagagcctttataatactgtctgcgtcatctggtgcattcacgcagaagagaaagtgaaacacactgaggaagca
aaacagatagtgcagagacacctagtggtggaaacaggaacagcagaaactatgccaaaaacaagtagaccaaca
gcaccatctagtggcaaaagaggaaattacccagtacaacaaataggtggtaactatacccacctaccattaagc
ccgagaacattaaatgcctgggtaaaattgatagaggagaagaaatttggagcagaagtagtgccaggatttcag
gcactgtcagaaggctgcatcccctatgacattaatcagatgttaaattgtgtgggagaacatcaagcagctatg
cagattatcagagaaattataaacgaggaggctgcagattgggacctgcagcacccacaacaagctccacaacaa
ggacagcttagggagccgtcaggatcagacattgcaggaacaactagtacagtagatgaacaaatccagtggatg
tacagacaacagaaccccataccagtaggcaacatttataggagatggatccaactggggttgcaaaaatgtgtc
aaaatgtataacccaacaaacattctagatataagacaagggccaaaagagccatttcagagctatgtagacagg
ttctacaaaagcttaagagcagaacaaacagatccagcagtaaagaattggatgactcaaacactgctgattcaa
aatgctaacccagattgcaagctggtgctgaaggggctgggtatgaatcccaccctagaagaaatgctgacggct
tgccaaggagtaggaggaccaggacaaaaggcaaaattaatggcagaagccctgaaagaggcccttgcaccaggg
ccactccctttgcagcagcccaacagaagggaccaagaaagccaattaagtgttggaattgtggaaagaggga
cactctgcaaggcaatgcagagccccaagaagacagggctgctggaaatgtggacaaatgggccatgttatggcc
aaatgcccagacagacaggcaggtttttaggctttggcccatggggaaagaagccccgcaatttccccatggcc
caaatgcatcaggggctgacaccaactgctccc
>#11 JQ864086 1299 nt
atgggcgcgagaaactccgtcttgtcagggaagaaagcagatgaattagaaaaaattaggttacggcccaacgga
aagaaaaagtatatgttgaagcatgtagtatgggcagcaaatgaattggacagattcggattagcagaaagcctg
ttggataacaaagaaggttgtcaaaaaattctttcggttttagctccattagttccgacaggttcagaaaattta
aagagcctttataatactgtctgcgtcatttggtgcattcacgcagaagagaaagtgaaacatactgaggaagca
aaacaaatagtgcagagacatctagtggtggaaacaggaacagcagacagaatgccagtaacaagcagaccaaca
gcaccacctagtggcagaggaggaaattacccagtgcagcaagtaggtggcaattatgtccacctacccttaagt
ccaagaacattaaatgcttgggtaaaattggtagaagagaaaaaatttggggcagaggtagtgccaggatttcaa
gcgctatcagaaggctgcactccctatgatatcaatcaaatgctaaattgtgtaggagaacatcaggcagccatg
caaattattagagagattataaatgaagaagctgccgattgggatttacaacacccaccgccaggtccaccacca
gcagggcaacttagagagccaagaggatcagacattgcaggaactactagtacagtagatgaacaaatccaatgg
atgtacaggcaacaaaaccctataccagtaggcaacatttatagaaggtggatccaattagggctgcagaaatgt

gtaagaatgtataacccaacaaacattttagatgtgaaacaaggaccaaaagagccatttcaaagctacgtagat
agattctataaaagtctaagagcagagcaaacagatccggcagtaaagaattggatgacccaaacactgctgatt
caaaatgctaacccagattgtaaattggtgctcaagggtctgggtatgaatcccactttagaagaaatgctgaca
gcctgtcagggaataggagggccaggacaaaaagctagattaatggcagaagcattgaaagaggcactgagacca
gaccaactcccatttgcagcagtccaacagaaaggacaaaggaggacaatcaagtgttggaattgtggaaaggag
ggacactctgcaagacaatgcagggcccctagaagacagggctgctgggggtgcggaaaaacgggtcatgttatg
gccaaatgccctgaaagacaggcg
>#12 AF334679 1194 nt
atgggcgcgagaggctccgtcttgtcagggaaaaaagcagatgaattagaaaaagttaggttacggcccggcgga
aggaaaaagtacatgcttaagcatattatatgggcagccagagaattagacagatttggatcggcagaaagcctg
ttggaaagcaaagaaggatgtcaaagaatattagcagtactagctccattaatgccaacaggctcggaaaattta
aaaagcttgtttagtactgtctgcgtagtttggtgtctgcacgcagaaatgaaagtgaaagacacagaggaagct
aagaaaacagtacagagccatctagtggtagaaagtggaactgcagaaaaattgccagctcaaagcagaccaaca
gctccacctagtggaggaaactatccagtgcagcaagtaggaaataattatgtgcacactccactgtccccacgg
acactcaatgcgtgggtcaaactggtagaagaaaagaaatttggagcagaagtagtgccaggatttcaggcacta
tcagaaggctgtacaccctatgacattaatcagatgcttaattgtgtaggtgaacatcaggcagccatgcaaatt
atcagagaaataattaatgaggaagcagcagactgggacctacaacacccaagaggacaacagccggcgcaaccg
gctggagggctaagggaaccatcaggatcagacatagcagggacaactagcaccccaagtgaacagatagaatgg
atgtatagggcacaaaacccagtcccagtgggagacatctatagaaggtggatccagctgggactccaaaaatgc
gtcaggatgtataacccaacaaacatcctggatgttaaacaaggtccaaaggagccctttcaaagttatgtagat
aggttctacaaaagtttgagagcagagcagacagatccggcagtgaagaattggatgacacaaacactgctgatt
cagaacgccaaccctgattgcaaactagtattaaaagggttgggcatgaatcccaccctagaagagatgctaaca
gcctgtcaaggggtaggaggcccaggacagaaggcccgcctcatggcagaggccatgaaagacgccctcacgggg
tctttagtggcagcacagtttaggggagcagcgaaaggccaagggaataagcctatcatcaggtgtttt
>#13 JX860414 1305 nt
atgggcgcgagaagctccgtcttgtcagggaaaaagacagatgaattagaaaaggttaggttacggcccaacgga
aagaaaaagtacatgttgaagcatgtagtgtgggcagcgaacgaattggatagatttggattggcagaaagcctg
ttggagaacaaagaaggttgtcaaaaaattctctcggtcttagctccattagtaccaacaggttcagaaaattta
aagagcctttataatactgtctgcgtcatttggtgcatccacgcagaacagaaagtgaaacatacagaggaagca
aaacaaatagtgcagagacatctagtggtggaaacaggaactgcagatacaatgccagccacaagtagaccaaca
gcaccacctagtggcagaggaggaaattaccctgtgcagcaaataggcggcaattatgtccacctacctttaagt
ccaagaacactgaatgcatgggtaaaattggtggaagagaagaaatttggagcggaggtagtgccaggattccag
gcactatcagaaggctgtaccccatatgatattaatcaaatgttaaattgtgtgggggatcatcaggcagccatg
caaattataagagaaatcataaatgaggaagcggctgattgggacctacaacacccacagccgatgggcccaata
ccagcagggcagctcagagacccgaggggatcagacattgcaggaactactagcacagtagaggagcagatagaa
tggatgtataggcagcaaaatcctataccagtaggtaacatctacagaagatggatccagcttgggctacaaaaa
tgtgttagaatgtataatccaacaaacattctagatgtaaagcagggaccaaaagaacctttttcaggcctatgta
gatagattctacaaaagcttaagggcagagcaaacagatccggcagtaaagaattggatgactcaaacgctgctg
attcaaaatgcaaacccagattgtaaagtgatcttaaaaggtctgggtatgaaccccaccctagaggaaatgatg
tcagcctgtcaaggagtgggtggaccaggacaaaaggctaggttaatggcagaggccttaaagagtgctttagca
ccaggagtacttccatttgcagcggcccaacagaaaggaggactgagacatacagtaaagtgctggaattgtgga
aaggagggacactctgcaaaacaatgcagagcccctaggagacaaggctgctggaaatgtggaaaaatgggccat
atcatggctaaatgtccagagagacaggtg
>#14 HM803689 1314 nt
atgggtgcgagagcgtcggttttgtcagggaaaaaactagatgcctgggaacaaattcggcttcgtcccggtagc
aagaaaaagtatatgctgaaacatctagtgtgggcaagtagggagctagagagatttggcctaagtgatacgttg
ttagattcagcagtaggatgtcagaaaattttaggagtcctgttgcctctagttccgacagggtcagaggggcta
aaaagcctctttaatttatgctgcgcactttggtgcgtacattcggaagtgaaagtgagagacacagaggaagct
atacaatatgtaaagaaacagtgccatctagaggacagagaagatagtgcagcagaaaaagaaaaaggtgccaca
gcgacatctagtggccaaaaaggaaattacccaataataacagtaaatcagcagccagagcaccagcccatttca
ccacggacacttaatgcttgggtgaaagtggtagaagagaaaaagtttggggctgaagtggtgcccatgttttcg
gcactttcagaaggatgcataccttatgatataaatcaaatgttaaatgcaataggagaacatcaaggagctttg
caaatagtgaaagaagtcatcaatgaggaagcagcagattgggatgctagacatcctaatctaggaccactacct
gcagggcagcttagagagcctacaggaagcgatattgcaggaacaacaagcagtgttgcagaacagatagcttgg
tcaacaagagctaacaacccaataatagtagggaacatttatagaaattggattattctggggttgcaaaaatgt

gtaaggatgtataacccagtaaatgttctggatattaaacagggaccaaaagagccattcaaggactatgtagat
aggttctataaagccttgagagcagagcaaacagatccagcagtaaaaaattggatgactcagacactgctgatc
cagaatgccaatcctgactgtaaaacagttctaaaaggtctaggcatgaacccctccttggaagaaatgttgcta
gcatgccaaggagttggaggaccccaacacaaggctagagtattggcagaagccatgcagcatgctaatagcatg
atgatggctcaaatgagagggcaaggaaggtcagggccaccaagaagaggtggagaaagaccagtaccaaaatgt
tacaattgtggaaaaacaggacatatttccaggttttgtaaggcccctagaagaaaaggatgctggaagtgtgga
gatgaggatcatcttatgaaggattgtaccaagcaggtt
>#15 HM803690 1314 nt
atgggtgcgagagcgtcaattttgacagggaaaaagctagatgcctgggagcagattcggcttcgccccgggagt
aagaaaaaatatatgttaaaacatctagcatgggcatgtagggaactagagagatttggcttaagtgatacattg
ttagattcagcaggggatgtgaaaaaatcttaggagtccttttgcctctggttccgacagggtcagaggggctg
aaaagcctctttaatttgtgctgcgcactttggtgcgttcacaaggaagtgaaagtgaaagacacagaagaagca
gtgcagtatgttaagaaacagtgccatctagtggagaaagaggatactgcaggagaaaaagaaaaaggtgccaca
atgacatctagtggtcaaagaggaaattaccccatagtaacaattaatcagcagccagaacaccagcctatttct
cccaggacgttgaatgcctgggtgaaggtggttgaggaaaagaagtttggagcagaagtagtgcccatgttttcg
gcactttcagagggctgcataccttatgatgtaaatcaaatgctaaatgccataggagaacatcaaggagcatta
caaatagtgaaagaagtaataaatgatgaagcagcagactgggatcttaggcatcccaatccagggccactacca
gcaggacagctaagagagccgacaggtagtgatattgcaggaaccaccagcaatattgctgaacagatagcctgg
acaaccagagcaaataacccaatagcagtaggaaacatttatagaaattggatagtactagggcttcaaaagtgt
gtaaaaatgtataacccagtaaacatcttggatataaagcagggaccaaaagaacctttaaagattatgtggat
aggttttataaagcacttagagcagaacaggcagatccagcagtaaaaaattggatgactcaatcacttttgatt
cagaatgcaaacccagactgcaagacagtgctgaaggggctaggaatgaatcccactctagaagaaatgctaaca
gcctgtcaaggagtaggaggaccacagtataaagccagggtcctagcagaagccatgcaacaagcgaatagcgct
atcatggctcaaatgagaggtgggcaaagatcaggcccgccaagaggacaaggaggaaagacattcttgaaatgt
tacaactgtggaaagttggggcacatttcaagatattgtaaagcccccagaagaaaaggatgctggaagtgtgga
gaggaggatcacatccttaaggattgccctaagcaggta
>#16 M58410 1242 nt
atgggcgggggtcactcagcactgtcagggagaagcctcgacacgttcgagaagattaggctacgtccgaacggg
aaaaagaagtaccaaattaaacatttaatatgggcaggaaaagaaatggaacgatttgggttacatgagaaactt
ttagaaacaaaagaaggctgtcaaaaaatcatagaagttttaaccccgttggaaccgacaggctccgaggggcta
aaagctctgtttaatttgtgctgcgtcatttggtgcattcacgcagaacagaaagtgaaagacacagaggaagct
gtagtaacagttaagcaacactaccatctagtggacaaaaatgagaaagcagctaaaaagaaaaatgagacaaca
gcgccacctggtggcgaatcaagaaattacccagtagtaaatcagaataatgcctgggtacaccagcctttgtct
ccgcgcacgttaaatgcgtgggtcaaatgcgtggaggaaaaaaggtggggagcagaagtagtccccatgttccaa
gcactctcagagggatgtctctcctatgatgtaaatcagatgctcaatgtaataggagaccatcaggggggcatta
caaattcttaaggaagtcattaatgaagaagcagcagagtgggacaggacacacagaccaccagctggcccgtta
ccagcagggcagctaagagacccgacagggtcagatatagcaggaactaccagctcaattcaggaacaaatagag
tggaccttcaatgccaatccaagaatagacgtaggggcacaatacagaaaatgggttattttgggcttacaaaag
gtagtgcagatgtacaatccccaaaaggtcctagacattcgacagggacctaaagaacccttccaggactatgta
gacagattctataaagccctgagagcagaacaagcaccacaggatgttaaaaattggatgacacaaactttgctt
atccagaatgccaatccggattgtaaattgattctgaaaggattgggaatgaatccaaccttggaggaaatgcta
atagcttgccagggagtaggagggccacaacataaggctaagctaatggtagaaatgatgagtaatggacagaat
atggtccaagtgggacctcagaaaaagggcccccgagggccgctaaaatgctttaattgtggcaaatttggacat
atgcaaagggaatgcaaggcaccaagacagatcaaatgcttt
>#17 LC114462 1314 nt
atgggtgcgggtacctcagcattgtcagggagaaaattagatgaatttgagaaaatacgacttcgcccgaacggt
aagaaaaaataccgattaaaacatataatatgggcaggcaaagaaatggagcgcttcggcctccatgagaaactc
ttagaaacagaggaaggatgtaaaaggataatagaagttctattacctttagaaccaacaggatcagaaggtttg
aaaagcttgtttaatctcgtgtgcgtgctgtattgcattcaccgagaacagaaagtgaaagacacagaggaagct
gtagtaacagtaaaacaacactgccacctagaggaaaaacaaaaaagtgcagctgcgccacctggtggcgaaaag
aaaaattacaacagtagagagacatctggtggccaaagtcagaactacccagcgcaacaacagggaaatgcttgg
gtacatgtgccgctctcccccaggacgttaaatgcctgggtgaaagcagtagaagagaaaaaatttggggcagaa
atagtgcccatgtttcaagccctctcggagggatgcaccccttatgacatcaatcaaatgctaaatgttttagga
gaccatcagggggcactacagatagtaaaagaaatcatcaatgaggaagcagcaaattgggacttacaacatccc
ccgccggctggacccctaccagcagggcaattgagagaccctaggggatcagacatagcaggtaccaccagcaat

gttcaagaacagctggagtggatctataccgccaaccctagagtagacgtaggggccatttataggagatggatc
atcttggggttacaaaaatgtgttaaaatgtacaacccagtctcagtgctagacatcagacaagggcctaaagag
cccttcaaagattatgtagatagattctacaaagctataagagcagaacaaacatcaggagaagtaaaacaatgg
atgacagagagtctgctcatccaaaacgccaacccagaatgtaaagtaattctaaagggtctgggaatgcaccca
acattggaagaaatgctcacagcctgtcaaggggtaggaggaccccagtacaaagccaaattgatggtggaaatg
atgcagcaaatgcagggacaaaacatggtgcagcaaggggggcccaagaggaccaccgcgaggaaaccctcgctgc
ttcaattgtggaaaatttggccatttacaaagaaattgcgcagagccaagaaagatgaaatgctttaaatgtgga
aaaataggacacatggccaaggactgcaaaggacaggtt
>#18 KR862336 1335 nt
atgggtgcgggttcctcagcattgtcagggagaaaattagaccgatttgaagaaatacgtttacgcccgaacgga
agacaaaagtataaattgagacacttaatatgggcaggcaaggagatggagcgcttcggcctccacgagaagtta
ctagaaacagaggaaggctgtaagaaaatcatagaagtactcttcccccctagaaccaacagggtcggaaggttta
aaaagtctgtttaatctcgtatgtgtgctttactgcattcatcgagaacagaaagtgaaagacacagaggaagct
gtagtaacagtaaaacaacagtgccatctagtggagaaagaaaaaactgcagcagcgccacctggtggcgaaaag
aaaaatagcaacaccacagtgacatctagtggtcagagtcaaaattacccagtacaacaacaagggaatgcctgg
gtccataccccactctctccaaggaccctaaatgcttgggtaaaagcagtagaggaaaagaagtttggagcagag
atagtccccatgttccaagcactctcggagggatgcactccgtacgatataaatcaaatgcttaatgtgcttgga
gaccaccaaggggccttacaaatagtaaaagagatcattaatgaggaagcagccaattgggaccaaacacatcca
ccaccagcaggtccattaccagcaggacagctgagagatccgagaggatcggacattgcaggaaccactagtaca
attcaggaacaaatagaatggatctacacagcaaatccaagaacagatgtgggagccatttacaggagatggata
atcctagggctgcaaaagtgtgtaaagatgtacaatccagtcagtgttttggatattaggcaagggccaaaagaa
gcctttaaagactatgtagacagattttacaaagccataagagcagaacaggcctcaggagaggtgaaacaatgg
atgacggaaactctcttaactcaaaatgccaacccagaatgcaaagtcatcctgaaaggcctaggtatgcacccc
acccttgaagaaatgttaacagcttgtcaaggagtaggaggacctcagtataaggctaaactgatggtagaaatg
atgcagcaattacagtcaaatcaaaacatggtccagcaggggacaggaggcccaaggggaccaaaaggcctaga
ggaccaccaagatgctttaattgtggaaaatttggacacttgcagagaaattgtccagaaccaagaaagagcaag
tgtttcaaatgcggcaaaacaggccacatggcaaaagattgcaggggacaggtgaatttt
>#19 KR862356 1332 nt
atgggtgcgggttcctcagcactgtcagggagacaattggaccaatttgagaaaatacgtttacgcccgaacgga
aagaaaaaattacaaattgaaacatttaatatgggcaggcaaagagatggagcgctttggcctccatgagaagtta
ctagaaacaaaggaaggctgtcagaagatcattgaggttctctttcctttggaaccaaccggatctgaaggacta
aaaagtctctttaatttggtgtgcgtattatattgtttgcacacagaacagaaagtgaaagatacagaggaagcg
gtggcagtagttagacaacactgccacctagtggaaaaagaaaaaagtacaacaacgccatctggtggccaaact
ggaaataacatgcagacagcgccacctggcggcaggagtcaaaactttccagtacaacaacagggaaatgcatgg
gtccatgtacccctctccccacgcaccttaaatgcctgggtaaaagcggtggaggagaaaaaatttggagcagaa
atagtgcccatgtttcaggcattatcagaaggctgtacaccctatgatataaaccagatgcttaatgtgctagga
gaccatcaaggggcattacaaatagtgaaggaaatcatcaatgaggaggccgcccagtgggacctgacacatcct
ccacaagcaggaccaataccagcaggacaattaagggacccacgaggctcggacattgcagggaccaccagctca
ttacaggaacagatcgaatggatttatacggccaaccctaaagtagatgtaggagccatctatagaagatggatt
atattaggcctacaaaaatgtgtaaaaatgtacaatccagtcagtgtcctagatatcagacagggtcccaaagaa
cctttcaaggattatgtagatagattctataaggcaataagagcagaacaggcttcaggagaagtaaaacaatgg
atgactgagagtctcctgatacagaatgccaatccagagtgtaaaatcatcctcaagggattgggcatgcacccc
accctggaagaaatgttgacagcatgtcaaggagtaggggggcccgcaatacaaggccaaattaatggtggaaatg
atgcaacaactgaatcaacagaatcaggtcatggtccaacaaggagggggaggacccagacgtggcccccccaaaa
ccaattaggtgctttaattgcggaaaatttgggcatatccaaaagaattgtacagaaccaaggaaacttagatgt
ctaaaatgtggcaaaccaggccacatggcaaaagactgcaggggacaggtgaatttt
>#20 KR862363 1329 nt
atgggagcgggttcctcagcattgtcaggtaggcaattagaccaatttgaacaaatacgcttacgcccgaacgga
aagaaaaagtataaattaaaacatataatatgggcaggcaaggagatggagcgcttcggcctccatgaaaagtta
ttagaaacaaaagaaggctgtcaaaagatcatagaagtcctttttcctctagaaccaacgggctcggaaggacta
aaaagtctttttaatctggtatgcgtactttactgcctgcatacagaacagaaagtgaaagacacagaagaggca
gtaacaacagtaaggaaacagtgccacctagtggaaaaacaaaaaattgcaacagagacatctagtggccaaaaa
gaaaatagcagctcaaaagagacacctagtggcagaagtggtaattacccagtgcaacaacagggaaatgcatgg
gtacacgttcctctatccccgcgcactctgaatgcgtgggtgaaagcagtggaggagaaaaagtttggggcagag
atagtacccatgttccaagctctatcagaagggtgcacaccatatgacatcaatcagatgttgaatgtgttagga

gatcaccaaggagccctgcagatagtaaaagaaatcattaatgaagaagcagcccagtgggacttgacacatcca
ccgccagcagggccattaccagcaggtcagctaagggatcccagaggatcagacatagcaggaacaactagtaca
ttacaggaacaaattgaatggatctacactgctaatcctaaggtagatgtaggtgctatatataggagatggatt
attttggggctgcaaaaatgtgtaaaaatgtacaacccggtcagtgtcctagacatcagacaaggaccaaaagaa
cctttcaaagactatgtggacagattctataaggccattagagccgaacaagcctcaggggatgtaaaaatgtgg
atgacagagaccctcctcgttcaaaatgccaaccctgagtgtaaagtcattctgaaagggttaggcatgcatccc
actttggaggagatgctaactgcctgccaagggggtaggggaccccagtataaggcaaaattgatggtggaaatg
atgtcccaaatgaatcaacagcaacagatcatggtacagcaggggaaccctagaaaggaccgccaagaccgtcc
gcaaaatgttttaattgtggaaggtttggacatctgcaaagaaactgcccagaacccagaaaaataaagtgcctt
aaatgtggaaagccaggacatatggcaaaagactgcaggggggcaggtaaatttt
>#21 M29975 1266 nt
atgggggcggctacctcagcactgaataggagacaattagatgaatttgagcatatacgacttcgcccgaacgga
aagaaaaagtatcaaattaaacatttaatatgggcaggcaagaagatggaccgcttcggcctccatgagaagtta
ttggagacagaggaaggttgtaaaaagatcatagaagttctctctcccctagaaccaacagggtcggaaggaatg
aaaagtctgtataatctggtgtgcgtattgctttgcgtccaccaagaaaagaaagtgaaagacacagaggaagct
ttagcaatagtaagacaatgctgccacctagtggacaaagaaaaaactgcagttacgccacctggtggacagcag
aaaaataacacaggaggaacagcgacacctggtggcagccaaaattttcccgcacaacagcaaggaatgcatgg
gtgcatgtaccactttcacctcgcaccctaaatgcatgggtaaaagcagtagaagagaaaaaatttggggcagaa
atagtacccatgttccaagccctctcagaaggctgcaccccatatgacatcaatcagatgcttaatgtcttagga
gatcatcaggggggccttgcaaatagtgaaagaaataattaatgaggaagcagcccagtgggatgtaacccaccca
ccgccggcaggccccttgccagcgggacagctcagggatccgggggggatcagatatagcagggaccactagtaca
gtgcaagagcagctagagtggatctatactgctaacccaagggtagatgtaggggccatctatcgaagatggatc
atcctagggttacaaaaatgtgtaaaaatgtacaatccagtgtctgttttagatatcagacaagggcccaaagaa
ccattcaaagattatgtagacagattctataaagcaataagagcagaacaagcttcaggagaagtcaaacaatgg
atgacagaatctttgctcattcagaatgccaacccagattgcaaagtaattttgaagggcctagggatgcacccc
actcttgaagaaatgctgacagcctgtcaagggggtgggaggcccaagttacaaagccaaagtcatggcagaaatg
atgcagaacctgcagagtcagaacatggtacagcagggaggtggaaggggaagaccaagaccccccgccaaagtgt
tacaactgtggaaaatttggccacatgcagaggcagtgtcctgagccaagaaaaataaaatgtctt
>#22 AF301156 1308 nt
atgggcaacgagcagggactgttagggaagaaaacattagaagacctacagaaagtccggctgaagaaagggaag
aaaggatgttataagataaaacatgttagatggatgtgcacagaggtaagccgctgtgttttaatatttgagctg
ttaaagtcagcaacaggagtggctcaaatattggaaaaggtgacgccattagtggacacaggatcagaagtgttg
aggtccctatatggatgttctgtgtgttactgcctgcacagaaaatggaatattgaggacactcaagaggcagaa
aagaaagtagaagaagcttataaaaagcaggccatgatagaaatggccagcaaggaggaggagaaagcaaaaaag
gaagcagagaaattggacatgccaatagtaacagggcctcaagggccagtgcatcaacccctgagccccaggacg
ttaggagcctgggttaagtgtgtagagggaggaattgctccttccttggctcccatgttcttagcttattctacg
ggggcaatagcttatgatatgaacttgatgctcaatattttggatactcatcaaggattccctcaggtcttgaag
gatgagattaataaaaaggcagaagagtatgacctactacatccggtccagcagccacaacagcaaggagcatta
aggcagcctacagcatcagatatcacaggcaacacaagctcagtagcagaacaagtggcttggggagagcccata
gccaacatttataggggctggatagtacagagtctggaaaaggtaatccagattgcaagaccatcctctgtacta
gatattagacagggaagtaaagaggattttaaaagctatgtagacagattttattcagccctaagggcagaacct
gcagcaggagaaatcaaggcctggatggctaataatttgctaatacagcatgccaaccctgactgcaaaagaatt
ctgaaaggactgcagaagccatctttggaagacatgctagcagcatgccagggagtgggaggaccagaccacaag
gcaaaggtgctagcagaggctatgcagcagttccagcaagagagaaccaacatgattgaagtaaagacagcaaaa
tgcttcaattgtcagggaatagggcacttagccagaatgtgtcccaaaagaccaataggaggagccggtcgagga
agaggccgaggacggggaggctttagaggagctcccagaaggccagtgaggtgttttacctgcaatcaggaagga
cacatgcaaagagattgtcccaacaagcaggca
>#23 AY159322 1311 nt
atgggcgcgagcgcgtcaggacttaggggagaaaaattggatgaactggaaaagattaggttacggccctccgga
aagaaaaagtaccagttaaaacatgttatatgggtaagcaaggaactagacagatttggcctacatgagaagttg
ttagaaagtcaggaaggatgcgaaaaaattcttagcgtactctttccactagttcctacagggtcagaaaattta
atttcgctgtacaacacctgttgttgcatttggtgcgtacatgcgaaagtgaaagtaacagatactgaggaagca
aaagagaaagtaaaacaacggtaccacctagtggttgaaagagagaatgcagcctcagaagaagaaaaggagca
acagcgacacctgctgttcgctcaaaaattaccccattcaggtaataaatcaaacaccagtacatcaggaatt
tcaccaagaaccttgaatgcatgggtaaaatgtatagaggagaaaaagttcagcccagaaatagtgcctatgttt

attgccttgtcagaaggatgcatcccgtatgacctcaatggcatgctcaatgccattggagatcatcagggagct
ctccaaatagtgaaggatgtcatcaatgaggaagctgcagactgggatcttagacaccccctgtgggacctatg
ccccaaggagtgctaagaaacccaacaggaaatgacattgcaggaacaacaagctccatagaagagcagatagaa
tggacaaccaggcagcaagaccaggtaaacgtaggaggtatatacaaacaatggatagtactagggctgcaaaaa
tgtgtaagcatgtataaccctgtaaatatttagacatcaaacagggaccaaaagagccttttaaggactatgtg
gatagattctacaaagctctgcgggcggagcgcacagatccacaggtcaaaacttggatgacgcagacattactc
atccagaatgccaacccagattgtaaagctaccctcaaagggttaggcatgaatcccacattggaagaaatgttg
ctggcctgccaaggggtaggaggacctaaatacaaggcacaaatgatggcagaggccatgcagcaagcacaggca
gctgtaatgatgcagaactcgggggggaccgccgcggggtcccccgagacaaccccctagaaaccctagatgcccc
aattgcggaaagtttggacatgtactgagggattgcagagccccaagaaagagaggctgttttaagtgtggagat
ccaggacatctaatgaggaactgcccaaagatggtg
>#24 AF367411 1311 nt
atgggcgcgagcgcgtcaggtcttaggggagaaaaattggacgagctggaaaagattaggttacggccctccgga
aaaaagaaataccagctgaaacacataatatgggtaagcaaggaactagacagatttggccttcatgaaaagctg
ttagaaagtaaggaaggatgcgagaaaattcttagcgtactctttccactagttcctacagggtcagaaaattta
atctcgctgtacaacacctgctgctgcgtatggtgcgtacatgcgaaagagaaagtaacagatacagaggaagcg
aaagagaaagtaaaacaaaagctccatctagtggccgaaaaggaaaatgcagcatcagaaaaagaacaaagagca
atagtgacacctagtggccgctcaaaaaattacccaatacagataataaatcagaccccagtccatcagggaatt
tcaccgcgcacgctgaatgcgtgggtaaaatgcatagaagagaagaagtttagcccagaaatagtgcccatgttc
atagccttgtctgaagggtgcctcccctatgatctcaatggcatgctcaatgctattggagaccatcagggagcg
cttcaaatagtgaaggatgtcatcaatgaggaagctgcagactggaacttgagacacccacaagtgggtcctatg
ccccaaggtgtcttgagaaacccaacgggcagtgatattgctggaacaacaagctccatagaagagcagatagaa
tggactacaagggagcaagatgcagtaaatgtgggaggaatatataaacaatggatagttttagggcttcagaag
tgtgtaagcatgtataatccagtaaacatcctggacataaagcaaggaccaaaggagccgttcaaggactatgtg
gacaggttttacaaagctctgcgggcggagcggactgatccacaagtcaaaacctggatgacacaaactttgctc
atccagaatgctaacccagattgtaaatccatccttaaagggctaggcatgaatccctctttagaagagatgccg
ctagcctgccaaggggtaggaggccctaaatataaagcacaaatgatggcagaggctatgaaggaagcccagtca
gctgtaatgatgcagaattcgggagggccaccgcggggcccccgagacaacctcctagaaacatcagatgccct
aactgtggaaaatttggtcatgggctgagggactgtataagccctcgaaaaaagggatgcttcaagtgtggagat
ctaggacacataatgagaaattgcccaaagatggtg
>#25 AF328295 1311 nt
atgggcgcgagcgcgtcaggtcttaggggagagaaattggatgaactggaaacaattaggttacggccctccgga
aagaagaaataccagttaaaacatataatatgggtaagcaaggaactagatcgatttggccttcatgaaaaacta
ttagaaactcaggaaggatgcgagaaaattcttagcgtactctttccactagttcctacagggtcagagaatctt
cttgcgctgtacaacacctgttgttgcatctggtgcgtacatgcgaaagtgaaagtaactgatacagaagaggca
aaaaagaaagtagaacaatgcttccatctagcggccaaaggagaaagtgcagcctcagaaaaagaagagaaagca
acagcgacacctagtggccgctcaaaaaattatcctattcagataataaatcaaacaccagtgcatcaaggaatt
tcaccaagaactttaaatgcctgggtcaaatgtattgaggaaaagaaattcagtccagaaatagtacctatgttc
atagccctatcagaaggatgcctcccatatgacctcaatggtatgttaaatgctattggggaacatcagggagct
ctccaaatagtgaaggatgtcatcaatgaggaagctgcagactgggatcttagacacccccaagtaggacctttg
cctcaaggggtgctgagaaacccaacaggaagtgacattgcaggaacaacaagcagcatagaagaacagatagaa
tggacaactagacagcaggaacaggtaaatgtaggggcaatctataagcagtggatagtcctagggctgcaaaaa
tgtgtgagcatgtacaacccagtgaacattctggacataaaacaagggccaaaagagcctttcaaggactatgtg
gacagattttataaagctctgcgggcggagcgaacagatccacaagtcaaaacctggatgacacaaacattgctc
atccaaaatgccaacccagattgtaagagcatcttgaaaggattgggcatgaacccctctttagaagaaatgtta
ctagcatgtcaaggagtgggggaccaaaatataaggcacaaatgatggcagaggccatgcagaagatgcagtca
gaagtcatgatgcagaactcgggggaccaccgcggggtcctccgagacaaccccctagaaaccccagatgtccc
aactgtggaaggtatggacatgttctgagagactgtagactccccagaaagaagggatgctttaagtgtggggac
acaggacatatgatgagaaactgcccaaaaatggtg
>#26 KM378564 1320 nt
atgggagcaagcgcgtcaggacttaggggagagaagcttgatgcttttgaaaaggttaggttacggccctccgga
aaaaagaaatacaggctcaagcatcttgtctgggtagcgaaggaactagatcgattcggcctacatgagagatta
ttagaaagtcaggaagggtgccaaaaaatttaggagtactctttcctctagttcctacagggtcggagaattta
atttctctctttaatacctgctgcgtagtttggtgcgttcactcgaaagtgaaagtatcagacacagaggaagct
gttcaaaaggtaaagcaacactgccatctagtggacaaaaatgagaatgcagcttcaaaaaatgaaaatggggaa

acagcgacatctagcggcaggtcaagaaattaccccattcaagttgttaatcaacaggcagtgcaccaggctatt
tcaccaagaactttaaatgcctgggtgaaggtgattgaggaaaagaagttcagtgcagaagtggtgcctatgttt
ttggccctgtcagaaggctgcattagctatgacatcaatcaaatgttgaatgctattggagatcaccaaggtgcc
ctccaaatagtaaaagacatcatcaatgaggaagcagcggactgggataggagacatcctcaagtaggacctctc
ccacaaggggtgcttaggaacccctcagggtcagacattgcagggaccaccagctctatagaagagcagatagaa
tggaccaccagagcccaagatgccatcaatgtgggagcaatttataggcaatgggtggtcttagggttgcaaaga
tgtgtaacaatgtataatccagtcaacatcttggatgtaaaacagggtcctaaagaaccattcaaggactatgta
gatagattttataaagccctcagagcagaacacacagatgcagcagtaaaaaattggatgacacaaacgctgctg
atacagaatgcaaatccagactgtaaagtgattctcaaaggcctagggatgaaccccagtctggaagagatgctg
ctggcttgccaaggagtaggaggaccaggccataaggcaagggtcatggcagaagctatgaaagaacaacaagca
gctataatgatgcagcagaattccctcagaggtccaccgcggggacccaggggacccctccaagaaaccctaaa
tgtccaaactgcgggcagttcggacataacctaagacaatgtacaaagcctagaaaaaagggatgctttaggtgt
ggagctttagatcatatgttaagaaattgcccaaaacaggtgaat
>#27 KM378563 1320 nt
atgggagcaagcgcgtcaggacttaggggagagaagcttgatgcttttgaaaaggttaggttacggccctccgga
aaaaagaagtataagctcaagcatcttgtctgggtagcgaaggaactagatcgattcggcctacatgagagatta
ttagaaagtcaggaagggtgccaaaaaatttaggagtactctttcctctagttcctacagggtcagagaatcta
atttctctctttaatacctgctgcgtagtttggtgcgttcactcgaaagtgaaagtaacagacacagaggaagct
gttcaaaaagtaaaacagcactgccatctagtggacaaaaatgagaatgcagcttcaaaaaatgaaaatggggaa
acagcgacatctagcggcaggtcaagaaattaccccattcaggttgttaatcaacaggcagtacaccaggctatt
tcaccaagaactttgaatgcctgggtgaaggtaattgaggaaaagaagttcagtgcagaagtggtgcctatgttt
ttggccctgtcagaaggctgcatcagctatgacatcaatcagatgttaaatgctattggagatcaccaaggtgct
ctccaaatagtaaaagacatcattaatgaggaagcagctgactgggataggagacatcctcaagtaggacctctc
ccgcaaggggtgcttaggaacccctcagggtcagacattgcaggaaccaccagctctatagaagagcagatagag
tggaccaccagagcccaggactccatcaatgtgggagcaatttatagacaatgggtggtcttagggttgcaaaga
tgtgtgacaatgtacaatccagtcaacatcttggatgtgaagcaaggtcctaaagaaccattcaaggactatgta
gatagattctacaaagccctcagggcagaacatacagatgcagcagtaaaaaattggatgacacaaacgttgctg
atacaaaatgcaaatccagactgtaaagtgattctcaaaggcctagggacgaaccccagtctggaagagatgctg
ttagcttgccaaggtgtaggaggaccaggccataaggcaagggtcatggcagaagctatgagagaacaacagaca
gctataatgatgcagcaaaatccccccaggggtccaccgcggggacccaggggacccctccaagaaaccctaga
tgtccaaactgcgggcagtatggacataccctaagacaatgtacaaagcctagaaaaaagggatgctttagatgt
ggagctttagatcatatgttaagaaattgcccaaaacaggtgaat
>#28 M27470 1293 nt
atgggtaatgggaactctgccttgttagggactgatttggataaatttgagaaaataagattaaagagaggtggt
aaaaaatgttatagattgaaacacctctgttggtgtaaaggtgaattagatagatttggcttatcggataaactc
cttgaaacacagcaaggatgtgaaaaaatcctctcagtatgttggccattatatgaccaaggatcagataatcta
aaagctttggtagggacagtctgtgttgtagcctgcatacacgcaggtatagaaattaagagcacacaagatgct
ttaaaaaaattaaaagtcataacaagaaaggaagaaaagcaggaggatgaaagtaagaatttccctgtacaaagg
gatgcagcaggacagtatcagtatactccaataagtcctaggattatacagacatgggtaaaaacagtggaagaa
aagaagtggaaaccggaggtcatccctctattctcagcattgacagaaggagcaatcagtcatgatttgaatatc
atgctgaatgcagtaggagatcatcagggagcaatgcaagtcttaaaagatgtaattaatgagcaagcagcagaa
tgggatctaacacatcctcaacaacaaccagcacaaccaggaggaggattaaggaccccttcaggctctgatata
gcaggaactacttctacagtggaagaacaattggcatggatgaatatgcaacaaaatgcaatcaatgtaggaaca
atctataagagttggattatactgggcatgaatagattggtaaaaagtcattgtccaataagtataacagatgta
agacagggaccaaaggaagcttttaaagactatgtagatagattctacaatgtaatgagagcagaacaagcttca
ggagaagtaaagatgtggatgcagcagcatctgcttatagaaaatgcaaacccagaatgcaagcagattttgaga
agcttagggaaaggagcaactttagaggaaatgttggaagcatgtcagggagtaggtgggccacaacataaagcc
agattaatggcagaaatgatgagaacagtggtaggacaatcacaaaattttgtgcagcagagagggcctcaaaga
ggaccagttagacaacctactggaaggaaacctatctgcttcaactgtaataaagaagggcatgtagcaaggttc
ttcaaggcccctagaaggaaagggtgctggaattgtggagcaatggatcatcagaaagctcaatgccctaagcca
gctcagcagcagagggtt
>#29 AF131870 1290 nt
atgggagcgggaaactccacagtggatagggaagttgtgaggagctttgaaagagtagctctgaaacctggaggg
aaaaagacctaccaggcaagacatgtagtatgggcaggtaaagaattagataggtttggacttaacaaggaactg
ttaagaacagtggaaggctgtcagaagattctgtcagtctgttggcctttgtatgcatctggttctaagaacctg

aaggccttagtaggtacagtatgtgtgcttgcctgttgtcatcaaggcattccagtaaaagacacacaggaggct
ctaaaaaaggtcagactagagccagcaaaaaatgaagaagcacaagcttctcaaaattttccagtgcagagagag
ggacaaaattacattcatcagcctctctcgcctagaacagtgcaaacatgggtgaagatagtggaagagaaggga
tggaaaccagaaacagtggcaatgtttgcagctttaatgacaggagcaattccagaagacatcaatgtgatgctg
aatgccatagggggagcatcaaggagctatgcagatgataaaagatcatatagtggaggaaagtgcagaatgggac
agaatgcaccctcagcagcagcctgcacagccaggaggaggcctaaggactcctaatggtacagacatagcaggg
gtaacatcaacagtagaagagcagatacagtggatctatggccttaatggagcaacaagagttgcagtacaagac
atctataaaggctgggtcatagagggcatggaaagagtagttagactccatcagactacctcagtagtagaaatt
agacagggaccaaaagagcctttcagagactatacagataggttcttcaaggctttaagagcagaatcaggctca
gaggaagtaaaagagtggatgaaagagaaaatgctagtgcaaaatgcaaacccagattgtaaattggtaataaag
gcactgggagaaactccatcattagaggagatgatgagagcctgccaaggggttgggggccgagccataaagga
aagatcctggcagaagcaatggcctctgcttttaggcagcaagtaggaagacaagctatggtgcagcaaaactta
ccacccagaaacagccaggggaggtttgtaagaataggaggaggaggacctaggaagcccatgacttgttttaat
tgtggtaaaccaggacatctagcaaatcagtgcagagagccaaagaaaggaccacccggctcctgctggaaatgt
ggaaagatgggacat
>#30 AF075269 1275 nt
atgggatcgggtaactctgtcctcagtaggcaaattgaaaaagatttttgtagtgtcaggctaagacctgggagt
aaaaagacttatcaaaaaagacatgtagaatgggcaacgaaagaattagacagatttggattgggatcacaactc
ttggaaacagcagaagggtgtaagaaaatcctatctgtctgctggcctttatatgcaactggttctaagaaccta
aaggccttagtagggacagtatgtgtcatctgctgctgtcacctaggagtaagaatatcagacactcaggaagcc
atcagtaaagtaaaaatagaaccagcaccagaagcagcagggaagaaacaacagacaggggggaaattatcccctg
ataagagaaaatcagagatgggtacatacacctttatcgcccagaacaatacaaacatgggtgaaaatagtggaa
gacagaggttggaaaccagaaacagtagcaatgttttcagccttaacagaaaaagctttgcctgatgatttaaat
gttatgttaaatgcaattggagatcatcagggcgccatgcaaataataaaagatcacatagtggaggaaggtgca
gagtgggatagacagcacccgcaacaacagccagctcaaccaggagggggtttaagaactcctaatgcaacagat
gtggcaggggtcacctcaacagtagaagaacaactagcctggactacagcagacactcctgtagatgtaggaaaa
atttataaagaatgggtcattcaggctatggaaaaagtagtcagaatccatcaaccagtaagtgtcatggacatc
aaacaaggacccaaagagccttttaaagaatatgcagataggttctttaaagcactgagagcagaaggaggctct
catgaagtaaaggagtggatgaaggagaaaatgcttgtgcagaatgcaaaccctgactgcaggttagttataaaa
gcactaggagaaggggcatctctagaagagatgatgaaagcttgtcagggagtaggaggacctgctcataaagga
aaaatattggcagaagcaatggcttcagccatacagcaacaagtaagacaaaacatggtgcaggtcactccgctt
agaaatgcgcaaggcaggtttgtgagaacaggaggaggaggtcccaggaaacccctcacttgcttcaattgtgga
aagccaggtcacacagcaagaatgtgcagacagcccagacaggaaggttgttggaactgtggcagtaaagagcat
>#31 AF188116 1251 nt
atgggatcggggaactccgtcctcagtaggcaaattgagaaagatttttgtaatgtcaggctaagacctgggagt
aaaaagacttatcagaaaagacatgtagactgggcaacgaaagaactagataggtttggctaggagcaaacctt
ctagaaacagcagagggctgtaagaagatttgggagtccgttggcctttatatcctacaggctctaaaaatctg
aaagctctagtagggacagtgtgtgtcatctgttgctgtcacctaggaataagagtcaatgatacacaggaagca
attaataaagtgaaaattgagcagaacaaacctgtgccagaaagtaaaaattttcctttgattagagaaaatcaa
agatgggtccataccccctctatcacccagaacaattcaaacatgggtgaaaatagtagaagataggggatggaag
ccagaaacagtggctatgttttcagctctgacagaaaaagccttgccagaagacttaaatgtcatgttaaatgct
ataggagaccatcaaggggcaatgcagataataaaagatcatatagcggaagaaggagctgaatgggatagacag
catccccagcagcaaccagcacaaccaggaggagggttaagaactccaaatgcaacagatgtggcagggatcaca
tcaacagtagaagaacagcaagcctggacaacagcagacacacccatagatgtagggaaaatctataaagaatgg
gtaatccaggctatggagaaagtggtaagaatacatcagccagtcagtgtaatggacataaaacagggtccaaaa
gaaccattcaaggactatgctgataggttctttaaggccttaagagcagaaggaggttcacatgaagtgaaagag
tggatgaaagagaaaatgctagtgcagaatgcaaaccctgactgcaaaatggtaataaaggccttaggagaaggt
gccactttagaagaaatgatgaaagcatgtcagggagtaggaggacctgcccataaaagaaaaatcttagcagag
gcaatggcaacagccatgcaggggcagatgaaacatctcatggtgcagcaaacaccacctagaaatgcacaaggg
agatttgtaagaacgggggggaggtggcccaagaagacctctcacttgtttcaattgtgggaagccaggacattca
gcaaaaaatgtgcaggcagcctagacaggaaggctgttggaactgtggtgct
>#32 AF188115 1290 nt
atgggatcggggaactctgtcctaagtaggcaaattgagggagaattctgcagtatcaggctaagacctggcagt
aaaaagacgtatcagaaaagacatattgtgtgggcaacaaaagaattagataggtttggtttaggagcacacttg
ctggagacagcagatggctgcaagagaattcttggagtctgttggccactatactccacaggctcaaagaattta

aaagcacttgtaggaactgtatgtgtgctgtgttgctgccatttagggataaaaatagcaaacaccgcagaagct
atgcaaaagataaaaatagaaccaactcaggaagacaggcagcagaaaagtggcaattatccactcatcagagaa
aatcagagatgggtacacacgccattgtcacctagaacaatacaaacttgggtaaaaatagtagaagatagagga
tggaaaccagaaacagtggcaatgttttctgccctcacagaaaaagctttgccagatgacttaaatgtcatgtta
aatgccataggagaccatcaagtggcaatgcagataattaaagatcatattgtggaagaaggggcagaatgggac
aggacacatccgcaacaacaacctgcacaaccaggaggagggcttagaacccctaatgcaacagatgttgcagga
gtcacctcaacagtagaggagcagctagcctggaccactgcagacaccccagtggatgtaggaaaaatttataaa
gaatgggtaattcaagctatggaaaaagtagttagaattcatcaaccagttagtgtaatggacatcaaacagggg
ccaaaagaaccattcaaagaatatgcagataggttctttaaagcattaagagcagaaggaggctcacatgaggta
aaagaatggatgaaagagaagatgctggtacaaaatgcaaacccagattgtaggatggtaataaaagccttagga
gaaggggctagtttagaagacatgatgaaggcctgtcagggagtaggaggaccagcccataaaggaaagattttg
gcagaggcaatggcaacagccatgcaaagtcaaatgagacagaatatggtacaagtgacaccaccaaggaatgag
cagggaaggtttgtaagaacaggaggaggaggccctagaaagcctttaacttgctttaattgtggaaagccaggt
catacagcaagaatgtgtagacagcctagacaggagggatgctggaactgtggtagcaaagaacataggtttgct
caatgtccaaagcct
>#33 AY523867 1389 nt
atgggagcaggggatcggcgatcctaacaggtagaaagcttgataaatatgagaaaattcggcttcggccgaag
gggaagaagcgctacctagttcgacatttagtttgggcaggaaaggagctggacagattcggcctctcagaccag
cttcttgaaagtaaagaaggttgtgaacaaatcataaaaaccatcttaccattagaaaaacatggctcagaaaat
ttaaaatcattatttggcataacagcagtagtctgggccgtccacgcagaaaaagacgtggaagatacggaacaa
gcaaaacagaaagtgaaagaggcttgcaattgggaagatgaggaaacagtgacatctagtggccaaaaagaaaat
agcaacgacacagcgacatctagtggcagagaaggaaaaatgcagctgccagcagcaatgccatctagtggcggc
tcaggaaattaccctctaattagaaacccacaaaatcaatggattcatgtaggggtcaacaccaggactttaaaa
acctgggtagaggcagtgaacagcaaaaaatttgatgcctcactggtcccactattccaattttaacagaaggt
tttataccttatgacctcaatgacatgcttaatgccattggagatcatcaaggagcaatgcaagtgatcaaagat
gtgattaatgaggaagggtctgaatgggacctacaacacccacaacctcaacagcagcagcctgtagcagggcta
agagatcctagtgcctcagacatagcaggaacaaccagtaccatccaagaacaaatagagtggataactagacag
aacaacccaattcaggttggacagatctacaggcagtggataattctaggattacaaaagtgtgtgcaagtctac
aatcctgtcaatattttggacatcaggcaaggacctaaagaaacatttaaggattatgttgatagattctatcat
tgtcttagagcagaacaagcggacccggctgtaaaaaattggctcacacagtcattactaattcagaatgcaaac
cctgagtgcagaagcattctgaaagcaatggtcaaaccaaccttggaagaaatgcttcaagcttgccaaggggta
ggaggaccacaatacaaagcaaaactattagcagaagcaatggtaatgacacaacacagcttgggcatgatacaa
gggccaagacaaggaagcaatcccaggagagggccaacaagatgctttaattgtgggcagttaggacatcttcaa
aaagactgccctaggccaaaaaagcttaagtgctttaactgtggaggaacgggacacatagcaaggcagtgtaga
caaccaagaaaaggacaaggcaaccctcctccacaggcc
>#34 AY655744 1374 nt
atgggggcacggcactcggcgatgctcaccggtagggccttagataaatatgagaaagtgagactgaggccgaaa
gggaaaaagaagtatatggtgaagcatattgtatgggcttccaaagagatggagaggtttggcctgagtgacgcc
ctgttagagaccaaagaagggtgcgttaagatcatagaagccatttacatgttagaaccgaatggctcagaaggg
ataaagtctctctttggaatcgtgtgtgttctatactgcattcatgctggtatagacattgaggatacagagcag
gccaaggcagaagtaaggaaacgctgccacctaggggagaaaaaggaaacaacagaggaggaaaaagtaagaaag
gcaacagtgacatctagtggccaaacaggaaattaccccataataagaaatgcacagcagcagtatcaacatcag
gcactgtcacctaggctgctaaagacatggattgccactatagaggagaaaaaatttgcccctgaaacagtggcg
ctcttctcggcactagcggaagggtgcatcccctatgacatcaatcaaatgcttaatgcagtaggagagcatcag
ggggcaatgcagatcataaaggacatcattaatgagcaggcatcagagtgggatcagatgcatccccagataggt
ccactaccagcaggacagttaagggaaccagcagggagtgacatagcagggacaacaagcactcccgctgaacag
gtggaatggaccaccagagcacagaaccccgtagatgtgggagcaatatacaaaagatgggtcatcatgggctta
cagcgctgtgtaaaaatgtacaatccagttaacatactggatgtgaaacaaggccccaggaaccattcaaagac
tatgtagacaggttctacagatgtcttagggccgaacaaacagatcaggcagtaaaaaattggatgacacagacg
ctgttggtccagaatgctaacccagagtgcaaggccatcttaaaagccatgggcccaggagccaccttagaagaa
atgctccaggcctgtcaaggggtaggaggaccccagcataagagcaggcttatggcagaagctatggcagaagcc
ttaaaacaaaacacccaacaagtcatagccatggtacaacaggggcaaggaggcccaagggggagaggggacct
agaaggaacccccccagggcagattaggtgttataactgtggaaaatttggccatatagcaaagaactgcccggcc
ccgccgagacagaaggtgccgccaggcacttgctataagtgtggcaaaccaggccacatagcaaaacaatgctcc
tcagcacaggccaatttttaggg

>#35 AY523865 1389 nt
atggggcggggcggtcggcaccattaaccggggcggctttagacagatttgaaaaagtaagattgagaccaaaa
ggcaaaaagaagtatatgataaagcacctcgtctgggcaggtaaggaactagacagatttggccttgcagcagca
ttattagaaaataaggaaggctgtcaggaaattttgactaggttacagcctttagaagcaacaggcagtgagaat
ttgaaatctctatatggcatagtctgtgtcatctgggcatgccatgccaacctctcggtggatgacaccgaggag
gccaaaaaggcagtgtacaggaggataaagcaggtggaacaggaggaagaattagaaatggccctaatccaaaga
cagaaagagaaggaagtagaacagaaacaacaaaagcaacaggaacaaagacagcaggagcctcaggcagcaccc
cagacaacaccatcagttaactaccctatcttaagacaggggcaacagtttgtgcatatgccattaagccccagg
atagtaaaaacctggataaatgctgtagaggaaaagaaattttctccagagatagttcctctttttcaagtcttg
gcagagggctgtacccccctatgacattaatggtctacttaatgcaattggagatcttcaggggctatgcagatc
attaaagatgtaattaatgaggaagcagcagagtgggatttacaacacccacaacaacagccgccacaggggcag
cttagggaaccatcaggagcagacatagcaggaacaaacagcacagtggaagaacagatagcatggatgaccaga
ccagcaggtcaaggacaaggacccatagatgtaggacaaatatatagaagatgggtcatactaggtctgcaaaga
tgtgtgaaaatgtacaatccaaccaacatcttggatgttaaacaagggcccaaggaaccattcaaagattatgtt
gacaggttctataagaccttaagagctgaacaagcagatcaggcagtaaaaaattggatgaccaccaccctcatg
attcaaaatgcaaaccctgattgcaggatcatcctgaaaggattaggtcagaatcccaccttggaagagatgcta
catgcatgccaaggggtgggaggcccacagcaaaaggccagacttatggcggaggctatggcctcagccttaaaa
gaggcagggtccctaggcatggtacaacaaaggagaggacccccctggttctagaagacagattagatgcttcaat
tgtggacaaattggacatctccaaaaggactgcaaaagacctagaaagaccaaatgttttaaatgtgggcaggaa
ggccacattgcaaaaaattgtggacagactccgagggca
>#36 AY523866 1380 nt
atgggggcggggcgatcggcgccattaacgggggcagctttagacagatttgagaaaataagattgagaccaaaa
ggcaagaaaaagtatttgataaagcacctcgtctgggcaagtagggaactagacagatttggccttgcaggagca
ttattggaaaccaaagaaggatgtcaagagatcttaactagattacaacctttagaggcatcaggcagtgaaggc
cttaagtccctatacggcatagtatgtgtcgtttgggcatgccacgccaacctctcggtagaagataccgaggag
gcaaaaaaggcagtggctagaaggataaagcaggcagagcaggaggaagagttagagatggcattaattcagaag
aaagcagaaaaggagttagagaaaaagcagaaaactcaggcagcagcagcagacacacagcaaaaaacaaaccaa
ccagctaattttccaatcctgagacaaggacagcaatatgtacatatgcctttaagtcctaggattgtgaaaacc
tggataaatgcagtggaggaaaagaaattttccccagagatagtgcccctatttcaagtattggcagaagggtgt
actccctatgatattaatggctgctaaatgccataggagatctacaaggggcaatgcaaattataaaagatgtg
attaatgaggaagcagcagagtgggacttacaacacccacagcaacagccaccacaaggtcagcttagagagcca
tcaggagctgacatagcaggaacaaacagcacagtggaagagcagatagcttggatgaccaggccagcaggacaa
ggagcaggcccaatagatgtaggacagatatacaggagatgggtcatcctaggcttacagagatgtgtaaaaatg
tacaatccaactaacattttggatgttaagcagggtcctaaggaacccttcaaagactatgtagatagattctac
aaaaccttgagggctgaacaagcagatcaggcagtcaaaaattggatgaccaccaccctaatgattcagaatgct
aacccagattgtagaataattctaaaagggttaggacagaaccccactttagaagaaatgttgcatgcatgccag
ggagtaggaggtccccaacagaaagcccgtctgatggcggaggctatggcctcggccttaaaagaagcaggctcc
ctggggatggtgcagcaaagaggaggacaaggaggaggtcctagaagacagcttagatgctttaattgtggacag
attgggcatgtgcaaagggattgtaaaaagcctagaaaagttaaatgcttcaaatgtggtaaggagggccacata
gcaaagaactgtggacaagtaccaagggca
>#37 FJ919724 1413 nt
atgggagcggggcgatcagcaccgttaacggggaaagctttagacaaatttgaaaaggtgaggttaaggccaaaa
ggtaaaaagaagtatatgataaagcacctcgtctgggcagctagggaattagataggttcggcctctcagaggca
ttgttagaaaacaaggagggatgtcaggagattctaaccaggttaacacctttggaagcaacaggcagtgagaat
ctcaagtctctttatgggacatgttgtgtcatatgggcctgccatgcaggtctccaaatagaggacacagaagaa
gcaaagaaggcagtaaaacttagaattgctcaagtagaagcagaggaccaattagaaatgcaggtcctgcagaaa
aaagcagaggcggaagcagcaaagaaacaaggggcagtatcagctcaagcccaaccagcccaggttcaatcagca
gccccacaacagggaacagtagtaaggggcaactacccaatcctcagacaaggggggacagtatgtgcatatgcct
ctaagtccaaggatagtaaaaacttggataaatgcagtagaggagaaaaagttctccccagagatagtccctctt
ttccaagttctagcagagggctgtaccccatatgacatcaatgggttactcaatgccataggagatttacaaggg
gcaatgcagataataaaggatgtaatcaatgaggaagcggccgagtgggatttacaacacccacaacagcagcca
ccccaaggacagttgagagaaccctcaggagcagatattgcaggaccaacagcacagtagaagaacaaattgca
tggatgacaagagcccagggagtggaggaggacccatagatgtaggacaaatttatagaagatgggtaattttg
ggtctacaaagatgtgtaaaaatgtataatcctaccaacatcttggatgtgaaacaagggcccaaagagccattc
aaggactacgtagacaggttctacaagaccttgagggcagaacaggcagaccaggcagtaaaaaattggatgacc

accacccttatgattcaaaatgctaacccagattgcaggacaatcttgaaggctttgggacctaatcctactcta
gaggagatgctgcatgcatgccaaggggtaggaggaccccaacagaaagcaaggctcatggcggaggcaatggtc
tcagccatgagggagggaggaggccttaccatggtacaacagaggggaccaggagggccaagacctgggaataga
attagatgctataattgtggacaaattggccatgttcaaaaggactgtaggaagcccagaacagttagatgcttc
aaatgtcaacagacagggcacatggcaaaaaactgcccaaaagcagctcctcagtcaaagaat
>#38 AF208027 1296 nt
atgggcgcgagaagctccgtcctgtcagggaaaaaagctgatgaattagaaaaagttaggttacggcccggcgga
aagaaaaaatacatgttaaaacatgttgtgtgggcagcgaacgaattggacagattcggattagcagagagcctg
ttggaatcaaaagaaggttgccaaaaaattctttcagtattagctccattagtaccaacaggttcagaaaattta
aagagcctttataatactgtctgcgtcatttggtgtttgcacgccgaacagaaagtaaaacatactgaagaagca
aaacaagtagtgcagagacatctagtggtagaaactggaactacagaaaaagtgcctgccacaagcagaccaata
gcaccacctagtggcagaggaggaaactacccagtgcagcaagtaggcggcaactatgttcatctgccattaagt
cctcgaactttaaatgcatgggtaaaattggtagaagaaaagaagttcggggcagaagtggtgccaggatttcag
gcgctgtcagaaggctgcctaccatatgatatcaatcaaatgctaaattgtgtaggagaacatcaagcagccatg
caaattattagagaaataattaatgaggaggcagcagaktgggatcttcaacatccacaacaagggccgccgcca
gcaggacaamtcagggaaccgacaggatcagatatagcagggaccactagtacaatagaagaacaaattcagtgg
acacacaggcagcaaaaccctataccagtaggaaatatytatagaagatggatccarttaggacttcaaaaatgt
gtcaggatgtayaatccaacaaacatcctagatgtaaaacaagggccaaaagaaccctttcagtcttatgtagat
agattctacaagagtctragagcagaacaaactgatccagcagtaaaaaattggatgacacagacactactgatt
caaaatgccaacccagattgtaaattagtactaaaggggttgggaatgaatcctactttagaggaaatgttgaca
gcctgtcaagggrtaggaggaccagggcaaaaagccaggcttatggctgaagcattaaaagaagccttaaaccct
acagccctaccatttgcagctgctcaacaaaaaacaggaggaaaaaggagcacaataaaatgttggaattgtggc
aaggagggacatacagttagacagtgcagagcccccagaagacaggggtgctggaagtgtgggaagccaggccac
attatggccaaatgtccagag
>#39 M30502 1566 nt
atgggcgcgagaaactccgtcttgagagggaaaaaagcagacgaattagaaaaagttaggttacggcccggcgga
aagaaaaagtacaggttaaaacatattgtgtgggcagcgaatgaattggataaattcggattggcagagagcctg
ttggagtcaaaagaaggttgccaaaagattctcagagttttagatccattagtaccaacagggtcagaaaattta
aaaagcctttttaataccgtctgcgtcatttggtgcttgcacgcagaagagaaagtgaaagatactgaggaagca
aagaaactagcacagagacatctagtggcagaaactggaactgcagagaaaatgccaaatacaagtagaccaaca
gcaccacctagtgggaaaagaggaaactaccccgtgcaacaagcgggtggcaactatgtccatgtgccactgagc
ccccgaactctaaatgcatgggtaaaattagtggaggaaaagaagttcggggcagaagtagtgccaggatttcag
gcactctcagaaggctgcacgccctatgatattaatcaaatgcttaattgtgtgggcgatcaccaagcagctatg
caaataatcagagagattattaatgaagaagcagcagactgggattcgcagcacccaataccaggccccttacca
gcaggacagctcagagacccaagagggtctgacatagcaggaacaacaagcacagtagatgaacagatccagtgg
atgtataggccacaaaatcccgtaccggtagggaacatctacagaagatggatccaaatagggctgcaaaagtgt
gtcagaaagtacaacccaactaacatcttagacataaaacagggaccaaaagaaccgttccaaagctatgtagac
aggttctacaaaagcttgagggcagaacaaacagacccagcagtaaaaaattggatgacccaaacgctgctaata
cagaatgccaacccagactgcaagttagtactaaaaggactggggatgaatcccaccctagaagagatgctaacc
gcctgccagggggtaggcggaccaggccagaaagccaggctaatggctgaagccctaaaagaggctatgggacca
agccctatcccatttgcagcagcccaacaaagaaaggcaattaggtattggaactgtggaaaggagggacactcg
gcaagacagtgccgagcacctagaagacagggctgctggaagtgtggcaagccaggacacatcatggcaaactgc
ccggaaagacaggcaggtttttagggttgggcccacggggaaagaagcctcgcaacttccccgtgacccaagcc
cctcaggggctgataccaacagcacctccggcagatccagcagcggaactgttggagagatatatgcagcaaggg
agaaagcagagggagcagagggagagaccatacaaagaggtgacggaggacttgctgcacctcgagcagagagag
acacctcacagagaggagacagaggacttgctgcacctcaattctctctttggaaaagaccagtag
>#40 AF082339 1221 nt
atgggcgcgagaaactccgtcttgagagggagaaaagcagacgaattagaaagaattaggttacggcccggcgga
aagaaaaaatatcagctaaaacatattgtgtgggcagcgaatgaattggacagattcggattggcagaaagcctg
ttggagtcaaaagaaggttgccaaagaattcttaaagttttagaaccattagtgccaacaggatcagaaaattta
aaaagcctttttaatactgtctgcgtagtttggtgcgtgcacgcagaagagaaagtgaaagatactgaaggagca
aaacaaataatacagagacatctagcggcagaaatagaaacagcagagaaaatgccaagcacaagtagaccaaca
gcaccacctagtgaacagggggaaacttccccgtacaacaagtagccggcaactacacccatgtgccgctgagc
ccccgaaccttagatgcttgggtaaaattagtagaagaaaagaagttcggggcagaagtagtgccaggatttcag
gcactctcagaaggctgcacgccctatgatattaatcaaatgcttaattgtgtgggcgaccatcaagcagccatg

caaataatcagggagattatcaatgaagaagcagcagactgggatgttgcacatcccataccaggccccttacca
gcagggcagcttagagaaccaagagggtctgacatagcaggaacaacaagcacagtagaagaacagatccagtgg
atgttcaggccacggaatcctgtgccagtagggaacatctatagaagatggatccagatagggctacagaagtgt
gtcaggatgtacaacccaaccaacatcctagacataaaacaaggaccaaaggagccattccaaagctatgtagat
agattctacaaaagcttaagggcagaacaaacagatccagcagtaaagaattggatgactcaaacactgctggta
cagaatgccaacccagactgcaaattagtgctgaaaggattagggatgaatcctaccttagaagagatgctaacc
gcctgtcagggagtaggggggaccaggccagaaagccagattaatggcagaagccttaaaggaggccatgacacca
gctcctatcccatttgcggcagcccaacaaagaaggacaattaagtgctggaattgtggaaaggaagggcactcg
gcaagacaatgccgagcaccc
>#41 U27200 1224 nt
atgggcgcgagaggctccgtcttgtcagggaagaagacagatgaattagaaaaggttaggttacggcccggcgga
aagaaaagatacatgctaaaacatatagtatgggcagtgaatgaattagagagatttggattggcagagagccgg
ttgggatcaaaagaaggatgccggaaaattcggaaagtcttaggaccattagtaccaacaggttcagaaaattta
aaaagtctttataataccgtctgcgtcatcttttgcctgcacgcagaagagaaagtgaaagatactgaggaagca
aaaaagatagcacagcgacatctagcagcggacacagaaaaaatgccagctatgagtaaaccaagtaaaccaact
agccgcctagcttatccagtgcagcaaatagctggcaattattcccacctgccgctaagcccccgaaccttaaat
gcctgggtaaaattagtagaagagaagaagttcggggcagaagtagtaccaggatttcaggcactatcagaaggg
tgcacccccttatgatattaatcagatgttaaattgtgtaggagaacatcaggcagccatgcaaattattaggga
gataattaatgaggaagcagcagactgggaccaacagcatccatcgccaggcccaatgccagcagggcaactcagg
gaaccaagagggtcagacatagcgggaaccaccagcacagtagaagaacagatacagtggatgtacagaccccaa
aatcctgtcccagtgggaaacatctatagaaggtggattcagttagggctacagaaatgtgtccggatgtacaat
cctactaatatactggacataaagcaagggccaaaagaacccttccaaagctatgtagatagattctacaaaagc
ttacgggcagagcaaacagacccagcagtaaaaaattggatgacacaaacactgctgattcagaatgctaaccca
gattgcaaattagtgcttaagggcttgggaatgaaccccacattagaagaaatgctaacagcctgccaggggata
ggaggcccagggcagaaggcaaggctaatggctgaggctttaaaagaagccttgacaccttccaccaatccgttt
gccgccgctcaaccaagagcagggaagaggacagtgacatgctggaactgcggcaaggcgggacatacagccagg
cagtgcaaggccccctagaaggcag
>#42 KP004991 1308 nt
atgggtgcgagagcgtctgtgttgtcagggagtaaattggatgagtgggaaaaaatccggttaaggccaggatct
aaaaagaaatatctgctaaaacatttagtatgggcaagcagggagctggaaagatttgcttgtaatcctgagcta
ttagaaacagcagaagggactgagaagctactgcagcagctagaaccagctctcaagacagggtcagaaagcctg
cagtcactctggaacacgatagtagtactctggtgtgttcacaacagattcaaagtggctgatacacagcaggca
atacaaaagttgaaggaagtgaaggggcataaaaacacaagtgcgcagcagacagatcaaaggagctcgcagcag
acaggacaaaattaccctatagtaccaaatgcacagggacaaatgacgcatcagcccctctcccccagaacttta
aatgcatgggtaaaggcagtagaagaaaaggcctttaaccctgagattattcctatgtttatggcattatcagaa
ggagctatcccatatgacatcaatactatgctaaatgccataggaggacatcaggggggccctacaagtgctgaag
gargtcatcaatgaagaagcagcagattgggacagaactcacccgccagtggtgggaccattgccaccagggcaa
ataagggaaccagcaggaagtgacattgctgggacaactagcacccagcaagagcaggtccactggactactagg
gctaacaatcctatcccagtaggagatatctacagaaaatggatagtaatggggttaaacaagatggtaaaaatg
tacagtccagtgagcatcttagacataaagcagggaccaaaagaaccatttagagattatgtagacaggttctay
aaaactttaagagctgaacaagcaacccaagaagtaaaaaattggatgacagagacactgcttgttcagaatgcc
aatccagattgcaaacagattctaaagtcattagggccaggggctaccttggaggaaatgatgatagcctgtcaa
ggggtaggaggaccaactcataaggctagagtactagcagaagcaatggcctctgtccaccaagatctaaaagga
ggatacacagcagtattcatgcaaagagggcaaaacccaaataagagagggcctataaaatgttttaactgtgga
aaagaagggcatttagcaaaaaattgtcgagcacctagaagaaagggttgctggaaatgtggacaggagggtcat
caaatgaaagattgcaaaaatggaaggcaggca
>#43 FJ424866 1293 nt
atgggtgcgagagcgtcagtgttgacaggggggacgattggatgcatgggaacgaattaggcttaagccaggaagt
aagaaaaagtatatgctaaaacatgtagtatgggcaagcagggagctggaaagatttgcatgtaatcctgagctt
atggaaacagccgagggctgtgaaaaaattattagggcaactagaatcagctctcaaaacagggtcagatggcttg
cgatctctgtggaacactttggtagtgctgtggtgtgtccaccggagaattgatgtatcagacactcagcaagcc
ttagaaaaatttaaggaagtcatgtcaaaaagaaagtcagacagccgccctgaaacaagtcagaactatccagtg
gtagcaaatgctcaagggcagatggtccatcagccgctgtctcccaggactctaaatgcctgggtgaaagcggta
gaagaaaaggccttcaatccagaaatcatccctatgttcatggcattatcagaagggtctatcccttatgatatt
aatactatgctgaatgccattggaggacatcaggggggcattacaggtattaaaggaagtcatcaatgaggaagca

gcagaatgggacaggatgcacccggcagtggtgggacctctgccagtgggacaactgagggagccgtcaggggga
gatattgcagggacaacaagtactcaacaagaacaagtagcttggataacaaggaataacaaccctgtgccagtg
ggtgacatatataggaaatggatagtgctagggttaaacaaagtagtaaaaatgtactgtccagtcagtatccta
gatattaagcagggacctaaggaaccattccgagactatgtagacagattctacaaaaccctcagagcagaacag
gcaagtcaagatgtaaaaaattggatgacagaaactttgttaattcagaatgctaatcctgattgtaagcaaatt
ctaaaagccctaggcccaggggcaaccctagaagaaatgatgaatgcttgtcagggagtggggggacctactcac
aaagcaagagtcttggcagaggcaatggcagcagcccactcagatttaaaaggaggatatacagcagtctttatg
caaaagggggaaacaatcaagggattaagaaggaccagtaaagtgcttcaattgtgggaaagtaggacatata
gcaaaaaattgtagggcacccagaagaaagggatgctggaaatgtgggcaagatgggcaccaaatgaaagactgc
agatcaggcagacaggcg
>#44 KP004990 1287 nt
atgggtgcgagagcgtcagtgttaacaggggggtcgattggatgcatgggaaagaattaggctcaagccaggaggt
aagaaaacctatatgctaaaacatgtagtatgggcaagcagagagctggaaagattcgcttgtaatcctgagctt
atggagacagcagaaggttgtgagaaattactaggacagcttgagccagctctcaaaacaggttcagaaggcctg
cagtcgctttggaacaccctggtagttctatggtgtgttcacaagcgaatagacgtagccgacacccagcaggcc
attacaaaatggaaggaggaaatgcaaaaacgaagaaaagagaaagaaagcagccccggaacaagtcaaaactat
cctatagtgcagaatgcgcaggggcaaatgatacatcagccactgtctcccaggactttaaatgcttgggtgaag
gcagtagaagagaaagccttcaaccctgagattatccctatgtttatggccttatcagaaggatctataccttat
gacattaatactatgcttaatgcaataggcgggcatcagggagcattacaggtgttgaaagatgtgattaatgag
gaagctgcagaatgggacaggttacaccctgccccgtagggccgttaccgccagggcagataagggaacctaca
ggaggggacattgcaggaaccaccagtactcagcaagagcagatagcatggataacaaaaaatcctcctatacca
gtaggagacatctatagaaaatggatagtgttgggactcaataaagtggtaaaaatgtatagccctattagcatc
ctggacataaagcagggacctaaggagccattcagagattatgtagataggttctataaaaccctcagagcagaa
caagccagtcaggatgtaaaaaattggatgacagataccttattagtacaaaatgctaatcccgattgcaagcag
attctgaaagccctaggaccaggggccaccttggaggaaatgatgaatgcttgtcaaggagtaggggggaccaaca
cataaggccagggtcttggcagaagctatggcagcagccaaccaagcyagccaagacttgaaaggggggatacaca
gcagtctttatgcaaagaggacagagagggccagttaagtgctttaactgtggaaagctagggcatatagcaaag
aactgtagagcaccgaggagaaaaggatgctggaaatgtggacaagagggtcatcagatgaaagactgcaaaata
ggaaaacaggca
>#45 KP004989 1287 nt
atgggggcgagagcgtcagtgttaacaggggggccgattggatgcatgggagagaattaggcttaagccaggaagt
aagaaaacatatatgctaaaacatgtagtatgggcaagcaaagagctggaaagatttgcatgtaatcctgagctt
atggaaacagcggatggctgtragaaattattagggcagttagaaccagctcttaaaacaggctccgatggcctg
cagtctctttggaacaccctagtagttctgtggtgtgttcacagaagacaggaagtaagtgacacccagcaggct
attgcaaaatggaaggaggaaatgcagaagaggaaaaaggcacaagagggcagcactggaacaagtcaaaattat
cctattgtgcagaatgcccaaggacaaatggtccatcagccgctgtctcccaggacrttraatgcctgggtaaag
gcggttgaagaaaaagccttcaaccctgaaatcatccctatgtttatggccttatcagaagggtctataccctat
gacatcaatactatgctcaatgcagtaggtgggcatcagggagcgttacaggtgttgaaggatgtaatcaatgag
gaagctgcagaatgggatagaacacatcccgccccagtagggcctttaccaccagggcaaattagagaaccaaca
ggaggggatattgcaggaagcactagcactcagcaggaacaaatagcatggataacaagagccaatcctatacca
gtaggagacatctatagaaagtggatagtgctgggacttaataaggtggtaaaaatgtacagccccattagcatc
ttggatataaagcagggacctaaagaaccatttagagattatgtggatagattctacaaaaccctcagagcagaa
caagccagccaggatgtaaagaattggatgacagaaactttactaatrcaaaatgctaatccagattgtaagcaa
attttgaaagctctgggaccaggggccaccctggaagaaatgatgaatgcctgtcaaggagtaggggggaccaaca
cataaggccagggtcttggcagaagctatggcagcagccaatcaagccagccaagacttgaaaggagggtataca
gcagtgttcatgcaaagaggacagagagggccagttaaatgctttaactgtggaaaggtcggacatatagcaaag
aactgtaaggcacctaggagaaaagggtgctggaaatgtggacaggaaggtcaccaaatgaaagattgtaaatca
gggagacaggcw
>#46 FJ424863 1293 nt
atgggtgcgagagcgtcagtattgacaggggggacgattggatgcgtgggaacgaattcggcttaagccaggaagt
aagaaaaagtatatgctaaaacatgtagtatgtgcaagcagggagctggaaaggtttgcatgtaatcctgagctt
atggaaacagcygagggctgtgaaaaattattagggcaactagaaccagctctcaaaacagggtcagatggctta
cgatctctgtggaacactttggtagtgttgtggtgtgtccaccggagaattgatgtatcagatactcagcaagcc
ttagaaaaatttaaggaagccatgtcaaaaagaaagtcagacagccgccctgagacaagtcagaactatccagtg
gtagcaaatgctcaagggcagatggtccatcagccgctgtctccaaggactctaaatgcctgggtgaaagcagta

gaagaaaaggccttcaatccagaaatcatccctatgttcatggcattatcagaagggtctatcccttatgatatt
aatactatgctgaatgccattggtggacatcagggagcattacaggtattaaaggaagtcatcaatgaggaagca
gcagaatgggacaggctgcacccggcagtagtaggacctctgccagtaggacagctgagggagccgtcaggggga
gatattgcagggacaacaagtactcaacaagaacaagtagcttggataacaaggaataataaccctgtgccagtg
ggtgacatatacaggaaatggatagtgctagggttaaacaaagtagtaaaaatgtactgtccagtcagtatccta
gatattaagcagggacctaaggaaccattccgagactatgtagacagattctacaaaaccctcagagcagaacag
gcaagtcaagatgtgaaaaattggatgacagaaactttgctaattcagaatgctaatcctgattgtaagcaaatt
ctaaaagccctagggccgggggcaaccctagaagaaatgatgaatgcttgtcagggagtgggaggacctactcac
aaagcaagagtcttrgcagaggcaatggcagcagcccactcagatytaaaaggggggatatacagcagtctttatg
caaaagggggaarcaatcaagggattaagaagggaccagtaaaatgcttcaattgtgggaaagtaggacatata
gcaaaaaattgtagggcacccagaagaaagggatgctggaaatgtgggcaagaggggcaccaaatgaaagactgc
agatcaggcagacaggcg
>#47 FJ424864 1293 nt
atgggtgcgagagcgtcagtgttgacaggggggacgattggatgcatgggaacgaattaggcttaagccaggaagt
aagaaaaagtatatgctaaaacatgtagtatgggcaagcagggagctggaaagatttgcatgtaatcctgagctt
atggaaacagccgagggctgtgaaaaattattagggcaactagaatcagctctcaaaacagggtcagatggcttg
cgatctctgtggaacactttggtagtgctgtggtgtgtccaccggagaattgatgtatcagacactcagcaagcc
ttagaaaaatttaaggaagtcatgtcaaaaagaaagtcagacagccgccctgagacaagtcagaactatccagtg
gtagcaaatgctcaagggcagatggtccatcagccgctgtctcccaggactctaaatgcctgggtgaaagcggta
gaagaaaaggccttcaatccagaaatcatccctatgttcatggcattatcagaagggtctatcccttatgatatt
aatactatgctgaatgccattggaggacatcagggggcattacaggtattaaaggaagtcatcaatgaggaagca
gcagaatgggacaggatgcacccggcagtggtgggacctctgccagtgggacaactgagggagccgtcaggggga
gatattgcagggacaacaagtactcaacaagaacaagtagcttggataacaaggaataacaaccctgtgccagtg
ggtgacatatataggaaatggatagtgctagtgttaaacaaagtagtaaaaatgtactgtccagtcagtatccta
gatattaagcaggggcctaaggaaccattccgagactatgtagacagattctacaaaaccctcagagcagaacag
gcaagtcaagatgtaaaaaattggatgacagaaactttgctaattcagaatgctaatcctgattgtaagcaaatt
ctaaaagccctaggcccagggggcaaccctagaagaaatgatgaatgcttgtcagggagtgggggggacctactcay
aaagcaagagtcttggcagaggcaatggcagcagcccactcagatttaaaaggggggatatacagcagtctttatg
caaaaggggggaaacaatcaagggattaagaagggaccagtaaartgcttcaattgtgggaaagtaggacatata
gcaaaaaattgtagggcacccagaagaaagggatgctggaaatgtgggcaagatgggcaccaaatgaaagactgc
agatcaggcagacaggcg
>#48 FJ424871 1293 nt
atgggtgcgagagcgtcagtgttgacaggggggacgattggatgcatgggaacgaattaggcttaagccaggaagt
aagaaaaagtatatgctaaaacatgtagtwtgggcaagcagggagctggagagatttgcatgtaatcctgagctt
atggaaacagccgagggctgtgaaaaattattagggcaactagaaccagctctcaaaacagggtcagatggcttg
cgatctctgtggaacactttggtagtgctgtggtgtgtccaccggagaatagatgtctcagatactcagcaagcc
ttagaaaaatttaaggaagccatgtcaaaaagaaagtcagacagccgccctgaaacaagtcagaactatccagtg
gtaacaaatgcgcaagggcagatggtccatcagccgctgtctccaaggactctaaatgcctgggtaaaagcggta
gaagaaaaggccttcaacccagaaataattcctatgttcatggcattatcagaagggtctatcccttatgatatt
aatactatgctgaatgccattggaggacatcagggggcattacaggtcttaaaggaagtcatcaatgaggaagca
gcagaatgggacaggcagcacccagcagtggtaggacctctgccagtgggacaactgagggaaccatcaggggga
gatattgcaggaacaacaagtactcaacaagaacaagtagcttggatgacaaggaataataatcctgtgccagtg
ggtgacatatacaggaaatggatagtgctagggttaaacaaagtagtaaaaatgtactgtccagtcagtatcctg
gatattaagcagggacctaaggaaccattcagagactatgtagacagattctacaaaaccctcagagcggaacaa
gcaagtcaagatgtaaaaaattggatgacagaaactttgctaattcagaatgccaatcctgattgtaagcaaatt
ctaaaagccctaggcccagggggcaaccctagaagaaatgatgaatgcctgtcagggagtgggaggacctactcac
aaggcaagagtcttggcagaagcaatggcagcagcccactcagatctaaaaggggggatatacagcagtctttatg
caaaaaggggggaaacaatcaagggattaagaagggggccagtaaaatgcttcaattgtgggaaggtaggacatata
gcaaaaaattgtagggcacccagaagaaagggatgctggaaatgtgggcaagatgggcaccaaatgaaagactgc
agatcaggcagacaggcg
>#49 JN091690 1371 nt
atgggtgcgagagcgtcagtgttgcggggagagaagcttgatagatgggaagccataaagcttagacccggaggc
aggaaaagatatttgttaaaacacctaatatgggccgggagtgagttacagcgcttcgcgatgaatcccggcctc
atggagagcaaagaaggctgctggaaaatcatcctccagctacagccatctgtagacattggttctccagaaata
atctccttgtttaacaccatttgcgtgctttattgcgtacatgaaggagaaaaagtgcaaaatacggaagaagca

gttaaaattgtgaaagtgaaactaacaaagcaagaaagtagctccgcagcgacatctagcggacagaataaaaat
acagaagagacacctagtggtagccaagagaatgcaggaagagcagcagcgccatctagtggcagactgtatcca
gttatcacagacatgcagggagcagcaaggcatcagcctatatcacccagaactttaaatgcttgggtaaggaca
gtggaagacaaagggttcaatccagaagtgataccaatcttttcagctttatctgaggggggctaccccttatgat
ttaaatactatgcttaatgctattggggatcatcaagcagcaatgcaaatgctaaaggaagtaatcaatgaggag
gcagcagactgggatagaacacacccagctcatgcaggaccccaacaagcggggatgctgcgagagccaacagga
gctgatattgcaggaaccactagctcagtacaagagcaagtgcagtggatgacaacccctcaggcacaaggagga
gtaccagtaggggacatctataaaagatggataattatagggttaaacaaattagttaggatgtacagccctgtt
agtattttagacattaaacagggaccaaaagaaccattcagagattatgtagacaggttctacaaatgcatcaga
gcagaacaggcctctcaaccagtaaagacttggatgacagaaactctgttagtccagaatgcaaatccagactgt
aaacacatttgaaggcactaggacaaggagcaaccttagaggaaatgcttacagcttgccagggagtaggagga
ccttcccataaggcaaaaatactggcagaagcaatggcatcagcaaacagtgcagttggggggaataaatatgtta
caaggaggaaagaaacccctgaggaaaggccagctgcagtgtttcaattgtgggaaaccagggcatacagccaga
aactgcagagccccaaggaaaaagggctgctggaggtgtggtcaggaaggacatcagatgaaagactgcacagga
agacccaacaattcaggggta
>#50 AF447763 1386 nt
atgggtgcgagagcgtcagtgttgaggggagataagctggatacatgggaatccataaggcttaaatccagaggc
aggaaaaaatatttaataaaacatctagtatgggccggaagcgaactacagcgtttcgcgatgaatcccggtctc
atggagaacgtagaaggctgctggaaaatcatcctccagctgcagccttcggtagacattggttctccagaaatc
atttctttgtttaataccatctgtgtactctactgcgtacacgcaggagaaagagtccaagatacggaagaagca
gtcaaaattgtgaaaatgaaactaactgtacagaaaaataactccacagcgacatctagtggacaaagacagaat
gcaggtgaaaaagaggaaacagtgccacctagtggcaatacaggaaacacagggagagcaacagagacacctagt
gggagtagactatacccagtgataactgatgcacagggagttgcaaggcatcagcctatttcacctagaactcta
aatgcctgggtaagggtaatagaagaaaaagggtttaatccagaagtaataccaatgttctcagcattgtctgag
ggagcaaccccttatgatctaaatagtatgctcaatgctgttggggaacatcaagcagcaatgcaaatgttgaag
gaagtcatcaatgaggaagcagcagagtgggacagagcacatcccgctcatgcaggaccccagcaagcagggatg
ctaagagagcccacaggggcagatattgcagggaccactagtacgctacaagaacaagtactgtggatgacaacc
ccacaggcacaaggaggagtgccagtaggagacatctataaaaggtggataattttaggattaaataaattagtc
agaatgtacagccctgttagcattttggacataaaacagggaccaaaagaaccattcagagattatgtagacaga
ttctacaaaacaatcagagcagaacaagcatctcaaccagtaaaaacttggatgacagaaactttactggtacaa
aatgcaaacccagattgtaagcatatcttaaaagccttggggcaaggagcaacattagaagaaatgctcacagcc
tgtcaaggagtgggaggaccctctcataaggcaaagattctggctgaagcaatggcctcagcaacagcaggggga
gtaaatatgctgcagggaggaaaaagaccacccttaaaaaagggtcagctgcagtgttttaactgtgggaaagta
ggccatacagcaagaaattgtagggctccaagaaagaaaggttgctggaggtgtggacaagagggacatcaaatg
aaggactgcaccaccagaaacaacagcactggggta
>#51 JQ768416 1389 nt
atgggtgcgagagcgtcagtattaaggggagaaaagcttgatacttgggaaagcattaggcttagacccggaggc
aagaaaaagtacttaataaaacatctagtatgggccggaagcgagctcgaacgcttcgcgatgaatcccggtctt
atggaaaacaaagaaggctgctggaaaatcatcctccagctacagccctcaatagacattggctctccagaaata
gtctccttgtacaacactgtctgtacactattttgtgtacacgcaggagaaaaggtacgggacacggaagaagcc
gttaaaattgtgaaagtaaaactaactacagtaaaagatgatgccgcagcgacatctagcggacaaggtcagaat
gcaggaggaagagggacagcagtgacacctagtggtaaaagtgaaaatgccagtagagtaacagtgccacctagt
gagggaggaaggttatatccaattgtgactgaccttcagggaacagcaagacatcagcctatttcacctagaact
ttaaatgcttgggtaaaaacagtggaagaaaagggatttagtccagaagtcattccaatgttctctgctttatct
gaaggggcaaccccttatgatttaaattccatgcttaatgctattggagaacatcaggctgcgatgcaaatgtta
aaggaagtaatcaatgaggaggcagcagagtgggacagactacatcccgctcatgcagggccccagcaggcaggg
atgctaagggagccaacaggagcagacattgcagggaccactagtacaacacaagaacaaatacagtggatgaca
acacctccagcacaaggaggagtaccagtaggggatatctataagagatggatcattctagggcttaataaattg
gttagaatgtataacccagtgagtattctggacataaagcaagggccaaaggaggcatttagagattatgtagat
agattctacaaaactatcagagcagaacaagcctctcagccagtgaaaacatggatgacagaaaccttgctgata
caaaatgcaaatccagattgcaagcatattctaaaagccttaggacagggagcaacattggaagaaatgctaaca
gcctgtcagggggtaggaggccctacccataaggcaaaaatcctggctgaagcaatggcaacagctactaataca
gtaggagtaaatatgctgcagggaggtaagaaaccattaagaaaggggcagcttcaatgttttaattgtggaaaa
ttagggcatacggcaaaaaactgtagagccccaaggaaaaaaggctgttggaggtgtggacaggagggacatcaa
atgaaagactgcacagccagaactaacaatactggggtt

>#52 JQ866001 1377 nt
atgggtgcgagagcgtcagtattgaggggagagaagttagatcgatgggaatcggtaaggcttcgcccaggaggc
aagaaaaaatatcttataaagcatctagtgtgggccggcaatgaattacagcgattcgctttgaatcccggcctg
atggaaacagcagatggttgcagaaagatcattatgcaactctcaccatcagtagaaatagggtcaccagaaata
atctccttatttaacaccattgcagtgttacattgcatacatgaaggagaaaaaattcaggacactgaagcagca
gtaaaaattgtgaaagagaaactaaaaataacgagcagtgaaacaacaggaaatgaggaaaccgcaaaagggaaa
aaagagacagcagtgacatctagtggtcagacagaaaaaatctaccccacattgccatctagtggtcagacaggg
agaaactaccctattgtttttgatgctgcgcaacagccaaggcatcagccaatttcacccagaactcttaatgct
tgggtaaaaacagtagaggagaaaaattttaagccagaagtaatcccaatgttctcagccttagcagagggagcc
atcccacatgatcttaatacaatgctaaatgcaattggggaccatcaaggggcaatgcaagtgctgaaggaagtt
atcaatgaagaagcagctgattgggacaggttacacccaactcatgcagggccgctagtcccagggactctaaga
gagcccacagggagtgacattgcaggaacaacaagtaccattgtggagcagatacaatgggtaactactcctcaa
aatcaaggaggagttccagtaggagatatctataaaagatggatcatcctagggctgaataaattagtgaaaatg
tacagccctgtaagcattctagatataagacagggaccaaaagaaccattcagagattatgtagacaggttctac
aaaactatcagagcagaacaagcttcccagccagtaaaaaattggatgacagaaactttactggtacaaaatgct
aacccagattgtaagaccatactgaaagcattgggaccaggggcaaccttggaagaaatgctgactgcctgtcag
ggagtaggggggaccagcacataaggcaagggtcctagcagaagccatgcagcaaacacagcagaatgttaatatg
gtacaggggagagcagcgcctcaaggaaaaagaggggcaggaaacataaaatgttttaattgtggaaagataggg
catacagcaagaaattgcagagccccaagaagaaagggatgttggaagtgtggacaggaaggtcatcagctgaag
gactgcacacagtcaaaaagtggagta
>#53 DQ373065 1308 nt
atgggtgcgagagcgtcagtattaacaggaggaaaattagatcaatgggaaaaaatttatttgagaccagggga
aagaaaaaatacatgatgaaacacttagtatgggcaagcagggagctggaaagattcgcgtgtaacccaggtctc
atggacacagcagaaggttgtgcccaattactcagacaattagaaccagctctcaaaacagggtcagaaggactg
cgctctctatttaacaccctggcagttctttactgtgttcataataacataaaggtacagaatacacaggaagct
ttagaaaaattaagagagaaaatgaaagcagaacaaaaagaacctgaaccagaacaagcagcaggggcagcggca
gcacctgaaagcagtatcagtaggaactatcctctggtacaaaatgctcaagggcaaatggtacatcagccgctg
tcacccagaaccttaaatgcttgggtaaaagtggtagaagaaaaggcctttaatccagaagtaataccaatgttt
atggccttgtcagaaggggcaacgccccaagacttaaacaccatgttaaatacagtaggggggacatcaggcagca
atgcagatgctgaaggaagttatcaatgaggaagcagcagagtgggacaggggacatccagtacatatggggcca
ataccaccaggacaagtaagggagccaagaggaagtgatatagcaggaacaactagtaccctggcggagcaagta
gcatggatgactgccaatcctcctgttccagtaggagatatttatagaagatggatagtcttgggattaaataag
attgtaagaatgtatagtcctgccagcattctagacatcaaacagggaccaaaagagacttttagagattacgta
gataggttttacaaaactctaagagcagagcaggccactcaggaagtaaagaattggatgacagagacactctta
gtacaaaatgcaaatcctgattgtaaaaatatcctgagagcattaggaccaggagcctccctggaggaaatgatg
acagcatgccaaggggtgggaggaccagctcataaggcaagagtactagcagaagccatgacacaggcacaaaca
gcaactagtgtttttatgcaaagaggaaattttaaaggcataagaaagaccatcaaatgctttaattgtggtaaa
gagggccatctagcaagaaactgtaaggcacctagaaaaaagggctgctggaaatgtgggcaagagggacatcaa
atgaaggactgcagaagtggagaaaggcaggcg
>#54 AF103818 1305 nt
atgggtgcgagagcgtcagttctgacaggggggacgattagatgcttgggaaaagattcggcttagacctggggga
aagaaaaagtatatgatgaaacatttagtttgggcaagcagggagctggaaagatttgcttgtaacccaggtcta
atggaaacagcagacggctgtctccaattattaaaacagttagaaccagctctaaaaacaggatcggaaggcctg
cgatcgctctttaacaccttagcggtcctttggtgtgtccacagtagggtgacagtggaggatacgcagcaggcc
ttagtgaaactgaaagaggtagtgcagttgcagaaacaacaggaagaaaaagaacaacagcaacaggaggcaagt
ggcagtaacattggtagcagcaattacccagtaatacagaacgctcagggacagatggtacatcaggcaatgtct
cccagaactttaaatgcctgggtaaaagcggtagaagaaaaggcctttaacccggaagtcatcccgatgttcatg
gccttatcagaaggggcgactccccaagatgtcaacactatgctcaatgctataggggggacatcagggagcaatg
caagttttaaaggaagccatcaatgaggaagcagcagattgagatagaacacatcctctgcatgcaggccctata
gcaccagggcagatgagagaacccaggggaagtgacattgcaggaaccaccagcaccttgcaagaacaagtgggc
tggatgacagcaaacccacccattccagtaggagacatatatagaagatgggtggtcctagggcttaataaagta
gtaaaaatgtactgtcccgttggtatcttagatatcaagcagggacctaaagagccctttagagactatgtagac
aggttctacaaaacattaagagcagaacaagcttctccagaagtaaaaaacctggatgacagagacccttttggta
caaaacgctaatccagattgtaaaactattttaagagccttgggacctggagccaccctagaagaaatggtgaca
gcatgtcaaggagtgggaggacccgctcataaggcaagggtcttagctgaagccatgtgccaaatgaaaaacccc

tcaagcgtatttctacagaaaggaaacgctgggaagccagggagaaaaatcaaatgttttaattgtggcaaagag
ggacatctagccagaaattgcagggctccaagaagaaaagggtgctggaagtgtgggcaggaaggacatcaaatg
aaagactgcacagcaggaaacagacaggct
>#55 EF535993 1290 nt
atgggtgcgagagcgtcaatattaacgggggacgattagatgcttgggaaaaaattaggctaaggccaggggga
aagaaaaaatatatgatgaaacatttagtctgggcaagcagggagctggaaagattcgcttgtaaccctggtcta
atggaaacaggagagggatgtgaacagctattaaaacagttagaaccagctctcaaaacaggatcagaaggcctg
cgatcgcttttaacactctagcggtactttggtgtgttcataaaagaattccagtggaagacacacagcaggct
ttaactaaactgaaagaaacagcggcccgrgaacaggaagtggcccaaccacagccgcagcaagacagcaatgtc
agtagaaacttcccagtagtacagaatgcacaagggcaattgatgcatcagcccatgtcacctagaactttaaat
gcttgggttaaggtagtggaagaaaagaattttaatccagaagtgataccaatgtttatggccctgtcagaggga
gcgactccccaggatgtcaatactatgcttaatgctataggagggcaccagggagctatgcaggtgttaaaggaa
gtcatcaatgaggaagcactggaatgggacaggacccatcctgtgcatgcaggtccaatagcaccaggacaactg
agagaggcacgaggaagtgacatagctggaaccactagcactctagcagaacagattgcttggatgacagcaaat
ccacccgttccagtaggagaaatctatagaagatgggtggttctagggcttaacaaagtggtaagaatgtatagc
ccagtcagtatcctagacattaagcagggacctaaggagcctttcagggactatgtagatagattctacaaaacc
ctgagggcagaacaggcaacacaggaggtaaaaacctggatgactgataccctcytggtacaaaatgctaaccca
gactgtaaaaatatcttgagggccttaggaccaggagccaccttagaagagatgatgacagcatgccaaggagtg
ggaggaccagcccataaggctagggttttggctgaagctatgagccaagtacaaaactccacaagtgtgtttttg
cagaggggcaataatgggagaccaactaggaaaatcaaatgctttaattgtggcaaagaagggcatttagcaaga
aactgcagggccccaagaaggaaggggtgctggaaatgtggacaagaggggcatcagataaaagattgtacagct
ggagacagacaggct
>#56 AY169968 1314 nt
atgggtgcgagagcgtcagtgttaacaggggggaaattggatcgctgggaaaaagttcggcttagacccggggga
aggaaaaaatatatgatgaaacatttagtatgggcaagcagagagctggaaagattcgcatgtaaccccgggcta
atggaaagtgcagaaggatgtcttaaattgctacagcagttagagccagctctgaaaaccggctcggaagggctg
cgatctttatttaacactctagcagtgttatggtgtgttcacagtaacatcgaggtagaagacacacagaaagca
ttagaacagcttaagaaacaccatggagaacaagtaagaaaaaatgagaacacagaaaacaatgaaagcagacag
ggcgctagtgcttcctctggcactagtgggaattaccctatagtacagaatgctcaagggcagatggtacatact
cccatctcccctagaaccttaaatgcatgggtaaaagtagtggaagagaaggcttttaatcctgaggtaatacca
atgttttcagctctgtcagaaggagctctacctttggatgttaacacaatgctgaatgctgtaggggacatcag
ggagccatgcaagttctaagagaggtcatcaatgaagaagctgcagaatgggatcggatgcatcctgttcatgcc
ggccccatagctccaggacagctaagagaaccacggggtagtgatatagcagggactaccagcacattgcaggag
caaattggatgggcaacagcaaacccacccatcccagtaggagacatctatagaagatgggtaattctagggcta
aacagagtagtaaaaatgtattgtccagtaagcatccttgatataaagcaaggaccaaaagagccctttaaagat
tatgtagacaggttttataaaaccatgagagctgagcaagcttctcaggaagtgaaaacttggatgacagacacc
cttttggtgcaaaatgctaacccagattgcaagcaaattctaaaggccctgggtccggggggcctccctagaagaa
atgatgacagcctgtcaaggggtaggaggaccagcccataaggcaagagtcttagctgaagctatgacaatggtc
caaagtcaagcaagaacagacattttctttcagaaagggccaggggcaaccccaagaagaaaaataaaatgctat
aattgtggaaaggaaggccatctggccagaaactgtaaggcaccaaggagaaagggctgctggaaatgtgggcag
gagggacaccaaatgaaagactgttcagggagacaagta
>#57 AF382828 1305 nt
atgggtgcgagagcgtctatattaaccggcgggcgattagatgcctgggagaaaattaggctcagaccaggggga
aggaaaagatacatgatgaaacatatagtatgggcaagcagggagctggaaagattcgcttgtgaccctggtcta
atggaaacaaaagaaggctgttatcagttattgaaacagctagaaccagctcttaaaacaggatcagaaggatta
cggtccttatttaatactttggctgtgttatggtgtgttcatgctagaatagaagtagaagatacacagcaagcc
ttacaaaagttgaaagaatttgtgcaaaaggaaacagcaacagcacaggaagcagcagcaaagcaaagtgagtca
aacaccactgagataagcagaaattacccagtggtgcagaatgcacagggacagatgatacatcaggccatgtca
cccagaactttaagtgcctgggttaaagcagtagaagaaaaagcctttagcccagaagtcattccaatgttcatg
gctttatcagaagggggcaacaccccaagatgtgaacacaatgctgaatgctataggaggacaccaaggagctatg
caggtactaaaagaggtaatcaatgaagaagcagcggagtgggatagattacacccattacatgcaggaccagtg
gcaccaggtcaaatgagagagccaagaggcagtgacattgcaggtactactagtaccttgcaagagcaagtagga
tggatgacatccaatccaccagtccctgtaggagagatttacagaagatgggtggtattagggcttaataaggtg
gtaagaatgtattgccctgttagcatcttggacataaaacaaggccgaaggagccttttagagactatgtagat
agattctacaaagttttaagagcagaacaggcttcacaagatgtgaaaaactggatgacagagaccctcctggta

cagaatgccaacccagattgtaagcaaatacttagggcactaggaccaggagctaccctagaggaaatgatgaca
gcctgccaaggagtaggaggcccggcacataaggctagagttctagcagaagctatgagccaaatacaacagaca
agctcaatattcatgcaaagagcaggggaagaactcctcctaggagagtcaagtgcttcaattgtggaaaggaa
ggtcatatagcaagaaattgtaaagcccctagaagaaagggatgttggaagtgtggaaaagaaggacatcagatg
aaggactgccaaaatgaaggaagacaggtg
>#58 EF535994 1290 nt
atgggtgcgagagcgtcagtattaacgggaggaaagttagatgattgggaaaaaatttatctccggccagggga
aagaaaaaatatatgatgaaacatttagtatgggcaagcagggagctggaaagattcgcttgtaaccccggccta
atggacacaacagaaggatgtgaacaattaattaaccagttgcagccagctctcacaacaggatcagaaggacta
agatctttatttaacaccctagcagtcctctggtgcgtacatcaaagaattagagtaaaagacacaaaagaggct
ttagacaaactagaagaagtgcagcaaaaaagcaaagtgaaagtaaaacaggaagccgcggcggcagaccccagc
ggcggcgagagcagaaacttcccgatagtaacaaatgctcagggacaaatggtccatcagtcgctctcacccaga
actttaaatgcatgggtaaaagtagtagaagaaaaggcatttagcccagaagtcatacccatgtttatggctcta
tcagaaggagccacccctcaggatttaaacaccatgctaaatacaataggggggacatcaggcagcaatgcaaatc
ttaaaagaagtgatcaatgaggaagctgcagagtgggacaggacacaccctatacatgcagggccaattcctcca
ggccaaatgagggaaccaagaggaagtgacatagcagggacaactagcaccattcaggaacaagtgggatggatg
accagtaaccctcctaccccggtaggagacatttacaagagatggatagttttagggcttaataaaatagtaaga
atgtatagccctgtcagtattctagatataaagcaaggaccaaaagaaccatttagagattatgtggacagattt
tacaaaactctaagagctgaacaagcaacccaagatgtaaaaaattggatgacagagaccttgctagtgcaaaat
gccaacccagattgtaagcaaattttaagagctctgggtccaggggcaaccttagaagaaatgatgacagcctgc
cagggagtaggggggcccctctcataaagccagagtattggcagaagcaatgagtcaggcaaacaccactgttatg
atgcaaagaggtaatgttaagattcagaaaagaattgtgaaatgctttaactgtggcaaggaaggtcacatagca
agaaactgtagagcccctagaagaaaaggctgttggaaatgtgggcaagaaggacaccaacagaaaaactgcccc
gatatgcaacgggcg
>#59 DQ373066 1269 nt
atgggtgcgagagcgtcaatattaaccggggaagattagatgcttgggaaaaaattaggctaagaccaggggga
aagaaaaaatacatgatgaaacatttagtatgggcaagcagagagctggaaagattcgcttgtaaccctggtcta
atggaaaatgcagaaggttgtgagaagttattgaaacagctagaaccagctcttaagacagggtcagaaggactg
cgctctttatttaacactttggcagtcctttggtgtgtacataagagaatagaagtggaagacacacaacaggct
ttacagaaactgaaagctgccgtgaacccagaacagggtgaaacaccatcagctagcgcgaacttccctgtagta
cgaaacgctcagggacagttgacgcataatccaatatcacccagaaccctaaatgcctgggtaaaagcagtagaa
gaaaaagctttcaatccagaggtcataccaatgttcatggcattatcagaaggggcaacgcctcaagacctcaat
actatgctaaatacaataggaggacatcaggcagccatgcaaattctaaaagaagtaatcaatgatgaggctgct
gagtgggacaggacacaccctacccatgcagggcctatagctccaggacaaatgagggaaccaagaggcagtgac
atagcaggcactactagctccctagaggagcaagtagggtggatgacagcaaaccctcctattccagtaggagat
atttatagaagatggatagtcctggggttaaataaaatagttaaaatgtacagtcctgttagcattttggacatt
aaacaaggaccaaaagagcctttcagggactatgtagacaggttctacaagaccttaagggctgaacaagcatca
caggatgttaaaaattggatgacagacaccctgttgatacagaatgctaacccagactgtaaaaacatcttaaga
gcattaggaccaggagccactctagaagaaatgatgacagcttgtcaaggggtaggaggaccctcacataaggct
agagtgctggcagaagccatgagccaggtaaacaaaacagaaatctttatgcaaagaggggacaaaatagacct
cctaataaaaagattaaatgctttaattgtggaaaagaaggacacctagcaaggaactgcagggcaccccgaaga
aagggctgctggaagtgtggacaggagggacatcagatgaaagattgtaatcaaaataatggaaggatt
>#60 DQ373064 1293 nt
atgggtgcgagagcgtcaatcttaacaggcggaaagttagatgattgggaaaaagttcggctaaggcccggggga
agaaaaagatatatgatgaaacatttagtatgggcaagcagggagctggagagatttgcttgtaaccccgggctt
atggaaacaacagaaggctgtgagcaattgattacacagttagaaccagctcttaagacagggtcagaaaacctt
aaatctttatttaacaccatagtggtcctctggtgcgtgcatcagagaatcgcgataaaagacaccaaggaggct
ttagatacactaacagcagttagacagaaacagcaaaaggaaaaggaagcagctgcagccgcggcaagggacaat
gacagtggtagcagaaactaccctatagtgacaaatgcacaagggcagccggtacaccaggccctctcacctaga
actttaaatgcctgggtcaaggtagtagaagaaaaggcatttagcccagaagtaatacccatgttcgcagcctta
gcagaaggagccacccctcaagatttaaataccatgctaaatacagtaggggggacaccaggcagccatgcagatc
ttaaaagaggtcattaatgaggaagcacaagaatgggataggctgcatcctatccatatggggccaatagcacca
ggccaaatgagagaaccaaggggaagtgacatagcaggaaccactagtactgtacaagaacaaataggatggatg
actagcaaccctcctataccagtaggagaaatttacaaaagatggataattatgggggctgaataaggtagtcaga
atgtacagccctgttagcatcctggacataaagcaagggccaaaagaaccattcagagactatgtggacaggttt

tacaaaacattaagagcagaacaagccacacaggaagtaaaaaattggatgacagacaccttgcttgtacagaat
gctaacccagattgcaaaagtatcctgagagcattgggtccaggagcctccttagaggaaatgatgacagcatgc
cagggagtcggggtccagcccataaagccagggtcttagcagaagctatgagtcaagcaaatgcagcaacagtc
attatgatgcaaaggggaaattttaaaggaccaaaaagagttattaaatgtttcaattgtggcaaagaagggcac
atagcaagaaattgtaaagccccaggagaaaaggctgttggaaatgtgggcaggaaggacatcggatgcaagat
tgtaacatggggaaggtt
>#61 KP861923 1278 nt
atgggtgcgagagcgtcagtattaaccggtagtaaattggatgattgggagaaaattaggttaaggccagggggt
aagaaaaaatatatgatgaaacatttagtatgggcaagcagggagctggacagatttgcttgtaaccccggcctt
atggaaacaggagaaggctgtgaacagttaattaaacagctagaaccagctctcggaacagggtcagagggattt
agatctctctttaatactttagtagtcctctggtgtgtacatcagagaattcaggtaaaagacaccaaagaagcc
ttagaccagctggcagaatggcaacaaaagcaaaagaaaaaggaagcagcagcgacagcagcagacagctcagtc
agcagaaactaccctgtagtagcaaatgcacagggacagatggtgcatcagccattatcacccagaaccttaaat
gcctgggtgaaagtggtagaagaaaaggcatttagcccagaagtaatacctatgttcatggcattagcagaagga
gccacccctcaagatttaaataccatgctgaatacagtaggggacatcaggcagccatgcaaatattaaaagaa
gtaattaatgaggaagcatcagaatgggataggatacatcccattcatatggggcctatagcaccaggccaaatg
agagagccaaggggaagtgatatagcaggaactactagtaccatacaggagcagataggctggatgactagtaac
cctcccattccagtgggagagatttataaaagatggatagttctggggctaaataaaatagtaagaatgtatagc
cctgtcagtattctagacataaagcagggaccaaaagaaccattcagagattatgtggacagattttataagacc
ttaagggcagagcaggccacacaggaagtaaaaacttggatgacagacaccttgcttgtgcagaatgctaaccca
gattgtaagaacatcttgagagcactgggtccaggagccaccctagaggaaatgatgacagcatgccagggagtg
gggggccctgcccataaggctagagtactggcagaggccatgagtcaagcaaatgctacagtaatgatgcagagg
ggaaattttaaaggccctaagagaactattaaatgctataactgtggcaaggaaggccacctagccaggaactgc
aaagcccccagaaggaaaggctgttggaaatgtggacaggaaggacacagaatgcaggattgtaaactaagcaag
gtt
>#62 AJ302647 1305 nt
atgggtgcgagagcgtctgtgttgtcagggagcaaattggatacatgggaacaaattaggttaaagccaggatgt
aaaaagaaatacagactaaaacatttagtatgggcaagcagggagctggaaagattcgcatgtaatcctgagcta
ctagaaactgcagagggcaatgaggaactgttacagcagttagagccagctctcaagacagggtcagaaagcctg
cagtcactctggaacacaatagcagtgctctggtgtgttcacaaaagatttaaagttgaagatacacagcaggca
atacagaaactaaaggaagtaatggggagcaggaagtctgcaggtgccgctaaggaagacacaagcgcaaggcag
acgggtcaaaactaccctgtagtagcaaatgcacagggacaaatggtacatcagtccctctcccccaggacttta
aatgcatgggtaaaggcagtagaagaaaaggcctttaaccctgaaatcatccctatgttcatggcattgtcagag
ggagctattccttatgatactaataccatgctaaatgccataggaggacatcaaggggctttacaagtgctaaaa
gaagtaatcaatgaggaagcagcagaatgggatagaactcacccacaagcggcagggccattgcctccagggcag
ataagggaaccaacaggaagtgacatcgctgggacaactagcacccagcaagagcaagttcactggattactagg
cccaaccaacctatcccagtaggagacatctatagaaaatggatagtgttagggttaaacaaagtagtaaaaatg
tacagcccagtgagcatcttagatattagacagggaccaaaggaaccatttagagattatgtagatcggttctac
aaaacattaagagctgaacaggcaactcaagaagtaaaaaattggatgacagaaaccctgcttgttcaaaatgcc
aacccagattgcaaacagattttgaaatcattagggccaggagctaccttagaagaaatgatgatagcttgtcag
ggagtaggaggaccaactcataaggccagagtactagcagaagcaatgtctgcagcccaagatctgaagggagga
tactcagcagtatttatgcaaagagggcaaaacccaggtaggaaagggcctataaaatgtttcaattgtggaaaa
gaaggacatctagcaagaaattgtcgagcacctagaaagaaaggttgctggaaatgtggacaggaaggtcaccaa
atgaaagattgcagaaatggaaaacaggca
>#63 AJ302646 1305 nt
atgggtgcgagagcgtctgtgttgacagggagcaaattggatgcatgggaacaaattaggttaaagccaggatgt
aaaaagaaatacagactaaaacatttagtatgggcaagcagggagctggacagatccgcatgtaatcctgagcta
ctagaaactgcagagggcaatgagaaactgttacagcagttagagccagctctcaagacagggtcagaaagcctg
cagtcactctggaacactatagcagtgctctggtgtgttcacaacagaattaaagttgaagatacgcagcaggca
atacagaaactaaaggaagtaatggggagcaggaagtctgcaggtaccgctaaggaagacacaagcgcaaggcag
acgggtcaaaactaccctatagtaacaaatgcacagggacaaatggtacatcagtccctctcccccaggacttta
aatgcatgggtaaaggcagtagaagaaaaggcctttaaccctgaaatcattcctatgttcatggcattgtcagag
ggagccattccctatgatactaataccatgctaaatgccataggaggacatcaaggggctttacaagtgctaaaa
gaagtaatcaatgaggaagcagcagaatgggatagaactcacccaccagcggcagggccattgcctgtagggcag
ataagggaaccaacaggaagtgacattgctggaacaactagcacccagcaagagcaagttcactggattaccagg

cccaaccaacctatcccagtaggagacatctatagaaaatggatagtgttaggattaaacaaagtggtaaaaatg
tacagcccagtgagcatcttagatattagacagggaccaaaagaaccatttagagactatgtagatcggttctac
aaaacattaagagctgaacaggcaactcaagaagtaaaaaattggatgacagaaaccctgcttgttcaaaatgcc
aacccagattgcaaacagattttgaaatcattagggccaggagctaccttagaagaaatgatgatagcttgtcag
ggagtaggaggaccaactcataaggccagggtattagcagaagcaatggctgcagcccaagatctgaagggagga
tacacagcagtatttatgcaaagagggcaaaacccaagtaggaaagggcctataaaatgtttcaattgtggaaaa
gagggacatctggcaagaaattgtcgagcacctagaaagaaaggttgctggaaatgtggacaggaaggtcaccaa
atgaaagattgcaaaaatggaagacaggca
>#64 AY169812 1308 nt
atgggtgcgagtgcgtccgtgttgacagggagtaaattggatgcatgggaacgaattcggttgaagccaggatct
aaaaagaaatatagactaaaacatttagtatgggcaagcagggagctggacagattcgcatgtaatcctgagcta
ctagaaactgcagagggcaatgagaagttgttacaacagttagaaccagctctcaagacagggtcagaaagcctg
cagtcactctggaacacaatagcagtgctctggtgtgttcacaacagatataaagttgaagatacgcagcaggca
atacaaaagttaaaggaagtaatggggagtaggaagtctgcaagtgccgctaaggaagaaacaagtgcaaggcag
acgggtcaaaattaccctgtagtaccaaatgcgcagggacaaatgatacatcaggccctctcccccaggacttta
aatgcatgggtaaaggcagtagaagaaaaggcctttaaccctgaaatcattcctatgttcatggcattgtcagag
ggagctattccctatgatattaataccatgctaaatgccataggaggacatcaaggggccttacaagtgctaaag
gaagtaatcaatgatgaagcagcagattgggatagaagtcacccaccagtggtagggccgttgccaccagggcaa
ataagggaaccaacaggaagtgacattgctgggacaactagctcccagcaagagcaagttcactggattactagg
gccaaccaccctatcccagtaggagacatctatagaaaatggatagtgttgggactaaacaaaatggtaaaaatg
tacagtccagtgagcatcttagatattaaacagggaccaaaagaaccatttagagattatgtagatcggttctac
aaaacattaagagctgagcaagcatctcaagatgtaaagaattggatgacagaaaccctgcttgttcagaatgcc
aacccagattgcaaacagattttgaaagcattagggccaggagctaccttagaagaaatgatggtagcctgtcag
ggagtaggaggaccaactcataaggccaaattgctagctgaagcgatggttaccgcccatcaagatctaaaagga
ggatacacatcagtattcatgcaaagaggacaaaatccaatgaggaagggacctataaaatgtttcaattgtgga
aaagagggacatatagcaaaaaattgtcgagcacctagaaagaaaggttgctggaaatgtggacaggaaggtcac
cagatgagagattgcagaaatggaaaacaggca
>#65 AY169816 1254 nt
atgggtgcgagtgcgtctgtgttgacaggaagtaaattggatgcatgggaaagaattaggttaaagccaggatct
aaaaagacatataagctaaaacatttagtatgggcaagcagggagctggacagattcgcatgtaatcctgggcta
ctagaaactgcagagggtaatgagaagctgctacagcagttagagccagctctcaagacagggtcagaaagtctm
cartcactctggaacacaataacagtgctctggtgtgttcacaacagatatcaagttatagacacgcaggaggca
atacaaaagttaaaggaagtcatggggagcaggaartctgcggaggccgctaaagaaaacaaaagcacaaagcag
gagggtcaaaattaccctatagtaacaaatgcgcaggggcaagtaacgcatcagcccatctcccccaggacttta
aatgcatgggtaaaggcagtagaagaaaargcctttaaccctgaaatcattcctatgttcatggcattgtcagag
ggagcaattccctatgatattaatactatgctaaatgccataggaggacatcagggagctttacaagtgctaaag
gaagtaattaatgaggaagcagcagattgggatagaactcacccaccaccggtagggccgttgccaccagggcag
ataagggaaccaacaggaagtgacattgctggaacaactagcacccagcaagagcaaattcaatggattaccagg
cccaatcaacctatcccagtaggagacatctacagaaaatggatagtgttaggactaaacaaaatggtgaaaatg
tacagcccagtgagcatcttagatattaagcagggaccaaaagaaccatttagagactatgtagatcgattctac
aaagtattaagagctgaacaagcatctcaagaagtaaaaaattggatgacagaaaccctgcttgttcaaaattcc
aatccagattgcaaacagattttgaaatcattaggrccaggagctaccttagaagagatgatggtagcctgtcaa
ggagtaggaggaccaactcataaggccagagtactagcagaagcaatggctacagcccagcaagatttgagagga
gggtgcacagcagtattcatgcaaagagggcaaaatcaaggaaggaaaggacctataaaatgtttcaactgtggg
aaagagggacatatagcaaaaaattgtcgagcccctaggagaaagggttgctgg
>#66 KU168282 1254 nt
atgggtgcgagagcgtctgtgttgacagggagtaaattggatgcgtgggaacaaattaggctaaagccaggatct
aaaaagaaatataggctaaaacatttagtatgggcaagcagggagctggaaagattcgcatgtaatcctggtcta
ctagaatctgcagagggtaatgagcaactgttacagcaattagagccagctctcaagacagggtcagagagcctg
caatctctctggaactcagtagcagtgctctggtgcgttcacaacagatttgatgttcaagatacacagcaggca
ataaaaaagttaaaggaagtaatggcgagcaggaagcctgccactgccgctaaggaggaaacaggctcaaggcag
acaagtcaaaattaccctgtagtaacaaatgcgcagggacaaatggtacatcaagccctttcccccaggacttta
aatgcatgggtaaaggcagtagaagaaaaagcctttaaccctgaaattattcctatgtttatggcattatcagaa
ggagctatttcctatgatatcaataccatgatgaatgccataggagatcaccaaggggctttacaagtgctgaag
gaagtaatcaatgaggaagcagcagaatgggatagaactcatccaccagcaatggggccgttaccaccagggcag

ataagggaaccaacaggaagtgacattgctggaacaactagcacacagcaagagcaaattcactggattactaga
gggaataaccctatcccagtaggagatatctatagaaaatggatagtgctaggattaaataaagtggtaaaaatg
tacagtccagtgagcatcttagatattaagcagggaccaaaagaaccattcagagactatgtagatcggttctac
aaaacattaagagctgagcaagctactcaagaagtaaaaaattggatgacagaaaccctgcttgttcagaattcc
aacccagattgtaaacaaattttgaaatcattaggaccaggagctactctagaagaaatgatggtagcctgccaa
ggagtaggagggccatctcacaaggcgagagtactagcagaagcaatggtttctgcccaacaagatctaaaagga
ggatacacagcagtattcatgcaaagagggcaaaatccaaaaagaaaagggcctataaaatgtttcaattgtgga
aaagagggacatatagcaaaaaattgtcgagcacctagaagaaaagggtgctgg
>#67 AY169809 1308 nt
atgggtgcgagagcgtctgtgttgaaagggagcaaattggatgcatgggaacaaattaagttgaagccaggatct
aaaaaagcatataggctaaaacatttagtatgggcaagcagagagctggaaagattcgcatgtaatcctgagcta
ttagaaactgcagagggtaatgagaaactgttacaacagttagagccagctctcaagacagggtcagaaagcctg
cagtcactctggaacacagtagcagtgctctggtgtgttcacaacagatttaaagttgaagatacgcagcaggca
atacaaaagttaaaggaagtaatggggaaaaggaagcctgtagatactgctaaggaagacacaaactcaaggcar
acaggtcaaaattaccctgtagtaccaaatgcacagggacaaatgatacatcaggccctctcccccaggactttg
aatgcatgggtaaaagcagtagaagaaaaagcctttaaccctgaaattattccaatgttcatggcattgtcagag
ggagctgttccctatgatatcaatactatgctaaatgccataggagggcatcaggggggctttacaagtgctgaag
gaagtaatcaatgaggaagcatcagactgggatagaacgcacccaccagcgatggggccgttgccaccagggcaa
ataagggaaccaacaggaagtgacattgctggaacaactagcacccagcaagagcaagttcactggactaccagg
cctaacaaccctatcccagttggagatatttatagaaaatggatagtgttaggattaaacaaaatggtaaaaatg
tacagtccagtgagcatcctagatattaggcagggaccaaaagagccatttagagactatgtagatcggttctac
aaaacattaagagctgagcaagcaactcaagaagtaaaaaattggatgacagaaaccctgcttgttcaaaatgcc
aacccagattgcaaacagattttaaaatcattagggccaggagctaccttagaagaaatgatgatagcctgtcaa
ggagtaggagggccaacacataaggccaaaatactagcagaagcaatggctacagcccagcaagatctaaaagga
ggatacacagcagtgttcatgcagagagggcaaggcccaattaggaaagggcctataaaatgtttcaactgtgga
aaagagggacatatagcaaaaaattgtcgagcacctagaaagaaaggctgctggaaatgtggacaggaaggtcat
caaatgaaagattgcagaaatggaaaacaggca
>#68 KU168283 1254 nt
atgggtgcgagtgcgtctgtgttgaaagggagcaaattggatgcatgggaacaaattaggttaaagccaggatct
aaaaagaaatatagactaaaacatttagtatgggcaagcagggagctggaaagattcgcatgtaatcctgagcta
ctagaaactgcagagggcaatgagaaactattacaacagttagagccagctctcaagacagggtcagacagcctg
cagtcactctggaacacaatagcagtgctctggtgtgttcataacagatataaagtagaagatacacagcaggca
acacaaaagctgaaggaagtaatggggagcagaaagtctgcagcggccgctaaggaagacacaagcgggaggcag
acgggtcaaaattaccctgtagtaccaaatgcacaaggacagatggtgcatcagcccatctcccccaggacttta
aatgcatgggtaaaggcagtagaggaaaaggcctttaaccctgaagtcatccctatgttcatggcattatcagag
ggagctattccctatgatattaatactatgctaaatgccataggaggacatcaaggggccttacaagtgctaaaa
gaagtaatcaatgaggaagcatcagattgggatagaactcatccaccagcgatgggaccgttaccaccagggcag
ataagggaaccaacaggaagtgacattgctggaacaactagcacccagcaagagcaagttcactgggttaccagg
gccaacaaccctatcccagtaggagatatctatagaaaatggatagtgctaggattaaacaaaatggtaaaaatg
tacagcccagtgagcatcttagatattaaacagggaccaaaagaaccatttagagactatgtagatcggttctat
aaaacattaagagctgagcaagcaagtcaagacgtaaaaaattggatgacagaaacattgcttgttcaaaatgcc
aacccagattgcaaacagattttgaaatcattagggcaaggagctaccttagaagagatgatgryagcctgtcag
ggagtgggaggaccaactcataaggccagagtgctagcagaagcaatggctacggcccagcaagatctgaaggga
ggatgcacagcaatatttatgcaaagagggcaaaatccaattaggaaaggacctataaaatgtttcaattgtgga
aaagagggacatctagcaagaaattgtcgagcgcctagaaagaaaggttgctgg
>#69 AY169813 1308 nt
atgggtgcgagagcgtctgtgttgacagggagtaaattagatgattgggaacaaattaggttaaggccaggatct
aaaaagaaatataggctaaaacatttagtatgggcaagcagggagctggaaagattcgcatgtaatcctgagctg
ttagaaactgcagaaggtaatgagaaactgttacaacagttagaaccagctctcaagacagggtcagaagacctg
aagtcactctggaacgcaatagcagtactctggtgcgttcataacagatataaagttggagatacacagcaagca
gtgcaaaaaattaaaagaagtaatgggaaacaggaagtctgcagataccgctaaagaaaccactagctcaaagcag
acaagtcaaaattacccagtagtagcaaacatgcaagggcaaatgatacatcagcccctctcccccaggacttta
aatgcatgggtaaaggcagtagaagaaaaggcctttaacccggaaattatccctatgtttatggcattgtcagaa
ggagctattccctatgacattaatactatgctaaatgccataggaggacaccaggggggctttacaagtgttgaag
gaagtcatcaatgaggaagcagcagattgggatagaactcacccaccggtggtagggccgttaccaccagggcaa

ataagggaaccaacaggaagtgacattgctggaacaactagcacccagcaagagcaagtccactggattaccagg
gctaacgcccctatcccagttggagatatctatagaaaatggatagtgctaggactaaataaaatggtaaaaatg
tacagtccagtgagcatcttagatattaaacagggaccaaaagaaccatttagagattatgtggataggttctac
aaaacattaagagctgaacaagcaacccaggaagtaaaaaattggatgacagaaaccctacttgttcagaatgcc
aatccagattgcaaacagattttgaaatccttagggccaggagctaccttagaagaaatgatggtagcatgtcag
ggagtaggagggccaactcataaggcaaaaatactagcagaagcaatggcctcagtccaacaagatctaaaagga
ggctacacagcagtatttatgcaaaggggacaaaatccaactaggggaagacctataaaatgtttcaattgtgga
aaagatggacatctagcaaaaaattgtcgagcacctagaaaaaaaggttgctggaaatgtggacaggaaggtcac
caaatgaaagattgcagaaatggaaaacaggca
>#70 AY169805 1308 nt
atgggtgcgagtgcgtcagtgttgacaggaagtaaattggatgcatgggaacaaattaggctaaagccaggatct
aaaaagaaatataggctaaaacatttagtatgggcaagcagggagctggacagattcgcatgtaatcctgagcta
ctagaaactgcagagggcaatgagcaactgttacagcaattagagccagctctcaagacagggtcagaaagcctg
cagtcactytggaacacaatagcagtgctctggtgtgttcacaacagatataaaattggagatacgcagcaggca
atacaaaagttaaaggaagtcatggggaacaggaagtctgcgagtgccgctaaggaagacacaagtgcaaagcag
gcaggtcagaattaccctgtagtatcaaatgcgcagggacaaatgatacatcagcccatctcccctaggacttta
aatgcatgggtaaaggcagtagaagaaaaggcctttaaccctgaaatcattcctatgttcatggcattgtcagag
ggagctattccctatgatattaatactatgctaaatgccataggaggacatcaaggggctttacaagtgctaaag
gaagtaatcaatgaggaagcatcggagtgggatagaactcacccaccaccgatagggccgttaccaccagggcag
ataagggacccaacaggaagtgacattgctgggacaactagcacccagcaagagcaagttcactgggttaccagg
aaccccaaccctatcccagtaggagacatctatagaaaatggatagtgttgggactaaacaaaatggtaaaaatg
tacagcccagtaagcatcttagatattagacagggaccaaaagagccatttagagactatgtagatcggttctac
aaaacattaagagctgagcaagcaactcaagaagtaaaaaattggatgacagaaaccctgcttgttcaaaatgcc
aacccagattgcaaacagattttaaaatcattagggccaggagctaccttagaagagatgatggtagcctgtcag
ggagtaggaggaccaactcataaggccaaaatattagcagaagcaatggctgcagcccatcaagatctgaaagga
ggatacacatcagtattcatgcaaagagggcaaaacccaattaggaaagggcctataaaatgtttcaactgtgga
aaagagggacatatagcaaaaaattgtcgagcacctagaaagcgaggttgctggaaatgtggacaggaaggtcat
caaatgaaagattgcagaaatggaaarcaggca
>#71 HQ179987 1287 nt
atgggtgcgagagcgtcagtgttgacaggggggccgattggatgcatgggaaagaattaggcttagaccaggaggt
aagaaaaaatataagctaaaacatgtagtatgggcaagcagagagctggaaagatttgcatgtaatcctgggctt
atggaaacagcggaaggctgcgaaaaattattagagcagttagaaccagctctcagaacaggctcygatggcctg
cagtctctttggaacaccctagtagttytatggtgtgttcacaaaagaatagagataagtgacacacagcaggcc
attacaaaatggaaggaggaaatgcagaaaagaaagaaaacatcagagggcagcactggaacaagtcaaaactat
cctatcgtgcagaatgcccaggggcaaatgacccatatgccgctgtcccccaggacgctgaatgcctgggtgaag
gcagtagaagaaaaagccttcaaccctgaaattatccctatgtttatggccttgtcagaaggggctatacctgat
gacatcaataccatgcttaatgcagtaggcggacatcagggagcgttgcaggtgttgaaagaggtaattaatgaa
gaggctgcagaatgggatagaacacatcccgttccagtaggaccattaccaccagggcagcttagagaaccaaca
ggaggggatattgcaggaaccactagtaccaaacaggaacagataacctggatgacaaggaacaatcctgtacca
gtaggggacatctatagaaartggatagtgttggggctcaataaagtggtaaaaatgtactgccccgttagcatt
ctggacataaagcagggacctaaggaaccatttagagattatgtagayagattctacaaaaccctcagagcagag
caagccagtcaggaagtaaaaagttggatgacagacaccttactagtacaaaatgctaatccagattgyaagcag
attttgaaagctctgggaccaggtgccaccttagaagaaatgatgaatgcctgtcaaggagtaggggggaccaaca
cataaggccagggtcttggcagaagctatggcagcagctaatcaagctagccaagaattaaaaggagggtataca
acagtttttatgcagagtggacagagaaagccagttaagtgctttaactgtggaaaagtaggacacatagcaaag
aactgcaaggcacctagaagaaggggrtgttggaagtgtggacaggaaggtcatcaaatgaaagactgcaaatca
ggaagacaggca
>#72 GU111555 1296 nt
atgggtgcgagagcgtcagtgttgacaggggggccgattggatgcatgggaaagaattaggcttaagccaggaagt
aagaaaaaatatatgctaaaacatatagtatgggcaagcagagagctggaaagatttgcatgtaatcctgagctt
atggaaacagcagaaggctgtgaaaaattattagagcagttagaaccagctctcagaacaggttctgatggcctg
cagtctctttggaacaccctagtagttttatggtgtgttcacagaagagtrgatataagtgacacacagcaggcc
attacaaaatggaaggaggaaatgcagagaagaaagaaagcactagagggcagcactggaactggaacaagtcaa
aactatcctattgtgcagaatgcccaggggcaaatgacccatctgccgctgtctcccaggacgctgaatgcctgg
gtgaaggcagtagaagagaaggccttcaaccctgaaattatccctatgtttatggccttgtcagaagggtctata

cccgatgacattaatactatgctcaatgcagtaggcggacatcagggagcrttrcaggtgttgaaagargtgatt
aatgaagaggctgcagaatgggatagaacacatcctgttccwgtrggaccgttaccaccagggcagcttagagac
ccaacaggaggggatattgcaggaaccactagtaacaagcaggaacagataacctggacaacaaggaacaataat
cctataccagtaggagatatctatagaaagtggatagtgttggggctcaataaagtggtaaaaatgtatagcccc
attagcatcctggacataaagcagggacctaaggaaccatttagagattatgtagatagattctacaaaacyctc
agagcagaacaagctagtcaggaagtaaagagttggatgacagacacattattaatacaaaatgctaatccagat
tgtaagcagattttgaaagctctgggaccaggggccaccttagaagaaatgatgaatgcctgtcaaggagtaggg
ggaccaacacataaggccagggtcttagcagaagctatggcagcagctaatcaagctaaccaagatttaaaagga
gggtatacaactgttttcatgcagagtggacagagawagccagttaagtgctttaactgtggaaaaataggacac
atagcaaagaactgtaaggcacctaggagaaaggatgctggaagtgtggacaggaaggtcatcaaatgaaagac
tgcaaatcaggaagacaggca
>#73 DQ017382 1305 nt
atgggtgcgagagcgtcagtgttaacaggggaaaattagataaatgggaagcaatttatttgagaccaggggga
aagaaaaaatacaaaatgaaacatttagtatgggcaagcagggagctggaaagattcgcttgtaacccaggtctc
atggacacagcgcacggctgtaaccagttactaaaccaattagaaccagctctcaatacagggtcagaaggactg
cgctccttatataayaccatagcagttctttattgtgtccatagtacaataccggtactcaatacacaagaggct
ttggacaagataaaagagaaaaaggaacagcacaagtctgaggcaaaaaatccagaagcaggggcagcggcagca
gctgataacaatatcagtaggaactatcctctagtccagaatgctcaaggacaaatggtacatcatccgctgaca
cccagaactttaaatgcttgggtaaaagtaatagaggagaaggcctttaatccagaagtaataccaatgtttatg
gccttgtcagaaggggcaacaccctcagatctaaattctatgttaaatacagtaggaggacatcaggcagctatg
caaatgctaaaggaagtcatcaatgaggaagcagcagaatgggacaggacgcatccagtccctgtgggaccacta
cccccagggcaactgagagaacctagaggragtgatatagcagggacaactagtactctgccagaacagataaca
tggatgacttctaatcctcctattccagtaggagatatttatagaagatggatagttttggggttaaacagaatt
gtgagaatgtatagtcctgtcagcattttagagatcaagcaaggaccaaaagaaccctttagagattatgtagac
aggttctacaaaactttaagagcagagcaggcaacacaggatgtaaagaattggatgacagaaacactcttagta
caaaatgcaaatccagattgtaarcagatcctaaaagcattaggaccatcagctagcttagaagagatgatgacg
gcctgccaaggagtgggggaccagggcataaggcaagagtgctagcagaggctatggcacaggcacaggcagca
actagtgtctttgtacaaagaggaaactttaaaggcacaaggagaaccattaaatgtttcaactgtggcaaagag
ggccatttggcaaggaactgtaaggctcctagaagarggggctgttggaagtgtgggcaagaaggacatcaaatg
aaagattgtaaaaatgaaggaagacaggct
>#74 AY532635 1314 nt
atgggtgcgagagcgtcagtgttaacaggggaaaattagatcaatgggaagcaatttatttgagaccaggggga
aagaaaaaatacagaatgaaacatttagtatgggcaagcagggagctggaaagattcgcttgtaacccaggtctc
atggacacagcgagcggctgtgcccagttactaaaccaattagaaccagctctcgcaacagggtcagaaggactg
cgctccttatataacgccctagcagttctttattgtgtccatagtaacatacaggtacacaatacacaagaggcg
ttggacaagataaaagagataaaagagaaaaaggaacggcacaagtccgagccaaaaaacccagaagcaggggca
gcggcagcagctgataacaatatcagtaggaattatcctctagtccagaatgctcaaggacaaatgatacatcag
ccgctgacacccagaactttaaatgcttgggtaaaagtgatagaggagaaggcctttaatccagaagtaatacca
atgtttatggccttgtcagaaggggcaacacccgcagatctaaatactatgttaaatacagtaggaggacatcag
gcagctatgcagatgctaaaggaagtcatcaatgaggaagcagcagaatgggacaggacgcatccartccctgyg
ggaccattacccccaggacaactgagagaacctagaggaagtgatatagcagggacaactagtactctgmtggaa
caggtgacctggatgacttctaatcctcctgttccagtaggagaaatctataaaaaatggatagttctggggtta
aatagaattgtgagaatgtatagtcctgtcagcattctagagatcaaacaaggaccaaaagaaccctttagagat
tatgtagacaggttctacaaaactttaagagcagagcaggcatcacaggatgtaaagaattggatgacagaaaca
ctcttagtacaaaatgcaaatccagattgtaaacagatactaaaagcattagggccagcagctaccttagaagaa
atgatgacggcctgtcagggagtgggggaccagcgcataaggcaagagtgctagcagaggctatgtcacaggta
cagacagcaactagtgtctttgcacaagggagaaactttaaaggcataaggagaaccattagatgtttcaattgt
ggcaaagagggccatttggcaagaaactgtaaggcccctagaagagggggctgttggaaatgtgggcaagaagga
catcaaatgaaagattgtaaaaatgaaggaagacaggct
>#75 AJ006022 1302 nt
atgggtgcgagagcgtcagtgttaacaggggaaaattagatcaatgggaatcaatttatttgagaccaggggga
aagaaaaaatacagaatgaaacatttagtatgggcaagcagggagctggaaagattcgcttgtaacccaggtctc
atggacacagcggacggctgtgccaagttactaaatcaattagaaccagctctcaagacagggtcagaagaactg
cgctctttatataacgctctagcagttctttattgtgtccatagtaggatacagatacacaacacacaggaagct
ttggacaagataaaagagaaacaggaacagcacaagcccgagccaaaaaacccagaagcaggggcagcggcagca

actgatagcaatatcagtaggaattatcctctagtccagactgctcaaggacaaatggtacatcagccgctgaca
cccagaaccttaaatgcttgggtgaaagtgatagaggagaaggcctttagtccagaagtaataccaatgtttatg
gccttgtcagaaggggcaacgccctcagatctaaatactatgttaaatacagtaggggacatcaggcagcaatg
cagatgctgaaggaagtcatcaatgaggaagcagcagactgggataggacacatccagtccctgtgggaccacta
cccccagggcaactgagagaccctagaggaagtgatatagcaggaacaactagcaccctggcagaacaggtggct
tggatgactgctaatcctcctgttccagtaggagatatttatagaagatggatagtcctggggttaaacagaatt
gtgagaatgtatagtcctgtcagcattctagagatcaaacaaggaccaaaagaacccttcagagactatgtagac
aggttctacaaaactctaagagcagagcaggcaacacaggaagtaaagaattggatgacagaaacactcttagta
caaaatgcaaacccagattgtaaacagctcctaaaagcattagggccaggagctaccttagaagagatgatgacg
gcctgccagggagtgggggaccagcacataaggcaagagtgctagcagaggctatgtcacaggtgcagcagcca
acaactagtgtctttgcacaaaggggaaactttaaaggcataaggaaacccattaaatgtttcaattgtggcaaa
gagggccatttggcaagaaactgtaaggcccctagaagaggaggctgttggaagtgtgggcaagaaggacatcaa
atgaaagattgtaaaaatgaaggaaga
>#76 MF7672 1305 nt
atgggtgcgaaagcgtcagtgttaacaggggaaaattaaataaatgggaagcaatttatttgaaaccaggggga
aaaaaaaatataaaaggaaacatttagtatgggcaagcagggagctggaaagattcgcttgtaacccaggtctc
agggacacagcgagtggctgtgcccagttaataaaccagttaaaaccacctctcaagacagggtcaaaaggactg
cgctctttatataacaccctggcagttctttattgtgtccatagtaacatatcggtacacaatacacaagaggct
ttggacaagataaaagagaaacaggaacagcacaagtctgagccaaagcaaccagaagcaggggcagcagcggca
gctgataacaatgtcagtaggaactatcctctaatccagaatgctcaaggacaaatggtacatcaggcgctgtca
cctagaactttaaatgcttgggtaaaagtaatagaggaaaaggcctttaatccagaagtaataccaatgtttatg
gccttgtcagaaggggcaacgccctcagatctaaattctatgttaaatacagtaggggacatcaggcggctatg
cagatgctaaaggaagtcatcaatgaggaagcagcagaatgggacaggacgcatccagcccctgtggggccacta
cccccaggacaattgagagaccctagaggaagtgatatagcagggacaactagtactctggcagaacaggtggca
tggatgactgctaaccctcctattccagtaggagatatttatagaagatggatagttctggggttaaacagaatt
gtgagaatgtatagtcctgtcagcattctagaratcaagcaaggaccaaaagaaccctttagagattatgtagac
agattctacaaaactttaagagcagagcaggcaacacaggatgtaaagaattggatgacagaaacactcttagtr
caaaatgcaaayccagattgtaaacagatyctaaaagcattagggccaggagctaccttagaagagatgatgacg
gcctgccagggagtgggggaccagcacacaaggcaagggtcctagcagaggctatgtcacaggcacaggcagca
actagtgtctttgcacaaggaggaaactttaaaggcacaagaaaaaccattaaatgttttaattgtggcaaagag
ggccatttggcaagaaactgtaaggcccctagaagaggagggtgttggaagtgtgggaaagaaggacatcaaatg
aaagattgcaaaagtgaaggaagacaggct
>#77 KY498771 1302 nt
atgggtgcgagagcgtcagtgttaacaggggaaaattagatcaatgggaatcaacttacttgagaccaggggga
aagaaaaaataccaaatgaaacatttagtatgggcaagcagggagctggacagattcgcttgtaacccaggtctc
atggactcagcggaaggctgtgcccagttaatgaaccaattagaaccagctctcaagacagggtcagaaggactg
cgctctttatataacaccctagcagttctttattgtgtccatcgtagaatatcagtacaaaatacacaagaagct
ttggacaaagtacatggggaaaaggaacagcacaacaagccaaaaaactcagaagcaggggcagcggcagcagtt
gacagcagtatcagtagaaattatcctctagtccagactgctcaaggacaaatgatacatcagccgctgacaccc
agaaccttaaatgcttgggtaaaagtgatagaggagaaggcctttagtccagaagtaataccaatgtttatggcc
ttgtcagaaggggcaacacccggagatctaaatactatgttaaatacagtaggaggacatcaggcagctatgcag
atgctgaaggaagtcatcaatgaggaagcagcagaatgggacaggctgcatccagtccctgtgggaccactgccc
ccaggacaactgagagaccctagaggaagtgatatagcaggaacaactagtactctgcaagaacaggtggcatgg
atgactgctaatcctcctgtcccagtaggagatatttatagaagatggatagtcctggggttaaacagaattgtg
agaatgtatagtcctgtcagcattctagagatcaaacaaggaccaaaagaaccctttagagactatgtagacagg
ttctacaaaactttaagagcagagcaggcaacacaggaagtaaagcagtggatgacagaaacactcttagtacaa
aatgcaaacccagattgtaaacagctcctaaaagcattagggccaggagctaccttagaagagatgatgacrgcc
tgccagggagtgggggaccagggcataaggcaagagtactagcagaggctatgtcacagatacagccaacaact
agtatctttgcacaaagaggaaactttaaaggcataaggagaaccattaaatgtttcaattgtggcaaagagggc
catttggcaagaaactgtaaggcccctagaagaaagggctgttggaaatgtgggaaagaaggacatcaaatgaaa
gattgcaacaatgaagggagacaggct
>#78 DQ017383 1305 nt
atgggtgcgagagcgtcagtgttaacaggggaaaattagataaatgggaagcaatttatttgagaccaggggga
aagaaaaaatatagaatgaaacatttagtatgggcaagcagggagctggaaagattcgcttgtaacccaggtctc
atggacacagcgaagggctgtgcccagttactaaaccagttagaaccagctctcaatacagggtcagaaggactg

cgctccttatataacaccttagcagttctttattgtgtccatagtacaataccggtacttaatacacaagaggct
ttggacaaaataaaagagaaaaaggaacagcacaagcctgaggcaaaaaatccagaagcaggggcagcggcagca
gctgataacaacatcagtaggaattatcctctagttcagaatgctcaaggacaaatgatacatcagccgctgaca
cccagaactttaaatgcttgggtaaaagtgatagaggagaaggcctttaatccagaagtaataccaatgtttatg
gccttgtcagaaggggcaacaccctcagatctaaattctatgttaaatacagtaggaggacatcaggcagctatg
caaatgctaaaggaggtcatcaatgaggaagcagcagaatgggacaggacgcatccagtccctgtgggaccacta
cccccagggcaactgagagagcctagaggcagtgatatagcagggacaactagtactctgccagaacagataaca
tggatgacttctaaccctcctattccagtaggagatatttatagaagatggatagttttggggttaaacagaatt
gtgagaatgtatagtcctgtcagcattctagagatcgagcaaggaccaaaagaaccctttagagattatgtagac
aggttctacaaagttttaagagcagagcaggcaacacaggatgtaaagaattggatgacagaaacactcttagta
caaaatgcaaatccagattgtaaacagatcctaaaagcattagggccaggatcctccttagaagagatgatgacg
gcctgccagggagtgggggaccagggcataaggcaagagtgctagcagaagctacggcacaggcacaggcagca
accagtgtctttgtacaaaggggaaactttaaaggcacaaggagaaccattaaatgtttcaattgtggcaaagag
ggccatttggcaaggaactgtaaggcccctaggagaggaggctgttggaaatgtgggcaggaaggacatcaaatg
aaagattgtaaaaatgaaggaagacaggct
>#79 AJ271370 1308 nt
atgggtgcgagagcgtcagtgttaacaggggaaaattagatcaatgggaagcaatttatttgaggccaggggga
aagaaaaaatacagattgaaacatttagtatgggcaagcagggagctggaaagattcgcttgtaacccaggtctc
atggacacagcgaatggctgtgcccagttaataaaccaattagaaccagctctcaagacagggtcagaaggactg
cgctctttgtwtaacaccctggcagttctttattgtgtccatagtaacataccggtacacaatacacaagaggct
ttagacaagataaaagagaaacaggaacagcacaagtccgagccaaagaaaccagaagcagggacagcggcggca
gctgacagcagtatcagtaggaattatcctctagtccagaatgctcaaggacaaatggtacatcagccgctgaca
cctagaactttaaatgcttgggtaaaagtaatagaggaaaaggcctttaatccagaaataataccaatgtttatg
gccttgtcagaaggggcaacgccctcagatctaaatagtatgttaaatacagtaggaggacatcaggcagctatg
cagatgctaaaggaagtcatcaatgaggaagcggcggagtgggacaggacgcatccagcccctgtgggaccacta
cccccagggcaaatgagagaccctagaggaagtgatatagcagggacaactagtactctggcagaacaggtggca
tggatgacttctaatcctcctattccagtaggagatatttatagaagatggatagttctggggttaaacagaatt
gtgagaatgtatagtcctgtcagcattctagagatcaagcaaggaccaaaagagccctttagagattatgtagac
aggttctacaaaactttaagagcagagcaggcaacacaggatgtaaagaattggatgacagaaacactcttagta
caaaatgcaaacccagattgtaaacagatcctaaaagcattaggaccaggagctaccttagaagagatgatgacg
gcctgccagggagtaggggaccagcacataaggcaagagtgctagcagaggctatggcacaggcacaaacagca
actagtgtctttgtacaaaggggaaactttaagggcataagaaaaaccattaaatgttttaattgtggcaaagag
ggccatttggcaagaaactgtaaggcccctagaagaaggggctgttggaagtgtgggcaagaaggacatcaaatg
aaagattgtaaaaatgagggaagmcaggctaat
>#80 DQ314732 1287 nt
atgggtgcgagagcgtcagtattaagtggggaaaattagatgcatgggaaaaaattcggttacggccaggggga
aggaaaaaatataagatgaaacatctaatatgggcaagcagagagttggaaagatttgcaattaaccctggcctt
ttagaaacagcagagggatgtcaacaaataatagaacagttacagtcaactctcaagacaggatcagaagaactt
aaatcattatataatacagtagcaaccctctggtgcgtgcaccagaggatagaggtaaaagacaccaaagaagct
ttagacaaaatagaggaagcacaaaataagagtcagcaaaagaaacagcaagcagctgctggcacaggaagcagc
agcaaagtcagccaaaattaccctatagtgcaaaacgcacaagggcaattggtgcatcagcctttatcacctaga
actttgaatgcatgggtgaaagtagtagaagaaaagggttttaacccagaggtaatacccatgttctcagcatta
tcagagggagccgccccacaagatttaaatatgatgctaaatatagtgggggacaccaggcagcaatgcaaatg
ttaaaagaaaccatcaatgaggaagctgcagaatgggatagggtacacccagtacatgcagggcctattccacca
ggccagatgagggaaccaaggggaagtgacatagcaggaactactagtacccttcaagaacaaataggatggatg
accaacaatccaccgatcccggtgggagacatctataaaagatggataatcctgggattaaataagatagtaaga
atgtatagccctactagcattttggacataagacaagggccaaaagagcccttcagagactatatagataggttc
tataaaactctcagagcagaacaagctacacaggaggtgaaaaactggatgacagaaaccttgctagtccaaaat
gcgaatccagactgtaggtccattttaaaagcattaggaagaggcgctacattagaagaaatgatgacagcatgc
cagggagtgggaggacctagccataaagcaagggttttggccgaggcaatgagccatgtacaacaagcaaatata
atgatgcagagaggcaattttaagggccagaaaagaattaagtgcttcaactgtggcaaagaaggacacctagcc
agaaattgcagggcccctagaaaaaagggttgttggaaatgtgggaaggaaggacatcaaataaaagactgcact
gagagacaggct
>#81 AF377956 1272 nt

gcaagagcgtcactattaagcgggggaaaattagatgatttggaaaaaattcggttaaggccagggggggaagaaa
aaatataggctgaaacatatagtatgggcaagcagggagctagaaagatttgcacttaatcctggccttttagag
acaaaggaaggctgtaaacaaataataggacaactacaaccatcccttcagacaggatcagaagagcttaaatca
ttattcaacacaatagtagtcctctattatgtacatcaaaggataaaaataggagacaccaaggaagctttagat
aagctacaggaagaacaagacaaaagtcagcaaaaaacacaaccagcagcggctgacaaaggggtcagtcaaaat
taccctatagtacagaatcttcagggacaaatggtacaccagtctctatcacctagaactttaaatgcatgggta
aaagtaatagaagagaaggctttcagcccagaagtaatacccatgttttcagcattatcagaaggagccacccca
caagatttaaacaccatgctaaacacagtgggggggacatcaagcagccatgcaaatgttaaaagataccatcaat
gaggaagctgcagaatgggacagattacatccagtgcaggcaggacccatcccaccaggtcagataagagaacct
aggggaagtgatatagcaggaactactagcaacctacaggaacaaatagcatggatgacaagcaacccacctgtc
ccagtaggagaaatctataaaagatggataatcctaggattaaataaaatagtaagaatgtatagccctgtcagc
attttggacataaaacaagggccaaaagaaccctttagagactatgtagacaggttctttaaaactctaagagct
gagcaagcttcacaggaagtaaaaggctggatgacagacaccttgttggtccaaaatgcgaacccagattgtaag
atcattttaaaaggattaggaacaggggctacactagaagaaatgatgacagcatgtcaggggggtgggggacct
ggccataaggcaagaattttggctgaggcaatgagccaagtaacatctacatccatattgatgcagaaaagcaac
tttaagggccaaagaagaaatgttaagtgtttcaactgtggcaaagaaggacatatagctaaaaattgcagggcc
cctagaaaaagggctgttggaaatgtggaaaggaaggacaccaaatgaaagactgcactgagagacaggct
>#82 AB254156 1284 nt
atgggtgcgagagcgtcaatattaagaggcggaaaattagatgcatgggaaaaaattaagttaaggccaggagga
aagaaacattatatgctaaaacacttagtatgggcaagcagggagctggaaagatttgcacttaaccctggcctt
ttagagacagcagaaggctgtaaacaaataatacaacagctaactccagctcttaagacaggaacagaggaactt
agatcattatttaacacagtagcaactctctattgtgtacataaaaatatagaggtacgagacaccaaggaagcc
ttagacaaaatagaggaagaacaaaagaaatgtcagcaaaaaccacagcaagcagaagcagctgacaaaggaaaa
gtcagtcaaaattaccctatagtgcagaatctccaagggcaaatggtacaccaaagcctatcaccgagaacttta
aatgcatgggtaaaagtaatagaggagaaggctttcagcccagaggtgatacccatgtttacagcattgtcagaa
ggagccaccccacaagatttaaacaccatgttaaatacagtgggggggacatcaggcagccatgcaaatgttaaag
gataccatcaatgaggaggctgcagaatgggatagggtacatccagtgcatgcagggccagttgcaccaggccaa
atgagagaaccgaggggaagtgacatagcaggaaccactagtacccttcaagaacagatagcatggatgacaagt
aacccacctattccagtaggagacatctataaaagatggataattctggggttaaataaaatagtaagaatgtat
agccctgtcagcattttggacataaaacaagggccaaaggaaccctttagagactatgtagaccggttctttaaa
actttaagagctgaacaatctacacaagaagtaaaaaattggatgacagacaccttgttggtccaaaatgcgaac
ccagattgcaagaccattttaagagcattaggaccaggggcttcattagaagaaatgatgacagcatgtcaggga
gtgggaggacctagccacaaagcaagagtgttggctgaggcaatgagccaagcaggcaatgcaaacataatgatg
cagagaggcaattttaagggccctagaaaaattgttaaatgtttcaactgtggcaaggaagggcacatagccaga
aattgcagggcccctaggaaaagggctgttggaaatgtggaaaggaaggacaccaaatgaaagactgtactgaa
agacaggct
>#83 MF373132 1287 nt
atgggtgcgagagcgtcaatatttagaaggacggaattggatacttggaaaaaaattaagttaaagccaggggga
aagaaaaagtatatgatgaaacatctagtatgggcaagcagagagctggaaagatttgcacttgaccctggcctt
ttagaaacatcagaaggctgtaaacgaataatacaacagctacagccatctcttcagacaggaacaaaagaactc
atatccttgtataatacagtagtagttctatattgtgtacataacaatatagcggtaaatgacactaaggaagcc
ttagacaaagtaaaggaagaacaaaacaaaagtcagcaaaaaacacagcaggcagcaacggctggcaacaaacag
gtcagtcaaaattatcctatagtgcagaaccttcaagggcaaatggtacatcaaaacatatcaccaagaaccttg
aatgcatgggtgaaagtaatagaggagaaggctttcagcccagaggtaatacccatgtttacagcattatcagaa
ggagccactccacaagatttaaacaccatgttaaatacagtgggggggacatcaagcagccatgcaaatgttaaaa
gacaccatcaatgaggaggctgcagaatgggataggttacatccagtgcaagcagggcctgtcgcaccaggccaa
ataagagatccaaggggaagtgacatagcaggaactactagtacccttcaggaacaaataacatggatgacaagt
aacccacctatcccagtaggagacatctataaaagatggataattctagggttaaataaaatagtaagaatgtat
agccctgttagcattttggacataaaacaagggccaaaggaaccctttagagactatgtagaccggttctttaaa
accttaagagctgaacaagctacacaagatgtgaaaaattggatgacagacaccttgttggtccaaaatgcgaac
ccagactgtaagaccattttaagggcattagggccaggagctacattagaagaaatgatgacagcatgtcaggga
gtgggaggacctggccacaaagcaagagtattggctgaggcaatgagccaagcaaacaatgtaaacataatgatg
cagagaaacaattttaagggcccaaaaagaattattaaatgtttcaactgtggcaaggagggcacatagccaga
aattgcagggcccctaggaaaaagggctgttggaaatgtggaaaggaaggacaccaaatgaaagactgtactaat
gaaaggcaggct

>#84 KP109483 1278 nt
atgggtgcgagagcgtcaatattaagagggggaaaattagataaatgggaaaaaattaggttaaggccaggggga
aagaaacattatatgttaaaacacttagtatgggcaagcagggagctggaaagatttgcacttaaccctggcctt
ttagagacagcagaaggctgtaaacaaataataaagcagctacacccagctctccagacaggaacagaggaactt
aaatcattattcaacacagtagcaactctctattgtgtacatgcagggatagaggtacgggacaccaaggatgcc
ttagacaaaatagaggaagaacaaaacaaaagtcagcaaaaaacacagcaggcaaaagaggctggcgggaaggtc
agtcaaaattatcctatagtgcagaatctccaagggcaaatggtacaccaggccatatcacctagaactttgaat
gcatgggtaaaagtagtagaggagaaggcttttagcccggaggtaatacccatgtttacagcattatcagaagga
gccaccccacaagatttaaacaccatgttaaatacagtgggggacatcaagcagccatgcagatgttaaaagat
accatcaatgaagaggctgcagaatgggatagaacacatccaatacatgcagggcctattgcaccaggccaaatg
agagaaccaaggggaagtgacatagcaggaactactagtacccttcaggaacaaatagcatggatgacaagtaac
ccacctgttccagtgggagacatctataaaagatggataattctggggttaaataaaatagtaagaatgtatagc
cctgttagcattttggacataaaacaagggccaaaggaaccctttagggactatgtagatcggttctttaaaact
ctaagagctgaacaagctacacaagatgtaaaaaattggatgacagacaccttgttgatccaaaatgcgaatcca
gattgtaagaccattttaagagcattaggaccaggggcttcaatagaagagatgatgacagcatgtcaaggagtg
ggaggacctagccacaaagcaagagtgttggctgaggcaatgagccaaacaaacaatgccataatgatgcaaaga
agcaattttaaaggctctaaaagaactattaaatgtttcaactgtggcaaggaagggcacttagccagaaattgc
agggcccctaggaaaaagggctgttggaaatgtggaaaggaaggacaccaaatgaaagactgtactgaaagacag
gct
>#85 KY658708 1281 nt
atgggtgcgagagcgtcaatattaagaggcgaaaaattagataaatgggaaaaaattaggttaaggccaggggga
aagaaacattatatgctaaaacatctagtatgggcaagcagggagctggaaggatttgcacttaaccctggcctt
ttagaaacagcagaaggctgtaaacaaataatgaaacagctacaaccagctcttcaaacaggaacagaggaactt
aggtcattatacaacacagtagcaactctctattgtgtacatgcagggatagaggtaagagacaccaaggaagcc
ttagacaagatagaggaagaacaaaacaaaagtcagcaaaaaacacagcaggcaaaagaggctgatgggaaggtc
agtcaaaattatcctatagtacagaatctacaagggcaaatggtacaccaggccatatcaccwagaactttgaat
gcatgggtaaaagtaatagaggagaaggcctttagcccagaagtaatacccatgtttacagcattatcagaagga
gccaccccacaagatctaaacaccatgttaaatacagtgggggacatcaagcagccatgcaaatgttaaaagat
accatcaatgaggaggctgcagaatgggatagattacatccagtacatgcagggcctgttgcaccaggccaaatg
agagacccaaggggaagtgacatagcaggaactactagtacccttcaggaacaaatagcatggatgacaagtaac
ccacctgttccagtgggagacatctataaaagatggataattctggggttaaataaaatagtaagaatgtatagt
cctgtcagcattttggacatcagacaagggccaaaggaaccctttagagactatgtagatcggttctttaaaact
ttaagagctgaacaagctacacaagatgtaaagaattggatgacagaaaccttgttggtccaaaatgcaaaccca
gattgtaagaccattttaagagcattaggaccaggggctacattagaagaaatgatgacagcatgtcagggagtg
ggaggacctggccacaaagcaagagtgttggctgaggcaatgagccaggcaaacagtgtaaacataatgatgcag
aaaagcaattttaaaggccctagaagaattgttaaatgtttcaactgtggcaaggaagggcatatagccaaaaat
tgcagggcccctaggaaaaaaggctgttggaaatgtggaaaagaaggacaccaaatgaaagactgtactgagaga
caggct
>#86 KC156114 1284 nt
atgggtgcgagagcgtcagtattgaaaggcggaaaattagatgcatgggaaaaaattaggttaaggccaggggga
aagaaaaaatataggctaaaacacttagtatgggcaagcagggagctggaaaaatttgcacttaaccctggcctt
ttagaaacatcagaaggctgtaagcaaatactgacacagctacaaccagctcttcaaacaggaacagaggaactt
aaatcattattcaacacagtagcaactctctattgtgtacataaaggatagaggtacatgacaccaaggaagcc
ttagacaaggtagaggaagaacaaaacaaaagtcagcaaaaaacacagcaggcagaagcggctgacaaaggaaaa
gtcagtcagaattatcctatagtgcagaatctccaagggcaaatggtacaccaggccttatcaccaagaactttg
aatgcatgggtaaaagtaatagaggagaaagctttcagcccagaggtgatacccatgtttacagcattatcagaa
ggagccaccccacaagatttaaacaccatgctaaatacagtgggggacatcaagcagccatgcagatgttaaaa
gataccatcaatgaggaggctgcagaatgggataggatgcatccagtgcatgcagggcctgctgcaccaggccag
atgagagagccaaggggaagtgacatagcaggaaccactagtaaccttcaggaacaaatagcatggatgacaagt
aacccacctattccagtgggagacatctataaaagatggataattctggggttaaataaaatagtaagaatgtac
agccccgtcagcattttggacataaaacaaggaccaaaagaaccctttagagattatgtagatcggttctttaaa
accttaagagctgaacaatcctcacaagaggtaaaaaattggatgacagacaccttgttgatccaaaatgcgaac
ccagattgtaaaaccattttaaaagcattaggatcaggggcttcattagaagaaatgatgacagcatgtcaggga
gtgggaggacctggccacaaagcaagagttttggctgaggcaatgagccaagtaaacaataccagtataatgatg
cagaaaagcaactttaaaggctctagaaaaattgttaaatgtttcaactgtggcaaggaagggcatatagccaga

aattgcagggcccccaggaaaagaggctgttggaaatgtggaaaggaaggacaccaaatgaaagactgtactgag
aggcaggct
>#87 AF005496 1296 nt
atgggtgcgagagcgtcagtattaagcggcggaaaattagatgcttgggagaaaattcggctaaggccaggggga
aagaaaaaatataggctaaaacatctagtatgggcaagcagggagctggaaagatttgcacttaaccccggcctt
ttagaaacaccagaaggctgtctacagataatagaacagatacagccagctattaagacaggaacagaagaactt
aaatcattatttaatctagtagcagtcctctattgcgtacatcgaaaaatagatgtgaaagacaccaaggaggct
ttagataagatagaggaaatacaaaacaaaagtcagcaaaaaacacagcaagcagcagctgataaggaaaaagac
aacaaggtcagtcaaaattatcctatagtacagaatgctcaagggcagatggtacaccaggccatatcacctagg
accttaaatgcatgggtaaaagtagtagaagaaaaggcttttagcccagaagtaatacccatgttttcagcatta
tcagaaggagccaccccacaagacttaaatgctatgctaaatacagtgggggggacatcaagcagccatgcagatg
ttaaaagatacaatcaatgaggaagctgcagaatgggacagggtacatccagtgcatgcagggcctattccacca
ggccaaatgagagaaccaaggggaagcgatatagcaggaactactagtaccctgcaggaacaaatagcatggatg
acaggcaatccagctatcccagtgggagacatctataaaagatggataatcctgggattaaataagatagtaaga
atgtatagtcctgtcagcattctggacataaaacaagggccaaaagaaccctttagagactatgtagacaggttt
tttaaaactttaagagctgagcaagccacacaggatgtgaagaattggatgacagaaaccttgttggtccaaaat
gcaaatccagattgcaagactatattaagagcattaggacaaggggcttcaatagaagaaatgatgacagcatgt
cagggagtgggaggacctagtcataaagcaagagttttggctgaggcaatgagccaagtaacaaatacaaataca
gccataatgatgcagaaaggcaactttaagggccaaagaaaatttgttaaatgcttcaactgtggcaaagaggga
cacatagccagaaattgcagggcccctaggaaaaagggctgttggaaatgtggaagagaaggacatcagatgaaa
gactgcacagagagacaggct
>#88 FJ389367 1293 nt
atgggtgcgagagcgtcagtattaagtgggggaaaattagatgcatgggaaaaaattcggttgaggccaggggga
aagaaaaaatatagaataaaacatctagtatgggcaagcagggagctggaaagatttgcactcaaccctggcctt
ttagaaacaacagaaggttgtcaacaaatattggaacagttgcaaccaactcttaggacaggaacagaggagatt
aaatcattatataatgcartagcaactctctattgtgtacatcaaaggatagaggtaaaagacaccaaagaagct
ctagaagaagtggaaaagatacaaaagaaaagtcagaaaaagacacagcaggcagcaatgggtgaaggaaacagc
agccaagtcagccaaaattatcctatagtgcaaaatgcacaagggcaaatggtacatcagcccctatcacctaga
actttaaatgcatgggtaaaagtagtagaagagaaggcattcaatccagaagtaatacccatgttttcagcacta
tcagaaggagccaccccacaagatttaaataccatgctaaacacagtgggggacatcaagcagctatgcaaatg
ctaaaagatactattaatgaggaagctgcagagtgggacaggacacatccaccacaggcagggcctatcccacca
ggccagataagggaaccaaggggaagtgatatagcaggaactactagtaacctgcaggaacaaataagatggatg
accagcaatccacctatcccagtgggagaaatytataaaagatggataatcctgggattaaataagatagtaaga
atgtatagccctgtcagcattttggatataagacaagggccaaaagarcctttagagattatgtagataggttc
tttaaaactttaagagctgaacaagctacacaggaagtaaaaaactggatgacagacaccttgttggtccaacat
gcaaacccagattgtaagaccatcttaagagcattagggccaggagctacactagaagaaatgatgacagcatgt
cagggagtgggaggacccggccataaagcaagggttttagctgaagcaatgagccaggcatcaaattcagcagca
gctataatgatgcaaaaaggcaattttaagggcccaagaagaattaagtgtttcaactgtggcaaggaaggacat
ctagccagaaattgcagggcccctaggaaaaagggctgttggaaatgtggaaaagagggacatcaaatgaaagac
tgcacagaaagacaggct
>#89 FJ389365 1242 nt
attcggctgaggccmgggggaargaaammrtayaractaaaacatwtagtatgggcaagcagggagctggayaga
tttgcacttaaccccggcctcttagaaacaggagaaggwtgtcagcagatamtgrmamagttgsaaccakctctc
magacaggaacagaggarattaaatcattatataatacagtagcaacyctctattgtgtacatcacaggatagar
ataaragacaccaaagaagctctagatgtartrgaaaaamtacaaaagaamagtcagcaagaaacacagcaggca
gcaaggagtacaggaaataacagccaagttagccaaaactatcctatagtgcagaatgcacaagggcaaatggta
caycaggccatatcaccyaggactttaaatgcatgggtaaaagtaatagaagaaaaggccttcagcccagaagta
atacccatgtttacagcattatcagaaggagccaccccacaagatttgaataccatgctaaacaccgtggggggg
catcaagcagctatgcagatgctaaaggatactattaatgaagaagctgcagagtgggataggatgcatccacca
caggcagggcctcttccaccaggccagmtaagagaacccaggggaagtgatatagcaggaactaccagtaccctg
caggaacaaataacatggatgaccagcaacccacctatyccagtgggagaactatataaaagatggataatcctg
ggttaaataaaatagtaagaatgtatagccctgtcagcattttggacataaaacaagggccaaaagaaccyttc
agagattatgtagatagrtttttttaaaactttgagagctgarcaagccacccaggatgtaaaaaattggatgaca
gacaccttgttggtccaaaatgcgaatccagattgtaaaaccatcttaagagcattaggaccaggagctacatta
gaagaaatgatgacagcatgtcaaggagtgggaggacccagccacaaagcaagagtgttagctgaggcaatgagc

caggcaacargtgcagcagcaaacrtaatgatgcagaaaagcaattttaagggcccgagaagaaatattaagtgt
ttcaactgtggcagggaaggacatctagccagaaattgcagggcccctaggaaaaagggctgttggaaatgtgga
caggagggacatcaaatgaaagactgcacagaragacaggct
>#90 MH705151 1284 nt
atgggtgcgagagcgtcagtattaagcgggggaaaattagatgcatgggagaaaattcggttaaggccagggggga
aagaaacattataaactgaaacatctagtatgggcaagcagggagctggaaagattcgcagttaaccctggcctt
ttagactcagcagaaggctgtcaacaaataatggaacagttacaaccagctctcaagacaggatcagaagaactt
acatcattatttaatacagtagcaaccctctattgtgtacatcaaaggatagatgtaagagacaccaaggaagct
ttagataaaatagaagaaataaagagcaagcaaaagacacagcaggtagcagctgacacaggaaacagcaacaag
gtcagtcaaaattaccccataatacaaaatacacaagggcaaatggtatatcagagcttatcacctaggactttg
aatgcatgggtaaaagcaatagaagaaaaggctttcagcccagaagtaatacccatgttcacagcattatcagag
ggagccgccccacaagatttaaatatgatgctgaacatagtgggggacaccaggcagctatgcaaatgttaaaa
gataccattaatgaagaagctgcagaatgggatagggtacatccagtacatgcagggcctattcaaccaggccag
atgagagaaccaaggggaagtgacatagcaggaactactagtacccttcaagaacaaataggatggatgacaagc
aatccacctatcccagtgggagaaatctataaaagatggataatcctgggattaaataaaatagtaagaatgtat
agccctgttagcattttggacataagacaagggccaaaagaacccttcagagactatgtagataggttctttaaa
actctcagagctgagcaagctacacaagaggtaaaaggttggatgacagaaaccttgctggtccaaaatgcgaat
ccagactgtaagaccattttaagagcattaggagcaggggctacattagaagaaatgatgacagcatgtcaggga
gtgggaggacccggccataaagcaagggttttggctgaggcaatgagtcaagtacaacatacaaacataatgatg
cagagaggcaatttaggggccagaaaagaatgattaaatgtttcaactgtggcaaagaaggacacctagccaga
aattgcagggcccctaggaaaaagggctgttggaaatgtgggaaggaaggacatcaaatgaaagaatgcacagaa
agacaggct
>#91 KF716478 1290 nt
atgggtgcgagagcgtcagtattgagtggtggacaattagataggtgggagaaaattcggctaaggccaggggga
aagaaaaaatatagactaaaacatctagtatgggcaagcagggagctggatagattcgcacttaaccctagcctt
ttagaaacaacagaaggatgtcaacaaatattagaacagttacaaccagctattaagacaggaacagaagaactt
agatcattatataatacagtagcaaccctctattgcgtacatcgaaagatagaggtaaaagacaccaaggaagct
ctagataaaatagaggaactacaaaagaaaagcaagcaaaagacacaacaggcagcggctgacacaggaagtaac
agcagcacggtcagccaaaattaccctatagtgcaaaatgtacaagggcaaatgatacaccagcccttgtcacct
aggactttgaatgcatgggtaaaagtaatagaagaaaagggtttcaatccagaagtaatacccatgttctcagca
ctatcagaaggagccaccccacaagatttaaatatgatgctaaacatagtgggggacaccaggcagctatgcaa
atgctaaaagataccatcaatgaggaagctgcagaatgggacaggttacatccagtacatgcagggcctgttgca
ccaggccagatgagagaaccaaggggaagtgatatagcaggaactactagtaacactcaagaacaaatagcatgg
atgacaggcaatccacctatcccagtgggagacatctataaaagatggataatcctgggattaaacaaaatagta
agaatgtatagtcctgttagcattttggatataaaacaagggccaaaagaacccttcagagactatgtagatagg
ttctttaaagctctcagagctgaacaagctacacaggaggtaaaaggttggatgacagaaacattactggtccaa
aatgcaaatccagattgtaagtctattttaagagcattaggatcaggggctacattagaagaaatgatgacagca
tgccagggagtgggaggacccagccataaagcaagggttttggctgaggcaatgagtcaagtccaacagccaaac
ataatgatacagagaggcaattttagaggccagaaaaagattaagtgtttcaactgtggcaaagaaggacaccta
gccagaaattgcagggcccctagaaaacagggctgttggaaatgtgggaaggaaggacaccaaatgaaagactgc
actgagagacaggct
>#92 AJ320484 1296 nt
atgggtgcgagagcgtcagtattaagcgggggaaaattagatgcatgggaaaaaattcggttacggccaggagga
aagaaaaaatatagactgaaacatatagtatgggcaagcagggagttagaacgattcgcacttaatcctggcctt
ttagagacatcagaaggctgtaaacaaataataggacagctacaaccagctgttcagacaggatcagaagaactt
aaatcattatttaatacagtagcaaccctctattgtgtacatgagaggatagaggtaaaggacaccaaggaagct
ttagaaaaaatagaggaagaacaagccaaaagtaagaaaaaagcacagcaagcaacagctgacacaagaaacagc
agccaggtcagccaaaattatcctatagtgcaaaacctacagggacaaatgatacaccaggccatatcacctaga
actttgaatgcatgggtaaaagtaatagaagagaaggctttcagcccagaagtaatacccatgtttacagcatta
tcagaaggagccaccccgcaagacttaaacaccatgctgaacacagtagggggggcatcaagcagccatgcagatg
ttaaaagagaccatcaatgaggaagctgcagaatgggataggctacatccaccgcaggcagggcctgttgcacca
ggccagatgagggaaccaaggggaagtgatatagcaggaactactagcacccttcaggaacaaatagggtggatg
acaagcaatccgcctgtcccagtaggagagatctataaaagatggataatcctggggttaaataaaatagtaaga
atgtatagtcctgtcagcattttggacataagacaaggtccaaaggaaccctttagagattatgtagatcggttc
tataaaactctaagagccgagcaagcttcacaagatgtaaaaaattggatgaccgaaaccttgttggtccaaaat

gcaaacccagattgtaaaactatcttaaaagcactgggaccaggggctacattagaagaaatgatgacagcatgt
cagggagtaggggggacccagtcataaagcaagagttttggctgaggcaatgagccaagcaacaaatttgaatggt
gccgcaatgatgcagagaagcaattttaagggcccaaggaaaattgttaagtgtttcaactgtggcaaagaaggg
cacatagcaagaaattgcaaggctcctaggaaaaagggctgttggaaatgtggcagtgaaggacaccaaatgaaa
gattgcactgaaagacaggct
>#93 KT200357 1299 nt
atgggtgcgagagcgtcagtattaagcgggggagaattagatagatgggagaaaattcggttaaggccaggggga
aagaaaaaatataggttaaaacatatagtatgggcaagcagggagctagaacgattcgcagtcaatcctggcctt
ttggagacatcagaaggctgcagacaaatactgggacagttacaaccgtcccttcagacaggatcagaagaactt
agatcactatataatacagtagcaaccctctattgtgtacatcaaaagatagatgtaaaagacaccaaggaagct
ttagacaaggtagaggaagagcaaaacaaaagcaagaaaagggcacagcaagctgcagctggcacaggaaacagc
aaccaggtcagcgtcagccaaaattaccctatagtgcagaaccttcaggggcaaatggtacatcaggccatatca
cctagaactttaaatgcatgggtaaaagtggtagaggagaaggctttcagcccagaagtaatacccatgttttca
gcattatcagaaggagctaccccacaagacttaaacaccatgttaaacacagtgggggggacatcaagcagccatg
caaatgctaaaagagaccatcaatgaggaagctgcagaatgggatagattgcatccagtacaggcagggcctgtc
gcaccaggccagataagagaaccaaggggaagtgacatagcaggaactactagtacccttcaagaacaaatagca
tggatgacacataatccacctatcccggtaggagaaatttataaaagatggataatcttgggattaaataaaata
gtaagaatgtatagccctgtcagcattctggacataagacaaggaccaaaggaacctttagagactatgtagat
cggttctataaaactctaagagctgaacaagcttcacaggatgtaaaaaattggatgacagagaccttgttagtc
caaaatgcgaacccagattgcaagactattttgaaagcactgggaccagcagctacactagaagagatgatgtca
gcatgccaaggagtgggggggcccggccataaagcaagagttttggctgaagcaatgggccaaataacaaataca
gctaccataatgatgcagaggggcaattttaagaaccaaaaaaggactgttaagtgtttcaattgtggcaaggaa
ggacacatagccagaaattgcagggcccctaggaaaaaaggctgttggaaatgtggaagggaaggacaccaaatg
aaagattgcactgagagacaggct
>#94 AY331292 1296 nt
atgggtgcgagagcgtcagtattaagcgggggaaaattagataaatgggaaaaaattcggttaaggccaggaggg
aaraaaaagtataaattaaaacatatagtatgggcaagcagggaactagaacgcttcgcagtcaatcctggcctg
ttagaaacaacagaaggctgcagacaaatactgggacagctacaaccatcccttcagacaggaacagaagaactt
agatcattatataatacaatagcagtcctctattgtgtacatcaaaggatagatataaaagacaccaaggaagct
ttagataagatagaggaagagcaagacaaaagtaagcaaaaggcacagcaagcagcagctgacacaggaaacagc
aacaaccaggtcagccaaaattaccctatagtgcagaacctccaggggcaaatggtacatcaggccatatcacct
agaactttaaatgcatgggtaaaagtagtagaagagaaggctttcagcccagaagtaatacccatgttttcagca
ttatcagaaggagccaccccacaagatttaaacaccatgctaaacacagtgggggggacatcaagcagccatgcaa
atgttaaaagagaccatcaatgaggaagctgcagaatgggatagattgcatccagtgcatgcagggcctattgca
ccaggccagatgagagaaccaaggggaagtgacatagcaggaactactwgtacccttcaggaacaagtaggatgg
atgacaagtaatccacctatcccagtaggagaaatttataaaagatggataatcctgggattaaataaaatagta
aggatgtatagccctaccagcattctggacataagacaaggaccaaaagagccctttagagactatgtagaccgg
ttttataaaactctaagagctgagcaagcttcacaggaggtaaagaattggatgacagaaaccttgttggtccaa
aatgcgaacccagattgtaagactattttaaaagcattgggaccagcagctacactagaagaaatgatgacagca
tgtcagggagtgggaggacccggccataaagcaagagttttggccgaagcaatgagccgagtaacaaattcaaat
gccataatgatgcagagaggcaattttaagaaccaaagaaagactgttaagtgtttcaattgtggcaaagaaggg
catatagccagaaattgcagggctcctaggaaaaagggctgttggaaatgtggcagggaagggcaccaaatgaaa
gattgtactgagagacaggct
>#95 KP109514 1293 nt
atgggtgcgagagcgtcagtattaagcgggggagaattagatagatgggaaagaattcggttaaggccaggagga
aggaaaaaatatcaattaaaacatatattatgggcaagcagggagctagaacgatacgcacttaatcctggcctg
ttagaaacatcagatggctgtaaacaaatattggcacaactacaaccagcccttcagacaggatcagaaggattt
agatcattattcaacacagtagcagtactctattgtgtgcatcaaaagatagagataaaagacaccaaagaagct
ttagataagatagaggaagagcaaaacaaatttaagaaaaaggcacaacaagcagcagctgacacaggaaacagc
agccaggtcagccaaaattaccctatagtgcagaacatgcaggggcaaatggtacatcagcccatatcaccaaga
actttgaatgcatgggtaaaagtagtagaagagaaggctttcagcccagaagtaatacccatgttttcagcattg
tcagaaggagccaccccacaagatttaaacacaatgctaaacacagtgggggggacatcaagcagccatgcaaatg
ttaaaagagaccatcaatgaggaagctgcagaatgggatagattacatccagtggctgcagggcctgttgcacca
ggccagctgagagacccaaggggaagtgacatagcaggaactactagtacccttcaggaacaaataggatggatg
acacataatccacctatcccagtaggagagatctataaaagatggataatcctgggattaaataaaatagtaagg

atgtatagtcctaccagcattctggacataaaacaaggaccaaaagaaccctttagagactatgtggaccggttc
tataaagttctaagagccgagcaagcatcacaggatgtgaaaaattggatgacagaaaccttgttggtccaaaat
gcaaacccagattgtaaaactattttaaaagcattaggaccagcagctaccctagaagaaatgatgacagcatgt
cagggagtgggaggacccagccataaggcaagaattttggcagaagcaatgagccaagtaacaaacccaactacc
atactgatgcagagaggcaattttaagaaccaaaaaagaattgttaagtgtttcaattgtggcaaagaagggcat
atagccaaaaattgcagggcccctagaaaaagggctgctggaaatgtggaaaggagggacaccaaatgaaggac
tgcactgagagacaggct
>#96 AF146728 1311 nt
atgggtgcgagagcgtcagtattaagcgggggagaattggatagatgggagaaaattcggttaaggccaggggga
aagaaaacatataaattaaaacatatagtatgggcaagcagggaactagaacgattcgcagttaatcctggcctg
ttagaaacatcagaaggctgtagacaaatactggtacagctacaaccatcccttccgacaggatcagaagaactt
aaatcattatttaatacagtagcaaccctctattgtgtgcatcaaaagatagagataagagacaccaaggaagct
ttagaaaagatagaggaagagcaaaacaaaagtaagaaaaaggcacagcaagcagcagctgacgcagcagcagct
gatgcaggaaatagcagcaaggtcagccaaaattaccctatagtgcagaacctccaggggcaaatggtacatcaa
cccatatcacctagaactttaaatgcatgggtaaaagtagtagaagagaaggctttcagcccagaagtaataccc
atgttttcagcattatcagaaggagccaccccacaagatttaaataccatgctaaacacagtaggggggacatcaa
gcagcagccatgcaaatgttaaaagagaccatcaatgaggaagctgcagactgggatagattacatccagtacat
gcagggcctattgcaccaggccagatgagagaaccaaggggaagtgacatagctggaactactagtacccttcag
gaacaaataggatggatgacaaataatccacctatcccagtgggagaaatctataaaagatggataattctggga
ttaaataagatagtaagaatgtatagccctaccagcattctggacataaaacaaggaccaaaggaaccctttaga
gattatgtagaccggttctatagaactctaagagccgagcaggcttcgcaggaggtaaaaaattggatgacagaa
accttgttggtccaaaatgcgaacccagattgtaggactattttaaaagcattgggaccagcagctacactagaa
gaaatgatgacagcatgtcagggagtaggggggacccggccataaagcaagagttttggccgaagcaatgagccaa
gtaacaaattcagctaccataatgatgcagagaggcaattttaggaaccaaagaaagactgtcaagtgtttcaat
tgtggcaaagagggccacatagccagaaattgcagggccccgaggaaaaggggctgttggaaatgtggaaaggaa
ggacaccaaatgaaagattgtactgagagacaggct

Supplementary File S4. Dataset of 96 nucleotide sequences of the gene *pol* from primate lentiviruses: 37 sequences from SIV infecting Old-World monkeys (#1 - #37), 4 from HIV-2 (#38 - #41), 20 from SIV infecting African apes (7 from SIVgor, #42 - #48, 13 from SIVcpz, #49 - #61), and 35 from HIV-1 (9 from group O, #62 - #70, 2 from group P, #71 and #72, 7 from group N, #73 - #79, and 17 from the pandemic group M, #80 - #96). From each genome sequence we selected the non-overlapping region that encodes the Pol protein. Each nucleotide sequence contains the NCBI ac. number and the length of the coding region.

```
>#1  AF468658  2763 nt
gaagtctccatgggggacaagtggtgtccatgttattagacacaggggcggacgataccattgtccaagaatca
gcaattgaattagacacaccatggacaccaaaaacggtagggggatagggggtttaatacaagtaaaggaacat
agacatgtagaagtgatctttaatgaaaaagaatcaaagccactgtacttgtagggcccacaccagtgaatata
ttgggaagaaattgtttaagcaagttaggagtaaccttaaacatggtgcagcagaaactagagcccgtagaagtg
cacctaaaatcaggaaaagaaggaccaaagattaaacaatggcccttgtctaaggaaaagatagaggccttaacc
caaataacacaagaaatgctcaagttaggccaattggaaaagatagggccagaaaatccatacaactcgccagtc
tttgccataaaaaagaaagacaaatcacaatggagaatgttaatagatttcaggaaattgaatgaggctacgcaa
gacttggctgaggtccagttggggattccgcacccagcaggactagaacaaaaagagcacgtaactataatagat
atgaaagacgcttattacagtgtaccattgtatgaagaattcagaaagtacactgcattttcagtaccatcagta
aacaatcagaccccagctgagaggtatcagttcaaagttttacctcagggttggaaggcgtctccaaccatttt
caggccactgtagcttccttgttatatcagattagagaccaggagccagatgtggtaataatacagtacatggat
gacttgttgataggatcagataggaaattggcagagcataggcaagtagtgcacaaaataagaaatttgttgacg
tcctataacatccagacccccgaggccaaacaccaacaagattatccagtcagatggctcgggtatgagttgcac
cctaagggatggaggctccaaccagtggagctcccagatcaagacatctggacagtaaatgacatccaaaaattg
gtagggaagctcaattgggtatcgcaaatacatcctggcataaggacaaaacagttatgcagatgtattagagga
gccaaagggctaacagaacaggtggagctcactgaggaagcacaaatagagttagcagaaaacagagagattcta
aaacaatcctcagagggcagctactacgacgcagaaaagccacttgtggtagaaattacctccctaggagaacag
caatggggatacatgttcagtcaggacagcaagatgctaagatcaggcaaatttgcaaaaccagaactgcacat
ataaactcttatcagcaattggcagatgctatgaccaaagtaggtagggaaagtcaggttacttggggaaaagta
cctgacaaattcaggatcccagtagtcaaggagcaatgggatacgtggtggatgaactattggcaggcaacatgg
attcccaccatagaagcagtacacacaccacatttgttgagacagtggtacaccctagtctcggaacccctagaa
aatgcagtcacctattatgtagacggggcagcaaataggacctctaaattaggaaaagctggctatgtgaccaat
acaggaaaatatagagccatagaactagaaggaactacaaatcaacaagcagaactccaggcagttcttttggca
ttaaaagaaggaccaccaagaatgaacttggtcacagattcccaatacgtaatggggatcttacaaagccaacca
gaagtatccacctctcccttagtggaacaaatcatacaagaactgttagggaaagaggccgtctacttgagttgg
gttccagcacacaaagggataggaggaaacacagaggtagacaaattagtcagcagagggatccgccaagtactc
ttcatggagaacatagagcccgcagtagaggaccatgaaaaatatcatagtaattggaagtatttaaggatcaa
tacaaaatcccggcactattggctaaagaaatagtaaacaagtgctccaaatgtcagatacatggggaaccaaaa
catgggcaagtaaatgcagaattaggcatatggcaaatggactgcacacatctggaaggcaaagttatcttagtg
gcagtccacgtagccagtgggtatgtttgggctaggataataccccaagaaacaggcagacaaactgcattaaag
ctattagaattagcagcaacatggccagtaactcacctacatacagataatggccccaatttcatctctaaggaa
ctggaggcagcttgttggtgggcaaacatacaacacagtacaggggtgccatacaacccgcaaagtcaaggggta
gtagaaaacatgaacaaacaactcaaggaaacaatccaaaagattagagatgaggtcacatacttagaaacagca
gtagcacaagcatgctacattcataattttaaaagaaagggaggaataggggatatgtgccctacagagaggtta
gtgaacatgatccacacagaattagaaacacaacacttaaacacacaaagttccaaatttcaaaaatttcgggtt
tattacaggcaaggagctaatcctcattggcaaggaccagcagtacttctctggaagggtgaaggagcagtagtg
gtccaaacccaagcaggtgagatcatcacagtccctagaaggaaagcaaagatcatcaagcca
>#2  AF468659  2781 nt
```

gaagtctccataggggggacaagtggtgtccatgttattagacacaggggcggacgacaccatcgtccaagactca
gcaattgaattagatacaccatggacaccaaaaatggtaggggggaataggggggtttaatacaagtaagggagcat
aaacatgtagaagtaatctttaatgagaaaagaatcaaagctactgtactggttggacccacaccagtaaatata
ttaggaagaaattgcttaagtaagttaggggtaactttaaacatggtgcagcagaagatagaacccatagaagtg
cacctaaaggcaggaaaagagggaccaaagatcaaacagtggcccctgtctagggaaaagatagaggccttaacc
caaataacacaagagatgcttagattaggtcaattggaaaaaataggaccagaaaatccatacaactcaccagtc
tttgccataaaaaagaaggacaaatcacaatggagaatgctaatagattttagaaaattaaatgaggctacgcaa
gacttggctgaggtccagttgggaattccgcacccagcaggaattccgcaccctgcaggactagagcaaaaggag
catgtaactataatagatatgaaagatgcttattatagtataccattgtatgaggatttcaggaagtacaccgct
ttttcagtaccttcagtaaataatcaggccccagctgaaaggtatcagttcaaagtcttaccgcagggttggaaa
gcgtcgccaaccatctttcaaaacattgtagcttccttgctacgacagattagagatcaggagccagatgtggta
ttaatacagtacatggatgatttactgataggatcagataggaaactgggagaacacaggcaagtagtgcacaaa
ataagaaatttactaacatcctataacatccaaacccccgaggccaaacaccaaccagattatccggtcaagtgg
ctcgggtatgagttgcatcctaagggatggaggctccagccggtggaactcccagaccaggatacctggacagtg
aatgacatccagaaattggtagggaaattaaattgggtatcacaaatacaccctggtataaggacaaaacagttg
tgcaaatgtattaggggagctaaagggctaacagaccaggtagagctcactgaggaggcacagatagaattagca
gaaaatagggagatcttaaaacaatcttcagagggtggctattatgacgcagaaaagccgctcgtggtggaaatt
acctccctaggagaacagcaatgggggtacatgttcagccaagaaggcaagatgttaaaatcagccaaattcgca
aagaccagaaatgcacacataaactcctatcagcaatttgcagatgctttgaccaaagtaggtagggagagtcag
gttacctggggaaaggtaccagacaaatttaggatcccagtagtcaaggaacaatgggatgcatggtggatgaat
tattggcaggcaacatggatacccaccatagaagcaatacacacaccacacctactgagacaatggtacaccccta
gtctcggaacccctagaagatgcagttacttattatgtagatggagcagcaaataggacctccaaactaggaaaa
gctggttatgtgaccaatacaggaaaacatagggccatagaattagaagaaactacaaatcagcaagcagaactc
catgcagttcttttggcattaaaagaagggccaccaaaaatgaatctggtcacagattcccagtatgtaatgggg
atcttgcaaagtcagccagaggtatccacctctgcgctagtggaacaaatcatacaagaactgctaaaaaaggaa
gccatctacttaagttgggttccggcacacaagggaataggaggaaacacagaggtggacaaattagtcagcaag
ggaatccgccaagtgcttttcctggaaaacatagagcctgcagtagaagaccatgaaaagtatcatagtaattgg
aagtacctaagagatcaatacaaaatccccgcactattggctaaagaaatagtgaacaaatgttccaagtgtcag
gtacatggggaaccaaaacatgggcaggtaaatgcagaattaggcatgtggcagatggactgcacgcatttggaa
ggaaaggtcatcttagtggcggtccacgtagcaagtgggtatgtctgggctaggataataccccaggaaacaggc
agacaaactgcattgaagctcttagaattagcagcaacatggcctgtaacccacctacatacagataatggcccc
aatttcgtctccaaggaattggaagcagcctgctggtgggcagacatacaacacaccacaggggtgccatacaac
ccgcaaagtcaaggggtagtagaaaacatgaacaaacaactcaaggaaacaatcacgaaaatcagagaagaggtc
acatacttagaaacagcagtagcacaggcatgctacattcataattttaaaagaaagggaggaatagggggatatg
tgccctacagaaagaatagtaaatatgattcacacagaattagaaacacaacacttaaacacacaaaattccaaa
tttcaaaaatttcgggtttattacaggcaaggagctaaccctctttggcaaggaccagcagtactcctctggaaa
ggtgaaggagcagtagtagtccaaactcaagcaggtgagattattacagtccctagaaggaaagcaaagatcatc
aagcca
>#3 EF070329 2760 nt
attaatgggcagccagtgtccatgctcctggacaccggagcggatgacaccatcatccaagactcagatataaaa
attagtggggaattgactcccaaagtagtgggaggtttaggggggctgatcaatgtaaaacaatataagggggtg
atagtaaaattcaatgagaaaagaatagtagccacagtcttggtgagtcctacacccatcaatatcttagggaga
aattgcttatcaaaattggaaataacactaaacatggtaatggcaggacagcaattgaaacccactaaagttagc
cttaaagaaagaaaaaaagggccaatggtaaaacaatggccactctctaaggagaagatagaggcactaaaagaa
attactcagaaaatgataaaattaggtcaactggaggaagcagggcctaataacccatataattcaccagtattc
gccataagaaagaaagacaagacaaaatggcgaatgttaatagatttcagaaagcttaatgaggtgacacaagaa
tttgcagaagtccagacagggatcccacatccctcagggttagcccaaaaggcacatgtcactatagtagatatg
caggatgccttttatagtgtcccattagacaaagaattccgaccctacacggccttctcggtccctgcagtgaac
aacatggaccagctaaaagattccaatttaaggtgttgccacaggggtggaaggatcacccaccatttttcaa
gccacaacagccaagcttctagagcaagttaggaaggacaatcaggacgccttaatagtccaatacatggatgac
ctgcttatagggtcagacagagaaataggagaacatagaaggctagtgaaaaagatcagagatctcctctcaagt
aaggggatacagaccccagaagacaaacaccagccagactacccggtagaatggctagggtacgagttacatccg
aaaggatggagaatcaaaccagtggagctgccagaccaggacacctggacagtgaatgaaattcagaagctggta
gggaagttaaactgggcagcccaagtctattcagggatcaaaactaaacatttatgcagatgtatcaggggaata
aaggggctaacagagctagtggagctcagtgagcaagcccaattagaactagcagaaaatagggaaatcctgaag

caagaggccggtggggcctattatgacccagaaaaaccccctagtcttagagatagtgtccttaggggaacaacaa
tgggggtacacctttacccaagacagaaacatgctaagaacaggaaagtttgccaagatcagaacagcccatagt
aacccctaccaacagctggcagaagccctatctagagctagtaaggaggccctagtctgttgggggaagacccca
gacaaatgcaggataccggtagtcaaggaacagtgggacaattggtgggcagactcatggcaaacaacttggatt
cccgacatagaagcagtacacaccccatacccttctgagacagtggtttaccttggtcccagagcccatagaaaca
gcacccacctactatgtggatggagcagcaaataggaactctaaactaggaagagctggatatgtaaccgatagg
ggacaggagagagctataaatttggaaaacacaaccaaccagcaagcagagctccaagccattctcttggctcta
atggatgggccaccagaaatgaacctagtaacagattctcaatatgcattgggaataatcacagcagcaccagag
gtatcagagtcccctctggtggagcagataatacagcaaatgctgagcaaaaatgccatctttatatcccgagta
ccagcccacaagggcataggagggaatgaggaagtagaccacctagttagcaggggaatccgccaggtactattt
atggaaaatatagacccggcagttgaagaccatgagaagtaccattctaattggaagtatctcagggataaatac
aacatacccaccatcttagccaaggaaatagtaaacaaatgctcagcatgtcaaacacatggggaaccaaaacat
gggcaagtcaatgcagacttggggggtatggcaaatggattgcacacatctggaaggcaaggtcatcttggtggca
gtacatgtagccagtagtttcatatgggccaggataatttcccaagaaactggaaggctaacagccctagagctg
tcaaacttggcagccacatggccaattagtcagatacacccagacaatggtaccaactttactagcaaggaattc
caagctgttgcatggtgggctaacatacagcacaccacaggtgtcccctacaatccacagagtcaaggagtagta
gaaaatgcaaacaaacagttaaaggaaaccattcacaaagtaagggaagaagtaacatatttagaaacagcagta
gcacaagcagtcttcattctaaataataaaagaaagggagggatagggggatatgacccctacagaaaggttagtt
aatatgctatacacagaactagaaatacaacaactacaaaatcacaatacaaaattttcaaaatttcgggtttat
tacagaagaggagcaaacccttattggctgagaccagctcgacttctttggaaaggagaaggagctctagtgatc
aaaacaaaggaaggtgagatcgtaacagtcccaagaagaaaagcaaaaataatcaaggat
>#4 EF070330 2754 nt
atagaaggacaagtggtgcacatgctgctcgacacaggggcagatgacaccatagtccaggattccaacattcag
atagataaaccatggaatcccaaattggtgggggaatcgggggaaacatatcagtcagagaatatagaggggta
caagtatctttcaatgagaaaacaattaaggcaacagtgttggtgggacctacacccatcaacataatggggaga
aattgtttaagtaaatttggaatcaccttgaacatgatacaggaaaaaatagaacccataaaagtggccttaaag
gaaggggcaaaaggaccaatggtcaaacagtggccactgactcaagaaaagattaaggccttagaaggcatagtt
cagcaaatgttaaaattagatcagatagaagagatagggccagacaatccttacaactctccatgttttgcaatc
aggaagaaagataaatctaaatggagaatgttaatagattttagacagctaaatgaggccacccaagagttcaca
gaggtacagctagggatcccacatccagcaggattagcggaacatgagcatgtcacaatagtagatatcaaggac
gccttttacagcgtcccattagatccagcattcagaaaatacacagccttctcgctgccccaggtaaataaccag
ggaccagcaagaaggtatcagttcaaagtgctgccacaggggtggaaaggatcccccaccatcttccaatacacg
gcagcaaaattattacaggaaatcagggaagcaaatccagatatcaccctcatccagtacatggatgatctctta
ataggatccaacagagagagatatcgggacataggagagtagtagcacagattaggaacatgctcttgaaactgggg
atacaaaccccggaagataagtaccagaaagactacccagtccagtggctagggtatgagttacaccccagaggg
tggaaaatacagccagtaacactcccacagcaagaaaactggacagtaaatgatatacagaagttggtaggcaaa
ttgaattgggcagcgcaagcctacccaggagttaaaaccaaacagctatgcaaatgtataaggggagttaaatcc
ctgacagaagaagttcaactaacagaggaagctcaattagaattggcagaaaaccaggaaattcttaagcaggca
gtccagggggcatactttgatccagaacaacccctggtggctgaaattgtctcgttaggagactcacaatggggg
tataacttcctccaaaataagggaatattaaaatctggaaaatttgccaaagtcagatcagtccataccaacagt
tatcagcagttatctgatgccatagccagaataggaagggaatcattagtcatttgggggaagcccccgaaaag
gtcaggattccagtgatcaaggagcagtgggatcaatggtggacagaacattggcaggtatcatggatacctgac
atagaggcagtacataccacccacctgctgcgacagtggttcacattggtgccagagccattgtcagaagctccc
acatattatgtagatggagcagcacataaagtatctaagttagggaaggcaggatatgttactaacacaggaaaa
gaaaaggtggttagcttagaaaatactaccaatcaaaaggcagaactagaagctgtcttgttggccttgaaagaa
ggccctcccagcatgaatatagtcacagattcccaatatgtgctggggatagtggcttctcaaccccaagaatcc
acctcgccccttggtagaagaaataattcaacaattactgaccaaggaggcggtatacctatcgtgggtgccagcc
cataaaggtataggaggaaatgaggatgtggacaaactggtaagccatggaattaggcaggtgctattcatggag
caaatagagcctgcaaaagaggaccatgaaaagtaccatagcaattggaagtatctcagggataagtacaacatc
ccagctttactggctaaggaaatagtaaacctttgtcccaaatgtcagacacatggggagccaaaaacaggacag
gtcaatgcagaattgggagtgtggcaaatggattgtactcacttagaaggaaaaatcatcttagtagccgtccac
gtggccagtggatacacgtgggcaaaaatattacccaggaaacaggaaggcaaacagccctaggcctgttagaa
ttagcagctctttggcctgtaacacaaatacacacagataatggagccaacttcattagtgaagaattcggagct
gcatgttggtgggctagcatagatcacaccacaggggtaccatacaaccctcaaagtcagggagtagtagaaaac
aaaaacaaacagttaaaagaaactatacagaaaatcagagaagaagtcacctatttagaaacagcagtagcacag

gcagtcttcatcatgaattttaaaaagaagggaggaataggggacctagcagctgctgagagaataattaatatg
ttacatacagaactagaactacaacacttacaatcacaaaaatccaaatttcaaaattttcgggtttattatagg
acaggctctgacccatcttggaagggacctgctgccttgttgtggaagggtgaaggagcagtagtcgtcaaaaca
gaacaaggacaggtaatcactgtacctagaagaaaggcaaaaataatcaagcca
>#5 AY340701 2769 nt
tctgtgctgataggggacaaaaggtgtccatgctattggatacaggtgccgacgacacaattatacaagatcag
cacgtaaatttaggggagaattggacacctaaagtagtagggggaataggggaatgatagaagttaaacaatac
aaagcaatacaagtgatatttgaagacagagaggtttgggcaacagtattagttggaccaactcctataaatatt
ttagggagaaatgtcttagctaagatgggggtaaccctcaacatggctgctggggagacctccaacccccatct
ttagaggtcactttaaaagcaggaaaagagggaccaaagttgaaacagtggccattatccagagaaaagatagag
gctcttacacaaattactcaagagatgcttaaattaggacaattggaaccaaccgagcccaataacccatataac
tcaccctgttttgccataaagaaaaaggacaagagcaaatggagaatgctcatagatttcagagaacttaacaag
gcaactcaggacttctttgaggtgcagttgggaatcccacacccagcgggacttgagaagatggaccatgtgaca
atagtagacatgaaggacgccttctacagcattcccttgtgggaaccgttcagaaaatacacggcgttctcaata
ccctctttaaacaatgctgaacctgctaaaaggtatcagttcaaggtcctcccgcagggctggaaagggtcccca
actgtgtttcaacatacagcggcagagctaccacaggagatcaggaaaaaatacccagaggtaactttaatacag
tacatggatgacctactgataggatcaaatcactctctgccagaacacaggaaaatagtaggggtcataagagcg
accctactaaggaaagggattcaaacacccccagagaaatttcaggatgactacccggtgcaatggttagggtat
gagctacacccatcccattggaaaattgccaaaatagagctaccagaccaggaacaatggactgtcaatgagatc
caaaagttgataggaaagcttaattgggcagcacaaatctattcaggaataaaaaccaaaaacctgtgcaaaagt
atcagaggggcacggagcctgacagatacagtggtcctctcagatttagctcaagctgagctagcggaaaatagg
gagattctcaaggaggaatccaccgggggatattacgaccccaacgagccattaaaggtggagttaacctcacta
gcagaaaaccaatggggctacagattcttccaagaaaaatcagtattgaagacaggaaaatttgccaaaattaga
agtacccactccaatacctatcagcagttagcagatgcgctagccaaattgggaaaagaggccatagtcacatgg
ggacgacttcccatttttcgcctaccagtggtgaaagagcaatgggacgcatggtgggcagataattggcaaatc
aattgggtaccagacatagaggcggtgtattccccacacctgctcagacagtggtatactctggtacaagacccc
atagaaggggctccaacatactacgtggatgggccgccaataggacatcaaaggaagggaaagcaggttatgta
accaactttggggcaagaaaggccgtcacattagaacagaccaccaaccaaaaggcagagctagaggccgtgtta
ctagccctcagagatggaccacctcagatgaacataattacagattctcagtatgtactgggcattctagcctcc
tgcccggagatctcagaaagtcccatagtggaggccatcatacaggaactcttaaaaaaggaccaggtattcttg
tcatgggtaccagctcacaaaggaataggaggaaatgaagaagtagatcgcctagtgggccagggaattaggcaa
gtcctgtttatggaaaatttggaacccgcaagggaagatcatgaaaagtatcatagtaattggaaatacttaaga
gatacctaccacattcctactctgttggccaaagaaatagtcaaccattgtcacaaatgtcaaaaccatggagaa
cccaaatcaggacaggtaaacgcagaagtaggagtatggcagatggactgcacccatctagagggaagcataatt
ttggtagcagtacatgtagccagtggttatgtatgggccaagatcctaccaagggaaacaggaaaatgtacaggg
atagccctgttggagctggccgctatgtggccggtcactcagatacatacagataatgggccaaatttcaccagc
caagaattcgaagcagctgcatggtgggcaaatattcatcacaccacaggggtaccctacaacccacaaagccaa
ggggttgtagagaatatgaatagacaactaaaagagaccattaagaaaatcagggatgaggttgaatacctccca
acagcattagcacaagcactgttcatcttaaattttaaaagaaagggaggaataggggatatggcccctgtagac
agatttataaacatgatacacacagaattagaactacaaacctcaaacaaccaaattacaaaattttcaaaattt
cgggtttatttcaggacaggtgccgaccctcagtggaaaggaccagcgcgcctactctggaaaggtgaaggtgcc
gtagtcataaaaactgaggagggcgatatcctgacagtccctaggagaaaggcaaagataatcaaaccc
>#6 KJ461716 2754 nt
tcctgcttagtggagaggcaagaagttgacctactattggacacgggtgcagatgatacaataatccatgatcag
gacatagaactaggagataactggcacccaaaaattataggggggagtaggtggcaatattagagtaaaagcatat
catagtgttgaaatagaatggcagggaaaaagagtaacagaggaagtattggtgggagacactcccatcaatata
atggggagaaatttcttaaccaaatttggggtgactttaaacatggtagtaggcaaatttaagccgacagaagta
cggctaagagaagggaaagatggacccaaagtcaaacaatggccattaagtgcagagaaaatacaggcactaaaa
gagatagtacaagatatggtagataaaggacaattggaaaaaatagggccagaaaacccatacaattctccagta
ttttgtattaggaagaaggacaaaacaaaatggaggatgctaattgatttcaggaaactaaatgagcacacacag
gacttagcagaagtacagttggggatcccacacccagcaggtttacccaagaaggcacaggtttccatagtagac
atcaaagatgcctactatgcaatcccgctacatgaggactttcaaaaatacacagcattcaccataccttcagtg
aacaacatgggaccaggagaaagatatcagtttaaggtactgccacaggggtggaaggcatctccaactatattt
cagaacacaataggagacctgttgcaacagatcaggaaaaaatacccacaatccttatgattcagtacatggat
gacctcctcatagggtcagatgaaacagtaacagaacacaggaaaatagtagagcaaattaggaacatccttcta

aaacaagggttacagacccctgaggaaaaataccagccacaaaggccagttaattggttagggtatgagcttaga
cctagagagtggacaattcctagaataacattgccagttaagcaggtctatacagtcaatgagatccaaaagtta
gtaggacaattaaattgggccagccaaatatatccagggataaaaacaaaggctctgtgtaaattaattagagga
acaaaaggcttaacagaagaagtacaggtcacagaagaggcagaaacagagcttgcagaaaaccaagccatttta
gaccaagaagctaaaggggatactatgatgcagagaagccattagaagtagatatcatacagttgggaggaacc
cagtgggggtacacagtaagacaagacaaagaagtactaaagacaggtaaatttgcaaaacaaagaagtgcacat
agtaacccattcaaacaattggtagatgccattatgaaaataggaaaagaaagcttagtatactggggagaata
ccacaattcaatgtcccagttaacaaagagcagtgggacgcatggtggaatgaacattggcaggtgtcctggatt
ccggatataaaaccagtgcacactccacctttgatacaaatgtggtaccaattagtacaggatccaatttcagaa
gcagaaacttggtatattgatggagcagcaaatagggaatctaaattagggaaagcaggatatgtgacagataga
ggaaaagagaaagtagtagcattagaacataccaccaatcaaaaagcagagttacaagcattacttctagctcta
caagatgggggaacaaaacagaacatagttacagactcccaatatgttctaggaatcataacaggagctcccaca
gaaacagaccatccaatactagagcaaataataacacagttacaaagcaaagaagcaatatacctctcatgggta
ccagcccataaaggcataggggcaatgaagcagtagacaaactagtaagcaaaggaatcagaagggtattgttc
ctggaacaaataccacaagcccaggaagaacatgaaaggtatcataacaattggcaagaccttagagacaggttc
cagataccagcattaatagcaaaagagatcttaaaagcatgccccaagtgtcaaggaaaaggagagccaatgcat
gggcaggtgaacatggaagtgggactctggcagatggattgtacacacttggaagggaaaatcataatagtagca
gtacatgtagccagtggatacacagaagccaagctaatcccacaagaaacaggaaaagagacggctatattcctg
ttacaactatgtgctagatggccagtcacacagatacacacagataatggaccaaatttcaccagccaggaatta
gcagcagcagcatggtgggctaatgtacaacacagcacaggtgtgccttacaatccacagagtcagggagtagta
gaaaacaagaacaagcaattaaaggaaacaataagcaaaatcagagatgaagtacaatacttagagacagcagta
gcaatggcactcctcattcttaattttaagaaaaggggaggaataggggggatgacaccagcagaaaggctaata
aacatgatacacacagacttagaactacaacaaaccaaatttccaaaattttcgaattttcgggtttattacagg
acagggaagagccctgagtggctagggccagcacaactcctgtggaaaggagaaggtgcagtggtaatcaagaca
actacaggtgagctgttgacagtcccaaaaaggaaagcaaagatcattaagccc
>#7 KJ461714 2754 nt
tcctgtttagtagagggacaagaagttgacctactattggacacgggtgcagatgatacaataattcatgatcag
gacatagacttaggagataactggcacccaaaaattataggggagtaggtggcaatattagggtaaaagcttat
catagtgttagcatagaatggcagggaaaaagagtaacagaggaagtattggtgggagacactcccatcaatata
atggggagaaatttttaaccaaatttggggtaactctaaatatggtagtaggcaaatttaagccaacagaggta
cggttaaaggaagggaaagatggacccaaagtcaaacaatggccattaagtgcagaaaaaatacaggcactaaaa
gagatagtacaagatatggtagacaaaggacaattggaaaaagtagggccagaaaacccatacaattctccagta
ttctgtattaggaagaaggacaaaacaaaatggaggatgctaattgatttcaggaaactaaatgagcacacacag
gacttagcagaagtacagttggggatcccacacccagcaggtttacccaagaaagcacaggtttccatagtagac
atcaaggatgcctactatgcagtcccgctacatgaggactttcaaagatacacagcattcacaataccctcagtg
aacaacatgggaccaggagaaagatatcagtttaaggtactgccacaggggtggaaggcatctccaaccatattt
cagaacacaataggagacctgttacaacagatcaggaaaaatacccacaagtcctcatgattcagtacatggat
gacctcctcatagggtcagacgagacaataacagagcacaggaaaatagtagagcaaattaggaacatccttcta
aaacaagggctacagacccctgaggaaaaataccagccacaaaggccagttaattggttagggtatgagcttagg
cctagagagtggacaattcctaaaataacattgccagttaagcaggcctatacagtcaatgagatccaaaagcta
gtaggacaattaaattgggccagtcaaatatacccagggataaagacaaaggctctgtgtaaattaattagggga
acaaaaggtctaacagaagaagtacaggtcacagaagaggcagaaacagagcttgcagaaaatcaagccatttta
gaccaagaagctaaaggggatactatgatgcagagaagccattagaagtagatatcatacaattagggggaacc
cagtgggggtatacagtaagacaagacaaagaagtactaaagacaggtaaatttgcaaaacaaagaagtgcacat
agtaacccattcaaacaattggcagatgctattatgaagataggaaaagagagcttagtatattggggagaata
ccacaatttaatgtcccagttaacaaagagcagtgggacgcgtggtggagtgaacattggcaggtgtcctggatt
ccggacataaaaccagtgcacaccccacctttgatacaaatgtggtaccaattagtacaggaaccaatcccagaa
gcagaaacttggtatattgatggagcagcaaatagggaatctaaattagggaaagcaggatatgtgacagataga
ggaaaagagaaggtggtagcattagaacacaccaccaatcaaaaggcagagttacaagcattgctcctagcttta
caagatgggggaacaaaacagaacatagttacagactcccaatatgttctaggagtcgtaacaggagctcccaca
gaaacagaccacccaatactagagcaaataataacacagttacagagcaaagaagcaatatacctctcatgggta
ccagcccataaaggtataggggcaatgaagcagtagacaaattagtaagcaaaggaatcagaagggtattgttc
ctggaacaaataccacaagcccaggaagaacatgaaaggtatcataacaattggcaagaccttagagacaggttc
caaataccagcattaatagcaaaagagatcttgaaagcatgccccaagtgtcaaggaaaaggagagccaatgcat
gggcaggtaaacatggaagtggggctctggcagatggattgtacacacttggaagggaaaatcataatagtagca

gtacatgtagccagtgggtacacagaagccaaactaatcccacaagaaacaggaaaagagacggctatattcctg
ttacaactatgtgctagatggccagtcacacagatacacacagacaatggaccaaatttcaccagccaggaacta
gcagcagcagcatggtgggctaatgtacaacatagtacaggtgtgccttacaatccacagagtcagggagtagta
gagaacaagaacaggcaattgaaggaaacaataggcaaaatcagagatgaagtacaatacttagagacagcagta
gcaatggcactcctcattcttaattttaagaaaaggggaggaataggggggatgacaccagcagaaaggataata
aacatgatacacacagaattagaactacaacaaaccaaaattccaaaattttcgaattttcgggtttattacagg
acagggaagagccctgagtggctagggccagcacaactcctgtggaaaggagaaggtgcagtggtaatcaagaca
actacaggtgagctgttgacagtcccaagaaggaaagcaaagatcattaagcct
>#8 KJ461715 2754 nt
tcctgtttagtggaggggcaagaagttgacctactattggacacgggtgcagatgatacaataattcatgatcag
gacatagaattgggagataactggcacccaaaaattataggggagtaggtggcaatattagagtaaaagcatat
cataatgttgacatagaatggcagggaaaaagagtaacaggggaagtgttggtgggagacactcccatcaatata
atggggagaaatttcttaactaaatttggggtgactctaaatatggtagtaggcaaatttaagccgacagaagta
cggttaaaagaagggaaagacggacctagagtcaaacaatggccattaagtgcagaaaaaatacaggcactaaaa
gagatagtgcaagatatggtaaataaaggacaattggaaaaaatagggccagaaaacccatacaattctccagta
ttttgtattaagaagaaggacaaaacaaaatggaggatgctaattgatttcaggaaactaaatgagcacacacag
gacttagcagaagtacagttggggatcccacacccagcaggtctacccaagaaggcacaggtttccatagtggac
attaaagatgcctactatgcagtcccgctacacgaggactttcaaaaatatacagcattcacgataccttcagta
aacaacatgggaccaggagaaagatatcagtttaaggtgctgccacaggggtggaaggcatctccaactatattt
cagaacacagtaggagacctgttacaacagatcaggaaaaaatacccacaaatcctcatgattcaatacatggat
gacctcctcatagggtcagatgagacagtaacagaacatagaaaaatagtagagcaaattaggaacatccttcta
aaacgagggttacaaacccctgaggacaaatatcagccacaaaggccagttaattggttagggtatgaacttagg
cctagagaatggacaattcctagaataacattgccagttaagcaggtctatacagtcaatgagatccaaaagtta
gtaggacaattaaattgggccagccaaatatacccagggataaaaacaaaggctctatgtaaattaattagaggg
acaaaaggcttaacagaggaagtgcaggtcacagaagaggcagaaacagagcttgtagagaaccaagccattta
gaccaagaagctaaaggggatactatgatgcagagaagccattagaggtagatatcacacaattgggagaaacc
cagtgggggtacacagtaagacaagataaagaagtactaaagacaggtaaatttgcaaaacaaagaagcgcacat
agtaacccattcaaacaattggtagatgccattatgaaaataggaaaagaaagcttagtatattggggaagaata
ccacaatttaatgtcccagttaataaagaacagtgggacgcgtggtggagtgaacattggcaagtgtcctggatt
ccggatataaaaccagtgcacaccccacctttgatacaaatgtggtaccaattagtacaagatccgatcccagga
gcagaaacttggtatattgatggagcagcaaatagggaatctaaattagggaaagcaggatatgtgacagacaga
gggaaagaaaaagtagtagcattagaacacaccaccaatcagaaagcagaattacaagcattacttctagctcta
caagatgggggaacaaaacagaacatagttacagattcccaatatgttctaggaatcataacaggagctcctaca
gaaacaaaccacccaatactagagcaaataataacacagttacaaagcaaagaagcaatatacctctcatgggta
ccagcccataagggtataggaggtaatgaagcagtagataaattagtaagcaaaggaatcagaaaggtattgttc
ctggaacaaataccacaagcccaggaagaacatgaaaggtatcataacaattggcaagaccttagagacaggttc
cgaataccagcattaatagcaaaagagattttaaaagcatgccccaagtgtcaaggaaaaggagagccaatgcat
gggcaggtaaacatggaagtgggactctggcagatggattgtacacacttggaagggaaaatcataatagtagca
gtacatgtagccagtgggtacacagaagccaaactaatcccacaagaaacaggaaaagagacggctatattcctg
ttacaactatgtgctagatggccaatcacacagatacacacagataatggaccaaatttcaccagccaagagttg
gcagcagcagcatggtgggctaatatacaacacagcacaggtgtgccttacaatccacaaagtcagggagtagta
gaaaataagaacaggcaattaaaagaaacaataagcaaaatcagagatgaagtacaatacttagagacagcagta
gcaatggcactcctcattcttaattttaagaaaaggggaggaataggggggatgacaccagcagaaaggctaata
aacatgatacatacagacttagaactacaacaaaccaaatttccaaaattttcgaattttcgggtttattacagg
acagggaagagcccagagtggctagggccagcacaactcctgtggaaaggagaaggtgcagtggtaatcaagaca
actacaggtgagctgttgacagtcccaaaaaggaaagcaaagatcattaagcct
>#9 JX860407 2751 nt
acagcgcaggtcgagggtcagcctgtggaagtattactggacacaggggctgatgactctatagtagcaggagta
gaattaggaacaaattacaccccaaagatagtaggaggaattggaggttttattaatactaaagaatataaaaat
gtagaaatagaagtactagggaaaagaattagaggaacactcatgacaggagataccccaatcaatattttggc
agaaatttactaacagctttgggtatgtccttaaacttcccaatggcccaagtggagccggtaaaagtaacacta
aaaccaggaaaagatggaccaaaagtaaggcaatggccactatcaaaagaaaagatacaggcattaaaagagatc
tgtgaacagatggaaaaggatggtcaattagaagaagctccacctaccaatccatacaatacacctacttttgct
ataaagaagaaagacaaaaacaagtggagaatgctaatagatttcagggaactaaataaagtcactcaggatttc
acagaagtgcagttgggaattccacacccagcaggattggcaaagagaaaaaggattacagtcctggatgtaggg

gatgcatatttctctatacccttagatgaggacttcaggcagtatactgctttcaccctaccatcggtaaataat
gcagagccaggaaaaagatatatctacaaggttctaccacaagggtggaaaggatcaccagcaattttcaacat
acaatgagaggtgtattggagccatttagaaaagcaaatccagatgtaaccctaattcagtacatggatgacatc
ctgatagccagtgacagaacagacttagaacatgacagagtagtgctgcagttaaaagaactcttaaacggaatg
ggcttctctactccggatgagaagttccagaaagaccctccatatcaatggatggggtatgaactgtggccaaca
aaatggaaattgcaaaaaatagaattgcctcagaaagacaattggacagtaaatgacattcagaaactagtagga
gtgttaaattgggcagcgcaagtctatccaggaatcaaaaccaaacatctttgtaaactgatcagaggaaaaatg
actctgacagaggaagtccaatggactgaattggcagaagcagagtatgaggaaaacaagatcattctcagtcag
gaacaagaaggatgttactaccaagagggaaagcctttagaggcaacggtagtgaaaaatcaggataatcagtgg
tcttacaaaatccaccaggaagataaagtattgaaagtaggcaaatttgccaaaataaagaatacacatacaaat
ggagttaggctattggcacatgtggtacaaaagataggaaaggaggctatagtgatctggggacggatcccaaaa
ttccatctaccagtagagagagaaatctgggagcaatggtggacagactactggcaggtgacctggataccagac
tgggactttgtatcaacacccccttttggtcagattagttttcaacctagtaaaggatcctcttgaaggagaagaa
acattctacacagatggatcatgcaacaggcagtctaaagaagggaaagcaggttatgtaacagatggaggaaaa
gacagggtaaaggtgttagaacagaccactaatcagcaagcagagctagaggcctttctcatggcattaacagac
tcaggaccaaaagcaaatatcatagtagattcacaatatgtaatgggaatagtagcaggtcaacctactgaatca
gaaaataagatagtaagccaaatcatagaagaaatgatcaaaaagacggcaatttatgtcacatgggtaccagct
cacaaagggctaggaggaaatcaagaagtagaccacctagtcagtcagggaatcagacaagtattgtttctagaa
aagatagaaccagctcaagaagagcatgaaaaataccacagcaatgtaaaagagttggtattcaaatatggcata
ccaagattggtagcaaaacaaatagtagatacctgtgacaaatgccaccaaaaaggggaagctatacatggacaa
gttaatgcagatctaggaacctggcaaatggattgcacacacttagaaggaaaaatagtcatagtggcagtacat
gtggctagtggattcatagaagcagaagtaattccacaagagacagggagacagacagcattattcctgttaaaa
ttggcaagtagatggcctatcacacatttgcatacagacaatggtgccaactttacttcacaagaagtaaaaatg
gtagcttggtggacaatatagagcacacctttggagtaccctataatccacagagccaaggagtagtggaagct
atgaatcaccacttgaaaacacaaatagacagaattagagagcaagcaaattcagtagaaaccatagtactaatg
gcagttcattgcatgaattttaaaagaaggggaggaataggggatatgaccccagcagaaagactaattaatatg
attaccacagaacaagaaatacaattccagcaatccaaaaattcaaaatttaaaaattttcgggtctattacaga
gaaggcagagatcagctgtggaaaggacccggtgaactattgtggaaaggggaaggagcagtcatcttaaaggta
gggacagatataaaggtagtgcccagaagaaaagctaagatcataaaagat
>#10 U79412 2751 nt
actgctcatattgagggacagcctgcagaagtactattggatacaggggctgatgattctattgtagcaggaata
gagttaggtccacattataccccaaaaatagtaggaggaataggaggttttattaatactaaagaatacaaaaat
gtagaaatagaagttttaggcaaaaggattaaaggacaatcatgacaggggacaccccgattaacatttttggt
agaaatttgctaacagctctgggaatgtctctaaatttccccatagctaaggtagagcctgtaaaagtcacctta
aagccaggaaaagatggaccaaaattgaggcagtggccattatcaaaagaaaagatagttgcattaagagaaatc
tgtgaaaagatggaaaaggatggtcagttggaggaagctcccccgaccaatccatacaacacccccacatttgct
ataaagaaaaaggacaagaacaaatggagaatgctgatagattttagggaactaaataaggtcactcaggacttt
acagaagtccaattaggaataccacaccctgcaggactagcaaaaaggaagaggatcacagtactggatgtaggt
gacgcatatttctccatacctctagatgaagaatttaggcagtacactgcttttactttaccatcagtaaataat
gcagaaccaggaaaacgatacatttataaggtcctgcctcaggggtggaaggggtcaccagccatcttccaacac
actatgagaaatgtgctggaacccttcaggaaggcaaatccagatgtgaccctagtccagtatatggatgacatc
ttagtagctagtgacaggacagacctggaacatgacagggtagttttacagttaaaggaactcttaaatagcata
gggttttctaccccagaagagaagttccaaaaagatcccccatttcaatggatggggtatgaattatggccaaca
aaatggaagttgcaaaagatagagttgccacaaaaagagacctggacagtgaatgatatacagaagttagtagga
gtattaaattgggcagctcaaatttatccaggtataaaaaccaaacatctctgtaggttaattagaggaaaaatg
actctaacagaggaagttcagtggactgagatggcagaggcagaatatgaggaaaataaaataattctcagtcag
gaacaagaaggatgttattaccaagaaggcaagccattagaggccacggtaataaagaatcaggacaatcagtgg
tcttataagattcaccaagaagacaaaatactgaaagtaggaaaatttgcaaagataaaaaatacacataccaat
ggagttagactattagcacatgtaatacagaaaataggaaaggaagcaatagtgatctggggacaggtcccaaaa
ttccacttaccagtcgagaaggatgtatgggaacagtggtggacagactattggcaggtaacctggataccagaa
tgggattttatctcaacgccgccactagtaagattagtcttcaatctagtgaaggaccctatagagggagaagaa
acctattatgtagatgggtcatgtaataaacagtcaaaagaagggaaagcaggatatatcacagataggggcaaa
gacaaagtgagagtcttagaacagactactaatcaacaagcagaattggaagcatttctcatggcattgacagac
tcagggccaaaggcaaatattatagtagattcacaatatgttatgggaataataacaggatgccctacagaatca
gagagcaggctagttaaccaaataatagaagaaatgattaaaaagacagaaatttatgtagcatgggtgccagca

cacaaaggtataggaggaaaccaagaaatagaccacctagttagtcaagggattagacaagtcctcttcttggaa
aagatagagccagcacaagaagaacatgataaataccatagtaatgtaaaagaattggtattcaaatttggatta
cccagactagtggccaaacagatagtagacacatgtgataaatgtcatcagaaaggagaagctatacatgggcag
gtaaattcagatctagggacttggcaaatggattgtacccatctagagggaaaaataatcatagttgcagtacat
gtagctagtggattcatagaagcagaagtaattccacaagagacaggaagacagacagcactatttctgttaaaa
ttggcaagcagatggcctattacgcatctacacacagataatggtgccaactttacttcgcaagaagtaaagatg
gttgcatggtgggcagggatagagcacacctttggggtaccatacaatccacagagtcagggagtagtggaagca
atgaatcaccatctaaaaatcaaatagatagaatcagggaacaagcaaattcaatggaaaccatagtattaatg
gcagttcattgcatgaattttaaaagaaggggaggaataggggatatgactccagcagaaaggttacttaacatg
atcactacagaacaagaaatacaattccaacaatcaaaaaactcaaaatttaaaaattttcgggtctattacaga
gaaggcagagatcaactgtggaagggacccggtgagctattgtggaaaggggaaggagcagtcatcttaaaggta
gggacagacattaaggtagtacccagaagaaaggctaagattatcaaagat
>#11 JQ864086 2751 nt
actgcctacattgaagaacagcccgtagaagtattattagatacaggggctgatgattcaattgtagcagggata
gaattaggtccaaattataccccctaaagtagtaggaggaataggaggcttcattaataccaaagaatataaagat
gtaaaaataaaagtcttaggcaaggtgattaagggaacaattatgacgggagataccccaattaatatttttggc
agaaatttgctaacagctatgggcatgtctttaaatctccccatagctaaggtggagcctataaaagtaacacta
aaaccagggaaagaaggaccaaaattaagacagtggccgctatcaaaagaaaagataattgcattaagagaaatc
tgtgaaaaaatggaaaaagatggccagttagaggaagcccctccaaccaatccgtataacacccccacttttgct
ataaagaagaaagacaaaaataaatggaggatgctaatagattttagagaattaaataaggtcactcaagacttt
acagaagtacagttaggaataccacaccctgcaggactagcaaagagaaggaggatcacagtattggatgtaggt
gatgcatatttctccatacctctagatgaagaattcaggcagtacactgcctttactttaccatcagtaaataat
gcagaaccaggaaaaagatacatctataaggtattacctcaagggtggaaggggtcaccagctatttttcagtat
actatgagaaatgtattagaacctttcaaaaaagcaaatccagatgtgaccctgatccaatacatggatgacgtc
ttaatagctagtgatagaacagatttagagcatgacagggtagttttacagttaaaggaacttctgaacggcata
ggattctctaccccagaagagaagttccagaaagatcccccattccagtggatgggatatgaattgtggccaacc
aaatggaaactgcagaaaatagagttgccacaaagagagatttggacagtaaatgacatacaaaaattagtagga
gtgctaaattgggcagcacaaatttatccaggaataaagactaaacatctttgcagactaatcagaggaaaaatg
actttaacagaagaggttcagtggactgagatggcagaggcagaatatgaagaaaacaagataattctcagtcaa
gaacaagaaggatgttactaccaagagggaaaaccaatagaggcaacagtaataaagagtcaggataatcaatgg
tcatataaaattcaccaagaagacaaagtactgaaagtaggcaaatttgcaaaggttaaaaatacacatacaaat
ggagtcagattactagcacacgtagtgcagaaaataggaaaagaagcactagtaatttggggagaggtgccaaaa
ttccatttgccagtagaaagagaaatttgggaacaatggtggacagattattggcaagtaacctggataccagat
tgggactttgtgtcaacacctcccttagtcagattagtcttcaacctagtaaaagagcctatacagggggcagaa
acattctatgtagatggatcctgtaataggcagtcaagagaaggaaaagcaggctatgtggcggataggggcaga
gacaaagcaaaacttttagaacagactaccaaccaacaagcagagttggaagccttctatctagccttagcagat
tcgggaccaaaagcaaatattatagtagattcccaatatgttatgggcataatagcaggtcaacccactgaatca
gaaagtaggttagtaaaccagataatagaggagatgattaaaaaagaagcaatttatgtagcatgggtgcctgca
cataaaggaataggaggaaatcaagaagtagatcacctggttagccagggaattagacaagtcctattcttagaa
aaaatagaaccagcacaagaagagcatgaaaagtaccatagtaatgtaaaagaattggtattcaaatttggttta
cctaggctagtagcaaaacagatagtagacacatgtgataaatgccaccagaaaggagaagccatacatgggcaa
gtaaatgcagaactagggacttggcaaatggactgtacgcacctagaaggcaaaataattatagttgcagtacat
gtggctagtggattcatagaggcagaagtaatcccgcaggaaacaggaagacaaacagcactgtttctgttaaag
ttagctggcagatggcctatcacacatctgcatactgataatggtgccaatttcacatcacaagaagtaaaaatg
gttgcctggtgggcagggattgaacagacctttggggtgccttataatccacagagccaaggagtagtggaagca
atgaaccatcatttaaaaacccagatagatagaattagagaacaagcaaactcaatagagactatagtactaatg
gcagttcattgtatgaattttaaaagaaggggaggaataggggatatgactccagcagaaagattagtcaatatg
atcaccacagaacaagaaatacaattccaacaatcaaaaaattcaaaatttaaaaattttcgggtctattacaga
gaaggcagagaccagctgtggaaaggacccggtgagctattgtggaaaggggaaggagcagtcatcctaaaggta
gggacagagatcaaggtagtaccaaggaggaaagctaaaattatcaaagac
>#12 AF334679 2751 nt
agagcactaatagagggtcagcccctagaggttctactggacacaggggcagacgactccatagtagcagggata
gagttaggtccagggtacacaccaagaattgtaggggggattggaggattcataaatacaaaagaatatagaaca
gtaaaaatagaagttttaggaaaggtaataagagaaacactaatgacaggagataccccgataaatatttttggc
agaaatattctagcagcattaggggtctctttaaattacccagtggcaaaggtagaatatacaaaagtaaagttg

aaggagggaatggatgggccaagattaaaacaatggcccttatctaaggaaaagatacaagcattaacagagatc
tgcgagaaaatggaaaaggagggacaattggaacgagcccctcctacaaatccttataatacaccgacctttgct
attaagaaaaagaataaggacaagtggagaatgctcatagatttcagagaattaaacaaaatgacacaagaattc
acagaagtacagctgggtatcccacatccggcaggcctaaaggaaatggaaaggataacagtgctagatataggg
gatgcctatttttcagtacccctagacccggaatttagacaatacacagcattcactataccatcagtgaacaac
caagaaccaggaaagaggtatatttacaaggtcctgccacaaggatggaagggatctcctgcaattttccaggca
accatgaggcaggtattagaacccttcagaaaagctaacccagatgtgcttctcgtccagtatatggatgattta
ctaattggcagtaacagaggcctaacagagcatgacaaaatggtaacacagttaagagacatgctcaacaatctg
gggttcagtaccccagaagacaagtttcaaaagaatccaccattacagtggatgggatatttgttataccccaag
aagtggaaattacagaaaatagaactccctgaaaaggagcgatggacagtaaatgacatacaaaaactagtggga
gttctaaattgggcagcacagatatatccagggattaaaacaaaaaacctgtgcaaaatgatcagagggaaaatg
actctgacagaagaagttcagtggacagaattagcagaagcagaattggcagagaatagaattattctaaatcaa
gagcaggaaggaagatattacagagaagatgaaccgctagaagcaacggttctaaagaatcaagacaatcagtgg
agttataagattcatcaaggtgacaggattctcaaggtagggaagtttgcaaaaataaaaaatacacatacaaat
ggaataaggttactggctaatgtagttcaaaagataggaaaagaaagtctagtgatatggggaaagacaccattt
tttcaccttccagtagaaagggaggtttgggatcaatggtggacagattattggcaagctacttggattccagag
tgggacttcatctccacaccaccgttaatcaggttagtgtttaatttagtaaaagatccaatagaaaaggaggaa
gtatattacattgatggctcctgcaatagaaacagtaaagaagggaaagcaggatatgtcacagacaggggaaaa
gaaaaggtcctgccattagaacaggctaccaatcagcaagcagagctgcaggccctgttattggccttgaaagat
tctccaagtaaggtcaatgtagtaacggactcacagtatgtcttgaacataataacaggacagccatcagaatcg
gattcagatatagtggcacaaattattgaacaactagttcagaaagaagcagtatacataggatgggttccagct
cataaaggcataggagggaacaatgaggtagaccgtttagttagtcaaggaataagacaggtcctgttcctagaa
agtatagagccagcacaagaagaccatgacaaatatcatagcaatgtaaaggagttagctcagaaatataacatc
ccacaattagtagctaaacagatagtaaatgcttgtaacaaatgccaacagaagggagaagcgatacatggtcag
acaaatgcagaggtaggcacatggcagatggactgcacccacttagaaggaaaggttatcatagtagcagtacat
gtggctagtggctttatagaagcagaggtaatacctagggaaacaggaagacagacagcattgttcctcttaaaa
atagcatcaagatggcctataaagcatctgcatacagacaacggtgccaatttcacctcccaggaggtgaagatg
gtagcatggtggttaggagtggaacagtcctttggagtaccgtacaacccacaaagtcagggagtagtagaagcc
atggacttacatcttaagaaaaacatagataaaataagggaacaagcagagtcagtagagaccctagtactaatg
gcagcacattgcatgaattttaaaagaaggggaggaataggggatatgactcctgcagaaagaatagtaaatatg
atcaccacagaactagaaacccaatacttaaattcacaaaattcaaaatttcaaaattttcgggtttattacaga
gaaggaagagaccaactgtggaagggaccagcagagctcctctggaaaggagaaggggctgttgtaattaaggta
gggacagaaataaaagtggtaccaagaagaaaagcaaaaatcatcaaagat
>#13 JX860414 2751 nt
acagcccatattgaaggtcagccagtagaagtattattagacacaggggctgacgactcaattgtagcaggaata
gaattaggattaaattataccccaaaagtagtaggagggataggaggattcattaataccaaggaatacaaaaat
gtagcaatagaagtattaggtaaaacaattaggggaacagtcatgacggggggataccccaatcaacatttttggc
agaaatctgttaacaagattagggatgtctttaaattttccagtagctagggtagaaccagtaaaagtgacacta
aaaccagggcaggatggaccaaaattaagacagtggccattatctaaagaaaaaatagaggcattgaaagagatc
tgtgaaaaaatggagaaggagggccagttggaggaagctcctcctactaatccatacaatacgcccacctttgcc
ataaagaaaaaggacaaaaataagtggagaatgctaatagacttcagagaactaaatagggttacacaggatttc
acagaagttcaactaggaataccacaccctgcaggactagcaaaaagaagaagaattacagtactggatgtaggg
gatgcatacttttccataccactggacccagaattcagacaatatactgcctttaccctaccatcagttaacaat
gcagagccaggaaagagatacatctacaaggtcttaccacaaggatggaaagggtcaccagcgattttccaacac
acaatgagaaatgtattggagccttttagaaaggccaatccagatataaccttagttcagtacatggatgacatc
ttgatagctagtgataggacagacttggaacatgacagggtggtcctgcagttaaaagaactattgaatggccta
gggttctccaccccagatgagaaatttcagaaagaccctccatatcattggatgggatatgagttatggcctaca
aaatggaagctgcaaaagatagagctaccacaaagagaaagctggacagtaaatgacattcagaagttagtagga
gtactaaattgggcagcacaaatttatccagggataaagacaaaacacctctgcaagctaattagaggaaagatg
gcattgacagaagaagtccaatggacagaaatggcagaggcagagtttgaggaaaatagaatcattcttaatcaa
gaacaagaagggtgttactaccaagaggataagcccttagaagcaaccatagtgaaaagccaagataatcagtgg
tcttataagatccatcaagaaaataaaatactgaaagtaggtaaatatgcaaaaataaagaatacacatacaaat
ggagtcaggttattggcacatgtagtacaaaagataggaaaagaagcaatagtaatttggggaaaagtgcctaaa
tttcatatgccagtagaaagagaaatttgggagcagtggtggacagactattggcaggtaacatggataccagaa
tgggatttcatatcaacaccccctttagttaggctagtgtttaatctggtaaaagaccctatagaaggggcagaa

acattctatgttgatggttcttgtaataggcagacgaaggtaggaaaagcaggatacataacagacagaggaaaa
gataaggtaaaactcttagaacagactaccaatcaacaagcagaattagaagcttttctcatggcagtaacagat
tcaggaccagaagcaaacattatagtagactctcagtatgtcatgggaatagtagcaggacaacctactgaatca
gaaagcagggtagtaaaccaaataatagaggagatgatcaaaaagacagcaatctatgtggcatgggtaccagcc
cataaaggcataggaggaaatcaggaaatagatcatttagttagtcaaggaatcagacaagtcctgtttctagag
aaaatagagccagcacaagaagaacatgagaagtaccatagtaatgtaaaagaactggtgttcaaatttagaata
cccagattagtagcaaaacagataattgatacttgtgacaaatgccaccaaaaaggggaggcaatacatggacaa
gtaaatgcagagttagggacttggcaaatggactgcacacacttagaaggaaaggtaatcatagtagcagtgcat
gtagctagtggattcatagaggcagaagtaataccacaggaaacaggaagacagacagcattgttcctgttgaaa
ttggcaagcagatggcccatcacacatttgcatacagacaatggtgccaatttcacctcgcaagaggtgaagatg
gtagcctggtgggcaggcatagaacagacctttggggtgccttataatccacagagccaaggggtagtagaagca
atgaatcatcacttaaaaactcaaatagacagaatcagagaacaagcaaactcagtagaaacaatagtactcatg
gcagttcattgcatgaattttaaaagaaggggaggaataggggatatgaccccagcagaaagattaatcaatatg
attaccacagaacaagaaatacaattccaacaatcaaaaaattcaaaatttaaaaattttcgggtctattacaga
gagggcagagaccaactgtggaaaggacccggtgagctattgtggaaaggggaaggagcagtcatcataaaggta
gggacagacattaaagtagtaccaaggagaaaagcaaaaattatcaaagac
>#14 HM803689 2754 nt
atggtcaatatagaagggcaaaaggtagaagccctcttagatacaggggcagatgacacagtaattaatgggata
gaattaggagaaaattggaagccaaaaattataggggggtattggtggatatataaatgtgaaacaatattacaat
tgtaagatagaaatagcagggaaagtaacacatgcccatgtattggtagggccaacacctgtaaatattattgga
agaaatgttttacacaaattaggagcctctttaaattttccaattagcaaggcagaaacaattgaagtgaaattg
aaaccaggacaagatgggcctaaagtaaaacaatggcctttaagcaaagaaaagatagaagcgttaacagaaatt
tgtaatgctatggaaaaagaaggaaaaatatccaaaatagggccagaaaatccttacaatacgcctatttttgc
attaggaaaaaggattctacaaaatggagaaaattggtagatttcagagaattaaataagagaactcaggacttc
tttgaggtacagctgggaataccacatcctggggggtttgaaaaaatgtaaacagataacagttttggacataggg
gatgcttatttttcatgtcctctatatgaacctttagaaaatatactgcattcacaataccagcaatcaataat
cagggaccaggagttaggtatcaatacaatgtgctcccacagggatggaagggatctccagctatatttcaaaca
actgcaaatagaatcttagagccattcagaaaagaaaatccagatgtagtaatctatcaatacatggatgattta
tttgtaggatctgataggacagaattagaacataatcagatgataaaaagactcagagaacatttgttgatctgg
gggtttgaaaccccagataaaaaatttcaggacaaacctccctttgaatggatggggtatgtactccatccagac
aaatggacagtacaagaaattaaattacctgagaaagaggaatggacagtaaatgatattcagaaattagtggga
aaattaaactgggccagtcaaatctatacaggaataaagacaaaagaattatgtaaattaattagaggagtaaaa
aggctagatgaaaaggtagaattcaccagagaagcagaactagaatatgaggaaaacaagctattgctgaaagaa
caaatgcatggagtctactatgatccagaaaaacctctgatagctaaagtacaaaagttaacaggagggcagtgg
tcttatcaggtagaacaaggagatggtaaaccattaaagacagggaaatatgccaagcaaaagactgcacacacc
aatgagatcaggatgctagcaggattggtacagaaaatatgtaaagaagcagtagtcatttggggaaaattgccc
aaatttgagctgccaatagagagagaggtatgggaacaatggtgggctgattattggcaggtatcatggattcct
gagtgggaattcgtgagcacgcccccattaattaggttatggtataatctggtaaaagatcccataccaggggaa
gaagtctattatgtagacggggcagctaacagaaatagcaaacagggaaaagcaggatacattacagacagagga
agagaaaaggtaaaagaattagaagataccactaatcaaaaggcagaattggaggcagtcttattagcattaaaa
gattcagggaaaaaggtaaatatagtaacagattcacagtatgtgtttggcatattggcaggaagtccagacact
agtgatagtcctcttgtacagcagatcatagaacaattaatagggaaagaggaagtgtacctctcctgggtacct
gcacataagggcataggaggaaatgaggaagtagacaaattagtaagtaaaggaattagacaagtcctcttttta
gatggaatagaaaaggctcaagaagagcatgaaaaatatcataataattggagagcaatggcagaagattttcag
ataccacaagtagtagcaaaggagatagtagcccaatgccctaaatgtcaggtgaaaggagaagcaatgcatgga
caggtagatgccagtccaaaaacatggcagatggactgcactcatttagaaggaaaggtcatcatagtagcagtc
catgtagccagtgggtatatagaagcagaagttttaccagcagaaacaggaaaagaaacagctcacttcctgcta
aagctagcagccagatggccagtaaaacatttgcacacagacaatggagcaaattttacgagctcagcagtacaa
gcagtttgttggtgggctcagatagagcacaccttcggggtaccctacaacccacaaagtcaaggagtagtagag
tccatgaatcaccagctaaaaactataataacacaaataagagatcaagcagaaaagatagagacagcagtacaa
atggcagtgctcattcacaattttaaaagaaaaggggggattgggggtacagtgcaggagaaagaataatagat
ataatagcatcagacttacaaacaactaaattacaaaatcaaatttcaaaaattcaaaattttcgggtttatttc
agagaaggaagggatcaacagtggaaaggacctgccactctaatctggaaaggagaaggagcggtagtaatccag
gacggacaagatttaaaggtagtcccaagaagaaagtgtaaaatcataaaagat
>#15 HM803690 2754 nt

tgggtcaccatagagggtcaaaaagtagaagccctttttagacacaggtgctgatgatacagtcatcagtgacata
gaattaggagataattggaagccaaaaattataggaggaatagggggatatataaatgtaaaacagtattataat
tgtaaaatagaaatagcagggaaagttacacatgctcatgtattagtaggtccaacaccggtaaatataattggg
agaaatgtgttacacaaattaggagcttcattaaattttccaataagtaaagcagaaataattaaagtagagtta
aaggaaggtcaggatggaccaagagtaaagcagtggcccctcagtaaagaaaaaatagaagccttaacagaaata
tgcaatgcaatggaaaaggagggaaaaattagtagaataggaccagagaatccatataatactccaatattttgt
attagaaagaaagattcgaccaaatggagaaagctagtagatttcagagaactaaataagaggacacaggatttc
tttgaggtacagctggggattccacatccaggaggattgaagaaatgcaagcagattactgtgctggacataggg
gacgcatattttcctgcccctctatgagcccttcagaaagtatacagcttttactataccagcaataaataat
caagggccaggtatcagatatcaatacaatgtgcttcctcagggatggaagggctccccagccatatttcaaacc
actgctaacaaaattttagaaccagtcaggaaacaaaatccagatttagtcatctatcagtatatggatgacctg
tttgtaggatcagacagaacagaattagaacacagtcaaatgataaagaaattgagggaacatttgctcgcctgg
ggatttgagacaccagaaaagaaatttcaagataaaccaccatttgaatggatggggtatgtactccacccagac
aagtggacagtgcaggaaattaagttgccagaaaaggaagaatggacagtgaatgacatacagaagttggtaggc
aaattgaactgggcaagtcagatctatgcaggaatcaaaactaaagaattgtgcaaattaattagaggaataaaa
agattagatgataaggtagaatttacaaaagaagcagaattagagtatgaagaaaataaactgctattaaaagaa
aaattgcatggggtatattatgacccagaaaaaccattgatagcaaaagtacagaaattagaaggaggacagtgg
tcatatcaggtagaacaaggagatggaaaacctctaaaaacaggaaaatatgccaagcagaagacagcacatacc
aatgagattagaatgctggcagggttagttcagaaaatttgtaaagaagcaatagttatttggggaaaattgcct
aaatttgaattgcctgtagaaagagaggtatgggaacaatggtgggctgattattggcaagtctcatggattcca
gaatgggaatttgtgagcacgccgcctttaattaggctgtggtataacctagtaaaggatcccataccaggagag
gaggtgtattatgtggatggggcagcaaacagaaatagtaaattaggaaaagcaggatacataacagatagagga
aaagaaaaagtaaaagaattagaagataccaccaatcagaaggcagaattagaggctgtacttttagctttaaaa
gattcaggaaaaagagtaaatatagtaacagactctcaatatgtgtttggcatattagcaggaagtccagacact
agtgagagtcccttagtacagcagataatagagcaactaatagggaaagaagaagtgtacctctcctgggtaccg
gcacataagggcataggaggaaatgaagaggtagataaattagtgagcagaggaattagacaagtattattcctt
gatggcatagaaaaagcacaagaagaacatgataaatatcatagcaattggagagctatggcagaagatttcaga
atacctcaagtagtagctaaggaaatagtggcccaatgtcctaagtgccaggtgaaaggggaggcagtccatggg
caaacagatgctagtccagggacatggcaaatggattgtactcatttggaaggaaaggtaatcatagtggcagtc
catgtagccagtggatatatagaagcagaagtcataccagcagaaacaggaaaagagacagcatacttcctgtta
aaattagcagcaagatggccagtgaaacacttacatacagataatggagctaatttcacaagtgcagcagtacag
gcagtctgttggtgggctcagatagagcacacctttggagtaccctacaatcctcaaagtcagggagtggttgaa
agtatgaatcatcaattgaagacaataatagggcaaattagagaccaagcagagaaattagaaacagcagttcaa
atggcagtgctcattcacaattttaaaagaaaggggggattggggggtacagtgcaggagaaagaataatagac
ataatagcatcagagttacaaacaaataaattacaaaatcaaatttcaaaaattcaaaattttcgggtttatttt
agagaaggcagggatcagcagtggaagggaccggccactctcatctggaaaggagaaggagcagtggtgatccag
gatggacaggatctgaaggtagtccctagaagaaagtgtaaaattgtaaaagat
>#16 M58410 2772 nt
cgagtcatcatagaaggaacgccagtgcaagccttgttagatacaggagcagatgacactataattcaagaaaag
gacttgcactttcccccacataaaccatggcgttccaaggtagtaggaggtataggaggagggattcatgtcaaa
gaatatcagggggtacaagtacaattggaggataaaatcatcaccggctcaattctaataggaagtacaccaatc
aatattataggaagaaatattttagctcaggcaggcatgaaattagttatgggagttctatctagtcagattgag
gaaacaaaagtacaactaaaagaagggaaagatggacctaaattgaaacaatggcccttatcaagagaaaaaatt
gaagctttaacagaaatatgcaaacaaatggaagaggagggaaaattatctaggataggaggagaaaatccttat
aatacaccagtgtttgccataaagaaaaaggataaaacacaatggagaatgcttgtagatttcagggaactaaac
aaagctactcaagactttttgaggttcagctgggaattcctcacccagcgggccttcagaaaaagaagcaaatc
acagtaatagacatagggatgcctattattcaataccattatgcaaggaattcagaaaatatacagcatttacc
atcccctcagtaaataatacagggccagggataaggtatcagttcaattgtctgcctcagggatggaaaggatct
cctacaattttccagaatacggcagcaaacattttagaggagatcaaaaggcacactcctgggttagaaattgtc
caatacatggacgatttgtggttggcgtcagaccatgatgagactagacataatcaacaggtagacatagtaaga
aagatgctgctagaaaaaggtctagaaaccccagacaagaaagtccaaagagaaccgccatgggaatggatgggg
tataaattgcatccgaataaatggaccattaacaaaatagaattaccccccttagaaggagaatggacagtaaac
aaaatacagaaggtagtaggagttctaaattgggcaagtcaaatttatccaggaattaaaaccaaacatacctgt
gccatgttgagagggaaaaagaacctcctagaagaaatagtatggacagaagaggcagaggcagaatataagaac
aatcaaggatagtgcaggaaacacaagaaggaacatactatgaccctctcaaagaattaatagcaacagttcaa

aagcaaggagaagggcaatggacataccaattcacccaagaaggggcagtattaaaggtgggaagatatgccaag
caaagagaaactcatactaatgatctaaggactctagcacaccttgtccaaaaaatctgtaaggaagcacttacc
atttggggaagacttccacgagtacaactcccagtagacaagaaaacatgggatatgtggtggcaggactattgg
caagtatcctggataccagaatgggagtttgttagcacaccactcctagtaaaactgtggtattccttagtaaaa
gaaccaatcaaaggagaagatgtttattatgtggatggggcagcatccaaagtgaccaaattaggtaaggcagga
tatctgtcagagagaggaaaaagtagaattagggaattagaaaacaccactaaccaacaagcagaattaacagca
gttaagatggcattggaggacagtggagaaaatgtaaatatagtcacagattctcaatatgtaatgaacatcttg
acagcatgtccacaggaaagtaactcacccttagtggaacagataatacaagccctaatgaaaaagaggcaggtc
tacttacaatgggtaccagctcataaggggataggaggcaatacagaaatagataaattagtaagcaaaggaata
agacagatcctcttcttagatagaatagaagaagcacaagatgaccatgcaaagtaccataacaattggagaagt
atggtacaggaatttggattacctaatatagtagcaaaagagatagtagcggcatgtcccaaatgccaaataaga
ggagaacctaagcatggacaggtagacgcctccattgaaacttggcagatggactgcacccatttagaaggaaaa
gttataatagtagcagtacatgtagccagtggattcatagaagcagaggtgatcccaagagaaactgggaaggag
acagcacactttctgctgaaactgttagcaagatggccagtgaaacatctacacactgataatggcccaaacttt
acctctcagaatgtggcagcggtgtgctggtggggtaatatagagcacaccactggaataccttataacccacag
tcacagggtagtgtagaaagcatgaacagacagctcaaggaaatcatctctcaaataagagatgattgtgagaga
ttggagacagcagtgcaaatggctacgcatatccacaattttaaaagaaagggaggaataggggggtatctctagt
gcagaaagattggttaatatgctaacaacacaactagaactaaatactctacaaaaccaaatccaaaaaattttg
aattttaaggtctactacagagaaggtagagatccagtgtggaaaggaccagcgcgactcatctggaaaggagaa
ggcgcggtggtaattaaagaggggggaagacatcaaggtagtccccaggagaaaggctaagattatcaaagat
>#17 LC114462 2766 nt
attgttagaatagagggtcagccaatcaaagtcttgttagatacaggagcagatgataccatcataaaagaacag
gacttagcattaacgggatcatggcgacccaaaatcataggaggaataggaggaggtttacaagttagggagtat
aataatgtagaagtaatattagaagacaaaacattaaaggggcagtcttattaggaccaacacctataaatatt
ataggaagaaatttttttagcccaggcaggagtcaaacttgttatggggcaattatcagaagcaatccccataact
ccagtcagactaaaagaaggggccaaaggaccttgtgtaaagcaatggcctctatctagggaaaagataaaagct
ttgacagatatttgtgactccttagaaaaagagggaaaaatatcaagaataggaggggaaaatgcatataacacc
ccagtattctgcataaaaaagaaggatcaatcgaaatggagaatgctaatagactttagggagctaaataaggcc
acccaggacttctttgaagtccagctaggaatacctcatccctcagggctacagaaaaagaaacaagtaactgtt
ttagacataggagatgcttattacagcattcctctagatccagagtttagaaaatatacagcctttactattcct
agcataaacaatcagggacccggacagaggtatcaatttaattgtttgccacaaggatggaaaggatcccccaca
atttttcagaatacagcagcaaaaatactggcccagattaaagaagaattaccggggctagaaattgtccagtat
atggatgacctctgggtagggtcaaactactcagaaagagaacatgatgaaaaagttagacagcttagagagaaa
ctattacagtggggctttgagaccccagaaaagaaagttcaatcagaacctccctatgaatggatgggatataaa
ctttggccaagcaaatggcagttacaaaagttagagatagaagaaaaagatcaatggacagtaaatgatattcag
aaattggcaggaaaacttaactgggcagcacaactatatccagggcttaaaaccaaacacatttgccgcctcatt
aggggaaaaaggggattgttagatccagtcctatggactcctgaggcagaactagaacttgaagagaatagaagc
attcttaagacagaacaagaaggggcatattatgacccacaaaagcccctaagagcagcagtccaaaagttagga
aatggacagtggggatatcaaataaaacaggagcaaaaggtattaaaagtaggaaaatatgagaaacagaaagca
atacatactaatgaacttagaatattagcaggacttgtgcaaaagatagctaaagagagcctagttatctgggga
accttacccaaatttgagttaccagtagaaagggaggtatgggaacaatggtggagtgattattggcaagtctct
tggataccagaatggaattcgtcagtgtcccacctctagtaaaattgtggtactctctggtcaaagatccaatt
ccaaaagaagatgtctattatgtagatggggcctgtaatagacaatcaaaagaaggaaaagcaggttatatcact
caatatggaaaacaaagagtcaaaactcttgagaatactactaatcaacaggcagaactgcaggctatcctcatg
gctctagaggatagcgggtctaaagttaatatagtaacagactcccaatatgcgttagggatcttgacagctcac
cccacacagagtgattccgccttagtaaatcaaataattgagcaattaatcaagaaggagcaagtataccttcaa
tgggtcccagcacataaaggcataggaggaaatgaagaggtagacaaattagtcagtaaaggaattaggcaagtg
ttattcttagaaaggatagaagaagcacaggaagaacatgagaggtaccataacaattggaaggaaatggcggac
acatttggcatcccacaagtagtggctaaagaaatagtagcagcatgtcctaaatgccaagtcaaaggagagccc
gttcatggccaggtagacgcgtccccaggggtctggcagatagactgtacccacatagaaggacacatcatcata
gtagctgtccatgtggccagtggcttcatagaagcagaggaaataccaagggaaacaggaaaggctacagccaaa
ttcctattaaagttactatcaagatggccagtaaaacagattcacactgacaatggaccaaattttgtatcacaa
gaagtcaaagctatttgttggtgggaaaaatagaacatacaacaggagtaccctataatcctcaatctcaaggg
tcagtagaatctatgaataaacaattgaaagaaataataggaaaaattagggatgactgccaatatgtcgaaaca
gcagtattaatggcatgccacattcacaattttaaaagaaagggaggaataggggggtatgaccagtgcagaaaga

ctactaagtatactaactgcacaaatagaaacacaaaccttacaacaaaaaatccaaaaaattttgaattttaga
gtctactacagagaaggaagagaccctgtgtggaagggaccagcacagctcatttggaaaggagaaggagcagtg
gtaataaaagaagcagacggagacttgaaagtaataccaagaagaaaggctaagattattaaagac
>#18 KR862336 2760 nt
gaaattctcataaatggcctcccagtcatggtccttttagatacaggggcagatgatacaattattaaagaaaca
gatattaatttggcaggaccatggcggcccaaattaataggaggcattgggggaggcttaagagtaaaagaatat
aacaatgttacagtacaaatagaggacaaaacattgattggcacgatcttaatagggccaaccccaattaatata
ataggaagaaacttttggctagtgcagggatgaaattagtaatgggacaattaagcgataaaatacccattaca
cctgctaaattaaaggagggagccaaaggtcccaaaataaagcaatggccactctcagcagaaaaaatcaaagcc
ctcacggacatatgtgaggacatggaaaaggaaggaaaactaaccagaataggaggagaaaatccttataacacc
ccaatcttctgtattaagaaaaaggatacctcacaatggaggatgttagtagatttagggaattaaataagatg
acccaagactttttgaggtacaattagggatcccacacccagcgggactgaggaaaatgaaacagatcacagtg
ctagacataggcaatgcctattacagtgtaccattagaccctgacttccgcaaatatactgcatttactatacct
gcaattaataatgagggaccagggaaaaggtaccaattcaactgtctcccgcagggatggaaaggatcaccaacc
atctttcaaaatacggcagcagggattctagagaaaattagggaagagttaaaagagttaactattatccagtat
atggatgatttgtgggtaggatccaattgtggggaaaggaagcatgatgaactgatagagcgactaaggacagag
cttctagcctggggatttgaaaccccagacaaaaaggtacagaaacaacctccctttgaatggatggggtataaa
ttatatcctcagaaatgggtagtacaacccatagaattagaagagaaagaacagtggacagttaatgatattcag
aaactagtaggaaaactaaattgggctgcacaaatatatccagggttaaaaactaaaagaatttgtcaattaata
aaaggaaagaaaaatttgttagaccctgtagtctggacagatgaagcagaggcagaatatgaagaaaataagatt
attctaaaaacagaacaagaagggacatattatgatccagaaaaacctctaaaggtagcagtgcaaaagctggga
gatggacaatggggatatcagttcaaacaagacaaaatcttaaagacagggaaattcatgaaacagaggaccact
catagtaatgagctaagaattttggcaggattggtacaaaagatagccaaagaaagcctagtaacatggggaata
ctccctaagtttgaactcccagtagaaagggaggtatgggaacaatggtgggcggagtattggcaagttggctgg
atacctgaatgggaatttgtcagtgtaccaccattggtcaccttatggtaccgcctcaccaaagaccccatacct
aaacaagatgtctactatgtagatggagcctgtaacagaaactccaaaatgggcaaagcaggatatatcactcaa
tatgggaaacagagagtgaaagaattggaaaatactaccaaccaacaggcagaattaacagctatccttatggca
ctacaagatagtggagatcaagtaaacatagtaacagactcccagtatgccttagggatcatatcaagtcacccc
acacaaagtgactcgcctctggtagaacagatcatccaagaattagtgaagaaaaaggcggtatacctacaatgg
gtaccagcacacaaaggcatagggggaaatgaagaggttgataaactagtcagcaaaggaataagacaagtacta
ttcatagaaaaaatagaagaggcccaagaagaacacgaaagatatcacaacaattggagagaaatggcagataca
tttggtattccccaagtagtagccaaagaaatagtagctcagtgccctaagtgccaaataaaaggagagcctata
catggacaagtggatgcttcaccaggagtgtggcagatggactgtacccatatggaaggacatgtgatcatcaca
gccgtacatgtagccagtgggttcatagaagcagaaataatacctagagaaacaggaaaagagacagcaaagttc
ctgttaaaaattctctccagatggccaataacaaagctacacacagacaatggccctaacttcatttcacaggaa
gtgaaggcaatgtgttggtggggaaaaatagagcacacaacaggagttccctataatccacaatcacaaggtca
gtggaaagtatgaataaacaattaaaggaaatcataggcaaaattagagatgactgtcaatacctagaaacagca
gtattaatggcctgccacatccacaattttaaaagaaagggaggaataggggatatgactagtgcagatagactc
ctgaacatcctaaccacacaaatagaaacaaaacaactacaacaaaaaattcaaaaacttttgaatttcaaggtg
tacttcagagaagggagagacccaatttggagaggcccagcaacactcatctggaaaggagaaggagcagtggta
attaaagaccaaggagaactaaaggtagtcccaagaagaaaagcaaagatcataaaagat
>#19 KR862356 2766 nt
acagtgtatattaacggcctccccattaaggctctcttagacacaggagcagatgatactatcataaaagagaca
gatatacaattagcaggacattggagaccaaagttaataggggggaataggaggaggccttaatgtaaaggaatat
ttaaatgtcaccctaaatttagaaggaaaagagttaatagggaccattttagtaggagaaacacctatcaatatt
ataggaagaaacttcttagccaatgctggtatgagattggtaatgggacaattatctgatgctatacccattacc
aaagtagctctaaaagaaggagctaaaggaccgtgtataaaacaatggccactttcaaggaaaaaatagaagcc
ctacagcaaatatgcacacaaatggaaaaggagggaaaattaaccaagataggggggagagattgcatataacacc
cctgtatttgcaataagaaagaaagacaaaacacaatggaggatgctaatagatttagggaattaaataaagta
acacaagacttctttgaggtccagttggggataccacatccaggaggccttcataaaaagaagcaaataacagtt
ttggatataggggatgcttattatagcattccattggacccggatttcagaaaattcactgcttttacgatacct
agtgtaaacaaccagggtccagggactagatacatatttaactgcctcccgcaaggatggaagggatccccaact
atttttcaaaatactgcagcaaagatattagaagaaataaaaacagaacttcctgctcttacaattattcagtat
atggatgatatgtgggtaggatcagatttaacaccatatgagcatgataaacagatcacaatactgagagaaagg
ctatctcaatggggcttggaaactccagaaaagaagctccaaaaggaacccccgtattcatggatgggatataag

ttgtggccacagaaatggcagctacagcccatagagctaccaaaccctgaggaatggactgtaaatgacatacaa
aaattagtagggatgctaaactgggctgcacaaatttacgcaggtcttaaaactaaacaaatatgcaaactgatc
agagggaagaaagacctgttggaaaaggtaacatggactcaagaagctgaaatagagctagcagaaaatagagaa
attctcaaaaccacacaggagggagcatactatgaccctacaaaaagtcttagggcaacaatacagaaattgcca
gatggacaatggagttatcaaataaaacaagaaggaaaccaaattttgaggacaggaaaatacatgaaggctaaa
gctacacatacaaatgacttaagagtactggccggtctagtacagaaaattgctaaggagagtataacttgttgg
gggactttacccgtctttgagttaccagtagaaagggaagtatgggaacagtggtggagtgattattggcaagtc
agctggataccagaatgggaatttgttagtgttccaccattagtcaccctatggtataggctaacgaaagatcct
atacccaaggtggacacctattatgtagatggatcctgcaacaggaatagcaaggaaggaaaggccggatatatc
acacaacaggggaagcaaaggattaggaccttagaaaataccaccaaccaacaagcagaattacaagcagtattg
atggccctagaagatagtggaaaacaggttaacataataacagattcccaatatgtgttaggggttatatcgtca
catcccacgcaaagtgactcgccactggtagaacaaattattgctgcattaattcaaaaggaagcggtgtattta
aactgggtaccagctcacaaagggataggaggaaatgaggaaatagacaaattagtgagccaagggattagacag
gtcctcttcctagaaaaaatagaagaagcacaagaggaacacgaacggtaccacaacaattggaaggagatggca
gatacctttggaatacctcaagtagtagcaaaagaaatagtagcacaatgtccaaaatgtcaaataagaggggaa
ccaatacatgggcaagtagatgcctcaccgggggtttggcaaatggactgtactcatctagaaggaaaaattatt
ataacagcagtacatgttgcaagtggctttatagaagcagaaattatacctagagaaacaggaaaggaaacagca
aaatttttactcaagctactttctagatggcctatcacaaaactacacacagataatgggcccaactttgtgtcg
caagaagtacaagcaatgtgttggtgggggaaaatagagcacacaacaggtataccatataaccctcaatcacaa
ggttcggtagagtctatgaatagacagttaaaggacattattggaaaaatcagggaagactgccaatacttagaa
acagcagtactcatggcctgccacattcataattttaaaagaaagggaggaataggggatatgactagtgctgat
agattactaaacatattaaccacccaactagaaaccaaaactttacaacaaaaaattcaaaaattcttgaatttc
aaggtctactacagagaaggccgtgatcctgtgtggaaaggaccagccgtgctcatttggaaaggtgaaggggca
gtggtgatcaaagaacagggagaactgaaagtcatacccagaagaaaggctaagattattaaggat
>#20 KR862363 2766 nt
accatacatataaatggcctcccaataaaagtacttcttgatacaggagcagatgacactattatcaaagaaaca
gacattcaattacaaggttcatggagacccaaattaataggaggtataggggggaggtattaatgtaaaagaatat
aataatgtagtagtagaaatagaagggaaagaactaattggcactgttttagtaggagaaactcctattaacatt
atcggaagaaatttcttagctaacgcagggatgaaattagtaatgggagcattatctacaaatataccaatcaca
aaagtgaatttgaaagaaggagcaaaaggaccttgcttaaaacaatggccactgtctaaagaaaaaattctagca
ttacaacaaatttgtcaggaaatggaaaaagaaggaaaattgactaagattggaggggaaaatgcctacaacaca
ccagtatttgcaataaggaagaaagacaaaacacagtggcgaatgttaatagactttagggagctaaataaagtc
actcaagacttctttgaagtgcaattaggaataccacatccgggggggattaaaaaagaagaaacaaatcactgtg
ctagatataggcgatgcctattatagcatccccctggatccagactttagaaaatacacagccttcactatacca
agcataaataatgaaggaccaggcactaggtatcaatttaattgtctaccacaaggatggaaaggctcacccacc
atcttccaaaatacagcagcaaagatattggaagaaataaaggcagacttacctgcattaactatcattcaatat
atggatgatttatgggtaggatctgacttaaccccatatgaacatgataaacaaattgaaaccctcagacagaga
ttaggacggtggggactagaaactcccgagaagaaattacagaaacaacctccctatgaatggatggggtacaaa
ctatggcctcataaatggcagttacaacctatagaattaaaagagccagaagaatggacagttaatgacattcag
agagtggtaggacaattaaattgggcagcacaattgtacccaggattaaaaacaaagaacatttgtaaactgata
agaggaaaaaagaacctttggaaccagtacaatggaccccggaagcagaggtagaatatgcagagaataaagag
attctcaaaacttcacaggagggagcctattatgacccctcaaaaaatctcaaagtagcagtacagaagcttcca
gaaggacaatggagctatcagtttaaacaagaaggaggaaacattctaaaaacaggaaaatacatgaaaaataaa
gcaactcacactaatgatcttagagtattggcaggattagttcaaaaaatagctaaagaaagcatagttatctgg
ggacaattacccattttgaattacctgtagaaaaagagacttgggagcaatggtggagtgactactggcaagta
gggtggataccagattgggaatttgtcagtgttcctccattagttacactttggtatcgactgacgaaagacccc
atcccaaaggaagatgtttattatgtagatgggtcatgcaacagagaaagtaagttaggcaaagcaggatacatc
actcagcaaggaaagcagaaagtcatcactttggaaaacacaacaaaccaacaggcagaattgcatgcagtactg
ctggccttacaagacagtggaaaaagggtaaacattgtgacagattcacaatatgttttaggtatcatctcttca
caccccacacagagtgattcaccactggtagaacaaataatagcccagttgattcaaaaggagttagtatattta
aattgggtgccagcccataaaggtataggggaaatgaggaaatagacaaattagtaagtcaaggaattaggcaa
gtactgttcctagaaaaaatagagcatgcacaggaagaacatgaaagataccataacaattggaaggaaatggcc
gacacatttgggataccacaggtggtagcaaaagaaatagtagcacagtgccccaagtgccagataaggggagaa
ccaatacatgggcaaattgatgcctcaccaggtgtgtggcaaatggattgtacccacttagaaggaaaaataatt
atcacagcagtccacgttgccagtgggttcatagaagcagaggtcataccaagggagacaggaaaagaaaccgcc

aaattccttctaaaaataatatcaaggtggcctatcacaaagttacacacagataatggcccaaattttgtatca
caggaagtacaagccatgtgttggtggggaaaggttgagcatacaacaggagtaccttacaatccacaatcacaa
ggttcagtagaatcaatgaatagacaattaaaagagattattggcaaaatcagagatgactgtcaattcttggaa
acagcagtacttatggcctgccacattcataattttaaaagaaagggaggagtaggggatatgactagtgcagaa
agattattgaatatgttaaccacacaattagaaatcaatcacttacaaaaacaacaacaaaaattttcaaatttc
aaggtctacttcagggaaggcagagatcctgtctggaaaggacccgcaacactcatctggaagggagaaggagca
gtggtaataaaagaccaagaagaattaaaagtagtcccaaggaggaaagcaaaaattataaaagat
>#21 M29975 2763 nt
acctgtataataggaggaactgccgttaaggcattattagatacaggggcagatgacactataataaaggataca
gatttacaattaaggggatcatggagaccaaaaatagtaggaggaattggggggagggttaaacgtaaaagaatat
gataatgtagaagtacaattggaagacaagatattaagaggaacagtcctcataggagcaactcccatcaatatc
ataggaagaaactttttagcccaggcaggagccaaattagtgatggggcaattgtcgcagacaataccaatcacc
ccggtacgcttaaaggaaggggccagaggaccacgattgaagcaatggccactctctaaagaaaaaataatagcc
ctgcaagaaatttgcaaaacattagaggaagaaggaaaattaagcagggtaggggggagacaatgcatacaataca
ccagtattctgtataaggaaaaaagacaaatcacagtggagaatgctggtagatttcagggaactcaacaaagct
acacaagacttctttgaagtccaattaggtataccccatccagcagggttaaagaaaatgaagcaaataaccatt
atagatgtgggggatgcatattatagcataccactggatcctgagtttagaaaatacacagctttcaccatccct
acggtaaacaatgagggaccaggcataagatatcaatttaattgcctaccgcagggctggaagggatccccgaca
attttccaaaacacagcatcaaaaattctagaagaaataaagaaagaattaaaacagctgacgattgtccagtac
atggatgacctctgggtaggatcacaagaagagggtccaaagcatgatcagctagtacaaacacttaggaataga
ttgcaagaatggggattagaaacaccagagaaaaaggtgcaaagagaacctccctttgagtggatgggatataaa
ttatggcctcataaatggaagttacaaagtatagaattagagaagaaagaacaatggacagtgaatgatcttcag
aaattggtagggaaattaaattgggcagcacaattatatccaggattgagaacaaaaaatatctgtaagctactt
agaggaaagaaaaatttattagacgtggtagaatggaccccagaggcagaagcagagtacgaagaaaacaaggag
atcctaaaaacagagcaagaaggtacttattatgcaccagaaaaaccccttagggcagcagtacagaaattagga
gatgggcaatggtcataccaattcaagcaggaaggaaaaatcttaaaggtagggaagttcgccaaacagaaagct
actcacaccaatgagttgcgtgtactagcaggagtagtacagaaaatagggaaagaggccctagtaatttgggga
caattacccacttttgaactcccagtggagagggacacatgggaacaatggtgggcagactattggcaagtcagt
tggatacccgaatgggactttgtcagtgttccgcccttagtaactttgtggtatacactgactaaggaacccatc
ccgggagaggatgtctactatgtagatggagcctgtaatagacagtcgaaagagggaaaagcaggctacataacc
caacaaggcaaacaaagagtacaacagctagaaaacacaacaaatcaacaagctgaactgacagccataaaaatg
gccttggaggatagcggccctaaagtcaatatagtaacagattcacaatatgcgatgggcatattgacagcacag
cccacacagagtgactccccactagtagaacaaataatagcacagatggtacagaaagaagccatctatctgcaa
tgggtacctgctcataaaggtatagggggcaatgaagaaatagacaaattagtaagcaagggagttagaagaata
ttgttcattggcaggatagaagaagcacaagaagaacatgataggtatcacagtaactggagaaatctagcagac
acatttggattgccacaaatagtagctaaagaaattgtagcaatgtgcccaaaatgtcaagtaaaaggggaacca
atacatggacaagtagatgcttcaccaggagtgtggcagatggactgcacacatatagaaggaaaaatagtgata
gtagcggtccatgtagccagtgggtttatagaagcagaggttatccctagggaaacaggaaaagagacagcaaag
ttcttgttaaaaataataggaagatggcccatcactcacctccatacagataatggaccaaatttcacttctcag
gaagtagctgctatgtgctggtggggaaaggtagaacacacaacgggggtaccatataatccacagtcccaggga
tctatagaaagtatgaacaaacaattgaaagagataattggaaaaataagagatgactgtcaatatacagaaaca
gcagtacttatggcctgccacattcacaattttaaaagaaaggaggaataggggggctaacagctgcagagaga
ctaataaatatgataacaacacaattagaaatcaacactctacaaaccaaaatccaaaaaattttgaattttaga
gtctactacagagaaggcagagatccagtgtggaagggacctgctcgcctgatctggaaaggagaaggcgcggta
gttctcaaggaaggtgaagaactgaaggtagttccgagaaggaaagcaaaaatcataaaagac
>#22 AF301156 2790 nt
atagtagaagtagaaggacaaaagtgccaagcgctgttagatacaggagcagatgatacagtttttaaagaagaa
gatgttcaattaacaggcgcttggacagcagtacaaattcagggaataggggggagcaatcagagtaaaacagtat
agagataaaattctaaagatgggaggaaaagaatacagaggagatatcttaataggacacaccccccataaatata
gtaggtagaaatatgttaaaagaagggaaattagtagtagctcaattatcagataggatcccagtcaccaaggtg
acgttaaaagagggcatggatgggccaaaagtaaagcagtggcccttaagcaaagaaaaaatagaaggtttacaa
aagatatgtgaaagattggaagcagagggaaaaatagaaaaagcagaattagggaatccatataatacaccaata
ttttgcattaggaaaaaagataaaaatgaatggaggaaattaatagatttcagggagctaaacaagagaacacaa
gacttcatggaagtccaattagggataccacacccaggaggtcttatagaaaagacccatataacagtattagat
ataggagatgcctatttctcaattcccttggatccggattatcaaaagtacacagcttttacaatcccatcagtg

aacaatttggcaccagggactagatatgtgtacaaagtgctaccgcagggttggaaagggtctccaacaatcttc
caaggaacagtagcaagaatcctagaaccgttcaggaagagaggacaattgcaaatagctcagtacatggatgat
ttatatctaggatcagatctgcctttagaggagcatagaagggcagtacaggagctcagagatgcactcttgtac
tatgggttagagacaccagaaaagaaataccaggcagatcctccatacaagtggatgggctatgaattacatcct
aagcaatggaaattacaaaaggtcaaaattccagaacaggataagtggacagtaaatgatatacaaaaattggta
ggagccttaaattggttaagccaattatacccaggaatcaggacaaaggagataagtaaatgcactaaaggtaag
aagcatttgttggaggaagtgcagcttagtgcagaagcagaagaggagctacaagataacaaacagataatcaaa
gaagaagttcagggagtgtactatataccacatgaggacatttgggtagacattttcaggttagggaaaggacaa
tggggctatgcagtctgccaaaaacatggaacgctaagaagaggaaaacataacacaggcaaaaatcagcacttt
aatagtatgcaggagttagcttcagttatacaaaagataggtagggaagccatagtcacctggggaaaggtgccc
aaaatgaaagtcccagcaaagagagaggattgggaacagtggtggagtgactattggcaaagtgcttggatccca
gagatagagtttgtctcatcctcttatgtagtaaaattagtatggaacttggttttagagcctctgaaggaggca
ccaacttattggacagatggaggatgctccaggaaattaggagcgggaaaggcaggatggataaattcaaaagga
gaagaagaagtagtcaatcttcatgaagggtcaaatcagcaagcagagctgacaggagttttgctagctctaaaa
catgggccaaaaagaatgaatttggtaacagacagtctttatgcattagggataattactgggcagccatatgat
cagcttccatctagtagaggggagattattcaggcaggtatggctaaagaagccattcatgtagcctggtgtcca
gcacataaagggataggaggaaatgaactgatagatcaaaaggtaggagtaagacaggtaatgtggatagataaa
atagaagcagcagaagaagaccatcaaaagtttcatagtaatgtgcagtatctaaaagaacaatttgggttaccc
actgtagtagcaaaagaaatttgggaaagatgttcagagtgtcagaacaaaggacaagctgttcatggccagcta
gattatagctatggcttatggcaattagattgtactcatgaggaaggaaaggttatcttagtagcagtacacgtc
tgtactctgttctgctgggcaaccatattaaagagagaaacaggggaagaaacaggtagggccctcataaaattg
gctagtcagtgggaggttagacaagttcacacagataatgggccaaattttgtaagtcagcactttaaggcagca
gtgtggtggctaggaatagcacacaccacaggacacccctataatcctcaatcacaaggagttgtagagcagaga
aacaaggatgttaaaagaaagattaagaaaatgaaagatcaagcagaaacattagaaagcaaagtagcaatggca
gtctatgcgctcaattttaaaagaaagggaggattagggggaagagtccatgggaaagacaagtagaaagagca
ataatagaattagatacacaaaacctaacaaaattacaaaatcaaaaatttaaaaatttaaggcctactggaaa
gagcacacaggagagtggcaaggaccaggagaactggtgtggaaaggtgaaggagcggtagtcatcagaaattct
caaggtaccttgtttgtgaaacctagaagaaaagtaaagatcaccagactccaatatggagaagatgtgggcagt
gagaatttgttatcc
>#23 AY159322 2748 nt
gaaatagaggggcaaaaggtagaggcacttctagatactggagcagacgatacagtaattaaagatttagagtta
acaggaaattggaagccacgaattataggaggaattggaggagcaatcagagtaagacagtattttaattgtaaa
gtagaaatagcaggaaaaattactcatgctgcagttctggtgggccctacccctgtaaatattataggtagaaat
gtacttaagaagttaggatgtactttaaattttcctattagtaagatagagacagtaaaagtaacactaaaacca
ggaacagatggaccgagaataaaacagtggccactgtctaaagaaaagatcctagccttacaagaaatatgcaat
caaatggaaaaagaaggcaagatctctagagtaggtccagaaaatccatacaacaccccagtattctgtataaaa
aagaaagatggaaccagctggagaaaattagtagattttagacaactgaacaaagtcactcaggatttctttgag
gtgcagttaggaatcacacatccaggaggcctaaagcaatgtgagcaaattacagtattggacataggagatgcc
tatttttcatgtcccttggatgtggactttagaaagtatactgcgttcaccattccatcggtgaacaaccagggc
ccaggaatcagataccagtataatgtcctaccacagggatggaaaggctctccagcaattttccaggcaacagca
gacaaaattctaaaggcttttaaggaaaagcatccagaagtcttaatctatcagtatatggatgacttgtttgtg
gggagtgatctaagtgcctctgaacacagtaaaatgatagtagaattaagggaacatttgcagttctgggggctc
gagaccccagacaaaaagttccaaaaggaacctccctttgaatggatgggatatgtgctgcacccaaagaaatgg
acagttcagaaaatacagctaccagaaaaagaaagctggacagtaaatgatattcagaaattagtagggaaactt
aattgggcaagccaaatctattccggaattaaaacaaaagagctctgtaaattgataagggggcaagaccccta
gataaaatagtggaatggactagagaagcagaactagaatatgaagaaaacaagctaatagtgcaggaggaagta
catggagtgtactaccagccagaaaaacctttaatggcaaaggtgcaaaagttaacacaaggacagtggagttac
caaatagaacaggaagacaacaaacctctcaaggtaggaaagtatgccaggacaaagaatgcccacacaaatgaa
ttgagggtacttgcagggttggtacaaaaggtagctaaggagtgtctagtaatctggggaaagctaccgaaattc
tatctcccgttagagagggaggtatgggaccaatggtggcatgactattggcaagtaacatggatcccagagtgg
gacttcatttcaacaccaccattaataaggttatggtacaacctgctaaaagacccaataccaggggaagatgta
tattatgtagatggggcagcaaacagaaactccaaggaaggcaaggcaggctattatacctctagaggtaaaagc
aaggtagtagccttggaagaaacgaccaatcaaaaggcagaattgacagctataaagatggctttgcaagattca
ggacccagggtgaatatagtcactgattcccagtatgcactaggcatactttcagcagccccagatcagtcagac
aaccccatagttagggaaataatagaactcatgataggcaaggaagagatttatctctcatgggtcccagcccac

aagggtataggaggtaatgaacagatagataaattagtaagtaagggaataagacaggtactatttttagaggga
atagataaagcccaggaagaacatgacaaatatcacaataattggagagcgttgtctcaggactttaacctaccc
cccatagtggcaaaagagatagttgcacaatgtccaaaatgtcagacaaagggagaacctgttcatgggcaggta
aatgcagatccagggacatggcagatggactgcacccatttagaaggaaaaatcatcatagtagcagtccatgta
gccagtggatatttagaagcagaggtaataccagcagagacaggaaaagaaacagcgcacttcctgttaaagcta
gcaggtaggtggccagtaaaacatctacacactgacaatggtcctaactttgtcagtgaaaaggtggccacagtc
tgctggtgggctcaaatagagcacaccacaggagtaccttacaacccacagagtcaaggagtagtagaagcaaaa
aatcatcatcttaagaagatcatagaacaagttagggatcaagctgaaaggctagaaacagcagtgcaaatggca
gtgctaattcacaatttaaaagaaaaggggggataggggagtacagtcctggagaaagaatagtagacataata
accacagacatcctaacaaccaaattacgacaaaatatttcaaaaattcaaaattttcaggtttattacagagaa
ggaagggaccaacagtggaaaggaccagcagagctcatttggaaaggagaaggcgctgtggtaatcaaagaagga
gttgatttaaaggtggttcctagaagaaaagcaaaaataattaaggac
>#24 AF367411 2748 nt
gagatagaagggcaaaaagtggaggccttactggacacaggagctgatgatacagtaatcaaagatctagattta
aaaggtaattggaaaccacagattattggaggaattggaggatcaatcaatgtaaaacagtttttcaattgtaaa
gtaacaatagcaggcaaaactacacacgcttcagtcctagtgggccccacacctgtaaatattgtaggtagaaat
gtgctgaagaaattaggatgtacactaaattttccagttagtaaagtagaaacagtaaaggtaacactaaaacca
ggaactgacggacctaaaataaaacaatggccattgtctaaggaaaagattttagccttacaagaaatatgtagt
caaatggagaaggaaggccagatctctaagataggtccagaaaatccttacaacacaccggtgttttgcatcaaa
aagaaagatggaaccagctggagaaaattggtagattttagacagttaaacaaagtaactcaggacttctttgag
gtgcagttgggaatcccacaccctggaggtctcaaacaatgtgagcaaattacagtactggacataggggatgcc
tatttctcatgtcctttggatgaggactttagaaagtacactgcattcaccattccatcggtgaataatcagggc
ccaggaatcaggtaccagtataatgtcctaccacagggatggaagggctccccagccattttccaggcaacagca
gataaaatcttacagcccttcagagagagacatccagatgtagtgatctatcaatatatggatgatctctttgtt
gggagtgatagagttgccccagaacacagcagaatgattcaagagttaagagaccacctcttgttttggggggctc
gagaccccagacaagaagtttcaaaaggaaccaccctttgagtggatgggatacatactgcaccctaagaaatgg
acagtgcagaaagtacagcttccagagaaagaagaatggacagtaaatgacatccaaaagttggtagggaaactt
aattgggcaagtcagatttattccggaattaaaacaaaagagctctgtagactaattagaggggcaaaacccccta
gatgaaaaagtagaatggactagagaagcagaattagaatatgaagaaaacaaactcatagtgcaggaggaagtg
catggagtatactatcaaccagagaagcctttaatggcaaaggttcaaaaattgacacaagggcaatggagttac
caaatagagcaggaggataataagccgctgaaggtagggaaatatgccagaacaaagaatgctcatacaaatgag
cttagagtgcttgcaggactagtacagaagattgcaaaagaagctttagtgatctggggaaaactccctaagttt
tatctgccaatagaaagagaagtctgggaccagtggtggccagaatattggcaagccacatggattcccgaatgg
gaatttgtatccactccacaccttatcgggttgtggtataacctgttgagagaacctgtgccaggagaagatgtg
tattatgtggatggagcagctaacagaaactccaaagaagggaaagcaggatatgtaacagctagaaataaatcc
agagtaatagccctagagaataccacaaaccaaaaagcagaattggaggcaataaaaatggcattacaagactca
ggaccaaaagtaaatatagtgacagattcacagtatgctatgggtatcctatcggcagcacccgaccaatcagac
aaccccatagtaagagaaattatagaactcatgatccacaaggaagcagtatacctagcttgggtaccagcacac
aaaggcataggagggaatgagcaagtagataaactagtgagcagaggagtaaggcaagtactgtttttagaaggc
atagataaagcacaagaagagcatgataaatatcacaataattggagggcattagcacaagacttctgtatacca
aacatagtggcaaaagaaatagtagcccagtgtccaaagtgtcaaacaaaaggagagccaatacatggccaggta
gatacatccccaggaacctggcaaatggactgcacacatatggaaggaaaagtaatcatagcagcagtccatgta
gcaagtaggtatctagaagcagaagtaatacccacagaaacaggaaaagaaacagcacacttcctgttaaaatta
gcaggtagatggccagtaaaacatctacacacagataatggccctaatttcaccagtgaaaaagtagccacagtc
tgttggtgggctcaaatagagcacactacaggaattccctacaatcctcagagtcaaggagtaatagaggcaaaa
aaccatcatctcaaacaaatcatagggcaagttagagaccaagcagagaaactagaaacagcagttcaaatggca
gtattaattcacaattttaaaagaaaaggggggataggggaatacagtccaggagaaagaatagtagacattata
gcaacagacctcctaacaactaaattacaacacaatattcaaaaaattcaaaattttcgggtttattacagagaa
ggaagggaccaacagtggaaaggaccagcagaactcatttggaaaggagaaggagcggtggtaatcaaagagggg
acagacttgaaggtagtaccaaggagaaaagcaaaaatcatcagagat
>#25 AF328295 2748 nt
gaaatagagggacaaaaggtagaagccctactggacactggagcagatgatacagtaatcaaagatgtagattta
acaggaaattggaaaccccaaattataggaggaattggaggagcaattagggtgaaacagtatttcaattgtaaa
ataacagtagcaggaaaaagtactcatgcttcagtgctagtgggccctacccctgtaaatattataggtagaaat
gttcttaagaagttaggatgtactctgaattttcctattagtaagatagagacagtaaaagtaacactaaaacca

ggaatggatggaccaagaattaaacagtggccactgtctaaagaaaagatttagccttacagacaatatgtgat
gaaatggaaaaggaaggcaaaatctctagaataggtccagaaaatccatataatacaccagtattttgtataaaa
aagaaagatggaaccagctggagaaaattagtagatttaggcaattaaatagagtgactcaggatttctttgag
gtgcagctaggaatcccacatccgggaggcttaaaacaatgtgaacaaatcacaatattggatataggagatgcc
tatttttcatgtcctttggatgagagctttagaaagtacactgcatttaccattccatcggtaaataatcaaggc
ccaggaatcaggtaccagtataatgttcttccacagggatggaaaggctctccggcaatatttcaagcaacggca
gacaaaattctaaaaacattcaaagaggaatacccagaagtattaatttatcaatacatggatgatctgtttgtg
ggaagtgatctgagtgcgtcagagcacaacaaaatggtaaataggttgagggaacatttaaggttctggggctc
gagaccccagacaaaaagttccaaaaagaacctccttttgagtggatggggtatgtgctgcacccacagaaatgg
acagtccaaaagatacagctgccagaaaaagagacatggacagtaaatgatattcagaaactggtaggaaaactc
aattgggcaagtcaaatctattccggaattaaaacaaaagagctctgcaaattgatcagaggggcaaaacctcta
gatgaagtagtagaatggactagggaagcagaattagaatatgaagaaaataagttaatagtgcaggaggaagta
catggagtttattatcagccaggaaaacctctgatggccaaggtacagaaactgacacagggacaatggagctat
cagatagaacaggaagagaataagcctcttaaggtagggaagtatgctaggacaaaacatgcccacacaaatgag
ttaagggtgcttgcaggattagtacaaaaaatagccaaagagtgcatagtgatttggggagaattgccaaaattc
tatctcccccctggagagggaagtatgggatcagtggtggcatgattattggcaggcaacatggatcccagaatgg
gaattcatctcaacaccaccactaataaggttgtggtataacctgttaaaagaaccaataccaggggaggatgtc
tattatgtggatggggcagcaaatagaaactctaaggaagggaaagcaggatactatacagctaggggaaaaagt
aaagtaatagccttagaaaacacaactaatcagaaagcagaattagaggcaatactgttagccttgaaagattca
ggtcctagggcaaatatagtcacagactcacaatatgcattaggcatactttcagccactccagatcgatcagac
aaccctatagttagagaaataatcaaccaaatgatagccaaagaagctgtatacttagcatgggtaccagctcat
aaaggcataggaggcaatgaacaaatagacaaattagtaagcaaaggaatcagacaagtactattcctggaagga
atagataaagctcaggaggaacatgacaaataccataataattggagatcattaagtcaggaatttagcataccc
cctatagtggcaaaagaaatagttgcacagtgcccaaagtgtcaggtaaaaggagaacctatccatggacaggtg
gatgcggccccaggaacatggcaaatggattgcacccatctagaaggaaaggttatcatagtagcagtccatgtg
gccagtggatatctagaagcagaagtgataccagcagaaacaggaaaagaaacagcacatttcctgctaaaacta
gcaggaagatggccagtgaaacatttacatactgacaatggtccaaactttgtcagtgagaaggtagccacagtg
tgttggtgggctcagatagagcacaccacaggaataccctataacccccaaagtcaaggagttgtggaagcaaaa
aatcatcatcttaaggtaattatagagcaagttagggatcaagcagagagactggagacagcagtacaaatggca
gtactcattcacaattttaaaagaaaagggggatagggagtacagtccaggagaaagaatagtggatataata
acaacagatattctaactaccaaattacaacaaaatatttcaaaaattcaaaatttcgggtttattacagagaa
ggaagggagcagctctggaaaggaccagcagaactcatttggaaaggagaaggagcagtggtaatcaaagaagga
acagacttaaaagttgtgcccagaagaaaagctaaaataatcagggat
>#26 KM378564 2748 nt
gagatagaagggcaaaaggtggaggctttactagacacaggagcagatgatacagtaattcaaggattagaatta
acaggaaaatggaagccacaaattataggaggaattggaggagctattaatgtgaagcaatattttaattgtgaa
atcaaggtagcaggtaaaattactcatgcttcagtgttggtaggccctacccctgtaaatattataggtagaaat
gtgctgtgtaaattaggagctactcttaactttcctgttagtcaagtagaaccagtaaaagtaacactaaaacca
ggaatggatgggccaaagataaaacaatggcccctctccaaggaaaaaattgaagccttaaaagagatatgtgag
caaatggagaaggaaggtcaaataagtagaataggtccagaaaatgcatacaacacaccggtgttttgtattaaa
aagaaagatggtaccaaatggagaaaattagtagatttcaggcagttaaataaagcaacacaggacttctttgag
gtgcagttggggataccacacccgggaggattaaaagagtgcgaacagatcacagtactggatgtaggtgatgcc
tacttctcatgccctctggatccggactttagaaagtacactgcattcaccattccatcggtgaataatcagggc
ccaggaatcaggtatcagtataatgtcctcccacagggatggaaaggctcacctgcaatcttccaagctacagct
gacaagatcttacagccattcagagagaaaaatccagatgtgattatttatcagtacatggatgatctgtttgta
ggaagtgatagaccaaagcatttacatgatcagatgattaaagaattgagaacacatttaaagttctggggctc
gagaccccggacaaaaaatttcaggacaatcctccttatgagtggatgggataccagctgcatccaaagaaatgg
acagtgcaagaaattcgtttgcctgataaagaaatttggactgttaatgatattcagaaattagtaggaatactc
aattgggctagtcaaatttattccggaattaaaacaaaagagctctgtaaattaattaggggagccaagcccctg
gatgaagaagtagaatggtctagggaagcagaactagaatatgaagaaaacaaactgatattaaaagaacaaatg
catggggtatattatcagccagaaaagcccctaaaagccaagatacaaaaattgggcaatggacaatggtcatat
cagatagaacaagatgacaacaagccactaaaaacaggaaaatatgctaaggtaaagaatagtcacaccaatgat
atgaggatgttagcaggactagtgcaaaaggtagcaaaagaagcactggtaatctggggaagattgcccattttc
tacctaccggtagaaagggaggtttgggaacaatggtggcaggagtactggcaggtaacatggattcctgattgg
gaatttgtatcaacacccccctctcatcaagttatggtataacttgttgagtgacccaataccaggagaagaagtt

tattatgtggatggagctgctaacagagttagcaaagaaggaaaagctggctatgttacctccagggaaaagaa
aaggtaatagccttagaagaaaccaccaatcagaaggctgaattacaggctattctgttagccttgaaagactct
ggtccaaaagtcaacatagtgacagattcacaatatgccatgggcataatatcttcctccccagaaatcagtgac
aaccctatagtgaatcaaattatagaacaaatgatcagtaaagaagcagtttacttaaattgggtaccagcacat
aaagggataggaggtaatgaggaggtggacaaattagttagtagaggaataagacaagtgttattcctagataat
atggaaaaggcacaggaagaacatgatttataccataataattggagatccttagcacaagaatttggtctacca
gggatagtggcaaaagaaatagtggcacagtgtcccaaatgtcaaatacatggagagccaatacatggccaagta
gatgcaagcccaggaacttggcaaatggactgcacacatctagaaggaaaaataatcatagtggcagtccatgta
gcaagtggatacatagaagcagaagtaataccagcagaaacaggagagaaaacagcttactttctgctaaagtta
gctggcagatggccagtatcacacttgcatacagataatggaccaaatttcaccagtgaaaaggtagcaacagta
tgctggtgggctaaaatagagcataccacaggagtaccctataacccccagagtcagggagtggtagaatcaatg
aacaatcagctaaagaaaattataggacaaatcagagatcaggcagaaaaactagagacagcagttcaaatggca
gtactgattcacaattttaaaagaaaaggggggatagggggagtacagtgcagcagaaagaatagtagacatcata
gccacagatctcttaacaagcaaactacaacaaaatattctcaaaattcaaaattttcgggtttattacagagag
ggaagagatcagttgtggaaaggaccagcagaacttgtctggaaaggggaaggagcagtggtcatcaaggaaggt
actgacttgaaagtaatacctagaagaaaggcaaagatcatcaaagat
>#27 KM378563 2748 nt
gaaatagaagggcaaaaggtagaggctttactagacacaggagcagatgatacagtaattcaaggattggaatta
acaggaaaatggaagccacaaattataggaggaattggaggaaccattaatgtgagacaatattttaattgtgaa
atcaagatagcagggaaaattactcatgcttcagtgttagtaggccctacccctgtaaatattataggtagaaat
gtactctgtaaattaggagctactcttaactttcctgttagtcaagtagaaccagtaaaagtaacactaaaacca
ggaatggatggaccaaagataaaacaatggcccctctccaaggagaaaattgaagccttgaaagaaatatgtgaa
caaatggagaaggaaggtcaaataagtagaataggtccagaaaatccatacaacacaccggtgttttgtattaaa
aagaaagatggtaccaaatggagaaaactagtagatttcaggcaattaaacaaagcaacacaggacttctttgag
gtgcagttagggataccacacccaggaggattaaaagcatgcgaacagatcaccgtactggatgtaggtgatgcc
tacttctcatgccctctggatccggactttagaaagtacactgcattcaccattccatcggtgaataatcagggc
ccaggaatcaggtatcagtataatgtccttccacagggatggaaaggctcacctgcaatcttccaagctacagct
gacaagatcttacagccattcagagagaagaatccagatgtgattatatatcagtacatggatgatctgtttgta
gggagtgatagaccaaagcatttacatgagcagatgattaaggaattaagaacacacttaaagttctgggggccc
gagaccccagacaaaaaatttcaggacaatcctccttatgagtggatggggtaccagctgcacccaaagaagtgg
acagtgcaagaaattcgcttgcctgataaagaaatttggactgttaatgatattcagaaattagtaggagtgctt
aattgggctagtcaaatttattccggaattaaaacaaaggagctctgtaaattaattaggggagccaaaccccta
gatgaagaagtagaatggaccagggaagcagaactagaatatgaagaaaacaaactgatattaaaagaacaaatg
catggggtatattatcagccagaaaaacccctaaaggccaagatacaaaaactgggcaatggacaatggtcatat
cagatagaacaagatgacaacaagccactaaaaacaggaaaatatgctaaggtaaaaaatagtcacaccaatgat
atgagaatgttagcaggactagtgcaaaagatagcaaaagaggcactggtgatctggggaagattgcccactttc
tacttaccagtagaaagggaagtttgggaacaatggtggcaggagtactggcaggtaacatggatcccggattgg
gaattcgtatcaacaccacatctcatcaagttatggtataatttgctgagtgacccaataccaggagaagaagtt
tattatgtggatggagccgctaacagagttagtaaagaaggaaaagctggctatgttacctccaggggaaagag
aaggtgatagccttagaagaaaccaccaatcagaaggctgaattacaggccatcctgttagccttaaaggactcc
ggtccaaaagtcaacatagtgacagattcacagtatgccatgggcataatatcatcctccccagaaatcagtgac
aacccgatagtgaatcaaattatagaacaaatgatcagtaaagaagcagtttacttaaattgggtaccagcacat
aaaggaataggaggtaatgaggaggtggacaaattagttagtagaggaataagacaagtgttatttctagataat
atggaaaaagcacaggaagaacatgatttataccataataattggagatccttagcacaagaatttggtctacca
gggatagtggcaaaagaaatagtggcacaatgtcctaagtgtcaaatacatggagaaccaatacatggccaagta
gatgcaagcccaggaacttggcaaatggactgcacacatttagaaggaaaaataatcatagtggcagtccatgta
gcaagtggatacatagaagcagaagtaataccagcagaaacaggagagaaaacagcctactttctgctaaaatta
gccggcagatggccagtgtcacacttgcacacagataatggaccaaatttcaccagtgaaaaggtagcaacagta
tgctggtgggctaaaatagagcataccacaggagttccctataacccccagagtcaaggagtggtagaatcaatg
aacaatcagttaaagaaaatcataggacaaattagagatcaggcagaaaaactagagacagcagttcaaatggca
gtactaattcacaattttaaaagaaaaggggggatagggggagtacagtgcagcagaaagaatagtagacattata
gcctctgatctcttaacaaataaactacaacaaaatattctcaaaattcaaaattttcgggtttattacagagag
ggaagagatcagttgtggaaaggaccagcagaacttgtctggaaggggaaggagcagtggtcatcaaggaaggt
actgacttgaaagtaatacctagaagaaaagcaaagatcattaaagat
>#28 M27470 2766 nt

gaggtctcagtggatggtgtcactataagagctctactagatacaggagctgatgataccatctttaatgaaaga
aatataaaattaaaaggaaattggcagccaaaaattatagggggaataggtggaaacttaagagtaaaacagtat
gataatgtatatgtagaaataagagggaagggaacatttgggacagtattgataggacctactccaatagatata
atagggagaaacataatggaaaaattaggaggaaaattaatattggcacaattgtctgataaaataccaataaca
aaagtgaaattaaaaccaggagtagatggacccagaataaaacaatggcctttaagtaaagagaaaatagttggt
cttcagaaaatatgtgatagattagaggaggaaggaaaaattagtagggtagatccaggaaataattacaataca
cctatctttgccataaagaagaaggataaaaatgaatggagaaaattaatagactttagagaattaaacaagtta
acacaggattttcatgaattacagttaggtatacctcacccagcaggaataaaaaagtgtaaaagaataacagtc
ctagatataggggatgcctattttagtatacctctggatccagattatagaccctatactgcctttacggtacca
tcagttaataatcaagcaccaggaaaaagatacatgtataatgttcttcctcaagggtggaagggaagtccatgt
atctttcaagggacagtagcatcactgctggaggtatttagaaagaaccatccaacagtacagttatatcaatac
atggatgatttgtttgtagggtcagactatacagcagaagagcatgagaaagctatagtagaattaagggcttta
ttaatgacatggaacttagaaacacctgaaaagaaatatcagaaagaacctccctttcattggatggggtatgag
ttacacccagataagtggaagatagaaaaggttcaactaccagaattagcagaacagccaacagtaaatgaaata
cagaaattggtaggtaaattaaattgggctgcacagttatatcctgggatcaaaacaaaacaactgtgcaagcta
ataagaggaggactaaacataacagagaaagtcacaatgacagaagaagcaagactggaatatgaacaaaataaa
gagatcttggctgaagaacaagaagggtcttattatgatcctaataaggaattatatgtaagatttcagaaaaca
acaggaggagatatatcatttcaatggaagcaaggaaataaggttttaagagcagggaaatatgggaaacagaaa
acagcacatagtaatgacctcatgaaattggcaggtgctacgcagaaggtaggaagagaaagtatagtaatctgg
ggttttgtaccaaaaatgcagatacccactacaagggagatatgggaagattggtggcatgagtattggcagtgt
acatggataccagaagtagaatttatcagcacacctatgttagaaagggaatggtatagcttgtccccagaacct
ctagaggggggtagaaacatattatgttgatggagcagctaacagggacagtaaaatgggaaaagcaggatatatt
acagatagaggttttcaaagggtagaagaatatctaaataccaccaatcagcagacagaattacatgcagtaaaa
ctagctctagaagatagtggaagttatgttaacatagtaacagattcacaatatgtagtaggtatactagcaagc
agacctactgaaacagatcaccccatagtaaaggaaataatagaattaatgaaaggaaaagaaaaaatttattta
agttggctaccagcacacaaagggataggagggaatgagcaaatagataagctagtaagttcaggaatcagaaaa
gtcttattcctacaaaatatagaaccagcacaggaagaacatgagaaatatcatagcaatgaagcacaattaaga
gagaaattccacttaccagctctagtagccaaacagattgtgcaaagttgcagtaagtgctgtcatcatggagag
cccataaagggacagacagatgcttcacttggagtctggcagatagattgcacacatctggaaaatcaaattatt
atagtagcagtgcatgtagcttcaggcttcatgaaggcagaagttataacagcagaaactggaaaaaagacagca
gagtttctgttaaagttagcagcacaatggcctattagtaaactacacacagataatgggcctaactttactagt
caggaagtagaaaccatgtgttggtggttagggatagaacacacatttggaataccctataacccacaaagtcag
ggggtagtggaaaataaaaataagtatctaaaagaattgattgagaaaataagagaagattgcaaagaattaaaa
acagcagtagccatggccacattcattcataattttaaacaaaggggaggactaggggggatgacagcaggagag
agaatagtaaatatgatcaatacagaattagaatatcaatatcaacaaaatcaaatttcaaaaaatttaaatttt
aaggtttacttcagagaaggaagagatcagctgtggaaaggacctggtatccttttgtggaaaggagaaggggca
gtagttttaaaatatcaagaagagataaagatagtacctagaagaaagtgtaaaataataaaagat
>#29 AF131870 2769 nt
aaggtcttcattaatgggcaacctgtgactgctttattggacacgggagcagatgacacaatagtgtctgaagat
tctgtgtccatagaaggtcactggaagcccagggtagtgggtggtataggggggacaaattagagtaaaagaatat
gcagatgtctttgtagaaattagggataaaacagctacaggaacagtgttggtggggccaacaccagtagatatc
atagggagaaatattctttcagccttaggggggaagattagttttagccactcttagtgaaaagatcccaattaca
aaagttaagttaaaagaagggggccgctggaccaaaagtgaaacaatggcccctgtcaagggagaaaatagaaggc
ctacagaaaatttgtgatagattggaagcagaaggaaagattagcagggcagacccagggaaccccttataacaca
ccaatttttgctataaagaagaaagacaaaaatgaatggagaaagttaatagattttagagtactaaatggtatg
actcaagactttcatgagttacagttagggattcctcatccagcaggattaaagaagtgtaaacagattacagta
gtagatgtgggggatgcatacttctcaataccattggatccagattatagaaagtatacagcattcacaataccg
tcagtaaataatcaggcaccaggaaaaagatatgtgtataatgtcctaccacagggctggaaaggaagtccttgc
atctttcagggaacggtagcgagcctcttagagaaatttagaagacagtatccagaagttcaattgtaccaatac
atggatgatttactcataggatctgattatgagaaaaagaagcatgaagaaatagtgaaacaattgaggcagcta
ctcatggaatggaatttagaaacaccagagaaaaaatatcagggagagccaccctataagtggatggggtacata
ttacacccagacagatgggagatagagaagataaaattacccccctctagaagaagagccaacagtgaatgacata
cagaaaattgtaggagttatcaattgggcttcacaattatatgaagggctcagaacaaaagaattatgtaaacta
attagagggaataaaccattagcagagaaagtaaagatgacagaagaagctagggaagaataccagagtaatcag
gaggtattacaggagtcagtatcaggcagttactatgaaccagataaagaattaatttgcagagtgcaaaaggta

aagcaaggaatattaacctttcaatggttgcagggaaaacaagtattaagggtaggaagatatcaaaagagaggg
gcagcacatgagaatccttgtcaacagttagcagctgccctacaaaagataggaagagaaagcatagttatatgg
ggatttgtgccaaagatacaggtacccatacaaagagagatatggagtcagtggtgggcagactactggcagtgt
acatggatccctgagttagaatttgtcagcactcctaagttggagcaagagtggtatactctagccacagaacca
gtgccaggagacacctactatgtggatggagcagctgaaaagctagaaaagagagggaaagcaggatatataacc
cagggaggaagatccagagttaagaaattggaaaacaccaccaatcagcaagcagaactagaggctataaaaatg
gccttggaggatagtaggagctcagttaatatagtaacagactcacagtatgccttaagactgctcagtaagaga
cccacagaaacagattcagagctagtaaaggagatcgtagagctcataaggcaaaaggaccaggtatacctaggt
tgggtaccggcacacaaaggaataggaggaaatcaagaaatagatcaactagttagccagggaatcagaaagaga
caggtaatgtttatagaaaagatagaaccagctgtagaagaacatggaagttccataacaatgcagcctctttg
caggagatgtttgacatacccctagtggtagcaaagcagatagtcaatgaatgtgcacaatgtcaacagaaaggg
gaagccataacaggacaagtagatgcctcagtgggcatctggcagatagattgcacccacatggaagaaaaagtc
ataatagtagcggtgcatgtggcttcagggtatatggtagcagaagtactacccaatgagcaaggaagacaaca
gccacttggctgttaaagctctgtgccatgtggccagtaaaacagatacatacagataatgggccaaatttcatc
agtaaagatgtggaggcagtatgttggtggttaggcatacagcatacaacagggataccttacaaccctcagagt
caaggagtagtggaagcaaagaacaaggtattaaaacaaattatcagcagaattagagaagatgcacaggaattg
aaaactgcagtactcatggctctgcacattcataattttaaacaaaggggaggactaggggggaatgacagcagca
gagaggtttatcaacatgattaatgcagatctagaaacacaatacttacagaaaataaattcaaaaattttaaaa
tttaaggtctactacagagaaggaagagatcctcagtggaaaggacctgctaaacttctgtggaaaggggaagga
gcagtagtcatcaaacagggtgagaacatcctggtagtacctaggagaaaagcaaaactagtaaaggac
>#30 AF075269 2775 nt
agtatattggtcaatgggcaaccagtgtcagcactgttggacactggggctgatgacacaatttttcagaaaat
tcagtaagaatagaaggaccctatactccaagagtagtaggggcatagggggcaaattagggtaaaagaatat
agagatgtctttatagaaatagcaggaaaaactacagtagggacagtcttattaggacctaccccagtagatatt
gtagggaggaatattttaactgcaatggggccaaattgatttagctcagttaagtgataaaattcctattaca
aaggtatctttaaaacctgggtgtgatggaccaagagtaaagcagtggcctctatcaaaagagaaaatagaaggc
ctacaagctatttgtgataggttagaaaaagaaggaaaaatatctccagtggatccagggaacccatacaatact
ccaatatttgctatcaagaagaaagacaaaaatgaatggagaaagttaattgatttcagaaagctcaatgaatta
actcaagactttcatgagctacaattaggtattccacacccagcaggtatcaagaaatgcaaacagattacagta
gtagacataggagatgcctatttcagtatccctttagatccaaattacagaaagtatacagcatttactatacct
tctctcaataatcaagagccaggaaaaagatatcagtacaatgtgctgccacaagggtggaaggggagcccttgt
atatttcagggaacagtagcaggacttctctcagagtttaggaaattaaatccagacatgatcatttaccaatat
atggatgatttattcataggatcagatagagagagaaaaggacatgatcaggcagtaaaagaactcagagaactt
cttatgacatggaacttagagacaccagaaaagaagttccaagcagagccaccctatcattggatgggttatgta
ctgcatcctgataggtgggaaatagaaaagattaaattaccagagatggatctaacaaaaactacagtaaatcaa
atacagaaactggtgggagtacttaattggcagctcaattgtatgatggtattaggacaaaagaactctgcaaa
ttaataaggggagtaaaacccttggaagaaatcataaactggacagaggaagccttggaagaatatggcagaac
aaagaggtacttaaagaaaagatgcagggagcctattatgacccagaaaaggaacttattgtcagggtacagcaa
aacaaaaaggggataattactttccagtggagacaaggaaataacatcttaagagctgggaggtatcaaagacag
aaggcagcacacacaaatcccctacagaaattagtagaagctattcagaagataggaaaagaaagcatagtcatc
tggggctttgtgccaaaaattcaagtcccagtgactagagaagtctgggagcactggtggagcgaccactggcag
gttacatggattccagacttagaattcatttctaccccgcaattagaacaagagtggtacatttgggaggcagaa
cccataataggggtagacacctactatgtagatggagcagcagaaaaggtaggaaaaacaggaaaagcaggatat
ataacacaatcagggaaagagaaagtaaaggagttaactgacaccacaaatcagcaagcagaactagaggcagtt
ctaatggcattacaagatagcaatagtaaagtaaatatagtaacagattcacaatatgtaatgaaaatattgtca
caaagaccaacagaaacagaacaccccatagtaaaagacatcatagaacaatgcaagcagaaagatcaagtttat
ctaggatgggtgcctgctcataaaggaataggaggtaatcaagaggtagaccacctagtaagtaaaggtataaga
cagaaacaggtcatgtttctagaaaagatagaacctgcagtagaagaacatagcaaattccataacaatgcaaag
gatctagaagaaaaatttaacctaccccccatggtagccaaacaaattgtcaatgactgtgcaaactgtcaaaag
aaaggagaagccataacaggacaagtggatgtctcagtgggtatttggcagctagactgtactcacttagaagga
caggtcattataaatgcagtccatgtggcctcaggcttcatggttgcagaagttataccagatgagacaggaaaa
acaacgtcacatttcctgttaaaattatgcagtagatggcctgtaaaacaaatacatacagacaatggtccaaat
tttgtaagtaaggaggtacaggcagtaacctggtggatagggatagaacatacaactgggataccttataaccct
cagagtcaaggggttgtagaagcaaaaaacaaagttttaaaaagtattatagaaagagtaagagaagatgcacag
caactaaaaacagcagtactaatggcagtccacattcataattttaaacaaaggggaggactagggggcttaca

ccagcagagaggtttattaatatgattaatgcagaactagaaacacaatatctacaaaaattaaattcaaaaatt
ttaaaatttaaggtttattacagacaaggaagagatcctcagtggaaaggaccagcgcaacttctgtggaaagga
gaaggtgctgtagtggtaaaagaaggggagaacatcttctcagtccccagaagaaaagcaaaactagtaaaagat
>#31 AF188116 2775 nt
aaagtactggtcaatggcaagaagtaactgccctattggacactggagcagatgatacaatcttttctgaaaat
tcagttagaatagaaggaccttatactccaagagtagtaggggggtataggggggacaaattaaagtaaaagaattc
agggatgtttttatacaaatagcagggaagaccacagtagggacagttttgctaggaccaaccccagtagatata
gtaggaagaaacatttttaaaccctctaggggctagattagtgttagcacaattaagtgagaaaatacctataaca
aaggtgcatttgaagccagggtgtgatgggccaagagtaaaacagtggccactatccaaagaaaaaatagaagga
ttacaagcaatttgtgatagattagaaaaggaagggaaaatatccccagttgatccaggaaatccttacaacacc
ccaatctttgctataaagaaaaaggacaaaaatgaatggagaaagttgatagatttcagaaaattaaatgagtta
acacaagactttcatgagctacagttaggaataccacacccagcaggaataaagaaatgcaaacaaattacagta
gtagatataggagatgcctatttcagcatacctttagatcctgattacagacaatatactgcgttcactataccg
tcactaaacaatcaggaaccaggaaaaagatatcagtataatgtgttgccacaaggatggaaggggagtccttgt
atttttcaagaaacagttagtggactccttgcagagttcaggaaattaaatccagacatactcctctaccagtac
atggatgatttgttcataggttcagacagggagaaagaaggacatagtcaggcagtaaaagaattaagagaactt
ttaataacttggaacttagaaactccagagaagaaatttcaagacaaaccaccctaccattggatggggtatgtc
ctgcacccagatagatgggaaatagagagagtaaaactgccagagatagacacaaggaaaactacagtaaatgaa
attcagaaactggtaggagttttaaattgggcagcacaactgtatgatggaatcagaacaaaagagctctgtaag
ttgattagaggagtaaagcccttggaacaactagtgacttggacagaggaagcactagaagaatatgagaccaat
aagacaattctaaaagaaaaagtacaggggggcttactatgatccaagtaaagaacttgtagtaagggtacaacag
aacaagaaaggggttataactttccaatggaggcaaggaaatgccattctaagagcaggaagatatcaaagacaa
aagagtgctcacatgaatccccttcagcaattagcagaagctgtacagaaaataggaaaagaaagcatagtcatt
tggggttttgtaccaaagatacaagtacctgtcactagagaaacatgggaaaattggtggataaacttttggcaa
gtaacttggataccagacttagaattcatctccacacctcaattagaacaggaatggtatagcttggaagcagaa
cccatagaaggtacagatacctactatgtggatggagctgcagaaagagtaggtaaaacaggaaaagcagggtat
ataactcaatcaggaaaagagaaaataaaagaattacaagatactactaatcagcaggcagaattagaggcagta
ttgttagctttacaagatagcaaatcaacagttaacatagtaactgactcccaatatgtaatgaaggtcttaagt
cagagacctacagaaactgagcatcctatagtgaagaacatcataaaggagctgcagaagaaagatgcagtttac
ctaggatgggtaccagctcataaaggtataggggggaaaccaggagatagatcaattagttattaaaggaattagg
acaaaacaggtcatgtttctggagaagatagaaccagctgtagaagagcatggaaagtttcacaataatgcaaaa
gacttagaagaaaagtatgggatacccccccatggtagctaagcaaatagtaaatgagtgtgctaagtgtcagcag
aagggagaagccataacaggtcaagtagatgtatcagtgggtatttggcaactggattgtactcacctagaaggg
caaatcatcatgaatgcagtccatatggcatcaggcttcatggttgcagaagtgattccagatgaaacagggaaa
actacagcaaattttttgttaaagttatgtagcagatggcctgtaaaacaaattcacacagataatggtccaaat
tttgtgagtaaagatgtacaggcagtgacctggtggttaagtatagagcacacaacagggataccatataaccct
caaagtcaaggagtagtagaggcaaaaaataaagttttaaagcaaatcatagaaaagagtaagagaagatgcacag
cagctaaagacagcagtgttaatggcagtccacattcataattttaaacaaaggggaggactggggggggttatca
ccagcagagagatacatcaatatgatcaatgcagatttagaaactcaatacatacaaaaattaaattcaaaaatt
ttaaaatttaaggtttactacagacaaggaagagatccacagtggagaggacctgctcaacttctgtggaaagga
gaaggtgctgtagtggtaaaggagggagagaacatttctctatccccaggaggaaagcaaaattagtgaaagat
>#32 AF188115 2775 nt
aacattctggtcaataggcagccagtgtctgctcttcttgacactagggcagatgataccatcttttcagaaaat
tcagttaaaatagagggaccttatacacctagagtagtaggggggaatagggggggacaaattagagtaaaagaatac
aaggatgtctttatagaaatagcagggaagacaacagtagggacagtactattaggacctacaccagtagacatt
gtaggaagaaatatttttaactagtgtgggagcaaaattagtactagcacaattaagtgacaaaatacctatcaca
aaggtcagattgaagccaggctgtgatggtcccagagtaaaacagtggcctttgtcaaaagaaaagatagagggc
ctacaagcaatttgtgatagattagaaaaagaagggaaaatatcaaaagcagatgcaggaaacccatataacaca
ccaatctttgccataaagaagaaggacaaaaatgagtggagaaaactgattgatttagacaactaaatgcttta
acacaagaccttcatgaagtacaattaggcattccacatcctgcaggaataaagaaatgcaagcaaataacagtg
gtagatataggagatgcctattttagtattcccctggatccagactatagacagtacacagcattcacagtacca
tctattaataaccaggaaccagggagaagatatcagtataatgtactaccccaaggatggaaggggagtccatgc
atttttcaagctacagtagcaggactcctatcagaatttagaaaattaaaccctgacataatcctgtatcaatac
atggatgatttactcatagggtcagatagggacaagaaagggcatgatcaggcagtaaaagaattaagagagctc
ttaatgacatggaatttagaaacaccagaaaagaaatttcaagcagagcctccctatcattggatggggtatgta

cttcatccagataggtgggaaatagagaaagttaaactgccagacatagaccttcagaagacaacagtaaacaac
atacagaagctagtaggagtcttgaattgggcagcacaactttatgagggaattagaactaaagaactttgtaaa
ttaattagggggtaaaagctctagaagaagttgtaacctggacagatgcagcattagaagaatatgaacaaaac
caacagatactaaaagaaaaactgcagggagcttattatgaccctactaaagaattggtagtaagagtacagcag
aataaaaaggggtcattaccttccagtggaggcaaggaaattctattctgagagcaggaaggtatcaaaggcaa
aaagcagctcacacaaatcccctacaaaagttggcagaggcagtacaaaaaataggaaaagagagtatagtcatt
tggggtcatgtgcctaagatccaggtaccagtaacaagagaagtgtgggatcaatggtggagtgatcattggcag
gtcacctggatacctgaattggagtttatctcaaccccacagttagaacaggaatggtatagcttagaagcagaa
cccatagaaggggtagatacctattatgtagatggagctgcagaaaaagtagggaaaacaggaaaagcaggttat
gtcacacaatcaggaaaggagaaagttaaagaattaacagataccaccaatcaacaagcagaattagaggctgtc
ttattggcattaaaagacagtaaaagcagggtcaacatagtaacagactcacaatatgtgatgaaagtcttatct
caacggcctacagaaacagaacaccctgtagtgaagcagattatagaggaatgtaagaagaaagaccaagtatat
ctaggatgggtaccagctcacaaaggtataggggaaatcaagaagtagaccacctagtcagtaaaggaattaga
caaatacaagtactgtttctagaaaaaatagagccagcacaggaagagcatagcaaatttcataataatgctaaa
gatctagaagaaaaatttcacctacccccctatggtagctaagcaaatagtgaacagttgtaataaatgtcagaca
aagggagaagccataacaggacaagtagatacttcaccaggtatttggcaaatagactgcactcacatggaggga
caggtaatcattaatgcaatccatgtggcctgtggatttatggtagcagaagtaattccagatgaaacagggaaa
acaacagcaaacttcctgttaaaactgtgcagcagatggccagtacagcaagtccatacagacaatggaccaaac
tttgtcagcaaggaggtacaagcagtaacctggtggctaggtattgaacactcaacagggattccttacaatccc
cagagtcagggagtagttgaagccaaaaataaagtgctaaagcaaatcatagaaagggttagagaagatgctcaa
caattaaagacagctgtcctaatggcagtacacattcataattttaaacaaaggggaggactaggggggctttca
ccagcagaaaggtttattaatatgattaatgcagacttagaaacacaatatttacaaaaactaaattcaaaaatt
ttaaaatttaaggtttactacagacaaggacgagatcctcagtggaagggacctgcacaaattctgtggaaagga
gaaggtgctgtagtggtaaaagaaggagataccatcttctcagtccccagaagaaaagctaagctagtaaaagat
>#33 AY523867 2769 nt
gaaataaatgtagaagggaccaaggtaacaatgttgctggacacaggggcagatgacaccataataagagaacaa
gacatccagttacatcaaccttggaccccaaaaatagtaggggggattaggaggaaacataagggtaagacaatat
aaccagatcagatttcaaatagaaagaccagatgggagaataaaggaagtagagggtagtcttctagtaggacct
acaccagtaaatattttaggaagaaacatattgagcaaattgggagcaaaattagtaatgatagcacaaaaatta
gaaccaatgaaagtccaactaaagccagggaaggagctacctaaattaaaacagtggcctctgacaagagaaaaa
cttgaggcactgcgagcaatagtttcagatatgcttgaaaaggggcaattggaaaaggcctcccctacaaatcct
tataataccccagtttttgtaatacaaaagaaggacaaattaaaatggaggatgcctatggattttaggaaactc
aatgaagcaacacaggacttttgggaagtacagttaggaatacctcatccaggagggctcagtacaaaaagaatg
acagtattagacttgaaagacgcctattacacagtacctctagatgaagagttcagacagtatacagcatttaca
gtgcccagtataaacaattcttctccaggagagagataccagttcaaggtgttgccacaaggatggaagggatcg
cccactatctttcaggcaacagtaaatcagatactccaacctataaggaaaagaattcagatataataatagta
cagtacatggatgaccttttggtagggtctaacagatcagaaaaggaacatggtcaaattgtaagtgaaattgta
aaaagtctattagcagtaggattctctattccaccagaaaaatggcaggacaaattccccttgcaatggttaggg
tacacgctacaccctgataagtggagtctacaaaaggttcaactaccagaaattacagaatcaccaacagttaat
gagttacagaaaataataggagtcctaaattgggcaagtcaaatatatcctggtatcaaatgtaaagcattgagt
aagtgcataagaggggcaaaagcacttaccgacattgtagaactgacagaagaggcagaagcagaaatggctgaa
aatagagagatactcaaagtagaacaaacagggtcttattatcagccagaaaaaccattagaagcacacattagc
aagttaggacaacaacagtgggggtacataattaaacaagggccacaagaaaaacctctgatcacaggaaaaaca
ggaaaagcatatgcgacccatcacaatgattaccaggcattggctcagctgatgaataaaataggaatacaagcc
ctatggatttggggaaaaatccctgaattccacctcccagtaaaaagggaagaatgggaaaaatggtggacagat
cactggcaagcaacatggttgccagaagtaaaatgtgtgcacaccccatattagttagatggtattacaatttg
gtttcagagccagtagaagcagaaacattttatgtcgatggggccgctaataggaatagcaaagaaggacaagca
ggttacataacagataggggtaaacagacattaaagaggttggaaaacacaaccaatcaaaaggctgagttagaa
gcagtattaatggcattacaagactcaggaccagaagttaacatagtaacagattctcagtatgcattaggaata
ttaatgaactgtcctacatcctcggaacacccagtagtcgaacagattatgcaaaaggctatggagaaagaaaag
atctacataacctgggttccagcccataaaggaataggaggaaatcaagaagtagatcaattagtaagtaaggga
attaggaaaatactgtttctggataacatcccaaaagcacaggaagaacatgagaaatatcacactaatgtagaa
tacataaggcaagaatttcatctacccagacaggtagccaaagctatagtagaaatgtgtcccaaatgccaaatc
agaggagaaccaaaacatgggcaagtagacacagatctaggtacatggcaaatggattgtacccacttagaagga
aaagtaatttgtgtagcagtaaacacagccagtgggtatacagaaactaaaatattgaaaagagaaacagggcaa

gaaacaggactattccttttgcaaatagcagcaagatggccaattaaacatatacatacagacaatggtccaaac
ttcatcagtgatgcctttgcggcagcctgttggtgggcaggagtcgaacacaccacaggaataccatacaatcca
caaagccagggaatagtagaaaacaaaaatagacaattaaaagaaaccatcaatcaaattagggaagaagtagaa
agattagagacagcggtggcaatggccacgttcattttaaattttaaaagaaagggaggaatagggggactaca
ccagcagataggtatattaacatgctatacacagaactacaactacaaaatacacacacacaaaaattttcgaat
tttaaagtatattatagacatggtacttctgattggcagggaccagcttctctactgtggaagggagaaggagca
gtagtgatccagacaccagatcaacaggtaatagcagtacctagaaggaaagcaaaaatcatcagcagt
>#34 AY655744 2760 nt
aaagtgctgatagaaggacaagaggtggaggtactcctagacacaggagcagatgacacaattatacaagtagga
gaaatagaattggaaggagtaccccaccaaaaaacagtaggggggattggcaggatacatacaagtgcaaagttat
agtgccatagaaatacagtgggaaaacaaaagagcaagggacacagttttgattggaccaacgccaattaacatt
ctagggagaaatttttttagcaaaatttagtgttacacttaacatggtcagctcagacataccgataaccaaggtt
aaattaaaagagggtaaggaacccccaaaatcaagcaatggcccttaaccagagaaaaaatagaagggctacaa
atcatcatagacgacatggtgaaggcaggacagttagaggaggttggaccggaaaatccatataattctccagtg
ttcgctatcagaaagaaggacaaaactcaatggagaatgctcatagacttccgcgccctaaatcaagcaacccaa
gacttcgcggaagtacaacaggggatacctcacccctcaggcctgcctcagatgaagcaaataacggtcctagac
atgaaggatgcctattacagcatccccttagacccagacttctcacagtacacagccttcaccatcccgtctctg
aacaatgataggccaggaagaagatatcaattcaaggtgctaccccaaggatggaagggttcgcccaccatcttc
caacatacctcggatcaattattgaggaaattcagggacaagtaccctcaggtagcactctttcaatacatggat
gacctattggtaggaagcaatgagagcttggaggaacatagaaggatagtaggagaactaagagctctcttaaac
aaggtagggatccagaccccagaggctaaataccaagacaaaccgcccattaaatggctagggtatgaattgtat
ccagataagtggaaagtacagcctattgaattgccagataaactcacatggacagtcaatgagttacagaagtta
ataggaaaacttaattgggcatgccaaatatattctggtattaaaacaaaggccctctgcaggtacctcagggga
gtaaaaggacttctagaagaagtacagttatcagaggaagcagaggcagaattagcagaaaacagggaaatctta
gcagaagaaactcacggtacatattaccaggaaggagagcccctggaggcagaactgaccaaattggcagaaggt
caatggggctatatgataaaacaaggcaaacggctcttaaaaacaggcaagtttgccaaacagagaacagcacac
agtaacccctatcaacagttaataggagctatgcagaaaataggcaaggagtccatagtcttttgggggcaggta
ccagtattcaggatcccagtggtcaaggaggaatgggaacagtggtggacagaacactggcaatgctcatggata
cccaccatcgtgcccattcacacaccaccattagttaggctatggtataacctagtgcaggagcccatagaagga
gcagacacgtattacgtagacggtgcagcccaccgagagtcaaaagaaggcaaggcaggatacgtgactgcaaca
ggaaaagaacatgtcatcaagttagaaaaaactaccaaccaaaaggcagaattggaagcagtactattggcactc
aaagattcagggcccaaggtaaatatagtcacagactctcagtatgtctatggcatattggcaggaagccccaca
gaaacagataacaagataatagaagacattatccagctactactaagcaaggaggcagtctacttggcttggctg
ccggcccacaaaggcataggaggaaatgaacaggtggataagttagtaagccaaggaatcaggaagatcctttt
gtagaacagatcccagaagcacaagaagaacatgagagataccataataattggagggatcttaaggcaagattt
aagttacccaccatagtagccaaggctattatagaagcatgccccaaatgtcaagtacaaggagaacctaagaca
ggacagaacaatgcagctgtagggacatggcaaatggattgcacccatttggagggacaaatcatctgtgtagca
gtacacgtggccagtgggtacatagagactaagattctgccaagggagacggggcgggagaccgccctctttctc
ttacaagtggccagcagatggcctataagccacctgcatacagacaatgggccaaactttgtctcagcagaaatg
caggccatggtatggtggctcaaaatagagcactccgcaggagttccatataatcctcaatcccagggagcagta
gaaaacaaaaataaacagctcaagaagacaatcacacaaattagggatgaagtacaatacctgtccacagcagtg
gcacaggcgactttcattttgaattataaaagaagaggggggattaggggatatgtgcccagcagaagcaataatt
aacatgatctacacagaactacaaactacacaactacaaaaccaaattcaaaatttttcggattttaaggtctat
tacagaaagggtgctaatcctctttggcagggaccagctcacctcatttggaaaggagaaggagcggttgtcttg
cgaacagacgaaggagaggtaatcacagtccctagaaggaaggcaaaaataattaagccc
>#35 AY523865 2754 nt
acagtacacatagaaggaagaaaagtccaggtgctaatagatacaggggcagatgataccattatttcagaaaaa
gatatagatttagaggcaccttggaccccaaaaacagtaggggggattaggaggtttcataaatgtcaaatgttac
ccagggatagaaattgcaatggctaataaggtagcagtatcagatctattagtaggagataccccaattaatatt
ctaggaagaaattatttagcaaaaatgggggtatctataaattttccagtattagattataccccagtaaaatta
aaagagggggcagatggaccaaagattaaacaatggccactttcaagggaaaaaatagaggccttaaaagatatc
ttaaagcccatgatagaggcaggacagatagtcccggcagcgcccaccaacccatataactctccagtgtttgta
ataaagaagaaggacaaaagtaagtggcgaatgctaatagacttaagagccttaaacaaggccacacaggaggtg
tgggaggtgcagacaggtataccacacccatctgcactaccacaaatggaacagatcactgtcttagatcttgca
gatgcctattattcaatagcactagacccaacatttgcaaaatacacagcattcacagtaccatctgtcaataat

atacaaccaggagaaagatatgagttcagagtactgcctcagggatggagcgcttcaccagccattttcaagcc
tcagtagggagacagttacagatctttagggaaaagaatccagagctaataatagtccagtatatggatgattta
ctagtagggtcagatttaaggaaagatcaacacctaaagaaggtagcacaattaagacagttcctcctagagagg
ggattgaggacaccacctgagaagtatcaggaagacccaccctttcactggatggggtatgagttacaccctaaa
aaatggcggttgatgccagttaccctaccagatgaagaagaatggacagtgcataaaattcagaagttagtagga
cagttaaattgggcaagccaaatttattcagggataaaaacaaaacacttatgtagggcaatcagaggggtcccg
ggattaacagacccggttaccctctctgaagaagctcaagcagaacttagagagaacaaggaaatcctgaagcaa
gaagtgtcaggagtttactataaagagcaggaaccattaatagcagaattatccaaattagggaaaggacaatgg
ggatatgtgatcagacaacctaagggaatattaaagacaggaaaattctctagagataaaggagctcactataat
gatttccatcaattagctaaggctatgtataaaataggaacagaaagcatagtttttggggaagaataccacag
ttcaggctcccagtggtaaaagaagaatggacaattggtggcataatcactggcaagccgcctggatcccagac
tgggaggcaatacataccccacatctggtcaagctctggtatgagctggtgtcagaacccataccagatgcagac
acctactatgtagatggggcagctaacagggagtccaaattagggaaagctggctatgtcacagagtggggaaaa
caaagtgtaaaatgcttagagaataccactaatcagaaagcagaattagaagccatattgttggccttggaagaa
ggcccatcaaaaatgaatatagtaacagactcccagtatgcactggggataattttagaacacccatctgagaca
gaacataagatagtagaaaaggtaatacaggccttgcaagggaaagaacaaatttatctagcatgggtaccagcc
cacaagggaataggaggaaatgaccaggtagataaattagtcagtaaaggaataaggaggatcctcttcttggaa
agggtagaggaagcccaagaggaccatgaaaaataccatgggaattggaaacaattaaggatgaataccaacta
cctacactcatagctaagcaaattataacacaatgccccaaatgtcagacaagaggggagcccacacatggtcag
gtggatgcctcaatgggcatctggcagatggactgcacccatctggaaaacaaaataataatagtggcagtccat
gtggctagtggatatatagagaccaagatcctaccagcagaaacaggaaaagagacagctctgttcctgttacag
ttaggagcaagatggccagtaactcagatacacacagacaatggacccaactttatcagtcaaccagtcaaggca
gcttgctggtggctaggtattgagcacaccacaggggtaccatataacccacaaagccagggagtggtagaaagt
aagaacaggattctaaaagataccatcaaacaaatcagagatgatgcagagagattggaaacagcagtagcaatg
gctacctacataattaattttaaaagaaggggaggaataggggatatgtctccgatagaaagactagtaaacatg
ataacaacagaaatagaaacaaaaactttacaacaaaaaatttcaaaacttttaggcttcaaggtctattacaga
gaaggtgctgatccaacttggaaagggccagcaaccctgctgtggaaaggagaaggtgcagtggtgtgcaagaca
gaggtaggagatatcaaagtggtccctagaaggaaagcaaaagtaatcaaagaa
>#36 AY523866 2754 nt
gcagtatatgtggaaggaaaaagggtccaggtgctaatagacacaggtgcagatgatacaattatttcagagaaa
gatttagatttagatgcaccttggaccccgaaaacagtgggggattaggggatttatcaatgtaaaatgctac
ccagggatagagattaaaatggcagacaaggtagctgtttcagatgtgttagtaggtgataccccaatcaacata
ttgggaaggaattatttggcaaagatgggggtatccataaattttccagtattggactttacaccagtaaagttg
aaggagggagctgatggcccaaggattaaacagtggcccctttcaagagaaaaaatagaagccttaaaagagatc
ataaggcctatggtagaagcaggacagatagtccccacagagcccactaacccatatagctctcctgtgtttgtt
ataaagaagaaggacaagaataaatggagaatgcttatagacctgagagcccttaataaggctacccaggatgtc
tgggaagtgcaaacagggatcccgcacccctcggggttgccacaaatggaacagataacagtcctagacttggca
gatgcctattattcaataccctggatccacagtttgcacgttatacagcattcacaataccttcggtgaacaat
acacaaccaggagagagatatgagttcagagtactacctcaaggctggagtgcatcaccagccatattccaggca
acagtgggcaggcagctgaaacttttaaggaaaaatacccagacttaattatagtccaatatatggatgacctta
ttggtaggatcaaatttagataaggtctcacatttagaacaggtaaaacagcttaggaaatttctcctagaaaag
gggttaaggacaccccggagaaataccaggaggatccccataccattggatggggtatgagttacatccaaag
aaatggcacttgttgccaatagagctaccagaagaggaggagtggacagtccacaaaatccagaaactggtagga
cagttaaattgggcaagtcagatatactcaggaataaagaccaaacacttgtgcaaggcaatcagaggggttcca
gggctaacagataaagtaagcctttcagaggaagcacaagcagaactgagggagaataaagaaactcccaaacag
gaagtctcaggggtatactacaaagaagatgagcccctaatatcagagatttccaaaataggaagaggacagtgg
ggatatgtaattagacaaacaaagggattgctaaaaacagggaaattctcaaaggacaagggaactcattataat
gactttcatcaactagctaaggcaatgtataaaataggcacagagagtatagtctattggggaaaagttcccaaa
tttaggttgccagtagtaaaggaagaatgggataactggtggcataaccactggcaggccacttggatccctgac
tgggaggcggtgcataccccacaactagtcaggctatggtatgagcttgtatcagagccaataccagatgctgac
acctattatgtagatggagcagctaatagggaatcaaaaatgggaaaagcaggctacatatcagaatggggaaaa
cagggagtaaaaagtctggaaaatactaccaatcaaaaggcagaattagaagccatcctactggccttggaggag
ggaccctccaagatgaacatagtaacagactcccagtatgctttaggaatattgttagaacaccccacggaaaca
gaacatgagatagtagaaaagatcatacaagccttacaagaaaaggaacaggtatatcttaattgggtaccagcc
cacaaaggtataggagggaatgatcaggtagacaaattggtaagtagaggaataaggagaatcctattcttggaa

agagtagaagaggctcaagaggatcatgagaaatatcatggtaattggaaacaattgagagatgaatttcagtta
cctaccttaatagccaagcagattattgcgcagtgtccaaagtgccagataaagggagagcctatgcatggtcaa
gtagatgcctccatggggtatggcaaatagattgcacccacctagaaacaaagtcatcatagtagcagtgcat
gtagctagtggatttatggagactaaggtcttatcagcagaaactggaaaggaaacagccttattcttattacag
ttgggggcaagatggcccattaaacagatacacacagacaatggaccaaatttcataagtcagccagtcaaggca
gcatgttggtggttgggaatagatcatactacaggggtaccatataacccacaaagtcaaggggtagtagaaagc
aaaaatagggtcctaaaagaaactatcaaacaaattagagaagatgcagaaagactagaaacagcagtagcaatg
gccaccttcattattaattttaaaagaaggggaggaataggggatatggcagcaatagagagactagtaaacatg
ataactacagacttggaaacacaaaccttacaacaaaaaatttcaaaacttttaggttacaaggtatattacaga
gaaggagctgacccaacttggaagggacccgctaccttactgtggaaaggagaaggagcggtggtgtgtaaaaca
gaggtaggagacataaaggtagtaccaagaagaaaagccaaggtgataaaagat
>#37 FJ919724 2760 nt
acaatagagatagagggaagaagggtgcaggttctattagatacaggggcagatgatactataattcatgagaaa
gatattcaacttacctcaccctttgtcccaaaaacagtaggggggtctgggaggttttataaatgtaagatgttat
ccagggatagaaatccaatgtcaaggtaaggtagcagtaggggaagtcctagtgggagagacacccattaatatt
ttagggaggaatttcttagcacccctaggcttctcgataaacatgctagagagtcagattcaatataccccagtg
aaattaaaagaagggaagtcaggaccaaaagtcaaacaatgggcctctagccagggaaaagatagaggctctcag
ggaaatagtgggaaagatggtggaggaaggccaatggctcctgcctcacccaccaatccatataactcacctgtg
tttgccattaaaaagaaagacaagaacaaatggagaatgttaatagatcttagagaattgaacaaggcaacacaa
ccagtttgggaggtccagacagggatcccgcacccagcaggtctgccacagaaaaggcagataacagttttggac
atagcagatgcatactattctataccattagatccatcctttgctcagtatacggcatttactataccatcagtt
aataacacagcccctggagagaggtatgagttcagggtgctgccacaaggatggagtgcatcaccagctatattc
caacacactgttcatcgggtattggaaggatttagaaagaaatacccagatgtactcatagtccagtacatggat
gacctattggtagggtcagacagaacagaagaacaacacagactagtagtcaaaacactaagagattatctccta
gagaggggggttaaaaacacccccagagaaatttcaggcagagccccccctatcagtggatgggatatgtccttcat
ccaaaaaagtggactacccaaccaattcagatccctgaggaagaggaatggactgttaataaaatacagaaacta
gtaggaatcttgaattgggcaagccaaatatattcagggataaaaacaaaacacctgtgcaagagtattagaggg
gttccagggttaacagaccctgtaaagttaacagaagaggcacaggccgagctggaagaagccagacagatctta
aaagaacaagtgtcagggacctattacaaagaacaggaggacctcatagcagacattactaagctttcagaaggg
caatggggatacaccattagacagtcaaagggaatcctgaaaacaggcaaatatgcaaaggcaaaaggggcacat
ttcaatgattttcatcagatagcaaaattgatgatgaaggtaggaacagaaagcatagtaacatggggaggtta
cccacatttaggttacctgtccagaagcaggactgggatggatggtggcatgagcattggcaagccacttggatc
ccagaatgggaggcggtacacactcctcccctagtcagattatggtattccttggtttcagaacctataaaagaa
gcagatacatattatgtggatggagcagctaacagagaatccaaggaagggaaagcagggtacatttcagagaga
ggaaaacagagtgtaaaggccttagaaaacactaccaatcaaaaggcagaattaacagcagttctcatggcctta
gaagatagtggggaaaaggtgaacatagtaacggactcccagtatgtactaaacatactaacagaacaccccaca
accactgaacatgaattagtagagaaaataattcaacaactacagaagaaacaggaagtatatcttcagtgggtg
ccagctcataaaggtctgggaggtaatgaacaaatagataaattagttagtaagggaattagaaagatcctttt
ctagaaagagtagaagaggctcaggaagatcacgataaataccacagtaattggaaacagctaagggaggagttt
aatctccccaccttagttgcaaagcaaataattaatcaatgtcccaaatgtcaaatacatggggaacccaaacat
ggacaagtcaatgcagacttgggtatttggcaaatggactgcacacatttagaaggaaaagtcataatagtagca
gttcatgtggctagtggatttacagaaacccaggtgttaaaggaggaaacaggaaaacagacagctttattcctg
ttacaattaggggctaggtggcccatacaacaagttcacacagacaatggacccaactttattagtcaggcattt
gctgccgcctgttggtggttagggatagagcacaccacaggggttccatacaacccacagagtcaaggagtagta
gaaaacaaaaatagacaattaaaggatacaatcacacaagttagggaagatgcacaacgattggaaacagcagta
gctatggctactcacattcttaattttaaaagaaggggaggaataggggatatgaccccttcagaaaggatagtc
aatatgattaatacagaactagaaataaaatacctacaacaaaaaatttcaaaattttcaggattcaaagtctac
tacagagaaggagctgatcccagttggaaaggcccagcaacactcctgtggaaaggagaaggagcagtggtaatc
aaattggacacaggggatttaaaagtagttcccagaagaaaagcaaaagtaataaaggac
>#38 AF208027 2751 nt
actgcatacatagaaagtcagccagtagaagtactattagacacaggggctgatgactctatagtagcaggaata
gaattaggaagtaattatagtccaaaaatagtaggaggaataggaggatttattaataccaaagaatacaaaaat
gtagaaattaaagtggtaggaaaaagaatacaggctactgttatgacaggggataccccctataaatatctttgga
aggaacattttagttaagctaggcatgtctctaaattttccaattgcaaaggtagagccagtaaaagtaaggcta
aaacctggcatggatgggccaaaaatcaggcaatggcccctatctaaggaaaaaatacaagcactcaggaaatt

tgtgacaaaatggaacaggaagggcaattggaagaagcacccccccaccaatccgtataacacacccacctttact
atcaagaagaaggacaaaaacaaatggagaatgttaatagacttcagagaacttaacagggtaacccaggacttt
actgaagtccagttgggcatacctcaccctgcaggtttggcagaaaaaaggagaataacagtactggatataggg
gatgcctacttcagcattcctctagatgtagatttcaggcagtacacagctttcactttaccatcagtaaacaat
gcagaaccaggaaaaagatacatctacaaggtcctaccgcaaggatggaagggctcaccagctatcttccaacat
acaatgaggaaggtactagaccccttcagaaaagccaatgaggatrtaattatyatccaatatatggatgacatt
cttgtggccagtgacagaactgatttagaacatgatcaagtagttttgcagctaaaggaactcttaaataaaacr
gggttctctacccctgatgaaaagttccaaaaagaccctccatatcaatggatgggatatgaattrtggccaaaa
aagtggaaactacaaaagattaacctgccmgaaaaagagacttggacagtaaatgatattcaaaaactagtaggg
gtactgaaytgggcagctcagctatatccaggaatcaaaacaaaaaacctgtgyaaattaattagaggtaaratg
accttgacggaagaagtacagtggacagaattggcagaagcagaattgcaagaaaacaagattatcctaraacag
garcaggaaggcgcctattacaaagaagragagcctttrgaagccactgtacagaaaaatttagacaatcagtgg
acatacaaagttcatcaaggaaacagaatcctaaaagtaggtaaatatgcaaagataaaaaatacccatacaaat
ggagtaagattattggctcatgtggtccaaaaratagggaaaggaagcactagtaatatggggaaaattacccttc
tttcatctgccagtagaaaggggacacctgggaacaatggtggacagactattggcaagtaacttggatcccagat
tgggactttgtgtctacaccgcatctagtaaggttagtctacaacctagtcaaagaacctctagagcagacagag
acctattatacagacgggtcctgcaataaaacctccaaggarggaaaagcaggctacgtcacagacagaggaaaa
gacaaggtaaargtcttagagcagacaacaaaccagcaggcrgaacttgaggcatttgcaatggccttgcaggac
tcaggtccaaaagtcaatatcatagtrgattcccagtatgtcatgggratcatagcaggrcaaccaacggaaaca
gaatccccgttagtaaataaaataattgaagaaatrattaaaaaagaarctctatatgtaggatgggtaccagct
cataaaggaataggaggtaatcaagaggtagaccrtctagtaagccagggaattagacaagtactattcttgraa
aaaatagaacctgctcaagaagaacatgaaaarttttcatagtaatgtaaaagagttaactcataagtttggcatt
cctcaattagtagcaaaacagatagtaaattcataccacagttgccaacagaaaggagaagccattcatggacag
gtaaatgcagaattgggtacttggcaaatggactgcacccatttagaaggaaaagtaatcatagtagcagttcat
gtggccagtggctttgtagaagcagaagtaatcccacaagaaacaggaaggcaaacagcattattcctgttaaaa
ttagcagggagatggcctatcacacacctacatacagacaatggtgccaacttcacctcgcaagaggtgaagatg
gtagcctggtgggtaggcatcgagcaagcatttggagtaccatacaacccccagagtcaaggggtagtagaatca
atgaatcatcatctaaagaaacaaatagataaaattagagatcaagcaaattcaatagaaaccatagtgttaatg
gcagtacattgcatgaattttaaaagaaggggaggaatagggggatatgacccctgcagaaagaataattaacatg
attactacagaacaagaaatacaatttcaacaaacaaaaaattcaaaatttaaaaattttcgggtctattacaga
gaaggcagagaccagctctggaaaggacctggtgagcttttgtggaaaggggaaggagctgtcatcataaaggta
gggacagaaatcaaagttgtacccagaagaaaagcaaaaatcataaaagat
>#39 M30502 2559 nt
atgacaggagataccccaatcaacatctttggcagaaatattctgacagccttaggcatgtcattaaatttacca
gttgccaagatagagccaataaaagtaacattgaagccagggaaagatggaccaaggctgaaacaatggccccta
acaaaagagaaaatagaagcactaaaagagatctgtgaaaaaatggaaaaagagggccagctagaagaggcacct
ccaactaatccttataatacccccacatttgcaattaagaaaaaggacaagaacaaatggaggatgctgatagat
tttagagaactaaataaggtgactcaagatttcacagaaattcagctaggaattccacacccggcaggactagcc
aaaaagaaaaggatctctatattagatgtaggggatgcctattttccataccactacatgaagattttaggcag
tatactgcatttaccctaccagcagtaaacaatatggaaccaggaaaaagatatatatataaagtcttgccacaa
ggatggaagggatcaccagcaattttttcaatacacaatgaggcaagtcttagaacctttcagaaaagcaaaccca
gatgtcattctcatccagtacatggatgatatcttaatagctagtgacaggacaggtttagagcatgacaaagtg
gtcctgcagctaaaagaacttctaaatggcctagggttttctactccagatgagaagttccaaaaagaccctcca
tttcaatggatgggctgtgaactatggccaactaaatggaagctgcagaaactacaactgccccagaaagacata
tggacagtcaatgacatccaaaagctagtgggagtcttaaattgggcggcacaaatctattcaggaataaaaacc
aaacacttatgtagactaattagaggaaaaatgacactcacagaagaagtgcagtggacagaactagcagaagca
gagctagaagaaaacaaaattatcttgagccaggaacaagaaggatattattaccaagaagaaaaagaattagag
gcaacaatccaaaaaagccaaggacatcaatggacatacaaaatacaccaggaagagaaaatcctaaaagtagga
aagtatgcaaagataaaaaatacccataccaatggggtcagattactagcacaggtagttcagaaaataggaaaa
gaggcactagtcatttggggacggataccaaaatttcacctgccagtggagagagagacctgggagcagtggtgg
gataactactggcaagtgacatggatcccagagtgggactttgtatctaccccaccactggtcaggttaacattt
aacctagtaggagatcctataccaggcgcagagaccttctacacagatggatcatgcaatagacagtcaaaagag
ggaaaagcaggatatgtaacagatagaggaaaagacaaagtaaaagtattagaacaaactaccaatcagcaggca
gaattagaagtctttcggatggcactggcagactcaggcccaaaggttaatatcatagtagattcacagtatgta
atggggatagtagcaggccagccaacagagtcagaaaatagaatagtgaaccagatcatagaagaaatgataaag

aaggaagcagtctatgttgcatgggtcccagcccataaaggcataggaggaaaccaggaagtagaccatttagta
agtcaaggcatcagacaagtattattcctggaaaagatagagcccgctcaagaggaacatgaaaaatatcatagc
attataaaagaactaacccataaatttggaataccccttctagtagcaagacagatagtaaactcatgtgcccaa
tgccaacagaaaggagaagccatacatgggcaagtaaatgcagaaataggcgtttggcaaatggactacacacac
ttagaaggaaaaatcattatagtagcagtacatgttgcaagtggattcatagaagcagaagtcatcccacaggaa
tcaggaaggcagacagcactcttcctattaaaactggccagtaggtggccaataacgcacttgcacacagacaat
ggccccaacttcacttcacaggaagtgaagatggtggcatggtgggtaggtatagaacaatcctttggagtacct
tacaacccacaaagccagggagtagtagaagcaatgaatcaccacctaaagaatcagataagtagaattagagaa
caggcaaatacaatagaaacaatagtactgatggcagttcattgcatgaattttaaaagaaggggaggaataggg
gatatgaccccagcagaaagactaatcaacatgattaccacagaacaagaaatacaattcctccaaagaaaaaat
tcaaattttaaaaatttccaggtctattacagagaaggcagagatcagctgtggaaaggacctggtgaactactg
tggaagggagaaggagcagtcatagtcaaggtagggacagacataaaagtagtaccaagaaggaaggccaagatt
atcagggac
>#40 AF082339 2751 nt
acagcctacattgagggccagccagtggaagttttactagacacaggggctgacgactcaatagtagcaggaata
gagttagggagcaactataccccaaaaatagtaggggggaataggggggattcataaataccaaagaatatgaagat
gtagaaataaaagtactaaataaaagagtaaaagccaccataatgacaggtgacaccccaatcaatatttttggc
agaaacattttgacagccttaggcatgtcattaaacctaccagttgccaagatagagccaatagaggtaagatta
aagccaggaaaagacgggccaaaattaagacaatggcccttaacaaaagaaaaaatagaggcactaaaagaaatc
tgtgaaaaaacggaaagagaaggccaattagaggaggcacctccaactaacccttataataccccccacatttgca
ataaagaagaaggacaaaaacaaatggagaatgctaatagattttagagaattaaacaaggtaactcaagatttc
acagagattcagttagggattccacatccagcaggattagccaagaaaagaagaatcactgtgctggatgtaggg
gatgcttacttttccataccactgcatgagagctttagacagtatactgcatttactctaccatcagtaaacaat
gcagaaccaggaaaaagatatatatataaagtcttaccgcagggatggaagggatcaccagcaattttcaacac
acaatgagacagatcttagagccattcagaaaggcaaaccaggatgtcattctcattcaatacatggatgatatc
ttaatagctagtgacaggacagatttagaacatgacaaggtggtcctgcagttaaaggaactcctaaatggccta
ggattttccaccccagatgagaagttccaaaaagaccctccatacaaatggatgggctatggactgtggccaact
aaatggaagctgcaaaaaatacaattgccccagaaagaagtatggacagtcaatgacatccaaaaacatgtgggt
gtcctaaattgggcagcacaaatctacccaggaataaagaccaaacacttatgtagactaattagaggaaaaatg
acactcacagaaggagtgcagtggacagaactagcagaagcagaactagaggagaacagaattatcttaagtcag
gaacaagaggggcactattaccaagaagaaaaggagttagaagcaacagtccaaaaagatcaagacaatcaatgg
acatataaaatacaccagggagaaaaaattctaaaagtggaaaagtatgcaaaaatgaaaaatacccataccaac
ggggtcagattgttagcacaggtagttcaaaaaataggaaaagaagcactggtcatttggggacgaataccaaga
tttcacctaccagtagaaagagaaacctgggaacagtggtgggatgactactggcaagtgacatggatcccagac
tgggactttgtatctaccccaccactggtcaggctagcatttaacctagtaaaagatcctatactaggcgcagag
accttctacacagacgggccctgtaataggcaatcaaaagaaggaaaagcaggatatataacagatagagggaga
gacaaggtgaaggtactagaacaaactaccaatcagcaagcagaattagaagccttcgcgctggcagtaacagac
tcaggtccaaaagccaatattatagtagattcacagtatgtaatgggaatagtagcaggccagccaacagaatca
gaaaatagaatagtaaatcaaatcatagaagaaatgataaaaaaggaagccatctatgttgcgtgggtcccagcc
cacaaaggcataggggggaaatcaggaagtagaccatttagtaagtcagggcatcagacaagtattgtttctagaa
aaaatagagccagctcaggaagaacatgaaaagtatcatagcaatgtgaaagaactatcccataaatttggatta
cccaatctggtggcaagacagatagtaaacacatgtgcccaatgtcagcagaagggagaggctatacatgggcaa
gtgaatgcagaactaggcacttggcaaatggactgcacacacttagaaggaaaagtcatcataatagcagtgcat
gttgccagtggattcatagaagcagaggtcatcccgcaggaatcaggaagacaaacagcactcttcctattaaaa
ctggctagtagatggccaataacacacttgcacacagatagtggtgtcaacttcacctcacaggaagtaaagatg
gtagcatggtgggttggtatagagcaatcctttggagtaccttacaatccacaaagccaaggagtagtagaagca
atgaatcaccacctaaaaaatcagataagcagaattagagagcaggcaaatacagtggaaacaatagtactaatg
gcagttcattgcatgaattttaaaagaaggggaggaataggggatatgaccccagcagaaagactcatcaatatg
atctccacagaacaagaaatacaattcctccaaacaaaaaatttgaaatttaaaaatttcccggtctattacagg
gaaggcagagatcagctgtggaaaggacctggggagctactgtggaaaggggacggagcagtcatagttaaggta
gggacagacataaaagtagtaccaagaaggaaggccaagatcatcagagac
>#41 U27200 2751 nt
aaagcaactattgagggtcaatcagtagaagtattactagacacaggagctgatgactcaatagtagcagggata
gaattaggcagcaattacaccccaaaaatagtaggtgggataggaggatttataaataccaatgaatacaaaaat
gtagaaatagaagtagtaggaaaaagagtaagagcaacagtaatgacaggggacaccccaataaacatttttggc

agaaatattttaaatagcttaggcatgactctaaatttcccagtagcaaggatagaaccagtaaaagtccagtta
aagcctgaaaaagatgggccaaaaatcagacaatggcccctatccaaagagaaaatactagccctcaaagaaatc
tgtgaaaaaatggaaaaagagggacagttagaagaggcgcctcctactaatccatacaattcgcccaccttcgcc
ataaaaaagaaagacaaaaacaaatggaggatgctaatagatttcagagaactaaacaaggtaacccaagaattt
acagaggtccagctgggtattcctcacccagcaggactggcatcaaagaaaagaataacagtactagatgtagga
gatgcctacttcagtgtcccactagatccagacttcagacaatatacagcatttactttgccagcagtaaataat
gcagaaccaggaaagagatatctttacaaagtcctaccacagggatggaagggatccccagcaattttccagtac
accatggcaaaggtactagaccctttcagaaaagccaacaatgatgtcactataatccagtacatggatgacatt
ctcgtggcaagtgacaggagcgatctggagcatgacagggtagtgtctcaactaaaagagctattaaataacatg
ggattctctactccagaagaaaagttccaaaaagaccctccattcaaatggatggggtatgagctctggccaaag
aaatggaaactgcaaaaaatacagctaccagaaaaagaggtttggacagtaaatgacattcagaagttagtggga
gtattaaattgggcagctcaacttttcccggggattaagaccaggcatatatgtaaactaataaggggaaagatg
accctaacagaagaggtacaatggactgaattggcagaggcagaattccaggaaaacaaaatcatcctagaacaa
gagcaggaaggatcctattacaaagaaggggtacctttagaagcaacagtgcagaaaaatctagcaaatcagtgg
acatacaagattcatcagggagataaaatcctaaaagtaggaaaatatgcaaaggttaaaaacactcacaccaat
ggagtaagactattggctcatgtagtccaaaaaataggaaaggaagcattggtcatctggggagagataccaatg
ttccatctaccagtagaaagagagacatgggatcagtggtggacagattactggcaagtaacctggatcccagaa
tgggattttgtctcaaccccaccattaataaggttagcctataacctggtcaaagacccccctagaaggagtagaa
acttactacacagatggatcctgtaacaaagcctcaaaagaagggaaagcaggatatgtcacagacaggggaaag
gataaagttaaaccattagaacaaacaacaaatcagcaagcagagcttgaagcatttgcactagcactacaggac
tcaggaccacaggtcaatatcatagtagattcacaatatgtcatgggaatagtagctgcacaaccaacagaaaca
gaatcaccgatagtaagagaaataattgaagaaatgatcaaaaaggaaaaaatatatgtaggatgggtaccagct
cacaagggactgggtggtaatcaggaagtagaccacctagtgagccaaggaattagacaaatcctatttctagaa
aaaatagaaccagctcaagaagaacatgaaaaatatcataataatgtaaaagaactagtccataaatttgggatt
ccacaattagtggcaagacaaatagtaaattcctgtgataaatgccaacaaaaaggggaagctattcatggacag
gtaaattcagaactagggacatggcaaatggactgtacacatttagagggaaaggttataatagtggcagttcat
gtagccagtggattcatagaagcagaagtaataccccaagaaacaggaagacagacagctctcttcctgttaaag
ctggccagcagatggcctatcacacacctgcacacagacaacggtgccaacttcacttcacaagatgtgaaaatg
gcagcctggtggatagggatagaacaaacattcggagtgccctataatccagaaagtcagggagtagtagaagca
atgaaccatcatctgaaaaatcagatagacagaattagagatcaggcagtatcaatagagacagttgtgttaatg
gcaactcactgcatgaattttaaaagaaggggaggaataggggatatgacccctgcagaaagaatagtcaacatg
ataactacagaacaagaaatacaattcctccaaacaaaaaatttaaaattccaaaatttccgggtctattacaga
gaaggcagagatcaactctggaagggacctggtgatctattgtggaaaggggaaggagcagtcatcataaaggta
gggacagaaatcaaagtaatacccagaagaaaagcaaagatcataaggaac
>#42 KP004991 2754 nt
acagcgaaaatcgggggccatctatgtgaagttttgctggatacaggggcagatgatacagtgctacatgacata
caattagaaggaaaatggacaccaaaaatgataggggggtataggggggttttataaaggtgaaagaatttaacaat
gtgacagtacaaatagaaggcagggaagttcagggaacagtattggtgggacctactcctgttaatattgttgga
agaaacatattgacagggttgggttgcacattaaatttccccataagccccatagccccagtgccagtaaaacta
aaaccaggaatggatggaccaaaagtaaaacaatggcccctatctagagaaaaaatagaagcattaacagcaata
tgtcaggaaatggaacaagaaggaaagatatcaagaatagggcctgaaaatccttataatacacccatatttgct
ataaaaaagaaagatagcactaagtggagaaaattagtagatttcagagagttaaataaaagaacacaagatttc
tgggaggtacagttaggtatcccacatcctggggtttaaagaaaaagcaatctgtcacagttttagatgtagga
gacgcctatttctcatgtccattagacccagatttcagaaaatatacagcttttactattcctagtgtaaacaat
gagacaccaggagtgagataccaatacaatgtcctcccgcaaggatggaaaggctctccagctatatttcaaagt
tcaatgactaagattctagatccattcagraaagacaatccagaaatagaaatttatcagtacatggatgacctc
tacgtagggtcagatttaccactagcagaacatagaaggaaagttgaggtgcttagggagcatctataccagtgg
ggatttactaccccagayaaaarcaccaaaaggagcctccctttttgtggatgggctatgagctccacccagat
aaatggacagtacagcccatcaaattgcctgacaaagaagtatggacagtaaatgatatacaaargctggtagga
aaattaaattgggcaagtcaaatctatcaaggaatcagagtaaargaactatgtaagttgattagaggaaccaag
tcattracagaagtagtacctctaagcaaagargcggaactagaactagargaaaatagggaaaggctaaaggaa
ccagtgcatggggtatattaccaacctgacaaagacttatgggttaatattcaaaaacarggagaagggcaatgg
acgtaccaggtctatcaagatgagcataaaaaccttaaaacaggaaaatatgctaggcaaaaagtctcacataca
aatgatataagacaactagcagaagtaattcagaaagtgtctcaagaatctatagttatttggggaaagttgcct
aagtttaagctgccagttactagagaaacttgggaagcatggtgggcagactattggcaagccacctggattcct

gaatgggaatttgttagtacacctccattgatcaaattatggtatcaattagaaagtgagcctatttcaggggca
gagacatactatgtagatggggcagccaatagggacacaaaattaggaaaagcagggtatgttacagaacaagga
aaacagaagataatcaaattagaggagactactaatcagaaagctgaattaatggcagtcttagtagcactgcaa
gactccaaaaaagaagtaaatatagtaacagactcgcagtatgtattaggtgttatctcagcccaacctacacaa
agtgattccccccctagttcagcagataatagaggaattgacaaaaaaggaaagagtataccttgcctgggttcct
gcccataagggcataggggaaatgaaaaaatagacaaactagtaagtaaagatattagaagagttctattccta
gaagggatagaccaggcacaagaggatcatgaaaaatatcatagcaattggagggcgttggccagtgactttgga
ctgccaccagtggtagccaaagaaatcattgctagctgccctaaatgtcatgtgaaaggggaagctatgcatggt
caagtagattgcagtccagggatatggcaactggactgcacgcatttagaaggaaaaattatattagtggcagtt
catgtggccagtggcttcatagaagcagaagtaatccctgcagaaacaggacaggaaactgcttacttcctgctt
aaattagcagcaaggtggccagtcaaagtaatacacaccgataatggaccaaactttaccagtgctgcagtaaag
gctgcttgttggtggcttaatataactcatgaatttggaattccatataatccacaaagtcaaggagtagtagaa
tctatgaataaagagttaaagaaaataatacagcaagttagagatcargcagagcagttgaaaacagcagttcaa
atggcagtatttgtccacaattttaaaagaaaaggggggattgggggtacactgcaggagataggataatagac
ataatagcttcacaaatacaaacaacagaattacaaaaacaaattttaaaaattcaaaattttcgggtctattac
agagacagcagagaccctatttggaaaggaccggcacagctcctgtggaaaggtgagggagcagtagtcatacaa
gacaagggagacattaaggtagtaccaagaagaaaggcaaaaataatcagggat
>#43 FJ424866 2754 nt
cctgtgaagataggggacatctatgtgaggctttgttagacacaggggctgatgatacagtagtagaacactta
tctttagaaggtaggtggaaaccaagaatgataggaggcataggaggatttattaaagtaaaagaatatgaagat
gtagaaatagaaatagaacataggaaagtagtagggacagttctagttggaccaacccctgtaaatattatagga
aggaatattttaacaaaaattggatgtaccctaaattttcccataagctccatagatgtagtacccgtatcttta
aagccagggatggatggacccaaagtcaagcaatggcctttgtcaaaagaaaaaatagaagccttaacagctatt
tgtcaggaaatggaacaggaaggcaagatatcaaaaattgggcctgaaaatccatataatacacctattttgcc
ataaaaaagaaagacagtactaaatggagaaaattggtagattttagagaattaaataaacgaacacaggatttt
tgggaagttcagttagggattcctcacccaggagggcttaaaaagaaaaaatcagtaacagtactggatgttggg
gacgcttatttttcttgtcccttagacccagattttagaaaatatacagctttcacaattcctagtgtgaataat
gagacaccaggggtaaggtatcagtacaatgtactaccccagggatggaaaggatctccagcaattttccagcac
tccatgacaaaaatacttgatcctttcagaaagaaaaacccagagatagaaatctatcagtacatggatgacttg
tatgtagggtcagatttacctataacagaacataggcagaaggtagaaaaactcagggaacatttatatgcatgg
ggattcaccactcctgacaaaaagcatcaaaaggaacctccctttttgtggatggggtatgagctgcaccctgat
aagtggacagtgcagcctataaagttaccagaaaaggaaagttggacagtcaatgatatccagagattggtaggt
aaactaaattgggctagtcaaatatatccagggataagagtaaaagaactctgcaaattaattaggggtactaaa
tcacttacagaagtagtgacccttagtaaagaagcagaattagaactagaagagaacagggaaatattaaaagaa
cccgtacatggagtgtattaccagcctgaaaaggaattaatagtgaatgtgcaaaaacaaggagaaggacagtgg
acttatcagatcttccaggatgaatacaagaatctaaagacaggaaagtatgctagacaaaaggccactcatact
aatgacattagacagttggcagaagtgatacaaaaggtctcacaagaaagcatagttatctggggaaaactgcct
aagttcaggctacccatatgtcggagtacctgggacgcttttggactgactattggcaggcttcctggatccct
gagtgggaatttattagtacacctcccctcatcaagctctggtatcaattggaaaaggaacccataccaggagca
gaaacctttatgtagatggagcagctaatagagaaagtaaacaaggaaaagcaggatatgtaacagataagggt
agacagaaaataagaaaactagagggcacaactaatcagaaagcagaactagaggcagtcctcttggccttagaa
gaatcaggaaaaaatgttaacatagtgacagactcccagtatgtactagggattattgcagcaagtcctgatcaa
agtgactccccccttagtgcagaaaataatagaggaaatgacaacgaaggaaaaagtctatttagcttgggtacca
gctcacaaaggtataggggaaatgaagaaatagacaaattagtcagtaaagatatcagaagagtcttattccta
gaaggcatagatcaagcgcaggaagatcatgaaaaatatcacagtaattggagaacactagccagtgactttggt
ctgccgccagtggtagccaaagaaatcatcaatagctgccctaagtgccatgtaaaggggaagctaggcatgga
caagtagattgcagcccaggtatctggcagttggattgcactcacttagaagggaaaatcattttagtagcagtc
catgtggccagcggcttcatagaagctgaagttattccagcagaaacagggcaggaaacagcctacttcctgctc
aagttagcagctaggtggccagtaaaaataatacacacagacaatgggcctaattttactagtgccacagtcaag
gctgcctgttggtggctcaatgtaacacatgagtttggaattccctataatccccaaagtcaaggagtagtggaa
tctatgaacaaagaattaaaaaagattatacaacaagtgagagatcaagcagaacatttaaagacagcagtgcag
atggcagtatttgtccacaattttaaaagaaaaggggggattgggggatacactgcaggagacaggataatagac
atgttagccacacaaatacaaacaacagaattacaaaaacaaattttaaaaattcaaaattttcgggtctattac
agagacagcagagaccctatttggaaaggaccagcgacgctcctgtggaaaggtgaaggggctgtagtcattcag
gacaaaggggacattaaagtagtccctaggagaaaggcaaaaataattagggat

>#44 KP004990 2754 nt
cctgtaaaaatagggggacatatttgtgaagctttattagatacaggggctgatgacacagtagtagataaaata
ccattagaaggaaggtgggtaccaaaaatgataggaggaataggaggatttattaaagtaaaagagtttgaaaat
gttaaaatagaaatagaaggtagagaagttcatgggacagtattagttggcccgaccccagttaatataatagga
aggaacattttgacatccattggctgtactttaaattttcctatcagccccatagaagtagtaccagtaaaattg
aaaccaggcatggacggcctaaggtgaagcaatggcctttatcaaaagagaaaatagaagcattaactgctatc
tgccaagatatggaacaggaaggaaaaataacaagaattgggcctgaaaatccctataacacccctatttttgca
ataaaaaagaaagatagcagtaaatggagaaagctagtagattttagagagttaaataaaagaactcaagatttt
tgggaagtccagctgggaatccctcacccaggaggtcttaagcagaagaagtcagtaacagtactagatgtggga
gatgcttatttctcctgtcccttagatccagatttcaggaagtacacagcttttactatacctagtgtaaataat
gaaacaccaggggtcagataccagtacaatgtgttacctcaagggtggaagggatcaccagccatcttccaacat
tccatgaccaagatcctagatccattcaggaaaaataatccagaaatagaaatttatcagtacatggatgattta
tatgtagggtcagacttgccactatctgaacataggcaacgagtcgaaaagcttagagaacatctctatgcttgg
ggatttacaactcctgataaaaaacatcagaaggaacccccttttcctttggatggggtatgaactccaccctgac
aagtggacagtgcaacccattaagttgccagaaaaagaaagctggacagtgaatgatatccagggactagtaggg
aaactaaattgggcaagtcaaatttatccaggaataaggataaaagagttatgcaaactcataaggggaactaaa
gctttaacagaagtagttaccttcactagagaagcagagttagaactagaagaaaataaagaaatcttaaaagaa
ccaaggcatggagtttattaccaaccagaaaaagaattaatagtagatatacagaaacaaggagagggacaatgg
acttatcagatatttcaggaagaacataagaatttgaagacagggaaatatgccaggcaaaaggctactcacacc
aatgacataaggcaattggcagaagtaatacagaaggtatcacaagagagcatagtaatatggggggaagctgccc
aaattcaggttaccagtaaatagaaccttgtgggaagcctggtggtcagattattggcaagccacctggatacca
gagtgggagtatgttagcacaccccctcttattaaactctggtaccagctagaaaaggaccccataccagagaca
gaaactttttatgtagatggggcagcaaatagagaaaccaagctaggtaaggctggctatgtaacagatagggga
agacaaaaaatagtcaaactagaaggaaccaccaatcaaaaagcagaactagaagcagtgctattagccttaaaa
gaatcaggaaagcaggctaatatagtaacagactctcagtatgtgttagggattattgcagcaactccagatcag
agtgactccccccctagtacaaaagataatagaagaaatgacaaaaaaagaaaaggtgtacctatcatgggtacca
gcacacaaaggcataggggggtaatgaagaaatagacaaattagtcagtaaagacatcaggagagtcttattttta
gaaggcatagatcaggcgcaggaagatcatgagaaatatcatagtaactggaggaccttagccagtgactttggg
cttccaccaatagtggcaaaagagattattaacagttgccctaaatgccatgtaaagggagaagccatgcatggg
caggtggactgcagtccaggaatatggcaattggactgtacccacacagaaggaaaaatcatccttgtagcagtc
catgtggccagtggcttcctggaagctgaagtaattccagcagaaacaggacaggaaactgcatacttcctgctt
aagctagcagcaagatggcctgtaaaagccatccacacagacaatgggcctaacttcgctagtgctgcagttaaa
gcagcttgttggtggctcaacataactcatgaatttgggataccctataatccccaaagtcaaggagtagtagaa
tccatgaacaaggaattaaagaaaattatactgcaagttagggaccaagctgaacatttaaaaacggcagttcaa
atggcagtatttgtccacaattttaaaagaaaaggggggattggggggtacactgcaggagataggattatagat
ataatggctacacaaatacaaacaacagaactacaaaaacaaattttaaaaattcaaaattttcgggtctattac
agagacagcagagaccctatttggaaaggaccagcgacgctcctgtggaaaggtgaaggggcagtagtcatacaa
gataagggagacattaaagtagtccctagaaggaaagcaaaaatcattagggat
>#45 KP004989 2754 nt
ccagtaaagatagggggacatatttgtgaagctttattagacacaggggctgatgacacagtagtagataaaata
cctttagarggtagatggaagccaaaaatgataggggaataggaggttttattaaagtaaaagaatttgaraat
gttaaaatagagatagaagggagagaagtttctgggacagtattagttggcccaaccccagtaaatatagttgga
agaaatatcctaacattagtgggttgtactctaaattttcccattagccctatagaagtagtaccagtaaaattg
aagccaggaatggacggcctagagtaaagcaatggcctctatcaaaagaaaaaatagaagctttaactgctatc
tgtcaagagatggaacaagagggaaaaataacaaaaattgggcctgaaaatccatataacacccctatctttgca
attaaaaagaaagatagcagcaaatggaggaaattagtagacttcagggaattaaataaaagaacacaagacttc
tgggaagtccagctgggaatccctcacccaggtggtcttaaacaaaggaaatcagtaacagtgttggatgtggga
gatgcttatttctcctgtcctttagatccagattttcggaagtatacagcttttacaatacctagtgtgaataat
gaaacaccaggagttagatatcagtacaatgtacttcctcagggctggaarggatctccagccattttccagcac
tcaatgactaaaatcctggaacccttttagaaaagagaacccagaaatagaaatttatcagtacatggatgattta
tatgtagggtcagatttgcctctgcctgaacataggcagcgrgtagagaagcttagagaacatctctatgcatgg
ggrtttacaactcctgataagaaacaycaaaaggaacctccattcctctggatgggctatgagctccatcctgat
aaatggacagtacaacccatcaagctaccagaaagagaaagctggacagtaaatgatatccagaaattagtaggc
aaattaaattggccagtcagatatatccaggaataagagtaaaagagttatgcaaactaattagaggactaaa
gccttaacagaggtgattcctcttactagagaagcagaattagagttagaagaaaataaagaaatcttaaaggaa

ccagtgcatggrgtctattatcagccagaaaaagatttaatagtagatatacaaaaacaaggagagggacaatgg
acttatcagatatttcaggaagaacataaaaacttgaaaacwggaaagtatgctaggcagaaggctacccatacc
aatgatataaggcaattggcagaagtaatacagaaagtatcacaagaaagtatagtaatatggggaaagctrccc
aaatttaggttaccagtaaataggactttatgggaagcctggtggtcagactattggcaggccacctggatacct
gagtgggaatttgttagcacacccctctcattaagctttggtatcaattggaaaaggaccccataccaggaaca
gaaacttttatgtagatggggcagcaaacagagaaaccaagctaggtaaagctggatatgtgacagataggggt
aggcagaaaatcattaaattggaagaaacaactaatcagaaagcagaattagaagctgtactattagccttaaaa
gaatcaggaaaacaagctaacatagtaacagactcccaatatgtattagggattatctcagcaactccagatcaa
agtgactccccctagtgcagagaataatagaagaaatgacaaaaaaggacaaggtgtacctttcatgggtacca
gcacataaaggcataggggtaatgaggaaatagacaaattagtcagcaaggacattagaagggtgttatttcta
gaaggcatagatcaggcacaagaagatcatgaaaaataccacagcaattggaagaccctagctagtgactttggg
cttccaccaatagtagcaaaagagatcatcaatagttgccctaaatgtcatgtaaaaggagaagccatgcatgga
caggtagattgcagtccagggatatggcagttagattgtacccacacagaaggaaaaattatccttgtggcagtc
catgtagccagtggcttcctggaagccgaggtaattccagcagaaacaggacaggaaactgcatactttctgctc
aagttagcagcaagatggcctgtgaaagtcatacacacagataatgggcctaacttcactagtgctgcagtaaaa
gcagcatgttggtggctcaacataacacatgagtttggaataccctacaatccycaragtcaaggagtrgtagaa
tctatgaacaaagaattaaagaagattatacttcaagtacgagaccaggctgagcatttaaagacagcagttcaa
atggcagtatttgtccacaattttaaaagaaaagggggattgggggtacactgctggagacagaatcatagat
atgatggctacacaaatacaaacaacagaactacaaaaacaaattttaaaaattcaaaattttcgggtctattac
agagacagcagagaccctatttggaaaggaccagcgacgctcctgtggaaaggtgaaggggcagtagtcatacaa
gataagggagacattaaagtagtccctagaaggaaagcaaaaataattagggat
>#46 FJ424863 2754 nt
cctgtgaagataggggacatctatgtgaggctttgttagacacaggggctgatgatacagtagtagaacactta
tctttagaaggtaggtggaaaccaagaatgataggaggcataggaggattcattaaagtaaaagaatatgaagat
gtagaaatagaaatagaacatagaaaagtagtagggacagttttagttggaccaacccctgtaaatatcatagga
aggaatattttaacaaaaattggatgtaccttaaatttcccataagctccatagatgtagtacccgtatcttta
aaaccagggatggatggacccaaagtcaagcaatggcctttgtcaaaagaaaaaatagaagccttaacagctatt
tgtcaggaaatggaacaggaaggcaagatatcaaaaattgggcctgaaaatccatataatacacctattttcgcc
ataaaaaagaaagacagtackaaatggagaaaattggtagattttagagaattaaataaaaggacacaagatttt
tgggaagttcagttagggattcctcacccaggagggcttaaaaagaaaagatcagtaacagtactggatgttggg
gacgcttatttttcttgtcccttagacccagatttagaaaatatacagctttcacaattcctagtgtgaataat
gagacaccaggggtaaggtatcagtacaatgtactaccccagggatggaaaggatctccagcaattttccagcac
tccatgacaaaaatacttgatcctttcagaaagaaaaacccagaaatagaaatctatcagtacatggatgacttg
tatgtagggtcagatttacccataacagaacataggcagaaagtagaaaaactcagggaacatttatatgcatgg
ggattcaccacccctgacaaaaagcatcaaaaggaacctccctttttgtggatggggtatgagctgcaccctgat
aagtggacagtgcaacctataaagttaccagaaaaggaaagttggacagtcaatgatatccagaaactggtaggg
aaattaaattggccagtcaaatatatccaggaataagagtaaaagaactctgcaaattaattaggggtactaaa
tcacttacagaagtagtgacccttagtaaagaggcagaattagaactagaagagaacagggaaatattaaaagaa
cccgtgcatggagtgtattaccagcctgaaaaggaattaatagtgaatgtgcaaaaacaaggagaaggacagtgg
acttatcagatcttccaggatgaatacaagaacttaaagacaggaaagtatgctaggcaaaaggccactcatact
aatgacatcagacagttggcagaagtaatacaaaaggtctcacaggaaagcatagttatctggggaaaattgcct
aagttcaggctacccatatgtcgaagtacctgggacgctttttggactgactattggcaggcttcttggattcct
gagtgggaatttgttagtacacccccccctcattaagctctggtatcaattggaaaaagaacccataccaggagca
gaaaccttttatgtcgatggagcagctaatagagaaagcaaacaaggaaaagcaggatacgtaacagataggggt
agacagaaaataaggaaactagagggcacaactaatcagaaagcagaactagaggcagtcctcttggccttggaa
gaatcagggaaaaatgttaatatagtgacagattcccagtatgtactagggattattgcagcaagtcctgatcaa
agtgactccccccttagtgcagaaaataatagaggaaatgacaacaaaagaaaaggtctatttagcttgggtacca
gctcacaaaggtataggggaaatgaagaaatagacaaattagtcagtaaagatatcagaagagtcttattccta
gaaggcatagatcaagcacaggaagatcatgaaaaatatcacagtaattggagagcactagccagtgactttggt
ctgccgccagtggtagccaaagaaatcatcaatagctgccctaagtgccatgtaaaagggaagctaggcatgga
caagtagattgcagcccaggtatctggcagttggactgcacccacttagaagggaaaatcattttagtggcagtc
catgtggctagcggcttcatagaagctgaagttattccagcagaaacagggcaggaaacagcctacttcctgctc
aagttagcagctagatggccagtaaaaataatacatacagacaacgggcctaattttactagtgccacagtcaag
gctgcctgttggtggctcaatgtaacacatgagtttggaattccctataatccccaaagtcaaggagtagtggaa
tctatgaacaaagaattaaaaaagattatacaacaagtgagagatcaagcagaacatttaaagacagcagtgcag

atggcagtatttgtccacaattttaaaagaaaaggggggattggggggatacactgcaggagacaggataatagac
atgttagccacacaaatacaaacaacagaattacaaaaacaaattttaaaattcaaaattttcgggtctattac
agagacagcagagaccctatttggaaaggaccagcgacgctcctgtggaaaggtgaaggggctgtagtcattcag
gacaaaggggacattaaagtagttcctaggagaaaggcaaaaataattagggat
>#47 FJ424864 2754 nt
cctgtgaagataggggacatctatgtgaggctttgttagacacaggggctgatgatacagtagtagaacactta
tctttagaaggtaggtggaaaccaagaatgataggaggcataggaggatttattaaagtaaaagaatatgaagat
gtagaaatagaaatagaacataggaaagtagtagggacagttctagttggaccaacccctgtaaatattatagga
aggaatattttaacaaaaattggatgtaccctaaatttcccataagctccatagatgtagtacccgtatcttta
aagccagggatggatggacccaaagtcaagcaatggcctttgtcaaaagaaaaaatagaagccttaacagctatt
tgtcaggaaatggaacaggaaggcaagatatcaaaaattgggcctgaaaatccatataatacacctatttttgcc
ataaaaaagaaagacagtactaaatggagaaaattggtagattttagagaattaaataaacgaacacaggatttt
tgggaagttcagttagggattcctcacccaggagggcttaaaaagaaaaaatcagtaacagtaytggatgttggg
gacgcttatttttcttgtcccttagacccagatttagaaaatatacagctttcacaattcctagtgtgaataat
gagacaccaggggtaaggtatcagtacaatgtactaccccagggatggaaaggatctccagcaattttycagcac
tccatgacaaaaatacttgatcctttcagaaagaaaaacccagagatagaaatctatcagtacatggatgacttg
tatgtrgggtcagatttaccyataacagaacataggcagaaggtagaaaaactcagggaacatttatatgcatgg
ggattcaccactcctgacaaaaagcatcaaaaggaacctcccttttttgtggatggggtatgagctgcaccctgat
aagtggacagtgcagcctataaagttaccagaaaaggaaagttggacagtcaatgatatccagagattggtaggt
aaactaaattgggctagtcaaatatatccaggaataagagtaaaagaactctgcaaattaattaggggtactaaa
tcacttacagaagtagtgacccttagtaaagaagcagaattagaactagaagagaacaggaratattaaaagaa
cccgtacatggagtgtattaccagcctgaaaaggaattaatagtgaatgtgcaaaaacaaggagaaggacagtgg
acttatcagatcttccaggatgaatacaagaatctaaagacaggaaagtatgctagacaaaaggccactcatact
aatgacatcagacagttggcagaagtgatacaaaaggtctcacaagaaagcatagttatctggggaaaactgcct
aagttcaggctacccatatgtcggagtacctgggacgcttttttggactgactattggcaggcttcctggatycct
gagtgggaatttattagtacacctcccctcatcaagctctggtatcaattggaaaargaacccataccaggagca
gaaacctttttatgtagatggagcagctaatagagaaagtaaacaaggaaaagcaggatatgtaacagataagggt
agacagaaaataagaaaactagagggcacaactaatcagaaagcagaactagaggcagtcctcttggccttagaa
gaatcaggaaaaaatgttaacatagtgacagactcccagtatgtactagggattattgcagcaagtcctgatcaa
agtgactccccccttagtgcagaaaataatagaggaaatgacaacaaaggaaaaagtctatttagcttgggtacca
gctcacaaaggtatagggggaaatgaagaaatagacaaattagtcagtaaagatatcagaagagtcttattccta
gaaggcatagatcaagcgcaggaagatcatgaaaaatatcacagtaattggagaacactagccagtgactttggt
ctgccgccagtggtagccaaagaaatcatcaatagctgccctaagtgccatgtaaaggggaagctaggcatgga
caagtagattgcagcccaggtatctggcagttggattgcactcacttagaagggaaaatcattttagtagcagtc
catgtggccagcggcttcatagaagctgaagttattccagcagaaacagggcaggaaacagcctacttcctgctc
aagttagcagctaggtggccagtaaaaataatacacacagacaatgggcctaattttactagtgccacagtcaag
gctgcctgttggtggctcaatgtaacacatgagtttggaattccctataatccccaaagtcaaggagtagtggaa
tctatgaacaaagaattaaaaaagattatacaacaagtgagagatcaagcagaacatttaaagacagcagtgcag
atggcagtatttgtccacaattttaaaagaaaaggggggattggggggatacactgcaggagacaggataatagac
atgttagccacacaaatacaaacaacagaattacaaaaacaaattttaaaattcaaaactttcgggtctattac
agagacagcagagaccctatttggaaaggaccagcgacgctcctgtggaaaggtgaaggggctgtagtcattcag
gacaaaggggacattaaagtagttcctaggagaaaggcaaaaataattagggat
>#48 FJ424871 2754 nt
cctgtgaggataggggacatctatgtgaggctttgttagacacaggggctgatgatacagtagtagaacactta
cctttagaaggtaggtggaaaccaagaatgataggaggcataggaggatttattaaagtaaaggaatatgaagat
gtagaaatagaaatagaacatagaaaagtagtagggacagtcttagttggaccaaccccggcaaatattatagga
agaaatgtgttaacaaaaattgggtgtaccctaaatttcccataagccccatagaggtagtacccgtatcttta
aagccagggatggatggacctaaagttaagcaatggcccttgtcaaaagaaaaaatagaagccttaacagccatt
tgtcaggaaatggaacaggaaggcaagatatcaaaaattgggcctgaaaatccatataatacacctatttttgcc
ataaaaaagaaagacagtaccaaatggagaaaactggtagattttagagagttaaataaacgaacacaagatttt
tgggaagttcagttagggatccctcacccaggagggcttaaaaagaaaaaatcagtaacagtactggatgttggg
gacgcttatttttcttgtcccttagacccagatttagaaaatatacagctttcacaattcctagtgtgaataat
gagacaccaggggtaaggtatcagtacaatgtactaccccagggatggaaaggatctccagcaattttccagcac
tccatgacaaaaatacttgatcctttcagaaagaaaaacccagaaatagaaatctaccaatacatggatgacttg
tatgtagggtcagatttacccataacagaacataggcagaaagtagaaaaactcagggaacatttatatgcatgg

ggattcaccaccccctgacaagaagcatcaaaaggaacctcccttttttgtggatggggtatgagctgcaccctgat
aaatggacagtgcagcctataaagttaccagaaaaggaaagttggacagtcaatgatatccagaaattggtaggc
aaattaaattgggctagtcaaatatatccaggaataagagtaaaagaactctgcaaattaatcaggggtactaaa
tcacttacagaagtagtgacccttagtaaagaagcagaactagaattagaagagaacagggaaatcttaaaagaa
ccagtacatggagtgtattaccagcctgaaaaggaattaatagtgaatgtgcaaaaacaagaagaagggcagtgg
acttatcagatcttccaggatgaacacaagaacttaaagacaggaaaatatgctaggcaaagggccactcatact
aatgacatcagacaattggcagaagtgatacaaaaggtctcacaggaaagcatagttatctggggaaaactgcct
aaattcaggctacccatatgccgaagcacctgggacgcttttttggactgactattggcaggcgtcctggattcct
gagtgggaatttattagtacaccccctctcattaagctctggtatcaattggaaaaagaacccataacaggagca
gaaacctttatgtagatggagcagctaatagagaaagcaaacaaggaaaagcaggatatgtaacagataggggt
agacagaagataagaaaactagagggcacaactaatcaaaaagcagaactagaggcagtcctcttggccttggaa
gaatcagggaaaaatgttaatatagtgacagactcccagtatgtgctggggattattgcagcaagtcctgatcaa
agtgactccccccttagtacagaaaataatagaggaaatgacaacaaaagaaaaagtctatttagcctgggtacca
gctcacaaaggtataggggggaaatgaggaaatagacaaattagtcagtaaagatatcagaagagtcttattccta
gaaggcatagatcaagcacaggaagatcatgaaaaatatcacagtaattggagagcactagccagtgactttggk
ctgccaccagtggtagccaaagaaatcatcaatagctgccctaagtgtcatgtaaaaggggaagctaggcatgga
caagtagattgcagcccaggtatctggcagttggattgcactcacttagaagggaaaatcattctagtagcagtc
catgtagctagtggcttcatagaagctgaagttattccagcagaaacagggcaggaaacagcttacttcctgctc
aagttagcagccaggtggccagtaaaagtaatacacacagataatgggcctaactttactagtgccacagttaag
gctgcctgttggtggctcaatgtaacacatgagtttggaattccctataatccccaaagtcaaggagtagtggaa
tctatgaacaaagaattaaaaaagattatacaacaagtgagagatcaagcagaacatttaaagacagcagtgcag
atggcagtatttgtccacaattttaaaagaaaaggggggattggggggatacactgcaggagacaggataatagac
atgttagccacacaaatacaaacaacagaattacaaaaacaaattttaaaaattcaaaattttcgggtctattac
agagacagcagagaccctatttggaaaggaccagcgacgctcctgtggaaaggtgaaggggctgtagtcattcag
gacaaaggggacattaaagtagttcctaggagaaaggcaaaaataattagggat

>#49 JN091690 2760 nt

actgtgaaagtacaaggacaagtctgtcaagctcttttagacactggagcagatgacagcgtttttttgtaatctt
aagctaaagggacagtggactccaaaaactataggggggaatagggggctttgtgccagtgaatgaatattacaac
attccagttcagataggaaataaagaagtaacagccactgttctagtaggggacacccccattaatataatagga
agaaatattctacaacaattaggatgtactctaaatttccccattagtaaagttgatgttgtaaaggtgaagcta
aaagagggaatggatgggccaaaggtaaaacaatggcccctctctaaagaaaaaattgaagctttaacagagatc
tgcaattccttagaagaggaaggaaagatttctgcaattgggccagaaaatccatataatacaccaatctttgca
attaagaaaaaggatggttctaaatggagaaaattggtagattttagagagttaaacaagagaacacaggatttc
tgggaattgcagctaggaataccccatccagcaggactaaagaagaggggcatggtaacagtactggatgtaggg
gatgcttattttttctattcctctggatccagacttcagacagtatactgcttttaccattcccagtcttaacaac
aatacaccaggaaaaagatttcagtataatgtgttgcctcaagggtggaaggggtcaccagcaattttttcaaaac
agtatgacaaaaatttttagatcccttcagaaaagaccacccagaggtggatgtctatcagtacatggatgatctt
tatgtagggtcagatttaaatgaaaatgaccataggcagctcataaaaaggttaaggcagcatttgttagcctgg
ggactagagactccagacaaaaagtatcaggaaaaacctccatttctgtggatgggttatgagctgcatccaaac
aaatggacagtgcaaaatattacactgccagagccagagcagtggacagtaaatcatattcagaaattagtaggt
aaattaaattgggctagtcaaatttacccaggaataaaaactaaggaactgtgcaagctcattagaggagtaaaa
gggctgactgacccagtacagatgactagggaagcagaactagaattagaagaaaacaaagaaattctaaaagaa
aaggtacaaggggcttattatgatcctaagctaccccctgcaggcagcagttcaaaagcaggggcaaggacaatgg
acataccagatttatcaggaagaaggaaaaaaacctgaaaacaggaaaatatgcaaaatcagcaggcactcatact
aatgagataaggcaactagcaggattgatacagaaaataggtaatgaaagcatcataatttggggaattgtgcct
aaattcttgctgccagtatccaaagaaacatggagtcaatggtggactgattattggcaagttacctggatacct
gagtgggagttcattaataccccaccattaatcagattatggtataatttgttagataaccccatcccagaagca
gaaactttttatgtggatggagcagctaatagagatagcaaaaaaaggaaagcaggatatgtcacagataggggg
aggtacaaggcaaaagagttagaaaacaccacaaatcagcaagcagaattatgggcagtggatttggcattaaaa
gattcaggagcacaagtaaacatagtgacagactcccaatatgttataggaattctacaaggactgcctgatcaa
agtgactcccccatagtagaacaaatcatacaaaaattaaccaataaagcagcagtctatttagcctgggttcca
gcccatagaggaatagggggcaatgaagaagtagacaagttagttagcaaaaatatcagaagagtactctttcta
gaaggaattaatgaggcacaggaagatcatgataaatatcatagtaattggaaagcattggctgatgaatacaat
cttcctcctgtagtagcaaaagaaatcatagcccaatgccccaagtgtcacataaaaggagaagccatacatgga
caagtagactgtagtccagaagtatggcagctagattgtacccacttggaagggaaaatcatcatagtagcagta

catgtggccagtggattcatagaggcggaggtcatacctgaggaaacaggaaaagaaacagcttacttcatatta
aaattagcaggcagatggcctgtaaagaagattcatacagataatgggccaaatttcactagtacagcagtaaag
gctgcatgttggtgggcgcaaatccaacatgaatttgggattccatacaatccccaaagtcaaggggttgtggaa
tccatgaataagcaattgaagcaaattattcagcaggttagggaacaagcggaacaactgaaaacagcagttgta
atggcagtctacattcataattttaaaagaaaaggggggattggggagtatactgcaggagaaagactattagac
ctactgactacaaacatacagacaaagcaattacaaaaacaaattttaaaaattcaaaattttcgggtttattat
agggacggcagagatccggtttggaagggaccagcgcgactgctgtggaaaggtgaaggagcagtagtaataaag
gaggggggaagatattaaagtagtgcccagaagaaaagttaagataataaaagactatgga
>#50 AF447763 2754 nt
acagtaaaagtccagggacaagtctgtcaagctcttttagatactggagcagatgacagtgtttttttgtaacatc
aaattaaagggacagtggacaccaaaaaccataggaggaataggaggatttgtaccagttagtgagtactataat
attccagtacaaattggcaataaagaagtcagagccactgtcctagtgggagaaaccccccattaatataataggt
agaaatattttaaagcaattaggatgtaccttaaattttcctattagcccaatagaggtagtaaaagtacaatta
aaagaaggaatggatgggccaaaagtaaagcagtggcccctctccaaggagaaaattgaggcattaacagaaata
tgtaagacattggaaaaggaaggaaaaatttctgcagttggaccagaaaacccatataacacaccaatttttgcc
attaagaaaaaggatacctctaaatggagaaaattagtagatttcagagaactgaataaaagaactcaagatttt
tgggagttacagctaggaataccccatccggcagggttaagaaaaagaaatatggtgacagtactggatgtaggg
gatgcctacttttccattcccctggatccagacttcagaaagtatacagcttttaccatacccagtctcaataat
aacacaccagggaaaagatttcagtataacgtgttacctcaaggttggaagggatctccagcaatttttcagagc
agtatgacaaaaatcctagatcctttcagaaaagaacacccagatgtggacatttaccaatatatggatgatctt
tacataggttcagatcttaatgaagaggaacataggaaactgataaagaagctgagacagcatctgttaacatgg
ggattagagacccctgacaaaaagtatcaggaaaaacctccattcatgtggatgggctatgagctacatccaaat
aaatggacagttcaaaatatcacattaccagaaccagagcagtggacagtgaatcatatccagaagttggtaggc
aaacttaattgggccagtcaaatttatcatggaataaaaactaaagaactatgcaaattgattagaggagtaaaa
ggattaactgagccagtagaaatgaccagggaagcagaattggagttagaagaaaataagcagattctaaaagaa
aaggttcaaggagcatactatgatcctaaattacctctgcaagcagcaatacagaagcaggggcaaggacagtgg
acatatcagatatatcaggaagaagggaaaaatttaaaaacaggaaaatatgcaaaatcaccaggtacccacacc
aatgagataagacaattagcaggactgatacagaaaataggcaatgagagcataataatttggggtattgtgcct
aaattttttattacctgtatccaaagagacatggagccagtggtggactgattactggcaagttacctgggtacct
gagtgggaatttattaacaccccaccactaatcaggctatggtacaatctgttgtctgaccccatcccagaagca
gaaaccttttatgtagatggggcagcaaacagagacagtaaaaagggaagagcaggatatgtaacaaacagaggc
agatacaggtcaaaggacttagagaacaccactaatcaacaagcagaattatgggcagtagatctagccttaaaa
gactcaggagcacaggtaaatatagtcacagattcccaatatgttatgggagttttacagggattaccagatcaa
agtgactcccccatagtagagcaaattattcaaaagttaacacaaaagacagcaatttatctagcatgggtacca
gcccataaaggtataggggggtaatgaagaagtagacaaattggttagtaaaaatattagaaaaatattattcctg
gatggaattaatgaagcacaggaagaccatgataaatatcacagtaattggaaagctttagctgatgaatataat
ctgcccccagttgtggctaaagaaattattgctcagtgtccaaaatgccatataaaaggagaggctatacatgga
caggtggactacagtccagaaatctggcaaatagactgtacccacctagaaggaaaggtcatcatagtagcagtg
catgtagctagtggtttcatagaagcagaagtcataccagaagaaacaggaagagaaaccgcttacttcatccta
aaattggcaggaagatggcctgtaaagaaaatacatacagataatggaccaaattttactagtacagcagtgaag
gcagcctgctggtgggcacaaattcaacatgaatttgggattccatataatcctcaaagtcaaggagtagtagaa
tctatgaataaacaattaaagcaaattatagagcaagtcagggaccaagcagagcaactgaggacagcagtaatc
atggcagtgtatatccacaattttaaaagaaaaggggggattggggagtacactgcaggggaaagactattagac
atactaactacaaatatacagacaaaacaattacaaaaacaaattttaaaagttcaaaattttcgggtttattat
agggacgccagagatccaatttggaagggaccagcgcgactactgtggaaaggtgaaggggcagtagtaataaaa
gaaggagaagacattaaagtagtacccaggagaaaagcaaaaatcataaaagag
>#51 JQ768416 2754 nt
gcagtcagagttcaaggacatctctgtcaagctctgttagacacaggtgctgatgacagtgttttctgcaattta
cagctaaaaggacagtggaaaccaaaaaccataggggggtatagggggggtttataccagtaagtgagtactataat
attccggtgcaaattggcaataaagaagtaagagccactgtcttagtgggggatacaccccctaaatataatagga
agaaacattttacaacaattgggctgtactttaaatttccccattagtaaagttgaagtagtaaaagtagaacta
aaacaaggcatggacgggccaaaagtaaaacaatggcctctgtctaaagagaaaattgaagccttaacagaaata
tgtagtactttagaaaaggaaggaaaaatctctgccataggaccagaaaatccttacaatacacccatatttgca
atcaagaaaaaggactcttctaaatggaggaagttagtggacttcagagaattaaataaaagaacacaggatttc
tgggagttacaactagggataccacaccctgcaggattaaaaaagagaaaagtggtaacagttctggatgtaggg

gatgcttatttttccattcctctagatccagactttagaaaatatacagcatttaccatccctagtctcaataac
aacacaccagggaagagatttcagtacaatgtgttacctcaaggatggaagggatcacctgctatatttcagagt
agcatgacaaaaatttttagaccctttcaggaaaaaacatccagaggtagatgtgtaccaatacatggatgatctt
tatgtaggatcagactacagtgaagaggagcacagaaagctaatgaaagagctaagacagcacttgttagcatgg
ggattagaaaccccagacaaaaaatatcaagaaaaacctccattcttatggatgggctatgagctacatccagat
aaatggacagtgcagaacatcaccctgccagaaccagaacagtggacagtgaatcacatccaaaagctggtaggc
aagttaaattgggcaagtcaaatctatgcaggaataaagacaaaagaattatgcaaattaataagaggagtaaaa
ggactaactgaccctgtggaactaacaagagaagcagaattagagttagaagaaaataaagaaattctaaaagaa
aaggtgcagggtgcatactatgaccctaaactaccattacaagcagcagtacaaaaacagggacagggtcaatgg
acatatcaaatatatcaggaagaaggaaaaaatttaaaaacaggcaagtatgcaaaatctccaggcacccacact
aatgagataaggcaattagcaggtcttatacagaaaataggtaatgaaagcatagtaatttggggcactgtacca
aagtttctcttgccagtgactaaggaaacatggagtcagtggtggacagattactggcaagtcacctgggtacct
gaatgggaattcattaacacccccccattgattagactatggtacaatttgctctctgaccccattccagaagca
gaaacattttatgtagatggggcagcaaatagggaaagtaaaaagggaaaagcaggatatgtcacagacaggggt
agatataaaaccagggaactagaaaataccacaaatcagcaagcagaactatgggctgtagatttagctctaaag
gattcagggtcacaagtaaacatagtaacagactcccagtatgtcatgggggtactgcaagggatgcctgatcaa
agtgactcccccatagttgaggaaatcatacaaaaactcatgcagaaaaatgcagtgtatctagcatgggtacct
gctcataaaggaataggaggcaatgaggaagtagataagctagtcagtaaaaatattaggaaagtcctattttta
gaagggataactgaggcacaggaagatcatgataaatatcatagtaattggaaagcaatggctgatgaatacaat
cttcccccctatagtagccaaggaaatcattgcccagtgttctaaatgtcacaaaaaaggagaagccattcatggc
caagtagactgtagcccagaagtatggcagctagattgcactcacctagaagggaaagtcatcatagtggcagtg
catgtggccagtggtttcatagaagctgaggtcataccagaggaaacaggaaaagaaacagcttacttcattctt
aaattggcagggagatggcctgtaaaaagaatacatacagacaatggaccaaattttactagcacagcagtaaag
gcagcatgttggtgggcacaaatccaacatgaatttggcataccttataatccccaaagtcaaggagtagtggaa
tctatgaataagcaattaaagcaaataatagagcaaattagagaccaagcagagcagttaaaaacagcagtagta
atggcagtctacattcacaattttaaaagaaaagggggattggggagtatactgcaggagaaagactgctagat
atactaaccacaaatatacagacacaacaactacaaaaacaaattttaaaagttcaaaattttcgggtttattat
agggacgccagagacccgatttggaagggaccagcgcggctactgtggaaaggtgaaggagcagtagttataaaa
gagggtgaggacatcaaagtagtacccagaaggaaagcaaaaataatcaaagaa
>#52 JQ866001 2754 nt
gaagtaacagtgcagggacaggtctgcacagccctttagacacaggggctgatgatacagtatttgcagacctt
aagttaaaaggatcatgcaaccctaggacaattggaggaatagggggatttgtccctgtacaagaatattacaat
gtaccaattaccatagcagggaaaacagtgaaaacaaaagtactaataggaccaaccccagtaaatataattgga
agaaatgttttaagacaacttggttgtactctaaatttccctattagtacagtagaaatagtaaaagtgaagctc
aaggaaggaatggatgggcctaaggtaaaacaatggccactatccagagagaaaattgaagccctaacagaaatt
tgtgaaaatttaagagcagaagggaaaatcattgaagtaggacctgacaatccttacaatactcccatctttgct
attaaaaagaaagacagtacaaaatggagaaaattagtagattttagagagctcaataaaaggacacaagatttt
tgggaggtacaattaggaataccacacccagcagggttaaaaaagagaaaagtcattactgtgttggatgttggg
gatgcttatttttcaatcccattagacccagaatttcaaaagtatacagcatttaccataccaagtgtcaataat
gaaaggcctggtaaaagatattgttacacagtgctaccacagggatggaaggggtctccagcaatcttccaatac
agtatgacaaaaatttttagatccattcaggaagaagcatcctgaaatagatgtttaccaatacatggatgacctg
tatgtaggatctgatttgacagctgaaaaacataaagaagtggttcaggagttaagaaaccacttgaaagcatgg
ggcttagaaacccccgataaaaaatatcaagagaaaccaccatttctctggatgggatatgaattacacccagag
aagtggacagtgcagcagataaaattaccagagccagaaacatggacagttaatgatattcagaaattagtgggt
aaattaaattgggcaagtcaaatttatccagggatcagaacaagagaactctgcaaattaattaggggagtcaaa
ggactaacagatgtagtacagttgactgcagaagcagaattagaattagaggaaaataaacaaattttaaaagaa
acagtacaggggacctactatgacccaaaggaacctcttaaggcagctgtacagaagcaaggaaaaggacaatgg
acttatcaagtctatcagaaagaaaatcaaatattaaaaacaggaaaatatgcaagaaaccatggtactcatact
aatgaattaaaacaattagcaggggtagtgcaaaaaattgggacagaaagcattataatatggggaagagtacct
gtattccaacttccagttacaaaggaagtgtggcaacaatggtggtctgactattggcaggtcagctggattcca
gaatgggagtttgttaacacccccccattaataaagttatggtacaatctgttaacagaacctatgccagaagca
gagacctattatgtagatggagcagcaaacagggactctaaattagggaaagcaggctatgtgacagatagagga
aaaagcagggtaatagaattaacagaaactactaatcagcaggcagaattacaggctatactgctggcattaaaa
gatgcaaaagaaaaggtcaacatagtgacagattcccaatatgtgctaggaatcttacaaggaactcctgaccaa
agtgactccccccctagtagaggaaataatacaagaattaatgaataaagaagcagtttatttaaattgggtacct

gctcacaaaggaataggagggaatgaagaagtagataaattagtaagcaaaaacattagaaaagttcttttccta
gaaggaatagatcaagcacaagaagatcatgaaagattccatagtaattggaaaatgttagctgatgaatataat
ttacccccatagtagctaaggaaatcatagctcaatgcaataaatgtcacaggaaaggagaagccatacatgga
caagtggattgtagcccagaaatatggcaactagactgcacacacatagaaggaaaagtggttctagtagcagtc
catgtagccagtgggttcatagaagcagaagtgttatcaagtgaaacaggcaaggaaacagctttcttcatttta
aaactagcaggaagatggccagtaaaaaagatacatacagataatggagccaatttactagtgctgcagtaaag
gcagcatgctggtgggcaaacatccaacaggaatttggaattccctacaatccacaaagtcaaggagtggtagaa
tccatgaataaacaattaaaaattattgtagaccaaattagagaacaagcagaacatttaaggacagcagtagct
atggcagtgtacattcacaattttaaaagaaaaggggggattgggggtacacaccaggagatagaatattagac
atattaacaacagacatacaaactaaagaattacaaaaacaaatttttaaacttcaaaattttcgggtctattat
agggactcaagagacccagtgtggaaaggaccagcccgtctcctgtggaaaggtgaaggggcagtagtcataaag
gagggagaagaaatcaaagtagtaccaagaaggaaggctaagattataaaggat

>#53 DQ373065 2745 nt

ataggaggagaaataaaagaagctcttctagatacaggagctgatgatacagtaatagaagaaatacagttggaa
ggaaaatggaaaccaaaaatgatagggggaattggagggtttatcaaagtaaaacagtatgataatgtgataata
gagatacaagggaaaaaagcagttggcacagtcttagtagggccaacacctgttaatattattggaagaaatttt
ctgacacaaattggttgtactctaaattttccaataagtcctattgaaaccataccagtgaaattgaagccaggt
atggatggcccgagagtaaaacagtggcctttgacagaagaaaaaataaaggcactaacagaaatttgtacagaa
atggaaaaagaaggaaaaatttccagaatagggcctgaaaatccatataatactccaatttttgctataaaaaag
aaagatagcactaaatggagaaaattagtagatttcagagagttaaacaaaagaactcaagattttgggaagta
caattaggaatcccacatccagcagggctaaagaagaaaaaatcagtaacagtgctagatgtaggagatgcttac
ttttcatgtcccttagatgaaaatttagaaagtatacagcttttaccatacctagtgtaaataatgaaacacct
ggtattagataccagtacaatgtgctgccacaaggctggaaaggatcaccagcaatctttcagagcactatgaca
aaaatattagaaccatttaggaagaacaatccagaactagtcatatatcagtacatggatgatctctatgtaggc
tctgatctagaaataacacaacatagagaggcagtggaaagacttaggagtcatcttttgacttggggatttaca
acccctgacaaaaagcatcagaaagaaccaccattttgtggatgggatatgaactccatccagataaatggaca
gtccagacaatacaattaccagagaaagacacatggactgtcaatgatatacaacagttagtaggaaaattaaac
tgggctagccagatctacccagggattaaagtaaaacaactttgcaaattaattagaggagcaaaagctctaaca
gaagtagtcacacttacaagagaagcagaattagagttagctgagaatagggagatattaaaagaacctgtacat
ggggcctattataacccagacaaagaattaatagcagagatccagaagcagggacaaggtcaatggacatatcag
atttatcaagacttacataaaaatttaaagacaggaaaatatgcaaaaatgaggtctactcatactaatgacata
agacaattaactgaagtggtacaaaaggtggcactagaaagtatagttatctggggaaaaactcctaaattcaga
ttaccagtgcaaaaagaagtatgggagacctggtggacagagtattggcaggcaacttggattcctgattgggag
tttgtcaacacccctccactagtaaaattatggtatcagttagagacagaaccaattagcggggcagaaacctat
tatgtagatggagcagctaatagggaaactaaattagggaaagcaggatttgtgacagataggggtaggcaaaag
gtgacctctatctcagaaactaccaaccaacaagctgaattacaggctgtcctcatggccttacaagatgcagga
caagaagtaaatatagtcactgactctcagtatgtcctaggaataattcactcacaaccagataaaagtgaatca
gaattagtaaatcagataatagaagaacttataaaaaaggaaagaatatatctttcctgggtacctgcacataaa
ggtattggaggaaatgaacagatagacaaattagtcagcacaggaatcaggaaagtcttattcctagatggtata
gataaggctcaagaagaacatgaaagatatcatagtaattggaaggcaatggccagtgattttaatctaccccc
atagtagctaaagaaatagtagccagttgtgataagtgtcaattgaaaggagaagccatacatggcaaatcaac
tgtagtccaggggtatggcaattagattgcacacatttggagggaaaaattatccttgtagcagtccatgtggcc
agtggctacttagaagcagaagtcattcctgcagaaacaggacaggaaactgcatattttatcctaaagttagct
ggaagatggccagtaaaagtcatacatactgataatgggagcaacttcaccagtgccactgtaaaagcagcctgt
tggtgggcaaatatccaacaggagtttggaataccctacaatcctcaaagtcagggtgcagtagaatccatgaat
aaagaattaaagaaaattataggacaaattagagatcaagcagaacatttaaaaacagcagtacaaatggcagtc
ttcattcacaattttaagagaaaaggggggattggggatacactgcaggggaaagaataatagacataatagca
acagatatacaaacaacaaagctacaaacacaaattttaaaagttcaaaattttcgggtttattacagagacagc
agagagcccacttggaaaggaccagccaaactactgtggaaaggagaaggggcagtggtaattcaagataacggg
gatataaaagtagtcccacgtagaaaggccaaaataattagagat

>#54 AF103818 2745 nt

gtagcagggcagatagtagaagctcttttagacacaggagctgatgatacagtactagacaacatacaaattgaa
gggacatggagaccaaaaatgatgggggaattggaggttttataaaagtaaaacaatatgatcacgtcaatata
gaaatagagggaagaaaagcacagggttcagttttagtgggaccaacaccagtaaatattattggcaggaatatt
ttaacacaaattggatgtactttaaattttcctatcagctctattaaaactgtaccagtaagattaaaaccagga

atggatggccctagagtaaagcaatggccattgacagcagaaaaaattaaggcattaacagaaatttgccaagaa
atggaaaaagaaggaaagattacaagagtagggccagaaaatccttacaatacaccaattttgctatcaaaaag
aaagatagcactaaatggagaaaattagtagactttagagaattaaataaaaggacacaagacttctgggaagtt
cagttaggcataccacacccagcaggattaaagaagaaaagatcagtaacagtcctagatgtaggggatgcctat
ttttcttgtccactggataaggaatttagaaaatacacagcatttaccatccctagtgtaaacaatgagacccca
ggaattagataccagtataatgtgttaccacaaggatggaaagggtcaccagcaattttccagagcagtatgaca
aaaattctagatcctttagaaaacaacatccagatgttataatctatcaatatatggatgatctctatgtaggg
tcagatctaaacttagaaaagcatagggaaaaggtagaactgctcagacaatatttgcttacttggggattcact
acccctgataagaagcatcaggaggaaccaccattcttatggatgggctatgaactccatccagataagtggaca
gtgcagcccatacagttaccacaaaaagaaatttggacagtcaatgatattcagaaattagtagggaaactgaac
tgggcaagtcagatatatccaggaataaaaataaagcagttatgcaaattaataaaaggagctaaagctctaact
gaagttgtaaattttacacatgaggcagaaatggagttagaagaaaacagagaaattctaaaggaaccagtacat
ggggtctattatgacccagaaaaagaattagtagcagaagtacaaaaacaaggaaggagtcagtggacataccaa
atttttcaagaacggcataagaatctaaagacaggaaaatatgccagacaaagatcagcacatactaatgatatc
agacagctagttgaagtggtgcaaaaaatagctactgagagcattgtcatttggggaaaggtaccaaaatttaaa
ttgctagtgcagaaggaagtctgggaaacatggtggtcagaatattggcaggccacctggattccagattgggaa
tttgtcaatacccctcccctcgtaaaactttggtataagttagagacagaggccatagagggggcagaaacattt
tatgtggatggagcagcccaacgagaaaccaagaaaggaaaggcaggatatgttactgataggggtagacaaaaa
attataactttagaaaatactactaatcaaaaggcagagctcacagcggtatacttagcattaaaagattcagag
aatacagttaatgtagtcactggctcccaatatgtcttaggaatcatccactctcagccagaccaaagtgaatca
gaattggtcaatcagataatagaagaattaataaagaaagaaaaagttatatctcatgggtaccagcacataaa
ggaattggaggaaatgaacaaatagataaactagtcagttcagggatcagaaaagttcttttcctagatggcata
gataaagcacaggaagaccatgacaaatatcatagtaattggacagctatggccagtgattttaacctgccacca
gtggtcgctaaggagatagtggccagctgtgataaatgtcagccaaaaggagaggccatacatgggcaggtagat
tgcagtccaggtatctggcagctagattgtacacatttagaaggaaaatcattatagtggcagtacatgtggcc
agtggatacctagaagcagaggtcattcctgcagaaacaggacaagagacagcctattttatcttaaaattagca
ggaagatggcctgtaaaagtgattcatactgataatggatctaactttacaagtagtacagttagagcagcttgc
tggtgggcaggcatacaacaagagtttggaattccatacaatccacaaagtcaaggagtggtagaatccatgaat
aaagaattaaagaaaatcataggacaaatcagggatcaagcagagcatttaaagacagctgtacagatggcagta
ttcattcacaattttaaaagaaaaggggggattggggggtatactgcaggagaaagaatcatagacatcatagca
tcagaactacaaacagacttattacaaaaacaaattttaaaagttcaaaattttcgggtctattacagggacagc
agagatccaatttggaaaggaccagccaaacttctgtggaaaggtgaaggggcagtagtaatcaaggaaaacgag
gaggttaaagtagtacccagaagaaaagcaaaaattataaaagac
>#55 EF535993 2745 nt
atagaggggcagctgatagaagctctgctagacacaggagctgatgatacagtgatagataatgtacaattaaca
ggaagatggaaaccaaaaatgataggggggaattggaggttttatcaaagtaaaacaatatgataacatagcaata
gaaattgaaggcagaaaagcaacaggcacagtgttggtgggaccaacaccagtgaatataattggcagaaatata
ttgacacagattggctgtactttaaatttccctataagtcccattggaactgtgccagtaaaattaaagccagga
atggatggccctagagtaaaacaatggcccttaacagaagagaaaatcaaagctttaacagaaatttgtcaggaa
atggaaaaagaaggaaagatttcaaaaatagggccagaaaatccttataatacaccaattttgccataaaaaag
aaagatggtgaaaaatggagaaaattggtagatttagagaattaaatagaagaacacaggacttctgggaagtc
caattaggcataccacacccggcaggattaaaaaagaaaaagtcagtaacagtactagatgtgggagacgcttat
ttttcctgcccactagatgaaagttttagaaaatacacagcatttaccatacctagtgtaaacaatgagacacca
ggaattagatatcaatataatgtgctaccacaaggatggaaagggtcaccagcaatttttcaaagcagcatgaca
aaaatcttagaaccatttagacaacagaatccagagttgacaatttaccagtacatggatgatctttatgtaggc
tctgayctagaaataggagaacatagaaaaaaggtagagttacttagacaacatctactcacttggggttttact
actcctgataagaagcatcaaaaagagccacctttttttgtggatgggctatgagcttcatccagataaatggaca
gtgcaaccaatacagttaccacaaaaagagacctggactgtcaatgatatccaaaagctggtaggaaaattaaat
tgggcaagtcaaatatatccagggataaaagtaaaacagctatgcaagctcataaaaggagcaaaagctttaact
gaagtagtggccatgacacaggaagcagaaatggaattggaagaaaatagagaaattttaaaagacccagtgcat
ggagtgtactatgacccagagaaggagttaatagcagaagtacaaaaacagggaaacagtcagtggacttatcaa
atttttcaggaacagcataagaacttgaaaacaggaagtatgctaggcaaagatcagcacatactaatgacata
agacaattagcagaggtagtacaaaaagtagcaactgaaagtatagtaatctggggaaaaacaccaaaattcaga
ttacctgttcaaaaggaggtctgggaaacctggtggtcagaatactggcaagccacctggatcccagactgggag
tttgtaaataccccctcccctggtaaaattatggtaccarttagaaacagaacccataccaggggcagaaacattc

tatgtagatggggcagccaacagagacacaaagaaaggaaaagcaggttatgtcacggatagaggtagacagaaa
atagtcaatctagaagataccactaatcagaaagcagagctgacagctgtttatctagccctacaagatgcagaa
catatagttaacatagtcactgattcccaatatgtgttaggaataattcactctcagccagatcaaagtgagtca
gaattggttaatttaataatagaagagttaataaaaaaggaaaaaagctacctctcatgggtgccagcacacaaa
ggaattggaggaaatgaacaggtagataaattagtcagctctggaattaggagagttctcttcttagatggtata
gataaggcacaagaagaacatgaaaaatatcataataattggagagctatggcaagtgacttcaacctcccacct
gtagtggccaaagaaatagtagccagttgtgacaagtgccaattaaaaggagaagccatgcatgggcaagtagac
tgcagcccaggaatgtggcaattagactgcacacatctagaagggaaagtcatcctagtggcagtacatgtagcc
agtggatacctagaggcagaggtcatccctgcagaaactggacaggaaacagcttatttattctaaaattagca
ggaagatggccagtaaaagtaattcacactgataatggatctaattttaccagtaatacagttaaggcagcctgt
tggtgggcaggcatccaacaggaatttggaattccttataacccacaaagccaaggagtagtagaatcaatgaac
aaagaattaaagaagatcataggacaaattagagaacaagcagagcatttaaagacagcagttcaaatggcagtg
ttcattcacaattttaaaagaaaaggggggattgggggtacactgcaggagaaaggattatagacattatagca
acagacatacaaacaactaatttacaaaaacaaattttaaaagttcaaaatttcgggtttattacagggacagc
agagatccaatttggaaaggacctgccagacttctgtggaaaggtgaaggggcagtagtaatcaaagataaagag
gaagttaaagtagtgcccaggaggaaagcaaaaataatcagagat
>#56 AY169968 2745 nt
atagaagggcaattatgtgaagccctgttagacacaggggctgatgatacagtaatacaagacattcaattacag
ggaatttggaaaccaaaaatgatagggggaattggaggctttattaaagtgaaacaatatgataatgtatcaata
gagatagaggggaaaaaagtgcaagggacagtgttggtgggaccaacacctgtcaatataattggagaaatatt
ttaacacagatagggtgcacactggtattccctatcagttcaattgaaactgtcccagtcaaattgaaaccagga
atggacgggccaagaataaagcaatggcctttatcagcagaaaaaattaaagccttgacagaaatatgtcaggaa
atggaaaaggaagggaagatatctaaaataggacctgaaaacccatacaatactcctatttttgcaattaaaaag
aaagatagttcaaaatggagaaaattagtagatttcagagaattaaataaaagaacccaagatttctgggaagta
caattgggcataccccacccagcagggttaaaagagaagaaatcagtaacagtactagatgtgggtgatgcatat
ttctcttgtcctctagataaggacttcaggaagtatacagcattcacaattcctagtgtaaataatgaaactcca
gggatcagatatcagtataatgttttaccacaaggatggaaagggtctccagctatctttcaaagtagtatgaca
aaaattctggaacccttcagagcaaaaaaccctgatatcactgtctaccaatacatggatgacttatatgtaggg
tctgatcttgacattacccaacacagacagaaggtagaagagttaaggcaacatttgcttcactggggggtttaca
accccagataaaaagcatcaaaaagaaccaccccttcttatggatggggtatgagcttcacccagataaatggaca
gtgcagccaataaagctaccagaaaaggaggtatggacagtcaatgatcttcagaaactcataggaaaattaaac
tgggcaagtcagatttatccaggaataaaaataaaacagttgtgcaaattaataagaggaacaaaaaggttaaca
gatgtagttcctctcacaccagaagcagaattagaattagcagaaaacagagaaatagttagtacaccagtacat
ggggtttattataatccagataaggagctcatagcagaagtacaaaaacagggagaagggcagtggacatatcag
atctttcaggaacaacatagaaatttaaagacaggaaaatatgccagacagagatcaacacatacaaatgatatt
agacaattggcagaagtcatacaaaaaattgctgtggaaagtatagtcatttggggaaaaacacctagatttaga
ttaccagtacaaaaagaaagttgggagacatggtggcagagtactggcaggccacctggatccccgagtgggag
ttcattaacacccccccattagtaaaattatggtatagtttagagacagaccctataccaacggcagacacatac
tatgtagatggagcagcaaatagggaaacaaagaaaggaaaagcaggatacataacagacaagggaaaacaaaaa
ataatcagtctagagaacaccactaatcaacaggcagagttaggggccttgtatctagctttacaagactctgaa
caacaagtgaacatagtaactgactctcaatatgtgttagggattattcagtcacaaccagatcacagtgaatca
gaattagtaaatcaaataatagaagaattaattaaaaaggacaaggtttacctttcctgggtacctgcacataag
ggcataggaggaaatgaacaagtagataagttagtcagttctggaatcaggaaagtactattcctggatggaata
gataaagcacaggaagaacatgaaagatatcatagcaattggaaagctttggcgggtgaatttaacttaccaccc
atagtagcaaaagagatagtggctaactgtgataaatgtcaggtaaaaggagaggctctgcatgggcaggttgat
tgtagcccaggaacatggcagatggattgcacacatttagaaggcaaaatcatcatagtagcagttcatatagcc
agtggatacatagaagcagaagttatcccagcagaaacaggacaagaaactgcatacttcatattaaaattagca
agcagatggccagtgaaagtcttacacacagataatgggacaaattttacaagtaatgcagttaaagcagcatgt
tggtgggccaacatcaaacaaaagtttggaattccctacaacccacaaagtcaaggagtggtagaatccatgaac
aaagaattaaagaaaatcattgaacaagttagagatcaggcagaacatctaaaaacagcagtacaaatggcagtg
ttcagtcacaattttaagagaaaaggggggattgggggtacactgcaggggaaagaataatagacataatagca
acagacatacaaacaaaagaactacaaaaacaaattttaaaaattcaaaaatttcgggtttattacagagacagc
agagacccgatttggaaaggaccggccaccctgctgtggaaaggtgaaggggcagtagtaattcaggatcaagga
gaaataaaagtagtacctagaaggaaagcaaaaataattagagat
>#57 AF382828 2745 nt

gtagcaggacaactatgtgaagccctactagatacaggggctgatgatacagtattagataatatacaactagaa
gggaaatggagaccaaaattaataggaggcattggaggatttattaaagtaaaacaatatgataatgtagaaata
gaaatagagggaaaaagagtagaaagcacagtactagtgggaccaactccagttaatattataggaagaaatgtg
ttaacccaattaggatgtactctaaattttccaataagtccaattactcctgtgccagtaaaattaaagccagga
acagatggaccaagggtaaagcaatggccattgtcaaaagaaaagatagaggcattaacagaaatttgtagggaa
atggaaaaagaaggaaaaatttcaagaataggtcctgaaaatccatataatactcccatatttgcaataaggaag
aaagacagtacaaagtggagaaaattagtagatttagagaattgaataaaagaacacaagatttttgggaagta
cagttaggcattccacaccctgcaggtttaaagaaaaagaaatcagtaacagtgccggatgtgggagatgcttat
ttttcctgccccttagatgaaaactttagaaagtatacagccttcaccatccctagtgtaaataatgaaactcca
ggtataaggtaccagtataatgttctaccacaaggatggaaagggtcaccagcaatcttccaaagcagcatgaca
aaggttttagaccctttcagacagaaaaacccagaattggtaatttaccaatacatggatgacttatatgtagga
tcagatttatctttggaagaacacaggaaaagagtagaacaactcagagaacatttactcagatggggttttacc
acccctgacaagaagcaccaaaaggaacctcctttctctggatggggtatgagcttcatccagacaaatggaca
gtgcagccaatacagctgcctcagaaagagaattggactgtaaatgacattcagaaactggtaggtaaattaaat
tgggcaagtcagatctatccaggaattaagataaaacaattgtgtaaattaatcagaggagctaaggctttaaca
gaaatagtcgagatgactagagaagcagaattagagttagaagaaaatagggaaattttaaaagaaccagtgcat
ggcacctactatgacccagaaaaagaactaatagcagaagttcaaaagcagaataatgggcaatggacttatcag
atttttcaggaaacaggaaggaatctaaaaacaggaaaatatgccagacaaagagcagctcctactaatgacata
agacaactagctgaagtggtgccaaaagtttcccctgaaagtataatcctttggggaactgttcctaaatttaag
ttaccagtgcaaagggaagtatgggaagcctggtggacagattattggcaagccacctggatccctgaatgggaa
ttcgtcagaaccccaccccttgtaaagttatggtatcaattggaaaaagaacctatacaaggagcagaaacctat
tatgttgatggggcagccaatagggagacaagaagaggaaaagcaggatatgttattaataatggaaaacagaag
gtcatttccttggaagagaccactaaccaaaaggcagaattagaggcactcagaatagccctacaggactcaggg
caagaagtaaatgtggtaacagactcccaatatgtgttgggaatcataaattcccaaccagaccaaagtgagtca
gaaatagtgaatcaaataatagaagagctaataaagaaagagaaggtctatttgacatgggtaccagcacacaaa
ggaataggaggaaatgaacaggtagataagttagtcagtagtggcatcagaaaggtgctatttttagatggtata
gataaggcacaagaagaccatgagagatatcatagcaactggagagccatggctgatgaattccaactcccaccg
gtagtagccaaagaaatagtagccacatgtgataaatgtcagcttaaaggagaagcaatacatggacaagtggat
tgcagtccagggatctggcagctggactgcacccacttagaagggaaaataatcctggtggcagtccatgtggca
agtggatacttagaagcagaagtaattccagctgagactggacaggaaactgcatattttatcttaaaattagca
gggagatggccagtaaagaagatacacacagataatggaacaaatttcaccagtgcagcagtaaaagcagcctgt
tggtgggcaggcataaaacaagaatttggaataccttataatccacaaagtcaaggggtagtagaatccatgaac
aaagaattaaaaaagattatagggcaagttagagaccaagcagaacacttaaagacagcagtacaaatggctgta
ttcattcacaattttaaaagaaaagggggggattggggggtacactgcaggagaaagaatagtagacatgctagca
acagacttacaaacacaagaactacaaaaacaaattctaaaaattcaaaaatttcgggtttattacagagacagc
agagaccccatttggaaaggaccagcctctctcctgtggaaaggtgagggtgcagtagttattcaggacaacgga
gatataaaagtagtacccagaagaaaagctaaaattattaaacat
>#58 EF535994 2739 nt
ggagttataaaagaggtactattagacacaggggcagatgatacagtactagaggaaatagaattagagggaaaa
tggaggccaaaaatgatagggggaattggaggttttataaaagtaagacaatatgatcagatacccatagaaatt
tgtggaaaaagagcaataggtacagtattagtagggcccacacctgtgaatattatagggagaaatatcttaact
caaattgggtgtactctaaattttcccattagcactatagaaactgtaccagtaaaattaaagccagggatggac
ggtccaagagttaaacagtggcccctaacagaagaaaaaataaaagcattgacagaaatttgtacagagatggaa
aaggaaggaaaaatttcaagaatagggccagaaaatccatataatacaccaatatttgctattaagaaaaaggat
agcacaaaatggagaaaattagtagacttcagagaattaaataaaagaacccaagatttttgggaagtccagcta
ggaataccacaccctgcgggactgaaaaagaaaaaatcagtaacagtgctggacgtaggggatgcttatttttca
tgcccccctagatgaagagtttagaaaatacactgccttcaccatacctagtgtaaataatgagacaccaggaata
aggtaccagtacaatgtactcccgcagggatggaaaggatcaccagctatttttcaaagcagtatgactaagatt
ctggaaccttatagaaaacagaatccagaaatagtcatctaccagtacatggatgatctatatgtaggatctgat
ttacatatagagcaacatagagaaaaggtggaagaattaagagctcatctattaaagtggggctttactacacct
gacaaaaagcatcaaaaagaaccaccattcctttggatgggatatgagctccatccagacaaatggacagtccag
cccatacagctgccagamaaagacagctggactgtcaatgacatacagaaattagtaggaaagttaaattgggca
agtcaaatatatccaggaattaaggtaaaacaattgtgtaagcttattagaggaacaaaagctttaacagaggta
gttrccctcacacaggaagcagaattagagttagcagaaaacagggagatattaagagaccctgtacatggggcc
tactatgacccctcaaaagaattgatagcagaaatacagaaacaaggtcaaggccaatggacttatcagatttat

caggaacagcataaaaatctgaaaacaggaaaatatgcaaaaatgagagctgcccattcaaatgatgtaaaacag
ttgactgaagtagtacagaaagtagccctggaaagtatagtcatctggggaaaaacacccaagtttagactgccc
atacagaaagaaacatgggaggcttggtggacagattactggcaagcaacttggatccctgaatgggaatatgtt
aacacccctcccctggtaaagctgtggtaccagttagaaacagaacccatagtgggggcagaaacctttatgta
gatggggcagcaaatagagaaaccaaattaggaaaagcaggatatgtaactgatagagggagacaaaagattatc
tccctaacagaaacaactaatcaaagagcagaactgcaagctatatacctagccctacaggattcagaagtagag
gtaaacatagttactgactcacaatatgcattagggatcattcaatcacagcctgacattagtgaatcagacata
gtaaatcagataatagagcagctagtgaaaaaggaaaagatttatctttcatgggtaccagcacacaaaggatt
ggaggaaatgaacaaatagacaaattagttagtgcaggaatcaggaaagtcctttttcctggatggaatagataag
gcccaagaagaacatgagaaataccacaacaattggaaagctatggcagatgaatttaatttgccacccatagta
gccaaagaaatagtagccagctgtgataaatgccaattaaaaggagaagccatgcatggacaggtagactgtagt
ccaggaatatggcaactagactgcacacatttagaaggaaagatcatcttagtagctgtacatgtagccagtgga
tacatagaggcagaagtcatacctgctgaaacagggcaagagacagcttattttgtgctgaaattagcaggaaga
tggccagtaaaggttatacacactgataatggcagcaactttactagtgctgctgtcaaggctgcatgttggtgg
gcaaatatccaacaggaatttggaattccctacaatcctcagagtcaaggagtagtagaatcaatgaacaaagag
ttaaagaaaatcatagggcaggtaagagatcaagcagaacatttaaagacagcagtgcaaatggcagtcttcatt
cacaattttaaaagaaaaggggggattggggggtacagtgcaggggaaagaatcatagatataatagcaacagat
atacaaactagagaactacaaaaacaaatttcaaaagttcaaaaatttcgggtttattacagggacagcagagaa
cctatctggaaaggaccagccaagcttctctggaaaggtgagggagcggtagtgcttcaagataaggaagatata
aaagtagtcccacgcagaaaggctaaaataatcagggac
>#59 DQ373066 2745 nt
attgaaggacaattaattgaagccttgctagacacaggggctgatgacacagtaattgaagacttaaatttgcca
ggaaaatggagtccaaaaatgatagggggaattggaggttttattaaagtaaaacaatttgaaagagtaaacata
gaaatagaaggaaaaaagataaccagtactgtattagtaggaccaacccctgtcaatataataggaagaaatgtc
ctaacaaaattagggtgtactctaaatttcccaataagtccaatagacacagtaaaagtgtttctaaagccaggg
atggacgggcccaaggtaaaacagtggcctctgtcaaaagaaaaaatagaggcattaactgaaatatgtaaagaa
atggaaaaggaaggaaaaatttcaaaaatagggccagaaaatccttacaatacacctatttttgcaattaagaaa
aaggacagcaccaaatggagaaaacttgttgatttcagagaacttaataagaggactcaagaattttgggaagta
caactgggcataccacacccagctgggttaaagaagaaaaaatcagtaacagtcttggatgtgggagatgcctat
ttttcatgtcctcttgacccagactttagaaaatttactgcattcaccattcctagcataaataatgaaactcca
ggaattagatatcaatataatgtacttcctcaaggatggaagggatctccttccatctttcagagtagcatgaca
aagatcttagaacccttttagaacaaagcatccagaaataattatttaccagtacatggatgacttgtatgtgggc
tcagatttacctatagaggaacatagaaagagagtagaagagctcagagcacatctgttaaaatggggatttaca
acaccagacaagaagcatcagaaagaacccccatttctctggatgggatatgagttacatccagataagtggaca
gtccagcaaataaaattgccagaacaggaagtgtggactgttaatgatattcagaagttagtaggcaaattaaat
tgggcaagtcaaatatatccaggaattaaaattaagcagctttgtaaattaattaggggagcgaagggtctgaca
gaagtagtagctctcaccccagaagctgaactagaactggcagaaaatcaagagatactaaaagaaccagtacat
ggagcctactatgaccctgaaaaggaattaatagcagaaatacagaagcaaggacagggtcagtggacatatcaa
atatttcaggagccacataaaaacctaaagacaggaaaatatggcaaacaaagagctgcacatactaatgatatt
aggcagcttgctgaaacagtacaaaaaatagctacagaaagcattgtaatatggggaaaaacaccaaaatttaga
ctccctgttcaaaaagaaatgtgggaagcctggtgggcagattactggcaggctacctggattcctgagtgggaa
ttcataaacaccccacccctagtaaagttatggtaccagttagagacagagcctatagaaggagcagaaacatac
tatgtagatggggcagccaacagagaaacaaaaagaggaaaagcaggctatgtgacaaacaggggagacaaaag
attcatagtcttgaagataccactaatcaaaaagcagaattacaagcagttttaatggccttacaggactcagga
caggaagtcaatgtggtcactgactcccaatatgtgctgggaatcttacaatcacacccagaccacagtgaatca
gatatagtaaatcagataatagaagagttaataaaaaaggaaaaaatttatctatcatgggtaccagcacacaga
ggtataggaggaaatgaacaggtagataaactagttagttcagggatcagaaaagttcttttcttagatggaata
gaaaaagctcaagaagaacatgaaagataccactctaattggaaggcaatggctcaggactttaacttaccacca
atagtggctaaagaaatagtagcccaatgtgataaatgtcagctaaaaggagaagcaatgcatggacaagttgac
tgtagcccaggaatatggcagattgattgcacccatttagaaggaaaaataattatagtggcagtccatgtggcc
agtggatacatagaagcagaagtcatccctgctgaaacaggacaggaaacagcatattttgtccttaagttagca
ggaagatggccagtaaagactatacacacagacaatgggcctaatttcactagtaatgcagtaaaggcagcatgt
tggtgggcaggtattagacaggaatttggaattccatacaacccacaaagtcaaggagtagtggaatctatgaat
aaagaattaaaaaagatcatagagcaagtcagagatcaggctgaacatttaaaaacagcagtacaaatggcagtc
ttcattcacaattttaaaagaaaaggggggattggggggtacagtgcaggagagagaataatagacatcatagca

acagacatacaaacaaaagaactacaaaaacaaattttaaaaattcaacaatttcgggtctattacagagacagc
aaagatccaatttggaaaggaccagcgaaactcctgtggaaaggtgaaggggcagtggtaattcaagatcaggac
gaaataaaagtggtgcctagaaggaaagctaagataattagggat
>#60 DQ373064 2745 nt
gtcgggggctacttgaaaggagcactgttagatacaggggcagatgatacagtgctagaagatttagaattaggg
ggaaggtggaaaccaaagatgataggaggaattggaggttttatcaaagtaaagcaatatgataatataccaata
gaaataggaggaaaaaaggcactaggtacagttttagtaggacccacaccagtaaatatcataggaaggaacatt
ttaactcaaattggatgtactctaaattttcctattagccctattgaaactgtaccagtacaattaaagccagga
atggatgggccaaaagtaaaacaatggcccttaacagaggaaaaaattaaagctttaacagaaatctgtacagaa
atggaacaggaaggaaaaatttcaagaatagggcctgaaaacccatataatactcctgtgtttgctataaaaaag
aaagatagcacaaaatggagaaaattagtagatttagagaattaaataaaagaactcaagactttgggaagtt
caattaggaatacctcaccctgcgggattaaagaagaaaaggtcagtcacagtattggatgtaggagatgcctac
ttttcatgcccccctagatgaaaactttagaaaatatactgcattcaccatacctagtataaacaatgaaacacca
ggaatcagatatcaatacaatgttctaccgcagggatggaaaggatcaccagctatatttcaaagtagcatgaca
aagattttagagccattcagaagacagaacccagatatagtaatttatcagtacatggatgatttatatgtaggg
tcagatttgaaaatagagcaacacaggaacaaggtagaagagctgagagctcacttgctaaagtgggggtttacc
acccctgacaaaaaacatcagaaggaaccaccatttctctggatgggatatgaactccatccagacaaatggaca
gtacagcccatacagttaccagaaaaggaaagttggactgtcaatgacatacagaaattggtgggaaaattaaat
tgggcaagtcaaatatacccaggcatcaagataaaacagatatgcaggcttcttaggggagtaaagaattcaaca
gaagtagtcacctttactagagaagcagaattagaattagcagaaaatagggaaatattaaaaaacccagtacat
ggggtctattatgacccggctaaagacctgatagcagaagtacaaaaacaaggacagagtcaatggacctatcaa
atctatcaggaacaatttaaaaatctgaaaacaggaaaatatgccagacaagggtctgctcataccaatgatgta
aaacagttagcagaggtaatgcaaaagatagccatagaaagcatagtcatatggggaaaaataccaaagtttaga
ttgcccattcaaaaagaagcatgggaagcatggtggacagattattggcaagccacttggatccctgaatgggaa
tatgttaatacccccttcccatgtcaagttttggtaccaattagagcaagaacctataccaggggcagaaacattt
tatgtagatggggcagctaacagagaaactaagttaggaaaagcaggatatgtaacagatagggggaagcagaaa
ataatccccttaacagaaacaaccaatcagaaggctgagttacaagccattcaattagccttacaagactcagga
cctgaggtaaatatagttactgattcacaatatgcattaggaatccttcaagcccaaccggatcagagtgattca
gaattggtgaatcaaatcatagaaaatctaataaaaaaggaaaaagtttacttaacatgggtacctgcacataag
ggaataggaggaaatgaacaagtagacaaattagtcagttcaggaattaggaaagtcctttttctggatggaata
gatcgagcccaggaagagcatgaaaaataccataataattggagagctatggccagtgatttcaatctgccaccc
attgtagcaaaagagatagtagctagttgtgataaatgtcagctaaagggagaggcaatgcatggacaggtagac
tgtagcccaggaatatggcagttagattgtacacacctagaaggaaaagtcatcttagtagcagtgcatgtagct
agtggctatatggaagcagaagtcatacctgcagaaacaggacaggaaacagcttattttatcttaaaattagca
tcgagatggccagtaaaaattatacatactgataatggtagcaattttactagcactgcagtcaaggctgcatgt
tggtgggcgcagatccagcaggaatttggaattccctacaatccccagagtcaaggagtggtagaatcaatgaac
aaagaacttaagaaaataatagggcaggtaagagatcaggcagagcacctaaaaacagcagtacaaatggcagta
ttcattcacaattttaaaagaaaaggggggattggggggatacagtgcaggagaaagaataatagacataatagca
acagatatacaaactacagaattacaaaaacaaatttcaaaagttcaaaaatttcgggtttattacagggaccgc
agagatcctatttggaaaggaccagccaaactactgtggaagggtgaaggagcagtagtgctgcaagatcaggaa
gaaataaaggtagtcccacgtagaaaagctaaaattattagggat
>#61 KP861923 2745 nt
ataggaggacacttaaaagaggcactgttagacacaggggcagatgatacagtgctagaagagctagaattaggg
ggaaaatggaaaccaaaaatgatagggggaattggaggttttatcaaagtaaagcagtatgaaaatataccaata
gaaatatgtggaaaaagagcagtaggtacagttttagtaggacccacacctgttaatataatagggagaaacata
ttaactcaaattggctgtactttaaattttcctatcagtcctattgaaactgtaccagtagcattaaagccaggg
atggatggaccaagagtcaaacaatggccattgacagaagaaaaaataaaagcattgacagaaatttgcacagaa
atggaaaaggaaggaaagatttctaaaattgggcctgaaaatccatacaatacaccagtatttgctataaaaaag
aaagatagtacaaaatggagaaaattagtagatttcagagaattaaataaaagaactcaagatttctgggaggtt
cagttaggaataccccaccctgcggggttaaagaagaaaaaatcagttactgtattggatgtgggggatgcttat
ttttcatgccccctagatgaaaattttaggaaatatacagcattcactatacctagtgtgaataatgagacacca
ggaatcaggtatcaatacaatgtactcccgcagggatggaaaggatcaccagccatatttcaagctagtatgaca
aaaattctagagccttacagaaagcagaatccagagataattatttaccaatacatggatgatttgtatgtaggc
tcagatttaaaaattgagctacacagacaaaaggtagaagagctgagagatcatctactaagatggggatttact
acccctgacaagaaacatcagaaggaaccaccattcctctggatggggtatgagctccatccagacaaatgggca

gtacagcctatacagttaccagacaaggagagctggactgtcaatgatatacaaaaactagtgggaaagttaaat
tgggcaagtcaaatatatccaggaattaaagtaaagcaattatgcaagctaattagaggaacaaagagtttaaca
gaggtagttaccctcacaaaagaagcagaattagaattggctgaaaacagggagatattggcaaacccagtacat
ggggtctattatgacccttcaaaagaattgatagcagaagtacaaaaacagggacagagtcagtggacatatcag
atatatcaagaacagtttaagaatttaaagacaggaaaatatgctaaaacaagatcagcccataccaatgatgta
aagcaattaacagaagtagtgcaaaaaatagcattagaaagcattgttatctggggtaagataccaaagtttaga
ctgcccatacaaaaggaaacatgggaagcctggtggactaattattggcaggctacctggatccctgaatgggaa
tatgttaatacccctcccctagtaaaactctggtaccagttagaaacagaaccaataacaggtgcagagacattc
tatgtagatggggcagctaatagagataccaagttagggaaagcagggtatgtaacagataggggaaacaaaag
ataatctccctaacagaaacaactaatcagaaagcagagttacaagctattcagctagctttacaggattcagga
tcagaagtaaacatagtaactgactcacaatatgcattaggaattatccaaggccagccagataaaagtgaatca
gaaatagtaaatcagataatagaaagtttaataaacaaggaaagggtctatctctcatgggtacctgcacacaaa
ggaattggaggaaatgaggaagtagacaaattagtcagttcagggattagaagagttctcttcctggatggaata
gacaaagcacaggaagagcatgaaaaataccataataattggaaagcaatggccagtgactttaatataccccca
atagtagcaaaagaaatagtagccagttgtgataaatgtcaattgaagggagaagcaatgcatggacaggtagac
tgcagtcctggaatatggcaactagattgcacccacctagaaggaaagatcatcctagtagcagtacatgttgcc
agtggctacatagaagcagaagtcatacctgcagaaacagggcaggagacagcatacttcatattaaaattagca
ggaagatggccagtaaaagttatacacactgataatggcagcaactttaccagtaatgcagtcaaggctgcatgt
tggtgggcacagatccaacaggaatttggaattccctacaatccccaaagtcaaggagtagtagaatccatgaat
aaagaactaaagaagattataggacaagtaagagatcaggcagaacatttaaagacagcagtacaaatggcagta
ttcattcacaattttaaaagaaaaggggggattggggggtacagtgcaggagagagaataatagacatcatagca
acagacatacaaactacagaactacaaaaacaaattttaaaagttcaaaaatttcgggtttattacagggacagc
agagatcctatttggaaaggaccagcaaaactactttggaagggtgaaggagcagtagtgcttcaagatcaggaa
gaaataaaggtagtcccacgtagaaaagcaaaaattattagggat
>#62 AJ302647 2754 nt
ccagcaagggttggggccatctatgtgaagttttactggatacaggggcagatgatacagtactaaataacata
caattggaaggaaaatggacaccaaaaatgataggggtataggaggttttataaaggtaaaagaatataatcaa
gtgccagtagaaatagagggaagggaagtactgggaacagtattggtgggacctactcctgttaatattattgga
agaaacatattgacaggattgggttgtacactaaatttccctataagtcccatagccccagtaccagtaaaacta
aaaccaggaatggatggaccaaaaataaaacaatggcccctatctaaagaaaaaatagaagccttgacagcaata
tgtcaggaaatggaacaagaaggaaaaatttcaagaataggacctgaaaatccttataatacacctatctttgct
ataaaaaagaaagatagcactaagtggagaaagctggtagactttagggaattaaacaagagaacacaagatttc
tgggaggtacagttaggtatcccacatccggggggtttaaagcaaaagcaatctgttacagtcctagatgtagga
gatgcttatttctcatgccccttagacccagatttcagaaaatatactgccttcactattcctagtgtgaacaat
gagaccccaggaataagataccagtacaatgtcctcccgcagggatggaaagggtcgccagctatattccaaagt
tcaatgacaaaaattttagatccatttaggaaaaacaacccagaattagaaatttgtcaatacatggatgactta
tatgtaggatcagatttacccctgacagaacatagaaagagagtagaattgcttagagaacacttatatcagtgg
ggattcactacccctgataaaaagcatcaaaaggaacctccctttttgtggatggggtatgagctccatccagac
aaatggacagtacaacccatccaattgcctaacaaggaggaatggacagtaaatgatatacaaaaactagtagga
aaattaaattgggcaagtcaaatctatcaaggaattagagtaaaagaattgtgtaagttaattagaggcaccaag
tcattgacagaagtagtacctttaagtaaagaggcagagctagaattagaggagaatagagaaaagttaaaagaa
ccagtgcatggtgtatactatcaacctgacaaagacttatgggttaatattcagaagcagggaaaggacaatgg
acttatcagatatatcaggatgaacataagaacctcaaaacagggaaatatactaagcaaaaggcctctcacaca
aatgatataagacaattagcagaagtactccagaaggtgtctcaagaagctataattatctggggaaaattgcct
aaatttaagctgccaatcactagagaaacttgggaaacatggtgggcagactattggcaagccacctggattcca
gaatgggaatttgtcagcacagccccattgatcaaattatggtaccagttagaaagtgaacctattataggggca
gaaacctattatgtagatggagcagctaacagagatacaaaactaggaaaagcaggatatgttacagaaaaaggg
aaacagaaaatagtaaaattagaggagaccaccaatcaaaaggctgaattaatggcagtattattagccttacag
gattccaaggaaacagtaaatatagtaacagattcacaatatgtattgggcatcatctcctcacagcctacacag
agtgagtcccctctagttcagcagataatagaggaactaacacaaaaggaacaggtgtttcttacatgggttcct
gcccataaaggcataggaggaaatgaaaaaatagataaattagtaagcaaggatattagaagagtcctattccta
gaaggaatagaccaggcacaagaagatcatgaaaagtatcatagcaattggagagcattagctagtgattttgga
ttgccaccagtggtggccaaagaaatcattgctaattgtcctaaatgtcatataaaggggaagcaattcatggt
caggtagactacagtccagaagtatggcaaatagattgcacacatctagaaggcaaaatcataatagttgctgtt
catgtggcaagtggattcatagaagcagaagtaataccagcagaaacaggacaagaaactgcctacttcctgtta

aaattagctgcaagatggcctgttaaaataatacatacagacaatgggcctaatttcacaagtgcaaccatgaag
gctgcatgttggtggacaggcataaaacatgagtttggaataccatataatccacaaagtcaaggagtagtagag
gccatgaacaaggaattaaaatcaattatacagcaggtgagggaccaagcagaacacttaaaaacagcagtacaa
atggcagtatttgttcacaattataaaagaaaagggggattgggggtacactgcaggagaaaggataatagac
atattagcatcacaaatacaaacaacagaattacaaaaacaaattttaaaattcaaaaatttcaggtctattac
agagacagcagagatcctatttggaaaggaccggcacagctcctgtggaaaggtgagggagcagtagtcatacaa
gataaaggagacattaaggtagtaccaagaagaaaggcaaaaataatcagacat
>#63 AJ302646 2754 nt
acagcaagggttggggggccatctatgtgaagttttactggatacaggggcagatgatacagtactaaccaacata
caattggaaggaaaatggacaccaaaaatgataggggggtataggaggttttataaaggtaaaagaatataatcaa
gtgccagtagaaatagagggaagggaagtactgggaacagtattggtgggacctactcctgttaatattattgga
agaaacatattgacaggattgggttgcacactaaatttccctataagtcccatagccccagtaccagtaaaacta
aaaccaggaatggatggaccaaaaataaaacaatggcccctatctaaagaaaaaatagaagccttgacagcaata
tgtcaggaaatggaacaagaaggaaaaatttcaagaataggacctgaaaatccttataatacacctatctttgct
ataaaaaagaaagatagcactaagtggagaaagctggtagactttagggaattaaacaagagaacacaagatttc
tgggaggtacagttaggtatcccacatccgggggggtttaaagcaaaagcaatctgttacagtcctagatgtagga
gacgcttatttctcatgcccccttagacccagatttcagaaaatatactgccttcactattcctagtgtgaacaat
gagaccccaggaataagataccagtacaatgtcctcccgcaaggatggaaagggtcgccagctatattccaaagt
tcaatgacaaaaattttagatccatttaggaaagacaacccagaattagaaatttgtcaatacatggatgactta
tatgtaggatcagatttacccctgacagaacatagaaagagagtagaattgcttagagaacacttatatcagtgg
ggattcactacccctgataaaaagcatcaaaaggaacctccctttttgtggatggggtatgagctccatccagac
aaatggacagtgcagcccatccaattgcctaacaaggaggaatggacagtaaatgacatacaaaaactagtagga
aagttaaattgggcaagtcaaatctatcaaggaattagagtaaaagagttgtgtaagttaattagaggcgccaag
tcattgacagaaatagtacctttaagtaaagaagcagagctagaattagaggagaatagagaaaagttaaaagaa
ccagtgcatggtgtatactatcaacctgacaaagacttatgggttaatattcagaagcagggatggggacaatgg
acttatcagatatatcaggatgaacataagaacctcaaaacagggaaatatactagacaaaggcctctcacaca
aatgatataagacaattagcagaagtactccagagggtgtctcaagaggctataattatctggggaaaattgcct
aaatttaagctgccaatcactagagaaacttgggaaacatggtgggcggactattggcaagccacctggattcca
gaatgggaatttgtcagtacaccccccattgatcaaattatggtaccagttagaaagtgaacctattatgggggca
gaaacctattatgtagatggagcagctaacagagatacaaaactaggaaaagccggatatgttacagaaaagggg
agacagaaaataattaaattagaggagaccaccaatcaaagggctgaattaatggcagtattattagccttacag
gattccaaggaaacagtaaatatagtacaagattcccaatatgtattgggcatcatctcctcacagcctacacag
agtgaatcctctctagttcagcagataatagaggaactaacaaaaaaggaacaggtgtatcttacatgggttcct
gcccataaaggcataggaggaaatgaaaaaatagataaattagtaagcaaggatattagaagagtcctattccta
gaaggaatagaccaggcacaagaagatcatgaaaagtatcatagcaattggagagcattagctagtgattttgga
ttaccaccagtggtggccaaagaaatcattgctaattgtcctcaatgtcatataaaaggggaagcaattcatggt
caggtagactgcagtccagaagtatggcaaatggattgcacacatctagaaggcaaaatcataatagttgctgtc
catgtggcaagtggattcatagaagcagaagtaataccagcagaaacaggacaggaaactgcctacttcctgtta
aaattagctgcaagatggcctgttaaggtaatacatacagacaatgggcctaattttacaagtgcagccatgaag
gctgcatgttggtgggccaacataaaacatgagtttggaataccatataatccacaaagtcaaggagtagtagag
gccatgaacaaggaattaaaatcaattatacagcaggtgagggaccaagcagaacacttaaaaacagcagtacaa
atggcagtatttgtgcacaattataaaagaaaagggggattgggggtacactgcaggagaaaggataatagac
atattagcatcacaaatacaaacaacagaattacaaaaacaaattttaaaattcaaaaatttcaggtctattac
agagacagcagagatcctatttggaaaggaccggcacagctcctgtggaaaggtgagggagcagtagtcatacaa
gataaaggagacattaaggtagtaccaagaagaaaggcaaaaataatcagacat
>#64 AY169812 2754 nt
acagcaaaagttggggggccatctatgtgaagtkttrctggatacaggggcagatgatacagtactaaacaacata
caattggaaggaaaatggacaccaaaaatgataggggggtatagggggytttataaaagtaaaagaatatgatgat
gtgacagtagaaatagagggaagacaagtacagggaacagtattggtgggacctacccctgttaatattattgga
agaaatatattgacaggattaggttgtacactaaattttcctataagccccatagccccagtrccagtaaaatta
aaaccaggaatggatggaccaaaaataaaacaatggcccctatctaaagaaaaaatagaagcyttgacagcaata
tgtcaggaaatggaacaagaaggaaaaatttcaagaataggacctgaaaatccttataatacacctatctttgca
ataaaaaagaaagatagtactaagtggagaaaattggtagactttagggaattaaacaagagaacacaagatttc
tgggaggtacagttaggtatcccacatccagggggtttaaagcaaaagcaatctgttacagtcctagatgtagga
gatgcttatttctcatgcccccttagacccagatttcagaaaatatactgccttcactattcctagtgtgaacaat

gagaccccaggaataagataccagtacaatgtcctcccgcaaggatggaaaggttcaccagctatattccaaagt
tcaatgataaraattctagatccatttaggagggacaacccagaattagaaatttgtcagtacatggatgaccta
tatgtaggatcagatttacccctgacagaacatagaagaagggtagaaatgcttagagaacacttatataagtgg
ggattcactacccctgataaaaagcatcaaaaggaacctccctttytgtggatggggtatgagctccatccagac
aaatggacagtacagcccatccaattacctaacaaggatgtgtggacagtaaatgatatacaaaaattagtagga
aagttaaattgggcaagtcaaatctattcaggaattagagtaaaagaattgtgtaaattaattagaggcaccaaa
tcattgacagaagtagtacctttaagtaaagaggcagagctggaattagaggagaacagagagaagctaaargaa
acagtgcatggtgtatactatcaacctgamaaagamttatgggttcagattcagaagcatgaawcagggcaatgg
acttaccagatatatcaggatgaatataagaacctcaaaacaggaaartatactaggcaaaaagcctcccacaca
aatgatataagactattaacagaagtagtccagaaggtggctcaagaagccatagttatctggggaaaattgcct
aaatttaagctgccagtcactagagaaacttgggaaacatggtggacagactwttggcaagccacctggattcca
gaatgggaattcgttagtacacccccattgatcaaattatggtacaggttagaaagtgaacctatcataggagca
gaaacctattatgtagatggagcagctaatagaaatacwaaactagggaaggcaggatatgttacagaacaaggg
aaacagaaaatagtaaaattagaggagaccaccaatcaaaaggctgarttaatggcgatactattagccctacag
gaytccaargaaacagtaaatatagtaacagattcacaatatgcattgggtatcatctcctcccaacctacrcag
agtgattccccattagttcagcagataatagaggaactaacaaaaaaggaacaggtgtatcttgcatgggttcct
gctcataaaggcataggaggaaatgaaaaaatagataaattagtgagcaaggatattagaagagtcctattcyta
gaaggaatagaccaggcacaagaagatcatgagaaatatcatagcaattggaaagcattagctagtgactttggg
ctaccaccagtagtggccaaagaaatcattgctagttgtcctaaatgtcatataaaaggagaagcaatgcatggt
caggtcgactgcagtccagaagtatggcaaatagattgcacacatttagaaggcaaggtcataatagttgctgtc
catgtggcaagtggattcatagaagcagaagtaataccagctgaaacaggacaagaaactgcctacttcctgtta
aaattagctgcaaggtggcctgttaaaattatacatacagacaacgggcctaattttacaagtgcaaccatgaaa
gctgcatgttggtggaccaacatacaacatgagtttggaataccttataatccacaaagtcaaggagtagtagaa
gccatgaataaggaattaaaatcaattatacagcaggtgagggaccaagcagaacacttaagaacagcagtacaa
atggcagtatttgttcacaattttaaaagaaaaggggggattgggggtacactgcaggagaaaggataatagac
atactagcatcacaaatacaaacaacagaattacaaaaacaaatttttaaaattcaaaaatttcaggtctattac
agagacagcagagaccctatttggaaaggaccggcacagctcctgtggaaaggtgagggagcagtagtcatacaa
gataaaggagaaattaaagtagtaccaagaagaaaggcaaaaataatcagacat
>#65 AY169816 2754 nt
acagcaagagttgggggccacctatgtgaagttttgctggatacaggggcagatgatacagtattaaccaacata
caattggaaggtaaatggaaaccaaaaatgataggaggtataggaggctttataaaggtaaaggaatatgagaat
gtgacagtagaaatagaaggaagagaagtacagggaacagtattggtgggacctactcctgttaatattattgga
agaaatatattgacaggactaggttgtacactaaatttccctataagccccatagccccagtgccagtaagacta
aaaccaggaatggatggaccaaaagtaaaacaatggcccctatctaaagaaaaaatagaagccttaacagcaata
tgtcaggaaatggarcaagaaggaaaaatttcaagagtaggacctgaaaatccwtataatacacctatctttgct
ataaaaaagaaagatggtaccaagtggagaaagctggtagactttagagaattaaacaagagaacacaagatttt
tgggaggtacagttaggtatcccacatccgggggggtttaaagcaaaagcaatctgttacagtcctagatgtagga
gatgcttatttctcatgtcccttagatccagattttagaaaatatactgctttcactattcctagtgtgaacaat
gagaccccaggaayaagataccagtacaatgtcctcccgcaaggatggaaagggtcaccrgctatattccaaagt
tcaatgacaaaaattctagayccatttagaaaagacaacccagaattagaaatatgtcagtatatggatgactta
tatgtaggatcagatttacccctgacagaacatagaaaargattgaattgcttagagaccacttatatcagtgg
ggattcactacccctgataagaagcatcaaaaggaacctccttttctgtggatggggtatgagctccacccagac
aaatggacagtgcagcctatccaattgcctaacaaggatgtgtggacagtaaatgatatacaaaaactaatagga
aagttaaactgggcaagtcaaatttaccaaggaattagagtaaaagaattgtgtaaattaattagaggcaccaag
tcattaacagaagtagtacccttaagtaaagaggcagagctagaattagaggaaaacagagaraggctaaaagaa
ccagtgcatggggtatattatcaacctgamaaagacttatgggttgatattcagaaacagggagaagggcaatgg
acttaccagatatatcaggataaatataagaacctcaaaacagggaaatatacwaggcaaaaggcctcccacaca
aatgatataagacaattagcagaagtactccagaaggtgtctcargaatctatagttatctggggaaaattgcct
aaatttaagctgccagtcactagagaaatttgggaagcatggtgggcggactattggcaagccacctggattcca
gaatgggaatttgtyagcacacccccattgattaaattatggtaccagttagaaagtgaacctattatgggggca
gaaacttattatgtagatggagcagctaatagagagacaaaactaggaaaggcaggatatgttacagaactaggg
aaacagaaaataataaaattagatgaaaccaccaatcaaaaggctgaattaatggccatattattagccttacag
gattccaaagaaaaagtaaatatagtaacagattcacaatatgcattgggcatcatttcctctcaacctacacag
agtgaatctcctatagttcagcagataatagaggaactaacaagaaaggaacaggtatatcttgcatgggttccg
gctcataaaggcataggggaaatgaaaaaatagataaattagtaagcaaggatattagaagagtcctgttctta

gaaggaatagaccaggcacaagaagatcatgaaaagtatcatagtaattggagagcattagctagtgaatttgga
ctaccaccagtagtggccaaggaaatcattgctagctgtcctaaatgtcatataaaggggaagcaatgcatggt
caggtagactgcagtccagaagtatggcagatggattgcacacatctagaaggcaaaatcataatagttgctgtc
catgtggcaagtggattcatagaagcagaagtaataccagcagaaacaggacaagaaactgcctacttcctgtta
aaactagctgcaagatggcctgttaaaatattacacacagacaatgggcctaattttacaagtgcaactatgaag
gctgcatgttggtgggccaacatacaacatgagtttggaataccatataatccacaaagccaaggagtagtagaa
gccatgaataaggaattaaaatcaattataggacaggtgagggaccaagcagaacacttaagaacagcagtacaa
atggcagtatttgttcacaattataaaagaaaaggggggattggggggtacactgcaggagagaggataatagac
atattagcaacacaattacaaacaacagaattacaaaaacaaattttaaaattcaaaattttcgggtctattac
agagacagcagagaccctatttggaaaggaccggcacagctcctgtggaaaggtgagggagcagtagtcatacaa
gayaagggagacattaaggtagtaccaagaaggaaggcaaaaataatcagagat
>#66 KU168282 2754 nt
acagcaagggttggggccatctatgtgaggctttactggatacaggggcagatgatacagtattaaatagcata
caattagaaggaagatggaaaccaaaaatgataggggtataggaggctttataaaagtaaaagaatatgataat
gtgacagtagaaatacaaggaaaggaagtasaggggacagtattggtgggacctactcctgttaatattattggg
agaaatatattgacaggattaggatgtacactaaatttccccataagtcccatagccccagtaccagtacagcta
aaaccaggaatggatggaccaaaagtaaaacaatggcccctatctagagaaaaaatagaagcactaacagcaata
tgccaagaaatggaacaggaaggaaaaatctcaaggataggacctgaaaatccttataatacacctatttttgct
ataaaaaagaaagatagcacaaaatggagaaagttggtagacttcagggaattaaataaaagaacacaagatttc
tgggaagtgcaattaggtattccacatccaggggggtttaaagcagaggcaatctgttacagtcttagatgtggga
gatgcttatttctcatgtcctttagacccagacttcagaaaatacactgccttcactatacctagtttgaacaat
gagacccccggagtaagataccagtacaatgtcctcccgcaagggtggaaaggttcaccagccatatttcagagt
tctatgacaaagatttagatccatttagaaaagacaacccagaaatagaaatttatcagtacatggatgactta
tatgtaggatcagatctaccattggcagaacatagaaagagggttgaattgcttagagaacatttatatcagtgg
ggattcactacccctgataaaaagcaycagaaagaacctccctttctatggatgggatatgagctccatccagac
aagtggacagtacagcccattcaattgcctaacaaggaagtatggacagtaaatgacatacaaaaagtaatagga
aaattaaattgggcgagtcaaatctatccaggaatcagagtaaaagaattgtgcaagttaattagaggaactaaa
tcattgacagaggtgataccgttaagtaaagaagcagaactagaattagaagaaaacagagaaaggctaaaagag
cctatacatggagtatattatcaacctgacaaagacttatgggttaatattcagaagcaaggagaagggcaatgg
tcttaccagatatatcaggatgaacataagaaccttaaaacagggaaatatactaggcaaaaggcctctcacaca
aatgacataagrcaattagcagaagtggtccagaaggcatctcaagaatctatagttatatggggaaaattacct
aaatttaagttgccagtcactagagaaacttgggaagcctggtgggcagattattggcaagccacctggattcct
gaatgggaatttgtcagcacrccccattgatcagattatggtaccggttggaaacagaacctattgtaggggca
gaaacctattatgtagatggagcagctaataggaatacaaaactaggaaargcaggatatgttacagaacaaggg
aaacagaaaataataaagttagaggagacaaccaatcagaaggctgaattaatggccatactattagccttgcag
gattccaaaaaacaagtaaacatagtaacagactcacaatatgcattgggcatcatagcctcccaaccaacacaa
agtgactcccctatagttcagcagataatagaagaactaaccaataaggaacaagtgtatcttacatgggtacca
gctcacaaaggcataggaggaaatgaaaaaatagataagttagtaagcaaagatattagaagagtcctgttccta
gaaggaatagatcaggcacaagaagatcatgaaaaataccatagcaattggagagcattagctagtgactttgga
ataccaccagtagtagctaaggaaatcattgctagttgtcctaaatgccatataaaggagaagcaatgcatggc
caagtagactacagcccagagatatggcaaatggattgcacacatttagaaggcaagatcataatagttgctgtc
catgtggcaagtggctttatagaagcagaagtaataccagcagaaacaggacaggaaactgcctatttcctgtta
aaattagcagcaagatggcctgtcaaagtaatacatacagacaatgggcctaattttacaagtgcagccatgaag
gctgcatgttggtggacaggcatacaacatgagtttggaataccatataatccacaaagtcagggagtagtagaa
gccatgaataaggaattaaagtctattatacagcaggtgagagaccaagcagagcatctaaaaacagcagtacaa
atggcagtctttgttcacaattttaaaagaaaaggggggattggggggtacactgcaggagagagattaatagat
atactagcatcacaaatacaaacaacagaattacaaaaacaaattttcaaaattcaaaattttcaggtctattac
agagatagcagagaccctatttggaaaggaccggcacaactactgtggaaaggtgagggggcagtagttatacaa
gataaaggagacattaaagtagtaccaagaagaaaggcaaaaataatcagaaat
>#67 AY169809 2754 nt
acagcaagggttggggccatctatgtgaagttttactggatacaggggcagatgatacagtactaaacaacata
caattggaaggaaaatggacaccaaaaatgataggggtataggaggctttataaaggtaaaagaatatagcaat
gtgaaagtagaaatagaaggaagggaagtacagggaacagtattagtgggacctacccctgttaatattattgga
agaaatatattgacaggattaggttgtacactaaatttccctataagccccatagccccagtaccagtgaaacta
aaaccaggaatggatggaccaaaagtaaaacaatggcccctatctaaagaaaaaatagaagctttaacagcaata

tgtcaggaaatggaacaagaaggaaaaatttcaagaataggacccgaaaatccttataatacacctatctttgct
ataaagaagaaagatagtaccaagtggagaaaattggtagacttyagagaattaaacaagagaacacaagatttc
tgggaggtacagttaggtattccacatccgggggtttaaagcaaaagcaatctgttacagtcctagatgtagga
gatgcttatttctcatgtcctttagacccagatttcagaaaatatactgccttcactattcctagtgtgaacaat
gagaccccaggagtaagataccagtacaacgtcctcccgcaaggatggaaaggatcgccagccatattccaragt
tccatgacaaaaattctagatccatttaggaaaagaacccagaaatagaaatttatcagtacatggatgactta
tatgtaggatcagatttgcccttggcagaacatagaaaaagggttgaattgcttagagagcatttatatcagtgg
ggattcacgacccctgataaaaacatcaaaaggaacctccctttctgtggatgggatatgagctccacccagac
aagtggacagtacagcccatccacttgcctaataaggacgtgtggacagtaaatgatatacaaaaactagtagga
aaattaaattgggcaagtcaaatctatcaagggattagagtaagagaattgtgtaagttaatcaagggcaccaag
tcattgacagaggtggtacctttaagtaaagaggcagaactagaattagaagaaaayagggaaaagctaaaagaa
ccagtacatggrgtatactatcaacctgacaaagatttatgggttaatattcagaagcaaggagagggacagtgg
acttaccagatatatcaggatgaacataagaacctcaagacagggaaatatactaggcaaagagcctcccacaca
aatgatataaggcaattggcagaagtactccagaaggtatctcaggaatcgatagttatctggggaaattgcct
aaatttaagctgccggtcactagagaaacttgggaaacatggtgggcagactattggcaagccacctggattcca
gaatgggaatttgttagcacacccccattgatcaagttatggtaccagttagaaagkgaacctattagggggca
gaaacatattatgtagatggagcagctaatagagatacaaaactaggaaaggcaggatatgttacagagcaaggg
aaacagaagataataaaattagaagagaccaccaatcaaaaggctgaattaatggcagtgttagtagccttacag
gattccaaggagaaagtaaacatagtaacagattcacaatatgtattgggtatcatctcctcccaacctacacag
agtgaatcccctatagttcaacagataatagaagaactgacaaaaaaggaacaggtgtaccttacatgggtccct
gctcataagggcataggggaaatgaaaaaatagataaattagtaagcaaagatattagaagagtcctattccta
gaaggaatagaccaggcacaagaagatcatgagaaataccatagcaattggagagcattagccagtgactttgga
ctaccaccagtggtggccaaggaaattattgctaattgtcctaaatgtcatataaaggggaagcaattcatggt
caggtagactgcagtccagaagtatggcaaatggattgcacacatgtagaaggcaaagttatcatagttgctgtc
catgtggcaagcggattcatagaagcagaagtgataccagcagaaacaggacaggaaactgcctatttcctgtta
aaattagcagcaagatggcctgttaaagtaatacatacagacaacggcctaattttacaagtgcaaccatgaaa
gctgcatgttggtggactaacatacaacatgagtttggaataccatacaatccacaaagtcaaggagtagtagaa
gccatgaataaggaattaaaatcaattatacagcaagtgagggaccaagcagagcacttaaggacagcagtacaa
atggcagtatttgttcacaattttaaaagaaaagggggattgggggtacactgcaggagagagattaatagac
atattagcatcacaaatacaaacaacagaactacaaaaacaaattttgaaaattcaaaatttcgggtctattac
agagacagcagagaccctatctggaaaggaccggcacagctcctgtggaaaggtgagggagcagtagtcatacaa
gacaaaggagacattaaggtagtaccaagaagaaaggcaaaaatactcagggac
>#68 KU168283 2754 nt
acagcaagggttgggggccatctatgtgaagctttactggacacaggggcagatgatacagtactaaccaacata
caattagagggaaaatggacaccaaaaatgataggggggtataggaggttttgtaaaagtaaaagaatatgatgac
gtgacagtagagaaagagggaagaagagtacagggaacagtattggtgggacctactcctgttaatattattgga
agaaatatattgacaggattaggttgtacactaaacttccctataagccccatagccccagtgccagtaaaacta
aaaccaggaatggatggaccaaaagtaaaacaatggcccctatctaaagaaaagatagaagccttgacagcaata
tgtcaagaaatggagcaagaaggaaaaatttcaagagtaggacctgaaaatccttataatacacctatctttgct
ataaaaaagaaggatagtactaggtggagaaagctagtagactttagagaattaaacaagagaacacaagatttc
tgggaggtacaattaggtatcccacacccgggggtttacagcaaaggcaatctgttacagtcctagatgtagga
gatgcttatttctcatgccccttagacccagatttcagaaaatatactgctttcactattcctagtgtgaacaat
gagaccccagggataagatatcagtacaatgtcctcccgcagggatggaaagggtcaccagctatattccaaagt
tcgatgacaaaaattctagatccatttaggaaaaacaacccagagctagaaatttgtcagtacgtggatgaccta
tatgtagcatcagatttacccctgacagaacatagaaaacgggtagaattgcttagagaacacttatatcagtgg
gggttcactacccctgataaaaagcatcaaaaggaacctccctttctgtggatggggtatgagctccatccagac
aaatggacagtacagcccatccaattgcctaacaaggacgtatggacagtaaatgatatacaaaaactagtagga
aagttaaattgggcaagtcaaatctatccaggaattagagtaaaagaattgtgtaagttaattagaggcaccaag
tcattgacagaaatagtacccttaagtaaagaagcagagctagaattagaggagaacagagaaaggctaaaagaa
ccagtgcatggtgtatactatcagcctgacaaagacttatgggttaatattcagaagcaaggagaagggcaatgg
acttatcagatatatcaggaggaacataagaacctaaaaacagggaaatactctaggcaaaaggcctcccacaca
aatgatataagactattagcagaagtagtccagaaggtgtctcaagaagctatagttatttggggaaaattgcct
aaatttaagctgccagtcgctagggagacttgggaaacatggtgggcggactattggcaagccacctggattcca
gaatgggaatttgtcagcacacccccattaatcaaattatggtacaggttagaaagtgaccctattagggggca
gaaacatattatgtagatggagcagctaatagagatacaaaattaggaaaggcaggatatgttacagaamgaggg

aaacagaaaataataaaattagargagaccaccaatcaaaaggctgaattaatggcagtattattagccttacag
gattccaaggaagcagtaaacatagtaacagattcacaatatgcattgggcatcatctcctcccaacctacacag
agtgattcccctatagttcagcagataatagaggaactaacaagaaaggaaagggtgtatcttgcatgggttcct
gctcataaaggcataggaggaaatgaaaaaatagataaattagtaagcaaggatattagaagagtcctatttcta
gaaggaatagaccaagcacaagaggatcatgaaaagtatcatagcaattggagagcattagctagtgactttgga
ctaccaccagtggtagccaaagaaatcattgctaattgtcctaaatgtcagacaaaaggagaagcaatgcatgga
caagtagactgcagtccagaagtatggcaaatagattgcacacatacagaaggcaaagtcataatagttgctgtc
catgtggcaagtggatatatagaagcagaagtaataccagcagaaacaggacaagaaactgcctacttcctgtta
aaattagctgcaagatggcctgttaaagtaatacatacagacaacggcctaattttacaagtgccaccatgaag
gctgcatgttggtggaccaacataaaacatgagtttggaataccatataatccacaaagtcaaggagtagtagaa
gccatgaataaagaattaaaatcaattatacagcaggtaagggaccaagcagaacacttaagaacagcagtacag
atggcagtatttgttcacaattttaaaagaaaaggggggattggggggtacactgcaggagaaaggataatagac
atattagcatcacaaatacaaacaacagaattacaaaaacaaattttaaaattcaaaaatttcaggtctattac
agagacagcagagatcctatttggaaaggaccggcacagctcctgtggaaaggtgaaggagcagtagtcatacaa
gataaaggagacattaaggtagtaccaagaaggaaggcaaaaataattagacat
>#69 AY169813 2754 nt
acagcaaaggttgggggccatctatgtgaggttttgctggatacaggggcagatgatacagtactaaacaacata
caattggaaggaagatggacaccaaaaatgataggggggaataggaggttttataaaagtaaaagaatatgataat
gtgacagtagaaatagaaggaaaaaaggtgcagggaacagtattggtgggacctactcctgttaatattattgga
agaaatatattgacaggattgggttgcacattaaacttccctataagccccatagacacagtaccagtaaaacta
aaaccaggaatggatggaccaaaagtaaaacaatggcccctatctagagaaaaaatagaagccttaacagcaata
tgtcaggaaatggaacaagaaggaaaaatttcaagaataggacctgaaaatccttataatacacccatctttgct
ataaaaaagaaagatagtactaagtggagaaaattggtagacttcagagaattaaataaaagaacacaagacttc
tgggaggtacagttaggtatcccacatccgggggtttaaagcaaaaacaatctgttacagttttagatgtagga
gatgcttatttctcatgtcctttagatgagaatttcagaaaatatactgctttcactattcctagtataaacaat
gagaccccaggaataagataccagtacaatgtcctcccacaaggatggaaagggtcaccagctatattccaaagt
tcaatgacaaaaattctagatccattcagaaaaaacaatccagaaatagaaatttgtcagtacatggatgactta
tatgtagggtcagatttgcccttggcagagcatagaaaaagggtagaatcacttagagagcatttatatcggtgg
ggattcactaccccagataaaaagcatcaaaaagaacctccatttatgtggatgggatatgagctccacccagac
aaatggacagtacagcccatccaattgcctgacaaggaagtgtggacagtaaatgatatccaaaaattagtagga
aagttaaattgggcaagtcagatatatcagggaattagagtaaaagaattatgcaagttaatcagaggaaccaag
tcattgacagaagtggtacctttgagtaaagaggcagaactagaactggaagaaaacagggaaaggctaaaagaa
ccagtacatggtgtatattatcaacctaacaaagacttatgggttaatattcagaaacaaggacaagggcaatgg
acttatcaggtgtatcaggatgaacataagaacctcaaaacaggaaaatatgctaggcaaaaggcctcccataca
aatgagataagacaaatagcagaagtagtccagaaggtggctcaagagtctatagtcatctggggaaaattgcct
aaatttaagctgccagtcactagagaaacttgggaagcatggtgggcagactattggcaagccacctggattcct
gaatgggaatttgtcagcacgcccccattaatcaaattatggtaccaattggaaagtgaacctattgtaggagca
gaaacctattatgtagatggagcagctaatagggagacaaaattaggaaaggcaggatatgttacagaacaagga
aaacagaaaataataaatttagaggagaccaccaatcaaaaggctgaattaatggcagtactaatagcattacag
gattccaaagaaaaggtaaacatagtaacagactcacaatatgtattgggcatcatctcctcccaacctacacaa
agtgattcccctatagttcagcagataatagaagaactgacaaaaaaggaacaaacgtatattacatgggttcct
gctcataagggcataggaggaaatgaaaaaatagataaattagtaagcaaagatattagaagggtcttattcctg
gaaggaatagatcaggcacaagaagatcatgaaaaatatcacagcaattggagagcattagctagtgactttgga
ctaccaccagtagtagccaaagaaattattgctagttgtcctaaatgtcatactaaaggggaagcaattcatggt
caggtagattacagtccagagatatggcaaatggattgcacacatttagaaggcaaagtcataatagttgctgtc
catgtggcaagtggattcatagaggcagaagtgataccagcagaaacaggacaagaaactgcctatttcctgtta
aaattagcakcaagatggcctattaaaatattacatacagataatgggcctaattttacaagtgcaaccatgaag
gctgcatgttggtgggcyaacataaaacatgagtttggcataccatataacccacaaagtcaaggggtagtagaa
gccatgaacaaggaattaaagtcaattatacagcaggtgagggaccaagcagagcatttaaaaacagcagtacaa
atggcagtatttgttcacaattttaaaagaaaaggggggattgggggtgtactgcaggagagagattaatagat
atgttagcatcacaaatacaaacaacagaactacaaaaacaaattttaaaattcaaaaatttcgggtctattac
agagacagcagagaccctatttggaaaggaccggcacaactcctgtggaaaggtgagggagcagtagtcatacaa
gataaaggagatatcaaggtagtaccaagaagaaaagcaaaaatactcaggtgt
>#70 AY169805 2754 nt

atagcaagggtgggagrccatctatgtgaagttttactggatacaggggcagatgatacagtactaaacaacata
caattggaaggaaaatggacaccaaaaatgataggggtataggaggttttataaaagtaaaagaatatgataat
gtgacagtagaaatagagggaagagaagtacagggaacagtattggtgggacctactcctgttaatatcattgga
aggaatatattgacaggattaggatgtacactaaatttccctataagccccatagccccagtgccagtaaaacta
aaaccaggaatggatggaccaaaaataaaacartggcccctatctaaagaaaaaatagaagccctgacagcaata
tgtcaggaaatggaacaggaaggaaaaatttcaagaataggrcctgaaaatccttataatacacccatctttgct
ataaaaaagaaagatagtactaagtggagaaarttggtagactttagggaattaaacaagagaacacaagatttc
tgggaggtacagttaggtatcccacatccgggggtytaaagcaaaagaaatctgttacagtcctagatgtagga
gatgcttatttctcatgccccttagacccagacttcagaccatatactgctttcactatcccaagtgtaaacaat
gagaccccaggaataagataccagtacaatgtcctcccgcaaggatggaaaggttcaccagccatattccaaagt
tcaatgataaaaattctagatccatttaggaaagacaacccagaattacaaatttgtcaatacatggatgacctc
tatgtaggatcagatttacctctgacagaacatagaaaaagggttgaattgcttagagaacacttatatcagtgg
ggattcactaccccctgataaaaagcatcaaaaggagcctccctttctgtggatggggtatgagctccatccagac
aaatggacagtacagcccatccaattgcctaacaaggaagtgtggacagtaaatgatatacaaaaactaatagga
aagttaaattgggcaagtcaaatctatcaaggaattagagtaaaagaattgtgtaagttaattagaggcaccaag
tcattgacagaagtagtacctttaagtaaagaggcagagttggaattagaggaaaacagagagaagctaaaagaa
ccagtgcatggtgtatactatcaacctgacaaagacttatgggttaatattcagaagcaaggagaagggcaatgg
acttaccaaatatatcaggatgaacataagaatctcaaaacagggaaatatgctaggcaaagagcctcccacaca
aatgagataagacaattagcagaagtactccaaaaggtgtcccaagaagctatagttatctggggaaaattgcct
aaatttaagctgccaattactagggaaacttgggaagcatggtgggcggactactggcaagccacctggattcca
gaatgggaatttgtcagtacacccccattgatcaaattatggtaccaattagaaagagaacctattataggggca
gaaacttattatgtagatggagcagctaatagaaatacaaaactaggaaaggcaggatatgttacagaacaaggg
aaacagaaaataataaaattagaagagaccaccaatcaaaaggctgaattaatggcagtattattagccttacag
gattccaaagaaaaggtaaatatagtaacagattcacaatatacattgggcatcatctcatcacaacctacacag
agtgaatcccctatagttcaacagataatagaggaactgacaaaaaaggaaaaggtgtatcttacatgggttcct
gctcataaaggcataggaggaaatgaaaaaatagataaattagtgagcaaggatattagaagagtcctattccta
garggaatagaccaggcacaggaagatcatgaaaaatatcatagcaattggagagcattagctagtgactttgga
ctaccaccagtggtggccaaggaaatcattgctagctgtcctaaatgtcatataaaaggagaagcaatacatggt
caggtagactgcagtccagaagtatggcagatagattgcacacatgtagaaggcaaagtcataatagttgctgtc
catgtggcaagtggattcatagaagcagaagtaataccagcagaaacaggacaggaaactgcttacttcctgtta
aaattagctgcaagatggcctgttaagataatacatacagacaatgggcctaattttacaagtgcaactatgaag
gctgcatgttggtggactaacatacaacatgagtttggaataccatataatccacaaagtcaaggagtagtagaa
gccatgaataaggaattaaaatcaatyatacagcargtgagggaccaagcagaacacttaagaacagcagtacaa
atggcagtatttgttcacaattttaaaagaaaagggggggattgggggggtacactgcaggagagaggataatagac
atactagcatcacacatacaaacaacagaattacaaaaacaaattttaaaaattcaaaattttcgggtctattac
agagacagcagagaccctatttggaaaggaccggcccagctcctgtggaaaggtgagggagcagtagtcatacaa
gataaaggagacattaaagtagtaccaagaagaaaggcaaaaataatcagagat
>#71 HQ179987 2754 nt
ccagtaaaaatagggggcatatttgtgaggctctattagatacaggggctgatgacacagtagtagataactta
cctttagaaggaagatggaaaccaaaaatgataggggaataggrggttttataaaagtaaaagaatttgaagaa
gttaagrtagaaatagaaggaagacaagtttatggractgtattagtgggyccaaccccagtaaatataatagga
agaaatattttgacattaattggatgtactttgagtttccccatcagccccatagaaatagtaccagtaaaatta
aaagcaggaatggatgggcctagggtgaagcaatggcccttgtcaaaggaaaaaatagaagctttaagagccatc
tgtcaagagatggaacaggaaggaaaaataacaaaaattgggcctgaaaatccatataacacccccattttttgca
ataaaaaagaaagatggcagcaagtggaggaaattagtagacttcagagaactaaataaaagaacacaagatttc
tgggaagtccaattgggratccctcatccaggaggtcttcagaaaagraaatcagtgaccatattagatgtaggr
gatgcttatttctcctgyccttttagatccagattttagaaaatatacagctttcactatacctagtgtaaataat
gacacaccaggacttagrtatgtgtayaatgttctgcctcagggatggaaggatcaccagccattttycagcat
tcaatgactaagatcttagaaccctttagraagagtaatccagaagtagaaatctaycaatacatggatgatyta
tatgtagggtcagatytgcctttgtctgaacatagacagcgggtagagaaactyagggarcacctctatgtatgg
gggttcacaactcctgacaaaaarcatcaaaaggagcctcctttcctctggatgggatatgagctccatcctgac
aagtggackgtgcaacccatcaaattaccagaaaaggaaagttggacagtaaatgacattcagaagctagtagga
aagttaaattgggctagtcaaatttatccaggaattagggtaaaagagttatgyaaactaattagaggaactaar
tccttaacagaagtagttgcytttactagagaagcagaattagaactagaggaaaataaagaaattttaaaagaa
ccagtgcatggagtttattaccaaccagaaaaggaattaatagtagacatacagaaacaggggggcggacaatgg

acttatcaggtattccaggaagaacataaaaacttgaaaacagggaaatatgccaggcaaaaggctacccacacc
aatgatataaggcaactggcagargtaatacagaaggtatcacaagaaagcatagtgatatggggaaaactaccc
aagtttagactaccagtaaatagaaatgtgtgggagacttggtggtcagactattggcaagccacctggatacct
gagtgggaatttgttagcacacccccctcttattaagctctggtatcagctagaaaaagaccccataccaggaaca
gaaacctttatgtagatggggcagcaaacagagagaccaagttaggyaaagctggatatgtaacagataggggt
aggcagaagataatcaaactagaagaracaactaatcaaaargcagaattagaagcagtgttgttagccttaaaa
gaatcaggagaacaggctaacatagtaacagactcccaatatgtgttaggrattatctcagcaactccagatcaa
agtgactcccccytagtgcaaaaaataatagaagaaatgacaaaaaaggaaaaggtatacctrtcatgggtacca
gcacacaaaggcataggggggtaatgaraayatagayaaattagtcagyaargacattagaagagtgttattcttg
gaaggcatagaccaagcacaggaggatcatgaaaaatatcacagtaattggagagccctagctagtgartttggc
cttccaccaatagtggcaaaagagattatyaacaattgccctaaatgtcatgtaaaggggaagccatgcatgga
caggtagactgcagtccaggaatttggcaactggactgtacccacatggarggaaaaattatcctcgtggcagtc
catgtrgccagtggcttcatggaagcagaagtaattccagtagaaacaggacaagaagctgcatactttgtgctc
aaattggcatcaagatggcctgtaaaagtaatacacacagacaatgggcctaactttactagcgctgcagtaaaa
gcagcctgttggtggcttaatataactcatgagtttgggataccctacaatccccaaagtcagggagtagtggaa
tcaatgaataaagaattaaagaaaattatacatcaggtacgagatcaagctgagcacttaaagacagcagttcaa
atggcagtatttgtccacaattttaaaagaaaaggggggattggggggtacactgctggagacaggatcatagat
attctggctacacaratacaaacaacagaattacaaaaacaaattttaaaaattcaaaattttcaggtctattac
agagacagcagagaccctatttggaaaggaccagcgacactcctgtggaaaggtgaaggggcagtagtcatacaa
gacaaaggagatattaaggtagtccctaggagaaaagcaaaaataattagaaat
>#72 GU111555 2754 nt
ccagtaagagtagggggcatatttgtgaggctttattagatacaggagctgatgacacagtagtagataaatta
cctttagaaggaagatggaaaccaaatatgataggaggaataggggggttttataacagtaaaasaatttgaagwa
gttaaggtagaaatagaaggaagagaagtttacggaactgtattagtrggcccaaccccagtaaayataatagga
agaaatattttgacattaattggatgtactctaagtttccccatcagctccatagaaatagtaccagtrawatta
aaggcaggaawggatgggcctaaggtaaagcmatggcccttrtcaaargaaaaaatagaagctttaagagstatt
tgtcaagagatggaacaggaaggaaaaataacmaaaattgggcctgaaaatccatataacaccccccatctttgca
ataaaaaagaaagatagcagcaartggaggaaattagtakacttcagagaactaaataagagaacacaagatttt
tgggaagtacaghtgggaatccctcatccaggaggtcttaagaaragraaatcagtgacagtattagatgtgggg
gatgcttatttctcctgtcctttagatccagattttagaaaatatacagcttttactatacctagtgtaaataat
gagacaccaggaattagatatcagtataatgttctgcctcagggatggaaaggatcgccagccatctttcagtat
tcaatgactaagatcttagaaccctttagggagagtaayccagaagtagaaatctatcaatacatggatgatcta
tatgtagggtcagatttgcctttgtctgaacatagacagcgggtagagaaacttagggaacacctctatgtatgg
gggtttacaactcctgacaaaaaacatcaaaaggagcctcctttcctctggatgggatatgagctccatcctgac
aagtggacggtgcaacccatcgtattaccagaaaaggaagcttggacagtaaatgacatccagaagctagtagga
aagttaaattgggcaagtcagatttatccaggaataagggtaaaagaattrtgcaaactaattagaggaactaaa
tccttaacagaagtagttgctcttactagagaagcagaattagaactagaggaaaataaagaaattttaaaagaa
ccagtgcatggagtttattaccaaccagaaaaggaattaatagtagacatacagaaacagggagagggacaatgg
acttatcagatataccaggaagaacataaaaacttgaagacagggaaatatgccaggcaaaaggccactcacacc
aatgatataaggcaactggcagaagtaatacagaagacatcacaagaaagcatagtaatatggggaaaattaccc
aagtttagactaccagtaaatagaagtatgtgggagacttggtggtcagactattggcaagccacctggatacct
gaatgggaatttgttagtacacccccctcttattaagctctggtatcagctagaaaaagaccccataccaggaata
gaaacctttatgtagatggggcagcaaacagagagactaaattaggyaaagctggatatgtaacagataggggt
aggcaraagataatcaaactagaaggaacaaccaatcaaaaagcagaattagaagcagtgctgttagccttaaaa
gaatcaggaaaacaggctaacatagtaacagactcccaatatgtrttagggatcatctcagcaactccagatcaa
agtgactccccccctagtgcaaaaaataatagaagaaatgacaaaraaggaaaaagtatacctatcatgggtacca
gcacacaaaggcataggggggtaatgaagatatagayaaaytagtcagtaaagacattagaagrgtgttattccta
gaaggcatagaycaagcacaggargatcatgaaaaatatcacagtaattggaaagccctagctagtgactttggc
cttccaccaatagtggcaaaagagattatyaacaattgtcccaaatgtcatgtaaaggggaagccatgcatgga
caggtagactgcagtccaggaatttggcarctggactgtacccacacagaaggaaaaattatccttgtagcagtc
catgtagccagtggtttcctggaagcagaagtgattccagcagaaacaggacargaaactgcataytttgtgctc
aaattggcagcaagatggcctgtaaaagtgatacacacagacaatgggcctaacttyactagygctgcagtaaar
gcagcctgttggtggcttaatataactcatgagtttgggataccctacaatccccagagtcagggagtagtggaa
tcaatgaataaagaattaaagaaaatcatacttcaagtacgagatcaagctgaacacttaaagacagcagttcaa
atggcagtatttgttcacaattttaaaagaaaaggggggattggggggtacactgctggagacaggatcatagat

attatggctacacaaatacaaacaacagaattacaaaaacaaattttaaaaattcaaaattttcgggtctattac
agagacagcagagaccctatttggaaaggaccagcgacgcttctgtggaaaggtgaaggggcagtagtcatacaa
gacaaaggagatattaaagtagtccctaggagaaaagcaaaaataatcagagat
>#73 DQ017382 2745 nt
ataggaaatgaagtaagagaagctcttttrgatacaggagctgatgatacagtaatagaagaaatacaattagaa
ggaagatggaaaccaaaaatgataggaggaattggaggatttatcaaagtaagacaatatgataatataacaata
gacatacaaggaaggaaagcagttggtacagtgttggtaggaccaacacctgttaatattataggaagaaacttt
ttaacacagattggctgtaccctaaattttccaataagtcctattaaaactgtaccagtaaaattaaaaccagga
atggatggcccaaaagtaaaacaatggcctttgacagcagaaaaaatagaggcattaagagaaatttgtacagaa
atggaaaaggaaggaaaaatttctagaatagggcctgaaaatccatataacactccaatttttgctataagaaag
aaagatagcaccaaatggagaaaattagtagatttcagagaattaaataaaaggacccaagacttttgggaagtg
cagctaggaattccacatccagcaggattaaagcagaaaaagtcagtaacagtcctagatgtaggagatgcttat
ttttcatgtcccttagatgaagattttagaaatatacagcttttaccatacctagtgtaaataatgagacacct
ggtattagataccagtataatgtgttaccacagggatggaaggatcaccagcaatttttcaaagttcaatgaca
acaattytagaaccatttagaaagaagcatccagaaataatcatttatcagtacatggatgacctctatgtggga
tctgacttagacctagcacaacatagagagacagtagaagaacttagaggtcatcttttgaagtggggctttaca
acccctgacaaaaaacatcaaaaggagccaccgttcctctggatgggatatgagctccatccagataaatggaca
gtccagccaataaagttaccagaaaaggatacatggactgtcaatgatatacaaaaattagtaggaaagttaaat
tgggcaagtcagatctatccaggaatcaaagtaagacagctttgtaaattaatcagaggaaccaaggctttgaca
gaagtagttacctttacagaagaagcagaattagaactagcagaaaatagggagatattaaaagaacccctacat
ggagtctattatgacccagaaaaagaattaatagcagaaattcaaaaacaaggacaaagtcagtggacatatcag
atttatcaggaatcayataaaaatttaaagacaggaaagtatgcaaaaatgaaatctgcccatactaatgatata
aaacagttaactgaagtagtacaaaaggtagcaacagaaagtatagtaatttggggaaagactcctaagtttaaa
ttaccmgtacaaaaggaagtgtgggaggcatggtggactgagaattggcaagcaacttggattccagagtgggaa
ttcgttaacactcctccccttgtaaaattatggtatcagttagaaacagagccaattagtggggcagaaacatac
tatgtagatggagcagctaataaagaaacaaaattgggaaaagcaggttttgtgacagatagaggaagacagaaa
gtggtctctcttgaaaacaccaccaatcaaaagactgagttacaagctatccttatggccttacaagatkcagga
caggaagtaaacatagtcactgattctcagtatgctatgggaataattcactcacaaccagataagagtgaatca
gaattggtgagccaaataatagaagaactcataaaaaaggaaagagtctatctctcttgggtrcctgcacataaa
ggtattggaggaaatgagcaggtagacaaattagttagctcaggaattagaaaagtattatttctagatggtata
gaaaaagcccaagaagagcatgaaagatatcacagtaactggaaagcaatggccagtgattttaacttgcccccc
atagtggcaaaagaaatagtagccagctgtgacaaatgccagctaaaaggggaagccatgcatggacaggtcaat
tgtagtccaggagtgtggcagttagattgtacacacttagaaggaaaaatcattcttgtagcggtccatgtggcc
agtggctacttagaagcagaagttattcctgcagaaacaggacakgaaacagcatattttattttaaagttagct
ggaagatggccagtaaaagttatacacactgataatggacccaatttcactagtgccactgtaaaagcagcctgt
tggtgggcaagtatcaaacaggaatttgggataccctacaatcctcaaagtcagggagcagtagagtccatgaat
aaagaattaaagaaaattataggacaaatcagagatcaagcagaacatctgaaaacagcagtacaaatggcggtc
ttcattcacaattttaaaagaaaaggggggattggggggtacactgcaggggaaagaataatagacataatagca
acagacatacagacaacaaaattacaaacacaaattttaaaagttcaaaattttcgggtttattacagagacagc
agagatcccatttggaaaggaccagccaaactgctgtggaaaggagaaggggcagtggtaatccaagataacggg
gatataaaagtagtcccacgtaggaaagcaaaaataattagagat
>#74 AY532635 2745 nt
ataggaaaagaagtaagagaagctcttctagatacaggagctgatgatacagtgatagaagagatacaattagaa
ggaaaatggaagccaaaaatgataggaggaattggaggatttatcaaagtaagacaatatgataatgtaacaata
gacatacaaggaaggaaagcagttggtacagtgttagtaggaccaacacctgttaatattataggaagaaacttt
ttaacacagattggctgtactttaaattttccaataagtcctattgaaactgtaccagtaaaattaaaaccaggg
atggatggcccaagagtaaaacaatggcctttgacagcagaaaaaatagaggcattaagacaaatttgtgcagaa
atggaacaggaaggaaaaatttctagaatagggcctgaaaatccatacaacactccaatctttgctataaaaaag
aaagatagcactaaatggagaaaattagtagatttcagggaattaaataaaaggacccaagattttgggaagta
cagctaggaattccacatccagcaggattacagaagaaaaaatcagtaacagttctagatgtaggagatgcttat
ttttcatgtcccttagatgaagattttagaaagtatacagccttcaccatacctagtacaaataatgagacacct
ggtattagataccagtataatgtgttgccacagggatggaaggggtcaccagcaatttttcaaagttcaatgaca
aaaattttagaaccatttagaaagaaacatccagagataatcatttatcagtacatggatgacctctatgtggga
tctgactcagaactagcacaacatagagagacagtggaagaacttagaagtcatcttttgaagtggggctttacg
acccctgacaaaaaacatcaaaaggagccaccgttcctctggatgggatatgagctccatccagataaatggacg

gtccagacaataaagttaccagaacaggatgtatggactgtcaatgatatacaaaaattagtaggaaagttaaat
tgggcaagtcagatctatccaggaatcaaagtaaaacagctttgtagattaatcagaggaaccaaagctttgaca
gaagtagtcaactttacaaaagaagcagaattagaattagcagaaaacagggagatattaagagaacccctacat
ggagtctactatgacccaggaaaagaactaatagcagaaattcaaaaacaaggacaagatcagtggacatatcag
atttatcaggagccatataaaaatttaaaaacaggaaaatatgcaaaaaggagatctgcccatactaatgatata
aaacagttagttgaagtggtacaaaaagtagcaacagaaggtatagtaatttggggaaagattcctaagtttaaa
ttaccagtacaaaaggaagtgtgggaggcatggtggactgacaattggcaagcaacctggattcctgagtgggaa
ttcgtcaacactcctccccttgtaaaattatggtatcagttagaaacagagccaatcactggggcagaaacatac
tatgtagatggagcagctaataaagaaacaaaattgggaaaagcaggttttgtgacagatagaggaaaacagaaa
gtggtctctattgaagacaccaccaatcaaaagactgagttacaagctatccttctggccttacaagattcagga
caggaagtaaacatagtcactgattctcagtatgctctgggaataattcactcacacccagataaaagtgaatca
gaattggtgagccaaataatagaagagctcataaaaaaggaaagagtttatctctcttgggtacctgcacataaa
ggtattggaggaaatgagcaggtagacaaattagttagctcaggaattagaaaagtattattcctagatggtata
gaaaaagcccaagaagatcatgaragatatcacagtaactggagagcaatggccagtgattttaacttaccctct
gtagtggcaaaagaaatagtagccagctgtgacaaatgccagctaaaaggggaagccatacatggacaggtcaat
tgtagtccaggagtgtggcagctagattgtacacatttagaaggaaaaatcattcttgtagcggtccatgtggcc
agtggctacttagaagcagaagttattcctgcagaaacaggacaggaaacagcatattttattttaaaattagct
ggaagatggccagtaaaagttatacacactgataatggacccaatttcactagtgccactgtaaaagcagcctgt
tggtgggcaaatatcacacaggaattcgggataccctacaatcctcaaagtcagggagtagtagaatccatgaat
aaagaattaaagaaaattataggacaaatcagagatcaagcagaacatctaaaaacagcagtgcaaatggcggtg
ttcattcacaattttaaaagaaaaggggggattggggagtacactgcaggggaaagaataatagacataatagca
acagacatacaaacaacaaaattacaaacacaaattttaaagattcaaaattttcgggtttattacagagacagc
agagatcccatttggaaaggaccagccaaacttctgtggaaaggagaaggggcagtggtaatccaagataacgga
gatataaaagtagtcccacgtaggaaagcaaaaataattagggat
>#75 AJ006022 2745 nt
atagggaaagaagtaagagaagctcttttagatacaggagctgatgatacagtaatagaagagctacaattagag
ggaaaatggaaaccaaaaatgataggaggaattggaggatttatcaaagtgagacaatatgataatataacagta
gacatacagggaagaaaagcagttggtacagtattagtaggaccaacacctgttaatattataggaagaaatctt
ttaacccagattggctgtactttaaattttccaataagtcctattgaaactgtaccagtaaaattaaaaccagga
atggatggcccaaaggtaaaacaatggcctttgacaacagaaaaaatagaggcattaagagaaatttgtacagaa
atggaaaaggaaggaaaaatttctagaatagggcctgagaatccatataacactccaatttttgctataaaaaag
aaagatagcactaaatggagaaaattagtagatttcagggaattaaataaaaggacccaagattttgggaagtg
cagctaggaattccacatccagcaggattaaagcagaaaaaatcagtgacagttttggatgtaggagatgcttat
ttttcatgtcccttggacaaagattttagaaagtatacagcttttaccatacctagtataaacaatgagacacct
ggtattagataccagtataatgtgctgccacaaggctggaaagggtcaccagcaatttttcagagtacaatgaca
aaaattctagaaccattcagagagaaacatccagagataatcatttaccagtacatggatgacctctatgtggga
tctgacttagaactagcacaacatagagaggcagtagaagaccttagagatcatcttttgaagtggggctttacg
acccctgacaaaaaacatcagaaggaaccccccgttcctctggatgggatatgaactccatccagacaaatggaca
gtccagccaataaagttaccagaaaaggatgtatggactgtcaatgatatacagaaattagtaggaaagttaaat
tgggcaagtcagatctatccaggaatcagagtaaaacagctctgtaaattaatcagaggaaccaaagctttgaca
gaagtagtcaactttacagaagaagcagaattagaactagcagaaaacagggagatattaaaagaacccctgcat
ggagtctattatgacccaggaaaagaattagtagcagaaattcaaaagcaaggacaaggtcagtggacatatcag
atttatcaggagttacataaaaatttaaaaacaggaaagtatgcaaaaatgagatctgcccatactaatgatata
aaacagttagttgaagtggtaaggaaagtggcaacagaaagtatagtaatttggggaaagactcctaaatttaga
ttaccagtacaaaaggaagtgtgggaggcatggtggaccgatcattggcaagcaacttggattcctgagtgggaa
tttgtcaacactcctccccttgtaaaattatggtatcagttagaaacagagccaatcagtggggcagaaactttc
tatgtagatggagcagctaatagggaaacaaaattgggaaaagcaggttttgtgacagataggggaagacagaaa
gtggtctctattgcagacaccaccaatcaaaaggctgagttacaagctatccttatggccttacaagagtcagga
cgggatgtaaacatagtcactgactctcagtatgctatgggaataattcattcacagccagataaaagtgaatca
gaattggtgagccaaataatagaagagctcataaaaaaggaaagagtttatctctcttgggtacctgcacataaa
ggtattggaggaaatgagcaggtagacaaattagttagctcaggaattagaaaaatattattcctagatggtata
gaaaaagcccaagaagatcatgacagatatcacagcaattggaaagcaatggccagtgattttaacttaccccc
atagtggcaaaagaaatagtagccagctgtgacaaatgccagctaaaggggaagccatgcatggacaggtcaat
tgtagtccaggagtgtggcaattagattgtacacacttagagggaaaaatcatccttgtggcggtccatgtggcc
agtggctacttagaagcagaagttattcctgcagagacaggacaggaaacagcatattttattttaaagttagct

```
ggaagatggccagtaaaagttatacacactgataatggatccaatttcactagtgccactgtaaaagcagcctgt
tggtgggcaaatatcaaacaggaatttgggataccctacaatcctcaaagtcagggagcagtagagtccatgaat
aaagaattaaagaaaattataggacaaatcagagatcaagcagaacatctaaagacagcagtgcaaatggcggtt
ttcattcacaattttaaaagaaaaggggggattggggggtacactgcaggggaaagaataatagacataatagca
acagacatacagacaacaaatttacaaacacaaattttaaaagttcaaaattttcgggtttattacagagacagc
agagatcccatttggaaaggaccagccaaacttctgtggaaaggagaaggggcagtggtaattcaagataacggg
gatataaaagtagtcccacgtaggaaagcaaaaataattagggat
>#76  MF7672  2745 nt
ataggaaaggaagtaagagaagctcttttagatacaggagctgatgacacagtaatagaagaaatacaattagaa
ggaaaatggaagccaaaaatgataggaggaattggaggatttatcaaagtaagacaatatgataatataacaata
gacatacaaggaaggaaagcagttggtacagtattagtaggaccaacacctgtcaatattataggaagaaacttt
ttaacacagattggctgtactttaaattttccaatcagttctattgaaactgtaccagtaaaattaaaaccagga
atggatggcccaagggtaaaacaatggccattgacagaagaaaaaatagaggcattaagagaaatttgcacagag
atggaaaaggaaggaaaaatttctagaatagggcctgaaaatccatataacaccccaatttttgctataaaaaag
aaagatagcactaaatggagaaaattagtagatttcagagagttaaataaaaggactcaagattttgggaagtg
cagctaggaattccacatccagcaggattaaagcagaaaaaatcagtgacagttttagatgtaggagatgcttat
ttttcatgtcccttagataaagagtttagaaagtatacagcttttaccatacctagtgtgaataatgagacacct
ggtattagataccagtataatgtgttgccacagggatggaagggatcaccagcaattttcaaagttcaatgaca
aaaattttagaaccattcaggaagagacacccagagatagtcctttatcartacatggatgacctctatgtggga
tcagacttagaaatagcacaacatagagagacagtagaagaacttagaggtcatcttttgaagtggggctttaca
accccctgacaagaaacatcaaaaggagcctccattcctctggatgggatatgagctccatccagataaatggacg
gtccagccaataaagttaccagaaaaggaggtatggactgtcaatgatatacagaaattagtaggaaaattaaat
tgggcaagtcagatctatccaggaatcaaagtaaaacatctttgtagattaatcagaggagccaaggctttgaca
gaagtagtcacttttacacaagaagcagaattagaactagcagaaaacagggagatattaaaagaaaccctgcat
ggagtctactatgacccagaaaaagaattaacagcagaaattcaaaaacaaggacaaggtcagtggacttatcag
atttatcaggagcaatataaaaatttaaaaacaggaaagtatgcaaaaatgaaatctgctcatactaatgatata
aaacagttagctgcagtggtacaaaaagtggcgacagaaagcatagtaatttggggaaaaattcctaagtttaaa
ttaccagtacaaaaggaagtgtgggaggcatggtggactgataattggcaagcaacttggattcctgagtgggaa
tttgtcaacacccctcccctwgtaaaattatggtatcagttagagacagagccaattattggggtagaaacatac
tatgtagatggagcagctaatagagaaacaaaattggggaaagcaggttttgtgacagacagaggaagacagaaa
gtggtctctattgaaacaccaccaatcagaaggctgagttacatgctatccttatggctttacaagagtcagga
caggaagtaaacatagtcactgactctcagtatgctatgggaataattcactcacaaccagatacaagtgaatca
gacttggtgggtcaaataatagaagaactcataagaaaggaaagagtctatctctcttgggtacctgcacataaa
ggcattggaggaaatgagcaggtagataaattagttagctcaggaattagaaaagtattattcctagatggtata
gaaaaagcccaagaagagcatgaaagatatcacagtaattggaaagcaatggccagtgattttaacttacccct
gtagtagcaaaagaaatagtagccagttgtgacaaatgccaaataaaaggggaagccatgcatggacaggtcaat
tgtagtccaggagtgtggcaattagattgtacccacttagaaggaaaaatcattctggtagcggtccatgtggcc
agtggctacttagaagcagaagttattcctgcagaaacaggacaggaaacagcatatttatttaaagttagct
ggaagaaggccagtaaaagtcatacacactgataatggaaccaatttcactagtaacactgtaaaagcagcctgt
tggtgggcaagtatcaaacaggaattcgggataccctacaatcctcaaagtcagggagcagtagaatccatgaat
aaagaattaaagaagattataggacaaatcagagatcaagcagaacatctaaaaacagcagtgcaaatggcagtc
ttcattcacaattttaaaagaaaaggggggattggggggtacactgcaggggaaagaataatagacataatagca
acagacatacaaacaacaaaattacaaacacaaattttaaaagttcaaaattttcgggtttattacagagacagc
agagatcccatttggaaaggaccagccaagctactgtggaaaggagaaggggcagtggtaatccaagataacggg
gatataaaagtagtcccacgtaggaaagcaaaaataattagggat
>#77  KY498771  2745 nt
atagggaaagaagtaagggaagctcttttagatacaggagctgatgatacagtaatagaagaactacaattagag
ggaaaatggaaaccaaaaatgataggggggaattggaggatttatcaaagtaagacaatatgataatgtaacagta
gatatacagggaagaaaagcagttggtacagtattagtaggaccaacacctgttaatattataggaagaaatctt
ttaacacagattggctgtactttaaattttccaataagtcctattgaaactgtaccagtaaaattaaaaccagga
atggatggcccaaaggtaaaacaatggcctttgacagcagaaaaaatagaggcattgagagaaatttgtacagaa
atggaaaaggarggaaaaatttctaaaatagggcctgagaatccatataacactccaatttttgctataaaaaag
aaagatggcactaaatggagaaaattagtagatttcagagaattaaataaaaggacccaagattttgggaagtg
cagttaggaattccacatccagcaggattaaagcagaaaaaatcagtgacagttttggatgtaggagatgcttat
ttttcatgtcctttggatgaagatttcaggaagtatacagcttttaccatacctagtataaacaatgagacacct
```

ggtattagataccagtataatgtgttgccacagggctggaaagggtcaccagcaatttttcaaagttcaatgaca
aagattctagacccatttagaaagaaacatccagagataatcatttatcagtacatggatgacctctatgtggga
tctgacttagaactagcacaacatagagaggtagtagaagaacttagaaggcatcttttggattggggctttacg
acccctgacaaaaaacatcaaaaggagccaccgtttctctggatgggatatgagctccatccagataaatggacg
gtccagccaataaagttgccagaaaaagagatatggactgtcaatgatatacaaaaattagtagggaaattaaat
tgggcaagtcagatctatccaggaatcaaagtaaaacagctgtgtagattaatcagaggaaccaaagctttgaca
gaagtagttgactttacaaaagaagcagaattagaactagcagaaaacagggagatattaaaagaaaccatgcat
ggagtctactatgacccaggaaaagaattaatagcagacattcagaaacaaggacaaggtcaatggacatatcag
atctatcaagagccatataaaaacctgaaaacagggaaatatgcaaaaatgagatctgcccatactaatgatata
aaacagttagttgaagtagtacagaaggtggcaacagaaagtatagtaatttggggaaggactcctaaatttaga
ttaccagtacaaaaggaagtgtgggagacatggtggactgatcattggcaagcaacttggattcctgagtgggaa
tttgtcaatacycctccccttgtaaaattatggtatcagttagaaacagagccaatcagtggggtagaaacatac
tatgtggatggagcagctaatagagaaacaaaattgggaaaagcaggttttgtgacagatagaggaagacagaaa
gtggtttctcttgcagacaccaccaatcaaaaggctgaattacaagctatccttatggccttacaagagtcagga
caggcagtaaacatagttactgactcccagtatgctatgggaataatacatgcacaaccagataaragtgaatca
gaattggtaagccaaataatagaagaactcataaaaaaggagaaagtttatctctcatgggtacctgcacataaa
ggtattggaggaaatgagcagatagacaaattagtcagctcaggaattaggaaagtattattcttggatggcata
gacaaagcccaagaggatcatgaaagatatcacagtaattggagagcaatggctagtgattttaacctaccccc
gtagtggcaaaagaaatagtagccagctgtgacaaatgccagctaaaaggggaagccatacatggacaggtcaat
tgtagtccaggagtatggcagttagactgtacacacttagaagggaaaatcattcttgtagcagtccatgtggcc
agtggctacttagaagcagaagttattcctgcagaaactggacaggaaacagcatattttattttaaagttagct
ggaagatggccagtaaaagttatacacactgataatggacccaatttcactagtgccactgtaaaagcagcttgt
tggtgggcaaacatcaaacaggaatttgggataccctacaatcctcaaagtcagggagcagtagagtccatgaat
aaagaattaaagaaaattataggacaaatcagagatcaagcagaacatctaaaaacagcagtgcaaatggcggtt
ttcattcacaattttaaaagaaaaggggggattggggggtacactgcaggggaaagaataatagatataatagca
acagacatacagacaacaaaattacaaacacaaattttaaaagttcaaaattttcgggtttattacagagacagc
agagatcccatttggaaaggaccagccaaacttctgtggaaaggagaaggggcagtggtaatccaagataacggg
gatataaaagtggtgccacgtaggaaagcaaaaataattagggat
>#78 DQ017383 2745 nt
ataggaaatgaagtaagagaagctcttttagacacaggagctgatgatacagtaatagaagaaatacaattagaa
gggagatggaaaccaaaaatgataggggggaattggaggatttatcaaagtaagacaatatgataatataacaata
gacatacaaggaaggaaagcagttggtacagtgttagtaggaccaacacctgttaatattataggaagaaacttt
ttaacacagattggctgtactctaaattttccattaagtcctattaaaactgtaccagtaaaattaaaaccagga
atggatggcccagaagtaaaacaatggcctttgacagcagaaaaaatagaggcgttaagagaaatttgtacagaa
atggaaaaggaaggaaaaatttctagaatagggcctgaaaatccatataacactccaatttttgctataaaaaag
aaagatagcactaaatggagaaaattagtagatttcagagaattaaataaaaggacccaagacttttgggaagtg
cagctgggaatcccacatccagcaggattaaagcagaaaaagtcagtaacagtcttggatgtaggagatgcttat
ttttcatgtcccttagatgaagattttagaaagtatacagtttttaccatacctagtgtaaacaatgagacacct
ggtattagataccagtataatgtgttaccacagggatgggagggatcaccagcaatttttcaaagttcaatgaca
acaattctagaaccatttagaaagaagcatccagagataatcatttatcagtacatggatgacctctatgtggga
tctgacttagaaatagcaaaacatagagagacagtagaagaacttagaggccatctgttgaagtggggctttaca
acccctgacaaaaaacatcaaaaggagccaccattcctctggatgggatatgagctccatccagataaatggaca
gtccggccaataaagttaccagaaaaggatacatggactgtcaatgatatacagaaattagtagggaaattaaat
tgggcaagtcagatttatccaggaattaaagtaagacagctttgcaaattaatcagaggaaccaaggctttgaca
gaagtagttacctttacagaagaagcaggattagaactagcagaaaacagggagatattaaaagaacccctacat
ggagtctattatgacccagaaaaggaattaatagcggaaattcaaaaacaaggacaaagtcagtggacatatcag
atttatcaggagtcacataaaaatttaaagacaggaaagtatgcaaaaatgagatctgcccatactaatgatata
aagcagttaactgaagtagtacaaaagatagcaacagaaagtatagtaatttggggaaagactcctaagtttaaa
ttaccagtacaaaaggaagtgtgggaggcatggtggactgagaattggcaagcaacttggattcctgagtgggaa
ttcgtcaacacccctccccctgtaaaattatggtatcagttagaaacagagccaattagtggggcagaaacatac
tatgtagatggagcagctaatagagaaacaaaattgggaaaagcaggttttgtgacagatagaggaagacagaaa
gtggtctctattaaagataccaccaatcaaaaaactgagttacatgctatcctcatggccttacaagagtcagga
caggaagtaaacatagtcactgactctcagtatgctatgggaataattcactcacagccagataaaagtgaatca
gaattggtgagccaaataatagaagaactcataaaaaaggaaagagtctatctctcttgggtacctgcacataaa
ggtattggaggaaatgagcaggtagacaaattagttagctcaggaattagaaaagtattattcctagatggtata

gaaaaagcccaagaagagcatgaaagatatcacagtaactggagagcaatggccagtgattttaacttrcccccc
atagtggcaaaagaaatagtagccagctgtgacaaatgccagctaaaaggggaagccatgcatggacaggtcaat
tgtagtccaggagtgtggcagttagattgtacacacttagaaggaaaaatcattcttgtagcagtccatgtggcc
agtggctacttagaagcagaagttattcctgcagaaacaggacaggaaacagcatattttattttaaagttagct
ggaagatggccagtaaaagttatacacactgataatggacccaatttcactagtgccactgtaaaagcagcctgt
tggtgggcaagtatcaaacaggaattcgggataccctacaatcctcaaagtcagggagcagtagagtccatgaat
aaagaattaaagaaaattataggacaaatcagagatcaagcagaacatctaaaaacagcagtgcaaatggcagtc
ttcattcacaattttaaaagaaaaggggggattggggggtacactgcaggggagagaataatagacataatagca
acagacatacagacaacaaaattacaaacacaaattttaaaagttcaaaattttcgggtttattacagagacagc
agagatcccatttggaaaggaccagccaaacttctgtggaaaggagaaggggcagtggtaatccaagataacggg
gatataaaagtagtcccacgtaggaaagcaaaaataattagagat
>#79 AJ271370 2745 nt
ataggaaaggaaataagagaagctcttttagatacaggagctgatgatacagtaatagaagaaatacaattagaa
ggaaaatggaaaccaaaaatgataggaggaattggaggatttatcaaagtaagacaatatgataatataacaata
gacatacaaggaagaaaagcagttggtacagtgttagtaggaccaacacctgttaatattataggaagaaacttt
ttaacacagattggctgtactttaaattttccaatcagtcctattgaaactgtaccagtaaaattaaaaccaggg
atggatggcccaagggtaaaacaatggcctttgacagcagaaaaaatagaggcattaagagaaatttgtacagaa
atggaaaaagaaggaaaaatttctagaatagggcctgaaaatccatataacactccaatttttgctataaaaaag
aaagatagcactaaatggagaaaattagtagatttcagggaattaaataaaaggacccaagaattttgggaagtg
caactaggaattccacatccagcaggattaaagcagaaaaaatcagtaacagttmtagatgtaggagatgcttat
ttttcatgtcccttagataaagatttagaaagtatacagcttttaccatacctagtataaataatgagacacct
ggtattagataccagtataatgtgttaccacagggatggaagggatcaccagcaatttttcaaagttcaatgaca
aaaattctagaaccatttaggaagaaacatccagagataattatttatcagtacatggatgacctctatgtggga
tctgacttagaaatagcacaacatagagagacagtagaagaacttagaggtcatcttttgaagtggggctttacg
accccctgacaaaaaacatcaaaaggagccaccgttcctctggatgggatatgagctccatccagataaatggacg
gtccagccaataaagttaccagaaaaggaggtatggactgtcaatgatatacaaaaattagtaggaaagttaaat
tgggcaagtcagatctatccaggaatcaaagtaaaacagctttgtaaattaatcagaggaaccaaggctttgaca
gaagtagtcacttttacacaagaagcagaattagaactagcagaaaacagggagatattaaaagaacccctacat
ggagtctattatgacccaggaaaagaattaatagcagaaattcaaaaacaaggacaaggtcagtggacatatcag
atttatcaggagccatataaaaatttaaaaacaggaaagtatgcaaaaaygagatctgctcatactaatgatata
aaagagttagctgcagtggtacaaaaggtggcaacagaaagtatagtaatttggggaaagactcctaagtttaaa
ttaccagtacaaaaggaagtgtgggagacatggtggactgagcattggcaagcaacttggattcctgagtgggaa
ttcgtcaacactcctccccttgtaaaattatggtatcagttagaaacagagccaattagtggggcagaaacatac
tatgtagatggagcagctaataaagaaacaaaattaggaaaagcaggttttgtgacagatagaggaagacagaaa
gtggtctctattgagaacaccaccaatcaaaaggctgagttacaagctatccttttggccttacaagagtcagga
caggaagcaaacatagtcactgactctcagtatgctatgggaataattcactcacaaccagataaaagtgaatca
gacttggtgggccaaataatagaagaactcataaaaaaggaaagagtctatctctcttgggtacctgcacataaa
ggtattggaggaaatgagcaggtagataawttagttagctcaggaattagaaragtattattcctagatggtata
gaaaaagcccaagaagaacatgaaagatatcacagtaattggaaagcaatggccagtgattttaacttaccccccc
atagtagcaaaagaaatagtagccagctgtgacaaatgccagctaaaaggggaagccatgcatgggcagatcaat
tgtagtccaggagtgtggcagttagattgtacacacttagaaggaaaaatcattcttgtagcagtccatgtggcc
agtggctacttagaagcagaagttattcctgcagaaacaggacaggaaacagcatattttattttaaagttagct
ggaagatggccagtaaaagttatacacactgataatggacccaattttattagtgccactgtaaaagcagcctgt
tggtgggcaggtatcaaacaggaattcgggataccctacaatcctcaaagtcagggagcagtagagtccatgaat
aaagaattaaagaaaattataggacaaatcagggatcaagcagaacatctaaaaacagcagtgcaaatggcggtt
ttcattcacaattttaaaagaaaaggggggattggggggtamacggcaggggaaagaataatagacataatagca
acagacatacaaacaacaaaattacaaacacaaattttaaaagttcaaaattttcrggtttattacagagacagc
agagatcctatttggaaaggaccagccaaacttctgtggaaaggagaaggggcagtggtaatccaagataacggg
gatataaaggtagtcccacgtaggaaagcaaaaataattagagat
>#80 DQ314732 2745 nt
ataggaggacagctgaaagaagctctattagatacaggagcagatgatacagtattagaagatataaatttgcca
ggaaaatggaaaccaaaaatgataggggggaattggaggttttatcaaggtaaggcagtatgatcaaatacctata
gaaatttgtggaaaaaaggctataggcacagtattagtaggacctacacctgtcaacataattggacgaaatatg
ttgactcagcttggttgtactttaaatttcccaattagtcctattgacactgtaccagtaacattaaagccagga
atggatggaccaaaggttaaacagtggccattaacagaagaaaagataaaagcattaacagaaatttgtaaagag

atggaagaggaaggaaaaatctcaaaaattgggcctgaaaatccatacaatactccagtatttgctataaagaaa
aaggacagcaccaaatggagaaaattagtagatttcagagagctcaataaaagaactcaggacttttgggaagtt
caattaggaataccgcatccagcaggtttaaaaaaggcaaaatcagtaacagtactagatgtgggagatgcatat
ttttcagttcctttagatgaaagctttagaaagtatactgcatttaccatacctagtataaacaatgagacacca
ggaatcagatatcaatacaatgtgctgccacagggatggaaaggatcaccggcaatattccagtgtagcatgaca
aaaatcttagagccctttagaataaaaaatccagaaatggttatctatcaatacatggatgacttgtatgtagga
tctgacttagaaatagggcagcacagagcaaaaatagaggagctaagagctcatctattgagctggggatttact
acaccagacaaaaagcatcagaaggaacctccgttcctttggatgggatatgaactccatcctgacagatggaca
gtccagcctatagaactgccagaaaaagacagctggactgtcaatgatatacagaaattagtgggaaaactaaat
tgggcaagtcaaatttatgcagggattaaggtaaagcaactgtgtaaactcctcaggggagctaaagcactaaca
gacatagtaccactgactgaagaagcagaattagagttggcagagaacagggagattctaaaacaccctgtgcat
ggagtatattatgacccgtcaaaagacttagtagcagaagtacagaaacaagggcaggaccaatggacatatcaa
atttatcaagagccatttaaaaatctgaaaacagggaaatatgccagaaaaaggtctgctcacactaatgatgta
agacaattaacagaagtggtgcaaaaaatagccacagaaggcatagtaatatggggaaagacccctaaatttaga
ctacccatacaaggagaaacatgggaaacatggtggatggagtattggcaggctacctggattcctgaatgggag
tttgtcaatacccctcctctagtaaaattatggtaccaattagaaaaagaccctatagtaggagcagagactttc
tatgtagatggggcagccagtagggagactaagctaggaaaagcagggtatgtcactaatagaggaagacaaaag
gtagtttccctaactgagacaaccaatcaaaagactgagttacatgcaatccatttagccttgcaggattcagaa
ccagaagtaaatatagtaacagactcacaatacgcattaggaatcattcaggcacaaccagacaggagtgaatca
gaagtagtcaaccaaataatagaggagctgataaaaaaggaaaaggtctacctgtcatgggtaccagcacacaag
gggattggaggaaatgaacaagtagataaattagtcagttcaggaatcaggaaggtgctattttttagatgggata
gataaggctcaagaagaacatgaaagatatcacagcaattggagaacaatggctagtgattttaatttgccacct
atagtagcaaaggaaatagtagccaactgtgataaatgtcaactaaaaggggaagctatgcatggacaagtagac
tgtagtccagggatatggcaattagattgcacacatctagaaggaaaagtcatcctggtagcagtccacgtggcc
agtggatatatagaagcagaagttattccagcagaaacaggacaggaaacagcatactttctgctaaaattagca
ggaagatggccagtaaaagtaatacacacagacaacggtagcaatttcaccagcgctgcagttaaagcagcctgt
tggtgggccaatgtccgacaggaattcgggatcccctacaatccccaaagtcaaggagtagtagaatctatgaat
aaagaattaaagaaaatcatagggcaggtaagagagcaagctgaacaccttaagacagcagtacaaatggcagta
ttcatacacaattttaaaaggaaaggggggattggggagtacagtgcaggggaaagaataatagacataatagca
acagacatgcaaactaaagaattacaaaaacaaattacaaaaattcaaaattttcgggtttattacagggacagc
agagacccaatttggaaaggaccagcaaaactactctggaaaggtgaaggggcagtagtaatacaagacaatagt
gatataaaggtagtaccaagaagaaaagcaaagatcattagggat
>#81 AF377956 2754 nt
acaataaaagtagaggggcaactaagggaggcactattagatacaggggcagatgatacagtattagaagatata
aatttgtcaggaaaatggaaaccaaggatgataggggggaattggaggttttatcaaagtaagacagtatgaccaa
atacccatagaaatttgcggacaaaaggctataggtactgtattagtaggacctacgcctgtcaacataattgga
agaaatatgttgactcagattggttgcactttaaattttccaattagttctattgaaactgtaccagtaaaatta
aagccaggaatggatggcccaaaggttaaacaatggccattgacagaagaaaaaataaaagcattaacagaaatc
tgtacagagatggaaaaagaaggaaaaatttcaaaaattgggccagaaaatccatacaatactccagtatttgcc
ataaagaaaaaggacagtactaaatggagaaaattagtagatttcagagaacttaataaaagaactcaagatttt
tgggaggttcaattaggaataccacaccctgcagggttaaaaaagaaaaaatcagtaacagtactagatgtgggg
gatgcatatttttcagttcccttagataaggagttcaggaagtacactgcattcaccatacctagtatcaacaat
gagacgccaggaattagatatcagtacaatgtgcttccacagggatggaaagggtcaccagcaatattccaaagt
agcatgacaaaaatcttagagccctttagagtaaaaaatccagaaatagttatttaccaatacatggatgatttg
tatgtagggtctgacttagaaataggacagcataggacaaaaatagaagagttaagagaacatctattgagatgg
ggatttactacaccagataaaaaacatcagaaggaacccccatttctttggatggggtatgaactccatcctgac
aaatggacagtacaggctatacaattgccagacaagagcagctggactgtcaatgatatacagaagttagtggga
aaactaaattgggcaagtcagatttatccagggattagagtaaagcacctatgcaaactccttagaggagccaaa
gcgctaacagacgtagtgccactaacagtagaagcagaattagaaatggcagagaacagggaaattctaaaagaa
ccagtacatggggtatattatgacccatcaaaagatctaatagcagaagtacagaaacaagggcacgaccaatgg
acatatcaaatttatcaagagccacataaaaatctgaaaacaggaaagtatgcaagaaggaggtctgcccacact
aatgatgtaaaacaattaacagaagtagtgcaaaaaatagccacagaaggcatagtaatatggggaaaagttcct
aaatttaggctacccatacaaaaagaaacatggaaacatggtggacagagtattggcaggccacctggattcct
gaatgggagtttgtcaatacccctcctctagtaaaattatggtatcaattagaaacagagcccatattaggagca
gaaactttctatgtagatggggcagctaatagagagaccaaaataggaaaagcaggatatgttactgacagagga

agacaaaaagttgtcccccctaactgagacaacaaatcagaagactgaattacaagcaattcatttagctttgcag
gactcaggatcagaagtaaacatagtaacagactcacagtatgcattaggaatcattcaagcacacccagataag
agtgaatcagagttagtcaaccaaataatagagcaattaatacaaaaagaaagggtctacctgtcatgggtacca
gcacataaaggaattggaggaaatgaacaagtagataaattagtcagtgctggcatcaggaaagtactgttttg
gatgggatagataaggctcaagaagaacatgaaaaatatcacagcaattggagagcaatggctagtgattttaat
ttgccacctgtagtagccaaagaaatagtagccagctgtgataaatgtcagctaaaggggaagccatgcatgga
caagtagactgcagtccagggatatggcaactagattgtacacatttagaaggaaaaattatcttggtagcagtc
catgtggctagtggttatatagaagcagaagttatcccagcagaaacgggacaggaaacagcctacttcctacta
aagttagcaggaagatggccagtaaaaataatacatacagataatggcagcaatttcaccagtactgtggttaag
gcagcctgttggtgggcaggtatccagcaggaatttggaattccctacaatcctcaaagtcaaggagtagtagaa
tctatgaataaagaattaaagaaaattataggacaggtaagagatcaagctgaacatcttaagacagcagtacaa
atggcagtattcattcacaattttaaaagaaaaggggggattggggggtacagtgcaggggaaagaataatagac
ataatagcaacagacatacaaactaaagaattacaaaaacaaattacaaaaattcaaaactttcgggtttatttc
agggacagcagagacccaatttggaaaggaccagcaaagctactctggaaaggtgaaggggcagtagtcatacaa
gacaataatgaaataaaagtagtaccaagaagaaaagcaaaaatcattagggat
>#82 AB254156 2745 nt
gtaggggccagataaaggaagctctcttagatacaggagcagatgatacagtattagaagaaataaatttgcca
ggaaaatggaaaccaaaaatgataggagggattggaggttttatcaaagtaagacagtatgatcaaataactata
gaaatttgtggaaaaagggctgtaggttcagtattagtagggcctacacctgtcaacataattggaagaaatatg
ttgactcagcttggatgtacactaaattttccaattagtcctattgagactgtaccagtgaaattaaagccagga
atggatgggccaaaagttaaacaatggccattgacagaagaaaaaataaaagcattaacagcaatttgtgaggaa
atggagaaggaaggaaaaattacaaaaattgggcctgaaaatccatataatactccagtacttgccataaaaaag
aaggacagtactaagtggagaaaattagtagatttcagggaactcaataaaagaactcaagacttttgggaagtt
caattaggaataccacacccagcagggttaaaaaaggagaaatcagtgacagtgctagatgtgggggatgcatat
ttttcagttcctttagatgagaacttcaggaaatatactgcattcaccatacctagtataaacaatgaaacacca
gggattagatatcaatataatgtgcttccacagggatggaaaggatcaccagcaatattccagagtagcatgaca
aaaatcttagagccctttaggacaaaaaacccagaaatagttatctatcaatatatggatgacttgtatgtagga
tctgacttagaaatagggcaacatagagcaaaaatagaggagttaagagaacatttattgaaatggggatttacc
actccagacaaaaaacatcagaaagaacccccatttctgtggatggggtatgaactacatcctgacaaatggaca
gtacagcctatacagctgccagaaaaagaaagctggactgtcaatgatatacagaagttagtgggaaaattaaac
tgggcaagtcagatttatccaggaattaaagtaaagcaactgtgtaagctccttaggggagccaaagcactaaca
gacatagtaccactgactgaagaagcagaattagaattggcagagaacagggaaattctaagagaaccagtacat
ggagtatattatgacccatcaaaagacttaatagctgaaatacagaaacagggacatgaccaatggacatatcaa
atttaccaagacccattcaaaaatctgaaaacagggaagtatgcaaaaatgaggactgcccacactaatgatgta
agacagttagtagaggcagtgcaaagaataactatggaaagtatagtaatatggggaaagactcctaaatttaga
ctacccatccaaaaagacacatgggagacatggtggacagactattggcaagccacctggattcctgaatgggag
tttgttaatacccctcccctagtaaaattatggtaccagctagagaaagaacccatagtaggagcagaaactttc
tatgtagatggagcagctaatagggaaactaaaataggaaaagcaggatatgttacagatagaggaagacagaaa
attgtttctctaactgaaacaacaaatcagaagactgaattacaagcaattcagctagctttgcaggattcagga
tcagaagtaaacatagtaacagactcacagtatgcattaggaataattcaagcccaaccagataagagtgaatca
gagttagttaatcaaataatagaacagttaataaacaaggaaaaagtctacctgtcatgggtaccagcacataaa
ggaattggaggaaatgaacaagtagacaaattagtaagtagtggaatcaggagagtgctgtttctagatggaata
gataaggctcaagaagagcatgaaaaatatcacagcaattggagagcaatggctagtgaattcaatctgccaccc
atagtagcaaaagaaatagtagctagctgtgataaatgtcagctaaaggggaagccatacatggacaagtggac
tgtagtccagggatatggcaattagattgtacacatttagaaggaaaaatcatcctagtagcagtccatgtagcc
agtggctacatagaagcagaggttattccagcagaaacaggacaggaaacagcatacttcatactaaaattagca
ggaagatggccagtcaaagtaatacacacagacaatggcagtaatttcaccagtgctgcagttaaggcagcctgt
tggtgggcaggtatccaacaagaatttggaattccctacaatccccaaagtcagggagtagtagaatccatgaat
aaagaattaaagaaaatcatagggcaagtaagagagcaagctgagcaccttaagacagcagtacaaatggcagta
ttcatccacaattttaaaagaaaaggggggattggggggtacagtgcaggggaaagaataatagacataatagca
acagatatacaaactaaagaactacaaaaacgaattatacaaattcaaaattttcgggtttattacagagacagc
agagaccctatttggaaaggaccagccaaactactctggaaaggtgaaggggcagtagtaatacaagataatagt
gacataaaggtagtaccaaggaggaaagtaaaaatcattaaagac
>#83 MF373132 2745 nt

gtagggggacagataaaggaggctctcttagacacaggagcagatgatacagtattagaagaaataaaattacca
ggaaattggaaaccaaaaatgataggggggaattggaggttttatcaaagtaagacagtatgatcaaatacttata
gaaatttgtggaaaaaaggctataggttcagtattagtaggacctacacctgtcaacataattggaagaaatatg
ttgactcaacttggatgcacactaaattttccaattagtcctattgaaactgtgccagtaaaattaaagccagga
atggatggcccaaaggtcaaacaatggccattgacagaagagaaaataaaagcattaatagaaatttgtgaagaa
atggaaaaggaaggaaaaattacaaaaattgggcctgaaaatccatataacactccagtatttgccataaaaaag
aagaacagtactaagtggagaaaattagtagattttagggaactcaataaaagaactcaagacttttgggaagtg
caattagggataccacacccaggagggttaaaaaagaacaaatcagtgacagtactagatgtaggggatgcatat
ttttcagttcctttagatgaaggtttcaggaaatatactgcattcaccatacctagtctaaacaatgagacacca
gggattagatatcaatataatgtgcttccacagggatggaaaggatcaccagcaatatttcagagtagcatgaca
aaaatcttagaaccctttagggcacaaaatccagaaatagtcatctaccaatacatggatgacttgtatgtagca
tctgatttagagatagggaaacatagagcaaaaatagaggaattaagagaacatctatggaggtggggattttac
acaccagataagaaacaccagaaagaaccaccatttctttggatggggtatgaactccatcctgacaagtggaca
gtacagcctatacagctgccagaaaaggatagctggactgtcaatgatatacagaagttagtgggaaaattaaac
tgggcaagtcaaatttatccagggattaaagtaaagcaactatgtaaactccttaggggaaccaaagcattaaca
gacatagtaccactgactgaagaagcagaattagaattggcagagaacagggaaattctaaaagaaccagtacat
ggagtatattatgacccatcaaaagacttaataactgaaatacagaaacagggaaatgatcaatggacatatcaa
atttaccaagaaccattcaaaaacctgaaaacagggaaatatgcaaaaatgaggactgcccacactaatgatgta
aagcagttaacagaggtagtgcaaaaagtagccatggaaagcatagtaatatggggaaaaactcctaaatttaga
ttacctatccagaaagaaacatgggaggcatggtggacagattattggcaagccacctggattcctgagtgggag
tttgtcaatacccctcccctagtaaagttatggtaccagctggagaaagaacctatagcaggagtagaaactttc
tatgtagatggagcagctaatagggaaactaaaatgggaaaagcaggatatgttactgacaggggaaggcagaaa
attgtttctctaactgaaacaacaaatcagaaaactgaattacaagcaattcagctagctttacaggactcagga
tcagaagtgaacatagtaacagactcacagtatgcattaggaattattcaagcacagccagataagagtgaatca
gagttagtcaatcaaataatagaacagttgataaaaaaggaaagggtctacctatcatgggtaccagcacataaa
gggattggaggaaatgagcaaatagataaactagtaagtactggaatcaggaaagtgctgtttctggatggaata
gataaggctcaagaagagcatgaaagatatcacaacaattggagagcaatggctgatgagtttaatctgccaccc
atagtagcaaaagaaatagtagccagctgtgataaatgtcagctaaaaggggaagccatgcatggacaagtagac
tgtagtccaggaatatggcaattagattgtacacatctagaaggaaaaatcatcctggtagcagtccatgtagcc
agtggctacatagaagcagaggttattccagcagaaacaggacaagagacagcatactacatattaaaattagca
ggaagatggccagtcaaagtaatacatacagacaatggcagtaatttcaccagtagtgcagttaaggcagcctgt
tggtgggcaggtatccaacaagaatttggaattccctacaatccccaaagtcagggagtagtagaatccatgaat
aaagaattaaagaaaatcatagggcaggtaagagatcaagctgagcaccttaagacagcagtacaaatggcagta
ttcattcacaattttaaaagaaaaggggggattggggggtacagtgcaggggaaagaataatagacatgatagca
acagatatacaaactaaagaactacaaaaacaaatcacaaaaattcaaaattttcgggtttattacagagacagc
agagaccctatttggaaaggaccagccaaactactctggaaaggtgaaggggcagtagtaatacaagataacagt
gacataaaggtagtaccaagaaggaaagtaaaaatcattaaggac
>#84 KP109483 2745 nt
gtagggggtcagataaaagaggctctcttagacacgggagcagatgatacagtgttaggagaaataaatttgcca
ggaaaatggaaaccaaaaatgataggaggaattggaggttttatcaaagtaagacaatatgatcaaatacctata
gaaatttgtggaaaaaaggctataggctcagtattagtaggacccacacctgtcaacataattggaagaaatatg
ttgactcagcttgggtgcacactaaattttccaatcagtcccattgaaactataccagtaaaattaaagccagga
atggatggcccaaaggttaaacaatggccattgacagaagagaaaataaaggcattaacagcaatttgtgatgaa
atggaaaaggaaggaaaaattacaaaaattgggcctgaaaatccatataacactccaatatttgccataaaaaag
aaggacagtactaagtggagaaaattagtagacttcagggaactcaataaaagaactcaagatttttgggaagtt
caattaggaataccacacccagcagggttaaaaaaaaaaaaatcagtgacagtactggatgtggggatgcatat
ttttcagttcctttatatgaagacttcaggaaatatactgcattcaccatacctagtataaacaatgaaacacca
gggattaggtatcaatataatgtgcttccacagggatggaaaggatcaccagcaatattccagagtagcatgaca
agaatattagagccctttagggcacaaaatccagaaatagtcatctatcaatatatggatgacttatatgtagga
tctgacttagagatagggcaacatagagcaaaaatagaggagttaagagaacatctgttaaagtggggatttacc
acaccagacaagaaacatcagaaagaacctccatttctttggatggggtatgaactccatcctgacaaatggaca
gtacagcctatacagctgccagaaaaggatagctggactgtcaatgatatacagaagttggtgggaaaattaaac
tgggcaagtcagatttacccaggaattaaagtaaggcaactttgtagactccttagggggctaaagcactaaca
gacatagtaccactaactggagaagcagaattagagttggcagaaaacagggaaattctaaaggaaccagtacat
ggagtatattatgacccatcaaaagacttgatagctgaaatacagaaacaggggcaggaccaatggacatatcaa

atttaccaagaaccattcaaaaatctgaaaacagggaagtatgcaaaaatgaggactgcccacactaatgatgta
aaacagttaacagaggctgtgcagaaaatagccatggaaagcatagtaatatgggaaaagactcctaaatttaga
ttacccatccaaaaagaaacatgggagctatggtggacagactattggcaagccacctggattcctgagtgggaa
tttgttaatacccctcccctagtaaaattatggtatcggctggaaacagaacctatagcaggagcagaaactttc
tatgtagatggagcagctaatagggaaactaaaacaggaaaagcagggtatgttactgacagaggaagaaataaa
gttgtttctctaactgaaacaacaaatcagaagactgaattgcaagcaatttgtctagctttgcaagattcagga
tcagaagtaaacatagtaacagattcacagtatgcattagggatcattcaagcacaaccagataagagtgaatca
gagttagttaaccaaataatagaacagttaataaagaaggaaagggtctacctgtcatgggtaccagcacataaa
ggaattggaggaaatgaacaagtagataaattagtaagtaatggaatcaggaaagtgctatttctagatggaata
gataaagctcaagaagagcatgaaaaatatcacagcaattggagagcaatggctagtgactttaatctrccaccc
atagtagcaaaagaaatagtagccagctgtgatcaatgtcagctaaaaggggaagccatgcatggacaagtagac
tgtagtccagggatatggcaattagattgtacacatttagaaggaaaaatcatcctggtagcagtccatgtagcc
agtggctacttagaagcagaggttattccagcagaaacaggacaagaaacagcatactatatactaaaattagca
gcaagatggccagtcaaagcaatacatacagacaatggtagtaatttcaccagygctgcagttaaggcagcctgt
tggtgggcaggcatccaacaggaatttggaattccctacaatccccaaagtcagggagtagtagaatccatgaat
aaagaattaaagaaaattatagggcagataagagatcaagctgagcaccttaagacagcagtacaaatggcagta
ttcattcacaattttaaaagaaaaggggggattggggggtacagtgcaggggaaagaatagtagacatgatagca
acagacatacaaactaaagaattacaaaaacaaattataaaaattcaaaattttcgggtttattacagagacagc
agagaccccatttggaaaggaccagccaaactactctggaaaggtgaaggggcagtagtgatacaagataataat
gacataaaggtagtaccaaggaggaaagcaaaaataattaaagac
>#85 KY658708 2745 nt
gtagggggccagataaaggaggctctcttagacacaggagcagatgatacagtattagaagaaataaatttgcca
ggaaaatggaaaccaaaaatgataggaggaattggaggttttatcaaagtaagacagtatgatcaaatagttata
gaaatttgtggaaaaaaggctataggtacagtattagtaggacctacacctgtcaacataattggaagaaatatg
ttgactcagcttggatgtacactaaattttccaattagtcccattgaaactgtaccagtaaaattaaagccagga
atggatggcccaaaggttaaacaatggccattgacagaagagaaaataaaagcattaacagaaatttgtgaagaa
atggagaaggaaggaaaaattacaaaaattgggcctgaaaatccatataacactccagtatttgccataaaaaag
aaggacagtactaagtggagaaaattagtagatttagggagcttaataaaagaactcaagacttttgggaagtt
caattaggaataccacacccagcagggttaaaaaagaagaaatcagtgacagtactggatgtgggggatgcatat
ttttcagttcctttagatgaaggcttcaggaaatatactgcattcaccatacctagtataaacaatgaaacacca
gggattagatatcaatataatgtgcttccacagggatggaaaggatcaccagcaatattccagagtagcatgaca
agaattttagagccctttagagcacaaaatccagaaatagtcatctatcaatatatggatgacttgtatgtagga
tctgacttagaaatagggcaacatagagcaaaaatagaagaattaagagaacatctgttaaagtggggatttacc
acaccagacaaaaaacatcagaaagaaccccctttctttggatggggtatgaactccatcctgacaaatggaca
gtacagcctataaagctgccagacaaggatagctggactgtcaatgatatacagaagttagtgggaaaattaaac
tgggcaagtcagatttacccagggatcaaagtaaggcaactttgtaaactccttaggggggccaaagcactaaca
gatgtagtaccactaactgaagaagcagaattagaattggcagagaacagggaaattctaaaagaaccagtacat
ggggtatattatgacccatcaaaagacttaatagctgaaatacagaaacaggggcatgaccaatggacatatcaa
atttaccaagaaccattcaaaaatctgaaaacaggaaagtatgcaaaaatgaggactgcccacactaatgatgta
aaacagttaacagaggcagtgcaaaaaatagcccaggaaagcatagtaatatggggaaagactcctaaatttaga
ctacccatccaaaaggaaacgtgggagacatggtggacagactattggcaagccacctggattcctgaatgggag
tttgttaatactcctcccctagtaaaattatggtaccagttggagaaagaacccataccaggagtagaaactttc
tatgtagatggagcagctaatagggaaactaaactaggaaaagcagggtatgttactgacagaggaaggcagaaa
attgtttctctaactgaaacaacaaatcagaagactgaattgcaagcaattcagctagctttgcaagattcagga
tcagaagtaaacatagtaacagactcacagtatgcattaggaatcattcaagcacaaccagataagagtgaatca
gagttagtcaaccaaataatagaacaattaatacaaaaggaaagggtctacctgtcatgggtacctgcacataaa
ggaattggaggaaatgagcaagtagataaattagtaagtcagggaatcaggaaagtgctgtttctagatggaata
gataaggctcaagaagagcatgaaaaatatcacaacaattggagagcaatggctgatgagtttaatctgccaccc
atagtagcaaaggaaatagtagctagctgtgataaatgtcaactaaaaggggaagccatacatggacaagtagac
tgtagtccagggatatggcaattagattgtacacatttagaaggaaaagttatcctggtagcagtccatgtagcc
agtggctacatagaagcagaggttatcccagcagaaacaggacaagaaacagcatactacatactaaaattagca
ggaagatggccagtcagagtaatacatacagacaatggtactaatttcaccagtgctgcagttaaggcagcctgt
tggtgggcaggtatccaacaggaatttggaattccctacaatccccaaagtcagggagtagtagaatccatgaat
aaagaattaaagaaaattatagggcaggtaagagatcaagctgagcaccttaagacagcagtacaaatggcagta
ttcattcacaattttaaaagaaaaggggggattggggggtacagtgcaggggaaagaataatagacataatagca

acagacatacaaactaaagaactacaaaaacaaattataaaaattcaaaattttcgggtttattacagagacagc
agagaccctatttggaaaggaccagccaaactactctggaaaggtgaaggggcggtagtaatacaagataacagt
gacataaaggtagtaccaaggaggaaagtaaaaatcattaaggac
>#86 KC156114 2745 nt
gtaggggggacagataaaggaggctctcttagacacaggagcagatgatacagtattagaagacataaatttgcca
gggaaatggaaaccaagaatgataggaggaattggaggttttatcaaagtaagacaatatgaacaaatacctata
gaaatttgtgggaaaaaggctataggtacagtgttagtaggacctacgcctgttaacataattggaagaaatatg
ttgacacagcttggatgtacactaaattttccaattagtcctattgaaactgtaccagtaaaattaaagccaggg
atggatggcccaagggttaaacaatggccattgacagaagagaaaataaaagcattaacagcaatttgtgaagaa
atggagaaggagggaaaaattacaaaaattgggcctgaaaatccatataacactccaatatttgccataaagaag
aaggacagtactaagtggagaaaattagtagatttcagggaactcaataaaagaactcaagattttgggaggtt
caattaggaataccacacccagcagggttaaaaaagaaaaaatcagtgacagtgctagatgtgggggatgcatat
ttttcagttcctttagatgaaagctttaggaaatatactgcattcaccatacctagtataaacaatgcaacacca
ggaattagatatcaatataatgtgctaccacagggatggaaaggatcaccagcaatattccagagtagcatgaca
aaaatcttagagcccttcaggacacaaaatccagatatagttatctatcaatatatggatgacttgtatgtggga
tctgacttagaaatagggcaacatagagcaaagatagagcaattaagagaacatttactgcaatggggacttacc
acaccagataaaaaacatcaaaaggaaccccccatttctttggatggggtatgaactccatcctgacaaatggaca
gtacagcctatacagctaccagacaaggatagctggactgtcaatgatatacagaagttagtgggaaaattgaac
tgggcaagtcagatttacccagggattaaagtaaggcaactttgtaaactccttaggggaaccaaagcactaacg
gacatagtaccactaactgaagaagcagaattagaattggcagagaacagggaaattctaaaagaaccagtacat
ggggtatattatgacccatcaaaagacttgatagctgaaatacagaaacaagggcatgaccagtggacatatcaa
atttaccaagaaccattcaaaaatctgaagacaggaaagtatgcaaaaatgaggactgcccacactaatgatgta
aaacagctaacagaggcagtgcaaaaaatagcccaggaatgcatagtaatatggggaaagactcctaaatttagg
ttacccattcaaaaagacatatgggaaacatggtggacagactattggcaagccacctggattcctgaatgggaa
tttagtaatacccctcccctagtaaaattatggtatcagcttgagaaagatcccatagcaggagcagaaactttc
tatgtagatggagcagctaatagggaaactaaactggggaaagcagggtatgtaactgataaaggaaggcagaaa
atcattactctaactgaaacaacaaatcagaaggctgaattacaagcaattcagcttgccttgcaggattcagga
tcagaagtaaacatagtaacagactcacagtatgcattaggaatcattcaggcacaaccagataagagtgaatca
gagttagtcactcaaataatagagcagttaataggaaaggaaaggatctacctgtcatgggtaccagcacataaa
ggaattggaggaaatgaacaagtagataaattagtaagtagtggaatcaggaaagtactgtttctagatggaata
gacaaggcccaagaagagcatgaaaaatatcacagcaattggagagcaatggctagtgagtttaatctgccaccc
atagtagcaaaagaaatagtagctagctgtgataaatgtcaacaaaaggggaagccatgcatgggcaagtagac
tgtagtccagggatctggcaattagattgtacacatttagaaggaaaaatcatcctggtagcagtccatgtagcc
agtggctacatagaagcagaggttatcccagcagaaacaggacaagaaacagcatactacatactaaaattagca
ggaagatggccagtcaaggtaatacatacagacaatggcagtaatttcaccagtaatgcagttaaggcagcctgt
tggtgggcaggtatccaacaggaatttggaattccctacaatccccaaagtcagggagtagtagagtccatgaat
aaagaattaaagaaaatcataggacaggtaagagatcaagctgagcacctaaagacagcagtacaaatggcagta
ttcattcacaattttaaaagaaaaggggggattggggggtacagtgcaggggagagaataatagacatgatagca
acagacatacaaactaaagaattacaaaaacaaattataaaaattcaaaattttcgggtttattacagagacagc
agagaccctatttggaaaggaccagccaaactactctggaaaggtgaaggggcagtagtaatacaagataacagt
gacataaaggtagtaccaaggaggaaagcaaaaatcattaaggac
>#87 AF005496 2745 nt
atagagggacagttaagggaagctctattagatacaggagcagatgatacagtattagaagagataaatttgccg
ggaaaatggaaaccaaaaatgataggggggaattggaggttttatcaaagtaagacagtatgagcaagtagccata
gaaatctgtggaaaaaaggctataggtacagtattagtaggacctacacctgtcaatataattggaaggaatata
ttgactcaaattggttgcaccttaaattttccaattagtcctattgaaactgtaccagtaaaattaaagccagga
atggatggcccaaaggttaaacaatggccattgacagaagaaaaaataaaagcattaacggaaatttgtacagag
atggaaaaagaaggaaaaatctcaagaatagggcctgagaatccatacagcactccaatatttgccataaaaaag
aaggatagtactaaatggagaaaattagtggatttcagagaactcaataaaagaactcaagacttctgggaagtt
cagttaggaataccacacccagcagggttaaaaaagaaaaaatcagtatcagtactggatgtgggggatgcatat
ttttcagtccctttagataaagaattcagaaagtatactgcattcaccatacctagtataaacaatgagacacca
gggattagatatcagtataatgtgcttccacagggatggaaaggatcaccagcaatattccagagtagcatgaca
aaaatcttagcgccctttagagaacaaaatcctgaaatggttatttaccaatacatggatgatttgtatgtagga
tctgacttagaaatagggcaacatagagcaaaaatagaggagttaagagctcatttgttgaaatggggatttacc
acaccagacaaaaaacatcagaaagaaccccccatttctttggatgggatatgaactccatcctgacaaatggaca

gtacagactgtaaaactgccagaaaaagacagctggactgtcaatgatatacagaagttagtgggaaaactaaat
tgggcaagtcagatttatccaaatattaaagtaaagcaactatgtaaactccttaggggggccaaagcattaaca
gacataataccactgacaaaagaggcagaattggaattggcagaaaacagggagattctgagagaaccaatacat
ggagtatattatgatccatcaaaagacttaatagcagaaatacggaagcaagggcaaggccaatggacatatcaa
atttatcaggagccatttaaaaatctgaagacaggaaaatatgcaaaaatgagaactgcccacactaatgatata
aaacaattaacagaagcagtgcaaaagatatctacagaaagcatagtaatatggggaaaaattcctaaatttaga
ctacctatacaaaaagaaacatgggagacctggtggacagagtattggcaagccacatggattcctgaatgggag
tttgttaacacccctcatctagtaaaattatggtatcagttagaaacagagcccatagcaggagcagaaacttac
tatatagatggggcagctaatagggaaactaaattaggaaaagcaggatatgtcactgatagaggaaagcaaaaa
gttgtctccctaacggaaacaacaaatcagaagactgaattacaagcaatttatctagctttgcaagattcaggg
ttagaagtgaacatagtgacagattcacagtatgcactaggaatcattcaagcacaacccgataagagtgaatca
gagttagttaatcaaataatagaggaattaataaagaaggaaaaggtctacctgtcatgggtaccagcacacaaa
ggaattggaggaaatgaacaagtagataaattagttagttctggagtcagaaaagtgctatttctagatgggata
gataaagctcaagaagaacatgaaaggtatcataacaattggagagcagtggctagtgattttaatctaccacct
atagtagcaaaagaaatagtagctagctgtgataaatgtcagctaaaaggggaagccatgcatggacaagtagac
tgtagcccaggaatatggcaattagattgcacacatttggaaggacaagttattctggtagcagtccatgtagcc
agtggctatatagaagcagaagtcatcccagcagaaacaggaaaggaaacagcatacttcctgttgaaactagca
agcagatggccagtaaaagtaatacatacagacaatggcagcaatttcacgagtgctgcggttaaggcagcctgt
tggtgggcagatatccaacaggaatttgggattccctacaatccccaaagtcagggagtagtagaatctatgaat
aaagaattaaagaagatcatagggcaggtaagagaccaagcagaacaccttaagacagcagtacaaatggcagta
ttcattcacaattttaaaagaaaaggggggattggggggtacagtgcaggggaaagaataatagacataatagca
acagacatacaaactaaagaattacaaaaacaaatttcaaacattcaaaaatttcgggtttattacagggacagc
agagacccaatttggaaaggaccagcaaaactcctctggaaaggtgaaggggcagtagtaatacaagacaatagt
gaaataaaagtagtaccaagaagagaggcaaaaatcattagggat
>#88 FJ389367 2754 nt
acagtaagagtaggggacagctaatagaagccctattagacacaggagcagatgatacagtattagaagatata
aatttacaaggaaaatggaaaccaaaaatgataggggaattggtggttttattaaagtaaaacagtatgatcaa
atacttatggaaatttgtggaaaaaaggctatagggacagtgttagtaggacctacacctatcaacataattgga
agaaatatgttgactcagattggttgtactttaaattttccaattagtcctattgaaactgtaccagtaaaatta
aagccaggaatggatggcccaaaggttaaacaatggccattgacagaagagaaaataaaagcattaacagaaatt
tgtacagaaatggaaaaagaaggaaaaatttcaaaaattgggcctgaaaatccatacaacactccaatatttgcc
ataaagaaaaaagacagtaccaagtggagaaaattggtagatttcagagagctcaataaaagaactcaagacttc
tgggaggtccaattaggaatacctcatcccgcggggttaaaaaagaaaaaatcagtaacagtactagatgtgggg
gatgcatatttttcagtgcccttatatgaagactttagaaagtatactgcattcaccatacctagtgtaaataat
gagacaccagggattagatatcagtacaatgtgcttccacagggatggaaaggatcaccagcaatatttcagart
agcatgacaaaaatcttagagccctttagaataaaaaatccagaaatagaaatctaccaatatatggatgattta
tatgtaggatctgacttagaaatagggcagcatagarcaaaaatagaggagttaagagaacatctgttaaaatgg
gggtttaccacaccagataaaaaacatcagaaagaacctccattcctttggatgggatatgagctccatcctgac
aaatggacagtacaacctatacagctgccaaataaagaaagctggactgtcaatgatatacaaaagttagtggga
aaactaaattgggcaagtcagatttatccaggaatyaaagtaaaacaactatgtaaactccttaggggggccaaa
gcactaacagacatagtaccactgactgcagaagcagaattggaactggcagagaacagggaaattctgaaagaa
cctgtacatggagtctattatgacccatcaaaagacytaatagcagaagtacagaaacaagggttagaccaatgg
acatatcaaatctatcaggagccatacaaaaatctgaaaacaggaaaatatgcaaaaaggggtctactcacact
aatgatgtaaaacaattaacagaagtagtgcaaaaaatagccacagagagcatagtaatatggggaaagactcct
aaatttaaactacccatacgaaaagaaacatggaagtatggtggacagaatattggcaggccacctggattcct
gactgggagtttatcaatactcctcctctagtaaaattatggtatcagttagaaacagaacccatmgcaggagta
gaaacttattatgtagatggggcagctaatagggaaacaaagttaggaaaggcaggatatgtcactgacagagga
aaacaaaaaattattaccctaactggaacaacaaaccaaaagactgaattacaagcaattcagctagctttgcag
gactcaggatcagaagtaaacatagtaacagactcacagtatgcattaggaattattcaagcacaaccagatagg
agtgaatcagaattagtcaatcaaataatagagcagctaataaaaaaggaaagggtctacctgtcatgggtacca
gcacacaaaggattggaggaaatgaacaagtagataaattagtyagtaatggaatcaggaaagtgttattttta
gatggcatagataaagcccaagaagagcatgaaagatatcacagcaattggagagcaatggctagtgattttaat
ctgccacctatagtagcaaaagaaatagtggccagctgtgataaatgtcagctaaaaggggaagccatgcatggc
caagtagactgtagtccaggaatatggcaattagattgtactcatttagaaggaaaaattatcctggtagcagtc
catgtggccagtggctatatagaagcagaagttatcccagcagaaacaggacaggaaacagcattcttcatatta

aaattggcaggcaggtggccagtgaaaatgatacacacagacaatggcagcaatttcaccagtgctgcagtaaag
gcagcatgttggtgggcagatatcacacaagaatttggaattccctacaatccccaaagccaaggggtagtggag
tctatgaataaagaattaaagaaaatcattgggcaggtcagggatcaagctgaacaccttaagacagcagtacag
atggcagtattcattcacaattttaaaagaaaaggggggattggggggtacagtgcaggggaaagaataatagac
ataatagcatcagacatacaaactaaagaactacaaaaacaaattacaaaagttcaaaattttcgggtttattac
agggacagcagagacccaatttggaaaggaccagcaaaactactctggaaaggtgaaggggcagtagtaatacaa
gacaataacgaaataaaggtagtaccaaggagaaaagcaaagatcattagggat
>#89 FJ389365 2745 nt
gtgggggacagctaatagaagccctrttagacacaggrgcagatgatacagtattagaacaataaatttacca
ggaaaatggaagccaaaaatgataggggggaattggaggatttatcaaagtaaaacagtatgatgatatacatata
gaaattgarggraaaaaggctatagggacagtattagtaggacctacacctgtcaacataattggaagaaatatg
ttgactcagattggytgtactttaaattttccaattagtcctattgaaactgtaccagtaaaattaaagccagga
atggatggcccaagggttaaacaatggccattgacagaagagaaaataaaagcattaacagaaatttgtaatgaa
atggaaaaggaagggaaaatttcaaagattgggcctgaaaatccatacaacactccaatatttgccataargaaa
aargacagtactaaatggagaaagttggtagatttcagagagctcaataaaagaactcaagatttctgggaagtc
caaytaggtatacctcatccckcggggttaaaaaagaaraaatcagtaacagtactggatgtgggagatgcatat
ttttcagttcccytacatgaagactttagaaagtatactgcattcactatacccagtayaaataatgagacacca
gggaytagatatcagtayaatgtgctyccmcagggatggaaaggatcaccagcaatatttcagagtagtatgacc
argatcttagagccctttaggayaaraaatccagaactggtgatctaycaatacatggatgatttatatgtagga
tctgacttagaaatagggcagcatagagcaaaaatagaggagttaagarakcatctrytgarrtggggattyacc
acaccagataaraaacatcagaaagaacctccattcctttggatgggataygagctccatcctgacaaatggacg
gtacaacctatacagctgccagacaaggaaagctggactgtcaatgatatacaraaattagtgggaaaactaaat
tgggcaagycagatytatcaagggattaaagtaaggcagctatgtaaactccttaggggggccaaagcactaaca
gacatagtaccactgactgargaagcagaattagaattggcagagaacagggagattctaaaagaacctgtacat
ggagtctactatgacccatcaaaagatttaatagcagaagtacagaaacaagggctygaccaatggacatatcaa
atttatcaagagccatataaaaatctgaaaacaggaaagtatgcaaaaaggggtctgcccacactaatgatgta
agacaattaacagaggcagtgcaaaaaatagccacagaragcatagtaatatggggaaaagttcctaaatttaaa
ctacctataaggaaagagacatgggaattatggtggacagaatattggcaggccacctggattcctgattgggag
tttgtcaatacycctcctctagtaaaattrtggtaycagttagagacagarcccatakyaggagcagaaacttac
tatgtagatggggcagctaatagggaaacaaaattaggaaragcaggatatgttactgacaaaggaaagcaaaar
gttatyaccatacaggaaacaacaaaccaaaaggctgaattacatgcaattcagctagccttacargactcaggg
tcagaggtaaacatagtaacagaytcacaatatgcattaggaatcattcaagcacaaccagatagragtgaatca
gaattagtcaatcaaataatagarcagctrataaamaaggaaaggrtctacttarcatgggtaccagcacacaaa
gggattggrggaaatgaacaagtagataaattagtcagtagtggaatcagaaaagtactatttttagatggcata
gataaagcccaagaagaacatgaaagatatcacagcaattggaaagcaatggctagtgattttaatctgccaccg
gtagtagcaaaagagatagtggccagctgtgataaatgtcagctaaaaggagaagccatgcatggacaggtagac
tgtagtccaggaatatggcaattagattgtacacatttagaaggaaagattatcctggtagcagtccatgtagcc
agtggctatatagaagcagaggttatcccagcagaaacaggacaggaaacagcatactttatattaaaattagca
ggaaggtggccagtaaaagtaatacatacagacaatggcagcaattttaccagtaatgcagtaaaggcagcttgt
tggtgggcaagtatcacacaagaatttggaattccctacaatccccaaagccaaggagtagtggaatctatgaat
aaagaattaaagaaaattattgggcaggtcagagatcaagctgaacatcttaagacagcagtacagatggcagta
ttcattcacaattttaaaagaaaaggggggattggggggtacagtgcaggggaaaggataatagacataatagca
tcagatctacaaactaaagaactacaaaaacaaattacaaaaattcaaaattttcgggtttattacagggacagc
agagacccaatttggaaaggaccagcaaaactactctggaaaggtgaaggggcggtagtaatacaagacaataac
gaaataaaagtagtaccaagaagaaaagcaaagatcattagggat
>#90 MH705151 2745 nt
ataggaggacagctaaaagaagctctattagatacaggagcagatgatacagtattagaagacataaatttgtca
ggaaaatggaaaccaagaatgataggggggaattggaggttttatcaaagtaaggcagtatgatcagatacttata
gaaatttgtggaaaaaaggctataggtacagtattggtaggacctacacctgtcaacataattggaagaaatatg
ttgactcagattggttgtactttaaattttccaattagtcctattgaaactgtaccagtaaaattaaagccagga
atggatggtccaaaggttaaacaatggccattgacagaagagaaaataaaagcattaacagaaatttgtaaagaa
atggaaaaggaaggaaaaatttcaaaaattgggcctgaaaatccatacaatactccagtatttgctataaagaaa
aaggacagcactaaatggaggaaattagtagatttcagagagctcaataaaagaacccaggacttctgggaagtt
caattagggataccacatccagcgggtttaaaaaagaaaaaatcagtaacagtactagatgtgggggatgcatat
ttttcagttcctttacatgaagacttcagaaagtatactgcattcaccatacctagtataaacaatgagacacca

ggaatcaggtatcagtacaatgtgcttccacagggatggaaaggatcaccagcaatattccaaagtagcatgaca
aaaatcttagagccctttagattaaaaaatccagaattagttatctatcaatacatggatgacttgtatgtagga
tctgatttagaaataggacagcatagaacaaaaatagaggagttaagagatcatctattgagatgggggtttact
acaccagacaaaaagcatcagaaagaacctccatttctttggatgggatatgaactccatcctgacaaatggaca
gtccagcctatacagctgccaaacaaagacagctggactgtcaatgatatacagaaattagtgggaaaactaaat
tgggcaagtcaaatttatccagggattaaagtaagacaactgtgtaaactcctcaggggagccaaagcactaaca
gatatagtaacactgactgaggaagcagaattagaactggcagagaacagggagattctaaaagaacctgtgcat
ggagtatattatgacccatcaaaagacttagtagcagaaatacagaaacaagggcaagaccaatggacatatcaa
gtttatcaagagccattcaaaaatctgaaaacaggaaaatatgcaagaaaaaggtctgctcacactaatgatgta
aggcaattagcagaagtggtgcaaaaagtgtccacagaaagcatagtaatatggggaaaaacccctaaattcaga
ttacccatacaaagagaaacatgggaagcatggtggatggagtattggcaggctacctggattcctgaatgggag
tttgtcaatacccctcctctagtaaaattatggtaccagttagaaaaggaccccatattaggagcagagactttt
tatgtagatggggcagctaatagagagactaagctaggaaaagcagggtatgtcactgacagaggaagacaaaag
gttgtttccctaactgagacaacaaatcaaaagactgaattgcatgcaatctacctagccttgcaggattcaggg
tcagaagtaaatatagtaacagattcacagtatgcattaggaatcattcaggcacaaccagacaagagtgaatca
gagctagttaatcaaatgatagagaagctaatagaaaaggaaaaagtctacctgtcatgggtaccagcgcacaag
ggaattggaggaaatgaacaagtagataaactagttagttctggtatcaggaaggtactgtttttagatgggata
gataaagctcaagaagaacatgaaagatatcacagcaactggagagcaatggctagtgattttaatctgccacct
atagtagcaaaggaaatagtagccagctgtgataaatgtcaactaaaaggggaagccatgcatggacaagtagac
tgcagtccagggatatggcaattagattgcacacatctagaaggaaaaataatcatagtagcagtccatgtagcc
agtggctatatagaagcagaagttatcccagcagaaacaggacaggagacagcatactttctgctaaaattagca
ggaagatggccagtaaaagtaatacacacagacaatggcagcaatttcaccagcgctgcagttaaagcagcctgt
tggtgggcaaatatccaacaggaatttggaattccctacaatccccaaagtcaaggagtagtggaatctatgaat
aaagaattaaagaagatcatagagcaagtaagagagcaagctgaacaccttaagacagcagtacaaatggcagta
ttcattcacaattttaaaagaaaaggggggattggggggtacagtgcaggggaaagaataatagacataatagca
acagacatacaaactaaagaattacaaaaacagattacaaaaattcaaaattttcgggtttattacagggacagc
agagacccaatttggaaaggaccagcaaaactactctggaaaggtgaaggggcagtagtaatacaggacaatagt
gacataaaggtagtaccaagaagaaaagcaaagatcattagggat
>#91 KF716478 2745 nt
ataggggccagctaagagaagctctattagatacaggagcagatgatacagtattagaagacataaatttgcca
ggaaaatggaaaccaaaaatgataggggaattggaggttttatcaaagtaagacagtatgatcagatactcata
gaaatttgtggaaaaaaggctataggtacagtgttggtaggacctacacctgtcaacataattggaagaaatatg
ttgacccagattggctgtactttaaattttccaattagtcctattgaaactgtaccagtaacattaaagccagga
atggatggcccaagaattaaacaatggccactgacagaagaaaaaataaaagcattaacagaaatttgtacagaa
atggaaaaggaaggaaaaatttcaaaaattgggcctgaaaatccatacaatactccagtatttgctataaagaaa
aaagacagcactaaatggagaaaattagtagatttcagagaacttaataaaagaactcaagacttttgggaagtt
caattaggaataccgcatccagcgggcttaaaaaagaaaaaatcagtaacagtactagatgtgggggacgcatat
ttttcagttcccttagataaaaatttagaaagtatactgcattcaccatacctagtataaacaatgagacacca
ggaatcaggtatcagtacaatgtgcttccacagggatggaaaggatcaccggcaatattccagagtagcatgata
aaaatcttagagccctttagagcaaaaaatccagaaataattatctatcaatacatggatgacttgtatgcagga
tctgatttggagatagggcagcatagagcaaaagtagaagaattaagagatcatctattaagttggggatttacc
acaccagacaaaaagcatcagaaggaacctccatttctttggatgggatatgaactccatcctgacaaatggaca
gtacagcctatacagctgccagaaaaagaaagttggactgtcaatgatatacagaaattagtgggaaaactaaat
tgggcaagtcaaatttatgcagggattaaggtaaagcaactgtgtaagctcctcagaggagccaaagcactaaca
gatatagtaccactgactgaggaagcagaattagaattggcagagaatagggaaattctaaaagaccctgtgcat
ggagcatattatgacccctcaaaagaattaatagcagaaatccaaaaacaagggcaggatcaatggacatatcaa
atttatcaagagccatttaaaaacctaaaaacaggaaaatatgcaagaaaaaggtctgctcatactaatgatgta
aaacaattaacagaagtggtgcaaaaagtggtccaggaaagtatagtcatatggggaaagacccctagatttaaa
ctacccatacaaaaagagacatgggaaacatggtggatggactattggcaggctacctggattcctgaatgggag
tttgtcaatacccctccccctagtaaaattatggtaccagttagagaaagaccccataataggagcagagactttc
tatgtagatggggcagccaatagggagactaagctaggaaaagcagggtatatcactgataaaggaagacaaaag
gttgtttccctaactgagacaacaaatcaaaagactgaactacatgcaatcaatctagccttgcaggattcagga
tcagaagtaaatatagtaacagactcacaatatgcattaggaatcattcaggcacaaccagacagaagtgagtca
gagttagtcaatcaaataatagaaaagctaataggaaaggacaaagtctatctgtcatgggtaccagcacacaag
ggattggaggaaatgaacaagtagataaattagtcagctctggaatcaggaaggtactatttttagatgggata

gataaggctcaagaagaacatgaaagatatcacagcaattggagagcaatggctagtgattttaatctgccaccc
atagtagcaaaggaaatagtagccagctgtgataaatgtcagctaaaagggaagccatgcatggacaagtagac
tgcagtccagggatgtggcaattagattgcacacatctagaaggaaaagtaattctggtagcagtccatgtagcc
agtggctatatagaagcagaagttatcccagcagaaacaggacaggagacagcatactttctgctaaagctagca
ggaagatggccagtaaaagtagtacacacagacaatggtagcaacttcaccagcgctgcatttaaagcagcctgt
tggtgggcaaatgtccaacaggaatatgggattccctacaatccccaaagtcaaggagtagtagaatctatgaat
aaggaattaaagaaaatcataggacaggtaagagatcaagctgaacaccttaagacagcagtacaaatggcagta
ttcattcacaattttaaaagaaaaggggggattggggactacagtgcaggggaaagaataatagacataatagca
acagacatacaaactagagaactacaaaaacaaattacaaaaattcaaaattttcgggtttattacagggacagc
agagagccactttggaaaggaccagcaaaactactctggaaaggagaaggggcagtggtaatacaggacaataat
gatataaaggtagtgccaaggagaaaagtaaagatcattaaggat
>#92 AJ320484 2745 nt
ataggggacagctaaaggaagctctattagatacaggagcagatgatacagtgttagaagacataaatttgcca
ggaaaatggaaaccaaaaatgatagggggaattggaggttttatcaaagtaagacagtatgatcaaatacccata
gaaatctgcggatataaagctataggtacagtattaataggacctacacctgtcaacataattggaagaaatttg
ttgactcagattggctgcactttaaattttccaattagtcctattgaaactgtaccagtaaaattaaagccaggg
atggatggcccaaaagttaaacaatggccattgacagaagaaaaaataaaagcactaacagaaatttgtgcagac
atggaaaaagaaggaaaaatttcaagaattgggcctgaaaatccatacaatactccagtatttgccataaagaaa
aaagacagtactaagtggagaaaattagtagatttcagagaacttaataagagaactcaagatttctgggaagtt
caactaggaataccacatcctgcagggctaaagaagaaaaaatcagtaacagtactggatgtgggtgatgcatat
ttttcagttcccttagatatagactttagaaagtatacagcattcaccatacctagtacaaacaatgaggcacca
ggaattagatatcagtacaatgtgcttccacagggatggaaaggatcaccagcaatattccaatgtagcatgaca
aaaatcttagaaccctttagaaaacaaaatccagaaatagttatctatcaatacatggatgatttgtatgtagga
tctgacttagaaatagggcagcatagaacaaaaatagatgaattaagggaacatctattgaggtggggacttatc
acaccagacaaaaaacatcagaaagaacctccatttctttggatgggttatgaactccatcctgataaatggaca
gtgcagcctataaagctgccagaaaaagaaagctggactgtcaatgatatacagaagttagtgggaaaattaaat
tgggcaagccagatttatccaggaattagagtaaaacacttatgtaaactccttaggggagccaaagcactgaca
gaagtaataccactgacaaaagaagcagaattagaactggcagaaaacaaggaaattctaaaagaaccagtacat
ggagtgtattatgacccatcaaaagacttaatagcagaagtacagaaacaagggcaagaccaatggacatatcaa
atttatcaagagccatttaaaaatctgaaaacaggaaagtatgcaaaaatgaggagtgcccacactaatgatgta
aaacaattaacagaggcagtgcaaaaaatagcccaagaatgcatagtaatatggggaaagactcctaaatttaga
ctacccatacaaaaggaaacatgggaagcatggtggaaagaatattggcaggccacctggattcctgagtgggag
tttgtcaatacccccccccttagttaaattatggtaccagttagagaaggaacccatactaggagcagaaactttc
tatgtagatggggcagctaatagagagaccaaattaggaaaagcaggatatgttactaacaaaggaagacagaaa
gttgtctctctaactgacacaacaaatcagaagactgagttacaagccattaatctagctttgcaggattcggga
ttagaagtaaacatagtaacagattcacaatatgcattaggaatcattcaggcacaaccagataaaagtgaatca
gagttagtcagtcaaataatagagcagttaataaaaaaggaaaaggtctacctagcatgggtaccagcacacaag
gggattggaggaaatgagcaagtagataaattggtcagtaatggaatcaggaaagtattattttggatggaata
gataaggctcaagaagaacatgagaaataccacaacaattggagagcaatggctagtgattttaacctgccacct
gtggtagcgaaagaaatagtagctagctgtgataaatgtcagctaaaaggagaagccatgcatggacaagtagac
tgtagtccaggaatatggcaattagattgtacacatctagaaggaaaagttatcctggtagcagttcatgtagcc
agtggctatatagaagcagaagtgatcccagcagaaacagggcaggaaacagcctactttatcttaaaattagca
ggaagatggccagtaaaagtagtacatacagacaatggcagcaatttcaccagttctgcagttaaggctgcctgt
tggtgggcaggcattaagcaggaatttggaattccccacaatccccaaagtcaaggagtagtggaatctatgaat
aaagaattaaagaaaattataggacaggtaagagatcaagctgaacatcttaagacagcagtacaaatggcagta
ttcattcacaattttaaaagaaaaggggggattggggagtacagtgcaggggaaagaataatagacataatagca
acagacatacaaactacaaaattacaaaaacaaatcataaaaattcaaaattttcgggtttattacagggacagc
agagatccaatttggaaaggaccagcaaagcttctctggaaaggtgaaggggcagtagtactacaagataatagt
gacataaaggtagtaccaagaagaaaagtaaagatcattagggat
>#93 KT200357 2745 nt
gtagggggcaactaaaggaagctctattagatacaggagcagatgatacagtactagaagaaataaatttgcca
gggaaatggaaaccaaaaatgatagggggaattggaggttttatcaaagtgagacagtatgatcaggtacccata
gaaattagtgggcataaagccataggtacagtattagtaggacctacacctgtcaacataattggaagaaatctg
ttgacacagattggatgcactttaaattttcccattagtcctattgaaactgtaccagtaaaattaaagccaggg
atggatggcccaaaagttaaacaatggccattgacagaagaaaaaattaaatgcattaatagaaatttgtgcagaa

atggaaaaggaaggaaaaatttcaaaaattgggcctgaaaatccatacaatactccaatatttgccataaagaaa
aaagacagtactaaatggagaaaattggtagatttcagagaacttaataagagaactcaagacttctgggaaatt
caattaggaataccacatccttcagggttaaaacagaaaaaatcagtaacagtattggatgtgggtgatgcatat
ttttcagttcccttagataaagactttaggaagtatactgcatttaccatacctagtgtgaacaatgagaaacca
gcaattagatatcagtacaatgtgcttccacagggatggaaaggatcaccagcaatattccaaagtagcatgaca
aaaatcttagagccttttagaaaacaaaatccagacatggttatctatcaatacatggatgatttgtatgtcgga
tctgacctagaaatagagcaacatagaacaaaaatagaggaactgagacaacatctgttgaggtggggtttaca
acaccagataaaaaacatcagaaagaacctccattcctttggatgggttatgaactccatcctgataaatggaca
gtacagcctataaagctgccagaaaaggacagctggactgtcaatgacatacaaaaattagtgggaaaattaaac
tgggcaagtcagatttatccagggattaaagtaaggcaattatgtaaactccttaggggaaccaaagcactaaca
gaagtagtatcgctaacaagagaagcagaactagaattggcagaaaacagggagattctcaaagaaccagtacat
ggagtgtactatgacccatcaaaagacttaatagcagaaatacagaagcaggggcaaggccaatggacatatcaa
atttatcaagagccatttaaaaatctgaaaacaggaaagtatgcaagaacgaggggtgcccacactaatgatgta
aaacaattaacagaggcagtgcaaaaaatagccacagaaggcatagtgatatggggaaagattcctaaattcaaa
ttacccatacaaaaagaaacatgggaaacatggtggacagagtattggcaagccacctggattcctgagtgggag
tttgtcaatacccctcccttggtgaaattatggtatcagttagagaaagaacctatagtaggagcagaaactttc
tatgtagatggagcagctaatagggaaactaaaataggaaaagcaggatatgttacagacagaggaagacaaaaa
gttgtccctctaacggacacaacaaatcagaagactgagttacaggcaattcatttagctttgcaggattcagga
ttagaagtaaatatagtaacagactcccaatatgcattaggaatcatccaagcacaaccagatcagagtgaatca
gagatagtcagtcaaataatagaacagttaataaaaaaggaaaaggtctacctggcatgggtaccagcacacaaa
ggaattggaggaaatgagcaagtagataaattggtcagtagtggaatcaggaaagtgctatttttagatggaata
gataaggcccaggaagagcatgagaaatatcacaataattggagagcaatggctagtgattttgacctgcccccct
gtagtagcaaaagaaatagtagccagctgtgataaatgtcagctaaaaggagaagccatgcatggacaagtagac
tgtagtccaggaatatggcagctagattgtacacacttagaaggaaaagttatcctagtagcagttcatgtagcc
agtggttatatagaagcagaagttattccagcagagacagggcaagaaacagcatactttattttaaaattagca
ggaagatggccagtaaaaacaatacatacagacaatggccccaatttcatcagtaatacagttaaggccgcctgt
tggtgggcggggatcaagcaagaatttggcattccctacaatccccaaagtcaaggagtagtagaatctatgaat
aaagaattaaagaaaattatagggcaggtaagagatcaggctgaacatcttaagacagcagtgcaaatggcagta
ttcatccacaattttaaaagaaaaggggggattggggggtacagtgcaggggaaagaataatagacatgatagca
tcagacatacaaactaaagcattacaaaaacaaattacaaaaattcaaaattttcgggtttattacagggacagc
agagatccactttggaaaggaccagcaaaacttctttggaaaggtgaaggggcagtagtaatccaagataatagt
gaaataaaagtagtgccaagaagaaaagtaaaaatcattagggat
>#94 AY331292 2745 nt
ataggggggcaactaaaggaagctctattagatacaggagcagatgatacagtattagaagaaatgaatttacca
ggaagatggaaaccaaaaatgataggggggaattggaggttttatcaaagtaaaacagtatgatcagatacttata
gaaatctgtggacataaggctataggtacagtattagtaggacctacacctgtcaacataattggaagaaacctg
ttgactcagattggttgcaccttaaattttcccattagtcctattgaaactgtaccagtaaaattaaagccagga
atggatggcccaaaagttaaacaatggccattgacagaagagaaaataaaagcattagtagaaatttgtacagaa
atggaaaaggaagggaaaatttcaaaaattgggcctgaaaatccatacaatactccagtatttgccataaagaaa
aaagacagtactaaatggagaaaattagtagatttcagagagcttaataagagaacacaagacttttgggaagtt
cagttaggaataccacatcccgcaggattaaaaaagaaaaaatcagtaacagtattggatgtgggtgatgcatat
ttttcagttcccttagataaagaatttaggaagtacactgcatttaccatacctagtataaacaatgaaacacca
gggattagatatcagtataatgtgcttccacagggatggaaaggatcaccagcaatattccaaagtagcatgaca
aaaatcttagagccttttagaaaacaaaatccagacatagttatctatcaatacatggatgatttgtatgtagga
tctgacttagaaatagggcagcatagaacaagaatagaggaactgagacaacatttgctgaagtggggatttacc
acaccagacaaaaaacatcagaagaaacctccattcctttggatgggttacgaactccaccctgacaaatggaca
gtacagcctatagtgctgccagaaaaagacagctatactgtcaatgatatacagaagttagtgggaaaattaaat
tgggcaagtcagatttacgcagggattaaagtaagacaattgtgtaarctccttaggggagccaaagcactaaca
gaagtaataccactaacaaaagaggcagagctagaactggcagaaaacagggagattctaaaagaaccagtacat
ggagtgtattatgacccatcaaaagacttaatagcagaaatacagaaacaggggcaaggtcaatggacatatcaa
atttatcaggagccatttaagaatctgaaaacaggaaagtatgcaagaatgaggggtgcccacactaatgatgta
aaacaattaacagaggcagtgcaaaaaataaccacagaaagcatagtaatatggggaaagattcctaaatttaga
ctccccatacaaaaagaaacatgggaaatatggtggacagaatattggcaagccacctggattcctgagtgggag
tttgtcaatacccctcccctagtgaaattatggtaccaattagagaaagaacccataataggagcagaaactttc
tatgtagatggggcatctaacagggaaactaaattaggaaaagcaggatatgtcactaacagaggaagacaaaaa

gttgtctccctaactgacacaacaaatcagaaaactgagttacaagcaatttatctagctttgcaggattcggga
tcagaagtaaacatagtaacagactcacaatatgcattaggaatcattcaagcacaaccagataagagtgaatca
gagctagtcaatcaaataatagagcagttaataaataaggaaaaggtctacctggcatgggtaccagcacacaaa
ggaattggaggaaatgaacaagtagataaattagtcagtgctggaatcaggaaagtactatttttagatggaata
gataaggcccaagaagaacatgagaaatatcacagtaattggagagcaatggctagtgattttaacctgccacct
gtagtagcaaaagaaatagtggccagctgtgataaatgtcagctaaaaggagaagccatgcatggacaagtagac
tgtagtccaggaatatggcaactagattgtacacatttagaaggaaaagttatcctggtagcagttcatgtagcc
agtggatacatagaagcagaagttattccagcagagacagggcaggaaacagcatatttcttttaaagttagca
ggaagatggccagtaacaacaatacatacagacaatggcagcaatttcatcagcactacagttaaggccgcctgt
tggtgggcggggataaagcaggaatttggcattccctacaaccctcaaagtcaaggagtagtagaatctatgaat
aaagagttaaagaaaattataggacaggtaagagatcaggctgaacatcttaagacagcagtgcaaatggcagta
ttcatccacaattttaaaagaaaaggggggattggggggatacagtgcaggggaaagaatagtagacataatagca
acagacatacagactaaagaactacaaaaacaaattacaaaaattcaaaattttcgggtttattacagggacagc
agagatccactttggaaaggaccagcaaagctcctctggaaaggtgaaggggcagtagtaatacaagataatagt
gacataaaagtagtgccaagaagaaaagcaaaaatcatcagggat
>#95 KP109514 2745 nt
atagggggcaactaaaggaagctctcttagatacaggggcagatgatacagtattagaggaaatgaatttacca
ggaagatggaaaccaaaaatgataggggggaattggaggttttatcaaagtaagacagtatgatcagatatccata
gaaatctgtgggcataaagctataggtacagtgttagtaggacctacacctgtcaacataattggaagaaacttg
ttgactcagattgggtgcactttaaattttcccattagtcctattgaaactgtaccagtaaaattaaaaccagga
atggatggcccaaaagttaaacaatggccattgacagaagaaaaaataaaagcactaacagaaatttgtgcagag
atggaaaaggaaggaaaaatttcaaaaattgggcctgaaaatccatataatactccaatatttgccataaagaaa
aaagacagtactaaatggagaaaattagtagatttcagagaactcaataagagaactcaagacttctgggaagtg
cagttaggaataccacatcctgcagggttaaaaaagaaaaaatcagtaacagtactggatgtgggtgatgcatat
ttttcagttcccttagataaagaattcagaaagtatactgcatttaccatacctagtataaacaatgagacacca
ggggttagatatcagtacaatgtgctcccacagggatggaaaggatcaccagcaatattccaaagtagtatgaca
aaaattttagatccttttagaaaacaaaatccagacctagttatctatcaatatatggatgatttgtatgtagga
tctgacttagaaataggacagcatagaacaaaaatagaggaactaagacaacatctgttgaggtggggacttacc
accccagacaaaaaacatcagaaagaacctccatttctttggatgggttatgaactccatcctgataaatggaca
gtacagcctatagtgctgccagaaaaagacagttggactgtcaatgacatacagaagttagtgggaaaattaaat
tgggcaagtcagatctactcagggattaaagtaaaacagttatgtaaactccttaggggaactaaatcactaaca
gaaataataccactaacagaagaagcagagttagaactggcagaaaacagggagattctaaaaacaccagtacat
ggagcgtattatgacccatcaaaagacttaatagcagaaatacagaagcaaggggaaggccaatggacatatcaa
atttatcaggaaccttttaaaaatctgaaaacaggaaaatatgcaagaatgaggggtgcccacactaatgatgta
aaactattaacagaggcagtgcaaaagatagccacagaaagcatagtaatatggggaaagactcctaaatttaga
ctacccatacaaaaagaaacatgggaaacatggtggacagagtattggcaagccacctggattcctgagtgggag
tttgtcaatacccctcccttagtgaaattatggtaccagttagagaaagaacccatagtaggagcagaaactttc
tatgtagatggggcagctaacagggaaactaaattaggaaaagcaggatatgtaactgacaaaggaagacaaaag
gttgtctccctaaatgacacaacaaatcagaagactgagttacaggcaattcatctagctttgcaggattcagga
ttagaagtaaacatagtaacagattcccaatatgcattgggaatcattcaagcacaaccagataaaagtgaatca
gagttagtcaatcaaataatagagcagttaataaaaaaggaaaaggtctacctagcatgggtgccagcacacaaa
ggaattggaggaaatgaacaagtagataaattagtcagtgctggaattaggaaaatactatttttagatggaata
gataaggcccaagaagaacatgagaaatatcacaataattggagagcaatggctagtgattttaacataccacct
gtagtagccaaagaaatagtagccagctgtgataaatgtcagctaaaaggagaagccatgcatggacaagtagac
tgtagtccagaaatatggcaactagattgtacacacttagaaggaaaagttatcctggtagcagttcatgtagcc
agtggatatatagaagcagaagttatcccagcagagacagggcaagaaacagcatacttcatcttaaaattagca
ggaagatggccagtaaaaacaatacatacagataatggcagcaatttcaccagtgctgcggttaaggccgcctgt
tggtgggcggggatcaaacaggaatttggcattccctacaatccccaaagtcaaggggtagtagaatctctaaac
aaagaattaaagaaaattgtaggacaagtaagagatcaggctgaacatcttaagacagcagtacaaatggcagta
ttcatccacaattttaagagaaaaggggggattggggggatacagtgcaggggaaagaatagtagacataatagca
acagacatacagactaaagaattacaaaaacaaattacaaaaattcaaaattttcgggtttattacagggacagc
agagatccactttggaaaggaccagcaaagcttctctggaaaggtgaaggggcagtagtaatacaagataatagt
gacataaaagtagtgccaagaaggaaagcgaaaatcattagggat
>#96 AF146728 2745 nt

atagggggcaactaaaggaagctctattagatacaggagcagatgatacagtattagaagaaatgtgtttacca
ggaagatggaaaccaaaaatgatagggggaattggaggttttatcaaagtaagacagtatgatcaggtattagta
gaaatttgtggacataaagctataggtacagtattagtaggacctacacctgtcaacataattggaagaaatctg
ttgactcagcttggatgtactttaaattttcccattagtcctattgagactgtaccagtaaaattaaagccagga
atggatggcccaaaagttaaacaatggccattgacagaagaaaaaataaaagcattagtagaaatttgtacagaa
atggaaaaggaaggaaaaatttcaaaaattgggcctgaaaatccatacaatactccagtatttgccataaagaaa
aaagacagtactaaatggagaaaattagtagatttcagagaacttaataagagaactcaagacttctgggaagtt
caattaggaataccacatcccgcagggttaaaaaagaaaaaatcagtaacagtactggatgtgggcgatgcatat
ttttcagttcccttagataaagacttcagaaagtataccgcattcaccatacctagtataaacaatgagacacca
gggattagatatcagtacaatgtgcttccacagggatggaaaggatcaccagcgatattccaaagtagcatgaca
aaaatcttagagccttttagaaaacaaaatccagacgtagttatctatcaatacatggatgatttgtatgtagga
tctgacttagaaatagggcagcacagaacaaaaatagaggaactgagacaacatctgttgaggtggggtttacc
acaccagacaaaaaacatcagaaagaacctccattcctttggatgggttatgaactccatcctgataaatggaca
gtacagcctatagtgctgccagaaaaagacagctggactgtcaatgacatacagaaattagtgggaaaattaaat
tgggcaagtcagatttattcagggattaaagtaagacaattatgtaaacttcttaggggaaccaaggcactaaca
gaagtaataccactaacagaagaggcagagctagaattagcagaaaacagggagattctaaaagaaccagtacat
ggagtgtattatgacccatcaaaagatttaatagcagaggtacagaagcaggagcaaggccaatggacatatcaa
atttatcaagaaccatttaaaaatctgaaaacaggaaagtatgcaagaatgaagggtgcccacactaatgatgta
aaacagttaacagaggcagtgcaaaaaatagccacagaaagcatagtaatatggggaaagactcctaaatttaag
ctacccatacaaaaggagacatgggaagcatggtggatggagtattggcaggccacctggattcctgagtgggag
tttgtcaatacccctcccttagtgaaattatggtaccagttagagaaagaacccatagtaggagcagaaactttc
tatgtagatggagcagctaatagggagaccaaattaggaaaagcaggatatgttactgacagaggaagacaaaag
gttgtcccccctaagtgacacaacaaatcagaagactgagttacaagcaattcagctagcgttgcaggattcggga
ttagaagtaaacatagtaacagactcacaatatgcattaggaatcattcaagcacaaccagataagagtgaatca
gagttagtcaatcaaataatagagcagctaataaaaaaggaaaagatctatttggcatgggtaccagcacacaaa
ggaattggtggaaatgataaggtagatagtttggtcagtgctgggatcaggaaagtactattcttggatggaata
gataaggcccaagatgatcatgagatatatcacagtaattggagggcaatggctaatgattttacctgccacct
atagtagcaaaagatatagtagccagctgtgataagtgtcagcaaaaaggagaagccatgcatggccaagtagac
tgtagtccaggtttatggcaactagattgtacacatttagaaggaaaagttatcctggtagcagtccatgtagcc
agtgggtatatagaagcagaagtcattccagcagagacagggcaggaaacagcatattttctattaaaattagca
ggacgatggccagtaaaaacagtacatacagacaatggccccattttcatcagtactgcggttaaggccgcctgt
tggtgggcagggattaagcaggaatttggcattccctacaatccccaacgtcaaggagtggtagactctatgaat
aacgacttaacgacaattataggacaggtaagagatcaggctgaacatcttaagacagcagtacaaatggcagta
ttcatccacaattttaaaagaaaaggggggattggggggtacagtgcaggggaaagaataatagacataatagca
tcagacatacaaaccaaagaattacaaaaacaaattacaaaagttcaaaattttcgggtttattacagggacagc
agagatccactttggaaaggaccagcaaagctcctctggaaaggtgaaggggcagtagtaatacaagataatagt
gacataaaagtagtgccaagaagaaaagcaaagattattagggat

Supplementary File S5. Dataset of 96 nucleotide sequences of the gene *env* from primate lentiviruses: 37 sequences from SIV infecting Old-World monkeys (#1 - #37), 4 from HIV-2 (#38 - #41), 20 from SIV infecting African apes (7 from SIVgor, #42 - #48, 13 from SIVcpz, #49 - #61), and 35 from HIV-1 (9 from group O, #62 - #70, 2 from group P, #71 and #72, 7 from group N, #73 - #79, and 17 from the pandemic group M, #80 - #96). From each genome sequence we selected the non-overlapping region that encodes the Env protein. Each nucleotide sequence contains the NCBI ac. number and the length of the coding region.

```
>#1  AF468658  2100 nt
gggaactggacgacagtgttctatggggtaccggtatggagagatgcaaaaccaccattgttctgtgcctcagat
gcagacataactagtaatgaaccaggaaacatttggatctctacggcatgcttgccctcagacccctcaccgtct
gaggtgccactaaacataacagaggaatttaatatctataaaaattatatggtagatgaggtgagagatgacatg
gtatctctatttaatcaggccctaagaccatgtgtcaaactgactccgatgtgtgtacgaatgaaatgtaagctc
ccaaatacgagcacaacaacaacaccgtccacaacaacagccacaacatcaccctcatccccaacgccaacacca
tggggaaattggggaggtaatggaacaggacagccaatatataactgttcctttaaccagactacagaatttagg
gacaaaaagagacagatgtatagcctcttttggaaagatgatattatgagggcacaggatggtaatgacactgac
tattatattataaattgcaacacatcatatgtcacgcaaaagtgtgtcaagacctccttccaaccggttcccata
cactactgtgctccgcccggctttgctatgctaaaatgcaatgatgccaatttcacaggagtgggcaagtgttcc
aatgtatcagcagtaacatgcacgcatggcattcaacctttagtagcaacatggcttcatctaaatggacttat
cagccagggaacaataccagagtaatgatgaatggcaaaaagaatgaatctatagtcataggctttggggaagat
tatcagcttactcttacatgtatcagacctggaaataaaaccctaaaaaatttacagataggagcaggaatgaca
ttctactcccaaattatagtaggggggagatactaggaaggcctattgcaaactcaaccatacaaagtgggatata
gcaataaggcaagccatgaaggccatgaagaaccattgggaaaagatcaacaatgataccacccctaataagact
caaataaggtggacctccgaacctaaaggagatctagaggtccaaactcattggttccaatgtcagggagaattt
ttctattgtaatttgtcagtactctttcaattcaacaacaatattacggcagtggatgacacaaacattaacaat
gtcacaagcaaatacaaaggacaatggatggcttgcagaattaggcaatttgtcacacaatggggatatgtgtct
aggtcgatctacttacctccacggcaaggtcatataaattgtacatctaacatcactggcctcctaatagacgga
gcgatgtatggacattccatcaatatgaccccatcggccgatgtagccgatgcatggaagtatgagttatccagg
tacaaggtggtggagatagatccactttcgctggccccaacctcggctcaaaggagaccacatccaggggtgcat
gtgaaaaagagggccatttcattgggcatatccttcttgacgtttctcagtgcggcaggtgccacgatgggcgca
gcggcaacagcgctgacggtacaatcccggtcattgttggctgggatagtgcagcagcaggagaacctgctgagg
gctgtagaggcccaacagagcttattacagctatctgtatggggcataaaacagctacaagcccgcctgtcttca
ctagagaaatacctgaggatcaaacaattctccaagcttggggctgtgcgaatcagccaatctgtcacaccata
gtaccttggaatgactcatgggctaagaactcgacgccagattgggagcacatgacctggcaggagtggagtaag
ttaattgaaaatgacacatataccatacagcaattattagaaaatgcaaaccatcaacaatcaaagaacatgaat
gacttattaaagttatccaaatgggactccctttggagttggtttgatatttccaattggctctggtacattaag
attttcataatggtagttgcagctttagtagctttgagaataataatgtttgttcttaatatgcttagaagggtt
>#2  AF468659  2067 nt
aattggacgacagtgtactatggggtaccggtatggagggacgcaactccaccattgttttgcgcctcagatcca
gatatagctagtaatgaaccaggaaacatttggatttctacagcatgtttgccctcggacccctcaccggcagag
gtgccgttaaacataacagagaaattcaacatctataaaaattacatggtagatgaggtgcgagatgacatggta
tcactatttaatcaggccctgaaaccatgtgtcaaattgactccaatgtgtgtggggatgtattgtaacctcaca
aacacatccacaagcgaaccaacaacaactccaaggccaccaaatgtctccacaaccacgcagtggggaagttgg
ggggggaaaatggaacaggacaacccttatataactgttcctttaatcagaccacagaatttagagaccaaaag
aaacagatgtatagcctcttctggagagaagacattatggaagagacccatggtaaccagagtggatattatatt
agaaattgtaatacctcatatatcactcaaaaatgtgtcaagtcctccttccaaccggttcccatacactactgt
gctccacccggctatgctatgctaaaatgcaatgatgttaatttcacaggagtaggcacatgctacaatgtgtca
```

gcagtaacctgcacacatggcattcaacccttagtagcaacatggctccatttaaatgggacctatcagccagga
aataataccagaataatgatgaatggtatgaagaatgaatctatagttataggctttggagaagattatcatctt
aatcttacatgtatcagacctggaaataaaaccataagaaatttgcaataggagcgggaatgacattttattcc
caagtgatagtaggaggaaatactaggaaagcctattgccgacttgatcctgataggtggaatagggcaataaga
gaagctatgaaagcaatgaatgaacattgggaaaataaaactgggagaaatgatactcaaataagatggacctca
gagcctaaaggagatctagaggttcaaactcattggttccaatgtcaaggagaatttttctattgcaatttgtca
atactctttcaacttaataataacaccattaacagcagcaatattgggaacatcacaagcaaatacaaaggacaa
tggctggcttgcaaaattaggcaatttgtcacccaatggggatatgtgtctaaatcaatctacttacctccacgg
cagggtcatataaattgcacatccaacattacaggtctgttgatagatggcgctatgtatgaaagctctatcaac
atgaccccgtcggccgatgtggctgatgcgtggaactatgaattgtccaggtacaaagtggtggagatagatccg
ctctcgatggccccaactccggctaaaaggaaagaacaccccgctgtggagaaaagagccctttcattgggaata
tccttcttgacgtttctcagtgcggcaggtaccacgatgggcgcagcggcaacagcgctgacggaacagtcccgg
tctttgttggctgggataatgcagcagcaggagaacctgctgagggctgtagaggcccaacagagcttattacag
ccatctgtatggggcataaaacagctacaaacccgcctgtcatcactagagaaatatttgagggatcaaaccatt
ctccaagcatggggctgtgcgaacaggccaatctgtcacaccatagtaccttggaacacctcatgggctaatggc
tcgcttccagactgggagaacatgacctggcaaaagtggagtatgttagttgagaatgacacatatacaatacag
cagttgttagagcaagcaaaccagcaacaagcatcaaacctgaatgagctgatgaagttgtccaaatgggactcc
ctttggagttggtttgatatttccgattggcagaggtacattaagatctttgtaatagtagttgcagccttaata
gctttaagaatagtaatgtttatccttaatatgcttagaagg
>#3 EF070329 2157 nt
ctaatattgagaaaacatgccaagcaatgggtcacggtgttttatagggtaccggtatcaaagaatgccacacca
cctctcttctgtgcatcaaatgctaatatagcaaacagagagccagggaacgtgttgataacgacagcatgccta
cccacggaccccgcgccacaggaggtacaactaaacatctcaaatgagcactttaatatatataagaactatatg
gtagatcagatgactgatgatgtgaagtccatctttactcaggcccttaagccgtgtgtgaaactaacacctatg
tgtgtaaaaatgaagtgtgaccttctcaatattagtatgtctagtaccaccgcagcaagcgccacaacaagcggg
catacaaccgtcaccccttggggcagatgggcagacaataatgagacacaattgaccatgtacaattgttccttc
aatcagaccaccgagttcagagatgtaaaaaaacagatgtactctctcttctttgtaaaagacctcatgaaaggg
gagaatgagacatattacattacaaactgcaacacttcttatatcacacagcagtgtgagaagagcagcttccaa
ccggtcccaatacaatactgtgcccctgcaggatatagtctcttaaaatgcaatgatgctaatttcacaggacaa
ggcatttgtaataatgtaaccgctaggcactgcactcatgacatattaccgctaattgctacttggctacaattg
aatggtacctacctaccaggcaatgacacagctgtaatgatgaatggtgataaaaatgaatctattgccataaaa
tttggagaaaactttagggtaaatctaacctgtattaggccaggaaataagacaattagaaatctacagatagga
gcaggcatgaccttctattctcagctgatagtggatggagacacccgcagggcatattgcaagatcaataaaact
caatgggatcctgctttgagacaggctatgatagctatgagagaccattggcacaatagactccaaaaagctaat
aagacaataaacaaaacggaaataaaaataaggtggacctcagagccaaagggagatagggaggttcagacacac
tggtttaattgccaaggggaattcttctactgcaacttgtcagtattattccaatttgagaataacaccagagaa
attaatgactcaaatatacatacagtaaaaaccaaatatcgccatgaccaatggatggtctgcagaataaggcag
tttgtaacccagtggggttacgtatcaaagtccatatacctgccccctcgaaaaggtcatgttcaatgtacatcc
catgtaacagggctattaatagatggggccatgtacggcaatacaataaacatgaccccctcagccaatgtctgg
acgcgtgcgtctagactgagagatacagtggtagagatagaccctctttccgtggcaccgacagaagtgcgcagg
agacctgtgccccacgtcaaggctagggcaatcagcttgggaataaccttcttaggattcctcagtgctgcggga
ggcacaatgggcgcagcggcgacagccctgacggtacagtctcggtctttgttggctgggatagtgcagcaacag
gctaatctgttgagagccgtagaagcccaacaacatctactacagttgtctgtttggggcataaaacaactacag
gctcgcctgacagcactggagaaattcgtgaaggaccaagccatactaaatctgtgggggtgcgccaataggcaa
atctgccatacctcggtgccatggaatgattcttgggcaaaccacacccaaccaggatgggacaacatgacctgg
caacagtggagcagattagtggacaatgacacagcagtcatccatggtctattggaaatggctcaacaacaacag
gaagaaaaccaacacaagttgcagaagcttttagaatggattccctttgggaatggtttgacatttccaaatgg
ctctggtatgtcaagatattttgcatggtagtagcaggcttaatagctttaagaatagttatgtttatattagga
atgcttagaaggatttcctttagggaagccttagggcgcttcatcgcacacgcacaa
>#4 EF070330 2157 nt
ataatatggggcaatcagtgggtcacagtgttctatggggtaccggtatggaaagatgcaactccaccactcttc
tgtgcatcagatgctaacatagcaaacagggaaccagggaatgtgtggatcaccacagcatgtcttcccacagat
cccgcaccccaggaagtacaattaaatatctctggggaatattttaatgtctataaaaattacatggtgacccag
atggttgatgatgtaaggtccatattcaaccaggccctaaaaccctgtgtcaaattgacccctatgtgtgtcaaa
atgaaatgcagtagaataaatgtaacaaggccatcaacaacaacaacagcatcaacaccaacaaaaacaacacca

gtgaccccttggggggaaatgggacgccaatggtacagagcaaagcatgttcaattgttcattcaatcagactaca
gaattcagagacaaaaagaagcagatgtactccttattttacactgatgatctgatgcgtgaggataatgatact
tattacatattaaactgtaacacctcatacataactcagcaatgtgaaaagagcagcttccaaccggttccgata
caatactgtgcacctgcagggtattctctattgaaatgtaatgatgctaattttacaggacaaggaaaatgtaac
aatgtgacagctacgcattgcacgcatggcatattacccttggttgctacctggttacagcttaatggcacatat
ttagaaggaaataatacagcagtaatgatgaatggggacaaaaatgaatctataggaataaagtttgggaaaac
tttagagttaacttaacctgcttaaggccaggcaataagactataaggaacctccagataggggcaggcatgact
ttctattcacagcttatagtaggaggggacacacgaagagcatactgcaagataaacaaaacacagtggatgta
gcattaaggcaagctatgttagcaatgagaaagcattggcatggccgcttggagaaacaaggagaaaatattaca
gaagaaaagataaaaatcaggtggacctcccagcccaaaggggataatgaggtccaaacacattggtttaattgt
gcaggggaattcttttactgcaatctatcagtgctgtttcaatttaacaacaatatcacacaaattaacgagagc
aatatacataatgtgacaaccaaataccaccaagatcaatggatggtctgtaggattagacagtttgtcacgcaa
tggggctatgtgtcaaaatccatctacttgcctccacgacggggacatgtgcagtgtacttctaatgtgactggg
ctccttattgatggagctatgtataaagacacagtcaacatgactccatccgcgaatgtgatggacgcctggcga
atggaactaaaaaattacaaggtagtggaaatagacccgctttccatggctcccacagaggtgcagaggaggcct
gttccccatgtaaaagcaagagcaatatctctaggcataacctttctgggatttctcagcgcggcaggaggcacg
atgggcgcagcggcgacagccctgacggtacagtctcggtcattactggccgggatagtgcagcagcaggcaaat
ctgctgagggccgtagaagcccagcaacatctgttacagttgtctgtctggggtataaaacagctacaggcccgc
ctgacagcgcttgagaagttcataaaggatcaagccttgttgaacctttggggatgtgcgaacaggcagatctgt
cacactagggtgccatggaatacatcatgggccaatcataccttaccaggctgggaaaacatgacctggcagcag
tggagcaagttggtggataatgatactaataccatacagggcctcctagaagcagcacaaaagcaacaggaagaa
aaccaacacaaattgcaaaaattattagaatgggactctttatgggaatggtttgacatctccaagtggctttgg
tatattaagatcttttgtatggtagtagcaggtttagtgctttttagaatattcatgtttgtgctaggcatactt
agggaacaagcccgccgctgcagtgaactcttagcgcgcctcttagcatacattcaa
>#5 AY340701 2088 nt
tggaccaccgtctactacggtgttccagtatggaaaccggcaactcctcctctcttttgtgcctctgatccaaat
tacgggtctaaagaggcagggaacaattggctggcgtcttcctgcctcccgacagaccctaccccgcaatctcta
tatttgaacatcactgaggagttcaatgcttatcaaaactacatggtggaagaaatggtagaggatatgaagtct
ctgttctcgcaggctttaaagccttgtgtgaaattaacacccatgtgtgttaggatgctctgtgttgaagttaac
accgtttcgaatgccagtaccactcctgcccctagtacccccacaccctggggaaattggggagggaatggaaca
ggacagccagtatataattgctcatttaaccagactacagaattcagggataaaaagaagcaaatgtatagctta
ttttggaaggaagacataatgaaggaggaaggaagcaatggcagtcactattacatcctgaattgcaacacatca
tacataacccaagcttgtgagaagtccaattatgagccagttcctttgcattattgtgcacccccaggctatgct
ttattgagatgtgatgaccccgcctttacgggacaagggagttgttctaatgtttctgcagtaacatgtactcat
gctatccaacctatagtagctacttggttccagttgaacagtacgggtaatgctcccaatacaacagtaatgatg
aataagcagaaaaatgagtctatagtagttagattggctaagcacctacatgtcaacattacttgcattaggcca
ggaaacaaaactattaggaatttacagataggagcaggcatgacattctattcgcaacttatagtgggaggtaat
actcgtaaagcctactgcaaagttaataagacccaatgggagactgccctacaggcagttcacgaggcagtaaaa
actgagtgggaaaagaaaaacaatggaaccaatgtgaccacaatatcttggagatttcaaccccaaggggacaag
gaggtccagactcactggtttaactgccagggagaattcttttattgtaatgtctcagctttattcattaataga
agaaccaacaaaacggacggatatctccattcgacgtgaataacaagcccaacaccacgtatcatggtgggtgg
ctagcatgtaccataagacaaatggtgacacaatggggatatgtgtcaaagagtatctacctacctccacggaaa
ggccatgtgcagtgtacatctaatatcactgccctcctgattacaggggagttgtatcaaaacaacgtgaccctt
gttccctcggcacaggtgagtgactcatggagatcagagctcagtcgatataaggtggtggaaattgaccccta
tctatggcacctacaacagcgcagagaaggactggggtacacagggaaaagagagcaattactttgggtatggcc
tttctgggcttcctcagcacggcaggaggcacaatgggcgcagcggcgacagccctgacggtacagtctcggtca
ttactggctgggatagtgcagcagcaggagaacctgctgagggccgtaacagcccaacagagcttactacagctt
actgtttggggagtcaagcagctacaggcccgcctgacggctgtagagaagttcataaaggaccaaacattgcta
aatgcatggggatgtgccaataaagccgtgtgtcacacaacagtgccgtggaacaattcttgggcaaaaggtcac
ttccctgagtgggacaatatgacatggcaacagtggagtgagttagtagataatgacacaatgaccattcagcag
ctcttggaagctgcgcaagagcagcaaggcaaaaaccaacatgagttaatgaagccgggacaatgggacttcctg
tggaattggtttgacatctccaaatggttgtggtacatcaaaatattcattatagtagtagcagctttgataggc
ttaagaatacttatgttcatactaggagttatcagtaggttacgcttgctagcctactgccag
>#6 KJ461716 2250 nt

atgacaaagtataagcttatagtattaggtatagcaattataataggtttaataacaatagatttgcaacagggg
gatgaaaatgatggatgggtaacagttttctatggggtaccagtgtggcataatgccacccctcctttgttttgc
acagcagatgcaagacatacatgggtcactactaattgcttgccaacagatccagcccctatagagacacctatg
aatatctcaggagaatggttcaatgtacacaccaattatatggtggaccatatggacaaagatatggcagcattg
tttctacaggctacaaagccatgtaccaaattgacccctatgtgtgttaaaatgaagtgtcagaattatactaaa
gaagctacaaccacaacaacaccaacatcatcaacaacatcaaaagcaccaccaacaacaacccaaaggcctgca
acagaatggtggggaggaaaagatcctcaatctttactcaattgcacttttaacatgaccccaggttttaaagat
aggaaagctcattattgggctccttttttatacattggatttatggcaacaatctaatggtactaatggaacaggg
gaatattatgtaaagtattgcaacacgtctgctatgacacaagcctgtgataaatttcattttcagcctttccca
gttcattactgtccaccagcagggtatgcattgttcaaatgcaatgacataccatggaatggacaagggccatgt
aaaaatgtgacagcagtacattgtacacatgcaattaatacattggctagtacatggctccagttaaatggcaca
tatgaaaagaatacagatgaggtgcaggttatgagaaagtataattccaattacagtgtagcttttatcttctca
gagagccaaatagtaaatttaacatgtgtaagaccagggaataaatcaataaggaacttgcaaataggagcaggc
atgaccttttactcacaactcatagtaggggaaatactagaagagcttattgcaaataaatggcacacagtgg
tacaatgctttaaatgctacctatagggccataaagaaggagtataatcttacacgggatcaaccaatcacctgg
agatctcaaccaagggggatagagaagtggagagtcactggttccagtgtcaaggggagttcttttactgtaat
ttatctaggatctttcagaaaaccaatttcacaaatgataccttctatccacgaaacataacaaaggaaggggca
gagttgagcaacatatggttcacatgtaccatcaggcaagtagtgaacagatggtcacatgtagaaaagttgatg
tacttaccaccaagaaggggacatgttcagtgtacatctaatgtcacaggaatcttggtggaatctgaatactat
ccaggaagtccatttaacatgaccccatcagccaatatcaaggacttgtggaaaatagatttaagaagatataag
gtggtggaaatagatccaattggcctagcacccactccaataaaaagatatgaaccaccaccaagcaaaagcgtt
gtcaaaagagcagccgcattgactctgggattcttgggatttctcagtacggcaggggcgacaatgggctcggtg
gcaaccgccttgacagtccagtcgcgttccctcttagaaggcatagtagcccagcaaaaggcgctacttgaggca
gttgagcaccaacaacagcttctacagctgtctgtgtggggatcagaaatctcaatgcccgccttaccgctatt
gagaaatatgtcaaggaccaagggatcttggcttcatatgggtgccaattcaaacaaatttgttatacctcagtt
ccttggaataaatcctttgctgatggagcacttcctgcttggcacaacatgacctggatagagtgggaaagaaaa
gtgactaatcatacaggtatcatcaatactttgttggtagaggcccaaaggaggcaagaagaaaacacacataag
ttacagaagttaggagaatgggataatctttggaattggtttgacatctccaagtggtttcagtggattaaaata
gcagtgttgatagtaataggtttaatagcattaagaatagtaatgtggcttattaatatattgtggtggtttgga
cgtgcttatcctcgcttgatactagcagcagaagcaacagcagaagcaaccagaagagcagtagcttacattcac
>#7 KJ461714 2346 nt
atgacaaagtatacgcttatagcattaggtatagcaattataataggtttagtaataatagatttgcaacaaggg
gaagaaaataatggatgggtaacagttttctatggggtaccagtgtggcagaatgcctcccctcctttgttttgc
acagcagatgcaagacatacatgggtcaccatgaactgcttgccaacagacccagcccctgtagagacacctatg
aatatcacaggagaatggttcaatgtgcacaccaattatatggtggaccacatggatcaagatatggcagcgctg
tttctacaggctacaaagccatgtaccaaattgacccctatgtgtgttaagatgaagtgtagcaattatactaga
gaggctacaacagcagcaccatcaacaacagcaccaacgaccaaagggcatacaacagaatggtggggaggaaaa
gatcctcaatctttactcaattgttcttttaacatgaccccaggttttaaagataggaaagctcattattgggct
ccttttttataaattggatttatggcaacataataatggtactaatggcacaggggaatattatgtaaagtattgc
aacacgtctgcaatgacacaagcctgtgacaaatttcattttcagcctttcccagttcattactgtccaccagca
gggtatgcattgttcaaatgcaatgacataccatggaatgggcaagggcaatgtaaaaatgtgtcagcagtacat
tgtacacatgcaattaatacattggctagtacatggctccagttaaatggcacatatgaaaagaacacagatgag
gtgcaggtcatgagaaagtataattccaattacagtgtagcttttatcttttcagagagccaaatagtaaatttg
acatgtgtaaggcctgggaataaaacaataaggaatttgcagataggatcaggcatgaccttttactcacaactc
atagtaggggagatactagaagagctttttgcagaataaatggcacacagtggtaccatgctttaaatgctact
tatagggctataaagaaggagtataatcttacacagaaccaaccaatcacctggagatctcaaccaagggggac
agagaagtagagagtcactggttccaatgccaaggggaattcttttattgtaacttatctaggatctttcagcaa
accaattttaccaatgacaccttctatccaagaaacataacagaggaaggggaaaggttgagcagaacatggttc
acatgtaccatcaggcaagtagtgaataggtggtcacatgtagaaaagttgatgtacttaccaccaagaaggga
catgttcagtgtacatctaatatcacaggagtcttggtggaatcagaatactatccagggagtccatttaatatg
accccatcagccaacatcaaggacctgtggaaaatagatttaagaagatataaggtggtggaaatagatccaatt
ggcttagcacccactccaattaaaaggtatgaaccaccatcaaacaccaaaagtgttgtcaaaagagcagccgca
ttgaccctgggattcttgggatttctcagtacggcaggggcgacaatgggctcggtggcaaccgccttgacagtc
cagtcgcgttccctcttagaaggcatagtagcccagcaaaaggcgctacttgaggcagttgagcaccaacaacag
cttctacagctgtctgtgtggggatcagaaatctcaatgcccgccttaccgctattgagaaatatgtcaaggac

caagggatcttggcttcatatgggtgccacttcaaacaaatttgttacacctcagttccttggaatatatctttt
gctgatggaggagtgcttcctgactggaataatatgacctggatagaatgggaaagaaaagtgaccaatcataca
ggcatcattaataatttgttggtagaggctcaaaggaggcaagaagaaaacacacataagttacagaagttagga
gaatgggataatctttggagttggtttgatatctccagatggtttcagtggattaaaatagcagtgttaatagta
ataggtttaatagcattaagaatagtaatgtggcttattaatatattggggttcttagcagttgtctgggacgac
ctgagagcgatcgtcttgtggatccaccggacattggtcgacttaggctggctgattcgtcatctgtggagcagt
ctaactgactggtggtttggaagtgcttatcctcgcttgatactagcagcagaagcaacagcagaagcaactaga
agagcaatagcttacattcaa
>#8 KJ461715 2262 nt
atgacaaaacataagcttatagtattaggtatagcaattataataggtttaataatagtagatttgcaacaaggg
gaagaaaataatggatgggtaacagttttctatggagtaccagtgtggcataatgccacccctcctttgttttgc
acagcagatgcaagacacacatgggtcactactaattgcttgccaacagatccagcccctatagagacacctatg
aatatctcgggagaatggttcaatgtacataccaattatatggtggaccatatggaccaagatatggcggcattg
tttctacaggctacaaagccatgtactaggttgacccctatgtgtgttaagatgaagtgtagtaattttactaaa
ccaacttcaacaccacaaccaactacatcatcaaaaccaacaacattgacagcgacaaccaaaaagcttgtaaca
gaatggtggggaggaaaagatcctcaatctttactcaattgctcttttaacatgaccccaggttttaaagatagg
aaagctcactattgggctcctttttataaattggatttatggcaacataagaatgatagcgaaactaatgtcaca
ggggaaggggaatattatgtaaagtattgcaacacgtctgcaataacacaagcctgtgataagtttcattttcaa
cctttcccagttcattactgcccaccagcagggtatgcattgttcaaatgcaatgacataccatggaatgggcaa
gggccatgtaaaaatgtgacagcagtacattgtacacatgcaattaatacattggctagcacatggcttcagcta
aatggcacatatgaaaagaacacagataaggtgcaggtcatgagaaagtataattccaattacagtgtagctttt
atcttctcagaaagccaaatagtaaatttaacatgtgtaagaccagggaataaatcaataaggaacttgcaaata
ggagcaggcatgacctttactcacaactcatagtgggaggaaatactagaagagcttattgcaaaataaatggc
acacagtggtactctgctttaaatgctacctatcgggctatacagaaggagtataatcttacagagaaccaatca
atcacctggagatctcaaccaaggggggatagagaagtagagagtcactggtttcagtgccaaggggaattcttt
tattgtaatttatctaggatctttcagaaaaccaatcttacaaataacaccttttacccacaaaacataacacag
gaaggggaagcgttgagcaacatatggttcacatgtaccatcaggcaagtagtgaacagatggtcacatgtagaa
aagttgatgtacctaccaccgagaaggggacatgttcagtgtacatccaacatcacaggaatcttggtggagtca
gaatactatccaggaagcccatttaacatgaccccatcagccaatatcaaggacctgtggaaaatagatttaaga
agatataaggtggtggaaatagacccaattggcctagcacccactccactaaaaaggtatgaaccaccaccaagc
aaaagcattgtcaaaagagcagccgcattgaccctgggattcttgggatttctcagtacggcaggggcgacaatg
ggctcggtagcgaccgccttgacagtccagtcgcgttccctcttagaaggcatagtagcccagcaaaaggcgcta
cttgaggcagttgagcaccaacaacagcttctacagctgtctgtgtggggggatcagaaatctcaatgcccgcctt
accgctattgagaaatatgtcaaggaccaagggatcttggcttcatatgggtgccaattcaaacaaatttgttac
acctcagttccttggaataaatcttttgctgatggtgaagcgcttcctgaatggaacaacatgacctggatagag
tgggaaagaaaagtgactaatcatacaggtatcatcaatgggttgttggtagaggcccaaaggaggcaagaagaa
aacacacataagttacagaagttaggagaatgggataatctttggagttggtttgacatctccagatggtttcag
tggattaaaatagcagtattaatagtaataggtttaatagcattaagaatagtaatgtggcttattaatatatta
tggtggttcggaagtgcttatcctcgcttgatactagcagcagaagcaacagcagaagcaactagcagagcagta
gcttacattcac
>#9 JX860407 2241 nt
atgggatgtcttgggaatcaactgcttatcgccatcttgctagtaagtgcttatgggatctattgtgctcaatat
gtcacagtattctatggtgttccagcatggaggaacgcgacaattcccctcttctgtgcaaccgagaatagggac
acttggggtacaactcaatgcttgccagataatgatgactattcagaactagcaatcaatgtcacagaggctttt
gatgcctggaacaatacagtcacagagcaagcaatagaggatgtctggaatctctttgagacatccataaaacca
tgtgtaaaattaaccccattatgtatagcaatgagatgcaataaagctgagacagacaagtggggactaacagga
acagcagcaccaccaacaacaaagacactaaagacaacatcaacaacaaaaccaccagtactcgaagtaaatgat
accgaaccctgtgtaaaacttaacaactgcacaggattggaaccagaaccaatgataggatgtaaattcaatatg
acaggattgaaaagaggcaggaaaaaggagtacaatgaaacatggtactctagtgatttggtctgtgagcaaggt
ggtaacaacacaaatgaaagcagatgttatatgaatcattgtaatacaagtgtaatccaagagtcctgtgacaaa
cattattgggatgctattagatttagatactgtgcaccaccaggctatgctttgcttagatgtaatgattcaaat
tattcaggttttatgcctaattgttctaaggtagtagtctcttcatgtacaaggatgatggaaacacaaacctct
acatggtttggttttaatggcactagggcagaaaatagaacttacatttattggcatggtcataggaatagtact
ataattagtttaaatagctattacaatttaacaataagatgtagaaggccagggaataaaacagtactaccagtc
accataatgtcagggttagttttccattcacaacccataaatgagaggccaagacaggcatggtgctggtttgga

ggaaaatggaagtcagcaataaaagaggtgaaggagaccttggtcaaacatcccaggtatacaggaaccaatgat
acaagtaaaattaatctgacagcaccaggaggaggagaccctgaggtcaccttcatgtggacaaattgtagagga
gaattcctctattgtaaaatgaattggttcctaaattgggtagaagatagaaacagaacgcagatggaggcgaga
aacaacaacgataaagagaggagaaagaggaactatgtgccatgtcacattagacaggtgatcaatacatggcac
agagtaggtaaaaatgtatatttgccgcctagagaaggagacctgacatgtaactccactgtgaccagtctaata
gcagaaatagactggtatgataaaaaccaaactaatatcaccatgagtgcagaggtggcagaactgtatcgattg
gaattggggggattacaaattagtagaaatcactccaattggcatagccccaacaaatgtaaagaggtacaccact
actactacctcaagagctaaaagaggggtctttgtgctagggttcttgggatttctcgcaacggcaggttctgca
atgggcgcggcgtcgttgacgctgactgctcagtctcggactttactggctgggatagtgcagcaacagcaacag
ctgttggacgtggtcaagagacaacaagaattgttgcgactgaccgtctggggaactaaaaacctccagacaaga
gtcactgccatcgagaagtacttaaaggaccaggcacagctaaattcatggggatgtgcgtttaggcaggtctgc
cacactactgtcgaatggccagccgcaaatgtgacaccagattggaataacatgacctggcaagagtgggagaga
aaggttgactatctagaggcaaatataacgcttctattagaagaagcacaaattcaacaagaaaagaacatgtat
gagttgcaaaaactaaatagctgggatgtatttggcaattggtttgatctcacctcttgggtaaaatatatacag
tatggaatatatatagtagtaggaattatattgttaagaatagtaatctatgtagtacagatgttagctaggtta
aggaaagggtataggccagttttctcttccccacctaattatcgccagcagctcacctatattgga
>#10 U79412 2226 nt
atgggatgtcttgggaatcagctgcttatcgccatcttgtttctaagtgcctatgggatctattgcattcaatat
gtcacagtcttttatggtgtaccagcttggaggaatgcgacaattcccctcttctgtgtaaccaggaatagggat
acttggggaacaactcagtgcctaccagataatgatgattattcagaattggcccttaatattacagaaagcttt
gatgcttgggagaatacagtcacagaacaggcaatagaggatgtatggcatctctttgagacctcaataaagcct
tgtgtaaaattaaccccattatgcattactatgaaatgcaacaaaagtgaaacagataaatggggattgacaaaa
tcatcaacaacaacagcagcaccaacaacaaaaacaacaacaacaaaggaaatagaagtggtcaatgaaaatagt
acttgtgtaaatcgtgataattgcacaggcttggaacaagagccaatgataagctgtaaattcaacatgacaggg
ttaaaaagagacaagaaaagagagtacaatgaaacttggtactctgcagatttggtttgtgaacaaggtaatagc
actgaagatgaaagtagatgttacatgaatcactgtaacacttctgttattcaagaatcttgtgacaaacattat
tgggatgctattagatttaggtattgtgcacctccaggttatgctttgcttagatgtaatgacacaaagtattca
ggctttatgcctaactgttctaaggtggtggtctcttcatgcacaagaatgatggagacacagacttctacttgg
tttggctttaatggaactagagcagaaaatagaacttatatttactggcatagcaaagataataggactataatt
agtttgaataagtataataatctaacaatgaaatgtagaagaccaggaaataagacagttttaccagtcaccatt
atgtctggattggttttccactcacaaccaatcaatgaaaggccaaaacaggcatggtgtaggtttgaaggaaat
tggaaggaggcaataaaagaggtgaagcagaccattgtcaaacatcccaggtatactggaactaacaatactgat
aaaatcaatttgacggctcctcgaggaggagatccggaagttaccttcatgtggacaaattgcagaggagagttt
ctctactgtaaaatgaattggtttctaaattgggtagaagataagaatctgactggaactacccagaagccacag
gaacagcataaaaggaattacgtgccatgtcatattagacaaataatcaacacttggcataaagtaggcaaaaat
gtttatttgcctccaagagagggagacctcacgtgtaactccacagtaaccagtctcatagcaaacatagattgg
attgatggaaaccaaactaatatcaccatgagtgcagaggtggcagaactgtatcgattggaattgggagattat
aaattagtagagatcactccaattggcttggcccccacaaatgtgaagaggtacactactggtggcaccccaaga
aataaaagaggggtctttgtgctagggttcttaggttttctcgcaacggcaggttctgcaatgggcgcggcgtcg
ttgacgctgaccgctcagtcccggactttattggctgggatagtgcagcaacagcaacagctgttggacgtggtc
aagagacaacaagaattgttgcgactgaccgtctggggaacaaagaacctccagactagagtcactgccatcgag
aagtacttaaaggaccaggcgcagctaaatgcttggggatgtgcatttagacaagtctgccatactactgtacca
tggccaaatgcaagtctaacaccaaattggaacaatgagacttggcaagagtgggagcgaaaggttgacttcttg
gaggaaaatataacggcccttctagaagaggcacaaattcaacaagaaaagaacatgtatgaattacaaaagttg
aatagctgggatgtgtttggcaattggtttgaccttgcttcttggataaggtatatacaatacggagtttatata
gttgtaggagtaatactgttaagaatagtcatctatatagtacaaatgctagctaagttaaggcaagggtatagg
ccagtgttctcttccccaccttcttatttccagcagctaacctacctacaa
>#11 JQ864086 2250 nt
atgggatgtcttgggaatcagctgcttatcgcgctcttgctagtaagtgctttagagatttattgtgttcaatat
gtaacagtattctatggtgtaccagcatggaagaatgcgacaattcccctcttctgtacaaccaggaatagggac
acttggggaacaacacaatgcttgccagataatgatgattactcagaattggcaatcagtatcacagaggctttt
gatgcttggaataatacagtcacagaacaagcaatagaggatgtgtggaacctctttgaaacatccattaagccc
tgtgtaaaactcaccccactatgtatagcaatgagatgtaataaaactgagacagataggtggggtttgacaagg
aacgcagggacaacaacaacaacaacaacaacaacaacagcagcaacaccaagtgtggcagaaaatgttataaat
gaaagtaatccttgcataaaaaataatagttgtgcaggcttggaacaggagcccatgataggttgtaaatttaac

atgacagggttaaaaagggacaaaaggatagaatataatgaaacatggtattcaagagatttaatctgtgagcag
tcagcgaatgaaagtgagagtaaatgttacatgcatcattgtaacaccagtgttattcaggaatcctgtgacaag
cattattgggatgctattagatttagatattgtgcaccgccaggttatgctttgcttaggtgtaatgattcaaat
tattcaggctttgctcctaactgttctaaggtagtggtttcttcatgcacaagaatgatggaaacgcaaacctct
acttggtttggcttcaatggtactagggcagaaaatagaacatacatttattggcatggcaaaagtaatagaacc
ataattagcttaaataagtattataatctaacaatgagatgtagaagaccaggaaataagacagttttaccagtc
accattatgtcagggttggtcttccattcgcaacccataaatgagagaccaaaacaggcctggtgctggtttgga
ggaagctggaaagaggccatccaggaagtgaaggaaaccttggtcaaacatcccaggtatacgggaactaatgat
actaagaaaattaatctaacagctccagcaggaggagatccagaagtcacttttatgtggacaaattgtagagga
gaattcttatactgcaaaatgaattggtttcttaattgggtagaggacagagaccaaaagagtagcagatggaga
caacaaaatacgagagagcgacagaagaaaaattatgtgccatgtcatattagacaaataatcaacacgtggcac
aaagtaggcaaaaatgtatatttgcctcctagggaaggagacctgacatgtaattccactgtaactagtctcata
gcagagatagattggaccaatagcaatgagaccaatatcaccatgagtgcagaggtggcagaactgtatcgattg
gagttgggagattacaaattagtagagattactccaattggcttggcccccacaagtgtaagaaggtacaccaca
actggtgcctcaagaaataagagaggggtctttgtgctagggttcttgggtttctcgcgacagcaggttctgca
atgggcgcggcgtcgctgacgctgtcggctcagtcccggactttgttggctgggatagtgcagcaacagcaacag
ctgttggatgtggtcaagagacaacaagaattgttgcgactgaccgtctggggaactaagaacctccagactaga
gtcactgctatcgagaagtacctgaaggatcaggcgcagctaaattcatggggatgtgcttttaggcaagtctgt
cacactactgtaccatggccaaatgaaacattggtgcctaattggagcaatatgacttggcaagagtgggaaaga
caggttgacttcctagaggcaaatataactcaattattagaagaagcacaaattcagcaagaaaagaatatgtat
gaattgcaaaaactaaatagctgggatatctttggcaattggtttgaccttacttcttggataagatatatacaa
tatggtgtactaatagttttaggagtagtagggttaagaatagtaatatatgtagtgcagatgttagctaggtta
agacagggttataggccagtgttctcttcccctcccgcttatgttgtcatcagaattggaatagcctacctacaa
>#12 AF334679 2256 nt
atggcgtgtcctggacttcacctgcttatagacatcttgtttttaagtgtgttagggacctggtgtgcacagtat
gtaacaatcttttatggtattcctgcatggaggaacgctacgatccccctcttctgtgcgacccagaatagagac
acatgggggaccgttcagtgcttgccagataatggagactattcagaattggcccttaatgttacagaggccttc
gatgcttgggataatacagtaactgaacaagcaatagaagatgtttggaatctctttgaaacatctattaaacct
tgtgtaaagttaactccattatgtattgctatgaaatgtaataaaaatgagacagacagatggggggttgacaaga
gcagctactactactagctcaccaactactactagccccttaactgctgctagcccatcaggagaggaaatcgtt
aacgacactatgtcttgtacaaagaacaacaattgctctggcatagagcaggaaccaatgataggttgtcaattt
aacatgacaggactaaaaagagaccagaaaaggcagtacaatgaaacctggtactccagagatctagtctgtgag
caaggaggaaatgaaagcagtagatgttacatgaatcattgcaatacaagtgttatacaagagtcatgtgacaag
cactattgggatgccatcagatataggtactgtgcaccaccaggctatgctttgcttagatgtaatgatacaaat
tattcaggctttgcacctaattgtagtaaggtagtagtatcatcatgtacaaggatgatggaaacacagacttct
acatggtttggctttaatggtactagagcagagaatagaacatatatatattggcatggcggaagtaatagaaca
ataattagcttaaataagtattataatttgacaataaagtgcagaagaccaggaaataagacagtcctaccagtc
accattatgtcaggtttggtctttcattcgcaaccaataaatgagaggccaagacaagcatggtgctggtttgga
ggaaaatggagagaagctatgcaggaggttaagaaaaccatagtcaaacaccccaggtatactggaaccaatgat
actaggaaaattaatctaacggcccgggaggaggggatccggaagtcacattcatgtggacaaattgcagagga
gaattcctttactgtaaaatgaattggttcctcaattgggtagaggataggaatacgagcagcccaaggtggaca
actcaaaccaaaaaggaacagcacaaaagaaattatgtaccttgccacatcaggcaaataattaatacgtggcac
agggtaggaaaaaatgtctatttgcctccaagggaaggagacttaacttgtaactccacagtgaccagcctaata
gcaaatattgattggattgacaacaatgagaccaatattaccatgagtgcagaagtggcagaattgtatcgattg
gaattggggggattataaattggtagagatcactccaattggcatggctcccacacatgtgaaaaggtacaccaca
agtacctcaaagaataaaagaggagtctttgtgctagggttcttgggtttctcgcgacggcaggttctgcaatg
ggcgcagcgtcgctaacgctgactgctcagtcccggactttattggctgggatagtgcaacaacagcaacagctg
ttggacgtggtcaagagacaacaagaattgttgcgactgaccgtctggggaactaaaaatcttcagactagagtc
accgccatcgagaaatacctaaaggatcaggcacagctaaattcatggggatgtgcatttaggcaagtctgccac
actactgtactatggccaaatgacagtcttgtcccagactggaacaatatgacatggcaagaatgggagaaaaag
gttgaattcctagaggcaaatataactcaaatgttggaggaagcacgactacagcaagagaaaaacatgtatgaa
ttacagaaattgaatagctgggatgtgtttggaaattggtttgaccttacctcctgggtaagatacatacagtat
ggagtctttctagtcataggaatagtattgttaagaatagttatctatgtagtacaaatgttaagtaggttaagg
cagggttataggcctgttttctcctccccccatcttatcatcagcaggagcttgtcagaagagaagcaggctat
atccgt

>#13 JX860414 2223 nt
atgggatgtcttggcaatcagctgcttatcgccagcttgctactaagtgcttgctcgatatattgcaagaaatat
gtaactgttttctatggcattcccgcatggaggaacgcgacagttcccctcttctgtgcaaccaagaatagggat
acttggggaacagtccaatgcttgccagataatgatgattactcagaattagctctcaatgtcacagaggccttt
gatgcttgggagaacacagtcacagaacaggcagtagaggatgtatggaacctctttaagacttctataaaaccc
tgtgttaaactaacaccactatgtatagctatgagatgcaataggacagagacaaatcgatggggactaacgggc
aactccgacacaacaacagtaacacctagcgccaaaactacaacagaaaaaggtgaaataaatgacactgacccc
tgcttcaaaggtaacaactgtacagggataggaagtgaagaattggtatcttgcaaattcaatatgacagggtta
aagagagacaaaaagaaggagtacaatgagacctggtactcaagggatttggtctgtgaacgagacggaaatgaa
atagcaaatgaaagtagatgctacatgaatcattgcaatacttctgtaatccaggaatcttgtgacaagcattat
tgggatgctataagatttagatattgtgcaccacctggctatgctttgcttagatgtaatgatacaaattattca
ggctttgcacctaattgcagtaaggtagtggtctcttcatgcacaaggatgatggaaactcaaacctctacatgg
tttggatttaatggcactagagcagaaaatagaacatacatatattggcatgggagagacaataggactataatt
agtttaaataagtattataatctaacaataaaatgtagaagaccaggaaacaagacagttatgcctgtgacaatc
atgtcaggcttggtcttccattcccaaccaataaatgacaggccaagacaagcctggtgttggtttggtggaaaa
tggaaatcagctatgcaggaggtgaaacaaacccttgtaaaacatcccagatatactggaaccaatgatactaaa
aacataaactttactgagccaggaaaaacctctgacccagaagtagcttttatgtggacaaattgcagaggagaa
ttcctctattgtaaaatgacttggttcctcaattgggttgaagacagaaacatgtcttcacaaaacaaggcagaa
caaaaaagacgtaactatgtgccttgccacattcggcagataattaacacttggcacaaggtagggaaaaatgta
tatttgcctcctagggaaggagaactgacatgtaattcaacagtaaccagtcttattgctaacatatattggatt
gatggaaacagaacaaatatcaccttcagtgcagaggtggcagaactgtacagattggagttggggggattacaaa
ttagtagaaattactccaattggctttgcacctaccgatgtaaaaagatattcctctgtaacgacaccgaggaat
aaaagaggtgtgttcgtgcttgggttcttgggatttctcgcaacggcaggttctgcaatgggcgcagcgtcgttg
acgctgtccgctcagtcccggactttgttggctgggatagtgcagcaacagcaacagctgttggacgtggtcaaa
agacaacaagaactgttgcggctgaccgtctggggggactaaaaaccttcagaccagagtcactgccattgagaaa
tacctacaggatcaggcaaagctaaattcatggggatgtgcattcaggcaagtctgccacactactgtaccgtgg
gtcaatgattcactccaaccagactggaacaatatgacttggcaagaatgggagaaacaggtagcttacctagag
gcaaatattacacagcaattggaagaagcacaaatactacaagaaaagaatatgtatgatttacaaaaattaaat
agttgggatgtttttggcaactggtttgacctcacgtcatgggtaaaatatgtctatttcggattctatgtagta
gcaggagtaatagtattaaggatagtaatctatgtaatacagatgttaggtaagttaagaaaggctataggcca
gtgttttcctcccctcccacttatcgccagcagctgacctacctccaa
>#14 HM803689 2172 nt
atgaagtgcctaagcttaatagtgttaagtagtctgattcttttagctttagcaaaacagaagccacaatatgtg
acagtattttatggagtaccagtatgggtcaacagtacagtacctatgttttgtgttactgacaacacaaactca
tggggtactttaaattgcatcccagaaggaggaatatcaccagaagtaccagttaatgtgtcagaaaaatttgat
gcttgggaaaatgccctgtatgaacaagcaaaagataatgtgtggaatctttatgattctaccctaaaaccttgt
gttaggttatcccctttatgtgtaacaatgaattgtactgtaataaatgggagttgggatggtataacaacacca
gctccatcaccagcacctacaacaccaacagcagcaaaaactacaacaaaagtagattgccctatgaataatgaa
acatgtgcagcagttccagatgaggatgttatgaattgtgagtttgcagtggcaggattaaaaagggatgaaaaa
tataaagctaatgacacgtggtactcaagagatctttggtgtgagaaagataaaaataataataagactactaag
aggcaatgttttatgcgacattgtaatactactagtattcagcaattctgtgagcctaaatactgggaaccttt
aggattagatattgcgctccaccaggatttgccctattggtatgcaaagataaaaattatacaggatttgatacc
tgtcataatgtgacagccacttcatgcacagatatgataaatacaacagtatcaacctcatttggactaaatgga
tcaattagtgaaaacagaacttggatttatcagagaaagcaatcgaatcggacagtaatagggttaaatagagag
tataatctaacagtggaatgtagaaggccatcaaataggacagtgaagggaatctcattggctacaggagtcttt
atctccttaagagtagagaaacgaccaaaaggagcttggtgtagattttatggcaattggacaggtgcttgggaa
gaagttaaaaaagaggtaataaaaacgaaggatataaagggacaaatgatactaagaaaatatacataagatca
cattatggagggatgatgaagctaaatacttttggttaaattgtgatggggaattcttatattgcaaattgaat
tggtttctcaacctattaaataatagaactgatggaaacataaatgagcgaagacaagccatgtttgtgccttgt
atcacaaaaatggtggttaatgattggtatacagtgtcaaaaaaggtatacacaccaccaagacctgatgcattg
aaatgcagggccacggtcacttacttgttggcagacatagattatatacataccaatgaaacaaatgttactctg
actgcagaggtaggagatctatgggcagcagagttaggaagatacaaagtagtagaaattaaaccaattggctat
gcacctacagatgttaaaaggtacgagacaagacagaaaagagtacctttggtgctgggttttctaggtttcctc
tcggcagcaggtactgcaatggcgcagcggcgacagccctgacagtccagtcccggcatttgcttgctgggata
ttgcagcagcagaagaagttgctggacatagttgagcagcagcagcacttgctgaggctcaccgtctgggggact

aaaaacctacaggcgcgtgtcactgctattgagaaatacctagcagaccaatctctattgaatacatttggctgt
gcatggaggcaagtctgtcatacaacagtgcagtggactttcaatgcaacacctgaatggaataaacagacatgg
cttgaatgggaaaggaatatatcaataatagaagggaatatctctttagcattacaggaagcccaggatcaacat
gagagaaatgtgcatgaactggagaaattaaataattggggagatgcctttagttggctgaggcttgactggtgg
atggaatatctaaaaataggagttttataatttaggagttataggactaagaatttctttcttctatgggga
tgtatcagtaagattagagcagggtatactcctttgttgtcgcccccaccttattatcatcagcagatacaa
>#15 HM803690 2145 nt
atgaaaagtgcaataggaatagtgataatagctagtctaataagtgctatagtaagtaaacaatgggtaacagta
ttctatggggttccggtatggaaaaatagtacagcaccaatgttttgtgttacagataatgcaaattcatggggc
actctaaattgtataccagaaggggggactgtcaccagaggtacctgtaaatgtctcagagcagtttgatgcatgg
gaaaacagtttgtatgaacaggccaaggacaatgtgtggcatctatatgattccaccctgaagccttgtgttaga
ctatcacccttatgtataaccatgaactgcacagcattaaatggaagctgggatggatcagtaacaacaacaaca
caaccaagaacaacaccatccgcaacagcatcaacaaatatagactgtggaataaacaatgaaacatgtagtgca
gtagaggatgaaaatgttatgcagtgtacatttgcagtagcagggttgaaaagagatgaaaagcataaatacaat
gatacttggtattcaagagacctctggtgtcagaaagctaacaatgtgacccaatgttttatgagacactgtaac
acaactagcatacaccagttttgtgaaccaagatactgggaacccttaggttaagatactgtgctccaccaggc
tttgccttgctaaaatgcaatgattataattacacgggttttgatacttgtcataatgtaacagctacttcatgt
acagacatgataaacacaacagtgtctactagttttggattaaatggatcaataagtgaaaatagaacttggatc
tatcaaagaaaacagagtaacagaacaatcatagggttgaatagtttctataacttgacagtaagttgtagaaga
ccatcaaataggacaataaagggaatttccttagccacaggagtatttatatctctaaaagtagaaaaaaggcca
aaaggagcttggtgcagattttatggaaattggaaaggagcttggaaagaggtaaaagagaaggtcatacaaaca
aagggatataagggaacaaatgacacaactaaaataaacataaggtcagtttatggaggagatgatgagactaaa
tatttttggctaaattgcgatggagaattcctctattgtaaactcaattggtttttgaattatttgaacaataag
acagagggtaatgagaaggaaagaaggcaagccatgtttgttccttgcgtcacgaaaatgattgttaatgattgg
tacacggtgtcaaggaaagtttatacaccgccaaggcaagatgcgttaacatgtaatgctacagtaagctatttg
ttagcagacatagagtatgtgaatcaaaatgagactaatgtgacactgtctgcagaagtaggagacctatgggca
gcagaattgggaagatacaaagtagtggaaattaaaccaattggatatgcaccaacagctataaaaaggtacgag
acaaaacagaaaagagtacctttggtgctgggttttctaggtttcctctcagcagcaggtactgcaatgggcgca
gcggcgacagccctgacagtccagtcccggcatttgcttgctgggatattgcagcagcagaagcagttgctggac
atagttgagcagcagcaacacttgctaaagctcaccgtctgggggactaaaaacctccaggcccgtgtcactgct
attgagaaatacctagcagaccaatctctattgaatacatttggttgtgcctggagacaagtgtgtcacacctca
gtgccttggagctttaataagacaccggactggcagaaacagacatggcttgaatgggaaaggaatgtctcatat
ttagaagcaaacataacagcctcattacaagaagcacaagatcagcatgagaaaaatgtacatgaactagaaaag
ttgaacaattggggagatgcatttagctggctaaggctggactggtggatggagtatataaaaataggaatcttt
gttatactaggcattatagggttaagaatagtcttcttgctatggagctcygttagtaagcttagagcaggatat
actcctttgctctctcccccaccctattatcattcacaggtgcaa
>#16 M58410 2166 nt
ttttccccttacgaagagaggagaccattacaaccatgtactaataaatgctattgcaaaaaatgctgttatcac
tgtgagctttgcttcctgcagaagggcttaggagtgcgttatatggggagattgcttataaaaatactaataata
gcaatagggataagtataggaataggtaacctgtatgtgacagtgttttatggaatcccagtatggaaaaattca
acagttcaggcattttgcatgacgcccaataccaatatgtgggcaaccaccaactgcataccagatgatcatgat
aatacagaggtgcctctaaacattacagaagctttcgaggcttgggataatccgctggtaaaacaagcagagagt
aatatacatctactctttgaacaaacgatgaggccttgtgttaagctctcccccatatgtattaaaatgtcctgt
gtagagctgaatggtacagccacgacaaaggccaccactactgcaactacaacaatgactacccccctgtcagaat
tgcagtacagagcagatagaaggagaaatggcagaggaaccagcatccaactgcacttttgcaattgcaggatat
caaagagatgtaaaaaagaattatagcatgacctggtatgatcaggagttagtctgcaataataaaacaggaagt
gaaaagggaagtaaggattgttacatgatacattgtaatgattcagtgataaaagaagcttgtgataaaacatat
tgggatactttaagagtaagatactgtgcaccagcagggtatgctttgctaaaatgtaatgataaggattataga
ggctttgctccaaagtgcaagaatgtttcagtagtgcattgtactagattaatcaatactactataactacaggg
ataggattaaatggtagtagatcagaaaatagaacagagatatggcagaaaggaggaaatgataatgatacagtt
ataataaagttgaataagttttacaacttgacagtgagatgccgaagacctggtaataaaacagtgttgccagta
acaatcatggcagggttagtatttcactctcagaaatataataccaggttaaaacaagcgtggtgccacttccaa
ggagattggaaaggggcatggaaagaagtcagagaagaagtaaagaaagtgaaaaatcttacagaagtaagcata
gaaaatatacatctgagaaggatatggggagatccagaatcagcgaatttttggttcaattgtcaaggtgaattt
ttctattgtaagatggactggtttatcaattatctaaacaatcgaacagaagatgcagaaggtactaataggacc

tgtgacaaagggaagccaggaccaggaccatgtgttcagagaacttatgttgcctgccatatacgacaagtagta
aatgattggtacactgtctctaaaaaggtatatgctccaccaagggaaggtcatttggagtgtaactcatcagtc
acggcactatacgtggcaatagattataacaacaagtctggcccaataaatgtgaccctaagtcctcaggtacgc
agcatatgggcgtacgaactgggagactataaattagtagagataacaccaattggctttgctcctacagatgta
agaagatatactggccccacaagagaaaaaagggtgccattcgtgctagggtttctaggcttcttgggagctgct
ggaactgcaatgggcgcagcggcaacaacgctgacagtccagtctcggcatttgcttgctgggatattgcagcag
cagaagaacttgctggcggctgtggaacagcaacaacagttgttgaagctgaccatttggggtgtgaaaaacctc
aatgcccgcgtcacagctctcgagaagtacctagaggatcaggcacggctaaattcatggggatgtgcgtggaaa
caagtatgtcacaccacagtgccatggaagtataataacactcctaagtgggacaatatgacttggttggagtgg
gagagacaaattaatgccttggaaggcaacataactcaactattggaagaagcacaaaatcaggaatcaaagaat
ctggatctgtaccagaaattggatgattggtcagggttctggtcatggttctcactgtcaacttgg
>#17 LC114462 2229 nt
atgaacataaagtacataaattttctaatagttataggtataggtataggactagtgttaagtaaacagttatat
gtgacagtcttttatggagtaccagtatggaaaaacagcacagtacaggccttttgtatgacacctaccaataga
ttatgggcaacaactaattgcatcccagatgatcatgactatacggaagtgcctctgaatattacagagccattt
gaagcatgggctgacagaaaccctttggtagcacaggcagggagtaatattcacctgctgtttgagcagacactc
aaaccatgtgtaaaattaagcccattatgtattaagatgtcatgtgtacccttaaatggctccaagacagggtca
acaacggcaccaagtacaacacaaaccacaaagtcaacacaaaggccttgcaagcaatatgagaaaaacaacacg
ttgcaggcatgtaacgacaccattatagaaagagaaatggatgaagagcccgcttcaaattgcacctttgccatg
gcaggatatattagggaccagaaaaagaattattcagtagtatgggatgatagggaaatttattgtaaaaatgga
agcgacaatagctccaaggtgaaagagtgctatatgattcattgcaatgactctgtgataaaggaagcttgtgac
aagacctattgggatgaattaaggctgagatattgtgctccagcaggatatgctttgcttaaatgtaatgatcat
gattataatggctataagcaaaattgctctaatgtgtcagtagtacattgcacaggattaatgaacaccacagtt
agtacaggcctattgcttaatggtagttattcagaaaatagaactcagatatggcagaaacatagagtgaataat
gattcagtgattatcctctttaacaagttttataatcttacagtaacttgtaaaaggccagggaataagacagtc
ttgccagtcaccattatggcaggattagtcttccactctcagaagtataacatgaggttaagacaagcttggtgc
cacttccagggcaattggaaaggagcctggaaggaagtacgagaggaaatagtgaaattaccaaaagataggtat
caaggaaccaacaatacagagcagataaaattgctgcgacaggatggggatccagaagcagctaacttgtggttc
aattgccaaggagaatttttctattgtaaaatggattggtttctaaattacataaacaatgccacaactacgttt
gatggaaacaagtgtggtaataaaacaaagaaaggaaatgctccaggaccctgtgttcaaaggacatatgttgcc
tgccacataagatcagttgtaaatgattggtatacagtaagcaaattaacctatgccccgccaagagaagggcac
ttacaatgtacttccacggtaacgggtatgacagtagagctaaattacaacaataagaacgtaacaaatgtgacc
ctaagtccccaaatagaagggatttgggcagcagaattgggtcgatacaaattggtggagattaccccaattggc
tttgcacccacagaggtcagaagatacacaggggacatgagagacaaaaaagagtccctttcgtgctagggttc
ctaggcttcttaggagctgctggaactgcaatgggagcagcggcgacagccctgacggtccagtctcagcattta
cttgctgggatattgcagcagcaaaagaacttgctggcggctgtggagcagcaacaacagatgttgaagctgacc
atttggggtgtgaaaaacctcaatgcccgcgtcacagctctggagaagtacctagaggatcaggcacggctgaat
gcatgggggtgtgcatggaaacaggtgtgtcataccacggtaccatggaaatggaacaatactcctagttgggac
aatatgacctggctcgaatgggaaaggcaaattcaaaaactggaaggcaacataacagggcttctggaggaagca
agggcacaggaagagaaaaatttggatgcgtatcaaaaattaacctcatggtcagacttctggagttggtttgac
ttaagcaagtggtttaacatcttaaaaataggattcttaatagtaataggaattataggattaagactcttatat
agtgtatacctttgtatagctagggttaggcaggggctttccagtacctgcaa
>#18 KR862336 2256 nt
gaggagagaagaccattgcaaaaatgtgataataagtgctactgtaaaagatgttgttatcattgccagctttgc
tttctgcagaagggtttaggtgtgaatatgcatttgcaaaagctcctatctacattcttagtgttaggcttttta
gcatgggtaggaataggtaaacaatggatcacagtgttttacggagtaccagtatggaaaaacagttcagttcaa
gctttctgcatgacacccactaacagactatgggcaacaacaaactgtataccagatgatcatgattacacagaa
ataccсttgaacattacagaacсttttgaggcttggccagatggaaaccctttagtggctcaagcagaaagtaat
atacacttgttgtttgagcagacactcaaaccatgtgtgaaattatctcccttgtgcataaaaatgagctgtgtg
cctctaggatctagtaaagctggtcctactactactcctgctagtagtaccaccgccaaaaacctaccatgtgtg
aagaatgagactgatccacagctgagggattgtaatgcaacaatcatagaacaggaaatggaggaggaaccagca
tcaaattgtacatttgcaatggcaggatatgttagggatcagaagaaaaactactcagtagtatggagtgatgca
gaaatttactgtaagaacagtacctacagtgccaccagtaacaccaaagactgttacatgatccattgtaatgat
tctgttattaaggaagcatgtgataaaacttattgggatgagttgcaacttaggtattgtgctccagcaggatat
gctttgctgaaatgtaatgattatgattataatggatataagcaaaactgcagtaatgtttcagtagtacattgc

acaaagcttataaatacaacaattagtacaggcttattgcttaatggaagctattcagaaaataggacacagatt
tggcaggaacatggagtgagcaacgactcagtgataatccattttaacaaacattacaatctgacagtaacatgt
agaagaccaggaaataaaacggtcttaccagtaacaatcatggcaggcttggtatttcattctcaaaaatataat
acaaaattaagacaagcttggtgtcacttccagggcaattggaaaggagcctggaaggaagtaagagaagaaatt
gtaaaactaccaaaggaaagatatcagggaaccaataacacagagaatataaaattgttaaggcaagatggagat
ccagaagcagccaatttatggtttaattgtcagggagaattcttctattgtaaaatggattggttccttaattac
atcaacaatcagacagttacctttgatggaaatacgtgtaataatactaagactagaccgagaaagggagcgcct
ggaccttgtgtgcaaagaacatatgttgcctgccacattcgatctgtcgtaaatgattggtacaaagtggcaaaa
ttaacctatgctccaccaagagaaggtcatttgcaatgtacctccacggtgacgggtatgacagtagaactgaat
tacaattcaggaaatcggacaaatgtgacactcagccctcaaataagaggcatatgggcagcagaattgggtagg
tataaattagtggaaattactccaattggctttgcaccaacagatgtcaggcgatatgaaggaccccaaagacag
aaacgggtgcctttcgtgctaggattcctaggattcttgggagcagcaggaactgcaatgggagcagcggcgaca
gccctgacggtccagtcccagcatttacttgcggggatattgcagcagcagaaaaatctgctggcggctgtggag
cagcaacaacaaatgttgaagctgaccattggggtgtgaaaaacctcaatgcccgcgtcactgctcttgagaag
tacctagaggatcaggcacggttaaacgcctggggatgtgcatggaaacaagtctgtcatacaatagtcccgtgg
caatggcacaatgtaacaccaaggtgggataacatgacctggttggagtgggaaaggcagatagcaaacttggaa
ggcaacatatcgcgacaattagaagaggccagggctcaggaagagaaaaacatggatgcatatcaaaaactgtca
gattggtcaagcttctggagctggtttgacctgagtcgttggttcaactatctaaagataggcttcttagtgata
gtagga
>#19 KR862356 2241 nt
cagcgcctcctccagggcttgcatgagcctttgaaggcatgtactaacaaatgctattgcaagaactgctgttat
cattgtcagttgtgttttctgcaaaaagggttaggtgtgaatatgctcttgcttaagatctgtctcttaggctta
atagggatattagtttatagccaccaccaacaatatatcacagtattctatggggtaccagtatggaaaaatagc
acagtccaagccttctgcatgacacctactactagattatgggctacaaccaattgcataccagacgatcatgat
tatacagaggttcctcttaacataacggaaccctttgaggcatgggcagatagaaatccattagtggcacaagca
gaaagcaatatacacctgttgtttgagcagacactcaaaccatgtgtcaaattaagcccactctgcataaggatg
agttgtgtagaattaaaatcctcaagaccaacaaccacggcgtcaacaagtactgtaacagcaccactatctgac
ccatgccagagaaacgagagtgagactgtaattggaaagtgtaatgacacaataatagaaaaggagatggaggaa
gagccagcttctaattgcacctttgctatggctgggtatgtaagagatcaaaagaagaactactcagtagtctgg
aatgatgcagaaattatgtgtaagggaaatgggaccaacgaggccggaaaaagagaatgctatatgattcattgt
aatgattcagtaataaaggaggcttgtgaaaagacatactgggatgagttaagactgagatattgtgcacctgca
ggcttcgctctactcaaatgtaatgatatggactataatggatataagcagaattgctctaatgtatcagtggta
cattgcacaggcttgatgaataccacggttagcacaggcttattgcttaatggtagttattctgagaatagaaca
caaatatggcaaaagcatagagtaaataatgattcagtgatagtactcttcaataagttttataatctcacagtt
agatgtagaagacctggcaataagacagtattgccagtgaccattatggcaggtctggttttccactcccagaag
tataacacgaggctaagacaagcgtggtgtcatttccaaggcgattggaggggagcctggagggaggtaaaagag
cacatagttaaattaccaaaagacaggtataaggggacaaatgacacagaaaagatctatctgcagagacaattt
ggtgacccagaagcagcaaatctttggttcaattgtcaaggagaattcttttattgtaagatggattggtttctt
aattatctaaacaacctaacagtagatgcagatcataataagtgcaacaattcgccaaaaggaaaagcaccagga
ccctgtgttcagagaacatacgttgcctgccatatccgatctgtcataaacgattggtataccatctctaaaagg
acttatgctcctccaagagaaggtcacttggagtgtctttccacggtcactggtatgacagtagaacttaattat
atatccaacaatagaacaaatgttaccctatcccctcagataggggtatatgggcagccgaattggggagatac
aaattagttgaaattacgccaattggttttgcacccacggacgtcaggcggtatacaggagggcacgacagagca
aaacgtgtacctttcgtgctaggattcctaggcttcttaggggctgctggaactgcaatgggagcagcggcgaca
gccctgacggtccagtctcagcatttacttgctgggatattgcagcagcagaagaacttgctggcggcagtggag
gctcaacagcagatgttgaagctgaccatttggggtgttaaaaacctcaatgcccgcgtcacagcccttgagaag
tacctagaggatcaggcgcggttgaatgcctggggggtgcgcatggaaacaagtatgtcataccactgtaccttgg
caatggcaaaacaggaccccacaatgggacaacatgacttggttagaatgggaaagacaaatagcggatttggag
agcaacataacagggcaactggtagcggctagagaacaagaagagaaaaatttggatgcttatcaaaaacttacc
tcgtggtcagattttttggagctggtttgacctgtcaaaatggtttaatattctaaaattaggcttc
>#20 KR862363 2229 nt
gaagaaagaagacctttggaagcatgtttaataaatgctactgtaagaaatgttgttatcactgccagctttgc
tttttgcaaaagggattaggtatcaatatgactatagtacctgtactagtttatttaggaatagggcttttatta
gcaaaggagcagtatattacagtattctatggagtaccagtatggaagaatagttcagttcaagcattctgcatg
acacccaccacaagattatgggctactacaaattgtatcccagatgatcatgactatacagaggttcctttgaat

atcacagaaccttttgaagcatgggctgatagaaatcccttggtagcacaagcagggagcaacatacatctattg
tttgagcagacactcaaaccttgtgtgaaattgtccccctatgtataaaaatgtcctgtgtagagcttaactct
tctagaatcactacaaccagtactccatcagccactgacaagccctcagacccttgtggtggtaatagtgcgacc
ataggaagatgtaacaatactcttatagagcaagaaatggaggatgagccagcttctaattgtacctttgcaatg
gcagggtatgtgagagatcagaaaaagaattattcagtggtatggaatgatgcagaaattatgtgcaaaagcaag
aacagcagtgacaataagactaaagagtgctatatgatacactgcaatgactcagtgatcaaagaggcatgtgaa
aaaacgtactggatgagctgagattaagatactgtgctccagcaggatatgctttattaaaatgtaatgatgaa
gattataatggatataagagaaattgctcaaatgtgtcagtagtgcattgcacaggactgatgaataccacagtc
agtacaggcttactgcttaatggtagttatagtgcaaataagactgaaatatggcagaagcacagggttaataat
gattcagtaatcatactgttcaataaacattataacctgacagtgagatgcaggagaccagggaacaaaactgta
ttacctgtaaccataatggcaggattggtcttccactcccagaagtataacacgaggttaagacaagcatggtgt
cacttccaaggcaattggcgaggggcctggaaggaagttaaggagaaaatagtacagttaccaaaggacagatat
caaggaaccaatgatacaaaacaaatattttgcaaagacaatttggagatcctgaggcagcgaatctatggttt
aattgtcaaggagagttcttctattgtaagatggattggttcctaaactaccttaataaccttacagtagatgca
aatcacaataaatgcacaaacagcaccaagaagggacacgcaccaggaccttgtgtacagaggacctatgttgct
tgccatatccgaacagttattaatgattggtacacagtatcaaagagaacatatgccccaccaagagaaggacac
ttggagtgtttatctacagtcacagggatgacagtggaattgaattataattcaaagaacagaactaatgtgaca
ttgagtccccaaatagaaggcatttgggcagcagaattgggcagatataaattagtggaaataacaccaattggc
tttgcacccacggatgtcaggcgatatacgggaggacacgaaagacaaaaacgtgtgcctttcgtgctagggttc
ctaggattcttgggagctgctggtactgcaatgggagcagcggcgacagccctgacggtccagtctcagcattta
cttgctgggatattgcagcagcagaagaacttgctggcggcagtggaggctcaacagcaaatgttgaagctgacc
atttggggagtaaaaaacctcaatgcccgcgtcacagctctcgagaaatacctagaggatcaggcacggctaaac
gcctgggggtgcgcttggaaacaagtttgtcataccactgtcccctggacttggagtaacaggactccagagtgg
aacaacatgacttggcttgagtgggaaagacaaatagcggacctggaaagcaacataacaggacaattagtcgct
gccagggaacaagaggaaaagaatttagatgcatatcaaaaattgacctcgtggtcagacttctggagttggttt
gacttatctaaatggtttaatatccttaagttaggcttctttgtaatcatagga
>#21 M29975 2217 nt
cttgagacatgtacaaataaatgcttttgcaaaaaatgctgttatcattgccaattctgcttcttacggaaagga
ctaggtattacctatatgacaaagttcttaggaattttatagtattaggaatagggataggaatagggataagt
acaaaacagcagtggataacagtgttctatggagtaccagtatggaaaaacagctcagtccaagcttttttgcatg
acacctactactaggttgtgggcaactactaattgcataccagatgatcatgactatacagaagtaccactgaat
ataacagagccatttgaagcatgggcagacagaaatcccttagtagcacaagcaggaagtaacattcacctgctg
tttgaacagacattaaagccctgtgtaaagctatcacctctatgtatcaaaatgaattgtgtagagttaaaaggc
tccgcaacctctaccccagcaacctctactacggcaggaaccaaactaccctgtgttagaaataaaacagactcc
aacctacagtcatgcaacgacaccatcatagaaaaggagatgaatgacgaggcagcgtcaaactgcacctttgct
atggctgggtacattagggaccaaaagaagaattactcagtagtatggaatgatgcagaaatcttttgtaagcgt
agtacatcgcataatgggacaaaagagtgctatatgatccactgtaatgattcagttataaaggaagcttgtgat
aagacatattgggatgaattaagactaagatattgtgctccagcaggatacgctttgcttaaatgtaatgattgg
gattatgcaggatttaagccagaatgttctaatgtttcagtagtgcattgcacaactttaatgaatacaacagta
accactggtctgttattgaatggaagctattcagaaaatcgaacccagatctggcaaaaacatggagtgagcaat
gactcagtgttaatcttgctcaataagcattataacctgacagttacatgcaaaaggccagggaataagacagtc
ttgccagtaacgataatggcaggattagtcttccactcacagaagtataatacaagactaaggcaggcctggtgc
cacttccagggcaattggaaaggagcttggaaggaagtacaagaggaaatagtaaaattaccaaaagaacggtac
caaggcaccaatgatacaaacaaaatcttttttgcaaagacaatttggagacccagaagcagcaaatctatggttc
aactgtcaaggggaattcttctactgtaaaatggactggtttttaaattatctgaataatttaacagtggatgct
gatcataatcattgtaaaaacaacgcagggaaaggtcgaagtccaggtccctgtgtacagagaacttatgttgcc
tgccatatccgatctgtcataaatgattggtatactatatcaaagaaaacatatgctccaccaagagaaggacat
ttgcagtgcacgtccacagttactgggatgacagtagagctaaactataataaccagaacaggacaaatgtaaca
ttgagtccccagatagaaaccatctgggcggcagaattgggcagatacaaattggtagagattacaccaattgga
tttgcacccacagaagtcaggcgatacacgggaggccaagagaggcaaaaacgagtcccgttcgtgctagggttc
ctaggcttcttgggagctgctgggactgcaatgggagcagcggcgacagccctgacggtccagtctcagcattta
cttgctgggatattgcagcagcagaagaatctgctggcggctgtgggagctcaacagcagatgttgaagctgacc
atttggggtgtgaaaaacctcaatgcccgcgtcacagctcttgagaagtacctggcggatcaggcacggttaaac
gcttgggggtgcgcgtggaaacaagtatgtcatacaacagtaccctggacgtggaataatacaccagagtggaat
aatatgacctggttggagtgggaaaaacagatagaaggattggagggcaacataacaaaacaattggaacaggca

agggaacaagaggaaaagaatttggatgcttatcaaaagttgtcagactggtcgagtttttggtcttggttcgat
ttttcaaaatggctgaacattttaaagataggctttttggca
>#22 AF301156 2166 nt
atgcttagatatctctttataaacttgctagtattaggtatagtgttaagtaataaatgggtaacagtatatcaa
ggagtacctgcatgggaagaagcagatgtaaatgatcagcagttcttctgcttttcttcctccccagagattcaa
caggtgttggggtgtttaccacctccgcctggaaagccagtagaacaaaacatgccaaatgtaacagaagctttt
gatcttttaagaatagcttcagtggggaggtatggataataacacagactaccttagaacaaaggctaagacca
tgtgctaagttgacagcatactgtgctccaatgatttgcaccaaagtaaataggactgaaaatggcactagtaca
gtagccccaacaacaacaaataatagtgcttcagattgggatgagtcaaattggaaagaatatccgtggtataat
tgcagaatgaattcaacagctttcttacttaaagatagaaaggagttagaattaggattttcagtagaagattta
actgtgcttggaaataaaaatgacagtaatagtatcagggcaacaatgaaagattgtgcaaattatacagttacc
caagtatgtgatatgactatagtggatccagttaggacagggttctgtgcagctccaggatacatgcttttaaga
tgtgatgataaaaagtgggatgggacaggagcatgcaacaatgtgacagcagtgtcctgtacccatgagtttaat
ataacagtcatgtctcatgtattagtcaatgcctcaaaagagttgtctgattgggcgaaagatagagagggagtc
tggaagaatgactcagggacaattgagtattattggtttccaaaagatatagctttagggtgtataagaagagga
aatagttctcacagaaatctaaacactgccaatggagcaaaattctactatgagcttattccttattcaaaaggc
atttatggaaggtgtcagtttgtaccaatgactgggcagaataagaaaaacaaaacacaattcgcaatagaaatc
aaaaagaatttaacagcttggctagagcgcatttcacgcaaaaatataactatcactccaaggaatggaaataga
acaagtgatccagaggctactttcacctttgtgatttgtcataggttattcttttactgtaatgcatctagttta
tggaaacatgattccccagtcatgaactgtaccattagaaagctggtgaatagttgggtcactcatgccaggatc
ctgtatgggccgcctccaggaggtcatctacaatgtaactgggagaaacagccagttattgcgttcatgggcacc
atagaaggagacaatgatggtaatgggtgtgcttatccagcagcaccaaattttaagcatgcattaagtactttg
gaattgggaagatacaaattggtaaaaatgaggacaactacttatgtcccaacagatataaaaagatcagtgaat
gtaaattggcatcatgggagacaaaagagaggcatctttgcattctccatcttagccctcttgagtggggccggt
gctgcaatgggctcagcgtctgtggcgctgacgatccaggctcaatcccttaatggaagggctagtgccagcagc
aaccgaatgttgctgaaactggtcgagacgcagtctgcactgctacagctcacggtctgggggggttaaaaacctc
caggtacgagttgccactatagaaggttatttggaggaacaagcaaagcttgccagcattgggtgtgcaaacatg
caaatttgtagaactattgtgccctggaacaagacctggggagaggaggatccatggcagaacatgacctggaaa
cagtggcatgagcgagtaagaaattacacagatataatagaggcagacttagtagaggcatatgacttacaagag
gaaaatgaaaagaaattggcagaattaggggattggactaattggttttcaggctttggcctattcaacatcttt
aagtatgtactctatgctgcatatgttgtaggaggtttaataggacttagaattattatggtagtaatagcttgc
ataagaggagctttcagagttaagggatttcagcagataggcaggactaatgtgtcttcacagatc
>#23 AY159322 2343 nt
atgttaagatatcttaggtatatagtcttaggaataatagtaagtgtaatagtaggagaacaatgggtgacagtg
tactatggcacacccaaatggcataaggcaaggacacacctgttttgtgcaacagacaataattcattctgggtg
accacaagctgtgtccccagtctgttgcactatgaagaacagcatatccccaatataacagagaattttacaggc
cctatagaagagaatgaaatagtgacacaagcttggggagcaatttcttccatgatagatgcagtattaaaacca
tgtgtaaggttaacaccttattgtgtgaagatgaaatgtacagaaggtcaaaatgaaacagagcaagcaacagct
aagaccacaacacctgtacccacaacaacaacccccctccactaccactagctccagcacaaataagacaacaact
cctgtgttggttgttgagaaacaaaataatgagacaacaacacagcaaaatagagtatgtaaattcaacacgaca
ggattatgtagggactgcaaattggaaatagaggaaaactttagatatgaagatgtaacttgcacaaagcttaat
aaaactggtagtgctactaatagtactgaacctgaatatgagtgttacatgacttcatgtaacgcaacagtaata
acacaggaccgtaacaaagcttccacagatagaatgacatttaggttgtgtgcacccccagggtttgtcttacta
aaatgtaatgaaaaattgaacaagacaaaattatgtgggaatgtgtctgcagtgcagtgcacagcaccgctgcca
gccacaatctctacaatgtttggcttcaatggaactaagcatgattatgatgagcttattcagacaaacccacga
aaaggaaaagatgagtttcatgaccataaatatgtatatagagtggataaaaaatggggattacaggtaaggtgt
agaaggaagggaaatagatctatcatttccacaccaagttctacaggattgctgttctatcatgggttggagcca
ggaaagaatcttagaaagggcaagtgtcagttagaagggaaatggggacaggctttacatagtctatcattagag
ctaagaaagataaatgatagcatttacaaagacaaccacaatatgacatgtaaaagcagtaacaacaaaaagaac
acaacagggtgtcatcttaaaactataagtataagtgagtccaccgttaaaggcgaaccaggagctgaaactatt
atgctcctctgtggaggagaatatttcttttgtaattggactaaaatatggaaggcatggaatagcaaacaaagt
tcagtttggtacccttacatgtctcgcaatattagacaaatagtaggtgattggcataaagtaggcaagaaaatt
tatatgcctcctgtgtctgggtttaataatgaaataagatgtactaatgatgtgacagaaatgttctttgaggtc
caaaaaactgatgatgacaatggatatatcataaaatttataccacaagattggattcaaaatcaatacacagca
gtgggggctcattacaaattggtgaaggtggatccgataggttttgcccccacagacatacacagacatcatttg

cctaatacaaggcaaaaaagaggagcggtcttacttggaatgctcggcctcctaggtttggcaggttccgcgatg
ggctcagtggcggtggccctgactgtccagtcccagactttactgaatgggattgtggagcagcagaaggttctg
ctgagcctgatagatcagcactccgagttattaaaactaactatctggggtgtaaagaatcttcaggtccgcctc
acagccttggaggaatacgtagcggaccaatcaaggctctcggtatggggatgttcattctcccaagtttgtcat
actagtgtaaaatggcccaataatagtatagtgcccaattggacctcagagacatggctggaatgggacagaaga
gtaaacagcatagtgacaaatatgaccatagacttacagagggcatatgaattagaacagaggaacatctttgag
ttgcaaaaattaggggacttaaacttccatgggctaactgggttcgaccttacatggtggcttaagtatgttaag
ataggactgttagtagtagtagttatcataggattaagaatgttagcttgcttatggtcagtattagggaagttt
aggcagaagccttggaagccagagcaagtcaggagctggctgaaaaggtcgacgctttacatttggctgaagaac
ctccaagcagtaattgaa

>#24 AF367411 2178 nt

atgcttaggtacataatattaggaataattgtaggactagggttaggaaaccaatgggtgacagtatattatggc
acacctaaatggcataaggcagagacacacctgttctgtgcaacagaaaatgattccttatgggtaacaactagt
tgtgttcccagcttgttgcactatgaagagcaaccaattccgaacataacatggaatttcacaggtcccatggaa
gaaaatgaggtagtaatgcaagcatggggagctatctcctccatgatagacgcagtattaaaaccttgtgtgaaa
ctgaccccatactgtgtcaagatgcagtgtacaaaaggaaaagaccttgctacccctatacccactacatccacc
acaactaccacaactaccaccaagacggtggcaaatactactgagttggacatagacaccaataacactgaaact
acaacacaacagaacagggtctgtaaattcaatactacaggattatgtagggattgcaaattggagatagaagaa
aattttagatatgaagatgtaacttgtacaaatggcacaaataacacatattcttgttatatgacacaatgcaat
acatcagtaataacccaggactgtaataaagcatccacagatgaaataaaatttagactgtgtgcacctccagga
tatgttctcttgaggtgcctagaaagattgaatgtctctaaaaaatgtaccaatatcacagcagtacagtgtaca
cagccattgccagccaccacctcaactatgtgtggatctagtgggacaaaacatgattataatgaattaatacag
acaaatacaaagaaaggcaaagaggagtttcatgaccataagtatgtgtatagagtagatgagaagtacaactta
caagtagtatgtagaagaaaaggaaacagatctgtgatctcaacacctagtgcaacgggattgatcttttatagt
gggttagaaccagggaaaaatcttaagaaaggtatgtgtcagttgaagggacaatggggaatagccatgcatgat
ctagcaatagaattaagaaaaatagacagctccatatggagaaatgtaacaaagggctattgcaaggggttaaaa
agaaaggaaaatagaacgggttgtgcccttaaaacaataaaggtctctgactacaccacgaaaggcgaaccagga
gcagaaactattatgctcctctgtggaggagaatacttcttttgtaattggacaaaaaatttggagaacatggaat
gatcaaaattcatcagtatggtatccatggatgtcatgtaatattagacaaataatagatgattggcataaagta
ggaaagaagatttatatgcctcctgtatctggatttaataatgagataagatgttctaatgatgtaacagaaatg
ttctttgaggttcaaaagacagaagagggatacatcatcaaatttgtgccacaagatgaggtacaaaaccgattt
acagcggtaggagcacactatcaactagtaaaagtggacccaattggctttgctcctacagaagtggcaagatat
catctgccagaagctaggcagaagaggggagcggtcttgcttgggatgttcggcctcctaggtttggcaggttcc
acgatgggctcagtggcggtggccctgactgtccagtctcaggctttgttgaatgggattgtggagcagcagaag
gttctgctgagcctgatagatcagcactccgagttattaaaactaactatctggggtgtaaagaatcttcaggcc
cgccttacagccttggaggactatgtagctgatcaagcaagactatccatgtggggctgctcattcgcccaggta
tgccacacacatgtaccatggccaaatgactctataacaccaaattggacctcagagacatggctggaatgggat
aaaagagtaacagctctaactgacaatatgacagtaaacttacagaaggcatatgaattggagcaaaagaatata
tatgagctagaaaaattaggagattggacttcctgggctagctggttcgactttacgtggtggcttaaatatgtt
aagataggtttactaatagtaatagtgattatagtgttaaggattttagcttgcttatggtcagtcttaggaaag
ttt

>#25 AF328295 2379 nt

atgcttagacatttaatcacagcaatagtaataataggtataggtataggaaaacaatgggtaacagtatattat
ggcacacctaaatggcacccagcaagaacgcatctttttttgtgcaactgacaacaactccctttgggtaaccaca
agttgtgtaccaagcttattgcattatgaggagcaactcatccccaatataacagaaaattttacagtccctata
caagagaatgaagtggtaaggcaagcatggggagctatttcctctatgatagatgcagttctaaaaccgtgtgta
aaattgacaccatactgtgtcaagatgcaatgtacaaagggagaaaacagtagtaccagcactaccactcccatg
cccactaccaccactcccaccactccagcagtgacggggctggaggtgacggtacagaataatgagagtaccaca
caagaaaatagggtatgtaaatttaacacaacaggattatgtagggattgtaaaatagagattaaagagagcttt
agatatgaggatgtgacctgtactaaaaaggaaaatgacactgaaactgaagaatgttatatgacacactgtaat
acatcagtaataacacaggattgtaataaggcctcaacagacaaaatgacatttagattatgtgcccctccaggg
tacattctgctaagatgtcgtgagaaattgaataaaactaaattatgtgccaatgtctcagcagtacagtgtact
gacccaatgcctgctactatatcaactatgtttggctttaatggaacaaaacataattatgatgagttaattttg
gtgaaccctcaaaaggagtttcatgaccataaatatgtttatagggtgaacaaaaaatgggggttgaaggtggta
tgtgataggaagggaaacaggtctatagtttcagtcccaagtgcatcaggattaattttctatcatgggttagaa

ccaggcaggaatctgaaaaaaggaatgtgcaggttaataggacaatggggaagagctttgaacgcattatcacgt
gaattaaagaaagtaaatgcaagtatttatagaggttttaatgcaagtggcccttgcaggggggataaacaataga
ggagtggtaaacaaaacagggtgtgccttgaaaacaatagaagtaagtaactacaccaccattggcgaaccagga
gcagaaacaattatgatcctctgtggaggagaatatttcttttgtaattggacaagaatttggaaaacctggaat
aaccaaaactcaaatgtttggtatccatatatgtcatgtaaaatcagacagatagtggatgattggcataaagtg
ggaaggaagatttatatgcctcctgtgtctggatttaataatcatataagatgcactaatgatgtcacagagatg
tttttgaggtccaactagttgaaggaaaaagatacctaataaagtttctgccacaagatgaggtacaaaatcaa
ttcacagcagtgggagcacattataaattggtaaaagtggatccgataggattcgcccctactgatgtacacaga
tatcatctaccagatgcaaagcagaagagaggagcagtcttgcttggaatgctcggcctcttgggcttggcaggt
tccgcgatgggctcagtggcggtggccctgactgtccagtcccaggctttgttgaatgggattgtggagcagcag
aagattctgctgagcctgatagatcagcactccgagttattaaaactaactatctgggtgtaaaaaatcttcag
gcccgcctcacagccttggaggactatgtagcggaccaatcaagactagcagtatggggatgctcattctcccaa
gtgtgtcatactaatgttccgtggccaaatgaaagtataactcctaattggacctcagaaacatggctggaatgg
gatagaagagtgacagctatcactaacaatatgacaatcgatttacagagggcatatgaattggaacaaaagaat
atgtatgaattacaaaaattgggagatttgacttcatgggctagctggttcgacctcacgtggtggctcaaatat
gttaagataggaatattgataataatggtagtaataggacttagaattttagcttgtttatggtctacaataggt
aggtttggagactatctccgacctcgcaacctcagacagccagacgaagaaagagaaggagagctcaacttagaa
gaacagaacatcaagtcagagagctccaagaaagaatttggcagaccttggaggccagagcaaataaggaattgg
ttgaaggagtcaacaatttacatttggctgaagaatctgcaggcagtaattgaa
>#26 KM378564 2259 nt
atgattagacatatagttataggcttatgcattttaggattttaggattaagtataggaaaacattgggtaaca
gtgttttatggaacccccaagtggagaccagcatccacacatctgatctgtgctacagataatcattcattctgg
gtaactacaagttgcataccaagtctattgcattatgaagaaacacaaattgaaaatatagaagaaaattttact
gtccctatgacaaaaaatgaagtgataaaacaagcatggggtgcattatcatctatgatagatgcagtgcttaaa
ccatgtgtcaaaatcaatccttattgtgtaaaaatggaatgtactgatggcccaccacaaccaacaacaactaca
gcaactagtactagtactactagtgctgccccaaatacaacagcagatttggagatagacagaaataacacagaa
ccaaaaacagaaacaaatagagtatgtaagtacaatgtgacaggattgtgtagggactgtaaagaagagatcaca
gaaaattttaggtatgatgatgtagtttgtaaatgtggcagtagagctgaatcaccagaagagaacaactgtact
gaaagtagaatatgttatgttcaccattgcaactcaaccgtgattactcaggattgcaataaggcttctactgat
atgatgaaattcagattgtgtgctccaccaggatatatatttctaagatgtaatgagaagctaaataaaactaga
aagtgtagaaatataacagcagtgcagtgtactagacacatgcctgccacaatctctagcatgtttggcttcaat
ggcacaaagcatgatcaagatgagttaatagagacaaggggaagaccagactttattgatcataaatacgtgttt
agggttgataggaaatggaaccttagaattctctgtagaagaaaggaaataggagtatagtgtcaacgccctca
gccacagggttgttgttttatcatgggttagaaccaggaagaaatctaaaaagaggaatgtgtatatttcaggga
cactggggaatggctttagaaagcctagccaaagaattgaaaaaagtaaatgcatccatttggagaaatactaat
aagaccagccaatgtaacggctcaaaggaaagacaaacgcaacagggtgcctgttgaagcagcttagggtagat
aattacaccgccgagggagatctggcatcagagaacctcatgatgatgtgtggaggagaatatttcttctgcaat
gttactaaaatatggaaaacatggaataatagatcaagtagtgtgtggtatccatatgcatcctgccacataaaa
caaataatagatgattgggcaaaagttggcaggaaaatttatatgcctcctgtatcaggatttaataatgagata
agatgtacacaagaagtcacagaaatgttctttgaggttcaccgtttggacacaaatgtaaccgatgattctcat
tatcagattaaatttataccacaagatgaagtgcaaaatcagtccacagcagtaggagcacattacaaattggtc
aaggtggagccaattggctttgctccaactgacgtgcatagatacaacctgccggaacacaggcagaaaagaggt
gcagtcgtgcttggaatccttggcctcttgagcttggcaggttccacaatgggctcagtggcgacggccatgact
gtccagtcccaggctttgttgaatgggatagtggagcagcaaaagcaactgctgcgcctggtggaacagcaacag
gagttacttaagttaactatctggggcgtaaagaacctccaggcccgccttacagctcttgaggaatacgtagga
gaccaagcaaagttgagcctctggggatgctcctttgcacaagtatgtcacacaaatgtagagtggcctaatgaa
acagtgactccaaattggacctcggagacatggatggagtggcaaaaaagagtggatagtatatcaaacaacatt
actcttgatttacagaaggcatatgaacaagaacaaaagaatatctttgaattgcaaaaattaggagatttaaca
tcatgggctaactggtttgattttacttggtggtttaaatatattaagataggattctttgtagtgatagtaata
atagggttaagaatcttagcagctttatggagtacagtaggtaggtttaggcagggttatcgccctcttccttat
atctttaag
>#27 KM378563 2307 nt
atgattagacatatagttataggcttacgcattttaggattttaggaataagtataggaaaacattgggtaaca
gtgttctatggaacccccaagtggagaccagcatccacacatctgatctgtgctacagataatcattcattctgg
gtaactacaagttgcataccaagtttattacattatgaggaaacaaaaattgaaaatatagaggaaaattttact

gtccctatgacagaaaatgaagtgataaaacaagcatggggtgcgctgtcatctatgatagatgcagtgcttaag
ccatgtgttaaaatcaatccttactgtgtgaaaatgatatgtaatgataccaaagaaataccaactacaacaaca
gtagctacaacagtggctactactactagtactacagctccaaaaaatgctagtgttgccccaacaacagcaaat
ggcactttggatatagatacaaataatacagaaaaagtaacagaagcaaacagagtatgtaagtacaatgtgaca
ggattgtgcagggactgtaaggaagaaatcacagaaaattttaggtatgatgatgtagtttgtaaatgtggcaat
gtaactgaatcaccagaaaataataattgtaatggtacaagaacatgttatgtgaccagttgcaactcaacagtg
attacccaggattgtaataaggcctccacagatatgatgaagttcagattgtgtgctccaccagggtatatattg
ctaagatgtaatgaaaaactgaataaaagtagtaagtgtaaaaatataacagcagtgcagtgtactagacacatg
cctgccacaatctctagtatgtttggcttcaatggcacaaagcatgatcaagatgagttaatagaaacaagagga
agaccagaatttgtggatcataaatatgtgtttagggttaataagaaatggaacctcaaaatcatgtgtagaaga
aaaggaaacaggagtgtaatatctacaccctcagctacagggttgttgttttatcatgggttagaaccagggaaa
aatctaaacagaggaatgtgtaaatttgagggacaatggggaaaggctttagaaagcttagccaaagaattaaaa
gcagtaaatgcctccatttggagaaatactactattaaagggaaagaatgtaaacgtctcaaagaaaccaaagac
acaaaaacagttgggacagggtgcatgttgaagcagcttaaggtagataattacaccaccaaggggatctagca
tcagagaacctcatgatgttgtgtggaggagaatacttcttctgtaatatctctaaaatatggagaacatggaat
aatagatcaagtaatgtgtggtatccatatgcatcctgccacataaaacaaatagtagatgattgggcaagagtg
ggcaggaaaatttatatgcctcctgtatcaggatttaataatgaaataagatgtacacaagatgtcacagaaatg
ttctttgagatccaaaaattggagacaaatgaaatcaatgattccaactatcagattaaatttgtgccacaagat
gaagtgcaaaatcagttcacagcaataggagcgcattacaaattggtcaaggtggagccaattggctttgctcca
actgatgtacatagatacaacctgccggaacacaggcagaagagaggtgcagtcgtgcttgggatccttggcctc
ttgagcttggcaggttccacaatgggctcggtggcgacagccatgactgtccagtctcaggctttgttgtctggg
atagtggagcagcaaaagcaactgctgcgcctggtggaacagcaacaggagttactcaagctaactatctggggc
gtaaagaacctccaggcccgcctcacagctcttgaggaatacgtaggagaccaagcaaggttaagcctctgggga
tgctcctttgcacaagtgtgtcacacaaatgtggtgtggcctaatgagtcagtgactccaaattggacctcagag
acatggatggagtggcaaaaaagagtagatagtatatcaaacaacattactcttgatttacagaaggcatatgaa
caagagcaaaagaatatttttgagttacaaaaattaggggacttaacatcatgggctaactggtttgatttcact
tggtggtttaaatatattaagataggattctttatagtaatagcaataataggattaagaattttagcagcttta
tggagcacagtaggtaggtttaggcagggttatcgccctcttccttatgtctttaag
>#28 M27470 2289 nt
atgtctacaggaaacgtgtaccaggaactaataagaagatacctggtagtggtgaagaagctatacgaaggtaag
tatgaagtgtccaggtctttttcttatactatgtttagcctactagtaggtattataggaaaacaatatgtgaca
gtcttctatggagtaccagtatggaaggaagctaaaacacatttgatttgtgctacagataattcaagtctctgg
gtaaccactaattgcataccttcattgccagattatgatgaggtagaaattcctgatataaaggaaaattttaca
ggacttataagggaaaatcagatagtttatcaagcatggcatgctatgggaagtatgttagataccatacttaag
ccatgtgtaaagattaacccatattgtgttaagatgcaatgtcaggaaacagaaaatgtatcagcaacaacagct
aagcctataactacacctactactacatctacagttgcaagtagtacagagatttacttagatgtagataaaaat
aatacagaagaaaaggtagagaggaatcatgtatgtaggtataacataacaggactatgcaggattcgaaggaa
gaaatagtaacaaatttagaggggatgatgtgaaatgtgaaaataatacttgctatatgaatcattgtaatgag
tcagttaatacagaagactgtcagaagggacttttgataagatgtattttaggttgtgtgcctccaggatatgtc
atgttaagatataatgagaagttaaataataataaattgtgtagcaatatatcagcagtgcagtgtactcagcac
ttagtagccacagtaagtagctttttggctttaatggaactatgcataaggaaggagaattgatacccatagat
gataaatataggggcccagaggaatttcatcaaaggaagtttgtctataaggtgccaggaaaatatggcttaaag
atagaatgtcacagaaaggaaataggtcagtagtgagtactccatcagctacaggattattatttatcatggg
ttagaacctggaaagaatttaaagaaaggcatgtgcaccttcaaaggacgttgggggttagcactttggagtcta
gctaaagaactaaataaattaaatgactccatcaaagtgaaccagacctgtaaaaattttactagcactggagag
gagaacaaacaaaacacggacaagcaaaaggagtttgccaaatgcataaagactcttaagatagataattatact
acatcaggagatagagcagcagaaatgatgatgatgacatgtcaaggtgaaatgttcttctgtaatgtaacaaga
atcatgagggcatggaatgatcctaatgagaagaagtggtatccttatgcctcatgtcaaattaggcaaatagta
gatgactggatgcaagtaggaagaaagatatatttaccacctacatcaggatttaataatcacataaggtgtaca
catagggtaacagaaatgtactttgaaatgcaaaagatagatagtaatgaaacaaaaatgcaaattaaattcttg
cctcccagtgaaacctccaatcaatttgttgcttatggagctcattataaattagtcaaaataatgccaattggc
atagcacctacagatgtgaaaagacacactttacctgaacatcataaagagaagagaggagcagtaatacttggt
atccttggtctgctctcgctggcaggatccgcgatgggctcagtgtcggtggcactgactgtccaatctcagtct
ttggtgactgggatagtggaacaacaaaaacagttgttgaagctcatagagcaacagtctgaactcttaaaactc
accatatggggagtaaagaatttacagactcgcctgaccagtttggagaattatatcaaggaccaagctttgctg

tctcaatgggggtgttcatgggcacaggtgtgtcatacttctgtagagtggactaatacaagcatcactccaaat
tggacatcagaaacttggaaggaatgggagacaagaactgattatctgcaacaaaacattacagaaatgttaaaa
caggcatatgatcgagagcaaagaaacacatatgaattacagaagttaggagaccttacatcttgggcaagttgg
tttgactttacttggtgggttcaatacttaaaatggggagttttcttagtgttaggaattataggattaagaatt
ttgttagccttatggaatacaataagtaggtttaggcag
>#29 AF131870 2277 nt
atgcggtgccctgaaatattgataggtttctctttgctgttaggtagtatagcaatacagtatgtaacagtattc
tatgggactcctaagtgggaaccagcagtagtgccattaatatgtgcttcagctaataatagcttgtgggtgact
acttcatgcttgccagatttacaaacttatgcagaagtgcccataacagggttagaagagaattttacagaggga
ataagcaataatcaaatagtgcagcaagcatggcaagctatgacctctatggtagatgccattatgaagccttgt
gtaaagatcaatccctattgtgtcaaaatgaagtgtccaccgaaaccgacaactccctcctccaactcaacagtc
aaaagtagctgtgattattggacgaccaccacggctaagactacaactcagactacaagtagtaccagctccact
gctagtacaactactccaatgcctctagattggaattgtactgacacagagaacatagcggagagcaataaagta
tgtaaatataatgtgacagggctttgtagagattgtaaaacggaggtggaacaaaacttagggatacagaagtt
acttgcaatggtaatgacacatgttatatgacacattgcaatgattcaattattacacaagactgccataagggt
attatgcaaaatgcttacttcaggctttgtgcacctgcggggtatatgttattaagatgtgatgaaaaattaaat
gctactaagaaatgtaaaaatataactgctaccccttgtactaattatatgacaagtacagttagtagcttttt
ggctttaatggaactagacataaggaagatgagttaataccaatcaataacaaggtaggacaaggggctgaggga
gaatatgtatggaaagtagcagctaagtgggggttagtaattcagtgcataaggaaagggaataggtcacaagta
agtactataagtagtacaggactgctgttttattatgggctagagccaggttctaagttaagactggcacagtgt
aaatttgtaggacaatggggaagagcctttgccaccttagggaaactattgaggcaggtggaacctttagctaac
atgagtaatggctgtacatttgataatataacaaagacatgcagatttaccaatggtacagattttaaaaagatg
attaagtttaaaccaatggagaagcatggagcagatgcagcaacagagatgttgatgatgacttgtggagaagag
atgttcttttgtaatttaacaagaattttcaaagtatggaatgacacaacctccaataaatggtatccttggcc
aattgccacataaaaagtgtgatagatgattggcgtcagtgggcaagaagatttacttgcccccaacatcaggg
tttaataacagaataagatgtgcaaacagagtgacagaagcatggttcacactggaaagggtggaagactggaag
gtaaatggatcaaacatctcagttgtagccttccagccacctaccaacaccttaaatcaatttgtgagcacagga
gcacattacaaactagtaagaattaggccaattgggtttgctccaacagatgagcaccggtacgcacccaggaag
gaaaagagggcagcacctgttgccctaggagctttggcacttctcagtgctgcgggtactgcaatgggcttagta
tcgacgatactaactgtccaggcccaagcagtcttgcaagggatattgcagcagcagaagcagttgctggtgctt
gttgagaagcagcaagagctactaagactcaccatatggggagtgaagaatctccaagcacgcctgacagcccta
gaggagtatgtgcaagaccaaagccttttagcttcttgggggtgtcaatggaaacaagtatgccatactaatgtg
ccctggaattataatatcacgcccaattggactaaggacacctggatggaatgggataggcaagtaaaaatgtat
gatgataatataacagctcttctccaggaggcatatgtaacagagttagagaatcaaaacaagttcaaacagcta
caagaatttaacttctggagttggttggacctgtctcaatggtttctatacataaaatatgcagtcctgattata
ggcattataatagcagctagaattcttagttttatcatacaacagatctataggatgtgtcagggatatagggtg
ttgtccccttctgcttatgttgaacag
>#30 AF075269 2280 nt
atggcatgtccaggattaggaattctgcttttgcttcttggcataatatggggaaaacaatatgtaacagtattt
tatggggtgccaaattgggatgataatgtttcagtgcccttgatttgtgcttcagccaatactagcctctgggtc
acgacatcttgcttgccagatttgcaatcttatgcagaggtacctatctataatatttcagaaaactttacaata
ccagtaaaagacaatcaggttatacagcaagcctggtctgctatgaatgctatggtagacagtattatgaaacca
tgtgtaaaaatcaatccatattgtgtgagaatgcagtgtggggaagttacaaaaacaccaacaacaacaccaaaa
actactacacagatgccttgttttatcaacgaacaggtaacagttaagaacccggggaatgaaacaagattagag
gaagatcttaattgcacaagagggcttaatgagaccacagagaggaatgcagaatgccagtataatgtaacaggg
ttatgcagagactgcaggacggaaataaaacaaagctttagatatgatgatgtaacgtgctcaggagagagggag
aacagaacctgttacatgactcattgtaatgattcaataataacacaagattgtaataaggggggtaatgcaaaat
gcttattttaggctttgtgctccagcaggctatatgctattgagatgcaatgaacaattaaactttagtaaaaaa
tgtgaaaatataacagctacaccatgtactggatatatgcttagtagtgtaagtagtttctttggctttaatgga
actaatcataccagggatgagcttattccactaactccaaataagatggaagatctaaatggtgcaaagtttgtg
tataaagtagcaggaaaatggggtttaattattagatgcataagaaaaggaaatagatcagaagtatccacaatt
agctctacaggatatttattttattatggcctagaacatggcagcagattaagattagcacagtgtaaatttgaa
ggccaatggggaagaatgtttaataatctaggcaaaatgctaaaggagttgaatgcagaagccatgaattataca
gaagggacagggacatgtgattctaagaaaacaacttgtggacggaaattaaaaggcttaccaatagctaatatg
actaggcatggagcagacttagcaacagagatgttaatgcatacttgtggagaggaaatgttcttttgtaatgta

actaggatattccaagaatggaacaataaaaattcagataaatggtatccttgggccaactgccacataaaaagc
atcattgatgattgggctacaataggaaagaagatttatctaccacctacttcaggtttcaacaatagaatcaga
tgtactcatagagtaacagaaatgttctttgagatggaaaaatgggaaccacatgaggacttaggaggcaaccta
agtataaagttcttgcctccatcatgggaaactaatcaatttgtggcagaagggtctaaatataaattgataaaa
ttgaatccaattgggtttgctcctacagatgagcacaggtacgcacccaggggaaggcaaaccagagcagcgcct
ctggctttaggagccttgggacttctcagtgctgcaggcactgcaatgggcttagtatcgacgatactaactgtc
caggcccaagcagtcttgcaagggatattgcagcagcagaagcagttgctggtgcttgttgagaaacagcaagaa
ctgttaagactcaccatatgggagtgaagaatctccaagcacgcctgacagcccttgaggagtatgtgaaacat
caagcgctccttgcctcttgggggtgtcaatggaaacaggtttgtcatactaatgtggagtggacatataatatc
acacccaattggaccaaggatacctggagagaatgggaatcaaaggtagcaatttatgacaagaacattacttct
ctgttacaagaagcatacaccacagagctagagaatcagaataaatttaagaagttacaagaatttaacttttgg
agttggttggatatctcacactggtttacttatgttaagtatgcagtgttaattatacttgtaatcatagggtta
agagtattgagctttataatacaaaatgtagtaaaaatgtgtagggggtatagggtgctctccccctctgtttat
attgaacaggactacaagtgggagaaagaa
>#31 AF188116 2295 nt
atggcatgtccaggtaacatattgctattgctgctctgcttgggactaacagcaggagaaaaggagaaacaatat
gtaacagtattctatggagtaccaaaatgggaagatgctgtagtacctttaatctgtgcttcagctaataatagc
ttgtgggtcactacatcttgtctcccagatctgcaatcttatgctcagatccctattcataatattactctaaat
tttactcaagaaattaaggacaatcaaataatacagcaggcatggggagctatgacgagcatggtggatgctatt
atgaagccatgtacaaaaataaatccttattgtgttcgcatgaaatgtacaggggaaggactaggtaagaagaga
acaacaacaacaacaacaccaacaacaacacccatgccttgctttataacagaatctactactactactacaaag
agttcagggactgaacaaccatctttaatagaagatacaaattgtacacgatggttaaatgagactacagaaatt
aatagggaatgttcatataatgtgacagggctttgtagagattgcaaggaagagataaagcaaaattttaggtat
gatgagacaacatgtaatgagaatggtacttgctacatgacccattgcaatgatagtattattctgcaagactgc
aataagggagttatgtcaaatgcttattttaggctttgtgcaccagctggctacatgcttttaaaatgcaatgaa
aagctaaacttttcagctaattgtactaatataacagctacaccatgcacagattacatgataagttcagttagt
agcttctttggctttaatggaaccaatcatacagaagacgagttaattccactaactccaaaaaagatgggagat
cttaatggagctaaatttgtttacaaggtagcaggaaaatggggactaatcattagatgtataagaaaaggaaat
aggtcagaagtctccacaatcagttcaacaggacttctgttttactatgggttagaacatgggtctagactaaga
ttggcacaatgcaaatttgagggtcaatggggaagaatgtttaacaatttaggaaggatgttaaagaagcttaat
gagactgctatgaattataaagaaggaaattgttccacaccaggaaaaccatgtggtagacaattaaagggtcta
cccatagcaaatatgacgagaagaggagtagatctcgcaacagagatgctcatgcacacttgtggtgaggaaatg
ttcttttgtaatgtcacaagaatcttccaggaatggaacaataaaaactcagataaatggtatccctgggccaat
tgccacataaaatcaattatagatgattgggcaactgttgggaagaaaatttatcttcctccaacatcaggattt
aacaatagaataagatgtactcacagagtgacagaaatgtggtttgaaatggaaaaatgggaaccacatgaagac
ttaggaggaaatctgtctattaagtttttgcctccctcatgggagacaaatcaatttgtggcaacaggagctcat
tacaaattgatcagactgagaccaattggctttgctccaacatcagagcatcggtacgcacccaggggaaggcaa
acaagagcagctccactagctctaggtgccttgggacttctcagtgctgcaggtactgcaatgggcttagtatcg
acgatactaactgtccaggcccaagtagtaatacaagggatattgcagcagcagaagcagttgctggtgcttgtt
gagaaacagcaagagctgctaagactcaccatctggggagtgaagaatctccaagcacgcctgacagctattgag
gagtacttgaaagaccaagcactacttgcctcttgggggtgtcagtggaaacaagtgtgtcatactaatgtacca
tggaattataatgttactcctaattggacaagggatacctggatagaatgggaaagacaagttggatctttagaa
gcaaacatcaccacactgttgcaagaggcttacacaacagagttagagaataggaataactttaaaagttacaa
gattttaatttttggagttggatggacctcactacttggttccaatatattaagtatgcagtgcttataatcata
ggtataataatactaagaattctaagctttatcatacaaagtgtagtgaagatgtgtaggggctatagggttctc
gccccctctgcttatgttgaacaggacttcaagtgggagaacgga
>#32 AF188115 2286 nt
atggactgtcttaagctcctacttttcttgctctttttaggagcacaaggggaggcaaagaagcagtatgtaaca
gtattttatggggtgcctaagtgggaagatgctgtagtgccattaatttgtgcttcagctaataatagcttatgg
gtcacaacatcatgcctaccagatttacagtcttatgcacagattcctattcacaacataagtttaaatttcact
caagagataaaagataatcaaatcatacagcaagcatggagtgctatgaccagtatggtggatactatcatgaag
ccttgtgtaaagataaatccatactgtgtgagaatgaaatgtacagggggaggaataggaccaaaaacaacacca
tcaccaataggaaaaacaacaacacccatgccttgctttatagcagaaacaaccacaaccaccaccaaagggaat
acaactatggaaagtttaatagaggacacaaattgtactagatggttaaatgagactactgagattaatagagaa
tgtaaatataatgtaacaggactatgtagggactgtaaagaagaaataaagcagagttttaggtatgatgaga ca

acctgtgataagaatggaacctgttatatgacccactgtaatgatagtattattcagcaagattgtaataaggga
gtaatatctaatgcttattttaggctttgtgcaccagcaggctatatgcttttaaaatgcaatgaaaaattgaat
ttctcagctaattgtaccaatataacagcaactccttgcacagactatatgattagtacagtgagtagtttcttt
ggctttaatggaaccaatcatacagaggataagttaattccactaactccaaaaaagatgggaaatctcctggac
gcgaagtttgtatataaagtatcaggaaaatggggactcattatcagatgtattaggaaaggaaacaggtcagaa
gtctctaccatcagttcaacaggacttctgttttactatggcttggaacatggtagcaggctgagattggctcag
tgtaaatttgaaggacaatggggaaggatgtttaataatttagggaaaatgcttcaaaagcttaatgcaacagca
atgaattatacaaatggacaatgtcctgagggaaaacagccttgtggtagacatctaaagggattacctatagca
aatatgacaagacgtggggaaggtcttgcaacagagatgttgatgcatacatgtggagaggaaatgtttttctgc
aatataacaagaatactccaagaatggaataataaaaactcagataaatggtatccctgggccaattgccacata
aaatcagtcatagatgattgggcaactgtagggaagaaaatctatatccctccaacatcaggctttaataacaga
ataagatgtactcatagagtaacagaaatgtggtttgaactggaaaagtgggagccagaggaaaacctgggggga
aatttgtctgtcagattcttgcctccctcatgggagacaaatcaatttgtggcaacgggagctcattataaatta
ataaggattagaccaattggctttgctccaacagctgagcaccagtacgcacccagtagtaagcagaagagagca
gcaccgctggctctaggtgccttgggacttctcagtgctgcaggtactgcaatgggcttagtatcgacgatacta
actgtccaggcccaagtagtaatacaagggatattgcagcagcagaagcagttgctggtgcttgttgagaaacag
caagagctcttaagactcaccatatggggagtgaagaatctccaagcacgcctgacagctattgaggagtaccta
aaggaccaagctctgcttgcttcctggggggtgtcaatggaaacaaatatgtcatactaatgtggaatggaattat
aatataactcctaattggacaagggatacctggattgagtgggacagacgagttggagtattagaagcaaatatc
tccaccctgttgcaagaagcctataccacagagttagaaaatagaaatgccttcaagaagctacaggagtttaac
ttttggagttggttggatattttatcatggttccaatatattaagtatgcagtactaataataataggtattata
gtgttaagaatagtaagctttattgtgcagaatatagtaaaaatgtgtaggggatatagggttcttgccccctct
gcttatgttgaacaggactacaagtgggagaaagga
>#33 AY523867 2172 nt
atgaaacttttaatatcataagtgtagtgttaggtatagtattaagtttaggcatagtagctaggttagaacaa
tatgtcacagtattttatggagttcccaattgggaagatgctcaagtacctatgttttgtgcaacccccccatacg
ggaggttgggcaacaaagaactgtgtaccatcttctgaacagatagaagtcagagttaatattagtggggaatat
ttcactgcgtggaattcttcccatggcataagacaacagcttttgcaggacatgtctgatcttttcctgcaggct
gataggccttgtgtaaaattatctcctatgtgtatcagaacgttatgtgtagaggacaaggacagtagtagtggt
aatcttaccacaacaatcagtccaacaacaacaacaaacaaagcagcaacaccacctgcatggtgggggacaac
tctacagaaactaggtacaattgttcatttaacatgacaggggttttaaggataagaaacatcagtataatgca
ttcttttacaagtcagatataatgagagaggaaggaaatgaaagttattactatttacagcattgtaatactagt
gtaatgcgcaatgcttgtgaaaaacagacttttcgcccatttcccattcaatactgtgctccaccgggatactca
ctgttgaaatgtaatgatacagaatttgaaggagatgctgagtgtcaaaatgtaacagcagtttcatgcacacat
cctttcaacactttggctagtacatggtttcagttaaatggcacctataaagcaaaagacaaagttaggtttatt
agacaaaaggataaaaatgaatcagtgattatactagtgccagaaaaattaggattacaactggtgtgtgaaaga
ccaggaaatgagtctataaaaaacatccaattggcagcaggatttttcttgccagtgattcaaggagattgaaa
acagggaaagctgcgaaaagagcattttgcaaagtcaaaggagaatggggaaagtttttgagcaggtccataat
gagagtataaaggtgtggaaaaatgtgacctcaacatcatggaggtcgcaaccacaaggagacctggaagtgaga
acacattggtttcaatgtggcggagaatttttttactgcaatgtttcaaagatttttgcaaatgtgtcaaatggc
tatgctaatccaagcaattatgcaaaaaatttatatctctcgtgtgccatacgacagattataaactattgggg
tatgtaaccaagctaatgtatttgccacctacagaagggcacattaagtgcacttctaatattactgctgtatta
acagatatagagtactatccaaattcacagttgaattttgcacctactgctaatgtggaagatgtgtggagagct
gatttattcaattataaattgattcggattaaacctattggctttgcgcctacaagtcaaaggcgctatgagctg
cctacgaaacagaaaagagcagcaccattggcacttgggttcctgggattactctcggcagcgggtactgcaatg
ggagcgctgcgacagcgcttacactccagtcccagactttgttggctgggatagtgcagcagcagcaaaagttg
ctggaggctgtagaggcacaacagcacctcttaggcttaacggtctggggtgtgaaaaaccttaacgcccgactc
actgctctagagacgtatctcagagaccaagccattatgtcaaattggggatgcgcatttaagcaaatttgtcac
acagcagtaacgtggcagcaagcatgtggcaataactctcgttgcccaacaccccaatgggaaaacatgacatgg
catacatgggaacggcaggtagataatttgacagaccacatagataatttactgagagaagctcaggagcagcaa
gagaagaatgtgcatgatcttactaaattacaagaatgggattccctgtggagctggtttgatctgtcgaaatgg
ttccagtaccttaagataggattctttgccatagcagctatagtgatattaagagtacttagttttgcttggggc
atagtaagaaatatgttgggaggctattctcctcttttgcaggacttgtaattgcctggggattcatcagt
>#34 AY655744 2166 nt

atgaaatacttagcagttatttttgcttagcttacttaggaaggtttataatagggaaccagtatgtcaccatc
tattatggagtaccagtgtggaaaaatgcaacacccccattgttttgtgccaccatggcaagggaccaatggtca
acatcccaatgcgtcccatcagatcctcagaatctcgaggtaccagtgaatctcactgagtacttcgatgtgtat
aaaaattacatggtggaccacatagcagaagacatggatagcctctttttgacagccacaagaccatgtactaag
ttaacaccaatatgtgtaaggatgaagtgtgaggacataacaaagaaccttacaatcagtgccaataccacaaca
ccagccccgacggccatgagcaatagcaatatgccgtggtggggagacaatacaacagaaccagtatataattgt
acatggaacatgacaggagcatttagggataagaggtatacctactttctcactggtacctagcagacctaatg
aaggaaccaggcaatggatccttctattatgccacaggatgcaatgtgtcagttatagcacaagcttgtgaaaaa
actcactaccaggcatttcctatacagtactgtgctccagcaggctttgccctgataaagtgtaatgatagccca
tggacaggtagaggaaagtgtcacaacatctcagcagtacactgtacagaccctatcaccacaatagcagctacg
tggttaattctcaatgggacaaaggaaaatcacacacaggtcatacatcaggctccaaagaatgaaagtgctata
atcctcctaggaaaggaacacaacctcacaatcacatgcataagaccaggaaataggacaataaaggacttacag
atagcagcaggactaatgtttcacagccagataatagcagggaagaacctcaacaggcctattgtaagctagag
ggcaattggactaaagccatggaatcggccaatgaagaaatagcaaaaaggtacaaggagcatatcaacaacaca
gataaagtgaacatcacatggacggcagtcaggagaggagacctagaggtacaacttttctggcatgcatgtcag
ggagaattcttctattgcaacataagttctatgttcctgaatacttcctatgagtataactacacatcaaaccaa
ggcaaattacaccaaggggtcataggaaagaagccgaggtcgagtgataggaccaagttctacatgtcatgccaa
ctgaggcaaatgatcaacaggtggagcagggtggagaagttaatgtacctcccacccagggaaggacacctgcag
tgcaaaagtaacatcacaggattgttggtggatatcaaccaccaccgcggctctagtgacataacagtagagccc
tcgtcacagatacgtgactcgtggcgagcagcagtttcacggtacaaggtgatagagatcgtgccttttgggtat
gcgccgacaaatatcagaaggtacgatgggcccgctaacaagcaaaaacgagcggccccgctagccttaggcttc
ataggatttctcagcacagcaggcactgccatgggcgcagtggcgacagccctgacggtgcagtctcgctctttg
ctgagtgggatagtgcagcagcaggaacacctgctgagggcaattgagcatcagcaacacctgttacaattaaca
gtgtggggcatcaagaatctcaatgcccgcctgaccgcgcttgagaaatacttagaggaccaggctaagctaaat
tcatgggggtgcgcatggaaacagatctgctacacctcggtaccgtggaacaaaacatggtcgaattacacagat
cctcaatggcagaatatgacatggcaagaatgggagatgaaagtagacaatcacacagggctcatatcacagttg
ttacaagaagcacaagagcaacaggaaagaaatgtccatgagttgcaaaagttaaatgactgggacagtctctgg
agctggttcaatttgtctgcctggtttagatggcttaggatagcagtaatagtagtagctagtcttatcttactt
aggattgtaatgtacatagttaatgtgctcaagttgctcaggcagggctacttacctcttccgttt
>#35 AY523865 2232 nt
atgaggcttatacagcttttgctacttattttagggttaatagtgataggtacacaggggaaacagaaaaagcaa
caatacttgacagtatattatggagtaccagtctgggtagatgccaaagttgatttgttctgtacagccaactca
tcagagtcaggatgggcagtgacagcatgcctgccacatgccttagtgagagaagaagtcccaatgccaaatgtg
acccaaaactttaatgcttttgataaccccatagaggagcagctatggcaggatatgactagtttgtacaaacag
tcattcaaaccatgtgtaaagctaacaccatattgtgttagcatgcagtgcataaaaacatcaacaaatccaaca
ccaacaaacacatcaacaacaacaacaacaatagcaacaacaacaaaaacaactacagattggtcaggggaaaac
ataaccatgacacaatattggaattgttcatttaatgtctcaggtccttatagagataagaaagaaaagagctca
gcagtatggctagaggatgatatccagtgggcggataataaagatggtagtgggaacagaacagggtacatgaaa
cactgtaatgactctgtcatcacccagtcttgtgaaactagccgctttaaccttttaagatcaggtactgtgca
ccagctggatatggtctactaagatgtgatgataaaaattttaatggtacagggttatgcaacaacgtgactgca
gtggcatgtacaaatctcatacacaccatggcatccacatgggtacaatttaatgggtctgatgaggagagggca
gaagagcttcatataataagaaaagaggtaaaaggtgaggtgcagaatggaagcattactataagagtgcccgct
aaatacaacttgacattaacatgtgtaaggccaggaaataagacctaccgagccatacacatggctacaggtctg
tcctttatactacctttatacaaaggctgaggataaagagagcacattgtagactaaacggaagctgggcaaac
gcaacaaaggaaatgcgacaaaagatattggaaatatttggaaaggcaaataggacaaataatctgacaatacac
tacccaaaaggagatagggaagtacaaagtgtctggtttcagtgtcatggagaattcttctattgtaacatttcc
aaagccctagatctcctactcctacaaaataacactagaaatagcacttggtctgacaagtggttgatgccttgt
aggataaatcagttagtaaacacttggtatacggtagggcaacatatctatctacctccgaaagaaggagagttg
aagtgcagctcacacatttctgcatttgtttttgatgtagatcactataatgggagtataacccttacaccatca
gcagacatcagggcagtatggagagcagacctatttaaatataaaatcatagaagtaaaacctataggattcgct
ccctccgcagtgagaaggtacgagggaccggagtcggtaagacacaagagggcagcaggaattgcttttggtctg
gtagcatttctcagtacagcaggtgctgctatgggcgcagcgtcaacagcgctgacggtacagtctcgctctttg
ctgagtgggatagtgcagcaacagcaagagctgttgaaggctgtggaggctcacgggcagctgttaacattaaca
gcctggggagtgagaaacctcaacacccgcctgactgctatagagaagtacttaaaagaccaagcaaaattaaat
gaatggggctgcgccttcaaacagatctgtcataccacggtcccttggaataactctctggaggatccagactgg

gacaatatgacctggcaggagtgggaaatgaaggtagcaaattatacagatgagtgggaaggagctttacagaga
gcacaagagcagcaggagaggaatgtacatgccctacaatcactacaagattgggactctttatggaattggttt
gacctgtccagatggttctggtggattagattagtagtatacataatagctgctttaatattgctaagaatagct
atgtttggagtgaatattggaagtagaggctttgaattactgcaagaaggaatatatagactgtcatttgctctt
tggcaaagactgagactcttgggggaacgctttagagtgcttgggggtacttacag
>#36 AY523866 2175 nt
atgcttagcttattaataataatagcaagtctgaccctggtaggggcagcaccaacaaagcaacaatgggtgaca
gtatattatggagtgcccgtatggcacaatgccactgttggcttattctgtacagctaatagttcagaatcagga
tgggctgtgacagcatgcttgccacatgcaatggttagagaacaagtccctatgcctaatgtgtcccagtatttt
gactctttttcaaatgttatggaacaacaattatgggacgacatgactagcttatacagacaatcttttaaaccc
tgtgtaaagttaaccccatattgtgtgcggatgaactgtagtagtacaacaacaacaacaacagcagcaacaaca
acaataaaaacaacaactgactggtcaggagagaataagacagtgacacagtattggaattgtaccttcaatgtt
acagggccatatagggacaagaaggaaatgcaaagtgcagtgtggttagaggatgacatacagtgggagacagaa
aaccaaacaaatactaacaaaagtataagacatgggtatatgaaccactgcaacaattcagttataacacagtcc
tgtgaaaccagtcgctttaagccttttaagataagatactgtgcaccagctggatatggtctgttaagatgtgat
gacagaaactttaatggtaccggcttgtgtaccaatgtgactgcagtggcatgtacaaatctcatacataccatg
gcatcaacatggatgcaattcaatggttcagatgaagaaagggcagaagacatacatatcataaggaagggcctg
gaaaataagaccattactgtcagaatcccaccaaagtataatctatctttggattgtgttaggccagggaacaag
acgtatagagctgttcatatggccacagggctatccttttatacaaccttcataccaaggctgaggataaagaga
gcacattgtaggatgagaggtaattggacaggagcagttaaggagataaggagcagattaaaggagatatataat
gtgacaaaaataacagtacactatccaggaggtgatagggaggtacaaaacacgtggtttcaatgccatggagag
ttcttttattgtaatgtttccaaagcatttaatctgctcatgaataaaactaataacataacctggagtaataac
actttgatgccttgtaagattaatcaattggtaaacacatggttcaaggtaggacaacatgtatatttgcctcca
aaggaaggagaattgaagtgtcattcttctgtttctgcacttgtttttgaggtagctcattatgggcaaaacatg
aacataacaccctcctcagatattaaggcagtttggagggcagatctatacaagtataagatcatagaagttaaa
cccatcgggatggcccctacaagtataagaaggtacgagggaccggagtctgcaagacaaaaaagggcggcgga
attgcttttggtctagtggcttttctgagtacagcaggtgctgctatgggcgcagcgtcaacagcgctgacggta
cagtcccgctctttgctgagtgggatagtgcagcaacagcaagagctgttgaaggctgtggaggctcacgggcat
ctgttgtcactaacagcatggggagtgagaaacctcaacacccgcctgactgctatagaaaagtacctaaaagat
cagagcaagttaaatgaatggggatgtgcctttaaacaaatctgtcacactacggtcccttggaaccatacatgg
ggagaacctgattggaataatatgacctggcaagagtgggaaaggaaagtagcaaactatacagatgaatgggaa
ggagccttacagagagcacaggaacaacaagagagaaatgtgcatgccttacagtcactgactgattgggattca
ttatggaattggtttgacctatccagatggttctggtggattagattagtagtgtatataatagcagctttgata
ttgcttaggatagcaatgtttggggttaatatcgggactaagagaggctttgagctcctgcaagaaacaatatat
agactttcatttgcattttggcaaagactgagactcttgggggaacatttaagagtggtctggggttattgtcaa
>#37 FJ919724 2127 nt
atgcttgctttcatattagctattatagtaattatcttaggacaaaagacagaacaggttagccagcaacaacag
tatgtgacagtattttatggggttccagtttggaaaaatgcctcagtgggattgttttgcacagcaaatggttca
gagtcaggatgggcagtgacagcctgcctaccacactccgtgataagagaacaggtacacatgccaaatgtatca
cagtactttgatgcattcaagaatcccatagaagaccaattatgggaagatatgacagggttatataaacaaaca
tacagaccctgtgtaaagttaacaccttattgtgtaaagatgcggtgtaagactgaaacaaccctaacatcaaac
ctaacaactccaaagacaacaactgattggagtggagaaaacaacaccatgtcacaatattggaattgctctttc
aatgtctcaggtccatatagagataagaaagaagagggacatgccatgtggctaacagatgatctggaatgggca
aatggaaatgagacaggggatactaggggagcttatatgaggtattgcaacaggtctgtaataactcaagcctgt
gacaaaagtcactttaaacctttcaaaataaggtactgtgcaccagctggatatggtctaatgagatgtgatgac
aaaaatttcaatggtacaggactttgtaccaatgtgtctgcagtggcatgtacaaacttaatacacacaatggca
tccacctggttacagttcaatgggtcagatgaggaaagagcagaagaaattcatgtcataagaaagggtcttgaa
aataagacaataacaattaggattcctcccgagtacaacctccgtttagactgtgtccgtccaggtaataagaca
tatagagcaactcacatggccacaggattatcattttacacaacctttgtagaaaggctaaggatcaaaagagca
cattgcagaatgcgaggtaattggagtggcgcagtttctgacataaaacaagtattaaagggcatatataatgtt
actaacataacaatgcattacccaggaggagatagagaggtacaaagggtatggttccaatgccatggagaatat
ttttattgcaatgtcagtaaggcctatcaacttttcaccaatgacaccataacatggagtaacaacactttgatg
ccctgcaaaattaatcaattggtaaacacctggtacaagatagggcagcatatatacttgccaccaagagaaggg
gagttgaggtgtgactcacatgtttctgcaataatttttgatgtggctcactatggacacatgttgaatttgacc
ccatcatcagatattaaggcagtgtggagagcagatctgtataagtacaaaatcatagaagtgcagccaataggc

tttgcacctactgctgtcagacgctacgagggaccagagtctgtgagacaaaaagagcagtgggaatggcttta
ggtctagtggcatttctcagcacagcaggtgctgctatgggcgcggcgtccacagcgctgacggtacagtctcgc
tctttgctgagcgggatagtgcagcagcaacaagagttgctgaaggctgttgaggctcacgggcacctgttaaca
ttgacagcgtggggagtgaaaaacctcaatacccgcctgaccgcgcttgagaaatacctaaaggaccaaagcaag
ttgaatgagtggggatgtgcttttaaacagatctgccacaccacagtgccttggaatggatcaataggacaacca
gattggaataacatgacatggcaggaatgggaacagaaggtagcaaactacacagatatatgggaagaagcatta
cagaaagcacaggaaaaacaggagcagaacgtacatgccctacagtcactacaagattgggactctttgtggaat
tggtttgacctatccaggtggttctggtggattagaatagtggtgtatgtaatagcaggattagtattgcttaga
ataataatgtttgtagtaaatataattagtctgagctttgctatttggactttgctacaaagacttggggaacaa
ctaagagtactttggggatacttgcaa
>#38 AF208027 2250 nt
atggcatatcttggcaatcagctgcttatcgcaaccttrctcttaagtacttatgggttctattgtaccaaatat
gtcactgtttttatggcatacccgcatggaggaacgcgagcgtacctctcttctgtgcaaccaaaaatagggac
acatggggcactacacagtgccttcctgataatgatgattacagtgaactagcattgaatgtaacagaagctttt
gatgcctgggataacacagtaacagaacaggcagtagaagatgtttggaatctgtttgagacatctacaaaacct
tgtgttaaattaacacctctgtgcattgccatgagatgcaataaaactgagacaaacaaatggggtctaacaggg
accaccactgtaacccctgcaacagttcccacaactaaaatggtgacagctgaactagtgaatagcactagccag
tgcttaatgtacgataattgtacaggtatacagtcagaatccatggttggctgcaaatttaacatgacagggcta
aaaagggatcagagaaaggaatacaatgagacttggtattcacaagatttgcaatgygaacagtccaataagagt
gagaatgagagtagatgctatatgaggcactgtaatacatcagtcattcaggaatcatgtgataaacattattgg
gatgctataagatttagatattgtgctccgccagggtttgctttgctaagatgtaatgatactaactattcagga
tttgcacctaattgctctaaagtagtggtctctacatgtacaagaatgatggaaacacaaacctctacatggttc
gggtttaatggaacaagggcagaaaatagaacatatatctattggcatgggagagacaataggactataataagt
ttaaataagtattataatttaacaataaratgtaggagaccagggaataaaacagtattaccaattactattatg
tcagggctagtcttccactctcaaccaatcaatgaaagacccagacaggcttggtgttggtttggaggcaaatgg
aaggaagccatgcaggaggtaaaggaaaccgtggtcaagcaccccagctataaaggaacaaatgacacaaagaam
ataacctttacaacaccaggagaaggttcagatccagaagtaaagttcatgtggactaattgcagaggagaattc
ttgtattgtaaaatgacttggtttctaaattggatagaggacagaaatatgactctcctaaagccccaggaacga
caaagacgcaattatgtaccctgccatattaggcagataattaacacatggcacaaagtaggaaaaaatgtgtac
ttgcccccccagagaaggtgatctgacgtgtaattcaacagtcaccagcctaatagcgaatatagaagagaatagg
tctaacaaccatacaaatatcattttcagtgcagaggtggcagaactgtatcgattagaattggggggattataaa
ttagtagaaataactccaattggctttgcacccacaaatgtaaagagatattcctctgtgacaccgaaaaataaa
agaggtgtatttgtgctagggtttttgggatttcttgcaatggcaggttctgcaatgggcacagcgtcgttgacg
ctgtcggctcagtcccggactttgttggctgggatagtgcagcaacagcaacagctgttggatgtggtcaagaga
caacaggaaatgttgcgactgaccgtctgggggacgaaaaacctccagactagagtcactgccatcgagaaatat
ctaaaggaccaggcacggctaaattcatggggatgtgcgtttagacaggtctgccacactactgtaccttgggat
gcccttggtgctaataaaacattggagccacaatggaataatatgacatggcaagaatgggaaaagcagatcaac
tttttggaggacaacatcacacggctattagaagaagcacaaattcagcaagaaaaaaatatgtatgagttacag
aaattaaatagctgggatgtatttggcaattggtttgacctaacctcctgggttaaatacgtttacttaggcctt
tatgtagtggcaggagtaatagtgttaagaatagtcatatatgtagtacaaatactaggaagacttaggcaaggg
tataggcctgtttyttcttcccctccctcttatgttcagcaggaacatctccggctagaagcagcttatttcagc
>#39 M30502 2172 nt
atggagcctggtaggaatcagctgtttgttgtcattttactaacaagtgcttgcttagtatattgtagccagtat
gtgactgttttctatggcatacccgcgtggaaaaatgcatctattcccttattttgtgcaactaaaaatagagac
acttgggggaccatacagtgcttgccagacaatgatgattatcaggaaataattttaaatgtgacagaggctttt
gatgcatggaataatacagtgacagaacaagcagtagaagatgtctggcatctatttgagacatcaataaaacca
tgtgtcaagctaacacctctatgtgtggcaatgaattgtagcagggttcaagggaataccacgaccccgaatccc
aggacctcgagttccacaacctcgagaccacccacatccgcagcctccataataaatgaaacttctaactgcata
gaaaacaacacatgcgcaggattagggtatgaggagatgatgcaatgtgagttcaatatgaaggggttagaacaa
gataagaaaaggaggtataaggacacatggtatttagaagatgtggtttgtgacaacacaacagctggcacatgt
tacatgagacattgcaacacatcaatcatcaaagagtcatgtgataagcactattgggatgctatgaggtttaga
tactgtgcaccaccgggctttgccctattaagatgtaatgataccaactattcaggctttgaacctaagtgcact
aaagtagtagctgcttcatgcacaaggatgatggaaacgcaaacttctacttggtttggctttaatggcactaga
gcagaaaatagaacatatatctattggcatggcagagataataggactatcattagcttaaacaagtattataat
ctcacaatgcgttgtaagagaccaggaaataagacagttttaccaataacacttatgtcaggattagtgtttcac

tctcagccaatcaacacaaggcctaggcaggcatggtgccggtttggaggcagatggagggaagccatgcaggag
gtgaagcaaacccttgtacaacatcccagatacaaaggaatcaatgatacagggaaaattaactttacgaaaccg
ggagcaggctcagacccggaagtggcatttatgtggactaactgcagaggagaatttctctactgtaacatgact
tggttcctcaattgggtagaagacaagaaccaaacacggcgcaactattgccatataaagcagataattaatacc
tggcataaagtagggaaaaatgtatatttgcctcctagggaaggggagttggcctgtgaatcaacagtaaccagc
ataattgctaacattgacatagataaaaatcggactcataccaacattacctttagtgcagaagtggcagaactg
taccgattagaactgggagactacaaattaatagaaataacaccaattggcttcgcacctacagatcagagaagg
tactcctcaactccagtgaggaacaaaagaggtgtgttcgtgctagggttcttgggttttctcgcgacagcaggt
tctgcaatgggcgcgcggtccctgacgctgtcagcccagtcccggactttactggccgggatagtgcagcaacag
caacagctgttggacgtagtcaagagacaacaagaaatgttgcgactgaccgtctggggaacgaaaaacctccag
gcaagagtcactgctatcgagaagtacctaaagcatcaggcacagctaaattcatggggatgtgcgtttagacag
gtctgccacactactgtaccgtgggtaaatgactctttatcgcctgactggaaaaatatgacatggcaggagtgg
gagaaacaagtccgctacctagaggcaaatatcagtcaaagtttagaagaagcccaaattcaacaagaaaagaat
atgtatgaattacaaaaattaaatagctgggatattcttggcaactggtttgacttaacctcctgggtcaagtat
attcaatatggagtgcatatagtagtgggaataatagctttaagaatagcaatctatgtagtgcaattgttaagt
agatttagaaagggctataggcctgttttctcttcccccccggttatctccaacaggcggcccaactgcaa
>#40 AF082339 2166 nt
atgatgtctagtagaaatcagctgcttgttactatcttactagctagtgcttgcttagtatattgtaaacaatat
gtgactgtttttatggcgtgccagcatggaaaaatgcatccattcccctcttttgtgcaaccaaaaatagagat
acttggggaaccatacagtgcttaccagacaatgatgattatcaggaaatagctttgaatgtgacagaggctttc
gatgcatgggataatacagtaacagaacaagcagtagaagatgtctggagactatttgagacatcaataaaacca
tgtgtcaagttaacacctttatgtatagcaatgaagtgtagcaacataagcacagagagcacaacccacatccccg
agcccagggagcacactcaaacccctgataaatgagagcgatccatgcataaaggcagacaactgccccagggga
ctaggggatgaagagatggtcaattgtcggttcaacatgacaggattacagagagataagccaaaacagtataat
gaaacatggtactcaaaagatgtggtttgtgaaccatttaacaccaccacaaaccagaccaggtgttacatgaac
cattgcaacacatcagtcatcacagagtcatgtgataagcactattgggatgctataaggtttagatactgtgca
ccacctggttacgccctactaagatgcgatgatatcaattattcaggctttgcacccaattgctctaaagtagta
gctgctacatgcacaaggatgatggagacgcaaacttctacttggtttggctttaatggcactagggcagaaaat
agaacatatatctattggcatggtagagataatagaactatcatcagcttaaacaaacattataatcttactatg
cattgtaagaggccaggaaataagacagttgtaccaataacacttatgtcagggttaatatttcactcccagcca
atcaataaaagacccagacaagcatggtgctggttcaaaggcgaatggaggaaagccatgcaggaggtgaaggaa
acccttgtaaaacatcccaggtataaaggaaccaatgacacaaaccaaattaactttacaaaaccaggaagaggc
tcagatgcagaagtggtatatatgtggactaactgcagaggagaatttctccattgcaacatgacttggttcctc
aattgggtggaaaacaaaacgggtcaggaacagcacaattatgcaccgtgccatataaagcaaataattaatatc
tggcacaaagcagggaaaaatgtatatttgcctcctagggaaggagagttgacctgcaactcaacagtaaccagc
ttgattgctaacattgacacggatggcaaccagacaaatattacctttagtgcagaggtggcagaactataccga
ttagaattgggggattataaattagtagagataacaccaattggcttcgcacctacatcagaaaggagatactcc
tctactccaaggaggaataaaagaggtgtgttcgtgctagggttcttaggttttctcgcgacagcaggttctgca
atgggcacggcagctttaacgctgtctgctcagtctcggactttattggccgggatagtgcagcaacagcaacag
ctgttggacgtggtcaagagacaacaggaaatgttgcgactgaccgtctggggaacgaaaaatctccaggcaaga
gtcactgctatcgagaaatacttaaaggaccaggcgcggctaaattcatggggatgtgcatttagacaagtctgc
cacactactgtaccatgggtaaataactccttaaaacctgattgggacaacatgacgtggcaagagtgggaacaa
caagtccgttacctagaggcaaatatcagtgaacagttagaacgggcacaaattcagcaagaaaagaatacgtat
gaactacaaaaattaaatagctgggatgtttttaccaactggcttgacttaaccgcctgggtcaagtatattcaa
tatggagtttatataatagtaggaatagtagctcttagaatagtaatatatgtagtgcaaatgttaagtagactc
aggaagggctataggcctgttttctcctcccctcccggttacatccaacaggcagcctacctgcag
>#41 U27200 2148 nt
atggcacatgttaataattacctacttgttacactcctgcttataagtatctatgggtatatgggcaagaacttt
gtcactgtcttctatggtatacccgcatggaaaaatgcatcaattcccctcttttgtgctaccagaaacagagat
acctggggaactgtacaatgcctcccagataatgatgactataccgaaatccaattaaatataacagaggctttt
gatgcatgggataatacagtgacagatcaggcaacaaaggatgtgtggagtctctttgagacctcaataaaacca
tgtgttaaattaaccccactgtgtgtgacaatgaagtgtaataagacgtggagctcagctagcaaagagaccact
acgtcctccgcgtccctcagatcttctactcagaccctactcaacgaagatagcaaatgtattcaaaatgacagc
tgtgcagggataggactagaagaaatgatagactgtcaattcaaaatgacaggattaaaaagagatgagtcaaaa
caatataaggacacctggtataaacaagatttagtgtgtgaaaaggggacaaggagtaatgaaagcaaatgttat

ataaaaacctgtaatacatcaattatccaagaatcatgtgacaaacattattgggatagtttaagatttaggtat
tgtgctcccccaggatttgctttgctaagatgcaatgatactaaatattcaggcttcatgcccaactgtagtaag
gtagtagtatctctgtacagaatgatggaaacacagacctctacatggtttggcttcaatggtacaagggcagaa
aacaggacatatatctattggcatggtaaagacaataggactatcataagcttaaattcatattataatctgaca
atgcactgtaaaaggccaggaaacaaaatggttgtgccaataagaaccgtgtcaggtattctcttccattcacag
cctatcaataaaagacctaaacaagcttggtgctggtttaaaggaaactggacagaagccatacaggaggtgaaa
gagaccattaaaaatcatcccagatattcaggaacaacaaatatctcacagataaggttagcagagcacgcgaga
agctcagatccagaagtaagatatatgtggactaactgtagggggagagtttctctactgtaatatgactttttc
ttaaactgggtagaaaatagaactgggctcaagagaaattatgcgtcatgccacatcagacagatagtcaacacg
tggcacaagattggaagaaatgtgtatttgcctccaagagagggtgaactctcctgtaattccactgttaccagc
ctcatagccaacattgactggatagataagaaccttactaatattactgtgagtgcagaagtgtcagaactgtat
aaattggaactgggggactacaaattagtagagataacaccaattggctttgcacctacaagtataaaaagatat
tcctcagtgacaccgaggaataaaagaggtgtacttgtgctagggtttttgggattccttgcgacggcaggttct
gcaatgggcgcggcgtccttgacgttgtcggctcagtcccggactttactggctgggatagtgcagcagcagcaa
cagctcgtggacgtggtcaaaagacaacaagaactgttgcggctgaccgtctggggggacgaaaaacctccaggca
agagtcactgccatcgagaaatatctcaaagaccaggcacaactaaattcatggggatgtgctttcagacaggtc
tgtcacacgactgtaccatgggtaaatgaatcccttaagccagactggaataacatgacatggcaacaatgggag
aggcaagtccgcttcttggatgcaaatataacaaaattactagaagaggcacagatacaacaagaaaagaacatg
tatgagttacagaaattaaatcaatgggatattttcagtaattggtttgacttcacctcctggatggcatacatc
aggttaggattatatatagtaataggaatagtagtattaagaatagcaatatacattatacagatgctagcaagg
cttaggaagggctataggccagtattctcctcccctccctcttatact
>#42 KP004991 2334 nt
ccactgtaygcaacagtctattatggggtccctgtatggagagatgcacaaactacattattctgtgcagcagat
gcaaaaatggcaagctcagagatgcataatgtatgggckacacaagcatgtgttcccacagacccacarccaata
gaactgaaactaaccaatgtgacagagacctttaacatytggaaaagtggaatggtagagcaaatgcaggaggat
attattagcttatggaccaaagtttaaagccmtgtgtcaagttaacagtcatgtgtgtgaccatgaattgcakc
cgawttaccccctaayacgacaacccatagccttaacatgacaggtccagcccctaggacaagtamtccaaccact
agcagccataacatgacaagtactccaaacattccagagctagaagtttacaactgtacttttaatgtcaccaca
gtattgaaagataaaaaaagtcaacaacaggcgttgttttacagagaagacttatctcagataggtgatrasaac
agcacctataggctgattaactgtaatacttctaccatctctcaagcttgtcccaaagtgtcctttgagccattg
ccaatacagtattgtgcaccggcaggctatgctttaatgaaatgtaaccaaagcaagtttaatgggacagggaca
tgtaatgagactttaataactcactgtacacatgggattagaccgacagtgtcaacacaattcatcttcaatggg
acgttagaaaaggaactcctgatgctaagtaaaaacatttcagacagtggaaaaacaattatagttaagttaaaa
aaggcagtaacattcaagtgtgaaaggacagggaacaacacgcgagggcaaattcagatagggcccatgacaatt
tacaattcagaaaacattgtgggcaaaaccaggaaagcctattgtgcatataatagaacagaatgggaacaggct
ctgaaaaccatcagtgaagcctttgcaaagttagagaatgtgacaaaggtacagtggaagaacagttcaggaggt
gatctagaggtagcaatgctgcattttaattgccatggtgagttcttttattgcaacaccacgacaatgtttaat
tatacttacaattgtagccaccatagctgtaaagctcaatcaaagaacatttcagagggcagcggggactcgtat
attccctgtaaattgaaacaagtagtaaattcatggatgagagtaggatcaggtctttttgccccacctatacgg
ggaactctcaaatgtatgtcaaatataacaggtttactactagaaagagatgtcccattkaacataackgccact
racagaaatataactctcagaccaacgggggagaaatgaaagatatatggagaagtgaactatacccatacaag
gtggtgcaagtcaaagcattatctgtggcacccacaaagataaaaagacccataattggtctgaacagagaaaaa
agaggagcaggactaggaatgctattccttggattcatgagtgcagcaggaagcactatgggcgcagcatctctg
acgctgacggtacaggccaagcaattattgcatggtatagtgcaacagcaaaataacatgctgagagctatagca
gcccagcaggaattgctgaggctctctgtatggggcattagacagctccgagctcgcctgctagctatagaaacg
tatctaagggatcagcagctcctaggactgtggggatgtgcagggaaattgrtttgttatactaatgtgccatgg
aataatacttggactaataaaagtratacagaattagagcacatytgggagaacctaacatggcaggaatgggac
aaactrgttgacaattacactgawgacatcttcctcaagatacagsaggcgaacacacaacaagaggttaatgaa
aagaagttactagagttagacaaatgggcagacttgtggagttggtttgacataactaaatggctgtggtacata
aaaatagctataatgatagtagragctttaataggactaagagttgtcatggtagtgcttagtttagtaaagaac
attaggaagggatatcaacctctctcgttacaggaagcttgcaaaacacttaaggctacagcacaatattggcta
caagaattgcaaagaagcrctactaacttactagatactgttgcagtggcagttgccaattggactgacagcatt
atcttaggtgtgcagagattcggaaggggaattcttaacatcccaagaagaataagacaagggttagaactcagt
ttattgtaa
>#43 FJ424866 2292 nt

gctacgcattatgctactgtgtattatggagtgccagtctggcgagaggcaaatgtaactttattctgtgcagca
gatgctaattatgtgagcaaagaacaacacaatatttgggcaacgcaggcatgtgtgcctaccgacccaaggcca
acagagtttccattggaaaatgtaacagaaacctttaatatctggaaaaattacatggtagatcaaatgcaggat
gatatagttagcttatgggatcagagcttaaagccttgtgtaaaactaacagttatgtgtgtcactctgaattgt
acacaactaaatacaacaaatacaacacagactgagatgacaaatacaactataccaccacatacaactatacca
ccacacatcccacagttagacatttacaactgtagctttaatgttaccacggttctaaaagataagacaacaaag
cagcaagctttattttatagacaagatataatagaaacaggtgaaacaggtacaaatgggatcaaagggtataga
ttaatcaattgtaatacctctactatatcgcaagcttgtcccaaagtctcctttgagccactacctatacagtat
tgtgcaccagcaggctatgccctgatgaaatgtaatgatatacaatttaatggtacaggagaatgcagaaacgtg
tcaatagttcattgtacccatgggatcaggccaacagtctccacacagttgatactcaatggaactttagcagaa
gggaatgccacaataatcagcaaaaatagtagtgatagtggaaaagatattatagtcaaactagccagaccagta
gaaattacctgtgaaaggacaggtaataatacaagaggacagatccaaatagggcccatgacaatttataactca
gaaaacatagtaggaaacacaagaaaggcttttgcaagtataatgagacaaactggcagggtgcgttgaaagac
acagtgcaggctctcaacgagagcctgatccacttccggaattcttcaggaggagatccagaagttacctttctg
cattttaattgtcatggagaatttttctactgtaataccactaaaatgtttagctacaattgcacaacagagggt
tgcaataccactgaaacaagcaataccactgaaacaactaaatatattccctgccatctaaaacaggtagtcagg
tcctggatgagagtaggctcaggcctatttgctccgcctattaggggaactctaagatgtatatctaacattaca
ggaataatattacaaaggatgcacccctaaaaactgatgagaacagcaccttaagaccaataggaggtgacatg
acaaacatttggaggagtgaactgtacccctacaaggtggtgagggtcaaggcattgactgtggcaccaacaaaa
gcaaagaggcctgttattggccataacagggaaaaacgtgcagcaggcctgggaatgctgtttcttgggttcatg
agtgcagcaggaagcacgatgggcgcagcggcagtaacgctgacggtacaggccaggcaagtcttgcatggtatc
gtgcagcagcaaaacaacatgctgagagctatagcagctcagcaagaattgctgagactctctgtttggggcata
agacagctccgagctcgcctgctagccatagaaacctacctaagagaccagcagctcctaggactgtggggatgc
acaggagattaatttgttacactaatgtgccatggaacaagacctggaccgggaaaaatgacactgaactagat
gacatttggggaaacatgacttggcaacaatgggacaaattggtagacaactacactgacacaatattttttagaa
atacaaagggcacaggaacagcaggaggctaatgaaaaggccctactagagttggataaatgggcagatctttgg
agttggcttgacataacacaatggctatggtatataaaaatattcataataataatagcagggctagtaggacta
agaattctaatggctataataaatatgtgtcacagggttaggcagggttactcacctctgtcatttcagttgaca
agagactgctttgcttttattgcttactggggacaagaacttaagcaaagtgctattagcttgctagactgtgtt
gctgtatggaccgctaattggactgatcaagtaatagcaatagctcaaagaataggtaggggcatcttaaacata
cccagaaggattagacaaggattagaaagaagcctattataa
>#44 KP004990 2352 nt
actggaagctatgttatgggtacacgttatgtcactgtatattatggagtgccagtgtggagggaagccaaaact
acattattctgtgcagcagatgcaaacctagctagcaaagagcagcataatatttgggccactcaagcctgtgtg
cctacagaccctactccaataaaggtcaagctaaatatatcagaaccctataacatttgggaaaattatatagta
aagcaaatgcaaaaaaacatcattagcttattaaatcagagcttaaaaccttgtgttaagctaacagtcatatgt
gttacaataaattgtagtaagtttaaaccagctactaccaatacaactagtgcaccaattaacgctagtctacaa
agtagcactactgcacaaaataacctaaacttttataactgttcttttaatgtaactacagtgctaaaagataaa
aaagaaaagaagcaagctctcttctataaagaaaaccttgtaccacttaatcaaacatctaaagaaaaactatat
aggctaattaattgtaacactactacaattacgcaagcctgtccaaaagtgtcttttaaaccattaccaatacag
tattgtgcaccagcaaaatatgcattaataaaatgtaaacaaaaaggttttaatrgtacarractttgcaatraa
acagtaataacacattgcacacatggaattagaccaactgtgtccacacagttaatatttaatggaagtttagca
gaagaagaggctctagtgatgtcaaaagatgttacggccactgggaaaaacatcataataaaattaagtacagga
gtaaacataacgtgtatcagaacaggtaataatacaagaggacaaacacaaataggacccatgacctggtataat
agtgtgaattatataggtaatatcaggagggcttattgccaggtggaggaaacagactggaagggaattttgaaa
aatgttagcaaagctctcataaaacgttacaatggcaaggtaaatgaaacccatacgtttgagtttaagaacgct
tctgggggagacccagaggttacacacctgcacttcaattgtcatggggagtttttttattgtaatacctctaaa
ctcttcaactatacctatgtctgtaatcagacagatcaaggtaatgctaattattcctgtcatagtactagtaca
ttagaaaatggaaccttagtaatacccctgcaaattaaggcaggtagttaattcatggatgagagtgggatcagga
ctttttgcaccacctgtcccgggaagcctaacgtgccactccaatatcacaggtctaatccttcaaagggactgg
ccactaaacaataatacgactactattttgaggcctgaaggaggagacatgaaagacatctggagaagtgagcta
tatccatataaggtagtccaagtaaaagctttggctgtggcacctacaaaaatctccagaccaacaattatggca
cagactcatcagagggaaaaaagaggagcaggcctcggaatgctattccttgggttcatgagtgcagcaggaagc
acgatgggcgcagcggcagtaacgctgacggtacaagccaagcaattattgcatggtatagtgcaacagcagaat
aatctgctcagagcgatagaggcacagcaagaattgctgaggctctctgtgtggggcataagacagctccgagct

cgcctgcttgccattgagacctacttaagggatcagcagctcctaggcctgtggggatgtacaggacaaatagtc
tgctataccaatgtgccatggaatcaaacctggactggtaggaatgacactgaattagatagcatttggaacaag
ttgacatggcaggaatgggataaattagtagataattacactgacaccattttgtagagatacaaaaagcaaat
gaacaacagaaagaaaatgagaaaaaactgttagagttagaccaatggacacagctgtggagctggtttgacata
acaaaatggttatggtacataaaaattttcatcatgatagtaggaggcctcatagggctaaaattttgctagct
ataattaacatagttaaaagagtcaggcagggatactcacctttgtcattacagtgcttgagagactgcttggct
gtgtgtggctattgggcacaagaactacaacagagtgcaactagcttattggacactgttgctgtgagagttgct
gactggactgaccaagtgatcctagtagggcaacgaatagggagaggcatcttgaacataccaagaagacttaga
caaggactagagaggagcttgctataa
>#45 KP004989 2430 nt
atgaaggatatgacagtaatgaagaagaagaagagcagcttaggaaactggggaatttgcttggctttgataata
tactttaatgctatcagttatgttaattgcacacrttatgttactgtatattatggagttccagtttggcaagat
gctaagactaccttgttttgtgcagcagatgcagatctagctagcaaagagcagcataatatttgggccacacaa
gcctgtgtgcccctagatccaacaccaatagagcttaagttgaatatcacagagtcctttaatatttgggaaaat
tatatggtagagcaaatgcargaggacatcgttagtttatgggatcagagtttaaagccttgtgttaaattgaca
tttctttgtgtcactatgaattgtagtgagtataaaggttataattgttctacagaagggaacacaacagctgca
ccatgcaatacaaccgaaaacactacaaaagagaacagcacaggaatgttgacctgtaattttaatgtgactaca
gtattaaaagataaaaaggaacagaaacaggcactcttctatagagaagacctagcaagcttagaaagcaataat
agttatagactaattaattgcaatagttctaccatcacacaagcatgtccaaaagtgtcttttgagccactacca
atacaatactgtgcaccagcagggtacgcactaatgaaatgcaacaggacagactttaatggcacaggaacttgt
aatgagacatctatagtacactgcacacatggaatcaggccaacagtttcaacccagttagtgctcaatggaact
ctagcaaaaggaaaacctttagtaattactaagaatgtctcggagactggagcgcctatcatagtgaaattgagt
gcaagtactgcgataacctgtataagacctggtaataataccagggggagaagttcaattaggacccatgacatgg
tacaacatgagacactacataggcgatatcagaaaagcacattgcactgtctctcggggaaattggacaaaggtc
ctacaaaatgtcagtgcagctctctgggaagcctatccagcagagtggaaaaataaaacacaagataaaaatcac
acaatactatttagggcaagctctggaggagacccagaagttgcttctttgcacttcaattgtcatggggaattc
ttttattgcaacacctctgcactgtttaaccatagctgtacaaagaatatgacaaagaacgaatggatatgtaca
ccaaacaatgccactggcacattaagattaccttgcaggctaaaacaggtagtgaactcatggatgagagtrggg
tcaggcttgtttgcgccacctatcccaggaagtttaacctgtaaatctaacatcacaggtatactgttggagaga
gaccttcctctgggaaatatgaccaacaccaccttaagacccataggaggggacatgaaaaatatttggagaagt
gaattgtacccctacaaggtggtccaagtaaaagctttaggggtggcacctacaaaaatctccagacccacaatt
atgggtcctcacagagaaaaaagaggagcaggcctgggaatgctattccttgggtttctgagtgcagcaggaagc
actatgggcgcagcggcagtcacgctgacggtacaggccaagcaattattgcatggtatagtgcaacagcagaat
aatctgctaagagcaatacaggcacagcaagaactgctgaggctttctgtatggggcataagacagctccgagct
cgcctgcttgccattgaaacctacctaaaggatcagcaactcttaggcctgtggggatgttcaggaaaattagta
tgttatactaatgtgccatggcagaaaaattggacgacttaccaaagtgacagtcaattagatgctatttgggat
aatttaacatggcaggaatgggacaaacaggtaaacaattacactgatctaatttttcttgaaatacaaatagca
caggaacagcaagaacagaatcagaagaaattgttagaattggaccaatgggcacagttgtggagctggttggac
ataacacagtggttgtggtacataaagattttatcatgatagtaggagggcttataggactaaggattttata
gctgtagttaatgtagttaagagagtcaggcagggatactcacctttgtcattgcagtgtgtgagagactgcttg
gctgtgtgtggctactgggtgcaagaattacaacgcagtgcaaccaatctgttggacactactgcagtggcagtt
gctgaatggactgatcaagtgatcttaatagggcaaagaatagggagaggtatactgaacatacccagragaatt
agacaagggctagagagaagtttactctaa
>#46 FJ424863 2277 nt
gctacgcattatgctactgtgtattatggagtgccagtctggcaagaggcaaatgtaactttattctgtgcagca
gatgctaattatgtgagccaggaacaacacaatatatgggcaacgcaggcatgtgtgcctaccgacccaagccca
atagagtttccattggaaartgtaacagagacctttgatatctggaacaataacatggtagatcaaatgcaggat
gatataattagcttatgggatcagagcttaaagccttgtgtaaaactaacagttatgtgtgtcactttgaattgt
acaaagacaacagagacgacaacrcagacaaatryaacaaatacaactataccaccatacatcccacagttagac
atttacaactgtagctttaatgttaccacggttctaaaagataagaaaacaaagcagcaagccttattttataag
caagatataataaaaacagatgagacgaatgagaccaaagagtatagtgagtataggttaatcaattgtaatacc
tctactatatcgcaagcttgtcccaaagtctcctttgagccactacctatacagtattgtgcaccagcrggctat
gccctgatgaaatgtaatgataaacaatttaatggtacaggagaatgcaaaaatgtgtcaatagttcattgtacc
catgggatcaggccaacagtctccacacagttgatactcaatggaactttagcaaaagagcatgccacaataatt
agcaaaaatagtactgatagtggaaaagatattatagtcaaactagccagatcagtagaaattacctgtgaaagg

acaagtaacaatacaagaggacagatccaagtagggcccatgacaatttataactcagaaaacatagtaggaaac
acaagaaaggctttctgcaagtataataagacaaactggcagaatgcattgaaagatacagtacgggctctcaaa
gcgaatcagatccgcttccagaattcttcaggaggagatccagaagttacctttctgcattttaattgtcatgga
gaatttttctactgtgataccactaaaatgtttaactacaattgtacaaaggagagctgtgattgcataacaggg
aattgcacaaattatatcccctgccatctgaaacaggtagtcatgtcctggatgagagtaggctcaggcctattt
gctccgcctattaggggaactttaagatgtaaatctaatattacaggaataatattacaaagggatgtacccctg
aataagactaatgagaatagtactgattacaacaccttaagaccaataggaggggacatgacaaacatttggagg
agtgagttgtacccctacaaagtggtaaaggtcaaggcattgtctgtggcaccaacaaaagcaaggagacctgtt
attagccataacagggaaaaacgtgcagcaggcctgggaatgctgtttcttgggttcatgagtgcagcaggaagc
acgatgggcgcagcggcagtaacgctgacggtacaggccaggcaagtcttgcatggtatcgtgcagcagcaamac
aacatgctgagagctatagcagctcagcaagaattgctgagactctctgtttggggcataagacagctccgagct
cgcctgctagccatagaaacttacctaagagaccagcagctcctaggactgtggggatgcacagggagattaatt
tgttacactaatgtgccatggaacaagacctggaccgggaaaaatgaaactgaactagatgacatttggggaaac
atgacttggcaacaatgggacaaattggtagacaactacactgacacaatattttagaaatacaaaaggcacag
gaacagcaggaggttaatgaaaaggccttactagagttggataaatgggcagatctttggagttggcttgacata
acacaatggctatggtatataaaaatattcataatgataatagcagggctagtaggactaagaattctaatggct
ataataaatatgtgtcacagggttaggcagggttactcacctctgtcatttcagttgacaagagactgctttgct
tttattgcttactggggacaagaacttaagcaaagtgctattagcttgctagactgtgttgctgtatggactgct
gattggactgatcaagtaatagcaatagctcaaagaataggtaggggcatcttaaacatacccagaaggattaga
caaggattagaaagaagcctattataa
>#47 FJ424864 2292 nt
gctacgcattatgctactgtgtattatggagtgccagtctggcgagaggcaaatgtaactttattctgtgcagca
gatgctaattatgtgagcaaagaacaacacaatatttgggcaacgcaggcatgtgtgcctaccgacccaaggcca
acagagtttccattggaaaatgtaacagaaacctttaatatctggaaaaattacatggtagatcaaatgcaggat
gatatagttagcttatgggatcagagcttaaagccttgtgtaaaactaacagttatgtgtgtcactctgaattgt
acacaactaaatacaacaaatacaacacagactgagatgacaaatacaactataccaccacatacaactatacca
ccacacatcccacagttagacatttacaactgtagctttaatgttaccacggttctaaaagataagacaacaaag
cagcaagctttattttatagacaagatataatagaaacaggtgaaacaggtacaaatgggatcaaagggtataga
ttaatcaattgtaatacctctactatatcgcaagcttgtcccaaagtctcctttgagccactacctatacagtat
tgtgcaccagcaggctatgccctgatgaaatgtaatgatatacaatttaatggtacaggagaatgcagaaacgtg
tcaatagttcattgtacccatgggatcaggccaacagtctccacacagttgatactcaatggaactttagcagaa
gggaatgccacaataatcagcaaaaatagtagtgatagtggaaaagatattatagtcaaactagccagaccagta
gaaattacctgtgaaaggacaggtaataatacaagaggacagatccaaatagggcccatgacaatttataactca
gaaaacatagtaggaaacacaagaaaggctttttgcaagtataatgagacaaactggcagggtgcgttgaaagac
acagtgcaggctctcaacgagagcctgatccacttccggaattcttcaggaggagattcagaagttacctttctg
cattttaattgtcatggagaatttttctactgtaataccactaaaatgtttagctacaattgcacaacagagggt
tgcaataccactgaaacaagcaataccactgaaacaactaaatatattccctgccatctaaaacaggtagtcagg
tcctggatgagagtaggctcaggcctatttgctccgcctattaggggaactctaagatgtatatctaacattaca
ggaataatattacaaagggatgcacccctaaaaactgatgagaacagcaccttaagaccaataggaggtgacatg
acaaacatttggaggagtgaactgtacccctacaaggtggtgagggtcaaggcattgactgtggcaccaacaaaa
gcaaagaggcctgttattggccataacagggaaaaacgtgcagcaggcctgggaatgctgtttcttgggttcatg
agtgcagcaggaagcacgatgggcgcagcggcagtaacgctgacggtacaggccaggcaagtcttgcatggtatc
gtgcagcagcaaaacaacatgctgagagctatagcagctcagcaagaattgctgagactctctgtttggggcata
agacagctccgagctcgcctgctagccatagaaacctacctaagagaccagcagctcctaggactgtggggatgc
acagggagattaatttgttacactaatgtgccatggaacaagacctggaccgggaaaaatgacactgaactagat
gacatttggggaaacatgacttggcaacaatgggacaaattggtagacaactacactgacacaatattttagaa
atacaaagggcacaggaacagcaggaggctaatggaaaggccctactagagttggataaatgggcagatctttgg
agttggcttgacataacacaatggctatggtatataaaaatattcataataataatagcagggctagtaggacta
agaattctaatggctataataaatatgtgtcacagggttaggcagggttactcacctctgtcatttcagttgaca
agagactgctttgcttttattgcttactggggacaagaacttaagcaaagtgctattagcttgctagattgtgtt
gctgtatggaccgctaattggactgatcaagtaatagcaatagctcaaagaataggtaggggcatcttaaacata
cccagaaggattagacaaggattagaaagaagcctattataa
>#48 FJ424871 2301 nt
gctaagactgagcattatgctactgtatattatggagtgccagtctggcgagaggcaaaagtaactttattctgt
gcagcagatgccagttttgtgagcaaagaacaacacaatatttgggcaacgcaggcatgtgtgcctaccgaccca

agcccaacagagtttctattggaaaatgtaacagagacctttgatatctggaaaaattacatggtagaacagatg
caggaggatataattagcttatgggatcagagcttaaagccttgtgtaaaactaacagtcatgtgtgtcactatg
acttgttcaaacgacacaaaggggagcaataaatcaactgcaactacaactagcgcaaacacacctacaagcaca
gagacaactataccaacaaacatcccagagttagatattttcaattgtacttttaatgtcaccacggttctaaaa
gataagaaaacaaagcagcaagccttattctacaagcaagatataacagaaacagataaaaatgagtataggtta
ataaattgtaacacctctactatatcacaagcttgtcccaaagtctcctttgagccactacctatacagtattgt
gcaccagcaggctatcccctgctgaaatgtaatgatgaacaatttaatggtacaggagaatgcaacaatgtgtca
atagttcattgtacccatgggatcaggccaacagtctccacacagttgatactcaatggaactttagcaaaaggg
aataatgccacaataatcagcagcaatagtagtgatagtgcaacagatattatagtcaaactagccaacccagta
gaaatcacctgtgaaaggacaggtaataatacaagaggacagatccaagtagggcccctgacaatttataactca
gaaaacataataggaaacacaagaaaggctttctgcaagtataatragacagcatggcagaaggcgttacaagct
acagtgcgggctctcaaaacgaacctgacccactacttcaacaagtcttcaggaggagatccagaggttacctct
ctgcactttaattgtcatggggaatttttctactgtaataccactaaaatgtttaactacaagtgtacaggggaa
cactgcaattgtacagacaaggagctttgcaataagactgcagaaatggagtatattccatgccgcctaaaacag
gtagtcaactcctggatgagagtaggctcaggcctatttgctccwcctmttaggggaactttwagatgtatatyt
watattacaggaatamtattacaaagagatacaccccysgctgagaataataataacaccaccttaagaccatta
ggaggtgaaatgagaaacatttggaggagtgaattgtacccctacaaggtggtaaaggtcaaggcattgtctgtg
gcaccaacaaaagcaaagaggcctgttgttagccataacagggaaaaacgtgcagcaggcctgggaatgctgttt
cttgggttcatgagtgcagcaggaagcacgatgggcgcagcggcaataacgctgacggtacaggccaggcaagtc
ttgcatggtatcgtgcaacagcaaaacaacatgctgagagctatagcagctcagcaagaattgctgagactctct
gtttggggcataagacagctccgagctcgcctgctagccatagaaacttacctaagagaccagcagctcctagga
ctgtggggatgcacagggaaattgatttgttacactaacgtgccatggaacactacctggaccgggaaaagtgat
agtgaactagatggcatttggggaaacttgacttggcaacaatgggacaaattggtagacaactacactgacaca
atatttttagaaatacaaaaggcacaagaacagcaggaggctaatgaaaaggccttactagagttggataaatgg
gcagatctttggagttggcttgacataacacaatggatatggtacataagaatattcataatagtaatagcaggg
ctagtaggactgaaaattctaatggctataataaatatatgtcacagggttaggcagggctactcacctttgtca
tttcagttgacaagagactgctttgcttttattgcttactggggacaagaacttaagcaaagtgctattagcttg
ctggactgtgttgctgtatggactgctgattggactgaccaagtaatagcaatagctcaaagaataggtaggggc
atcttaaacataccaagaaggattagacaaggattagaaagaagcctatta
>#49 JN091690 2268 nt
gtccattatgctacagtattttatggggtgcctgtttggaaagatgcaaaacctcaattgttttgtgcctctgat
gcagacattactagtagagacccacataatatatgggctactcataactgtgtaccccctagatccacatccctat
gaagttccattatcaaatgtctcagtagactttgacatggaaaacaactatatggtggaagaaatgaaaaaggat
ttaattgcactcttccaacaaagctttaaaccctgtgttaagttaacacctttctgtgtaacaatgaactgtgta
aaacttaaggagacaacaactacaatagctaccatcacaaaccccccttgtaaacttgacaggaatagaagattat
aaagtgtataattgtagttttaatcagaccactgagtttagagacaagaaaaagcagatttattctttattttac
agagaagatattatgaaaacagacaataacgatagttattatttgcataattgcaacacctcagccattacccaa
gaatgcgaaaagtcaagctttgaaccaattcctattagatattgtgctccagcaggatatgctatgttgaaatgt
aacagtgcaaactttacaggagtaggaacttgtaacaatgtaagtgtagtacattgcacacatggaatatatccc
atgatagccacagccttacatctaaatggaacacttgaaaagcacaacacaacagcttattttgcaacacccaca
cacaataagccactgttgataaaattcaataagtcagtagacatgaattgtacaagaacaggcaataattcaagg
ggacaagtacaaatcggtccaggcatgaccttttataatatagaaaacatagttggaaataccaggaaagcttat
tgtacggtaaactaccaggaatggagtcatgccataggagaagctaaaaaagtggccgaagaagccctaaagaag
aatattacctttcggtggaaccaaggaggagatctagaagttaccaacttctggtttaattgtcaaggagaattt
ttctattgcaacctcactaactggacaaatataacgtggctagagacgtggctaaagactaacaatagttcccgt
ccccttgtagcaccatgtaaacttaggcagatagtaaaccattggggtatagtatctaaaggtatctacctacct
cctagaaggggaaatttaacatgcagatctaacattacagggttcattacgacatgggataatgacacggagaac
gtgcttgttacctttttcagccaaagtagaagactattggaaggtagaaatgtcacgatacaaagtagtggaaatt
cagcccttggccatagctcctactacaggaaaaaggccggagattagagccaatcacacaaggtcaaaaagagat
gttggcataggactgttgttcctgggatttctcagtgcagcaggaagtacaatgggcgcagcgtcaatagcgctg
acggcacaggccagaggattgctctctggtattgtacagcagcagcacaacctgctgcaggccatagaagctcaa
cagcacttgttacagctttctgtgtggggcattaaacagctccaagccagaatgcttgcagtagagaaatatatc
agggatcaacagcttttaagtgtttggggatgtgccaataaattggtatgccacagtaatgtagtgtggaatatg
acatgggctgaaaatggaactacttgccctcagggttcagaagactattataattgcatctggaacaacttgaca
tggcagcagtgggaaaagctagtggctaattctacagaagaaatatatacattattagagaaagcacaagtgcaa

caagaaactaacaaaaaagaactctttgagttagataagtggagcactctttgggattggtttgacattacacaa
tggctgtggtatattaagatagctatacttatagtagcagggttagtaggacttagaattgttatgtttatagtt
aatgtaattaggcaagttaggcagggttatatgcccctgttttcacagagtagacaacttttcgattggcttagc
aatacatatagtatcttaagaacctcgcttatacaggcaatagataggttagctaacttcacaggctggtggacg
gacttggttatagcaggagtagcatttgtagcccaaggcatcaggaatatcccaaggagaattagacagggccta
gaaatagccttaaattaa
>#50 AF447763 2304 nt
acttattacaccacagtgttttatggagtacctgtttggaaagaggcccaaccaaccttgttttgtgcctctgat
gctgatattactagtagagataaacacaacatatgggcaacacataactgtgtgcctttagatcccaatccttat
gaagtaaccctagccaatgtgtcaataaggtttaatatggaagaaaattacatggtgcaagagatgaaagaagat
atattatcactttttcaacagagttttaagccttgtgtaaaattaacaccattttgcataaagatgacatgtaca
atgactaataccacaaataaaaccctgaattcggcaacaacaaccttaacaccaacagtaaatttgagttctata
cctaactatgaggtgtataattgttcatttaatcagacaactgagtttagagataagaaaaaacaaatatattcc
ttgttttatagagaagatattgtaaaagaggatggtaacaataatagttattatttacataattgcaatacctca
gtcattactcaagaatgtgataaatctacttttgaaccaattcccatcagatactgtgctccagcaggctttgcc
ctgttaaaatgtagagatcagaatttcacagggaaaggacaatgctccaatgtctcagtagttcactgtacacat
gggatttatcctatgatagccacagcattacacttaaatgggtccctggaagaagaagaaacaaaagcttacttt
gttaatacctcagttaatacacccttattagtaaaatttaatgtatcaataaatttaacgtgtgaaagaacagga
aacaatacaagaggtcaagtacagataggtccaggtatgacctttataatatagaaaatgtagtaggggacacc
aggaaagcttattgttcagtcaatgcaacaacatggtacaggaacttagattgggctatggctgccataaacaca
accatgagggccagaaatgaaacggtacaacaaacgttccaatggcagagggatggagaccctgaggtcactagc
ttctggttcaattgtcaaggagaattcttttactgtaatctcacaaattggactaatacctggacagctaataga
accaataatactcatggtactcttgttgcaccatgcagactgaggcagatagtaaatcattggggtatagtgtca
aaaggggtttaccttcccccaaggaggggaacagtaaaatgtcactcaaacatcacaggacttatcatgacagca
gaaaaagacaacaataatagttataccccccaattttctgctgtagtagaagactattggaaagtagaattagca
agatataaagtggtggaaattcagcccttgtcagtggctccaaggccaggaaaaaggcctgaaattaaggccaat
catactaggtcaagaagagatgtgggcataggactgttgtttcttggatttcttagtgcagcaggaagtacaatg
ggcgcagcgtcaatagcgctgacggcacaggccagaggattactctctggtattgtacagcagcaacaaaacctg
cttcaggccatagaagcgcaacaacacttgttgcagctctctgtatggggcattaagcagctccaggccagaatg
cttgcagtagagaaatacataagagaccaacagctcctaagcctctggggatgtgctaacaaattggtgtgtcac
agtagtgtgccatggaacctcacctgggctgaagattctacaaagtgcaatcacagtgatgcaaagtactatgac
tgtatatggaacaatttgacttggcaggaatgggatcgattagtagaaaactctacaggaaccatatactccctg
ttagagaaagcacaaacacaacaggagaaaaacaaacaagagttgttagaattagacaaatggagcagtctttgg
gattggtttgatataacacaatggctgtggtatataaaaatagctataatcatagtagcaggattagtaggactt
agaattctcatgtttatagttaatgtagttaagcaagttaggcagggttatacacccctattttcacagactatc
atagtaggagttagacagatcattgagtggagcagtaatacttatgctagcttaagagttttgctaatacaagcc
atagacagacttgctaactttacagggtggtggacagatttaatcatagaaggagtggtttacatagccagggga
atcagaaatattcctagaagaattagacagggtctggaactagccttaaattaa
>#51 JQ768416 2331 nt
acatactatgctacagtcttttatggagtacctgtttggaaagatgctacaccaccactgttttgtgcttctgat
gcagatgttgctagtacacaggcacacaacatctgggcaacacataattgtgttcccctagatcctcatccttat
gaagtgcctttaccaaatgtatccatagaatttgatatgtctactaattatatggtagaagagatgaaaacagat
ttgatctcactttttcagcaaagttttaaaccatgtgtaaaattaacacccttttgtataagaatgacatgtgca
aatgtaacagaagcaacaacaacaacaccaccaacaacgtcaactagcacaacagaaccaccaacatcaaagacc
acagtgaatacgtcagagatagaagattataaggtgcttaattgcagctttaatcaaacaactgagtttagggat
aagaaaaagaacatttactctttattctatagggaagatgttatgaaagaaagccagaataaaacaaacaatagc
gaatattattatttgcataattgcaataccacagccataactcaaagttgtagtaagtcaagttttgaaccaatt
cccattagatactgtgctccagcagggtatgctatgctaaaatgtaatgatgcaaattttacaggggcaggagaa
tgcacaaatgtaagtgtagtccactgtacacatgggataatgcccatgatagcctcatggttacatttaaatgga
acatatgaaaaggagaaaacgaaggtgtatcatacaaatgtaacaaataatccaccactattagttaagttcaat
gagacaatacacataacctgtgaaagaacagggaacaatacaagaggtcaggtacagataggaccagggatgacc
ttctataacagagaaaacatctttggagatactaggaaagcctttgctatgttaatgcaactgagtggcggaga
actcttgagatggccaaaacagccctcagagaagccacaaaaaaacctaccctgaatatcacacggccaaagggg
gacccagaggtagagaacttttggtttaattgtcagggagaatttttctactgcaatctaacaaaatggattaac
gagacctggcttaacgagacggaccagaccaatcttgtagcaccctgtagactcagacaaatagttaaccattgg

ggcattgtggctaaagccatctacctccctccaagaagaggaacagtaaaatgtgtgtcaaacatcacgggtttc
ctgatgacaaacgaggaaacgggtgctaccttctccggtaaagtagaggattattggagggtggaattagcaaag
tacaaaattgtagaaattcagcccctatcagtggctcctaccacaggaaaaaggccagagattaaggccaatcat
acaagatcaaaaagggacgttggcatagggctgttgtttctgggatttctcagcgcagcaggaagtacaatgggc
gcagcgtcaatagcgctggtggcacaggccagaggggtcctctctggtattgtacagcagcagcacaacctgctg
caggcaatagaggcgcaacagcatttgttgcagcttactgtatggggcgtcaaacagctccaagccagactgctt
gcaatggagaagtacatcagagaccaacagctcttaagtgtttggggatgtgccaacaaattggtgtgtcatagt
actgtggtatggaatgacacttgggctgctaggaaaccctgttcaaacacatctggacctacagattatgattgc
atttggcaaaacttaacatggcaacaatggagtgaattagtagacaactctacagacacaatttacactttatta
gaggttgcacaaatacaacaggaaaagaataagaaagaattattagaactagataaatggagcactctctgggac
tggtttgatattacacaatggctgtggtatattaaattggctataatcataatagcaggcttaataggacttaga
attctaatgtttatagttaatgtgcttaggcaggttaggcagggttatatgcccttgttttcacagttcatacag
accataataatagtacatagaaacttagtgcttaagtggagactatttagagattggcttgccaatgcttatgtg
attgtaaagacttcttttataaggaacttagataggcttgcaaatttcacagcctggtggactgacatactaata
gaaggagcagttaacattttagaggcattagaaacatcccaaccagaatcagacaaggtttagaaatagcttta
aattaa
>#52 JQ866001 2289 nt
ctaggaattggaacatattacaccacagtctattatggggtgccagtatggaaagaggcaacaccaacattgttc
tgtgcttcctcagcaaaggtagcaagcacacaaccacataatatttgggccacacataattgtgtgcctcttgat
cctcagccatatgaaatacctattaacattactactgaatttaatatggaaactaattatatggtagaagagatg
aaaaaagatttaatctctctgtttcaacagagtttgaaaccttgtgtaaagttgacaccctttgtgtaaaaatg
aactgcagtgaattcacaagaaatagtacaacaggaaataatgcaacaaaaaataatacaagtctagaagataaa
ataaaaagcaatcttacaagtctagaaatgtatgaatgctttttaatcaaactacagaatttagagacaaaaag
aagcagatatattcattgttctataaagaggatataatgaaagacaaaaacgacagtactaatacctcttattat
ttaataaattgcaataccacagccataactcaggaatgtgagaagtcttcatttgaaccagttcctattcagtac
tgtgctccaccaggatatgctatgctaaagtgtaaagatgaaaatttcaggggaaaaggaaactgtacaacagtg
aggattgtacattgtacacatagtatatttccaatgatagctacagctttacatttaaatgggtctgtagaagaa
ggagaaacaaaggcatactatgtagaagccaaacataatgcaccattattaataaaattcaataaagcaacggtc
tttaacataacttgtgtaagacccggcaacaacacaagaggacaggtacagatagggccaggcatgaccttctat
aatataagaaatgttattggagacactagaaaggcattttgctatgtaaacaaaacactatgggaaaatgctact
gaggcagcaaaagtagcaataaacgaaactcttaaaaacaccacgttcaaaaacaccacctatacatttaggaca
gatggggatctaaagtggatctcaggaggagaccaagagactcagactcattggttcaactgtcaaggagaattt
ttctattgtgacctcaaaaattggacaaacaatggcatcgagatcgatggaatgctgatagcaccatgtcgactt
agacagatagtaaatcattggggaatagtatccaggggaatttatctccctcccagggagggacaggtcaaatgt
gtatctagtattacaggattcataatgacagctgagacgaatgacactgatggcactatcacacccaccttctct
gcaaaggtggaagactactggaaagtagaaatgagtagatacaaagtagtggagatacagccattatcagtggca
cccactaaaggaaaaagaccagtagttggtgacccagaagcaaaaagtagaagtaaaagagaaataggaatggga
atgatctttatgggatttctcagtgcagcaggaagtactatgggcgcagcgtcaatagcgctgacggtacaagcc
agaggattgctctctggtatagtacagcagcaagcaaatttgctgcaggcaatagaggctcaacaacatttgttg
cagctctcggtgtggggcataaaacagctccaagccagactgctagcagtggagaaatacctaagagatcaacag
ctcctgggtctatggggatgtgctaacaaattgatctgtcatagttcattagaatggaatagctcttggttaaac
aaaaccctgtgcggtaataagacaaacaattatgaaaacaattatgagtgcatatggtctcaatacacctggcaa
cagtgggatgaggaaataaggaacatctcagatgttatctaccttagtttagaaacagctcaggtgcaacaagaa
agaaatcacaagcaactcctagaactagataaatggagctcattatggaactggtttgacatcactaattggctg
tggtatattaaaatagctataataattgtagccagcttagtaggcttaagaatagtaatgtttatcataaattta
gtggggaagcttaggcagggctattcacttttgtctccacagcagcattgcattgtttgcatagatactttagca
gagttcactggctggtggacagacggggttatagaagcactaagagtagcagtagacataataagacatatacca
actagaatcaggcagggattagaaattgctctaaactaa
>#53 DQ373065 2193 nt
ttgtgggtaacagtgtactatggggtacctgtctggagagatgcagagacagttctattctgtgcttcagatgct
aaggcccatagtacagaggctcacaatatttgggccacgcaagcatgcgtccccactgaccccaacccacaagaa
gtactaataccaaatgtgacagaacgttttgatatgtggaaaaacaatatggtagatcaaatgcaagaagacatt
attagcttgtgggaacagagtttaaaaccctgtgttaagttaaccccattatgtgtaaccctatcatgtagctca
tggaggagtgtgaacaatagtgttaaccaaacaaaccacgtacaaatgcaaaattgctcatttaatgtgaccact
gagttaagagataaaaagaagcaagtctactctctgttttatatgggagatataatacccttagacactaataat

agcagtggtaataacagtcaatataggttaataaattgtaacaccacagctgtgacacaggcctgtcctaaaatt
tcctttgagccaattccaatatattattgtgcaccaccaggatttgccattataaaatgtaatgatcaagatttt
aatggaactggggaatgtaacaatgtaagtactgtacaatgtacccatggaataaagccagtgatatccacacag
ttgatcctaaatggcagcttagcaacatcaaatatcgtaataagaaataattcaaaagacacactcttggtgcaa
ttaaatgaaagtatcccaataaattgcacaagaccaggaaataagacaagaggacaagtacaaataggacctggt
atgacattctataacatagaaaatataataggagacactaggcaagcatattgtgaggtaaacaggacatgggaa
caaatatggaatacaacaaaacaaataataataaacaacagaaagaatattacatttatacccaatccaggcgga
gacctagaagtaacaaacttaatgatcaattgtggaggagaattcttttactgtaataccagtcaattgtttaca
aatcaaaatggcaacacaactggcaatatcaccttacaatgcagaataagacaaattgtaaatttatggacaaga
gtaggaaaaggaatttatgctccgccaattaaaggacctattaactgtctctctaacatcactgggataatactt
gactatacaaagtctggaactgagaagtacaccatatatcccacagggggagacatgactaacctctggaggcag
gagttgtataagtataaggtggtcagcatagaacccataggagtagcaccaggaaaagctaaaagacacacagtg
actagacaaaaaagagcagcctttggactaggtgcgctgtttcttgggtttcttggagcagcagggagcactatg
ggcgcagcgtcaataacgctgacggtacaggcccggaaattattatctgggatagtgcagcagcagaataatctg
ctgagagcaatagaggcgcaacaacacttgctgcaactctcagtttggggcattaaacagctccaagcaagagtc
cttgctatagaaagatacctgagggaccagcaaatcctaggcctatggggctgctcaggaaaatctgtgtgttac
accaatgtgccttggaataccacttggagcaacaataactcctatgacacaatatggggaaatatgacatggcaa
aattgggatgagcaagtaagaaattattcaggtgtcatttttgggctgctagagcaagcacaagagcaacaaagc
ataaatgaaaagtcactcttggaattggatcaatggtcaagtttgtggaactggtttgacattacaaaatggctg
tggtacataaaaatttttcataatggtagtagcaggcattgtaggcataagaataataagcataataatgtctatg
gtagcaagagttaggcagggatattctcccctctcgttgcaggaactcagaatatatttgtggggaataatagct
tactggggaagggagctaaaaattagtgctataaacttgcttgatactacagctgttgcagtagcagaagggaca
gatagaatcatagagctagtgcaaagaataggacggggaatactacacatacctaggagaatcagacaaggtcta
gaaagagcacttttataa
>#54 AF103818 2130 nt
ttatgggctacagtatattatggggtacctgtatggagagatgtagagacaaccttattctgtgcctctgatgca
aaggcatacaagcaggaggcccacaacatttgggccacacaggcatgtgtccctactgaccccaatccacaagaa
gtacatttgccaaatgtgactgaaaagtttgacatgtgggaaaataatatggcagaacaaatgcaggaagatatt
attagcctatgggaccagagcttaaaaccttgtataaagttaaccccattgtgtgttactatgacttgtcttaac
cccgatagtaatagtagtgctgtaaatactactgatataatgagaaactgttcttttaatataactactgaatta
agagacaaaaagaaacaagtgtattccttattttatgtagatgatctagctcatatcaataataatacctataga
ctgataaattgcaacaccaccgctatcacacaggcttgtcctaagacctcctttgagccaattccaatacactat
tgtgcaccaccaggctttgccatcctaaaatgtaatgaaaaagatttcaaaggaaagggagagtgtaaaaatgtt
agtacagtgcagtgtactcatggcataaaaccagtggtgactacacagctcataataaatggcagtttagcaact
aaaaatgttactgtaagaagtaaaaactttgcagacattattctagtacaattctcagagggagtcaatatgact
tgtattagaccaggaaacaatacagtgggaaatgtacaactaggaccaggaatgacttttataacataccaaag
atagtaggagatgtaagagaagcacattgtaacatctcaaaactgacatgggagaaacagagaaaatacacttta
gagataataaaaaaggaagcaaacctgacaaaggtagagttaattccaaatgcaggaggagacccagaagtggta
aatatgatgcttaattgtggaggagaatttttttattgtaatacaattcccctatttaacatgacctacaacaat
accgacaacaccactatcacacttaagtgtagaataagacaaattgtaaatcagtggatgagagtaggaaaagga
atctttgccccaccaatcaaaggtgtgctaagttgtaactcaaatataacgggaatgattcttgacataagcata
agcgcagtcaataacgatagtaggaatataacagtgatgcctacaggaggagatatgacggctttatggaaaaat
gaattacataagtataaggtggtcagcatagaacctataggagtggcaccaggtaaggccaaaaggcatacagtg
aaaagagaaaaaaagagcagccttcggactaggtgcactgttcctggggtttcttggagcagcaggaagcactatg
ggcgcagcatcagtagtgctgacggtacaggcccgacaattattgtcagggatagtgcaacagcagaataatctg
ctaagagcaatagaggcccaacagcatttattgcaactatcagtttggggcattaaacagcttcaagccagagta
cttgctgtggaaagataccttaaggatcagcagatcctaggtctgtggggctgctcaggaaaaacaatttgttat
accactgtgccttggaatgatacctggagtaacaacctctcctatgatgctatttggggcaatctaacttggcaa
gaatgggacagaaaagtaagaaactattcaggtactattttagtcttatagaacaagcacaagaacagcagaat
acaaatgaaaaatcactcttggaattggatcaatggtcaagtctatggaactggtttgatattaccaactggctg
tggtatataaaaatatttttaatagtagtagcaagcttagtaggaatcagaattgtaggtgtgatattttcacta
gtagcaaaagttaggcagggttattctcccctctcgttacaggctacaagcttattagatacaacagcaatagca
gtagctgaaggaactgatagaattatagaattaacaagaaggctctttctaggtattatacacataccaagaaga
attaggcaaggcctagagagaagcttatta
>#55 EF535993 2190 nt

gatcagttgtgggcaacagtgtactatggagtacctgtgtggaaagatgcggagacaacactcttttgtgcagca
gatgcttcagcacttaacaaagaagcccacaatatttgggcctcgcaagcctgtgttcccacagaccctaaccca
caagaagtgcatgtaccaaatataactgaaacctttgacatgtgggaaaataacatggtagaacaaatgcagact
gatattattagtctatgggatcaaagtctaaaaccatgtgttaagttgacaccactatgtgtcactatgcattgt
attccatacaacataagcagcactgggaatgacactaggaatagctctacaagccagatggtaaaaaaatgcttc
tttaatatgactactgaattaagagataaacagaagcaagtatattctttattttatgtagatgatatagtasmk
attaatggtamtaaktcctataggctaataaactgtaataccacagctattacacaagcatgccctaaaacctcc
tttgagccaattccaatacattattgtgcaccccaggttttgcaatcatgaaatgcaatgagccagattttaat
ggaacaggaaaatgtagaaatataagtacagtacaatgtactcatggcatcaagccagtggtaactacacartta
attcttaatggaagtctagctgagaacagcacgaytgytaggactgaasacatgamaraaatggatragaccatc
atagtacagtttcataaaagggtccagatgacatgtataagaccaggaaataacagcagaggacagatacaaata
ggaccagcaatgtcattttataacctagaaaatattataggagataccagacaagcatattgtaatrtcagcgcc
gcagaatgggaacaaaggttmaawgacactrtgagggctataaaaagtcttaagccaggcagtaacatcaccttc
acagatcaacaaggaggagacccggaaatagtaaacatgatgttcaattgtggggagagtttttttattgtaac
accaccccattgttcaataacagttggacaaatacaactagctcagatataaatagctcaaatataataataaac
tgtagaataaggcaaattgtaaactcctggatgagagtgggcaagggaatttacgccccaccaattagaggaact
ataagttgcacatcaaatattactgggctcctgctcgagttagacagaggactcccgagaaatggactaatacc
aataacaagttaacactataccctacaggaggagagatgagagatttgtggaggctggaattacataaatacaaa
gtagtcagcatagaacccataggagtggcaccaagtaaagctaaaaggcacacagtgacaagagaaaaaagagca
gcctttggactaggtgcgctgtttcttgggtttctaggagcagcaggaagcactatgggcgcagcatcactaacg
ctgacggtacaggcccggcaattattgtcagggatagtgcaacagcagaacaacctgctgagagcaatagaggcg
caacagcatttgttgcaactctcagtatggggcattaaacagctccaagcaagagtccttgctgtggaaagatac
cttaaagatcagcaaatcctaggtctgtggggctgctcaggaaaatccatctgttatactactgtgccttggaat
agtacttggggtgctaatacctcctatgatgaaatctggaataacctaacatggcaagactgggacaagagagta
aaaaattattcaggagtcatctttgaccttatagagcaagcacaagagcaacaraacacaaatgagagaagctta
ttggaattggatcaatggtcaagtttgtggaattggtttgatatttctaattggctgtggtacataaaaatattt
ctaatggcagtagcaggcttaataggcataagaatagtaggagttatcatgtccttaatagcaaaggttaggcag
ggatattctcccctctcgttgcaggctacaagcttattagatacaacagcaattgcagtagctgaaggaacagat
agaattatagaagttgtgcaaagaataggtagaggtattctacatataccaagacgcattagacaaggcttagag
agaagcttgctataa
>#56 AY169968 2202 nt
gaatgggtaacagtgtattatggagtacctgtgtggagagaagtcaacacagtactcttctgtgcttcagatgct
aaagcacatagcacagaggctcataacatatgggccacacacgcatgtgtacctacagaccctaatcctcaagaa
gtagttcttcataatgtaacagaggactttaatatgtggaataatcagatggtagaacagatgcaagaagacatc
agtagtctctgggaccagagtcttaaaccatgtgtaaagctaactcctttatgtgtaacaatgaaatgtagtaac
gttactagacaaaatagcactagcaactctaacaataaccctaccctcaagaacaatacttcaggtaagaatgaa
actggagttttacaaatgaaaaactgtacatttaacacaacaacagaattgagagataaaaagaaacaggtctat
tcattattttatgtagatgatctgcaaagcctaggaagtggaactggtgatacatatacaatgattaattgtaac
accacagccataacacaggcatgtccaaaagtgtcctttgagcctattccaatacactattgtgcaccagcagga
tttgcaattctcaaatgtaatgatgtaaacttttcagggaaaggaaaatgtagaaatgtaagcacagtccattgt
acacatggaatcaaaccagtagtaacaacccaattgatcattaatgggagtctagctacagaaaacataacagta
agagtaaacaatgcctctaaaaatacccatgattggatagtacaactaagcacagcagtgaatctgacgtgtaag
agggtagggaataacacaagaggcaaagtacagataggaccaggaatgaccttctataatatggaccatatattt
ggtgatacaaggaaagccttttgcgaactcaatgggacaacatggaatgaaacactgcaaaaagttagagaaagc
ttaataaaggaaataaaagcaaatgcaaatggaacctacaatataacgtttgagccaagcagtggaggtgaccca
gaaatagcaaatcacatgttcaactgtggaggagaattcttttactgcgatactagaaaaatgtttaatgaaagt
gaaccttttcacgagaacatgacaataccatgcagaattagacaaatagtgaattcatggatgagagtaggaagg
ggaatctatgcacctccaataccaggacatataacttgtaactctctaattactgggctcatattgacacgagac
catgttaataatactaataatacatttagaccaataggaggagacatgaaaaacatttggaggagtgaactctat
aaatacaaagtggtgcgcatagaaccattatctgtggcaccaaccaaagcaaaaaggcatacagtaggagaaagg
agacagaagagagcagcctttggcctgggagcactgttccttgggtttctgggtgcagcaggaagcacaatgggc
gcagcggcagtcacgctgacggtacaggcccgacagttactatcgggaatagtgcaacaacagaacaacctgttg
agagcaatagaggcccaacagcatctattgcaactaacagtctggggagtaaaacaactccaagccagattactt
gctgtagaaagatacctacaggatcagcagattctaggcctttggggctgctctggaaagtctatctgttatacc
actgtgccttggaacaaaacatggtcaggcaagtctatgagtgacatttggaacaatttaacatggcaacaatgg

gacaaattgatcactaattacacagggacaattttggtttattagaagaggcacaatcacaacaagagaaaaat
gaaaaggacttattggagttggatcaatgggcatctttgtggaactggtttgacataaccaactggctgtggtac
ataaaaattttccttatggcagtaggagggattataggtttaagaattatcatgtcagttgtctcagtgatcagg
agagtcaggcagggttactcacccctctcgttgcaggctacaagcttattagatgcaacagctattgcagtagga
gaaggcacagatagaatcataagagcagtgcaaatagtattcagaatcataggcaacataccccggcgcataaga
caaggcctagagagaactttactttaa
>#57 AF382828 2184 nt
caatggtgggtaacagtatactatggggttccagtctggaaggaggctactacaccattgttctgtgcagcaaat
gcaacagctttaaaagaggaacctcataatatttggccacacaagcctgtgtgcctacagacccctctccagaa
gaggtcatcctaattaatgtgacagaagaatttaatgtatgggacaatgctatggtagagcaaatgcaggaggat
attactagcctatgggaccagagccttagaccttgtgtaaaattgaacccttttatgtgtgcaattaacgtgtact
tctataaatgctactggaaacgaaactaatgctactggaaacggaattgaaaaggatgggttggcacaagacatg
agaaattgtactttcaatacaaccacagaattaagagataaaaaacagcaaatttattccttattctggaaaaat
gatcttgtaggtactaacaatacgtttaggttaattaattgcaatactacagcaatcacacaagcttgtccaaaa
acctcctttgaaccaattccaatacattactgtgcaccagctggctttgccctgttgaaatgtaatgataaagat
tatccaggaaaaggaaagtgtaaaaatgtaagtacagtgcattgcacacatggaattaagccaactgtaactact
cagttgatactcaatggcagcttagcagaagaagaagaggtggtgttcagaactaagaatatgacagctccaggt
ctcagtgacacagttatagtacagttaaaaagggcaattcccattaattgcagtagaccaggaaacaatacagga
agagcaataaaccttagcccagggacaacattcttcaacacggaagccctaataggaaaccctaggaaagcgtcc
tgccaccttaatggtacactatggaataacattctaaacagaataaaacaaaagattaaaaacagcacaacgtgg
cacagggggacatcacattcacaaagcatccaggaggagacccagaagtggtaaatttcatgtttaactgtgga
ggggagttcttttattgtaatacatcaagattaattacctgtaacagtagtgacacaagtgagtacattctccca
tgcaaaattaggcaagtagtaaattcttggatgagagtggggaaaggtatctttgccccgcctagaagaggaact
ataacctgtaattccaccattacaggactcctcttagaagtacagaatggcacggggaacaacacagaagtttac
ctgtcaggggagatatgagagatatttggaggagtgaattatataaatataaaatagtaaaaattgagccactg
gggtggcaccaacaaaagcaaagaggtacacagtggcaaaagcacttgatagatccaaaagagcagcctttgga
ctgggggcgctgtttcttggattccttggagcagcaggaagcactatgggcgcagcatctgtgatgctgacggta
caagctcgaaacctgctttcgggaatagtgcagcagcaaaataacttgctgcgagccatagaagctcagcaacac
ctgctgcagctatcagtctggggcataaaacagctccaggcgagggtccttgctgtggaaagatatctaaaggat
cagcaagtcctagcactatggggctgctctggaaaaactgtgtgctactctactgtaccctggaatacttcctgg
aacagtaacaaatcttatgaggacatttggagtaatctgacctggcagcaatgggataagttagtagaaaatcat
acaggaaccattttcaattattacagagagcacatgagcaacagaatagcaatgaaaaagagctattggaattg
gatcaatggtcctcattatggaattggtttgacataacaaactggctgtggtatataaaaatgtttataatctta
gtgggaggggttataggtttaagaattgtaataggtgtagtaaatataataagaagaagtaggcagggatactca
cctttatcgtttcaggctattagcctgcttgatactacagcaattatagtagccgaaagaactgatacaataata
gaagtagcaactagaatagggagaggcatattgcatataccaagaagaatcaggcaaggtttagaaagagcttta
ctagaataa
>#58 EF535994 2151 nt
tctgaaaataatttatgggtaacagtgtactatggagtacctgtatggaaagaggcaaaaacaactctttttgt
gcatcagacgctaaagcacagatagctgaggcacacaacatatgggcttctcaagcttgtgtgcctacagatcct
aaccctgaagaaataacgttagaaaatgtaacagaagagtttgatgcatggaacaataacatggtagaccaaatg
caagaagatctcattagcctatgggatcaaagcctaaagccatgtgtaaaactgacccccctctgtgtgacctta
aactgttccacaaatacaactacagaggcatctcaggtgcagtataattgttctttcaatgtaactacagaatta
agagataagaagaagcaagtgtactccttattttatagagaggacataacaagtcttgatagcaataagacagtt
aaaaatggaacttataggttaattaactgtaataccacagccataacccaggcttgtccaaaaacatcatttgaa
ccaattcctatttactattgtgcaccagctggatttgcccttttaaaatgtaatgaccagaatttcaagggaaaa
gggacatgtagaaatgttagcacagtgcattgtacacatggtattaagcctgtagtctctacccaattcctcttg
aatggaagcctggcagaaggaaacaagactgtagttagggtaagaagtaaatctaatacagagactatcattgtg
cagctagctacagcaatatatatcaattgtaccagactaggtaataaaaccatagagggcatacctatagggcca
ggacaaatttttataggactaaaacggtggtaggcgacaccagaggagcagagtgcagaattaatggacagcc
tggaatgagactctgagacaggtaaaggaggcattaaacaacacatatcgaaatctcaatctcagtctcacagaa
ataaactttgaaggtgcatccggcggagatctagaagtaactacacattatttcaattgtggaggagaattcttt
tactgtaatacatcaaacttattcaatcaatccactattaacaatattacacacattccgtgcagaataagacaa
attgtaaatcagtggcaaggagtaggaaaaggaatctttgcacctcctattagaggaacaattcaatgtaattcc
acaatcacaggcctactcttaacaagagatggtaaaaatgagactgaaacttttagacctacaggaggagacatg

agaaataattggagaagtgaattatacaaatacaaggtagtaaaaattgaacccctaggattagcaccaaccaag
gcaaaaaggagaacagtgcaaagagaaaagagggcagtaggactgggagtgatgttccttggattcatgggagca
gcaggaagcactatgggcgcagcggcactgacgctgacggtacaggccagaaatctactctctggtatagtgcaa
cagcaaagcaatttgctgaaagctatagaggcgcaacagcatctgttgcaactctcagtctggggcattaaacag
cttcaggcgcgagttcttgctgtggaaaggtatctaaaggatcaacaactcctaggactatggggctgctctgga
aaactcatttgcactactaatgtgccttggaataatacttggagtaaaaaattaggaacaaacctatccttttgg
gataacatgacatggatgcaatgggagaaagagattgataactacacagaaactatctatgaacttctaacacga
tctcagaatcaacaggaagtcaatgagcaagaattgttagctctggataaatggtcaagcctgtggaattggttc
gatataacacagtggctgtggtatataaaattatttgtcatgatagtaggaggcttaataggcataagaatagtt
tttgctatgctttctatagtcaatagagttaggcagggatactcacctttgtcgttccaggctataagcttgctt
gacgccacagcaatagcagtagcagagggaacagatagaatyatagaaatagcacaaagagttggcagaggcatt
ttgcatatacctagaagaattagacaaggtttagaaagagctttgctataa
>#59 DQ373066 2169 nt
gaaaattggtgggtcactgtttattatggggtcccagtatggagggaggcaaaaaccaccctttctgtgcctcc
gatgctaagagttatagtacagaagctcataacatctgggccacccaagcttgtgtacctactgatcccactcct
caagaggtactcttacctaatgtcactgaagagtttaacatgtgggaaaattacatggtagatcaaatgcaggaa
gacataataagtctctgggaacaaagtcttaaaccatgtgtaaaattaacacccttatgtgtgacccttacttgt
aataatcccactaatacttcctgcactaactctactgacgatcgcttgggagacatgagaaattgtagctttaat
gtcacaactgaactaagagataagaaaagacaagtgtattccttattctatgtggaggatataacagcaatagga
aataatagtacttacaggttgattaattgtaataccacagccatcactcaagcttgtccaaagacttcctttgaa
ccaattccaatacattactgtgcaccagcaggatttgcactcctaaaatgtaatgatatagattacaaagggaat
gaaacttgtaaaaatgtaagtacagtacattgtacacatggcattaaaccagtggcaaccacccaactgatttg
aatgggagtacagcagacaatcagactgtggccagaatagacccttcagagaatttagcaataatacagctcaaa
gatccagtaaagattacatgtaggagaccaggaaataacacaagaggtcaaatacaaataggaccagcaatgact
ttttataacatagaaaatgtagtaggggacactagaaaagcatattgtgagataaatgggacacagtgggcaaaa
gccctcaatgaaacaaaagaagttctaaggaacatactgaggaagaacataagctttatggtcccttctgggga
gatccagaagtgactaaccatcactttaactgcgggggagaattcttctactgtaatacaagtgagatcatcaat
atcactaagataaacaaaactgagaatatgacaataatcccttgtagaataagacaaatagtgaattcctggatg
cgtgtagggaaaggaattttgcaccaccaattagaggtaacattacctgcacttctaatatcacaggtatgcta
ctagagatacataagaaccgagaagatcagggagaagatcaggatcagaataacacatacgtttgtctaacaggg
ggtaacatgaaagatatttggagaagtgaactatacaaatacaaaatagttgagatacaaccactaggagtagca
cctactaaaagtagaagatatgcagtagaaaaacaacaccatagagaaaagagagcccttggactaggagcactg
tttcttgggttcctgggagcagcaggaagcactatgggcgcagcatcagtcgtgctgacggtacaagcccggcaa
ttgttaacgggcatagtgcagcagcagaataatctgctaagagcaatagaagctcaacaacatctgttgcagctt
tctgtctggggaatcaaacagctccaggcgagagttcttgctgtggaaaggtacctaaggatcagcaactccta
ggtctatggggctgtacaggaaaaactatttgtcccactgctgtacgctggaacaagacatggggaaatattagt
gactatcaggttatttggaataattacacatggcaacaatgggatagagaagtaaataattacacaggactcatc
tacacattgctagaggaggctaatacacagcaggagaaaaatgaaaaggagttattggaattggattcatgggct
aatttatggagttggtttgacataacaaattggttgtggtacataaagatgttcttaatagtagtaggaggcatt
ataggcttaagaatatgctttgcaataggatcactaatcaatagggttaggaagggatactcacctttgtcattg
caggccataagcttattagatactaccgctattgcagtaggagaaggaactgatagaatcttagaagcaataaca
cgcttgggcaggggcatttacatatacctagaagaattagacaaggcttagaaagagctttgctttaa
>#60 DQ373064 2160 nt
agcactaaaaatgaaaatttatgggtgacagtgtactatggagtgcctgtctggaaagaagcagaagcaactctc
ttttgtgcgtcagatgcaaaagcacaaaaggcagaggcacacaatatatgggcctctcaagcttgtgtacccaca
gatcctgatcctaaagcaatagaactaagcaatgtaacagaaaattttaatatgtggacaaatgcaatggtaaat
cagatgcaacaggatgtaattagcttatgggatcaaagcctaaaaccatgtataaagctaacccctctttgtgtt
actttacattgcagtatccctaaatttgataactctagcattaatagctctaatgagactatgaaaaattgtagc
ttcaatacaacaacagaactaagagataagaagaaacagacttactcattgttttatgtagatgatttaactcag
ataaacaagacaaccgagagctataagcttataaattgtaatactacagccataacccaggcttgcccaaagatt
tcttttgagccaatacctatacattattgtgctccagctggttttgctcttctaaggtgtaacaataaaacatat
aatggtagtggtccatgtaacaatgttagtacagtacactgtactcatggaattaaaccagttatttctactcaa
ttgatcctgaatggtagtctagcagaagaaaaaatcatgattaggtacaaggctgataaggttctagtacagctc
aatacaagtatacacatcaattgtactagggtaggaaataagacaataaaaggaatcccccctaggaccaggacag
ctgttctatggaacagaaactgtagtaggagacacaagagaggctcattgtgaaattaaccagacacattggtat

aaaatactaaaccaggtgaaaagagagctaaccacggtcttcaatgaatctaacaaaactgtacactttgcaaac
agttcaggtggagatccagaagtggcaaaccttcattttaattgtggaggagaatttttttattgtaacacctcg
gccttgttcaatgacaccttgttacgcaataaaaccttgctcaatgaaaccttgcacaataacttgacactacct
tgtaggattagacaaattgtaaacctatggctaagagtaggaaagggaatatttgctccacctattagaggtaac
atccgctgtaattccactattactgggttgatcctagaaaaacacacaaacacagacaatatcaccttccgtcca
ataggaggggatatgacagacatctggagaagtgaattatataactataaaatagtaaaaattgaaccattagga
gtagcgcctaccagggcgagaaggaggactgtggatagagaaaaaagagcagcagggctgggagtgtttttcctt
gggttcttgggagcagcagggagcactatgggcgcagcatcattaacgctgacggtacaggccagacaattattg
actggcatagtgcaacagcagagtaatttgctgagagctatagaggcgcagcaacacttgctgcaactctcagtc
tggggcatcaaacagctccaagcaagagtccttgctatcgaaagatacctaaaggatcaacaactcctaggaata
tggggttgctcaggaaaactcatctgcaccacttctgtgccatggaatagaacttggagtaacaaaacatataat
gaaatttgggacaacatgacatggatggaatgggacagggaagtaagaaattacacagaaattatttatgggtta
atagagcaagcacaagaccagcaggaaaataatgagaaaaaacttttagaactggatcattggacaagcctgtgg
aattggtttgacataagtcactggctgtggtacataaaaatattcattatgataataggaggcttaatagtgtgt
aggataattttgctgtgctcgctatagtaaatagagtcaggcagggatactcacctctgtcatttcaggccact
agcttgcttgatacaacagcaatagcagtagctgagggaacagatagaatcatagaaatagtacaaagaattggt
agaggaatattacatatacctagaaggattagacaaggtttcgaaagggctttattataa
>#61 KP861923 2400 nt
agtaaggaggaactgtgggtgacagtgtactatggagtacctgtatggagagatgcagaaacaactctcttttgt
gcatcagatgcaaaggcacaagaaacagaggcacataatatctgggcctctcaagcttgtgtacccacagatcct
aaccctgaagaaatacacctaccaaatgtaacagaatactttgatatgtggaaaaataatatggtggaacaaatg
caggaagatataattagtttgtgggatcaaagtttaaaaccatgtgtaaagttgactcctctctgtgttacttta
aactgcactaattgtatttggggaaatggcacttttagggaatggaatgacacaagggaagacttgagaaactgc
agctttaatgctggggatataagagacaagaaaaagagggtccattcactattttatgctgaagatattatacta
atagaccgcaccagaactacaaagcagggaaattcaacacaagcccccgccacgacgcctatagcgagtaccaag
gtacccgatagcaatgacacatgcagccccaacaatactctagaatatagacttataagttgtaatacctcagtc
ataacccaggcctgcccaaaaacttcttttgaaccaatacctatacactattgtgctccagctggttttgctctt
ttaaaatgtaatgatacaaattttagtggacagggtatatgtagaaatgtcagcacagttcattgtacacatgga
attaggccagtggtgtcaactcacctacttttaaatggcagtttagcaaaaggggatatcatgataagggtaaag
aacaaaaaccaaagtactgacaccattatagtacaattgaacaagagtataaatattacttgtattagaccaggg
aataacaccataaaaggcattcctatagggccaggacaacacttctatggtacagaaacggtagtaggtgataca
aggaaagcctactgcaaaatcaatggaacacaatggtttgatacattaaagagagtagcttgggaattaaaggaa
acattcaatcttaccagagtaacatttgatcaagcatcacccggagatccagagatacaaaaacactacttcaat
tgtggaggagaattcttttattgtgagacatcttcattgtttaatgctacaatagatattacaccaaatactaca
ctgaatgagacgacctgggattatccagctgagaacaatattaaccaagaaatgataatccattgtagaataaaa
caaattgtatcaatgtggcagagagtgggaaaaggaatattttctcctcctattagaggaaccattcaatgtaac
tccactattacaggactactgctaactcgcgatggtaataacaaagcagatagtgagaacaaaacaaggattgaa
acaggacagaacaaagcagaagagattaataggactgacatttttagacctgcaggaggagaaatgagagacaat
tggagaagtgaattatatagatacaaggtggtgaaaattgaaccactaggactagcacccaccaaggcaaaaaga
aggactgtaaatagagaaaaaagagcaccaggactaggactgatgttccttgggttcttgggagcagcagggagc
actatgggcgcagcatcattaacgctgacggtacaggccaggcaattattgactggcatagtgcaacagcaaagc
aatttgctgagagctatagaggcgcaacagcacttgttgcaactctcagtctggggcattaaacagctccaggca
agagtccttgctattgaaagatacctaaaggatcaacagctcctaggaatctggggatgctcaggaaaactcatc
tgcactactgatgtaaagtggaatgaaacatggagtaataaatcttataatgagatctgggataacatgacatgg
atgcagtgggacaaagaagtcagaaattacacagaaaccatttatgatttaatagaacaagcacaaaaccaacag
gagtacaatgaaaaaagtttgttagagcttaaccagtgggcaagcctgtggaattggtttgatatatcaaattgg
ctgtggtatataaaaatattcattatgatagtaggaggattaataggctgtagaatagtttttgctttgctatct
atagtaaatagagtcaggcagggatactcacctctgtcatttcagggtgttctacagtattggggaagggaacta
aaagttagtgctataagcttgcttgatacaacagcaatagcagtagcagaaggaacagataggataatagaagta
gcacaaagatttggtagaggcatacttaatatccccacacggattagacaaggattagaaagagctttgctataa
>#62 AJ302647 2280 nt
caacactatgcaacagtctatgctggggtacctgtatggaagaggcaaccccagtactattctgtgcttcagat
gttaacttaacgagcactgagcagcataatatttgggcatcacaagcctgtgttccaacagacccctctccatat
gaatatcctttaaaaaatgtaacagataacttcaatatatgggaaaattacatggtagaacaaatgcaggaagat
attattagtttatgggaacagagtttaaaaccttgtgttcaaatgacttttctgtgtgtacaaatgaattgtaca

aatgtaaatgatgagaccaacagctccgtaaagaatgataccagcagctcagagaaccttatgaaaaagtgtgag
tttaatgtaaccacagttctcaaagacaaaaaggagaaacaacaggctctattctatgtatcagatttgatgaaa
gtgaatgaaaataatgacacaatgtatacattaattaattgtaattccacaaccattaagcaaacctgtccaaag
gtatcgtttgaggcaattccaatacactattgtgctccagcgggatatgccatctttaagtgtaacaacacaggg
tttaatggaacaggcccatgcacaaatgttacagtagttacttgtacacatggcatcagaccaacagtaagtact
caactaatattaaatgggacaatctctgaaggaaagataagaattatgggaaaaaatatttcagacactgggaaa
aatatcatagtgaccataaattctactataaacatgacctgtgagagaccaggaaatcagacagtacaaaagata
ctaacaggtccagtggcctggtacagcatgggcctgaaaaataacctaactaactcaagggcagcctcttgcaag
tataacagctctgtttgggaagaagccttaaaacaaacagctgaaaggtatttagaacttatgaacaatacaaat
acagttaacataacattcaatcacagcactggtggagacccagaggtaacccatttgcattttaactgtcatgga
gaattcttctattgtaacacatctcagatgtttaattataccttttcatgtacaagaaccaactgtattagacaa
agtaacagtagcattaatggcacaatatcttgcaggataaaacaggtggtaaggtcatggatacagggagggtcg
ggactttatgcacctcccaggccaggttacctaacatgcaactcatccataactggaatgattctacaattggat
aagacatggaaccgcaccaacaacagcgaatccacatttagaccataggggggagatatgaaagatatatggaga
actgaattgttcaaatacaaagtagtaaagataaaaccttttagtgtggcacctacaaaaattgcaagaccagtc
ataggcactggcactcgaagagaaaaaagagcagtaggattgggaatgctgttcttggggggttctaagtgcagca
ggaagcactatgggcgcagcggcaacaacgctggcggtacagacccacactttgatgaagggtatagtgcaacag
caggacaacctgctaagagcaatacaggcccagcagcaattgctgaggctatctgtatggggtatcagacaactc
cgagctcgcctgctagccttagaaaccttaatacagaaccagcaactcctaaacctatggggctgtaaaggaagg
ctagtctgctacacatcagtaaaatggaacaggacatggacaaataataatactgatttagatacaatttgggga
aatctaacatggcaggaatgggatcagcagataagcaacataagcgccaccatatatgatgaaatacaaaaggca
caagtacagcaggaacacaatgagaaaaagttgctggagttagatgaatgggcttctatttggaactggctggac
ataactaaatggttgtggtatataaaaatagcaataatcatagtaggggcactaataggggtgagaattgttatg
atagtacttaatctagtgagaaacattaggcatggatatcaacccctctcatttcaggcttgcagaatttgcata
gctgtaatacaatactggctacaagagttgcagaatagtgctacaagcctactagatacccttgcagtggcagtt
gccaattggactgacggtataattttagggctacaaaggataggaagaggaattcttaacatcccaagaagaatt
agacagggcttagaaagagctttattgtaa
>#63 AJ302646 2313 nt
cagcactatgcaacagtctatgctggggtacctgtatgggaagaggcaaccccagtattattctgtgcttcagat
gctaacctaacaagcactgagcagcataatatttgggcatcacaagcctgtgttcccacagacccctctccatat
gaatatcctttaaccaaggtgacagataatttcaatatatggaaaaattacatggtagaacaaatgcaggaagac
attattagtttatgggaacagagcttaaaaccttgtgttcaaatgactttcctgtgtgtacaaatgaattgtaca
aactatgtacaagggaattatacaaacaatagctccataaataatgataccagcagtccagagaaccttgtgaaa
cagtgtgaatttaatgtaaccacagttgtcaaagacaaaaaggagaaaaaacaggctctattctatgtatcagat
ttgatgaagataaatgaagcaaatgacacaaaggacatgtatacattaattaattgtaactccacaaccattaag
caagcctgtccaaaggtatcgtttgagccaattccaatacactattgtgctccagcgggatatgccatctttaag
tgtaacagcacagagtttaatggaacaggcccatgcaacaacattacagcagttacttgtacacatggcatcaaa
ccaacagtaagtactcaactaatattaaatgggacactctctgaaggaaacataagaattatgggaaaaaatatt
tcagacaatatgaaaaatatcatagtgaccctaaattctactataaacatgacctgtgtgagacaaggaaatcag
tcagtacaagagatacaaataggtccaatggcctggtacagcatgagcctggcccaagagggaaaaccaaataac
tcaaggatagcctattgcaagtataacatctctgattggaaaaagccttaaaacagacagctgaaaggtattta
gaccttaggaacaatacaaatacagttaacataacattcgagcgcagcattggtggagattcagaggtaacccat
ttgcatttcaactgtcatggagagttcttctattgtaacacatctaagatgtttaattataccttttcatgtata
ggaaccaactgtactagtaaccaaaatagcagcaacagtaatgacactcggatatattgcaggataaaacaggtg
gtaaggtcatggatacagggagggtcaggactctatgcacctcccaggaaaggtaacctaacatgcagctcactc
ataactggaatgattctacaattggatatgccatggaacagcaccaacaacagcaacgccacatttagaccaaca
gggggagatatgaaagatatatggagaactgaattgttcaaatacaaagtagtaaaggtaaaaccttttagtgtg
gcacctacaaaaattgcaaggccagtcataggcactggcactcaaagagaaaaaagagcagtaggattgggaatg
ctattcttaggggttctaagtgcagcaggaagcactatgggcgcagcggcaacagcgctggcggtacagacccaa
tctttgatgaagggtatagtgcaacagcaggacaacctgctaagagcaatacaggcccagcagcaattgctgagg
ctatctgtatggggtatcagacaactccgagctcgcctgctagccttagaaaccttaatacagaaccagcaactc
ctaaacctatggggctgtaagggaaagctaatctgctacacatcagtaaaatggaacacgacatggacaaattgc
acaaataccaataagttagatgatatttgggacaagctaacatggcagcaatgggatcagcagataagcaacgta
agttccatcatatatgaggaaatacgaaatgcacaagtacagcaggaacaaaatgagaaaaagttgctggagtta
gatgaatgggcttctatttggaactggctggacataactaaatggttgtggtatataaaaatagcaataatcata

gtaggggcactaataggggtgagaattgttatgatagtacttaatctagtgaaaaacattaggcagggatatcaa
cccctctcatttagagcttgcagactttgcatagctgtaatacaatactggctacaagagttgcagaatagtgct
acaagcctactagataccattgcagtggcagttgccaattggactgtcacaataattttagggatacaaggata
ggaagaggaattcttaacatcccaagaagaattagacagggcttagaaagaagtttattataa
>#64 AY169812 2256 nt
cagctgtatgcaacagtttatgctggggtacctgtatgggaagaggcagccccagtattattctgtgcctcagat
gctaacctaacaagcactgagaagcataatgtttgggcatcacaagcctgtgttcccacagaccccactccacat
gaatatttattaaaaaatgtgacagatarcttyratatatggaaaaattacatggtagaacaaatgcaggaagac
attattagtctatgggaccagagtttaaaccttgtgttcaaatgactttcatgtgtgtacaaatgaattgtaca
gacatagaaaacaaaaacagtagctcagaaaccctatgaaaaagtgtgagtttaatataaccacagttctcaag
gacaaaaaggagaaaaaacaggctttattctatagagcagatttgactgaaataagagacaacagcacaaacagc
acaatatatacattaattaattgtaattccacaaccattaagcaagcctgtccaaaggtatcttttgagccaatt
ccaatatactactgtgctccagcaggatatgccattttttaagtgtaacaatgcagaatttaatggaacaggcaaa
tgcaacaacatttcagtggtgacttgtacacatggcatcaagccaacagtgagtactcaactgatattaaatggg
acactctcaaaagaaaaaataagaattatgggacaaaatatttcggccactgcacaaaatatcatagtgaccta
agttctgctgtaaacataacctgtgtgagaccagggaataagacagtacaagagatgagaataggtccgatggcc
tggtacagcatggccataagtggaacaaacagctcaagggtagcctattgccagtataacaccactgagtgggaa
aaagccttaaaaaacacagctgaaaggtatttggaacttataaataagacagaagagaatacaacaataatgttc
aaccggagccatgatggcgatgtagaggtaacccatttgcattttaactgtcatggagagttcttctattgtaac
acatctgggatgtttaattataccttttttatgtaatgggaccaactgtaaaaaccaaagtacccatagcaataat
ggcacgataccttgcaagttgaaacaggtggtaagatcatggatgaggggagggtcgggactctatgcacctccc
atcccaggtaacctaacatgcatgtcacacataactggaatgattctacaaatagatgggccatggaacaacaac
accacaaacaatacatttagaccaataggggggagatatgaaagatatatggagaaatgaattgttcaactacaaa
gtagtgagggtaaaaccttttagtgtggcacctacatcaattgcaaggccagtcataggcactggcactcataga
gagaaaagagcagtaggattgggaatgctattcttggggggttctaagtgcagcaggtagcactatgggcgcagcg
gcaacagcgctgacggtacagacccactctctgataaagggtatagtgcaacagcaggacaacctgctaagagca
atacaggcccagcaggaattgctgaggctatctgtatggggtatcagacaactccgagctcgcctgctagcctta
gaaaccttcatacagaatcagcaactcctaaacctatggggctgtaarggaagactaatctgctacacatcagta
aaatggaacgasacatggagwrgtacaactaatatagaccaaatttggggaaacttaacatggcaggaatgggat
cagcagatagacaacgtaagtgccrccatatatgaggaaatacaaagggcacaagtacagcaggaacaaaatgag
aaaaagttgctggagttagaygaatgggcttctctttggaattggcttgacataactaaatggttgtggtatata
aaaatagcaataatcatagtaggagcactaataggtgtgagaattgtcatggtagtgcttaatctagtgagaaac
attaggcagggatatcaacccctctcgttacaggcttgcagaatttgtgcagctgtaatacatttctggctacaa
gaattgcagaagagcgctactagcctactagatacctgtgcagtagcagttgctaattggactgacggcataatc
ttagggrtacaaaggctaggaagaggaattcttaatatcccccgaagaataagacagggcttggaaagaagttta
ttgtaa
>#65 AY169816 2280 nt
cagctgtttgcaacagtctatgctggggtgcctgtatgggaagatgcagcaccagtattattctgtgcttcagat
gctaatctaacaagcactgagcagcataatatttgggcagcacatgcctgtgttcctacagatccctctccatat
gaatatccattggacaatgtgacagattattttaatgtatggaaaaattatatggtagaacaaatgcaggaagac
attattagtttatgggaacagagtcttaaaccttgtgttcaaatgactttcctgtgtgtacaaatgaattgtacc
aacttaaatgacacagtaaatggaaccagcagctcagaaagcgatatgcaaaggtgtgagtttaatgtaaccact
gttgtaaaagacaaaaaggagaaaaaacaggctttattctatagatcagatttgatggaattaaaggataaggat
aatagcacaaacacaacaatgtatacattaattaattgtaactccacaaccgtcacgcaagcctgtccaaaggta
tctttccagccaattccaatacactattgtgctccagcaggatttgctatctttaagtgtaacagcacagaattc
aatggaacaggcacatgcgaaaacataacagtagttacttgtacacatggcatcaagccaacagtaagtactcaa
ctaatattaaatgggacactctctaaaggaaaaataagaatgatgtcaacaaatatttcagacagtgggaagaat
atcatagtgaccctaaattctaccataaacatgacctgtagtagaccagcattggaagtacagagcatgggcgta
ggtccaatggccgtatacagtgcacacctaagggaatcagggacaaacggatcaaggatagcttactgtgagtat
aataccagtgattgggaaaaagcattaagacaaacagctgagaggtatttagaacttgtaaacaacacaggtagt
attagcatgacattcaacaaaagcaacgatggtggagatccagagacaacccatttacattttaattgtcatgga
gaattcttttattgtaacacatctaatttatttaattataccttttcatgtaatggaaccacctgtaatgatagc
caaagtaccaccaatgacacacagataccttgcaaactgagacaggtagtaaggtcatggataaaggacagtca
ggattctatgcacctcccatcaagggtaacctgacatgtacatcaaacataacaggaatgattctacaaatggat
gcaccatggaacagcaccagaaacaacagcgaaaaccaaactgcaacatttagaccaacaggggggagaaatgaaa

gatatatggagaactgagttgttcaactacaaagtagtaagagtaaaacctttagtgtggcacccacatcaatt
gcaaggccagttgtaggcactagcactcatagagaaaaaagagcaataggattaggaatgctattcttggggtt
ctaagtgcagcaggtagcactatgggcgcagcggcaacagcgctgacggtacagacccacactttgataaagggt
atagcgcaacagcaggacaacctgctaagagcaatacaggcccagcagcaattgctgaggctatctgtatggggt
atcagacaactccgagctcgcctgctagccttagaaaccttaatacagaatcagcaactcctaaacctgtggggc
tgcaaaggaaagctagtctgctacacatcagtaaaatggaatgaatcatggacaggaaacgaaagcatttgggaa
gaactaacatggcaggaatgggatcggcagataagaaacataagctccaccatatatgaggaaatacaaaaggca
caagtacagcaggaagtaaatgagagaaaggtgctggagttggatgaatgggcttctctttggagttggcttgac
ataactaaatggttgtggtatataaaaatagcaataatcatagcaggagcactaataggcctgagagttatcatg
atagtacttaatctagtgaagaacattaggcagggatatcaacccctctcgttgcaggcttgcagaatttgtgca
gctgtaacacaatattggctacaagaattgcagaatagtgctacaagtttgctagatacagttgcagtggcagta
gccaattggactgacggtataatctcagggatacaagcaataggaagaggaattcgtaacatcccaactagaatt
agacagggcttagagagaagtctattgtaa
>#66 KU168282 2301 nt
ccacaaatgtatgcaacagtctattctggggtaccggtatgggaagatgcaaaaccaacactattctgtgcttca
gatgttaacttgacaagcacagagaaacataatatttgggcatcacaagcctgtgttcccacagaccctactcca
aatgaatatcgtctagagaatgtgacagataaatttgatatatggaaaaattatatggtggaacaaatgcatgaa
gacattattagtttatgggatcagagtttaaagccttgtgtgcagttgactttcttatgtgtacaaatgaactgt
acagatgtaaaatcaaatacaacaaatacaaccatcctaaaagaagaagcaacagccataaaaagctgtagcttt
gatgtaactacagttctcaaagacaaaaaggagaaaaaacaggctctatttatgtaacagatctggctaaggtt
gacccaacagatccaaattcaacagatacaacaacctatacattaattaattgtaactccacaaccatcaggcaa
gcctgtccgaaggtaagctttgagcccatccctatacattattgtgctccagcaggatatgccatctttaagtgt
aatgaaacaaactttaatggaacaggcacatgccacaatgttacaatggttacttgtacacatggcatcaagcca
acagtaagtactcagctaatattgaatgggacaatatctaaagacaaaacaagaattatgggacaaaatatcaca
gacagtgggaagaatatcataataaccctaaatactactataaaaatgaattgcacgaaagaaggaaataatgag
atacaagaaataaaaatagggcccatgtcatggctcagtatggcagttactgcaaatgaaactggctcaaaatca
aggatagcttattgtaattatagtagcaccgaatgggaaaaggccttaaaactaacagctgaaagatatgtacaa
cttttaaacataacagaaaaagttaccataacattcaaaaatagcagtggggggagatgcagaaataagccgtttg
cattttaactgtcatggagaattcttttattgtaacacatctaagatgtttaactatacttttgactgtacaagt
ggcaactgtactgagatcaattccatgaataaaactgatgagcataatggactaccttgcaggttaagacaggta
gtaagatcatggataaagggagagtcaggactttatgcacctcccatcagaggcaacctcacatgccagtccaac
ataactggaatgattctacaactagatgaaccatataaccgcacatcagatgcaaatgtcacacttagaccaagt
gggggagacatgcaagatatatggagagctcaattgtacaactacaaagtagtacagataaaacctttagtgta
gcacctacaagaatctcaaggccaacaataaaccttaacacccctcgcagagaaaaaagagcagtaggattggga
atgctgttcttgggggcgctaagccttgcaggtagcactatgggcgcagtggcaacaacactgacggtacagacc
cacagtgtactgaagggtatagtgcaacagcaggacaacctgctgagagcgatacaggcccagcaacacctgctg
aggctatctgtatggggtatcagacaactccgagctcgcctgcaagccttagagacccttgtacagaatcagcaa
cgcctaaacctatggggctgtaagggaaggataatctgttacacatcagtaaaatggaacacatcatggggagat
tacaatgacagtatttggaaccactatacatggcagcaatgggaccaacaaatagataatataagctccattatc
tatgatgaaatacaagcagcacaagaccaacaggaaaggaatgtaaaggcactgctggagctagatgaatgggcc
tctctttggaactggtttgacataactaaatggctgtggtatataaaaatagctataatcatagtaggagcactg
ataggtataagagttatcatggtagtacttaatctagtgaggaacattaggcagggatatcaacccctctcgttg
caggctttcaaactttgtggagctgtaacacaatactggctacaagaattgcagactagtgctataaatctgttt
gatacttttgcagtggcagttgctaattggactgacagctttatcttaggtatacaaagaataggacgaggattc
cttaacatcccaagaagggttagacaaggcgcagaaatagcattattgtaa
>#67 AY169809 2286 nt
cagttgtatgcaacagtctatgctggggtgcctgtatgggaagaagcaaacccaatactattctgtgcttccgat
gctaacttaacaagcactgagaagcacaatatttgggcagcacaagcctgcgttcccacagaccccactccacat
gaatatccattatccaatgtgacagataacttcaatatatgggaaaattacatggtggaccagatgcatgatgat
atcactgatttgtggaagcagagtttaaagccatgtgttgaattgactttcctgtgtgtacaaatgaattgcaca
gatgttactcatactaataatgtaaccatctcgcaggctgtaaccagccccgtaaaaaagtgtgatttcaatgta
accacaattttcaaagacaaaagggagaaaaaacaggctctattctataaagcagatttgcttgaaacaaaagaa
ttagaatcaaacagaacaatgtatacattaattaactgtaactccacaaccatcaagcaagcctgtccaaaagta
acttttgaacctattccaatacactactgtgctccagcaggctatgccatctttaagtgtaacaatagcgaattt
aacggaaaaggtgcatgcagtaatattacagtagttacttgtacacatggcatcaagccagcagtaagtactcaa

ctaatactaaatggacactctctaaaggaaaaataagaattatgggaaaaaatatttcagacagtgggcaaagt
atcctagtgactctaaattctactataaacataacctgtgagagaccaggaaatcggaaaagagaggagataaaa
ataggtccaatggcttggtacagtatgtcagtagaaaacaacataacatcaagggaagcttattgcacatataat
gtcagtaaatgggcaacagccctaaagcaaacagctgagaggtatctagaacttgtaaacaatacacaggctagc
ataacattcaatcaaagcagtggtggagatccagagataaccaatttacatttcaattgtcatggagaattcttt
tattgtaacacaacggggttgtttaattatacctttctgtgtgaaaactccatctgtaacttcacccataacaat
acttcagaggagcgcaatctaacttgtagaattagacaggtggtaaggtcatggatgaagacagggtcgggactc
tatgcacctcccatcgaaggtaacctaacatgtaggtcaaacataactggaatgattctagaaatggataaacca
tggaacaaaagtacaaacaatgaaacagccatatttagaccaataggggagatatgaaagaaatatggagaact
gaattgttcaactacaaagtagtaagggtaaaacctttagtgtggcacctacaaaaattgcaaggccagtcata
ggcactggcactcctagacagaagagagcagtaggattgggaatgctattcttggggggttctaagtgcagcagga
agcactatgggcgcaacggcaacaacgctggcggtacagacccacactttaatgaagggtatagtgcaacagcag
gacaacctgctaagagcaatacaggcccagcagcatttgctgaggctatctgtatggggtattagacaactccga
gctcgcctgctagccttagaaacctttatacggaatcagcaattcctaaacctgtggggctgtaagggacaatta
gtctgctacacatcagtaaaatggaacacaacatggggaagcaatattcctaatattactaatctrgaggaaatt
tggggcaaactaacctggcaggaatgggatcagcaggtaagcaatgtaagctccatcatatatgaggaaatacaa
aaggcacaagtacagcaggaacaaaatgagaagaagttgctggagttaaatgaatgggcctctatttggaattgg
cttgacataactaaatggttgtggtatataaaaatagctataatcatagtaggagcactagtaggtgtgagagtt
gtyatgataatacttaatctagtgagaaacattaggcagggatatcaacccctatcgttacaggcttgtagaatt
ggtagagctgtaatacaatactggctacaagaattgcagaatagtgctataagtctggtagataccttggcagtg
gtagttgccaattggactgacggcatcattttaggactacaacgcataggaagaggaattcttaacatcccaaca
agaattaggcagggcctagaattaggtttattgtaa
>#68 KU168283 2226 nt
cagctgtatgcaacagtctatgctggggtacctgtatgggaagaggcaaccccagtattattctgtgcttcagat
gcgaacctaacaagcactgagcagcataacatttgggcatcacaagcctgtgtccccacagatcccactccatat
gaatatccattaaagaatgtgacagataacttcaatatatggaaaaattacatggtagaacaaatgcaggaagac
attattagtttatgggaacagagcttaaaaccttgtgttcaaatgactttcctgtgtgtacaaatgaattgtaca
gacataatgaaacagtgtgcctttaatataaccacagttctcaaagacaaaaaggagaaaaaacaggctctgttc
tatgtatcagatttacataaattctatgataacgagacaaatagcacaatgtatagattaactaactgtaattcc
tcaaccattaggcaagcctgtccaaaggtatcttttcagccaattccaatacactattgtgctccagcaggatat
gccatctttaagtgtaacagctcagaatttaatggaacaggtacatgccgcaacattacagtagttacttgtaca
catggcatcaagccaacagtgagtactcaactaatattaaatggacactctctgaaggaaaaatcagaattatg
gggaaaaaatatctcggacagtggcaaaagcatcttagtgaccctaaattctactataaacataacctgtgagaga
ccatggaatctgtcattacaagagatacgcataggtccaatggcctggtatagcattgctttacagacacaaccc
caggcaaggttagctcattgcacgtataataccactgattgggaaaaagccttaaaacaaacagctgaaaggtac
ttagaacttgtaaactatacaagtggtaatgttaacataacattcaatagaagcaatgakggaggagatgcagaa
ataacccagctacattttaactgtcacggagagttcttctattgtaacacatctagtctgtttaattatagcttt
tcatgtaacggaaccaactgtactattaaccaaactaaaaatgagacttggataccttgcaggataaaacaggta
gtaaggtcatggatacagggagggtcaggactctacgcacctcccatccgaggtgcactaaaatgtgagtcaaac
ataactggaataattctagaaatggatcagccatggaataaaagcagcccagatgtcacatttagaccataggg
ggagacatgaaagacatatggagaactgaattgttcagatacaaagtagtaaaggtaaaacctttagtgtggca
cctacaaaaattgcaaggccagtcataggcactaacactcatagagaraaaagagcagtaggattgggaatgcta
ttcttggggggttctaagtgcagcaggaagcactatgggcgcagcggcaacaacgctggcggtacagacccacact
ttgatgaagggtatagtgcaacagcaggacaacctgctaagagcaatacaggcccagcagcaattgctgaggcta
tctgtatggggtatcagacaactccgagctcgcctgctagccttagaaaccttaatacagaatcagcagctccta
aacctatggggttgtaagggaaggataatctgctacacatcagtaaaatggaacagtacatggggaaatgtcacc
agtatgagtgaagtttgggacaaactaacctggcaggaatgggatcagcagatagacaacataagcaatgttata
tttgatgaaatacaaagagcacaagtacagcaggaacaaaatgagaagaagttgctggagttagatgaatgggct
tcaatttggaattggcttgacataactaaatggttgtggtatataaaaatagcaataatcatagtaggagcacta
ataggtgtaagaattgtcatggtagtacttaatctagtgagaaacattaggcagggatatcaacccctctcgttt
caggcttgcagaatttgtgcagctgtaggacaatactggctacaagaattgcagaatagtgctacaaacctatta
gataccattgcagtggcagttgccaattggactgacggcataatattaggaatacaaaggataggaagaggaatt
cgtaacatcccaaggagaactagacagggcttagaacaaagtttattgtaa
>#69 AY169813 2250 nt

aatctatatgcaacagtctattctggggtgcctgtgtgggaagatgcaascccaacattattctgtgcttcagat
gttaaccttactagcactgagagccataatatttgggcatcacaggcctgcgtccccacagacccctcaccacat
gagtatccactaattaatgtgacagatagattcaatatatggaagaattacatggtagatcaaatgcaggaagac
attattagtttatgggagcagagtctaaaaccttgtgttcaaatgactttcctgtgtgtacaaatgaattgtaca
gatgtcaatcaaacattgagtaatgcgacacaagggatggaagtaaaaaagtgtgaatttaatgtaactacagtt
ttaaaagacaaaaaggaaaagaaacaggctctattctatgtatcagatttggctaaggttcaagatagtggtcag
aatggaacatatacattaattaattgtaattccacaaccatcaagcaagcctgcccaaaggtttcttttgagccc
atcccaatacattattgtgctccagcaggatatgccatctttaagtgtaatgacacagcatttaatggaacaggc
ttatgcaagaacatttcagtagttacttgtacacatggtatcaagccaacagtaagtactcaactaatactaaat
ggaacaatctctgaaggaaacataagaatgctgggaaaaaatctgacaagtaatgagaatatcctagtgactcta
aattccactataagcataacctgtgagagaccatacaatcagtcaatacaagagttaaggataggtccaatggct
tggtacagcatgacattagaacaaaacaaaacaggcagtaacataaggacagcttattgcaattataatggctct
gactgggaaggtacattaaaaggagtagctgaaagatatttagaacttataaatcagacaaacaatgctaccgtg
acattcagtaatagtacgggtggagatccagagataagcttttttgcattttaactgtcatggagaattcttttat
tgtgacacaagtaagatgtttaattataccttttaattgtaacaagaccaaatgtaataatagtaaaaatagtacc
cataataacactatgacaacaataccttgcagaataaaacaggtagtaaggtcatggataagggggagagtcggga
ctctatgcacccccccattaaaggtaatttaacctgcatatcaaacataactggaatgattctattaagggacgag
ccagggaataacagcagacacacccttaggccagtaggggggagatatgaaagatatatggagaactgaattagtc
aattacaaagtagtaaaaataaaacctttttagtgtagcacctacaaaaattacaaggccagtcataagcacccat
agagaaaaaaagagcagtaggattgggaatgctattcttaggggttctaagtgcagcaggtagcactatgggcgca
gcggcagtgacgctgacggtacagacccaaactttgttgaagggtatagtgcaacagcaggacaacctgctaaga
gcaatacaggcccagcaacagttgctgaggttatctgtatggggtatcagacaactccgagctcgcctgcaagcc
ttagaaacctttatacagaatcagcaactcctaaacctctggggctgtaagggaaagctaatttgctatacatca
gtaaaatggaacaaaacatggggaggagatgagtcaatctggggtaacctaacatggcagcagtgggatcagcag
atagagaatgtaagctccatcatatatgaagaaatacaaaaggcacaagaacaacaggaacaaaatgagaagaag
ttgctggaattagatgaatgggcctctctttggaattggcttgacataactaaatggttgtggtatataaaaata
gctataattatagtaggagcactaataggtgtgagaattatcatggtaatacttagtctagtgaagaacattagg
cagggatatcaacccctctcgttacaggcctgcagacaaacaagagctttattacaatattggctacaagaattg
caaaaaagtgctacaagcttgctagataccattgcaataatagttggcaattggactgatagtatcatcttagga
ctacaaagaataggaaggggaattcttaacatcccaagaagaattagacagggccttgaacgctgcttagtgtaa
>#70 AY169805 2334 nt
ctgtatgcaacagtctatgctggggtacctgtatgggaagaggcagaaccagtattattctgtgcctcagatgct
aacctaacaagcactgagcagcataatgtttgggcatcacaagcctgtgtccccacagaccccactccacatgag
tatccattacgcaatgtgacagataaatttaatatatggaaaaattacatggtagaccaaatgcaggaagacatt
attagcctatgggaacagagcttaaaaccttgtgttcaaatgactttcctgtgtatacaaatgaattgtacagac
atacaaatcaattctaaagaaaacaactctacaaacgatagctcaataaaccctataagcacagagaaccctaac
agcatagagaaccctatgaaaaattgtgagtttaatgtaactacagttctcaaagataaaaaggagaaaagacag
gctctattctatgtagcagacttggatagagtgaataatagtgaaataggtaacacagcatatacattaattaay
tgtaactccacaaccatcaagcaagcctgtccaaaggtatcttttgagccaattccaatacactattgtgctcca
gcaggatttgccatcttcaagtgtaacagcacagagtttaatggaacaggcacatgccacaacattacagtagtt
acttgtacacatggtatcaagccaacagtaagtactcagctaatattaaatggaacactttcggaaggaaagata
agaataatagcaaaaaatatttcagctactggcaacaatatcatagtgaccttaaatactactataaacatgacc
tgccagagaccaggacatcaagatgtacaagagataatgacaggtccactggcctggtatagcatggaactaaag
aaagacacaaacacatccaaatcaagaatagcttattgtgagtataataccaagaagtgggaagaggccttaaga
cagacagctgaaaggtatttagaattagtaaacaatacaggtgttagagaaatgatattcaattacaatactgga
ggagatccagagataacccagttgcattttaattgtcatggagagttcttttactgcaacacatctcagatgttt
aattatatctttaactgtaccggaaccacctgtaattataccaaaaacatccaaaattatacctttagccaaaat
accactagcactaagataccttgcagattgaggcaggtagtaaggtcatggatgaggggaggatcggaccctat
gcacctcccatccgaggtaacttaacatgtggatcaaacataactggaatgattctagaaatggatgagccatgg
aacgagagccacagggaaagcatatttagaccaacaggggaaatatgaaagatatatggagaactgaattgctc
agatacaaagtagtaaggataaaacctttttagtgtggcacctacaaaaattacaaggccagtcataggctttggc
acccgcagagaaaaaagagcagtgggcttgggaatgctattcttggggggttctaagtgcagcaggtagcactatg
ggcgcagcggcaacagcgctgacggtacagacccacactttgataaagggtatagtgcaacagcaggacaacctg
ctaagagcaatacaggcccagcagcacttgctgaggctatctgtatggggtatcagacaactccgagctcgcctg
ctagccttagaaaccttaatacagaatcagcaactcctaaactcatggggctgtaaaggaaggctagtctgttac

acatcagtaatatggaacacatcagaatggaaaggaaatgagtctatttggggcaacctcacatggcagcaatgg
gatcaggagatagacaatgtaagtgccaccatatttgaagaaatactaaaagcacaaatacagcaggaaacaaat
gagaaaaagctgctggagttagatgaatgggcttcyatttggaattggcttgacataactaaatggttgtggtat
ataaaaacagcaataatcatagtaggagcactaataggtataagaattgtcatggtaatacttaatttagtgaga
aacattaggcagggatatcaacccctctcgttccaggcttgcagaatttgtgcagctgtaatacaatactggctt
caagaattgcagaatagtgctacaaatctagtagatactattgcagtagcagtagctaattggactgacagtata
atcttagggatacaaagaataggaagaggaatctggaacgtccccaggagaattagacagggatttgaaagaagt
ttgttataa
>#71 HQ179987 2349 nt
gctattagctatactagtggaacacgttatgttactgtatattatggggttccagtttggagagatgctaaaatt
accttattctgtgcagcagatgcatcgctaactagcarwgagcagcataatatttggccacccaggcctgtgtg
cccacagaccctagaccaatagaggtcaggatagataatgtaacagagtcttttaatatttggacaattatatg
gtgacacaaatgcaggaagacatcattagcttatgggatcagagccttaaaccttgtgtaaaattgacagttcta
tgtgttactatgrattgtagcgactgcagcacggttgactgtactaataactcctccagggttaataacagcacc
gacagcaccaacaccagcaaaaccaatccattagaattatttcagtgcagttttaatacaaccacaatagtaaaa
gataaacagcagacacagcaagcactcttttatagagcagacctaacaaaattggataatgacaatagtacatat
agattaattaattgcaacaccactaccattacacaggcatgcccaaaagtgaactttgagccgctacccatacag
tattgtgcaccagcagggtatgcactaatgaaatgcaatcagacaggatttaatggtacaggaccttgtaataag
acagttataacacactgtacacatggaattaagccaacagtgtcaacacaattaatactcaatggaactctagca
aagggagagcccttagtaattactcagaatgtgtcagatacaggaaaagtcatcatagtaaaattaaatgagagt
gtgtcactcacctgtataagaccaggtaataacacaagaggacaagtgcaactaggagtcatgacatggtataat
atgaaacactacgtaggcgatatcagggcagctcattgtaatgtttctagggaaaattggaaaagaaccttagaa
tgggtcagtgaggccatctggaaggcttatcctcaacattccaaccatacatccaaacacaaccatacatttgtt
tttaaaaacagtacaggggggagacccagaggtttcttcgctgcacttcagttgtcatggggaattcttttattgt
aacaccagcagcctgtttaactttagttatacttggaatgacaccacctggcgggctgcaggcaatcataataca
agtgaaaatataacactgtcttgtagactgaagcaagtagttaattcatggatgagagtgggatcaggcttgttt
gcgacacctattgaggggggaattaaagtgtcaatccaacattacaggtttaatgttagaaagagaactaccttac
aatgacagcagcaagaacaccactttaagtcctataggggggagacatgacaaatatctggagaagtgagttatat
ccctacaaagtagttcaagtaaaagctctggccgtggcacctacaaaaatttccagacctacaattatggctcat
gatggacataggaagaaaagggcagcaggcctaggaatgctattccttgggtttctgagcgcagcaggaagcact
atgggcgcagcgggaatcacgctgacggtacaggccaagcaattattgcatggtatagtgcagcagcagaacaat
atgctaagagctatagaggcacagcaagaattgctgagactctctgtgtggggcataagacagctccgagctcgc
ctgcttgccattgaaacctatttaagggatcagcaactcctaggactgtggggatgctcaggacaaatartatgt
tatactaatgtgccatggaatagtacctggaccaacaaaaatgaaacagaattagatggcatttggggtaatcta
acatggcaggagtgggacaaactggtggataattacactgacacaatttacttggaaatacagaaagcacaagag
cagcagaaggaaaatgaaagaaagctattagaattagacaaatgggcacaattgtggagctggatggacataaca
aaatggttgtggtacataaaaattttcattatgatagtaggaggaattataggactaaaaatcttaatggctata
ggtaatgtagtcaggaaagtcaggcagggataytcacctgtrtcgttgcagtgcctgcgagactgctttgctgcg
tgtggctattggactcaagaattaaaacaragtgcaaccagcytgttgaacactgttgctatatcagttgcaaat
tggactgatcaagtaatagcagtagggcaacaaataggaagaggcttcttgaacataccaagaaggttaagacaa
gggctagaaagaagcttactgtaa
>#72 GU111555 2322 nt
gctattagctgtgctagtggaatacactatgttactgtatattatggggttccagtttggagaaatgctgaagtt
accttgttttgtgcagcagatgcatcgctaactagcaaagagcagcataatatttggccacccaggcctgtgtg
cccaccgaccctacaccaatagagatcccgataaatgtaacagagtcttttaatatttggaaaaattatatggtg
acacaaatgcaggaagacatcattagcttatgggatcagagccttaaaccttgtgtaaaattgacaatcctatgt
gtcactatgaattgtagtgaatgccacagggaggctagctgtaataataacagtagctcctctgtgaatakcacc
gccgacaatccattagatctatttaagtgcagttttaatacaaccacagtattgaaagataaaaagcagggacag
caagcactcttctatagaacagacctaatagcactgaatgagacagcaaataatacactatatagattaattaat
tgcaacaccactaccattacacaagcatgcccaaaagtgacttttgagccactacccatacagtattgtgcacca
gcagggtatgcactaatgaaatgcaatcagaaaggatttaatggtacaggaccttgtaatcagacagtcataaca
cactgtacacatggaattaagccaacagtatcaacacaattgatactaaatggaactctagcagaggaagagccc
ttagtaattactcaaaatgtgtcagacacaagatatgttatcatagtaaaattaaataagaatgtgtcaatcacc
tgtgtaagaccaggtaataacacaagaggacaagtgcaaataggacccatgacctggtataatatgaaattctac
acaggtgatatcaggaaagcttattgtaacatttccatgcaaaattggacagaaaccttaaaatctgtcagtgag

gccatctggaaagcttatcctcaacccccccaaccaaaaccatacatttgtctttcgaaacagtacagggggagac
ccagaggtttcttttctgcactkcaattgtcatggggaattcttttattgtaacaccagtaccctgtttaattat
agctatacttgtaatacctcgggtgcatgtacaggtaaacacacaggtgagaacatgacaataacactgccttgt
agactaaagcaagtagttaattcatggatgaaagtgggatcaggcttgtttgcgccacctatcgagggacaattg
cagtgccactccaacattacaggtttaatattagacagagcatcaccttataatgccaacagcagcaacaccact
ttaagccctacaggggggagaaatgagaaatatctggagaagtgagttatatccctacaaggtagttcaagtaaag
gctctggctgtggcacctacaagagtctccagacctacaatcatggctcatgatgctcataggaaaaaaaggga
gcaggcctaggaatgctattccttgggtttatgagtgcagcaggaagcactatgggcgcagcggcagttacgctg
acggtacaggccaggcaattattgcatggtatagtgcagcagcagaacaatatgctacgagctatagaggcacag
caagaattgctgagactctctgtgtggggcataagacagctccgagctcgcctgcttgccattgaaacctactta
agggatcagcaactcctaggactgtggggatgctcaggacaaacagtatgttatactaatgtgccatggaatagg
agctggaccaacaaaagtgagactgaattagatggcatttggactaatctaacatggcaggaatgggacaaamtg
gtggataattacactgacacaatttatttagaaatacaacgagcacaagatcarcaaaaggctaatgaaaaaaag
ttattagagttagaccaatgggcacaattgtggaactggttggacataacacaatggttgtggtacataaagatt
ttcattatgatagtaggaggaataataggactaagaatcttgttagctataattaatgtagtcaggagaatcagg
cagggatactcacctgtatcgttacagtgcctgcgagactgctttgctgcgtgtggctattggactcaagaatta
aaacaaagtgcaaccagcttattggacactgttgctatatcagttgcagggtggactgatcaagtaataatagta
gggcaacaaataggaagaggctttttgaacataccaagaaggataagacaagggatagaaagaagcttgcta
>#73 DQ017382 2190 nt
caacattgggtaacagtgtactatggggtgccagtatggagagacacagagacagttcttttctgtgcttcagat
gctaaagcccatagtacagaggctcacaacatctgggccacacaagcatgtgttcccactgatcccaatccacaa
gaagtgccattaatcaatgtgactgaacattttgatatgtggaaaaataacatggcagaacaaatgcaagaggac
attatcagtttatgggaacagagcttaaagccctgtgttmaattaaccccattatgtgtaactatgcattgtwac
aatagcaatgggkttaatgrgacagttgacaatggggaattagatataggatacaaacaaatgaaaaattgttca
ttcaatgtaaccactgagagaaaagataaaaagaagctagcttactctctgttttatgcagaagatgtagtgcaa
ctcaatgagagtgacagtacgaatcaaacatataggctaataagttgtaaaaccacatctgtaacacaagcctgt
cctaagaccacctttgagccaattccaatacattactgtgcaccaccaggctttgccattatgaratgtaatgaa
ggaaactttagtggaaaaggggaatgtaaaaatgtgagtactgtacaatgcacacatggaataaagccaacgata
tctactcagttaatcmtaaatgggagcctagatacagatgatattgttmttagayrtsataawgataatatgttg
gtgcaatggaatmagacagtgtcaataaattgtacaaggccagggaataatayaggaggacaggtgcagatagga
cctgctatgacattttataacatagagaaaataataggggacattagacaagcacactgtaatgtctctgaagaa
tggaaatcaatgtgggatagaacaaaagagaaaataaagggcctcctgggaaacaacacaacctttaagtctcgg
gtgaacattggaggagacccagaagtaagacacttcatgttcaattgtggaggagagtttttcctttgtaacact
tccagattatttgatgagaacgggactgtaaatgggactattattctgccctgtagaataaaacaaattgtaaat
ttgtggacaagagtaggaaaaggaatttatgcaccaccaattcggggaaatattacctgtaactccagtattact
ggactaattctagaagttagtggaaatagtacagtatatccttcaggaggaaacatggttaatctctggagacaa
gagttgtacaagtacaaagtagttagcatagaacccataggagtagcaccaggtaaagctaaaagacgcacagtg
aatagagaaaagagagcagcctttggactaggtgcgctgtttcttgggtttcttggtgcagcagggagcactatg
ggcgcagcgtcaataacgctgacggtacaggcccggaacttattatctgggatagtgcaacagcagaataatctg
ttgagagcaatagaggcgcaacaacaattgttgcaactctcaatctggggcattaaacagctccaagcaaaagtc
cttgctatagaaagataccttagggatcagcaaatcctaagtctatggggctgctcaggaaaaactatatgctat
accactgtgccttggaatgagacttggagcaatcatacctcctatgattcaatctggggaaatttaacctggcaa
caatgggatgagaaagtaagaaactattcaggtgtcatttttgaccttatagagcaggcacaagagcagcagaac
acaaatgagaaatcactcttggaattggatcaatggacaagtctgtggaactggtttgatatttcaaactggctg
tggtacataaaaatagctataatggtagtagcaggcattataggcataagaatcataagtgtaataataactata
atagcaagagttaggcagggatattctcccctctcgttgcagctcagaaaacgcttgtggggaatactagcatat
tggggaaaagagttaaaagatagtgctatcagcttgcttaatacaacagctattgcagtagcaggggggracagat
aggcttatagaattagcacaaagaataggaaggggaatattacacatacctagaagaatcagacagggcctagaa
agaacactttttataa
>#74 AY532635 2211 nt
aaatattgggcaacagtgtactatggagtaccagtatggagagatgtagaaacagttcttttctgcgcctcagat
gctaaagcccatagtacagaggctcacaacatctgggccacacaagcatgtgtccccactgatcccaatccacaa
gaagtaccactagacaatgtgactgaaccttttaatatgtgggaaaataaaatggcagaacaagtgcaagaggac
attattagtttatgggaacagagcttaaagccctgtgttaaattaaccccattatgtgtaactatgaattgtagc
aatagcaatggaaatcggacaacagatgagaaagaaaaaccaggaaatgggacagacctagaagcaagacacatg

aaaaattgctcattcaatataaccactgaaatacatgacaaaaagaagcaagcttactctctgttttatgtagaa
gatgtagtgccactcaatgatgggaataatagtacatacaggctaataaattgtaataccacagctgtaacacaa
gcttgtcctaagactacctttgagccaattccaatacattactgtgcaccaccaggctttgccattatgaaatgt
aatgaagcaaattttaatggaacaggggaatgtaaaaatgtgagtactgtacaatgcacacatggaataaagcca
gtgatatccactcagttaatcctaaatgggagcttagataaagatattgttattagaaataatagtggtggaaat
ctgttggtgcaatggaatgagatagtgacaatgaattgtacaaggccagggaataatacaggaggacaggtacaa
ataggacctgctatgacattttataacatagaaaaaatagtaggagacattagacaagcacactgtaatgtctct
aacgaatggagatcaatgtggaataaaacaaaagagaaaataaagagcctcctgggaaacaacataacattcaag
gctcaggaaaagaatggaggagacccagaagtaacacacttaatgttcaattgtggaggagagtttatctattgt
aatacttccagattattcaatgagagcatgaatacaaatgggactaatgggactattactctgccctgtagaata
agacaaattgtaaatttgtggacaagagtaggaaaaggaatttatgcaccgccaattcggggaaatcttacctgt
aactccactattactggactgattctagaacatagtggtggcagtaatggcacagtatatcccacaggaggaaac
atggtcaatctctggagacaagagttgtataagtacaaaacagtcagtatagaacccataggagtagcaccaggt
aaagctaaaagacgcacagtgagtagagaaaaaagagcagcctttggactaggtgcgctgtttctggggtttctt
ggagcagcagggagcactatgggcgcagcgtcaataacgctgacggtacaggcccggacattgttatctgggata
gtgcaacagcagaataatctggtgagagcaatagaggcgcaacaacatttgttgcaactctcaatctggggcatt
aaacagctccgtgcaaaagtccttgctatagaaagataccttagggatcagcaaatcctaagtctatggggctgc
tcaggaaaaactatatgctataccactgtgccttggaatgagacttggagcaataatacctcttatgatacwatc
tggggtaatttaacctggcagcaatgggataggaaagtaagaaactattcaggtgtcatttttgagcttatagar
aaggcacaagaacaacagaacacaaatgagaaatcactcttggaattggatcagtgggcaagtctgtggaactgg
tttagtatcacaaactggctgtggtacatcaagatagctataatggtagtagcaggcattataggcataagaatc
ataagtgtaataataactataatagcaagagttaggcagggatattctcccctctcgttgcagctcagaacacgc
ttgtggggaataatagcatattggggaaaagagttaaaggatagtgctatcagcttgcttaacacaatagctatt
gtagtagcagagggaacagacaggcttatagaattagcacaaagaataggaaggggaatattacacatacctaga
agaatcagacagggcctagaaagagcacttctataa
>#75 AJ006022 2220 nt
caacattgggtaacagtgtactatggggtaccagtatggagagaagcagagacaactcttttctgtgcttcagat
gctaaagcccatagtacagaggctcacaacatctgggccacacaagcatgtgttcctactgatcccaatccacaa
gaagtgctattacccaatgtaactgaaaaatttaatatgtgggaaaataaaatggcagaccaaatgcaagaggat
attatcagtctgtgggaacagagcttaaagccctgtgttaaattaaccccattatgtgtaactatgctttgtaac
gatagctatggggaggaaaggaacaatacaaatatgacaacaagagaaccagacataggatacaaacaaatgaaa
aattgctcattcaatgcaaccactgagctaacagataaaaagaagcaagtttactctctgttttatgtagaagat
gtagtaccaatcaatgcctataataaaacatataggctaataaattgtaataccacagctgtgacacaagcttgt
cctaagacttcctttgagccaattccaatacattactgtgcaccaccaggctttgccattatgaaatgtaatgaa
ggaaactttagtggaaatggaagctgtacaaatgtgagtactgtacaatgcacacatggaataaagccagtgata
tccactcagttaatcctaaatggaagcttaaatacagatggaattgttattagaaatgatagtcacagtaatctg
ttggtgcaatggaatgagacagtgccaataaattgtacaaggccaggaaataatacaggaggacaggtgcagata
ggacctgctatgacattttataacatagaaaaaatagtaggagacattagacaagcatactgtaatgtctctaaa
gaactatgggaaccaatgtggaatagaacaagagaggaaataaagaaaatcctgggaaaaacaacataaccttc
agggctcgagagaggaatgaaggagacctagaagtgacacacttaatgttcaattgtagaggagagtttttctat
tgtaacacttccaaattatttaatgaggaattacttaacgagacaggtgagcctattactctgccttgtagaata
agacagattgtaaatttgtggacaagggtaggaaaaggaatttatgcaccaccaattcggggagttcttaactgt
acctccaatattactggactggttctagaatatagtggtgggcctgacaccaaggaaacaatagtatatccctca
ggaggaaacatggttaatctctggagacaagagttgtataagtacaaagtagttagcatagaacccataggagta
gcaccaggtaaagctaaaagacgcacagtgagtagagaaaaaagagcagcctttggactaggtgcgctgtttctt
gggtttcttggagcagcagggagcactatgggcgcagcgtcaataacgctgacggtacaggcccggacattatta
tctgggatagtgcaacagcagaatattctgttgagagcaatagaggcgcaacaacatttgttgcaactctcaatc
tggggcattaaacagctccaggcaaaagtccttgctatagaaagataccttagggatcagcaaatcctaagtcta
tggggctgctcaggaaaaacaatatgctataccactgtgccttggaatgagacttggagcaacaatacctcttat
gatacaatctggaataatttaacctggcaacaatgggatgagaaagtaagaaactattcaggtgtcatttttgga
cttatagaacaggcacaagaacaacagaacacaaatgagaaatcactcttggaattggatcaatgggacagtctg
tggagctggtttggtattacaaaatggctgtggtatataaaaatagctataatgatagtagcaggcattgtaggc
ataagaatcataagtatagtaataactataatagcaagagttaggcagggatattctcccctttcgttgcagctc
agaacacacttgtggggaatacttgcatattggggaaaagagttaaggatagtgctatcagcttgcttaataca

acagctattgtagtagcagaaggaacagataggattatagaattagcacaaagaataggaaggggaatattacac
atacctagaagaatcagacaaggcctagaaagagcactgatataa
>#76 MF7672 2214 nt
aattattgggtaacagtgtactatggagtaccagtatggaaggaagcacataccgttcttttctgcgcctcagat
gctaaagcccatagtgcagaggctcacaacatctgggccacacaagcatgtgtccccactgatcccaatccacaa
gaagtgctattaactaatgtgactgaatattttaatatgtggaaaaataaaatggcacagcaaatgcaagaagac
attatcaacttatgggaacaaagcttaaagccctgtgtttcattaactccattatgtgtaactatgctttgtaac
gatagcaatggatggaatgagacagtaaaccakactgcaagcccggacwtgaaacaaatgagaaattgctcatat
aatgtcaccaatgagataagagataaaaagaagcaagtttactctctgttttatgtagatgatgtagtaccactc
aatgacagtgtgacaacatataggctaataaattgcaataccacagctgtgacacaagcttgtcctaagacctcc
tttgagccaattccaatacattactgtgcaccaccaggctttgccattatgaaatgtaatgaaggaaattttagt
ggaaatggaacttgtaaaaatgtgagtactgtacaatgcacacatggaataacaccaataatatccactcagtta
atcctaaatggaagcttagatacaaaggatatagttattagacatcatagagatagaggagntagaggtgatctg
ttggtgcaatggaatgagacagtgtcaatgaactgtacaaggccaggaaataattcagtaggacaggtgcagata
ggacctgctatgacattttacaacatagagaaaatagtaggagacattagacaagcacactgtaatgtctctgga
aactgggcatcaatgtggaacaaaacaaaagaaaaaatacagaacctactgggaaaaaayacaacakaattcrag
gttcgggggaaaaatggaggagacccagaagtaacacacttaatgttcaattgtagaggagagtttttcctctgc
aacactactaacttatttaatgacagcaagaatgcaactaatgggggtattactctgccctgtagaataaaacaa
tttgtgaatttgtggacaagagtaggaaaaggaatttacgcaccaccaattcggggagagctttcctgtaacttc
aatattactggactaatgctagaacatagtggtaacaccaacagtacaatattcctactatatccctcaggagga
aacatggttaatctctggagacaagagctgtataagtacaaagtagtcagtatagagcccataggagtagcacca
ggtaaagccaaaagacacacagtgggtagagaaaaaagagcagcctttggactaggtgcgctgtttctcgggttt
cttggagcagcagggagcactatgggcgcagcgtcaataacgctgacggtacaggcccggagkttattatctggg
atagtgcaacagcagaataatctgctaagagcaatagaggcgcaacaacatttgttgcaactctcaatctggggc
attaaacagctccaggcaaaagtccttgctatagaaagataccttaragatcagcaaatcctaagtctatggggc
tgctcaggaaaacttatatgctacaccactgtgccttggaatagcacctggagcaataatatctcttatgatgrc
atctggaataatttaacctggcaacaatgggatgagaaagtaaaaaactattcaggtgtcattttcgrtcttata
gagcaggcacaagaacaacaaaacacaaatgagaaatcactcttggaattggatcaatgggcaagtctgtggaac
tggtttgatattacaaactggctgtggtacataaaaatagctataatggtagtagcaggcattataggcataaga
atcataagtgtagtaataactataatagcaagagttaggcagggatattctcccctctcgttgcagctcaggata
cgcttgtggggaataatagcatattggggagcagagttaaaagagagtgccattagcttgtttaatacaacagct
attgtagtagcagagggaacagataggattatagaattagcacaaagaataggaaggggaatcttacacatacct
agaagaatcagacagggcctagaaagagcacttttataa
>#77 KY498771 2229 nt
caacattgggtaacagtgtactatggggtaccagtatggagagacgcagagacagttcttttctgcgcctcagat
gctaaagcccatagtacagaggtccacaacatctgggccacacaagcatgtgttcccactgatcccaacccacag
gaagtgctattacccaatgtaactgaaaattttaatatgtggaaaaataacatggcagaacaaatgcaagaagac
atcatcagtctatgggaacagagcttaaagccctgtgttaaattaaccccattatgtgtaactatgctttgcaac
aatagcaatggaaatatgtcagttgagggtaccacccagaggactacaacaagtagtacaggtataggaaataag
ctaatgaaaaattgctcatttaatgcaaccactgagagaaaagataaaaagaagacagtttactctctgttttat
gcagaagatgtagtaaaaatctcaaatgatagtacttataggctaataagttgtaatactacagttgtgacacag
gcttgtcctaagatctcttttgaaccaattccaatacattactgtgcaccaccaggctttgccattataaaatgt
aatgaaggaaattttagtggaaatggaacttgtaaaaatgtgagtactgtacaatgtacacatggaataaagcca
gtgatatctactcagttaatcctaaatggaagtttaagcacaggtgacattgttattagaaatagcagtcgcggt
actctgttggtgcaatggaatgagacagtgcaaataaattgtacaaggccaggaaataatacaagtggacaggtg
cagataggacctgctatgacattttataacatagagaaaatagtaggagacattagacaagcgcactgtaatgtc
tcctgggaaagttggggaaaaatgtggaatagaacaaaagacaaaataaacagggaggtcaactggaccaactgg
atctaccgggatagggagaagaaggaaggagatccagaagtaacccaattcatgttcaattgtagaggagaattt
ttctattgtaacacttctaaattgtttaaggagagtaaatcgtctaacaggacagggagtattactctgccctgt
agaataaaacaatttgtaaacttgtggacaagggtaggaaaaggaatttatgcaccaccagttaggggaaatctt
acttgtaattccagtattactggactgrttytagaatatagtrakgyagataatggtactaatgttacagtatat
ccctcaggaggaaacatgattgatctttggagacaagagttgcatagatacaaagtagttagtatagaacccata
ggagtagcaccaggtaaagctaaaagacgcacagtgagtagagaaaagagagcagtctttggactgggtgcgctg
tttcttgggtttcttggagcagcagggagcactatgggcgcagcgtcaataacgctgacggtacaggcccggaca
ttactatctgggatagtgcaacagcagaacaatctgttgagagctatagaggcgcaacaacatttgttgcaactc

tcaatttggggcattaaacagctccaagcaaaagtccttgctatagaaagataccttagggatcagcaaatccta
agtctatggggctgctcaggaaaagctatatgttataccactgtgccttggaatgagacttggagcagtaatacc
tcctatgagacaatctggaataatttaacctggcaagaatgggataagaaagtaagaaactattcaggtgtcatt
tttgaccttatagagcaggcacaagaacaacagaacacaaatgaaaaatcactcttggaattggatcagtgggca
agtctgtgggactggtttagtattacaaactggctgtggtatataaaaatagctataatgatagtagcaggcatc
ataggcataagaatcataagtgtaataataactataatagcaagagttaggcagggatattcacctcccctctcg
ttgcagctcagaatatacttgtggggaataatagcatattggggaaaagagttaaaagatagtgctatcagcttg
cttaatacaacagctattgtagtagcagaaggaacagataggcttatagaattagcacaaagaataggaaggggga
atattacacatccctagaagactcagacaaggcctagagagagtacttgtataa
>#78 DQ017383 2220 nt
caacattgggtaacagtgtactatggggtaccagtatggagagaaacagatacagttcttttctgtgcttcagat
gctaaagcccatagtacagaggctcacaacatctgggccacacaagcatgtgttcccactgatcccaacccacaa
gaagtacatttaatcaatgtgactgaaaaatttgatatgtggaaaaataacatggcagaacaaatgcaagaggac
attattagtttatgggaacagagcttaaaaccctgtgttaaattaaccccattatgtgtaactatgcattgtaac
gatagcaatgggtttaatgagacagttgggaatgggacattagacataggatacaaacaaatgaaaaattgctca
ttcaatgtaaccactgagagagaagatagaaagaagaaagcttactctctgttttatacagaagatgtagtgcaa
ctcaatgacagtgacagtgacagtaatacatatactctaataaattgtaagaccacagttgtaagacaagcttgt
cctaagaccacctttgagccaattccaatacattactgtgcaccaccaggctttgccattatgagatgtaatgaa
ggaaactttagtggaaaaggggaatgtaaaaatgtgagtactgtacaatgcacacatggaataaagccagtgata
tccactcagttaatcatgaatgggagcctagatgagacagatattgttattagaaatgatagtggtactatattg
ggtaatatattggtgcaatggaataagacagtattaataaattgtacaaggccagggaataatacaggaggacag
gtgcagataggacctgctatgacattttataacatagagaaaataataggggacattagacaagcacactgtaat
gtctctgaagaagaatggaaatcaatgtggaatagaacaaaagaaaaaataaaggacattctgggaaacaaaaca
acctttgaggctcgggggaacgatggaagtgacccagaagtaacacacttaatgttcaattgtggaggagagttt
ttcctttgtaacacttccagattatttaatgagagcaagaacgagactacaaatgggactattattctgccctgt
agaataaaacaaattgtaaatttatggacaagagtaggaaaaggaatttatgcaccaccaattcggggaaatatt
acctgtaactccaatattactggactgattctagaatatagtaatagcacaaatggtacagtagtatatccttca
ggaggaaacatggttaatctctggagacaagagttgtataaatacaaagtagtgagtatagaacccataggagta
gcaccaggtaaagctaaaagacgcacagtgagtagagaaaagagagcagcctttggactaggggcgctgtttctt
gggtttcttggtgcagcagggagcactatgggcgcagcgtcaataacgctgacggtacaggcccggacattatta
tctgggatagtgcaacagcagaataatctgttgagagcawtagaggcrcaacaacamttgttgcaactctcaatc
tggggcattaaacagctccragcaaaagtccttgctatagaragataccttagggatcagcaaatcctargtcta
tggggctgctcaggaaaaactatatgctataccactgtgccttggaatgagacttggagcartmatacctcytat
gakrcaatctggggwaatttaacctggcarmaatgggatragaaagtaagaaactattcaggtrytatttttgam
cttmtagagcaggcacaagaacaacaraacacaaatgagaaatcactcttggaattggatcaatgggcaartctg
tggaactggtttgatattacaagctggctgtggtacataaaaatagctataatggtagtagcaggcattataggc
ataagaatcataagtgtaataataactataatagcaagagttaggcagggatattctcccctctcgttgcagctc
agrawacgcytgtggggartactagcatattggggaaaagagttaaaagatagtgctatcagcttgcttrataca
ayagctattgcagtagcagagggaacagataggmtyatagaattagcacaaagaataggaaggggaatattacac
gtccctagaagaattagacagggcctagaaaggacacttgtataa
>#79 AJ271370 2205 nt
ccacattgggtaacagtgtactatggagtaccagtatggagagacgcagagacagttcttttctgcgcttcagat
gctaaagcccatagtacagaggctcacaacatctgggccacacaagcatgtgtccccactgatcccaatccacaa
gaagtgctattaaccaatgtgactgaatattttaatatgtgggaaaataaaatggcagaacaaatgcaagaggac
attatcagtttatgggaacagagcttaaagccctgtgttaaattaaccccattatgtgtaactatgctttgtaac
aatagcaatgggaatagtgcagggaatagtactaccaataggacagaggatctagaagacagacaaatgaaaaat
tgctcattcaatataaccactgagataagagatagaaagaagcaagtttactctctgttttatgtagaagatgta
gtgccaatcaaagatgggactgacaataatacatataggctaataaattgtaataccacagctgtgacacaagct
tgtcctaagactacctttgagccaattccaatacattactgtgcaccaccaggctttgccattatgaaatgtaat
gaaggaaatttagtggaaatggaagctgtacaaatgtgagtactgtacaatgcacacatggaataaagccagtg
atatccactcagttaatcctaaatggaagcttagatacagatgatattgttattagacatcatgggggtaatctg
ttggtgcaatggaatgagacagtgtcaataaattgtacaaggccaggaaataatacaggaggacaggtgcagata
ggacctgctatgacattttataatatagaaaaaatagtaggagacgttagacaagcatactgtaatgtctctgaa
gaatggggatcaatgtggaataaaacaaaaaagaagataaaaagactcctgggaaacaacacaactttcaaagct
caggataaaaatggaggagacctagaagtaacacacttaatgttcaattgtrraggagagtttttctattgcaac

acttccagattatttaatgagagcgagaacaagactaataagactattattctgccctgtagaataaaacaaatt
gtaratttgtggacaagagtagkaaaaggaatttatgcaccaccaattcggggaaatcttagctgtarctccagt
atcactggactgattctagaacatagtggtgaaaacggtaacaagacagtatatccctcaggaggaaacatggtt
aatctctggagacaagaactgtataagtacaaagtagttagtatagaacccataggagtagcaccaggtaaagcc
aaaagacgcacagtgagtagagaaaagagagcagcctttggactaggtgcgctgtttcttgggtttcttggagca
gcagggagcactatgggcgcagcgtcaataacgctgacggtacaagcccggacattattatctgggatagtgcaa
cagcagaataatctgttgagagcaatagaggcgcaacaacatttgttgcaactctcaatctggggcattaaacag
ctccgagcaaaagtccttgcgatagaaagataccttagagatcagcaaatcctaagtctatggggctgctcagga
aaaactatatgctataccactgtgccttggaatgatacytggagcagtaatacctcctatgatacaatctggrgt
aatctcacctggcaacaatgggatcggaaagtaaggaactattcaggtgtcatttttgatcttatagagcaagca
caagaacaacaaaacacaaatgagaaagcactcttggaattggatcaatgggcaagtctgtggaactggtttgat
attacaaaatggctgtggtacataaaaatagctataatggtagtagcaggcattataggcataagaattataagt
gcaataataactataatagcaagagttaggcagggatattctcccctctcgttgcagctcagaatacgcttgtgg
ggaataatagcatattggggaaaagagttaaaagatagtgctatcagcttgcttaatacaacagctattgtagta
gcagagggaacagataggtttatagaattagcacaaagaataggaaggggaatattacacatacctagaagaatc
agacagggcctagaaagagcacttttataa
>#80 DQ314732 2235 nt
gcctcaaacaacttgtgggttacagttcattatggggttcctgtgtggagagatgcagataccaccctattttgt
gcatcggatgccaaggcacatgaaacagaagtgcacaatgtctgggccacacatgcctgtgtacccacagacccc
aacccacaagaaatatacatggaaaatgtaacagaaaattttaacatgtggaaaaataacatggtagagcagatg
caggaggatgtaataagtttatgggatcaaagtataaaaccatgtgtaaagttaactcctctctgcgttacttta
aattgtaccaataaggttcgggcgatcaatgtcacatcaagctctagcataggaaatataacagatgaattaaga
aactgttcttttaatataaccacagaactaagagataagaaacagaaggtccatgcactttttttataagcttgat
atagtacctattgaaaataacaatagtagtaacaatagtagtgagtataggttaataaattgtaatacttcagtc
attaagcaggcttgtccaaagatatcctttgatccaattcctatacattattgtactccagctggttatgcgatt
ttaaagtgtaatgataagaatttcaatgggacagggccatgtaaaaatgtcagctcagtacaatgcacacatgga
attaagccagtggtatcaactcaattgctgttaaatggcagtctagcagaagaagagatagtaatcagatctgaa
aatctcacaaacaatgtcaaaaccataatagtacatcttaataaatctgtagaaatcaattgtaccagaccctcc
aacaatacaagaaaaagtataactataggaccaggacaaatgttttacagaacaggagaaataataggagatata
agaaaagcatattgtgagattaatggaacagaatggaatgaaactttaagacaggtagctgtaaaattaaaagag
cactttaataagacaataatatttaaaccaccctcaggggggagatctagaaattacaatgcatcattttaattgt
aaaggggaattttttctattgcaatacaacacaactgtttaatagtacttgggtaggaaatgaaaccaaaaagggg
gataatggcactataagaaatgaaaccaaaaaggggggataatggcactatcatacttccatgcaggataaagcaa
attataaacatgtggcaggaaacaggacaagcaatgtatgctcctcccatcagtggaacaattaattgtgtatca
aatattacaggactactattgacaagagatggtggaggtaataatgactcgagtcagaacgagaccttcaggcct
ggagggggaaatataaaggacaattggagaagtgaattatataaatataaagtagtagaaattgaaccactagga
atagcacccaccagggcaaagagaagagtggtggagagagaaaaaaagagcagtgggaataggagctatgatcttt
gggttcttaggagcggcaggaagcactatgggcgcggcgtcaataacgctgacggtacaggccagacaattattg
tctggtatagtgcaacagcaaagcaatttgctgagggctatagaggcgcagcagcatctgttgcaactcacagtc
tggggcattaaacagctccaggcaagagtcctggctgtggaaagatacctaaaggatcaaaagctcctaggactt
tggggctgctctggaaaaatcatctgcaccactgctgtgccctggaactccacttggagtaataaatcttatgaa
gagatttgggagaacatgacatggatagaatgggagagagaaattagcaattacacaaaacaaatatatgagata
cttacagaatcgcaaaaccagcaggataagaatgaaaaggatttgttagaactggataaatgggcaagtctgtgg
acttggtttgacataacccattggctgtggtatataagaatatttataatgatagtaggaggtttaatagggtta
agaataatttttgctgtgcttgctatagtacatagagttaggcagggatactcacctttgtctttccaggctatt
tctttgtttgatgcttcagcaatagcagtagcggggtggacagatagggttatagaagtagcacaaggagtttgg
agagctattctccacatacctaggagactcagacagggcttggaaagggctttgctataa
>#81 AF377956 2190 nt
gctgcagataacttgtgggtcacagtctattatggagtacctgtgtggaaagaagcaaccactactctattttgt
gcatcagatgctaaagcatatgaaagagagatacataatgtctgggctacatatgcctgtgtacctacagacccc
aacccacaagaattagttctgggaaatgtaacagaaaattttaacatgtggaaaaataacatggtagaccagatg
catgaagatataatcagtttatgggatcaaagcctaaagccatgtgtacagataaccccactctgtgtaacctta
aattgtactgatgttcctgttaacatcactaatgggaacagcaccctggataacatcaccctggaagaacaaggg
gaaataaaaaactgttctttcaatatcaccacagagataaacgatattaagaaaaaagaatctgcaatttttat
aggcttgatgtagtaccaatcaataatagtactagtgaatataggctactaagttgtaatacctcaaccgttaca

caggcttgtccaaaggtgtcctttgatccaattccaatacattattgtgctcctgctggttttgcgattctaaag
tgtaatgataaagagttcaatggacagggttatgtaggaatgtcagcacagtacaatgtacacatggaattaaa
ccagtagtgtcaactcaactactgttaaatggcagcctagcagaaggagatatagtaattagatctgaaaatatc
tcagataatgcaaaaaccataatagtacagtttaatagatctgtagcaattaactgtacaagacccaccaacatt
acaagaagaagtatgcgtataggaccaggacgagtattttatgcaacaggtaccgtactaggagatataagaaag
gcatattgtaccattaatggaacactgtggaataaaactttagaaggagtagctaaagaggtccaaagccacctt
aataaatcaataacatttgcgccatcatcaggaggggacctagaagttacaacacatagttttaattgtagagga
gagtttttctactgcaacacagtagctctgtttaatgcaactaacatgactaatgcaatgaacaggtccaatggc
attatcactcttccatgtagaataagacaaattgtaaacatgtggcaaagagtaggacgagcaatgtatgccgct
cccattgctggacaaattcagtgtaactcaagcatcacaggtctaatattgacaagagatggtgggaaaaataat
accaataatgacaccctcagacctggaggggagatatgagagacaattggagaagtgaactgtataaatataag
gtagtaaaaattgaaccactaggagtagcacccaccaaggcaaaaagacaagtggtgaagagagaaagagaaaaa
agagcagtgggaataggagctgtgctccttgggttcttgggagcagcaggaagcactatgggcgcggcgtcaatg
acgctgacggtacaggccagacaattattgtctggtatagtgcaacagcaaaacaatttgctgaaggctatagaa
gcgcaacagcatctgttgcagctcacagtctggggcattaaacagctccaggcgagaatcctggctgtggaaaga
tacctaaaggaccaacagctcctagggatttggggatgctctggaaaactcatctgcaccactaatgtgccctgg
aattctagttggagtaataaatctcagaatgaaatttgggagaacatgacctggatgcagtgggaaaaagagatc
agtaattacacaggcacaatatacaaattaatagaaaatgcacaaaaccagcaggaaaagaatgaacaggactta
ttggcattggacaaatgggacaatctgtggagttggtttactataacaaattggttgtggtacataaaattattc
ataatgatagtaggaggcttgataggattaagaatagtttttgctgtgcttgctgtaataaatagggttaggcag
ggatactcacctttgtcgttacaggctattgatctgcttaataccacagcaatagtggtagctgaagggacagat
agaatcatagaagttctgcaaagagctggtagagctattctccacatacctagaagaataagacagggtgcagaa
agggctttgctataa
>#82 AB254156 2259 nt
atggagggcttgtgggtcactgtacactatggggtacctgtgtggaaagaagcaaagactactctattttgtgca
tcagatgctaaagcatatgagacagaagtgcataatgtctgggctacacatgcctgtgtacccacagaccccagc
ccacaagaagtgtatttaaaaaatgtaacagaaaattttaatatgtgggaaaataacatggtggatcagatgcat
gaggatataatcagtttgtgggatcaaagcctaaaaccatgtgtaaagttaaccccactctgtgtcactttagac
tgtagtcataatattaccattaggaatagtacctccagtgagaataatagtacctccagtaccaacagtacaatc
ttgagtgaaataatgagaaattgtactttcaatgtaaccacagaactaagggataagcagacaaaagaatatgca
ctttttataaacttgatatagtaaaacttgagaagaactctagtgaatatagattaataaattgtaattcatca
accgtaacacaggcctgtccaaaagtctcttttgacccaattcctatacattattgtgctccagctggttatgcg
attctaaagtgtaataataattatacattcaatggaacaggaccatgtaataatgtcagcacagtacaatgtaca
catggaattaaaccagtggtgtcaactcaactactgttaaatggtagcctagcagagggagggatagtaattaga
tctaaaaatctgacagacaatgccaaaacaataatagtacatcttaatgaatctgtaagaattgtgtgtacaaga
cctggcaataatacaagaaaaagtgtaaggataggaccaggacaagcattctttgcaacaggagaagtaatagga
gatataagaaaggcacattgtaacattactgagaggacatggaataacactttacaaaagataagtagaaaatta
gaaaaacactttccgaacaaaactataaaatttgcaccatcctcaggaggggacccagaaattacaacacatagc
tttaattgtagaggagaatttttctattgcgacacatcaaacctgtttaacagcacatttaatgttaacagcaca
tttaatggtacagataatgcaaatagcacacagaacatcacactccaatgcagaataaaacaaattataaacatg
tggcaggaggtaggacgagcaatgtatgcccctcccattgcaggaaacataacatgtatttcaaatatcacagga
atactattaacacgtgatggaaatgataattacacagagacattcagacctataggaggaaatatgaaggacaat
tggagaagtgaattatacaaatataaagtggtagaaattaagccattaggagtagcacccaccaaggcaaaaagg
agagtggtggagagacaaaaaagagcagtgggactaggagctgtgttccttgggttcttaggagcagcaggaagc
actatgggcgcagcgtcaataacgctgacggtacaggccagacaattgttgtctggtatagtgcaacagcaaagc
aatttgctgagagctatagaggcgcaacagcacatgttgcaactcacagtctggggcataaaacagctccaggca
agagtcctggctatagaaagatacctaaaggatcaacagctcctagggatttggggctgctctggaaagctcatc
tgcaccactgctgtgccttggaactctagttggagtaataaaacatataatgagatttgggataatatgacctgg
atgcagtgggatagagaaatcaataattacacagatacaatatactggttgcttgagaaatcacaaaaccagcag
gaacaaaatgaaaaagatttactagaattggacagttgggatagcctgtggagttggttaagcataacaaattgg
ctgtggtatataagaatattcataatgatagtaggaggtttgataggtttaagaataatttttgctgtgctttct
atagtaaagagagttaggcagggatactcacctttgtcgtttcagtatttgggaagccttgtgcagtattggggt
ccggaactaaaaaagagtgctattagcctgcttgataccatagcaatatcagtagctgagggaacagataggatt
atagaattgctaagaagaatttgtagagctatctgtagcatacctagaagaataagacagggctttgaagcagca
ttgcaataa

>#83 MF373132 2289 nt
gggaagggggacaaggaggacttgtgggtcacagtatattatggggtacctgtgtggagagatgcacaaacctct
ctgttttgtgcatcagatgctaaagcatatgatacagaggtgcataatgtctgggctacacatgcctgtgtaccc
acagaccccaacccacaagaaatggttttaaaaaatgtaacagaaaattttgacatgtggaaaaataacatggtg
gatcagatgcatcaggatataatcagtttatgggatcaaagcctaaagccatgtgtaaagatgaccccactctgt
gttactttaaactgtacaaatgctacaactgctatgaatgctacaattgataataacatgcagggagaaataaaa
aattgtactttcaatgtaaccacagaaataagagataaggtaaagaaggtacatgcactttttataaacttgat
atagcaccacttggtaacagtgctagtagtgataacagttctagtggtggtaaaggtactaggaataatgagtat
aatgattatatattaataaattgtaatacctcagccatagctcaagcctgtccaaaggtctcttttgacccaatt
cctatacattattgtgctccagcgggttatgcgattctaaagtgtaatgataagacattcaatggaacaggacca
tgcaataatgtcagcacagtacaatgtacacatggaattaagccagtggtatcaactcaattactgttaaatggt
agcctagcagaaggagacataataattagatctgagaatatgacaagcaatactaaaacaataatagtacatctt
aatgaatctgtatcaattaattgtacaagacccaacaataatacaagagaaagtttaaggataggaccaggacaa
gcattatatgcaacaggagatataataggagatataagacaagcacattgtaatattagtaaagaaaggtggaat
aaaactttagaaagagtaaagaaaagattagcagagtacttccctaatacaacaatactctttgcaccacccca
ggaggggacctagaagttacaacacatagctttaattgtagaggagaatttttctattgcaatacatcaaacctg
tttaatagtagtaaaactaatataacagcaaatgaaaatggaaccatcacactcccatgcagaataaaaccaatt
gaaaccttggggcaggggggtaggaaaagcaattattgcccccctccattaaaggaaaattacctggtacttccaaa
ttcccaggcttaccactggccccgagtaggggtgaaaaaataggaacctatggaaatttggcaaaaagagaattt
tccccgggggggggagatatgagtgacaattggagaagtgaattatataaatataaagtggtagaaattaatcca
ttaggagtagcacccactaaggcaaaaaggagagtggtggagagagaaaaaaagagcagtgggaataggagctgtg
ttccttgggttcttgagtgcagcaggaagcactatgggcgcagcgtcaataacgctgacggtacaagccagacaa
ttgttgtctggtatagtgcaacagcaaaacaatttgctgagagctatagaggcgcaacaacatatgttgaaactc
acagtctggggcattaagcagcttcaggcaagagtcctggctatagaaagatacctaaaggatcaacagctccta
gggatttggggctgctctggaaaactcatctgcaccactgctgtgccttggaatgaaagttggagtaataagtct
caaaatgagatttgggataacatgacctggatgcagtgggatagggaaatcaataattacacaaatgaaatatac
agattgcttggggaatcgcaaatccagcaggagaaaaatgaacaggatttactagcgctggacaagtggcaaaat
ctgtggagttggtttgacataacaaactggctgtggtatataaaaatgttcataatgatagtaggaggcttgata
ggtttaagaatagtttttgctgtgctttctatagtaaatagaattaggcagggatactcacccttatcgttgcag
tatctgggaagtcttgtgcagtattggggttctgagctaaaaaggagtgctattagtctgcttgataccacagca
atagcagtaggtgaaggaacagataggattgtagaatttatactacgagtttgtagagctatctacaacatacct
cgaagaataagacagggctttgaagcagctttgcaataa
>#84 KP109483 2292 nt
gtgggaggaaacttgtgggtcacagtttattatggggtacctgtgtggagagaagcaaaaactactctattctgt
gcatcagatgctaaagcatatgagaaagaagtgcataatgtctgggctacacatgcctgtgtacccacagacccc
aacccacaagaaatagatttgaatgtaacagaaaattttaacatgtggaaaaatgacatggtggagcagatgcat
gaggatgtaatcagtttatgggatcaaagcctaaagccatgtgtaaagttgaccccaatttgtgtcactttagaa
tgtacagatgctaatattacctgcaatagtactactagcagtaataattgtaccagctatgagatcaacaaggag
gacatgggggaaataaaaaattgctctttcaatacaaccacagaattaatagataagcagaagaaagtgcatgca
ctcttttatagacttgatatagtatcactagaaaaggacaactctagtaagaagaacgactctaatgagtattat
agattaataaattgtaatacctcagccataacacaagcctgtccaaaggtcacttttgacccaattcctatacac
tattgtgctccggctggttatgcgattctaaagtgtaaaaataagacattcaatggacggaccatgcaataat
gttagcacagtacaatgtacacatggaattaagccagtggtatcaactcaactactgttaaatggtagcctagca
gaagaagagataataattagatctgaaaatctgacaaacaatgccaaaataataatagtacatcttaatcaagct
gtagaaattgtatgtacaagacctggcaataatacaagaaaaagtataaggatagggccaggacaaacattctat
gcaacaggagacataataggagacataagacaagcacattgtaacattagtgaagctaagtggaataaaaccttg
cgagaggtaagtaaaaaattagcagaacacttccctaataaaacaataatatttaactcatcctcaggaggggac
ctagaaattacaacacatagctttaattgtggaggagaatttttctattgtaatacatcaagcctgtttaatagt
acatttaatagtacatacatgactaatgatacagacatgaattcaaactcaaccatctcaatcccatgccgcata
aaacaaattataaacatgtggcaggaggtaggacgagcaatgtatgcccctcccattgcaggaaacataacatgt
aaatcaaatatcacaggactactattggtacgagatggaggaaactcaaatgatacaaatgagccagagatattc
cgacctcaaggaggagatatgagggacaattggagaagtgaattatataaatataaagtggtagaaattaagcca
ttgggagtagcacccactgcagcaaaaaggagagtggtgggaagagaaaaaagagcagtgggactaggagctatg
atccttgggttcttgggagcagccggaagcactatgggcgcggcgtcaataacgctgacggtacaggccagacaa
ttgttgtctggtatagtgcaacagcaaagcaatttgctgagggctatagaggcgcaacagcatatgttgcaactc

acggtctggggcattaaacagctccagacaagagtcctggctatagaaagatacctaaaggatcaacagctccta
gggatttggggctgctctggaaaactcatctgcactactgctgtaccttggaactccagttggagtaacaaatct
caagaccagatttggaatgatacgacctggatgcagtgggataaagaaattagtaattacacagacataatatac
acgttgcttgaaaaatcgcaaaaccagcaggaaataaatgaaaaggatttattagcattggacagttggaacagt
ctatggaattggtttgacataacaaagtggctgtggtatataaaaatattcataatgatagtaggaggcttgata
ggtttaagaataatttttgctgtgctttctatagtgaatagagttaggcagggatactcacctttgtcgtttcag
acctatctaggaagtcttgtgcagtattggggtctggaactgaaaaagagtgctattagtctgtttgataccata
gcaatagcagtagctgaaggaacagataggcttatagaattcatacaaagaactggtagagctatctgcaacata
cctagaagaataagacagggttttgaagcagctttgcaataa
>#85 KY658708 2241 nt
agtgtggtgggaaacttgtgggtcacagtttattatggggtacctgtgtggaaagatgcaaaaactactctattc
tgtgcatcagatgctaaagcatatgaaaaagaagtgcataatgtctgggctacacatgcctgtgtacccacagac
cccaacccacaagaaatagttttggaaaatgtaacagaaaattttaacatgtggaaaaatgacatggtggatcag
atgcatgaggatataatcagtttatgggaccaaagcctaaagccatgtgtaaagttgaccccactctgtgtcact
ttaaaatgtgtaaatgttagtaataaatttaacctacaaaatagcatgaatgaagatccgaaggaagaaataaga
aattgctctttcaatgcaaccacagaattaagagataagaaacagaaagtgcatgcactcttttataaacttgat
atagtaccacttcatggcaacaactctagtgagtatagattaataaattgtaatacctcaaccataacacaagcc
tgtccaaaggtctcttttgatccaattcctatacattattgtactccagctggttatgcgattttaaagtgtaat
aatcagacattcaatgggacaggaccatgcaataatgtcagctcagtacaatgtacacatggaattaggccagta
gtatcaactcagttactgttaaatggtagcctagcaaaaggagagataataattagatctgaaaatctgacaaac
aatgccaaaataataatagtacatcttaataaacctgtaaaaattgtgtgtgtaaggcccaacaataatacaaga
aaaagtgtaaggataggaccaggacaaacattctatgcaacaggtgaaataataggagacataagacaagcatat
tgtatcattaataaaactgaatggaataacactttacaaggggtaagtaaaaaattaggagaacacttctctaat
aaaacaataaaatttgaaccgtcatcaggaggggacctagaaattacaacacatagctttaattgtagaggagaa
tttttctattgcaacacatcacaactgtttaatagtacatacagtcccagttttaatggtacagaaggtaaatta
aatggaccatcacaatcccatgtagaataaaacaaattataaacatgtggcagaaagtaggacaagcaatgtat
gcccctcccattgcaggaaacctaacatgtgaatcaaatatcacaggattactattgacacgtgatggaggaaaa
acaggttcaaatcacccagagatattcagacctggaggaggggatatgagggataactggagaagtgaattatat
aaatataaagtggtagaaattaagccattgggagtagcacccactgaggcaaaaaggagagtggtggagagagaa
aaaagagcagtgggaataggagctgtgttccttgggttcttgggagcagctggaagcactatgggcgcagcgtca
ataacgctgacggtacaggccagacaattgttgtctggtatagtgcagcagcaaagcaatttgctgagggctata
gaggcgcaacagcatctgttgcaactcacagtctggggcattaagcagctccagacaagagtcttggctatggaa
agatacctaaaggatcaacagctcctagggatttggggctgctctggaaaactcatctgcaccactgccgtgcct
tggaactccagttggagtaatagatctcatgatgagatttgggataacatgacctggatgcagtgggatagagaa
attaataattacacagacacaatatacaggttgcttgaagaatcacaaaaccagcaggagaaaaatgaaaaggat
ttattagcattggacagttggcaaaatctgtggaattggtttagcataacaaattggctgtggtatataaaaata
ttcataatgatagtaggaggcttgataggtttaagaataatttttgctgtgctttctatagtgaatagagttagg
cagggatactcacctctgtcgtttcagtatctgggaagccttgtgcagtattggggtctagagctaaaaaagagt
gctattagtctgtttgataccatagcaatagcagtagctgaaggaacagataggattatagaattaatacaaaga
atttgtagagctattcgcaacatacctagaagaataagacagggctttgaagcagctttgcaataa
>#86 KC156114 2226 nt
gggatggggcaattgtgggtcacagtctattatggggtacctgtatggaaggaagcaaaaactactctattttgt
gcatcagatgctaaagcatattcccaagaagcacataatatctgggctacacatgcctgcgtacctacagacccc
aacccacaagaaatggttttagaaaatgtaacagaaaattttaacatgtggaaaaatgacatggtagatcagatg
catgaggatataatcagtttatgggatcaaagcctaaagccatgtgtaaagttgaccccactctgtgtcacttta
aactgtactactgttaatattacccaggaaaataatccccttaatgatagtgatagtaattacatgaaaaactgc
tctttcaatatgaccacagaactaaaagataaaaagaaggaagagagggcattgtttcatagacttgatctagta
ccacttaatgagaatagtagcaagtatgagaatagtaacaagtatatattaacaaattgtaggacctcaaccata
gcacaggcctgtccaaagatctcgtttgatccgattcctatacattattgtgctccagctggttatgcgattcta
aaatgtaataataaggcattcaatggatcaggaccatgcactaatgtcagcacagtacaatgtacacatggaatt
aagccagtggtatcaactcagctactgttaaatggtagcctagcagaagagataataatcagatttgaaaatctg
acaaacaatgtcaaaacaataatagtacaccttaatgaatctgtagaaatttcgtgtacaagacccaacaataat
acaagaaaaagtgtgaggataggaccaggacaaacattctatgcaacaggagaaataataggagatataagacaa
gcatattgtaccattaatgaaggaaaatggaataaaactttagaaaggataaggaacaaattagcagagtacttc
cctaataaaacaatacaatttcaaagcgcctcaggaggggacctagaaattacaacacatagctttaattgtgga

ggagaattttctattgcaacacatcaagattgtttaatcatacatttaatgagacatcagacgaagacatcaca
ctccaatgtaggataaaacaaattataaacatgtggcaggggtaggacgagcaatatatgcccctcccattgca
ggaaacataacatgtaactcaaatatcacaggactactattgacacgtgatggagggaagagtaatagtagtaat
cagacagagatattcagacctgcaggaggaaatatgagggacaattggagaagtgaattatataaatataaagtg
gtagaaattaaaccattaggaatagcacccactaaggcgaaaaggagagtggtggagagagaaaaaagagcggca
ataggagctatgttccttgggttcttgggagcagcaggaagcactatgggcgcggcgtcaataacgctgacggta
caggccagacaattattgtctggtatagtgcaacagcaaagcaatttgctgagagctatagaggcgcaacagcat
atgttgcaactcacggtctggggcattaaacagctccaggcaagagtcctggctatagaaagatacctaaaggat
caacagctcctaggaatttggggctgctctggaaaactcatctgcaccactaatgtgccttggaactctagttgg
agtaataaacctgaagaagaaatttggggaacatgacctggatgcaatgggagagagaaattgataattataca
gacacaatatacagtttgcttgaaaaatcgcaaaaccagcaggaacaaaatgaacaagatttactagcattggac
agttggaaaaacctgtggaattggtttagcataacaaaatggttgtggtatataaaaatattcataatgatagta
ggaggcttaataggtttaagaataatttttgctgtgctttctatagtgaatagagttaggcagggatactcacct
ttatcgttccagtatctaggaagtcttgcgcagtattggggtctggaactaaaacaaagtgctattaacctgctt
gataccatagcaatagcagtagctgaaggaacagataggatcatagaagtaatacaaagaatttgtagagctatc
tgcaatatacctacaagaataagacagggctttgaagcagctttgcaataa
>#87 AF005496 2187 nt
gctgcacaaaacttgtgggttacagtatattatggggtacctgtgtggaaagaggcaaaaaccactctattctgt
gcatcagatgctaaggcatatgagacagaaaagcataatgtctgggctacacatgcatgtgtacccacagacccc
aacccacaagagatggtcatggagaatgtaacagagagctttaatatgtgggaaaataacatggtggagcagatg
catacagacataatcagtttatgggatcaaagcttgaaaccatgtgtaaaattaaccccactctgtgttactcta
aactgtactaatgtcagaaacaatacctctaacagcactagcagtatggaggcaggaggggaactaacaaattgc
tctttcaatgtaactacagtactaagagataagcagcagaaagtacatgcactcttttatagacttgatgtagta
ccaattgataacaatagtactcagtataggctaataaattgtaatacctcagtcattacacaggcttgcccaaag
gtgtcctttgaacctattcccatacattattgtgctccagctggctttgcgattctaaagtgtaacaataaaaca
ttcaatggaacaggattatgtacaaatgtcagtacagtacaatgtacacatggaattagaccagtggtatcaact
caactgctattaaatggaagcctagcagaagaacagatcataattagaactaaaaatatctcagacaataccaaa
aacataatagtacagcttaagacaccagtaaacattacatgtaccaggcctaacaataatacgagaacaagtata
catttagggccaggacgagcattctatgcaacaggtgacatcataggagatataagacaagcacattgtaatatt
agtagaacagactggaataagactttacaccaggtagttacacaattaggaatacacttgaacaatagaacaata
agctttaagccaaactcaggaggggacatggaagttagaacacatagttttaattgtagaggagaattttctat
tgcaatacatcagggctgtttaatagtagttgggaaatgcatactaattacacatcaaatgacacaaagggaaac
gaaaacattacactgccatgcagaataaaacaaattgtaaacatgtggcagagagtaggacgagcaatgtatgcc
cctcccatccaaggaaacattatgtgtgtatcaaatattacaggactaatattgacaattgacgagggtaacgcg
tctgcagaaaattataccttcagacctggaggaggagatatgagggacaattggagaagtgaattgtataaatat
aaagtagtaaaaattgaaccactgggaatagcacccaccaagacaaggagaagagtggtggagagagaaaaaaga
gcagtgggaatgggagcttctttccttgggttcttgggagcagcaggaagcactatgggcgcggcgtcaataacg
ctgacggtacaggccaggcaattattgtctggtatagtgcagcagcaaagcaatttgctgagagctatacaggcg
cgacagcatatgttgcagctcacggtctggggcattaaacagctccaggcaagagtcctcgctgtggaaagatac
ctaagggatcaacagctcctggggatttggggctgctctggaaaactcatctgcaccactaatgtgccttggaac
tctagttggagtaataaatcacagagtgaaatctgggacaacatgacttggatggaatgggataaacaaattagc
aattacacagaggaaatatacaggttgcttgaagtctcgcaaacccagcaggaaaagaatgaacaggacttatta
gcattggacaaatgggcaagtctgtggacttggtttgacatatcacattggctgtggtatataaaaatattcata
atgatagtaggaggtttaataggtttaagaataatttttgctgtgctttctatagtaaatagagttaggcaggga
tactcacctttgtcttttcaggctattgatttgcttaacaccacagcaatagcagtagctgagggaacagatggg
attatagtaatagtgcaaagagcttggagagctattctccacatacctagaagaataagacagggctttgaaaga
agcttgctataa
>#88 FJ389367 2205 nt
gcctcaaataccttgtgggtcacagtctattatggagtacctgtgtgggaagatgcagataccaccctattttgt
gcatctgatgctaaatcatatagtactgaaggccataatgtctgggctacacatgcctgtgtacccacagacccc
aacccacaagagatatctctgataaatgtaacagaaaattttaacatgtggaaaaataacatggtagaacagatg
catgaggatataatcagtttatgggacgaaagcctaaagccatgtgtacagctaacccctctctgtgttacttta
aactgtgtgccagcaaatggcactaacataactggctctggcactgtgaatataaccgaacagatgaaaaactgc
tcgttcaatataaccacagaaataagggataggaagaagcargaatacgcgctttttttataaacttgatatagta
ccaatagaggataatagtaatagtaataataatagttataggatgataaattgtaatgtctcaaccattaaacaa

gcttgtccaaagatgtcctttgacccaattcccatacattattgtgctccagctggttttgcgattttaaagtgt
agggacgaggagttcaatggaacaggaacatgtaaaaatgtcagtacagtacaatgtacacatggaattaagcca
gtggtatcaactcaattactgctgaatggcagtttggcagaaggagacataatgattagatctaaaaacatcaca
gacaatgccaaaatcataatagtgcagcttaataaatctatagaaattaattgtaccagacctggcaataataca
agaaaaagtgtaagaatcggaccaggacaagtgttctatacaacaggtgaaataataggagatataagacaagca
tactgtaatattgcaaaaaaagactgggataatatgctaagggaagtggctataaaactaaggaaaagccttaac
agcaacaagaccataacctttaactcatctgcaggaggggacctagaaattgcaacacatagttttaattgtaga
ggagaattttctattgtaatacatcaggcctgtttaatatatcatataatagtacagagaatagtacttataat
gaagggaatgagacttatacactcccatgtaaaataaaacaaattgtgagaatgtggcaaagagtgggacaagca
atgtatgcccctcccatcaaaggaaacattacatgtagatcaaacattacaggactactattaacaagagatggt
ggggagaatgataataataacagtagtgaggtcttcagacctgcaggaggagacatgagggacaattggagaagt
gaattatataagtataaaatagtaaaaataaaaccattaggagtagcacccaccaaggcaaggagaagagtggtg
ggagagagaaaaaagagcagtagttggactaggagctgttttccttgggttcttaggagcagcaggaagcactatg
ggcgcggcgtcaataacgctgacggtacaggtcagacaattattgtctggcatagtgcaacagcaaagcaatttg
ctgagagctatagaggcacagcatcatctgttgcaactcacagtctggggcattaaacagctccaggcaagactc
ctggctgtggaaagatacctaaaggatcaacagctcctagggatctggggctgctctggaaaactcatctgcacc
actaatgtgccctggaatgctagttggagtaataaatcttatgaggccatttgggagaacatgacctggatagaa
tgggaaagggaaattaacaattatactcaacaaatatacagcctaattgaagaatcgcagaaccagcaggaaaag
aatgaacaagacttattagcattagaccaatgggcaagtttgtggaattggtttgacatatcaagatggctatgg
tatataaaaatatttataatgataataggaggtttaataggtttaagaatagtttttgctgtgcttgctataata
aatagagttaggcagggatactcacccttgttgttccaggctattaatttgcttgatacggtagcaatagcagta
gctaattggacagatagggttatagaagtagtacaaagagcttgtagggcttttctcaacatacctagaaggata
agacaaggcgtagaaagagcattgccataa
>#89 FJ389365 2190 nt
gcttcctcagacttgtgggtcacagtctattatggggtaccagtgtgggaagatgcagatactcctctattttgt
gcatctgatgctcaagcacatagtactgaaagccataatgtctgggccacacatgcctgtgtacccacagatccc
aacccacaagaaatgcttctgaaaaacgtaacagaaccttttaacatgtggaaaaataacatggtagaacagatg
aatgatgatataatcagtttatgggatgaaagcctaaagccatgtgtacagctaacccctctctgtgttacttta
aactgtgctgatgtttgtcttaagaacagcactgctactaatgattgtcctaagaacagcactgggaataacact
gtgagtaatagtattacaaaagaaatgacaaactgctctttcaatataaccacagaaataaaagataagcagaag
aaagaatacgcgctttttttataaacttgatatggtgcaactggatggtagaaatgactcttatgggttaataaac
tgtaatgtctcagccattaaacaagcttgtccaaaggtgtcttttgacccaattcccatacattattgtgctcca
gctgggtttgcgattttaaagtgcaaggataagaatttcaatggaacaggaacatgtaaaaatgttagttcagta
caatgcacacatggaattaagccagtggtatcaactcaattactactgaatggtagtctagcagaagaagaaata
gtaattagaactgaaaacatctcagacaatgccaaaatcataatagtgcagcttaataaatccatagaaattaat
tgtaccagacccagtaacaatacaagaagaagtataagtcttggacctggacggcgttctatgcaacaggtgat
gtagtaggagatataagacaagcacactgtaatgtaagtagacaacaatggaatgacacggtacagaaagtaact
gcatcactacaaaagatctttaaaaaaggcaatataacctttaagccacccacaggaggggacctagaaatgaca
acacatagttttaattgtagaggggaattttctattgtaatacatcagacttgtttaatatcagtacaacaaat
agtactaatgatactatcacactcccatgtaagataaaacaaattgtgagaatgtggcagagagtgggacaagca
atgtatgcccctcccattgcaggagaaattacatgcaggtcaaatattacaggactactattaacaagagatggt
gggggtggaggtaatcaaacaaatgaaaccttcagacttgcaggaggaaatatgagggacaattggagaagtgaa
ttatataagtataaagtagtaaagattaaaccactaggaatagcacccaccaagtcaaggagaagagtggtggag
agagaaaaaagagcagttggaataggagctgccttccttgggttcttaggagcagcaggaagcactatgggcgcg
gcgtcaataacgctgacggtacaggtcagacaattattgtctggcatagtgcaacagcaaagcaatttgctgaga
gctatagaggcgcagcaacatctgctgcaactcacagtctggggcattaagcagctccaggcaagagtcctggct
ctggaaagatacctaaaggatcaacagctcctagggatttggggctgctctggaaaactcatctgcaccactaat
gtgcgctggaacactagttggagtaataratcttttratgagatttgggataacatgacctggatacaatgggaa
agggaagtcagcaattacacacaagaaatatacagactaattgaagaatcgcaaaaccagcaggaaaagaatgaa
caagacttattagcactggaccagtgggcaagcttgtggaattggtttgacatatcaaggtggctgtggtatata
agaatattcataatgatagtaggagggttaataggtttaagaatagtttttgctgtgctttctatagtaaataga
gttaggcagggatactcacctttatctttccagttgcttgatacaattgcaatagcagtagctaactggacagat
agggttatagaagtagcacaagcagctggtagagcttttcttcacatacctagaaggataagacaaggctttgaa
agagctttgctataa
>#90 MH705151 2268 nt

gctacaggaaacttgtgggtcacggtatattatggggtacctgtgtggaaagacgcagagaccaccctattttgt
gcgtcagatgctaaagcatatgatacagaagtgcataatgtctgggctacacatgcctgtgtacccacagacccc
agcccacaagaaatagacctggaaaatgtaacagaaaagtttaacatgtggaaaaataacatggtagagcagatg
catatggatataattagtctatgggatcaaagcctaaagccatgtgtaaagttaacccctctctgtgttacttta
gattgtagccatagcatcaccaccatcaatagcaccaccatcaatagcaacagcacaagtgatatatcaggagaa
ataaaaaactgctctttcaatatgaccacagaactaagagataagacaaagaaagtatattcactttttataga
cttgatgtggtacaaattgatgaaaataatggtagtaataacagtgagtatagactaataaattgtaatacctca
gccattacacaggcttgtccaaaggtaacctttgagccaattcccatacattattgtgccccagctggttttgcg
attctaaagtgtaaggaggagaatttcaatggaacagggatatgcaggaatgtcagcacagtacaatgcacacat
ggaattaagccagtagtatcaactcaactgctgttaaatggcagcctagcagaaggagaggtagtgattagatct
gaaaacttcacaaacaatgccaaaatcataatagtacagtttaatgagtctgtacaaattaattgtaccagacct
aacaacaatacaagaagaagtttccgtataggaccaggacagacattctatgcaacaggtgaaataataggaaat
ataagacaagcacattgtaatgtcagtaaaacagactgggagagcactttacaaaggtagctgcacaattaggc
aaagaatttaaaccaacacaacaatctttgctaactcctcaggaggggatatagaaattacaacacatagtttt
aattgtagaggggaatttttctattgcaatacatcagacctgtttaatagcacttggcggaaactggcaaatggc
acttggagcaacataaataatgcaacgtcaaatgacactataactctccaatgcaaaataaagcaaattgtaaac
atgtggcagagagtaggacaagcaatgtatgcccctcccatccaaggagtaatacagtgtgtatcaaacattaca
ggactactcttaacaagagatggtgggagtagtaacagggcaaatgagaccttcagacctggaggaggagatatg
agggacaattggagaagtgaattatataagtataaagtagtaaaaattgaaccactaggagtagcacccaccagg
gcaaggagaagagtggtggagagagaaaaaagagcaattggaatgggagctcttttccttgggttcttaggaaca
gcaggaagtactatgggcgcggcgtcaataacgctgacggtacaggccagacaattattgtctggtatagtacaa
cagcagagcaatctgctgagggctatagaggctcaacagcatctgttgaaactcacagtctggggcattaaacag
ctccaggcaagagtcctggctgtggaaagatacctaaaggatcaacagctcctaggaatttggggttgctctgga
aaactcatctgcaccactactgtgccctggaactctagttggagtaatagatctcaggaggaaatatggaacaac
atgacctggctgcaatgggaaaaagaaattagcaattacacagacataatatatagtctacttgaagaatcacag
aaccagcaggaaaagaatgaacaagacttattggcattggacaagtgggcaagtctgtggaattggtttgacata
acaaattggctatggtatataaaaatatttataatgatagtaggaggcttaataggattaagaatagtatttgct
gtgctttctataataaatagagttaggcagggatactcacctctctcgtttcagtacctgtggaaccttctgtta
tattgggttcgggaactgaaaattagtgctattaatttgcttgataccacagcagtagcagtagctgagtggaca
gataggattatagaaataggacaaagacttgctagagctattctcaacatacctagaagaatcagacagggctta
gaaagggctttgctataa
>#91 KF716478 2232 nt
gctacagacaaattgtgggttactgtctactatggggtacctgtatggaaagacgcagagaccaccttattttgt
gcatcagatgcgaaagcatatgagaaagaaatgcacaatgtctgggctacacatgcctgtgtacctacagacccc
aacccacaagaagtagctttggaaaatgtgacagaagagtttaatatgtggaaaaataacatggtagaacagatg
cagacagatataatcagtctatgggaccaaagcctaaagccatgtgtagagttaacccctctctgcgtcactttg
gagtgtagcaatatcaacagcaccaataacaccgccaccaatttcaccaatttcatgggagaagacataaaaaac
tgctctttcaatatgaccacagaattaagggataaggagaagaaagtatattcacttttctataaagtggatata
acaccattggaaaacagcaacgaaactaagtataggttaataaattgtaatacctcagccattaaacaagcctgt
ccaaaggtatcctttgagccaattcccatacattattgtgctccagctggttttgcgatcctaaagtgtagagat
gaggagttcaatggaacagggccatgcaaaaatgtcagcacagtacaatgcacacatggaatcaagccagtagta
tcaactcaactactattaaatggcagtctagcagaaaagaaggtaatgattagatctgaaaatttcacaaacaat
gctaaaaacatcatagtacaatttaataagtcagtagaaattaattgtaccagacctaacaacaacaccagaaaa
agtgtacatataggaccaggacgagcattctatgcaacagatataataggggacataagatgggcatattgtaat
gtcagtagatcagaatggaaggaaactttacaacaggtagtcaaacaattaggaaaccattggaataaaacaata
cactttaataattctgcaggaggggatttagaaattacaacacatagttttaattgtggaggagaattttctat
tgtaatacatcaagcctgtttaatagtacttgggtatatggtaatggaagctggggtcaaatagcacggggtta
aatagtacaatgataaataatactattttcagtgataccataattcttcaatgcagaataaagcaaattataaat
atgtggcagagagcaggacaagcaatatatgcccctcccatcaaaggagtaataagctgtagatcaaacatcaca
ggactaatattaacaagagatggtgggagtaacagcaatgcaagtacaaatggaagtgaaaccttcagacctgga
ggaggagatatgagggacaattggagaagtgaattatataagtataaagtagtaaaagttgaaccactaggaata
gcaccctccaaggcaaggagaagagtggtggcgagagaaaaaagagcagttggaataggagctgtactccttggg
ttcttaggaacagcaggaagcactatgggcgcagcgtcaataacgctgacggtacaggccagaaaattattgtct
ggcatagtgcaacagcaaagcaatttgctgagggctatagaggctcaacagcatctgttgaaactcactgtctgg
ggcattaaacagctccgggcaagagtcctggctgtggaaaggtacctaaaggatcaacagctcctaggaatttgg

ggctgctctggaaaactcatctgccccactactgtgccctggaactctagttggagtaataaaagttacagtgag
atatgggataacatgacctggctgcagtgggataaagaaattagcaattacacagacctaatatatggctaatt
gaagaatcgcaaaaccagcaggaaaagaatgaactagacttattagcattaaacaagtgggcagagctgtggaat
tggtttgacttatcaaactggctgtggtatataagaatatttataatgatagtaggaggcttaataggattaaga
atagtttttgctgtgctttctataataaatagagttaggcagggatactcacctttgtcgttccaggctattagt
ttgcttgataccatagcaatagtagtagctggctggacagatagggctatagaaataggacaaggaattggtaga
gctatcctcaacatacctagaagaatcaggcagggcttagaaagggctttgctgtaa
>#92 AJ320484 2220 nt
gttgcagagaagaagtgggtcacagtgtattatggggtacctgtgtggaaagaagcaacaaccactctattttgt
gcatcagatgctaaatcatataaaacagaggtacataatatctgggctacacatgcctgtgtaccaacagacccc
aacccacgagaaatagaactggaaaatgtcacagaaaactttaacatgtggaaaaataacatggtggagcagatg
catgaggatatcatcagtttatgggatcaaagcctaaaaccatgtgtaaaattaaccccactctgtgtcacttta
aactgcactgatgcaaggaggaatgagactaggaataatattacaggaatggaaaacaatgatcaaatagaaatg
aaaaactgctctttcaatataaccacaaaattaatagataagaagaagcaagtacatgcactttttatagactt
gatgtggtacaaatagataatgatactagtaatagcaactatagcaactatagattaataaattgcaatacctca
gccattacacaggcttgtccaaaggtaacttttgagccaattcccatacattattgtgccccagctggttttgca
attctaaagtgtagagataagaagttcaatggaacaggaccatgcaaaaatgtcagcacagtacaatgcacacat
ggaattaggccagtagtgtcaacccaactgctgttgaatggcagtctagcagaagaagagataataattagatct
gaaaatctcacaaacaatgctaaaaccctaatagtacagcttaatgagtctgtagaaatcaattgtacaaggccc
tactacaaccagataagacaaagaacatctataggacaagggcaagcactctatacaacaagagtaacgggagat
ataagaaaagcatattgcaatattagtaaagcaggatggaataaaactttacagcaggtagcaaaaaaattagga
gacctctttaaccagacaacaataatttttaaaccatcctcgggaggagacccagaaattacaacacacagcttt
aattgtggaggggaatttttctactgcaatacatcaaaactgtttaacagtgcatggaatgacagtacatggaat
atagggaataataatacagggtcagataatgagacaatcattatcccatgcagaataaaacaaattataaacatg
tggcagggagtaggaaaagcaatgtatgcccctcccatcgaaggatggatcaattgtgcatcaaatattacaggg
ctcttactggtaagggatggtggtggtgcaaatgatagtcagaacgagaccttcagacctcaaggaggagatatg
agagacaattggagaagtgaattatacaagtataaagtagtaaaaattgaaccactaggaatagcacccaccaag
gcaaagagaagagtggtggaaagagaaaaaagagcaataggactaggagctatgttccttgggttcttgggagca
gcaggaagcacgatgggcgcagcgtcattgacgctgacggtacaggccagacaattattgtctggtatagtgcaa
catcaaaacaatttgctgatggctatagaggcgcaacagcatctgttgcaactcacagtctggggcattaaacag
ctccaggcaagaatcctggctgtggaaagatacctacaggatcaacagctcctaggaagttggggggtgctctgga
agacacatttgcaccactactgtgccctggaactctagttggagtaataaatctatagatgacatttggaataac
atgacctggatggagtgggaaaaagaaattgacaattacacaggtgtaatatacagattaattgaggaatcgcaa
acccagcaagaaaagaatgaacaagaactattgcaattggacaaatgggcaagtttgtggaattggtttagcata
acaaaatggctgtggtatataaaaatattcataatgatagtaggaggcttaatagggttaagaatagtttttact
gtgctttctttagtaaatagagttaggcagggatactcacctctatcgtttcaggctattagcttgtttaacgct
acagcagtagcagtagctgaggggacagatagggttatagaagtagtacaaagatttttagaggtattcttaac
gtacccacacgaataagacagggcttggaaagggcgttactataa
>#93 KT200357 2187 nt
gctacagaacaattgtgggtcacagtctattatggggtacctgtgtggaaggaggctaccaccactctattctgt
gcatcagatgctaaagcctataaaacagaggcacataatgtttgggccacacatgcctgtgtacccacagaccct
aacccacaagaagtagaattggtaaatgtaacagaaaattttaacatgtggaaaaataacatggtagaacagatg
catgaggatataattagtttatgggatgaaagcctaaaaccatgtgtgaaattaaccccactctgtgttacttta
aattgcagtaatgctaatttcaattgcaataatactaataacgctagttgtctagagatgcaggcagaagtaaaa
aattgctctttcaatatctccacaagcataaaaaacaaagaaaagaaagaacatgcactttttatagtcttgat
gtaatgtcaatagataatacaagctatgcattgagacagtgtaacacctcagtcattaaacaggcctgtccaaag
gtatcctttacaccaattcccatacattattgtgccccagctggttttgcgattctaaaatgtaaggataagaaa
ttcaatggaacaggaccatgtaaaaatgtcagcacagtacaatgtacacatggaattaagccagtagtgtcaact
caactgctgttaaatggcagtctagcagaagaagaggtagtgattagatctgaaaatctcacagacaatgctaaa
accataatagtgcagctgaatgaaactgtaaaaattaattgtacaagacccagtaacaacacaagaaaaggtata
cacataggaccggggagtgcattttatgcaacaggagaaataataggagatataagacaagcacattgtaacgtt
agtagaacagaatggaacaaaactttaggatacatagttgaaaaattaagagaacaatttaagaatacaacaata
atctttaaccagtcctcaggaggggacccagaaattgaaatgcacagttttaattgtggaggggaatttttctac
tgtaatacaacacagctgttcaatagtacttgggagccagggaagaatactactacagagggaaatgacactatc
acgctcccatgcagaataaaacagattataaacaggtggcaggaagtaggaaaagcaatgtatgcccctcccatc

agaggacgaattaattgtacatcaaatattacagggctgttattaacaagagatggtggtaacaataataaccag
acaaataggacagagacctttagacctcaaggagggaatatgaaggacaattggagaagtgaattatataaatat
aaagtagtacaagttgagccattaggaatagcacccaccaaggcaaggagaagagtggtgcagagagaaaaaaga
gcagtgggactaggagctgtgttccttgggttcttaggagcagcaggaagcactatgggcgcagcgtcaataacg
ctgacggtacaggccagacaattgttgtctggtatagtgcaacagcagaacaacctgctgagggctattgaagcg
caacaacatatgttgcagctcacagtctggggcatcaaacagctccgggcaagagtcctagctctggaaagatac
ctacaggatcaacagctcctaggcatttggggttgctctggaaaactcatttgcaccactaatgtgccttggaat
gctagttggagtaacaaaacccgtagtgagatttgggaaaacatgacctggatgcagtgggaaagagaaattagc
aattacacagcaacaatatactccttaattgaagaatcgcaaatccaacaagaaaagaatgaacaagaattatta
gaattagataagtgggcaagtttgtggaattggtttgacataacaaaatggctgtggtatgtaaaaatattcata
atgatagtagcaggcttagtaggtttaagaatagcatttactatattttctttagtaaatagagttaggcaggga
tactcaccattatcattccaggcagttagtttatttaatgccacagctataacagtagccgaagggacagatagg
gctatagaattacttcaaagcattggtagagctattctccacatacctagaagaataagacagggcttagaaagg
gctttgcaataa
>#94 AY331292 2226 nt
gctgaaaaagactcgtgggtcacagtctattatggggtacctgtgtggaaagaagcaatcaccactctattttgt
gcttcagatgctaaagcatataagacagaggtacataatgtttgggccacacatgcctgtgtacccacagacccc
aacccacaagaagtagtattggcaaatgtgacagaacattttaatatatgggaaaataacatggtagaacaaatg
catgaggatataatcagtttatgggatgaaagcctaaagccatgtgtaaaattaaccccactctgtgttacttta
aattgcactgactatattaataacagtactgaaagtaagaaaaccaatgaaggcttgaacagtactatagaaatg
aaaaactgctctttcaaaataaccacaggcatgagagataagatgcagaaagcatatgcactttttttataagctt
gatatagtaccaatagatgatgataatattaataagactagcaccaaccataattataccagctataggttaata
agttgtaacacctcagtcattacacaggcctgtccaaaggtatcctttgagcccattcccatacattttttgtgcc
ccggctggttttgcgattctaaagtgtaacaacaagagttacacaggaaaaggaccatgtacaaatgtcagcaca
gtacaatgtacacatggaattaggccagtagtatcaactcaactgctgttaaatggcagtctagcagaagaagag
atagtrattagatctgccaatttctctaacaatgctaaaaccataatagtacagctaaataaaactgtagtaatt
aattgtataagacctaacaacaatacaagaaaaagtatacatataggaccagggagagcattttatgcaacagga
gaaataataggagatataagacargcacattgtacccttaatggaacacaatggaataacactttaagacagata
gctttaaaattaaaagaacaatttaagggtaaaacaatagtctttaatcactcctcaggaggggacccagaaatt
gtaaagcactcttttaattgtgcaggggaatttttctactgtgatacaacaaaactgtttaatagtacttggcat
ggtaatgaaacatcttggaataatactgaaaagttaaatgacactatcatactcccatgcagaataaaacaaatt
ataaacatgtggcaggaagtaggaaaagcaatgtatgcccctccaatcgaaggactaattaactgttcatcaagt
attacggggctgctattaacaagagatggtggtaagagcaataatcagactgagatcttcagacctggaggagga
gatatgagggacaattggagaagtgaactatataaatataaagtagtaaaaattgaaccagtaggagtagcaccc
accaaggcaaagagaagagtggtgcagagagacaaaagagcagtgggactaggagcgttgttcattgggttcttg
ggagcagcaggaagcactatgggcgcagcgtcaatgacactgacggtacaggccagacaattattgtctggtata
gtgcaacagcagaacaacttgctgagggctattgaggcgcaacaacatctgttgcaactcacagtctggggcatc
aagcagctccaagcaagagtcctggctgtggaaagatacctaaaggatcaacagctcctggggatttggggttgc
tctggaaaactcatttgcaccactgatgtgccttggaatgatagctggagtaataaatctctggataagatttgg
aataacatgacctggatgcagtgggaaagagaaattgacaattacacagatgtaatatacaacttacttgaagaa
ttgcaaaaccaacaagaaaagaatgaacaagaattattgcaattagataaatgggcaagtttgtggacttggttt
gacataacaaaatggctgtggtatataaaaatagtcataatgataataggaggcttaataggattaagaatagtt
tttgttgtactttctatagtgaatagagttaggcagggatactcaccattgtcgtttcaggctgttagcttgctt
aattccactgccatagtagtagctgaggggacagataggattatagaagtagtactaagagcttttagagctgtt
cttcacatacctacaagaataagacagggcttagaaagggctttgctataa
>#95 KP109514 2166 nt
gctgaggaattgtgggtcacagtctattatggggtacctgtgtggaaagaagcagtcaccactctattttgtgca
tcagatgctaaagcatatgacaaagaggtacataatgtttgggccacacatgcctgtgtacccacagaccccaac
ccacaagaaatagaattggaaaatgtgacagaaaattttgacatgtggaacaataacatggtagaccagatgcat
gaggatataattagtttatgggatcaaagcctaaaaccatgtgtaaaattaaccccagtctgtgttactttacat
tgctgtaagttgaatgatactaagtgccatgatttgaatgtcactaatagtactacccctgaaggtaacaatacc
actaatattactaacaatggtggtgatgatttaagaaattgctctttcaatatgaccacaataatgacagagaag
atgcagaaaaattatgcacttttctatacatctgatatagtacctatagataatacagataatactagtgactat
aggttgataagttgtaacacctcaatccttacacaagcctgtccaaaggtatcctttgagccaattccaatacat
tattgtgccccggctggttttgcaatcttaaagtgtaaagacaagacattcaatggaacaggaccatgtacaaat

gtcagcacagtacaatgtacacatggaattaggccagtagtatcaactcaattgttgttaaatggcagtctagca
gaggaagacatagtaattagatctaaaaatttctcggacaatgctaaaaccataatagtacagctgaatgaatct
ataccaattgtgtgtgtaagacccaacaacaatacaagaaaaagtatatctataggaccagggagaacatggtat
gcaacaggagacataataggagacataagacaagcatattgtaacattactgttgggaattggagtaacacttta
caacggatagataaaaaattaaaagaacaatttagaaataaaacaataaactttaatcaatcctcaggaggggac
ccagaaattgtaatgcacagctttaattgtggaggggaatttttctactgtaatacaacacagctatttaataat
acttggcttacttggaaggccaatgcaagtgatcctgaaaaaaataacacaaaaaatatcacactcccatgcaga
ataaaacaaattataaacatgtggcaggaagtaggaaaagcaatgtatgcccctcccatcgcaggacaaattaaa
tgtgtatcaaatattacagggttactattaacaagagatggggggtcaggacaataacactaacggtaacgagacc
ttcagacctggaggaggagatatgagggacaattggaggagtgaattatataaatataaagtagtaaaaattgaa
ccattaggaatagcacccactaaggcaaagagaagagtggtgcagagagaaaaaagagcagcactaggagctatg
ttccttgggttcttgggaatggcaggaagcactatgggcgcagcgtcaataacgctgacggcacaggccagacaa
ttgttgtctggtatagtgcaacagcagaacaatctgctgagggctattgaggcgcaacaacatttgttgcaactc
acagtctggggcatcaagcagctccaggcaagaatcctggctgtggaaagatacctacaggatcaacagctcctg
gggatttggggttgctctggaaaactcatttgcaccactactgtgccttggaatgctagttggagtaataaatct
ctggataccatttggaataacatgacctggatgcagtgggatagagaaattaacaattacacaagcttaatatac
accttacttgaaaaatcgcagaaccagcaagaaaagaatgaacaagaattattggaattggataagtgggcaaat
ctgtggaattggtttgacataacaaattggctgtggtatataaaaatattcataatgatagtaggaggcttgata
ggtttaagaatagtttttactgtactttctataataaatggagttaggcagggatactcaccattatcgtttcag
gcaattagcttgatcaacgccgcagcagtagcagtagctgaggggacaggtaggccacgcacctag
>#96 AF146728 2220 nt
gcaacagaaaaattgtgggtcacagtctattatggggtacctgtgtggaaagaagcaaccaccactctattttgt
gcatcagatgctaaagcatatgataaagaggtacataatgtttgggccacacatgcttgtgtacccacagacccc
aacccacaagaaatattactagaaaatgtgacagaagagtttaacatgtggaaaaataacatggtagaacagatg
catgaggatataatcagtttatgggatcaaagcctaaagccatgtgtacaattaaccccactctgtgttacttta
aattgcactgatgagttgacgaatgttacttttactaatagtaggcatgtgactaatagtagttatgtgggaagt
atggaaaaaggagaaatgaaaaactgctctttcaacatcaccacaagcataagagataagaggcacaaagaattt
gcactttttataaacttgatgtagtacaaatagatggtagtaatactagctatagattaataaattgtaatacc
tcagtcattacacaggcctgtccaaaggtatcctttgagccaattcccatacattattgtgccccggctggtttt
gcgattctaaagtgtaacaataagacgttcaatggaaaaggaccatgtgcaaatatcagcacagtacaatgtaca
catggaattaggccagtagtgtcaactcaactgttgttaaatggcagtctagcagaaaaagagatagtaattaga
tctgacaatttcacggacaatgctaaaagcataatagtacagctgaatgaatctgtagaaattcattgtatgaga
cccaacaacaatacaagaaaagggatatatgtaggaccagggagacacatctatgcaacagagaaaatagtagga
gatataagacaagcacattgtaacatcagtagaacaaactggactagcgtgctaagacagatagctgtaaaatta
agagaacgatttaagaataaaacaatagtctttaatcactcctctggaggggacccagaaattgtaaggcacagt
tttaattgtggaggggaatttttctactgtaattcaacacaactgtttaatagtacttggtttaatagtactggg
aatgatactgaaagggcaactaacaatacagaaaatatcacactcccatgtagaataaaacaaattataaacatg
tggcagaaagtaggaaaagcaatgtatgcccctcccatcaacggacagattagatgttcatcaaatattacaggg
ctgatactaacaagagatggtggtaatcaagagaacaagaccgagatcttcagacctggaggaggagatatgagg
gacaattggagaagtgaattatataaatataaagtagtaagaattgaaccattaggagtagcacccaccaaggca
aagagaagagtggtgcagagagaaaaaagagcagtgggaatgataggggcaatgatccttgggttcttgggagca
gcaggaagcactatgggcgcagcgtcactggcgctgacggtacagaccaggcaattgttgtctggtatagtgcaa
cagcagaacaatctgctgagggctattgaggcgcaacagcatctgttgcaactcacagtctggggcatcaaacag
ctccaagcaagaatcctggctgtggaaagatacctagaggatcaacagctcctagggatttggggttgctctgga
aaactcatttgcaccacttctgtgccttggaatgctagttggagtaataaatctctaagtgacatttgggataac
atgacctggatgcagtgggaaagagaaattggcaattacacaggcttaatatatcatttacttgaagaatcgcag
aaccaacaagaaaagaatgaacaagaattattggcattagataagtgggcaagtttgtggaattggtttagcata
acaaaatggctgtggtatataaaaatattcataatgatagtaggaggcttagtaggtttaagaatagtttttgct
gtactttctctagtgaagaaagttagggagggatactcaccattgtcattgcaggctattagcttgctcaacgcc
acagctatagcagtagctgagggaacagatagggttatagaagtagtacaaagagcttgtagagctattctccac
atacctagaagaatcagacagggcttggaaaggcttttactataa

Supplementary File S6. Dataset of 96 nucleotide sequences of the regulatory/accessory genes taken together (*tat*, *rev*, *vif*, *vpr*, and *nef*) from primate lentiviruses: 37 sequences from SIV infecting Old-World monkeys (#1 - #37), 4 from HIV-2 (#38 - #41), 20 from SIV infecting African apes (7 from SIVgor, #42 - #48, 13 from SIVcpz, #49 - #61), and 35 from HIV-1 (9 from group O, #62 - #70, 2 from group P, #71 and #72, 7 from group N, #73 - #79, and 17 from the pandemic group M, #80 - #96). From each genome sequence we selected the non-overlapping region that encodes the Tat, Rev, Vif, Vpr, and Nef proteins. Each nucleotide sequence contains the NCBI ac. number and the length of the coding region.

```
>#1  AF468658  1314 nt
gcaactaaacatcatatatggtcaggaaaaactccatttgtgtatatccaccactatcaactacaacatcaaa
ggtttacacaaaacaaaattcgattagcattagatactaggcaggtaggagaggaagtagaagccacatatat
tgagataaccatcctatgggacacaactagtcatggaccagccagtctatcccgatccacctactaccaacag
gcagtcacaatagagtgggtttacaacagaagtaagctaggagcaagggaaacagacataacatggtacagta
ctaatttgaccccagacgtagcaatgcaaatcatacacacaaggtactttccatgttttcagaacgaagacgt
acggagggcaataaggggtgaacagctcttaggatattgtgaccacccggaagctcacccacaggtaggaaca
cctcacacgcttgaaaggctagccttctttgcgtacgttagatacgtaacggagctccctgccgatccggatt
ggacagtggatcaggcagcaatagcatgtgcaatagattatgtcaggagagtgcagacattactctttagaca
ctttagagatgggtgtttccacagatacaatagaattgtcagaaggtacccagtaatcaggcccttaaggggt
accgccccaccagatagtaactctgttcctgacccggcagagtgtcttttgcctaagtggcaacagccaggag
ccgcgccaaacaccccgtgctctgcttgctattgcaaaaggtgtgcttttcattgtcagttgtgttttctaag
gaagggactaggtctttccctttccgaatcaccctccaggcaaaattgcatcgattatgaaccatcatgccca
gtgagaccacaggtgccgcttagagaccccacctacaaacttatggtggatctctcgcacttttttgaaagaaa
agggaggactggaggccatgttttattgtgaagatagacaccaaaagctagagagctattgctattatgagtg
gggcattgtgcctggatggctccagtggactccaggaccaggaatccgctacccaactatgccaggcttctgc
tggtgcttacggccagtggccatgactgaggactctgagcccggggatgatcaatatctgttgaaccatcccg
cctatcagggacagcaagaggaccaccatagggagatcttggtcttctccttctgctctaggctagccttgaa
atcaggctggcagatgaaccagctgcagcaggaggagcggaagaagcgcctaaccgcaaaccgcttcctatag
>#2  AF468659  1251 nt
aggaaggtagactggttgcttagagctactaaacaccacatatggtcaggcaaaacaccatttgtatatgtcc
atcactaccaattgcaacaccaaagatttacacaaaacaagattcgattagcaatggatactaggaaggtagg
ggaagaagtagaagcaacatatatagagataactatcctatgggacacaaccagtcatggaccagctagctta
tcccggtccacctattaccaacaagcagtcataatagaatgggtatacaacagaagcaaactaggagcaagag
agggagacataatttggtacagcactaatctgaccccaggagtagcaatgcaaattatacatggaaaatactt
tccatgctttcagaacgaagacatacggagggcaataaggggtgagcagatcctaggaaattgtgaacacccg
gaagctcacctgcaggtaggaacacctcagacgcttgagcagttagccttctttgcatatgtcaaacatgtga
cggagctccctgccgatccagattggacagtggatcaggcagcaatagcatgtgcaatagattacatcagggt
gcaaacacttctctttagacactttaaagacccggaagagtgtctcttgcccaactggcagcagccaggagcg
gcgcccgccaccccgtgctctgcttgctactgcaagaagtgtgcccttcactgtcagttgtgctttctaagga
agggactaggtctttccctccataggtccagtgagccaaaacccaagagaggaagcctcgaggagtatgaacc
atcatgcccagtaagaccacgggtaccacttagagaccccacctacaaagtcatggtggatctctctcacttt
ttgaaagaaaagggaggactggagggcatgttttactgtgaggacaggcaccagaagttggaacaatatgcct
atctggagtggggcctggtgcctggatggctctcattcactccaggcccaggaacccgctatcctacgatacc
aggattctgcatctgtctgcggccagtagccacaactgaggactctgagcctggggatgatgaatatctgctg
acccaccctgcctatcagggacggagtgaagaccagcataaggagttcttggtcttctccttctgctccaagc
tggccataaagtcaggcatacagctagaccagctgcagcaggaggagcggaagatgcgcctaaccgcaaaccg
cttcctatag
>#3  EF070329  1371 nt
tgcactaaacattatatcttctcagggaaagctccatttacatatgtccaccactatcaattgcagcatcaga
gattcacccagaacaaaataaaaatacctttgagcataaacagaatagaaggaggcccaacagagactacata
catagagataaccatattgttagatgtcacaaacgtagggccagccagtttatcaaggtccacctattggcag
```

```
caatcctatatcctgaagtggagatacatcagggcagccccacaaaatagagagatagacatggtgcattacg
agaccttcctagacccagaagtgaccatgcagattatacacacacactacttctcttgtttccaacagagaga
catccaaagggcaatcagaggagaacagctcctaagaaagtgtgagcacatcaagacacactacccgaaggta
ggaactcctctatctctagaaaagctagcattctttgcatacatcaaacatacagtcacagagctcccagcag
atccacaatggtctgtagatcaggcagccataacatgtgccattgattacattaaatcagtccaaaccctctt
gttcagacactatagggacggatgttaccatagctatgctcaaacaatcaggagatacccaccgttgcgccca
ttgagagggacgcagcccccagactctaattccatgccaaatgctgaccccacaccatctttgagaccctcca
gatatagaatggatgaaatggacccttcagttgaaggcctgcctccggaccagcgtcccggagcggcaccccc
cacaccttgcaccaactgttactgcaaatgttgcgcgtttcactgtatattgtgcttccagaaaaaagcatta
ggtatctcaaacaagggccgcaggcaaatcatagaagaacatgagtgctcctgcccagtacgcccgaaagtcc
cgattcgtgatgtcaactacaaactcatgatagacctctctcactatttgaaaaaaaagagaggactagaagg
aatgtttgtgtgcatagacagactgcaaaaactagagacttatgcctatgtaaaatcaaaaatagtcccaaga
tcactaatatacacggatggcccaggcacccgttacccacggcaaccagggttcctgtggtgcttgagaccgg
tagccatgacagaggagtcggagccaggagatgaccagtacctcctgacacacccggcctatgtgggtcggga
tgaagaccaccacaaagagttcctggtcttctcctttgctctaggctggccctaaagtcaggcccgcagctt
aatcaaatacaacaaaaagagcggaagagacgcttaaccgcaaaccgcatcctctag
>#4  EF070330  1359 nt
gctacaaaacatcatatcttttcaggaaaaacaccatttgtgtatgtacaccactaccaggtacaacaccaaa
gattttcccaaaacaagatcaagctagcattgacacaaaacaccttagaatcaggagagacagaagtcacctt
catagagataacagtcctatgggatgtgactaatgtaggaccagcaagcctatccaaaagtacctattggaag
cagtcatacatcctggaatgggtatacatgaggaaaggtccaagagatagagaacggactatgtttggtatc
acacatacctcacacctgagatagccatgaagatcatacacacccaccacttctcttgtttctatagccagga
tatcccgagggtcatcagaggacaaccacccttaggggattgtgaacacccaggagcacatacaaaggtagga
ccccctcccactctacaggtattagcactattaacattaaagaaagtaacggagctaccagccgatcctaatt
ggacagtggatcaggctgcaatagcatgtgccatagattacattaggagaacacaaacactccttttcaggca
ttatcgagagggatgttatcataggtactctaacacaatcaggagataccccaacataagacccttgagaggg
acacaagctcctccttctaattcaatgcctaatgctgatcctactcctcctttgagaccctctagatatagga
tggatgaggatccttcagtagaaaaccttcccccggaacagcgccccggcgcagcgccgaccacgccttgcag
ctcctgctattgtaaacggtgtgcttttcattgcatgttgtgcttccaaaagaaagctttaggtatctccgga
agcaatgagcggccactcatcgaagaacatgagcgttcatgcccagtgcggccgcgagtgcccctacgggatc
ccacatacaagatgatgattgacctctctcattatttaaaagaaaagggaggactggaagggatgttttattg
tgaggaaagacaccaaaaattggagacttatacctatgtggactggggaattgtaccaggatggctacagttt
acagagggcccggggacccggtacccaacactgccagggttcctgtggtgcctaagggaagtcgccataactg
aggactctgaggagggggatgaggaatatctcctcacccatgcagcctaccaaggcaaggaggaggaccccca
taagcagttcttggtcttctcattctgctctcgcctcgccatgaagtcaggcaggcaattagatcaaatacag
caagaagaacggaagaggcgcttaaccgcaaaccgcatcctatag
>#5  AY340701  1395 nt
tgcactaaatggcacatactcacaggaagtgcaccctttgtctatgtacatcactaccagctgcacaatcaga
gattctcacagaacaaaattaaactccccttagacctaggaatcactcaagaaggagagtcatgggcaacata
tttggagataacaatttattgggacttaacaaacgtgggacctagtgcactctcaccaacagtgtacaacaaa
caggcctacacaatagaatgggtttattggatgcaaaaggtggaacgggtccctttagatcccgtgtggaatc
caaacccaagagatagagaggtcagaacatggtatgctacccatctgacgccggacctggcgaatcagatagt
acatacacattattttgcatgcttccaacagttggacgtgcgcagagcaatcagaggagagaagctcttggga
cagtgtcaacatctagcaacacactacccgaaggtagggaccccgttgaccttagagaaattagccttccttg
ccctagtgcgaccggcctggaatgcagaccaggcatgggcagcatgtgccattgattatactagatgggtcca
aaccatcctttataggcactacagggaaggctgttaccatagatatgcagagcaaataagaagataccctgtt
cttaggccaatgagagggactgctcccggcccaaccagttctgttccacaggccgatccagacaatcccagaa
gaccttccagatatagaatggatgaacctgttgacccagacttacccaaagaacaacacccccccagcaacccc
acgcacaccttgtactaactgcttttgtagagtttgttgctttcactgtcagatttgcttcctcacaaaaggc
ctcgggatttcaggcagaaatggcaaacaacgccggcaacctctggacgacgacgacgacgacgacggtgtcg
gttgtccagtaaggccccgagttccgcttcgcgaccctacttggaagctcatgatggacctctctcattattt
gaaagaaaagggaggactgggagagatgttttactgtgaagacagacatagaaagatagagcaatatgcctat
ctggaatggggcttaatcccaggatggctacaatatacggaagggccaggggtcaggtacccgactatgcccg
ggttcctgtggtgcttgcgcccggtagccaccacggaggattccgaggagggagatgaggacttcctcctaac
ccatccggcctatcaaggccggatggaggacccccatagacagttcttggtcttctcattctgctccaagctg
gcagtcaagagcggcagacagctagcacagcttcagcaagaggagcggaagaagcgcttagccgcaaaccgca
tcctctag
>#6  KJ461716  1377 nt
ctaactaagtatctcaagtataaaacaaaggaattagaggaagtacaatggatacatcattaccaattatatg
cacataggtatacacaaaacaaagcaatcatcccacttaccttgaaagtatcacaggattatgtggaagcaac
```

agccatagaagtacaaatattgtgggatctgtcaagttgtagaagtagggatccacctggaacaagagacaca
caagcccttactattagttggacttacaccaggttgactaagggagacagagagatagatagaatcagctatg
agacagagattactccgcacttagctgatgcaatgatacatgtggagcatttctcttgttttacagctcagga
tttcactagagcaatcagaggacagcaattgctaggtgagtgccatcatccacattatttaggcaaagagata
cccacattgcaatacctagcactaagggcaatacaatggacagaacagcaggcaatggcagctactgcaatag
attacataaggatcgttcaaaagttattgtgggatcattataagaaaggatgctatcacagataccatgctag
agtaaggaggtttccacaactgagaccactgagaggtacagcaggacaggacacgccccatgacagtgatccg
ccacaggtgctaagaccctctcgttatcatccagataacgatccatctcaagaggggttagaaaaatggcata
gggcgcctgcacaacctccaactgcttgcactccatgcttttgcaaaaactgcatatatcactgcatattgtg
cttccaacgaaaaggacttggcatccgcgaagaaacagccgagacagccaagagaggggaagttgtacctgta
agatacaacgaaggtgacagcacccgagatgatgatgaagaaggagtgggtttcccagtatgcccacaagtac
ccctcagacaaatgacattcaagacggctgtggatttctcctggtttttaaaagaaaagggaggactggaagg
gttgttttattcccctgagagacacaagaaattggacctatatgcttataatgtatggggactagttccaggg
tggcaaggatacacccaggggccaggcacaaggttcccaacatgctttggcatcctctgggaattggtcccgg
tggagatagggccacagggcctgggagaaggagatgaaagagcatcgttgctccatgcggggcaacaagtcta
ccaggaccccccattgtgagaccctggtatggcacttcaatcccacccttgcctttgagccaggcatactgaag
gctgacaagttgggacagaagcctttgcctgggcaaaagacctttctaacaggaagagactag
>#7 KJ461714 1374 nt
ctaactaagtatctcaagtataaaacaaaggaattagaggaagtacaatggatacatcattaccaattatatg
cacataggtatacacaaaacaaagtaatcatcccacttaccttgaaagtatcacaggattatgtggaagcaac
agccatagaagtacaaatattgtgggaactatcaagttgtagaagtagggatccacctggagtaagagacaca
caaggcctcactattagttggacttacaccagattgcacaagggagacagagagatagatagaatcagctatg
agacagagattactccgcaattagctgatgcaatggtacatgtggagcatttctcttgttttacagctcagga
tttcaatagagcaatcagaggacagcaattgctaggtgagtgccatcatccacattatttaggtaaagaggta
cccacattacaatacttagcactaagggcaatacaatggacagaacagcaggcgtgggcagctactgcaatag
attacataaagatcattcaaaagttattgtgggaccactataagaaaggatgctatcacagataccatgctag
agtaaagaggtatccacacctaagaccactgagaggtacagcaggacaggatacgccccatgatagtgatcca
ccacaggtgctaagaccctctcgttatcatccagacaatgatccctcccaagaggaggtagaaaaatggcata
gggcgcctgcacaacctccaactccttgcaattcctgcttttgcaaaagctgcatatatcactgcatattgtg
ctttcaacgaaaaggacttggcatccgcgaagaagcagcagcagccaagagaggagaagttgtacccatgagg
tacaatgaaggtgacaacacccgagatgatgatgaagaagaagtgggtttcccagtacgcccacaagtaccca
tcagaaacctgacattcaaaatagctgtggatttctcctggtttttaaaagaaaagggaggactggaagggtt
gtattattcccctgagagacacaagaaattggacctatatgcttatcatgtatggggagtagttccagggtgg
caaggatacacccctggcccaggcccgaggttcccaacatgttttggcatcctctgggaattggtcccggtgg
atgtggggccacagggcctgggagaaggagatgaaagggcattgttgctccatgcggggcaacaagcatacca
ggacctccatggtgagaccctggtgtggcacttcaatcccacccttgcctttgagccaggcatactgaaggct
gacaagttgggccagcagccattgcctgggatgaagagctgcataacaggaagagactag
>#8 KJ461715 1368 nt
ctaacaaagcatctcaaatataaaacaaaggaattagaggaagtacaatggatacatcattaccaattatatg
cacataggtatacacaaaacaaagcaatcatcccacttaccttgaaagtatcacaggattatgtggaagcaac
agccatagaagtacaaatattgtgggaactgtcaagttgtagaactaggaatccacctggagtaagagacaca
caagctcttactattagttggacttacaccagattgaataaaggagacagagagatagatagaatcagctatg
agacagagattactccgcaattagctgatgcaatggtacatgtggagcatttctcttgttttacagctcagga
tttcactagagcaatcagaggacagcaattgctaggtgagtgccatcatccacattatttaggcaaagagata
cccacattgcaatacctagcactaagggcaatacaatggacagaacagcaggcatgggcagctactgcaatag
attatataaagatcattcaaaagttattgtgggatcattataagaaaggatgctttcacagataccatgttag
agtaagaagatttccacagcttagaccactgagaggtacagcaggacaggaaacgccccacgacagtgatccg
ccacaggtgctaagaccctctcgttatcatccagataatgatccatcccaagaggggttagaaaaatggcata
gggcgcctgcacaacctccaactccttgcaattcatgcttttgcaaaagctgcatatatcactgcatattgtg
cttccaacaaaaaggacttggcatccgcgaagaagcagcccaaccaggagaagtagtacccttaaggtacaac
gagggtgactacacccgaactgatgatgaagaagaagtgggtttcccagtacgcccacaagtacccatcagaa
caatgacattcaaaacagctgtggatttctcctggtttttaaaagaaaagggaggactggaaggctatttta
ttcccctgagagacaccagaaattggacctatatgcttatcatgtatggggactgactccagggtggcaggga
tacacccaagggccaggcccgaggttcccaacatgttttggcatcctctgggaattggtcccggtggaggtag
ggccacagggcctgggagaaggagatgaaagggcaaagttgctccatgcagggcaacaatcattccaggaccc
ctatggtgaggtcctggcgtggcacttcaatcccacccttgcctttgagccaggcatactaaaggctgacaag
ttgggacagcagcctttgcctgggcaggagacctttctaacaggaagagactag
>#9 JX860407 1167 nt
ctcataaagcatttaaaatataacactaaagacctacagcaggcatgttatgtcccccatcataaagttggat
gggcatggtggacttgcagcagggtaatctttccattacaaggagaatctcatttagaagtacaagggtactg

gaacttaacaccagagaaaggatggctcagtacttatgctgtaaggataacttggtacagcagaaatttttgg
acagatgtaacaccagattatgcagatacattactgcatggatcttatttctcttgctttacagcgggagaag
taagaagagccatcaggggagaacagttactgtcttgctgcaagttcccaagagctcatagaaatcaggtacc
aagtctgcaatatctagcactgagtgtagtaagtatggcagaagctcccccagaggatgaggccccacagaga
gagccgtgggatgaatgggtaatggaagtcctggaggagctcaaagaagaagctaaaaggcattttgatcttc
gcttgctaactgcgcttggtaactacatttatgataggcctcagatgtataatgaaggccagtacatgaatac
cccctggagaaaccctgcaagagaaagagccaaattagcatataggaaacaaaatatggatgatgtagatgag
gaggatgatgaattagtaggggtagcagtacatccaaaagttccattaagagcaatgtcatacaaattggcaa
tagacatgtcacatttataaaagaaaaggggggactggatggatttattacagtgagagaagacataggat
tctagacatgtacctagaaaaggaagaaggaataataccagattggcagaattacacctctgggccagggata
agatacccaatgttctttggctggttatggaaactggtaccagtagatgtatcagatgaagctcaagaagatg
agacccactgtctggtgcatccagcacagacatatcagtgggatgacccttggggagaagtcctagcatggaa
gtttgatccaacattagcttatacctatcaggcatttgttaaatacccagaagagtttggatataagtcaggc
ctgtcagaggaagaggttaagagaaggctaaccgcaagaggccttttaaaatggctgacaagaaggaaacca
>#10 U79412 1173 nt
ctcataaaatatctgaaatataaaactaaagatctacaaaaggtttgctatgtgccccatcataaggtcggat
gggcatggtggacctgcagcagagtaatcttcccactacaagaagaaagccagttagaagtacaagggtattg
gaatttaacaccagaaagagggtggctcagtacttatgcagtgagaataacctggtactcaaggaacttttgg
acagatgtaacaccagactatgcagacattttactgcatagcacttatttcccttgctttacagcgggagaag
tgagaagggccatcaggggagaacaactgctgtcttgctgcaggttcccgagagctcataagaaccaggtacc
aagtctacagtacttagcactgagagtagtaagtatggaagaaagacctccagaagatgaaggcccacaaagg
gaaccatgggatgaatgggtagtggaggttctggaggaactgaaagaagaagctttaaaacattttgatcctc
gcttgctaactgcgcttggtaattatatctatgatagaggacagaaatacaatcagggacagtttatgaatac
tccatggaaaaacccagctggagagagggaaaaattagcatacagaaaacaaaatatggatgatatagatgag
gaagatgatggcttggtaggggtaccagtgaggccacgagttcccttaagagcaatgacttacaaattggcag
tagatatgtctcatttataaaagaaaaggggggactggaagggatttattacagtgaaagaagacataaaat
cttagacatatacttagaaaaggaagaaggcatcgtgccagattggcaggattacacctcgggaccaggacct
agatacccaaagacatttggctggctatggaaattagtccctgtaaatgtatcagatgaggcacaggagggtg
aggagaattatttactgcatccagctcaaacttcccagtgggatgacccttggggagaggttctagtatggaa
gtttgatccaactctagcctacacttatgaggcatatattagatacccagaagagtttggaagcaagtcaggc
ctgtcagaggaagaggttagaagaaggctaaccgcaagaggcctcttaaaaatggctgacaagaaggaaacta
gctga
>#11 JQ864086 1164 nt
ctcatcaaacacctgaaatataacactaaagacctacagatggcttgttatgtgccccatcataaagttggat
gggcatggtggacttgcagcagagtaattttcccattaagagatgagactcatttggaagtacaaggatattg
gaatttggcaccagaaaaaggatggctcagtactcatgcagtaagaataacctggtactccagaaatttctgg
acagatgtaacaccagattatgcagacactttactgcatagcacttatttcccttgcttttcagagggagaag
tacgaagggccatcaggggagagaaattgctgtcttgctgcaagttcccgaaagctcataaaaatcaggtacc
aagcctacagtatctagcactaacagtagtaagtatggcagaaagacctccagaagatgaagccccacagagg
gaaccatgggatgaatgggtagtggaagttctggaggaattaaaagaagaagccctgaaacattttgatcctc
gcttgctaactgcgcttggtaactatattcctcagaaatatagtgagggacaatatatgaataccccctggag
gaacccagcaacagaaagagaaaaattaggttatagacaacaaaacatggatgatgtggatgatgaagatgat
gacttagtaggtgtctcagtgcacccaagagtccccttaagggccatgacatacaaattggcaatagacatgt
ctcatttataaaagaaaaggggggactggaagggatttattacaatgagaaaagacatagaatattagatat
gtacatggaaaaggaagaaggaataataccagattggcaaaattacacatcagggccaggaactagataccct
atgtactatgggtggctctggaaattagtcccagtagatgtctcagatgaagctcaggaagacgagacacatt
gcctgatgcatccggcacagactcatcagtgggatgaccccctggggagaggtactggcatggaagtttgatcc
agaattagcttatagctataaggcatttattaagtacccagaagagtttggtagtaagtcaggcttgtcagag
gaagaggtaaagagaaggctaaccgcaagaggccttattaaaatggctgacaagaaggaaacaagctga
>#12 AF334679 1278 nt
agaatagtggagcgctggcatagctgcattaaattccataagtataaaacaagagagctagagaaagcctgct
atgtgccacatcacaaagtaggatgggcatggtacacagcctctagggtgattttccccttggaggagggaag
tcacttggaggtacaagtgtactggaatctgaccccagaaaaaggatggttatcaagctatgcagttaggatt
acttggtactcagaaaaatttggacagatgtaaccccagatgtagcagatcagttagcacatagtacttatt
tcccttgctttgcagcgcacgcggtgagacaagccatcagaggagagcaggtgttatcctactgtggctacgc
ggtagcccatcactctagtgtccaaagcctacagctgttggccctaaaggtagtattacaaatggaacatgct
ccagaggatgagaccaacccaagagaaccatgggatgagtggataagagatgtactggaagaattgaaagaag
aggcactgaaacattttgaccctcgcttgcttactgctttaggaaattacgtatatgatacttacgggatac
aatagaaggggcaggggaaataattaagatattacaaaaggcattgttcttacactttagggatggatataaa
tacaatgtaggggaatttatgaacaccccatggagaaaccctgctacagaaagacaaaagcaacagtataggc

agcagtgtatggatgatatagatgaggatgatgatgagctggctggagtagttgtctggccaaaagtacccct
aagagcaatgtcatacaaattggcaatagacatgtcacattttataaaagaaaaggggggactggatgggatt
tattacagtgagaggagacatagaatcctagatatatacctagaaaaagaggaaggtatcatcccagattggc
agaattatacaagtgggccaggaataaggtacccaaagttctttgggtggctctggcagctggtgccagtaga
cgtctcggatgaggcaaacaatgatgagacccattgcttggtacatccagcccagacatatcagcattcagac
ccttggggaaaggtcctagcctggaagtttaatcctcacctagcctacacatatgaagcatttgtaaggcacc
cagaagagtttgggtggaagtcaggcttgacagaggaagaggttgagagaaggttagctaacaagccaaaacc
gcagaagaagatggcggataagaaggaaacaagctga
>#13 JX860414 1167 nt
ctcataaagcatttgaaatataacactaaagagctacagaaagcctgctatgtgccccatcataaggttggat
gggcttggtggacttgcagcagagtgatcttcccattagaaggggaatctcacttagaggtacaaggatattg
gaacttgacaccagagaagggatggctcagtacttatgcagtaagaatcacctggtacaccaggaggttctgg
tcagatgtaacaccagactatgcagacacgttactgcatggcacttatttcccttgttttacagcgggagaag
tgcgaagggccatcaggggagaacaattgctgtcttcctgcaggttcccgaaagctcataagaaccaggtacc
aagcttacagtatctagcattaagaatagtaagcatggcagaaagacctccagaagatgaaggcccacagaga
gaaccatgggatgagtgggtagtggaagttctggaagaattaaaagaagaagctctgaagcattttgatactc
gcttgctaactgcgcttggtaattatatctatgataggggagagagttacagtcagggtcagtttatgaatac
cccctggagaaacccagctagagagagagaaaaattggcatataggcaacaaaatatagatgatatagatgat
gataatgatgatttaataggagtaccagtacactcaaaagtccccttaagatgcatgacttacaaattagcag
tagacatgtcacattttataaaagaaaaggggggactggaagggatttattacaatcagagaagacatagaat
actagacatttacttagaaaaggaggaaggaatcatccctgactggcagaattacacatcaggaccaggaata
agatacccaatgatgttcggatggctgtggaaactagtcccagtagaggtctcagatgaggcacagaatgatg
agacacattgtctggtgcatccagcacagacgggccaatgggatgacccgtggggaaaggtcttggcatggaa
gtttgaccccactctggcttatacctatgaggcatatattaagtacccagaagattttggggacaagtcaggc
ttgtcagaggaagaggttaagagaaggctaaccgcaagaggccttttaaaaatggctgacaagaaggaaact
>#14 HM803689 1236 nt
cagtggcactccctagtaaaatatcatatgtataaaggaaagaaagaagcaagagaatgggaatatgtacccc
atttcaaagtaccttgggggtggtggtctcattcagaggtccacattcccttgggaaataacactaagataaa
ggtgaccacctattggaatctaacaacagaaaaggggtggttgggaacttatggggcagctcttgcttatata
gaccagaaatgtgaccctccctacttcacagatatagacccaatagtggcagacagcttaatacataagatat
atttcccctgttttacagataaggcaattagacaagctatattgggagaaaaggttttgctgtgtggattcca
gaggggacatagagatcaggtagggacactccaatacctagcaatacaagcttgggctagagagcaagtaaag
aaaatggagatgcccccggaagatgaaggccctcagagagagccaatgaatgaatggctactagacacgctta
tggagctacaggaagaagcagctaagcattttacaatggagcttttacatgcagtagggaattacatatatga
gcaatatggggatagcttagagggagttagagtactaattactttgcttcaaagggctctgttcttgcactat
agaaaatgttattgcaagaaatgtgcatatcactgccagctttgctttcttcaaaaggcattaggcattaacg
gaggaataacaatagaaacacagcaaagtgtagatgctatagattggtatgaggagacagatgataccttggt
aggtttcccagtgaaaccacaagtaccattaagacctatgacttttaaattggcaatagatctctcacatttt
ttaaaagaaaaggggggactggaagggatttattactccaccaggagacatagaatcttagatatatacttag
aaaatgagcatggaattatccctgattggcagaattacacaaaaggaccaggtattagatacccaatgatgtt
tggatggctgtggaaattagtaccagtagatgtgattgatgaggctaaggaagatgaagagcatagcctgcta
cacccagctgagacttgtgggatggaagaccccctggggagaggtcttagcttggaagtttgatcctatgctag
caatagattatgtgggctataaactgcacccagaggactttggagagaagaagaacaagacccaataa
>#15 HM803690 1236 nt
caatggcactctctagtaaaataccatcagtacaaaggaaaaaaggaagcaaaggaatgggaatatgtgcccc
attttaaagtaccttggggatggtggtctcattcagaaatccacgttcccttgggaaaaaacactaaggttaa
aattaccacttattggaatctcacagcagaaaagggatggttaagtacttatggagtagggatagcctatata
gataacacttgtgacccttgctactttacagatatagatcctattctggctgacaagatgatacataatgtct
attttccctgttttacagatcaggcaattaggaaagctcttttaggagagaaagtgttagtgtgtggctttca
gcgaggacacagagatcaggtagggactcttcagtaccttgctttacaagcttgggttaaatcccagttacag
aaaatggaaattccccaggaggatgaaggccctcaaagggaaccatatgataaatggcttttagacacgctta
tggagctacaagaagaggcagctaagcattttaccatggagcttttgcaggcagtaggaaattacagttatga
gcagcatggagaaagtatagaaggggttaagaaaatgataactttgttgaatagagctttgtttttgcactat
agagcttgctattgtaagaagtgtgcttaccactgtcagctttgctttcttcaaaaggcaataggcatacaag
gagggtttactatagaaacacagcagtcagtagatgctatggattggtatgaggagacagatgacaccttggt
aggatttccagtcaagccacaagtaccattaagagcaatgagtttcaaattggcaatagacatgtcccacttt
ttaaaagaaaaggggggactggaagggatttattattcaataagaagacatagaattctagatatatacttag
aaaatgagcatggaataattcctgattggcagaattacactccaggacctggtcccaggtacccgaccttctt
cggatggttgtggatgctggtaccagtggatgtgagtgatgaggcaaaagaggatgaggaacatagtctcctg

catccagcagaaagtagtgggattgaggacccttggggagaaacattggcttggaagtttaatcctatgttag
cagtagattacataggctatagactgcaccctgacttctttggagagagaaagaacaagacccagtaa
>#16 M58410 963 nt
caattgccttgggaatacagacatcattggcaggtgcaatggcagttttggacctacagccagttcattatcc
ccttatcaaaagatgattacatagaagtgaatatttatcacaacctcaccccagaaagaggatggctctcaag
tcatggagtagggttatcctattaccatcaaaagggatataagacagaagtagatccaggaacagcagacaga
atgatacacctatattattttaactgttttacagatagagccatccaacaggctatcagagggggagaagtata
cgtggtgcacattcaaggaaggacataaaggtcaggtacaatcactgcaacttttggcactagttgcatatac
aaccccatggaatgagataggctacaagtactatagaattgttcaaaagtctatgtttgtacatttcagatgt
ggttgtagaaggagaggacctttaggctatgttaaaataggattttagtgatagtgattattctaggattaa
gatttgcatgggtattatggggatgtatcagaaatattaggcagggatataatcctctcccccagggtaaaat
gactccagacggccgccgcctgcaagaaggagacacctttgatgagtgggatgatgatgaagaagaagtaggc
ttccctgtgcaacctcgagtccccttaagacagatgacctataaattagcagtggacttttcccactttttaa
aatcaaaggggggactggatgggatatattactctgaaagaagagaaaagatcctgaatttgtatgccttgaa
cgagtggggaataatagatgattggcaagcttactcaccaggcccggggataaggtacccgagagtctttggc
ttctgctttaagctagtcccagtggacctgcatgaggaggcacgcaactgtgagagacactgtctgatgcatc
cagcacagatgggg
>#17 LC114462 1155 nt
catataagtagatggcaaggcatagtaagatactggatggaaaaaaggggaatacagtgggaatataagatgc
actataaaattcattgggcatggtacactatgtgtcaatacattatccctctggaaacaggagacatctatgt
agacatgttctggcacttgaccccacagacgggatggctctccacatatgcagtagggatacagtatttaagt
taccaggggaattataggacagaattagatccagggacagctgacagtatgatccacctccactacttcaatt
gctttacagaaagagccatccagaaagccatcaggggagaaaggttcgtcttctgcaactacccagaaggaca
caaacagacagggcaagtacagacccttcaattcctagccctacaagcagtacaagtgccaatgagtgagcga
gcctataggtattataggattgttcagaaagctctgtttgtacatttcaggtgtggatgtagaaggaggacac
catttgaaccttacgaggagaggagaaatttacaaccctgtaataataagtgcttttgcaagcgctgctgtta
tcattgccagctttgtttcctacaaaaaggattgggtataaataagagaccaacaaagggtaggatgactccg
gaaggacgacgtctgcaagagggagacacttgggaagaatggagtgatgatgaagaagaaataggcttcccag
tcaagcctagagtgcctctccgccaaatgacttacaaacttgcagtggatttctcgcacttttttaaaagaaaa
gggaggactggaagggatttattactcagaaaggagagataagatcctgaatttatatgccttaaatgaatgg
ggaatcttggatgattggaatgcatggacaccaggaccaggaataagatatccgcgctgctttggcttctgct
ttaagctagtaccagtagagttacatgaagaagcacagaattgtgagaggcactgcctggtccaccccgccca
gatgatagaagaccccgatggaatcaaccatggagaagtcctggtttggaagtttgacccaatgctggcggtt
gagtttaaggcagggatacacttttcagacatgcatgcgacagttggtaaaggaaactag
>#18 KR862336 1032 nt
tggcaaggaatagttaggttttggatgaaaaaacgaggattaaattggcaatatcagatgcattatcagatac
attgggcatggtatacaatgagcagatacacaattcctcttacttcaggagatatagaagtagacatctattg
gcacttgacaccagaaaaaggatggctctctacttatgcagtaggaatacagtatctttccaagataggacaa
tatagaacagaattagacccccaaacagctgacagtatgattcactgtcattactttaattgttttacagaaa
gagccattcaaaaggcaatcagaggggaaagattcgtcttctgcacttatccagagggacataaacagacagg
gcaagtacaaacccttcaatttcttgccttggtagcagtacaaagagcatacaaatattatagactggtgcaa
aaagccctgtttgtacatttcagatgtggatgtaagcgcatgacagcctttgatccttacattataggattga
gattactctatacagtatacttgtgcatctctagggttaggcagggttataccccttgtctcaacagaaagc
ttggcagtaccttcacggagaagaaaaaggcagaccaaggaatggaaaaatgagaccagaaggtagatatcta
caggaaggagacacctgggatgaatggagtgatgatgaggaggaagtaggctttccagtgaagccaagagtac
caataagacagatgacttacaagcttgcagttgattttcgcactttttgaaagaaaagggaggactggatgg
gatttattactccgagaggaggaataagatcctggatctgtatgccctgaatgaatggggaatcattgatgat
tggaatgcctggacaccaggacctggcattcgttacccgcgctgctttggtttctgctttaagttggtaccag
tagatctgcatgaagaagcacaaacatgtgaaagacattgccttgttcacccagctcagatgggagaagaccc
cgatggaata
>#19 KR862356 1065 nt
tggcagggaattgttagattctggatgaccaaaagacaactcccatggaagtatgaaatgcattaccaaattc
actgggcatggtatacgatgagcaggtacaggattccactcaaaaatggagaaattgttatagatctctattg
gcatttaaccccagaaaaaggatggctctcaacttatgcagtagggatacaatatttaggtaatcaaggacac
tatagaacagaaatagacccccatacagcagatagtatgatacatacagagtatttcacttgttttacagaaa
gagccatccaaaaggcgatcagaggagagagattcatcttctgcaactacccagagggacataaacaactagg
gcaggtacagaccctacagtttctagccttacaggtagttcaaattcctagactagagagggcatataagtat
tacagactggtgcaaaaagccttgttcgttcacttcaggtgtggctgcaggcgcaggacaccttttgaaccct
acgaagagagaaggatggacaaggggggaggcgtttatcatcataggcataatagggttaagattgctttacac
agtatacacatgcatagctagggttaggcagggttattctcctctgtctccacagacagctttacagcttcta

caaccaagaaaaaggaatggaaaacttacagaggacggaaggagactacaagcaggagataactgggaagatt
ggactgatgacgaagatgaagtgggcttcccagtgagaccacaagtccctctgagacaaatgacttacaaatt
ggcagtggatttctctcatttttgaaagaaaagggaggactgagtgggatttactactcagaaagaagagat
aagatcctgactctctacgcactcaacgaatggggaatcatagatgattggaacgcttggaccccgggacctg
ggatccgctatccccggaccttcgggttctgcttcaaattggtacctgtaaacctgcatgaggaggcagagac
ttgtgagagacattgccttgtccaccccgcccagctgggagaa
>#20 KR862363 1005 nt
ttttggatgaataagaggggcctcccatggcaatatacaatgcattacaaaattcactgggcatggtacacaa
tgagtagatacacaatacctgtagggaggggacaaatagtaatagatctttattggcatttgaccccagaaaa
aggatggctctctacatatgcagtaggcatacagtatttggacttgcaaagctcctactggacagaagtggac
ccagaaacagcagacagtatagtacattgtcactattttaattgctttacagagagagccgtccagaaggcaa
tcagaggggaaagattcatcttctgcaactacccagagggacataaacagacagggcaagtacaaactctcca
actcctagctctcattaaagttcagagagcatataaatattacagattagttcaaaaggccttgtttgtccat
ttcagatgtggttgtagacgtaggggaccttttgatccctacataataggcttaaggttgctatatagtgtgt
atctttgcatagcaagggttaggcagggttattctccccttcttccgcagacagcttttcaatacttgcagaa
gcccaggaagaaaaatgggaacatgactgcagaagggaggagattacaacaaggagatgagtggagcgaatgg
tccgatgatgaagaagaagtcggcttcccagtcaaaccacgagttccacttaggcagatgacctataaacttg
cagtggatttctctcacttttaaaagaaaagggaggactgagtgggatttactactcagaaagaagaaataa
gatcctgaccctttatgctctcaatgaatggggaatcattgacgactggaatgcctggaccccaggtccagga
atcaggtacccgcgcaccttcggcttttgctttaagcttgttccagtagtcctgcacgaggaagcagaaacat
gtgagagacattgcctcgtccaccccgcgcagatgggagaagacccggatgggata
>#21 M29975 1260 nt
tattggatgaataaaaggaatctgaaatgggaatacaaaatgcattatcaaatcacttgggcatggtacacta
tgagcagatatgtaataccccctcccaggaagtggagaaatccatgtggatatctattggcatttagctccaaa
acaaggatggctctcaacttatgcagtaggaatacaatatgttagcctagtaaatgataaatatagaacagaa
ttagatcccaatacagcagactccatgatacattgtcattattttacctgttttacagatagagccatccaac
aggcactaaggggaaacaggttcatcttctgtcaatttccaggaggacataaactaacaggtcaggtaccctc
cttgcaatatttagcattactagcccatcaatattatcgcttagttcagaaagctctctttgtgcatttccgg
tgtggatgtcgcaggagacaaccctttgagccatacgaggagaggagacaatacaggaaacccgtaataggcg
ttatagggttaagattgctttacacattatatacttgcatagctagggttaggcagggttactctcctttatc
tcctcagcaattggttcttgacactcagcatctggttacacaacagctgcctgaccctccttctcaagcttag
atgggcttggggagctcaaagccgcagcacaagaagcagttaaccatctggcgagctttgcacgcaacgcggc
acaccagatatggcttgcttgcagatccgcttatcgggcaatcatcaactctccaagaagagtgcgacaaggg
cttgaggaagtccttaattaggaagagaaatggcaacatgactccagaaggaagacgtctacaggacggggac
caatgggatgaatggtcagatgaagaagatgaagtgggatttccagtaagaccaagagtgccactaagacaaa
taacatacaaacttgcagtagatttttcgcacttttaaaagaaaagggaggactggatgggatttattactc
cgataggagaaataagatccttaatctgtatgccctcaatgaatggggaatcattgatgattggaacgcatgg
tcaaaaggacctgggataagatacccgaggtgctttggcttctgcttcaagctagtaccggttgccctgcatg
aggaagcagaaacatgtgaaaggcattgcttggtacacccagcacaactgcatgaagaccctgatggtataaa
tcatggagaaatattggca
>#22 AF301156 963 nt
aagcagtttccagaatgccaatattattatttaggaaaaacagagtcacccgaatggtggacagaaggtaaaa
tagagttttacttagggtcagctattaagttaagcataatatacttgggcactttaaccccagatagatatca
tagaggaccaccagtctcttatgaattacagataggtaagtgggaaacagatttaagctggcaacaagcagta
gggtacatgcacatcataaggggggaagtgtgtacgcatagaggaagaagcaagaaaagctataaggggcttac
cctggaatccttgtgatttccaggtaggacacctaggagtgttgaagttgcaagaaaacataacccggaggc
cagaaggcacttccctttgactcacatctcagtgataatgtggggttgtactagaaggcactgggggagaccg
tggacggctggtctggcttatgtgagaatcatggagtgtgccctctacttgcacgttcatgcgggggcgtgtt
gcccttgcccgctttgctatagacatccagatgtatattaccacaacccagaaccagaggacgtagaagatgt
agaagcaggatgttatgtgatgtctcgactgccagtacgacaggcgaccattaagctactggtggacatctct
tgctttttgaaagaaaagggtggactggaggggattattagaacccctgagagagatgacctgatagagcagt
atgcctacattgagtggggctgcttaaaaggttggttggagtatgaagatgagctgggagaggatggagcatt
aaaagaggacagaaagcctctggttgctggatggctttggaaattagtctacatagagcaacttggagagtat
gcatacagctatgacttgagtctgttgagcgttacaagcaggaagaagaagaaaccacaacaggttgccatag
agatggtggactga
>#23 AY159322 1293 nt
caggacgtcaagttgagagattggaattcaatagtaaaatatcataagtataaaggagagaaacatttagaaa
attggcaattataccccccattttcaatgctcaggatggtggacacactctcaaaagaagatcccctttaagga
tggttcaaagattatcatcaccgtcttgtggaacttgaccccagaaaaagggtggctgtctcaatacgccttt
acagtagaatatcaaaaggaaaattatttcacttacatagacccagtgacggcagatagaataatccacgggg

aatatttcccatgttttacagatcaagcaataagaaaagccttgtttggagaaagactggtagcttgttactt
cccctggggacatagaggtcaggtagggactctacagtttttggccttacaagcctatctcagaggtagaaag
atggaacagccacctgaggatgaggctccccagagaggaccatataataaatggctggtaggtactttggcag
aaatacaagaggaagctttaaagcatttcgataggcgcttgctacatgcagtaggctcatgggtgtatgagca
acaaggagacactttagaaggtgtccaacagctaataagcattttgcaaagagcactgttcttacactttaga
aagtgcttctgtaaagtttgttgctatcattgtcagctgtgcttcttgcagaaagctttaggggtacattatc
gggatctaaggcaccagcaggatgtaactctcccctcagaaaaagagcaaccaagtgatgaagaggaagaggt
aggctttccagtctacccaacgcagccagtgcctgaagcaacctacaaggacttgatagacatgtcccacttt
ttaaaagaaaagggggactggaagggatttggtggtctaaaagaagagaagagatcttggacctttatgcac
aaaacgagtggggactaatacctaattggcagaactacacaaagggaccggggataaggtatccgaagacttt
cgggttcttgttcaagctggtcccggtggcagtgtcgccccccctggaggaggatgaatgcaacaggctgcta
aactcttctcaaatgggaatccaggaagacccagagggagagaggttgatgtggaagtttgattcagggcttg
cttatacgttctatgctcctataattaggccagaggaatacaagtgtgtgacaagtctgagctatgaagccta
caagaaggaggaaaagcctgactgctgcaagaggaagtggtggcagttctag
>#24 AF367411 1023 nt
agacatgcagtagaaagatggaattcactggtaaaataccacaaatataaaggagaaaaacatttggacaaat
gggatttaaaggcacattttcaatgctcaggatggtggacacactcacaaaaatatattcccctagaagaaga
tgaacagatcataatcaccatcctgtggaacttaaccccagaaaaaggatggttatcaacttatgccatgaca
atagaatataagttcacaaactactacactcacatagaccccccaaacagcagacaggatgatacattggaaat
atctcccctgttttacagaacaagcaataagacaagccctgctaggaaagagactaacagtgtgctacttcca
ctggggacataaaggaaggtagggagcctgcagtacttggctttgctaagttatacagcctactgtaacatg
gagcaaccacccgaggacgaggcccctcaaagagaaccatacaatgaatggctaattgacaccttagcagaag
ttcaagaagaagctttaaagcattttgatagacgcttattgcatgcagtaggaagctggatctacaaaaccta
tggggatacattagaaggggtgcgacagcttatctcaattcttcaaaaagctctgtctatgcactatagagaa
gaggaagaaacgggctttccagtatatccacagtgcccagtaagagaacccacctacaaggacttggttgata
tgccccactttttaaaagaaaagggggactggaagggatttggcattctaaaagaagagaagagatcttgga
cttatatgctcaaaatgaatggggattcattcctacctggcaatcttatactgatggcccagggataagatac
ccgaagacatttaggttcttattcaagctttgcccggtggcagtgcctccagaccaggagaacaatgagtgta
acaaattgctccagtcctctcagctgggaatccaggaagaatccttggggagagaggctagtgtggaagtttg
a
>#25 AF328295 1230 nt
aaatggaattcattagtcaaataccacaaatataaggaagaaaaacacttagataagtgggaactattccacc
actttcaatgctcaggatggtggacgcactctcaaaagattatccccttcaaagatggctcaaagattattat
tactgccctatggaacttgaccccagaaaaaggatggttatctcaatatgcaatcacagcagaatacaacaaa
ggggactattatactcacatagacccagtaacagcagacagaatgattcattgggaatatttcccatgtttta
cagctgccgctgtgagaaaggtgctgtacggagaaagaatagtagcgtgttacagcccctggggacataaagg
tcaggtagggactctacaactcttggctttaagagcctacatcaagttttgcagaatggaacagccgccagag
gatgaggctccacaaagagaaccatataatgagtggctgatagatacattagcagaaattcaagaagaagctt
tgaagcattttgataggcgcttattgcatgcagtaggaagctggatttatgaaacatatggggacaccttgga
gggggtgcagaagcttatcaccctcttacagagagctctatttctgcattttagacatggatgcagggaaagc
cgaattggacaagaaggaggcaaatataaccccccttagatcctttccaaggccgaacaaccccttgtaaatgt
tattgcaagaaatgttgctatcactgccagtgttgcttcttgcagaaagctttagggatacattatcatgtct
acagaaccaggagaactcggaagagacttctgggaaaaatatcacagtagatgatgagcaaaaagaagaaagg
ggctttccagtttatcccacacagcctgtgcgtgaagcaacctacaaaaacctgatagatatgtcccactttt
taaaagaaaagggggactggaagggatttggttctctagaagaagagaagagatcttaaacctgtatgctca
gaatgaatggggattcataccagattggcaagaatacacttcagggccagggataagatacccgaagagattt
gggttcttattcaagttaatcccagtacaagtaccaccagaccaggagaaccaggagtgtaacaggctgctta
actcctctcagctaggaatccaggaagaatccatgggggagaggctgatgtggaagtttga
>#26 KM378564 1305 nt
aggtacatggtagagagatggaactccctgtgtaaatatcacaagtataagggagaaaagtatttagaaagat
ggcattatgcacctcacttccaatgctcaggttggtggactcatagtcagtggaccatccccttccaagacaa
aagtaaaatagtagtaacagtcctgtggaacctcaccccagacaaagggtggttaagtaagtatgccataaca
atagaacatattcaggagaagttttgcaccttcatagacccaacaacagcagataaaataatccacttggaat
atttcccttgttttacagatcaagcaattagaggtgtcctgctgggatttagaattcaagcttgctatttccc
tagagggcataagggtcaggtagggagtcttcagtatcttgccttgcaagcacacataaaatatcttaagatg
gaaaggccacctgaggatgaaggaccgccaagggaaccctatgatgagtggctagtagatactttagtagaaa
tacaagaggaagctttgaagcattttgataggcgcttactgcataatgtaggatcatgggtctatgaaagata
cggagattcattagaaggagtacagcagcttataacaatcttgcaaagagcgcttttcttgcattttagaatg
gatgccaggaaggtagacttggaccagcaggacgctggaatccatttcgaacctttccaagggcgaaccagca
attgtagcttagatactactcttatgcaagcagcagcagcagcagagaaagatgtcacccttcccactgaaga

agaacaaccctcagaagaagaagaagaagtaggctttccagtatatcctcgatgtcctctaagagaaccaaca
tataaagatctggtagacttttcccacttttaaaagaaaaggggggactgaaggggatttggtggtctcaca
agagagagacaatcttggacttatatgctcaaaatgaatggggcttcataaaaggctggcaaagctacaccag
aggacctggtatcaggtacccaaaagtgtttgggttcctgttcaagctggtccctgtggaggtagatgaagat
gtggtaaatcagccgtgcaacagcctgttaaactccagccaaatgggtcctattgatgacccaaacggagaaa
gactcatgtggcagtttgatccaaccctggcatattccttcaaggcttgtattcaccatccagaggagtttgg
acatgctagaagtatagagcacatagcagaggagccagcctgctgcaagaggaagtggtggcag
>#27 KM378563 1302 nt
aggtacatggtagaaaggtggaactccctgtgcaaatatcataagcataagggagaaaagtatttagaaagat
ggtattatgcacctcacttccaatgttcaggttggtggactcatagtcagtggaccatccccttcaaagacaa
gagtaaaatagtagtaacagtcctgtggaacctcaccccagacaaagggtggttaagtaaatatgccataaca
atagaacatattcaggagaagttttgtaccttcatagacccaacaacagcagatagaataatccacttggaat
atttcccttgttttacagatcaagcaataagaggtgtcttactgggatatagaattcaagcttgctatttccc
tagagggcacaagggccaggtagggagtcttcagtatcttgccttgcaagcacacataaagtaccttaggatg
gaaaggccaccggaggatgaaggaccgccaagggaaccctatgatgagtggctagtagatactttagtagaaa
tacaagaggaagctttgaagcattttgataggcgcttattgcataatgtaggatcatgggtctatgaaagata
tggagattcattagaaggagtgcagcagcttataagaatcttgcaaagagcacttttcttgcattttagaaaa
tgctggtgcaaacactgcgcttaccactgtcagctttgctttcttcaaaaaggcttagggatacgttatagct
tagatactactcctatacaagcagcagcagcagcagagaaagatgtcacccttcccacagaagaagaacaacc
ctcagaagaagaagaagaagaagtaggctttccagtatatcctcaatgcccgttgagagaaccaacttacaaa
gagttagtagacttctcccacttttaaaagaaaaggggggactgaaaggatttggtggtctctcagaagag
aatcaatcttggatctttatgctcaaaatgaatggggcttcataaaaggctggcaaagctacaccaaggacc
tggcatcaggtacccaaaggtatttgggttcctgttcaagttggtccctgtggatgtagatgatgacctgata
gatcagccgtgtaacaggctgttgaactccagccagatgggtcctattgatgactcagaaggagaaagactca
tgtggcagtttgatgcaagcctggcatacacctttcaggcttgtattcaccatccagaggagttcggacatgt
tacaagtataagacatgataaaagagaagagccagattgttgcaagaggaagtggtggcag
>#28 M27470 1362 nt
cagagaatagaaaagtggcactggttagtaagaagacagatggcatgggccactgcaaataatgaggaaggat
gttggtggctgtatcctcattttatggcttataatgaatggtatacttgcagtaaagtagtgattataataaa
tagggacataagattaatagttagaagctattggcatttgcaaatagaggtaggatgcttaagtacttatgca
gtaagcatagaagcagtagttagaccgccaccctttgagaaagagtggtgtacagagataactccagaggtag
cagatcatctaatacatttacattttatgactgcttcatggacagtgcagttatgaaagccatcaggggaga
agaagtgttaaaagtttgtagatttccagctggccataaagcacaaggtgttctctctttgcagtttctctgc
ttgagagtcatccaagtatcagaagatcaaggacctcccagagagccatacaatcagtggctagcagatacta
tggaggaaataaaggaagaagcaagaaagcacttccctctcattatcctaaatgcagtatcagaatattgtgt
gcaaaacacagggagtgaggaagaggcctgtgagaaatttattaccttaatgaatagagccatttgggtccac
ctagctcaagggtgtcaactgctagaagagtattatcagcctttgcaagcttgtgagaatatgggttcctcgc
agtccaagaagcgatcagaagcttgggttcgctactcgtcagctttgcggcaattagttggagggccggttac
accggatggctacaagcaaatagaatcttcacagggtgcagagaagcaatcattgctgcggggacgtgcatat
ggcacatactcagaaggattagacaaagtgcagaacgaccccttaactaaagatgagaaacttgacttaacac
agcaggatccagaagaggaggaagaagttggatttcctgtgtgtcgccaagtttccttaagagtgccatcata
caaagatctgatagacttctctcattttataaaagaaaaggggggactgggagggatatattatagcaggaga
agagaagaaatcctagatctctatgcagagaatgagtggggatttgaacctggatggcaacagtatacgacag
gtccaggaaccagatatcctaagacatttggattcctgtttaagctggaaccagtgagcagagctataggaga
tgagtatgcagctaacaatcatctgttacactcctcccagttatgtcctcaggaagatccagaaggagagacc
ctcatgtggtctgggaccctcatcttgcctatgactttgcagcattaa
>#29 AF131870 1023 nt
agagaatggaagatagaagacagtataagatagttagaatagtatggcttatagatagaattgctgtagaga
aatttttagatttaagaagaatgcatagggaaacaaaagatgattgggtatctatgtatggaacaggtactgg
atgggagtggtacacatacaataaaattattattccagtaacttatgggacagtagtagtaaggatctatgga
cacctcacaccagcaaaagggttgattaaccagtggggatgtagtatggaatggatttataacagttatcaaa
cagaaatagatcctcttgtggcagatcagatgatccactttaagtattttgattgttggacctctaggtgtat
cagaagagctatgttaggggaaaaaatactacatgagtgcagaaatcaagtagctcacaaaggcctggttctc
tctttgcagtttctctgcttgagagttctcgccttgttcattcatctgccaatgtgtccttcttacatcagaa
tcagaagtggatcaggattggaggcctgtgataacaaatgctggtgtaggagatgctgctttcactgtcaact
ttgctttctgcagaaagggttagggatccactatccaacatataagcagctaatagatctctcacattttata
aaagaaaagggtggactggaagggctctggtactccagaaccagagaagaaatcttggatttgtatgcagaaa
atgagtggggcttcataactggctggcaagattacaccaaagggcctggagtgagatatccaaaggcctttgg
ctggctatggaagctagcaccagtcaccattgatgaagacagggatcctaatcatccttgtcaagccctactg
cattccagccagcaaggggttaatgaagacccttggggagagagacttatctggacatttgatcccactctgg

```
cctatgattttagggccatacaaaagcaccctgaagagttcaagcatgttactagtctgcagtgggaggtgta
g
>#30  AF075269  876 nt
tccttaatagaaaagtttttagacatagtcaggttaaacacagaggcaaaagaaaattgggtaggcttacatg
gaacaggtgtaggttgggaattctatacctatcataaattagtgatacctctagagtcaggaactttagtagt
cagaatatatgcacatttggcagcaggaaggggatggatctcacaatgggcagtttccatagaatgggtctat
agagactatcaaacagaaatagaccccatactagcagaccaaatgatccactataaatattttaactgcttca
tacaaagagacattagaagggcacttctgggagaaagaataactatgtgttgccagcctttaggacataaagg
ttgtgttctctctttgcagtttatctgcttgagacaactacaagctttgtttcttcacctcccaacttgtccc
agcgccctgcaagcgtgtcagaataccccctacctataagactcttatagacctctcacattttataaaagaaa
agggtggactggaagggctatattggtcccagagaagacaggacatcttaattctctactgtgagaatgagtg
gggtctcattggagacttcatgaactacacagatggaccaggaacaagataccctctgacatttggatggttg
tggcagttagaaccagtggcttgtgatgagtataaagatccctcagatgagacacaatgccttttgcattcaa
gccagctgggggtcctggaagacccctggggagagaggcttatctggcacttcaatccaatgttggcagtgga
ctttatagccctcaaaaagcagcctgcgaagatacaaaatactgctttcgcttttgactgcaagaggaagtag
>#31  AF188116  951 nt
agcttagtagagaaatttctagatatcaaaaggaatcattcagaaagtaaaaatgattgggttagtttacatg
gtacaggagtaggatgggagttttatacttacaataaattactaattccattagatacaggaactttagtagt
aagaatttatgctcatctggcagcagggagaggatggatttcacagtgggcagtatcaatagaatggatctat
ggaaactatcagacagaagtggatcccattctagctgatcaaatgcttcatgtgaagtattttgactgttggg
tgcatagggacatagttagggccataaggggagaaaagattctgcacagctgtaaaatgcctactggacataa
aggttgtgttctctcattgcagtacctctgcttgagacaactacaaagagctctctatttacatctgcctcaa
tgtcccagtccactccaatcctgccagaataagtgccactgcaaagtttgttgctttcactgtatactttgct
tccagcagaaggcgttagggatacggtattctacctataaaaccttgatagacctctcacattttataaaaga
aaagggtggactggaagggctatattggtcccagagaaggaatgacatcttaacactctactgtgagaatgaa
tggggtctcattggagacttcatgaactacacagatggaccaggaactcgttacccactcgcctttggatggc
tgtggcagttggagccagtagcatgtgatgagtttaaagatccctcagatgagagacaatgtttgctacattc
aagccagttggggggtcctagaagacccctggggagaaaggctcatctggcactttaatcccatgttagcagtg
gactatgtagccctgagaaagcaaccagcaagtgttcaagcaactgctatgagatttaactgcgagaggaagt
ag
>#32  AF188115  948 nt
aacctgatagagaaattcctagatattaggagagcacataaagaaactcaggttgattgggtaagcttacatg
gaacaggggtaggctgggaattttatacttataataaaatagtaatacctttagagataggaaccttagtagt
aagatgctatgcacacttggcagcaggaagaggatggatctctcagtgggcagtatcaatagaatggatttat
gggaactatcaaacagaaatagatcccattctagcagatcaaatgatacactgtaaatattttaactgctttt
ctacaagggatattaggaaggctttatggggagaaaagataatggcaatctgcgctatgcccactggacataa
aggttgtgttctctcattgcagtttctctgcttgagacaactacaagcactctttattcatttgcctacctgt
cccagcccactgagtgtctgtgacaacaagtgctgctgcaaaaggtgttgctttcattgtattctttgctttc
agcagaagggattagggatacgatatcctacttataagactctgatagacctctcacattttataaaagaaaa
gggtggactggaagggctatattggtcccagagaagacaggacatcttgtgtctttactgtgagaatgaatgg
ggtctcattggagacttcatgacctacacagatggaccagggacacgttacccattaacatttggatggttgt
ggcagctggaaccagtggcctgtgatgagtacacagatccctcagatgccagccaatgcctgctgcactcaag
ccagctgggggtccaggaggacccctggggagagaggcttatctggcacttcagtcctatgctagcagtggac
tttgtagccctcagaaaacaacctaagagcatacaagcatcatctcttagcttgaactgtaagaggaagtag
>#33  AY523867  1101 nt
aaaatgcagcatatattaaaggtacacaagtataaaagcaaacaattagagaaagccacatataaacatcatt
atcagattacatgggagtggtggactcgagcacaatgggaaataccagtaggacaaggtgtattaattataaa
attctatcataatttgacaccagagaaaggcctgttaagaacagagggagtaggattaatttgggcccatccg
tctggatggagtacagaagtaacaccacaaacagcagatgcattagtacatagtcaatacttcccttgttttt
cagatagggcagtccaacaagcacttagaggggagaagttgacttcgcactgctggaatttccataaagaaca
ggtactttcattgcaatatttggctttacaaaagtatttaagcaagtgcatgctagatatgcatagagcactc
aactttatgctttttgaacattttgctgcattagaaggaacatttctctcaaatgggcctcagactccttgta
gcagatgctattgcaaaaagtgttgctatcattgttatagatgctttcttcaaaaaggcttagggataacacc
catcttggatcccaaaagagactttgactctgggggaaaaaatggaatgcgggagacatctgtcatgatgag
ggagatgaggatctagtaggctttcctgttttaccacaagtacccttgagacccttaacttacaaacttgcca
tagatctctcccattttataaaaagaaagggaggactgcagggcatgtttttctgtcaaaagagacatgagat
tctacagctgtatttaaagaatgagcatggtgtgatagatgacatcacctatacgagtgggccaggaacaaga
tacccgctgatcttcgggtggttatgggaattggcccctaatgaaatagaagggtacttggtagatgaggaag
acacactgatgatgcacccagcagcaggagtgggggcttcagaagacccacatagggagaatctcatgtggaa
```

cttcaatccacacttagcctatacaccaggctgggaaatggctcgtcagcaattagagagacaaacaggaaag
cgctag
>#34 AY655744 1143 nt
tttcacatacataaaaccaagtacttacaaaagttgatttgggttcatcactttcaattgagacacgcctcct
atacacaagcaaagtggatactcccgttagaagaaaaggacaccagagtagaatatttaaccataacacagta
ttggtgtttagcaccagaccatggatatacgtccgattgggacacaatatccattgagtggtcagtctcggat
atagtccgagacgaaaaggtgtacgccactgaagtaaccccaaatgtggcagatcacttgattcataccaaat
atttcccatgttttacagcgtcagcagtcagacaagccatcagaggggaggtgctactagcagtttgtggagt
cagacacaaaggtagggacacgccaccgtctctacagttcctagccttgacagcagcagttagatggacagga
gaacaggcatgggctgcctcagtcatagattttgctaggaagggtaatcaaatgttactcctacatttccagc
aagggtgctattgctattgcaagaaatgttgttaccactgccagctttgcttcttgcagaagggtttaggtat
ccgggaagaatggcaagcaacaggagcggactacatgggcaaggcattaacacaaggagaggtttggtatgaa
aaagaacaagaagaaaatgacatggaactagtgggcttccctgtgaggcctaaagttccgttgagaacctgta
cttataaactaggtatagacttctcgttctttttgaaagaaaagggaggactgaaggggatattttacaacaa
acgtaggcatgcaatcttgaatctgtatgcccacaatgaatggggcatactgccagactggcagaactacaca
gagggaccaggaacaagatacccattgtgctttggtatcctgtggaaactctgcccagtagagatccatgagg
atgacacggaggacggacatctcctcccgcatccagcatatgatgggcaggcagaagacccatggggagaatc
cctggtctgggtctttgatgagaagctggcctacacccccggagcgaagatggccgagtgggacaggttggag
agagagaagcggatgctgctagctccaccgcaaaccgcatcctcttga
>#35 AY523865 1197 nt
ttagataggtggtgcagtgctataaaaagagaaaaatacaagaccaaagaattagatactgtacaatggatac
accattttgaattaaggaactcatattatacacaaacaaaaatagtattccccatcacaaaagaaggggagt
agtagtagaattactatggtgcttgaccccagagaaaggatggctacccactatggcagtagcaatagcatgg
gaaacaaaaacatggaagacagaactaacaccagatttagcagatcaccttatacacctaagatactttccat
gtttctcccaacaggcagtgagacaagccataaggggagagatcctactgcaacagtgtcagcagtaccatag
gggaccagaaggtaaaggaccaccttcactacagtacatagcacttagagtagcagtaaaatacataaaaata
gttcaaaaaatgttatggttgcacctgagagaagcttgctttcacagggagagagaggctaccagagggtatc
ctaacattaggccattgacaggaagaaatagagaagttagggacggagaataaatggagagcatagatccatt
tgaagaaagagctaatacaccccaccccgcttgtatgtgcaaagcctgcattctacattgccagctgtgtttt
atacaaaagggtctagggatttccttctggggaagtcctagaagagaaataaagtgcaccaataagcaacaac
aagacttagatcagacagaagcagtaggcttcccagtacgcccccaggtatccatccgagaccccacatataa
gttaatgatagactactctcatttttttgaaagaaaagggtggactggaggatgtttttttactctgccagaagg
catgctattttagagcttcatgcccagaatgaatggggaattataccaggctggctacagtacacaaagggac
ctggagttaggtatccaatttacttcgggttcctctttaaactggtgccagtagaaatagcagacccagatta
tgagaatgatgagaggaacatcttgttacatgatgcccaccaggggatgatggaagacccccatagagagagg
ttggtttggaagtttgacagcactctggcctattgttataaggcaggccatgctgaacaaagagagcatacta
gaagatgtatgttccctaagaggaagtag
>#36 AY523866 1206 nt
ttagataagtggtgtagtgccataaaaagaggaaagtataagaccaaagaattacaagcaataaagtggatac
accattatgaattaagaagggcattctacactcagacaaaaatagtcttccccgtcacagaagaaggaaaagt
agtagtggaaatattctggagcctggctccagaaaaagggtggatgccctctatggcggtaggaatctcttgg
gagcagacagggtggcagacagaattaacacctgacctagctgatcatttgatacacttaagatacttccctt
gtttctcccaacaggcagtcacacaagccctaaagggagagaaactactacaacagtgtcagcaataccatag
gggaccagaaggtaaaggaccaccctcattacagtacttagcactaaggacagcagtaaaatatataaaaata
gtacaaagaatgctttggcttcatctgagagaagcatgcttccatagagagagggaagctactaggagatacc
ccaatattagaccccctaacaggaagaaacagggaggtcagggatggagaataaatggaagaaatagatccatt
caaggaaacagctaatacaccccatcctaattgtatgtgcaaagcctgtactatgcattgccagctgtgcttt
atgcaaaaaggtctagggatctacgaaccactctatgcagacccccacagagagataaaatgcactcagagag
cccaggagtgttatgatgaggaacatgggataggctttgctgtcagaccaagggtcccaattagggaacccac
ttacaaattgatgatagactactctcattatttaaaagaaaagggtggactggaggatatttttttactctgct
agaagacatgctatattagaaatccatgcccaccatgaatggggaataataccaggttggttgaagtacacag
aaggcccaggaccaaggtatccaacatactttgggttcctatttaagctagtgccagtagagatagcagatcc
tgactatgagaatgatgagagaaatatcctcttacatgatgcccaccaaggacaagcagaagacccccataag
gagaggctagtatggaagtttgattcctcccttgcctattgctataaagcaggacatgaagcccataaaccag
agcataacagaagatgcatgttccctaagaggaagtag
>#37 FJ919724 1158 nt
ttagatagatggtgtagtgctataaaaaagggaaaatataaaaccaaagaattggctcctataacatggcaac
atcactttgaactcagaaatagttattttactcagacaagaataatatttcctatcacaaaagaaggaaaagt
agtagtagaactcttctggtgcctagcgccagaaaaaggatggttgccaggaatggcagtaggtctcagttgg
gagcagggaaactggcagacagagctatctccagaattggcagaacacttaatacacttaagatatttcccct

gttttactcaacaggcggtgaggcaggccattctgggggagaagctgctcacgtgctgccagcaatatcatag
gggagcaggaggtaacggaccaccttcattacagtacatttgcttaagggtagccataaaaagggcagcagct
tcaagacattaccctaacatcagaccactgtcaggaagaaatcaggaggttagggatcaggaataaatggagg
aggaaatggacctattccaagggagagggagaggagaagcaaatcaccctagttgctattgcaaaatctgtac
ttttcattgctacttgtgcttcacgcaaaaaggcttagggatcacggaaacctttataggaaatccaagaaga
gaaatcaagtgtacacagagacaacaacaagatttgaccaacaagaggaagtgggatttcctgtcaaaccca
gggtcccaattagagaccccacctataaattaatgatagactactctcatttttaaaagaaaagggtggact
ggaggatatttttactcagccaggaggcatgcaatcttggaactgcatgcccaaaatgaatgggaataata
cctggttggctccagtacactgagggcccagggattagatatcctaagtactttgggttcctctttaagctag
tgccagtagagatagcagacccagactatgagaatgatgaaaggaacatcttgttacatgatgcccaccaggg
tcagatggaagacccttataaggagaggctggtatggaagtttgattcacaactagcctactgctataaagca
ggccatgaagctcatactaaagagacacatactaggagatgtatgttccctaagaggaagtag
>#38 AF208027 1230 nt
ctgataaaattcttaaagtttaaaacaaaagacctacagaaggcggtttatgttcctcatcacaaagttggat
gggcttggtggacttgcagcagggtaatattcccattaacaaaagaagcacatttggaaatacaagggtattg
gaacttgaccccctgaaaagggatggttaagccagtatgcagtaagattaacctggtacacaagaaaattttat
acagatgtgacaccagagacagcagatcaattattgcatggatcttattttgattgctttacagctggtgaag
taagaagagccatcaggggagagcagatattgtcctgctgcaactatccaacagctcataaaaggcaggtgcc
tagcttgcaatttctagccttacaagtagtgcaaaaaggaatggcagaagaaatcccccccagaagatgaggcc
ccccagagagagccatgggatgaatgggtagtagaagttctggaagaaatcaaagaagaagccttaaaacatt
ttgaccctcgcttgctaactgcgcttggtaactatatctatgataggaaatgttattgcaaaaaatgctgcta
tcattgtcagttatgctttcttaacaagggactcggagtatgtgggcaacaatacacgcagggtcaatttatg
aataccccatggagaaacccagcaacagaaagagcaaagttagcatacaggyagcaaaataatgatgatgtag
atagtgatgataatgacttagtaggagtaccagtctaccccaaagtcccgctaagagtaatgagttacaaatt
ggcaatagacatgtcacattttataaaagaaaagggggactggaagggatttattacagtgaaagaagacat
agaatcctagacatatacatggaaaaggagcaaggtataattccagattggcagaattacacagcaggaccag
gtataagatatccartgacgtttggctggctatggaaactagtaccagtcacggtctctgatgaggcacagga
ggacgagactcattgcttggtacatccagcacaaactagcccatgggatgacccggagaccgaagtgttggcg
tggaagtttgaccctacattagcctatgactacagggcatttatattgcatcctgaggagttcaggtggaagt
cagggctaccagaagcagtatggaaagagaaactaaaacagagaggactgcctatagaataa
>#39 M30502 1029 nt
acttatttctcttgctttacggcaggtgaagtaagaagagccatcagaggggaaaagttattgtcctgctgca
actatccccaagctcataaagcacaggtaccatcacttcaatacctagccctagtagtagtacaacaaactga
agcaccaacagagtttcccccagaagatgggaccccacggagggacttagggagtgactgggtaatagaaact
ctgagggaaataaaggaagaagccttaagacattttgatccccgcttgctaattgctcttggctactatatcc
ataatagacaaggagaagaactccttgcccagctgcaccgaccccctagagccatgcactaacaaatgctattg
taagcgatgcagtttccattgccagctgtgtttctcgaaaaaggggctcggaatatcaggacagcagtatcag
cagggagagtacatgaacagcccatggagaaacccagcaacagaaagacagaaagatttgtataggcagcaaa
atatggatgatgtagattctgatgatgatgacctaataggagttcctgttacaccaagagtaccacggagaga
aatgacctataaattggcaatagatatgtcacattttataaaagaaaaaggggactgcaaggatgttttac
agtaggaggagacatagaatcctagacatatacctagaaaaagaggaagggataataccagattggcagaatt
atactcatgggccaggagtaaggtacccaatgtacttcgggtggctgtggaagctagtatcagtagaactctc
acaagaggcagaggaagatgaggccaactgcttagtacacccagcacaaacaagcagacatgatgatgagcat
ggggagacattagtgtggcagtttgactccatgctggcctataactacaaggccttcactctgtacccagaag
agtttgggcacaagtcaggattgccagagaaagaatggaaggcaaaactgaaagcaagagggataccatatag
tgaataa
>#40 AF082339 1182 nt
cttgtcaagtacctaaagtacagaacaaaagatctagaaaaggtgtgctatgttccccaccataaggtgggat
gggcatggtggacttgcagcagggtaatattcccattacaaggaagaagtcatctagagatacaggcatattg
gaacctaacaccagaaaaaggatggctctcctcttatgcagtaagaataacttggtatacagaaaagttctgg
acagatgttaccccagactgtgcagactccctaatacatggcacttatttctcttgctttacggcaggtgaag
taagaagagccatcagaggggaaaagttattgtcctgctgcaattatccccaggcccataagtcacaggtacc
gtcactccaatttctggccttagtggtagtgcaacaaactgaagcaccaacagagtttcccccggccggaatg
ggaccccaccagggagccagggatgagtgggtaatagaagtcctgagagaaataaaagaagaagctttaaggc
attttgaccctcgcatgctaattgctcttggcggctatatctatactagaggacagcggtatcagcagggaga
ttttatgaataccccatggagaaccccagcaacagaaagagagaaagaattgtacaagcaacagaatatggat
gatgtagatttagatgatgatgatagcctagtaggggtctctgtcacaccaagagtacaattaagaacaatga
catacaaattggcagtagatatgtcacatttaataaaagaaaggggggggactggaaggatgttttacagtga
gagaagacatagaatcttagacatatacttagaaaggaagaagggataattccagattggcagaactatact
catgggccaggaataaggtacccgatgttctttgggtggctgtggaagctagtaccagtagatgtcccacaag

aaggggaggacactgagactcactgcctgctacacccagtacaaacaagcaggcatgatgacacgcatgggga
gacattagtttggagatttgaccctaagctggctcatgattacaaagcctttattctacacccagaggaattt
gggtacaagtcaggcctgccagaagatgagtggaaggcaagactgaaagcaagagggataccatttagtaaga
acaggaacagctga
>#41 U27200 1188 nt
tacctgaaatataggacaaaagacttgcaacaggtctcttatgttcctcaccataaggtaggatgggcttggt
ggacttgcagtagagtaatatttcccctgaaagaaggagcacatctagaagtccaaggatactggaacctgac
cccagaaaggggattcttgagttcctatgctgtaagactaacatggtatgagaggagcttttatacagatgta
actcctgatgtagcagaccgattactgcatgggtcttatttctcttcgtttacagctaatgaagtaaggagag
ccatcaggggagaaaagatattgtcccactgcaactacccatcagctcatacaggccaggtaccaagtttaca
gtttctagccctaagagttgtacaagaaggaaaagcagaagcagtcccagagattcctccagaggataaaaac
ccacaaagagaaccgtgggaacagtgggtagtggacgtcctggaggaaataaaacaagaagccttaaagcatt
ttgacccccgcttattaactgcacttggcaattttatctacaatagggcatgcagtaatacatgctactgtaa
aaaatgttcctaccattgccagctttgctttcttaaaaagggcctggggatatgtggacaaaagactttggga
gcagaaggggggaggaaaacaagattcagatgaggatgatgaggacaatgaagtaggggtccgtgtaagacccg
gggtcccactgaggccaatgacattcaaactagcagtagacatgtctcatttttttaaaagaaaaggggggaact
ggaagggatttctatagtgagagaaggcataaaatactagacacatacttagaaaatgaagaaggcattgtg
tctggatggcagaactacacacatgggccaggagtaagatatcccaagttctttggctggctatggaagctag
tgccaataaatatgatagcagaaccagaggacgaggaaacccattgtctggtgcatccagcacagacctccgc
atgggatgacccccacgaagagacccttgtctggcagtttgactccctcctagcatatgactatgtggccttc
agcaggttcccagaggagtttgggtatcagtcaggaatgccagagaaagagtggaaggctaaactgagagcaa
gaggaatacctacagagtag
>#42 KP004991 1380 nt
tataggtccaaaaaggccaaagattggttttacagacaccattatgaagccacaaatccaagagtcagttcag
gtgtatatattccagtaggtggtgcttgcataatagtgaccacatattggggtttgatgccargggaagmagc
tgarcaattgggaaatggggttagtatagaatggcagctaaggaattatagaacacagattgatcctgaaaca
gcagacaggatgatacacctgtattatytttcmtgttttacagagtcagcaatcaggaaagccatcttagggc
aaaaggtgttacccacttgtgaataccctgcaggacatagtcaggtagggacccttcaattcctagctttgag
ggtattagtaaaaacaagaaaatttagacctcccttacccagtgtccagaagttagcagaagatgcagaagca
ttaaaacatttcctagaccttggctacacgccttgggacaacacatttatgaaacttatggagacacttggg
agggagtaacagccattataaggatcttacaacaattaatatttacccattatagaattggctgccatcatag
tagaataggaattatcccatctaacgcaagaggaagaagaagaagagtgccccccttggcatcatcctgggagt
caaccccaaaccccttgtaataactgctyttgcaaaaawtgcgcctatcattgctatctttgtttcacaaaga
agggtttggggatctccatggggaatgcctggaagaaaagcagctttgtgggatggccagcagtaagggatag
aatgaggcaaacttcccctgaccctgagccatgtgcccctggggtaggaatagtttctagagaattagcacag
aaaggaggcataccaagctcatacacacctcagaacaatgcagcccttgcgttcctagacagtcatacagagg
aagaagtaggtttcccagttacacctcaagtgcctctaagaccaatgacctttaaaggagcgtttgacctcag
cttctttttaaaagaaaagggaggactggatgggctaatttactccccacagagagcagagattctggatctt
tgggtcyatcacactcagggattcttccctgattggcagaactacacaccagggccaggaaytagatacccac
tgacctatgggtggttgtttaagttagtaccagtatcagaagctgaggcagaagaaatgggcaataagaatga
gagggctaaactcctacacccagcttgyaatcatggctatggggatgagcatggacaaatactaaagtggcag
tttgatagatcactaggcaacaaccatgttgctctggcccttcacccagagtactttaagaactaa
>#43 FJ424866 1401 nt
taccataaatatagaactaaacaagcaaaacagtggttctataggcatcactatgaagcagtcaatcctaggg
taagttcaggggtatacataccagtaggccccgctgaaattatagtaaccacatattggggactcatgccagg
agaaaaggaagaacaattaggccatggggtaggcatagaatggagacagggaaaatacataacacaattagat
ccagaaacagcagataggataatccatctttattactttcaatgttttacagaatctgcagtgaggaaagcaa
ttgtggggggaaaaactcctgtacaagtgtaactacccggcaggacatagtcaggtagggacattgcaatactt
agcattaagagctttagtagggaaagtcagggtaagaccaccttttccaagtgttcagaaattagcagcagat
atgttagaagaacttaagcaagaagcagtaagacactttcctaggccctggttgcaccaattgggacagcatg
tttatgatacttatggagacacttgggaaggagtgacagcaataatcagaatcttacagcaagtaatctttgt
tcattatagaattgggtgtcatcatagcagaatagggattttgccaccctctagaagaggaagaagactgcca
ccatggcagcaaccagggagccagcccccaaccccatgcaacacatgctattgtaaaaagtgctgttatcact
gctatgtttgtttcacaaagaagggtttgggaatctccatgggaaacacatggaaaaagagtagcttcatagg
atggccagcagtaagggaaagaatgagacaaaccaaccctgagccaaagccggctccaggggtgggtgaagtg
tctagagcattagcacaaagaggagggatacctgcaaaatatactccacaaaacaatcaagctttagcattcc
tagaagctcatgaagatgaagaggtaggatttccagtcagacctcaagtgccgttaagaccaatgacctttaa
aggggcatttgacctcagcttctttttaaatgaaaaggggggactggaagggttagttttctctcaggccaga
agtgatatcttagacctctgggtttatcatactcagggatacttccctgactggcagaattacacaccagggc
cagggataagatatcccctgacctttggatggctgtttaagttagtgccagtctcagaggcagaagcggagga

gatgggaacatcaaatgagaaggccatgctgctgcatccagcgtgcagccatgggagagacgacccatatgga
gagatccttgtctggagattcgatagagcattgggaacaactcatgttgccttacagaagcacccagaactgt
tcaagagagactaa
>#44 KP004990 1392 nt
tacaggtctaaaagagcaaagaattggttttatagacatcattatgaggcagttaatcctagagttagttcag
gggtctatattccagtagggacagcaatgattattgtaaccacctactgggggctcatgcctggtgaaaaaga
agatcagttggggcatggagcaagtgtagtatggagtcaaggcaaatacagcacacaaatagatccagagaca
gcagatagactaattcacctccactacttccaatgtttttcagattcggctgtgaggaaggcaatactagggg
atagattactgaccaggtgtgaatattcagcaggacatagtcaggtaggctccctacagtacttagccttaag
agtgttagtaggaaaagtaagaaggaagccacccctccctagtgttcagttattgacagaggacaccttggag
gaactcaaagcagaggctgtaagacactttcctaggccttggttacactccctaggacaacatatatatgata
cctatggggacacctgggaaggagtaactgcaattattagaatcctacaacaattaatttttatccactatag
aattggatgccctcatagcagaatagggatagcgccacaccctcaaagggggagaagattgcctccatggcaa
cagccagggagtcagccctcaagcccgtgtaacaattgtttctgtaaagcttgctgctatcactgctatgttt
gtctcgtaaagaagggtttgggaatctccatggggaatgcatggaagaaaagtagtttagtagggtggccagc
agtcagagacagaatacaccagactaccccagagatagacccagcacctggggtggggaaaatctccagagaa
ttggcaaaagagaaagcaatacctagcaagtacaattcaagaaataatgaggcattagctttcttagaggctc
atgaagatgaggaagtagggtttccagtgaagccccaagtacccctaaggccaatgactttcaaaggagcatt
tgatctcagcttctttttaaatgaaaagggaggactggatgggttagtttattcacctgagagagccgagatt
ctagatctctgggtctatcacactcagggcttcttccctgattggcagaattacaccccaggtccaggagtca
gatggcccctgacctttgggtggatgtttaaactagtacccgtctctgaggcagaagctgagtacatgggaaa
taaggatgagagagctaagttgttacacccagcttgcacctatgggttttcagatccacacaaggagatccta
gtctggaagtatgacagatcactaggaacacagcatgttgccctgatgaaacacccagagctgtttatcaaag
actaa
>#45 KP004989 1389 nt
tatagatctaaagaagcaaaaaattggttttataggcaccactatgaggcaactaatcctagagttagttcag
gagtttacataccagtaggaacagcaactattattgtaactacatattggggactcatgcctggggaaagaaa
agarcaattagggcatggggcaagtgtggagtggagacaaggtagatacatcacacagatagatccagaaaca
gctgataggctaattcatctccactacttccaatgtttttcagattcggctgtgaggaaggcaatactagggg
acagattattgcacaggtgtgaatactcagcaggacatagtcaggtaggatccttacagtacctagccttaag
ggtgttagtagggaaagtaaaaaggaggccacccccttcctagtgtccaggtgttgacagaagatacactagag
gaactcaaggcggaagcagtgagacacttccctaggccttggttgcaattattagggcagcacatctatgata
cctatggggacacctgggagggagtaactgcaattattaggatcctacaacaattggtctttgttcattatag
aattggatgccagcatagtaggataggtatattgccaccctcacgaagaggaagaagactgcctccatggcag
cagccagggagtcagccctcaagcccatgtaacacttgcttctgtaaagtctgctgctatcattgctatgttt
gtttcgtaaagaagggtttgggaatctccatggggaacgcatggaagaaaagtagtctggtaggatggccagc
agtcagagacaggatgcaccagactaccccagaagaagcagcacctggggttggggaagtctccagagaattg
gcaaagggaaaaggaatacccagtaaatacaatgcaaggaacaatgcagccttagctttcttagatgctcata
gagaggaagaagtaggattccccgtcagacctcaagtgcccttgagatgcatgaccttcaaagggggcatttga
cctcagcttctttttaaatgaaaagggaggactggatgggttaatttactcacctgagagagcagaaatccta
gatctctgggtctatcacactcagggattcttccctgactggcagaattacactccagggccaggggtcagat
atcctttgacctttgggtggctgtttaagctagtgccagtctctgaggcagaagctgaggaaatgggaaataa
gaacgagagagctaagctgttacacccagcctgtgcttatggattctcggatccacataaggagattttgatg
tggaagtttgacagatcactaggaatcacacatgttgccctacaaaagcacccggaactgttttctaaagact
ga
>#46 FJ424863 1401 nt
taccataaatatagaactaaacaagcaaaacagtggttctataggcatcactatgaagcagtcaatcctaggg
taagttcaggggtgtacataccagtaggccccgctgaaattatagtgaccacatattggggactcatgccagg
agaaaaggaagaacaattaggccatggggtaggcatagaatggagacagggaaaatacataacacaattagat
ccagaaacagcagataggataatccatctctattactttcaatgttttacagaatctgcagtgaggaaagcaa
ttgtgggggaaaaactcctgtgcaagtgtaactacccagcaggacatagtcaggtagggacattgcaatactt
agcactaagagctttagtagggaaagtcagggtaagaccaccttttccaagtgttcagaaattagcagcagat
gtgttagaagarcttaagcaagaagcagtaagacactttcctaggccctggttgcaccaattgggacagcatg
tttatgatacttatggagacacttgggaaggagtgacagcaataatcagaatcttacagcaagtaatctttgt
tcattatagaattgggtgccatcatagcagaatagggattttgccaccctctagaagaggaagaagactgcca
ccatggcagcagccagggagccagcccccaaccccatgcaacacatgctattgtaaaaagtgctgttatcact
gctatgtttgtttcacaaagaagggtttgggaatctccatgggaaacacatggaaaaagagtagcttcatagg
atggccagcagtaagggaaagaatgagacaaaccagccctgagccaaagccggctccgggggtgggtgaagtg
tctagagtgttagcacaaaggggagggatacctgcaaaatatactccacgaaacaatcaagctttagcgttcc
tagaagctcatgaagatgaggaggtaggatttccagtcgyacctcaagtgcctttaaggccaatgaccttta

agggcatttgacctcagcttcttttaaaagaaaagggggactggaagggttagttttctctcaggccaga
agtgagatcttagatctctgggtttatcatactcagggatacttccctgactggcagaattatacaccagggc
cagggataagatatcccctgacctttggatggctgtttaagttagtgccagtctcagaggcagaagctgagga
gatgggaacagcaagtgagagagctatactgctgcatccagcgtgcagccatgggagagacgacccacatgga
gagatccttgtctggagattcgatagagcattgggaacaactcatgttgcattacagaagcacccagaactgt
tcaagagagaataa
>#47 FJ424864 1401 nt
taccataaatatagaactaaacaagcaaaacagtggttctataggcatcactatgaagcagtcaatcctaggg
taagttcaggggtatacataccagtaggccccgctgaaattatagtgaccacatattggggactcatgccagg
agaaaaggaagaacaattaggccatggggtaggcatagaatggagacagggaaaatacataacacaattagat
ccagaaacagcagataggataatccatctttattactttcaatgttttacagaatctgcagtgaggaaagcaa
ttgtgggggaaaaactcctgtacaagtgtaactacccggcaggacataggcaggtagggacattgcaatactt
agcattaagagctttagtagggaaagtcagggtaagaccaccttttccaagtgttcagaaattagcagcagat
atgttagaagaacttaagcaagaagcartaagacactttcctaggccctggttgcaccaattgggacagcatg
tttatgatacttatggagacacttgggaaggagtgacagcaataatcagaatcttacagcaagtaatctttgt
tcattatagaattgggtgtcatcatagcagaatagggattttgccaccctctagaagaggaagaagactgcca
ccatggcagcaaccagggagccagcccccaaccccatgcaacacatgctattgtaaaaagtgctgttatcact
gctatgtttgtttcacaaagaagggtttgggaatctccatgggaaacacatggaaaaagagtagcttcatagg
atggccagcagtaagggaaagaatgagacaaaccaaccctgagccaaagccggctccaggggtgggtgaagtg
tctagagcattagcacaaagaggagggatacctgcaaaatatactccacaaaacaatcaagctttagcattcc
tagaagctcatgaagatgaagaggtaggatttccagtcaaacctcaagtgccgttaagaccaatgacctttaa
aggagcatttgacctcagcttcttttaaatgaaaagggggactggatgggttagttttctctcaggccaga
agtgatatcttagacctctgggtttatcatactcagggatacttccctgactggcagaattacacaccagggc
cagggataagatatcccctgacctttggatggctgtttaagttagtgccagtctcagaggcagaagcggagga
gatgggaacatcaaatgagaaggccatgctgctgcatccagcgtgcagccatgggagagacgacccatatgga
gagatccttgtctggagattcgatagagcattgggaacaactcatgttgccttacagaagcacccagaactgt
tcaagagagactaa
>#48 FJ424871 1398 nt
taccataaatatagaactaaacaagcaaaacagtggttctataggcatcactatgaagcaatcaatcctaggg
taagttcaggggtatacataccagtaggccccgctgaaattatagtgaccacatattggggactcatgccagg
agaaaaggaagaacaattaggcaatggggtaggcatagaatggagacaaggaaaatacataacacaattagat
ccagaaacagcagataggataatccatctctattactttcaatgttttacagaatctgcagtgaggaaagcaa
ttgtgggrgaaaaactcctgtgcaagtgtaactacccagcaggacatagtcaggtagggacattgcaatactt
agcattaagagctttagtagggagagtcagggtaagaccaccctttccaagtgttcagaaattagcagcagat
ttgttagaagaactaaaacaagaagcagtaagacactttcctaggccctggttgcaccaattgggacagcacg
tttatgatacttatggagacacttgggaaggagtgacagcaataatcagaatcttacagcaagtaatctttgt
tcattatagaattgggtgtcatcatagcagaatagggattttgccaccctctagaagaggaagaagactgcca
ccatggcaacagccagggagccagcccccaaccccatgcaacacatgctattgtaaaaagtgctgttatcact
gctatgtttgtttcacaaagaagggtttgggaatctccggaaacgcatggaaaaagagtagctttgtaggatg
gccagcagtaagggaaagaatgaggcaaaccagccctgagccagatccggctccaggggtgggtgaagtgtct
atagcattagcacaaagaggagggatacctgcaaaatatactccaaaaaacaatcaagctttagcattcctag
aagctcatgaaaatgaggaagtaggctttccagtcgtacctcaagtgcctttaaggccaatgacctttaaagg
ggcatttgacctcagcttcttttaaaagaaaagggaggactggaagggttagttttctctcaggccagaagt
gatatcttagacctctgggtttatcacactcagggatacttccctgactggcagaattacacaccagggccag
ggtaagatatcccctgacctatggatggctgtttaagttagtgccagtctcagaggcagaagctgaggagat
gggaacagcaagtgagagagctatattgctgcatccagcgtgcagccatgggagagacgacccacatggagag
acccttgtctggaggttcgatagagcattgggaacaactcatgttgccctacagaagcacccggaactgtttc
agagagactaa
>#49 JN091690 1374 nt
ttagttaaacaccatatattcacaagtaaatgttgcagcagctggaaatatagacaccattatgaaactgata
taccaaaaagagcaggagaaatacatattcccattaatgagaaagcaaaaatagtagtgttacactattgggg
tctagcctgtggggagagaccatggcatttaggacatggagtaggattggaatggagacaaggaaaatatgtc
acacaactagaccctgcaacagctgaccagttaattcatactaagtattttgcatgctttactgcaggagcaa
ttcgtcaagcaatcttaggagagagaattctgacattctgccaatttcgatcaggacacagacaggtaggaac
tttacaatatttagccttttgaaggtagtggagattcaatacaagacagctccaagacacaggaggccgttg
ccatctgtactaaagttaacagaggacattctagaagaaataaaacaagaagctgttaggcattttccaaggc
aaatattacaaggagtagggaattgggtctttacagtgcatggagactcctgggaaggagtacaagaattaat
cagaattctgcagaaagctttgtttgctcactatcgacatggttgtgttcacagcagaataggacctatagat
cctcaagtagccccatggaatcatccaggagctgcacctaagacaccatgttcaaattgttattgtaaaaagt
gctgctttcattgcccgctttgctttacgaaaaaagcattaggaatctccatgggaaacatctttggcaagtg

gcctggagcccaaagggcaatccaagcgatacatgagagtaatcctacaataaaagggcaagcatcacaggac
ctccaggcaaggggagggttgactactagcactctaggaacatcagcagatgtaataaattactctcatgacc
actctgaagaggaagtcggcttcccagttaggccagcagtaccaatgagacccatgacagaaaaactagcagt
agatctgtcatggttcctaaaagaaaagggggactggatgggttacctttctccacaaagagagcagccatc
ttagatatgtggatgtataatacacaaggaatattcccagactggcagaactacactcctggaccaggaatca
gatacccactatgcagagggtggctgtttaaactggtaccagtagacccacctgaagatgatgagaggaacat
cttgctacacccttactgttctcatggaagagaagatcctgcaggagaaggagagaatctgatctggtgcttt
gacagcagtctatcaagaagacacatagccagagagagatatccggagtacttcaaataa
>#50 AF447763 1380 nt
acatggacaagcctagttaaacatcatatctttacaaccaaatgctgtaaagattggaagtatagacatcatt
atgaaactgatacaccaaaaagagcaggggaaatacacatacctctaacagaaagatcaaaattagtggtttt
acattattggggtctagcctgtggagaaagaccatggcatctaggtcatggcataggattagaatggagacaa
ggaaaatacagtacacaaatagaccctgaaacagcagaccaattgattcacactaggtattttacctgttttg
ctgcaggagcagttcggcaagcaatattaggagaaagaatattgacattctgccactttcaatcaggacacag
acaggtagggactctgcaattcttagctttcagaaaggtagttgagagccaagataaacagccaaaggacca
aggaggcccttgccatctgttacaaaactaacagaggacatcctagaagaaataaaacaagaagcagtgaaac
actttccaagaccaatattacaggggtaggaaattgggtcttcaccatttatggagactcctgggagggagt
acaggaattaatcaagatcttgcagagagctttgtttacccactatcgccatggttgtatccacagcagaata
ggacccatagatcctcaggtagcaccatggaacatccaggagctgcacctgaaacaccttgtacaaactgtt
actgtaaaaaatgctgctttcattgcccagtttgctttacgaaaaaagcattaggaatctccatgggaaacat
atttggtagatggcctggggcccggaaagccatcgaagatcttcataacacctcaagtgagcctgtaggacag
gcctcacaagacctccagaataaaggaggtctcactactaacaccctaggtacctcagcagatgtgttagaat
actctgcagaccatactgaagaagaagtaggttttccagtcagaccagcagtacccatgagacccatgacaga
gaagctagcaatagatctgtcatggttcttaaaagaaaagggggactggatgggctatttttctctccaaaa
agagcagccatcctagacacctggatgtataatacacagggtgtctttccagactggcagaactacacccctg
gaccaggaatcagatacccactgtgtaggggatggttatttaagttggtaccggtagacccaccagaagatga
tgagaagaacatcttgctacatccagcctgtagccatggaactaccgatccagatggagagactctgatctgg
cgctttgacagcagcctagcaagaaggcacatagccagagaaagatatccggagtacttcaaataa
>#51 JQ768416 1383 nt
acctggaatagtctagtgaaacatcatatctttacaactaaatactgcaaagagtggaaatatagacaccatt
atgagacagatgtgccaaaaagagcaggggaaatacacatacctcttgaaggaaaggcaagaatagttgtgct
acattattggggattagcttgtggggagagaccatggcatttgggacatggagtagggatagaatggagacaa
ggaagatatagcacacagatagaccctgaaacggcagaccagcttattcacactaagtactttgcatgtttta
ctgcaggagcagtgcggcaagcaatcttaggagaaaggatactgacgttctgccattatcaaacaggacatag
acaggtagggactttacaatatctagcttttctaaaagtagttgaagctcaatacaagacagcaaagaaacct
ggaagacctttgccagctgtagttaaattaacagaagacgtgttagaggaattaaaacaagaagcagttaggc
actttccaagaccagtactgcagggagtaggaaattgggtgtttacagtttatggagattcctgggaaggagc
tcaagagttaatcaaaattctgcaaagggccttgttttctcattatcgacatggttgtgttcacagcagaata
ggacctatagatcctcaggtagccccatggatgcatccaggagctgcacctgagacaccttgtacaaattgtt
actgtaaaaagtgctgctttcattgcccgctttgctttacgaaaaaagcattaggaatctccatgggaaacat
tttggcagatgggctggtgctactgaggtcctacgcagattgcatgctgcacctgctcatgatgggcaggcc
tcaaaagaacttcatgaaagaggagggctcactaacaataccataggaacagaaaaagatgttgcccattact
ctcaagaccatacagaagaggaagttgggttcccagtcagaccagctgtaccaatgagacccatgacagaaaa
attagcaacagatctgtcatggttcttaaaagaaaagggggactggatgggttaatttactctacacgtaga
gcagctatcctggatacctggatgtataacacacaaggagtattcccagactggcagaattacacccctgggc
caggagtaagatacccactctgcagagggtggttatttaagctagtaccagtaactccacctgaggacgatga
aaggaacatcttgctgcatccagctaactctcatggaagagaggacccagatggagacggcgagactctcatg
tggcagtttgacaccagcctagcaagaagacacatagccagagagagacatccggagtacttcaaataa
>#52 JQ866001 1368 nt
gcttggaacagcctagtaaaacaccatatttataagagtaaacattgtaaagaatggaaatatagacatcatt
atgatatagaccaccccagaaagggaggagaagtgcatatacccataactgagaaagcaaaattggtaataac
aatatattggggcctacactgtggggaaaaagcttggcatcttggacatggggcaagcatagaatggagacaa
gggagatactggacagaagtagacccagaaacagctgaccaaattatacatagtagatattttccttgtttta
cagaaagggccataagacaagctattttgggagaaaagatcctacagcattgccatttccctacaggacattc
taaggtagggagcctacagtttctagcctttaaaaaagtgctagaacagaacaagcagaagcctcatagacct
ccattaccctcagtaactgtcctggcagaggatacattggaagaaataaaaaatgaggcaatgagacattttc
caagaaatacactacaaaacataggcaactgggtgtttcagaactatggagattcatgggaaggagtacaagt
gcttataactctcttacaaaaagccctgtttactcattatagatacgggtgtgcccatagcagaatagggata
ggaccaaggaggccttggttacacccaagctctacaccaagcacgccctgcaattcttgctattgtaaagtat
gctgttatcactgccaactttgctttaccaagaaggctctcggcatctcaatgggagctgtactaggaaccag

attcccagggatagacaacatgttgaacagaattagaaacacaccagcagcagatgcaccagggtcacaagat
ttgcataacagaggaggccttacaacaaacaccataggaacagaacatgatgtattacaacattcaggagacc
atacagaagaagcagtaggcttcccagtaagacctcaagtgccaatgagacctatgacagaaaaattggcagt
agacctctcatggtttttaaaagaaaagggggactggaagggctatattggtctccaaagagagcagcaata
ctagatacctggatgtttaacactcaaggaatattccctgattggcagaattacacaccagggccaggagtta
gatatcctctgtgtagaggttggttatttaagttagtaccagcagacccaccagaagaggatgaaaggaatct
actcatgcaccctgcttgctcctatggaagagatgaccctaatggagaactgttggtatggaagtttgactct
gaactggctagaagacacatagccagagagagacatccggagtacttcaaataa
>#53 DQ373065 1389 nt
ttggtaaagcatcacatgtatgtgtcaaagaaggcacgaggttggttctatagacatcattatgaaacagata
atccaaaaataagttcagaaatacacatcccattaggagaagcaaaattagtaatagttacctattggggatt
aatgccaggagaaaggccatggcatttaggacatggagtatccatagaatggagacaaggaatatacaggaca
caaatagatcctgaattggcagacaagctaatacacctttattattttgattgttttacagcctctgccatca
ggcaagcggtcttagggagaccagtaatacctaagtgtgaataccctgcagggcataaacaggtaggctccct
acaatatctggcattaatagcctgggtgggagtacagaagagaagaccacccttacctagtgtgaccaagtta
acagaggatttgttggaagacctaaagaatgaggctctgcgccactttcctcggccttggctacatggactag
ggcaatacttctataatacatatggagatacctgggagggagtagaggccatcattaggacactacaacaact
gttgtttatacattataggattggctgtcaacatagcaggataggaatcactcctcaaaggagaaggttagag
ccctggaatcacccaggaagtcaacctaagacagcttgcaataattgccattgtaaagtttgttgctatcact
gtgtgtattgcttcacaaaaaaaggcttaggcatctcaatgggaaaagtttggtcaaagagtagccttgtggg
gtggccacaagtcatagatagaatgagaagacagcaagatccagcagaaggggtaggagcagtttctcaagat
ctagctaatcgaggggctattaccataagaaatactaaggagaataatcaagccctagcttggctagaagagc
aaaaagaggaagcagtaggctttccagtatgtcctcaggtaccattaagaccaatgacatacaaagaagcttt
tgatctttccttctttttaagagaaaagggggactggaagggctagtttggtcaaccaagagacaagaaatt
ctagacctctgggtttatcatactcaaggaatcttccctgactggcagaactacactccaggaccaggcgtca
gataccccctgacctttggatggtgcttcaagctagtaccactgtcacctgaggaggtagaagaagctaatca
aggagacaacaatgtcctgttacatcccatgtgccaacatggaatggaagatccagacaaagaagtgctggtg
tggcgctttgacagctccctagcaagagtacacagagcaagagagctgcatccggagttctaccagaactgct
ga
>#54 AF103818 1422 nt
gcttggaatagtctggtaaaatatcacatgtatagatcaaagaaagctaaaggatggttctacaggcatcatt
atgagagcactaatcccagagtaaattcagaagtacatatccctatagataaagcaaaaatagtgattataac
gtattgggggttgaaaacaggagaacaagcctggcacttagggcatggagtatcaatagaatggagacaagga
acttacaagacacaaatagatcctgagatggcagacaagctaattcatctgtattattttgattgttttacag
cctctgccatcagaaaagctgttttagggagaccagtaattcccaaatgtgaataccctacagggcacaataa
ggtaggctctttgcaattcttggctttaaaggccttagtaggacagagcaaaagaagaccacccttacctagt
gtgactaaattgacagaagatgtcttagaagaactaaaagaagaggctttgcgccattttcctagaccctggc
tacaaggcttaggacagtacatctacagtacttatggagacacttgggagggagtagaagcaattataaggat
cttacaacaacttttgtttatccattataggattggctgtaatcatagcaggatagggatttccttggcaagg
agaactcctcaagggagaaggatagagccctggaagcagccaggaagccagcctaagactgcttgcaataatt
gctactgtaaaaagtgttgctttcactgtgtggtttgttttacgaagaagggcttaggaatttccggtaacaa
gtggtctaagagtagcatagtaggatggccagaagtcagaaacagattgagacaaactcagacaacagcagca
gcagaaggagtaggacctgtctctcaagacttagcagaacatggggctatcactactagaaatacaccacaga
acaaccaaactctggcatggttagatgagatgactaatcatgaaagtgaagtaggcttccctgttcgccctca
agtaccattaaggcccatgacttacaaacaagcattcgatttaggcttctttttaaaagaaaagggggactg
gaagggttagtttattccagaagaagacaagagatcctagatctctgggtttaccatacacaaggaatcttcc
ctgactggcagaattacaccccagggcctggagtaagatatcccctaacatatgggtggtgctttaagttagt
ccctctcacagaagaggaagtagagcaggccaataaaggagagaccaacatactgctgcaccccatgtgccag
catggaatggaagatgaacatggcgaagtcttaatctggcagtttgacactgaactggctcggagacacagag
ctaaagagctgcatccagagtacttccggaactga
>#55 EF535993 1392 nt
ctagtaaaataccatatgtacagaacaaagaaagcaagaaaatggttttataggcaccattatgaaagttcta
accccagagtaagttcagaaatacatatccctttagaaaaagcaaaattagtggtcaccacctattggggatt
aatgccaggagagcatgaatggcacctaggacatggagtgtccatagaatggagatatgggtcatacagaaca
caaatagatcctgaaatggcagatagactaattcaccttcattattttgattgttttgcagcttctgccatca
gaaaagctgttttagggacacaagtagttcctaagtgtgagtacccagcagggcaccaacaggtagggtccct
acaatatttagccttaaaggcctgggtaggagtagaaaagaaaagaccacccttgcctagtgtcgccaagtta
gcagaagatctattggaggaactgaaaaatgaggctgtacgacactttcctcgagcatggttacatggattag
gacaacacatatatgacacatatggagatacatgggcaggtgtagaagctatcattagaatactgcaacagct
cttgtttattcattataggattggctgtcaacatagcagaatagggattaaccctgggaggaggagactagag

ccctggcaacatccaggaagccaacctagaactgcttgcaataattgctattgtaaatgttgctgctttcatt
gcatggtctgttttgagaaaaaaggcttaggaatctccatgggcaacaaatggtcaaaaagcagcctggtagg
atggccagaagtaagagctagaataagacaagctcaaccagctgcaccaggggtaggatcagtctcacgagat
ttagaggcacatggagcaataaccactagaaataccccacaaaacaatcaaactctagcatggctagaagaaa
tgcagggacatgaagaggaggtaggctttccagttagaccacaagtaccccttagacctatgacatttaaagg
agcttttgatttaggctacttttaaaagaaaaggggggactggaagggctaatttactccagaagaagacaa
gagatcttggatctctgggtctatcatacacaaggattcttccctgattggcagaattacacaccaggaccag
gagtcagatatccactaacatttgggtggtgctttaaactggttccactgacaccagaagaagtggaaaaagc
caatgaaggagataccaacatactgcttcaccccatctgccagcatgaaatagaagatgaacatggagaagtc
ttggtttggagatatgacagcaggctagctttgagacacattgctagagaacaacatccggagtactaccagg
actga
>#56 AY169968 1410 nt
gcatggaatagcctagttaaatatcatatgtataaaacaaagaaagcaaaagattggttctacagacaccact
atgaacatcctaatccaagagtttcttcagaggtacacattccttttaaagataattcaaaattaataatcac
aacctattgggctttaaccacaggagaaagagcgtggcatttaggtcatggagtctccatacagtggaggatg
ggatcatatgttacacaggtagatccttatgtagcagataaattgatccactctcaatattttgattgttttg
cagactctgccatcagaagagctatattagggcatattgtagaaccccggtgtgcttacagagaaggacacag
gcaggtaggcaccctacaattcctagctttaaaagctttagtaaaagaaaaaaggcttagaccacccttaccc
agtgtagctaaattaacagaggatatattagaagacttaaagaatgaagctgtaaaacactatcccagaagtt
ggcttcaccaattaggccagcatatttataccacctatggggacacgtgggaaggggtggaggcaattataag
aatcctacaacaactactgtttgttcattttagagttgggtgccaccatagcagaataggtataataccacaa
aggagaagagcattggagccatggaagcatccaggcagccagccaaaaacagcttgtaataattgctattgta
aagcttgttgtttccattgtatgtattgcttcacaaagaaaggcttaggaatttccatgggagctcagtggtc
taaaagtagcctagtaggatggcctgaaatcagaaaaagaataaaagaggctccaatagcagcagaaggagta
ggagaggtttctaaggatctagaaaaatatggagctatcactagtagacatacaccaagtactaatcaaactt
tagcatggttagatgaaatgaaacatcatgaagaagaagtgggctttccagttagaccacaggtgccattaag
accaatgacttacaaaggagcctttgacctttcccacttttttaagaggaaaggggggactggaagggttagtt
tactcccagaaaaggcaagaaatcttagatctctgggtttatcatacacaaggcttcttccctgactggcaga
actacaccccaggaccaggagagagatacccaccctgctttggatggtgctttaaactagtacctttgacaac
tgagcaagtggaagcagccaatgagggagataacaactgcttactgcaccctatatgtcagcatggaatggag
gatgaaagtaaagaagtcttaatatggcgctttgacagcagactggctttaagacatatagcaagagagaaac
atccggagtattacaaagactga
>#57 AF382828 1392 nt
ttagtaaagtttcatatgtacaagtcaaaaaaggccagaggatggttttataggcaccattatgatagtccca
atcctaaagtaagttcagaagtccacatacccataagagatgcaaaaatagtggtctgtacatactggaattt
aatgccaggagaacaaccatggcatctaggccatggagtgtccatagagtggaggcagagaacatatagaact
cagatagatcctcaaacagcagatcggctgatacactgttattattttgattgttttgcagcctctgctgtca
gaagagccatcctaggggagattgtgacaaaggtgtgtgaattccctaggggggcatcctcaagttcagtcact
acaatatctggccatagtacaagtagtaaagacaaagaaaaagaagccacccctacctagtgtcactatctta
gcagaagatatattggaggacttaaaaaacgaggctgtcagacattttcctagaccatggctacatcagttag
gacaattaatctacaataattatggagatacatgggaaggagtagaggccataatcagaatacttcaacactt
gctgtttgtgcattttaggattggctgtcaacatagcaggatagggattgaacgacagaggagaagaaggaga
cttgagccctggaatcatccaggaagtcagccaaaaacagcttgcaattcatgctactgcaaaaggtgctgct
atcattgtatgctttgcttcaccagcaaaggcttaggtatttccatgggcaacaaatggtctaaaagcagcat
tgtaggatggccgcaggtcagagagagacttaggcggactcaggaagcagcagaaggagtaggagaagtgtct
caggatttagcaaggcgtggagcaataacttccagacataccccctcagactaatcaaactcttgcatggctag
aagaaatggaacaggaggatgtagggtttccagtaagacctcaggtacctttaagaccaatgacatataaagc
agcctttgatctcagccacttttttaaaagaaaaggggggactggaagggttagtttattccaagagaagacaa
gagatcctggacctctgggtttatcatacccaaggatacttccctgattggaataactatactcctggaccag
gagtgcgttatccgttgacctatggatggtgcttcaagctagtgccagtatcaccagaggcagtagaagaagc
caacaagggagaggataatctcctattacatcccctgtgcactcatggatttgaggatgaagagaaggaggtt
ctgatatggaagtttgacagtcaactagccctgcggcatcttgcaagagaaaaacatcctgagtattacagag
actga
>#58 EF535994 1404 nt
gcatggaacagtttggtaaaacatcacatgtatgtgtctaaaaaggcacgaggttggttttacagacatcatt
atgaaagtgagaatccaaaagttagttcagaaattcacattccagtaggagatgccaaattagtaatagtaac
ctattggggactcatgccaggagaaagagactggcatttaggacatggggtctctatagaatggagacagaaa
agatatagcacacaaatagatcctgaattggcagaccacctaattcatttgcattattttgattgttttgcag
agtctgccataaggaaagccatactaggacaaatagttagccctagatgtgaattcccagcaggacacacaaa
ggtaggcactctacaatatttggcaatctcagcactgctaaaaggaagccaaagaaaccacctctacctagt

gttagtaaattagcagaagacttattagatgaactcaaacaggaagcagtaagacattttcctagacagtggc
ttcatgatttaggacagcacatttataacacatatggagacacttgggcgggggttgaggctatcataaggat
cctgcaacaattgctgtttattcattacagaattggctgccaacatagcagaataggcattctgccacaagga
agaaggagattagaaccctggaatcatccaggaagccagcctaaaccccctgtaacaattgctattgtaaaa
tgtgttgctatcactgtcaattttgcttcacaagaaaaggcttaggaatttacatgggaaacaaatggtcaaa
aagtagcctggtaggatggcctgcagtaagggacagacttagaaggacacctccagcagcagaaggagtaggg
ccagtctcgcgagacctagagagacatggggctataacaagtaggaatactccacagacaaatgagactcttg
cctggttagaggagcaaagggacgaagaagtaggatttccagtaagaccccaagtgcctttaaggcccatgac
ctataaagcggcctttgatcttagccacttttttaaaagaaaagggggggactggaagggctaatctactcccaa
agaaggcaagagatccttgatctctgggtctatcatacacaaggcttcttccctgattggcaaaactacacac
caggaccaggggtcagatacccggtgaccttcggctggtgcttcaagctagtgcctgtagatccaacagatgt
ggaggaagcaaatgaaggagacaacaatgtgctattacatcctatgtgccagcacggacaagaagatgaacac
agagaagtgctgatgtggcagtttgacagccacttggccctgacacacagagcccgagagctgcatccggagt
actacaaagactgctga
>#59 DQ373066 1401 nt
acctggaatagtttagtgaaatatcatatgtacagatcaaagaaagcaaaaaattggttctatagacatcact
atgatggcaccaaccctaggatggcttcacagatatatattccaataaatcaggctaagctaattgtcacaac
atattggggactaatgccaggagaaagagattggcatctgggacatggagtagcaatagaatggagagaaaga
ggttacagtacccaggtagaccctgaaacagcagataggctaatacaccttcattattttgattgttttacag
caactgccatcaggaacgctatcttagaaaaggcagtcctccctaagtgtgaattcccagaagggcacagcaa
ggtagggtctttacaatactgggctctaaagcagttggttaaaaccaagaaaaatagaccacctctgccctca
gtagcaaagttagcagaagatacattggaagaaattaaacaagaagcagttagacactttcccagaccctggt
tacatgcactaggagaacatatttatgaaacatatggagactcctgggcgggagtacaagctataataagaat
tttgcaacaattactgtttgtccatttcagaattggctgccatcatagcagaataggcataaaccctcagaga
agaaggagattagaaccatggaaccatccagggagcaaacctagaacagaatgcaataagtgcttttgcaaaa
agtgttgttatcattgtatactttgctttacaaagaaaggcttaggcatctccatgggagccaagtggtcaaa
aagtagcctagtaggatggccagaaataagagagagagaataaaaagagctccaacagcagcagatggagtaggg
gaagtatcacaagacatagcaagccgtggagcagtcacaattagaaataccccacaaaccaatgagactttag
cttggctagaagaaatgcaggatgaagaagtgggatttccagtcaaaccacaagtgcccttaagacctatgac
ctttaagggtgcctttgatctctctcacttttttaaaagaaaagggggggactggaagggttaatctggtcaagg
aaaagacaggagatccttgatttatgggtttatcacacacaaggttacttccctgactggcaaaactacaccc
cggggccaggagtgcgctacccactaaccttcgggtggtgctttaaactggtccctttgccacctgaggaggt
ggagaaagccaatgaaggagaaaacaactgcctgctacatcccatgtgccagcatggaatggaggatgaggaa
agagaagtactggtatggacttttgatagcaagctggctctcagacatctagcaagagagaaacatccggaat
attacagagactga
>#60 DQ373064 1404 nt
gcatggaacagtttggtaaaatatcatatctatgtatccaggaaagcaagaggctggttttatagacatcatt
atgagagtgaaaatccaaaagtaagttcagaaatacatatcccactaggggaggccaaattagtaatagtaac
ttattggggacttacaccaggagaacgggactggcacttaggtcatggggtatctatcgagtggaaacagaag
aaatatagtacacaaatagaccctgagttggcagatcatatgattcatttatattattttaattgttttgcag
aatctgccataaggaaggccatattaggacgagtagttagccctagatgtgaatttccagcaggacacaagaa
ggtaggaaccctacaatatttggcaatctctgccttattacaaagacagccaacaaggccgcctttgcctagt
gtcagcaaattagcagaagatgtattagaagaactcaaacaggaagcagtaagacactttcctagaccatggc
tccaccaattgggacaatatatatatgacacctatggagacacctgggagggagttcaagctatcataagaat
cctgcaacaactcctgtttattcattacagaattggctgtcaacatagcagaataggcattacaccacaaaga
agaaggagactagatccctggaatcatccaggaagtcagcctaaaactccctgtaacaattgcttttgtaaaa
agtgttgctatcactgtccattttgcttcacaaagaaaggcttaggaatttccatgggaagcaaatggtcaaa
aagtagcctggtaggatggcctgaagttagaagaagaattagacaagaaactgtagcagcagaaggagtagga
ccagcctcacaagacctagctaggcatggggcaattacaagtagtaataccccacaaactaatgagaccattg
cctggttagaggagcaaaaggaggaagaaatatggtttccagtaaaacctcaggtaccattaaggccaatgac
ttttaaagaggcttttgatcttggctacttttttaaaagaaaagggggggactggaagggttagtttactctaaa
aagagacaagaaatccttgacctcggggtttatcacacacaaggaatcttccctgactggcagaattacaccc
cagggccaggattagatatccaataacctttggcgggtgcttcaagctagtaccactaaccccagaagaggt
ggaggaggctaataaaggagagaacaatctactactgcacccaatatgccagcatggaatggaagatgaagac
aaagaagtgctgatatggaaatatgacagccagctcgccctaagacacatagctcgagagctgcattcggagt
actacaagaactgctga
>#61 KP861923 1389 nt
ttggtaaaacaccacatgtatgtgtccaaaaaagcaaaaaattggttttatagacatcattatgaaagtacaa
gtccaaaagtaagctcagaaatacacattccaataggagatgctaaattagtaatagtaacttattggggtct
gcaaccaggagaaagagactggcatctgggacatggagtgtctatagaatggagacagagaagatatagtaca

cagatagatcctgagttggcagaccacctgattcatttgcattattttaattgttttgcagaatctgccataa
ggaaggccctattaggacacatagttagccctaagtgtgaatatccagcaggacacttaaaggtaggctcctt
acaatatttggcaatatcagccttgttcaaaagcaacccaaaaagaccacccttgcctagtgtaagtaaacta
acagaggatgtgctagaagaacttaaacaagaagcagtaagacattttcctagacagtggcttcatggcttag
gacagtacatatacaacacatatggagatacctgggcgggagttgaagccataataaggattctgcaacaatt
gctgtttattcatttcagaattggctgtcaacatagcaggataggcattgtggttcgaagaaggacaatagag
ccctggaatcacccaggaagccaacctaaaactgcatgtaacaattgttattgtaaaagctgtagctttcatt
gtccactttgcttcatgaaaaaaggcttaggcatttccatgggagccaaatggtcaaagagcagcctagtagg
atggcctgaggtgagagaaagaatcagacaaacacaaccagcagcagaaggagtaggaccagtgtcaagagac
ctagacaatcatggagcaatcacagtcagaaatactccacaaactaatcaaacccttgcctggttagaagagc
agaaagaggaagaagtaggtttcccagtaaggcctcaggtaccattaagaccaatgaccttcaaaggtgcgtt
tgatctcagccacttttaaaagaaagaggggactggaagggctaatttattccagaagaagacaagagatc
cttgatctctgggtttatcacacacaaggcttcttcccagactggcaaaactacacaccaggaccaggcgtca
gatatccactgacctttggctggtgcttcaagttagtaccagtgcctccagaggaagtggaaagagccaatga
aggagagaacaatgtgctgctgcatccaatgtgccagcatggcatggaagatgaagatcgagaggtcctgcaa
tggcgctttgacagtcacctcgccctgagacacatagccagagagcaacatccggagtactacaaagactgct
ga
>#62 AJ302647 1401 nt
tacaggtcagggaagaccagagactggtattacagacaccattttgaatctaaaaatccaagagtcagctcag
gtgtatatattccagtaggagggccttggatagtagtgaccacatattggggattgatgccaggggaaagaga
tgaacatttgggacatggggttagcatagactggaggtacaggaagtatacaacacagattgaccctgaaaca
gcagacagaatgatacatacatattattttgcctgttttacagagtcagcaatcaggaaagccatcttagggc
agagagtactgaccaggtgtgaatactctgcaggacatagtcaggtagggacactgcaactactagctctaag
agtggtagtaaaagaaaaaagacataagcctcccctacccagtgtccagaagttaacagaagatctcctagaa
gaactaaaagcagaagcagtaagacatttccctagaccttggctacaggccttgggacaatacatttatgaca
cttatggggacacttgggtaggagttatggccattataagaatcttacaacacctgctatttgcccattttag
aattggatgccaacatagtagaataggaattaacccatctaacacaagaggaagaggaagaagaatgcccccct
tggcatcaccctggaagtcagccccagaccccttgtaataagtgctattgcaaagcgtgctgctaccattgct
atgtttgttttgcaagcaagggtttgggactctccatgggaaacgtattgggtaaagataaatttaaggatg
gtcagcagtcagagaaagaatgagaaaaacttcccctgagcctgaaccatgtgcacctggagtaggacaagtc
tccagggaattagcagctagaggagggatatcaaattcccatactcctcaaaacaatgcagcccttgcattcc
tagaaagtcaccaagatgaagacgtaggcttcccagtaagacctcaagtgcctctaaggccaatgacctataa
aggagcatttgacctcagcttctttttaaaagaaaagggaggactggatgggttaatttactcccctgaaaga
gcagagatcctggatctttgggtgtatcacactcagggattcttccctgattggcagaattacacaccaggac
caggaacaagattcccactgacatttgggtggctatttaagctagtaccagtgtcagaagctgaggcagaaga
actaggaaataagtgtgacagggctaaactcctgcatccagtttgcaaccatggctttgaagatccacacaag
gagatgctgaaatggcagtttgatagatcactaggcagcacccatgttgctctgataacccacccagagctct
ttctcaaggactaa
>#63 AJ302646 1422 nt
tacaggtcagggaagaccagagactggtattacagacaccattttgaatctagaaatccaagagtcagttcag
gtgtatatattccagtagggggggccttggatagtagtgaccacatattggggattgatgccaggggaaagaga
tgaacatttgggacatggggttagtatagaatggaggtacaagaagtataaaacacagattgaccctgaaaca
gcagacagaatgatacatatatattattttgcctgttttacagagtcagcaatcaggaaagccatcctagggc
agagagtactgaccaggtgtgaataccctgcaggacatagtcaggtagggacactgcaactactagctctaag
agtggtagtaaaagaaaaaagaaataagcctcccctacccagtgtccagaagttaacagaagatctcctagaa
gaactaaaagcagaagcagtaagacatttccctagaccttggctacaggccttgggacaatacatttatgaca
cttatggggacacttgggtaggagttatggccattataagaatcttacaacaactgctatttgcccattttag
aattggatgccaacatagtaggataggaattaccccatctaacacaagaggaagaggaagaagaaatggatcc
agtagatcctgaatgccccccttggcatcaccctggaagtcagccccagaccccttgtaataagtgctattgca
aagcatgctgctaccattgctatgtttgttttgcaagcaagggtttgggaatctccatgggaaacgtattggg
taaagatatatttaaggatggtcagcagtcagagaaagaatgagaggaacttcccctgatcctgaaccatgt
gcacctggagtaggacaaatctccagggaattagcagctagaggagggataccaagttcctatactcctcaaa
acaatgcagcccttgcattcctagaaagtcaccaagatgaagaagtaggcttcccagtaagacctcaagtgcc
tctaaggccaatgacctataaaggagcatttgacctcagcttctttttaaaagaaaagggaggactggatggg
ttaatttactcccataaaagagcagagatcctggatctttgggtatatcacactcagggattcttccctgatt
ggcagggctacacaccaggaccagggccaagattcccactgacatttgggtggttatttaagttagtaccagt
gtcagaagctgaggcagaggaactaggaaacaagtgtgagagggctagcctcctgcatccagcttgcaaccat
ggctttgaagacaaccacgggcaaatactgaaatggcagtttgatagatcactaggcagcacccatgttgcta
tggtaaccaacccagagctctttaacaaggactaa
>#64 AY169812 1407 nt

tacaggtctgggaaggccaggaactggtattacaggcatcattttgaatctagaaatccaagattcagttcag
gtgtacatattccagtagggatggcttgtatagtagtgaccacatattggggactgatgccaggggaaagaga
ggatcagatgggacatggggttagtatagaatggcagtacaaacagtatacaacacagattgaccctgaaaca
gcagacaggataatacatctgtactatttacttgttttacagagtcagcaatcaggaaagccattctagggc
agagagtattgcccaggtgtgaatacactgcaggacatagtcaggtagggacactgcaactattagctctaag
agcagtagtaaaagcaaaaagaaataagcctcccctacccagtgtccagaaattaacagaagatactctagaa
gaactaaaaacagaagcagtaagacattttcctaggcyttggctacakagcttrggacaatacatttatgaga
cttatggggacacttgggtaggagttatggcaattataagaatcttacaacacctgctattcacccattttag
aattggatgccaacatagtagaataggaattaacccatctaacaccagaggaagaggaagaagaatgcccccca
tggcatcaccctggaagtcagccccygaccccttgcaacacttgctattgcaaaagatgctgctatcattgct
atgtttgtttcacaagaaagggtttgggaatctccatgggaaacgtattggggaaagataaatttaaaggatg
ggcagcagtaagagagagagaatgagaaaaacttcctctgatcctgatcctcaaccatgtgcacctggagtaggg
ccagtgtccagggaactagcagctagaggaggagtaccaagttcctatactcctcaaaacaatgcagcccttg
cattcctagaaagtcatgaagatgaagatgtaggtttcccagtargacctcaagtgcctctaaggccaatgac
ctataaagcagcatttgacctcggtttctttttaaaagaaaagggaggactggatgggttaatttactcccat
argagagcagagatcctagatctttgggtvtatcacactcarggattttccctgattggcagaactacacac
caggaccagggactagatacccactgacatttggatggttatataagctagtaccagtgwcagaaacygaggc
aaargaactaggaaacacatgtgagagggctatgctcctgcatccagcttgtgcacatggctttgahgatcca
cacggagaaatactgaaatggcagtttgatagatcactagcctccacccatgttgctaagataactcacccag
agctcttccccaaggactaa
>#65 AY169816 1407 nt
tacaggtctagaaagaccaaggactggtattacagacatcattatgaatccagaaatccaagagtcagttcaa
gtgtatatattccagtagagaragcctatatagtagtgaccacatattggggattgatgccaggagaaagaga
tgwacatmtgggacatggagttagtatagaatggcagtataaaaaatataaaacacagattgaccctgaaaca
gcagacaggatgatacatctgcattatttcacctgttttacagacacagcaatcaggaargccatcctagggc
agagagtactgaccaagtgtgaataccctgcaggacatagtcaggtagggacactacagctactagctctaac
agcaatagtaaaaacaagaaacagtaggccccccctacccagtgtccagaaattaacagaagatctcttagaa
gaattaaaarcagaagcagtaagacattttcctaggccctggctacatgccttgggacaatacatttatgaga
cttatggggacacttgggaaggagtkatggcaattacaagaatcttacaacaactgctgtttacccattatag
aattggatgccaacatagtagaataggaatttacccaactaatacamgaggaagaggaagaagagtgcctgct
tggcaccaccctggaagtcaaccccagaccccttgtaataagtgctattgcaaagaatgctgctatcattgct
atgtttgtttcacaagcaagggtttgggaatctccatgggamacgtattgggaaaaaataaatatgagggatg
ggcagcagtaagacaaagaatgaggagaacwagaatttcccatgagcctgaaccatgtgcacctggagtagga
cagatttccagggacttagcagctagaggrgggataccaatttcccatactcctgaaaacaatgcagcccttg
cattcctagaaagtcaccaagatgaagaagtgggtttcccagtaaggcctcaagtacctctaaggccaatgac
ctataaaggagcatttgacctcagcttctttttaaaagaaaagggaggactggaagggttaatttactcccat
aagagagcagaaatcctggatctttgggtgcatcacactcagggattcttccctgattggcagaactatacac
caggaccaggaactagattcccactgacatttgggtggctatttaagctagtaccagtgacagcagaagaagc
agaaagactaggaaatacatgtgagagggctcatctcctgcacccagcgtgttcccatggctttggtgatcca
catggggaaatactaatgtggaagtttgatagatctctaggcaacacccatgttgctcagataacccacccag
agctcttccagaaggactaa
>#66 KU168282 1458 nt
tacagatctagaaaggctaagagatggaattatagacatcattatgaaacaaggcatccaaaaattagttcag
ttatatacattccaatagcagaagctgagataatggttaccacatattggggattaatgccaggggaaagaga
ggaacacttgggacatggggtcagtatagaatggcaatacaagaagtataaaacacagattgatcctgaaaca
gcagacaggatgatacatctgcattattttacctgttttacagagtcagcaatcaggaaggccattctagggc
agagagtgctgaccaggtgtgaatacactgcaggacatagtaaggtagggacactacagttcttagccttaaa
agcattagtgaaagtaaagaaaaacaagcctcccctacccagtgtccaaaagttaacagaagacagatggaac
aagcaccagagaatcaagggccagctagagagcaatccaatgtgtggacatctcctggaggagctaaaacaag
aagcagtgagacatttccctaggacttggctacaagccttagggcaatacatttatgatacttatggagacac
ttgggagggagttatagcaattataagaatcttacaacaaatactgtttatccattatagaattggatgccag
catagtagagtaggaattattccatctaatacaagaggaagaggaagaagaatgcctccttggcatcaccctg
ggagcaggccccaaaccccttgtaataattgctattgcaaacgctgctgctatcactgcctcctttgtttcac
aaagaagggtttgggaatctccatggggaatgcctggagcaaaagcaaatttgcaggatggtcagaagtaaga
aggagaatgagacaaacctcccctgagcctcagccatgtgcacctggagtaggagaagtctccagggcattag
cagacagagggggaataccaaattcctacaatcctcaaaacaatgcagctctcgcattcctagaaagccacac
agatgaggaagtaggtttcccagtaaaacctcaagtgcctctaagaccaatgacctacaaagcagccgttgac
atcagcttctttttaaaagaaaagggaggactggaagggttaatttactcccataagagagcagaaatcctgg
atctctggatatatcacactcagggattttccctgattggcagtgttacacaccgggaccaggaactagatt
cccactgacatttggatggctatttaagttagtgccagtgtcaagagaggaggcagaaggactgggtaataca

cgtgaggatgctagtctgctacatccagcttgtaatcatggcgctgaggatgaacacggggagatgctaaaat
ggcagtttgatagaacattaggcagcacacatatagccctgcaaaagcacccagagctcttccccaagtaa
>#67 AY169809 1407 nt
tacaggtctaggaaaaccaaggactggttttacagacatcattatgagtctagaaatccaagagttagttcag
gtgtatatattccagtagggrtaggttgtataatagtgaccacatattggggattaatgccaggggaaaggga
tgagcagttgggacatggagttagtatagaatggcagtacaagaggtataaaacacagattgaccctgaaaca
gcagacaggatgatacatctgtattatttcacctgttttacagagtcagcaatcaggaaagccaccctagggc
agagagtgctgaccaggtgtgaataccctgcaggacatagtcaggtagggacrctgcaactattagckctgag
aatagtagtaaaagaaaaaaggaataagccccccctacccagtgtccagaagttaacagaagatctcctggaa
gaactgaaagcagaagcagtgagacatttccctaggaattggttacaagacctgggacaattcatttatgaga
cttatggagacacttgggtaggagttatggccattataagaatcttacaacagatactatttacccattttag
aattggatgccracacagtagaataggaattaacccatctaacacaagaggaagaggaagaagagtgcccccct
tggcaccaccctggaagtcagccccagaccccttgtaatgcttgctattgcaaaaagtgctgctatcattgct
atctttgtttcacaagcaagggtttgggaatctccatgggaaacgcattraagaaaagtagctttgtgggatg
gccagcagtaagagaaagaatgagaaaagctcccagtagtgatcctgagccatgtgcacctggagtaggagaa
ctctccagagaattagcagctagaggagggataccaggctcatatactcctcaaaataatgcagcccttgcat
tcctagaaagccaccgagaagaagaagaagtaggtttcccagttaaacctcaggtgcctttaaaaccaatgac
ctataaaggagcatttgacctcagcttctttttaaaagaaaagggaggactggatgggttaatttactcccat
actagagcagagatcctggacctttgggtgcataacactcagggattcctccctgattggcagaactacacac
caggaccaggaaccagattcccactgacatttggatggttatttaagctagtaccagtgtcagaagctgaggc
agaagaattaggcaataagtgtgaaagggctagactcctccatccagcttgtgatcatggttttggagatcac
cacggggaaatactgaaatggcagtttgatagatcactaggcaacactcatgttgctatgataacccacccag
agctcttccacaaggactaa
>#68 KU168283 1401 nt
tacagatctagaaagaccaaggaatggtattacagacaccattttgaatccagaaatccaagattcagttcag
gtgtacatatcccagtagggaaggcttgggtagtagtgaccacctattggggattgatgccaggggaaaaaga
tgaacatttgggacatggggttagtatagaatggcagtacaagagatatacaacacagattgaccctgaaaca
gcagacaggataatacatcagtattattttacctgttttacagagtcagcaatcaggaaagccgtcctagggc
acagagtgctgaccaaatgtgaatactctgcaggacatagtcaggtagggacattgcaactactagctctaag
agcagtagtaaaagaaaaaagaaataagcctcccctacccagtgtccagaaattaacagaagataccctagaa
gatctaaaagcagaagcagtaagacattttcctagaccatggctacatgccttgggacaatacatatatgaaa
cttatggagacacttgggtaggagttatggcaattatgagaatcttacaacaactgctatttacccattttag
aattggatgccaacatagtagaataggaattaacccatctaacacaagagaaagaggaagaagaatgcccccct
tggcatcaccctggaagtcaaccccagaccccttgtaataagtgctattgcaaaagatgctgctatcattgct
atgtttgtttcacaagaaagggtttgggaatctccatgggaaacgcattgaggaaaagcccatttccaggatg
ggcagcgataagagagagaatgagaagaacctcccctgagcctgaaccatgtgcacctggagtaggacaaatc
tccaaggaattagcagctagaggagggataccaagttcctatactcctcaaaacaatgcagcccttgcattcc
tagaaagtcaccaagaggaagaagtaggtttcccagtaagacctcaagtgcctctaaggccaatgacctataa
agcagcatttgacctcagcttctttttaaaagaaaagggaggactggaagggctaatttactcccatcagaga
gcagagatcctggatctttggatttatcatacccaaggattcttccctgattggcagaattacacaccaggac
caggaactagattcccactgacatttgggtggttatttaagctagtaccagtgtcagaagctgaggcagaaga
actaggaaataaatgtgagagggctaaactcctgcatccagcttgcaaccatggctcggaagatgcacacgga
cagatactgaaatggcagtttgatagatcactaggcaacacccatgttgctatgataacccatccagagctct
tcaacaaggactaa
>#69 AY169813 1422 nt
ctggtgaaataccataagtacaggtctaaaaaggcccagagatggcattatagacatcattatgaaaccagaa
atccaagaattagctcaggtgtatatattccagtaggtacagctaatatagtggtgactacatattggggatt
aatgccaggggaaagagatgagcatttaggacatggagtcagtatagaatggcaatacaaaaaatatagtaca
cagattgaccctgaaacagcagatagamtratacatctgcattatttcacctgttttacagaracagcaatca
ggagtgccattttagggcagagagtgctgatcaggtgtgaataccctgcaggacatagccaggtagggacact
gcaactcctagcattaagagcagtggtaaaagacaaaagaagtaaacctcccctacccagtgtccagaagtta
acaggagacaccctggaagaactaaaagcagaagcagtaagacactttcctaggccttggctacaaaacttag
gacaatacatctatgatacttatggagacacctgggaaggagttatggcaatcataagaatcttacaacagtt
gatatttatccattttagaattggatgtcaacatagtagaataggaattaccccatctaacgcaagaggaaga
ggaagaagagtgccccccctggcatcaccctggaagtcagcccccaaccccttgcaacgcttgctattgcaaaa
aatgctgttatcattgctatctttgtttcacaaagaagggtttgggaatctccatggggagtgcatggagtaa
aagtaaattttcaggatggtcagcagtaagagatagaatgagacaaacctcccctaatcctgagccmtgtgca
cctggggtaggagaaatttcyagacagttatcagaaagagggggataacaaattcttatactcctcaaaaca
atgcagcccttgcattcctcgaaagccaccaagaggaggaagaagtagggttcccagtaagacctcaagtgcc
tctaaggccaatgacctataaaggagcatttgacctcggcttctttttaaaagaaaagggaggactggatggg

ttaatttactcccatagaagaaaagagatcctggatctttgggtgtatcacactcagggattcttccctgatt
ggcagaactacacaccaggaccaggaaccagatacccattgacatttggatggccatttaaactagtaccagt
gacarmagaagaggcagaaagaatgggcaatcagtgtgaaagggctatgctcctacatccamtttgtaaccat
ggtagtgaggatgaagaaggggaaatactacaatggaagtttgatagttcattagcgcgcacccatgttgctt
taataactcacccagagctcttcaacaaggactaa
>#70 AY169805 1401 nt
tacaggtctaggaagaccaaggactggtattacagacatcattatgaatgtagaaatccaagaatcasttcwa
gtgtatatattccaatagggccggcttttatagtagtgaccacatattggggattgatgccaggagaaagrga
tgaacatatgggacatggggttagtatagaatggcagtacaagaagtatgcaacacagattgaccctgaaaca
gcagacaggatgatacatctgcactattttacctgttttacagaatcagcaatcaggaaagccatcctagggc
aaagagtactgaccaagtgtgaataccctgcaggacatagtcaggtagggacactacaactactagctctaag
agctgtagtgaaagaaaaaagacataagcctcccctacccagtgtccagaaattaacagaagatctcctagaa
gagctaaaagcagaagcagtaagacatttccctaggccttggctacagggcttgggacaatacatttatgaya
cttatggggacacttgggaaggggttatggcaattataagagtcttacaactactgatatttgcccattttag
aattggatgccaacatagtagaataggaattaacccatctaacacaagaggaagaggaagaagaatgccccct
tggcatcaccctggaagtcagcccctgaccccttgtactaagtgctattgcaaaaaatgctgctatcattgct
atgtttgtttcacaagcaagggtttgggaatctccatgggaaacgcattgaggaaaagcaaatttgcaggatg
gccagcagtaagagaaagaatgagaagggcttcccctaaacctgaagattgtgcacctggagtaggagaagtc
tccagggaattagcatctagaggaggaataccaagttcctatactcctcagaacaatgcagcccttgcatttc
tagagagccaccaagatgaagatgtaggtttcccagtaacaccccaagtgcctctaaggccaatgacctwtaa
aggagcatttgacctcagcttctttttaaaagaaaagggaggactggatgggttaatttattcccagcaaaga
gcagagatcctggatctttggatctatcacactcagggattcttccctgattggcagaactacacaccagggc
caggaactaggttcccactgacatttggatggttatttaagctagtaccagtgacagaagatgaggcaaaaag
actaggaaatgtgtgtgagagggctcatctcctgcatccagcttgtaaccacggctttgaagatccacacgga
gagatactgaaatggcagtttgacagatcactgggcaacacccatgttgctaagataacccacccagagttct
tccccaaggactaa
>#71 HQ179987 1413 nt
tatcacaaatataggtctaaaaagacaagaaagtggttttataggcatcattatgagacaacccatcctagga
ttagttcagcagtttatattccagtaggaacagcaaccattattgtgactacytattgggggctcatgcctgg
ggaaagagaagaacaattaggacatggagcaagtgtggagtggagacaaggtaaatacaccacacagatagat
ccagaaacagcagataggctaattcatctccactactttcaatgtttttcagattcggctgtgaggagggcaa
tactaggggacagggtattgaayamatgtgaatactcagcaggacatagtcaggtaggctccttgcagtattt
agccttaaaagtggtagtagggaaggtaaaraggaagccacccctccctagtgtccagatattgacacaagac
gaactcaaggaggaagcagtaagacacttccctaggccttggttacactcattaggacagtatatctataata
cctatggggacacctgggagggagtaactgcaattattaggatcctacaacaattaatytttatccattatag
aattggatgccaacatagtagaataggtatcttgacaccctctcgaagaggaaggagactgcctccatggcaa
cagccagggagtcagccctcaagcccatgtaacaattgctactgcaaagcctgctgctatcattgctatgttt
gtttcacaaagaagggtttgggaatctccatggggaatgcatggaagaaaagtagcttagtgggctggccagc
agtcagggaaaaaataaagcagactaccccgactacccctgacccgactaccccagtaacacctgcacccggg
gttgggggaaatttccaaagaattagcacaaggaaaaggaatacccagtaaatttagttcaaagaacaatgcag
cattggccttcttggatgctcatgaggaagaagaagtaggrttcccagtcaggcctcaagtacccttaagatg
catgacatacaaggcagcatttgacctcagcttctttttaaaagaaaagggaggactggatgggttagtttac
tcacctgagagagcagagatcctagatctctggatctatcacactcagggattcttccctgactggcagaatt
acactccagggccaggagaaagatatcccctgacctttgggtggctgtttaaactagtaccagtctctgaggt
agaagctgaggaaatgggagataagcaggagaaagctaagctgctacatccagcctgcacttatgggttttca
gatcctcataaggagatcctagtgtggaagtttgacagctcacttggaagagaacatgttgccttacaaaagc
acccggaactgtttattaaagactaa
>#72 GU111555 1395 nt
ctggtcaaatatcacaaatataggtctaaaaagacaaagaattggttttataggcatcattatgagacagtcc
atcctaggattagttcaggagtttacattccagtaggagcagcaaccattgttgtgactacttattgggggct
tatgcctggggaaagagaagaacaattaggacatggagcaagtgtggagtggagacaaggtaaatacaccacc
cagatagatccagaaacagcagataggctaattcatctccactactttcaatgtttttcagattcggctgtga
ggagggcaatactaggggacagggtattgaacaagtgtgaatactcagcaggacatagtcaggtaggctcctt
gcagtttttagccttaaaagtggtagtagggaaagtaaaaaggaagccacccctccctagtgtccagatattg
acacaagatgaactcaaggcggaagcagtaagacccttccctytgcaatacttacactcattaggacaataca
tctataatacctatggggacacctgggagggagtaactgcaattattaggatcctacmacaattaatttttat
ccattatagaattggatgtcaycatagtagaataggtatattgccaccctctcgaagaggaaggagactgcct
ccatggcagcagccagggagtcagccctcaagcccatgtaacaattgctactgcaaagcctgctgctatcatt
gctatgtttgtttcacaaagaagggtttggggatctccgggaatgcatggaagaaaagtagcttagtgggctg
gccagcagtcagagaaagaatgaatcagactgccccagaaacacctgcatctggggttggggagatttccaaa

gaattagcacaaggaaaaggaatacccagtaaatataattcaaagaacaatgcagcattagccttcttagaag
ctcatgaagatgaagaagtaggattcccagtcaggcctcaagtacccgtaagacccatgacatacaaggcagc
atttgacctcagcttcttttaaaagaaaagggggactggaagggttaatttattcacctgggagagcagaa
atactagatctctgggtctatcacactcaggggttcttccctgactggcagcattacactccagggccaggag
aaagattcccactgacctttgggtggctgtttaaactagtaccagtctctgaggtagaagttgaggaaatggg
agatgagcaggagaaagctaagctgctacatccagcctgcacttatggggattcagatcatcatagggagatc
ttaatgtggaagtttgacagatcactgggagaaagacatgttgccttacaaaagcacccggaactgtttacta
aagactaa
>#73 DQ017382 1407 nt
ttagtaaaacatcatatgcatgtgtcaaaaaaggcaaaaggatggtattatagacatcattatgaatcaaggc
atccaaaaacaagttcagaagtacatatcccagtaggtcaggcaacattagtgatagtcacttattggggatt
aacaacaggagaacagccttggcatctaggacatggagtatccatagaatggagactaagaaaatacaagaca
caggttgatcctgaaatggcagacaagctaatacatctttattattttgattgttttacagattctgccataa
ggcaagcggtcttagggagacaagtatttcctaggtgtgaatatcctgcagggcacaaccaggtagatacctt
acaatatctagcattaacagcttgggtgggaataaagaagagaaagccacccttacctagtgtggccaagtta
acagaagatttattagaagacttaaaaaatgaagctgtgcgccattttccaaggatttggctacatgggttag
ggcaacacatctatgacacttatggagacacctgggagggagtagaggcaattatcaggatgytacaacaatt
actgtttatccattataggattggttgccagcacagcagaatagggattactcctcaaaggagaagattagag
ccctggaatcatccaggaagtcaacccaaaacagcttgcaataagtgctattgtaaaaattgctgctatcatt
gtgtggtttgcttcacaaagaaaggcttaggcatctcaatggggaagagttggtcaaagagcagtgtagtagg
atggccagaaatcagagaaagaatgagaagacaaagacaaactcaagcagcagcagcagcagtaggagtagga
gcagcttctcaagacctagctaatcgagggcccatcacccacaagcaatactagagataataatgaaactgtag
cttgggtagaagcacaagaagaagaagaggaagtaggctttccagtacgccctcaagtaccattgaggccaat
gacctttaaagcagcttttgatctttccttcttttttaaaagaaaagggggactggaagggctagtttggtcc
agaaaaaggcaagagattctagacctctggatttataacacacaaggctacttccctgactggcagaactaca
caccagggccaggagtcaggtatcccctgacatttggatggtgcttcaaactagtaccattgtcagctgaagc
agtagaagaagctaatgaaggagacaacaatgccctcttacatcccatatgtcaacatggagtagatgatgat
cacaaagaagtgctggtgtggcggtttgacagctccctggcaagaagacacatagcaagagagctgcatccgg
acttctacaagaactgctga
>#74 AY532635 1410 nt
ttagtaaagcatcatatgtatgtgtcaagaaaggcagaagggtggtattatagacatcattatgaaacaaatc
atccaaaaataagttcagaagtacatatcccagtaggtctggcaaggttagtgataaccacttattggggact
aacaacaggagaaaggtcttggcatctaggacatggagtatccatagaatggaaactaggaaaatacaagaca
caagttgatcctgaaatggcagacaagctaatacatctttattattttaattgttttacagcctctgccataa
ggcaagcggtcttagggagaccagtattccctaggtgtgaatatccggcagggcacaatcaggtaggcacttt
gcaacatctagcactcacagcctgggtgggagtaaagaagagaaagccacccttacctagtgtgactaagtta
acagaagatttattagaagaattaaaaaatgaagctgtgcgccattttccaaggacttggctacatgggttag
ggcaacacatctataacacatatggagacacctgggaggggtagaggcaattattaggatactacaacaatt
actgtttatccattataggattggctgccagcatagcagaatagggattactcctcgggggagaaggttagag
ccctggaatcatccaggaagccaacccaagacagcttgcaatggatgctattgtaaatattgctgctatcact
gtatgtgttgcttcacaaagaaaggcttaggcatatcaatgggaaaaatttggtcaaagagcagcatagtagg
atggccagaaatcagagaaagaatgagaagacaacgacctcacgagccagcagtagagccagcagtaggagta
ggagcagcttctcaagatctagccaatcgagggccctcacccacaagcaatactagaaccaataatccaactg
tagcttgggtagaagcacaagaagaagaaggggaagtaggctttccagtacgccctcaggtaccattgaggcc
aatgacctataaagcggctgttgatctttccttcttttaaaagaaaagggggactggaagggctaatttgg
tccagaaaaaggcaagacgttttagatctctgggtttatcacacacaaggcttcttccctgactggcagaact
acacaccagggccaggaactagattcccctgacctttggatggtgtttcaaactagtaccattgtcagctga
agcagtagaagaggctaatgaaggagacaacaatgccctcttacatcccctatgccaacatggagtagatgat
ggacacaaagaagtgctgcagtggcagtttgacagctccctggcaagaagacacatagcaagagagctgcatc
cggacttttacaagaactgctga
>#75 AJ006022 1407 nt
ttagtaaaacatcatatgtatgtgtcaaaaaaggcaaaaggatggtattatagacatcattatgaaacacatc
acccaaaaataagttcagaagtacatatcccagtaggtcaggcaagattagtgacagtcacttattgggggct
aacaacaggagaacagtcttggcatctaggacatggagtatccatagaatggagactaagaaaatacaagaca
caagttgatcctgaaatggcagacaagctaatacatcttcattattttgattgttttacagcctctgccataa
ggcaagcggtcttagggagaccagtattacctaggtgtgaatatccagcagggcacaaacaggtaggcaccct
acaatatctagcactaacagcctgggtgggagcaaagaagagaaagccacccttacctagtgtgactaagcta
acagaagatttattagaagaattaaaaaatgaagctgtgcgccattttccaaggatttggctacatgggttag
gacaacacatctataacacatatggagacacctgggaggggtagaggcaattatcaggatactacaacaatt
actgtttatccattataggattggctgccagcacagcagaatagggatcactcctcaaaggagaaggttagag

ccctggaatcatccaggaagccaacctaaaacagcttgcaataattgctattgtaaaagatgttgctatcact
gcttatattgcttcacaaagaaaggcttaggcatctcaatgggaaagatttggtcaaagagcagcctagtagg
atggccagaaatcagagaaagaatgagaagacaaacgcaagaaccagcagtagagccagcagtaggagcagga
gcagcttctcaagatctagctaatcgaggggccatcaccataagaaatactagagacaataatgaaagtatag
cttggctagaagcacaagaagaagaagaggaagtaggctttccagtacgccctcaggtaccattaaggccaat
aacctataaacaggcttttgatctttccttcttttaaaagataaggggggactggaagggctagtttggtcc
agaaaaaggcaagatattctagacctctggatgtatcacacacaaggcatcctccctgactggcataactaca
caccagggccaggaattagataccccgtaacctttggatggtgcttcaaactagtaccattgtcagctgaaga
agtagaagaggctaatgaaggagacaacaatgccctcttacaccccatatgtcaacatggagcagatgatgat
cataaagaagtgttggtgtggcgatttgacagctccctagcaagaagacatgtagcaagagagctgcatccgg
agttttacaagaactgctga
>#76 MF7672 1407 nt
ttagtgaaacatcatatgtatgtgtcaaaaaaggcaaaagggtggttctatagacatcattatgaaacaaatc
atccaaaaataagttcagaagtacatatcccaatgggtgaggcgaaattagtgataatcacttattgggggct
aacaacaggagaacagtcttggcatctaggacatggagtatccatagaatggagacaaggaaaatacaggaca
caagttgatcctgaaatggcagacaagctaatacattgttattattttaattgttttacagcctctgccataa
ggcaagcggtcttagggagaccagtattacctaggtgtgaatatccagcagggcaccaacaggtaggcactct
acaatatctagcactaaaagcctgggtgggaataaaaaagagaaagccacccttacctagtgtttctaagcta
acagaagacttactagaagaattaaaaaatgaagctgtgcgccattttccaaggacttggttacatgggttag
gagaatacatctatcacacatatggagacacctgggaggggtagaggcaattatcaggatactacaacaatt
actgttcatccattataggattggctgtcagcacagcagaatagggattactgttcaaggaagaaggttagag
ccctggaagcatccaggaagccaacccaagacagcttgcaataattgctattgtaaaaaatgctgctatcact
gtgtgtgttgcttcacaaagaaaggcttaggcatctcaatggggaagagttggtcaaagagcagaataccagg
atggccagaaatcagagaaagaatgagraaacaaacgcaagaatcagcagtagagccagcagtaggagtagga
gcagcctctcaagacctagctaatcgaggagccctcaccaccagcaatactaaagataataatcaaactgtag
cttgggtagaagcacaagaagaagaggaggaggtaggctttccagtacgccctcaagtaccggtgaggcctat
gacctataaagcagcctttgacctctccttcttttaaaagaaaaggggggactggaagggctagtttggtcc
agaaaaaggcaagagattctggacctctgggtttatcacacacaaggcttcttccctgactggcagaactaca
caccagggccaggaatcagatacccnctgacctttggatggtgcttcaaactagtaccattgtcagatgaagc
agtagaagaagccactgaaggagacaacaatgccctcctgcatcccatatgtcaacatggagtagatgatgat
cacaaacaagtgctggtgtggaggtttgacagcgccctggcaagaagacacatagcaaaagagctgcatccgg
acttttacaagaactgctga
>#77 KY498771 1395 nt
ttagtaaagcatcatatgtatgtgtcaaaaaaggcaaaagggtggtattatagacatcattatgaaacaactc
atccaaaaataagttcagaagtacatatcccagtaggtcaggcaagattagtgataaccacttattggggact
gacaacaggagaacagccttggcatctaggacatggagtatccatagaatggagactaagaaaatacaggaca
caagttgatcctgaaatggcagatcagctaatacatttttattattttaattgttttacagcttctgccatca
ggcaagcgatcttagggagaccagtatcacctaggtgtgaatatccagcagggcataaacaggtaggcacctt
acaatatctagcactaacagcctgggtggaagtaaagaagagaaagccacccttacctagtgtgactaagtta
acagaagatttattagaggaattaaaaaatgaagctgtgcgccaytttccaaggatttggctacatgggttag
gacaatacatctacaacacttatggagacacatgggaggggtagaggcaattattaggatgctacaacaatt
actgtttatccattataggattggctgtcagcacagcagaatagggatcactcctcaaaggagaaggatagag
ccctggaatcatccaggaagccgacccaagacagcttgcaataattgctattgtaaaaaatgttgctatcact
gtgtggtttgcttcacaaagaaaggcttaggcatctcaatgggaaagagttggtcaaaaagcagcatagtagg
atggccagaaatcagagaaagaatgagaagacaaccagcagagccagcaataggagtaggagcagcttctcaa
gacctagctaatcgaggggcaatcaccacaagtaatactgcaagcaataatgaaactgtagcttggctagaag
cacaagaagaagaggaagaagtaggctttccagtacgccctcaggtgccattaaggccaatgaccatgaaagc
ggctgttgacctttccttcttttaaaagaaaaggggggactggaagggctaccttggtcccagagaaggcaa
gacattctagacctctgggtttataacacacaaggcttcttccctgattggcagaactacacaccagggccag
gagttagataccccctgacctttggatggtgcttcaaattagtgccaatgtcaactgaagaagtagaagaagc
taataaaggagaaaacaatgccctattgcatcccatatgtcaacatggagtagatgatgctcacaaagaagtg
ctggtgtggcagtttgacagctccctggcaagaagacacgtagcaagagagctgcatccggagttctacaagg
actgctga
>#78 DQ017383 1410 nt
ttagtaaaacatcatatgcatgtgtcaaaaaaggcaaaaggatggtattatagacatcattatgaaacaaggc
atccaaaaataagttcagaagtacatatcccagtaggtcaggcaagattagtgatagtcacttattggggact
aacaacaggagaacagccttggcatctaggacatggagtatccatagaatggagactaagaaaatacaagaca
caggttgatcctgaaatggcagacaagctaatacatctttattattttgattgttttacagcgtctgccataa
gacaagcaatcttagggagacaagtatttcctaggtgtgaatatcctgcagggcacaatcaggtaggcacctt
acaatatctagcattaacagcttgggtgggaataaagaagagaaaaccacccttacctagtgtggctaagtta

acagaagatttattagaagacttaaaaaatgaagctgtgcgccattttccaaggatttggctacatggattag
ggcaacacatctatgacacatatggagacacatgggaaggagtagaggcaattatcaggatgctacaacaatt
actgtttatccattataggattggttgccagcacagcagaatagggattactcctcaaaggagaagattagag
ccctggaatcatccaggaagccaacccaaaacagcttgcaataattgctattgtaaaaaatgctgctatcatt
gtgtggtttgcttcacaaagaaaggcttaggcatctcaatggggaaaattttgtcaaagaacagagtagcagg
atggtcagaaatcagagaaagaatgagaaggcaaactcaagagccagctcaagagccagcagcagtaggagta
ggagcagcttctcaagacctagctaatcgaggggccatcaccacaagcaatactagagataataatgaaactg
taacttggctagaagcacaggaagaggaagaggaagtaggctttccagtacgccctcaagtaccaataaggcc
aatgacctataaagcagcttttgatctttccttcttttaaaagaaaaggggggactggaagggctagtttgg
tccagaaagaggcaagagattctagacctctgggtttatcacacacaaggcttcttccctgactggcagaact
acacaccagggccaggagttagatacccccctgacctttggatggtgcttcaaactagtaccattgtcagctga
agcagtagaagaagccaatgaaggagacaacaatgccctcctgcaccccatatgtcaacatggagtagatgat
gagcacaaagaagtgctggtgtggcggtttgacagctccctggcaagaagacacttagcaagagagctgcatc
cggacttttacaagaactgctga
>#79 AJ271370 1392 nt
ttagtaaaacaccatatgtatgtgtcaaaaaaggcaaaagggtggtattatagacatcattatgaaacaaagc
atccaaagacaagttcagaagtrcatatcccagtaggtcmggcaarattagtgatagtcacttattggggact
aacaacaggagaacagccttggcatctaggacatrgagtatccatagaatggagacaaggaaaatacaagaca
caagttgatcctgaaatggcagacaagctaatacattgttattattttaattgttttacagcttctgccataa
ggcaagcggtcttagggagaccagtgttacctaggtgtgamtatccggcagggcacaamcaggtaggcactct
acaatatctagcamtaacagcctrggtgggagtaaagaagagaaggccacccttacctagtgtgactaagtta
acagaagatttattagaagaattaaaaaatgaagctgtgcgccattttccaaggatttggctacatgggttag
gacaacacatctataacacatatggagacacctgggaggggggtagaggcaattatcaggatactacaacaatt
actgtttatccattataggattggctgccagcacagcagaatagggattactccccaaaggagaaggttagag
ccctggaatcatccaggaagccaacccaagacagcttgcaataagtgctattgtaaaaaatgctgctatcact
gtatgtgttgcttcacaaagaaaggcttaggcatctcaatgggaaaaatttggtcaaagagcagcctagtagg
atggccagaaatcagagaaagaataagaagacaaactccagagccagcagtaggagtaggagcagtttctcaa
gacctagctaatcgaggggccatcaccacaagcaatactaaagataataatcaaactgtagcttggctagaag
cacaagaagaasaggaggtaggctttccagtacgccctcaagtaccgctgaggccaatgacctataaagcggc
ttttgatctttccttcttttaaaagaaaaggggggactggaagggctagtttggtccagaaaaaggcaagaa
attctagacctctgggtttatcacacacaaggtttcttccctgactggcagaactacacaccagggccaggag
ttagataccccytgtgctttggatggtgcttcaaactagtaccattgtcagaggaagcagtagaagaagctaa
tgaaggagacaataatgccctcctgcatcccatatgtcaacatggagtagatgatgatcacaaacaagtgctg
gtgtggcggtttgacagctccctggcaagaagacacgtagcaaaagagctgcatccggacttctacaagaact
gctga
>#80 DQ314732 1386 nt
ctagtaaaacatcatatgtatatctcaaagaaagctaaaaagtggttttatagacatcattatgaaagtcagc
atccaaaggtaagttcagaagtacatatcccactaggagaggctacattagtaataagaacatattggggtct
gcagacaggagaaaaggactggcaattgggtcatggagtctccatagaatggaggcaaagaaaatatagcaca
cagatagatcctgacctagcagaccaactgattcatctacattattttgactgtttttcagaatctgccataa
ggaaagccatattaggacaagtagttagacgtaggtgtgaatacccatcaggacataacaaggtaggatccct
acaatatttggcactgaaagcattaacaacaccaaaaaggataaagccacctctgcctagtgttaagaaatta
acagaagatctgttagaggagcttaaaaacgaagctgttagacattttcctaggccttggcttcatggcctag
gacagcacatctatgacacttatggggatacttgggaaggggttgaagctataataagaattttgcaacaact
actgtttgttcatttcagaattgggtgtcaacatagcagaataggcattatacgagggagaagaggcaggcta
gagccctggaatcatccgggaagtcagcctacaactgcttgtagcacgtgttactgtaaaatatgttgctggc
attgccaactatgctttctaaaaaaaggcttaggcatctccatgggaggcaagtggtcaaaaagtagcatagt
gggatggcctcagatcagggagaaaataaagcaaactcctccagcagcagaaggagtaggtgcagtatctcaa
gatctagataaacatggagcagtaacaagtagtaatatgaataatgctgattgggactggctgaaagcacaag
aggaagaggaggtaggctttccagtcaggccacaggtacctctaagaccaatgacatttaagggagctttcga
tctaagcttcttttaaaagaaaaggggggactggagggctaatttactccaagagaagacaagagatcctt
gacttgtgggtctatcatacacaaggtttcttccctgattggcacaactacacaccagggccagggatcagat
atccactatgttttggatggtgcttcaagctagtaccagttgacccaagagaagtagaggaggacaacaaagg
agaaaacagcagcctgttacaccccataagccagcatggaatagaggacgaagaaagagaagtgctgatgtgg
aagtttgatagtgccctagcacgaagacacatagcccgagaaatgcacccagagttctataaaaactgctga
>#81 AF377956 1404 nt
acatggaacagtttagtaaaacatcatatgtatgtttcaaggagagctaaaggatggttttatagacatcact
atgaaagcaggcatccaagagtaagctcagaagtacacatcccactaaaggatgattctaaattagtaatagt
aagctattggggtctacatacaggagaaagagattggcatttgggtcaaggagtctccatagaatggaggcag
aaaagatataggacacaagtagaccctggcttggcagaccaattaattcatttgtattactttgattgtttt

cagaatctgccataaggaaagccatattaggacagagagttagtcctaggtgtaactatcaagcaggacataa
caaggtaggatccctacaatatttggcactaacagcattaataaccccaaagaagataaagccgcctttgcct
agtgtcaggaaactagtagaggatcttttagaggagcttaagcatgaagctgttagacatttccctagggagt
ggctccatggcttaggacagcatatctacaacacctatggggatacttgggagggagttcaagctataataag
aacactgcaacaactactatttatccatttcagaattgggtgtcatcatagcagaataggcattattcgacaa
agaagactaagactagagccctggaatcatccaggaagtcagcctgagactccttgtaataaatgttactgta
aaaagtgttgctttcattgccaattgtgctttacaaggaagggcttaggcatctccatgggtggcaagtggtc
aaaaagtagtaaagttggatggcctactataagggaaagaataagacaaacccctgcagcagcaccaggggtg
ggagcagtgtctcaagacttagataaacatggggcaattacaagcagccagactagggatactaatagtgact
tggcatggctagaagcgcaagaggatgaggaggtaggtttcccagtcagacctcaggtacctttaagaccaat
gacttataagggagctttcgatctcagtttctttttaaaagaaaagggggactggaagggttaatttactcc
aagagaagacaagaaatccttgatctgtgggtctaccacacacaaggctacttccctgattggcagaactaca
caccagggccagggactagatatccactgacctttggatggtgcttcaagctagtaccagttgagccagagga
ggtagaaaaggccaatgaaggagagaacaactgtttattgcaccctatgagccaacatggaatggaggacgaa
gacagggaagtgttaaagtggaagtttgacagctccctagcacggagacacagagccagagagatgcatccgg
agtactacaaagactga
>#82 AB254156 1425 nt
acatggaatagtttagtaaagcaccatatgtatgtttcaaagagagctaagggatggttctacagacatcatt
ttgaaagcagacatccaagagtaagttcagaagtacacatcccattaggggatgctagattaatagtaaagac
atattggggtttgcagacaggagaaagagattggcatttgggtcatggagtctccatagaatggagattgaga
aggtatagcacacaagtagaccctagcctggcagaccaactaattcatatgcattattttgattgttttgcag
actctgccataaggaaagccatattaggacatatagttagccctagatgtgactatcaagcaggacataacaa
ggtaggatctttacaatacttggcactgacagcattgataaaaccaaaaaagagaaagccacctctgcctagt
gttagcaaattggtagaggatcttttagaagagctcaagcaggaagctgtcagacactttcctagaccatggc
ttcatggcttaggacagcatatctatgagacctatggggatacatggacgggagttagagtgataataagaat
cctgcaacaattactgtttactcatttcaggattgggtgccagcatagcagaataggcattttgcaacagaga
agagcaagactagagccctggaagcatccaggaagtcagcctagaaccgcttgtaattcatgttactgcaaac
gctgcagctatcattgtccagtttgctttctgacaaaaggcttaggcatttccatggggaacaagtggtcaaa
aggcagtatagttggatggcctgctgtaagagaaagattaagacgagccagaccagcagcagagggagtagga
acagcagcagagggagtaggaacagcatctcaagacttagataaacatggggcacttacaaccagcaacacag
ccaccaacaatgctgattgtgcctggctggaagcacaagaggaagaagaagtaggctttccagtcagacctca
ggtacctttaagaccaataacttataaggcagcattcgatctcagcttctttttaaaagaaaagggggactg
gaagggttaatttactccaagaaaaggcatgagatccttgatttatgggtctatcacacacaaggcttcttcc
ctgattggcaaaactacacaccgggaccaggggtcagatatccactgacctttggatggtgcttcaagctagt
gccagttgacccaagtgaagtagaagaggccaatgaaggagagaacaactgtttgctacatcctataagccag
catggaatagaggatgaagacagagaagtcttaaagtggcagtttgacagcagcctagcacgcagacacctgg
cccgcgagctacatccggagtactacaaaaactgctga
>#83 MF373132 1407 nt
acatggaatagtttagtaaagcaccacatgtatatctcaaagagaaccagggggatggttttatagacatcatt
ttgaaagcagacatccaaaaataagttcagaagtacacatcccattaggggaggctaaattagtaataataac
atattggggtttgcaaacaggagaaagagattggcacttgggtcatggagtctccatagaatggagactgaga
agatatagcacacaagtagaccctagcctggcagaccaactaattcatatgcactattttgattgttttgcag
actctgccataaggaaagccatattaggacatatagttagccctaggtgtgactatcaagcaggacataataa
ggtaggatctctacaatatctggcactgacagcattgataaaaccaaaaaggattaagccacctctgcctagt
gttaagaaattagtagaggatcttttagaggaactcaagcaggaagctgtcagacactttcctagaccatggc
tccatagcttaggacaacatatctatgaaacttatggggatacttggatgggagttgaagccataataagaat
tttgcaacaattactgtttatccatttcagaattgggtgtatgcatagccgaataggcattatgcgacaaaga
agaacaagactagagccctggaaccatccaggaagtcagcctaaaactccttgcaataattgctattgtaaat
actgtagctatcattgtctagtttgctttcagacaaaaggcttaggcatttccatgggggcaaatggtcaaa
atgtagtccagtaggatggtctgagataagagacagaataaaaagaactcccccggcagcagagggagtagga
gcagcatcccgagacttagagagacatggggcactgacaaccagcaacacacccagcaataatgctacttgtg
cctggctggaagcgcaagaggaggaaggagatgtaggctttccagtcagacctcaggtacctttaagaccaat
gacttataaggcagcattcgatctcagcttctttttaaaagaaaagggggactggaagggttaatttactct
aagcaaaggcaagacatccttgatttgtgggtctatcacacacaaggtttcttccctgattggcaaaactaca
caccaggaccaggggtcagatatccactgacctttggatggtgcttcaagctagtgccagttgacccaaagga
agtggaagaagacaataaagatgagaacaacagcttgctacaccctgtgagcctgcatggagtggaggatgaa
gacaaagaagtactaatgtggaagtttgacagtcacctagcacgcagacacatggcccgcgagctacatccgg
agtattacaaagactgctga
>#84 KP109483 1407 nt

acatggaatagtttagtaaaacaccatatgtatatttcaaagagagctaagggatggttttacagacatcatt
atgacagcagacatccaaaagtaagttcagaagtacacatccccttaggggatgctagactagtaataaaaac
atattggggggttgcaaacaggagaaagagattggcacttgggccatggagtatccatagaatggagattgaga
ggatatagtacacaagtagaacctggcctggcagaccagctaattcatatgcattattttgattgttttgcag
actctgccataagaaaagccatattaggacacatagttattcctaggtgtgactatccagcaggacataataa
ggtaggatctctacaatacttggcactgacagcactgataaaaccaaaaaagataaggccacctctgcctagt
attaggaaattagtagaggatcttctagaggaactcaagcaggaagctgtcagacactttcctagaccatggc
ttcatggcttaggacaatatgtctatgagacctatggggatacttggacaggagttgaaactatggtgagact
actgcaacaattactgtttattcatttcagaattgggtgccagcatagcagaataggcattttgcgacagaga
agagcaagactagagccctggaaccatccaggaagtcagcctaaaactgcttgcaataactgttattgtaaaa
aatgtagctaccactgtctagtttgctttcagaaaaaaggcttaggcatttccatgggggcaagtggtcaaa
acgcagcatagttggatggcctaatataagagagagaatgagacgagctgagccagcagcagaaggagtagga
gcagcatctcaagacttagataaacatggagcgcttaccagcagcaacacagacaccaataatgctgattgtg
cttggctgagagcgcaagaggaggaagaagatgtaggctttccagtcagacctcaggtgcctttaagaccaat
gacttttaagggagcatttgatctcagcttctttttaaaagaaaagggggactggaagggttaatttactct
aagaaaaggcaagagatccttgatttgtgggtctatcacacacaaggctacttccctgactggcaaaactaca
caccaggaccaggggtcagatacccactgacctttgggtggtgcttcaagctgctaccaattgacccaaggga
agtagaagaggccaacaaagaagaagacaaccgcttgctacaccctatgtgccagcatggaatggaggatgaa
cacagagaagtattaaagtggaagtttgacagtcaactagcacttaaacacagggcccgcgagctacatccgg
agttttacaaagactgctga
>#85 KY658708 1422 nt
acatggaatagtttagtaaagcaccatatgcatatttcaaaaagagctaagggatggttttacagacatcatt
atgaaagcagacatccaaaagtaagttcagaagtacacatcccattaggggaggctagattagtaataaaaac
atattggggtttgcaaacaggagaaagagattggcatttgggtcatggagtctccatagaatggagattgaga
aaatatagcacacaagtagaacctggcctggcagaccagctaatccatatgcactattttgattgttttgcag
actctgccataagaaaagccatattaggagaaatagttattcctaggtgtgactatcaagcaggacataatca
ggtaggatctctacaatacttggcactgacagcattgataaaaccaaaacagagaaagccacctctgcctagt
gttaggaaattagtagaggatcttctagaggaaatcaagcaggaagctgtcagacactttcctagaccatggc
ttcatggcttaggacaatatgtctatgaaacctatggggatacttggacaggagtagaaactctaataagaat
actgcaacaactactgtttattcatttcagaattgggtgccaacatagcagaataggcattttgcgacagaga
agagcaagaatggagccagtagatcctaacctagagccctggaaccatccaggaagtcagcctagaactgctt
gtactaaatgctattgtaaatactgttgctaccattgcctagtttgctttcagacaaaaggcttaggcatttc
catgggggcaagtggtcaaaatgcagtggatggcctgctgtaagagaaagaatgagaagaactgagccagca
gcagagggagtaggagcagcatctcaagacttagataaatatggagcacttacaagcagcaacacaagcacca
ataatgctgattgtgcctggctggaagcgcaagaggaggaaggagatgtaggctttccagtcagacctcaggt
gcctttaagaccaatgacttataaaggagcattcgatctaggcttctttttaaaagaaaagggggactggaa
gggttagtttactccaagaaaagacaggaaatccttgatttgtgggtctatcacacacaaggcttcttccctg
actggcaaaactacacaccgggaccaggggtcagatatccactgacctttggatggtgcttcaagctggtgcc
agttgacccaggggaagtggaagaggccaacaagggagaagacaactgtttgctacaccctatgagccagcat
ggaatggaggatgaacacggagaagtattaaagtggaagtttgacagtcacctagcacgcagacacatggccc
gcgagctgtatccggagtattacaaagactgctga
>#86 KC156114 1413 nt
acatggaatagtttagtaaagcaccatatatgggcctcaaagaaagctagtggatggttttacagacatcatt
atgaaagcagacatccaaaagtaagttcagaagtacacatcccattaggggatgctagattaataataacaac
atattggggtttgcaaacaggagaaagagattggcatttgggtcatggagtctccatagaatggagattgaaa
agatatagcacacaagtagaccctggcctggcagaccagctaattcatatgcattattttgattgttttgcag
actctgccataagaaatgccatattaggacacatagtttttcctaggtgtgactatcaagcaggacataataa
ggtaggatctctacaatatttggcactgatagcattgataaaaccaaaaaggataaagcctcctctgcctagt
gttaggaaattagtagaggatcttttagaggaactcaagcaggaagctgtcagacactttcctagaccatggc
tccataacttaggacaacatatctatgaaacctatggggatacttggacaggagtcgaagtgataataagaat
tctgcaacaactactgtttatccatttcagaattgggtgccagcatagcagaataggcatcttgagacagaga
agagcaagactagatccctggaaccatccaggaagtcagcctaagactgcttgtaataattgttattgtaaag
gctgcagctatcattgtctagtttgctttcagaaaaaaggcttaggcatttccatgggggcaagtggtcaaa
aagcagtatagttggatggcctgctgtaagagaaagaataagacaagctaggccagagccagcagcagaggga
gtaggagcagcgtctcaagacctagataaacatggggcacttacaaccagcaacacagtttataacaatgctg
cttgtgcctggatagaagcgcaagaggagggagaagaggtaggctttccagtcaaaccccaggtgcctttaag
accaatgacttataaaggagcattcgatctcagcttctttttaaaagaaaagggggactggatgggttaatt
tactcccagcaaagacaggatatccttgatttgtgggtctataacacacaaggctacttccctgattggcaaa
actacacaccaggaccagggatcagatatccactgacctttggatggtgctacaagttagtgccagttgaccc
aaggacagtagaagaggccaacagtggagaaaacagctgcttgctccaccctatgagccagcatggaatggag

gatgcagacagagaagtattaatatggaagtttgacagcagcctagcacgcagacacctggcccgcgagctac
atccggagtattacaaagattgctga
>#87 AF005496 1404 nt
acatggaaaagcttagtaaagtaccatatgcatatttcaaggaaagctagaggatggttttatagacatcatt
ttgaaagcactcatccaaggataagttcagaagtacacatcccattaggagaagctaggttagtcataaccac
atactggggtctgaatacaggagaaagagaatggcatttaggccagggagtctccatagaatggagactgaaa
aggtatagcacacaagtagagcctggcctggcagaccaactaattcatatgcattattttgattgtttttcag
aatctgccataaggaaagccatattaggacgtgtagttagacctaggtgtaactatccagcaggacataaaca
ggtaggaactctacaatacttggcattaacagcattagtggcaccaaaaaagataaagccacctttgcctagt
gttagaaagctagtagaggatcttttagaggagattaagaatgaggctgttaggcattttcctagagtatggc
tccatcaattaggacagcatatctataacacctatggagatacttgggtaggagttgaagctttaataagaac
gctgcaacaactactgtttattcatttcagaattgggtgccaacatagcagaataggaattactcgacagaga
agagtaagactagagccctggaaccatccaggaagtcagcctcaaactgcttgtaacaattgttattgtaaaa
agtgctgctatcattgccaaatgtgctttttaaagaaaggcttaggaatttccatgggaggcaaatggtcaaa
aagtaggatgggtgggtggtctactataagggaaagaatgaggcgagctgaaccagtagcagaaggggtagga
gcagtgtctcgagatttggatagacgcggggcagtcacaattaataatacagcatctactaatcgtgatgccg
cctggctggaagcacaagaggacggggaggaagtaggctttccagtcaggcctcaggtacctttaagaccaat
gacctataagggagcttttgatctcagccatttttttaaaagaaaaggggggactggatgggttaatttactcc
aagcaaagacaggacatccttgatttatgggtctataacacacaaggctacttccctgactggcagaactaca
caccagggccaggggagagatttcccctgacctttgggtggtgcttcaagctagtaccagtaaatccacagga
ggtagaacaggccaatgaaggagagaacaacagcttgctacaccccatgagcctgcatggaatggaggatgac
gggagagaagtgctgatgtggaaatttgacagtcgactagcattgacacacttggcccgagtaaagcatccgg
agtacaaagactgctga
>#88 FJ389367 1407 nt
acatggaacagyttagtraaacatcatatgtatgtctcaaagaaagctaaaggctggttttatagacatcact
atgaaagcaggcatccaaaagtaagttcagaagtacacatcccaataggagatgctaaaatagtagtaagaac
atattggggtctgcatacaggagaaaaagattggcacctaggtaatggggtctccatagaatggaggcagaga
agatatagcacacagatagatcctgacctagcagaccaactgattcatctacattattttaactgtttttcag
actctgccataaggaaagccatattaggagaaatagttagacctaggtgtgaatatcaagcaggacataataa
ggtaggatccctacaatatttggcactgaaagcattagtaacctcaccaagggcgaagccacctttgcctagt
gttaggaaattaacagaagatctgttagaagaacttaaggctgaagctgttagacattttcctaggccctggc
ttcatggcttaggacagcatatctataacacttatggggatacttgggaaggagttgaagccataataagaat
actgcaacaactattgtttgtccatttcagaattgggtgccaacatagcagaataggcattgttccacaaaga
caaagaagagtaaggctagagccctggaatcatccggggagtcagcctaaaactgcttgtaccaaatgctatt
gtaaagcgtgttgctggcattgccaagtttgctttctgaacaaaggcttaggcatctccatgggaggcaagtg
gtcaaaaggatgcatggccggatggcctgaggtaagggaaagaatgagacaaaccactccagcagaaggagta
ggagcagcatctcaagatttagctaggcatggagcgatcacaagtagcaatacaccatccactaatgctgctt
gtgcctggctagaagcacaagaggaagactcagaggtaggctttccagtcagaccacaggtacctctgagacc
aatgacttttaagggcgcttttgatctcagcttctttttaaaagaaaaggggggactggatggctaatttac
tccaagaaaagacaagatatccttgacctgtgggtctataatacacaaggattcttcccagattggcaggact
acacaccagggccagggactagactcccactgacctttgggtggtgcttcaaactagtaccactggaaccaac
agagatagaggaagccaataaaggagagaacaacagtttattacaccccatctgccagcatggaatggaggac
gaggacagagaagtgctagtatggagatttgacagtagcctagcacggagacacttggcccgagagctgcatc
cggacttctacaaagactga
>#89 FJ389365 1413 nt
rcatggaacagtttartaaaacatcatatryatatctcaaagaargctaaagrytggtcttatagacaccact
atgaaagcaggcatccaagagtaagttcagaagtacacattccactaggagaagctaaaatagtagtaagaac
atattggggtctgcatacaggagaaaaagactggcarttgggtcatggggtctccatagaatggaggcaggga
aggtatagcacacaaatagatcctgacctagcagaccaaytgattcatctgcagtattttgattgttttacag
actctgcmataagacaagccatattaggacaaagagttagacctaggtgtgaatatcaagcaggacataataa
ggtaggatctytacaatatytggcactgcaagcattagtaaaaycaaraaagagaaggccacctttgcctagt
gttaggaaattaacagaagatgaacttaaaaatgaagctgttagacattttcctagaccctggctccatggct
taggacaatatatctataacacttatggagatacttgggaaggagttgaagccataataagaatgctacaaca
cttgctgtttatccatttcagaattgggtgccaacatagcagaataggcattagtcctctacggagaagagta
aggctagagccctggaatcatccrgggagtcagcctaaaactgcttgtaacaactgctattgtaaagtgtgct
gctggcattgtcaagtttgctttctgaacaaaggcttaggcatctccatgggatgcaagatgtcaaaagaatg
gtccagagtaagggaaaggatgagacgaaccccccaacagcagatggagtaggagcagaagcagcagcagat
ggagtaggagcagcatctcaggatttagctaggcatggagcactcacaagcagcaatacagcatccaccaatg
ctgcttgtgcctggctagaagcacaacaggaggactcagaggtaggctttccagtcagaccacaggtaccttt
gagaccaatgacctttaaaggtgcttttgatctcagcttctttttaaaagaaaaggggggactggatgggcta

atttactccaagcaaagacaagatatccttgacctgtgggtctataatacacaaggatacttcccagattggc
agaattacacttcagggccaggaattaggtacccactgacctttgggtggtgcttcaaactagtaccaatgga
tccagcagaggtagaggaagccaatagaggagagaacaacagtctattacaccccatctgccagcatggaatg
gaagatgaagacagaaccgtgctggtatggaagtttgacagtggcctagcacggagacacttagcccgagagc
tgcatccggagtattataaagactga
>#90 MH705151 1512 nt
acatggaacagtttagtaaaacatcatatgtatgtttcaaagaaagctcaaggttggttttatagacatcact
atgaaagcaggcatccaaaaataagttcagaagtacacatcccactaggtgatgctaggatagtggtaagaac
atattggggtctgcatacaggagaaagagactggcacttgggtcatggggcctccatagaatggaggcagaga
agatatagcacacaaatagatcctgacctagcagaccaattaattcatctgcattattttgactgttttgcag
actctgccataagaaaagccatcttaggagaaatagttagacctaggtgtgaatatcaagcaggacataacca
ggtaggatctctacaatatttggcactgaaagcattagtaaaaccaaaaagggcaaagccacctttgcctagt
gttaagaaattaacagaagatctgttagaagagctgaagcatgaagctgttagacatttccctaggccgtggc
tccatggattaggacaacatatctataacacctatggggatacttgggaaggggttgaagctataataagaat
gttgcaacaactactgtttgttcatttcagaattgggtgccaacatagcagaataggcattgttcgagggaga
agagtcaggctagagccctggaatcatccgggaagtcagcctaaaactgcttgtaataaatgtcattgtaaaa
agtgttgctatcattgccaagcttgctttctcaagaaaggcttaggcatctcctatggcaggaagaagcggag
accccgacgaagaactcctcagggcagtaaggatcatcaaaatcctatacaaaagatgggtggcaagtggtca
aaaagcagcatagtaggatggcctgatgttagggaaagaatgagacgatgtcctgaagttagggaaagaatga
gacgagctcctccagcagcagaaggagtaggagcagtgtctcaagatttagataagcatggagcaatcacaag
caggaatacagcaactaccaatgctagttgtgcctggctggaagcacaagaggaagaaggggaggtaggcttt
ccagtcaggccacaggtacctttaagaccaatgacttataaggcagctgtggatctcagccacttttaaaag
aaaagggggactggatgggttaatttggtcccagaaaagacaagacatccttgatctgtgggtctacaacac
acaaggcttcttcccagattggcagaattacacaccagggccagggcctagattcccaataacatttggatgg
tgctttaagctggtaccagttgatccagctgaagtagaagaggctactgaaggagagaacaacagcttattac
accctatatgccaacatggagcggaggacactgagagagaagtattaaagtggaagtttgacagtcgcctggc
attaaaacacagagctcaagagctgcatccggagttctacaaagactgctga
>#91 KF716478 1425 nt
acatggcacagtttagtaaaacatcatatgtatgtctcaagaaaagctaaagattggcattatagacatcact
atgaaagtaggcatccaaaagtaagttcagaagtgcacatcccactaggggaggctagaatagtagtaagaac
atattggggtctgcatacaggagaaaaggactggcaattgggccatggggtctccatagaatggagactaaaa
agatatagcacacaaatagatcctgacctggcagatcaactaattcatctgcattatttaactgtttttcag
aatctgccataaggagagccatattaggacaagtagttagccctagttgtgaataccaagcaggacataacaa
ggtaggatccctgcaatatttggcactgaaagcactagtaacaccaacaaggacaaagccacctttgcctagt
gttaagaaactaacagaagatctgttagaagagcttaagcatgaagctgttagacattttcctaggccatggc
tccatggactaggacaacatatctatgaaacctatggggacacttgggaaggagttgaagctataataagaat
tttacaacaattactgtttgttcatttcagaatcgggtgccaacatagcagaataggcattaacattcgcagg
agacgaggcaggctagagccctggaaccatccaggaagtcagcctgcaaccgcttgtaataagtgttactgta
aaaagtgttgctttcactgtcaagcgtgctttctgaacaaaggcttaggcatctccatgggtagcaagtggtc
aaaaagcagcatagtgggatggcctcaggttagggaaagaatgagacgagctcctccacaagctcctccaact
cctccagcagcaaaaggagtaggagcagtatctcaagatctagagaaacatggagcaatcacaagcagtaata
taaatcacccgagttgcacctggctggaagcgcaagaggaagaggaggaggtaggctttccagtcaggccaca
agtacctttaaggccaatgacttacaagggagctcttgacctcagccactttttaaaagaaaagggggggactg
gatgggctagtttactccaagaagagacaagagatccttgatctgtgggtatacaacacacaaggctatttcc
ctgattggcagaattacacaccagggccaggggtcagatacccactaacatttggatggtgtttcaagctagt
accagtagatccagaggaagtagagaaggctaatgagggagagaacaatagcctgttacaccctgtgtgccaa
catggaatggatgatgaagagagagaaacattgatatggagatttgacagccacctggcttttaaacacagag
cccaagagttacatccggagtactacaagaactgctga
>#92 AJ320484 1404 nt
acttggaaaagtttagtaaaacaccatatgtatgtttcaaagaaagctcaaggatggttgtatagacatcact
atgacagccctcacccaaaaataagttcagaagtacacatcccattaggagacgctagactggtagtaaaaac
atattggggcctgcatacaggagaaagagaatggcatctgggtcaaggagtctccatagaatggagaaaagg
agatatagcacacaagtagaccctggcctagcagaccaactaattcatatgtattattttgattgttttgcag
aatctgctataagaaaagccatattaggacaattagttagtcctaggtgtgaatatcaagcaggacataacaa
ggtaggatccttacagtatttggcactaacagcattaataaaaccagaaaaaacaaagccacctttgcctagt
gttaggaagctaacagaagatcttttagaggaacttaagagtgaggctgttagacattttcctaggatatggc
tccatggattagggcaatatatctatgaaacttatggggatacatgggcaggagttgaagctctaataagaac
cctgcaacaactactgtttattcatttcagaattgggtgtcaacatagcagaataggcattattcaacagaga
agaccaagattagagccctggaaccatccaggatgtcagcctaggacttcttgtaacaattgctattgtaaaa
agtgttgctatcattgccaaaaatgcttcttaacgaaaggcttaggcatctcaatgggtggcaaatggtcaaa

aagtaaaattgggtggcctgctgtaagggacagaatacaaagaactagtccagcagcagaaggggtgggagca
gcatctcgagacctggaaaaacatggggcaatcacaagtagcaatacagcagagactaatcctgactgtgcct
ggctagaagcacaagaagaggaagaggaggtgggttttccagttagacctcaggtaccgttaagaccaatggc
ttacaaggcagcttttgatctaagccacttttttaaaagaaaaggggggactggaagggttaatttactccaag
aaaagacaagaaatccttgatctttgggtctaccacacacaaggctatttccctgattggcaaaactacacac
cagggccagggattagatacccactgacctttggttggtgcttcgagctagtaccagtggatccagaggaggt
agaagaggccaataaaggagagaacaactgcttgttacaccctatgagccagcatggaatggaggacccggag
agagaggtgttagtgtggagatttaacagcagactagcatttgaacacaaggcccgaatacagcatccggagt
tctacaaagactgctga
>#93 KT200357 1407 nt
acatggaaaagtttagtaaaacaccatatacatgtttcaaagaaagctaagaaatgggtttatagacatcact
atgaaagcattaatccaaaaataagttcagaagtacacatcccactaggggatgatacattagtagtaacaac
atattggggtctgaacacaggagaaagagaatggcatttgggccagggagtctccatagagtggaggaaaagg
agatatagaacacaagtagaccctgacctagcagaccaactaattcatctgtattattttgattgtttttcag
aatctgctataagacatgccatattaggatatagagttagccctaggtgtgaatttcaagcaggacataataa
ggtaggatccttacagtacttggcactagtagcattaataacaccaaaaaggacaaaaccacctgtgcccagt
gttagcaaactaacagaggatcttttagaagaacttaagagagaagctgttagacattttcctaggacatggc
tccatgacttaggacaatatatctatgaaacttatggggatacctggactggagtggaagccataataagaat
attacaacaactactgtttattcatttcagaattggatgtcatcatagcagaataggcattattcgacagaga
agagcaaggctagagccctggaaacatccaggaagtcagcctaagactccttgtaccaaatgctattgtaaaa
agtgttgctttcattgccaagtttgtttcataacaaaaggcttaggcatctccatgggtggcaagtggtcaaa
acttagtaggggatgggaggttgtaagggaaagaatgagacgaaccacaccagtagaaccagcagcaagaggg
gtgggagcagtatctcaagatttggaaaggcatggagcaatcacaagtagcaatacagcagctactaatcctg
attgtgcctggctagaagcacaggaggaagaggacgtgggttttccagtcagacctcaggtacctttaagacc
aatgacttacaaaggagcacttgatcttagccacttttttaaaagaaaaggggggactggaagggctaattcac
tcccagagaagacaagatatccttgatctgtggatctaccacacacaaggctacttccctgattggcaaaact
acacaccagggccaggggtcagatttccactgacctttggatggtgtttcaagctagtaccagttgatccaga
tcaggtagaaaaggccaatgaaggagagaacagctgcctcttacaccccatgggcctgcatggaatggatgac
ccagagaaagaagtgttaatgtggaggtttgacagccgcctagcatttcatcatgtggcccgagagctgcatc
cggagtactttaaaaactga
>#94 AY331292 1404 nt
acatggaaaagtttagtaaaataccatatgtatagatcaaagaaagctaggggatggttttatagacatcatt
atgacagcactcatccaaaaataagttcagaagtacacattccactagggaatgcgagattggtaataacaac
atattggggtctgcatacaggagaaagagactggcatttgggtcagggagtctccatagaatggagggaaagg
aaatatagaacacaagtaacccctgacctagcagaccgactaattcatctgtattactttgattgtttttcag
aatctgctataagaaaggccatattaggacatatagttagtcctagttgtgaatatcaagcaggacataacaa
ggtaggatctctacagtacttggcactagcagcattaataacaccaaaaaagataaagccacctttacctagt
gttacaaaactgacagaggatcttttagaggagcttaagagtgaagctgttaggcactttcctaggatatggc
tccatggcttagggcaatatatctatgaaacctatggagatacttgggaaggagtggaagccataataagaac
tttgcaacaactgctgttcattcatttcagaattgggtgtcaacatagcagaataggcattactcgacagagg
agagcaagattagagccctgggagcatccaggaagtcaacctaagactgcttgtacctcttgctattgtaaaa
agtgttgctatcattgccaagtctgcttcataacaaaaggcttaggcatctccatgggtggcaagtggtcaaa
aagtagtatggttggatggcctgctgtaagggagagaatgagacgagctgaaccagcagcagagggggtggga
gcagtctctcgagatctggaacgacatggagcgatcacaagtagcaatacagcaaaaaacaatgctgctcttg
cctggctagaagcacaagaggaggaggaggtgggttttccagttagacctcaggtacctttaagaccaatgac
ttacaaggcagctatagatcttagccacttttttaaaagaaaaggggggactggaagggctaatttactcccag
aaaagacaagatatccttgatctgtgggtttaccacacacaaggctacttccctgattggcagaactacacac
cagggccagggaccagattcccactgacctttggatggtgcttcaaactagtaccagttgagccagagaaagt
agaagcagccaatgaaggagagaacaactgcttgttacaccctatgagcctgcatgggatggaggactcggag
ggagaagtgctacagtggaagtttgacagccgcctagcgcttcgtcacatggcccgagagaagcatccggagt
actacaaggactgctga
>#95 KP109514 1398 nt
acatggcacagtttagtaaaacaccatatacatgtttcaaagaaagctcagggatggatttatagacatcatt
atgaaaacactcatccaaaaataagttcagaagtacacattccaataggggaagctaaattggtgataaagac
atattggggtctgcatacaggagaaagagactggcatttgggtcagggagtctccatagaatggaggaaaagg
agatataacacacaagtagaccctggtttagcagaccaactaattcatctgtatcactttgattgtttttcag
aatctgctataagaaatgccatattaggaaatagagttagtcctaggtgtgaatatcaagcaggacataacaa
ggtaggatctctacaatatttggcactaacagcattaataacaccaaagaagacaaagccacctttgcctagc
gttacaaaactgacagaggatcttttagaggagcttaagcatgaagctgttaggcattttcctagaccatggc
ttcatggcctagggcaacatatctatgaaacttatggaatacttgggcaggagtggaggctataataagaat

actgcaacaactgctgtttattcatttcagaattgggtgtcaacatagcagaataggcattattccacagaga
agagcaagactagagccttggaagcatccaggaagtcagcctaaaactgcttgtaacaattgctattgtaaaa
agtgttgctttcactgccaagtttgtttcacaaaaaaaggcttaggcatctccatgggtagcaagtggtcaaa
attgggtggatggcctactgtaagagagagaatgagacgagctcaaccagctcaaccagcagcagatggggtg
ggagcagcatcgcgagacctggaaaaacatggagcaataacaagtagtaatacagcagctaacaatcctgaca
gtgcctggctagaagcacaagaggaagagcaggaggtgggctttccagtcagacctcaggtaccattaagacc
aatgacttacaagggagctttagatcttagccactttttaagagaaaaggggggactggaagggctaatttac
tcccagaaaagaagagacatccttgatctgtgggtctacaacacacaaggctacttccctgattggcagaact
acacaccagggccagggactaggtggccattaacctttggatggtgcttcaagttagtaccagtggagccaga
gaacagtgaaggagagaccaacagtttgctacaccctgcaagcctgcatgggatggaggacccagagggagaa
gtattagtgtggaagtttgacagccgcctagccttccatcacatggcccgcgagctgcatccggagtactaca
aggactgctga
>#96 AF146728 1395 nt
acatggaaaagtttagtaaaacaccatctatataagtcagggaaagctaggagatgggtttatagacatcact
atgaaagcactcatccaagaataagttcagaagtacacatccctctaggggaagctagattggtaataacaac
atattggggtctgcatacaggagaaagagactggcatttaggccagggagtctccatagaatggagaaaaaga
agatatagcacacaagtagaccctggcctagcagaccaactaattcatatgtattattttgattgtttttcag
aatctgctataagaaatgccatattagaacgtatagttagtcctagttgtgaacatcaagcaggacataacaa
ggtaggatctttacagtacttagcactagcagcattaataacaccaagacagacaaagccacctttgcctagt
gtcacgaaactgacagaggatcttttagaggaactcaagagtgaagctgtcagacattttcctaggatatggc
tacatggcctagggcagcatatctataacacctatggggatacttgggcaggagtggaagccttaataagaag
tctgcaacaactgctgtttattcatttcagaattgggtgtcgacacagcagaataggcattaatcctcagagg
agagcaagactagagccctggaagcatccaggaagtcagcctaagactgcttgtaccaattgctattgtaaac
aatgttgctttcattgccaagtttgcttcatacgaaaaggcttaggcatctccatgggtggcaaggggtcaaa
acgtattaggtctgaatggcctactgtaagggaaagaataatacaagctgagccagcagcagctggggtggga
gcagcatctcgagacctggaaaaacatggagcaatcacaagcagcaatattaataacgctgattgtgtttggc
tacaagcacaggaggaggaggaggtgggttttccagtcagacctcaggtacccttaagaccaatgactttcaa
ggcagctcacgatcttagcttcttttaaaagaacaggggggactggaagggctaatttactcccaaagaaga
caagatatccttgatctatggatctatcacacacaaggctacttccctgattggcagaattacacaccagggc
cagggacccgatatccactgacctttggatggtgcttcaagttagtaccagttgagccagatcaggtagaaaa
ggccaatgaaggagagaacatcagtttgttacaccctatgagcctgcacgggatggaggacaaagagaaagaa
gtgttaatgtggaaatttgacagccgcctagcagttcatcacatggcccgagagctgcatccggagtattaca
aaaactga
